# Evolutionary Pattern and Large-Scale Architecture of Mutation Networks of 2009 A (H1N1) Influenza A Virus

**DOI:** 10.3389/fgene.2018.00204

**Published:** 2018-06-07

**Authors:** Chengmin Wang, Nan Lyu, Lingling Deng, Jing Wang, Wenwen Gu, Hua Ding, Yan Wu, Jing Luo, Liang Wang, Xueze Lyv, Xiaodong Liu, Yi Tao, Hongxuan He

**Affiliations:** ^1^Key Lab of Animal Ecology and Conservation Biology, National Research Center for Wildlife-Borne Diseases, Institute of Zoology, Chinese Academy of Sciences, Beijing, China; ^2^Department of Infectious Diseases, Hangzhou Center for Disease Control and Prevention, Hangzhou, China; ^3^Beijing Animal Husbandry Station, Beijing, China

**Keywords:** evolutionary pattern, influenza A virus, scale-free mutation network, H1N1, complete genome

## Abstract

The adaptive evolution of influenza virus is an important question, but predicting its evolutionary future will be more challenging. Here, we investigated the mutation characteristic of influenza virus based on the complete genome data of 2009 (H1N1) influenza A virus. By assuming that evolution proceeds via the accumulation of mutations, we analyzed the mutation networks at four different time stages and found that the network structure follows the characteristics of a scale-free network. These results will be important for epidemiology and the future control of influenza viruses. Furthermore, we predicted the predominant mutation virus strain by using the early mutation network of influenza viruses, and this result was consistent with the WHO recommendation for the candidate vaccine of influenza virus. The key contribution of this study is that we explained the biological significance of this scale-free network for influenza pandemic and provided a potential method for predicting the candidate vaccine by using the early-stage network.

## Introduction

The emergence of a new A (H1N1) virus in the United States and Mexico in March and early April 2009 caused the first influenza pandemic of the 21st century (Ginsberg et al., 2009), which reminded the public of the serious threat of future influenza pandemics. Influenza A virus has a single-strand RNA genome comprising eight individual segments that encode proteins HA, NA, PB1, PB2, PA, NP, M1, M2, NS1, and NEP, where the HA and NA proteins are integral membrane glycoproteins (Webster et al., 1992). The molecular evolutionary studies of 2009 A (H1N1) influenza viruses showed that the new A (H1N1) virus derived from several viruses that had been circulating in pigs for years (Dawood et al., 2009; Vijaykrishna et al., 2010). Phylogenetic data suggested that the reassortment of swine lineages may have occurred years before emergence in humans (Cohen, 2009; Garten et al., 2009; Smith et al., 2009). The continuing evolution of influenza A virus is most prominent in the surface glycoproteins but can also occur in other gene segments (Webster et al., 1992). However, given the evolution of 2009 A (H1N1) influenza viruses in the epidemic, a more challenging problem is to determine the large-scale architecture of the mutation networks for each of the eight gene segments via viral evolution. In a previous study, the evolutionary characteristics of 2009 A (H1N1) influenza virus were investigated (Wang et al., 2013) by constructing a mutation network of non-structural genes using influenza virus genome databases (Bao et al., 2008) in which the progression of the pandemic was divided into four time stages: April/2009, June/2009, October/2009, and March/2010; for convenience, these four time points are denoted stages I, II, III, and IV, respectively.

## Materials and Methods

### Database Preparation

The sequence data of the 2009 novel H1N1 virus used in this study were from the influenza sequence database, which includes all human influenza A viruses with a full-length H1N1 subtype of *HA*, *NA*, *PA*, *PB1*, *PB2*, *NP*, *MP*, and *NS* genes from April 2009 to March 2010 (**Supplementary Tables S5**–**S12**). Multiple alignment of the nucleotide sequences was achieved by using Clustal W.

### Construction of the Mutation Networks

The haplotype distribution of virus gene sequences was analyzed by using DNAsp version: 5.10.1^1^ (Librado and Rozas, 2009), and the mutation networks for the eight genes (2009 A/H1N1) at different time stages were calculated and constructed by using the MJ method in NETWORK version 4.6.1.0. We also used this software to generate networks matrices for further analysis.

### Connectivity Distribution *P*(*k*)

**FIGURE 1:**
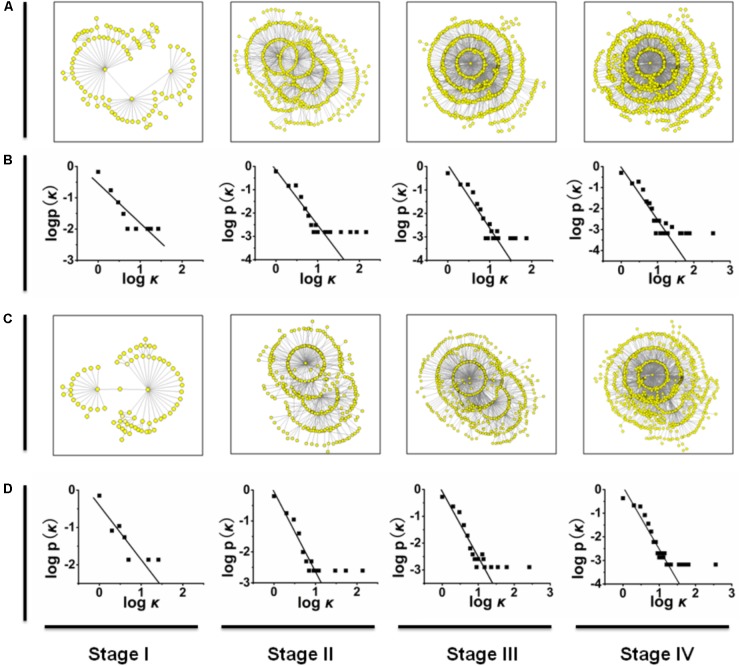
**(A,C)** The mutation networks of *HA* and *NA* genes at different time stages. **(B,D)** The relationships between P(k) and *k* in the mutation networks of *HA* and in the mutation networks of *NA*. Similarly, the mutation networks of other genes are shown in **Supplementary Figure S1**.

Scale-free networks are characterized by a power-law decay of the degree (*k*), i.e., *P*(*k*) ∼*k*^-α^ with an exponent of α. We summarize the connectivity distribution *P*(*k*) for each degree to test whether the gene mutation networks are consistent with and then satisfy the power law required for a scale-free network. To reduce noise, we apply the logarithmic binning as shown in **Figure 1**.

### Pathway Lengths

For each pair of nodes, the shortest pathway is calculated by using Floyd’s algorithm. We then determine the mean diameter of the network through averaging the shortest pathway length between two nodes.

### Network Dynamics

**FIGURE 2:**
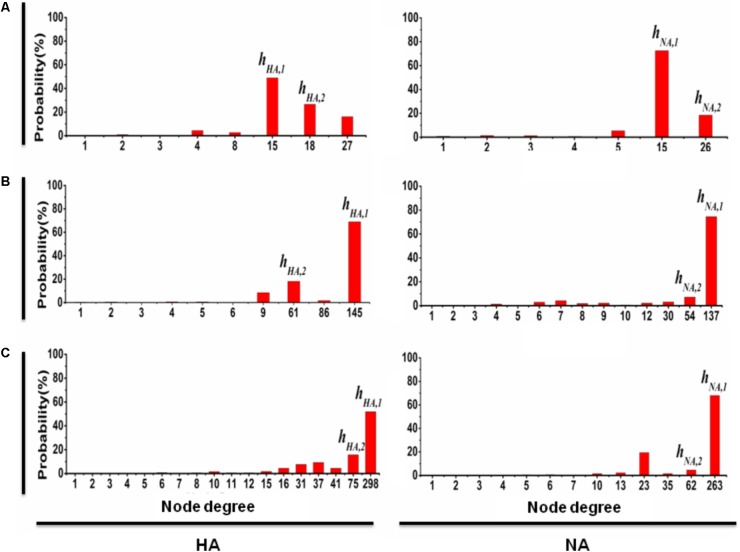
The probability that a new mutation type of *HA* (*NA*) is from the predominant mutation types. At time stage *j* (where *j* = II, III, and IV correspond to **A, B**, and **C**, respectively), the probability that a new mutation of *HA* is from the pre-mutation types is shown on the left; similarly, the probability that a new mutation of *NA* is from the pre-mutation types is shown on the right, where the *x*-axis denotes the degree of the pre-mutation types and the *y*-axis denotes the probability that a new mutation is from the pre-mutation types with a particular degree. The results clearly show that any time stage, most of new mutation types of *HA* (*NA*) are the direct variation of predominant mutation types h_HA,1_ and h_HA,2_ (h_NA,1_ and h_NA,2_). Similar results are obtained for the other six genes (**Supplementary Figure S2**).

To explore the development of gene mutation network, we summarize the origins of those newly detected nodes for each time stage after stage I. **Figure 2** shows the probabilities that the new mutations were generated from the origins with different node degrees.

## Results

### Construction of Mutation Network for Each Gene of Influenza Virus

To reveal the mutation pattern of the pandemic 2009 A (H1N1) influenza virus, using the genome data of 2009 A (H1N1) influenza virus (Influenza Virus Resource^2^), we constructed mutation networks (or graphs) for each of the eight gene segments (*HA*, *NA*, *PB1*, *PB2*, *PA*, *NP*, *MP*, and *NS*) at stages I, II, III, and IV, which are plotted in **Figures 1A,C** (see also **Supplementary Figure S1**). For convenience, the mutation networks in **Figure 1** and **Supplementary Figure S1** can be denoted Π(S,j), where *S* = *HA*, *NA*, *PB1*, *PB2*, *PA*, *NP*, *MP*, and *NS* and *j* = I, II, III, and IV. For example, Π(HA,I) represents the mutation network of gene fragment *HA* at time stage I. In each of the mutation networks Π(S,j), each node represents a particular mutation virus type, the link (or edge) between a pair of nodes denotes the connectivity of these two mutation types because of the mutation (i.e., one mutation type is a direct variation of the other), and the distance between two nodes is defined as the number of links in the shortest pathway connecting these two nodes. The graphic matrices of the mutation networks were calculated by using NETWORK version 4.6.1.0^3^ (see Supplementary Material).

### Mutation Network Follows the Characteristics of a Scale-Free Network

Basically, a network (or graph) can be characterized by its size and degree distribution (Onnela et al., 2007). The size of a network is measured by the total number of nodes, the degree of a node is defined as the number of links connected to this node, and the degree distribution, denoted by P(k), denotes the proportion of the nodes with degree *k* (also called the connectivity distribution; Barabási and Albert, 1999). Based on standard graphic theory (Faloutsos et al., 1999), we analyzed the scale properties of the mutation networks in **Figures 1A,C** and **Supplementary Figures S1A,C,E,G,I,K**, and showed that each of these mutation networks follows a power-law degree distribution P(k) ≈ k^-γ^ with γ > 0, or the mutation networks of H1N1 (2009) influenza virus belong to the class of scale-free networks (Onnela et al., 2007; see **Figures 1B,D** and **Supplementary Figures S1B,D,F,H,J,L**, in which the logarithm of P(k) is linearly regressed on the logarithm of *k*; Faloutsos et al., 1999). For example, the degree distributions of the mutation networks Π(HA,j) can be approximated as P(k) ≈ k^-1.267^ for *j* = I, P(k) ≈ k^-2.356^ for *j* = II, P(k) ≈ k^-2.705^ for *j* = III, and P(k) ≈ k^-2.501^ for *j* = IV (see **Figures 1B,D**). One of the most important properties of scale-free networks is that they are extremely heterogeneous, with a topology dominated by a few highly connected nodes (hubs) that link the remaining less connected nodes to the system (Callaway et al., 2000). Thus, for each of the eight gene segments of H1N1 (2009) influenza virus, the scale properties of the mutation networks imply that the new mutations at any time stage come mainly from a few predominant mutation types.

However, for each of the mutation networks in **Figure 1** and **Supplementary Figure S1**, the ratio of the total number of links to the total number of nodes (denoted by N_link_/N_node_, where N_link_ represents the total number of links and N_node_ is the total number of nodes, which equals the half of average degree) and the network diameter were calculated. For each of the eight gene segments, the changes in the ratio N_link_/N_node_ from time stage I to IV are monotonically incremental, but these increments are small compared to the increase in the total number of mutation types from the time stage I to IV (see **Supplementary Table S1**). These results suggest that for the evolutionary dynamics of H1N1 (2009) influenza virus in the pandemic, the increase in the number of mutation types cannot result in a large change (i.e., increase) in the average degree of the mutation networks. In standard graphic theory, the diameter of a network is defined as the shortest path averaged over all pairs of nodes (Barabási and Albert, 1999). Previous studies on the non-biological networks have shown that the diameter of a network logarithmically increases with the addition of new nodes (Bianconi and Barabási, 2001; Santiago and Benito, 2008). However, an investigation of the metabolic networks showed that the diameter of the metabolic network is the same for all 43 organisms, regardless of the number of substrates found in a given species (Jeong et al., 2000). Similar to this study, the diameters of the mutation networks of H1N1 (2009) influenza virus are shown in **Supplementary Table S2**, and we found that for each of the eight gene segments, the mutation networks Π(S,j) for *j* = I, II, III, and IV have similar network diameters (i.e., the difference between these four network diameters is not significant), or the diameter of the mutation network should be relatively independent of the change in the total number of mutation types. Thus, for each of the eight gene segments, the changes in the ratio N_link_/N_node_ and the diameter of the mutation network from the time stage I to IV also imply that most of new mutation types should derive from a few predominant mutation types at any time stage.

The power-law degree distribution also indicates that in a scale-free network, a few hubs dominate the overall connectivity of the network (Chung and Lu, 2006; Durrett, 2007). This idea implies that the removal of some hubs from a scale-free network will lead to a large link-lost, defined as a ratio (1 - N_link,after_)/ N_link,before_, where N_link,after_ and N_link,before_ denote the numbers of links after and before removing the hubs from the network, respectively. For each of the mutation networks shown in **Figure 1** and **Supplementary Figure S1**, when some hubs (or mutation types) are removed from the network, the size of the link-lost should reflect the importance of these hubs in the evolution of pandemic H1N1 (2009) influenza virus (i.e., the larger the link-lost size is, the greater the probability is that the new mutation types are direct variations of a few predominant mutation types). For each of the eight gene segments, we found that the mutation networks Π(S,j) for *j* = I, II, III, and IV have the same two mutation types with maximum degree. For convenience, we denote these two mutation types by h_S,1_ and h_S,2_ for all *S* = *HA*, *NA*, *PB1*, *PB2*, *PA*, *NP*, *MP*, and *NS*, and the biological information of h_S,1_ and h_S,2_ is listed in **Supplementary Table S3**.

### The Predominant Mutation Types Are Essential for Structure of Scale-Free Network

The results of the link-lost at time stages I, II, III, and IV for each gene segment, when both h_S,1_ and h_S,2_ are removed from the mutation networks, are shown in **Supplementary Table S4**, in which the decline of the size of the link-lost from time stage I to IV is slow. We also calculated the probabilities that a new mutation type is a direct variation of h_S,1_ or h_S,2_ in the period from time stages I to II, II to III, and III to IV (see **Figure 2** and **Supplementary Figure S2**). For example, the probability that a new mutation type of gene fragment *HA* is from h_HA,1_ (h_HA,2_) is 49% (26%) at time stage II (left in **Figure 2A**), 69% (16%) at time stage III (left in **Figure 2B**), and 52% (15%) at time stage IV (left in **Figure 2C**). Similarly, for gene fragment *NA*, the probability that new mutation type is from h_NA,1_ (h_NA,2_) is 72% (18%) at time stage II (right in **Figure 2A**), 74% (7%) at time stage III (right in **Figure 2B**), and 67% (5%) at time stage IV (right in **Figure 2C**). This result shows clearly that for the evolution of 2009 A (H1N1) influenza virus in the pandemic, the mutation types h_S,1_ and h_S,2_ for each of the eight gene segments (i.e., for *S = HA, NA, PB1, PB2, PA, NP, MP*, and *NS*) are considered the predominant mutation types.

### Predication of Candidate Vaccine Using Early-Stage Network

**FIGURE 3:**
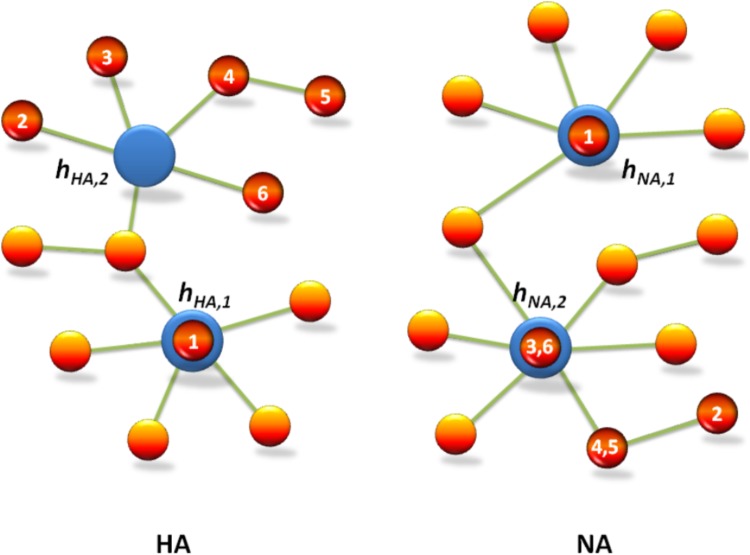
Mutation network analysis is consistent with the WHO recommendation for the candidate vaccine of 2009 A (H1N1) influenza virus. The red circles indicate vaccine strains recommended by WHO, which are denoted by No. 1–6, i.e., A/New York/18/2009, A/Texas/15/2009, A/Texas/5/2009, A/California/7/2009, A/California/4/2009, and A/England/195/2009, respectively. The blue circles indicate predominant mutation types h_HA,1_ and h_HA,2_ in *HA*, and h_NA,1_ and h_NA,2_ in *NA*, which are predicted by the mutation network analysis of 2009 A (H1N1).

The preparation of the candidate vaccine of 2009 A (H1N1) influenza virus was based on the WHO recommendation to use six wild-type candidate vaccine virus strains: A/NewYork/18/2009, A/England/195/2009, A/Texas/05/2009, A/California/04/2009 and A/California/07/2009, and A/Texas/15/2009. For the mutation networks of gene segments *HA* and *NA*, (i) the mutation types of *HA* and *NA* in A/NewYork/18/2009 are precisely h_HA,1_ and h_NA,1_, respectively; (ii) the distance from the mutation type of *HA* in A/England/195/2009, in A/Texas/05/2009, and in A/California/07/2009 to h_HA,2_ is 2, and the distance from the mutation type of *HA* in A/California/04/2009 to h_HA,2_ is 3 (where the distance from the mutation type of *HA* in A/California/04/2009 to the mutation type of *HA* in A/California/07/2009 is 1); and (iii) the mutation type of *NA* in both A/England/195/2009 and A/Texas/05/2009 is exactly h_NA,2_, and the distance from the mutation type of *NA* in both A/California/04/2009 and A/California/07/2009 to h_NA,2_ is 1 (see **Figure 3**). These results clearly show that our mutation network analysis not only is consistent with the WHO recommendations (see also **Table 1**) but also provides insight for predicting the predominant virus strain in the pandemic of influenza virus by using the early mutation networks.

**Table 1:** Comparison of the predicted predominant mutation types based on the mutation network of April/2009 (stage I) to the WHO’s recommendation for the candidate vaccine strains based on *HA* and *NA* genes.

Gene	Predominant mutation type	Mutation site	Predicted vaccine strains based on mutation network analysis	WHO’s recommendation for vaccine strains
HA	hHA,1	658A	A/New York/18/2009 A/California/14/2009 A/Texas/45/2009 A/England/202/2009	A/New York/18/2009 A/California/7/2009 A/California/4/2009 A/Texas/5/2009 A/England/195/2009 A/Texas/15/2009
	hHA,2	658T	A/Texas/19/2009 A/England/197/2009 A/Mexico/4115/2009	
NA	hNA,1	742 G(248 D); 316A(106 I)	A /British Columbia/GFA0401/2009 A/California/14/2009 A/New York/25/2009 A/Texas/23/2009 A/New York/18/2009	A/New York/18/2009 A/California/7/2009 A/California/4/2009 A/Texas/5/2009 A/England/195/2009 A/Texas/15/2009
	hNA,2	742A(248N); 316 G(106 V)	A/England/196/2009 A/New York/3178/2009 A/Texas/04/2009 A/Texas/5/2009 A/England/195/2009	

## Discussion

A key objective for research of influenza A is to improve the accuracy of vaccine strain choice. A better understanding of the evolutionary and epidemiological rules governing antigenic drift, viral fitness, the role of the source region, and establishment of predominance would be particularly helpful for the selection of vaccine strains when considerable variation among antigenically novel strains is observed and it is unclear which, if any, will become predominant. The World Health Organization (WHO) maintains a global surveillance program (McHardy and Adams, 2009). A panel of experts meets twice a year to review antigenic, genetic, and epidemiological data and decides on the vaccine composition for the next winter season in global (Russell et al., 2008). An update of the vaccine strain is recommended if an emerging antigenic variant is detected and judged likely to become predominant. Problems arise when an emerging variant is not identified early enough for an update of the vaccine composition. Thus, gaining a detailed understanding of the evolution and epidemiology of the virus is of the utmost importance, as it may lead to earlier identification of novel emerging variants. As existing databases grow, new statistical and computational techniques are being developed for interpretation of these large-scale, population-level genomic datasets in combination with epidemiological and phenotypic information.

Evolution is a dynamic principle that connects the past and the future. According to this principle, the fitness differences between individuals in a population are important driving forces of evolution (Lässig and Łuksza, 2014). The most prominent characteristic of influenza virus is its continuing evolution through gene mutation, which is why new flu vaccines are needed every year. Therefore, the evolutionary pattern and vaccine prediction have become the most important issues.

**FIGURE 4:**
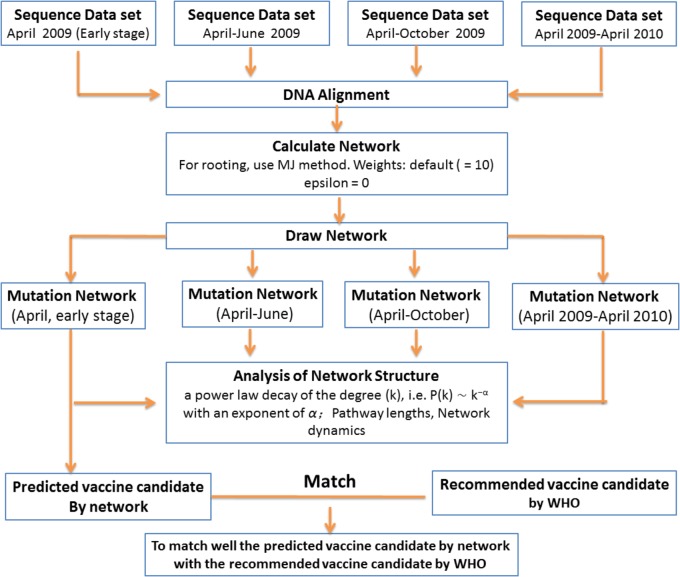
Scheme for prediction of evolutionary pattern in short-term pandemic of influenza viruses.

In general, most attempts to predict the evolution of influenza viruses have focused on identifying specific features within genetic sequences that might indicate fitness. However, such approaches require information on the viruses, but this information is often not available. In the present study, we developed a more general method to predict candidate vaccines from virus genetic sequences. The basic idea is simple (**Figure 4**). In the first step, because the fitness differences within a population are carried by genetic mutations, a “gene mutation network” for influenza virus population – which shows how each strain of the virus is related to other strains based on the accumulation of multiple mutation sites – was constructed according to the different mutation sites of the virus sequences. In the next step, the dynamic changes of the gene mutation network were analyzed with the increasing number of new virus strains according to the different stage of the influenza pandemic (e.g., stages I, II, III, and IV in our study). The basic structure of this dynamic network will show a visible imprint of the natural selection process. In the last step, by using this insight and methods borrowed from mathematics, the network structure was analyzed to select the predominant mutation virus type in the pandemic as a candidate vaccine.

Recently, there were two important progresses in predictive fitness models and predicting evolution for influenza based on genealogical trees. Łuksza and Lässig (2014) developed a fitness model for hemagglutinin that predicts the evolution of the viral population from one year to the next, and this model depends on two factors: adaptive epitope changes and deleterious mutations outside the epitopes. Moreover, Neher et al. (2014) demonstrated that the branching patterns of reconstructed genealogical trees contain information on the relative fitness of the sampled sequences based on the assumption that evolution proceeds by the accumulation of small effect mutations. However, compared to the abovementioned studies, we investigated the mutation pathway of influenza virus in the pandemic by using network theory. By using the genome data of 2009 (H1N1) influenza A virus, all the mutation networks at different time stages were constructed, and the network structure follows the standard power-law degree distribution (scale-free network). This result indicates that the mutation of virus is mainly dominated by a few predominant virus strains, or at any time stage, most of the new mutation types should be the direct variations of the predominant mutation types.

The key strength of our method is that it uses only the historical data on influenza A/H1N1 (2009) virus contained in a network. Thus, it can be applied in cases where we do not know which functions undergo adaptive evolution or where in the genome these functions are encoded. These results are important for influenza prevention and control: the dynamical mutation networks clearly show the mutation pathway of influenza virus in short-term evolutionary patterns, and visually demonstrate the role of the predominant virus strains in early stages of the pandemic (**Figure 2**). It is reasonable that the predominant virus strains in early stages were selected as candidate vaccines (**Figure 3**). Additionally, these dynamic mutation networks also show the developmental trend of the pandemic that spread rapidly in early stages and gradually slowed in late stages.

Inferring evolutionary patterns from genealogical trees has a long history. Geneticists use probabilistic methods to map mutations onto specific tree branches (Łuksza and Lässig, 2014). Determining how often these mutations appear in different lineages can tell us which fitness effects are predominant in a population (McDonald and Kreitman, 1991; Strelkowa and Lässig, 2012). From the statistics of the genealogical tree itself, epidemiologists infer the growth rate of pathogen populations and use this information to predict the future course of a pandemic (nd Stadler, 2010; Łuksza and Lässig, 2014).

This research based on 2009 H1N1 virus is particularly important with respect to the emergence of novel viruses with pandemic potential. Especially, the time period between the detection of the virus and the onset of a pandemic is too short to produce a specific vaccine for immediate vaccination of the population. Such a framework (**Figure 4**) could utilize epidemiological, genomic, and antigenic information and detailed knowledge of the genetic and epidemiological characteristics of antigenic drift to assess the likelihood of strains rising to predominance. Altogether, the current predictions are approximately halfway between random picks and optimal predictions. The current methods have advantages or disadvantages for influenza prediction and should be the ultimate test used in the future.

## Conclusion

We elucidate the evolutionary characteristic of 2009 A (H1N1) influenza viruses in the pandemic by using the mutation networks for each of the eight gene segments at four time stages based on the standard graph theory. The results demonstrate that all the mutation networks satisfy the power-law degree distribution. This property strongly implies that the evolution pattern of 2009 A (H1N1) influenza viruses in the pandemic should be mainly determined by some predominant mutation types (or predominant pandemic virus strains). Furthermore, for each gene segment, we identify the two predominant mutation types, and show that most of new mutation types at any time stage are directly derived from these two predominant mutation types. Our mutation network analysis of the predominant mutation types is also consistent with the WHO recommendation for the candidate vaccine of 2009 A (H1N1) influenza virus. These results not only provide a new insight for revealing the evolutionary dynamics of 2009 A (H1N1) influenza viruses in the pandemic but also demonstrate that the analysis of mutation networks is valuable for predicting the predominant virus strain in the influenza pandemic.

## Author Contributions

HH, CW, and YT designed the study. CW, JW, WG, YW, and JL collected the data. CW, NL, LD, HD, XuL, LW, and XiL analyzed the data. CW, HH, and YT wrote and edited the manuscript.

## Conflict of Interest Statement

The authors declare that the research was conducted in the absence of any commercial or financial relationships that could be construed as a potential conflict of interest.

## Footnotes

1 http://www.ub.edu/dnasp/

2 http://www.ncbi.nlm.nih.gov/genomes/FLU/FLU.html

3 http://www.fluxus-engineering.com/

## Supplementary Material

FIGURE S1 Mutation networks of *PB1*, *PB2*, *NP*, *MP*, *NS*, and *PA* genes at different time stages (from April 2009 to March 2010). **(A,C,E,G,I,K)** The mutation networks of *PB1*, *PB2*, *NP*, *MP*, *NS*, and *PA* genes at different time stages. **(B**,**D**,**F**,**H**,**J,L)** The relationships between P(k) and *k* in these mutation networks.

FIGURE S2 The probability that a new mutation type of *PB1* (*PB2*, *NP*, *MP*, *NS*, and *PA*) is from the pre-mutation types. At time stage *j* (where*j* = II, III, and IV correspond to **A, B**, and **C**, respectively), the probability that a new mutation type is from the pre-mutation types for each of *PB1*, *PB2*, *NP*, *MP*, *NS*, and *PA* genes is shown, where the *x*-axis denotes the degree of the pre-mutation types and the *y*-axis denotes the probability that a new mutation is from the pre-mutation types with a particular degree.

TABLE S1 The ratio N_link_/N_link_N_node_. N_node_ of the mutation networks for each of the eight genes at different time stages.

TABLE S2 The diameter of the mutation networks for each of the eight genes from time stage I to time stage IV.

TABLE S3 Biological information of the two predominant mutation types in each of the eight genes.

TABLE S4 The link-lost when the two predominant mutation types are removed for each of the eight genes.

TABLE S5 GenBank number of *HA* gene sequences used in this study.

TABLE S6 GenBank number of *NA* gene sequences used in this study.

TABLE S7 GenBank number of *PB2* gene sequences used in this study.

TABLE S8 GenBank number of *PB1* gene sequences used in this study.

TABLE S9 GenBank number of *PA* gene sequences used in this study.

TABLE S10 GenBank number of *NS* gene sequences used in this study.

TABLE S11 GenBank number of *NP* gene sequences used in this study.

TABLE S12 GenBank number of *M* gene sequences used in this study.
