# Supplementary figures and images for: Evolutionary Pattern and Large-Scale Architecture of Mutation Networks of 2009 A (H1N1) Influenza A Virus
Source: Front Genet. 2018 Jun 7;9:204. doi: 10.3389/fgene.2018.00204 (PMC6008563; doi:10.3389/fgene.2018.00204)

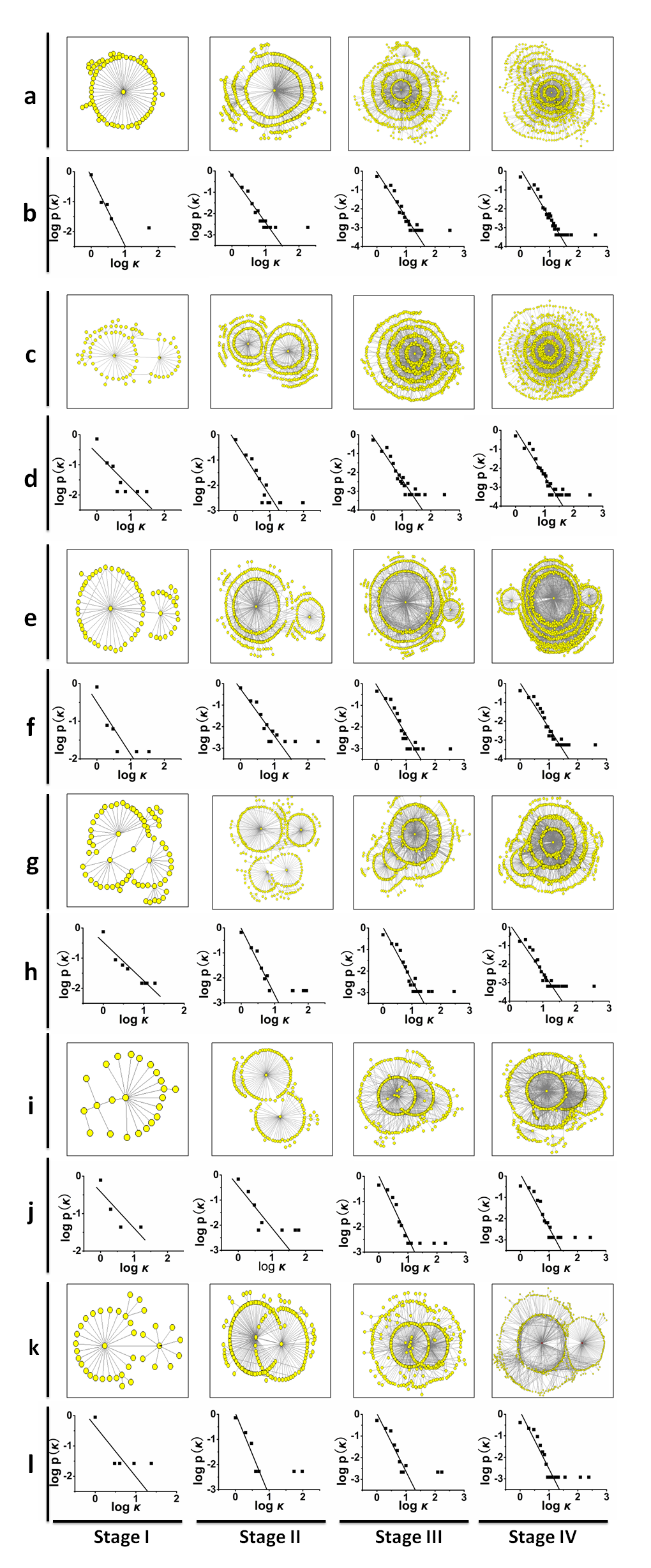

Supplement: FIGURE S1 — Mutation networks of PB1, PB2, NP, MP, NS, and PA genes at different time stages (from April 2009 to March 2010). (A,C,E,G,I,K) The mutation networks of PB1, PB2, NP, MP, NS, and PA genes at different time stages. (B,D,F,H,J,L) The relationships between P(k) and k in these mutation networks. [file Image_1.TIF]

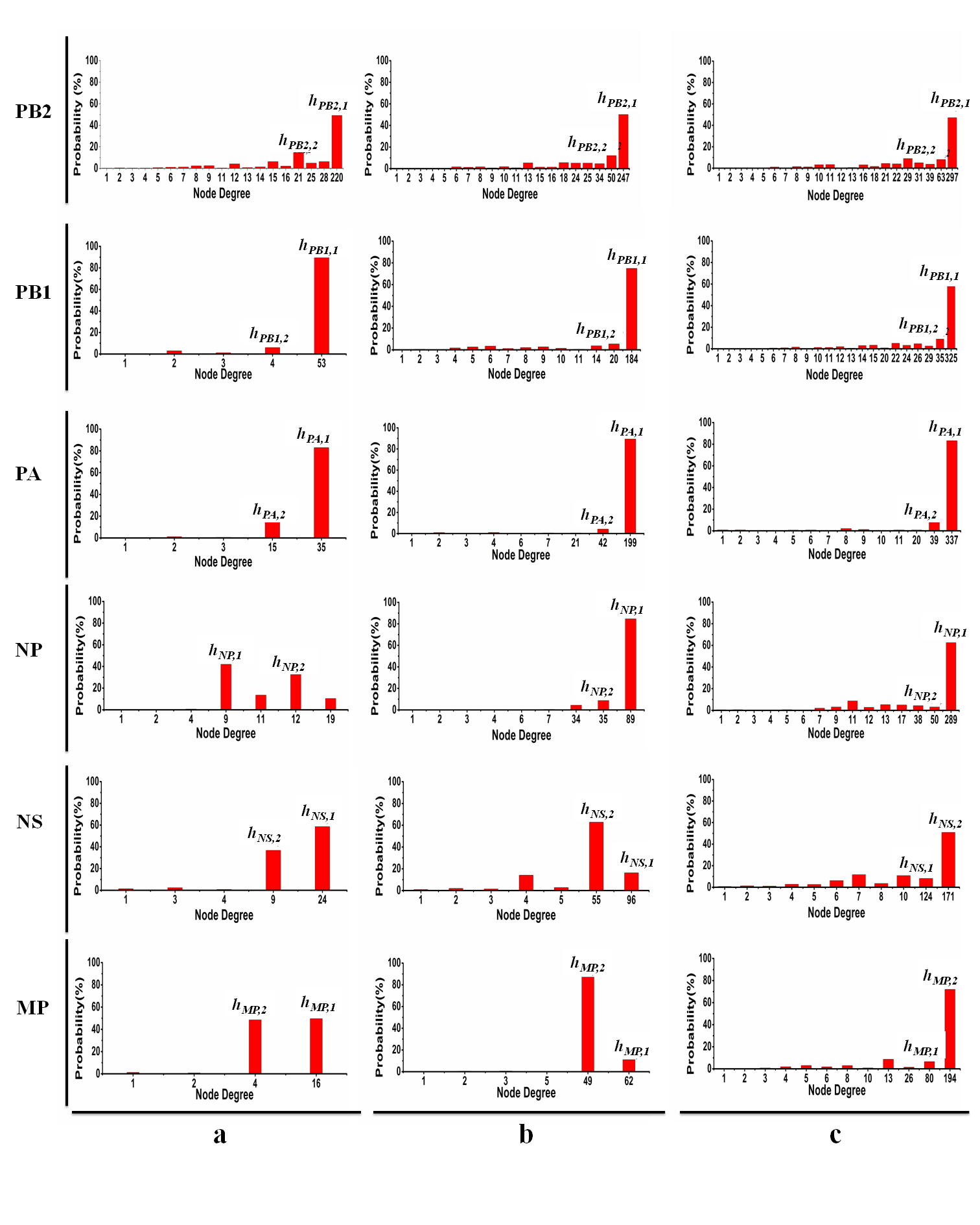

Supplement: FIGURE S2 — The probability that a new mutation type of PB1 (PB2, NP, MP, NS, and PA) is from the pre-mutation types. At time stage j (wherej = II, III, and IV correspond to A, B, and C, respectively), the probability that a new mutation type is from the pre-mutation types for each of PB1, PB2, NP, MP, NS, and PA genes is shown, where the x-axis denotes the degree of the pre-mutation types and the y-axis denotes the probability that a new mutation is from the pre-mutation types with a particular degree. [file Image_2.TIF]
