# Supplementary material for: Evolutionary Pattern and Large-Scale Architecture of Mutation Networks of 2009 A (H1N1) Influenza A Virus
Source: Front Genet. 2018 Jun 7;9:204. doi: 10.3389/fgene.2018.00204 (PMC6008563; doi:10.3389/fgene.2018.00204)
Supplement: TABLE S1 — The ratio Nlink/NlinkNnode. Nnode of the mutation networks for each of the eight genes at different time stages. [file Table_1.doc]

**Table S1. The ratio** $N_{link}/N_{Node}$ **of the mutation networks for each of the eight genes at different time stage.**

| **Ratio** $N_{link}/N_{Node}$ | | | | | | | | |
| --- | --- | --- | --- | --- | --- | --- | --- | --- |
| **Time Stage** | ***HA*** | ***NA*** | ***MP*** | ***NP*** | ***PA*** | ***NS*** | ***PB1*** | ***PB2*** |
| **I** | 1.05102 | 1.027 | 0.957 | 1.015 | 1 | 0.974 | 1.013 | 1.209 |
| **II** | 1.16641 | 1.121 | 1.115 | 1.137 | 1.143 | 1.080 | 1.107 | 1.253 |
| **III** | 1.2644 | 1.208 | 1.332 | 1.321 | 1.339 | 1.248 | 1.250 | 1.265 |
| **IV** | 1.29742 | 1.352 | 1.499 | 1.430 | 1.429 | 1.407 | 1.329 | 1.310 |

**Table S2. The diameter of the mutation networks for each of the eight genes from time stage I to time stage IV.**

| **Stage** | ***HA*** | ***NA*** | ***MP*** | ***NP*** | ***PA*** | ***NS*** | ***PB1*** | ***PB2*** |
| --- | --- | --- | --- | --- | --- | --- | --- | --- |
| I | 3.715±1.264 | 3.708±1.349 | 2.656±1.115 | 4.106±1.651 | 2.805±0.861 | 2.503±0.697 | 2.805±0.861 | 4.121±1.369 |
| II | 4.460±1.509 | 4.140±1.489 | 3.513±1.294 | 4.353±1.830 | 3.532±1.136 | 2.861±0.835 | 3.889±1.463 | 4.352±1.423 |
| III | 4.342±1.432 | 4.170±1.488 | 3.337±1.079 | 4.247±1.568 | 4.037±1.406 | 3.342±1.035 | 4.833±1.727 | 4.886±1.656 |
| IV | 4.498±1.420 | 4.546±1.555 | 3.740±1.283 | 4.681±1.603 | 4.590±1.529 | 3.847±1.196 | 5.636±2.061 | 5.638±1.965 |

**Table S3.** Biological information of the two predominant mutation types in each of the eight genes.

| **Gene** | **Predominant mutation type** | **Mutation site** | **Representative virus** |
| --- | --- | --- | --- |
| ***HA*** | ***h_HA,1_*** | 658A | A/New York/18/2009 |
|  | ***h_HA,2_*** | 658T | A/Texas/19/2009 |
| ***NA*** | ***h_NA,1_*** | 316A (106I) | A/New York/18/2009※ |
|  | ***h_NA,2_*** | 742A (248N) | A/England/195/2009※ |
| ***PB1*** | ***h_PB1,1_*** | 300A, 1058A (353K) | A/Mexico/4108/2009 |
|  | ***h_PB1,2_*** | 1058G (353R), 300G | A/England/196/2009 |
| ***PB2*** | ***h_PB2,1_*** | 2163G | A/Mexico/4108/2009 |
|  | ***h_PB2,2_*** | 2163A | A/California/04/2009 |
| ***PA*** | ***H_PA,1_*** | 1741A | A/California/14/2009 |
|  | ***H_PA,2_*** | 1741C | A/England/197/2009 |
| ***NP*** | ***h_NP,1_*** | 1143A, 1248A | A/New Jersey/01/2009 |
|  | ***h_NP,2_*** | 1143G, 1248G | A/Mexico/4482/2009 |
| ***NS*** | ***h_NS,1_*** | 367G (123V) | A/Toronto/3184/2009 |
|  | ***h_NS,2_*** | 367A (123I) | A/California/04/2009 |
| ***M*** | ***h_M,1_*** | 492G | A/California/04/2009※ |
|  | ***h_M,2_*** | 492A | A/New York/27/2009 |

**Table S4. The link-lost when the two predominant mutation types are removed for each of the eight genes.**

| **Removal** |  | **Link-Lost (%)** | | | | | | | |
| --- | --- | --- | --- | --- | --- | --- | --- | --- | --- |
|  | **Time Stage** | ***HA*** | ***NA*** | ***MP*** | ***NP*** | ***PA*** | ***NS*** | ***PB1*** | ***PB2*** |
| ***h_S,1_+h_S,2_*** | I | 92.192 | 94.366 | 100 | 84.662 | 98.625 | 98.571 | 98.820 | 95.298 |
|  | II | 64.229 | 86.064 | 95.802 | 88.559 | 96.935 | 99.453 | 90.779 | 91.081 |
|  | III | 51.735 | 65.810 | 85.586 | 60.902 | 43.576 | 95.507 | 51.634 | 30.996 |
|  | IV | 36.051 | 32.591 | 40.946 | 33.425 | 28.673 | 49.719 | 28.067 | 15.851 |
| ***h_S,1_*** | I | 79.445 | 87.989 | 100 | 56.445 | 90.271 | 39.039 | 98.185 | 83.971 |
|  | II | 39.168 | 65.019 | 76.815 | 43.253 | 87.858 | 52.577 | 86.353 | 46.453 |
|  | III | 38.582 | 50.399 | 52.673 | 40.334 | 39.712 | 53.056 | 44.782 | 24.310 |
|  | IV | 28.085 | 22.586 | 25.229 | 16.929 | 19.914 | 25.620 | 14.573 | 11.831 |
| ***h_S,2_*** | I | 56.121 | 41.744 | 100 | 47.354 | 48.950 | 93.243 | 7.997 | 33.219 |
|  | II | 19.423 | 39.060 | 56.377 | 32.766 | 16.392 | 80.959 | 12.936 | 33.743 |
|  | III | 9.699 | 22.430 | 15.026 | 27.456 | 3.442 | 32.106 | 3.113 | 4.395 |
|  | IV | 7.7714 | 11.398 | 5.643 | 18.194 | 9.803 | 17.574 | 14.643 | 2.608 |

**Table S5.** GenBank number of *HA* gene sequences used in this study.

**Table S6.** GenBank number of *NA* gene sequences used in this study.

**Table S7.** GenBank number of *PB2* gene sequences used in this study.

**Table S8.** GenBank number of *PB1* gene sequences used in this study.

**Table S9.** GenBank number of *PA* gene sequences used in this study.

**Table S10.** GenBank number of *NS* gene sequences used in this study.

**Table S11.** GenBank number of *NP* gene sequences used in this study.

**Table S12.** GenBank number of *M* gene sequences used in this study.
