# Supplementary material for: Evolutionary Pattern and Large-Scale Architecture of Mutation Networks of 2009 A (H1N1) Influenza A Virus
Source: Front Genet. 2018 Jun 7;9:204. doi: 10.3389/fgene.2018.00204 (PMC6008563; doi:10.3389/fgene.2018.00204)
Supplement: Supplementary file 4 [file Table_2.PDF]

Name	Sequence Acc	Subtype	Collection	Country	Strain Name
HA	GQ162194	H1N1	04/02/2009	Mexico	A/Mexico/3955/2009(H1N1)
HA	GQ117044	H1N1	04/01/2009	USA	A/California/04/2009(H1N1)
HA	FJ966082	H1N1	04/01/2009	USA	A/California/04/2009
HA	GQ149662	H1N1	04/03/2009	Mexico	A/Mexico/4108/2009(H1N1)
HA	GQ149689	H1N1	04/03/2009	Mexico	A/Mexico/4108/2009(H1N1)
HA	GQ149668	H1N1	04/03/2009	Mexico	A/Mexico/4108/2009(H1N1)
HA	GQ149654	H1N1	04/03/2009	Mexico	A/Mexico/4108/2009(H1N1)
HA	GQ162170	H1N1	04/03/2009	Mexico	A/Mexico/4108/2009(H1N1)
HA	GQ223112	H1N1	04/03/2009	Mexico	A/Mexico/4108/2009(H1N1)
HA	GQ149692	H1N1	04/07/2009	Mexico	A/Mexico/4115/2009(H1N1)
HA	FJ969511	H1N1	04/08/2009	USA	A/California/10/2009(H1N1)
HA	KC780060	H1N1	04/09/2009	USA	A/California/08/2009
HA	KC781785	H1N1	04/09/2009	USA	A/California/07/2009
HA	KF009554	H1N1	04/09/2009	USA	A/California/07/2009
HA	GQ377052	H1N1	04/09/2009	USA	A/California/08/2009(H1N1)
HA	KU933485	H1N1	04/09/2009	USA	A/California/07/2009
HA	CY181401	H1N1	04/09/2009	USA	A/California/07-JR003/2009
HA	CY181425	H1N1	04/09/2009	USA	A/California/07-JR010/2009
HA	CY181393	H1N1	04/09/2009	USA	A/California/07-JR002/2009
HA	CY181409	H1N1	04/09/2009	USA	A/California/07-JR008/2009
HA	CY266191	H1N1	04/09/2009	USA	A/California/07/2009
HA	CY181417	H1N1	04/09/2009	USA	A/California/07-JR009/2009
HA	FJ971076	H1N1	04/09/2009	USA	A/California/08/2009(H1N1)
HA	FJ969540	H1N1	04/09/2009	USA	A/California/07/2009(H1N1)
HA	FJ981613	H1N1	04/09/2009	USA	A/California/07/2009(H1N1)
HA	FJ966974	H1N1	04/09/2009	USA	A/California/07/2009(H1N1)
HA	KC781677	H1N1	04/10/2009	USA	A/Michigan/30/2009
HA	GQ162182	H1N1	04/13/2009	Mexico	A/Mexico/4176/2009(H1N1)
HA	GQ149674	H1N1	04/14/2009	Mexico	A/Mexico/4482/2009(H1N1)
HA	FJ981615	H1N1	04/14/2009	USA	A/Texas/04/2009(H1N1)
HA	GQ149671	H1N1	04/14/2009	Mexico	A/Mexico/4482/2009(H1N1)
HA	GQ149647	H1N1	04/14/2009	Mexico	A/Mexico/4486/2009(H1N1)
HA	GQ162202	H1N1	04/14/2009	Mexico	A/Mexico/4486/2009(H1N1)
HA	GQ162190	H1N1	04/14/2009	Mexico	A/Mexico/4482/2009(H1N1)
HA	GQ149623	H1N1	04/14/2009	Mexico	A/Mexico/4486/2009(H1N1)
HA	GQ162185	H1N1	04/14/2009	Mexico	A/Mexico/4486/2009(H1N1)
HA	GQ221798	H1N1	04/14/2009	USA	A/Texas/04/2009(H1N1)
HA	CY044235	H1N1	04/14/2009	USA	A/San Antonio/PR921/2009(H1N1)
HA	CY147771	H1N1	04/14/2009	Mexico	A/Mexico/24033/2009
HA	FJ998208	H1N1	04/14/2009	Mexico	A/Mexico/InDRE4487/2009(H1N1)
HA	GQ149677	H1N1	04/14/2009	Mexico	A/Mexico/4482/2009(H1N1)
HA	GQ377072	H1N1	04/14/2009	USA	A/Texas/04/2009(H1N1)
HA	GQ303340	H1N1	04/14/2009	Mexico	A/Mexico/InDRE4487/2009(H1N1)
HA	FJ966982	H1N1	04/14/2009	USA	A/Texas/04/2009(H1N1)
HA	FJ981612	H1N1	04/14/2009	USA	A/Texas/04/2009(H1N1)
HA	GQ457487	H1N1	04/15/2009	USA	A/Texas/05/2009(H1N1)
HA	GQ168861	H1N1	04/15/2009	USA	A/Texas/05/2009(H1N1)
HA	GQ122097	H1N1	04/15/2009	USA	A/Texas/15/2009(H1N1)
HA	GQ162172	H1N1	04/15/2009	Mexico	A/Mexico/4269/2009(H1N1)
HA	GQ339881	H1N1	04/15/2009	Mexico	A/Mexico/4283/2009(H1N1)
HA	FJ966959	H1N1	04/15/2009	USA	A/Texas/05/2009(H1N1)
HA	GQ402194	H1N1	04/16/2009	Canada	A/Canada-QC/RV1595/2009(H1N1)
HA	FJ966960	H1N1	04/16/2009	USA	A/California/06/2009(H1N1)

HA	KC781723	H1N1	04/16/2009	USA	A/California/06/2009
HA	CY147763	H1N1	04/17/2009	Mexico	A/Mexico/24032/2009
HA	CY147819	H1N1	04/17/2009	Mexico	A/Mexico/24043/2009
HA	GQ162195	H1N1	04/17/2009	Mexico	A/Mexico/4502/2009(H1N1)
HA	GQ149641	H1N1	04/19/2009	Mexico	A/Mexico/4603/2009(H1N1)
HA	GQ149684	H1N1	04/19/2009	Mexico	A/Mexico/4603/2009(H1N1)
HA	GQ149630	H1N1	04/19/2009	Mexico	A/Mexico/4603/2009(H1N1)
HA	GQ149634	H1N1	04/19/2009	Mexico	A/Mexico/4604/2009(H1N1)
HA	GQ162197	H1N1	04/20/2009	Mexico	A/Mexico/4575/2009(H1N1)
HA	GQ162200	H1N1	04/20/2009	Mexico	A/Mexico/4635/2009(H1N1)
HA	CY147835	H1N1	04/20/2009	Mexico	A/Mexico/24045/2009
HA	GQ162191	H1N1	04/20/2009	Mexico	A/Mexico/4595/2009(H1N1)
HA	GQ162183	H1N1	04/20/2009	Mexico	A/Mexico/4627/2009(H1N1)
HA	CY147827	H1N1	04/20/2009	Mexico	A/Mexico/24044/2009
HA	GQ162204	H1N1	04/20/2009	Mexico	A/Mexico/4646/2009(H1N1)
HA	GQ162174	H1N1	04/20/2009	Mexico	A/Mexico/4593/2009(H1N1)
HA	GQ160607	H1N1	04/21/2009	USA	A/California/13/2009(H1N1)
HA	GQ232010	H1N1	04/21/2009	USA	A/California/13/2009(H1N1)
HA	CY043094	H1N1	04/21/2009	USA	A/Brawley/40082/2009(H1N1)
HA	GQ117067	H1N1	04/22/2009	USA	A/Arizona/01/2009(H1N1)
HA	GQ377093	H1N1	04/22/2009	USA	A/California/12/2009(H1N1)
HA	GQ117097	H1N1	04/22/2009	USA	A/Indiana/09/2009(H1N1)
HA	GQ160606	H1N1	04/22/2009	USA	A/California/12/2009(H1N1)
HA	CY043086	H1N1	04/22/2009	USA	A/Brawley/40081/2009(H1N1)
HA	CY147699	H1N1	04/23/2009	Mexico	A/Mexico/24021/2009
HA	CY147611	H1N1	04/23/2009	Mexico	A/Mexico/24007/2009
HA	FJ984385	H1N1	04/23/2009	USA	A/Texas/06/2009(H1N1)
HA	GQ232002	H1N1	04/23/2009	USA	A/Texas/10/2009(H1N1)
HA	GQ232076	H1N1	04/23/2009	USA	A/Texas/11/2009(H1N1)
HA	GQ168642	H1N1	04/24/2009	USA	A/Iowa/04/2009(H1N1)
HA	GQ168644	H1N1	04/24/2009	USA	A/Kansas/03/2009(H1N1)
HA	FJ984364	H1N1	04/24/2009	USA	A/New York/23/2009(H1N1)
HA	GQ160579	H1N1	04/24/2009	USA	A/California/11/2009(H1N1)
HA	FJ984397	H1N1	04/24/2009	USA	A/Ohio/07/2009(H1N1)
HA	GQ232019	H1N1	04/24/2009	USA	A/Texas/17/2009(H1N1)
HA	GQ232028	H1N1	04/24/2009	USA	A/Illinois/01/2009(H1N1)
HA	FJ984360	H1N1	04/24/2009	USA	A/New York/31/2009(H1N1)
HA	GQ200250	H1N1	04/24/2009	USA	A/New York/21/2009(H1N1)
HA	CY041549	H1N1	04/24/2009	USA	A/New York/3210/2009(H1N1)
HA	FJ974026	H1N1	04/24/2009	Canada	A/Toronto/3141/2009
HA	GQ338349	H1N1	04/24/2009	USA	A/Michigan/04/2009(H1N1)
HA	GQ232049	H1N1	04/24/2009	USA	A/New York/25/2009(H1N1)
HA	FJ998207	H1N1	04/24/2009	Canada	A/Canada-NS/RV1535/2009(H1N1)
HA	FJ998209	H1N1	04/24/2009	Canada	A/Canada-ON/RV1527/2009(H1N1)
HA	GQ117059	H1N1	04/24/2009	USA	A/Kansas/02/2009(H1N1)
HA	GQ200221	H1N1	04/24/2009	USA	A/Texas/12/2009(H1N1)
HA	GQ221814	H1N1	04/24/2009	USA	A/New York/16/2009(H1N1)
HA	GQ221818	H1N1	04/24/2009	USA	A/California/11/2009(H1N1)
HA	FJ984401	H1N1	04/24/2009	USA	A/Ohio/07/2009(H1N1)
HA	GQ117024	H1N1	04/24/2009	USA	A/New York/22/2009(H1N1)
HA	GQ168628	H1N1	04/24/2009	USA	A/New York/23/2009(H1N1)
HA	GQ168633	H1N1	04/24/2009	USA	A/Texas/08/2009(H1N1)
HA	GQ117100	H1N1	04/24/2009	USA	A/Ohio/07/2009(H1N1)
HA	GQ168661	H1N1	04/24/2009	USA	A/New York/31/2009(H1N1)

HA	GQ117051	H1N1	04/24/2009	USA	A/Texas/08/2009(H1N1)
HA	GQ160601	H1N1	04/24/2009	USA	A/New York/24/2009(H1N1)
HA	GQ168617	H1N1	04/24/2009	USA	A/New York/26/2009(H1N1)
HA	CY065150	H1N1	04/24/2009	United Ki	A/Scotland/91800799/2009
HA	GQ117062	H1N1	04/24/2009	USA	A/Kansas/03/2009(H1N1)
HA	GQ168650	H1N1	04/25/2009	USA	A/New York/08/2009(H1N1)
HA	GQ168886	H1N1	04/25/2009	USA	A/New York/09/2009(H1N1)
HA	GQ168668	H1N1	04/25/2009	USA	A/New York/13/2009(H1N1)
HA	GQ160567	H1N1	04/25/2009	USA	A/New York/34/2009(H1N1)
HA	FJ984375	H1N1	04/25/2009	USA	A/New York/10/2009(H1N1)
HA	GQ117086	H1N1	04/25/2009	USA	A/New York/20/2009(H1N1)
HA	GQ117091	H1N1	04/25/2009	USA	A/Texas/07/2009(H1N1)
HA	CY086977	H1N1	04/25/2009	USA	A/New York/3217/2009(H1N1)
HA	GQ117082	H1N1	04/25/2009	USA	A/New York/20/2009(H1N1)
HA	FJ984355	H1N1	04/25/2009	USA	A/New York/18/2009(H1N1)
HA	GQ232064	H1N1	04/25/2009	USA	A/New York/18/2009(H1N1)
HA	GQ117103	H1N1	04/25/2009	USA	A/Massachusetts/07/2009(H1N1)
HA	GQ231996	H1N1	04/25/2009	USA	A/New York/34/2009(H1N1)
HA	GQ231999	H1N1	04/25/2009	USA	A/New York/33/2009(H1N1)
HA	GQ377103	H1N1	04/25/2009	USA	A/New York/45/2009(H1N1)
HA	CY065142	H1N1	04/25/2009	United Ki	A/Scotland/91800797/2009
HA	CY041130	H1N1	04/25/2009	USA	A/New York/3215/2009(H1N1)
HA	CY044163	H1N1	04/25/2009	Mexico	A/Mexico/48N/2009(H1N1)
HA	CY040637	H1N1	04/25/2009	USA	A/New York/3191/2009(H1N1)
HA	GQ160602	H1N1	04/25/2009	USA	A/New York/39/2009(H1N1)
HA	GQ168652	H1N1	04/25/2009	USA	A/New York/11/2009(H1N1)
HA	CY041122	H1N1	04/25/2009	USA	A/New York/3214/2009(H1N1)
HA	GQ168671	H1N1	04/25/2009	USA	A/Texas/09/2009(H1N1)
HA	GQ168851	H1N1	04/25/2009	USA	A/New York/06/2009(H1N1)
HA	CY147675	H1N1	04/25/2009	Mexico	A/Mexico/24015/2009
HA	GQ377061	H1N1	04/25/2009	USA	A/Texas/20/2009(H1N1)
HA	CY097876	H1N1	04/25/2009	Mexico	A/Mexico/47N/2009
HA	GQ457497	H1N1	04/25/2009	USA	A/New York/15/2009(H1N1)
HA	FJ984337	H1N1	04/25/2009	USA	A/New York/12/2009(H1N1)
HA	FJ984347	H1N1	04/25/2009	USA	A/New York/11/2009(H1N1)
HA	FJ969509	H1N1	04/25/2009	USA	A/New York/19/2009(H1N1)
HA	GQ232017	H1N1	04/25/2009	USA	A/New York/39/2009(H1N1)
HA	GQ232038	H1N1	04/25/2009	USA	A/California/17/2009(H1N1)
HA	GQ231995	H1N1	04/25/2009	USA	A/New York/35/2009(H1N1)
HA	CY041613	H1N1	04/25/2009	USA	A/New York/3002/2009(H1N1)
HA	CY041541	H1N1	04/25/2009	USA	A/New York/3209/2009(H1N1)
HA	CY040888	H1N1	04/25/2009	Mexico	A/Mexico/47N/2009(H1N1)
HA	GQ221786	H1N1	04/25/2009	USA	A/New York/19/2009(H1N1)
HA	GQ117032	H1N1	04/25/2009	USA	A/Texas/09/2009(H1N1)
HA	GQ117040	H1N1	04/25/2009	USA	A/California/14/2009(H1N1)
HA	GQ168655	H1N1	04/25/2009	USA	A/New York/09/2009(H1N1)
HA	GQ168673	H1N1	04/25/2009	USA	A/New York/12/2009(H1N1)
HA	GQ160527	H1N1	04/25/2009	USA	A/New York/37/2009(H1N1)
HA	GQ160543	H1N1	04/25/2009	USA	A/New York/35/2009(H1N1)
HA	GQ160556	H1N1	04/25/2009	USA	A/New York/18/2009(H1N1)
HA	GQ117116	H1N1	04/25/2009	USA	A/New York/13/2009(H1N1)
HA	FJ984394	H1N1	04/25/2009	USA	A/New York/19/2009(H1N1)
HA	GQ231981	H1N1	04/26/2009	USA	A/South Carolina/10/2009(H1N1)
HA	CY041050	H1N1	04/26/2009	USA	A/New York/3166/2009(H1N1)

HA	GQ338409	H1N1	04/26/2009	USA	A/Texas/37/2009(H1N1)
HA	CY039893	H1N1	04/26/2009	USA	A/New York/1669/2009(H1N1)
HA	GQ221788	H1N1	04/26/2009	USA	A/Arizona/02/2009(H1N1)
HA	GQ221794	H1N1	04/26/2009	USA	A/South Carolina/09/2009(H1N1)
HA	GQ117043	H1N1	04/26/2009	USA	A/Massachusetts/06/2009(H1N1)
HA	GQ117056	H1N1	04/26/2009	USA	A/South Carolina/09/2009(H1N1)
HA	GQ117112	H1N1	04/26/2009	USA	A/Michigan/02/2009(H1N1)
HA	CY041066	H1N1	04/26/2009	USA	A/New York/3169/2009(H1N1)
HA	GQ160538	H1N1	04/26/2009	USA	A/New Mexico/04/2009(H1N1)
HA	GQ168676	H1N1	04/26/2009	USA	A/Washington/11/2009(H1N1)
HA	GQ465677	H1N1	04/26/2009	Canada	A/Canada-PQ/RV1586/2009(H1N1)
HA	GQ457482	H1N1	04/26/2009	USA	A/Nevada/03/2009(H1N1)
HA	JN171868	H1N1	04/26/2009	Canada	A/Montreal/01/2009
HA	CY041058	H1N1	04/26/2009	USA	A/New York/3168/2009(H1N1)
HA	GQ117079	H1N1	04/26/2009	USA	A/Arizona/02/2009(H1N1)
HA	CY041170	H1N1	04/26/2009	USA	A/New York/3186/2009(H1N1)
HA	GQ168620	H1N1	04/26/2009	USA	A/Texas/19/2009(H1N1)
HA	CY041530	H1N1	04/26/2009	USA	A/New York/3205/2009(H1N1)
HA	CY041605	H1N1	04/26/2009	USA	A/New York/3207/2009(H1N1)
HA	GQ221805	H1N1	04/26/2009	USA	A/Arizona/03/2009(H1N1)
HA	CY041098	H1N1	04/26/2009	USA	A/New York/3176/2009(H1N1)
HA	GQ221820	H1N1	04/26/2009	USA	A/Colorado/04/2009(H1N1)
HA	GQ160574	H1N1	04/26/2009	USA	A/Texas/22/2009(H1N1)
HA	CY041734	H1N1	04/26/2009	USA	A/New York/3206/2009(H1N1)
HA	GQ232003	H1N1	04/27/2009	USA	A/Oregon/04/2009(H1N1)
HA	CY065246	H1N1	04/27/2009	United Ki	A/England/XFL00071/2009
HA	CY041514	H1N1	04/27/2009	USA	A/New York/3203/2009(H1N1)
HA	GQ232023	H1N1	04/27/2009	USA	A/District of Columbia/01/2009(H1N1)
HA	GQ338364	H1N1	04/27/2009	USA	A/Minnesota/02/2009(H1N1)
HA	GQ338414	H1N1	04/27/2009	USA	A/Wisconsin/07/2009(H1N1)
HA	CY040822	H1N1	04/27/2009	USA	A/New York/3260/2009(H1N1)
HA	GQ338368	H1N1	04/27/2009	USA	A/Ohio/08/2009(H1N1)
HA	CY040042	H1N1	04/27/2009	USA	A/New York/3007/2009(H1N1)
HA	GQ160526	H1N1	04/27/2009	USA	A/Florida/04/2009(H1N1)
HA	GQ160605	H1N1	04/27/2009	USA	A/Wisconsin/08/2009(H1N1)
HA	GQ200268	H1N1	04/27/2009	USA	A/District of Columbia/03/2009(H1N1)
HA	GQ200273	H1N1	04/27/2009	USA	A/Wisconsin/09/2009(H1N1)
HA	GQ168664	H1N1	04/27/2009	USA	A/Washington/12/2009(H1N1)
HA	CY040015	H1N1	04/27/2009	USA	A/New York/3014/2009(H1N1)
HA	CY147883	H1N1	04/27/2009	Mexico	A/Mexico/24052/2009
HA	CY147867	H1N1	04/27/2009	Mexico	A/Mexico/24050/2009
HA	CY147843	H1N1	04/27/2009	Mexico	A/Mexico/24047/2009
HA	CY039901	H1N1	04/27/2009	USA	A/New York/1682/2009(H1N1)
HA	GQ160591	H1N1	04/27/2009	USA	A/Virginia/04/2009(H1N1)
HA	CY040007	H1N1	04/27/2009	USA	A/New York/3012/2009(H1N1)
HA	CY039999	H1N1	04/27/2009	USA	A/New York/3008/2009(H1N1)
HA	CY147779	H1N1	04/27/2009	Mexico	A/Mexico/24036/2009
HA	CY070671	H1N1	04/27/2009	United Ki	A/England/XFL00077/2009
HA	GQ338391	H1N1	04/27/2009	USA	A/Washington/08/2009(H1N1)
HA	GQ402191	H1N1	04/27/2009	Mexico	A/Mexico/InDRE13547/2009(H1N1)
HA	CY065238	H1N1	04/27/2009	United Ki	A/England/551/2009
HA	CY065198	H1N1	04/27/2009	United Ki	A/England/197/2009
HA	CY147715	H1N1	04/27/2009	Mexico	A/Mexico/24024/2009
HA	CY147891	H1N1	04/27/2009	Mexico	A/Mexico/24053/2009

HA	CY147899	H1N1	04/27/2009	Mexico	A/Mexico/24054/2009
HA	CY147875	H1N1	04/27/2009	Mexico	A/Mexico/24051/2009
HA	CY147851	H1N1	04/27/2009	Mexico	A/Mexico/24048/2009
HA	GQ221812	H1N1	04/27/2009	USA	A/Colorado/03/2009(H1N1)
HA	GQ160599	H1N1	04/27/2009	USA	A/Nebraska/03/2009(H1N1)
HA	CY041082	H1N1	04/27/2009	USA	A/New York/3172/2009(H1N1)
HA	GQ117119	H1N1	04/27/2009	USA	A/Colorado/03/2009(H1N1)
HA	CY065214	H1N1	04/27/2009	United Ki	A/England/205/2009
HA	CY069773	H1N1	04/27/2009	United Ki	A/England/209/2009
HA	CY065190	H1N1	04/27/2009	United Ki	A/England/202/2009
HA	CY147707	H1N1	04/27/2009	Mexico	A/Mexico/24022/2009
HA	CY065222	H1N1	04/27/2009	United Ki	A/England/207/2009
HA	GQ894929	H1N1	04/27/2009	USA	A/Alabama/02/2009(H1N1)
HA	GQ465678	H1N1	04/27/2009	Canada	A/Canada-ON/RV1589/2009(H1N1)
HA	CY147859	H1N1	04/27/2009	Mexico	A/Mexico/24049/2009
HA	GQ221809	H1N1	04/27/2009	USA	A/Arizona/04/2009(H1N1)
HA	GQ200237	H1N1	04/27/2009	USA	A/Georgia/01/2009(H1N1)
HA	GQ160550	H1N1	04/27/2009	USA	A/Texas/23/2009(H1N1)
HA	CY040838	H1N1	04/27/2009	USA	A/New York/3262/2009(H1N1)
HA	CY040023	H1N1	04/27/2009	USA	A/New York/3049/2009(H1N1)
HA	CY041565	H1N1	04/27/2009	USA	A/New York/3225/2009(H1N1)
HA	CY069765	H1N1	04/27/2009	United Ki	A/England/196/2009
HA	CY041522	H1N1	04/27/2009	USA	A/New York/3204/2009(H1N1)
HA	CY041114	H1N1	04/28/2009	USA	A/New York/3193/2009(H1N1)
HA	CY040701	H1N1	04/28/2009	USA	A/New York/3235/2009(H1N1)
HA	CY041490	H1N1	04/28/2009	USA	A/New York/3184/2009(H1N1)
HA	GQ232057	H1N1	04/28/2009	USA	A/Delaware/08/2009(H1N1)
HA	CY041186	H1N1	04/28/2009	USA	A/New York/3188/2009(H1N1)
HA	CY041202	H1N1	04/28/2009	USA	A/New York/3199/2009(H1N1)
HA	CY041506	H1N1	04/28/2009	USA	A/New York/3196/2009(H1N1)
HA	CY041154	H1N1	04/28/2009	USA	A/New York/3183/2009(H1N1)
HA	CY041557	H1N1	04/28/2009	USA	A/New York/3211/2009(H1N1)
HA	CY041573	H1N1	04/28/2009	USA	A/New York/3227/2009(H1N1)
HA	CY041589	H1N1	04/28/2009	USA	A/New York/3236/2009(H1N1)
HA	GQ338394	H1N1	04/28/2009	USA	A/Delaware/06/2009(H1N1)
HA	GQ160586	H1N1	04/28/2009	USA	A/North Carolina/05/2009(H1N1)
HA	GQ160594	H1N1	04/28/2009	USA	A/Delaware/02/2009(H1N1)
HA	GQ221823	H1N1	04/28/2009	USA	A/Delaware/05/2009(H1N1)
HA	GQ168636	H1N1	04/28/2009	USA	A/Delaware/04/2009(H1N1)
HA	CY041090	H1N1	04/28/2009	USA	A/New York/3173/2009(H1N1)
HA	CY041106	H1N1	04/28/2009	USA	A/New York/3178/2009(H1N1)
HA	CY147731	H1N1	04/28/2009	Mexico	A/Mexico/24026/2009
HA	GQ160541	H1N1	04/28/2009	USA	A/Delaware/03/2009(H1N1)
HA	CY147747	H1N1	04/28/2009	Mexico	A/Mexico/24028/2009
HA	GQ160582	H1N1	04/28/2009	USA	A/New Hampshire/02/2009(H1N1)
HA	GQ160531	H1N1	04/28/2009	USA	A/Tennessee/05/2009(H1N1)
HA	GQ160578	H1N1	04/28/2009	USA	A/Washington/09/2009(H1N1)
HA	GQ200208	H1N1	04/28/2009	USA	A/Oregon/06/2009(H1N1)
HA	CY040629	H1N1	04/28/2009	USA	A/New York/3234/2009(H1N1)
HA	GQ168631	H1N1	04/28/2009	USA	A/North Carolina/06/2009(H1N1)
HA	CY040717	H1N1	04/28/2009	USA	A/New York/3238/2009(H1N1)
HA	GQ464408	H1N1	04/28/2009	Spain	A/Catalonia/63/2009(H1N1)
HA	GQ465673	H1N1	04/28/2009	Canada	A/Canada-AB/RV1531/2009(H1N1)
HA	GQ402192	H1N1	04/28/2009	Mexico	A/Mexico/InDRE13551/2009(H1N1)

HA	CY047318	H1N1	04/28/2009	USA	A/New York/3124/2009(H1N1)
HA	CY047326	H1N1	04/28/2009	USA	A/New York/3167/2009(H1N1)
HA	CY050067	H1N1	04/28/2009	USA	A/Cherry Point/WR1355/2009(H1N1)
HA	CY073394	H1N1	04/28/2009	USA	A/Brawley/WR1635P/2009(H1N1)
HA	CY065230	H1N1	04/28/2009	United Ki	A/England/200/2009
HA	GQ166661	H1N1	04/28/2009	United Ki	A/England/195/2009
HA	CY065254	H1N1	04/28/2009	United Ki	A/England/211/2009
HA	CY147739	H1N1	04/28/2009	Mexico	A/Mexico/24027/2009
HA	CY147923	H1N1	04/28/2009	Mexico	A/Mexico/24057/2009
HA	CY147787	H1N1	04/28/2009	Mexico	A/Mexico/24038/2009
HA	CY147939	H1N1	04/28/2009	Mexico	A/Mexico/24060/2009
HA	CY147947	H1N1	04/28/2009	Mexico	A/Mexico/24061/2009
HA	CY147915	H1N1	04/28/2009	Mexico	A/Mexico/24056/2009
HA	GQ338400	H1N1	04/28/2009	USA	A/Pennsylvania/10/2009(H1N1)
HA	CY041146	H1N1	04/28/2009	USA	A/New York/3181/2009(H1N1)
HA	GQ221826	H1N1	04/28/2009	USA	A/Idaho/02/2009(H1N1)
HA	GQ232044	H1N1	04/28/2009	USA	A/Rhode Island/02/2009(H1N1)
HA	CY041498	H1N1	04/28/2009	USA	A/New York/3195/2009(H1N1)
HA	GQ232037	H1N1	04/28/2009	USA	A/Arizona/06/2009(H1N1)
HA	GQ338355	H1N1	04/28/2009	USA	A/Delaware/07/2009(H1N1)
HA	CY065206	H1N1	04/28/2009	United Ki	A/England/204/2009
HA	CY041830	H1N1	04/28/2009	USA	A/New York/3348/2009(H1N1)
HA	GQ323564	H1N1	04/28/2009	USA	A/Utah/02/2009(H1N1)
HA	CY046331	H1N1	04/28/2009	USA	A/Wisconsin/629-D01505/2009(H1N1)
HA	CY053071	H1N1	04/28/2009	USA	A/Houston/130S/2009(H1N1)
HA	CY040709	H1N1	04/28/2009	USA	A/New York/3237/2009(H1N1)
HA	GQ168623	H1N1	04/28/2009	USA	A/Washington/07/2009(H1N1)
HA	GQ221791	H1N1	04/28/2009	USA	A/Oklahoma/01/2009(H1N1)
HA	CY041581	H1N1	04/28/2009	USA	A/New York/3230/2009(H1N1)
HA	CY147931	H1N1	04/28/2009	Mexico	A/Mexico/24058/2009
HA	CY147907	H1N1	04/28/2009	Mexico	A/Mexico/24055/2009
HA	CY041482	H1N1	04/29/2009	USA	A/New York/3189/2009(H1N1)
HA	HM567552	H1N1	04/29/2009	United Ki	A/England/213/2009
HA	CY041162	H1N1	04/29/2009	USA	A/New York/3185/2009(H1N1)
HA	GQ338372	H1N1	04/29/2009	USA	A/Nevada/04/2009(H1N1)
HA	GQ338375	H1N1	04/29/2009	USA	A/Texas/36/2009(H1N1)
HA	GQ338339	H1N1	04/29/2009	USA	A/Louisiana/02/2009(H1N1)
HA	GQ465675	H1N1	04/29/2009	Canada	A/Canada-NS/RV1554/2009(H1N1)
HA	GQ200231	H1N1	04/29/2009	USA	A/District of Columbia/02/2009(H1N1)
HA	GQ200255	H1N1	04/29/2009	USA	A/Oklahoma/02/2009(H1N1)
HA	GQ200270	H1N1	04/29/2009	USA	A/Oregon/05/2009(H1N1)
HA	CY147803	H1N1	04/29/2009	Mexico	A/Mexico/24040/2009
HA	CY040031	H1N1	04/29/2009	USA	A/New York/3099/2009(H1N1)
HA	CY147795	H1N1	04/29/2009	Mexico	A/Mexico/24039/2009
HA	GQ402193	H1N1	04/29/2009	Mexico	A/Mexico/InDRE13555/2009(H1N1)
HA	GQ402189	H1N1	04/29/2009	Mexico	A/Mexico/InDRE13494/2009(H1N1)
HA	GQ402190	H1N1	04/29/2009	Mexico	A/Mexico/InDRE13495/2009(H1N1)
HA	CY047310	H1N1	04/29/2009	USA	A/New York/3074/2009(H1N1)
HA	CY046299	H1N1	04/29/2009	USA	A/Wisconsin/629-D01486/2009(H1N1)
HA	GQ365658	H1N1	04/29/2009	Germany	A/Bayern/62/2009(H1N1)
HA	CY065342	H1N1	04/29/2009	United Ki	A/England/223/2009
HA	CY040854	H1N1	04/29/2009	USA	A/New York/3264/2009(H1N1)
HA	CY041194	H1N1	04/29/2009	USA	A/New York/3190/2009(H1N1)
HA	CY086969	H1N1	04/29/2009	USA	A/New York/3198/2009(H1N1)

HA	GQ465674	H1N1	04/29/2009	Canada	A/Canada-NS/RV1551/2009(H1N1)
HA	GQ338416	H1N1	04/29/2009	USA	A/Indiana/12/2009(H1N1)
HA	CY041726	H1N1	04/29/2009	USA	A/New York/3247/2009(H1N1)
HA	GQ247724	H1N1	04/29/2009	Brazil	A/Sao Paulo/1454/2009
HA	GQ377087	H1N1	04/29/2009	USA	A/Oregon/07/2009(H1N1)
HA	CY073402	H1N1	04/29/2009	USA	A/Brawley/WR1636P/2009(H1N1)
HA	CY039527	H1N1	04/29/2009	Netherlar	A/Netherlands/602/2009
HA	CY065262	H1N1	04/29/2009	United Ki	A/England/XFL00137/2009
HA	CY046307	H1N1	04/29/2009	USA	A/Wisconsin/629-D01642/2009(H1N1)
HA	CY147755	H1N1	04/29/2009	Mexico	A/Mexico/24030/2009
HA	GQ160565	H1N1	04/29/2009	USA	A/Maryland/05/2009(H1N1)
HA	CY040862	H1N1	04/29/2009	USA	A/New York/3265/2009(H1N1)
HA	GQ214151	H1N1	04/29/2009	France	A/Paris/2573/2009(H1N1)
HA	CY040830	H1N1	04/29/2009	USA	A/New York/3261/2009(H1N1)
HA	GQ232014	H1N1	04/29/2009	USA	A/Texas/28/2009(H1N1)
HA	GQ232033	H1N1	04/30/2009	USA	A/Louisiana/04/2009(H1N1)
HA	CY064740	H1N1	04/30/2009	Mexico	A/Mexico City/024/2009(H1N1)
HA	CY046843	H1N1	04/30/2009	USA	A/Wisconsin/629-D02361/2009(H1N1)
HA	CY046259	H1N1	04/30/2009	USA	A/Wisconsin/629-D01773/2009(H1N1)
HA	CY046387	H1N1	04/30/2009	USA	A/Wisconsin/629-D00750/2009(H1N1)
HA	GQ323483	H1N1	04/30/2009	USA	A/California/18/2009(H1N1)
HA	CY073366	H1N1	04/30/2009	USA	A/San Diego/WR1631P/2009(H1N1)
HA	CY064708	H1N1	04/30/2009	Mexico	A/Mexico City/020/2009
HA	GQ894862	H1N1	04/30/2009	USA	A/Florida/10/2009(H1N1)
HA	CY046355	H1N1	04/30/2009	USA	A/Wisconsin/629-D01529/2009(H1N1)
HA	CY046515	H1N1	04/30/2009	USA	A/Wisconsin/629-D02141/2009(H1N1)
HA	CY046411	H1N1	04/30/2009	USA	A/Wisconsin/629-D00447/2009(H1N1)
HA	CY049828	H1N1	04/30/2009	USA	A/Cherry Point/WR0080/2009(H1N1)
HA	GQ214138	H1N1	04/30/2009	France	A/Paris/2590/2009(H1N1)
HA	GQ214144	H1N1	04/30/2009	France	A/Paris/2580/2009(H1N1)
HA	CY041621	H1N1	04/30/2009	USA	A/New York/3100/2009(H1N1)
HA	CY046419	H1N1	04/30/2009	USA	A/Wisconsin/629-D00767/2009(H1N1)
HA	CY064700	H1N1	04/30/2009	Mexico	A/Mexico City/019/2009
HA	GU931801	H1N1	04/30/2009	Hong Kong	A/Hong Kong/415742/2009(H1N1)
HA	CY073350	H1N1	04/30/2009	USA	A/San Diego/WR1629P/2009(H1N1)
HA	GQ465681	H1N1	04/30/2009	Canada	A/Canada-SK/RV1788/2009(H1N1)
HA	GQ377045	H1N1	04/30/2009	USA	A/Tennessee/07/2009(H1N1)
HA	GQ377075	H1N1	04/30/2009	USA	A/Washington/18/2009(H1N1)
HA	CY047350	H1N1	04/30/2009	USA	A/New York/3365/2009(H1N1)
HA	CY046451	H1N1	04/30/2009	USA	A/Wisconsin/629-D01558/2009(H1N1)
HA	CY046475	H1N1	04/30/2009	USA	A/Wisconsin/629-D00015/2009(H1N1)
HA	HM567560	H1N1	04/30/2009	United Ki	A/England/217/2009
HA	CY065270	H1N1	04/30/2009	United Ki	A/England/214/2009
HA	CY065286	H1N1	04/30/2009	United Ki	A/England/216/2009
HA	GQ232009	H1N1	04/30/2009	USA	A/Kentucky/05/2009(H1N1)
HA	GQ377101	H1N1	04/30/2009	USA	A/California/19/2009(H1N1)
HA	CY040645	H1N1	04/30/2009	USA	A/New York/3194/2009(H1N1)
HA	CY040693	H1N1	04/30/2009	USA	A/New York/3232/2009(H1N1)
HA	GQ232052	H1N1	04/30/2009	USA	A/Ohio/10/2009(H1N1)
HA	GQ232054	H1N1	04/30/2009	USA	A/South Dakota/05/2009(H1N1)
HA	GQ338358	H1N1	04/30/2009	USA	A/Hawaii/09/2009(H1N1)
HA	GQ168606	H1N1	04/30/2009	Hong Kong	A/Hong Kong/01/2009(H1N1)
HA	GQ338402	H1N1	04/30/2009	USA	A/Pennsylvania/09/2009(H1N1)
HA	GQ323443	H1N1	04/30/2009	USA	A/Florida/11/2009(H1N1)

HA	GQ323451	H1N1	04/30/2009	USA	A/Rhode Island/03/2009(H1N1)
HA	GQ465676	H1N1	04/30/2009	Canada	A/Canada-NS/RV1565/2009(H1N1)
HA	CY065278	H1N1	04/30/2009	United Ki	A/England/215/2009
HA	AB568123	H1N1	05/2009	Japan	A/Kobe/9015/2009
HA	CY062375	H1N1	05/2009	Kuwait	A/Kuwait/N05052/2009(H1N1)
HA	CY062374	H1N1	05/2009	Kuwait	A/Kuwait/N05051/2009(H1N1)
HA	AB568143	H1N1	05/2009	Japan	A/Kobe/9336/2009
HA	AB568153	H1N1	05/2009	Japan	A/Kobe/91248/2009
HA	CY087288	H1N1	05/2009	Hong Kong	A/Hong Kong/29992/2009
HA	CY062376	H1N1	05/2009	Kuwait	A/Kuwait/N05053/2009(H1N1)
HA	AB568138	H1N1	05/2009	Japan	A/Kobe/9185/2009
HA	CY062373	H1N1	05/2009	Kuwait	A/Kuwait/N05050/2009(H1N1)
HA	CY107558	H1N1	05/2009	United Ki	A/Scotland/Glasgow_412118/2009
HA	GQ377035	H1N1	05/2009	USA	A/Alaska/04/2009(H1N1)
HA	AB568133	H1N1	05/2009	Japan	A/Kobe/9154/2009
HA	AB568148	H1N1	05/2009	Japan	A/Kobe/9358/2009
HA	GQ258462	H1N1	05/2009	Australia	A/South Australia/2001/2009(H1N1)
HA	AB568128	H1N1	05/2009	Japan	A/Kobe/9109/2009
HA	CY039986	H1N1	05/2009	Thailand	A/Nonthaburi/102/2009
HA	CY053103	H1N1	05/01/2009	USA	A/Houston/15H/2009(H1N1)
HA	CY046315	H1N1	05/01/2009	USA	A/Wisconsin/629-D01308/2009(H1N1)
HA	GQ377085	H1N1	05/01/2009	USA	A/California/20/2009(H1N1)
HA	CY047342	H1N1	05/01/2009	USA	A/New York/3345/2009(H1N1)
HA	CY046491	H1N1	05/01/2009	USA	A/Wisconsin/629-D02028/2009(H1N1)
HA	CY046427	H1N1	05/01/2009	USA	A/Wisconsin/629-D00677/2009(H1N1)
HA	CY064748	H1N1	05/01/2009	Mexico	A/Mexico City/025/2009(H1N1)
HA	GQ214156	H1N1	05/01/2009	France	A/Paris/2592/2009(H1N1)
HA	CY041178	H1N1	05/01/2009	USA	A/New York/3187/2009(H1N1)
HA	GQ232067	H1N1	05/01/2009	USA	A/Louisiana/03/2009(H1N1)
HA	GQ323473	H1N1	05/01/2009	USA	A/Louisiana/05/2009(H1N1)
HA	GQ338378	H1N1	05/01/2009	USA	A/Oklahoma/03/2009(H1N1)
HA	GQ323560	H1N1	05/01/2009	USA	A/Florida/09/2009(H1N1)
HA	CY053095	H1N1	05/01/2009	USA	A/Houston/150S/2009(H1N1)
HA	CY064732	H1N1	05/01/2009	Mexico	A/Mexico City/023/2009(H1N1)
HA	GQ249333	H1N1	05/01/2009	France	A/Paris/2591/2009(H1N1)
HA	GQ465679	H1N1	05/01/2009	Canada	A/Canada-AB/RV1644/2009(H1N1)
HA	CY046291	H1N1	05/01/2009	USA	A/Wisconsin/629-D01817/2009(H1N1)
HA	CY053087	H1N1	05/01/2009	USA	A/Houston/14H/2009(H1N1)
HA	GQ457517	H1N1	05/01/2009	USA	A/California/27/2009(H1N1)
HA	CY046971	H1N1	05/01/2009	USA	A/New York/3443/2009(H1N1)
HA	GQ231987	H1N1	05/01/2009	USA	A/Missouri/02/2009(H1N1)
HA	CY064716	H1N1	05/01/2009	Mexico	A/Mexico City/021/2009
HA	CY041637	H1N1	05/01/2009	USA	A/New York/3307/2009(H1N1)
HA	CY053079	H1N1	05/01/2009	USA	A/Houston/140S/2009H1N1)
HA	CY046459	H1N1	05/01/2009	USA	A/Wisconsin/629-D01861/2009(H1N1)
HA	CY046187	H1N1	05/01/2009	USA	A/Wisconsin/629-D01793/2009(H1N1)
HA	CY073378	H1N1	05/01/2009	USA	A/San Diego/WR1633P/2009(H1N1)
HA	CY065334	H1N1	05/02/2009	United Ki	A/England/201/2009
HA	CY046339	H1N1	05/02/2009	USA	A/Wisconsin/629-D02455/2009(H1N1)
HA	CY046195	H1N1	05/02/2009	USA	A/Wisconsin/629-D02312/2009(H1N1)
HA	CY046379	H1N1	05/02/2009	USA	A/Wisconsin/629-D01980/2009(H1N1)
HA	CY046235	H1N1	05/02/2009	USA	A/Wisconsin/629-D01735/2009(H1N1)
HA	CY046395	H1N1	05/02/2009	USA	A/Wisconsin/629-D01189/2009(H1N1)
HA	CY046435	H1N1	05/02/2009	USA	A/Wisconsin/629-D02262/2009(H1N1)

HA	GQ894905	H1N1	05/02/2009	USA	A/Texas/29/2009(H1N1)
HA	CY041074	H1N1	05/02/2009	USA	A/New York/3170/2009(H1N1)
HA	CY041597	H1N1	05/02/2009	USA	A/New York/3177/2009(H1N1)
HA	CY041806	H1N1	05/02/2009	USA	A/New York/3408/2009(H1N1)
HA	CY064756	H1N1	05/02/2009	Mexico	A/Mexico City/026/2009(H1N1)
HA	CY069781	H1N1	05/02/2009	United Ki	A/England/221/2009
HA	CY064724	H1N1	05/02/2009	Mexico	A/Mexico City/022/2009
HA	CY073386	H1N1	05/02/2009	USA	A/San Diego/WR1634P/2009(H1N1)
HA	CY049867	H1N1	05/02/2009	USA	A/Cherry Point/WR0101/2009(H1N1)
HA	HM567568	H1N1	05/02/2009	United Ki	A/England/221/2009
HA	CY065302	H1N1	05/02/2009	United Ki	A/England/218/2009
HA	CY065310	H1N1	05/02/2009	United Ki	A/England/246/2009
HA	CY049875	H1N1	05/02/2009	USA	A/Cherry Point/WR0102/2009(H1N1)
HA	CY065318	H1N1	05/02/2009	United Ki	A/England/219/2009
HA	CY065326	H1N1	05/02/2009	United Ki	A/England/220/2009
HA	CY046851	H1N1	05/02/2009	USA	A/Wisconsin/629-D00244/2009(H1N1)
HA	CY049859	H1N1	05/02/2009	USA	A/Cherry Point/WR0100/2009(H1N1)
HA	CY073426	H1N1	05/02/2009	USA	A/San Diego/WR1641P/2009(H1N1)
HA	GQ131023	H1N1	05/02/2009	South Ko	A/Korea/01/2009
HA	CY046203	H1N1	05/02/2009	USA	A/Wisconsin/629-D02408/2009(H1N1)
HA	CY046267	H1N1	05/02/2009	USA	A/Wisconsin/629-D00933/2009(H1N1)
HA	GQ365666	H1N1	05/02/2009	Germany	A/Brandenburg/19/2009(H1N1)
HA	CY046211	H1N1	05/03/2009	USA	A/Wisconsin/629-D00869/2009(H1N1)
HA	CY046371	H1N1	05/03/2009	USA	A/Wisconsin/629-D01705/2009(H1N1)
HA	CY046227	H1N1	05/03/2009	USA	A/Wisconsin/629-D01296/2009(H1N1)
HA	CY046275	H1N1	05/03/2009	USA	A/Wisconsin/629-D01092/2009(H1N1)
HA	CY069789	H1N1	05/03/2009	United Ki	A/England/222/2009
HA	CY046347	H1N1	05/03/2009	USA	A/Wisconsin/629-D01017/2009(H1N1)
HA	CY065294	H1N1	05/03/2009	United Ki	A/Scotland/4/2009
HA	CY065350	H1N1	05/03/2009	United Ki	A/England/247/2009
HA	CY046283	H1N1	05/03/2009	USA	A/Wisconsin/629-D02473/2009(H1N1)
HA	CY046883	H1N1	05/03/2009	USA	A/Wisconsin/629-D00498/2009(H1N1)
HA	GQ251035	H1N1	05/03/2009	Italy	A/Italy/05/2009(H1N1)
HA	CY046243	H1N1	05/03/2009	USA	A/Wisconsin/629-D01606/2009(H1N1)
HA	CY053150	H1N1	05/04/2009	USA	A/Houston/22H/2009(H1N1)
HA	CY046499	H1N1	05/04/2009	USA	A/Wisconsin/629-D00378/2009(H1N1)
HA	CY046363	H1N1	05/04/2009	USA	A/Wisconsin/629-D00790/2009(H1N1)
HA	CY046219	H1N1	05/04/2009	USA	A/Wisconsin/629-D01251/2009(H1N1)
HA	CY046403	H1N1	05/04/2009	USA	A/Wisconsin/629-D00807/2009(H1N1)
HA	CY046443	H1N1	05/04/2009	USA	A/Wisconsin/629-D01226/2009(H1N1)
HA	CY046915	H1N1	05/04/2009	USA	A/Wisconsin/629-D01764/2009(H1N1)
HA	CY046467	H1N1	05/04/2009	USA	A/Wisconsin/629-D01962/2009(H1N1)
HA	CY046483	H1N1	05/04/2009	USA	A/Wisconsin/629-D00349/2009(H1N1)
HA	GQ232070	H1N1	05/04/2009	USA	A/Missouri/03/2009(H1N1)
HA	CY043203	H1N1	05/04/2009	USA	A/New York/3338/2009(H1N1)
HA	GQ232007	H1N1	05/04/2009	USA	A/Vermont/03/2009(H1N1)
HA	CY041645	H1N1	05/04/2009	USA	A/New York/3313/2009(H1N1)
HA	CY049843	H1N1	05/04/2009	USA	A/Craven/WR0093/2009(H1N1)
HA	GQ323464	H1N1	05/04/2009	USA	A/Kentucky/06/2009(H1N1)
HA	CY041782	H1N1	05/04/2009	USA	A/New York/3337/2009(H1N1)
HA	GQ365674	H1N1	05/04/2009	Germany	A/Brandenburg/20/2009(H1N1)
HA	GQ377043	H1N1	05/04/2009	USA	A/Montana/07/2009(H1N1)
HA	GQ232073	H1N1	05/04/2009	USA	A/Wisconsin/10/2009(H1N1)
HA	KC781882	H1N1	05/04/2009	USA	A/Texas/45/2009

HA	CY046859	H1N1	05/04/2009	USA	A/Wisconsin/629-D01415/2009(H1N1)
HA	GQ323470	H1N1	05/04/2009	USA	A/Kentucky/07/2009(H1N1)
HA	CY069821	H1N1	05/04/2009	United Ki	A/England/250/2009
HA	CY053142	H1N1	05/04/2009	USA	A/Houston/220S/2009(H1N1)
HA	CY069805	H1N1	05/04/2009	United Ki	A/England/XFL00230/2009
HA	CY045482	H1N1	05/04/2009	Germany	A/Sachsen-Anhalt/101/2009(H1N1)
HA	CY046251	H1N1	05/04/2009	USA	A/Wisconsin/629-D02432/2009(H1N1)
HA	CY050142	H1N1	05/04/2009	USA	A/Wisconsin/629-D01058/2009(H1N1)
HA	GQ232035	H1N1	05/04/2009	USA	A/Wisconsin/11/2009(H1N1)
HA	CY065358	H1N1	05/04/2009	United Ki	A/England/XFL00200/2009
HA	GQ402196	H1N1	05/04/2009	Canada	A/Canada-SK/RV1767/2009(H1N1)
HA	CY046523	H1N1	05/04/2009	USA	A/Wisconsin/629-D00183/2009(H1N1)
HA	CY046507	H1N1	05/04/2009	USA	A/Wisconsin/629-D02425/2009(H1N1)
HA	CY046875	H1N1	05/04/2009	USA	A/Wisconsin/629-D00396/2009(H1N1)
HA	CY043171	H1N1	05/04/2009	USA	A/New York/3460/2009(H1N1)
HA	CY046867	H1N1	05/04/2009	USA	A/Wisconsin/629-D02272/2009(H1N1)
HA	CY046531	H1N1	05/04/2009	USA	A/Wisconsin/629-D00714/2009(H1N1)
HA	CY046323	H1N1	05/04/2009	USA	A/Wisconsin/629-D01810/2009(H1N1)
HA	CY046891	H1N1	05/04/2009	USA	A/Wisconsin/629-D01880/2009(H1N1)
HA	CY049820	H1N1	05/05/2009	USA	A/Craven/WR0019/2009(H1N1)
HA	GQ457511	H1N1	05/05/2009	USA	A/Montana/06/2009(H1N1)
HA	GQ323520	H1N1	05/05/2009	USA	A/Michigan/05/2009(H1N1)
HA	CY069813	H1N1	05/05/2009	United Ki	A/England/249/2009
HA	CY069829	H1N1	05/05/2009	United Ki	A/England/XFL00303/2009
HA	CY065414	H1N1	05/05/2009	United Ki	A/England/254/2009
HA	CY065382	H1N1	05/05/2009	United Ki	A/England/XFL00304/2009
HA	CY069797	H1N1	05/05/2009	United Ki	A/England/XFL00228/2009
HA	CY065366	H1N1	05/05/2009	United Ki	A/England/XFL00275/2009
HA	GQ457501	H1N1	05/05/2009	USA	A/Texas/30/2009(H1N1)
HA	GQ231993	H1N1	05/05/2009	USA	A/Ohio/09/2009(H1N1)
HA	GQ231990	H1N1	05/05/2009	USA	A/South Dakota/04/2009(H1N1)
HA	HM567576	H1N1	05/05/2009	United Ki	A/England/249/2009
HA	GQ169382	H1N1	05/06/2009	Thailand	A/Thailand/104/2009(H1N1)
HA	CY041750	H1N1	05/06/2009	USA	A/New York/3323/2009(H1N1)
HA	GQ329088	H1N1	05/06/2009	France	A/Strasbourg/2611/2009(H1N1)
HA	GQ329106	H1N1	05/06/2009	France	A/Strasbourg/2609/2009(H1N1)
HA	CY041822	H1N1	05/06/2009	USA	A/New York/3352/2009(H1N1)
HA	GQ402198	H1N1	05/06/2009	Canada	A/Canada-SK/RV1798/2009(H1N1)
HA	CY041814	H1N1	05/06/2009	USA	A/New York/3351/2009(H1N1)
HA	CY045490	H1N1	05/06/2009	Germany	A/Sachsen-Anhalt/97/2009(H1N1)
HA	CY046555	H1N1	05/06/2009	USA	A/Wisconsin/629-D00228/2009(H1N1)
HA	CY043195	H1N1	05/06/2009	USA	A/New York/3324/2009(H1N1)
HA	CY047334	H1N1	05/06/2009	USA	A/New York/3364/2009(H1N1)
HA	GQ377064	H1N1	05/06/2009	USA	A/Virginia/05/2009(H1N1)
HA	CY046603	H1N1	05/07/2009	USA	A/Wisconsin/629-D00487/2009(H1N1)
HA	GQ402195	H1N1	05/07/2009	Canada	A/Canada-QC/RV1759/2009(H1N1)
HA	GQ323509	H1N1	05/07/2009	USA	A/Washington/14/2009(H1N1)
HA	GQ465683	H1N1	05/07/2009	Canada	A/Canada-SK/RV1797/2009(H1N1)
HA	GQ323551	H1N1	05/07/2009	USA	A/Massachusetts/08/2009(H1N1)
HA	GQ323495	H1N1	05/07/2009	USA	A/Arkansas/03/2009(H1N1)
HA	CY070672	H1N1	05/07/2009	United Ki	A/England/251/2009
HA	CY065374	H1N1	05/07/2009	United Ki	A/England/XFL00300/2009
HA	CY100448	H1N1	05/07/2009	Mexico	A/Mexico City/IBT22/2009
HA	CY065406	H1N1	05/07/2009	United Ki	A/England/253/2009

HA	CY050198	H1N1	05/07/2009	Mexico	A/Mexico City/001/2009
HA	GQ465682	H1N1	05/07/2009	Canada	A/Canada-SK/RV1794/2009(H1N1)
HA	CY065398	H1N1	05/07/2009	United Ki	A/England/XFL00311/2009
HA	CY100456	H1N1	05/07/2009	Mexico	A/Mexico City/IBT23/2009
HA	GQ338361	H1N1	05/07/2009	USA	A/Wisconsin/12/2009(H1N1)
HA	CY065430	H1N1	05/08/2009	United Ki	A/England/256/2009
HA	CY046539	H1N1	05/08/2009	USA	A/Wisconsin/629-D01313/2009(H1N1)
HA	CY046579	H1N1	05/08/2009	USA	A/Wisconsin/629-D01909/2009(H1N1)
HA	CY043123	H1N1	05/08/2009	USA	A/New York/3389/2009(H1N1)
HA	GQ227545	H1N1	05/08/2009	Sweden	A/Stockholm/29/2009(H1N1)
HA	GQ465680	H1N1	05/08/2009	Canada	A/Canada-PQ/RV1758/2009(H1N1)
HA	CY041766	H1N1	05/08/2009	USA	A/New York/3354/2009(H1N1)
HA	GQ165815	H1N1	05/08/2009	Japan	A/Narita/1/2009
HA	GQ165814	H1N1	05/08/2009	Japan	A/Narita/1/2009
HA	GQ338405	H1N1	05/08/2009	USA	A/New York/57/2009(H1N1)
HA	CY070673	H1N1	05/08/2009	United Ki	A/England/255/2009
HA	CY069837	H1N1	05/08/2009	United Ki	A/England/XFL00364/2009
HA	CY050214	H1N1	05/08/2009	Mexico	A/Mexico City/003/2009(H1N1)
HA	CY050206	H1N1	05/08/2009	Mexico	A/Mexico City/002/2009(H1N1)
HA	CY046587	H1N1	05/08/2009	USA	A/Wisconsin/629-D02452/2009(H1N1)
HA	CY050222	H1N1	05/08/2009	Mexico	A/Mexico City/004/2009(H1N1)
HA	CY050879	H1N1	05/08/2009	Mexico	A/Mexico City/005/2009(H1N1)
HA	CY065390	H1N1	05/08/2009	United Ki	A/England/252/2009
HA	GQ402197	H1N1	05/08/2009	Canada	A/Canada-SK/RV1793/2009(H1N1)
HA	CY046571	H1N1	05/08/2009	USA	A/Wisconsin/629-D00905/2009(H1N1)
HA	GQ323446	H1N1	05/09/2009	USA	A/Maine/03/2009(H1N1)
HA	CY046611	H1N1	05/09/2009	USA	A/Wisconsin/629-D01055/2009(H1N1)
HA	CY070674	H1N1	05/09/2009	United Ki	A/England/258/2009
HA	CY050254	H1N1	05/09/2009	Mexico	A/Mexico City/012/2009
HA	CY050246	H1N1	05/09/2009	Mexico	A/Mexico City/011/2009
HA	CY050871	H1N1	05/09/2009	Mexico	A/Mexico City/017/2009
HA	CY070676	H1N1	05/09/2009	United Ki	A/England/262/2009
HA	CY045946	H1N1	05/09/2009	Canada	A/Toronto/C2781/2009(H1N1)
HA	CY050863	H1N1	05/09/2009	Mexico	A/Mexico City/013/2009
HA	CY092101	H1N1	05/09/2009	Mexico	A/Mexico City/016/2009
HA	CY062635	H1N1	05/09/2009	Mexico	A/Mexico City/014/2009
HA	CY069845	H1N1	05/09/2009	United Ki	A/England/XFL00376/2009
HA	CY050230	H1N1	05/09/2009	Mexico	A/Mexico City/006/2009(H1N1)
HA	CY050174	H1N1	05/09/2009	Mexico	A/Mexico City/015/2009
HA	CY046563	H1N1	05/09/2009	USA	A/Wisconsin/629-D01919/2009(H1N1)
HA	CY050847	H1N1	05/09/2009	Mexico	A/Mexico City/007/2009(H1N1)
HA	CY065438	H1N1	05/09/2009	United Ki	A/England/257/2009
HA	CY050238	H1N1	05/09/2009	Mexico	A/Mexico City/010/2009
HA	CY046595	H1N1	05/10/2009	USA	A/Wisconsin/629-D01482/2009(H1N1)
HA	GQ166223	H1N1	05/10/2009	China	A/Sichuan/1/2009(H1N1)
HA	CY046547	H1N1	05/10/2009	USA	A/Wisconsin/629-D00179/2009(H1N1)
HA	GQ183633	H1N1	05/10/2009	Finland	A/Finland/553/2009(H1N1)
HA	CY050166	H1N1	05/10/2009	Mexico	A/Mexico City/008/2009(H1N1)
HA	CY065422	H1N1	05/10/2009	United Ki	A/England/XFL00354/2009
HA	GQ200287	H1N1	05/10/2009	China	A/Shandong/1/2009(H1N1)
HA	CY050887	H1N1	05/10/2009	Mexico	A/Mexico City/018/2009
HA	JQ173156	H1N1	05/10/2009	Finland	A/Helsinki/P15/2009
HA	CY050444	H1N1	05/10/2009	USA	A/Wisconsin/629-D01482/2009(H1N1)
HA	GQ323455	H1N1	05/11/2009	USA	A/Texas/32/2009(H1N1)

HA	CY041758	H1N1	05/11/2009	USA	A/New York/3413/2009(H1N1)
HA	CY065446	H1N1	05/11/2009	United Ki	A/England/259/2009
HA	CY069853	H1N1	05/11/2009	United Ki	A/England/260/2009
HA	CY070675	H1N1	05/11/2009	United Ki	A/England/261/2009
HA	GQ249337	H1N1	05/11/2009	France	A/Paris/2629/2009(H1N1)
HA	CY065454	H1N1	05/11/2009	United Ki	A/England/XFL00418/2009
HA	CY043163	H1N1	05/11/2009	USA	A/New York/3454/2009(H1N1)
HA	CY043219	H1N1	05/11/2009	USA	A/New York/3455/2009(H1N1)
HA	GQ232060	H1N1	05/11/2009	USA	A/North Dakota/04/2009(H1N1)
HA	CY050855	H1N1	05/12/2009	Mexico	A/Mexico City/009/2009(H1N1)
HA	CY043155	H1N1	05/12/2009	USA	A/New York/3418/2009(H1N1)
HA	GQ323576	H1N1	05/12/2009	USA	A/Texas/34/2009(H1N1)
HA	CY065462	H1N1	05/12/2009	United Ki	A/England/XFL00419/2009
HA	CY043179	H1N1	05/12/2009	USA	A/New York/3488/2009(H1N1)
HA	CY043299	H1N1	05/12/2009	USA	A/New York/3498/2009(H1N1)
HA	CY065470	H1N1	05/12/2009	United Ki	A/England/XFL00473/2009
HA	CY065486	H1N1	05/12/2009	United Ki	A/England/264/2009
HA	CY065478	H1N1	05/12/2009	United Ki	A/England/263/2009
HA	CY070677	H1N1	05/12/2009	United Ki	A/England/XFL00475/2009
HA	CY069861	H1N1	05/12/2009	United Ki	A/England/XFL00421/2009
HA	GQ323574	H1N1	05/12/2009	USA	A/Texas/31/2009(H1N1)
HA	CY050328	H1N1	05/12/2009	USA	A/Wisconsin/629-D01851/2009(H1N1)
HA	CY065494	H1N1	05/12/2009	United Ki	A/England/265/2009
HA	CY065510	H1N1	05/13/2009	United Ki	A/England/XFL00482/2009
HA	CY050372	H1N1	05/13/2009	USA	A/Wisconsin/629-D01154/2009(H1N1)
HA	CY052959	H1N1	05/13/2009	USA	A/Houston/1H/2009(H1N1)
HA	KC781320	H1N1	05/13/2009	USA	A/Mississippi/01/2009
HA	CY065502	H1N1	05/13/2009	United Ki	A/England/XFL00481/2009
HA	CY051903	H1N1	05/13/2009	USA	A/Houston/10S/2009(H1N1)
HA	CY053063	H1N1	05/13/2009	USA	A/Houston/10H/2009(H1N1)
HA	CY053055	H1N1	05/13/2009	USA	A/Houston/100S/2009(H1N1)
HA	CY046675	H1N1	05/13/2009	USA	A/Wisconsin/629-D01305/2009(H1N1)
HA	CY043211	H1N1	05/13/2009	USA	A/New York/3420/2009(H1N1)
HA	CY043283	H1N1	05/13/2009	USA	A/New York/3449/2009(H1N1)
HA	CY046651	H1N1	05/14/2009	USA	A/Wisconsin/629-D01837/2009(H1N1)
HA	CY046667	H1N1	05/14/2009	USA	A/Wisconsin/629-D00064/2009(H1N1)
HA	CY046659	H1N1	05/14/2009	USA	A/Wisconsin/629-D00997/2009(H1N1)
HA	CY046683	H1N1	05/14/2009	USA	A/Wisconsin/629-D00978/2009(H1N1)
HA	CY041790	H1N1	05/14/2009	USA	A/New York/3463/2009(H1N1)
HA	CY043131	H1N1	05/14/2009	USA	A/New York/3514/2009(H1N1)
HA	CY050380	H1N1	05/15/2009	USA	A/Wisconsin/629-D01295/2009(H1N1)
HA	CY050388	H1N1	05/15/2009	USA	A/Wisconsin/629-D01839/2009(H1N1)
HA	CY065518	H1N1	05/15/2009	United Ki	A/England/266/2009
HA	GQ225373	H1N1	05/15/2009	China	A/Beijing/01/2009(H1N1)
HA	CY065526	H1N1	05/15/2009	United Ki	A/England/XFL00532/2009
HA	CY048925	H1N1	05/15/2009	Malaysia	A/Malaysia/820/2009(H1N1)
HA	CY044973	H1N1	05/15/2009	USA	A/New York/3744/2009(H1N1)
HA	CY046627	H1N1	05/15/2009	USA	A/Wisconsin/629-D01839/2009(H1N1)
HA	CY083296	H1N1	05/15/2009	USA	A/San Diego/WRAIR1647P/2009
HA	CY043187	H1N1	05/16/2009	USA	A/New York/3499/2009(H1N1)
HA	CY043251	H1N1	05/16/2009	USA	A/New York/3545/2009(H1N1)
HA	CY043235	H1N1	05/16/2009	USA	A/New York/3502/2009(H1N1)
HA	CY041774	H1N1	05/16/2009	USA	A/New York/3532/2009(H1N1)
HA	CY044981	H1N1	05/16/2009	USA	A/New York/3747/2009(H1N1)

HA	CY046691	H1N1	05/16/2009	USA	A/Wisconsin/629-D00434/2009(H1N1)
HA	CY043243	H1N1	05/16/2009	USA	A/New York/3509/2009(H1N1)
HA	GQ219580	H1N1	05/16/2009	Japan	A/Osaka-C/1/2009(H1N1)
HA	GQ219578	H1N1	05/16/2009	Japan	A/Osaka/1/2009(H1N1)
HA	GQ183617	H1N1	05/16/2009	China	A/Beijing/01/2009(H1N1)
HA	CY046635	H1N1	05/16/2009	USA	A/Wisconsin/629-D00967/2009(H1N1)
HA	CY046643	H1N1	05/16/2009	USA	A/Wisconsin/629-D02227/2009(H1N1)
HA	CY046619	H1N1	05/16/2009	USA	A/Wisconsin/629-D00575/2009(H1N1)
HA	CY043291	H1N1	05/16/2009	USA	A/New York/3469/2009(H1N1)
HA	CY070678	H1N1	05/16/2009	United Ki	A/England/267/2009
HA	GQ219579	H1N1	05/16/2009	Japan	A/Osaka/2/2009(H1N1)
HA	CY048933	H1N1	05/16/2009	Malaysia	A/Malaysia/854/2009(H1N1)
HA	CY051984	H1N1	05/16/2009	Norway	A/Norway/1359/2009(H1N1)
HA	CY062540	H1N1	05/16/2009	Mexico	A/Mexico city/CIA9/2009(H1N1)
HA	GQ183625	H1N1	05/16/2009	China	A/Beijing/02/2009(H1N1)
HA	CY045085	H1N1	05/17/2009	USA	A/New York/3574/2009(H1N1)
HA	CY043227	H1N1	05/17/2009	USA	A/New York/3501/2009(H1N1)
HA	CY044128	H1N1	05/17/2009	USA	A/New York/3516/2009(H1N1)
HA	CY083288	H1N1	05/17/2009	USA	A/San Diego/WRAIR1646P/2009
HA	CY043307	H1N1	05/17/2009	USA	A/New York/3534/2009(H1N1)
HA	GQ221694	H1N1	05/17/2009	China	A/Guangdong/1/2009(H1N1)
HA	GQ219581	H1N1	05/17/2009	Japan	A/Osaka-C/2/2009(H1N1)
HA	GQ402199	H1N1	05/17/2009	Canada	A/Canada-QC/RV1954/2009(H1N1)
HA	GQ219576	H1N1	05/17/2009	Japan	A/Hyogo/2/2009(H1N1)
HA	GQ219575	H1N1	05/17/2009	Japan	A/Hyogo/1/2009(H1N1)
HA	CY070286	H1N1	05/17/2009	United Ki	A/England/93000102/2009
HA	CY069869	H1N1	05/17/2009	United Ki	A/England/271/2009
HA	GQ329100	H1N1	05/17/2009	France	A/Paris/2650/2009(H1N1)
HA	CY041798	H1N1	05/17/2009	USA	A/New York/3468/2009(H1N1)
HA	CY050396	H1N1	05/17/2009	USA	A/Wisconsin/629-D00989/2009(H1N1)
HA	CY052967	H1N1	05/18/2009	USA	A/Houston/20S/2009(H1N1)
HA	CY044909	H1N1	05/18/2009	USA	A/New York/3612/2009(H1N1)
HA	GQ377069	H1N1	05/18/2009	USA	A/New York/59/2009(H1N1)
HA	GQ223440	H1N1	05/18/2009	China	A/Guangdong/1/2009(H1N1)
HA	GQ261272	H1N1	05/18/2009	Japan	A/Himeji/1/2009(H1N1)
HA	GQ268003	H1N1	05/18/2009	China	A/GuangzhouSB/01/2009(H1N1)
HA	CY052991	H1N1	05/18/2009	USA	A/Houston/3H/2009(H1N1)
HA	HQ011408	H1N1	05/18/2009	China	A/Guangdong/01/2009(H1N1)
HA	CY044088	H1N1	05/18/2009	USA	A/New York/3740/2009(H1N1)
HA	CY051911	H1N1	05/18/2009	USA	A/Houston/2H/2009(H1N1)
HA	CY052983	H1N1	05/18/2009	USA	A/Houston/30S/2009(H1N1)
HA	HM569702	H1N1	05/18/2009	Argentina	A/Argentina/19659/2009(H1N1)
HA	JN171869	H1N1	05/18/2009	Canada	A/Quebec/QC144876/2009
HA	GQ287621	H1N1	05/19/2009	Japan	A/Shiga/2/2009(H1N1)
HA	CY083312	H1N1	05/19/2009	USA	A/San Diego/WRAIR1649P/2009
HA	CY050412	H1N1	05/19/2009	USA	A/Wisconsin/629-D00752/2009(H1N1)
HA	CY052999	H1N1	05/19/2009	USA	A/Houston/50S/2009(H1N1)
HA	GQ323489	H1N1	05/19/2009	USA	A/Arizona/09/2009(H1N1)
HA	GQ323486	H1N1	05/19/2009	USA	A/Arizona/09/2009(H1N1)
HA	CY043267	H1N1	05/19/2009	USA	A/New York/3563/2009(H1N1)
HA	CY046771	H1N1	05/19/2009	USA	A/Wisconsin/629-D00722/2009(H1N1)
HA	CY053007	H1N1	05/19/2009	USA	A/Houston/5H/2009(H1N1)
HA	CY044136	H1N1	05/19/2009	USA	A/New York/3528/2009(H1N1)
HA	CY044196	H1N1	05/19/2009	Taiwan	A/Taiwan/T0724/2009(H1N1)

HA	CY047358	H1N1	05/19/2009	USA	A/New York/3741/2009(H1N1)
HA	CY044064	H1N1	05/19/2009	USA	A/New York/3568/2009(H1N1)
HA	CY043139	H1N1	05/19/2009	USA	A/New York/3529/2009(H1N1)
HA	CY044917	H1N1	05/19/2009	USA	A/New York/3613/2009(H1N1)
HA	CY044885	H1N1	05/19/2009	USA	A/New York/3580/2009(H1N1)
HA	CY046755	H1N1	05/19/2009	USA	A/Wisconsin/629-D00160/2009(H1N1)
HA	CY046739	H1N1	05/19/2009	USA	A/Wisconsin/629-D00084/2009(H1N1)
HA	CY046715	H1N1	05/19/2009	USA	A/Wisconsin/629-D00752/2009(H1N1)
HA	CY045143	H1N1	05/19/2009	USA	A/New York/3577/2009(H1N1)
HA	GQ402200	H1N1	05/20/2009	Canada	A/Canada-MB/RV1964/2009(H1N1)
HA	GQ290106	H1N1	05/20/2009	China	A/Beijing/502/2009(H1N1)
HA	GQ414764	H1N1	05/20/2009	Brazil	A/Sao Paulo/2056/2009
HA	CY044048	H1N1	05/20/2009	USA	A/New York/3566/2009(H1N1)
HA	CY046707	H1N1	05/20/2009	USA	A/Wisconsin/629-D01787/2009(H1N1)
HA	CY046723	H1N1	05/20/2009	USA	A/Wisconsin/629-D02427/2009(H1N1)
HA	CY046731	H1N1	05/20/2009	USA	A/Wisconsin/629-D01521/2009(H1N1)
HA	CY044056	H1N1	05/20/2009	USA	A/New York/3567/2009(H1N1)
HA	CY044893	H1N1	05/20/2009	USA	A/New York/3582/2009(H1N1)
HA	CY053174	H1N1	05/20/2009	USA	A/Brownsville/260S/2009(H1N1)
HA	JN171909	H1N1	05/20/2009	Canada	A/Montreal/MTL120171/2009
HA	CY053015	H1N1	05/20/2009	USA	A/Houston/60S/2009(H1N1)
HA	CY044933	H1N1	05/20/2009	USA	A/New York/3623/2009(H1N1)
HA	CY044869	H1N1	05/20/2009	USA	A/New York/3571/2009(H1N1)
HA	CY043259	H1N1	05/20/2009	USA	A/New York/3552/2009(H1N1)
HA	GQ225381	H1N1	05/20/2009	China	A/Beijing/3/2009(H1N1)
HA	CY043147	H1N1	05/20/2009	USA	A/New York/3551/2009(H1N1)
HA	CY053023	H1N1	05/20/2009	USA	A/Houston/6H/2009(H1N1)
HA	CY053182	H1N1	05/20/2009	USA	A/Brownsville/270S/2009(H1N1)
HA	CY044965	H1N1	05/21/2009	USA	A/New York/3653/2009(H1N1)
HA	CY070679	H1N1	05/21/2009	United Ki	A/England/XFL00651/2009
HA	CY052975	H1N1	05/21/2009	USA	A/Houston/20S/2009(H1N1)
HA	CY089872	H1N1	05/21/2009	USA	A/Wisconsin/629-D02275/2009(H1N1)
HA	GQ267839	H1N1	05/21/2009	Japan	A/Sakai/1/2009(H1N1)
HA	GQ402203	H1N1	05/21/2009	Canada	A/Canada-MB/RV1982/2009(H1N1)
HA	GQ261275	H1N1	05/21/2009	Japan	A/Sakai/2/2009(H1N1)
HA	GQ219586	H1N1	05/21/2009	Russia	A/Moscow/IIV01/2009(H1N1)
HA	AB514227	H1N1	05/21/2009	Japan	A/Saitama/43/2009(H1N1)
HA	CY044877	H1N1	05/21/2009	USA	A/New York/3573/2009(H1N1)
HA	CY053039	H1N1	05/21/2009	USA	A/Houston/7H/2009(H1N1)
HA	CY053198	H1N1	05/21/2009	USA	A/Brownsville/300S/2009(H1N1)
HA	CY053190	H1N1	05/21/2009	USA	A/Brownsville/290S/2009(H1N1)
HA	CY070680	H1N1	05/21/2009	United Ki	A/England/273/2009
HA	CY044901	H1N1	05/21/2009	USA	A/New York/3611/2009(H1N1)
HA	CY053047	H1N1	05/21/2009	USA	A/Houston/9H/2009(H1N1)
HA	CY051919	H1N1	05/21/2009	USA	A/Houston/90S/2009(H1N1)
HA	GQ232099	H1N1	05/21/2009	Italy	A/Milan/UHSR1/2009(H1N1)
HA	GQ184630	H1N1	05/21/2009	Russia	A/Moscow/01/2009(H1N1)
HA	CY046963	H1N1	05/21/2009	USA	A/New York/3576/2009(H1N1)
HA	CY053031	H1N1	05/21/2009	USA	A/Houston/70S/2009(H1N1)
HA	GQ377047	H1N1	05/22/2009	USA	A/Texas/39/2009(H1N1)
HA	CY053214	H1N1	05/22/2009	USA	A/Brownsville/31H/2009(H1N1)
HA	CY046763	H1N1	05/22/2009	USA	A/Wisconsin/629-D02298/2009(H1N1)
HA	GQ255897	H1N1	05/22/2009	Russia	A/Kaluga/01/2009(H1N1)
HA	GQ329082	H1N1	05/22/2009	France	A/Paris/2670/2009(H1N1)

HA	CY044204	H1N1	05/22/2009	Taiwan	A/Taiwan/T1338/2009(H1N1)
HA	GQ261277	H1N1	05/22/2009	Japan	A/Shiga/1/2009(H1N1)
HA	CY053206	H1N1	05/22/2009	USA	A/Brownsville/31OS/2009(H1N1)
HA	CY089179	H1N1	05/22/2009	Chile	A/Chile/19/2009
HA	CY045093	H1N1	05/22/2009	USA	A/New York/3614/2009(H1N1)
HA	CY044949	H1N1	05/22/2009	USA	A/New York/3627/2009(H1N1)
HA	CY046747	H1N1	05/22/2009	USA	A/Wisconsin/629-D02064/2009(H1N1)
HA	CY044212	H1N1	05/22/2009	Taiwan	A/Taiwan/T1339/2009(H1N1)
HA	GQ225365	H1N1	05/22/2009	China	A/Fujian/1/2009(H1N1)
HA	GQ402202	H1N1	05/22/2009	Canada	A/Canada-MB/RV1977/2009(H1N1)
HA	CY049907	H1N1	05/23/2009	Dominicar	A/Santo Domingo/0574/2009(H1N1)
HA	GQ338335	H1N1	05/23/2009	USA	A/New York/58/2009(H1N1)
HA	CY053127	H1N1	05/23/2009	USA	A/Houston/20OS/2009(H1N1)
HA	GQ225357	H1N1	05/23/2009	China	A/Shanghai/1/2009(H1N1)
HA	GQ232093	H1N1	05/23/2009	China	A/Beijing/4/2009(H1N1)
HA	GQ329066	H1N1	05/23/2009	France	A/Rennes/2671/2009(H1N1)
HA	GQ323530	H1N1	05/23/2009	USA	A/Illinois/04/2009(H1N1)
HA	CY070681	H1N1	05/23/2009	United Ki	A/England/278/2009
HA	CY075083	H1N1	05/23/2009	Chile	A/Chile/28/2009(H1N1)
HA	CY053135	H1N1	05/23/2009	USA	A/Houston/20H/2009(H1N1)
HA	CY070682	H1N1	05/23/2009	United Ki	A/England/279/2009
HA	CY070684	H1N1	05/23/2009	United Ki	A/England/288/2009
HA	CY070683	H1N1	05/23/2009	United Ki	A/England/285/2009
HA	CY049915	H1N1	05/23/2009	Dominicar	A/Santo Domingo/0574T/2009(H1N1)
HA	CY053222	H1N1	05/23/2009	USA	A/Brownsville/34OS/2009(H1N1)
HA	CY053230	H1N1	05/23/2009	USA	A/Brownsville/34H/2009(H1N1)
HA	CY050420	H1N1	05/23/2009	USA	A/Wisconsin/629-D02206/2009(H1N1)
HA	GQ225349	H1N1	05/23/2009	China	A/Zhejiang/1/2009(H1N1)
HA	CY050404	H1N1	05/23/2009	USA	A/Wisconsin/629-D00451/2009(H1N1)
HA	CY087294	H1N1	05/24/2009	Hong Kong	A/Hong Kong/419301/2009
HA	CY070711	H1N1	05/24/2009	United Ki	A/England/368/2009
HA	CY041960	H1N1	05/24/2009	Dominicar	A/Santo Domingo/572N/2009(H1N1)
HA	CY070689	H1N1	05/24/2009	United Ki	A/England/293/2009
HA	CY044957	H1N1	05/24/2009	USA	A/New York/3629/2009(H1N1)
HA	CY070690	H1N1	05/24/2009	United Ki	A/England/300/2009
HA	CY087296	H1N1	05/24/2009	Hong Kong	A/Hong Kong/419305/2009
HA	CY070687	H1N1	05/24/2009	United Ki	A/England/291/2009
HA	CY070685	H1N1	05/24/2009	United Ki	A/England/289/2009
HA	CY070688	H1N1	05/24/2009	United Ki	A/England/292/2009
HA	CY070686	H1N1	05/24/2009	United Ki	A/England/290/2009
HA	HM567592	H1N1	05/24/2009	Ireland	A/Ireland/1/2009
HA	CY045938	H1N1	05/24/2009	Canada	A/Toronto/C0270/2009(H1N1)
HA	GQ402201	H1N1	05/24/2009	Canada	A/Canada-MB/RV1975/2009(H1N1)
HA	GQ402187	H1N1	05/24/2009	Canada	A/Canada-MB/RV2023/2009(H1N1)
HA	CY046699	H1N1	05/24/2009	USA	A/Wisconsin/629-D01445/2009(H1N1)
HA	GQ253492	H1N1	05/24/2009	China	A/Shanghai/37T/2009(H1N1)
HA	GQ323579	H1N1	05/24/2009	USA	A/West Virginia/01/2009(H1N1)
HA	CY041983	H1N1	05/24/2009	Dominicar	A/Santo Domingo/0573N/2009(H1N1)
HA	CY044925	H1N1	05/25/2009	USA	A/New York/3617/2009(H1N1)
HA	CY075123	H1N1	05/25/2009	Chile	A/Chile/42/2009(H1N1)
HA	CY043275	H1N1	05/25/2009	USA	A/New York/3651/2009(H1N1)
HA	CY075099	H1N1	05/25/2009	Chile	A/Chile/32/2009(H1N1)
HA	CY075091	H1N1	05/25/2009	Chile	A/Chile/31/2009
HA	CY070692	H1N1	05/25/2009	United Ki	A/Scotland/6/2009

HA	CY050428	H1N1	05/25/2009	USA	A/Wisconsin/629-D02324/2009(H1N1)
HA	JQ409126	H1N1	05/25/2009	Finland	A/Helsinki/Vi1/2009
HA	CY075107	H1N1	05/25/2009	Chile	A/Chile/36/2009(H1N1)
HA	CY075115	H1N1	05/25/2009	Chile	A/Chile/40/2009(H1N1)
HA	CY044941	H1N1	05/26/2009	USA	A/New York/3625/2009(H1N1)
HA	GQ385300	H1N1	05/26/2009	Canada	A/Toronto/0462/2009(H1N1)
HA	CY075131	H1N1	05/26/2009	Chile	A/Chile/56/2009(H1N1)
HA	GQ375284	H1N1	05/26/2009	Russia	A/Moscow/03/2009(H1N1)
HA	GU014804	H1N1	05/26/2009	Japan	A/Shizuoka-C/97/2009(H1N1)
HA	CY046955	H1N1	05/26/2009	USA	A/New York/3654/2009(H1N1)
HA	CY045151	H1N1	05/26/2009	USA	A/New York/3655/2009(H1N1)
HA	GQ247726	H1N1	05/26/2009	Russia	A/Moscow/IIV02/2009(H1N1)
HA	CY070691	H1N1	05/26/2009	United Ki	A/England/309/2009
HA	HM567832	H1N1	05/26/2009	Ireland	A/Ireland/3/2009
HA	HM567584	H1N1	05/26/2009	United Ki	A/England/303/2009
HA	CY051247	H1N1	05/26/2009	USA	A/Wisconsin/629-S0035/2009(H1N1)
HA	HM567808	H1N1	05/26/2009	Ireland	A/Ireland/2/2009
HA	GQ283488	H1N1	05/26/2009	Finland	A/Finland/554/2009(H1N1)
HA	CY044072	H1N1	05/26/2009	USA	A/New York/3702/2009(H1N1)
HA	GQ255900	H1N1	05/26/2009	Russia	A/Moscow/02/2009(H1N1)
HA	CY043110	H1N1	05/26/2009	Dominicar	A/Santo Domingo/568T/2009(H1N1)
HA	CY046779	H1N1	05/26/2009	USA	A/Wisconsin/629-D01894/2009(H1N1)
HA	HM567600	H1N1	05/26/2009	United Ki	A/England/313/2009
HA	CY043102	H1N1	05/26/2009	Dominicar	A/Santo Domingo/565T/2009(H1N1)
HA	CY049931	H1N1	05/27/2009	USA	A/Craven/WR1001/2009(H1N1)
HA	CY075139	H1N1	05/27/2009	Chile	A/Chile/88/2009(H1N1)
HA	CY075147	H1N1	05/27/2009	Chile	A/Chile/95/2009(H1N1)
HA	CY053901	H1N1	05/27/2009	Argentina	A/Argentina/HNRG14/2009(H1N1)
HA	CY046811	H1N1	05/27/2009	USA	A/Wisconsin/629-D00698/2009(H1N1)
HA	GQ368664	H1N1	05/27/2009	Brazil	A/Sao Paulo/2233/2009
HA	GQ232085	H1N1	05/27/2009	China	A/Guangdong/02/2009(H1N1)
HA	CY051985	H1N1	05/27/2009	Norway	A/Norway/1573/2009(H1N1)
HA	HM567688	H1N1	05/27/2009	United Ki	A/England/350/2009
HA	CY069877	H1N1	05/27/2009	United Ki	A/England/350/2009
HA	CY070712	H1N1	05/27/2009	United Ki	A/England/369/2009
HA	CY076755	H1N1	05/27/2009	Canada	A/Ontario/142358/2009
HA	CY050895	H1N1	05/27/2009	USA	A/Wisconsin/629-D00949/2009(H1N1)
HA	CY053254	H1N1	05/27/2009	USA	A/Brownsville/36H/2009(H1N1)
HA	CY046835	H1N1	05/27/2009	USA	A/Wisconsin/629-D01883/2009(H1N1)
HA	CY053238	H1N1	05/27/2009	USA	A/Brownsville/350S/2009(H1N1)
HA	CY050436	H1N1	05/27/2009	USA	A/Wisconsin/629-D01886/2009(H1N1)
HA	GQ283484	H1N1	05/27/2009	Italy	A/Italy/49/2009(H1N1)
HA	CY046787	H1N1	05/27/2009	USA	A/Wisconsin/629-D02276/2009(H1N1)
HA	CY053246	H1N1	05/27/2009	USA	A/Brownsville/35H/2009(H1N1)
HA	CY045005	H1N1	05/28/2009	USA	A/New York/3709/2009(H1N1)
HA	CY070715	H1N1	05/28/2009	United Ki	A/England/372/2009
HA	CY053261	H1N1	05/28/2009	USA	A/Brownsville/37H/2009(H1N1)
HA	GQ283493	H1N1	05/28/2009	Finland	A/Finland/555/2009(H1N1)
HA	CY049068	H1N1	05/28/2009	Singapore	A/Singapore/ON129/2009(H1N1)
HA	CY075155	H1N1	05/28/2009	Chile	A/Chile/158/2009(H1N1)
HA	GQ368665	H1N1	05/28/2009	Brazil	A/Mato Grosso/2329/2009
HA	CY044220	H1N1	05/28/2009	Taiwan	A/Taiwan/T1773/2009(H1N1)
HA	GQ402204	H1N1	05/28/2009	Canada	A/Canada-MB/RV2013/2009(H1N1)
HA	GQ411908	H1N1	05/28/2009	China	A/Shanghai/60T/2009(H1N1)

HA	CY070696	H1N1	05/28/2009	United Ki	A/England/318/2009
HA	CY070714	H1N1	05/28/2009	United Ki	A/England/371/2009
HA	CY046827	H1N1	05/28/2009	USA	A/Wisconsin/629-D02144/2009(H1N1)
HA	CY053309	H1N1	05/28/2009	USA	A/Brownsville/370S/2009(H1N1)
HA	CY070702	H1N1	05/28/2009	United Ki	A/England/336/2009
HA	CY070713	H1N1	05/28/2009	United Ki	A/England/370/2009
HA	CY070693	H1N1	05/28/2009	United Ki	A/England/314/2009
HA	CY070701	H1N1	05/28/2009	United Ki	A/Scotland/7/2009
HA	CY070699	H1N1	05/28/2009	United Ki	A/England/327/2009
HA	CY070716	H1N1	05/28/2009	United Ki	A/England/373/2009
HA	CY123459	H1N1	05/28/2009	Singapore	A/Singapore/ON129/2009
HA	HM567608	H1N1	05/28/2009	United Ki	A/England/328/2009
HA	CY064791	H1N1	05/28/2009	China	A/Guangdong/SB1/2009(H1N1)
HA	CY050903	H1N1	05/28/2009	USA	A/Wisconsin/629-D01779/2009(H1N1)
HA	CY050911	H1N1	05/28/2009	USA	A/Wisconsin/629-D01262/2009(H1N1)
HA	CY046907	H1N1	05/28/2009	USA	A/Wisconsin/629-D01083/2009(H1N1)
HA	CY046795	H1N1	05/28/2009	USA	A/Wisconsin/629-D01026/2009(H1N1)
HA	CY046899	H1N1	05/28/2009	USA	A/Wisconsin/629-D00829/2009(H1N1)
HA	CY070694	H1N1	05/28/2009	United Ki	A/England/316/2009
HA	CY070698	H1N1	05/28/2009	United Ki	A/England/323/2009
HA	GQ288372	H1N1	05/28/2009	China	A/Fuzhou/01/2009(H1N1)
HA	CY070695	H1N1	05/28/2009	United Ki	A/England/317/2009
HA	CY050150	H1N1	05/28/2009	USA	A/Wisconsin/629-D00117/2009(H1N1)
HA	CY050927	H1N1	05/29/2009	USA	A/Wisconsin/629-D00426/2009(H1N1)
HA	HM567632	H1N1	05/29/2009	United Ki	A/England/342/2009
HA	CY051255	H1N1	05/29/2009	USA	A/Wisconsin/629-S0086/2009(H1N1)
HA	CY075163	H1N1	05/29/2009	Chile	A/Chile/180/2009(H1N1)
HA	GQ414767	H1N1	05/29/2009	Brazil	A/Sao Paulo/2275/2009
HA	GQ414765	H1N1	05/29/2009	Brazil	A/Sao Paulo/2193/2009
HA	GQ402205	H1N1	05/29/2009	Canada	A/Canada-MB/RV2018/2009(H1N1)
HA	GQ377095	H1N1	05/29/2009	USA	A/California/24/2009(H1N1)
HA	GQ223435	H1N1	05/29/2009	China	A/Guangdong/05/2009(H1N1)
HA	GQ414766	H1N1	05/29/2009	Brazil	A/Sao Paulo/2261/2009
HA	GQ250161	H1N1	05/29/2009	China	A/Guangdong/03/2009(H1N1)
HA	GQ359765	H1N1	05/29/2009	Sweden	A/Stockholm/31/2009(H1N1)
HA	HM780470	H1N1	05/29/2009	China	A/Guangdong/06/2009(H1N1)
HA	CY071031	H1N1	05/29/2009	USA	A/New York/NHRC0002/2009(H1N1)
HA	CY070700	H1N1	05/29/2009	United Ki	A/England/333/2009
HA	HM567840	H1N1	05/29/2009	Ireland	A/Ireland/4/2009
HA	CY070709	H1N1	05/29/2009	United Ki	A/England/362/2009
HA	CY070697	H1N1	05/29/2009	United Ki	A/England/320/2009
HA	CY050935	H1N1	05/29/2009	USA	A/Wisconsin/629-D01847/2009(H1N1)
HA	CY046946	H1N1	05/29/2009	Norway	A/Norway/1613/2009(H1N1)
HA	CY044080	H1N1	05/29/2009	USA	A/New York/3715/2009(H1N1)
HA	CY050919	H1N1	05/29/2009	USA	A/Wisconsin/629-D02213/2009(H1N1)
HA	CY046949	H1N1	05/30/2009	Norway	A/Norway/1623/2009(H1N1)
HA	CY046819	H1N1	05/30/2009	USA	A/Wisconsin/629-D02013/2009(H1N1)
HA	CY089880	H1N1	05/30/2009	USA	A/Wisconsin/629-D01829/2009(H1N1)
HA	CY046803	H1N1	05/30/2009	USA	A/Wisconsin/629-D00223/2009(H1N1)
HA	GQ360060	H1N1	05/30/2009	Sweden	A/Stockholm/33/2009(H1N1)
HA	GQ402206	H1N1	05/30/2009	Canada	A/Canada-MB/RV2020/2009(H1N1)
HA	HM567664	H1N1	05/30/2009	United Ki	A/England/347/2009
HA	HM567648	H1N1	05/30/2009	United Ki	A/England/345/2009
HA	CY044228	H1N1	05/30/2009	Taiwan	A/Taiwan/T1821/2009(H1N1)

HA	CY053277	H1N1	05/30/2009	USA	A/Brownsville/39H/2009(H1N1)
HA	HM567680	H1N1	05/30/2009	United Ki	A/England/349/2009
HA	CY055303	H1N1	05/30/2009	Singapore	A/Singapore/GN285/2009(H1N1)
HA	HM567624	H1N1	05/30/2009	United Ki	A/England/341/2009
HA	HM567672	H1N1	05/30/2009	United Ki	A/England/348/2009
HA	HM567640	H1N1	05/30/2009	United Ki	A/England/343/2009
HA	HM567656	H1N1	05/30/2009	United Ki	A/England/346/2009
HA	CY053269	H1N1	05/30/2009	USA	A/Brownsville/390S/2009(H1N1)
HA	CY045962	H1N1	05/30/2009	Canada	A/Toronto/T5294/2009(H1N1)
HA	HM567616	H1N1	05/30/2009	United Ki	A/England/337/2009
HA	HM569659	H1N1	05/30/2009	Argentina	A/Argentina/07-09GP/2009(H1N1)
HA	GQ377107	H1N1	05/31/2009	USA	A/Utah/04/2009(H1N1)
HA	HM567848	H1N1	05/31/2009	Ireland	A/Ireland/5/2009
HA	CY065534	H1N1	05/31/2009	United Ki	A/England/359/2009
HA	HM567720	H1N1	05/31/2009	United Ki	A/England/364/2009
HA	HM567728	H1N1	05/31/2009	United Ki	A/England/365/2009
HA	CY070703	H1N1	05/31/2009	United Ki	A/England/339/2009
HA	CY070708	H1N1	05/31/2009	United Ki	A/England/358/2009
HA	CY070706	H1N1	05/31/2009	United Ki	A/England/353/2009
HA	CY070704	H1N1	05/31/2009	United Ki	A/England/340/2009
HA	HM567712	H1N1	05/31/2009	United Ki	A/Scotland/8/2009
HA	HM567736	H1N1	05/31/2009	United Ki	A/England/366/2009
HA	HQ011423	H1N1	05/31/2009	China	A/Guangdong/55/2009(H1N1)
HA	CY045029	H1N1	05/31/2009	USA	A/New York/3812/2009(H1N1)
HA	CY070707	H1N1	05/31/2009	United Ki	A/England/357/2009
HA	CY070710	H1N1	05/31/2009	United Ki	A/England/363/2009
HA	CY070723	H1N1	05/31/2009	United Ki	A/England/405/2009
HA	GQ253498	H1N1	05/31/2009	China	A/Shanghai/71T/2009(H1N1)
HA	HM567696	H1N1	05/31/2009	United Ki	A/England/351/2009
HA	CY071023	H1N1	05/31/2009	USA	A/New York/NHRC0001/2009(H1N1)
HA	CY044861	H1N1	05/31/2009	USA	A/New York/3726/2009(H1N1)
HA	GQ293077	H1N1	05/31/2009	China	A/Zhejiang/2/2009(H1N1)
HA	CY045183	H1N1	05/31/2009	USA	A/New York/3960/2009(H1N1)
HA	CY069626	H1N1	06/2009	Singapore	A/Singapore/471/2009(H1N1)
HA	CY069634	H1N1	06/2009	Singapore	A/Singapore/478/2009(H1N1)
HA	CY069641	H1N1	06/2009	Singapore	A/Singapore/527/2009(H1N1)
HA	AB568158	H1N1	06/2009	Japan	A/Kobe/91508/2009
HA	AB568163	H1N1	06/2009	Japan	A/Kobe/91524/2009
HA	CY047768	H1N1	06/2009	Argentina	A/Argentina/14332/2009
HA	CY087290	H1N1	06/2009	Hong Kong	A/Hong Kong/33230/2009
HA	HM204567	H1N1	06/2009	India	A/Mum/NIV261/2009
HA	HM204566	H1N1	06/2009	India	A/Delhi/NIV57/2009
HA	KT898916	H1N1	06/2009	Italy	A/Pavia/VR9857/2009
HA	CY047787	H1N1	06/2009	Argentina	A/Argentina/7785/2009
HA	JN381207	H1N1	06/2009	Taiwan	A/Taiwan/1768/2009
HA	JN381203	H1N1	06/2009	Taiwan	A/Taiwan/1753/2009
HA	JN381205	H1N1	06/2009	Taiwan	A/Taiwan/1766/2009
HA	JN381210	H1N1	06/2009	Taiwan	A/Taiwan/97196/2009
HA	JN381211	H1N1	06/2009	Taiwan	A/Taiwan/97264/2009
HA	JN381212	H1N1	06/2009	Taiwan	A/Taiwan/97291/2009
HA	JN381206	H1N1	06/2009	Taiwan	A/Taiwan/1767/2009
HA	JN381204	H1N1	06/2009	Taiwan	A/Taiwan/1765/2009
HA	JN381209	H1N1	06/2009	Taiwan	A/Taiwan/1776/2009
HA	JN381208	H1N1	06/2009	Taiwan	A/Taiwan/1775/2009

HA	CY045226	H1N1	06/2009	Taiwan	A/Taiwan/115/2009
HA	CY045234	H1N1	06/2009	Taiwan	A/Taiwan/126/2009
HA	CY045242	H1N1	06/2009	Taiwan	A/Taiwan/137/2009
HA	CY069619	H1N1	06/2009	Singapore	A/Singapore/276/2009(H1N1)
HA	CY096808	H1N1	06/2009	Thailand	A/Thailand/ICRC-CBI6/2009
HA	CY096807	H1N1	06/2009	Thailand	A/Thailand/ICRC-CBI10/2009
HA	HQ695920	H1N1	06/2009	Taiwan	A/Taiwan/14/2009
HA	GQ359758	H1N1	06/2009	China	A/Zhejiang/DTID-ZJU01/2009(H1N1)
HA	JF429402	H1N1	06/2009	France	A/Lyon/969/2009
HA	HQ104926	H1N1	06/2009	Germany	A/Hamburg/NY1580/2009
HA	CY047774	H1N1	06/2009	Argentina	A/Argentina/18426/2009
HA	GU369647	H1N1	06/2009	Turkey	A/Ankara/04/2009(H1N1)
HA	CY052349	H1N1	06/01/2009	Brazil	A/Santa Catarina/423/2009(H1N1)
HA	CY070717	H1N1	06/01/2009	United Ki	A/England/376/2009
HA	GQ365368	H1N1	06/01/2009	Sweden	A/Stockholm/35/2009(H1N1)
HA	CY044989	H1N1	06/01/2009	USA	A/New York/3751/2009(H1N1)
HA	CY065622	H1N1	06/01/2009	United Ki	A/England/395/2009
HA	CY065550	H1N1	06/01/2009	United Ki	A/England/381/2009
HA	CY069885	H1N1	06/01/2009	United Ki	A/England/383/2009
HA	CY065574	H1N1	06/01/2009	United Ki	A/England/385/2009
HA	CY070721	H1N1	06/01/2009	United Ki	A/England/403/2009
HA	CY070705	H1N1	06/01/2009	United Ki	A/England/352/2009
HA	HM567704	H1N1	06/01/2009	United Ki	A/England/360/2009
HA	HM567816	H1N1	06/01/2009	Ireland	A/Ireland/7/2009
HA	FN423713	H1N1	06/01/2009	Luxembou	A/Luxembourg/43/2009
HA	HM567768	H1N1	06/01/2009	United Ki	A/England/383/2009
HA	JN171910	H1N1	06/01/2009	Canada	A/Montreal/MTL129721/2009
HA	CY056387	H1N1	06/01/2009	Nicaragua	A/Managua/3119.01/2009(H1N1)
HA	CY089888	H1N1	06/01/2009	USA	A/Wisconsin/629-D00881/2009(H1N1)
HA	CY053910	H1N1	06/01/2009	Argentina	A/Argentina/HNRG17/2009
HA	CY050967	H1N1	06/01/2009	USA	A/Wisconsin/629-D01725/2009(H1N1)
HA	CY050959	H1N1	06/01/2009	USA	A/Wisconsin/629-D00941/2009(H1N1)
HA	CY054691	H1N1	06/01/2009	Nicaragua	A/Managua/4467.05/2009(H1N1)
HA	CY065566	H1N1	06/01/2009	United Ki	A/England/384/2009
HA	CY065582	H1N1	06/01/2009	United Ki	A/England/386/2009
HA	CY065646	H1N1	06/01/2009	United Ki	A/England/402/2009
HA	GQ356787	H1N1	06/01/2009	Brazil	A/Sao Paulo/2303/2009
HA	CY047366	H1N1	06/01/2009	USA	A/New York/3795/2009(H1N1)
HA	HM567744	H1N1	06/01/2009	United Ki	A/England/374/2009
HA	CY070725	H1N1	06/01/2009	United Ki	A/Scotland/9/2009
HA	CY076763	H1N1	06/01/2009	Canada	A/Ontario/147265/2009
HA	CY065542	H1N1	06/01/2009	United Ki	A/England/361/2009
HA	CY045175	H1N1	06/01/2009	USA	A/New York/3959/2009(H1N1)
HA	CY065558	H1N1	06/01/2009	United Ki	A/England/382/2009
HA	CY050943	H1N1	06/01/2009	USA	A/Wisconsin/629-D00374/2009(H1N1)
HA	CY050951	H1N1	06/01/2009	USA	A/Wisconsin/629-D00724/2009(H1N1)
HA	CY071039	H1N1	06/01/2009	USA	A/New York/NHRC0003/2009(H1N1)
HA	CY053285	H1N1	06/01/2009	USA	A/Brownsville/400S/2009(H1N1)
HA	CY047374	H1N1	06/01/2009	USA	A/New York/3753/2009(H1N1)
HA	GQ338381	H1N1	06/01/2009	USA	A/Rhode Island/04/2009(H1N1)
HA	CY050983	H1N1	06/02/2009	USA	A/Wisconsin/629-D00592/2009(H1N1)
HA	GQ287625	H1N1	06/02/2009	Japan	A/Tokushima/1/2009(H1N1)
HA	CY051271	H1N1	06/02/2009	USA	A/Wisconsin/629-S0146/2009(H1N1)
HA	GQ329093	H1N1	06/02/2009	France	A/Paris/2709/2009(H1N1)

HA	CY045021	H1N1	06/02/2009	USA	A/New York/3803/2009(H1N1)
HA	GU014776	H1N1	06/02/2009	Japan	A/Chiba-C/48/2009(H1N1)
HA	CY070722	H1N1	06/02/2009	United Ki	A/England/404/2009
HA	CY070718	H1N1	06/02/2009	United Ki	A/England/378/2009
HA	HM567752	H1N1	06/02/2009	United Ki	A/England/377/2009
HA	HM567784	H1N1	06/02/2009	United Ki	A/England/393/2009
HA	HM567776	H1N1	06/02/2009	United Ki	A/England/391/2009
HA	CY051223	H1N1	06/02/2009	USA	A/Wisconsin/629-D00022/2009(H1N1)
HA	CY065614	H1N1	06/02/2009	United Ki	A/England/394/2009
HA	CY051279	H1N1	06/02/2009	USA	A/Wisconsin/629-S0148/2009(H1N1)
HA	GQ329070	H1N1	06/02/2009	France	A/Caen/2716/2009(H1N1)
HA	GQ329076	H1N1	06/02/2009	France	A/Paris/2722/2009(H1N1)
HA	CY051263	H1N1	06/02/2009	USA	A/Wisconsin/629-S0143/2009(H1N1)
HA	CY069893	H1N1	06/02/2009	United Ki	A/England/391/2009
HA	CY076771	H1N1	06/02/2009	Canada	A/Ontario/147723/2009
HA	CY065590	H1N1	06/02/2009	United Ki	A/England/388/2009
HA	CY069901	H1N1	06/02/2009	United Ki	A/England/393/2009
HA	CY044997	H1N1	06/02/2009	USA	A/New York/3828/2009(H1N1)
HA	CY045167	H1N1	06/02/2009	USA	A/New York/3819/2009(H1N1)
HA	CY045013	H1N1	06/02/2009	USA	A/New York/3796/2009(H1N1)
HA	CY045159	H1N1	06/02/2009	USA	A/New York/3818/2009(H1N1)
HA	HM567760	H1N1	06/02/2009	United Ki	A/England/380/2009
HA	CY053293	H1N1	06/02/2009	USA	A/Brownsville/430S/2009(H1N1)
HA	HM567864	H1N1	06/02/2009	United Ki	A/England/416/2009
HA	CY050975	H1N1	06/02/2009	USA	A/Wisconsin/629-D00367/2009(H1N1)
HA	CY049076	H1N1	06/02/2009	Singapore	A/Singapore/ON141/2009(H1N1)
HA	GQ334346	H1N1	06/02/2009	Japan	A/Shizuoka/759/2009(H1N1)
HA	CY053301	H1N1	06/02/2009	USA	A/Brownsville/43H/2009(H1N1)
HA	GQ330645	H1N1	06/03/2009	Russia	A/Moscow/IIV03/2009(H1N1)
HA	JN171911	H1N1	06/03/2009	Canada	A/Montreal/MTL130931/2009
HA	KC781928	H1N1	06/03/2009	USA	A/Virginia/24/2009
HA	KC781439	H1N1	06/03/2009	USA	A/Utah/27/2009
HA	CY070720	H1N1	06/03/2009	United Ki	A/England/390/2009
HA	CY070724	H1N1	06/03/2009	United Ki	A/England/410/2009
HA	HM567800	H1N1	06/03/2009	United Ki	A/England/401/2009
HA	CY107627	H1N1	06/03/2009	United Ki	A/Scotland/Paisley_413869/2009
HA	CY107599	H1N1	06/03/2009	United Ki	A/Scotland/Glasgow_413364/2009
HA	CY107635	H1N1	06/03/2009	United Ki	A/Scotland/Glasgow_413886/2009
HA	CY107621	H1N1	06/03/2009	United Ki	A/Scotland/Glasgow_413848/2009
HA	CY054683	H1N1	06/03/2009	USA	A/Wisconsin/629-D00589/2009(H1N1)
HA	CY069932	H1N1	06/03/2009	United Ki	A/England/425/2009
HA	GU014808	H1N1	06/03/2009	Japan	A/Yamaguchi/21/2009(H1N1)
HA	CY065606	H1N1	06/03/2009	United Ki	A/England/392/2009
HA	CY071047	H1N1	06/03/2009	USA	A/New York/NHRC0004/2009(H1N1)
HA	CY051231	H1N1	06/03/2009	USA	A/Wisconsin/629-D01664/2009(H1N1)
HA	HM567792	H1N1	06/03/2009	United Ki	A/England/399/2009
HA	CY107585	H1N1	06/03/2009	United Ki	A/Scotland/Paisley_412666/2009
HA	CY107592	H1N1	06/03/2009	United Ki	A/Scotland/EastKilbride_412770/2009
HA	CY107614	H1N1	06/03/2009	United Ki	A/Scotland/Paisley_413739/2009
HA	CY107751	H1N1	06/03/2009	United Ki	A/Scotland/Paisley_413960/2009
HA	CY107547	H1N1	06/03/2009	United Ki	A/Scotland/Livingston_412757/2009
HA	CY107565	H1N1	06/03/2009	United Ki	A/Scotland/Glasgow_412286/2009
HA	CY107578	H1N1	06/03/2009	United Ki	A/Scotland/Glasgow_412313/2009
HA	CY065598	H1N1	06/03/2009	United Ki	A/England/389/2009

HA	CY065630	H1N1	06/03/2009	United Ki	A/England/398/2009
HA	CY045495	H1N1	06/03/2009	Germany	A/Baden-Wurttemberg/448/2009(H1N1)
HA	GQ411897	H1N1	06/03/2009	Canada	A/Toronto/T5308/2009(H1N1)
HA	CY055415	H1N1	06/03/2009	USA	A/Wisconsin/629-D00809/2009
HA	CY123472	H1N1	06/03/2009	Singapore	A/Singapore/ON141/2009
HA	CY107745	H1N1	06/03/2009	United Ki	A/Scotland/Paisley_412182/2009
HA	CY044096	H1N1	06/03/2009	USA	A/New York/3895/2009(H1N1)
HA	AB514226	H1N1	06/03/2009	Japan	A/Saitama/55/2009(H1N1)
HA	GQ255901	H1N1	06/04/2009	Russia	A/Moscow/03/2009(H1N1)
HA	CY065686	H1N1	06/04/2009	United Ki	A/England/423/2009
HA	CY070719	H1N1	06/04/2009	United Ki	A/England/379/2009
HA	CY070728	H1N1	06/04/2009	United Ki	A/England/427/2009
HA	CY076779	H1N1	06/04/2009	Canada	A/Ontario/152439/2009
HA	CY076787	H1N1	06/04/2009	Canada	A/Ontario/152846/2009
HA	CY051023	H1N1	06/04/2009	USA	A/Wisconsin/629-D00017/2009(H1N1)
HA	CY051039	H1N1	06/04/2009	USA	A/Wisconsin/629-D00725/2009(H1N1)
HA	CY051031	H1N1	06/04/2009	USA	A/Wisconsin/629-D00658/2009(H1N1)
HA	CY069909	H1N1	06/04/2009	United Ki	A/England/412/2009
HA	HM567856	H1N1	06/04/2009	United Ki	A/England/415/2009
HA	CY051287	H1N1	06/04/2009	USA	A/Wisconsin/629-S0173/2009(H1N1)
HA	CY065654	H1N1	06/04/2009	United Ki	A/England/411/2009
HA	CY089896	H1N1	06/04/2009	USA	A/Wisconsin/629-D01420/2009(H1N1)
HA	CY065662	H1N1	06/04/2009	United Ki	A/England/414/2009
HA	CY065694	H1N1	06/04/2009	United Ki	A/England/428/2009
HA	CY051015	H1N1	06/04/2009	USA	A/Wisconsin/629-D00453/2009(H1N1)
HA	CY070727	H1N1	06/04/2009	United Ki	A/England/422/2009
HA	HM567880	H1N1	06/04/2009	United Ki	A/England/424/2009
HA	CY043342	H1N1	06/04/2009	Denmark	A/Denmark/524/2009(H1N1)
HA	CY051047	H1N1	06/04/2009	USA	A/Wisconsin/629-D00008/2009(H1N1)
HA	CY043334	H1N1	06/04/2009	Denmark	A/Denmark/523/2009(H1N1)
HA	CY070164	H1N1	06/04/2009	United Ki	A/Scotland/13/2009
HA	CY050991	H1N1	06/05/2009	USA	A/Wisconsin/629-D01903/2009(H1N1)
HA	CY052138	H1N1	06/05/2009	USA	A/New York/3980/2009(H1N1)
HA	GQ280797	H1N1	06/05/2009	USA	A/California/04/2009(H1N1)
HA	CY044104	H1N1	06/05/2009	USA	A/New York/3898/2009(H1N1)
HA	GQ396732	H1N1	06/05/2009	USA	A/Utah/10/2009(H1N1)
HA	CY065702	H1N1	06/05/2009	United Ki	A/England/430/2009
HA	HM567544	H1N1	06/05/2009	United Ki	A/England/456/2009
HA	HM567920	H1N1	06/05/2009	United Ki	A/England/443/2009
HA	HM567824	H1N1	06/05/2009	Ireland	A/Ireland/10/2009
HA	HM567896	H1N1	06/05/2009	United Ki	A/England/432/2009
HA	HM567912	H1N1	06/05/2009	United Ki	A/England/436/2009
HA	CY058524	H1N1	06/05/2009	Nicaragua	A/Managua/4905.02/2009(H1N1)
HA	CY058164	H1N1	06/05/2009	Nicaragua	A/Managua/4016.02/2009(H1N1)
HA	CY072966	H1N1	06/05/2009	Nicaragua	A/Managua/2750.01/2009(H1N1)
HA	CY065638	H1N1	06/05/2009	United Ki	A/England/400/2009
HA	CY053896	H1N1	06/05/2009	Argentina	A/Argentina/HNRG13/2009(H1N1)
HA	JN171912	H1N1	06/05/2009	Canada	A/Montreal/MTL133695/2009
HA	CY072910	H1N1	06/05/2009	Nicaragua	A/Managua/2652.01/2009(H1N1)
HA	CY065670	H1N1	06/05/2009	United Ki	A/England/418/2009
HA	CY065710	H1N1	06/05/2009	United Ki	A/England/431/2009
HA	CY065718	H1N1	06/05/2009	United Ki	A/England/433/2009
HA	CY069924	H1N1	06/05/2009	United Ki	A/England/417/2009
HA	CY065678	H1N1	06/05/2009	United Ki	A/England/419/2009

HA	GQ368667	H1N1	06/05/2009	Brazil	A/Distrito Federal/2611/2009
HA	GQ351319	H1N1	06/06/2009	Italy	A/Firenze/10/2009(H1N1)
HA	GQ287623	H1N1	06/06/2009	Japan	A/Shiga/3/2009(H1N1)
HA	GQ411907	H1N1	06/06/2009	China	A/Shanghai/143T/2009(H1N1)
HA	JX625982	H1N1	06/06/2009	United Ki	A/England/421/2009
HA	CY070726	H1N1	06/06/2009	United Ki	A/England/420/2009
HA	CY070731	H1N1	06/06/2009	United Ki	A/England/448/2009
HA	CY058084	H1N1	06/06/2009	Nicaragua	A/Managua/1455.01/2009(H1N1)
HA	CY083471	H1N1	06/06/2009	Nicaragua	A/Managua/WRAIR1689P/2009(H1N1)
HA	HM567904	H1N1	06/06/2009	United Ki	A/England/435/2009
HA	CY065726	H1N1	06/06/2009	United Ki	A/England/434/2009
HA	CY083256	H1N1	06/06/2009	USA	A/San Diego/WRAIR1642P/2009(H1N1)
HA	CY083599	H1N1	06/06/2009	Nicaragua	A/Managua/WRAIR8964F/2009(H1N1)
HA	HM567872	H1N1	06/06/2009	United Ki	A/England/421/2009
HA	JN171913	H1N1	06/06/2009	Canada	A/Montreal/MTL133868/2009
HA	CY053119	H1N1	06/07/2009	USA	A/Houston/170S/2009(H1N1)
HA	GQ287619	H1N1	06/07/2009	Japan	A/Fukuoka-C/1/2009(H1N1)
HA	CY069940	H1N1	06/07/2009	United Ki	A/England/438/2009
HA	CY070729	H1N1	06/07/2009	United Ki	A/England/441/2009
HA	HM567936	H1N1	06/07/2009	United Ki	A/England/450/2009
HA	GQ334338	H1N1	06/07/2009	Japan	A/Fukuoka-C/3/2009(H1N1)
HA	CY070732	H1N1	06/07/2009	United Ki	A/England/449/2009
HA	HM567944	H1N1	06/07/2009	United Ki	A/England/451/2009
HA	CY070730	H1N1	06/07/2009	United Ki	A/England/447/2009
HA	GQ334330	H1N1	06/07/2009	Japan	A/Fukuoka-C/2/2009(H1N1)
HA	CY058076	H1N1	06/07/2009	Nicaragua	A/Managua/171.01/2009(H1N1)
HA	CY051055	H1N1	06/08/2009	USA	A/Wisconsin/629-D02068/2009(H1N1)
HA	CY051071	H1N1	06/08/2009	USA	A/Wisconsin/629-D00579/2009(H1N1)
HA	CY051495	H1N1	06/08/2009	USA	A/Wisconsin/629-S0189/2009(H1N1)
HA	CY051295	H1N1	06/08/2009	USA	A/Wisconsin/629-S0186/2009(H1N1)
HA	HM567888	H1N1	06/08/2009	United Ki	A/England/429/2009
HA	CY083152	H1N1	06/08/2009	Dominican	A/Santo Domingo/WRAIR1044N/2009
HA	KC781793	H1N1	06/08/2009	USA	A/Wisconsin/26/2009
HA	CY051327	H1N1	06/08/2009	USA	A/Wisconsin/629-S0203/2009(H1N1)
HA	CY051503	H1N1	06/08/2009	USA	A/Wisconsin/629-S0206/2009(H1N1)
HA	CY052130	H1N1	06/08/2009	USA	A/New York/3976/2009(H1N1)
HA	CY051303	H1N1	06/08/2009	USA	A/Wisconsin/629-S0197/2009(H1N1)
HA	CY051311	H1N1	06/08/2009	USA	A/Wisconsin/629-S0201/2009(H1N1)
HA	CY071546	H1N1	06/08/2009	USA	A/California/WR1323P/2009(H1N1)
HA	JN171870	H1N1	06/08/2009	Canada	A/Quebec/QC145573/2009
HA	CY076795	H1N1	06/08/2009	Canada	A/Ontario/156785/2009
HA	CY045207	H1N1	06/08/2009	USA	A/New York/4057/2009(H1N1)
HA	CY051063	H1N1	06/08/2009	USA	A/Wisconsin/629-D00101/2009(H1N1)
HA	CY058132	H1N1	06/08/2009	Nicaragua	A/Managua/516.01/2009(H1N1)
HA	GQ421199	H1N1	06/08/2009	Italy	A/Ancona/01/2009(H1N1)
HA	CY051319	H1N1	06/08/2009	USA	A/Wisconsin/629-S0202/2009(H1N1)
HA	CY051351	H1N1	06/09/2009	USA	A/Wisconsin/629-S0226/2009(H1N1)
HA	CY052122	H1N1	06/09/2009	USA	A/New York/3969/2009(H1N1)
HA	GQ287627	H1N1	06/09/2009	Japan	A/Yokohama/1/2009(H1N1)
HA	CY065734	H1N1	06/09/2009	United Ki	A/England/445/2009
HA	CY070165	H1N1	06/09/2009	United Ki	A/Scotland/16/2009
HA	CY095847	H1N1	06/09/2009	China	A/Shenzhen/71/2009
HA	CY095785	H1N1	06/09/2009	China	A/Shenzhen/40/2009
HA	CY095792	H1N1	06/09/2009	China	A/Shenzhen/42/2009

HA	JN171914	H1N1	06/09/2009	Canada	A/Montreal/MTL136104/2009
HA	JN171871	H1N1	06/09/2009	Canada	A/Quebec/QC145697/2009
HA	CY058100	H1N1	06/09/2009	Nicaragua	A/Managua/168.02/2009(H1N1)
HA	CY051511	H1N1	06/09/2009	USA	A/Wisconsin/629-S0208/2009(H1N1)
HA	CY052290	H1N1	06/09/2009	USA	A/Texas/42091791/2009(H1N1)
HA	CY051343	H1N1	06/09/2009	USA	A/Wisconsin/629-S0222/2009(H1N1)
HA	CY051335	H1N1	06/09/2009	USA	A/Wisconsin/629-S0220/2009(H1N1)
HA	KF849772	H1N1	06/09/2009	Thailand	A/Thailand/SirirajICRC_CBI_9/2009
HA	KF849771	H1N1	06/09/2009	Thailand	A/Thailand/SirirajICRC_CBI_8/2009
HA	CY045970	H1N1	06/09/2009	Canada	A/Toronto/T5362/2009(H1N1)
HA	CY069948	H1N1	06/09/2009	United Ki	A/England/444/2009
HA	CY045037	H1N1	06/09/2009	USA	A/New York/3967/2009(H1N1)
HA	CY045053	H1N1	06/09/2009	USA	A/New York/4052/2009(H1N1)
HA	CY049883	H1N1	06/09/2009	El Salvad	A/San Salvador/WR0167N/2009(H1N1)
HA	CY051239	H1N1	06/09/2009	USA	A/Wisconsin/629-D01199/2009(H1N1)
HA	GQ365426	H1N1	06/09/2009	Japan	A/Iwate/1/2009(H1N1)
HA	HM567928	H1N1	06/09/2009	United Ki	A/England/446/2009
HA	CY043350	H1N1	06/09/2009	Denmark	A/Denmark/528/2009(H1N1)
HA	CY073342	H1N1	06/10/2009	USA	A/San Diego/WR1628P/2009(H1N1)
HA	CY044112	H1N1	06/10/2009	USA	A/New York/3992/2009(H1N1)
HA	CY052350	H1N1	06/10/2009	Brazil	A/Santa Catarina/460/2009(H1N1)
HA	CY052266	H1N1	06/10/2009	USA	A/Texas/42102708/2009(H1N1)
HA	CY052298	H1N1	06/10/2009	USA	A/Texas/42103399/2009(H1N1)
HA	CY051391	H1N1	06/10/2009	USA	A/Wisconsin/629-S0255/2009(H1N1)
HA	CY107757	H1N1	06/10/2009	United Ki	A/Scotland/Paisley_415110/2009
HA	CY070158	H1N1	06/10/2009	United Ki	A/England/471/2009
HA	CY107654	H1N1	06/10/2009	United Ki	A/Scotland/Glasgow_414473/2009
HA	KC780423	H1N1	06/10/2009	USA	A/Minnesota/22/2009
HA	CY107642	H1N1	06/10/2009	United Ki	A/Scotland/Glasgow_413941/2009
HA	CY090000	H1N1	06/10/2009	USA	A/Wisconsin/629-S0235/2009(H1N1)
HA	CY051367	H1N1	06/10/2009	USA	A/Wisconsin/629-S0248/2009(H1N1)
HA	CY051399	H1N1	06/10/2009	USA	A/Wisconsin/629-S0252/2009(H1N1)
HA	CY051359	H1N1	06/10/2009	USA	A/Wisconsin/629-S0245/2009(H1N1)
HA	CY051375	H1N1	06/10/2009	USA	A/Wisconsin/629-S0247/2009(H1N1)
HA	JN171872	H1N1	06/10/2009	Canada	A/Quebec/QC145783/2009
HA	CY045045	H1N1	06/10/2009	USA	A/New York/4006/2009(H1N1)
HA	CY045191	H1N1	06/10/2009	USA	A/New York/4014/2009(H1N1)
HA	CY045199	H1N1	06/10/2009	USA	A/New York/4048/2009(H1N1)
HA	CY051383	H1N1	06/10/2009	USA	A/Wisconsin/629-S0254/2009(H1N1)
HA	CY046063	H1N1	06/10/2009	Italy	A/Italy/130/2009(H1N1)
HA	CY045215	H1N1	06/10/2009	USA	A/New York/4074/2009(H1N1)
HA	GQ365446	H1N1	06/11/2009	Japan	A/Sapporo/1/2009(H1N1)
HA	CY053111	H1N1	06/11/2009	USA	A/Houston/160S/2009(H1N1)
HA	GQ421200	H1N1	06/11/2009	Italy	A/Ancona/02/2009(H1N1)
HA	HM569703	H1N1	06/11/2009	Argentina	A/Argentina/19464/2009(H1N1)
HA	GQ286175	H1N1	06/11/2009	Chile	A/Castro/JXP/2009(H1N1)
HA	CY065742	H1N1	06/11/2009	United Ki	A/Scotland/10/2009
HA	HQ228028	H1N1	06/11/2009	Finland	A/Finland/568/2009
HA	JN171915	H1N1	06/11/2009	Canada	A/Montreal/MTL138378/2009
HA	CY051887	H1N1	06/11/2009	USA	A/Texas/42114261/2009(H1N1)
HA	CY054283	H1N1	06/11/2009	Brazil	A/Santa Catarina/459/2009(H1N1)
HA	CY057206	H1N1	06/11/2009	Nicaragua	A/Managua/167.01/2009(H1N1)
HA	GQ365410	H1N1	06/11/2009	Japan	A/Akita/1/2009(H1N1)
HA	GQ351314	H1N1	06/11/2009	Hong Kong	A/Hong Kong/2369/2009(H1N1)

HA	CY089992	H1N1	06/11/2009	USA	A/Wisconsin/629-D01183/2009(H1N1)
HA	CY063486	H1N1	06/11/2009	USA	A/Boston/110/2009(H1N1)
HA	CY051407	H1N1	06/11/2009	USA	A/Wisconsin/629-S0269/2009(H1N1)
HA	CY051079	H1N1	06/11/2009	USA	A/Wisconsin/629-D01326/2009(H1N1)
HA	CY051879	H1N1	06/11/2009	USA	A/Texas/42113095/2009(H1N1)
HA	CY045077	H1N1	06/12/2009	USA	A/New York/4072/2009(H1N1)
HA	CY051855	H1N1	06/12/2009	USA	A/Texas/42122969/2009(H1N1)
HA	HQ228025	H1N1	06/12/2009	Finland	A/Finland/565/2009
HA	CY073318	H1N1	06/12/2009	USA	A/San Diego/WR1625P/2009(H1N1)
HA	GQ894841	H1N1	06/12/2009	USA	A/Utah/06/2009(H1N1)
HA	CY051111	H1N1	06/12/2009	USA	A/Wisconsin/629-D02263/2009(H1N1)
HA	CY049891	H1N1	06/12/2009	El Salvad	A/San Salvador/0169T/2009(H1N1)
HA	CY051839	H1N1	06/12/2009	USA	A/Texas/42123701/2009(H1N1)
HA	KF700940	H1N1	06/12/2009	Brazil	A/Minas Gerais/490/2009
HA	KF700937	H1N1	06/12/2009	Brazil	A/Minas Gerais/490/2009
HA	KT180977	H1N1	06/12/2009	Singapore	A/Singapore/DMS1/2009
HA	JN171873	H1N1	06/12/2009	Canada	A/Quebec/QC145950/2009
HA	CY070157	H1N1	06/12/2009	United Ki	A/England/463/2009
HA	CY052194	H1N1	06/12/2009	USA	A/Texas/42121926/2009(H1N1)
HA	HM569683	H1N1	06/12/2009	Argentina	A/Argentina/19618/2009(H1N1)
HA	KF700939	H1N1	06/12/2009	Brazil	A/Minas Gerais/490/2009
HA	KF700938	H1N1	06/12/2009	Brazil	A/Minas Gerais/490/2009
HA	HQ228026	H1N1	06/12/2009	Finland	A/Finland/566/2009
HA	CY058212	H1N1	06/12/2009	Nicaragua	A/Managua/578.01/2009(H1N1)
HA	CY058092	H1N1	06/12/2009	Nicaragua	A/Managua/655.02/2009(H1N1)
HA	CY050158	H1N1	06/12/2009	USA	A/New York/4095/2009(H1N1)
HA	CY073326	H1N1	06/12/2009	USA	A/San Diego/WR1626P/2009(H1N1)
HA	GU014792	H1N1	06/12/2009	Japan	A/Kanagawa/137/2009(H1N1)
HA	GQ463200	H1N1	06/13/2009	China	A/Hunan/SWL3/2009(H1N1)
HA	GU014780	H1N1	06/13/2009	Japan	A/Hiroshima/200/2009(H1N1)
HA	CY058220	H1N1	06/13/2009	Nicaragua	A/Managua/5221.03/2009(H1N1)
HA	CY073310	H1N1	06/13/2009	USA	A/San Diego/WR1624P/2009(H1N1)
HA	CY073334	H1N1	06/13/2009	USA	A/San Diego/WR1627P/2009(H1N1)
HA	CY075171	H1N1	06/13/2009	Chile	A/Chile/1586/2009(H1N1)
HA	GQ894816	H1N1	06/13/2009	USA	A/Illinois/07/2009(H1N1)
HA	GQ866951	H1N1	06/13/2009	Thailand	A/Thailand/CU-B5/2009(H1N1)
HA	CY051103	H1N1	06/13/2009	USA	A/Wisconsin/629-D01618/2009(H1N1)
HA	CY050999	H1N1	06/13/2009	USA	A/Wisconsin/629-D00665/2009(H1N1)
HA	CY064500	H1N1	06/13/2009	USA	A/Boston/115/2009(H1N1)
HA	CY054723	H1N1	06/13/2009	USA	A/California/VRDL6/2009(H1N1)
HA	JN171874	H1N1	06/13/2009	Canada	A/Quebec/QC145995/2009
HA	CY123692	H1N1	06/13/2009	Singapore	A/Singapore/ON199/2009
HA	JQ409142	H1N1	06/13/2009	Finland	A/Helsinki/Vi3/2009(H1N1)
HA	JQ409134	H1N1	06/13/2009	Finland	A/Helsinki/Vi2/2009
HA	HQ228029	H1N1	06/13/2009	Finland	A/Finland/569/2009
HA	CY052314	H1N1	06/13/2009	USA	A/Texas/42132413/2009(H1N1)
HA	CY058108	H1N1	06/13/2009	Nicaragua	A/Managua/2152.01/2009(H1N1)
HA	CY083607	H1N1	06/13/2009	Nicaragua	A/Managua/WRAIR8997F/2009(H1N1)
HA	CY083503	H1N1	06/13/2009	Nicaragua	A/Managua/WRAIR1693P/2009(H1N1)
HA	CY054747	H1N1	06/13/2009	USA	A/California/VRDL9/2009(H1N1)
HA	GQ259909	H1N1	06/13/2009	China	A/Jiangsu/1/2009(H1N1)
HA	CY057214	H1N1	06/13/2009	Nicaragua	A/Managua/109.01/2009(H1N1)
HA	CY050262	H1N1	06/13/2009	China	A/Qingdao/148/2009(H1N1)
HA	JN171916	H1N1	06/13/2009	Canada	A/Montreal/MTL139735/2009

HA	CY063494	H1N1	06/13/2009	USA	A/Boston/112/2009(H1N1)
HA	HQ228027	H1N1	06/13/2009	Finland	A/Finland/567/2009
HA	KC782097	H1N1	06/13/2009	USA	A/Rhode Island/15/2009
HA	KC781650	H1N1	06/13/2009	USA	A/Maryland/32/2009
HA	CY045061	H1N1	06/13/2009	USA	A/New York/4099/2009(H1N1)
HA	CY058228	H1N1	06/13/2009	Nicaragua	A/Managua/5363.02/2009(H1N1)
HA	CY058172	H1N1	06/13/2009	Nicaragua	A/Managua/792.01/2009(H1N1)
HA	CY051095	H1N1	06/13/2009	USA	A/Wisconsin/629-D00196/2009(H1N1)
HA	CY058116	H1N1	06/13/2009	Nicaragua	A/Managua/1637.01/2009(H1N1)
HA	CY058148	H1N1	06/13/2009	Nicaragua	A/Managua/4085.02/2009(H1N1)
HA	CY064460	H1N1	06/13/2009	USA	A/Boston/96/2009(H1N1)
HA	GQ365436	H1N1	06/13/2009	Japan	A/Kagoshima/1/2009(H1N1)
HA	CY047382	H1N1	06/13/2009	USA	A/New York/4100/2009(H1N1)
HA	GQ527163	H1N1	06/14/2009	Singapore	A/Singapore/TLL53/2009(H1N1)
HA	GQ457465	H1N1	06/14/2009	USA	A/Utah/05/2009(H1N1)
HA	CY049923	H1N1	06/14/2009	USA	A/New Bern/WR0670/2009(H1N1)
HA	CY051151	H1N1	06/14/2009	USA	A/Wisconsin/629-D01147/2009(H1N1)
HA	CY054739	H1N1	06/14/2009	USA	A/California/VRDL8/2009(H1N1)
HA	CY062522	H1N1	06/14/2009	Mexico	A/Mexico city/CIA6/2009(H1N1)
HA	GQ527164	H1N1	06/14/2009	Singapore	A/Singapore/TLL54/2009
HA	GQ351290	H1N1	06/14/2009	Italy	A/Italy/85/2009(H1N1)
HA	GQ527166	H1N1	06/14/2009	Singapore	A/Singapore/TLL52/2009
HA	CY054707	H1N1	06/14/2009	USA	A/California/VRDL4/2009(H1N1)
HA	CY052391	H1N1	06/14/2009	USA	A/Texas/42142537/2009(H1N1)
HA	GQ527165	H1N1	06/14/2009	Singapore	A/Singapore/TLL51/2009(H1N1)
HA	CY051119	H1N1	06/14/2009	USA	A/Wisconsin/629-D00916/2009(H1N1)
HA	CY123717	H1N1	06/14/2009	Singapore	A/Singapore/ON202/2009
HA	GQ457479	H1N1	06/14/2009	USA	A/Utah/07/2009(H1N1)
HA	GQ457470	H1N1	06/14/2009	USA	A/Utah/11/2009(H1N1)
HA	CY123649	H1N1	06/14/2009	Singapore	A/Singapore/ON193/2009
HA	CY051127	H1N1	06/14/2009	USA	A/Wisconsin/629-D00119/2009(H1N1)
HA	GQ457514	H1N1	06/15/2009	USA	A/California/25/2009(H1N1)
HA	CY064492	H1N1	06/15/2009	USA	A/Boston/109/2009(H1N1)
HA	GU014798	H1N1	06/15/2009	Japan	A/Niigata/690/2009(H1N1)
HA	CY058548	H1N1	06/15/2009	Nicaragua	A/Managua/4935.03/2009(H1N1)
HA	CY055455	H1N1	06/15/2009	USA	A/California/VRDL12/2009(H1N1)
HA	CY060750	H1N1	06/15/2009	Canada	A/Ontario/9739/2009(H1N1)
HA	CY063502	H1N1	06/15/2009	USA	A/Boston/113/2009(H1N1)
HA	CY075179	H1N1	06/15/2009	Chile	A/Chile/1598/2009(H1N1)
HA	CY075195	H1N1	06/15/2009	Chile	A/Chile/1600/2009(H1N1)
HA	CY075211	H1N1	06/15/2009	Chile	A/Chile/1624/2009(H1N1)
HA	CY051007	H1N1	06/15/2009	USA	A/Wisconsin/629-D01619/2009(H1N1)
HA	CY051135	H1N1	06/15/2009	USA	A/Wisconsin/629-D01152/2009(H1N1)
HA	CY051431	H1N1	06/15/2009	USA	A/Wisconsin/629-S0293/2009(H1N1)
HA	HM569701	H1N1	06/15/2009	Argentina	A/Argentina/19531/2009(H1N1)
HA	CY052274	H1N1	06/15/2009	USA	A/Texas/42151049/2009(H1N1)
HA	CY064476	H1N1	06/15/2009	USA	A/Boston/106/2009(H1N1)
HA	CY060742	H1N1	06/15/2009	Canada	A/Ontario/9698/2009(H1N1)
HA	CY123709	H1N1	06/15/2009	Singapore	A/Singapore/ON201/2009
HA	CY123825	H1N1	06/15/2009	Singapore	A/Singapore/ON226/2009
HA	KC781705	H1N1	06/15/2009	USA	A/Oregon/10/2009
HA	CY089928	H1N1	06/15/2009	USA	A/Wisconsin/629-D00889/2009(H1N1)
HA	CY083336	H1N1	06/15/2009	USA	A/San Diego/WRAIR1654P/2009
HA	CY054715	H1N1	06/15/2009	USA	A/California/VRDL5/2009(H1N1)

HA	HM569675	H1N1	06/15/2009	Argentina	A/Argentina/19527/2009(H1N1)
HA	HQ228030	H1N1	06/15/2009	Finland	A/Finland/571/2009
HA	JN171917	H1N1	06/15/2009	Canada	A/Montreal/MTL141466/2009
HA	CY064468	H1N1	06/15/2009	USA	A/Boston/103/2009(H1N1)
HA	CY051815	H1N1	06/15/2009	USA	A/Texas/42152486/2009(H1N1)
HA	CY070159	H1N1	06/15/2009	United Ki	A/England/479/2009
HA	CY051423	H1N1	06/15/2009	USA	A/Wisconsin/629-S0279/2009(H1N1)
HA	CY051415	H1N1	06/15/2009	USA	A/Wisconsin/629-S0280/2009(H1N1)
HA	CY070160	H1N1	06/15/2009	United Ki	A/England/480/2009
HA	KC781551	H1N1	06/15/2009	USA	A/Massachusetts/16/2009
HA	CY058124	H1N1	06/15/2009	Nicaragua	A/Managua/5453.01/2009(H1N1)
HA	CY051087	H1N1	06/15/2009	USA	A/Wisconsin/629-D01844/2009(H1N1)
HA	HM752472	H1N1	06/15/2009	Thailand	A/Thailand/CU-MV5/2009
HA	CY052106	H1N1	06/15/2009	USA	A/Wisconsin/629-D00739/2009(H1N1)
HA	GU014802	H1N1	06/15/2009	Japan	A/Shizuoka/793/2009(H1N1)
HA	CY075187	H1N1	06/15/2009	Chile	A/Chile/1599/2009(H1N1)
HA	CY075203	H1N1	06/15/2009	Chile	A/Chile/1603/2009(H1N1)
HA	GU014772	H1N1	06/15/2009	Japan	A/Aichi/198/2009(H1N1)
HA	GQ365455	H1N1	06/16/2009	Japan	A/Utsunomiya/2/2009(H1N1)
HA	CY052210	H1N1	06/16/2009	USA	A/Texas/42163291/2009(H1N1)
HA	CY058156	H1N1	06/16/2009	Nicaragua	A/Managua/2330.02/2009(H1N1)
HA	CY049836	H1N1	06/16/2009	Colombia	A/Bogota/WR0090N/2009(H1N1)
HA	CY051159	H1N1	06/16/2009	USA	A/Wisconsin/629-D00541/2009(H1N1)
HA	CY051463	H1N1	06/16/2009	USA	A/Wisconsin/629-S0314/2009(H1N1)
HA	CY064484	H1N1	06/16/2009	USA	A/Boston/108/2009(H1N1)
HA	CY074045	H1N1	06/16/2009	Argentina	A/Argentina/HNRG49/2009
HA	CY053965	H1N1	06/16/2009	Argentina	A/Argentina/HNRG45/2009(H1N1)
HA	CY052383	H1N1	06/16/2009	USA	A/Texas/42163295/2009(H1N1)
HA	JN171918	H1N1	06/16/2009	Canada	A/Montreal/MTL141633/2009
HA	CY044171	H1N1	06/16/2009	USA	A/Bethesda/SP506/2009(H1N1)
HA	CY051143	H1N1	06/16/2009	USA	A/Wisconsin/629-D01734/2009(H1N1)
HA	CY046062	H1N1	06/16/2009	Italy	A/Italy/129/2009(H1N1)
HA	GQ334355	H1N1	06/16/2009	Japan	A/Utsunomiya/1/2009(H1N1)
HA	GU014774	H1N1	06/16/2009	Japan	A/Aichi/202/2009(H1N1)
HA	GQ455032	H1N1	06/17/2009	China	A/Nanjing/2/2009(H1N1)
HA	CY107675	H1N1	06/17/2009	United Ki	A/Scotland/Glasgow_419977/2009
HA	GQ866959	H1N1	06/17/2009	Thailand	A/Thailand/CU-H9/2009(H1N1)
HA	CY052098	H1N1	06/17/2009	USA	A/New York/4238/2009(H1N1)
HA	CY051871	H1N1	06/17/2009	USA	A/Texas/42173957/2009(H1N1)
HA	CY064588	H1N1	06/17/2009	USA	A/Boston/127/2009(H1N1)
HA	CY055447	H1N1	06/17/2009	USA	A/California/VRDL11/2009(H1N1)
HA	CY051543	H1N1	06/17/2009	USA	A/New York/4403/2009(H1N1)
HA	CY064572	H1N1	06/17/2009	USA	A/Boston/125/2009(H1N1)
HA	CY045978	H1N1	06/17/2009	Canada	A/Toronto/T9842/2009(H1N1)
HA	CY107668	H1N1	06/17/2009	United Ki	A/Scotland/Glasgow_419865/2009
HA	CY047779	H1N1	06/17/2009	Argentina	A/Argentina/7649/2009
HA	CY047811	H1N1	06/17/2009	Argentina	A/Argentina/7967/2009
HA	CY061539	H1N1	06/17/2009	Argentina	A/Argentina/HNRG83/2009
HA	JN171875	H1N1	06/17/2009	Canada	A/Quebec/QC146397/2009
HA	CY054843	H1N1	06/17/2009	USA	A/California/VRDL31/2009(H1N1)
HA	CY107193	H1N1	06/17/2009	United Ki	A/Scotland/Paisley_417557/2009
HA	CY107661	H1N1	06/17/2009	United Ki	A/Scotland/Glasgow_419707/2009
HA	CY054731	H1N1	06/17/2009	USA	A/California/VRDL7/2009(H1N1)
HA	GQ392029	H1N1	06/17/2009	Italy	A/Italy/127/2009(H1N1)

HA	CY107772	H1N1	06/17/2009	United Ki	A/Scotland/EastKilbride_419809/2009
HA	CY051439	H1N1	06/17/2009	USA	A/Wisconsin/629-S0319/2009(H1N1)
HA	JN171919	H1N1	06/17/2009	Canada	A/Montreal/MTL144431/2009
HA	CY049971	H1N1	06/17/2009	Dominicar	A/Santo Domingo/1066N/2009(H1N1)
HA	CY089725	H1N1	06/17/2009	USA	A/Boston/139/2009
HA	CY047819	H1N1	06/17/2009	Argentina	A/Argentina/7980/2009
HA	CY053936	H1N1	06/17/2009	Argentina	A/Argentina/HNRG31/2009
HA	CY044120	H1N1	06/17/2009	USA	A/New York/4197/2009(H1N1)
HA	GU014750	H1N1	06/17/2009	Japan	A/Hiroshima/201/2009(H1N1)
HA	CY044179	H1N1	06/18/2009	USA	A/Silver Spring/SP509/2009(H1N1)
HA	CY054803	H1N1	06/18/2009	USA	A/California/VRDL26/2009(H1N1)
HA	HQ228034	H1N1	06/18/2009	Finland	A/Finland/578/2009
HA	GQ280264	H1N1	06/18/2009	China	A/Nanjing/1/2009(H1N1)
HA	CY051455	H1N1	06/18/2009	USA	A/Wisconsin/629-S0339/2009(H1N1)
HA	CY073302	H1N1	06/18/2009	USA	A/Silver Spring/WRAIRSP510P3/2009(H1
HA	CY073278	H1N1	06/18/2009	USA	A/Silver Spring/WRAIRSP510P18/2009(H
HA	CY073286	H1N1	06/18/2009	USA	A/Silver Spring/WRAIRSP510P2/2009(H1
HA	HQ228032	H1N1	06/18/2009	Finland	A/Finland/576/2009
HA	CY051447	H1N1	06/18/2009	USA	A/Wisconsin/629-S0345/2009(H1N1)
HA	JN171877	H1N1	06/18/2009	Canada	A/Quebec/QC146561/2009
HA	CY064644	H1N1	06/18/2009	USA	A/Boston/135/2009(H1N1)
HA	CY064628	H1N1	06/18/2009	USA	A/Boston/133/2009(H1N1)
HA	HQ228038	H1N1	06/18/2009	Finland	A/Finland/582/2009
HA	CY089944	H1N1	06/18/2009	USA	A/Wisconsin/629-D01875/2009(H1N1)
HA	KC781851	H1N1	06/18/2009	USA	A/Oregon/13/2009
HA	CY070161	H1N1	06/18/2009	United Ki	A/England/481/2009
HA	CY047827	H1N1	06/18/2009	Argentina	A/Argentina/8019/2009
HA	HQ228039	H1N1	06/18/2009	Finland	A/Finland/583/2009
HA	HQ228040	H1N1	06/18/2009	Finland	A/Finland/584/2009
HA	CY070163	H1N1	06/18/2009	United Ki	A/England/485/2009
HA	CY089904	H1N1	06/18/2009	USA	A/Wisconsin/629-D01115/2009(H1N1)
HA	CY053928	H1N1	06/18/2009	Argentina	A/Argentina/HNRG3/2009(H1N1)
HA	JN171876	H1N1	06/18/2009	Canada	A/Quebec/QC146496/2009
HA	HQ228041	H1N1	06/18/2009	Finland	A/Finland/585/2009
HA	CY073270	H1N1	06/18/2009	USA	A/Silver Spring/WRAIRSP510P12/2009(H
HA	HM569691	H1N1	06/18/2009	Argentina	A/Argentina/19656/2009(H1N1)
HA	CY070229	H1N1	06/18/2009	United Ki	A/England/92880010/2009
HA	KC781810	H1N1	06/18/2009	USA	A/Oregon/11/2009
HA	CY073294	H1N1	06/18/2009	USA	A/Silver Spring/WRAIRSP510P20/2009(H
HA	CY051167	H1N1	06/18/2009	USA	A/Wisconsin/629-D02063/2009(H1N1)
HA	CY047795	H1N1	06/18/2009	Argentina	A/Argentina/7937/2009
HA	CY044187	H1N1	06/18/2009	USA	A/Silver Spring/SP510/2009(H1N1)
HA	GQ365428	H1N1	06/18/2009	Japan	A/Iwate/2/2009(H1N1)
HA	CY043118	H1N1	06/18/2009	USA	A/Bethesda/SP508/2009(H1N1)
HA	CY064556	H1N1	06/18/2009	USA	A/Boston/123/2009(H1N1)
HA	GU014778	H1N1	06/18/2009	Japan	A/Chiba-C/51/2009(H1N1)
HA	CY064660	H1N1	06/18/2009	USA	A/Boston/138/2009(H1N1)
HA	CY070166	H1N1	06/18/2009	United Ki	A/England/495/2009
HA	CY075219	H1N1	06/18/2009	Chile	A/Chile/2009/2009(H1N1)
HA	CY073262	H1N1	06/18/2009	USA	A/Silver Spring/WRAIRSP510P10/2009(H
HA	GU014794	H1N1	06/18/2009	Japan	A/Kanagawa/140/2009(H1N1)
HA	GQ359771	H1N1	06/19/2009	Chile	A/Puerto Montt/Bio87/2009(H1N1)
HA	CY064532	H1N1	06/19/2009	USA	A/Boston/120/2009(H1N1)
HA	CY049364	H1N1	06/19/2009	Singapore	A/Singapore/ON313/2009(H1N1)

HA	HQ228033	H1N1	06/19/2009	Finland	A/Finland/577/2009
HA	CY051559	H1N1	06/19/2009	USA	A/New York/4459/2009(H1N1)
HA	CY051847	H1N1	06/19/2009	USA	A/Texas/42191653/2009(H1N1)
HA	CY064540	H1N1	06/19/2009	USA	A/Boston/121/2009(H1N1)
HA	CY064620	H1N1	06/19/2009	USA	A/Boston/132/2009(H1N1)
HA	CY049987	H1N1	06/19/2009	Mexico	A/Mexico City/WR1087T/2009(H1N1)
HA	CY123921	H1N1	06/19/2009	Singapore	A/Singapore/ON313/2009
HA	CY123900	H1N1	06/19/2009	Singapore	A/Singapore/ON279/2009
HA	HQ228035	H1N1	06/19/2009	Finland	A/Finland/579/2009
HA	HQ228036	H1N1	06/19/2009	Finland	A/Finland/580/2009
HA	CY075227	H1N1	06/19/2009	Chile	A/Chile/2239/2009(H1N1)
HA	HQ228037	H1N1	06/19/2009	Finland	A/Finland/581/2009
HA	CY083088	H1N1	06/19/2009	Colombia	A/Bogota/WRAIR0442T/2009
HA	CY087307	H1N1	06/19/2009	Hong Kong	A/Hong Kong/423432/2009
HA	JN171920	H1N1	06/19/2009	Canada	A/Montreal/MTL144954/2009
HA	JN171878	H1N1	06/19/2009	Canada	A/Quebec/QC146631/2009
HA	CY052234	H1N1	06/19/2009	USA	A/Texas/42191647/2009(H1N1)
HA	CY061347	H1N1	06/19/2009	Argentina	A/Argentina/HNRG104/2009
HA	CY049084	H1N1	06/19/2009	Singapore	A/Singapore/ON279/2009(H1N1)
HA	CY052415	H1N1	06/19/2009	USA	A/Texas/42192947/2009(H1N1)
HA	CY061536	H1N1	06/19/2009	Argentina	A/Argentina/HNRG72/2009
HA	CY047851	H1N1	06/19/2009	Argentina	A/Argentina/8673/2009
HA	CY047803	H1N1	06/19/2009	Argentina	A/Argentina/7953/2009
HA	CY045069	H1N1	06/19/2009	USA	A/New York/4290/2009(H1N1)
HA	GQ494354	H1N1	06/20/2009	Russia	A/Moscow/IIV05/2009(H1N1)
HA	CY064524	H1N1	06/20/2009	USA	A/Boston/118/2009(H1N1)
HA	CY064580	H1N1	06/20/2009	USA	A/Boston/126/2009(H1N1)
HA	CY049060	H1N1	06/20/2009	Singapore	A/Singapore/ON305/2009(H1N1)
HA	CY075235	H1N1	06/20/2009	Chile	A/Chile/2851/2009(H1N1)
HA	JN171880	H1N1	06/20/2009	Canada	A/Quebec/QC146628/2009
HA	CY054763	H1N1	06/20/2009	USA	A/California/VRDL20/2009(H1N1)
HA	CY052322	H1N1	06/20/2009	USA	A/Texas/42202026/2009(H1N1)
HA	CY123913	H1N1	06/20/2009	Singapore	A/Singapore/ON305/2009
HA	KT181112	H1N1	06/20/2009	Singapore	A/Singapore/DMS385/2009
HA	CY051175	H1N1	06/20/2009	USA	A/Wisconsin/629-D00428/2009(H1N1)
HA	CY123908	H1N1	06/20/2009	Singapore	A/Singapore/ON304/2009
HA	CY047875	H1N1	06/20/2009	Argentina	A/Argentina/9004/2009
HA	HQ228031	H1N1	06/20/2009	Finland	A/Finland/575/2009
HA	JN171879	H1N1	06/20/2009	Canada	A/Quebec/QC146618/2009
HA	CY072982	H1N1	06/20/2009	Nicaragua	A/Managua/5273.02/2009(H1N1)
HA	CY046061	H1N1	06/20/2009	Italy	A/Italy/128/2009(H1N1)
HA	CY051191	H1N1	06/20/2009	USA	A/Wisconsin/629-D00496/2009(H1N1)
HA	CY052242	H1N1	06/20/2009	USA	A/Texas/42201798/2009(H1N1)
HA	CY051863	H1N1	06/20/2009	USA	A/Texas/42201824/2009(H1N1)
HA	CY064652	H1N1	06/20/2009	USA	A/Boston/137/2009(H1N1)
HA	CY051535	H1N1	06/21/2009	USA	A/New York/4402/2009(H1N1)
HA	CY052330	H1N1	06/21/2009	USA	A/Texas/42211898/2009(H1N1)
HA	CY058532	H1N1	06/21/2009	Nicaragua	A/Managua/4514.03/2009(H1N1)
HA	CY070226	H1N1	06/21/2009	United Ki	A/England/92860086/2009
HA	CY123934	H1N1	06/21/2009	Singapore	A/Singapore/ON360/2009
HA	CY123938	H1N1	06/21/2009	Singapore	A/Singapore/ON361/2009
HA	CY123942	H1N1	06/21/2009	Singapore	A/Singapore/ON362/2009
HA	CY075901	H1N1	06/21/2009	India	A/Pune/NIV161/2009
HA	CY070162	H1N1	06/21/2009	United Ki	A/England/483/2009

HA	CY047843	H1N1	06/21/2009	Argentina	A/Argentina/8574/2009
HA	CY053945	H1N1	06/21/2009	Argentina	A/Argentina/HNRG36/2009(H1N1)
HA	JN171921	H1N1	06/21/2009	Canada	A/Montreal/MTL082953/2009
HA	CY047835	H1N1	06/21/2009	Argentina	A/Argentina/8551/2009
HA	CY047859	H1N1	06/21/2009	Argentina	A/Argentina/8989/2009
HA	CY051183	H1N1	06/21/2009	USA	A/Wisconsin/629-D02220/2009(H1N1)
HA	CY064604	H1N1	06/21/2009	USA	A/Boston/130/2009(H1N1)
HA	CY058140	H1N1	06/22/2009	Nicaragua	A/Managua/5665.01/2009(H1N1)
HA	CY111611	H1N1	06/22/2009	Hong Kong	A/Hong Kong/H090-661-V10/2009
HA	CY049356	H1N1	06/22/2009	Singapore	A/Singapore/ON368/2009(H1N1)
HA	CY051199	H1N1	06/22/2009	USA	A/Wisconsin/629-D02024/2009(H1N1)
HA	CY049851	H1N1	06/22/2009	Bolivia	A/La Paz/WR0096T/2009(H1N1)
HA	CY052258	H1N1	06/22/2009	USA	A/Texas/42221280/2009(H1N1)
HA	CY064596	H1N1	06/22/2009	USA	A/Boston/129/2009(H1N1)
HA	CY064548	H1N1	06/22/2009	USA	A/Boston/122/2009(H1N1)
HA	GU014810	H1N1	06/22/2009	Japan	A/Yamaguchi/23/2009(H1N1)
HA	CY120493	H1N1	06/22/2009	Hong Kong	A/Hong Kong/H090-661-V10/2009
HA	CY123946	H1N1	06/22/2009	Singapore	A/Singapore/ON368/2009
HA	CY123929	H1N1	06/22/2009	Singapore	A/Singapore/ON355/2009(H1N1)
HA	KC781885	H1N1	06/22/2009	USA	A/Utah/21/2009
HA	KC781712	H1N1	06/22/2009	USA	A/New York/98/2009
HA	CY070225	H1N1	06/22/2009	United Ki	A/England/617/2009
HA	CY123346	H1N1	06/22/2009	Singapore	A/Singapore/GP687/2009
HA	JN588774	H1N1	06/22/2009	Cambodia	A/Cambodia/T028/2009
HA	CY047907	H1N1	06/22/2009	Argentina	A/Argentina/9452/2009
HA	CY053971	H1N1	06/22/2009	Argentina	A/Argentina/HNRG5/2009(H1N1)
HA	CY053947	H1N1	06/22/2009	Argentina	A/Argentina/HNRG37/2009
HA	JN171924	H1N1	06/22/2009	Canada	A/Montreal/MTL083280/2009
HA	JN171923	H1N1	06/22/2009	Canada	A/Montreal/MTL083230/2009
HA	CY070174	H1N1	06/22/2009	United Ki	A/England/515/2009
HA	CY064668	H1N1	06/22/2009	USA	A/Boston/140/2009(H1N1)
HA	CY054819	H1N1	06/22/2009	USA	A/California/VRDL28/2009(H1N1)
HA	CY049372	H1N1	06/22/2009	Singapore	A/Singapore/ON355/2009(H1N1)
HA	CY053939	H1N1	06/22/2009	Argentina	A/Argentina/HNRG32/2009(H1N1)
HA	JN171922	H1N1	06/22/2009	Canada	A/Montreal/MTL083803/2009
HA	JN256797	H1N1	06/22/2009	Hong Kong	A/Hong Kong/H090-661-V1(0)/2009
HA	CY070167	H1N1	06/22/2009	United Ki	A/England/503/2009
HA	CY123969	H1N1	06/22/2009	Singapore	A/Singapore/ON406/2009
HA	CY054771	H1N1	06/22/2009	USA	A/California/VRDL21/2009(H1N1)
HA	GU014764	H1N1	06/22/2009	Japan	A/Niigata/700/2009(H1N1)
HA	CY055471	H1N1	06/22/2009	USA	A/California/VRDL18/2009(H1N1)
HA	CY075243	H1N1	06/22/2009	Chile	A/Chile/2362/2009(H1N1)
HA	KT180978	H1N1	06/22/2009	Singapore	A/Singapore/DMS4/2009
HA	KT181131	H1N1	06/22/2009	Singapore	A/Singapore/DMS562/2009
HA	KT181125	H1N1	06/22/2009	Singapore	A/Singapore/DMS516/2009
HA	CY047867	H1N1	06/22/2009	Argentina	A/Argentina/8994/2009
HA	CY049092	H1N1	06/22/2009	Singapore	A/Singapore/ON354/2009(H1N1)
HA	CY055463	H1N1	06/23/2009	USA	A/California/VRDL14/2009(H1N1)
HA	JN171881	H1N1	06/23/2009	Canada	A/Quebec/QC146767/2009
HA	CY054590	H1N1	06/23/2009	Thailand	A/Thailand/0316-00-N0/2009(H1N1)
HA	CY054592	H1N1	06/23/2009	Thailand	A/Thailand/0419-00-N0/2009(H1N1)
HA	CY051551	H1N1	06/23/2009	USA	A/New York/4434/2009(H1N1)
HA	JN171882	H1N1	06/23/2009	Canada	A/Quebec/QC146839/2009
HA	HQ228042	H1N1	06/23/2009	Finland	A/Finland/592/2009

HA	CY123953	H1N1	06/23/2009	Singapore	A/Singapore/ON392/2009(H1N1)
HA	CY065158	H1N1	06/23/2009	United Ki	A/England/541/2009
HA	CY070171	H1N1	06/23/2009	United Ki	A/England/511/2009
HA	CY047963	H1N1	06/23/2009	Argentina	A/Argentina/9711/2009
HA	CY047971	H1N1	06/23/2009	Argentina	A/Argentina/9721/2009
HA	CY047891	H1N1	06/23/2009	Argentina	A/Argentina/9333/2009
HA	CY070175	H1N1	06/23/2009	United Ki	A/England/516/2009
HA	CY047899	H1N1	06/23/2009	Argentina	A/Argentina/9384/2009
HA	JN171925	H1N1	06/23/2009	Canada	A/Montreal/MTL083987/2009
HA	KT181128	H1N1	06/23/2009	Singapore	A/Singapore/DMS547/2009
HA	CY053950	H1N1	06/23/2009	Argentina	A/Argentina/HNRG39/2009
HA	CY049300	H1N1	06/23/2009	Singapore	A/Singapore/ON384/2009(H1N1)
HA	KF849769	H1N1	06/23/2009	Thailand	A/Thailand/SirirajICRC_CBI_4/2009
HA	CY051479	H1N1	06/23/2009	USA	A/Wisconsin/629-S0410/2009(H1N1)
HA	CY123330	H1N1	06/23/2009	Singapore	A/Singapore/GP669/2009
HA	KF849768	H1N1	06/23/2009	Thailand	A/Thailand/SirirajICRC_CBI_3/2009
HA	KF849767	H1N1	06/23/2009	Thailand	A/Thailand/SirirajICRC_CBI_2/2009
HA	KF849770	H1N1	06/23/2009	Thailand	A/Thailand/SirirajICRC_CBI_7/2009
HA	JX625411	H1N1	06/23/2009	United Ki	A/England/92880002/2009
HA	CY123338	H1N1	06/23/2009	Singapore	A/Singapore/GP674/2009
HA	CY047883	H1N1	06/23/2009	Argentina	A/Argentina/9180/2009
HA	GU014800	H1N1	06/23/2009	Japan	A/Niigata/717/2009(H1N1)
HA	GQ365418	H1N1	06/23/2009	Japan	A/Fukushima/1/2009(H1N1)
HA	CY049292	H1N1	06/23/2009	Singapore	A/Singapore/ON380/2009(H1N1)
HA	CY047915	H1N1	06/24/2009	Argentina	A/Argentina/9576/2009
HA	CY054827	H1N1	06/24/2009	USA	A/California/VRDL29/2009(H1N1)
HA	CY064612	H1N1	06/24/2009	USA	A/Boston/131/2009(H1N1)
HA	CY107487	H1N1	06/24/2009	United Ki	A/Scotland/Edinburgh_17714/2009
HA	CY107479	H1N1	06/24/2009	United Ki	A/Scotland/Edinburgh_17645/2009
HA	CY123958	H1N1	06/24/2009	Singapore	A/Singapore/ON396/2009
HA	CY123965	H1N1	06/24/2009	Singapore	A/Singapore/ON402/2009(H1N1)
HA	CY070168	H1N1	06/24/2009	United Ki	A/England/507/2009
HA	CY107784	H1N1	06/24/2009	United Ki	A/Scotland/EastKilbride_420874/2009
HA	JN588780	H1N1	06/24/2009	Cambodia	A/Cambodia/T155/2009
HA	CY107465	H1N1	06/24/2009	United Ki	A/Scotland/Fife_17712/2009
HA	CY107819	H1N1	06/24/2009	United Ki	A/Scotland/StAndrews_17693/2009
HA	CY053942	H1N1	06/24/2009	Argentina	A/Argentina/HNRG33/2009(H1N1)
HA	CY061358	H1N1	06/24/2009	Argentina	A/Argentina/HNRG105/2009
HA	CY047955	H1N1	06/24/2009	Argentina	A/Argentina/9705/2009
HA	CY070198	H1N1	06/24/2009	United Ki	A/Scotland/20/2009
HA	CY054779	H1N1	06/24/2009	USA	A/California/VRDL22/2009(H1N1)
HA	KT180979	H1N1	06/24/2009	Singapore	A/Singapore/DMS5/2009
HA	CY053953	H1N1	06/24/2009	Argentina	A/Argentina/HNRG40/2009(H1N1)
HA	CY070169	H1N1	06/24/2009	United Ki	A/England/508/2009
HA	CY107804	H1N1	06/24/2009	United Ki	A/Scotland/EastKilbride_420881/2009
HA	HM569700	H1N1	06/24/2009	Argentina	A/Argentina/19911/2009(H1N1)
HA	CY070172	H1N1	06/24/2009	United Ki	A/England/512/2009
HA	CY123973	H1N1	06/24/2009	Singapore	A/Singapore/ON457/2009
HA	KT181099	H1N1	06/24/2009	Singapore	A/Singapore/DMS223/2009
HA	KF612093	H1N1	06/24/2009	Bolivia	A/Santa Cruz/46631/2009
HA	CY107790	H1N1	06/24/2009	United Ki	A/Scotland/EastKilbride_420875/2009
HA	CY107779	H1N1	06/24/2009	United Ki	A/Scotland/EastKilbride_420703/2009
HA	CY107797	H1N1	06/24/2009	United Ki	A/Scotland/EastKilbride_420880/2009
HA	CY107687	H1N1	06/24/2009	United Ki	A/Scotland/Glasgow_420768/2009

HA	CY107682	H1N1	06/24/2009	United Ki	A/Scotland/Glasgow_420755/2009
HA	CY083623	H1N1	06/24/2009	Peru	A/Trujillo/WRAIR9309F/2009
HA	CY049308	H1N1	06/24/2009	Singapore	A/Singapore/ON402/2009
HA	CY087216	H1N1	06/24/2009	USA	A/Houston/210S/2009
HA	CY047939	H1N1	06/24/2009	Argentina	A/Argentina/9588/2009
HA	CY047947	H1N1	06/24/2009	Argentina	A/Argentina/9597/2009
HA	CY049332	H1N1	06/24/2009	Singapore	A/Singapore/ON479/2009(H1N1)
HA	CY052146	H1N1	06/24/2009	USA	A/New York/4398/2009(H1N1)
HA	CY070173	H1N1	06/24/2009	United Ki	A/England/513/2009
HA	CY070181	H1N1	06/24/2009	United Ki	A/England/526/2009
HA	CY047923	H1N1	06/24/2009	Argentina	A/Argentina/9579/2009
HA	JN588781	H1N1	06/24/2009	Cambodia	A/Cambodia/T156/2009
HA	CY107693	H1N1	06/24/2009	United Ki	A/Scotland/Glasgow_422823/2009
HA	CY051471	H1N1	06/24/2009	USA	A/Wisconsin/629-S0435/2009(H1N1)
HA	CY049380	H1N1	06/25/2009	Singapore	A/Singapore/ON442/2009(H1N1)
HA	CY083575	H1N1	06/25/2009	Peru	A/Lima/WRAIR8648F/2009
HA	JN171884	H1N1	06/25/2009	Canada	A/Quebec/QC146934/2009
HA	CY075259	H1N1	06/25/2009	Chile	A/Chile/2911/2009(H1N1)
HA	CY049979	H1N1	06/25/2009	Dominican	A/Santo Domingo/WR1068N/2009(H1N1)
HA	CY052154	H1N1	06/25/2009	USA	A/New York/4401/2009(H1N1)
HA	CY052306	H1N1	06/25/2009	USA	A/Texas/42254309/2009(H1N1)
HA	GU014788	H1N1	06/25/2009	Japan	A/Hiroshima/230/2009(H1N1)
HA	CY083567	H1N1	06/25/2009	Peru	A/Lima/WRAIR8511F/2009
HA	JN588777	H1N1	06/25/2009	Cambodia	A/Cambodia/T075/2009
HA	JN588784	H1N1	06/25/2009	Cambodia	A/Cambodia/T272/2009
HA	KC781506	H1N1	06/25/2009	USA	A/Utah/22/2009
HA	CY070224	H1N1	06/25/2009	United Ki	A/England/92860065/2009
HA	JN171926	H1N1	06/25/2009	Canada	A/Montreal/MTL085214/2009
HA	JN171883	H1N1	06/25/2009	Canada	A/Quebec/QC146883/2009
HA	CY049324	H1N1	06/25/2009	Singapore	A/Singapore/ON478/2009(H1N1)
HA	CY075251	H1N1	06/25/2009	Chile	A/Chile/2909/2009(H1N1)
HA	KJ130196	H1N1	06/25/2009	New Zeala	A/New Zealand/2047/2009
HA	CY064676	H1N1	06/25/2009	USA	A/Boston/141/2009(H1N1)
HA	CY051527	H1N1	06/25/2009	USA	A/New York/4400/2009(H1N1)
HA	CY053968	H1N1	06/25/2009	Argentina	A/Argentina/HNRG48/2009
HA	CY044147	H1N1	06/25/2009	Colombia	A/Bogota/0466N/2009(H1N1)
HA	CY049939	H1N1	06/25/2009	Dominican	A/Santo Domingo/WR1056T/2009(H1N1)
HA	CY058204	H1N1	06/25/2009	Nicaragua	A/Managua/791.03/2009(H1N1)
HA	CY050182	H1N1	06/25/2009	USA	A/New York/4438/2009(H1N1)
HA	CY083527	H1N1	06/25/2009	Peru	A/Lima/WRAIR1696P/2009
HA	CY049148	H1N1	06/25/2009	Singapore	A/Singapore/ON504/2009(H1N1)
HA	CY057222	H1N1	06/25/2009	Nicaragua	A/Managua/611.01/2009(H1N1)
HA	CY049388	H1N1	06/25/2009	Singapore	A/Singapore/ON496/2009(H1N1)
HA	CY049100	H1N1	06/25/2009	Singapore	A/Singapore/ON511/2009(H1N1)
HA	CY049156	H1N1	06/25/2009	Singapore	A/Singapore/ON507/2009(H1N1)
HA	CY049396	H1N1	06/25/2009	Singapore	A/Singapore/ON506/2009(H1N1)
HA	CY070235	H1N1	06/25/2009	United Ki	A/England/92900231/2009
HA	CY049316	H1N1	06/25/2009	Singapore	A/Singapore/ON439/2009(H1N1)
HA	CY058540	H1N1	06/26/2009	Nicaragua	A/Managua/462.01/2009(H1N1)
HA	CY049172	H1N1	06/26/2009	Singapore	A/Singapore/ON588/2009(H1N1)
HA	CY075267	H1N1	06/26/2009	Chile	A/Chile/2994/2009(H1N1)
HA	CY075275	H1N1	06/26/2009	Chile	A/Chile/3019/2009(H1N1)
HA	CY052114	H1N1	06/26/2009	USA	A/Wisconsin/629-D01973/2009(H1N1)
HA	CY064516	H1N1	06/26/2009	USA	A/Boston/117/2009(H1N1)

HA	CY064564	H1N1	06/26/2009	USA	A/Boston/124/2009(H1N1)
HA	CY123996	H1N1	06/26/2009	Singapore	A/Singapore/ON496/2009
HA	CY124004	H1N1	06/26/2009	Singapore	A/Singapore/ON506/2009
HA	CY124012	H1N1	06/26/2009	Singapore	A/Singapore/ON520/2009
HA	CY124020	H1N1	06/26/2009	Singapore	A/Singapore/ON547/2009
HA	CY123980	H1N1	06/26/2009	Singapore	A/Singapore/ON477/2009
HA	JX625387	H1N1	06/26/2009	United Ki	A/England/506/2009
HA	CY075283	H1N1	06/26/2009	Chile	A/Chile/3056/2009
HA	CY053956	H1N1	06/26/2009	Argentina	A/Argentina/HNRG41/2009
HA	CY064895	H1N1	06/26/2009	USA	A/Boston/136/2009(H1N1)
HA	CY049180	H1N1	06/26/2009	Singapore	A/Singapore/ON590/2009(H1N1)
HA	CY049340	H1N1	06/26/2009	Singapore	A/Singapore/ON582/2009(H1N1)
HA	CY049188	H1N1	06/26/2009	Singapore	A/Singapore/ON594/2009(H1N1)
HA	CY070236	H1N1	06/26/2009	United Ki	A/England/92900239/2009
HA	CY070197	H1N1	06/26/2009	United Ki	A/Scotland/19/2009
HA	CY054595	H1N1	06/26/2009	Nepal	A/Nepal/NP09A-284/2009
HA	CY123988	H1N1	06/26/2009	Singapore	A/Singapore/ON479/2009
HA	CY070234	H1N1	06/26/2009	United Ki	A/England/92900124/2009
HA	CY054755	H1N1	06/26/2009	USA	A/California/VRDL19/2009(H1N1)
HA	KF612100	H1N1	06/26/2009	Bolivia	A/Santa Cruz/97831/2009
HA	CY064636	H1N1	06/26/2009	USA	A/Boston/134/2009(H1N1)
HA	CY049404	H1N1	06/26/2009	Singapore	A/Singapore/ON575/2009(H1N1)
HA	CY053166	H1N1	06/26/2009	USA	A/Houston/240S/2009(H1N1)
HA	CY049108	H1N1	06/26/2009	Singapore	A/Singapore/ON581/2009(H1N1)
HA	CY049348	H1N1	06/26/2009	Singapore	A/Singapore/ON593/2009(H1N1)
HA	CY049164	H1N1	06/26/2009	Singapore	A/Singapore/ON584/2009(H1N1)
HA	CY070176	H1N1	06/26/2009	United Ki	A/England/517/2009
HA	CY075291	H1N1	06/26/2009	Chile	A/Chile/3123/2009(H1N1)
HA	KC782290	H1N1	06/26/2009	USA	A/Texas/48/2009
HA	CY049196	H1N1	06/27/2009	Singapore	A/Singapore/ON621/2009(H1N1)
HA	CY051207	H1N1	06/27/2009	USA	A/Wisconsin/629-D01414/2009(H1N1)
HA	CY054811	H1N1	06/27/2009	USA	A/California/VRDL27/2009(H1N1)
HA	CY124035	H1N1	06/27/2009	Singapore	A/Singapore/ON580/2009
HA	CY083455	H1N1	06/27/2009	Peru	A/Lima/WRAIR1687P/2009
HA	CY049212	H1N1	06/27/2009	Singapore	A/Singapore/ON635/2009(H1N1)
HA	CY075914	H1N1	06/27/2009	India	A/Che/NIV246/2009
HA	CY103923	H1N1	06/27/2009	Brazil	A/Pernambuco/42/2009
HA	GU290047	H1N1	06/27/2009	Czech Rep	A/Prague/197-81395/2009(H1N1)
HA	CY124028	H1N1	06/27/2009	Singapore	A/Singapore/ON575/2009
HA	CY075913	H1N1	06/27/2009	India	A/Che/NIV245/2009
HA	CY049220	H1N1	06/27/2009	Singapore	A/Singapore/ON639/2009(H1N1)
HA	CY083583	H1N1	06/27/2009	Peru	A/Lima/WRAIR8689F/2009(H1N1)
HA	CY061369	H1N1	06/27/2009	Argentina	A/Argentina/HNRG106/2009
HA	CY049204	H1N1	06/27/2009	Singapore	A/Singapore/ON628/2009(H1N1)
HA	CY070177	H1N1	06/27/2009	United Ki	A/England/518/2009
HA	CY053959	H1N1	06/27/2009	Argentina	A/Argentina/HNRG42/2009
HA	CY070232	H1N1	06/28/2009	United Ki	A/England/973/2009
HA	CY046952	H1N1	06/28/2009	Norway	A/Norway/2010/2009(H1N1)
HA	CY054835	H1N1	06/28/2009	USA	A/California/VRDL30/2009(H1N1)
HA	CY052186	H1N1	06/28/2009	USA	A/Texas/42281289/2009(H1N1)
HA	CY070218	H1N1	06/28/2009	United Ki	A/England/92840014/2009
HA	HQ228043	H1N1	06/28/2009	Finland	A/Finland/593/2009
HA	JN171927	H1N1	06/28/2009	Canada	A/Montreal/MTL085707/2009
HA	CY070199	H1N1	06/28/2009	United Ki	A/Scotland/21/2009

HA	KT180980	H1N1	06/28/2009	Singapore	A/Singapore/DMS7/2009
HA	CY052399	H1N1	06/29/2009	USA	A/Texas/42291877/2009(H1N1)
HA	CY058196	H1N1	06/29/2009	Nicaragua	A/Managua/1127.02/2009(H1N1)
HA	CY070200	H1N1	06/29/2009	United Ki	A/Scotland/22/2009
HA	GQ457519	H1N1	06/29/2009	China	A/Changsha/78/2009(H1N1)
HA	CY043078	H1N1	06/29/2009	Japan	A/Japan/1070/2009(H1N1)
HA	CY103936	H1N1	06/29/2009	Brazil	A/Pernambuco/66/2009
HA	GU014768	H1N1	06/29/2009	Japan	A/Saitama/85/2009(H1N1)
HA	CY070233	H1N1	06/29/2009	United Ki	A/England/92900097/2009
HA	CY070180	H1N1	06/29/2009	United Ki	A/England/525/2009
HA	CY070179	H1N1	06/29/2009	United Ki	A/England/524/2009
HA	CY070170	H1N1	06/29/2009	United Ki	A/England/510/2009
HA	CY053907	H1N1	06/29/2009	Argentina	A/Argentina/HNRG16/2009
HA	CY070192	H1N1	06/29/2009	United Ki	A/England/540/2009
HA	CY070191	H1N1	06/29/2009	United Ki	A/England/539/2009
HA	CY070178	H1N1	06/29/2009	United Ki	A/England/520/2009
HA	CY123354	H1N1	06/29/2009	Singapore	A/Singapore/GP835/2009
HA	CY049995	H1N1	06/29/2009	Mexico	A/Mexico City/WR1100N/2009(H1N1)
HA	CY064508	H1N1	06/29/2009	USA	A/Boston/116/2009(H1N1)
HA	CY054787	H1N1	06/29/2009	USA	A/California/VRDL23/2009(H1N1)
HA	CY058180	H1N1	06/29/2009	Nicaragua	A/Managua/3189.01/2009(H1N1)
HA	CY063558	H1N1	06/29/2009	USA	A/Boston/152/2009(H1N1)
HA	CY069757	H1N1	06/30/2009	United Ki	A/England/633/2009
HA	CY064684	H1N1	06/30/2009	USA	A/Boston/142/2009(H1N1)
HA	CY051215	H1N1	06/30/2009	USA	A/Wisconsin/629-D02292/2009(H1N1)
HA	JX625395	H1N1	06/30/2009	United Ki	A/England/527/2009
HA	CY047754	H1N1	06/30/2009	Argentina	A/Argentina/12508/2009
HA	CY047752	H1N1	06/30/2009	Argentina	A/Argentina/12295/2009
HA	CY052045	H1N1	06/30/2009	Brazil	A/Parana/2529/2009(H1N1)
HA	CY075307	H1N1	06/30/2009	Chile	A/Chile/3242/2009(H1N1)
HA	CY075315	H1N1	06/30/2009	Chile	A/Chile/3244/2009(H1N1)
HA	CY073418	H1N1	06/30/2009	USA	A/San Diego/WR1640P/2009(H1N1)
HA	CY054867	H1N1	06/30/2009	USA	A/California/VRDL34/2009(H1N1)
HA	CY047759	H1N1	06/30/2009	Argentina	A/Argentina/13777/2009
HA	CY052202	H1N1	06/30/2009	USA	A/Texas/42303371/2009(H1N1)
HA	CY070239	H1N1	06/30/2009	United Ki	A/England/92900287/2009
HA	CY070237	H1N1	06/30/2009	United Ki	A/England/92900284/2009
HA	CY051487	H1N1	06/30/2009	USA	A/Wisconsin/629-S0464/2009(H1N1)
HA	GU014784	H1N1	06/30/2009	Japan	A/Hiroshima/216/2009(H1N1)
HA	CY051647	H1N1	06/30/2009	USA	A/New York/4691/2009(H1N1)
HA	CY120101	H1N1	06/30/2009	Hong Kong	A/Hong Kong/H090-663-V10/2009
HA	CY123362	H1N1	06/30/2009	Singapore	A/Singapore/GP875/2009
HA	CY069749	H1N1	06/30/2009	United Ki	A/England/527/2009
HA	CY070247	H1N1	06/30/2009	United Ki	A/England/619/2009
HA	CY070211	H1N1	06/30/2009	United Ki	A/England/611/2009
HA	CY070238	H1N1	06/30/2009	United Ki	A/England/92900286/2009
HA	JN256798	H1N1	06/30/2009	Hong Kong	A/Hong Kong/H090-663-V1(0)/2009
HA	CY111619	H1N1	06/30/2009	Hong Kong	A/Hong Kong/H090-663-V10/2009
HA	CY053962	H1N1	06/30/2009	Argentina	A/Argentina/HNRG44/2009
HA	GQ392017	H1N1	06/30/2009	Singapore	A/Singapore/TLL01/2009
HA	CY070216	H1N1	06/30/2009	United Ki	A/England/606/2009
HA	CY054795	H1N1	06/30/2009	USA	A/California/VRDL25/2009(H1N1)
HA	CY058188	H1N1	06/30/2009	Nicaragua	A/Managua/5586.01/2009(H1N1)
HA	CY075323	H1N1	06/30/2009	Chile	A/Chile/3295/2009(H1N1)

HA	CY049963	H1N1	06/30/2009	Dominicar	A/Santo Domingo/WR1059N/2009(H1N1)
HA	CY075299	H1N1	06/30/2009	Chile	A/Chile/3220/2009(H1N1)
HA	CY047744	H1N1	07/2009	Taiwan	A/Taiwan/526/2009
HA	GU560025	H1N1	07/2009	Kazakhsta	A/Astana/830/2009(H1N1)
HA	GU369648	H1N1	07/2009	Turkey	A/Ankara/05/2009(H1N1)
HA	AB568198	H1N1	07/2009	Japan	A/Kobe/91586/2009
HA	AB568183	H1N1	07/2009	Japan	A/Kobe/91569/2009
HA	AB568203	H1N1	07/2009	Japan	A/Kobe/91615/2009
HA	AB568188	H1N1	07/2009	Japan	A/Kobe/91571/2009
HA	AB568168	H1N1	07/2009	Japan	A/Kobe/91533/2009
HA	JN381214	H1N1	07/2009	Taiwan	A/Taiwan/71433/2009
HA	JN381213	H1N1	07/2009	Taiwan	A/Taiwan/71354/2009
HA	JN381215	H1N1	07/2009	Taiwan	A/Taiwan/71526/2009
HA	AB568178	H1N1	07/2009	Japan	A/Kobe/91559/2009
HA	HM204570	H1N1	07/2009	India	A/Mum/NIV968/2009
HA	GU117765	H1N1	07/2009	Japan	A/Nagasaki/I01/2009(H1N1)
HA	AB572352	H1N1	07/2009	Zambia	A/Zambia/CZC1/2009(H1N1)
HA	AB568193	H1N1	07/2009	Japan	A/Kobe/91585/2009
HA	AB568173	H1N1	07/2009	Japan	A/Kobe/91537/2009
HA	AB568213	H1N1	07/2009	Japan	A/Kobe/91709/2009
HA	AB568208	H1N1	07/2009	Japan	A/Kobe/91660/2009
HA	HM204569	H1N1	07/2009	India	A/Che/NIV658/2009
HA	CY050363	H1N1	07/2009	Singapore	A/Singapore/279M/2009(H1N1)
HA	JN381216	H1N1	07/2009	Taiwan	A/Taiwan/97338/2009
HA	GU369646	H1N1	07/2009	Turkey	A/Ankara/03/2009(H1N1)
HA	GQ866928	H1N1	07/2009	Thailand	A/Thailand/CU-H106/2009(H1N1)
HA	HM204568	H1N1	07/2009	India	A/Pune/NIV807/2009
HA	CY075355	H1N1	07/01/2009	Chile	A/Chile/3375/2009(H1N1)
HA	CY073410	H1N1	07/01/2009	USA	A/San Diego/WR1639P/2009(H1N1)
HA	CY054851	H1N1	07/01/2009	USA	A/California/VRDL32/2009(H1N1)
HA	CY054907	H1N1	07/01/2009	USA	A/California/VRDL40/2009(H1N1)
HA	CY054947	H1N1	07/01/2009	USA	A/California/VRDL50/2009(H1N1)
HA	CY065174	H1N1	07/01/2009	United Ki	A/England/634/2009
HA	GQ421201	H1N1	07/01/2009	Italy	A/Ancona/04/2009(H1N1)
HA	CY051831	H1N1	07/01/2009	USA	A/Texas/43011033/2009(H1N1)
HA	CY070212	H1N1	07/01/2009	United Ki	A/England/92820897/2009
HA	CY070182	H1N1	07/01/2009	United Ki	A/England/528/2009
HA	CY070210	H1N1	07/01/2009	United Ki	A/England/605/2009
HA	CY070213	H1N1	07/01/2009	United Ki	A/England/92820911/2009
HA	CY070187	H1N1	07/01/2009	United Ki	A/England/533/2009
HA	CY070203	H1N1	07/01/2009	United Ki	A/England/665/2009
HA	CY070196	H1N1	07/01/2009	United Ki	A/England/545/2009
HA	CY053913	H1N1	07/01/2009	Argentina	A/Argentina/HNRG18/2009
HA	CY083591	H1N1	07/01/2009	Peru	A/Lima/WRAIR8893F/2009(H1N1)
HA	CY070184	H1N1	07/01/2009	United Ki	A/England/530/2009
HA	CY070240	H1N1	07/01/2009	United Ki	A/England/974/2009
HA	CY070215	H1N1	07/01/2009	United Ki	A/England/92820918/2009
HA	CY070186	H1N1	07/01/2009	United Ki	A/England/532/2009
HA	CY063542	H1N1	07/01/2009	USA	A/Boston/149/2009(H1N1)
HA	CY047770	H1N1	07/01/2009	Argentina	A/Argentina/14591/2009
HA	KF612092	H1N1	07/01/2009	Bolivia	A/Santa Cruz/12541/2009
HA	KP637526	H1N1	07/01/2009	Thailand	A/Thailand/SN08270/2009
HA	CY070189	H1N1	07/01/2009	United Ki	A/England/537/2009
HA	CY070185	H1N1	07/01/2009	United Ki	A/England/531/2009

HA	JX625403	H1N1	07/01/2009	United Ki	A/England/685/2009
HA	JX625427	H1N1	07/01/2009	United Ki	A/England/92920018/2009
HA	CY070190	H1N1	07/01/2009	United Ki	A/England/538/2009
HA	CY107205	H1N1	07/01/2009	United Ki	A/Scotland/Paisley_425827/2009
HA	JX625419	H1N1	07/01/2009	United Ki	A/England/667/2009
HA	CY047772	H1N1	07/01/2009	Argentina	A/Argentina/15765/2009
HA	CY047766	H1N1	07/01/2009	Argentina	A/Argentina/14192/2009
HA	CY070188	H1N1	07/01/2009	United Ki	A/England/535/2009
HA	CY070228	H1N1	07/01/2009	United Ki	A/England/667/2009
HA	CY070254	H1N1	07/01/2009	United Ki	A/England/92920018/2009
HA	CY075331	H1N1	07/01/2009	Chile	A/Chile/3349/2009(H1N1)
HA	CY070298	H1N1	07/01/2009	United Ki	A/Scotland/93040048/2009
HA	CY070183	H1N1	07/01/2009	United Ki	A/England/529/2009
HA	CY070201	H1N1	07/01/2009	United Ki	A/England/546/2009
HA	CY054931	H1N1	07/01/2009	USA	A/California/VRDL45/2009(H1N1)
HA	CY063780	H1N1	07/01/2009	Singapore	A/Singapore/ON0801/2009(H1N1)
HA	CY051575	H1N1	07/01/2009	USA	A/New York/4500/2009(H1N1)
HA	CY107458	H1N1	07/01/2009	United Ki	A/Scotland/Dumfries_425403/2009
HA	KF612096	H1N1	07/01/2009	Bolivia	A/Santa Cruz/21641/2009
HA	GQ465672	H1N1	07/01/2009	Canada	A/Canada-SK/RV2486/2009(H1N1)
HA	CY053919	H1N1	07/01/2009	Argentina	A/Argentina/HNRG21/2009(H1N1)
HA	CY051567	H1N1	07/01/2009	USA	A/New York/4476/2009(H1N1)
HA	GU014782	H1N1	07/01/2009	Japan	A/Hiroshima/207/2009(H1N1)
HA	CY063518	H1N1	07/01/2009	USA	A/Boston/146/2009(H1N1)
HA	CY075347	H1N1	07/01/2009	Chile	A/Chile/3369/2009(H1N1)
HA	CY107811	H1N1	07/01/2009	United Ki	A/Scotland/EastKilbride_425383/2009
HA	CY075339	H1N1	07/01/2009	Chile	A/Chile/3361/2009(H1N1)
HA	CY075371	H1N1	07/02/2009	Chile	A/Chile/3553/2009(H1N1)
HA	CY111635	H1N1	07/02/2009	Hong Kong	A/Hong Kong/H090-667-V10/2009
HA	CY054859	H1N1	07/02/2009	USA	A/California/VRDL33/2009(H1N1)
HA	CY049947	H1N1	07/02/2009	Dominican	A/Santo Domingo/WR1057N/2009(H1N1)
HA	CY111627	H1N1	07/02/2009	Hong Kong	A/Hong Kong/H090-665-V10/2009
HA	CY103941	H1N1	07/02/2009	Brazil	A/Pernambuco/82/2009
HA	CY070294	H1N1	07/02/2009	United Ki	A/England/93040014/2009
HA	CY070222	H1N1	07/02/2009	United Ki	A/England/92860040/2009
HA	CY061380	H1N1	07/02/2009	Argentina	A/Argentina/HNRG107/2009
HA	CY054596	H1N1	07/02/2009	Nepal	A/Nepal/NP09A-294/2009
HA	CY070195	H1N1	07/02/2009	United Ki	A/England/544/2009
HA	CY070242	H1N1	07/02/2009	United Ki	A/England/92900351/2009
HA	CY070204	H1N1	07/02/2009	United Ki	A/England/610/2009
HA	CY072774	H1N1	07/02/2009	Nicaragua	A/Managua/5235.03/2009(H1N1)
HA	CY054597	H1N1	07/02/2009	Nepal	A/Nepal/NP09A-295/2009
HA	CY124059	H1N1	07/02/2009	Singapore	A/Singapore/ON802/2009(H1N1)
HA	KF612094	H1N1	07/02/2009	Bolivia	A/Santa Cruz/94841/2009
HA	CY111643	H1N1	07/02/2009	Hong Kong	A/Hong Kong/H090-667-V23/2009
HA	CY124115	H1N1	07/02/2009	Singapore	A/Singapore/ON811/2009
HA	CY120062	H1N1	07/02/2009	Hong Kong	A/Hong Kong/H090-667-V23/2009
HA	CY124087	H1N1	07/02/2009	Singapore	A/Singapore/ON806/2009(H1N1)
HA	CY070194	H1N1	07/02/2009	United Ki	A/England/543/2009
HA	CY120109	H1N1	07/02/2009	Hong Kong	A/Hong Kong/H090-665-V10/2009
HA	CY124100	H1N1	07/02/2009	Singapore	A/Singapore/ON808/2009
HA	CY124072	H1N1	07/02/2009	Singapore	A/Singapore/ON804/2009
HA	CY124079	H1N1	07/02/2009	Singapore	A/Singapore/ON805/2009(H1N1)
HA	CY124043	H1N1	07/02/2009	Singapore	A/Singapore/ON799/2009

HA	CY124051	H1N1	07/02/2009	Singapore	A/Singapore/ON800/2009
HA	JN256799	H1N1	07/02/2009	Hong Kong	A/Hong Kong/H090-665-V1(0)/2009
HA	CY070219	H1N1	07/02/2009	United Ki	A/England/92840702/2009
HA	CY075363	H1N1	07/02/2009	Chile	A/Chile/3467/2009(H1N1)
HA	CY054891	H1N1	07/02/2009	USA	A/California/VRDL38/2009(H1N1)
HA	CY124123	H1N1	07/02/2009	Singapore	A/Singapore/ON812/2009
HA	CY124095	H1N1	07/02/2009	Singapore	A/Singapore/ON807/2009(H1N1)
HA	CY124107	H1N1	07/02/2009	Singapore	A/Singapore/ON809/2009
HA	CY070214	H1N1	07/02/2009	United Ki	A/England/612/2009
HA	CY124067	H1N1	07/02/2009	Singapore	A/Singapore/ON803/2009(H1N1)
HA	JN256801	H1N1	07/02/2009	Hong Kong	A/Hong Kong/H090-667-V2(3)/2009
HA	CY070217	H1N1	07/02/2009	United Ki	A/England/92820956/2009
HA	CY070193	H1N1	07/02/2009	United Ki	A/England/542/2009
HA	CY070208	H1N1	07/02/2009	United Ki	A/England/666/2009
HA	CY055196	H1N1	07/02/2009	Malaysia	A/Malaysia/3758/2009
HA	CY049955	H1N1	07/02/2009	Dominican	A/Santo Domingo/WR1058N/2009(H1N1)
HA	JN256800	H1N1	07/02/2009	Hong Kong	A/Hong Kong/H090-667-V1(0)/2009
HA	CY052046	H1N1	07/02/2009	Brazil	A/Rio de Janeiro/2459/2009(H1N1)
HA	CY120117	H1N1	07/02/2009	Hong Kong	A/Hong Kong/H090-667-V10/2009
HA	JN588785	H1N1	07/02/2009	Cambodia	A/Cambodia/T282/2009
HA	CY072758	H1N1	07/02/2009	Nicaragua	A/Managua/435.01/2009(H1N1)
HA	CY075672	H1N1	07/02/2009	Thailand	A/Thailand/CU-H104/2009(H1N1)
HA	CY075379	H1N1	07/03/2009	Chile	A/Chile/3586/2009(H1N1)
HA	CY083463	H1N1	07/03/2009	Peru	A/Lima/WRAIR1688P/2009
HA	CY070251	H1N1	07/03/2009	United Ki	A/England/92900391/2009
HA	CY070205	H1N1	07/03/2009	United Ki	A/England/92800056/2009
HA	GQ414768	H1N1	07/03/2009	Brazil	A/Sao Paulo/43812/2009
HA	CY083615	H1N1	07/03/2009	Peru	A/Lima/WRAIR9202F/2009(H1N1)
HA	CY070207	H1N1	07/03/2009	United Ki	A/England/603/2009
HA	HQ228044	H1N1	07/03/2009	Finland	A/Finland/594/2009
HA	CY070206	H1N1	07/03/2009	United Ki	A/England/602/2009
HA	CY087024	H1N1	07/03/2009	USA	A/New York/4557/2009(H1N1)
HA	CY124146	H1N1	07/03/2009	Singapore	A/Singapore/ON822/2009
HA	CY122555	H1N1	07/03/2009	Singapore	A/Singapore/GP1020/2009
HA	CY124131	H1N1	07/03/2009	Singapore	A/Singapore/ON816/2009
HA	CY124139	H1N1	07/03/2009	Singapore	A/Singapore/ON819/2009
HA	HQ228047	H1N1	07/03/2009	Finland	A/Finland/597/2009
HA	GQ502906	H1N1	07/03/2009	Canada	A/Toronto/R8557/2009(H1N1)
HA	GU014786	H1N1	07/03/2009	Japan	A/Hiroshima/220/2009(H1N1)
HA	CY050263	H1N1	07/04/2009	China	A/Qingdao/283/2009(H1N1)
HA	JN171885	H1N1	07/04/2009	Canada	A/Quebec/QC147411/2009
HA	CY049236	H1N1	07/04/2009	Singapore	A/Singapore/ON896/2009(H1N1)
HA	CY049427	H1N1	07/04/2009	Singapore	A/Singapore/ON905/2009(H1N1)
HA	CY049252	H1N1	07/04/2009	Singapore	A/Singapore/ON901/2009(H1N1)
HA	CY049435	H1N1	07/04/2009	Singapore	A/Singapore/ON907/2009(H1N1)
HA	CY049443	H1N1	07/04/2009	Singapore	A/Singapore/ON912/2009(H1N1)
HA	CY062517	H1N1	07/04/2009	Mexico	A/Mexico city/CIA5/2009(H1N1)
HA	CY062491	H1N1	07/04/2009	Mexico	A/Mexico city/CIA1/2009(H1N1)
HA	CY055200	H1N1	07/04/2009	Malaysia	A/Malaysia/4039/2009
HA	CY049419	H1N1	07/04/2009	Singapore	A/Singapore/ON903/2009(H1N1)
HA	CY049244	H1N1	07/04/2009	Singapore	A/Singapore/ON899/2009(H1N1)
HA	HQ228045	H1N1	07/04/2009	Finland	A/Finland/595/2009
HA	CY049228	H1N1	07/04/2009	Singapore	A/Singapore/ON894/2009(H1N1)
HA	HQ228046	H1N1	07/04/2009	Finland	A/Finland/596/2009

HA	CY055198	H1N1	07/04/2009	Malaysia	A/Malaysia/4038/2009
HA	CY050264	H1N1	07/05/2009	China	A/Qingdao/292/2009(H1N1)
HA	AB530161	H1N1	07/05/2009	Japan	A/Nagasaki/HA-4/2009(H1N1)
HA	CY054875	H1N1	07/05/2009	USA	A/California/VRDL35/2009(H1N1)
HA	CY061542	H1N1	07/05/2009	Argentina	A/Argentina/HNRG91/2009
HA	CY070277	H1N1	07/05/2009	United Ki	A/England/671/2009
HA	CY061540	H1N1	07/05/2009	Argentina	A/Argentina/HNRG84/2009
HA	KT180981	H1N1	07/05/2009	Singapore	A/Singapore/DMS10/2009
HA	CY122563	H1N1	07/05/2009	Singapore	A/Singapore/GP1022/2009
HA	CY049459	H1N1	07/05/2009	Singapore	A/Singapore/ON925/2009(H1N1)
HA	CY049451	H1N1	07/05/2009	Singapore	A/Singapore/ON921/2009(H1N1)
HA	CY049116	H1N1	07/05/2009	Singapore	A/Singapore/ON923/2009(H1N1)
HA	JX625451	H1N1	07/05/2009	United Ki	A/England/671/2009
HA	CY075403	H1N1	07/06/2009	Chile	A/Chile/3819/2009(H1N1)
HA	CY075395	H1N1	07/06/2009	Chile	A/Chile/3766/2009(H1N1)
HA	CY075720	H1N1	07/06/2009	Thailand	A/Thailand/CU-H133/2009(H1N1)
HA	CY049276	H1N1	07/06/2009	Singapore	A/Singapore/ON980/2009(H1N1)
HA	CY070300	H1N1	07/06/2009	United Ki	A/Scotland/93040050/2009
HA	CY083344	H1N1	07/06/2009	USA	A/San Diego/WRAIR1656P/2009
HA	JN171928	H1N1	07/06/2009	Canada	A/Montreal/MTL088751/2009
HA	CY062505	H1N1	07/06/2009	Mexico	A/Mexico city/CIA3/2009(H1N1)
HA	CY069250	H1N1	07/06/2009	Nicaragua	A/Managua/4943.09/2009(H1N1)
HA	CY072566	H1N1	07/06/2009	Nicaragua	A/Managua/4197.01/2009(H1N1)
HA	KC781697	H1N1	07/06/2009	USA	A/Georgia/08/2009
HA	KC781606	H1N1	07/06/2009	USA	A/Arizona/14/2009
HA	KF667880	H1N1	07/06/2009	Singapore	A/Singapore/376Y/2009
HA	CY045511	H1N1	07/06/2009	Canada	A/Toronto/R8564/2009(H1N1)
HA	CY122587	H1N1	07/06/2009	Singapore	A/Singapore/GP1092/2009
HA	CY065166	H1N1	07/06/2009	United Ki	A/England/92860032/2009
HA	CY122603	H1N1	07/06/2009	Singapore	A/Singapore/GP1097/2009
HA	CY075702	H1N1	07/06/2009	Thailand	A/Thailand/CU-H128/2009(H1N1)
HA	CY070209	H1N1	07/06/2009	United Ki	A/England/604/2009
HA	CY070202	H1N1	07/06/2009	United Ki	A/England/92800004/2009
HA	CY075708	H1N1	07/06/2009	Thailand	A/Thailand/CU-H130/2009(H1N1)
HA	CY054923	H1N1	07/06/2009	USA	A/California/VRDL43/2009(H1N1)
HA	CY062498	H1N1	07/06/2009	Mexico	A/Mexico city/CIA2/2009(H1N1)
HA	CY122611	H1N1	07/06/2009	Singapore	A/Singapore/GP1099/2009
HA	CY083631	H1N1	07/06/2009	Peru	A/Arequipa/WRAIR9939F/2009
HA	CY063550	H1N1	07/06/2009	USA	A/Boston/151/2009(H1N1)
HA	CY049260	H1N1	07/06/2009	Singapore	A/Singapore/ON975/2009(H1N1)
HA	CY049268	H1N1	07/06/2009	Singapore	A/Singapore/ON978/2009(H1N1)
HA	CY049284	H1N1	07/06/2009	Singapore	A/Singapore/ON981/2009(H1N1)
HA	CY122687	H1N1	07/06/2009	Singapore	A/Singapore/GP1137/2009
HA	CY122595	H1N1	07/06/2009	Singapore	A/Singapore/GP1094/2009
HA	CY075387	H1N1	07/06/2009	Chile	A/Chile/3760/2009(H1N1)
HA	CY075714	H1N1	07/06/2009	Thailand	A/Thailand/CU-H132/2009(H1N1)
HA	CY075744	H1N1	07/07/2009	Thailand	A/Thailand/CU-H140/2009(H1N1)
HA	CY070302	H1N1	07/07/2009	United Ki	A/Scotland/93040058/2009
HA	GU014748	H1N1	07/07/2009	Japan	A/Gifu-C/67/2009(H1N1)
HA	CY070227	H1N1	07/07/2009	United Ki	A/England/632/2009
HA	CY070248	H1N1	07/07/2009	United Ki	A/England/92900378/2009
HA	CY070314	H1N1	07/07/2009	United Ki	A/England/982/2009
HA	CY075920	H1N1	07/07/2009	India	A/Mum/NIV398/2009(H1N1)
HA	CY070221	H1N1	07/07/2009	United Ki	A/England/92860024/2009

HA	CY070223	H1N1	07/07/2009	United Ki	A/England/92860057/2009
HA	CY070246	H1N1	07/07/2009	United Ki	A/England/92900376/2009
HA	CY070220	H1N1	07/07/2009	United Ki	A/England/607/2009
HA	CY122619	H1N1	07/07/2009	Singapore	A/Singapore/GP1102/2009
HA	CY070249	H1N1	07/07/2009	United Ki	A/England/92900383/2009
HA	CY045954	H1N1	07/07/2009	Canada	A/Toronto/T0106/2009
HA	KC781859	H1N1	07/07/2009	USA	A/Washington/52/2009
HA	CY053158	H1N1	07/07/2009	USA	A/Houston/23H/2009(H1N1)
HA	JN256802	H1N1	07/07/2009	Hong Kong	A/Hong Kong/H090-678-V1(0)/2009
HA	JN588778	H1N1	07/07/2009	Cambodia	A/Cambodia/T093/2009
HA	CY075732	H1N1	07/07/2009	Thailand	A/Thailand/CU-H137/2009(H1N1)
HA	CY075726	H1N1	07/07/2009	Thailand	A/Thailand/CU-H134/2009(H1N1)
HA	CY075411	H1N1	07/07/2009	Chile	A/Chile/3905/2009(H1N1)
HA	CY075419	H1N1	07/07/2009	Chile	A/Chile/3935/2009(H1N1)
HA	CY075738	H1N1	07/07/2009	Thailand	A/Thailand/CU-H139/2009(H1N1)
HA	CY122647	H1N1	07/07/2009	Singapore	A/Singapore/GP1122/2009
HA	CY122571	H1N1	07/07/2009	Singapore	A/Singapore/GP1087/2009
HA	CY122579	H1N1	07/07/2009	Singapore	A/Singapore/GP1090/2009
HA	CY073885	H1N1	07/07/2009	Nicaragua	A/Managua/2729.02/2009(H1N1)
HA	CY122655	H1N1	07/07/2009	Singapore	A/Singapore/GP1124/2009
HA	GU014766	H1N1	07/07/2009	Japan	A/Niigata/749/2009(H1N1)
HA	CY120125	H1N1	07/07/2009	Hong Kong	A/Hong Kong/H090-678-V10/2009
HA	CY122679	H1N1	07/07/2009	Singapore	A/Singapore/GP1136/2009
HA	CY111651	H1N1	07/07/2009	Hong Kong	A/Hong Kong/H090-678-V10/2009
HA	HQ228048	H1N1	07/07/2009	Finland	A/Finland/598/2009
HA	CY063534	H1N1	07/07/2009	USA	A/Boston/148/2009(H1N1)
HA	CY111659	H1N1	07/08/2009	Hong Kong	A/Hong Kong/H090-681-V10/2009
HA	KT180989	H1N1	07/08/2009	Singapore	A/Singapore/DMS11/2009
HA	CY051607	H1N1	07/08/2009	USA	A/New York/4567/2009(H1N1)
HA	CY070244	H1N1	07/08/2009	United Ki	A/England/631/2009
HA	CY070252	H1N1	07/08/2009	United Ki	A/England/92900398/2009
HA	CY070253	H1N1	07/08/2009	United Ki	A/England/636/2009
HA	CY070241	H1N1	07/08/2009	United Ki	A/England/92900350/2009
HA	CY083096	H1N1	07/08/2009	Peru	A/Lima/WRAIR0519F/2009
HA	CY051591	H1N1	07/08/2009	USA	A/New York/4565/2009(H1N1)
HA	KC780219	H1N1	07/08/2009	USA	A/Utah/33/2009
HA	CY122731	H1N1	07/08/2009	Singapore	A/Singapore/GP1163/2009
HA	CY122695	H1N1	07/08/2009	Singapore	A/Singapore/GP1142/2009
HA	CY122624	H1N1	07/08/2009	Singapore	A/Singapore/GP1107/2009
HA	CY122747	H1N1	07/08/2009	Singapore	A/Singapore/GP1167/2009
HA	CY122663	H1N1	07/08/2009	Singapore	A/Singapore/GP1127/2009
HA	CY122671	H1N1	07/08/2009	Singapore	A/Singapore/GP1132/2009
HA	CY122711	H1N1	07/08/2009	Singapore	A/Singapore/GP1147/2009
HA	CY122631	H1N1	07/08/2009	Singapore	A/Singapore/GP1114/2009
HA	CY122763	H1N1	07/08/2009	Singapore	A/Singapore/GP1174/2009
HA	CY122703	H1N1	07/08/2009	Singapore	A/Singapore/GP1146/2009
HA	KC782220	H1N1	07/08/2009	USA	A/Alaska/30/2009
HA	CY070245	H1N1	07/08/2009	United Ki	A/England/92900362/2009
HA	CY071474	H1N1	07/08/2009	USA	A/California/WR1313P/2009(H1N1)
HA	CY064692	H1N1	07/08/2009	USA	A/Boston/143/2009(H1N1)
HA	CY120133	H1N1	07/08/2009	Hong Kong	A/Hong Kong/H090-681-V10/2009
HA	CY070310	H1N1	07/08/2009	United Ki	A/England/979/2009
HA	CY122639	H1N1	07/08/2009	Singapore	A/Singapore/GP1120/2009
HA	CY122716	H1N1	07/08/2009	Singapore	A/Singapore/GP1156/2009

HA	CY122720	H1N1	07/08/2009	Singapore	A/Singapore/GP1160/2009
HA	CY071522	H1N1	07/08/2009	USA	A/California/WR1319P/2009(H1N1)
HA	CY122724	H1N1	07/08/2009	Singapore	A/Singapore/GP1162/2009
HA	CY051599	H1N1	07/08/2009	USA	A/New York/4566/2009(H1N1)
HA	CY122739	H1N1	07/08/2009	Singapore	A/Singapore/GP1164/2009
HA	CY122771	H1N1	07/08/2009	Singapore	A/Singapore/GP1178/2009
HA	CY122755	H1N1	07/08/2009	Singapore	A/Singapore/GP1169/2009
HA	JN256803	H1N1	07/08/2009	Hong Kong	A/Hong Kong/H090-681-V1(0)/2009
HA	CY050190	H1N1	07/08/2009	USA	A/New York/4576/2009(H1N1)
HA	KC780277	H1N1	07/08/2009	USA	A/Utah/40/2009
HA	GU014756	H1N1	07/08/2009	Myanmar	A/Myanmar/JP90/2009(H1N1)
HA	CY050266	H1N1	07/09/2009	China	A/Qingdao/322/2009(H1N1)
HA	CY049483	H1N1	07/09/2009	Singapore	A/Singapore/ON1067/2009(H1N1)
HA	CY070301	H1N1	07/09/2009	United Ki	A/Scotland/88/2009
HA	CY095721	H1N1	07/09/2009	China	A/Zhejiang/X1/2009
HA	CY095841	H1N1	07/09/2009	China	A/Zhejiang/7/2009
HA	CY095748	H1N1	07/09/2009	China	A/Zhejiang/X2/2009
HA	CY095777	H1N1	07/09/2009	China	A/Zhejiang/4/2009
HA	CY070311	H1N1	07/09/2009	United Ki	A/England/980/2009
HA	HQ228050	H1N1	07/09/2009	Finland	A/Finland/600/2009
HA	HQ228049	H1N1	07/09/2009	Finland	A/Finland/599/2009
HA	HQ228061	H1N1	07/09/2009	Finland	A/Finland/611/2009
HA	CY063510	H1N1	07/09/2009	USA	A/Boston/145/2009(H1N1)
HA	CY063526	H1N1	07/09/2009	USA	A/Boston/147/2009(H1N1)
HA	CY075435	H1N1	07/09/2009	Chile	A/Chile/4181/2009(H1N1)
HA	KP637718	H1N1	07/09/2009	Thailand	A/Thailand/SR08021/2009
HA	CY051615	H1N1	07/09/2009	USA	A/New York/4568/2009(H1N1)
HA	CY095763	H1N1	07/09/2009	China	A/Zhejiang/3/2009
HA	CY095728	H1N1	07/09/2009	China	A/Zhejiang/10/2009
HA	CY120165	H1N1	07/09/2009	Hong Kong	A/Hong Kong/H090-684-V10/2009
HA	CY123398	H1N1	07/09/2009	Singapore	A/Singapore/ON1074/2009
HA	CY122779	H1N1	07/09/2009	Singapore	A/Singapore/GP1182/2009
HA	CY111699	H1N1	07/09/2009	Hong Kong	A/Hong Kong/H090-684-V10/2009
HA	CY095817	H1N1	07/09/2009	China	A/Zhejiang/5/2009
HA	CY070230	H1N1	07/09/2009	United Ki	A/England/635/2009
HA	CY075427	H1N1	07/09/2009	Chile	A/Chile/4064/2009(H1N1)
HA	CY050265	H1N1	07/09/2009	China	A/Qingdao/320/2009(H1N1)
HA	CY054899	H1N1	07/09/2009	USA	A/California/VRDL39/2009(H1N1)
HA	CY049539	H1N1	07/09/2009	Singapore	A/Singapore/ON1118/2009(H1N1)
HA	CY049491	H1N1	07/09/2009	Singapore	A/Singapore/ON1072/2009(H1N1)
HA	CY070299	H1N1	07/09/2009	United Ki	A/Scotland/93040049/2009
HA	JN256808	H1N1	07/09/2009	Hong Kong	A/Hong Kong/H090-684-V1(0)/2009
HA	CY095906	H1N1	07/09/2009	China	A/Zhejiang/8/2009
HA	KC780623	H1N1	07/09/2009	USA	A/Utah/35/2009
HA	CY095979	H1N1	07/09/2009	China	A/Zhejiang/9/2009
HA	CY055204	H1N1	07/09/2009	Malaysia	A/Malaysia/4847/2009(H1N1)
HA	CY055202	H1N1	07/09/2009	Malaysia	A/Malaysia/4737/2009(H1N1)
HA	CY051583	H1N1	07/09/2009	USA	A/New York/4556/2009(H1N1)
HA	KP637798	H1N1	07/09/2009	Thailand	A/Thailand/SS08193/2009
HA	CY066183	H1N1	07/09/2009	USA	A/Boston/144/2009(H1N1)
HA	GQ392022	H1N1	07/09/2009	Russia	A/Moscow/IIV04/2009(H1N1)
HA	JN256810	H1N1	07/10/2009	Hong Kong	A/Hong Kong/H090-693-V1(0)/2009
HA	CY111667	H1N1	07/10/2009	Hong Kong	A/Hong Kong/H090-681-V22/2009
HA	KC780172	H1N1	07/10/2009	USA	A/Utah/46/2009

HA	CY070283	H1N1	07/10/2009	United Ki	A/England/93000013/2009
HA	CY070256	H1N1	07/10/2009	United Ki	A/England/92920036/2009
HA	CY054939	H1N1	07/10/2009	USA	A/California/VRDL48/2009(H1N1)
HA	CY049531	H1N1	07/10/2009	Singapore	A/Singapore/ON1113/2009(H1N1)
HA	CY070282	H1N1	07/10/2009	United Ki	A/England/92980708/2009
HA	CY074053	H1N1	07/10/2009	Argentina	A/Argentina/HNRG66/2009
HA	CY111715	H1N1	07/10/2009	Hong Kong	A/Hong Kong/H090-693-V10/2009
HA	JN256804	H1N1	07/10/2009	Hong Kong	A/Hong Kong/H090-681-V2(2)/2009
HA	CY065182	H1N1	07/10/2009	United Ki	A/England/675/2009
HA	CY050083	H1N1	07/10/2009	Taiwan	A/Taiwan/T0826/2009(H1N1)
HA	CY120181	H1N1	07/10/2009	Hong Kong	A/Hong Kong/H090-693-V10/2009
HA	CY070258	H1N1	07/10/2009	United Ki	A/England/92920061/2009
HA	CY070231	H1N1	07/10/2009	United Ki	A/England/630/2009
HA	CY075443	H1N1	07/10/2009	Chile	A/Chile/4182/2009(H1N1)
HA	GU014752	H1N1	07/11/2009	Japan	A/Mie/41/2009(H1N1)
HA	JN256806	H1N1	07/11/2009	Hong Kong	A/Hong Kong/H090-683-V1(0)/2009
HA	CY070312	H1N1	07/11/2009	United Ki	A/England/981/2009
HA	CY106568	H1N1	07/11/2009	Mexico	A/Merida/2189-CIR/2009
HA	CY120149	H1N1	07/11/2009	Hong Kong	A/Hong Kong/H090-683-V10/2009
HA	KT181027	H1N1	07/11/2009	Singapore	A/Singapore/DMS12/2009
HA	CY054915	H1N1	07/11/2009	USA	A/California/VRDL41/2009(H1N1)
HA	CY070272	H1N1	07/11/2009	United Ki	A/England/976/2009
HA	CY070284	H1N1	07/11/2009	United Ki	A/England/93000014/2009
HA	CY070269	H1N1	07/11/2009	United Ki	A/England/687/2009
HA	JX625443	H1N1	07/11/2009	United Ki	A/England/687/2009
HA	CY055206	H1N1	07/11/2009	Malaysia	A/Malaysia/5027/2009(H1N1)
HA	CY111683	H1N1	07/11/2009	Hong Kong	A/Hong Kong/H090-683-V10/2009
HA	CY055208	H1N1	07/11/2009	Malaysia	A/Malaysia/5057/2009(H1N1)
HA	CY070271	H1N1	07/11/2009	United Ki	A/England/975/2009
HA	CY055212	H1N1	07/11/2009	Malaysia	A/Malaysia/5112/2009(H1N1)
HA	CY054955	H1N1	07/11/2009	USA	A/California/VRDL51/2009(H1N1)
HA	CY070313	H1N1	07/11/2009	United Ki	A/England/93060032/2009
HA	KC782134	H1N1	07/11/2009	USA	A/Utah/34/2009
HA	CY070263	H1N1	07/12/2009	United Ki	A/England/637/2009
HA	CY070243	H1N1	07/12/2009	United Ki	A/England/92900359/2009
HA	GQ421203	H1N1	07/12/2009	Italy	A/Ancona/05/2009(H1N1)
HA	CY050267	H1N1	07/12/2009	China	A/Qingdao/333/2009(H1N1)
HA	CY055210	H1N1	07/12/2009	Malaysia	A/Malaysia/5103/2009(H1N1)
HA	CY070270	H1N1	07/12/2009	United Ki	A/England/92960009/2009
HA	CY070261	H1N1	07/12/2009	United Ki	A/England/92940308/2009
HA	CY070250	H1N1	07/12/2009	United Ki	A/England/92900386/2009
HA	JN412818	H1N1	07/12/2009	Indonesia	A/Jakarta/002/2009
HA	CY052423	H1N1	07/12/2009	USA	A/Texas/43122467/2009(H1N1)
HA	CY052346	H1N1	07/12/2009	Brazil	A/Parana/4597/2009(H1N1)
HA	CY049475	H1N1	07/12/2009	Singapore	A/Singapore/ON1060/2009(H1N1)
HA	CY049467	H1N1	07/12/2009	Singapore	A/Singapore/ON1050/2009(H1N1)
HA	CY049547	H1N1	07/12/2009	Singapore	A/Singapore/ON1126/2009(H1N1)
HA	CY072958	H1N1	07/12/2009	Nicaragua	A/Managua/585.01/2009(H1N1)
HA	HQ228051	H1N1	07/12/2009	Finland	A/Finland/601/2009
HA	CY072878	H1N1	07/12/2009	Nicaragua	A/Managua/4606.06/2009(H1N1)
HA	HQ228052	H1N1	07/13/2009	Finland	A/Finland/602/2009
HA	CY083320	H1N1	07/13/2009	USA	A/Great Lakes/WRAIR1652P/2009
HA	CY054603	H1N1	07/13/2009	Thailand	A/Thailand/THB0389/2009(H1N1)
HA	CY075761	H1N1	07/13/2009	Thailand	A/Thailand/CU-H203/2009(H1N1)

HA	CY055218	H1N1	07/13/2009	Malaysia	A/Malaysia/5175/2009(H1N1)
HA	CY049555	H1N1	07/13/2009	Singapore	A/Singapore/ON1138/2009(H1N1)
HA	CY049563	H1N1	07/13/2009	Singapore	A/Singapore/ON1144/2009(H1N1)
HA	CY049515	H1N1	07/13/2009	Singapore	A/Singapore/ON1099/2009(H1N1)
HA	CY111755	H1N1	07/13/2009	Hong Kong	A/Hong Kong/H090-704-V10/2009
HA	CY049507	H1N1	07/13/2009	Singapore	A/Singapore/ON1093/2009(H1N1)
HA	CY120068	H1N1	07/13/2009	Hong Kong	A/Hong Kong/H090-693-V20/2009
HA	CY120205	H1N1	07/13/2009	Hong Kong	A/Hong Kong/H090-704-V10/2009
HA	CY120141	H1N1	07/13/2009	Hong Kong	A/Hong Kong/H090-681-V32/2009
HA	CY123390	H1N1	07/13/2009	Singapore	A/Singapore/ON1069/2009
HA	CY123367	H1N1	07/13/2009	Singapore	A/Singapore/ON1048/2009
HA	JN256805	H1N1	07/13/2009	Hong Kong	A/Hong Kong/H090-681-V3(2)/2009
HA	JN256815	H1N1	07/13/2009	Hong Kong	A/Hong Kong/H090-704-V1(0)/2009
HA	JN256811	H1N1	07/13/2009	Hong Kong	A/Hong Kong/H090-693-V2(0)/2009
HA	CY111675	H1N1	07/13/2009	Hong Kong	A/Hong Kong/H090-681-V32/2009
HA	CY111731	H1N1	07/13/2009	Hong Kong	A/Hong Kong/H090-698-V10/2009
HA	CY070338	H1N1	07/13/2009	United Ki	A/England/654/2009
HA	CY070257	H1N1	07/13/2009	United Ki	A/England/92920053/2009
HA	CY070278	H1N1	07/13/2009	United Ki	A/England/688/2009
HA	CY070276	H1N1	07/13/2009	United Ki	A/England/92960080/2009
HA	CY075451	H1N1	07/13/2009	Chile	A/Chile/4257/2009(H1N1)
HA	KC780130	H1N1	07/13/2009	USA	A/Utah/36/2009
HA	CY052407	H1N1	07/13/2009	USA	A/Texas/43132503/2009(H1N1)
HA	CY120076	H1N1	07/13/2009	Hong Kong	A/Hong Kong/H090-698-V10/2009
HA	CY049499	H1N1	07/13/2009	Singapore	A/Singapore/ON1087/2009(H1N1)
HA	CY070264	H1N1	07/13/2009	United Ki	A/England/625/2009
HA	CY070260	H1N1	07/13/2009	United Ki	A/England/92940035/2009
HA	KP637654	H1N1	07/13/2009	Thailand	A/Thailand/SP08279/2009
HA	CY070259	H1N1	07/13/2009	United Ki	A/England/624/2009
HA	CY070255	H1N1	07/13/2009	United Ki	A/England/620/2009
HA	JN256814	H1N1	07/13/2009	Hong Kong	A/Hong Kong/H090-701-V1(0)/2009
HA	JX625459	H1N1	07/13/2009	United Ki	A/England/688/2009
HA	AB530249	H1N1	07/13/2009	Japan	A/Nagasaki/HA-6/2009(H1N1)
HA	JN256812	H1N1	07/13/2009	Hong Kong	A/Hong Kong/H090-698-V1(0)/2009
HA	KC781489	H1N1	07/13/2009	USA	A/Minnesota/12/2009
HA	CY054604	H1N1	07/13/2009	Thailand	A/Thailand/THB0390/2009(H1N1)
HA	CY123382	H1N1	07/13/2009	Singapore	A/Singapore/ON1063/2009
HA	CY123374	H1N1	07/13/2009	Singapore	A/Singapore/ON1058/2009
HA	CY083104	H1N1	07/13/2009	Peru	A/Piura/WRAIR0603F/2009(H1N1)
HA	CY055214	H1N1	07/13/2009	Malaysia	A/Malaysia/5162/2009(H1N1)
HA	GU014770	H1N1	07/13/2009	Japan	A/Wakayama/57/2009(H1N1)
HA	CY055216	H1N1	07/13/2009	Malaysia	A/Malaysia/5163/2009(H1N1)
HA	CY049523	H1N1	07/13/2009	Singapore	A/Singapore/ON1105/2009(H1N1)
HA	CY120197	H1N1	07/13/2009	Hong Kong	A/Hong Kong/H090-701-V10/2009
HA	CY111747	H1N1	07/13/2009	Hong Kong	A/Hong Kong/H090-701-V10/2009
HA	CY111723	H1N1	07/13/2009	Hong Kong	A/Hong Kong/H090-693-V20/2009
HA	CY083511	H1N1	07/13/2009	Peru	A/Piura/WRAIR1694P/2009(H1N1)
HA	HM189400	H1N1	07/13/2009	Russia	A/Moscow/CRIE-S/2009
HA	CY075756	H1N1	07/13/2009	Thailand	A/Thailand/CU-H199/2009(H1N1)
HA	CY070339	H1N1	07/13/2009	United Ki	A/England/983/2009
HA	CY070280	H1N1	07/13/2009	United Ki	A/England/92980009/2009
HA	CY070266	H1N1	07/13/2009	United Ki	A/England/627/2009
HA	HQ228054	H1N1	07/14/2009	Finland	A/Finland/604/2009
HA	CY070273	H1N1	07/14/2009	United Ki	A/England/629/2009

HA	CY070274	H1N1	07/14/2009	United Ki	A/England/652/2009
HA	CY073697	H1N1	07/14/2009	Nicaragua	A/Managua/3490.03/2009(H1N1)
HA	CY054979	H1N1	07/14/2009	USA	A/California/VRDL55/2009(H1N1)
HA	CY049571	H1N1	07/14/2009	Singapore	A/Singapore/ON1156/2009(H1N1)
HA	GQ499336	H1N1	07/14/2009	USA	A/Washington/28/2009(H1N1)
HA	CY051631	H1N1	07/14/2009	USA	A/New York/4620/2009(H1N1)
HA	CY051623	H1N1	07/14/2009	USA	A/New York/4607/2009(H1N1)
HA	CY052250	H1N1	07/14/2009	USA	A/Texas/43143450/2009(H1N1)
HA	CY120213	H1N1	07/14/2009	Hong Kong	A/Hong Kong/H090-708-V10/2009
HA	CY122787	H1N1	07/14/2009	Singapore	A/Singapore/GP1358/2009
HA	CY123406	H1N1	07/14/2009	Singapore	A/Singapore/ON1079/2009
HA	CY123454	H1N1	07/14/2009	Singapore	A/Singapore/ON1121/2009
HA	CY123438	H1N1	07/14/2009	Singapore	A/Singapore/ON1107/2009
HA	CY123414	H1N1	07/14/2009	Singapore	A/Singapore/ON1091/2009
HA	CY123422	H1N1	07/14/2009	Singapore	A/Singapore/ON1095/2009
HA	JN256809	H1N1	07/14/2009	Hong Kong	A/Hong Kong/H090-684-V2(3)/2009
HA	CY111691	H1N1	07/14/2009	Hong Kong	A/Hong Kong/H090-683-V20/2009
HA	CY111707	H1N1	07/14/2009	Hong Kong	A/Hong Kong/H090-684-V23/2009
HA	CY120173	H1N1	07/14/2009	Hong Kong	A/Hong Kong/H090-684-V23/2009
HA	CY070275	H1N1	07/14/2009	United Ki	A/England/653/2009
HA	CY070267	H1N1	07/14/2009	United Ki	A/England/628/2009
HA	CY070262	H1N1	07/14/2009	United Ki	A/England/92940310/2009
HA	HQ228053	H1N1	07/14/2009	Finland	A/Finland/603/2009
HA	KC782050	H1N1	07/14/2009	USA	A/Maryland/33/2009
HA	CY054963	H1N1	07/14/2009	USA	A/California/VRDL52/2009(H1N1)
HA	CY049595	H1N1	07/14/2009	Singapore	A/Singapore/ON1177/2009(H1N1)
HA	JN256816	H1N1	07/14/2009	Hong Kong	A/Hong Kong/H090-708-V1(0)/2009
HA	CY070279	H1N1	07/14/2009	United Ki	A/England/92980008/2009
HA	CY123430	H1N1	07/14/2009	Singapore	A/Singapore/ON1102/2009
HA	CY123446	H1N1	07/14/2009	Singapore	A/Singapore/ON1114/2009
HA	JN256807	H1N1	07/14/2009	Hong Kong	A/Hong Kong/H090-683-V2(0)/2009
HA	CY120157	H1N1	07/14/2009	Hong Kong	A/Hong Kong/H090-683-V20/2009
HA	GU014758	H1N1	07/14/2009	Myanmar	A/Myanmar/JP101/2009(H1N1)
HA	CY049579	H1N1	07/14/2009	Singapore	A/Singapore/ON1163/2009(H1N1)
HA	CY049587	H1N1	07/14/2009	Singapore	A/Singapore/ON1168/2009(H1N1)
HA	CY111763	H1N1	07/14/2009	Hong Kong	A/Hong Kong/H090-708-V10/2009
HA	KT181071	H1N1	07/14/2009	Singapore	A/Singapore/DMS13/2009
HA	CY070265	H1N1	07/14/2009	United Ki	A/England/626/2009
HA	HQ228055	H1N1	07/15/2009	Finland	A/Finland/605/2009
HA	CY111779	H1N1	07/15/2009	Hong Kong	A/Hong Kong/H090-712-V1_4/2009
HA	CY063566	H1N1	07/15/2009	USA	A/Boston/153/2009(H1N1)
HA	CY054605	H1N1	07/15/2009	Thailand	A/Thailand/THB0402/2009(H1N1)
HA	CY054607	H1N1	07/15/2009	Thailand	A/Thailand/THB0408/2009(H1N1)
HA	GU576500	H1N1	07/15/2009	Italy	A/Ancona/10/2009(H1N1)
HA	CY055222	H1N1	07/15/2009	Malaysia	A/Malaysia/5193/2009(H1N1)
HA	CY055220	H1N1	07/15/2009	Malaysia	A/Malaysia/5192/2009(H1N1)
HA	CY049603	H1N1	07/15/2009	Singapore	A/Singapore/ON1187/2009(H1N1)
HA	HM569699	H1N1	07/15/2009	Argentina	A/Argentina/07AR/2009(H1N1)
HA	JN256817	H1N1	07/15/2009	Hong Kong	A/Hong Kong/H090-712-V1(0)/2009
HA	JN256818	H1N1	07/15/2009	Hong Kong	A/Hong Kong/H090-712-V1(4)/2009
HA	CY111771	H1N1	07/15/2009	Hong Kong	A/Hong Kong/H090-712-V10/2009
HA	CY075921	H1N1	07/15/2009	India	A/Che/NIV551/2009
HA	CY070268	H1N1	07/15/2009	United Ki	A/England/92960005/2009
HA	CY070337	H1N1	07/15/2009	United Ki	A/England/621/2009

HA	CY054606	H1N1	07/15/2009	Thailand	A/Thailand/THB0405/2009(H1N1)
HA	CY120229	H1N1	07/15/2009	Hong Kong	A/Hong Kong/H090-712-V14/2009
HA	HQ228056	H1N1	07/15/2009	Finland	A/Finland/606/2009
HA	KC967086	H1N1	07/15/2009	Brazil	A/Rio de Janeiro/3701/2009
HA	HM189415	H1N1	07/15/2009	Russia	A/Moscow oblast/CRIE-4145/2009
HA	HM189369	H1N1	07/15/2009	Russia	A/Moscow/CRIE-Th/2009
HA	CY120221	H1N1	07/15/2009	Hong Kong	A/Hong Kong/H090-712-V10/2009
HA	JX625435	H1N1	07/15/2009	United Ki	A/England/92960004/2009
HA	HM189416	H1N1	07/15/2009	Russia	A/Moscow oblast/CRIE-4142/2009
HA	GU290055	H1N1	07/15/2009	Czech Rep	A/Prague/196-81511/2009(H1N1)
HA	CY083048	H1N1	07/15/2009	Peru	A/Lima/WRAIR0285F/2009
HA	CY050268	H1N1	07/16/2009	China	A/Qingdao/359/2009(H1N1)
HA	HM189367	H1N1	07/16/2009	Russia	A/Moscow/CRIE-ION/2009
HA	CY055526	H1N1	07/16/2009	Australia	A/Australia/1/2009(H1N1)
HA	GU576501	H1N1	07/16/2009	Italy	A/Ancona/11/2009(H1N1)
HA	CY055224	H1N1	07/16/2009	Malaysia	A/Malaysia/5218/2009(H1N1)
HA	CY054609	H1N1	07/16/2009	Thailand	A/Thailand/THB0415/2009(H1N1)
HA	CY111739	H1N1	07/16/2009	Hong Kong	A/Hong Kong/H090-698-V20/2009
HA	CY120189	H1N1	07/16/2009	Hong Kong	A/Hong Kong/H090-698-V20/2009
HA	CY070287	H1N1	07/16/2009	United Ki	A/England/93000118/2009
HA	CY070321	H1N1	07/16/2009	United Ki	A/England/93100717/2009
HA	CY054608	H1N1	07/16/2009	Thailand	A/Thailand/THB0411/2009(H1N1)
HA	JN256821	H1N1	07/16/2009	Hong Kong	A/Hong Kong/H090-715-V1(0)/2009
HA	KC780279	H1N1	07/16/2009	USA	A/Utah/41/2009
HA	CY120952	H1N1	07/16/2009	China	A/Guangdong/801/2009
HA	JN256813	H1N1	07/16/2009	Hong Kong	A/Hong Kong/H090-698-V2(0)/2009
HA	HM189426	H1N1	07/16/2009	Russia	A/Moscow/CRIE-PTD/2009
HA	CY083359	H1N1	07/16/2009	USA	A/San Diego/WRAIR1658P/2009
HA	CY051639	H1N1	07/16/2009	USA	A/New York/4677/2009(H1N1)
HA	HQ228076	H1N1	07/16/2009	Finland	A/Finland/627/2009
HA	CY072838	H1N1	07/16/2009	Nicaragua	A/Managua/265.01/2009(H1N1)
HA	CY111803	H1N1	07/16/2009	Hong Kong	A/Hong Kong/H090-715-V10/2009
HA	CY072894	H1N1	07/16/2009	Nicaragua	A/Managua/3214.04/2009(H1N1)
HA	CY054591	H1N1	07/17/2009	Thailand	A/Thailand/0404-00-N0/2009(H1N1)
HA	CY075459	H1N1	07/17/2009	Chile	A/Chile/4406/2009(H1N1)
HA	CY055059	H1N1	07/17/2009	Australia	A/Australia/3/2009(H1N1)
HA	GU576506	H1N1	07/17/2009	Italy	A/Ancona/17/2009(H1N1)
HA	CY055226	H1N1	07/17/2009	Malaysia	A/Malaysia/5225/2009(H1N1)
HA	CY083280	H1N1	07/17/2009	USA	A/South Carolina/WRAIR1645P/2009
HA	CY046064	H1N1	07/17/2009	Italy	A/Italy/131/2009(H1N1)
HA	CY046065	H1N1	07/17/2009	Italy	A/Italy/132/2009(H1N1)
HA	CY120253	H1N1	07/17/2009	Hong Kong	A/Hong Kong/H090-721-V10/2009
HA	JX625467	H1N1	07/17/2009	United Ki	A/England/92980479/2009
HA	CY070281	H1N1	07/17/2009	United Ki	A/England/92980479/2009
HA	JN256822	H1N1	07/17/2009	Hong Kong	A/Hong Kong/H090-721-V1(0)/2009
HA	CY103911	H1N1	07/17/2009	Brazil	A/Pernambuco/107/2009
HA	CY070285	H1N1	07/17/2009	United Ki	A/England/93000095/2009
HA	CY050269	H1N1	07/17/2009	China	A/Qingdao/362/2009(H1N1)
HA	HQ228077	H1N1	07/17/2009	Finland	A/Finland/628/2009
HA	KT181080	H1N1	07/17/2009	Singapore	A/Singapore/DMS14/2009
HA	CY055067	H1N1	07/17/2009	Australia	A/Australia/4/2009(H1N1)
HA	CY070325	H1N1	07/17/2009	United Ki	A/England/93120033/2009
HA	GU576502	H1N1	07/17/2009	Italy	A/Ancona/15/2009(H1N1)
HA	GU576504	H1N1	07/17/2009	Italy	A/Ancona/16/2009(H1N1)

HA	KC781466	H1N1	07/17/2009	USA	A/Idaho/08/2009
HA	CY120261	H1N1	07/17/2009	Hong Kong	A/Hong Kong/H090-722-V10/2009
HA	CY083136	H1N1	07/17/2009	Peru	A/Lima/WRAIR0681F/2009
HA	HQ228075	H1N1	07/17/2009	Finland	A/Finland/626/2009
HA	CY070315	H1N1	07/17/2009	United Ki	A/England/690/2009
HA	CY070288	H1N1	07/17/2009	United Ki	A/England/638/2009
HA	JN256823	H1N1	07/17/2009	Hong Kong	A/Hong Kong/H090-722-V1(0)/2009
HA	CY097884	H1N1	07/17/2009	USA	A/South Carolina/WRAIR1645P/2009
HA	HM189427	H1N1	07/17/2009	Russia	A/Moscow/CRIE-TRV/2009
HA	CY111811	H1N1	07/17/2009	Hong Kong	A/Hong Kong/H090-721-V10/2009
HA	CY111819	H1N1	07/17/2009	Hong Kong	A/Hong Kong/H090-722-V10/2009(H1N1)
HA	CY070289	H1N1	07/17/2009	United Ki	A/England/93000137/2009
HA	CY075773	H1N1	07/17/2009	Thailand	A/Thailand/CU-H243/2009(H1N1)
HA	CY083351	H1N1	07/18/2009	USA	A/San Diego/WRAIR1657P/2009
HA	KC780327	H1N1	07/18/2009	USA	A/Utah/37/2009
HA	CY120237	H1N1	07/18/2009	Hong Kong	A/Hong Kong/H090-712-V20/2009
HA	JN256820	H1N1	07/18/2009	Hong Kong	A/Hong Kong/H090-712-V2(4)/2009
HA	CY111787	H1N1	07/18/2009	Hong Kong	A/Hong Kong/H090-712-V20/2009
HA	CY111795	H1N1	07/18/2009	Hong Kong	A/Hong Kong/H090-712-V24/2009
HA	CY055075	H1N1	07/18/2009	Australia	A/Australia/6/2009(H1N1)
HA	HM189429	H1N1	07/18/2009	Russia	A/Moscow oblast/CRIE-RMA/2009
HA	CY070297	H1N1	07/18/2009	United Ki	A/England/639/2009
HA	HM189420	H1N1	07/18/2009	Russia	A/St.Petersburg/CRIE-PHP/2009
HA	JN256819	H1N1	07/18/2009	Hong Kong	A/Hong Kong/H090-712-V2(0)/2009
HA	CY120245	H1N1	07/18/2009	Hong Kong	A/Hong Kong/H090-712-V24/2009
HA	GU014806	H1N1	07/18/2009	Japan	A/Tokushima/2/2009(H1N1)
HA	CY052048	H1N1	07/18/2009	Brazil	A/Rio Grande do Sul/4509/2009(H1N1)
HA	CY055534	H1N1	07/18/2009	Australia	A/Australia/7/2009(H1N1)
HA	GQ915017	H1N1	07/19/2009	Brazil	A/Sao Paulo/53206/2009
HA	CY083128	H1N1	07/19/2009	Peru	A/Lima/WRAIR0672F/2009
HA	KC781037	H1N1	07/19/2009	USA	A/Washington/74/2009
HA	GU433025	H1N1	07/19/2009	Russia	A/Tomsk/03/2009(H1N1)
HA	KC780160	H1N1	07/19/2009	USA	A/Utah/47/2009
HA	CY050270	H1N1	07/19/2009	China	A/Qingdao/375/2009(H1N1)
HA	CY054610	H1N1	07/20/2009	Thailand	A/Thailand/THB0419/2009(H1N1)
HA	CY055565	H1N1	07/20/2009	Australia	A/Australia/12/2009(H1N1)
HA	CY055230	H1N1	07/20/2009	Malaysia	A/Malaysia/5237/2009(H1N1)
HA	CY075467	H1N1	07/20/2009	Chile	A/Chile/4438/2009(H1N1)
HA	CY050271	H1N1	07/20/2009	China	A/Qingdao/383/2009(H1N1)
HA	CY052375	H1N1	07/20/2009	USA	A/Texas/43200999/2009(H1N1)
HA	HQ228074	H1N1	07/20/2009	Finland	A/Finland/625/2009
HA	KP637398	H1N1	07/20/2009	Thailand	A/Thailand/MR08208/2009
HA	CY070999	H1N1	07/20/2009	Guam	A/Guam/NHRC0030/2009(H1N1)
HA	CY070305	H1N1	07/20/2009	United Ki	A/England/93040124/2009
HA	CY070308	H1N1	07/20/2009	United Ki	A/England/978/2009
HA	CY070291	H1N1	07/20/2009	United Ki	A/England/93020046/2009
HA	CY070306	H1N1	07/20/2009	United Ki	A/England/93040132/2009
HA	HQ228079	H1N1	07/20/2009	Finland	A/Finland/630/2009
HA	HQ228078	H1N1	07/20/2009	Finland	A/Finland/629/2009
HA	CY053904	H1N1	07/20/2009	Argentina	A/Argentina/HNRG15/2009(H1N1)
HA	CY070304	H1N1	07/20/2009	United Ki	A/England/93040114/2009
HA	CY070290	H1N1	07/20/2009	United Ki	A/England/93020044/2009
HA	CY054612	H1N1	07/20/2009	Thailand	A/Thailand/THB0421/2009(H1N1)
HA	CY054611	H1N1	07/20/2009	Thailand	A/Thailand/THB0420/2009(H1N1)

HA	CY055581	H1N1	07/20/2009	Australia	A/Australia/14/2009(H1N1)
HA	CY055550	H1N1	07/20/2009	Australia	A/Australia/10/2009(H1N1)
HA	CY055558	H1N1	07/20/2009	Australia	A/Australia/11/2009(H1N1)
HA	CY055589	H1N1	07/20/2009	Australia	A/Australia/15/2009(H1N1)
HA	CY055228	H1N1	07/20/2009	Malaysia	A/Malaysia/5234/2009(H1N1)
HA	CY055542	H1N1	07/20/2009	Australia	A/Australia/9/2009(H1N1)
HA	CY052049	H1N1	07/20/2009	Brazil	A/Rio Grande do Sul/4772/2009(H1N1)
HA	CY052050	H1N1	07/20/2009	Brazil	A/Rio Grande do Sul/4782/2009(H1N1)
HA	CY055573	H1N1	07/20/2009	Australia	A/Australia/13/2009(H1N1)
HA	CY055605	H1N1	07/21/2009	Australia	A/Australia/20/2009(H1N1)
HA	CY069090	H1N1	07/21/2009	Guam	A/Guam/NHRC0026/2009(H1N1)
HA	CY055287	H1N1	07/21/2009	Malaysia	A/Malaysia/5259/2009(H1N1)
HA	CY111827	H1N1	07/21/2009	Hong Kong	A/Hong Kong/H090-722-V20/2009(H1N1)
HA	CY055629	H1N1	07/21/2009	Australia	A/Australia/24/2009(H1N1)
HA	CY070322	H1N1	07/21/2009	United Ki	A/England/642/2009
HA	CY070309	H1N1	07/21/2009	United Ki	A/England/93040152/2009
HA	CY070323	H1N1	07/21/2009	United Ki	A/England/676/2009
HA	HQ228062	H1N1	07/21/2009	Finland	A/Finland/612/2009
HA	CY092185	H1N1	07/21/2009	Australia	A/Australia/17/2009
HA	CY083208	H1N1	07/21/2009	USA	A/New Jersey/WRAIR1504P/2009(H1N1)
HA	CY069258	H1N1	07/21/2009	Nicaragua	A/Managua/5364.01/2009(H1N1)
HA	CY068970	H1N1	07/21/2009	Guam	A/Guam/NHRC0011/2009(H1N1)
HA	CY073434	H1N1	07/21/2009	USA	A/San Diego/WR1650P/2009(H1N1)
HA	CY069050	H1N1	07/21/2009	Guam	A/Guam/NHRC0021/2009(H1N1)
HA	CY072854	H1N1	07/21/2009	Nicaragua	A/Managua/3438.02/2009(H1N1)
HA	CY055597	H1N1	07/21/2009	Australia	A/Australia/16/2009(H1N1)
HA	GU014754	H1N1	07/21/2009	Japan	A/Mie/52/2009(H1N1)
HA	KC782042	H1N1	07/21/2009	USA	A/West Virginia/06/2009
HA	CY046066	H1N1	07/21/2009	Italy	A/Italy/133/2009(H1N1)
HA	HM569667	H1N1	07/21/2009	Argentina	A/Argentina/08AR/2009(H1N1)
HA	CY070307	H1N1	07/21/2009	United Ki	A/England/93040139/2009
HA	CY070318	H1N1	07/21/2009	United Ki	A/England/93100019/2009
HA	CY070303	H1N1	07/21/2009	United Ki	A/England/93040063/2009
HA	CY054613	H1N1	07/21/2009	Thailand	A/Thailand/THB0423/2009(H1N1)
HA	KC782036	H1N1	07/21/2009	USA	A/Washington/47/2009
HA	CY054987	H1N1	07/21/2009	USA	A/California/VRDL58/2009(H1N1)
HA	CY075922	H1N1	07/21/2009	India	A/Pune/NIV652/2009
HA	CY054971	H1N1	07/21/2009	USA	A/California/VRDL53/2009(H1N1)
HA	KC781564	H1N1	07/21/2009	USA	A/Virginia/17/2009
HA	JN256824	H1N1	07/21/2009	Hong Kong	A/Hong Kong/H090-722-V2(0)/2009
HA	CY070316	H1N1	07/21/2009	United Ki	A/England/93080005/2009
HA	HQ228080	H1N1	07/21/2009	Finland	A/Finland/631/2009
HA	KC620386	H1N1	07/21/2009	Germany	A/Giessen/6/2009
HA	CY068962	H1N1	07/21/2009	Guam	A/Guam/NHRC0010/2009(H1N1)
HA	CY055232	H1N1	07/21/2009	Malaysia	A/Malaysia/5258/2009(H1N1)
HA	CY055295	H1N1	07/21/2009	Malaysia	A/Malaysia/5259/2009(H1N1)
HA	CY055613	H1N1	07/21/2009	Australia	A/Australia/21/2009(H1N1)
HA	CY069034	H1N1	07/21/2009	Guam	A/Guam/NHRC0019/2009(H1N1)
HA	GU433033	H1N1	07/21/2009	Russia	A/Novosibirsk/02/2009(H1N1)
HA	CY055234	H1N1	07/22/2009	Malaysia	A/Malaysia/5262/2009(H1N1)
HA	CY069026	H1N1	07/22/2009	Guam	A/Guam/NHRC0018/2009(H1N1)
HA	GU014760	H1N1	07/22/2009	Myanmar	A/Myanmar/JP131/2009(H1N1)
HA	CY070293	H1N1	07/22/2009	United Ki	A/England/93040011/2009
HA	CY070296	H1N1	07/22/2009	United Ki	A/England/93040019/2009

HA	HQ228063	H1N1	07/22/2009	Finland	A/Finland/613/2009
HA	CY070317	H1N1	07/22/2009	United Ki	A/England/613/2009
HA	CY061537	H1N1	07/22/2009	Argentina	A/Argentina/HNRG82/2009
HA	CY083367	H1N1	07/22/2009	USA	A/South Carolina/WRAIR1660P/2009
HA	CY054614	H1N1	07/22/2009	Thailand	A/Thailand/THB0427/2009(H1N1)
HA	CY054602	H1N1	07/22/2009	Thailand	A/Thailand/THA0364/2009(H1N1)
HA	CY073617	H1N1	07/22/2009	Nicaragua	A/Managua/4747.01/2009(H1N1)
HA	CY055637	H1N1	07/22/2009	Australia	A/Australia/25/2009(H1N1)
HA	CY055653	H1N1	07/22/2009	Australia	A/Australia/27/2009(H1N1)
HA	KX013005	H1N1	07/22/2009	Germany	A/Jena/MSV-VI5258/2009
HA	KJ549778	H1N1	07/22/2009	Germany	A/Jena/5258/2009
HA	CY070295	H1N1	07/22/2009	United Ki	A/England/93040018/2009
HA	CY073649	H1N1	07/22/2009	Nicaragua	A/Managua/4590.03/2009(H1N1)
HA	CY055236	H1N1	07/22/2009	Malaysia	A/Malaysia/5270/2009(H1N1)
HA	CY055238	H1N1	07/22/2009	Malaysia	A/Malaysia/5272/2009(H1N1)
HA	CY055240	H1N1	07/22/2009	Malaysia	A/Malaysia/5277/2009(H1N1)
HA	CY055621	H1N1	07/22/2009	Australia	A/Australia/23/2009(H1N1)
HA	CY070292	H1N1	07/22/2009	United Ki	A/England/93040009/2009
HA	KF667881	H1N1	07/22/2009	Singapore	A/Singapore/384P/2009
HA	KJ690424	H1N1	07/22/2009	Uganda	A/Uganda/MUWRP-059/2009
HA	GQ496142	H1N1	07/22/2009	Russia	A/Vladivostok/IIV17/2009(H1N1)
HA	KC222636	H1N1	07/22/2009	Germany	A/Jena/VI5258/2009(H1N1)
HA	GU014762	H1N1	07/22/2009	Myanmar	A/Myanmar/JP133/2009(H1N1)
HA	CY055645	H1N1	07/22/2009	Australia	A/Australia/26/2009(H1N1)
HA	CY068938	H1N1	07/22/2009	Guam	A/Guam/NHRC0008/2009(H1N1)
HA	CY073641	H1N1	07/22/2009	Nicaragua	A/Managua/1973.02/2009(H1N1)
HA	CY054615	H1N1	07/22/2009	Thailand	A/Thailand/THB0428/2009(H1N1)
HA	CY054279	H1N1	07/23/2009	Brazil	A/Rio de Janeiro/5090/2009(H1N1)
HA	CY052347	H1N1	07/23/2009	Brazil	A/Rio Grande do Sul/5395/2009(H1N1)
HA	CY055684	H1N1	07/23/2009	Australia	A/Australia/32/2009(H1N1)
HA	CY065952	H1N1	07/23/2009	Japan	A/Japan/NHRC0001/2009(H1N1)
HA	AB530462	H1N1	07/23/2009	Japan	A/Nagasaki/HA-10/2009(H1N1)
HA	AB530463	H1N1	07/23/2009	Japan	A/Nagasaki/HA-11/2009(H1N1)
HA	CY070320	H1N1	07/23/2009	United Ki	A/England/614/2009
HA	CY050272	H1N1	07/23/2009	China	A/Qingdao/399/2009(H1N1)
HA	AB530461	H1N1	07/23/2009	Japan	A/Nagasaki/HA-9/2009(H1N1)
HA	JX625498	H1N1	07/23/2009	United Ki	A/England/93760756/2009
HA	KC781873	H1N1	07/23/2009	USA	A/South Carolina/29/2009
HA	JQ065120	H1N1	07/23/2009	China	A/Shenzhen/9/2009
HA	JQ065115	H1N1	07/23/2009	China	A/Shenzhen/4/2009
HA	JQ065114	H1N1	07/23/2009	China	A/Shenzhen/3/2009
HA	JQ065113	H1N1	07/23/2009	China	A/Shenzhen/2/2009
HA	JQ065117	H1N1	07/23/2009	China	A/Shenzhen/6/2009
HA	JQ065121	H1N1	07/23/2009	China	A/Shenzhen/10/2009
HA	JQ065118	H1N1	07/23/2009	China	A/Shenzhen/7/2009
HA	CY119354	H1N1	07/23/2009	Malaysia	A/Malaysia/2050837/2009
HA	JQ065116	H1N1	07/23/2009	China	A/Shenzhen/5/2009
HA	CY055242	H1N1	07/23/2009	Malaysia	A/Malaysia/5283/2009(H1N1)
HA	CY065960	H1N1	07/23/2009	Japan	A/Japan/NHRC0002/2009(H1N1)
HA	CY070319	H1N1	07/23/2009	United Ki	A/England/672/2009
HA	KF700936	H1N1	07/23/2009	Brazil	A/Rio Grande do Sul/5470/2009
HA	KC781866	H1N1	07/23/2009	USA	A/Kansas/14/2009
HA	CY103912	H1N1	07/23/2009	Brazil	A/Pernambuco/117/2009
HA	KT181089	H1N1	07/23/2009	Singapore	A/Singapore/DMS16/2009

HA	KC967087	H1N1	07/23/2009	Brazil	A/Rio de Janeiro/5007/2009
HA	JQ065112	H1N1	07/23/2009	China	A/Shenzhen/1/2009
HA	CY055244	H1N1	07/23/2009	Malaysia	A/Malaysia/5286/2009(H1N1)
HA	CY055668	H1N1	07/23/2009	Australia	A/Australia/29/2009(H1N1)
HA	CY055661	H1N1	07/23/2009	Australia	A/Australia/28/2009(H1N1)
HA	JQ065119	H1N1	07/23/2009	China	A/Shenzhen/8/2009
HA	JQ065122	H1N1	07/23/2009	China	A/Shenzhen/11/2009
HA	CY065936	H1N1	07/23/2009	Guam	A/Guam/NHRC0002/2009(H1N1)
HA	CY068978	H1N1	07/23/2009	Guam	A/Guam/NHRC0012/2009(H1N1)
HA	CY073593	H1N1	07/23/2009	Nicaragua	A/Managua/5295.02/2009(H1N1)
HA	CY068914	H1N1	07/23/2009	Japan	A/Japan/NHRC0003/2009(H1N1)
HA	CY055676	H1N1	07/23/2009	Australia	A/Australia/31/2009(H1N1)
HA	CY055692	H1N1	07/24/2009	Australia	A/Australia/35/2009(H1N1)
HA	CY055708	H1N1	07/24/2009	Australia	A/Australia/37/2009(H1N1)
HA	CY055716	H1N1	07/24/2009	Australia	A/Australia/39/2009(H1N1)
HA	CY111843	H1N1	07/24/2009	Hong Kong	A/Hong Kong/H090-742-V10/2009(H1N1)
HA	GQ496149	H1N1	07/24/2009	Russia	A/Vladivostok/IIV18/2009(H1N1)
HA	GU290188	H1N1	07/24/2009	USA	A/Utah/42/2009(H1N1)
HA	AB530467	H1N1	07/24/2009	Japan	A/Nagasaki/HA-15/2009(H1N1)
HA	CY111835	H1N1	07/24/2009	Hong Kong	A/Hong Kong/H090-738-V10/2009
HA	CY070324	H1N1	07/24/2009	United Ki	A/England/93120020/2009
HA	CY062529	H1N1	07/24/2009	Mexico	A/Mexico city/CIA7/2009(H1N1)
HA	CY092193	H1N1	07/24/2009	Australia	A/Australia/38/2009
HA	CY069282	H1N1	07/24/2009	Nicaragua	A/Managua/3467.01/2009(H1N1)
HA	CY072574	H1N1	07/24/2009	Nicaragua	A/Managua/3231.02/2009(H1N1)
HA	CY055724	H1N1	07/24/2009	Australia	A/Australia/40/2009(H1N1)
HA	KP637454	H1N1	07/24/2009	Thailand	A/Thailand/PY08016/2009
HA	KP637662	H1N1	07/24/2009	Thailand	A/Thailand/SP08294/2009
HA	AB530465	H1N1	07/24/2009	Japan	A/Nagasaki/HA-13/2009(H1N1)
HA	GU292341	H1N1	07/24/2009	Finland	A/Finland/614/2009
HA	CY120269	H1N1	07/24/2009	Hong Kong	A/Hong Kong/H090-738-V10/2009
HA	CY128083	H1N1	07/24/2009	Viet Nam	A/Viet Nam/811/2009
HA	CY128075	H1N1	07/24/2009	Viet Nam	A/Viet Nam/817/2009
HA	JN256825	H1N1	07/24/2009	Hong Kong	A/Hong Kong/H090-738-V1(0)/2009
HA	CY051823	H1N1	07/24/2009	USA	A/Texas/43242018/2009(H1N1)
HA	KC782281	H1N1	07/24/2009	USA	A/Utah/42/2009
HA	KC782285	H1N1	07/24/2009	USA	A/Utah/42/2009
HA	CY055700	H1N1	07/24/2009	Australia	A/Australia/36/2009(H1N1)
HA	AB530464	H1N1	07/24/2009	Japan	A/Nagasaki/HA-12/2009(H1N1)
HA	KC780098	H1N1	07/24/2009	USA	A/Utah/42/2009
HA	KF700931	H1N1	07/24/2009	Brazil	A/Rio Grande do Sul/5661/2009
HA	GU290190	H1N1	07/24/2009	USA	A/Utah/42/2009(H1N1)
HA	AB530466	H1N1	07/24/2009	Japan	A/Nagasaki/HA-14/2009(H1N1)
HA	JN256826	H1N1	07/24/2009	Hong Kong	A/Hong Kong/H090-742-V1(0)/2009
HA	CY120277	H1N1	07/24/2009	Hong Kong	A/Hong Kong/H090-742-V10/2009
HA	CY051655	H1N1	07/24/2009	USA	A/New York/4728/2009(H1N1)
HA	CY069290	H1N1	07/24/2009	Nicaragua	A/Managua/4951.02/2009(H1N1)
HA	AB530468	H1N1	07/25/2009	Japan	A/Nagasaki/HA-16/2009(H1N1)
HA	CY069266	H1N1	07/25/2009	Nicaragua	A/Managua/1264.03/2009(H1N1)
HA	HM189377	H1N1	07/25/2009	Russia	A/Moscow/CRIE-GSE/2009
HA	KC780085	H1N1	07/25/2009	USA	A/Utah/20/2009
HA	KC780096	H1N1	07/25/2009	USA	A/Utah/20/2009
HA	CY055732	H1N1	07/25/2009	Australia	A/Australia/42/2009(H1N1)
HA	CY103913	H1N1	07/25/2009	Brazil	A/Pernambuco/126/2009

HA	CY070329	H1N1	07/25/2009	United Ki	A/England/93160009/2009
HA	KC782202	H1N1	07/25/2009	USA	A/Utah/20/2009
HA	CY075923	H1N1	07/25/2009	India	A/Pune/NIV759/2009
HA	CY128091	H1N1	07/25/2009	Viet Nam	A/Viet Nam/818/2009
HA	CY051663	H1N1	07/25/2009	USA	A/New York/4735/2009(H1N1)
HA	GU057010	H1N1	07/26/2009	China	A/Chengdu/03/2009(H1N1)
HA	CY055748	H1N1	07/26/2009	Australia	A/Australia/44/2009(H1N1)
HA	GU057014	H1N1	07/26/2009	China	A/Chengdu/18/2009(H1N1)
HA	GU057015	H1N1	07/26/2009	China	A/Chengdu/21/2009(H1N1)
HA	GU057016	H1N1	07/26/2009	China	A/Chengdu/31/2009(H1N1)
HA	GU057018	H1N1	07/26/2009	China	A/Chengdu/39/2009(H1N1)
HA	GU057020	H1N1	07/26/2009	China	A/Chengdu/47/2009(H1N1)
HA	GU057021	H1N1	07/26/2009	China	A/Chengdu/54/2009(H1N1)
HA	CY070327	H1N1	07/26/2009	United Ki	A/England/93160003/2009
HA	CY070343	H1N1	07/26/2009	United Ki	A/England/655/2009
HA	CY075779	H1N1	07/26/2009	Thailand	A/Thailand/CU-H300/2009(H1N1)
HA	GU057019	H1N1	07/26/2009	China	A/Chengdu/40/2009(H1N1)
HA	GU057012	H1N1	07/26/2009	China	A/Chengdu/06/2009(H1N1)
HA	GU057017	H1N1	07/26/2009	China	A/Chengdu/33/2009(H1N1)
HA	CY128107	H1N1	07/26/2009	Viet Nam	A/Viet Nam/823/2009
HA	KC781459	H1N1	07/26/2009	USA	A/Hawaii/22/2009
HA	KT181093	H1N1	07/26/2009	Singapore	A/Singapore/DMS17/2009
HA	KC967084	H1N1	07/26/2009	Brazil	A/Rio de Janeiro/5901/2009
HA	CY055740	H1N1	07/26/2009	Australia	A/Australia/43/2009(H1N1)
HA	GU057011	H1N1	07/26/2009	China	A/Chengdu/04/2009(H1N1)
HA	GU057013	H1N1	07/26/2009	China	A/Chengdu/14/2009(H1N1)
HA	CY055764	H1N1	07/27/2009	Australia	A/Australia/47/2009(H1N1)
HA	CY054617	H1N1	07/27/2009	Thailand	A/Thailand/THB0434/2009(H1N1)
HA	CY111859	H1N1	07/27/2009	Hong Kong	A/Hong Kong/H090-745-V10/2009(H1N1)
HA	GQ915076	H1N1	07/27/2009	Singapore	A/Singapore/TLL10/2009(H1N1)
HA	CY070328	H1N1	07/27/2009	United Ki	A/England/615/2009
HA	CY070326	H1N1	07/27/2009	United Ki	A/England/673/2009
HA	HQ228064	H1N1	07/27/2009	Finland	A/Finland/615/2009
HA	HQ228065	H1N1	07/27/2009	Finland	A/Finland/616/2009
HA	CY053922	H1N1	07/27/2009	Argentina	A/Argentina/HNRG23/2009(H1N1)
HA	JF789634	H1N1	07/27/2009	Kazakhsta	A/Astana/S1/2009
HA	HM752481	H1N1	07/27/2009	Thailand	A/Thailand/CU-MV14/2009
HA	CY070345	H1N1	07/27/2009	United Ki	A/England/657/2009
HA	CY054616	H1N1	07/27/2009	Thailand	A/Thailand/THB0433/2009(H1N1)
HA	GU226572	H1N1	07/27/2009	Iran	A/Lorestan/1599/2009(H1N1)
HA	CY065928	H1N1	07/27/2009	Guam	A/Guam/NHRC0001/2009(H1N1)
HA	CY072622	H1N1	07/27/2009	Nicaragua	A/Managua/5339.04/2009(H1N1)
HA	CY072726	H1N1	07/27/2009	Nicaragua	A/Managua/4407.02/2009(H1N1)
HA	CY075785	H1N1	07/27/2009	Thailand	A/Thailand/CU-H302/2009(H1N1)
HA	CY052367	H1N1	07/27/2009	USA	A/Texas/43272683/2009(H1N1)
HA	AB530470	H1N1	07/27/2009	Japan	A/Nagasaki/HA-18/2009(H1N1)
HA	AB530469	H1N1	07/27/2009	Japan	A/Nagasaki/HA-17/2009(H1N1)
HA	CY128099	H1N1	07/27/2009	Viet Nam	A/Viet Nam/835/2009
HA	CY128123	H1N1	07/27/2009	Viet Nam	A/Viet Nam/841/2009
HA	JF789637	H1N1	07/27/2009	Kazakhsta	A/Astana/S4/2009
HA	HM998844	H1N1	07/27/2009	China	A/Inner Mongolia/01/2009(H1N1)
HA	KF667883	H1N1	07/27/2009	Singapore	A/Singapore/386T/2009
HA	KF700932	H1N1	07/27/2009	Brazil	A/Rio Grande do Sul/5922/2009
HA	CY120084	H1N1	07/27/2009	Hong Kong	A/Hong Kong/H090-745-V10/2009

HA	JN256828	H1N1	07/27/2009	Hong Kong	A/Hong Kong/H090-745-V1(0)/2009
HA	HQ595234	H1N1	07/27/2009	Brazil	A/Sao Paulo/50280/2009
HA	JF789636	H1N1	07/27/2009	Kazakhsta	A/Astana/S3/2009
HA	HQ595237	H1N1	07/27/2009	Brazil	A/Sao Paulo/51533/2009
HA	KF667882	H1N1	07/27/2009	Singapore	A/Singapore/385K/2009
HA	CY122819	H1N1	07/27/2009	Singapore	A/Singapore/GP2224/2009
HA	KC781835	H1N1	07/27/2009	USA	A/South Carolina/28/2009
HA	JQ173148	H1N1	07/27/2009	Finland	A/Helsinki/P14/2009
HA	KC967090	H1N1	07/27/2009	Brazil	A/Rio de Janeiro/5773/2009
HA	KC967088	H1N1	07/27/2009	Brazil	A/Rio de Janeiro/5721/2009
HA	CY083328	H1N1	07/27/2009	USA	A/Cape May/WRAIR1653P/2009
HA	JN187125	H1N1	07/27/2009	Taiwan	A/Taiwan/2826/2009
HA	CY055756	H1N1	07/27/2009	Australia	A/Australia/45/2009(H1N1)
HA	CY055772	H1N1	07/27/2009	Australia	A/Australia/48/2009(H1N1)
HA	CY051986	H1N1	07/27/2009	Norway	A/Norway/2674/2009(H1N1)
HA	JF789635	H1N1	07/27/2009	Kazakhsta	A/Astana/S2/2009
HA	CY073657	H1N1	07/27/2009	Nicaragua	A/Managua/3225.03/2009(H1N1)
HA	CY122811	H1N1	07/27/2009	Singapore	A/Singapore/GP2223/2009
HA	CY073529	H1N1	07/27/2009	Nicaragua	A/Managua/466.01/2009(H1N1)
HA	CY054995	H1N1	07/27/2009	USA	A/California/VRDL61/2009(H1N1)
HA	CY054621	H1N1	07/28/2009	Thailand	A/Thailand/THB0441/2009(H1N1)
HA	CY070341	H1N1	07/28/2009	United Ki	A/Scotland/24/2009
HA	HQ595235	H1N1	07/28/2009	Brazil	A/Sao Paulo/51307/2009
HA	CY070342	H1N1	07/28/2009	United Ki	A/Scotland/25/2009
HA	HQ595236	H1N1	07/28/2009	Brazil	A/Sao Paulo/51366/2009
HA	CY083240	H1N1	07/28/2009	USA	A/Virginia/WRAIR1511P/2009
HA	CY054619	H1N1	07/28/2009	Thailand	A/Thailand/THB0437/2009(H1N1)
HA	CY128115	H1N1	07/28/2009	Viet Nam	A/Viet Nam/850/2009
HA	KC781671	H1N1	07/28/2009	USA	A/Washington/29/2009
HA	KF667885	H1N1	07/28/2009	Singapore	A/Singapore/392N/2009
HA	CY119530	H1N1	07/28/2009	Malaysia	A/Malaysia/2072040/2009
HA	CY120285	H1N1	07/28/2009	Hong Kong	A/Hong Kong/H090-742-V33/2009
HA	CY120293	H1N1	07/28/2009	Hong Kong	A/Hong Kong/H090-746-V10/2009
HA	CY122843	H1N1	07/28/2009	Singapore	A/Singapore/GP2252/2009
HA	CY122803	H1N1	07/28/2009	Singapore	A/Singapore/GP2221/2009
HA	CY122827	H1N1	07/28/2009	Singapore	A/Singapore/GP2238/2009
HA	CY123477	H1N1	07/28/2009	Singapore	A/Singapore/ON1576/2009
HA	JN256830	H1N1	07/28/2009	Hong Kong	A/Hong Kong/H090-746-V1(0)/2009
HA	CY054280	H1N1	07/28/2009	Brazil	A/Rio de Janeiro/5826/2009(H1N1)
HA	CY054618	H1N1	07/28/2009	Thailand	A/Thailand/THB0435/2009(H1N1)
HA	JN256827	H1N1	07/28/2009	Hong Kong	A/Hong Kong/H090-742-V3(3)/2009
HA	CY070340	H1N1	07/28/2009	United Ki	A/Scotland/23/2009
HA	KF667884	H1N1	07/28/2009	Singapore	A/Singapore/390T/2009
HA	GQ499334	H1N1	07/28/2009	USA	A/Washington/29/2009(H1N1)
HA	KC781634	H1N1	07/28/2009	USA	A/Washington/49/2009
HA	HM189385	H1N1	07/28/2009	Russia	A/Chelyabinsk/CRIE-KAI/2009
HA	CY111851	H1N1	07/28/2009	Hong Kong	A/Hong Kong/H090-742-V33/2009
HA	CY054620	H1N1	07/28/2009	Thailand	A/Thailand/THB0438/2009(H1N1)
HA	CY055780	H1N1	07/28/2009	Australia	A/Australia/51/2009(H1N1)
HA	CY051671	H1N1	07/28/2009	USA	A/New York/4738/2009(H1N1)
HA	CY073633	H1N1	07/28/2009	Nicaragua	A/Managua/4960.01/2009(H1N1)
HA	CY111875	H1N1	07/28/2009	Hong Kong	A/Hong Kong/H090-746-V10/2009(H1N1)
HA	CY065944	H1N1	07/29/2009	Guam	A/Guam/NHRC0003/2009(H1N1)
HA	CY070351	H1N1	07/29/2009	United Ki	A/England/622/2009

HA	CY051987	H1N1	07/29/2009	Norway	A/Norway/2690/2009(H1N1)
HA	AB535738	H1N1	07/29/2009	Japan	A/Nagasaki/HA-24/2009(H1N1)
HA	CY052348	H1N1	07/29/2009	Brazil	A/Rio Grande do Sul/7019/2009(H1N1)
HA	AB530471	H1N1	07/29/2009	Japan	A/Nagasaki/HA-22/2009(H1N1)
HA	HQ595239	H1N1	07/29/2009	Brazil	A/Sao Paulo/51801/2009
HA	HQ595238	H1N1	07/29/2009	Brazil	A/Sao Paulo/51583/2009
HA	CY070334	H1N1	07/29/2009	United Ki	A/England/674/2009
HA	CY083224	H1N1	07/29/2009	USA	A/California/WRAIR1507P/2009
HA	JN171886	H1N1	07/29/2009	Canada	A/Quebec/QC148621/2009
HA	CY054622	H1N1	07/29/2009	Thailand	A/Thailand/THB0442/2009(H1N1)
HA	GU226571	H1N1	07/29/2009	Iran	A/Khorasan/1583/2009(H1N1)
HA	CY073553	H1N1	07/29/2009	Nicaragua	A/Managua/2978.04/2009(H1N1)
HA	CY052282	H1N1	07/29/2009	USA	A/Texas/43292238/2009(H1N1)
HA	CY070332	H1N1	07/29/2009	United Ki	A/England/643/2009
HA	CY128451	H1N1	07/29/2009	Viet Nam	A/Viet Nam/001-1880/2009
HA	KC781541	H1N1	07/29/2009	USA	A/South Carolina/16/2009
HA	CY122872	H1N1	07/29/2009	Singapore	A/Singapore/GP2297/2009
HA	KF667886	H1N1	07/29/2009	Singapore	A/Singapore/394T/2009
HA	JN256832	H1N1	07/29/2009	Hong Kong	A/Hong Kong/H090-751-V1(0)/2009
HA	CY111891	H1N1	07/29/2009	Hong Kong	A/Hong Kong/H090-751-V10/2009
HA	CY070333	H1N1	07/29/2009	United Ki	A/England/616/2009
HA	CY054282	H1N1	07/29/2009	Brazil	A/Rio Grande do Sul/7108/2009(H1N1)
HA	CY120301	H1N1	07/29/2009	Hong Kong	A/Hong Kong/H090-751-V10/2009
HA	CY070331	H1N1	07/29/2009	United Ki	A/England/93160032/2009
HA	CY070336	H1N1	07/29/2009	United Ki	A/England/93200002/2009
HA	KF667887	H1N1	07/29/2009	Singapore	A/Singapore/395T/2009
HA	CY122795	H1N1	07/29/2009	Singapore	A/Singapore/GP2215/2009
HA	KC781917	H1N1	07/29/2009	USA	A/South Carolina/31/2009
HA	KC967083	H1N1	07/29/2009	Brazil	A/Rio de Janeiro/5900/2009
HA	KT181094	H1N1	07/29/2009	Singapore	A/Singapore/DMS18/2009
HA	KP317222	H1N1	07/29/2009	India	A/Delhi/018/2009
HA	KC967089	H1N1	07/29/2009	Brazil	A/Rio de Janeiro/5772/2009
HA	CY054593	H1N1	07/29/2009	Thailand	A/Thailand/0440-00-N0/2009(H1N1)
HA	CY054623	H1N1	07/29/2009	Thailand	A/Thailand/THB0444/2009(H1N1)
HA	CY122835	H1N1	07/29/2009	Singapore	A/Singapore/GP2242/2009
HA	KC781490	H1N1	07/29/2009	USA	A/Hawaii/25/2009
HA	CY075791	H1N1	07/29/2009	Thailand	A/Thailand/CU-H313/2009(H1N1)
HA	CY055788	H1N1	07/29/2009	Australia	A/Australia/53/2009(H1N1)
HA	JN256831	H1N1	07/30/2009	Hong Kong	A/Hong Kong/H090-746-V2(0)/2009
HA	JN256839	H1N1	07/30/2009	Hong Kong	A/Hong Kong/H090-758-V1(0)/2009
HA	JN256840	H1N1	07/30/2009	Hong Kong	A/Hong Kong/H090-759-V1(0)/2009
HA	CY111883	H1N1	07/30/2009	Hong Kong	A/Hong Kong/H090-746-V20/2009
HA	JN412821	H1N1	07/30/2009	Indonesia	A/Jakarta/005/2009
HA	JN412822	H1N1	07/30/2009	Indonesia	A/Jakarta/006/2009
HA	JN389446	H1N1	07/30/2009	Indonesia	A/Jakarta/001/2009
HA	CY111939	H1N1	07/30/2009	Hong Kong	A/Hong Kong/H090-757-V10/2009
HA	CY070330	H1N1	07/30/2009	United Ki	A/England/93160014/2009
HA	CY111947	H1N1	07/30/2009	Hong Kong	A/Hong Kong/H090-758-V10/2009
HA	GU226570	H1N1	07/30/2009	Iran	A/Ghom/1550/2009(H1N1)
HA	HQ595240	H1N1	07/30/2009	Brazil	A/Sao Paulo/52142/2009
HA	CY051679	H1N1	07/30/2009	USA	A/New York/4747/2009(H1N1)
HA	AB530473	H1N1	07/30/2009	Japan	A/Nagasaki/HA-26/2009(H1N1)
HA	AB530474	H1N1	07/30/2009	Japan	A/Nagasaki/HA-27/2009(H1N1)
HA	AB530476	H1N1	07/30/2009	Japan	A/Nagasaki/HA-29/2009(H1N1)

HA	KM013725	H1N1	07/30/2009	Thailand	A/Bangkok/SIMI501/2009
HA	KM013731	H1N1	07/30/2009	Thailand	A/Bangkok/SIMI507/2009
HA	CY123479	H1N1	07/30/2009	Singapore	A/Singapore/ON1609/2009
HA	CY123481	H1N1	07/30/2009	Singapore	A/Singapore/ON1616/2009
HA	CY122887	H1N1	07/30/2009	Singapore	A/Singapore/GP2317/2009
HA	JN256838	H1N1	07/30/2009	Hong Kong	A/Hong Kong/H090-757-V1(0)/2009
HA	CY120349	H1N1	07/30/2009	Hong Kong	A/Hong Kong/H090-757-V10/2009
HA	CY120365	H1N1	07/30/2009	Hong Kong	A/Hong Kong/H090-759-V10/2009
HA	CY055804	H1N1	07/30/2009	Australia	A/Australia/56/2009(H1N1)
HA	AB530472	H1N1	07/30/2009	Japan	A/Nagasaki/HA-25/2009(H1N1)
HA	CY122859	H1N1	07/30/2009	Singapore	A/Singapore/GP2290/2009
HA	CY122867	H1N1	07/30/2009	Singapore	A/Singapore/GP2293/2009
HA	CY111955	H1N1	07/30/2009	Hong Kong	A/Hong Kong/H090-759-V10/2009
HA	AB535739	H1N1	07/30/2009	Japan	A/Nagasaki/HA-30/2009(H1N1)
HA	CY054594	H1N1	07/30/2009	Thailand	A/Thailand/0445-00-NO/2009(H1N1)
HA	CY070335	H1N1	07/30/2009	United Ki	A/England/644/2009
HA	KF700935	H1N1	07/30/2009	Brazil	A/Rio Grande do Sul/7313/2009
HA	CY072630	H1N1	07/30/2009	Nicaragua	A/Managua/1836.02/2009(H1N1)
HA	CY122852	H1N1	07/30/2009	Singapore	A/Singapore/GP2286/2009
HA	KC781726	H1N1	07/30/2009	USA	A/Oregon/27/2009
HA	KF700933	H1N1	07/30/2009	Brazil	A/Rio de Janeiro/6529/2009
HA	HM189380	H1N1	07/30/2009	Russia	A/Nizhni Novgorod/CRIE-MEV/2009
HA	KP637326	H1N1	07/30/2009	Thailand	A/Thailand/KS08277/2009
HA	KY925947	H1N1	07/30/2009	Brazil	A/Porto Alegre/LACENRS-1771/2009
HA	AB530475	H1N1	07/30/2009	Japan	A/Nagasaki/HA-28/2009(H1N1)
HA	HQ595241	H1N1	07/30/2009	Brazil	A/Sao Paulo/52249/2009
HA	KC782297	H1N1	07/30/2009	USA	A/Kentucky/16/2009
HA	CY120357	H1N1	07/30/2009	Hong Kong	A/Hong Kong/H090-758-V10/2009
HA	CY122879	H1N1	07/30/2009	Singapore	A/Singapore/GP2315/2009
HA	CY122848	H1N1	07/30/2009	Singapore	A/Singapore/GP2266/2009
HA	CY070344	H1N1	07/31/2009	United Ki	A/England/656/2009
HA	CY120317	H1N1	07/31/2009	Hong Kong	A/Hong Kong/H090-751-V23/2009
HA	JN256843	H1N1	07/31/2009	Hong Kong	A/Hong Kong/H090-765-V1(0)/2009
HA	JN256829	H1N1	07/31/2009	Hong Kong	A/Hong Kong/H090-745-V2(4)/2009
HA	JN256833	H1N1	07/31/2009	Hong Kong	A/Hong Kong/H090-751-V2(0)/2009
HA	CY111963	H1N1	07/31/2009	Hong Kong	A/Hong Kong/H090-761-V10/2009
HA	CY111867	H1N1	07/31/2009	Hong Kong	A/Hong Kong/H090-745-V24/2009
HA	HQ595243	H1N1	07/31/2009	Brazil	A/Sao Paulo/53137/2009
HA	CY070347	H1N1	07/31/2009	United Ki	A/England/659/2009
HA	CY070356	H1N1	07/31/2009	United Ki	A/England/663/2009
HA	CY083216	H1N1	07/31/2009	USA	A/New Jersey/WRAIR1506P/2009(H1N1)
HA	CY071578	H1N1	07/31/2009	USA	A/South Carolina/WR1326P/2009(H1N1)
HA	CY120389	H1N1	07/31/2009	Hong Kong	A/Hong Kong/H090-765-V10/2009
HA	CY111899	H1N1	07/31/2009	Hong Kong	A/Hong Kong/H090-751-V20/2009(H1N1)
HA	CY120739	H1N1	07/31/2009	Brazil	A/Brazil/AVS07/2009
HA	CY111907	H1N1	07/31/2009	Hong Kong	A/Hong Kong/H090-751-V23/2009
HA	CY111915	H1N1	07/31/2009	Hong Kong	A/Hong Kong/H090-751-V24/2009(H1N1)
HA	JN256841	H1N1	07/31/2009	Hong Kong	A/Hong Kong/H090-761-V1(0)/2009
HA	KY925404	H1N1	07/31/2009	Brazil	A/Igrejinha/LACENRS-1803/2009
HA	CY111971	H1N1	07/31/2009	Hong Kong	A/Hong Kong/H090-762-V10/2009
HA	CY111979	H1N1	07/31/2009	Hong Kong	A/Hong Kong/H090-765-V10/2009
HA	CY070983	H1N1	07/31/2009	Guam	A/Guam/NHRC0007/2009(H1N1)
HA	KF700934	H1N1	07/31/2009	Brazil	A/Rio de Janeiro/6827/2009
HA	CY068930	H1N1	07/31/2009	Guam	A/Guam/NHRC0005/2009(H1N1)

HA	KY926019	H1N1	07/31/2009	Brazil	A/Estrela/LACENRS-313/2009
HA	KF667892	H1N1	07/31/2009	Singapore	A/Singapore/410W/2009
HA	CY071570	H1N1	07/31/2009	USA	A/South Carolina/WR1325P/2009(H1N1)
HA	KY925200	H1N1	07/31/2009	Brazil	A/Porto Alegre/LACENRS-1786/2009
HA	HQ595242	H1N1	07/31/2009	Brazil	A/Sao Paulo/52281/2009
HA	KC782112	H1N1	07/31/2009	USA	A/South Carolina/30/2009
HA	KC782299	H1N1	07/31/2009	USA	A/Illinois/10/2009
HA	CY055246	H1N1	07/31/2009	Malaysia	A/Malaysia/5670/2009(H1N1)
HA	JN256834	H1N1	07/31/2009	Hong Kong	A/Hong Kong/H090-751-V2(3)/2009
HA	KF667889	H1N1	07/31/2009	Singapore	A/Singapore/404C/2009
HA	KF667897	H1N1	07/31/2009	Singapore	A/Singapore/419B/2009
HA	KF667890	H1N1	07/31/2009	Singapore	A/Singapore/405S/2009
HA	JN256835	H1N1	07/31/2009	Hong Kong	A/Hong Kong/H090-751-V2(4)/2009
HA	CY119362	H1N1	07/31/2009	Malaysia	A/Malaysia/2068631/2009
HA	KF667894	H1N1	07/31/2009	Singapore	A/Singapore/416C/2009
HA	KF667895	H1N1	07/31/2009	Singapore	A/Singapore/417C/2009
HA	KF667896	H1N1	07/31/2009	Singapore	A/Singapore/418W/2009
HA	KF667888	H1N1	07/31/2009	Singapore	A/Singapore/401T/2009
HA	KF667891	H1N1	07/31/2009	Singapore	A/Singapore/406K/2009
HA	KF667893	H1N1	07/31/2009	Singapore	A/Singapore/413Y/2009
HA	CY120309	H1N1	07/31/2009	Hong Kong	A/Hong Kong/H090-751-V20/2009
HA	CY120325	H1N1	07/31/2009	Hong Kong	A/Hong Kong/H090-751-V24/2009
HA	CY120373	H1N1	07/31/2009	Hong Kong	A/Hong Kong/H090-761-V10/2009
HA	CY120381	H1N1	07/31/2009	Hong Kong	A/Hong Kong/H090-762-V10/2009
HA	JN256842	H1N1	07/31/2009	Hong Kong	A/Hong Kong/H090-762-V1(0)/2009
HA	AB568228	H1N1	08/2009	Japan	A/Kobe/91799/2009
HA	GU369655	H1N1	08/2009	Turkey	A/Ankara/11/2009
HA	GU369657	H1N1	08/2009	Turkey	A/Ankara/13/2009(H1N1)
HA	CY050339	H1N1	08/2009	Singapore	A/Singapore/9061364/2009(H1N1)
HA	CY062377	H1N1	08/2009	Afghanistan	A/Afghanistan/N09787/2009(H1N1)
HA	CY062387	H1N1	08/2009	Afghanistan	A/Afghanistan/N09840/2009(H1N1)
HA	CY062378	H1N1	08/2009	Afghanistan	A/Afghanistan/N09795/2009(H1N1)
HA	CY062383	H1N1	08/2009	Afghanistan	A/Afghanistan/N09828/2009(H1N1)
HA	CY062380	H1N1	08/2009	Afghanistan	A/Afghanistan/N09817/2009(H1N1)
HA	GU968909	H1N1	08/2009	China	A/Beijing/SE931/2009(H1N1)
HA	AB568233	H1N1	08/2009	Japan	A/Kobe/91821/2009
HA	AB568238	H1N1	08/2009	Japan	A/Kobe/91823/2009
HA	AB568218	H1N1	08/2009	Japan	A/Kobe/91738/2009
HA	AB568248	H1N1	08/2009	Japan	A/Kobe/91873/2009
HA	AB568223	H1N1	08/2009	Japan	A/Kobe/91780/2009
HA	AB568243	H1N1	08/2009	Japan	A/Kobe/91825/2009
HA	HM446345	H1N1	08/2009	Thailand	A/Thailand/CU-H88/2009
HA	HM204573	H1N1	08/2009	India	A/Jalna/NIV9436/2009
HA	HM204574	H1N1	08/2009	India	A/Ytml/NIV9438/2009
HA	HM204571	H1N1	08/2009	India	A/Blore/NIV1189/2009
HA	HM204575	H1N1	08/2009	India	A/Mum/NIV9312/2009
HA	JN381222	H1N1	08/2009	Taiwan	A/Taiwan/98031/2009
HA	JN381223	H1N1	08/2009	Taiwan	A/Taiwan/98158/2009
HA	JN381218	H1N1	08/2009	Taiwan	A/Taiwan/90060/2009
HA	JN381221	H1N1	08/2009	Taiwan	A/Taiwan/97888/2009
HA	JQ364989	H1N1	08/2009	China	A/Hangzhou/3/2009
HA	AB745121	H1N1	08/2009	Japan	A/Tottori/ST215/2009
HA	GU369666	H1N1	08/2009	Turkey	A/Ankara/22/2009
HA	GU369652	H1N1	08/2009	Turkey	A/Ankara/08/2009

HA	GU369662	H1N1	08/2009	Turkey	A/Ankara/18/2009
HA	CY062379	H1N1	08/2009	Afghanist	A/Afghanistan/N09800/2009(H1N1)
HA	JN381220	H1N1	08/2009	Taiwan	A/Taiwan/97674/2009
HA	CY062381	H1N1	08/2009	Afghanist	A/Afghanistan/N09821/2009(H1N1)
HA	GU369650	H1N1	08/2009	Turkey	A/Ankara/06/2009(H1N1)
HA	CY050347	H1N1	08/2009	Singapore	A/Singapore/9061365/2009(H1N1)
HA	AB568253	H1N1	08/2009	Japan	A/Kobe/91890/2009
HA	CY062386	H1N1	08/2009	Afghanist	A/Afghanistan/N09836/2009(H1N1)
HA	HM204572	H1N1	08/2009	India	A/Dhule/NIV9433/2009
HA	CY050355	H1N1	08/2009	Singapore	A/Singapore/9061366/2009(H1N1)
HA	CY062382	H1N1	08/2009	Afghanist	A/Afghanistan/N09827/2009(H1N1)
HA	CY062384	H1N1	08/2009	Afghanist	A/Afghanistan/N09831/2009(H1N1)
HA	JN381219	H1N1	08/2009	Taiwan	A/Taiwan/97565/2009
HA	JN381217	H1N1	08/2009	Taiwan	A/Taiwan/90052/2009
HA	CY062385	H1N1	08/2009	Afghanist	A/Afghanistan/N09833/2009(H1N1)
HA	CY055812	H1N1	08/01/2009	Australia	A/Australia/57/2009(H1N1)
HA	HQ595244	H1N1	08/01/2009	Brazil	A/Sao Paulo/53829/2009
HA	GQ915022	H1N1	08/01/2009	Brazil	A/Sao Paulo/54392/2009
HA	GQ915018	H1N1	08/01/2009	Brazil	A/Sao Paulo/53225/2009
HA	CY072154	H1N1	08/01/2009	USA	A/Texas/AF2648/2009(H1N1)
HA	CY075821	H1N1	08/01/2009	Thailand	A/Thailand/CU-H491/2009(H1N1)
HA	KT181095	H1N1	08/01/2009	Singapore	A/Singapore/DMS19/2009
HA	KC781511	H1N1	08/01/2009	USA	A/Colorado/11/2009
HA	KC780436	H1N1	08/01/2009	USA	A/Texas/71/2009
HA	KC781561	H1N1	08/01/2009	USA	A/Iowa/07/2009
HA	CY055820	H1N1	08/01/2009	Australia	A/Australia/58/2009(H1N1)
HA	CY070355	H1N1	08/01/2009	United Ki	A/England/662/2009
HA	GQ915019	H1N1	08/01/2009	Brazil	A/Sao Paulo/53823/2009
HA	GQ915020	H1N1	08/01/2009	Brazil	A/Sao Paulo/53838/2009
HA	CY071562	H1N1	08/01/2009	USA	A/South Carolina/WR1324P/2009(H1N1)
HA	JN588775	H1N1	08/01/2009	Cambodia	A/Cambodia/T057/2009
HA	CY119370	H1N1	08/02/2009	Malaysia	A/Malaysia/2076204/2009
HA	CY055836	H1N1	08/02/2009	Australia	A/Australia/60/2009(H1N1)
HA	CY118199	H1N1	08/02/2009	Malaysia	A/Malaysia/2076212/2009
HA	CY120341	H1N1	08/02/2009	Hong Kong	A/Hong Kong/H090-751-V34/2009
HA	CY111923	H1N1	08/02/2009	Hong Kong	A/Hong Kong/H090-751-V31/2009(H1N1)
HA	JN256836	H1N1	08/02/2009	Hong Kong	A/Hong Kong/H090-751-V3(1)/2009
HA	CY111931	H1N1	08/02/2009	Hong Kong	A/Hong Kong/H090-751-V34/2009
HA	CY083168	H1N1	08/02/2009	USA	A/New Jersey/WRAIR1499P/2009(H1N1)
HA	GQ915021	H1N1	08/02/2009	Brazil	A/Sao Paulo/53845/2009
HA	JN256837	H1N1	08/02/2009	Hong Kong	A/Hong Kong/H090-751-V3(4)/2009
HA	CY055828	H1N1	08/02/2009	Australia	A/Australia/59/2009(H1N1)
HA	KC781816	H1N1	08/02/2009	USA	A/Nevada/09/2009
HA	KC781853	H1N1	08/02/2009	USA	A/Nevada/10/2009
HA	CY070353	H1N1	08/02/2009	United Ki	A/England/93280011/2009
HA	CY054601	H1N1	08/02/2009	Thailand	A/Thailand/PMKSV0305/2009(H1N1)
HA	CY070352	H1N1	08/02/2009	United Ki	A/England/661/2009
HA	CY120333	H1N1	08/02/2009	Hong Kong	A/Hong Kong/H090-751-V31/2009
HA	AB530478	H1N1	08/03/2009	Japan	A/Nagasaki/HA-32/2009(H1N1)
HA	AB530479	H1N1	08/03/2009	Japan	A/Nagasaki/HA-33/2009(H1N1)
HA	AB530481	H1N1	08/03/2009	Japan	A/Nagasaki/HA-35/2009(H1N1)
HA	GU576508	H1N1	08/03/2009	Italy	A/Ancona/56/2009(H1N1)
HA	CY055844	H1N1	08/03/2009	Australia	A/Australia/61/2009(H1N1)
HA	CY054624	H1N1	08/03/2009	Thailand	A/Thailand/THB0454/2009(H1N1)

HA	KC781824	H1N1	08/03/2009	USA	A/North Dakota/12/2009
HA	KC781767	H1N1	08/03/2009	USA	A/New Hampshire/17/2009
HA	JN171887	H1N1	08/03/2009	Canada	A/Quebec/QC148795/2009
HA	CY070346	H1N1	08/03/2009	United Ki	A/England/658/2009
HA	CY070348	H1N1	08/03/2009	United Ki	A/England/664/2009
HA	CY070350	H1N1	08/03/2009	United Ki	A/England/645/2009
HA	HQ595245	H1N1	08/03/2009	Brazil	A/Sao Paulo/54026/2009
HA	HQ595246	H1N1	08/03/2009	Brazil	A/Sao Paulo/55565/2009
HA	CY119378	H1N1	08/03/2009	Malaysia	A/Malaysia/2076039/2009
HA	KF667898	H1N1	08/03/2009	Singapore	A/Singapore/420T/2009
HA	KF667899	H1N1	08/03/2009	Singapore	A/Singapore/425H/2009
HA	KF667903	H1N1	08/03/2009	Singapore	A/Singapore/440F/2009
HA	KF667901	H1N1	08/03/2009	Singapore	A/Singapore/427O/2009
HA	CY070357	H1N1	08/03/2009	United Ki	A/England/646/2009
HA	AB530477	H1N1	08/03/2009	Japan	A/Nagasaki/HA-31/2009(H1N1)
HA	JX625475	H1N1	08/03/2009	United Ki	A/England/646/2009
HA	JN412823	H1N1	08/03/2009	Indonesia	A/Jakarta/007/2009
HA	CY119386	H1N1	08/03/2009	Malaysia	A/Malaysia/2077462/2009
HA	CY120405	H1N1	08/03/2009	Hong Kong	A/Hong Kong/H090-771-V10/2009
HA	CY122959	H1N1	08/03/2009	Singapore	A/Singapore/GP2714/2009
HA	CY111995	H1N1	08/03/2009	Hong Kong	A/Hong Kong/H090-771-V10/2009
HA	CY106976	H1N1	08/03/2009	Hong Kong	A/Hong Kong/H090-770-V10/2009
HA	CY072678	H1N1	08/03/2009	Nicaragua	A/Managua/209.01/2009(H1N1)
HA	CY072558	H1N1	08/03/2009	Nicaragua	A/Managua/451.01/2009(H1N1)
HA	KF667902	H1N1	08/03/2009	Singapore	A/Singapore/437Y/2009
HA	KF667904	H1N1	08/03/2009	Singapore	A/Singapore/441T/2009
HA	CY055868	H1N1	08/03/2009	Australia	A/Australia/65/2009(H1N1)
HA	CY055860	H1N1	08/03/2009	Australia	A/Australia/64/2009(H1N1)
HA	CY055852	H1N1	08/03/2009	Australia	A/Australia/63/2009(H1N1)
HA	AB530480	H1N1	08/03/2009	Japan	A/Nagasaki/HA-34/2009(H1N1)
HA	JN256845	H1N1	08/03/2009	Hong Kong	A/Hong Kong/H090-771-V1(0)/2009
HA	CY070349	H1N1	08/03/2009	United Ki	A/England/660/2009
HA	KF667900	H1N1	08/03/2009	Singapore	A/Singapore/426N/2009
HA	CY068946	H1N1	08/04/2009	Japan	A/Japan/NHRC0004/2009(H1N1)
HA	GU220604	H1N1	08/04/2009	Serbia	A/Serbia/3761/2009(H1N1)
HA	CY073601	H1N1	08/04/2009	Nicaragua	A/Managua/4451.02/2009(H1N1)
HA	CY073609	H1N1	08/04/2009	Nicaragua	A/Managua/1256.04/2009(H1N1)
HA	JN171888	H1N1	08/04/2009	Canada	A/Quebec/QC148853/2009
HA	CY070358	H1N1	08/04/2009	United Ki	A/England/93280030/2009
HA	JN256848	H1N1	08/04/2009	Hong Kong	A/Hong Kong/H090-774-V1(0)/2009
HA	CY112019	H1N1	08/04/2009	Hong Kong	A/Hong Kong/H090-774-V10/2009
HA	CY055876	H1N1	08/04/2009	Australia	A/Australia/66/2009(H1N1)
HA	CY070354	H1N1	08/04/2009	United Ki	A/England/93280013/2009
HA	CY120421	H1N1	08/04/2009	Hong Kong	A/Hong Kong/H090-774-V10/2009
HA	KF667906	H1N1	08/04/2009	Singapore	A/Singapore/444W/2009
HA	KF667905	H1N1	08/04/2009	Singapore	A/Singapore/443N/2009
HA	KT181096	H1N1	08/04/2009	Singapore	A/Singapore/DMS20/2009
HA	HM189384	H1N1	08/04/2009	Russia	A/Rostov oblast/CRIE-SHAV/2009
HA	CY119522	H1N1	08/04/2009	Malaysia	A/Malaysia/2076756/2009
HA	CY123486	H1N1	08/04/2009	Singapore	A/Singapore/ON1666/2009
HA	CY122895	H1N1	08/04/2009	Singapore	A/Singapore/GP2640/2009
HA	CY122903	H1N1	08/04/2009	Singapore	A/Singapore/GP2649/2009
HA	CY070975	H1N1	08/04/2009	Guam	A/Guam/NHRC0006/2009(H1N1)
HA	KC781497	H1N1	08/04/2009	USA	A/Montana/15/2009

HA	GU576510	H1N1	08/05/2009	Italy	A/Ancona/60/2009(H1N1)
HA	CY120437	H1N1	08/05/2009	Hong Kong	A/Hong Kong/H090-779-V12/2009
HA	CY071490	H1N1	08/05/2009	USA	A/California/WR1315P/2009(H1N1)
HA	CY073569	H1N1	08/05/2009	Nicaragua	A/Managua/5230.01/2009(H1N1)
HA	KC781905	H1N1	08/05/2009	USA	A/Colorado/12/2009
HA	KC781764	H1N1	08/05/2009	USA	A/Maryland/15/2009
HA	KC781662	H1N1	08/05/2009	USA	A/Wisconsin/45/2009
HA	CY072598	H1N1	08/05/2009	Nicaragua	A/Managua/1077.04/2009(H1N1)
HA	GQ915023	H1N1	08/05/2009	Brazil	A/Sao Paulo/55312/2009
HA	CY103915	H1N1	08/05/2009	Brazil	A/Pernambuco/177/2009
HA	CY075893	H1N1	08/05/2009	India	A/Mum/NIV1126/2009
HA	CY075894	H1N1	08/05/2009	India	A/Mum/NIV1134/2009
HA	CY122943	H1N1	08/05/2009	Singapore	A/Singapore/GP2697/2009
HA	JN256849	H1N1	08/05/2009	Hong Kong	A/Hong Kong/H090-779-V1(0)/2009
HA	JN256850	H1N1	08/05/2009	Hong Kong	A/Hong Kong/H090-779-V1(2)/2009
HA	CY103916	H1N1	08/05/2009	Brazil	A/Pernambuco/181/2009
HA	CY103914	H1N1	08/05/2009	Brazil	A/Pernambuco/175/2009
HA	AB535740	H1N1	08/05/2009	Japan	A/Nagasaki/HA-37/2009(H1N1)
HA	CY055035	H1N1	08/05/2009	USA	A/California/VRDL69/2009(H1N1)
HA	CY055884	H1N1	08/05/2009	Australia	A/Australia/69/2009(H1N1)
HA	CY049643	H1N1	08/05/2009	Singapore	A/Singapore/GP2651/2009(H1N1)
HA	KC782102	H1N1	08/05/2009	USA	A/Massachusetts/29/2009
HA	CY071498	H1N1	08/05/2009	USA	A/California/WR1316P/2009(H1N1)
HA	KJ690408	H1N1	08/05/2009	Uganda	A/Uganda/MUWRP-066/2009
HA	KJ690503	H1N1	08/05/2009	Uganda	A/Uganda/MUWRP-067/2009
HA	HM189388	H1N1	08/05/2009	Russia	A/St.Petersburg oblast/CRIE-SEV/2009
HA	HM189428	H1N1	08/05/2009	Russia	A/Moscowv/CRIE-MOV/2009
HA	KY926119	H1N1	08/05/2009	Brazil	A/Teutonia/LACENRS-711/2009
HA	JN256847	H1N1	08/05/2009	Hong Kong	A/Hong Kong/H090-772-V1(0)/2009
HA	GQ915024	H1N1	08/05/2009	Brazil	A/Sao Paulo/55115/2009
HA	CY112027	H1N1	08/05/2009	Hong Kong	A/Hong Kong/H090-779-V10/2009
HA	CY120413	H1N1	08/05/2009	Hong Kong	A/Hong Kong/H090-772-V10/2009
HA	CY122919	H1N1	08/05/2009	Singapore	A/Singapore/GP2672/2009
HA	CY119394	H1N1	08/05/2009	Malaysia	A/Malaysia/2082543/2009
HA	CY112003	H1N1	08/05/2009	Hong Kong	A/Hong Kong/H090-771-V20/2009
HA	CY112011	H1N1	08/05/2009	Hong Kong	A/Hong Kong/H090-772-V10/2009
HA	CY112035	H1N1	08/05/2009	Hong Kong	A/Hong Kong/H090-779-V12/2009
HA	CY120429	H1N1	08/05/2009	Hong Kong	A/Hong Kong/H090-779-V10/2009
HA	KY925619	H1N1	08/05/2009	Brazil	A/Nonoai/LACENRS-714/2009
HA	AB530482	H1N1	08/05/2009	Japan	A/Nagasaki/HA-36/2009(H1N1)
HA	JN256846	H1N1	08/05/2009	Hong Kong	A/Hong Kong/H090-771-V2(0)/2009
HA	CY122927	H1N1	08/05/2009	Singapore	A/Singapore/GP2680/2009
HA	CY122935	H1N1	08/05/2009	Singapore	A/Singapore/GP2693/2009
HA	CY071482	H1N1	08/05/2009	USA	A/California/WR1314P/2009(H1N1)
HA	KC782043	H1N1	08/05/2009	USA	A/North Carolina/28/2009
HA	CY049635	H1N1	08/05/2009	Singapore	A/Singapore/GP2641/2009(H1N1)
HA	CY055892	H1N1	08/06/2009	Australia	A/Australia/70/2009(H1N1)
HA	GU576512	H1N1	08/06/2009	Italy	A/Ancona/61/2009(H1N1)
HA	KC781944	H1N1	08/06/2009	USA	A/Nevada/14/2009
HA	KC781471	H1N1	08/06/2009	USA	A/Nebraska/13/2009
HA	CY070359	H1N1	08/06/2009	United Ki	A/England/618/2009
HA	JN256844	H1N1	08/06/2009	Hong Kong	A/Hong Kong/H090-770-V2(2)/2009
HA	CY122951	H1N1	08/06/2009	Singapore	A/Singapore/GP2709/2009
HA	JN412824	H1N1	08/06/2009	Indonesia	A/Jakarta/008/2009

HA	CY120397	H1N1	08/06/2009	Hong Kong	A/Hong Kong/H090-770-V22/2009
HA	CY049675	H1N1	08/06/2009	Singapore	A/Singapore/GP2695/2009(H1N1)
HA	CY049683	H1N1	08/06/2009	Singapore	A/Singapore/GP2703/2009(H1N1)
HA	JN596858	H1N1	08/06/2009	Russia	A/Orenburg/01/2009(H1N1)
HA	JQ714171	H1N1	08/06/2009	China	A/Tianjinjinnan/SWL164/2009
HA	KP317205	H1N1	08/06/2009	India	A/Karnataka/001/2009
HA	CY111987	H1N1	08/06/2009	Hong Kong	A/Hong Kong/H090-770-V22/2009
HA	JN412825	H1N1	08/06/2009	Indonesia	A/Jakarta/009/2009
HA	KC781609	H1N1	08/06/2009	USA	A/California/33/2009
HA	KF667907	H1N1	08/06/2009	Singapore	A/Singapore/448D/2009
HA	JX625483	H1N1	08/06/2009	United Ki	A/England/93320016/2009
HA	CY049667	H1N1	08/06/2009	Singapore	A/Singapore/GP2687/2009(H1N1)
HA	CY049699	H1N1	08/06/2009	Singapore	A/Singapore/GP2715/2009(H1N1)
HA	CY049691	H1N1	08/06/2009	Singapore	A/Singapore/GP2711/2009(H1N1)
HA	GU045582	H1N1	08/06/2009	Singapore	A/Singapore/TLL38/2009(H1N1)
HA	CY049659	H1N1	08/06/2009	Singapore	A/Singapore/GP2686/2009(H1N1)
HA	CY051687	H1N1	08/06/2009	USA	A/New York/4761/2009(H1N1)
HA	CY075896	H1N1	08/06/2009	India	A/Pune/NIV1166/2009
HA	CY122911	H1N1	08/06/2009	Singapore	A/Singapore/GP2658/2009
HA	CY068922	H1N1	08/06/2009	Guam	A/Guam/NHRC0004/2009(H1N1)
HA	CY071538	H1N1	08/07/2009	USA	A/California/WR1321P/2009(H1N1)
HA	KC781776	H1N1	08/07/2009	USA	A/Michigan/21/2009
HA	CY071530	H1N1	08/07/2009	USA	A/California/WR1320P/2009(H1N1)
HA	CY073521	H1N1	08/07/2009	Nicaragua	A/Managua/5399.04/2009(H1N1)
HA	HM624085	H1N1	08/07/2009	Australia	A/Perth/267/2009
HA	KJ690463	H1N1	08/07/2009	Uganda	A/Uganda/MUWRP-069/2009
HA	CY072614	H1N1	08/07/2009	Nicaragua	A/Managua/2604.02/2009(H1N1)
HA	CY119402	H1N1	08/07/2009	Malaysia	A/Malaysia/2086505/2009
HA	CY112067	H1N1	08/07/2009	Hong Kong	A/Hong Kong/H090-785-V10/2009
HA	CY054625	H1N1	08/07/2009	Thailand	A/Thailand/THB0472/2009(H1N1)
HA	KT181097	H1N1	08/07/2009	Singapore	A/Singapore/DMS21/2009
HA	JN256854	H1N1	08/07/2009	Hong Kong	A/Hong Kong/H090-785-V1(0)/2009
HA	CY120461	H1N1	08/07/2009	Hong Kong	A/Hong Kong/H090-785-V10/2009
HA	KC781736	H1N1	08/08/2009	USA	A/California/37/2009
HA	CY087016	H1N1	08/08/2009	USA	A/New York/4764/2009(H1N1)
HA	CY071466	H1N1	08/08/2009	USA	A/California/WR1312P/2009(H1N1)
HA	CY073625	H1N1	08/08/2009	Nicaragua	A/Managua/5595.03/2009(H1N1)
HA	CY073585	H1N1	08/08/2009	Nicaragua	A/Managua/4064.01/2009(H1N1)
HA	CY075797	H1N1	08/08/2009	Thailand	A/Thailand/CU-H354/2009(H1N1)
HA	JN256851	H1N1	08/08/2009	Hong Kong	A/Hong Kong/H090-779-V2(0)/2009
HA	JN256852	H1N1	08/08/2009	Hong Kong	A/Hong Kong/H090-779-V2(1)/2009
HA	JN256853	H1N1	08/08/2009	Hong Kong	A/Hong Kong/H090-779-V2(2)/2009
HA	JN256855	H1N1	08/08/2009	Hong Kong	A/Hong Kong/H090-787-V1(0)/2009
HA	CY112051	H1N1	08/08/2009	Hong Kong	A/Hong Kong/H090-779-V21/2009
HA	GU045583	H1N1	08/08/2009	Singapore	A/Singapore/TLL40/2009(H1N1)
HA	GU045584	H1N1	08/08/2009	Singapore	A/Singapore/TLL42/2009(H1N1)
HA	CY120445	H1N1	08/08/2009	Hong Kong	A/Hong Kong/H090-779-V21/2009
HA	CY120469	H1N1	08/08/2009	Hong Kong	A/Hong Kong/H090-787-V10/2009
HA	CY120453	H1N1	08/08/2009	Hong Kong	A/Hong Kong/H090-779-V22/2009
HA	CY120092	H1N1	08/08/2009	Hong Kong	A/Hong Kong/H090-779-V20/2009
HA	KP317212	H1N1	08/08/2009	India	A/Gujarat/008/2009
HA	CY112075	H1N1	08/08/2009	Hong Kong	A/Hong Kong/H090-787-V10/2009
HA	CY112059	H1N1	08/08/2009	Hong Kong	A/Hong Kong/H090-779-V22/2009
HA	CY112043	H1N1	08/08/2009	Hong Kong	A/Hong Kong/H090-779-V20/2009

HA	CY055248	H1N1	08/08/2009	Malaysia	A/Malaysia/8860/2009(H1N1)
HA	KC781567	H1N1	08/08/2009	USA	A/Kansas/17/2009
HA	KC781733	H1N1	08/09/2009	USA	A/Oregon/30/2009
HA	CY060542	H1N1	08/09/2009	Canada	A/Ontario/25389/2009
HA	CY119418	H1N1	08/09/2009	Malaysia	A/Malaysia/2089888/2009
HA	KC781740	H1N1	08/09/2009	USA	A/Wyoming/08/2009
HA	CY119410	H1N1	08/09/2009	Malaysia	A/Malaysia/2089841/2009
HA	CY103917	H1N1	08/09/2009	Brazil	A/Pernambuco/231/2009
HA	GQ915025	H1N1	08/09/2009	Brazil	A/Sao Paulo/57831/2009
HA	AB530483	H1N1	08/10/2009	Japan	A/Nagasaki/HA-38/2009(H1N1)
HA	AB530484	H1N1	08/10/2009	Japan	A/Nagasaki/HA-39/2009(H1N1)
HA	CY055003	H1N1	08/10/2009	USA	A/California/VRDL64/2009(H1N1)
HA	KC781892	H1N1	08/10/2009	USA	A/Alaska/34/2009
HA	CY071514	H1N1	08/10/2009	USA	A/California/WR1318P/2009(H1N1)
HA	CY072686	H1N1	08/10/2009	Nicaragua	A/Managua/554.01/2009(H1N1)
HA	CY119426	H1N1	08/10/2009	Malaysia	A/Malaysia/2098001/2009
HA	CY119442	H1N1	08/10/2009	Malaysia	A/Malaysia/2098053/2009
HA	KT181098	H1N1	08/10/2009	Singapore	A/Singapore/DMS22/2009
HA	CY055011	H1N1	08/10/2009	USA	A/California/VRDL65/2009(H1N1)
HA	CY055494	H1N1	08/10/2009	USA	A/California/VRDL74/2009(H1N1)
HA	CY055900	H1N1	08/10/2009	Australia	A/Australia/72/2009(H1N1)
HA	CY071506	H1N1	08/10/2009	USA	A/California/WR1317P/2009(H1N1)
HA	KC781659	H1N1	08/10/2009	USA	A/Florida/14/2009
HA	CY054626	H1N1	08/10/2009	Thailand	A/Thailand/THB0474/2009(H1N1)
HA	KC781537	H1N1	08/10/2009	USA	A/Florida/13/2009
HA	CY073545	H1N1	08/10/2009	Nicaragua	A/Managua/3083.01/2009(H1N1)
HA	HM014332	H1N1	08/10/2009	China	A/Guangzhou/GIRD07/2009(H1N1)
HA	JX625230	H1N1	08/10/2009	Paraguay	A/Paraguay/85HCl/2009
HA	JX625231	H1N1	08/10/2009	Paraguay	A/Paraguay/86HCl/2009
HA	HM189401	H1N1	08/10/2009	Russia	A/Tver/CRIE-BVN/2009
HA	KY935468	H1N1	08/10/2009	Brazil	A/Rio Grande/LACENRS-1406/2009
HA	HQ595247	H1N1	08/10/2009	Brazil	A/Sao Paulo/59118/2009
HA	KC781494	H1N1	08/10/2009	USA	A/Vermont/17/2009
HA	KC781626	H1N1	08/10/2009	USA	A/Delaware/10/2009
HA	CY119434	H1N1	08/10/2009	Malaysia	A/Malaysia/2098044/2009
HA	CY051989	H1N1	08/10/2009	Norway	A/Norway/2924/2009(H1N1)
HA	CY119450	H1N1	08/11/2009	Malaysia	A/Malaysia/2097717/2009
HA	CY123499	H1N1	08/11/2009	Singapore	A/Singapore/ON1754/2009
HA	CY103918	H1N1	08/11/2009	Brazil	A/Pernambuco/247/2009
HA	CY073537	H1N1	08/11/2009	Nicaragua	A/Managua/4012.01/2009(H1N1)
HA	CY123494	H1N1	08/11/2009	Singapore	A/Singapore/ON1747/2009
HA	HQ595233	H1N1	08/11/2009	Brazil	A/Sao Paulo/21711/2009
HA	CY051991	H1N1	08/11/2009	Norway	A/Norway/3059/2009(H1N1)
HA	CY072662	H1N1	08/11/2009	Nicaragua	A/Managua/2339.03/2009(H1N1)
HA	CY072646	H1N1	08/11/2009	Nicaragua	A/Managua/2275.01/2009(H1N1)
HA	KC781664	H1N1	08/11/2009	USA	A/Utah/30/2009
HA	KC781474	H1N1	08/11/2009	USA	A/Vermont/18/2009
HA	KC781941	H1N1	08/11/2009	USA	A/Alaska/33/2009
HA	HQ595249	H1N1	08/11/2009	Brazil	A/Sao Paulo/60208/2009
HA	CY069274	H1N1	08/11/2009	Nicaragua	A/Managua/5212.04/2009(H1N1)
HA	CY123489	H1N1	08/11/2009	Singapore	A/Singapore/ON1746/2009
HA	CY123501	H1N1	08/11/2009	Singapore	A/Singapore/ON1757/2009
HA	CY123506	H1N1	08/11/2009	Singapore	A/Singapore/ON1760/2009
HA	CY072702	H1N1	08/11/2009	Nicaragua	A/Managua/5036.01/2009(H1N1)

HA	CY118219	H1N1	08/11/2009	Malaysia	A/Malaysia/2098038/2009
HA	JQ431197	H1N1	08/11/2009	France	A/Reunion/2336-4-M1E/2009
HA	HQ595248	H1N1	08/11/2009	Brazil	A/Sao Paulo/59456/2009
HA	CY115862	H1N1	08/11/2009	Netherlar	A/Netherlands/947b/2009
HA	CY055027	H1N1	08/11/2009	USA	A/California/VRDL68/2009(H1N1)
HA	CY072590	H1N1	08/11/2009	Nicaragua	A/Managua/1356.03/2009(H1N1)
HA	CY073561	H1N1	08/11/2009	Nicaragua	A/Managua/5119.06/2009(H1N1)
HA	CY055260	H1N1	08/11/2009	Malaysia	A/Malaysia/9254/2009(H1N1)
HA	JN631055	H1N1	08/11/2009	China	A/Shanghai/1300T/2009
HA	KC967085	H1N1	08/11/2009	Brazil	A/Rio de Janeiro/9911/2009
HA	CY051988	H1N1	08/11/2009	Norway	A/Norway/2917/2009(H1N1)
HA	CY060518	H1N1	08/11/2009	Canada	A/Ontario/222656/2009
HA	HQ595232	H1N1	08/12/2009	Brazil	A/Sao Paulo/13735/2009
HA	JN256856	H1N1	08/12/2009	Hong Kong	A/Hong Kong/H090-793-V1(0)/2009
HA	CY083232	H1N1	08/12/2009	Guam	A/Guam/WRAIR1510P/2009
HA	CY072582	H1N1	08/12/2009	Nicaragua	A/Managua/4186.02/2009(H1N1)
HA	CY128131	H1N1	08/12/2009	Viet Nam	A/Viet Nam/13032001/2009
HA	CY128139	H1N1	08/12/2009	Viet Nam	A/Viet Nam/13032002/2009
HA	CY112083	H1N1	08/12/2009	Hong Kong	A/Hong Kong/H090-793-V10/2009
HA	CY120477	H1N1	08/12/2009	Hong Kong	A/Hong Kong/H090-793-V10/2009
HA	KY925199	H1N1	08/12/2009	Brazil	A/Alegrete/LACENRS-1453/2009
HA	KY925641	H1N1	08/12/2009	Brazil	A/Porto Alegre/LACENRS-1598/2009
HA	HM189407	H1N1	08/12/2009	Russia	A/Moscow oblast/CRIE-SKV/2009
HA	HM189402	H1N1	08/12/2009	Russia	A/Belgorod/CRIE-KLG/2009
HA	KC781856	H1N1	08/12/2009	USA	A/Oregon/31/2009
HA	CY120715	H1N1	08/12/2009	Brazil	A/Brazil/AVS03/2009
HA	HQ595250	H1N1	08/12/2009	Brazil	A/Sao Paulo/61625/2009
HA	CY055266	H1N1	08/12/2009	Malaysia	A/Malaysia/9541/2009(H1N1)
HA	CY083391	H1N1	08/12/2009	USA	A/San Diego/WRAIR1663P/2009
HA	CY072718	H1N1	08/12/2009	Nicaragua	A/Managua/3359.01/2009(H1N1)
HA	CY055254	H1N1	08/12/2009	Malaysia	A/Malaysia/9131/2009(H1N1)
HA	CY055250	H1N1	08/12/2009	Malaysia	A/Malaysia/9117/2009(H1N1)
HA	CY055908	H1N1	08/12/2009	Australia	A/Australia/73/2009(H1N1)
HA	CY055258	H1N1	08/12/2009	Malaysia	A/Malaysia/9237/2009(H1N1)
HA	CY055256	H1N1	08/12/2009	Malaysia	A/Malaysia/9147/2009(H1N1)
HA	CY055252	H1N1	08/12/2009	Malaysia	A/Malaysia/9118/2009(H1N1)
HA	CY120723	H1N1	08/13/2009	Brazil	A/Brazil/AVS04/2009
HA	HM624086	H1N1	08/13/2009	Australia	A/Perth/260/2009
HA	CY075480	H1N1	08/13/2009	Russia	A/Saint-Petersburg/RII5/2009(H1N1)
HA	KY926117	H1N1	08/13/2009	Brazil	A/Santo Angelo/LACENRS-1537/2009
HA	JQ431206	H1N1	08/13/2009	France	A/Reunion/1722-7-M1E/2009
HA	HM189382	H1N1	08/13/2009	Russia	A/Perm/CRIE-SHEO/2009
HA	CY073577	H1N1	08/13/2009	Nicaragua	A/Managua/921.02/2009(H1N1)
HA	CY055262	H1N1	08/13/2009	Malaysia	A/Malaysia/9343/2009(H1N1)
HA	HQ595252	H1N1	08/13/2009	Brazil	A/Distrito Federal/62652/2009
HA	CY123511	H1N1	08/13/2009	Singapore	A/Singapore/ON1767/2009
HA	CY072990	H1N1	08/13/2009	Nicaragua	A/Managua/527.04/2009(H1N1)
HA	CY055264	H1N1	08/13/2009	Malaysia	A/Malaysia/9451/2009(H1N1)
HA	JX625232	H1N1	08/13/2009	Paraguay	A/Paraguay/98HC1/2009
HA	KT181100	H1N1	08/13/2009	Singapore	A/Singapore/DMS23/2009
HA	HM189425	H1N1	08/13/2009	Russia	A/Nizhni Novgorod/CRIE-KDAn/2009
HA	KY925216	H1N1	08/13/2009	Brazil	A/Venancio Aires/LACENRS-1539/2009
HA	KY925840	H1N1	08/13/2009	Brazil	A/Vacaria/LACENRS-1720/2009
HA	KF612098	H1N1	08/13/2009	Bolivia	A/Santa Cruz/87212/2009

HA	CY060734	H1N1	08/13/2009	Canada	A/Ontario/35273/2009
HA	JQ431207	H1N1	08/13/2009	France	A/Reunion/1722-8-M1E/2009
HA	CY072638	H1N1	08/13/2009	Nicaragua	A/Managua/3642.04/2009(H1N1)
HA	HQ595253	H1N1	08/13/2009	Brazil	A/Mato Grosso do Sul/64045/2009
HA	CY123515	H1N1	08/14/2009	Singapore	A/Singapore/ON1782/2009
HA	CY123513	H1N1	08/14/2009	Singapore	A/Singapore/ON1774/2009
HA	KY925045	H1N1	08/14/2009	Brazil	A/Sao Gabriel/LACENRS-1626/2009
HA	CY051695	H1N1	08/14/2009	USA	A/New York/4777/2009(H1N1)
HA	CY122967	H1N1	08/14/2009	Singapore	A/Singapore/GP2982/2009
HA	CY122980	H1N1	08/14/2009	Singapore	A/Singapore/GP3009/2009
HA	KP637830	H1N1	08/14/2009	Thailand	A/Thailand/IY07083/2009
HA	KC782222	H1N1	08/14/2009	USA	A/California/34/2009
HA	CY075803	H1N1	08/14/2009	Thailand	A/Thailand/CU-H393/2009(H1N1)
HA	KF700929	H1N1	08/14/2009	Brazil	A/Santa Catarina/13898/2009
HA	HM189424	H1N1	08/14/2009	Russia	A/Saratov/CRIE-MDA/2009
HA	KY926399	H1N1	08/14/2009	Brazil	A/Pelotas/LACENRS-1658/2009
HA	KY925687	H1N1	08/14/2009	Brazil	A/Triunfo/LACENRS-1611/2009
HA	CY112091	H1N1	08/14/2009	Hong Kong	A/Hong Kong/H090-793-V20/2009
HA	CY120485	H1N1	08/14/2009	Hong Kong	A/Hong Kong/H090-793-V20/2009
HA	JQ431212	H1N1	08/14/2009	France	A/Reunion/1658-4-M1E/2009
HA	KP317206	H1N1	08/14/2009	India	A/Kerala/002/2009
HA	HQ595251	H1N1	08/14/2009	Brazil	A/Distrito Federal/62218/2009
HA	JN256857	H1N1	08/14/2009	Hong Kong	A/Hong Kong/H090-793-V2(0)/2009
HA	CY055271	H1N1	08/14/2009	Malaysia	A/Malaysia/10226/2009(H1N1)
HA	CY058244	H1N1	08/15/2009	Nicaragua	A/Managua/3275.01/2009(H1N1)
HA	CY058604	H1N1	08/15/2009	Nicaragua	A/Managua/5747.02/2009(H1N1)
HA	CY052847	H1N1	08/15/2009	USA	A/Texas/44152535/2009(H1N1)
HA	KY925327	H1N1	08/15/2009	Brazil	A/Santo Antonio da Patrulha/LACENRS-
HA	CY052871	H1N1	08/15/2009	USA	A/Texas/44151841/2009(H1N1)
HA	CY058236	H1N1	08/15/2009	Nicaragua	A/Managua/473.02/2009(H1N1)
HA	CY072710	H1N1	08/15/2009	Nicaragua	A/Managua/5307.01/2009(H1N1)
HA	GU576514	H1N1	08/16/2009	Italy	A/Ancona/66/2009(H1N1)
HA	HM189370	H1N1	08/16/2009	Russia	A/Orenburg/CRIE-VNV/2009
HA	CY072654	H1N1	08/16/2009	Nicaragua	A/Managua/4570.01/2009(H1N1)
HA	GU057030	H1N1	08/16/2009	China	A/Jiangyin/68/2009(H1N1)
HA	GU057026	H1N1	08/16/2009	China	A/Jiangyin/17/2009(H1N1)
HA	GU057028	H1N1	08/16/2009	China	A/Jiangyin/47/2009(H1N1)
HA	CY122975	H1N1	08/16/2009	Singapore	A/Singapore/GP2987/2009
HA	CY122987	H1N1	08/16/2009	Singapore	A/Singapore/GP3012/2009
HA	KT181101	H1N1	08/16/2009	Singapore	A/Singapore/DMS24/2009
HA	GU057031	H1N1	08/16/2009	China	A/Jiangyin/69/2009(H1N1)
HA	GU057029	H1N1	08/16/2009	China	A/Jiangyin/48/2009(H1N1)
HA	GU057027	H1N1	08/16/2009	China	A/Jiangyin/34/2009(H1N1)
HA	GU057022	H1N1	08/16/2009	China	A/Jiangyin/01/2009(H1N1)
HA	GU057023	H1N1	08/16/2009	China	A/Jiangyin/02/2009(H1N1)
HA	GU057024	H1N1	08/16/2009	China	A/Jiangyin/15/2009(H1N1)
HA	GU057025	H1N1	08/16/2009	China	A/Jiangyin/16/2009(H1N1)
HA	CY055796	H1N1	08/16/2009	Australia	A/Australia/54/2009(H1N1)
HA	CY072670	H1N1	08/16/2009	Nicaragua	A/Managua/1054.02/2009(H1N1)
HA	CY058564	H1N1	08/17/2009	Nicaragua	A/Managua/4218.01/2009(H1N1)
HA	CY123130	H1N1	08/17/2009	Singapore	A/Singapore/GP3278/2009
HA	CY123019	H1N1	08/17/2009	Singapore	A/Singapore/GP3044/2009
HA	KY925011	H1N1	08/17/2009	Brazil	A/Santa Cruz do Sul/LACENRS-2013/20
HA	JQ431205	H1N1	08/17/2009	France	A/Reunion/2245-3-M1E/2009

HA	HM189399	H1N1	08/17/2009	Russia	A/Murmansk/CRIE-PVR/2009
HA	AB530486	H1N1	08/17/2009	Japan	A/Nagasaki/HA-41/2009(H1N1)
HA	CY122995	H1N1	08/17/2009	Singapore	A/Singapore/GP3021/2009
HA	CY123517	H1N1	08/17/2009	Singapore	A/Singapore/ON1786/2009
HA	KF667909	H1N1	08/17/2009	Singapore	A/Singapore/464L/2009
HA	JQ714172	H1N1	08/17/2009	China	A/Tianjinhongqiao/SWL172/2009
HA	CY123519	H1N1	08/17/2009	Singapore	A/Singapore/ON1790/2009
HA	KF667908	H1N1	08/17/2009	Singapore	A/Singapore/463B/2009
HA	AB530485	H1N1	08/17/2009	Japan	A/Nagasaki/HA-40/2009(H1N1)
HA	CY123003	H1N1	08/17/2009	Singapore	A/Singapore/GP3026/2009
HA	KC781569	H1N1	08/17/2009	USA	A/Maryland/27/2009
HA	KC781638	H1N1	08/17/2009	USA	A/Nevada/13/2009
HA	CY072606	H1N1	08/17/2009	Nicaragua	A/Managua/2155.01/2009(H1N1)
HA	CY060510	H1N1	08/17/2009	Canada	A/Ontario/10296/2009
HA	HM624087	H1N1	08/17/2009	Australia	A/Perth/268/2009
HA	JN588779	H1N1	08/17/2009	Cambodia	A/Cambodia/T154/2009
HA	CY058612	H1N1	08/18/2009	Nicaragua	A/Managua/2323.02/2009(H1N1)
HA	CY123027	H1N1	08/18/2009	Singapore	A/Singapore/GP3069/2009
HA	CY123051	H1N1	08/18/2009	Singapore	A/Singapore/GP3093/2009
HA	KF667911	H1N1	08/18/2009	Singapore	A/Singapore/4690/2009
HA	JQ431217	H1N1	08/18/2009	France	A/Reunion/1632-5-M1E/2009
HA	CY051703	H1N1	08/18/2009	USA	A/New York/4780/2009(H1N1)
HA	CY058580	H1N1	08/18/2009	Nicaragua	A/Managua/989.01/2009(H1N1)
HA	CY055019	H1N1	08/18/2009	USA	A/California/VRDL67/2009(H1N1)
HA	KC781514	H1N1	08/18/2009	USA	A/Wisconsin/49/2009
HA	KF667910	H1N1	08/18/2009	Singapore	A/Singapore/468H/2009
HA	CY123035	H1N1	08/18/2009	Singapore	A/Singapore/GP3074/2009
HA	CY123011	H1N1	08/18/2009	Singapore	A/Singapore/GP3040/2009
HA	CY070360	H1N1	08/18/2009	United Ki	A/England/93520038/2009
HA	KC781463	H1N1	08/18/2009	USA	A/Texas/56/2009
HA	HQ595254	H1N1	08/18/2009	Brazil	A/Sao Paulo/64519/2009
HA	CY058252	H1N1	08/18/2009	Nicaragua	A/Managua/3244.01/2009(H1N1)
HA	CY058260	H1N1	08/18/2009	Nicaragua	A/Managua/164.01/2009(H1N1)
HA	CY060444	H1N1	08/18/2009	Brazil	A/Bahia/12606/2009(H1N1)
HA	CY058596	H1N1	08/18/2009	Nicaragua	A/Managua/3435.01/2009(H1N1)
HA	CY063574	H1N1	08/18/2009	USA	A/Boston/154/2009(H1N1)
HA	CY063754	H1N1	08/18/2009	Singapore	A/Singapore/GP3084/2009(H1N1)
HA	CY058572	H1N1	08/18/2009	Nicaragua	A/Managua/4757.02/2009(H1N1)
HA	CY058556	H1N1	08/18/2009	Nicaragua	A/Managua/2093.01/2009(H1N1)
HA	CY051990	H1N1	08/19/2009	Norway	A/Norway/3036/2009(H1N1)
HA	CY123059	H1N1	08/19/2009	Singapore	A/Singapore/GP3104/2009
HA	KY925117	H1N1	08/19/2009	Brazil	A/Tenente Portela/LACENRS-2073/2009
HA	CY123548	H1N1	08/19/2009	Singapore	A/Singapore/ON1813/2009
HA	KR861649	H1N1	08/19/2009	India	A/Bangalore/2960-03/2009
HA	GU576517	H1N1	08/19/2009	Italy	A/Ancona/69/2009(H1N1)
HA	JQ431215	H1N1	08/19/2009	France	A/Reunion/0148-6-M1E/2009
HA	GU576515	H1N1	08/19/2009	Italy	A/Ancona/68/2009(H1N1)
HA	KT181102	H1N1	08/19/2009	Singapore	A/Singapore/DMS25/2009
HA	CY123524	H1N1	08/19/2009	Singapore	A/Singapore/ON1802/2009
HA	CY123540	H1N1	08/19/2009	Singapore	A/Singapore/ON1811/2009
HA	JQ431210	H1N1	08/19/2009	France	A/Reunion/0148-4-M1E/2009
HA	CY072846	H1N1	08/19/2009	Nicaragua	A/Managua/4702.04/2009(H1N1)
HA	CY075809	H1N1	08/19/2009	Thailand	A/Thailand/CU-H427/2009(H1N1)
HA	CY123532	H1N1	08/19/2009	Singapore	A/Singapore/ON1808/2009

HA	CY123043	H1N1	08/19/2009	Singapore	A/Singapore/GP3081/2009
HA	CY103922	H1N1	08/20/2009	Brazil	A/Pernambuco/414/2009
HA	CY051719	H1N1	08/20/2009	USA	A/New York/4789/2009(H1N1)
HA	AB530488	H1N1	08/20/2009	Japan	A/Nagasaki/HA-44/2009(H1N1)
HA	CY055279	H1N1	08/20/2009	Malaysia	A/Malaysia/12617/2009(H1N1)
HA	CY055916	H1N1	08/20/2009	Australia	A/Australia/79/2009(H1N1)
HA	CY072766	H1N1	08/20/2009	Nicaragua	A/Managua/287.01/2009(H1N1)
HA	CY075815	H1N1	08/20/2009	Thailand	A/Thailand/CU-H429/2009(H1N1)
HA	CY123075	H1N1	08/20/2009	Singapore	A/Singapore/GP3178/2009
HA	JQ431213	H1N1	08/20/2009	France	A/Reunion/2923-3-M1E/2009
HA	CY128163	H1N1	08/20/2009	Viet Nam	A/Viet Nam/13032011/2009
HA	CY128147	H1N1	08/20/2009	Viet Nam	A/Viet Nam/13032008/2009
HA	CY128179	H1N1	08/20/2009	Viet Nam	A/Viet Nam/13032016/2009
HA	KF667912	H1N1	08/20/2009	Singapore	A/Singapore/480Y/2009
HA	JX625233	H1N1	08/20/2009	Paraguay	A/Paraguay/125HCl/2009
HA	JX625241	H1N1	08/20/2009	Paraguay	A/Paraguay/1CMI/2009
HA	KP317207	H1N1	08/20/2009	India	A/Bihar/003/2009
HA	KY925593	H1N1	08/20/2009	Brazil	A/Rosario do Sul/LACENRS-2110/2009
HA	KY925861	H1N1	08/20/2009	Brazil	A/Porto Alegre/LACENRS-2121/2009
HA	AB601602	H1N1	08/20/2009	Japan	A/Yamagata/232/2009
HA	CY072870	H1N1	08/20/2009	Nicaragua	A/Managua/2999.03/2009(H1N1)
HA	AB530487	H1N1	08/20/2009	Japan	A/Nagasaki/HA-42/2009(H1N1)
HA	CY128171	H1N1	08/20/2009	Viet Nam	A/Viet Nam/13032015/2009
HA	CY128155	H1N1	08/20/2009	Viet Nam	A/Viet Nam/13032009/2009
HA	HQ595262	H1N1	08/21/2009	Brazil	A/Goias/69866/2009
HA	CY123067	H1N1	08/21/2009	Singapore	A/Singapore/GP3175/2009
HA	CY123083	H1N1	08/21/2009	Singapore	A/Singapore/GP3179/2009
HA	HQ595255	H1N1	08/21/2009	Brazil	A/Goias/66430/2009
HA	CY052162	H1N1	08/21/2009	USA	A/New York/4788/2009(H1N1)
HA	GU576521	H1N1	08/21/2009	Italy	A/Ancona/80/2009(H1N1)
HA	MF629794	H1N1	08/21/2009	India	A/Trivandrum/MCVR261/2009
HA	CY128187	H1N1	08/21/2009	Viet Nam	A/Viet Nam/13032019/2009
HA	CY070361	H1N1	08/21/2009	United Ki	A/England/93540705/2009
HA	JX625237	H1N1	08/21/2009	Paraguay	A/Paraguay/145HCl/2009
HA	KF667915	H1N1	08/21/2009	Singapore	A/Singapore/484S/2009
HA	KF667913	H1N1	08/21/2009	Singapore	A/Singapore/482H/2009
HA	KF667914	H1N1	08/21/2009	Singapore	A/Singapore/483A/2009
HA	JQ431196	H1N1	08/21/2009	France	A/Reunion/2433-3-M1E/2009
HA	GU576519	H1N1	08/21/2009	Italy	A/Ancona/75/2009(H1N1)
HA	CY051743	H1N1	08/21/2009	USA	A/New York/4810/2009(H1N1)
HA	CY051711	H1N1	08/21/2009	USA	A/New York/4787/2009(H1N1)
HA	CY051727	H1N1	08/21/2009	USA	A/New York/4790/2009(H1N1)
HA	CY060526	H1N1	08/21/2009	Canada	A/Ontario/235657/2009
HA	JQ431216	H1N1	08/21/2009	France	A/Reunion/1572-3-M1E/2009
HA	KC781947	H1N1	08/22/2009	USA	A/California/36/2009
HA	KC781529	H1N1	08/22/2009	USA	A/Hawaii/26/2009
HA	CY060446	H1N1	08/22/2009	Brazil	A/Minas Gerais/14602/2009(H1N1)
HA	JQ431218	H1N1	08/22/2009	France	A/Reunion/1709-4-M2E/2009
HA	CY062415	H1N1	08/22/2009	Kuwait	A/Kuwait/N13002/2009(H1N1)
HA	CY072862	H1N1	08/22/2009	Nicaragua	A/Managua/4147.01/2009(H1N1)
HA	JX625235	H1N1	08/22/2009	Paraguay	A/Paraguay/138HCl/2009
HA	CY063788	H1N1	08/22/2009	Singapore	A/Singapore/ON1868/2009
HA	CY072750	H1N1	08/22/2009	Nicaragua	A/Managua/5227.04/2009(H1N1)
HA	CY072798	H1N1	08/22/2009	Nicaragua	A/Managua/432.01/2009(H1N1)

HA	KT181103	H1N1	08/22/2009	Singapore	A/Singapore/DMS26/2009
HA	JX625234	H1N1	08/22/2009	Paraguay	A/Paraguay/136HCl/2009
HA	JQ714173	H1N1	08/22/2009	China	A/Tianjinnankai/SWL177/2009
HA	KY925956	H1N1	08/23/2009	Brazil	A/Ijui/LACENRS-2326/2009
HA	CY123122	H1N1	08/23/2009	Singapore	A/Singapore/GP3212/2009
HA	CY123114	H1N1	08/23/2009	Singapore	A/Singapore/GP3205/2009
HA	JQ714174	H1N1	08/23/2009	China	A/Tianjinnankai/SWL178/2009
HA	JX625236	H1N1	08/23/2009	Paraguay	A/Paraguay/141HCl/2009
HA	CY060534	H1N1	08/24/2009	Canada	A/Ontario/237882/2009
HA	CY123566	H1N1	08/24/2009	Singapore	A/Singapore/ON1858/2009
HA	CY051735	H1N1	08/24/2009	USA	A/New York/4791/2009(H1N1)
HA	CY060447	H1N1	08/24/2009	Brazil	A/Rio de Janeiro/14601/2009(H1N1)
HA	HQ595264	H1N1	08/24/2009	Brazil	A/Goias/69877/2009
HA	HQ595261	H1N1	08/24/2009	Brazil	A/Goias/69862/2009
HA	JQ431221	H1N1	08/24/2009	France	A/Reunion/2378-1-M1E/2009
HA	CY123099	H1N1	08/24/2009	Singapore	A/Singapore/GP3187/2009
HA	CY072814	H1N1	08/24/2009	Nicaragua	A/Managua/3421.02/2009(H1N1)
HA	JQ431208	H1N1	08/24/2009	France	A/Reunion/2133-5-M1E/2009
HA	JQ431209	H1N1	08/24/2009	France	A/Reunion/2133-6-M1E/2009
HA	KT181140	H1N1	08/24/2009	Singapore	A/Singapore/DMS623/2009
HA	CY119458	H1N1	08/24/2009	Malaysia	A/Malaysia/2126061/2009
HA	CY060502	H1N1	08/24/2009	Canada	A/Ontario/10016/2009
HA	CY123556	H1N1	08/24/2009	Singapore	A/Singapore/ON1852/2009
HA	JQ714175	H1N1	08/24/2009	China	A/Tianjinhedong/SWL182/2009
HA	CY119538	H1N1	08/24/2009	Malaysia	A/Malaysia/2126082/2009
HA	CY123561	H1N1	08/24/2009	Singapore	A/Singapore/ON1855/2009
HA	CY062414	H1N1	08/24/2009	Kuwait	A/Kuwait/N12991/2009(H1N1)
HA	HM189359	H1N1	08/25/2009	Russia	A/Yaroslavl/CRIE-KMP/2009
HA	CY062416	H1N1	08/25/2009	Kuwait	A/Kuwait/N13003/2009(H1N1)
HA	CY062413	H1N1	08/25/2009	Kuwait	A/Kuwait/N12988/2009(H1N1)
HA	CY065792	H1N1	08/25/2009	Netherlan	A/Netherlands/1039/2009(H1N1)
HA	HQ595256	H1N1	08/25/2009	Brazil	A/Sao Paulo/67664/2009
HA	HQ595263	H1N1	08/25/2009	Brazil	A/Goias/69870/2009
HA	CY123135	H1N1	08/25/2009	Singapore	A/Singapore/GP3292/2009
HA	CY058588	H1N1	08/25/2009	Nicaragua	A/Managua/65.02/2009(H1N1)
HA	KC781519	H1N1	08/25/2009	USA	A/Utah/51/2009
HA	KT181138	H1N1	08/25/2009	Singapore	A/Singapore/DMS618/2009
HA	KT181137	H1N1	08/25/2009	Singapore	A/Singapore/DMS617/2009
HA	KF667916	H1N1	08/25/2009	Singapore	A/Singapore/491H/2009
HA	KC781349	H1N1	08/25/2009	USA	A/Idaho/10/2009
HA	CY056395	H1N1	08/25/2009	USA	A/New York/4925/2009(H1N1)
HA	CY062511	H1N1	08/25/2009	Mexico	A/Mexico city/CIA4/2009(H1N1)
HA	HM189381	H1N1	08/25/2009	Russia	A/Tumen/CRIE-KIN/2009
HA	KC780679	H1N1	08/25/2009	USA	A/West Virginia/13/2009
HA	KT181142	H1N1	08/25/2009	Singapore	A/Singapore/DMS638/2009
HA	KC781598	H1N1	08/25/2009	USA	A/Idaho/11/2009
HA	HQ595260	H1N1	08/25/2009	Brazil	A/Piaui/69692/2009
HA	KT181145	H1N1	08/26/2009	Singapore	A/Singapore/DMS653/2009
HA	CY123571	H1N1	08/26/2009	Singapore	A/Singapore/ON1876/2009
HA	HQ595259	H1N1	08/26/2009	Brazil	A/Sao Paulo/69529/2009
HA	HQ595257	H1N1	08/26/2009	Brazil	A/Sao Paulo/68417/2009
HA	CY055924	H1N1	08/26/2009	Australia	A/Australia/80/2009(H1N1)
HA	JX625238	H1N1	08/26/2009	Paraguay	A/Paraguay/153HCl/2009
HA	KF667917	H1N1	08/26/2009	Singapore	A/Singapore/495W/2009

HA	JQ431219	H1N1	08/26/2009	France	A/Reunion/2224-3-M3E/2009
HA	CY073017	H1N1	08/26/2009	Hong Kong	A/Hong Kong/WRAIR0113N/2009(H1N1)
HA	KF667918	H1N1	08/26/2009	Singapore	A/Singapore/496T/2009
HA	CY074974	H1N1	08/26/2009	Thailand	A/Thailand/CU-C161/2009(H1N1)
HA	JQ431211	H1N1	08/26/2009	France	A/Reunion/0159-3-M1E/2009
HA	CY052004	H1N1	08/27/2009	Norway	A/Norway/3371/2009(H1N1)
HA	CY123576	H1N1	08/27/2009	Singapore	A/Singapore/ON1888/2009
HA	JN187126	H1N1	08/27/2009	Taiwan	A/Taiwan/6078/2009
HA	HQ712184	H1N1	08/27/2009	Australia	A/Brisbane/2014/2009
HA	HQ595258	H1N1	08/27/2009	Brazil	A/Sao Paulo/69431/2009
HA	HQ595266	H1N1	08/27/2009	Brazil	A/Sao Paulo/70189/2009
HA	CY119466	H1N1	08/27/2009	Malaysia	A/Malaysia/2135565/2009
HA	KF667919	H1N1	08/27/2009	Singapore	A/Singapore/501L/2009
HA	HQ595268	H1N1	08/27/2009	Brazil	A/Sao Paulo/70218/2009
HA	KC781581	H1N1	08/27/2009	USA	A/Kentucky/17/2009
HA	KT181147	H1N1	08/27/2009	Singapore	A/Singapore/DMS665/2009
HA	CY055043	H1N1	08/27/2009	USA	A/California/VRDL70/2009(H1N1)
HA	AB538394	H1N1	08/27/2009	Japan	A/Nagano/RC1/2009(H1N1)
HA	CY072902	H1N1	08/27/2009	Nicaragua	A/Managua/3434.02/2009(H1N1)
HA	CY051993	H1N1	08/27/2009	Norway	A/Norway/3273/2009(H1N1)
HA	CY123592	H1N1	08/27/2009	Singapore	A/Singapore/ON1896/2009
HA	CY123091	H1N1	08/27/2009	Singapore	A/Singapore/GP3183/2009
HA	CY103924	H1N1	08/27/2009	Brazil	A/Pernambuco/431/2009
HA	KC781646	H1N1	08/27/2009	USA	A/South Carolina/21/2009
HA	KC781628	H1N1	08/27/2009	USA	A/Virginia/19/2009
HA	KT181149	H1N1	08/27/2009	Singapore	A/Singapore/DMS676/2009
HA	KF667920	H1N1	08/27/2009	Singapore	A/Singapore/502J/2009
HA	KC781967	H1N1	08/27/2009	USA	A/Mississippi/05/2009
HA	AB538389	H1N1	08/27/2009	Japan	A/Nagano/RC1/2009(H1N1)
HA	CY123584	H1N1	08/27/2009	Singapore	A/Singapore/ON1892/2009
HA	CY073025	H1N1	08/27/2009	Hong Kong	A/Hong Kong/WRAIR0114T/2009(H1N1)
HA	JX625239	H1N1	08/28/2009	Paraguay	A/Paraguay/165HCl/2009
HA	KF667921	H1N1	08/28/2009	Singapore	A/Singapore/506T/2009
HA	CY055051	H1N1	08/28/2009	USA	A/California/VRDL71/2009(H1N1)
HA	CY052767	H1N1	08/28/2009	USA	A/Texas/44282651/2009(H1N1)
HA	CY051992	H1N1	08/28/2009	Norway	A/Norway/3214/2009(H1N1)
HA	CY072942	H1N1	08/28/2009	Nicaragua	A/Managua/1244.01/2009(H1N1)
HA	CY073665	H1N1	08/28/2009	Nicaragua	A/Managua/4209.04/2009(H1N1)
HA	CY103926	H1N1	08/28/2009	Brazil	A/Pernambuco/458/2009
HA	HQ595265	H1N1	08/28/2009	Brazil	A/Sao Paulo/69955/2009
HA	HQ595267	H1N1	08/28/2009	Brazil	A/Sao Paulo/70195/2009
HA	HQ595270	H1N1	08/28/2009	Brazil	A/Sao Paulo/70311/2009
HA	HQ595271	H1N1	08/28/2009	Brazil	A/Sao Paulo/70325/2009
HA	HQ595272	H1N1	08/28/2009	Brazil	A/Sao Paulo/70333/2009
HA	HQ595275	H1N1	08/28/2009	Brazil	A/Sao Paulo/70448/2009
HA	HQ595273	H1N1	08/28/2009	Brazil	A/Sao Paulo/70346/2009
HA	CY123616	H1N1	08/28/2009	Singapore	A/Singapore/ON1911/2009
HA	CY123608	H1N1	08/28/2009	Singapore	A/Singapore/ON1908/2009
HA	CY123600	H1N1	08/28/2009	Singapore	A/Singapore/ON1900/2009
HA	KC780565	H1N1	08/28/2009	USA	A/Minnesota/15/2009
HA	KC781501	H1N1	08/28/2009	USA	A/South Carolina/19/2009
HA	KT181152	H1N1	08/28/2009	Singapore	A/Singapore/DMS698/2009
HA	KC782315	H1N1	08/28/2009	USA	A/Minnesota/16/2009
HA	HQ712183	H1N1	08/28/2009	Australia	A/Brisbane/2015/2009

HA	KT181104	H1N1	08/28/2009	Singapore	A/Singapore/DMS28/2009
HA	CY054600	H1N1	08/28/2009	Thailand	A/Thailand/PMKE0009/2009(H1N1)
HA	HQ595278	H1N1	08/29/2009	Brazil	A/Sao Paulo/71003/2009
HA	HQ595276	H1N1	08/29/2009	Brazil	A/Sao Paulo/70480/2009
HA	HQ595274	H1N1	08/29/2009	Brazil	A/Sao Paulo/70429/2009
HA	KP637846	H1N1	08/29/2009	Thailand	A/Thailand/IY07086/2009
HA	KC781641	H1N1	08/29/2009	USA	A/California/35/2009
HA	CY119474	H1N1	08/29/2009	Malaysia	A/Malaysia/2139971/2009
HA	CY128195	H1N1	08/29/2009	Viet Nam	A/Viet Nam/13032030/2009
HA	CY052226	H1N1	08/30/2009	USA	A/Texas/44302551/2009(H1N1)
HA	CY052218	H1N1	08/30/2009	USA	A/Texas/44301765/2009(H1N1)
HA	JN588782	H1N1	08/30/2009	Cambodia	A/Cambodia/T217/2009
HA	CY052615	H1N1	08/30/2009	USA	A/Texas/44302533/2009(H1N1)
HA	CY062417	H1N1	08/30/2009	Kuwait	A/Kuwait/N13012/2009(H1N1)
HA	CY052623	H1N1	08/30/2009	USA	A/Texas/44302167/2009(H1N1)
HA	HQ595277	H1N1	08/30/2009	Brazil	A/Sao Paulo/70564/2009
HA	CY072790	H1N1	08/30/2009	Nicaragua	A/Managua/1984.02/2009(H1N1)
HA	CY103925	H1N1	08/30/2009	Brazil	A/Pernambuco/447/2009
HA	KC781525	H1N1	08/30/2009	USA	A/South Carolina/20/2009
HA	KC781761	H1N1	08/30/2009	USA	A/South Carolina/32/2009
HA	JQ065141	H1N1	08/31/2009	China	A/Shenzhen/30-SZCDC/2009
HA	JQ065124	H1N1	08/31/2009	China	A/Shenzhen/13/2009
HA	CY123645	H1N1	08/31/2009	Singapore	A/Singapore/ON1927/2009
HA	JQ065143	H1N1	08/31/2009	China	A/Shenzhen/32-SZCDC/2009
HA	JQ065126	H1N1	08/31/2009	China	A/Shenzhen/15/2009
HA	JQ065140	H1N1	08/31/2009	China	A/Shenzhen/29/2009
HA	JQ065136	H1N1	08/31/2009	China	A/Shenzhen/25-SZCDC/2009
HA	KT181155	H1N1	08/31/2009	Singapore	A/Singapore/DMS722/2009
HA	KT181105	H1N1	08/31/2009	Singapore	A/Singapore/DMS29/2009
HA	JQ065129	H1N1	08/31/2009	China	A/Shenzhen/18/2009
HA	JQ065137	H1N1	08/31/2009	China	A/Shenzhen/26/2009
HA	JQ065139	H1N1	08/31/2009	China	A/Shenzhen/28/2009
HA	JQ065127	H1N1	08/31/2009	China	A/Shenzhen/16/2009
HA	JQ065142	H1N1	08/31/2009	China	A/Shenzhen/31/2009
HA	KY926268	H1N1	08/31/2009	Brazil	A/Palmitinho/LACENRS-2595/2009
HA	CY052338	H1N1	08/31/2009	USA	A/Texas/44312415/2009(H1N1)
HA	JQ065125	H1N1	08/31/2009	China	A/Shenzhen/14/2009
HA	JQ065130	H1N1	08/31/2009	China	A/Shenzhen/19/2009
HA	CY052671	H1N1	08/31/2009	USA	A/Texas/44313703/2009(H1N1)
HA	CY051994	H1N1	08/31/2009	Norway	A/Norway/3278/2009(H1N1)
HA	AB540654	H1N1	08/31/2009	Japan	A/Nagasaki/HA-46/2009(H1N1)
HA	GU576522	H1N1	08/31/2009	Italy	A/Ancona/86/2009(H1N1)
HA	CY072974	H1N1	08/31/2009	Nicaragua	A/Managua/3507.01/2009(H1N1)
HA	CY071402	H1N1	08/31/2009	Taiwan	A/Taipei/WR0193T/2009(H1N1)
HA	CY063759	H1N1	08/31/2009	Singapore	A/Singapore/GP3441/2009(H1N1)
HA	CY062418	H1N1	08/31/2009	Kuwait	A/Kuwait/N13013/2009(H1N1)
HA	HQ228057	H1N1	08/31/2009	Finland	A/Finland/607/2009
HA	CY123640	H1N1	08/31/2009	Singapore	A/Singapore/ON1924/2009
HA	JQ065132	H1N1	08/31/2009	China	A/Shenzhen/21/2009
HA	JQ065134	H1N1	08/31/2009	China	A/Shenzhen/23/2009
HA	JQ065133	H1N1	08/31/2009	China	A/Shenzhen/22-SZCDC/2009
HA	JQ065131	H1N1	08/31/2009	China	A/Shenzhen/20/2009
HA	CY072926	H1N1	08/31/2009	Nicaragua	A/Managua/4063.01/2009(H1N1)
HA	JQ065135	H1N1	08/31/2009	China	A/Shenzhen/24/2009

HA	CY073673	H1N1	08/31/2009	Nicaragua	A/Managua/5248.03/2009(H1N1)
HA	CY123624	H1N1	08/31/2009	Singapore	A/Singapore/ON1914/2009
HA	JQ065128	H1N1	08/31/2009	China	A/Shenzhen/17-SZCDC/2009
HA	CY123632	H1N1	08/31/2009	Singapore	A/Singapore/ON1921/2009
HA	JQ065138	H1N1	08/31/2009	China	A/Shenzhen/27/2009
HA	JQ065123	H1N1	08/31/2009	China	A/Shenzhen/12/2009
HA	HM189479	H1N1	09/2009	South Ko	A/Korea/CJ01/2009(H1N1)
HA	HM189483	H1N1	09/2009	South Ko	A/Korea/CJ19/2009(H1N1)
HA	AB530489	H1N1	09/2009	Japan	A/Nagasaki/NU-1/2009(H1N1)
HA	GU968910	H1N1	09/2009	China	A/Beijing/SE1099/2009(H1N1)
HA	CY062391	H1N1	09/2009	Afghanist	A/Afghanistan/N10734/2009(H1N1)
HA	CY062429	H1N1	09/2009	Kuwait	A/Kuwait/N13085/2009(H1N1)
HA	GU968912	H1N1	09/2009	China	A/Beijing/SE1510/2009(H1N1)
HA	GU968911	H1N1	09/2009	China	A/Beijing/SE1113/2009(H1N1)
HA	GU968914	H1N1	09/2009	China	A/Beijing/SE1609/2009(H1N1)
HA	AB745122	H1N1	09/2009	Japan	A/Tottori/ST227/2009
HA	JN381230	H1N1	09/2009	Taiwan	A/Taiwan/98928/2009
HA	CY062389	H1N1	09/2009	Afghanist	A/Afghanistan/N10725/2009(H1N1)
HA	JN381229	H1N1	09/2009	Taiwan	A/Taiwan/98911/2009
HA	JN381225	H1N1	09/2009	Taiwan	A/Taiwan/98584/2009
HA	CY062394	H1N1	09/2009	Afghanist	A/Afghanistan/N10757/2009(H1N1)
HA	CY062397	H1N1	09/2009	Afghanist	A/Afghanistan/N10767/2009(H1N1)
HA	GU369656	H1N1	09/2009	Turkey	A/Ankara/12/2009
HA	GU369654	H1N1	09/2009	Turkey	A/Ankara/10/2009
HA	CY189537	H1N1	09/2009	Norway	A/Norway/3568/2009
HA	JQ364990	H1N1	09/2009	China	A/Hangzhou/4/2009
HA	CY062396	H1N1	09/2009	Afghanist	A/Afghanistan/N10765/2009(H1N1)
HA	HM189481	H1N1	09/2009	South Ko	A/Korea/CJ09/2009(H1N1)
HA	JN381228	H1N1	09/2009	Taiwan	A/Taiwan/98812/2009
HA	JN381227	H1N1	09/2009	Taiwan	A/Taiwan/98706/2009
HA	JN381226	H1N1	09/2009	Taiwan	A/Taiwan/98677/2009
HA	JN381224	H1N1	09/2009	Taiwan	A/Taiwan/98229/2009
HA	JN381231	H1N1	09/2009	Taiwan	A/Taiwan/99006/2009
HA	JN381232	H1N1	09/2009	Taiwan	A/Taiwan/99011/2009
HA	CY062392	H1N1	09/2009	Afghanist	A/Afghanistan/N10736/2009(H1N1)
HA	CY062395	H1N1	09/2009	Afghanist	A/Afghanistan/N10760/2009(H1N1)
HA	CY062390	H1N1	09/2009	Afghanist	A/Afghanistan/N10731/2009(H1N1)
HA	CY062403	H1N1	09/2009	Afghanist	A/Afghanistan/N10974/2009(H1N1)
HA	GU369660	H1N1	09/2009	Turkey	A/Ankara/16/2009
HA	HM189480	H1N1	09/2009	South Ko	A/Korea/CJ04/2009(H1N1)
HA	GU968913	H1N1	09/2009	China	A/Beijing/SE1547/2009(H1N1)
HA	CY062393	H1N1	09/2009	Afghanist	A/Afghanistan/N10749/2009(H1N1)
HA	CY062404	H1N1	09/2009	Djibouti	A/Djibouti/N11092/2009(H1N1)
HA	HM204576	H1N1	09/2009	India	A/Nsk/NIV10348/2009(H1N1)
HA	HM189482	H1N1	09/2009	South Ko	A/Korea/CJ15/2009(H1N1)
HA	HM189484	H1N1	09/2009	South Ko	A/Korea/CJ24/2009(H1N1)
HA	CY065123	H1N1	09/01/2009	Netherla	A/Netherlands/1064b/2009(H1N1)
HA	KP019826	H1N1	09/01/2009	China	A/FuZhou/SWL11202/2009
HA	CY119330	H1N1	09/01/2009	Malaysia	A/Malaysia/2142295/2009(H1N1)
HA	JX625229	H1N1	09/01/2009	Paraguay	A/Paraguay/20HBO/2009
HA	CY070362	H1N1	09/01/2009	United Ki	A/England/93640020/2009
HA	CY052679	H1N1	09/01/2009	USA	A/Texas/45010998/2009(H1N1)
HA	HQ228058	H1N1	09/01/2009	Finland	A/Finland/608/2009
HA	CY055479	H1N1	09/01/2009	USA	A/California/VRDL72/2009(H1N1)

HA	HQ595279	H1N1	09/01/2009	Brazil	A/Sao Paulo/23316/2009
HA	CY052003	H1N1	09/01/2009	Norway	A/Norway/3370/2009(H1N1)
HA	CY052019	H1N1	09/01/2009	Norway	A/Norway/3206-3/2009(H1N1)
HA	JQ714176	H1N1	09/01/2009	China	A/Tianjinnankai/SWL198/2009
HA	KC782038	H1N1	09/01/2009	USA	A/Texas/58/2009
HA	KF860844	H1N1	09/01/2009	Spain	A/Castilla La Mancha/4430/2009
HA	CY128203	H1N1	09/01/2009	Viet Nam	A/Viet Nam/13032035/2009
HA	CY128459	H1N1	09/01/2009	Viet Nam	A/Viet Nam/001-1889/2009
HA	CY128064	H1N1	09/01/2009	Viet Nam	A/Viet Nam/001-1890/2009
HA	CY119338	H1N1	09/01/2009	Malaysia	A/Malaysia/2142299/2009(H1N1)
HA	KC781445	H1N1	09/01/2009	USA	A/New York/99/2009
HA	CY063761	H1N1	09/01/2009	Singapore	A/Singapore/GP3491/2009(H1N1)
HA	CY123657	H1N1	09/01/2009	Singapore	A/Singapore/ON1934/2009
HA	KC782308	H1N1	09/01/2009	USA	A/Wisconsin/53/2009
HA	CY119346	H1N1	09/01/2009	Malaysia	A/Malaysia/2143696/2009(H1N1)
HA	CY073681	H1N1	09/01/2009	Nicaragua	A/Managua/4736.02/2009(H1N1)
HA	CY123662	H1N1	09/01/2009	Singapore	A/Singapore/ON1937/2009
HA	HQ595280	H1N1	09/01/2009	Brazil	A/Sao Paulo/71897/2009
HA	CY055486	H1N1	09/01/2009	USA	A/California/VRDL73/2009(H1N1)
HA	CY070366	H1N1	09/01/2009	United Ki	A/England/93760003/2009
HA	CY055932	H1N1	09/02/2009	Australia	A/Australia/81/2009(H1N1)
HA	CY055940	H1N1	09/02/2009	Australia	A/Australia/82/2009(H1N1)
HA	CY052855	H1N1	09/02/2009	USA	A/Texas/45023717/2009(H1N1)
HA	CY052879	H1N1	09/02/2009	USA	A/Texas/45024243/2009(H1N1)
HA	CY051751	H1N1	09/02/2009	USA	A/New York/4820/2009(H1N1)
HA	CY052775	H1N1	09/02/2009	USA	A/Texas/45021632/2009(H1N1)
HA	KC781304	H1N1	09/02/2009	USA	A/Kentucky/19/2009
HA	KC781801	H1N1	09/02/2009	USA	A/South Carolina/37/2009
HA	KC782085	H1N1	09/02/2009	USA	A/Kentucky/18/2009
HA	KC781972	H1N1	09/02/2009	USA	A/South Carolina/35/2009
HA	KC781758	H1N1	09/02/2009	USA	A/South Carolina/36/2009
HA	JQ431204	H1N1	09/02/2009	France	A/Reunion/2154-4-M4E/2009
HA	JQ431223	H1N1	09/02/2009	France	A/Reunion/2956-2-M1E/2009
HA	CY123137	H1N1	09/02/2009	Singapore	A/Singapore/GP3485/2009
HA	CY074990	H1N1	09/02/2009	Thailand	A/Thailand/CU-H567/2009
HA	CY075924	H1N1	09/02/2009	India	A/Nsk/NIV9968/2009
HA	HQ595281	H1N1	09/02/2009	Brazil	A/Goias/72059/2009
HA	CY070371	H1N1	09/02/2009	United Ki	A/England/93820003/2009
HA	CY070363	H1N1	09/02/2009	United Ki	A/England/93720014/2009
HA	CY123149	H1N1	09/02/2009	Singapore	A/Singapore/GP3515/2009
HA	CY123667	H1N1	09/02/2009	Singapore	A/Singapore/ON1943/2009
HA	HQ228066	H1N1	09/02/2009	Finland	A/Finland/617/2009
HA	CY072742	H1N1	09/02/2009	Nicaragua	A/Managua/2958.01/2009(H1N1)
HA	CY069098	H1N1	09/02/2009	Guam	A/Guam/NHRC0027/2009(H1N1)
HA	KY925715	H1N1	09/02/2009	Brazil	A/Osorio/LACENRS-2638/2009
HA	CY123145	H1N1	09/02/2009	Singapore	A/Singapore/GP3495/2009
HA	CY050019	H1N1	09/02/2009	Mexico	A/Mexico City/WR1306N/2009(H1N1)
HA	JX625240	H1N1	09/02/2009	Paraguay	A/Paraguay/171HCl/2009
HA	KC781622	H1N1	09/02/2009	USA	A/Idaho/09/2009
HA	JQ431220	H1N1	09/02/2009	France	A/Reunion/2692-2-M2E/2009
HA	KT181156	H1N1	09/02/2009	Singapore	A/Singapore/DMS737/2009
HA	CY070364	H1N1	09/02/2009	United Ki	A/England/93740004/2009
HA	CY050140	H1N1	09/02/2009	China	A/Qingdao/1008/2009(H1N1)
HA	KT181159	H1N1	09/02/2009	Singapore	A/Singapore/DMS752/2009

HA	JQ431214	H1N1	09/03/2009	France	A/Reunion/2969-5-M1E/2009
HA	CY052935	H1N1	09/03/2009	USA	A/Texas/45032753/2009(H1N1)
HA	CY051759	H1N1	09/03/2009	USA	A/New York/4823/2009(H1N1)
HA	CY074998	H1N1	09/03/2009	Thailand	A/Thailand/CU-H572/2009
HA	CY050003	H1N1	09/03/2009	Mexico	A/Mexico City/WR1297N/2009(H1N1)
HA	CY051775	H1N1	09/03/2009	USA	A/New York/4841/2009(H1N1)
HA	CY128211	H1N1	09/03/2009	Viet Nam	A/Viet Nam/13032036/2009
HA	HM006714	H1N1	09/03/2009	China	A/Xian/001/2009(H1N1)
HA	CY052591	H1N1	09/03/2009	USA	A/Texas/45033774/2009(H1N1)
HA	CY052903	H1N1	09/03/2009	USA	A/Texas/45032708/2009(H1N1)
HA	KC781653	H1N1	09/03/2009	USA	A/New Hampshire/18/2009
HA	KC781469	H1N1	09/03/2009	USA	A/New Mexico/05/2009
HA	CY052607	H1N1	09/03/2009	USA	A/Texas/45034157/2009(H1N1)
HA	CY123147	H1N1	09/03/2009	Singapore	A/Singapore/GP3502/2009
HA	CY087192	H1N1	09/03/2009	USA	A/Texas/45034240/2009(H1N1)
HA	CY103927	H1N1	09/03/2009	Brazil	A/Pernambuco/513/2009
HA	CY070365	H1N1	09/03/2009	United Ki	A/England/93740191/2009
HA	CY123683	H1N1	09/03/2009	Singapore	A/Singapore/ON1964/2009
HA	CY123675	H1N1	09/03/2009	Singapore	A/Singapore/ON1955/2009
HA	KC781949	H1N1	09/03/2009	USA	A/Maryland/28/2009
HA	KT181106	H1N1	09/03/2009	Singapore	A/Singapore/DMS30/2009
HA	CY052599	H1N1	09/03/2009	USA	A/Texas/45033567/2009(H1N1)
HA	CY051767	H1N1	09/03/2009	USA	A/New York/4824/2009(H1N1)
HA	CY050273	H1N1	09/03/2009	China	A/Qingdao/477/2009(H1N1)
HA	CY090081	H1N1	09/03/2009	Nicaragua	A/Managua/4032.03/2009
HA	AB558293	H1N1	09/03/2009	Japan	A/Sendai/TU373/2009
HA	JQ431222	H1N1	09/04/2009	France	A/Reunion/2956-1-M2E/2009
HA	JQ431198	H1N1	09/04/2009	France	A/Reunion/0215-4-M1E/2009
HA	CY050098	H1N1	09/04/2009	China	A/Qingdao/1020/2009(H1N1)
HA	HM072415	H1N1	09/04/2009	Taiwan	A/Taiwan/6662/2009
HA	AB558295	H1N1	09/04/2009	Japan	A/Sendai/TU377/2009
HA	CY063793	H1N1	09/04/2009	Singapore	A/Singapore/ON2018/2009
HA	CY068986	H1N1	09/04/2009	Guam	A/Guam/NHRC0013/2009(H1N1)
HA	CY062422	H1N1	09/04/2009	Kuwait	A/Kuwait/N13041/2009(H1N1)
HA	CY123688	H1N1	09/04/2009	Singapore	A/Singapore/ON1972/2009
HA	HM072417	H1N1	09/04/2009	Taiwan	A/Taiwan/7717/2009
HA	KC781588	H1N1	09/04/2009	USA	A/Alaska/35/2009
HA	AB558294	H1N1	09/04/2009	Japan	A/Sendai/TU376/2009
HA	KT181164	H1N1	09/04/2009	Singapore	A/Singapore/DMS804/2009
HA	CY070373	H1N1	09/04/2009	United Ki	A/England/93820011/2009
HA	CY128219	H1N1	09/04/2009	Viet Nam	A/Viet Nam/13032037/2009
HA	KC781594	H1N1	09/04/2009	USA	A/Missouri/08/2009
HA	CY052783	H1N1	09/04/2009	USA	A/Texas/45043852/2009(H1N1)
HA	CY062421	H1N1	09/04/2009	Kuwait	A/Kuwait/N13039/2009(H1N1)
HA	CY052863	H1N1	09/04/2009	USA	A/Texas/45042604/2009(H1N1)
HA	CY070991	H1N1	09/04/2009	Guam	A/Guam/NHRC0029/2009(H1N1)
HA	CY069010	H1N1	09/04/2009	Guam	A/Guam/NHRC0016/2009(H1N1)
HA	HM006715	H1N1	09/04/2009	China	A/Xian/002/2009(H1N1)
HA	CY050100	H1N1	09/04/2009	China	A/Qingdao/1006/2009(H1N1)
HA	CY062419	H1N1	09/04/2009	Kuwait	A/Kuwait/N13020/2009(H1N1)
HA	HM072416	H1N1	09/04/2009	Taiwan	A/Taiwan/6663/2009
HA	CY065880	H1N1	09/04/2009	Netherlar	A/Netherlands/1093/2009(H1N1)
HA	HQ228081	H1N1	09/04/2009	Finland	A/Finland/632/2009
HA	KT181168	H1N1	09/05/2009	Singapore	A/Singapore/DMS835/2009

HA	CY052919	H1N1	09/05/2009	USA	A/Texas/45052569/2009(H1N1)
HA	CY075892	H1N1	09/05/2009	India	A/Ngp/NIV11203/2009
HA	AB558296	H1N1	09/05/2009	Japan	A/Sendai/TU378/2009
HA	JQ431200	H1N1	09/05/2009	France	A/Reunion/0116-3-M1E/2009
HA	JQ431203	H1N1	09/05/2009	France	A/Reunion/2154-2-M5E/2009
HA	KC781582	H1N1	09/05/2009	USA	A/Alaska/36/2009
HA	HM006716	H1N1	09/05/2009	China	A/Xian/004/2009(H1N1)
HA	CY071834	H1N1	09/05/2009	USA	A/South Carolina/WRSP520/2009(H1N1)
HA	JN588776	H1N1	09/05/2009	Cambodia	A/Cambodia/T068/2009
HA	CY072806	H1N1	09/05/2009	Nicaragua	A/Managua/572.02/2009(H1N1)
HA	CY052887	H1N1	09/06/2009	USA	A/Texas/45062346/2009(H1N1)
HA	CY051783	H1N1	09/06/2009	USA	A/New York/4844/2009(H1N1)
HA	HM006717	H1N1	09/06/2009	China	A/Xian/006/2009(H1N1)
HA	CY052631	H1N1	09/06/2009	USA	A/Texas/45061670/2009(H1N1)
HA	KC781952	H1N1	09/06/2009	USA	A/Delaware/12/2009
HA	CY070374	H1N1	09/06/2009	United Ki	A/England/93820019/2009
HA	KT181107	H1N1	09/06/2009	Singapore	A/Singapore/DMS31/2009
HA	CY051895	H1N1	09/06/2009	USA	A/Texas/45062633/2009(H1N1)
HA	CY123161	H1N1	09/06/2009	Singapore	A/Singapore/GP3583/2009
HA	KC781822	H1N1	09/06/2009	USA	A/District Of Columbia/09/2009
HA	CY052639	H1N1	09/06/2009	USA	A/Texas/45062584/2009(H1N1)
HA	GU108486	H1N1	09/06/2009	China	A/Zhejiang-Yiwu/11/2009(H1N1)
HA	CY052951	H1N1	09/06/2009	USA	A/Texas/45061755/2009(H1N1)
HA	CY072157	H1N1	09/06/2009	USA	A/Texas/AF2651/2009(H1N1)
HA	CY062420	H1N1	09/06/2009	Kuwait	A/Kuwait/N13028/2009(H1N1)
HA	JQ431201	H1N1	09/07/2009	France	A/Reunion/0116-3-M2E/2009
HA	CY123163	H1N1	09/07/2009	Singapore	A/Singapore/GP3594/2009
HA	CY057878	H1N1	09/07/2009	USA	A/Wisconsin/629-D01987/2009(H1N1)
HA	AB558298	H1N1	09/07/2009	Japan	A/Sendai/TU384/2009
HA	CY052791	H1N1	09/07/2009	USA	A/Texas/45072273/2009(H1N1)
HA	CY052815	H1N1	09/07/2009	USA	A/Texas/45072656/2009(H1N1)
HA	CY052823	H1N1	09/07/2009	USA	A/Texas/45072128/2009(H1N1)
HA	CY119482	H1N1	09/07/2009	Malaysia	A/Malaysia/2156796/2009
HA	CY123159	H1N1	09/07/2009	Singapore	A/Singapore/GP3560/2009
HA	JQ431199	H1N1	09/07/2009	France	A/Reunion/0215-4-M2E/2009
HA	CY060550	H1N1	09/07/2009	Canada	A/Ontario/25913/2009
HA	KC782191	H1N1	09/07/2009	USA	A/Kentucky/20/2009
HA	CY052807	H1N1	09/07/2009	USA	A/Texas/45071344/2009(H1N1)
HA	CY052687	H1N1	09/07/2009	USA	A/Texas/45071524/2009(H1N1)
HA	CY070378	H1N1	09/07/2009	United Ki	A/Scotland/93860049/2009
HA	CY070379	H1N1	09/07/2009	United Ki	A/Scotland/93860051/2009
HA	GU112090	H1N1	09/07/2009	China	A/Zhejiang/DTID-ZJU02/2009(H1N1)
HA	CY062400	H1N1	09/07/2009	Afghanist	A/Afghanistan/N10786/2009(H1N1)
HA	CY052007	H1N1	09/07/2009	Norway	A/Norway/3478/2009(H1N1)
HA	GU189649	H1N1	09/07/2009	China	A/Zhejiang/DTID-ZJU03/2009(H1N1)
HA	HM006718	H1N1	09/07/2009	China	A/Xian/009/2009(H1N1)
HA	AB558297	H1N1	09/07/2009	Japan	A/Sendai/TU380/2009
HA	CY070380	H1N1	09/08/2009	United Ki	A/Scotland/93860052/2009
HA	CY123758	H1N1	09/08/2009	Singapore	A/Singapore/ON2111/2009
HA	CY123165	H1N1	09/08/2009	Singapore	A/Singapore/GP3621/2009
HA	CY056995	H1N1	09/08/2009	USA	A/Wisconsin/629-D00734/2009(H1N1)
HA	CY123722	H1N1	09/08/2009	Singapore	A/Singapore/ON2020/2009
HA	CY123724	H1N1	09/08/2009	Singapore	A/Singapore/ON2024/2009
HA	JQ065144	H1N1	09/08/2009	China	A/Shenzhen/33/2009

HA	JQ065147	H1N1	09/08/2009	China	A/Shenzhen/36/2009
HA	CY051998	H1N1	09/08/2009	Norway	A/Norway/3364-2/2009(H1N1)
HA	JQ065146	H1N1	09/08/2009	China	A/Shenzhen/35/2009
HA	JQ065154	H1N1	09/08/2009	China	A/Shenzhen/43/2009
HA	JQ065149	H1N1	09/08/2009	China	A/Shenzhen/38/2009
HA	JQ065150	H1N1	09/08/2009	China	A/Shenzhen/39/2009
HA	CY062426	H1N1	09/08/2009	Kuwait	A/Kuwait/N13066/2009(H1N1)
HA	KR861650	H1N1	09/08/2009	India	A/Bangalore/6053-14/2009
HA	KT181169	H1N1	09/08/2009	Singapore	A/Singapore/DMS847/2009
HA	CY052695	H1N1	09/08/2009	USA	A/Texas/45083819/2009(H1N1)
HA	JQ065151	H1N1	09/08/2009	China	A/Shenzhen/40-SZCDC/2009
HA	HQ228059	H1N1	09/08/2009	Finland	A/Finland/609/2009
HA	JQ065153	H1N1	09/08/2009	China	A/Shenzhen/42-SZCDC/2009
HA	HQ228060	H1N1	09/08/2009	Finland	A/Finland/610/2009
HA	KC781477	H1N1	09/08/2009	USA	A/Nebraska/18/2009
HA	CY070384	H1N1	09/08/2009	United Ki	A/Scotland/93880057/2009
HA	CY123726	H1N1	09/08/2009	Singapore	A/Singapore/ON2028/2009
HA	CY123701	H1N1	09/08/2009	Singapore	A/Singapore/ON2009/2009
HA	JQ065145	H1N1	09/08/2009	China	A/Shenzhen/34/2009
HA	JQ065152	H1N1	09/08/2009	China	A/Shenzhen/41/2009
HA	JQ065148	H1N1	09/08/2009	China	A/Shenzhen/37-SZCDC/2009
HA	JQ065155	H1N1	09/08/2009	China	A/Shenzhen/44-SZCDC/2009
HA	KC780076	H1N1	09/08/2009	USA	A/Oregon/32/2009
HA	KC782105	H1N1	09/08/2009	USA	A/Iowa/08/2009
HA	KC781600	H1N1	09/08/2009	USA	A/Wyoming/14/2009
HA	CY128227	H1N1	09/08/2009	Viet Nam	A/Viet Nam/13032040/2009
HA	KT181171	H1N1	09/08/2009	Singapore	A/Singapore/DMS869/2009
HA	CY057003	H1N1	09/08/2009	USA	A/Wisconsin/629-D02367/2009(H1N1)
HA	HM006719	H1N1	09/08/2009	China	A/Xian/010/2009(H1N1)
HA	AB558299	H1N1	09/08/2009	Japan	A/Sendai/TU385/2009
HA	CY073689	H1N1	09/08/2009	Nicaragua	A/Managua/5953.01/2009(H1N1)
HA	CY052170	H1N1	09/09/2009	USA	A/New York/4855/2009(H1N1)
HA	CY052735	H1N1	09/09/2009	USA	A/Texas/45091417/2009(H1N1)
HA	CY052005	H1N1	09/09/2009	Norway	A/Norway/3433/2009(H1N1)
HA	CY052006	H1N1	09/09/2009	Norway	A/Norway/3440/2009(H1N1)
HA	CY050043	H1N1	09/09/2009	Mexico	A/Mexico City/WR1310N/2009(H1N1)
HA	CY069106	H1N1	09/09/2009	Guam	A/Guam/NHRC0028/2009(H1N1)
HA	CY069082	H1N1	09/09/2009	Guam	A/Guam/NHRC0025/2009(H1N1)
HA	CY057011	H1N1	09/09/2009	USA	A/Wisconsin/629-D02041/2009(H1N1)
HA	HM006720	H1N1	09/09/2009	China	A/Xian/017/2009(H1N1)
HA	CY050112	H1N1	09/09/2009	China	A/Qingdao/1170/2009(H1N1)
HA	CY083399	H1N1	09/09/2009	USA	A/Great Lakes/WRAIR1664P/2009
HA	CY083375	H1N1	09/09/2009	USA	A/Pendleton/WRAIR1661P/2009(H1N1)
HA	AB558300	H1N1	09/09/2009	Japan	A/Sendai/TU386/2009
HA	JN187127	H1N1	09/09/2009	Taiwan	A/Taiwan/937/2009
HA	CY070369	H1N1	09/09/2009	United Ki	A/England/93780689/2009
HA	KC780192	H1N1	09/09/2009	USA	A/Texas/70/2009
HA	CY123728	H1N1	09/09/2009	Singapore	A/Singapore/ON2035/2009
HA	CY051799	H1N1	09/09/2009	USA	A/New York/4866/2009(H1N1)
HA	CY052655	H1N1	09/09/2009	USA	A/Texas/45093846/2009(H1N1)
HA	KC781586	H1N1	09/09/2009	USA	A/Maine/08/2009
HA	CY070368	H1N1	09/09/2009	United Ki	A/England/93780041/2009
HA	CY062423	H1N1	09/09/2009	Kuwait	A/Kuwait/N13053/2009(H1N1)
HA	HM568136	H1N1	09/09/2009	United Ki	A/England/H093780041/2009

HA	KR861651	H1N1	09/09/2009	India	A/Bangalore/6206-12/2009
HA	KT181172	H1N1	09/09/2009	Singapore	A/Singapore/DMS870/2009
HA	CY052719	H1N1	09/09/2009	USA	A/Texas/45091402/2009(H1N1)
HA	CY052663	H1N1	09/09/2009	USA	A/Texas/45093670/2009(H1N1)
HA	CY052703	H1N1	09/09/2009	USA	A/Texas/45093214/2009(H1N1)
HA	CY052727	H1N1	09/09/2009	USA	A/Texas/45091405/2009(H1N1)
HA	KC781691	H1N1	09/09/2009	USA	A/Ohio/15/2009
HA	KC781571	H1N1	09/09/2009	USA	A/Kansas/18/2009
HA	CY057019	H1N1	09/09/2009	USA	A/Wisconsin/629-D02133/2009(H1N1)
HA	AB558301	H1N1	09/09/2009	Japan	A/Sendai/TU387/2009
HA	CY070381	H1N1	09/09/2009	United Ki	A/England/93880044/2009
HA	CY051791	H1N1	09/09/2009	USA	A/New York/4856/2009(H1N1)
HA	CY123167	H1N1	09/09/2009	Singapore	A/Singapore/GP3624/2009
HA	HQ228067	H1N1	09/09/2009	Finland	A/Finland/618/2009
HA	CY123730	H1N1	09/09/2009	Singapore	A/Singapore/ON2040/2009
HA	CY051807	H1N1	09/10/2009	USA	A/New York/4870/2009(H1N1)
HA	AB558313	H1N1	09/10/2009	Japan	A/Sendai/TU407/2009
HA	AB558309	H1N1	09/10/2009	Japan	A/Sendai/TU399/2009
HA	CY063795	H1N1	09/10/2009	Singapore	A/Singapore/ON2063/2009
HA	HM568144	H1N1	09/10/2009	United Ki	A/England/H093780034/2009
HA	HM189389	H1N1	09/10/2009	Russia	A/Moscow oblast/CRIE-IVD/2009
HA	CY052895	H1N1	09/10/2009	USA	A/Texas/45102952/2009(H1N1)
HA	CY052759	H1N1	09/10/2009	USA	A/Texas/45101422/2009(H1N1)
HA	GU576524	H1N1	09/10/2009	Italy	A/Ancona/97/2009(H1N1)
HA	CY052743	H1N1	09/10/2009	USA	A/Texas/45101424/2009(H1N1)
HA	KC780809	H1N1	09/10/2009	USA	A/Montana/17/2009
HA	KC781813	H1N1	09/10/2009	USA	A/Nevada/15/2009
HA	CY070370	H1N1	09/10/2009	United Ki	A/England/93800021/2009
HA	CY065912	H1N1	09/10/2009	Netherlar	A/Netherlands/1084/2009(H1N1)
HA	KT181176	H1N1	09/10/2009	Singapore	A/Singapore/DMS905/2009
HA	KC781559	H1N1	09/10/2009	USA	A/Washington/57/2009
HA	CY052511	H1N1	09/10/2009	USA	A/Texas/45103737/2009(H1N1)
HA	CY052647	H1N1	09/10/2009	USA	A/Texas/45103259/2009(H1N1)
HA	CY052751	H1N1	09/10/2009	USA	A/Texas/45103759/2009(H1N1)
HA	CY052519	H1N1	09/10/2009	USA	A/Texas/45104048/2009(H1N1)
HA	CY065904	H1N1	09/10/2009	Netherlar	A/Netherlands/1083/2009(H1N1)
HA	AB558308	H1N1	09/10/2009	Japan	A/Sendai/TU398/2009
HA	CY123169	H1N1	09/10/2009	Singapore	A/Singapore/GP3668/2009
HA	JN588786	H1N1	09/10/2009	Cambodia	A/Cambodia/T320/2009
HA	JN187128	H1N1	09/10/2009	Taiwan	A/Taiwan/940/2009
HA	AB558314	H1N1	09/10/2009	Japan	A/Sendai/TU408/2009
HA	CY123732	H1N1	09/10/2009	Singapore	A/Singapore/ON2054/2009
HA	CY119514	H1N1	09/10/2009	Malaysia	A/Malaysia/2097960/2009
HA	CY123734	H1N1	09/10/2009	Singapore	A/Singapore/ON2058/2009
HA	KC781750	H1N1	09/10/2009	USA	A/North Carolina/37/2009
HA	CY052527	H1N1	09/10/2009	USA	A/Texas/45103998/2009(H1N1)
HA	CY057042	H1N1	09/10/2009	United Ki	A/England/93960032/2009
HA	CY052503	H1N1	09/10/2009	USA	A/Texas/45104026/2009(H1N1)
HA	CY050051	H1N1	09/10/2009	Mexico	A/Mexico City/WR1311T/2009(H1N1)
HA	CY050059	H1N1	09/10/2009	Mexico	A/Mexico City/WR1312N/2009(H1N1)
HA	AB535742	H1N1	09/10/2009	Japan	A/Nagasaki/HA-48/2009(H1N1)
HA	HQ228082	H1N1	09/10/2009	Finland	A/Finland/633/2009
HA	CY072918	H1N1	09/10/2009	Nicaragua	A/Managua/4972.02/2009(H1N1)
HA	CY057027	H1N1	09/10/2009	USA	A/Wisconsin/629-D00357/2009(H1N1)

HA	CY070367	H1N1	09/10/2009	United Ki	A/England/93780034/2009
HA	KY924993	H1N1	09/10/2009	Brazil	A/Campina das Missoes/LACENRS-2882/2
HA	CY123740	H1N1	09/11/2009	Singapore	A/Singapore/ON2075/2009
HA	CY123736	H1N1	09/11/2009	Singapore	A/Singapore/ON2064/2009
HA	CY123738	H1N1	09/11/2009	Singapore	A/Singapore/ON2067/2009
HA	CY057358	H1N1	09/11/2009	USA	A/Wisconsin/629-D02485/2009(H1N1)
HA	CY073513	H1N1	09/11/2009	Nicaragua	A/Managua/3014.02/2009(H1N1)
HA	JN588787	H1N1	09/11/2009	Cambodia	A/Cambodia/T354/2009
HA	KT181178	H1N1	09/11/2009	Singapore	A/Singapore/DMS913/2009
HA	CY083064	H1N1	09/11/2009	Colombia	A/Bogota/WRAIR0435N/2009
HA	CY052535	H1N1	09/11/2009	USA	A/Texas/45113882/2009(H1N1)
HA	CY052495	H1N1	09/11/2009	USA	A/Texas/45113371/2009(H1N1)
HA	CY070375	H1N1	09/11/2009	United Ki	A/England/93840010/2009
HA	CY062424	H1N1	09/11/2009	Kuwait	A/Kuwait/N13055/2009(H1N1)
HA	CY052911	H1N1	09/11/2009	USA	A/Texas/45113911/2009(H1N1)
HA	CY072822	H1N1	09/11/2009	Nicaragua	A/Managua/5115.01/2009(H1N1)
HA	AB558310	H1N1	09/11/2009	Japan	A/Sendai/TU400/2009
HA	KC780106	H1N1	09/11/2009	USA	A/Alaska/38/2009
HA	CY123154	H1N1	09/11/2009	Singapore	A/Singapore/GP3545/2009
HA	KC781675	H1N1	09/11/2009	USA	A/Georgia/23/2009
HA	CY128235	H1N1	09/11/2009	Viet Nam	A/Viet Nam/13032044/2009
HA	CY103929	H1N1	09/11/2009	Brazil	A/Pernambuco/565/2009
HA	AB558315	H1N1	09/11/2009	Japan	A/Sendai/TU409/2009
HA	CY062388	H1N1	09/11/2009	Kuwait	A/Kuwait/N10035/2009(H1N1)
HA	CY070391	H1N1	09/11/2009	United Ki	A/England/1005/2009
HA	CY083072	H1N1	09/11/2009	Colombia	A/Bogota/WRAIR0435T/2009
HA	CY057374	H1N1	09/12/2009	USA	A/Wisconsin/629-D01850/2009(H1N1)
HA	CY070372	H1N1	09/12/2009	United Ki	A/England/93820004/2009
HA	CY052487	H1N1	09/12/2009	USA	A/Texas/45122036/2009(H1N1)
HA	KT181108	H1N1	09/12/2009	Singapore	A/Singapore/DMS33/2009
HA	CY052431	H1N1	09/12/2009	USA	A/Texas/45120922/2009(H1N1)
HA	CY052439	H1N1	09/12/2009	USA	A/Texas/45122538/2009(H1N1)
HA	CY052455	H1N1	09/12/2009	USA	A/Texas/45122369/2009(H1N1)
HA	CY052463	H1N1	09/12/2009	USA	A/Texas/45121606/2009(H1N1)
HA	CY062402	H1N1	09/12/2009	Afghanist	A/Afghanistan/N10790/2009(H1N1)
HA	CY057366	H1N1	09/12/2009	USA	A/Wisconsin/629-D00643/2009(H1N1)
HA	CY052471	H1N1	09/12/2009	USA	A/Texas/45122033/2009(H1N1)
HA	CY052551	H1N1	09/12/2009	USA	A/Texas/45122774/2009(H1N1)
HA	CY052479	H1N1	09/12/2009	USA	A/Texas/45122282/2009(H1N1)
HA	CY052559	H1N1	09/12/2009	USA	A/Texas/45122722/2009(H1N1)
HA	CY052927	H1N1	09/12/2009	USA	A/Texas/45121004/2009(H1N1)
HA	CY052943	H1N1	09/12/2009	USA	A/Texas/45122886/2009(H1N1)
HA	CY052178	H1N1	09/12/2009	USA	A/New York/4881/2009(H1N1)
HA	CY050113	H1N1	09/12/2009	China	A/Qingdao/1215/2009(H1N1)
HA	CY072830	H1N1	09/12/2009	Nicaragua	A/Managua/1909.02/2009(H1N1)
HA	CY072734	H1N1	09/12/2009	Nicaragua	A/Managua/1419.02/2009(H1N1)
HA	CY062399	H1N1	09/12/2009	Afghanist	A/Afghanistan/N10782/2009(H1N1)
HA	CY070382	H1N1	09/12/2009	United Ki	A/England/93880053/2009
HA	CY103930	H1N1	09/12/2009	Brazil	A/Pernambuco/567/2009
HA	CY062398	H1N1	09/13/2009	Afghanist	A/Afghanistan/N10771/2009(H1N1)
HA	CY069018	H1N1	09/13/2009	Guam	A/Guam/NHRC0017/2009(H1N1)
HA	CY052799	H1N1	09/13/2009	USA	A/Texas/45131576/2009(H1N1)
HA	CY052583	H1N1	09/13/2009	USA	A/Texas/45132647/2009(H1N1)
HA	CY052575	H1N1	09/13/2009	USA	A/Texas/45131774/2009(H1N1)

HA	KC781828	H1N1	09/13/2009	USA	A/Connecticut/13/2009
HA	CY049651	H1N1	09/13/2009	Singapore	A/Singapore/ON2081/2009(H1N1)
HA	CY052567	H1N1	09/13/2009	USA	A/Texas/45132788/2009(H1N1)
HA	CY052831	H1N1	09/13/2009	USA	A/Texas/45131305/2009(H1N1)
HA	CY070377	H1N1	09/13/2009	United Ki	A/England/93860044/2009
HA	KC780880	H1N1	09/13/2009	USA	A/Texas/67/2009
HA	KC780877	H1N1	09/13/2009	USA	A/Texas/67/2009
HA	CY052839	H1N1	09/13/2009	USA	A/Texas/45132202/2009(H1N1)
HA	CY052543	H1N1	09/13/2009	USA	A/Texas/45132214/2009(H1N1)
HA	CY052447	H1N1	09/13/2009	USA	A/Texas/45130742/2009(H1N1)
HA	CY063797	H1N1	09/13/2009	Singapore	A/Singapore/ON2083/2009
HA	CY062425	H1N1	09/13/2009	Kuwait	A/Kuwait/N13060/2009(H1N1)
HA	CY062401	H1N1	09/13/2009	Afghanist	A/Afghanistan/N10788/2009(H1N1)
HA	CY069042	H1N1	09/14/2009	Guam	A/Guam/NHRC0020/2009(H1N1)
HA	CY070383	H1N1	09/14/2009	United Ki	A/England/93880056/2009
HA	KC780922	H1N1	09/14/2009	USA	A/Kansas/19/2009
HA	KC781778	H1N1	09/14/2009	USA	A/Wisconsin/50/2009
HA	CY123174	H1N1	09/14/2009	Singapore	A/Singapore/GP3710/2009
HA	JX625560	H1N1	09/14/2009	United Ki	A/England/94560024/2009
HA	KC780830	H1N1	09/14/2009	USA	A/Kansas/20/2009
HA	CY123745	H1N1	09/14/2009	Singapore	A/Singapore/ON2081/2009
HA	CY123179	H1N1	09/14/2009	Singapore	A/Singapore/GP3733/2009
HA	JF429396	H1N1	09/14/2009	France	A/StEtienne/1691/2009
HA	KC781753	H1N1	09/14/2009	USA	A/Texas/60/2009
HA	AB558303	H1N1	09/14/2009	Japan	A/Sendai/TU393/2009
HA	JN171929	H1N1	09/14/2009	Canada	A/Montreal/MTL215389/2009
HA	CY052711	H1N1	09/14/2009	USA	A/Texas/45140902/2009(H1N1)
HA	CY055423	H1N1	09/14/2009	USA	A/New York/4887/2009(H1N1)
HA	AB539047	H1N1	09/14/2009	Japan	A/Nagasaki/HA-49/2009(H1N1)
HA	CY072934	H1N1	09/14/2009	Nicaragua	A/Managua/5590.02/2009(H1N1)
HA	CY071007	H1N1	09/14/2009	Guam	A/Guam/NHRC0031/2009(H1N1)
HA	CY071015	H1N1	09/14/2009	Guam	A/Guam/NHRC0032/2009(H1N1)
HA	CY072886	H1N1	09/14/2009	Nicaragua	A/Managua/12.01/2009(H1N1)
HA	CY063799	H1N1	09/14/2009	Singapore	A/Singapore/ON2090/2009
HA	AB558302	H1N1	09/14/2009	Japan	A/Sendai/TU392/2009
HA	CY053474	H1N1	09/15/2009	Taiwan	A/Taiwan/143/2009
HA	AB558304	H1N1	09/15/2009	Japan	A/Sendai/TU394/2009
HA	CY069066	H1N1	09/15/2009	Guam	A/Guam/NHRC0023/2009(H1N1)
HA	CY090089	H1N1	09/15/2009	Nicaragua	A/Managua/3755.03/2009
HA	KC781817	H1N1	09/15/2009	USA	A/Texas/59/2009
HA	KC781533	H1N1	09/15/2009	USA	A/Arizona/16/2009
HA	KC781667	H1N1	09/15/2009	USA	A/South Carolina/26/2009
HA	AB558305	H1N1	09/15/2009	Japan	A/Sendai/TU395/2009
HA	AB558306	H1N1	09/15/2009	Japan	A/Sendai/TU396/2009
HA	AB558311	H1N1	09/15/2009	Japan	A/Sendai/TU402/2009
HA	CY070388	H1N1	09/15/2009	United Ki	A/England/93900683/2009
HA	KC782053	H1N1	09/15/2009	USA	A/South Carolina/24/2009
HA	CY123753	H1N1	09/15/2009	Singapore	A/Singapore/ON2095/2009
HA	CY119490	H1N1	09/15/2009	Malaysia	A/Malaysia/2173411/2009
HA	KC781042	H1N1	09/15/2009	USA	A/Massachusetts/31/2009
HA	KT181109	H1N1	09/15/2009	Singapore	A/Singapore/DMS34/2009
HA	KT181182	H1N1	09/15/2009	Singapore	A/Singapore/DMS952/2009
HA	KT181181	H1N1	09/15/2009	Singapore	A/Singapore/DMS946/2009
HA	CY070385	H1N1	09/15/2009	United Ki	A/England/93880062/2009

HA	CY057382	H1N1	09/15/2009	USA	A/Wisconsin/629-D02192/2009(H1N1)
HA	CY070387	H1N1	09/15/2009	United Ki	A/England/93900114/2009
HA	CY062427	H1N1	09/15/2009	Kuwait	A/Kuwait/N13070/2009(H1N1)
HA	CY057390	H1N1	09/15/2009	USA	A/Wisconsin/629-D00036/2009(H1N1)
HA	CY071730	H1N1	09/15/2009	Mexico	A/Mexico City/WR1697T/2009(H1N1)
HA	CY050117	H1N1	09/15/2009	China	A/Qingdao/1261/2009(H1N1)
HA	CY057406	H1N1	09/15/2009	USA	A/Wisconsin/629-D00832/2009(H1N1)
HA	CY070376	H1N1	09/15/2009	United Ki	A/England/93860037/2009
HA	CY057398	H1N1	09/15/2009	USA	A/Wisconsin/629-D01469/2009(H1N1)
HA	KC780082	H1N1	09/16/2009	USA	A/South Carolina/18/2009
HA	CY075895	H1N1	09/16/2009	India	A/Rtng/NIV11650/2009
HA	CY062428	H1N1	09/16/2009	Kuwait	A/Kuwait/N13081/2009(H1N1)
HA	HQ228068	H1N1	09/16/2009	Finland	A/Finland/619/2009
HA	KT181184	H1N1	09/16/2009	Singapore	A/Singapore/DMS962/2009
HA	CY057422	H1N1	09/16/2009	USA	A/Wisconsin/629-D00485/2009(H1N1)
HA	KC781875	H1N1	09/16/2009	USA	A/Arizona/15/2009
HA	KC782046	H1N1	09/16/2009	USA	A/South Carolina/17/2009
HA	KC781536	H1N1	09/16/2009	USA	A/South Carolina/22/2009
HA	KR861652	H1N1	09/16/2009	India	A/Bangalore/6974-08/2009
HA	KC781829	H1N1	09/16/2009	USA	A/Florida/20/2009
HA	CY050121	H1N1	09/16/2009	China	A/Qingdao/1269/2009(H1N1)
HA	KC782215	H1N1	09/16/2009	USA	A/South Carolina/18/2009
HA	CY057414	H1N1	09/16/2009	USA	A/Wisconsin/629-D01802/2009(H1N1)
HA	KC781310	H1N1	09/16/2009	USA	A/Wisconsin/66/2009
HA	KC781556	H1N1	09/16/2009	USA	A/South Carolina/18/2009
HA	CY050119	H1N1	09/16/2009	China	A/Qingdao/1268/2009(H1N1)
HA	AB558316	H1N1	09/16/2009	Japan	A/Sendai/TU412/2009
HA	AB558317	H1N1	09/16/2009	Japan	A/Sendai/TU413/2009
HA	CY123183	H1N1	09/16/2009	Singapore	A/Singapore/GP3804/2009
HA	CY120731	H1N1	09/16/2009	Brazil	A/Brazil/AVS06/2009
HA	KC782109	H1N1	09/16/2009	USA	A/North Carolina/35/2009
HA	KC782207	H1N1	09/16/2009	USA	A/South Carolina/18/2009
HA	CY070390	H1N1	09/16/2009	United Ki	A/Scotland/93980120/2009
HA	CY047713	H1N1	09/16/2009	China	A/Hangzhou/2/2009(H1N1)
HA	CY072782	H1N1	09/16/2009	Nicaragua	A/Managua/5401.01/2009(H1N1)
HA	CY057430	H1N1	09/16/2009	USA	A/Wisconsin/629-D01935/2009(H1N1)
HA	KC780851	H1N1	09/16/2009	USA	A/Kansas/21/2009
HA	CY090065	H1N1	09/17/2009	Nicaragua	A/Managua/4877.02/2009
HA	CY123690	H1N1	09/17/2009	Singapore	A/Singapore/ON1982/2009
HA	HM189393	H1N1	09/17/2009	Russia	A/Murmansk/CRIE-PAI/2009
HA	KC781956	H1N1	09/17/2009	USA	A/South Carolina/27/2009
HA	KC781959	H1N1	09/17/2009	USA	A/Minnesota/13/2009
HA	CY128243	H1N1	09/17/2009	Viet Nam	A/Viet Nam/13032048/2009
HA	CY123696	H1N1	09/17/2009	Singapore	A/Singapore/ON1999/2009
HA	KC781603	H1N1	09/17/2009	USA	A/South Carolina/38/2009
HA	KC781655	H1N1	09/17/2009	USA	A/South Carolina/23/2009
HA	KC781614	H1N1	09/17/2009	USA	A/South Carolina/25/2009
HA	KP637542	H1N1	09/18/2009	Thailand	A/Thailand/SN08312/2009
HA	CY050130	H1N1	09/18/2009	China	A/Qingdao/1364/2009(H1N1)
HA	CY119498	H1N1	09/18/2009	Malaysia	A/Malaysia/2180812/2009
HA	AB558322	H1N1	09/18/2009	Japan	A/Sendai/TU418/2009
HA	CY053490	H1N1	09/18/2009	Taiwan	A/Taiwan/167/2009
HA	CY053482	H1N1	09/18/2009	Taiwan	A/Taiwan/156/2009
HA	KY926278	H1N1	09/18/2009	Brazil	A/Pelotas/LACENRS-3045/2009

HA	CY070394	H1N1	09/18/2009	United Ki	A/England/706/2009
HA	HQ228070	H1N1	09/18/2009	Finland	A/Finland/621/2009
HA	KT181189	H1N1	09/18/2009	Singapore	A/Singapore/DMS982/2009
HA	KT181110	H1N1	09/18/2009	Singapore	A/Singapore/DMS35/2009
HA	HQ228071	H1N1	09/18/2009	Finland	A/Finland/622/2009
HA	KT181186	H1N1	09/18/2009	Singapore	A/Singapore/DMS978/2009
HA	AB558307	H1N1	09/18/2009	Japan	A/Sendai/TU397/2009
HA	CY123181	H1N1	09/18/2009	Singapore	A/Singapore/GP3791/2009
HA	CY103932	H1N1	09/18/2009	Brazil	A/Pernambuco/592/2009
HA	CY070386	H1N1	09/18/2009	United Ki	A/England/93900113/2009
HA	CY070389	H1N1	09/18/2009	United Ki	A/England/93960018/2009
HA	KC782185	H1N1	09/18/2009	USA	A/Texas/76/2009
HA	KC780059	H1N1	09/18/2009	USA	A/Virginia/20/2009
HA	KC780958	H1N1	09/18/2009	USA	A/North Carolina/58/2009
HA	CY053498	H1N1	09/18/2009	Taiwan	A/Taiwan/177/2009
HA	KT181188	H1N1	09/18/2009	Singapore	A/Singapore/DMS980/2009
HA	CY103931	H1N1	09/18/2009	Brazil	A/Pernambuco/590/2009
HA	CY053506	H1N1	09/18/2009	Taiwan	A/Taiwan/206/2009
HA	CY071754	H1N1	09/18/2009	Mexico	A/Mexico City/WR1706T/2009(H1N1)
HA	CY057438	H1N1	09/18/2009	USA	A/Wisconsin/629-D01522/2009(H1N1)
HA	HM568152	H1N1	09/18/2009	United Ki	A/England/H093900113/2009
HA	CY047714	H1N1	09/19/2009	China	A/Hangzhou/3/2009(H1N1)
HA	CY055502	H1N1	09/19/2009	USA	A/California/VRDL75/2009(H1N1)
HA	KC781617	H1N1	09/20/2009	USA	A/Delaware/11/2009
HA	KC781058	H1N1	09/20/2009	USA	A/North Carolina/44/2009
HA	CY075890	H1N1	09/20/2009	India	A/Kol/NIV105774/2009
HA	CY052008	H1N1	09/20/2009	Norway	A/Norway/3569/2009(H1N1)
HA	KC781870	H1N1	09/20/2009	USA	A/Washington/59/2009
HA	HM214474	H1N1	09/20/2009	China	A/Hangzhou/06/2009(H1N1)
HA	HM214472	H1N1	09/20/2009	China	A/Hangzhou/04/2009(H1N1)
HA	CY068994	H1N1	09/20/2009	Guam	A/Guam/NHRC0014/2009(H1N1)
HA	JN187129	H1N1	09/20/2009	Taiwan	A/Taiwan/8575/2009
HA	CY047712	H1N1	09/20/2009	China	A/Hangzhou/1/2009(H1N1)
HA	AB539739	H1N1	09/21/2009	China	A/Shanghai/P1/2009(H1N1)
HA	KC781545	H1N1	09/21/2009	USA	A/Utah/31/2009
HA	KJ690518	H1N1	09/21/2009	Uganda	A/Uganda/MUWRP-092/2009
HA	CY055510	H1N1	09/21/2009	USA	A/California/VRDL76/2009(H1N1)
HA	CY070395	H1N1	09/21/2009	United Ki	A/England/707/2009
HA	HM189395	H1N1	09/21/2009	Russia	A/Murmansk/CRIE-JAS/2009
HA	HM189394	H1N1	09/21/2009	Russia	A/Murmansk/CRIE-LKA/2009
HA	KT180983	H1N1	09/21/2009	Singapore	A/Singapore/DMS1006/2009
HA	HM189392	H1N1	09/21/2009	Russia	A/Murmansk/CRIE-DII/2009
HA	CY070406	H1N1	09/21/2009	United Ki	A/England/989/2009
HA	CY057454	H1N1	09/21/2009	USA	A/Wisconsin/629-D00748/2009(H1N1)
HA	CY057446	H1N1	09/21/2009	USA	A/Wisconsin/629-D01355/2009(H1N1)
HA	CY083431	H1N1	09/21/2009	USA	A/San Diego/WRAIR1668P/2009
HA	HQ228069	H1N1	09/21/2009	Finland	A/Finland/620/2009
HA	CY070433	H1N1	09/21/2009	United Ki	A/England/94160046/2009
HA	KC780127	H1N1	09/21/2009	USA	A/Mississippi/02/2009
HA	HQ228083	H1N1	09/22/2009	Finland	A/Finland/634/2009
HA	CY123762	H1N1	09/22/2009	Singapore	A/Singapore/ON2131/2009
HA	KC781346	H1N1	09/22/2009	USA	A/Washington/58/2009
HA	HM214478	H1N1	09/22/2009	China	A/Hangzhou/10/2009(H1N1)
HA	KC781980	H1N1	09/22/2009	USA	A/Kansas/22/2009

HA	CY070396	H1N1	09/22/2009	United Ki	A/England/708/2009
HA	CY070440	H1N1	09/22/2009	United Ki	A/Scotland/94160459/2009
HA	CY057478	H1N1	09/22/2009	USA	A/Wisconsin/629-D01237/2009(H1N1)
HA	CY057470	H1N1	09/22/2009	USA	A/Wisconsin/629-D00402/2009(H1N1)
HA	CY103933	H1N1	09/22/2009	Brazil	A/Pernambuco/609/2009
HA	GU211227	H1N1	09/22/2009	Russia	A/Russia/01/2009(H1N1)
HA	CY055518	H1N1	09/22/2009	USA	A/California/VRDL78/2009(H1N1)
HA	CY057462	H1N1	09/22/2009	USA	A/Wisconsin/629-D00839/2009(H1N1)
HA	CY062430	H1N1	09/22/2009	Kuwait	A/Kuwait/N13093/2009(H1N1)
HA	CY070419	H1N1	09/22/2009	United Ki	A/England/723/2009
HA	CY070414	H1N1	09/22/2009	United Ki	A/England/720/2009
HA	CY070392	H1N1	09/22/2009	United Ki	A/England/1006/2009
HA	CY123760	H1N1	09/22/2009	Singapore	A/Singapore/ON2125/2009
HA	CY128443	H1N1	09/22/2009	Viet Nam	A/Viet Nam/001-1716/2009
HA	CY123775	H1N1	09/23/2009	Singapore	A/Singapore/ON2136/2009
HA	KC781574	H1N1	09/23/2009	USA	A/Rhode Island/16/2009
HA	CY070441	H1N1	09/23/2009	United Ki	A/Scotland/94160462/2009
HA	CY083447	H1N1	09/23/2009	Mexico	A/Mexico City/WRAIR1679N/2009(H1N1)
HA	CY062431	H1N1	09/23/2009	Kuwait	A/Kuwait/N13111/2009(H1N1)
HA	KC780974	H1N1	09/23/2009	USA	A/Kansas/23/2009
HA	CY056419	H1N1	09/23/2009	USA	A/New York/4947/2009(H1N1)
HA	CY070393	H1N1	09/23/2009	United Ki	A/England/705/2009
HA	KC781062	H1N1	09/23/2009	USA	A/Florida/23/2009
HA	CY057494	H1N1	09/23/2009	USA	A/Wisconsin/629-D00287/2009(H1N1)
HA	CY056403	H1N1	09/23/2009	USA	A/New York/4944/2009(H1N1)
HA	CY056411	H1N1	09/23/2009	USA	A/New York/4945/2009(H1N1)
HA	CY071698	H1N1	09/23/2009	Mexico	A/Mexico City/WR1687T/2009(H1N1)
HA	HM998845	H1N1	09/23/2009	China	A/Inner Mongolia/03/2009(H1N1)
HA	HM998846	H1N1	09/23/2009	China	A/Inner Mongolia/04/2009(H1N1)
HA	CY057486	H1N1	09/23/2009	USA	A/Wisconsin/629-D00346/2009(H1N1)
HA	CY070400	H1N1	09/23/2009	United Ki	A/England/1008/2009
HA	CY083487	H1N1	09/23/2009	Mexico	A/Mexico City/WRAIR1691N/2009(H1N1)
HA	CY083407	H1N1	09/23/2009	USA	A/San Diego/WRAIR1665P/2009
HA	HQ228072	H1N1	09/23/2009	Finland	A/Finland/623/2009
HA	CY123188	H1N1	09/23/2009	Singapore	A/Singapore/GP3839/2009
HA	CY070397	H1N1	09/23/2009	United Ki	A/England/709/2009
HA	CY123767	H1N1	09/23/2009	Singapore	A/Singapore/ON2135/2009
HA	KJ690400	H1N1	09/23/2009	Uganda	A/Uganda/MUWRP-093/2009
HA	CY103919	H1N1	09/24/2009	Brazil	A/Pernambuco/397/2009
HA	KC781361	H1N1	09/24/2009	USA	A/Montana/18/2009
HA	KT181111	H1N1	09/24/2009	Singapore	A/Singapore/DMS37/2009
HA	CY057502	H1N1	09/24/2009	USA	A/Wisconsin/629-D00565/2009(H1N1)
HA	CY070401	H1N1	09/24/2009	United Ki	A/England/712/2009
HA	HQ595283	H1N1	09/24/2009	Brazil	A/Sao Paulo/82668/2009
HA	CY070399	H1N1	09/24/2009	United Ki	A/England/711/2009
HA	CY071738	H1N1	09/24/2009	Mexico	A/Mexico City/WR1701T/2009(H1N1)
HA	KC782335	H1N1	09/24/2009	USA	A/Illinois/18/2009
HA	CY057510	H1N1	09/24/2009	USA	A/Wisconsin/629-D00410/2009(H1N1)
HA	CY054883	H1N1	09/24/2009	USA	A/California/VRDL36/2009(H1N1)
HA	CY054598	H1N1	09/24/2009	Thailand	A/Thailand/PMKD0028/2009(H1N1)
HA	CY070403	H1N1	09/24/2009	United Ki	A/England/714/2009
HA	CY070405	H1N1	09/24/2009	United Ki	A/England/1009/2009
HA	CY103934	H1N1	09/24/2009	Brazil	A/Pernambuco/624/2009
HA	CY070402	H1N1	09/24/2009	United Ki	A/England/713/2009

HA	CY070409	H1N1	09/24/2009	United Ki	A/England/94060040/2009
HA	HQ595282	H1N1	09/24/2009	Brazil	A/Sao Paulo/82666/2009
HA	CY119506	H1N1	09/24/2009	Malaysia	A/Malaysia/2190140/2009
HA	KC781833	H1N1	09/24/2009	USA	A/Vermont/19/2009
HA	HM189362	H1N1	09/25/2009	Russia	A/Kaliningrad/CRIE-ZD/2009
HA	CY054599	H1N1	09/25/2009	Thailand	A/Thailand/PMKD0030/2009(H1N1)
HA	KC780265	H1N1	09/25/2009	USA	A/Minnesota/21/2009
HA	KC780711	H1N1	09/25/2009	USA	A/Nebraska/19/2009
HA	HM189365	H1N1	09/25/2009	Russia	A/Kaliningrad/CRIE-KG/2009
HA	HM189363	H1N1	09/25/2009	Russia	A/Kaliningrad/CRIE-MA/2009
HA	HM189364	H1N1	09/25/2009	Russia	A/Kaliningrad/CRIE-SHD/2009
HA	CY070398	H1N1	09/25/2009	United Ki	A/England/710/2009
HA	AB558312	H1N1	09/25/2009	Japan	A/Sendai/TU406/2009
HA	CY069058	H1N1	09/25/2009	Guam	A/Guam/NHRC0022/2009(H1N1)
HA	CY070418	H1N1	09/25/2009	United Ki	A/England/94080379/2009
HA	CY071746	H1N1	09/25/2009	Mexico	A/Mexico City/WR1704T/2009(H1N1)
HA	CY050368	H1N1	09/25/2009	South Kor	A/Korea/S1/2009
HA	KC780156	H1N1	09/25/2009	USA	A/Mississippi/03/2009
HA	KC781324	H1N1	09/26/2009	USA	A/Michigan/24/2009
HA	CY069074	H1N1	09/26/2009	Guam	A/Guam/NHRC0024/2009(H1N1)
HA	CY070415	H1N1	09/26/2009	United Ki	A/England/94080026/2009
HA	CY070407	H1N1	09/26/2009	United Ki	A/England/715/2009
HA	HM189366	H1N1	09/26/2009	Russia	A/Kaliningrad/CRIE-DA/2009
HA	CY070420	H1N1	09/26/2009	United Ki	A/England/1011/2009
HA	CY057518	H1N1	09/26/2009	USA	A/Wisconsin/629-D00891/2009(H1N1)
HA	KC780907	H1N1	09/27/2009	USA	A/Wisconsin/51/2009
HA	CY070411	H1N1	09/27/2009	United Ki	A/England/717/2009
HA	HQ595284	H1N1	09/27/2009	Brazil	A/Sao Paulo/83218/2009
HA	CY070413	H1N1	09/27/2009	United Ki	A/England/1012/2009
HA	CY070435	H1N1	09/27/2009	United Ki	A/England/94160055/2009
HA	CY070408	H1N1	09/27/2009	United Ki	A/England/1010/2009
HA	JN187130	H1N1	09/28/2009	Taiwan	A/Taiwan/4909/2009
HA	CY070442	H1N1	09/28/2009	United Ki	A/Scotland/90/2009
HA	KC782302	H1N1	09/28/2009	USA	A/California/38/2009
HA	CY070412	H1N1	09/28/2009	United Ki	A/England/718/2009
HA	JN187131	H1N1	09/28/2009	Taiwan	A/Taiwan/8542/2009
HA	AB558321	H1N1	09/28/2009	Japan	A/Sendai/TU417/2009
HA	AB558324	H1N1	09/28/2009	Japan	A/Sendai/TU426/2009
HA	CY070416	H1N1	09/28/2009	United Ki	A/England/721/2009
HA	CY123783	H1N1	09/28/2009	Singapore	A/Singapore/ON2145/2009
HA	KJ690479	H1N1	09/28/2009	Uganda	A/Uganda/MUWRP-098/2009
HA	CY070404	H1N1	09/28/2009	United Ki	A/England/94020500/2009
HA	KC781909	H1N1	09/28/2009	USA	A/Texas/62/2009
HA	CY057526	H1N1	09/28/2009	USA	A/Wisconsin/629-D01391/2009(H1N1)
HA	KP019827	H1N1	09/28/2009	China	A/FuZhou/SWL11479/2009
HA	CY070434	H1N1	09/28/2009	United Ki	A/England/1023/2009
HA	JQ431202	H1N1	09/28/2009	France	A/Reunion/1698-3-M1E/2009
HA	CY070436	H1N1	09/29/2009	United Ki	A/England/94160067/2009
HA	KC780899	H1N1	09/29/2009	USA	A/Alaska/39/2009
HA	CY070424	H1N1	09/29/2009	United Ki	A/England/94120045/2009
HA	CY070417	H1N1	09/29/2009	United Ki	A/England/722/2009
HA	CY123193	H1N1	09/29/2009	Singapore	A/Singapore/GP3869/2009
HA	CY060558	H1N1	09/29/2009	Canada	A/Ontario/26184/2009
HA	KC782077	H1N1	09/29/2009	USA	A/Pennsylvania/25/2009

HA	CY058276	H1N1	09/29/2009	USA	A/Wisconsin/629-D01015/2009(H1N1)
HA	KC780998	H1N1	09/29/2009	USA	A/Texas/66/2009
HA	AB558318	H1N1	09/29/2009	Japan	A/Sendai/TU414/2009
HA	KC780993	H1N1	09/29/2009	USA	A/Texas/66/2009
HA	CY072950	H1N1	09/29/2009	Nicaragua	A/Managua/5258.02/2009(H1N1)
HA	CY070444	H1N1	09/30/2009	United Ki	A/Scotland/91/2009
HA	CY123198	H1N1	09/30/2009	Singapore	A/Singapore/GP3912/2009
HA	CY070427	H1N1	09/30/2009	United Ki	A/England/726/2009
HA	CY075884	H1N1	09/30/2009	India	A/Delhi/NIV0951830/2009
HA	CY083439	H1N1	09/30/2009	USA	A/Ft.Benning/WRAIR1669P/2009
HA	CY057044	H1N1	09/30/2009	United Ki	A/England/94280035/2009
HA	AB558320	H1N1	09/30/2009	Japan	A/Sendai/TU416/2009
HA	CY070421	H1N1	09/30/2009	United Ki	A/England/724/2009
HA	CY075885	H1N1	09/30/2009	India	A/Delhi/NIV0951831/2009
HA	JX875011	H1N1	09/30/2009	USA	A/Kentucky/80/2009
HA	CY070422	H1N1	09/30/2009	United Ki	A/England/94080396/2009
HA	KC780027	H1N1	09/30/2009	USA	A/Wisconsin/67/2009
HA	KP019828	H1N1	09/30/2009	China	A/FuZhou/SWL11510/2009
HA	KT181113	H1N1	09/30/2009	Singapore	A/Singapore/DMS39/2009
HA	CY070443	H1N1	09/30/2009	United Ki	A/Scotland/29/2009
HA	KC780282	H1N1	09/30/2009	USA	A/South Dakota/11/2009
HA	CY065920	H1N1	09/30/2009	Netherlan	A/Netherlands/2290/2009
HA	KC780073	H1N1	09/30/2009	USA	A/Oklahoma/04/2009
HA	KP317214	H1N1	09/30/2009	India	A/Chattisgarh/010/2009
HA	CY123788	H1N1	09/30/2009	Singapore	A/Singapore/ON2159/2009
HA	HQ228085	H1N1	09/30/2009	Finland	A/Finland/636/2009
HA	CY062359	H1N1	10/2009	Egypt	A/Egypt/N11635/2009(H1N1)
HA	CY062364	H1N1	10/2009	Egypt	A/Egypt/N11652/2009(H1N1)
HA	CY062411	H1N1	10/2009	Bahrain	A/Bahrain/N11890/2009(H1N1)
HA	CY062370	H1N1	10/2009	Ghana	A/Ghana/N12981/2009(H1N1)
HA	CY062372	H1N1	10/2009	Ghana	A/Ghana/N12987/2009(H1N1)
HA	CY062408	H1N1	10/2009	Bahrain	A/Bahrain/N11659/2009(H1N1)
HA	CY062410	H1N1	10/2009	Bahrain	A/Bahrain/N11661/2009(H1N1)
HA	CY062363	H1N1	10/2009	Egypt	A/Egypt/N11651/2009(H1N1)
HA	CY062354	H1N1	10/2009	Egypt	A/Egypt/N10305/2009(H1N1)
HA	CY062362	H1N1	10/2009	Egypt	A/Egypt/N11649/2009(H1N1)
HA	HQ228087	H1N1	10/2009	Finland	A/Finland/642/2009
HA	GU968915	H1N1	10/2009	China	A/Beijing/SE2365/2009(H1N1)
HA	GU968916	H1N1	10/2009	China	A/Beijing/SE2449/2009(H1N1)
HA	JQ364992	H1N1	10/2009	China	A/Hangzhou/1708/2009
HA	HM189486	H1N1	10/2009	South Kor	A/Korea/CJ63/2009(H1N1)
HA	HM189488	H1N1	10/2009	South Kor	A/Korea/CJ109/2009(H1N1)
HA	HM189489	H1N1	10/2009	South Kor	A/Korea/CJ112/2009(H1N1)
HA	AB745131	H1N1	10/2009	Japan	A/Tottori/ST243/2009
HA	JN381240	H1N1	10/2009	Taiwan	A/Taiwan/99422/2009
HA	JN381241	H1N1	10/2009	Taiwan	A/Taiwan/99502/2009
HA	JN381235	H1N1	10/2009	Taiwan	A/Taiwan/99127/2009
HA	JQ364991	H1N1	10/2009	China	A/Hangzhou/1693/2009
HA	JQ364994	H1N1	10/2009	China	A/Hangzhou/1715/2009
HA	AB745129	H1N1	10/2009	Japan	A/Tottori/ST241/2009
HA	AB745134	H1N1	10/2009	Japan	A/Tottori/ST246/2009
HA	CY071940	H1N1	10/2009	Egypt	A/Egypt/N11243/2009(H1N1)
HA	CY067614	H1N1	10/2009	China	A/Qingdao/1517/2009
HA	AB745124	H1N1	10/2009	Japan	A/Tottori/ST235/2009

HA	AB745125	H1N1	10/2009	Japan	A/Tottori/ST237/2009
HA	AB745128	H1N1	10/2009	Japan	A/Tottori/ST240/2009
HA	JN381236	H1N1	10/2009	Taiwan	A/Taiwan/99144/2009
HA	HM189487	H1N1	10/2009	South Ko	A/Korea/CJ68/2009(H1N1)
HA	AB745130	H1N1	10/2009	Japan	A/Tottori/ST242/2009
HA	JN381237	H1N1	10/2009	Taiwan	A/Taiwan/99330/2009
HA	CY062369	H1N1	10/2009	Ghana	A/Ghana/N12979/2009(H1N1)
HA	GU369664	H1N1	10/2009	Turkey	A/Ankara/20/2009
HA	CY062361	H1N1	10/2009	Egypt	A/Egypt/N11643/2009(H1N1)
HA	JQ364993	H1N1	10/2009	China	A/Hangzhou/1709/2009
HA	CY067615	H1N1	10/2009	China	A/Qingdao/1530/2009
HA	AB745133	H1N1	10/2009	Japan	A/Tottori/ST245/2009
HA	GU369665	H1N1	10/2009	Turkey	A/Ankara/21/2009
HA	GU369663	H1N1	10/2009	Turkey	A/Ankara/19/2009
HA	CY062409	H1N1	10/2009	Bahrain	A/Bahrain/N11660/2009(H1N1)
HA	HM189485	H1N1	10/2009	South Ko	A/Korea/CJ40/2009(H1N1)
HA	AB745135	H1N1	10/2009	Japan	A/Tottori/ST247/2009
HA	AB745132	H1N1	10/2009	Japan	A/Tottori/ST244/2009
HA	CY062412	H1N1	10/2009	Bahrain	A/Bahrain/N11892/2009(H1N1)
HA	HM189412	H1N1	10/2009	Russia	A/Perm/CRIE-PMI/2009
HA	HQ228086	H1N1	10/2009	Finland	A/Finland/641/2009
HA	CY071939	H1N1	10/2009	Egypt	A/Egypt/N10843/2009(H1N1)
HA	CY071941	H1N1	10/2009	Egypt	A/Egypt/N11254/2009(H1N1)
HA	AB745127	H1N1	10/2009	Japan	A/Tottori/ST239/2009
HA	AB745137	H1N1	10/2009	Japan	A/Tottori/ST252/2009
HA	JN381233	H1N1	10/2009	Taiwan	A/Taiwan/90230/2009
HA	CY062356	H1N1	10/2009	Egypt	A/Egypt/N10477/2009(H1N1)
HA	CY067613	H1N1	10/2009	China	A/Qingdao/1508/2009
HA	JN381234	H1N1	10/2009	Taiwan	A/Taiwan/90233/2009
HA	JN381238	H1N1	10/2009	Taiwan	A/Taiwan/99334/2009
HA	JN381239	H1N1	10/2009	Taiwan	A/Taiwan/99384/2009
HA	AB745136	H1N1	10/2009	Japan	A/Tottori/ST249/2009
HA	AB745123	H1N1	10/2009	Japan	A/Tottori/ST234/2009
HA	AB745126	H1N1	10/2009	Japan	A/Tottori/ST238/2009
HA	CY062360	H1N1	10/2009	Egypt	A/Egypt/N11640/2009(H1N1)
HA	CY062368	H1N1	10/2009	Ghana	A/Ghana/N12975/2009(H1N1)
HA	CY062371	H1N1	10/2009	Ghana	A/Ghana/N12984/2009(H1N1)
HA	CY062355	H1N1	10/2009	Egypt	A/Egypt/N10306/2009(H1N1)
HA	CY083415	H1N1	10/01/2009	USA	A/San Diego/WRAIR1666P/2009
HA	JX625544	H1N1	10/01/2009	United K	A/Northern Ireland/94480417/2009
HA	CY123790	H1N1	10/01/2009	Singapore	A/Singapore/ON2168/2009
HA	CY062210	H1N1	10/01/2009	USA	A/California/VRDL81/2009(H1N1)
HA	KC780964	H1N1	10/01/2009	USA	A/Alaska/40/2009
HA	KC782079	H1N1	10/01/2009	USA	A/Minnesota/14/2009
HA	CY062202	H1N1	10/01/2009	USA	A/California/VRDL80/2009(H1N1)
HA	KC781991	H1N1	10/01/2009	USA	A/North Carolina/45/2009
HA	CY118267	H1N1	10/01/2009	Malaysia	A/Malaysia/2205822/2009
HA	CY063219	H1N1	10/01/2009	USA	A/Wisconsin/629-D01351/2009(H1N1)
HA	CY070426	H1N1	10/01/2009	United K	A/England/725/2009
HA	CY062194	H1N1	10/01/2009	USA	A/California/VRDL79/2009(H1N1)
HA	CY063801	H1N1	10/01/2009	Singapore	A/Singapore/ON2172/2009
HA	CY062218	H1N1	10/01/2009	USA	A/California/VRDL82/2009(H1N1)
HA	HM189372	H1N1	10/01/2009	Russia	A/Salehard/CRIE-HSN/2009
HA	KC780262	H1N1	10/01/2009	USA	A/Florida/21/2009

HA	CY071714	H1N1	10/01/2009	Mexico	A/Mexico City/WR1695N/2009(H1N1)
HA	CY068954	H1N1	10/01/2009	Guam	A/Guam/NHRC0009/2009(H1N1)
HA	CY069002	H1N1	10/01/2009	Guam	A/Guam/NHRC0015/2009(H1N1)
HA	KC780269	H1N1	10/01/2009	USA	A/Connecticut/14/2009
HA	KC782147	H1N1	10/01/2009	USA	A/Illinois/13/2009
HA	CY062226	H1N1	10/01/2009	USA	A/California/VRDL83/2009(H1N1)
HA	CY056571	H1N1	10/01/2009	USA	A/New York/5755/2009(H1N1)
HA	CY070425	H1N1	10/01/2009	United Ki	A/England/1014/2009
HA	CY070437	H1N1	10/01/2009	United Ki	A/England/94160072/2009
HA	CY070428	H1N1	10/01/2009	United Ki	A/England/727/2009
HA	CY070438	H1N1	10/01/2009	United Ki	A/England/729/2009
HA	CY128251	H1N1	10/01/2009	Viet Nam	A/Viet Nam/13032051/2009
HA	AB558319	H1N1	10/01/2009	Japan	A/Sendai/TU415/2009
HA	CY070423	H1N1	10/02/2009	United Ki	A/England/94120043/2009
HA	KC780845	H1N1	10/02/2009	USA	A/Iowa/09/2009
HA	KC780761	H1N1	10/02/2009	USA	A/Maryland/29/2009
HA	KC781365	H1N1	10/02/2009	USA	A/Nevada/16/2009
HA	KC780138	H1N1	10/02/2009	USA	A/New Mexico/08/2009
HA	KT181114	H1N1	10/02/2009	Singapore	A/Singapore/DMS40/2009
HA	CY070439	H1N1	10/02/2009	United Ki	A/England/94160078/2009
HA	CY057542	H1N1	10/02/2009	USA	A/Wisconsin/629-D00853/2009(H1N1)
HA	CY070447	H1N1	10/02/2009	United Ki	A/England/730/2009
HA	CY070457	H1N1	10/02/2009	United Ki	A/England/94200009/2009
HA	CY070501	H1N1	10/02/2009	United Ki	A/England/757/2009
HA	CY083423	H1N1	10/02/2009	USA	A/San Diego/WRAIR1667P/2009
HA	CY057534	H1N1	10/02/2009	USA	A/Wisconsin/629-D00250/2009(H1N1)
HA	CY052009	H1N1	10/02/2009	Norway	A/Norway/3689/2009(H1N1)
HA	CY057550	H1N1	10/03/2009	USA	A/Wisconsin/629-D02337/2009(H1N1)
HA	CY055431	H1N1	10/03/2009	USA	A/New York/4977/2009(H1N1)
HA	CY103935	H1N1	10/03/2009	Brazil	A/Pernambuco/643/2009
HA	CY070429	H1N1	10/03/2009	United Ki	A/England/1018/2009
HA	CY070445	H1N1	10/03/2009	United Ki	A/Scotland/89/2009
HA	CY070446	H1N1	10/04/2009	United Ki	A/Scotland/94180012/2009
HA	CY057558	H1N1	10/04/2009	USA	A/Wisconsin/629-D00690/2009(H1N1)
HA	CY056483	H1N1	10/04/2009	USA	A/New York/4995/2009(H1N1)
HA	CY070454	H1N1	10/04/2009	United Ki	A/England/731/2009
HA	JN171889	H1N1	10/04/2009	Canada	A/Quebec/QC151409/2009
HA	KC780377	H1N1	10/04/2009	USA	A/New York/101/2009
HA	CY070448	H1N1	10/04/2009	United Ki	A/England/728/2009
HA	CY070432	H1N1	10/04/2009	United Ki	A/England/94160004/2009
HA	KP317209	H1N1	10/04/2009	India	A/Uttar Pradesh/005/2009
HA	KC781326	H1N1	10/04/2009	USA	A/Texas/61/2009
HA	KC782119	H1N1	10/04/2009	USA	A/Alaska/37/2009
HA	CY070465	H1N1	10/04/2009	United Ki	A/England/94240372/2009
HA	HQ228084	H1N1	10/05/2009	Finland	A/Finland/635/2009
HA	CY071674	H1N1	10/05/2009	Mexico	A/Mexico City/WR1673N/2009(H1N1)
HA	CY056443	H1N1	10/05/2009	USA	A/New York/4985/2009(H1N1)
HA	CY056427	H1N1	10/05/2009	USA	A/New York/4981/2009(H1N1)
HA	CY056475	H1N1	10/05/2009	USA	A/New York/4990/2009(H1N1)
HA	CY056451	H1N1	10/05/2009	USA	A/New York/4986/2009(H1N1)
HA	CY055439	H1N1	10/05/2009	USA	A/New York/4979/2009(H1N1)
HA	CY057566	H1N1	10/05/2009	USA	A/Wisconsin/629-D00834/2009(H1N1)
HA	KT181115	H1N1	10/05/2009	Singapore	A/Singapore/DMS41/2009
HA	KC780176	H1N1	10/05/2009	USA	A/Minnesota/24/2009

HA	KC780640	H1N1	10/05/2009	USA	A/California/39/2009
HA	CY070430	H1N1	10/05/2009	United Ki	A/England/94120625/2009
HA	CY070451	H1N1	10/05/2009	United Ki	A/England/733/2009
HA	CY070452	H1N1	10/05/2009	United Ki	A/England/734/2009
HA	JX625528	H1N1	10/05/2009	United Ki	A/Northern Ireland/94480397/2009
HA	CY123797	H1N1	10/05/2009	Singapore	A/Singapore/ON2180/2009
HA	CY070466	H1N1	10/05/2009	United Ki	A/England/94240376/2009
HA	KC781375	H1N1	10/05/2009	USA	A/Oregon/35/2009
HA	AB558330	H1N1	10/05/2009	Japan	A/Sendai/TU433/2009
HA	KJ690542	H1N1	10/05/2009	Uganda	A/Uganda/MUWRP-102/2009
HA	CY070431	H1N1	10/05/2009	United Ki	A/England/732/2009
HA	CY070453	H1N1	10/05/2009	United Ki	A/England/735/2009
HA	AB558332	H1N1	10/05/2009	Japan	A/Sendai/TU437/2009
HA	AB558323	H1N1	10/05/2009	Japan	A/Sendai/TU420/2009
HA	HM776031	H1N1	10/06/2009	South Kor	A/Seoul/Y-01/2009(H1N1)
HA	CY056459	H1N1	10/06/2009	USA	A/New York/4987/2009(H1N1)
HA	CY057574	H1N1	10/06/2009	USA	A/Wisconsin/629-S1252/2009(H1N1)
HA	CY056467	H1N1	10/06/2009	USA	A/New York/4988/2009(H1N1)
HA	KM289092	H1N1	10/06/2009	Peru	A/Peru/2010731296/2009
HA	KC780932	H1N1	10/06/2009	USA	A/Colorado/13/2009
HA	KC780935	H1N1	10/06/2009	USA	A/Colorado/13/2009
HA	CY070455	H1N1	10/06/2009	United Ki	A/England/94180058/2009
HA	CY070487	H1N1	10/06/2009	United Ki	A/England/1080/2009
HA	CY075898	H1N1	10/06/2009	India	A/Ngp/NIV14725/2009(H1N1)
HA	CY075887	H1N1	10/06/2009	India	A/Delhi/NIV0951833/2009
HA	CY083080	H1N1	10/06/2009	Colombia	A/Bogota/WRAIR0440T/2009
HA	KP317210	H1N1	10/06/2009	India	A/Goa/006/2009
HA	AB558325	H1N1	10/06/2009	Japan	A/Sendai/TU428/2009
HA	CY058284	H1N1	10/06/2009	USA	A/Wisconsin/629-D01102/2009(H1N1)
HA	KC782129	H1N1	10/06/2009	USA	A/Texas/77/2009
HA	KC780268	H1N1	10/06/2009	USA	A/Delaware/13/2009
HA	AB535743	H1N1	10/06/2009	Japan	A/Nagasaki/HA-50/2009(H1N1)
HA	CY057043	H1N1	10/06/2009	United Ki	A/England/94280034/2009
HA	CY056435	H1N1	10/06/2009	USA	A/New York/4984/2009(H1N1)
HA	CY075886	H1N1	10/06/2009	India	A/Delhi/NIV0951832/2009
HA	KC782091	H1N1	10/06/2009	USA	A/Washington/60/2009
HA	CY070449	H1N1	10/06/2009	United Ki	A/Scotland/30/2009
HA	CY058292	H1N1	10/07/2009	USA	A/Wisconsin/629-D02100/2009(H1N1)
HA	CY062357	H1N1	10/07/2009	Egypt	A/Egypt/N10296/2009(H1N1)
HA	KM203128	H1N1	10/07/2009	India	A/Kerala/RGCBA923/2009
HA	CY062358	H1N1	10/07/2009	Egypt	A/Egypt/N10297/2009(H1N1)
HA	CY075899	H1N1	10/07/2009	India	A/Pune/NIV14886/2009
HA	KC781052	H1N1	10/07/2009	USA	A/North Carolina/57/2009
HA	CY070450	H1N1	10/07/2009	United Ki	A/England/94180043/2009
HA	CY070510	H1N1	10/07/2009	United Ki	A/England/758/2009
HA	CY070474	H1N1	10/07/2009	United Ki	A/England/746/2009
HA	CY083112	H1N1	10/07/2009	Ecuador	A/Quito/WRAIR0617N/2009
HA	CY083120	H1N1	10/07/2009	Ecuador	A/Quito/WRAIR0617T/2009(H1N1)
HA	JN171930	H1N1	10/07/2009	Canada	A/Montreal/MTL236425/2009
HA	KC780131	H1N1	10/07/2009	USA	A/Oregon/33/2009
HA	KR861653	H1N1	10/07/2009	India	A/Bangalore/8330-09/2009
HA	CY075492	H1N1	10/07/2009	USA	A/Boston/584/2009(H1N1)
HA	CY070464	H1N1	10/07/2009	United Ki	A/Scotland/31/2009
HA	KC781032	H1N1	10/07/2009	USA	A/Wyoming/15/2009

HA	CY070456	H1N1	10/07/2009	United Ki	A/England/94200007/2009
HA	KC780895	H1N1	10/07/2009	USA	A/Idaho/13/2009
HA	CY063803	H1N1	10/08/2009	Singapore	A/Singapore/ON2193/2009
HA	KC882400	H1N1	10/08/2009	USA	A/Texas/79/2009
HA	KT181116	H1N1	10/08/2009	Singapore	A/Singapore/DMS42/2009
HA	KC780440	H1N1	10/08/2009	USA	A/Tennessee/17/2009
HA	KC781229	H1N1	10/08/2009	USA	A/South Carolina/39/2009
HA	CY070476	H1N1	10/08/2009	United Ki	A/England/737/2009
HA	CY070462	H1N1	10/08/2009	United Ki	A/England/736/2009
HA	CY070458	H1N1	10/08/2009	United Ki	A/England/995/2009
HA	JN187132	H1N1	10/08/2009	Taiwan	A/Taiwan/7418/2009
HA	CY070459	H1N1	10/08/2009	United Ki	A/England/1040/2009
HA	CY070461	H1N1	10/08/2009	United Ki	A/England/740/2009
HA	KC780713	H1N1	10/08/2009	USA	A/Vermont/20/2009
HA	CY128267	H1N1	10/08/2009	Viet Nam	A/Viet Nam/13032060/2009
HA	CY128259	H1N1	10/08/2009	Viet Nam	A/Viet Nam/13032059/2009
HA	AB558326	H1N1	10/08/2009	Japan	A/Sendai/TU429/2009
HA	CY057598	H1N1	10/09/2009	USA	A/Wisconsin/629-D01080/2009(H1N1)
HA	CY057582	H1N1	10/09/2009	USA	A/Wisconsin/629-D01749/2009(H1N1)
HA	CY062234	H1N1	10/09/2009	USA	A/California/VRDL84/2009(H1N1)
HA	AB535744	H1N1	10/09/2009	Japan	A/Nagasaki/HA-52/2009(H1N1)
HA	AB535745	H1N1	10/09/2009	Japan	A/Nagasaki/HA-53/2009(H1N1)
HA	CY063067	H1N1	10/09/2009	USA	A/California/VRDL87/2009(H1N1)
HA	CY063075	H1N1	10/09/2009	USA	A/California/VRDL88/2009(H1N1)
HA	CY062242	H1N1	10/09/2009	USA	A/California/VRDL85/2009(H1N1)
HA	CY062250	H1N1	10/09/2009	USA	A/California/VRDL86/2009(H1N1)
HA	KC781685	H1N1	10/09/2009	USA	A/Hawaii/27/2009
HA	CY070484	H1N1	10/09/2009	United Ki	A/England/741/2009
HA	CY070475	H1N1	10/09/2009	United Ki	A/England/756/2009
HA	CY070477	H1N1	10/09/2009	United Ki	A/England/738/2009
HA	JX625513	H1N1	10/09/2009	United Ki	A/England/1072/2009
HA	JX625536	H1N1	10/09/2009	United Ki	A/Northern Ireland/94480404/2009
HA	CY123802	H1N1	10/09/2009	Singapore	A/Singapore/ON2195/2009
HA	KC781047	H1N1	10/09/2009	USA	A/Kentucky/24/2009
HA	JX625606	H1N1	10/09/2009	United Ki	A/Northern Ireland/94940020/2009(H1N
HA	KC781838	H1N1	10/09/2009	USA	A/Arkansas/07/2009
HA	CY071762	H1N1	10/09/2009	Mexico	A/Mexico City/WR1708T/2009(H1N1)
HA	HQ228097	H1N1	10/09/2009	Finland	A/Finland/654/2009
HA	CY057590	H1N1	10/09/2009	USA	A/Wisconsin/629-D02090/2009(H1N1)
HA	KP637462	H1N1	10/09/2009	Thailand	A/Thailand/PY08063/2009
HA	CY128275	H1N1	10/09/2009	Viet Nam	A/Viet Nam/13032061/2009
HA	KP317215	H1N1	10/10/2009	India	A/Madhya Pradesh/011/2009
HA	CY089187	H1N1	10/10/2009	USA	A/Boston/583/2009(H1N1)
HA	KC780978	H1N1	10/10/2009	USA	A/Nebraska/20/2009
HA	KC782194	H1N1	10/10/2009	USA	A/Hawaii/28/2009
HA	KC782176	H1N1	10/10/2009	USA	A/Tennessee/18/2009
HA	CY070463	H1N1	10/10/2009	United Ki	A/England/1044/2009
HA	KC780395	H1N1	10/10/2009	USA	A/New Mexico/06/2009
HA	CY057606	H1N1	10/10/2009	USA	A/Wisconsin/629-D00557/2009(H1N1)
HA	KC297141	H1N1	10/10/2009	Saudi Ara	A/Jeddah/1296/2009
HA	KC297146	H1N1	10/10/2009	Saudi Ara	A/Jeddah/1365/2009
HA	CY070472	H1N1	10/10/2009	United Ki	A/England/94260047/2009
HA	CY095963	H1N1	10/11/2009	China	A/Zhejiang/87/2009
HA	CY095949	H1N1	10/11/2009	China	A/Zhejiang/85/2009

HA	CY096045	H1N1	10/11/2009	China	A/Hubei/99/2009
HA	CY070460	H1N1	10/11/2009	United Ki	A/England/94220046/2009
HA	CY083056	H1N1	10/11/2009	USA	A/Colorado/WRAIR0353S/2009
HA	CY070470	H1N1	10/11/2009	United Ki	A/England/94260020/2009
HA	CY075900	H1N1	10/11/2009	India	A/Pune/NIV15492/2009
HA	CY095942	H1N1	10/11/2009	China	A/Zhejiang/84/2009
HA	CY095935	H1N1	10/11/2009	China	A/Zhejiang/83/2009
HA	CY095971	H1N1	10/11/2009	China	A/Zhejiang/88/2009
HA	CY090057	H1N1	10/11/2009	Nicaragua	A/Managua/4606.07/2009
HA	CY072694	H1N1	10/11/2009	Nicaragua	A/Managua/3013.01/2009(H1N1)
HA	CY095955	H1N1	10/11/2009	China	A/Zhejiang/86/2009
HA	CY095735	H1N1	10/11/2009	China	A/Hubei/100/2009
HA	CY070469	H1N1	10/11/2009	United Ki	A/Scotland/33/2009
HA	CY123208	H1N1	10/11/2009	Singapore	A/Singapore/GP4000/2009
HA	CY070511	H1N1	10/11/2009	United Ki	A/England/94340805/2009
HA	CY070468	H1N1	10/11/2009	United Ki	A/Scotland/32/2009
HA	KC782113	H1N1	10/11/2009	USA	A/Arkansas/08/2009
HA	CY096023	H1N1	10/12/2009	China	A/Zhejiang/96/2009
HA	KC780288	H1N1	10/12/2009	USA	A/Pennsylvania/26/2009
HA	KC781100	H1N1	10/12/2009	USA	A/Texas/65/2009
HA	KC261318	H1N1	10/12/2009	Mexico	A/Mexico City/INERPHAC1/2009(H1N1)
HA	KC782087	H1N1	10/12/2009	USA	A/Texas/64/2009
HA	KC781115	H1N1	10/12/2009	USA	A/Wisconsin/52/2009
HA	CY070473	H1N1	10/12/2009	United Ki	A/England/747/2009
HA	HM568064	H1N1	10/12/2009	United Ki	A/England/H094240645/2009
HA	CY096016	H1N1	10/12/2009	China	A/Zhejiang/95/2009
HA	CY095999	H1N1	10/12/2009	China	A/Zhejiang/92/2009
HA	CY096037	H1N1	10/12/2009	China	A/Zhejiang/98/2009
HA	HQ228095	H1N1	10/12/2009	Finland	A/Finland/652/2009
HA	CY055964	H1N1	10/12/2009	USA	A/San Diego/INS03/2009(H1N1)
HA	CY056212	H1N1	10/12/2009	USA	A/San Diego/INS53/2009(H1N1)
HA	CY063227	H1N1	10/12/2009	USA	A/Wisconsin/629-D00675/2009(H1N1)
HA	CY055972	H1N1	10/12/2009	USA	A/San Diego/INS04/2009(H1N1)
HA	CY055948	H1N1	10/12/2009	USA	A/San Diego/INS01/2009(H1N1)
HA	CY095986	H1N1	10/12/2009	China	A/Zhejiang/90/2009
HA	CY096005	H1N1	10/12/2009	China	A/Zhejiang/93/2009
HA	KC780955	H1N1	10/12/2009	USA	A/Wisconsin/60/2009
HA	KC780835	H1N1	10/12/2009	USA	A/Virginia/21/2009
HA	CY052011	H1N1	10/12/2009	Norway	A/Norway/3797/2009(H1N1)
HA	CY057622	H1N1	10/12/2009	USA	A/Wisconsin/629-S1313/2009(H1N1)
HA	CY100464	H1N1	10/12/2009	Mexico	A/Mexico City/INER2/2009
HA	KP637278	H1N1	10/12/2009	Thailand	A/Thailand/IY07093/2009
HA	CY055956	H1N1	10/12/2009	USA	A/San Diego/INS02/2009(H1N1)
HA	CY056875	H1N1	10/12/2009	USA	A/San Diego/INS51/2009(H1N1)
HA	CY057614	H1N1	10/12/2009	USA	A/Wisconsin/629-S1309/2009(H1N1)
HA	CY056883	H1N1	10/12/2009	USA	A/San Diego/INS54/2009(H1N1)
HA	CY057230	H1N1	10/12/2009	USA	A/New York/5045/2009(H1N1)
HA	CY055980	H1N1	10/12/2009	USA	A/San Diego/INS05/2009(H1N1)
HA	CY070480	H1N1	10/12/2009	United Ki	A/Scotland/35/2009
HA	CY070486	H1N1	10/12/2009	United Ki	A/England/94300096/2009
HA	CY056204	H1N1	10/12/2009	USA	A/San Diego/INS52/2009(H1N1)
HA	KC780765	H1N1	10/12/2009	USA	A/Washington/70/2009
HA	HQ228116	H1N1	10/12/2009	Finland	A/Finland/674/2009
HA	KC781096	H1N1	10/12/2009	USA	A/Texas/63/2009

HA	KT181117	H1N1	10/12/2009	Singapore	A/Singapore/DMS43/2009
HA	CY070467	H1N1	10/12/2009	United Ki	A/England/1067/2009
HA	CY070491	H1N1	10/12/2009	United Ki	A/England/749/2009
HA	CY083942	H1N1	10/12/2009	USA	A/San Diego/INS194/2009(H1N1)
HA	CY055988	H1N1	10/12/2009	USA	A/San Diego/INS06/2009(H1N1)
HA	CY070471	H1N1	10/12/2009	United Ki	A/England/742/2009
HA	CY063235	H1N1	10/12/2009	USA	A/Wisconsin/629-D02442/2009(H1N1)
HA	CY096011	H1N1	10/12/2009	China	A/Zhejiang/94/2009
HA	CY096030	H1N1	10/12/2009	China	A/Zhejiang/97/2009
HA	CY070478	H1N1	10/12/2009	United Ki	A/Scotland/34/2009
HA	CY123203	H1N1	10/12/2009	Singapore	A/Singapore/GP3991/2009
HA	CY063243	H1N1	10/12/2009	USA	A/Wisconsin/629-D00830/2009(H1N1)
HA	HM568072	H1N1	10/12/2009	United Ki	A/England/H094220579/2009
HA	CY066527	H1N1	10/12/2009	USA	A/San Diego/INS193/2009(H1N1)
HA	KM289093	H1N1	10/12/2009	Peru	A/Peru/2010731297/2009
HA	CY095992	H1N1	10/12/2009	China	A/Zhejiang/91/2009
HA	CY070481	H1N1	10/13/2009	United Ki	A/England/739/2009
HA	CY123804	H1N1	10/13/2009	Singapore	A/Singapore/ON2202/2009
HA	CY070489	H1N1	10/13/2009	United Ki	A/England/750/2009
HA	CY056491	H1N1	10/13/2009	USA	A/New York/5074/2009(H1N1)
HA	CY056507	H1N1	10/13/2009	USA	A/New York/5083/2009(H1N1)
HA	CY056523	H1N1	10/13/2009	USA	A/New York/5130/2009(H1N1)
HA	CY056515	H1N1	10/13/2009	USA	A/New York/5086/2009(H1N1)
HA	CY090073	H1N1	10/13/2009	Nicaragua	A/Managua/4076.03/2009
HA	AB558334	H1N1	10/13/2009	Japan	A/Sendai/TU439/2009
HA	CY071610	H1N1	10/13/2009	USA	A/Ft Carson/WR1448P/2009(H1N1)
HA	CY055996	H1N1	10/13/2009	USA	A/San Diego/INS09/2009(H1N1)
HA	CY083830	H1N1	10/13/2009	USA	A/San Diego/INS07/2009(H1N1)
HA	CY057318	H1N1	10/13/2009	USA	A/New York/5667/2009(H1N1)
HA	CY070495	H1N1	10/13/2009	United Ki	A/England/94320741/2009
HA	CY070479	H1N1	10/13/2009	United Ki	A/Scotland/94280017/2009
HA	HQ228096	H1N1	10/13/2009	Finland	A/Finland/653/2009
HA	CY057334	H1N1	10/13/2009	USA	A/San Diego/INS08/2009(H1N1)
HA	CY056004	H1N1	10/13/2009	USA	A/San Diego/INS10/2009(H1N1)
HA	CY056499	H1N1	10/13/2009	USA	A/New York/5079/2009(H1N1)
HA	CY070483	H1N1	10/13/2009	United Ki	A/England/1073/2009
HA	CY123213	H1N1	10/13/2009	Singapore	A/Singapore/GP4007/2009
HA	AB576885	H1N1	10/14/2009	Japan	A/Hokkaido/256/2009(H1N1)
HA	CY083495	H1N1	10/14/2009	USA	A/Chicago/WRAIR1691P/2009
HA	HQ228102	H1N1	10/14/2009	Finland	A/Finland/659/2009
HA	CY070512	H1N1	10/14/2009	United Ki	A/England/94340816/2009
HA	CY070485	H1N1	10/14/2009	United Ki	A/England/94300093/2009
HA	CY056891	H1N1	10/14/2009	USA	A/San Diego/INS55/2009(H1N1)
HA	CY057630	H1N1	10/14/2009	USA	A/Wisconsin/629-S1348/2009(H1N1)
HA	CY062407	H1N1	10/14/2009	Afghanist	A/Afghanistan/N11219/2009(H1N1)
HA	KC780743	H1N1	10/14/2009	USA	A/Utah/32/2009
HA	KC780756	H1N1	10/14/2009	USA	A/Michigan/25/2009
HA	KC782083	H1N1	10/14/2009	USA	A/Kansas/24/2009
HA	JN600356	H1N1	10/14/2009	India	A/Assam/2220/2009
HA	KT181118	H1N1	10/14/2009	Singapore	A/Singapore/DMS44/2009
HA	CY081526	H1N1	10/14/2009	Cambodia	A/Cambodia/NHRCC00006/2009(H1N1)
HA	CY056012	H1N1	10/14/2009	USA	A/San Diego/INS11/2009(H1N1)
HA	CY081502	H1N1	10/14/2009	Cambodia	A/Cambodia/NHRCC00003/2009(H1N1)
HA	CY052015	H1N1	10/14/2009	Norway	A/Norway/4023/2009(H1N1)

HA	CY061227	H1N1	10/14/2009	USA	A/San Diego/INS101/2009(H1N1)
HA	JN935017	H1N1	10/14/2009	Viet Nam	A/Hue/7/2009
HA	JN896300	H1N1	10/14/2009	Viet Nam	A/Hue/6/2009
HA	JN788951	H1N1	10/14/2009	USA	A/Wisconsin/UW-1/2009
HA	KC782073	H1N1	10/14/2009	USA	A/Washington/68/2009
HA	CY070482	H1N1	10/14/2009	United Ki	A/England/94280027/2009
HA	CY070513	H1N1	10/14/2009	United Ki	A/England/796/2009
HA	KC780549	H1N1	10/14/2009	USA	A/Delaware/20/2009
HA	CY123218	H1N1	10/14/2009	Singapore	A/Singapore/GP4012/2009
HA	CY081494	H1N1	10/14/2009	Cambodia	A/Cambodia/NHRCC00002/2009(H1N1)
HA	CY081510	H1N1	10/14/2009	Cambodia	A/Cambodia/NHRCC00004/2009(H1N1)
HA	CY081534	H1N1	10/14/2009	Cambodia	A/Cambodia/NHRCC00007/2009(H1N1)
HA	AB558331	H1N1	10/14/2009	Japan	A/Sendai/TU436/2009
HA	CY083479	H1N1	10/14/2009	USA	A/Chicago/WRAIR1690P/2009
HA	CY057638	H1N1	10/14/2009	USA	A/Wisconsin/629-S1350/2009(H1N1)
HA	AB558327	H1N1	10/14/2009	Japan	A/Sendai/TU430/2009
HA	CY090008	H1N1	10/14/2009	USA	A/Wisconsin/629-S1368/2009(H1N1)
HA	CY081542	H1N1	10/14/2009	Cambodia	A/Cambodia/NHRCC00008/2009(H1N1)
HA	CY081486	H1N1	10/14/2009	Cambodia	A/Cambodia/NHRCC00001/2009(H1N1)
HA	CY081518	H1N1	10/14/2009	Cambodia	A/Cambodia/NHRCC00005/2009(H1N1)
HA	CY056020	H1N1	10/14/2009	USA	A/San Diego/INS12/2009(H1N1)
HA	CY052010	H1N1	10/14/2009	Norway	A/Norway/3779/2009(H1N1)
HA	CY061235	H1N1	10/14/2009	USA	A/San Diego/INS102/2009(H1N1)
HA	KC780759	H1N1	10/15/2009	USA	A/Minnesota/17/2009
HA	CY092920	H1N1	10/15/2009	USA	A/Maryland/NHRC0002/2009
HA	AB558328	H1N1	10/15/2009	Japan	A/Sendai/TU431/2009
HA	KC780246	H1N1	10/15/2009	USA	A/New York/100/2009
HA	AB558335	H1N1	10/15/2009	Japan	A/Sendai/TU440/2009
HA	CY075891	H1N1	10/15/2009	India	A/Kol/NIV105777/2009
HA	CY056076	H1N1	10/15/2009	USA	A/District of Columbia/INS24/2009(H
HA	CY081550	H1N1	10/15/2009	Cambodia	A/Cambodia/NHRCC00010/2009(H1N1)
HA	CY082984	H1N1	10/15/2009	Cambodia	A/Cambodia/NHRCC00009/2009(H1N1)
HA	CY080344	H1N1	10/15/2009	Cambodia	A/Cambodia/NHRCC00011/2009(H1N1)
HA	KC782056	H1N1	10/15/2009	USA	A/Arizona/17/2009
HA	CY070493	H1N1	10/15/2009	United Ki	A/England/743/2009
HA	CY071386	H1N1	10/15/2009	Jordan	A/Amman/WR0060N/2009(H1N1)
HA	KC780340	H1N1	10/15/2009	USA	A/New York/102/2009
HA	CY070514	H1N1	10/15/2009	United Ki	A/England/94340856/2009
HA	CY070503	H1N1	10/15/2009	United Ki	A/England/748/2009
HA	CY070498	H1N1	10/15/2009	United Ki	A/Scotland/36/2009
HA	CY071602	H1N1	10/15/2009	USA	A/Ft Carson/WR1446P/2009(H1N1)
HA	KC781147	H1N1	10/15/2009	USA	A/Washington/61/2009
HA	JX625568	H1N1	10/15/2009	United Ki	A/Northern Ireland/1/2009
HA	CY057238	H1N1	10/15/2009	USA	A/New York/5158/2009(H1N1)
HA	CY066535	H1N1	10/15/2009	USA	A/San Diego/INS195/2009(H1N1)
HA	HM072418	H1N1	10/15/2009	Taiwan	A/Taiwan/7855/2009
HA	CY070488	H1N1	10/15/2009	United Ki	A/England/751/2009
HA	CY056899	H1N1	10/15/2009	USA	A/San Diego/INS57/2009(H1N1)
HA	CY063251	H1N1	10/15/2009	USA	A/Wisconsin/629-D00692/2009(H1N1)
HA	CY070499	H1N1	10/15/2009	United Ki	A/England/764/2009
HA	KC781969	H1N1	10/15/2009	USA	A/Mississippi/04/2009
HA	KC780252	H1N1	10/16/2009	USA	A/North Carolina/49/2009
HA	KC781025	H1N1	10/16/2009	USA	A/North Carolina/43/2009
HA	CY092928	H1N1	10/16/2009	USA	A/Maryland/NHRC0003/2009

HA	CY070504	H1N1	10/16/2009	United Ki	A/England/759/2009
HA	CY070516	H1N1	10/16/2009	United Ki	A/England/94360579/2009
HA	CY070497	H1N1	10/16/2009	United Ki	A/Scotland/37/2009
HA	KC781222	H1N1	10/16/2009	USA	A/New Hampshire/19/2009
HA	CY070521	H1N1	10/16/2009	United Ki	A/Scotland/40/2009
HA	CY056539	H1N1	10/16/2009	USA	A/New York/5150/2009(H1N1)
HA	CY063259	H1N1	10/16/2009	USA	A/Wisconsin/629-D00740/2009(H1N1)
HA	JN896302	H1N1	10/16/2009	Viet Nam	A/Hue/10/2009
HA	CY057246	H1N1	10/16/2009	USA	A/New York/5161/2009(H1N1)
HA	CY123223	H1N1	10/16/2009	Singapore	A/Singapore/GP4049/2009
HA	KC780510	H1N1	10/16/2009	USA	A/North Carolina/46/2009
HA	CY062405	H1N1	10/16/2009	Afghanist	A/Afghanistan/N11215/2009(H1N1)
HA	CY056531	H1N1	10/16/2009	USA	A/New York/5141/2009(H1N1)
HA	CY056643	H1N1	10/16/2009	USA	A/New York/6079/2009(H1N1)
HA	AB558329	H1N1	10/16/2009	Japan	A/Sendai/TU432/2009
HA	CY062406	H1N1	10/16/2009	Afghanist	A/Afghanistan/N11216/2009(H1N1)
HA	CY073049	H1N1	10/16/2009	Dominican	A/Santo Domingo/WRAIR1414N/2009(H1N
HA	HQ228073	H1N1	10/16/2009	Finland	A/Finland/624/2009
HA	CY070500	H1N1	10/16/2009	United Ki	A/England/763/2009
HA	JN171931	H1N1	10/16/2009	Canada	A/Montreal/MTL244624/2009
HA	AB558336	H1N1	10/16/2009	Japan	A/Sendai/TU441/2009
HA	CY070496	H1N1	10/16/2009	United Ki	A/Scotland/38/2009
HA	CY070494	H1N1	10/16/2009	United Ki	A/England/755/2009
HA	CY070490	H1N1	10/16/2009	United Ki	A/England/1081/2009
HA	CY070533	H1N1	10/16/2009	United Ki	A/England/94420095/2009
HA	KC781681	H1N1	10/16/2009	USA	A/Arizona/18/2009
HA	KC782327	H1N1	10/16/2009	USA	A/North Carolina/46/2009
HA	CY063083	H1N1	10/17/2009	USA	A/California/VRDL89/2009(H1N1)
HA	CY063107	H1N1	10/17/2009	USA	A/California/VRDL92/2009(H1N1)
HA	CY070492	H1N1	10/17/2009	United Ki	A/England/745/2009
HA	CY070506	H1N1	10/17/2009	United Ki	A/England/94340249/2009
HA	CY107347	H1N1	10/17/2009	United Ki	A/Scotland/StAndrews_09V500252/2009
HA	CY058046	H1N1	10/17/2009	USA	A/Texas/46172734/2009(H1N1)
HA	CY058038	H1N1	10/17/2009	USA	A/Texas/46172731/2009(H1N1)
HA	CY063091	H1N1	10/17/2009	USA	A/California/VRDL90/2009(H1N1)
HA	CY063115	H1N1	10/17/2009	USA	A/California/VRDL93/2009(H1N1)
HA	GU198201	H1N1	10/17/2009	China	A/Nanjing/3/2009(H1N1)
HA	KC781131	H1N1	10/17/2009	USA	A/Nevada/17/2009
HA	CY107361	H1N1	10/17/2009	United Ki	A/Scotland/Perth_09V500259/2009
HA	CY057059	H1N1	10/17/2009	United Ki	A/England/94760028/2009
HA	CY056547	H1N1	10/17/2009	USA	A/New York/5173/2009(H1N1)
HA	CY070502	H1N1	10/17/2009	United Ki	A/England/760/2009
HA	HM780477	H1N1	10/17/2009	China	A/Guangdong/0752/2009(H1N1)
HA	CY070515	H1N1	10/17/2009	United Ki	A/England/94360008/2009
HA	CY070519	H1N1	10/17/2009	United Ki	A/Scotland/94380033/2009
HA	CY107354	H1N1	10/17/2009	United Ki	A/Scotland/Perth_09V500257/2009
HA	CY063099	H1N1	10/17/2009	USA	A/California/VRDL91/2009(H1N1)
HA	KC781670	H1N1	10/17/2009	USA	A/Arkansas/06/2009
HA	CY057646	H1N1	10/18/2009	USA	A/Wisconsin/629-D02342/2009(H1N1)
HA	CY058062	H1N1	10/18/2009	USA	A/Texas/46181292/2009(H1N1)
HA	CY070508	H1N1	10/18/2009	United Ki	A/England/762/2009
HA	CY070518	H1N1	10/18/2009	United Ki	A/England/94360601/2009
HA	CY071554	H1N1	10/18/2009	Jordan	A/Amman/WR1324N/2009(H1N1)
HA	CY075500	H1N1	10/18/2009	USA	A/Boston/591/2009(H1N1)

HA	CY128283	H1N1	10/18/2009	Viet Nam	A/Viet Nam/13032071/2009
HA	CY057662	H1N1	10/18/2009	USA	A/Wisconsin/629-D00099/2009(H1N1)
HA	CY058684	H1N1	10/18/2009	USA	A/Texas/46182018/2009(H1N1)
HA	CY073041	H1N1	10/18/2009	Jordan	A/Amman/WRAIR1339N/2009(H1N1)
HA	CY058054	H1N1	10/18/2009	USA	A/Texas/46181235/2009(H1N1)
HA	CY070509	H1N1	10/18/2009	United Ki	A/England/761/2009
HA	CY060566	H1N1	10/18/2009	Canada	A/Ontario/296008/2009
HA	KC780115	H1N1	10/18/2009	USA	A/New Mexico/07/2009
HA	CY070505	H1N1	10/18/2009	United Ki	A/England/1002/2009
HA	CY083160	H1N1	10/18/2009	Dominicar	A/Santo Domingo/WRAIR1404T/2009
HA	KC781125	H1N1	10/18/2009	USA	A/North Carolina/53/2009
HA	CY057654	H1N1	10/18/2009	USA	A/Wisconsin/629-D00544/2009(H1N1)
HA	CY057686	H1N1	10/19/2009	USA	A/Wisconsin/629-S1388/2009(H1N1)
HA	CY052012	H1N1	10/19/2009	Norway	A/Norway/3855/2009(H1N1)
HA	CY063267	H1N1	10/19/2009	USA	A/Wisconsin/629-D02082/2009(H1N1)
HA	KC780516	H1N1	10/19/2009	USA	A/Delaware/19/2009
HA	CY063805	H1N1	10/19/2009	Singapore	A/Singapore/ON2230/2009
HA	HQ595285	H1N1	10/19/2009	Brazil	A/Sao Paulo/89876/2009
HA	CY058708	H1N1	10/19/2009	USA	A/Texas/46193311/2009(H1N1)
HA	AB558349	H1N1	10/19/2009	Japan	A/Sendai/TU459/2009
HA	AB558338	H1N1	10/19/2009	Japan	A/Sendai/TU447/2009
HA	AB558339	H1N1	10/19/2009	Japan	A/Sendai/TU444/2009
HA	AB558337	H1N1	10/19/2009	Japan	A/Sendai/TU443/2009
HA	CY057064	H1N1	10/19/2009	United Ki	A/England/94780023/2009
HA	KJ690456	H1N1	10/19/2009	Uganda	A/Uganda/MUWRP-111/2009
HA	CY071594	H1N1	10/19/2009	Turkey	A/Ankara/WR1429T/2009(H1N1)
HA	KC780787	H1N1	10/19/2009	USA	A/Nevada/18/2009
HA	CY060574	H1N1	10/19/2009	Canada	A/Ontario/29801/2009
HA	CY057678	H1N1	10/19/2009	USA	A/Wisconsin/629-S1384/2009(H1N1)
HA	JX403978	H1N1	10/19/2009	China	A/Liaoning/1/2009(H1N1)
HA	CY056060	H1N1	10/19/2009	USA	A/District of Columbia/INS20/2009(H
HA	AB558341	H1N1	10/19/2009	Japan	A/Sendai/TU446/2009
HA	CY057670	H1N1	10/19/2009	USA	A/Wisconsin/629-D00132/2009(H1N1)
HA	CY058692	H1N1	10/19/2009	USA	A/Texas/461917783/2009(H1N1)
HA	CY071642	H1N1	10/19/2009	Taiwan	A/Taipei/WR1489N/2009(H1N1)
HA	CY057254	H1N1	10/19/2009	USA	A/New York/5186/2009(H1N1)
HA	JN171932	H1N1	10/19/2009	Canada	A/Montreal/MTL246716/2009
HA	CY083838	H1N1	10/19/2009	USA	A/San Diego/INS14/2009(H1N1)
HA	CY083639	H1N1	10/19/2009	USA	A/San Diego/INS13/2009
HA	AB558348	H1N1	10/19/2009	Japan	A/Sendai/TU458/2009
HA	AB558340	H1N1	10/19/2009	Japan	A/Sendai/TU445/2009
HA	CY107368	H1N1	10/19/2009	United Ki	A/Scotland/Dundee_09V500266/2009
HA	CY070517	H1N1	10/19/2009	United Ki	A/England/772/2009
HA	CY070520	H1N1	10/19/2009	United Ki	A/Scotland/39/2009
HA	CY070507	H1N1	10/19/2009	United Ki	A/England/94340253/2009
HA	CY056907	H1N1	10/19/2009	USA	A/San Diego/INS60/2009(H1N1)
HA	CY058700	H1N1	10/19/2009	USA	A/Texas/46192760/2009(H1N1)
HA	CY060755	H1N1	10/19/2009	USA	A/Texas/46193632/2009(H1N1)
HA	CY061243	H1N1	10/19/2009	USA	A/San Diego/INS103/2009(H1N1)
HA	HQ228099	H1N1	10/20/2009	Finland	A/Finland/656/2009
HA	CY070524	H1N1	10/20/2009	United Ki	A/England/94400123/2009
HA	CY056859	H1N1	10/20/2009	USA	A/San Diego/INS15/2009(H1N1)
HA	CY057278	H1N1	10/20/2009	USA	A/New York/5221/2009(H1N1)
HA	CY064458	H1N1	10/20/2009	China	A/Guangdong/5024/2009(H1N1)

HA	CY056555	H1N1	10/20/2009	USA	A/New York/5199/2009(H1N1)
HA	CY056028	H1N1	10/20/2009	USA	A/San Diego/INS16/2009(H1N1)
HA	CY060763	H1N1	10/20/2009	USA	A/Texas/46201823/2009(H1N1)
HA	CY070523	H1N1	10/20/2009	United Ki	A/England/94400003/2009
HA	KM203129	H1N1	10/20/2009	India	A/Kerala/RGCBB360/2009
HA	KR861654	H1N1	10/20/2009	India	A/Bangalore/8973-04/2009
HA	CY070522	H1N1	10/20/2009	United Ki	A/England/94380515/2009
HA	HM568048	H1N1	10/20/2009	United Ki	A/England/950/2009
HA	CY056563	H1N1	10/20/2009	USA	A/New York/5227/2009(H1N1)
HA	KC781068	H1N1	10/20/2009	USA	A/Minnesota/23/2009
HA	HQ595286	H1N1	10/20/2009	Brazil	A/Sao Paulo/90572/2009
HA	CY123225	H1N1	10/20/2009	Singapore	A/Singapore/GP4078/2009
HA	CY107520	H1N1	10/20/2009	United Ki	A/Scotland/Edinburgh_20665/2009
HA	AB558350	H1N1	10/20/2009	Japan	A/Sendai/TU460/2009
HA	AB558333	H1N1	10/20/2009	Japan	A/Sendai/TU438/2009
HA	CY057270	H1N1	10/20/2009	USA	A/New York/5217/2009(H1N1)
HA	CY123809	H1N1	10/20/2009	Singapore	A/Singapore/ON2224/2009
HA	CY107375	H1N1	10/20/2009	United Ki	A/Scotland/Dundee_09V500321/2009
HA	CY120922	H1N1	10/20/2009	China	A/Guangdong/1201/2009
HA	JX625622	H1N1	10/20/2009	United Ki	A/Northern Ireland/94940033/2009(H1N
HA	JX625591	H1N1	10/20/2009	United Ki	A/Northern Ireland/94620068/2009(H1N
HA	AB558342	H1N1	10/20/2009	Japan	A/Sendai/TU450/2009
HA	CY073072	H1N1	10/20/2009	Turkey	A/Ankara/WRAIR1426N/2009(H1N1)
HA	CY061251	H1N1	10/20/2009	USA	A/San Diego/INS104/2009(H1N1)
HA	KR861655	H1N1	10/20/2009	India	A/Bangalore/8975-19/2009
HA	HQ228098	H1N1	10/20/2009	Finland	A/Finland/655/2009
HA	CY057262	H1N1	10/20/2009	USA	A/New York/5196/2009(H1N1)
HA	HQ228101	H1N1	10/20/2009	Finland	A/Finland/658/2009
HA	JN171890	H1N1	10/21/2009	Canada	A/Quebec/QC152267/2009
HA	JN393307	H1N1	10/21/2009	Viet Nam	A/Hue/15/2009
HA	JN171933	H1N1	10/21/2009	Canada	A/Montreal/MTL250022/2009
HA	CY057294	H1N1	10/21/2009	USA	A/New York/5276/2009(H1N1)
HA	HQ228100	H1N1	10/21/2009	Finland	A/Finland/657/2009
HA	CY071650	H1N1	10/21/2009	Ecuador	A/Quito/WR1589N/2009(H1N1)
HA	CY073080	H1N1	10/21/2009	Turkey	A/Ankara/WRAIR1428T/2009(H1N1)
HA	CY071626	H1N1	10/21/2009	Taiwan	A/Taipei/WR1472T/2009(H1N1)
HA	AB558402	H1N1	10/21/2009	Japan	A/Sendai/TU1001/2009
HA	AB558351	H1N1	10/21/2009	Japan	A/Sendai/TU461/2009
HA	CY066551	H1N1	10/21/2009	USA	A/San Diego/INS198/2009(H1N1)
HA	CY057694	H1N1	10/21/2009	USA	A/Wisconsin/629-S1398/2009(H1N1)
HA	CY070528	H1N1	10/21/2009	United Ki	A/Scotland/94420029/2009
HA	CY063807	H1N1	10/21/2009	Singapore	A/Singapore/ON2241/2009
HA	HM567976	H1N1	10/21/2009	United Ki	A/England/1111/2009
HA	CY060771	H1N1	10/21/2009	USA	A/Texas/46214103/2009(H1N1)
HA	HM568088	H1N1	10/21/2009	United Ki	A/England/H094380096/2009
HA	CY083669	H1N1	10/21/2009	USA	A/San Diego/INS62/2009(H1N1)
HA	KC780885	H1N1	10/21/2009	USA	A/North Carolina/38/2009
HA	KC781003	H1N1	10/21/2009	USA	A/North Carolina/38/2009
HA	KC780121	H1N1	10/21/2009	USA	A/Pennsylvania/27/2009
HA	KC780784	H1N1	10/21/2009	USA	A/South Dakota/13/2009
HA	KC780915	H1N1	10/21/2009	USA	A/New Mexico/12/2009
HA	AB558345	H1N1	10/21/2009	Japan	A/Sendai/TU453/2009
HA	AB558346	H1N1	10/21/2009	Japan	A/Sendai/TU454/2009
HA	HM568056	H1N1	10/21/2009	United Ki	A/England/1116/2009

HA	HM568096	H1N1	10/21/2009	United Ki	A/England/766/2009
HA	CY066543	H1N1	10/21/2009	USA	A/San Diego/INS197/2009(H1N1)
HA	AB558343	H1N1	10/21/2009	Japan	A/Sendai/TU451/2009
HA	CY056132	H1N1	10/21/2009	USA	A/San Diego/INS34/2009(H1N1)
HA	CY057702	H1N1	10/21/2009	USA	A/Wisconsin/629-S1397/2009(H1N1)
HA	AB558344	H1N1	10/21/2009	Japan	A/Sendai/TU452/2009
HA	AB558347	H1N1	10/21/2009	Japan	A/Sendai/TU455/2009
HA	CY056915	H1N1	10/21/2009	USA	A/San Diego/INS61/2009(H1N1)
HA	KC780937	H1N1	10/21/2009	USA	A/Louisiana/09/2009
HA	CY071634	H1N1	10/21/2009	Taiwan	A/Taipei/WR1485T/2009(H1N1)
HA	CY070527	H1N1	10/21/2009	United Ki	A/England/94400574/2009
HA	JX625614	H1N1	10/21/2009	United Ki	A/Northern Ireland/94940023/2009(H1N
HA	KC782152	H1N1	10/21/2009	USA	A/New Mexico/10/2009
HA	CY056092	H1N1	10/21/2009	USA	A/District of Columbia/INS27/2009(H
HA	HQ228103	H1N1	10/21/2009	Finland	A/Finland/660/2009
HA	CY089195	H1N1	10/22/2009	USA	A/Boston/593/2009(H1N1)
HA	CY057302	H1N1	10/22/2009	USA	A/New York/5297/2009(H1N1)
HA	CY071458	H1N1	10/22/2009	Mexico	A/Mexico City/WR1090N/2009(H1N1)
HA	CY065987	H1N1	10/22/2009	Mongolia	A/Ulaanbaatar/6525/2009(H1N1)
HA	AB558403	H1N1	10/22/2009	Japan	A/Sendai/TU1003/2009
HA	AB539046	H1N1	10/22/2009	Japan	A/Nagasaki/HA-56/2009(H1N1)
HA	CY070530	H1N1	10/22/2009	United Ki	A/Scotland/94420090/2009
HA	CY057710	H1N1	10/22/2009	USA	A/Wisconsin/629-S1407/2009(H1N1)
HA	CY057060	H1N1	10/22/2009	United Ki	A/England/94780010/2009
HA	CY060795	H1N1	10/22/2009	USA	A/Texas/46223444/2009(H1N1)
HA	HM568112	H1N1	10/22/2009	United Ki	A/England/798/2009
HA	CY060803	H1N1	10/22/2009	USA	A/Texas/46223582/2009(H1N1)
HA	CY060811	H1N1	10/22/2009	USA	A/Texas/46224042/2009(H1N1)
HA	HQ263268	H1N1	10/22/2009	USA	A/Bethesda/NIH106-D0/2009
HA	JQ065172	H1N1	10/22/2009	China	A/Shenzhen/980/2009
HA	JQ065165	H1N1	10/22/2009	China	A/Shenzhen/860/2009
HA	KC780683	H1N1	10/22/2009	USA	A/Ohio/16/2009
HA	JQ065160	H1N1	10/22/2009	China	A/Shenzhen/49-SZCDC/2009
HA	JQ065159	H1N1	10/22/2009	China	A/Shenzhen/48-SZCDC/2009
HA	JQ065175	H1N1	10/22/2009	China	A/Shenzhen/1009/2009
HA	JQ065174	H1N1	10/22/2009	China	A/Shenzhen/1006/2009
HA	JQ065166	H1N1	10/22/2009	China	A/Shenzhen/899/2009
HA	JQ065161	H1N1	10/22/2009	China	A/Shenzhen/50/2009
HA	JQ065180	H1N1	10/22/2009	China	A/Shenzhen/1132/2009
HA	JQ065184	H1N1	10/22/2009	China	A/Shenzhen/1230/2009
HA	JQ065163	H1N1	10/22/2009	China	A/Shenzhen/833/2009
HA	JQ065181	H1N1	10/22/2009	China	A/Shenzhen/1138/2009
HA	JQ065179	H1N1	10/22/2009	China	A/Shenzhen/1060/2009
HA	JQ065168	H1N1	10/22/2009	China	A/Shenzhen/921/2009
HA	JQ065183	H1N1	10/22/2009	China	A/Shenzhen/1167/2009
HA	JQ065171	H1N1	10/22/2009	China	A/Shenzhen/959/2009
HA	CY070526	H1N1	10/22/2009	United Ki	A/England/94400248/2009
HA	CY070525	H1N1	10/22/2009	United Ki	A/England/765/2009
HA	AB558355	H1N1	10/22/2009	Japan	A/Sendai/TU466/2009
HA	AB558352	H1N1	10/22/2009	Japan	A/Sendai/TU462/2009
HA	AB558354	H1N1	10/22/2009	Japan	A/Sendai/TU463/2009
HA	AB558363	H1N1	10/22/2009	Japan	A/Sendai/TU489/2009
HA	JQ065157	H1N1	10/22/2009	China	A/Shenzhen/46/2009
HA	CY066559	H1N1	10/22/2009	USA	A/San Diego/INS199/2009(H1N1)

HA	JQ065176	H1N1	10/22/2009	China	A/Shenzhen/1042/2009
HA	JQ065178	H1N1	10/22/2009	China	A/Shenzhen/1057/2009
HA	JQ065158	H1N1	10/22/2009	China	A/Shenzhen/47-SZCDC/2009
HA	JQ065169	H1N1	10/22/2009	China	A/Shenzhen/929/2009
HA	JQ065173	H1N1	10/22/2009	China	A/Shenzhen/981/2009
HA	JQ065156	H1N1	10/22/2009	China	A/Shenzhen/45-SZCDC/2009
HA	CY060779	H1N1	10/22/2009	USA	A/Texas/46221665/2009(H1N1)
HA	CY057286	H1N1	10/22/2009	USA	A/New York/5271/2009(H1N1)
HA	JQ065164	H1N1	10/22/2009	China	A/Shenzhen/853/2009
HA	CY089203	H1N1	10/22/2009	USA	A/Boston/594/2009(H1N1)
HA	CY057068	H1N1	10/22/2009	United Ki	A/England/94780031/2009
HA	JQ065167	H1N1	10/22/2009	China	A/Shenzhen/907/2009
HA	JQ065177	H1N1	10/22/2009	China	A/Shenzhen/1045/2009
HA	JQ065182	H1N1	10/22/2009	China	A/Shenzhen/1162/2009
HA	HQ228124	H1N1	10/22/2009	Finland	A/Finland/685/2009
HA	KC780825	H1N1	10/22/2009	USA	A/New Mexico/13/2009
HA	KP019829	H1N1	10/22/2009	China	A/FuZhou/SWL11704/2009
HA	CY060787	H1N1	10/22/2009	USA	A/Texas/46222134/2009(H1N1)
HA	JQ065170	H1N1	10/22/2009	China	A/Shenzhen/956/2009
HA	JQ065162	H1N1	10/22/2009	China	A/Shenzhen/786/2009
HA	AB558358	H1N1	10/23/2009	Japan	A/Sendai/TU474/2009
HA	CY057310	H1N1	10/23/2009	USA	A/New York/5447/2009(H1N1)
HA	AB558364	H1N1	10/23/2009	Japan	A/Sendai/TU490/2009
HA	JN171891	H1N1	10/23/2009	Canada	A/Quebec/QC152365/2009
HA	CY070531	H1N1	10/23/2009	United Ki	A/Scotland/94420091/2009
HA	KC781975	H1N1	10/23/2009	USA	A/New Mexico/14/2009
HA	KC780556	H1N1	10/23/2009	USA	A/West Virginia/12/2009
HA	AB558353	H1N1	10/23/2009	Japan	A/Sendai/TU464/2009
HA	HQ595287	H1N1	10/23/2009	Brazil	A/Sao Paulo/91656/2009
HA	CY092936	H1N1	10/23/2009	USA	A/Maryland/NHRC0004/2009
HA	CY056100	H1N1	10/23/2009	USA	A/District of Columbia/INS28/2009(H
HA	AB558356	H1N1	10/23/2009	Japan	A/Sendai/TU467/2009
HA	AB558365	H1N1	10/23/2009	Japan	A/Sendai/TU491/2009
HA	CY128291	H1N1	10/23/2009	Viet Nam	A/Viet Nam/13032074/2009
HA	CY052014	H1N1	10/23/2009	Norway	A/Norway/4021/2009(H1N1)
HA	CY058300	H1N1	10/23/2009	USA	A/Wisconsin/629-D02018/2009(H1N1)
HA	AB558357	H1N1	10/23/2009	Japan	A/Sendai/TU468/2009
HA	CY065864	H1N1	10/23/2009	Netherlar	A/Netherlands/1493b/2009
HA	CY071618	H1N1	10/23/2009	USA	A/Ft Carson/WR1449P/2009(H1N1)
HA	CY060819	H1N1	10/23/2009	USA	A/Texas/46233104/2009(H1N1)
HA	CY070529	H1N1	10/23/2009	United Ki	A/England/778/2009
HA	CY060827	H1N1	10/24/2009	USA	A/Texas/46240925/2009(H1N1)
HA	HM189373	H1N1	10/24/2009	Russia	A/Moscow/CRIE-BVA/2009
HA	CY052013	H1N1	10/24/2009	Norway	A/Norway/4020/2009(H1N1)
HA	CY060582	H1N1	10/24/2009	Canada	A/Ontario/304434/2009
HA	HQ263281	H1N1	10/24/2009	USA	A/Bethesda/NIH107-D0/2009
HA	CY060835	H1N1	10/24/2009	USA	A/Texas/46241654/2009(H1N1)
HA	CY075508	H1N1	10/24/2009	USA	A/Boston/595/2009(H1N1)
HA	CY128299	H1N1	10/24/2009	Viet Nam	A/Viet Nam/13032076/2009
HA	CY070532	H1N1	10/25/2009	United Ki	A/Scotland/42/2009
HA	CY090016	H1N1	10/25/2009	USA	A/Wisconsin/629-D02330/2009(H1N1)
HA	HQ595288	H1N1	10/25/2009	Brazil	A/Sao Paulo/91768/2009
HA	CY092912	H1N1	10/25/2009	USA	A/Maryland/NHRC0001/2009
HA	CY056124	H1N1	10/26/2009	USA	A/District of Columbia/INS33/2009(H

HA	CY056084	H1N1	10/26/2009	USA	A/District of Columbia/INS25/2009(H
HA	CY056108	H1N1	10/26/2009	USA	A/District of Columbia/INS29/2009(H
HA	CY056180	H1N1	10/26/2009	USA	A/District of Columbia/INS43/2009(H
HA	CY064764	H1N1	10/26/2009	USA	A/District of Columbia/INS32/2009(H
HA	AB536768	H1N1	10/26/2009	Japan	A/Nagasaki/HA-57/2009(H1N1)
HA	CY056923	H1N1	10/26/2009	USA	A/San Diego/INS63/2009(H1N1)
HA	CY058324	H1N1	10/26/2009	USA	A/Wisconsin/629-S1416/2009(H1N1)
HA	HQ228106	H1N1	10/26/2009	Finland	A/Finland/663/2009
HA	HQ228104	H1N1	10/26/2009	Finland	A/Finland/661/2009
HA	AB558360	H1N1	10/26/2009	Japan	A/Sendai/TU477/2009
HA	JN171892	H1N1	10/26/2009	Canada	A/Quebec/QC152717/2009
HA	JQ612499	H1N1	10/26/2009	Hungary	A/Hungary/40/2009
HA	CY075006	H1N1	10/26/2009	Thailand	A/Thailand/CU-H847/2009
HA	CY123817	H1N1	10/26/2009	Singapore	A/Singapore/ON2250/2009
HA	HM568104	H1N1	10/26/2009	United Ki	A/England/797/2009
HA	KC780053	H1N1	10/26/2009	USA	A/Washington/75/2009
HA	KC780689	H1N1	10/26/2009	USA	A/Rhode Island/19/2009
HA	CY050844	H1N1	10/26/2009	Mongolia	A/Ulaanbaatar/5882/2009(H1N1)
HA	KR861656	H1N1	10/26/2009	India	A/Bangalore/9274-06/2009
HA	HQ228105	H1N1	10/26/2009	Finland	A/Finland/662/2009
HA	HQ228110	H1N1	10/26/2009	Finland	A/Finland/667/2009
HA	KC780981	H1N1	10/26/2009	USA	A/Iowa/10/2009
HA	CY056140	H1N1	10/26/2009	USA	A/San Diego/INS35/2009(H1N1)
HA	CY057045	H1N1	10/26/2009	United Ki	A/England/94600034/2009
HA	KP019830	H1N1	10/26/2009	China	A/FuZhou/SWL11724/2009
HA	CY075485	H1N1	10/26/2009	Russia	A/Belgorod/RII4/2009(H1N1)
HA	CY070564	H1N1	10/26/2009	United Ki	A/England/94620048/2009
HA	CY056036	H1N1	10/26/2009	USA	A/District of Columbia/INS17/2009(H
HA	AB558359	H1N1	10/26/2009	Japan	A/Sendai/TU476/2009
HA	CY058308	H1N1	10/26/2009	USA	A/Wisconsin/629-S1417/2009(H1N1)
HA	CY058316	H1N1	10/26/2009	USA	A/Wisconsin/629-S1414/2009(H1N1)
HA	JX875039	H1N1	10/26/2009	USA	A/Kentucky/96/2009
HA	KC780967	H1N1	10/26/2009	USA	A/New Mexico/11/2009
HA	CY123230	H1N1	10/27/2009	Singapore	A/Singapore/GP4148/2009
HA	JN171893	H1N1	10/27/2009	Canada	A/Quebec/QC153013/2009
HA	AB558368	H1N1	10/27/2009	Japan	A/Sendai/TU498/2009
HA	HQ228109	H1N1	10/27/2009	Finland	A/Finland/666/2009
HA	HQ228107	H1N1	10/27/2009	Finland	A/Finland/664/2009
HA	AB558367	H1N1	10/27/2009	Japan	A/Sendai/TU497/2009
HA	AB558366	H1N1	10/27/2009	Japan	A/Sendai/TU496/2009
HA	CY058628	H1N1	10/27/2009	USA	A/Wisconsin/629-S1431/2009(H1N1)
HA	CY058636	H1N1	10/27/2009	USA	A/Wisconsin/629-S1425/2009(H1N1)
HA	AB558361	H1N1	10/27/2009	Japan	A/Sendai/TU481/2009
HA	AB536769	H1N1	10/27/2009	Japan	A/Nagasaki/HA-58/2009(H1N1)
HA	AB535746	H1N1	10/27/2009	Japan	A/Nagasaki/HA-59/2009(H1N1)
HA	CY067635	H1N1	10/27/2009	Norway	A/Oslo/INS219/2009(H1N1)
HA	CY057047	H1N1	10/27/2009	United Ki	A/England/94600039/2009
HA	AB535747	H1N1	10/27/2009	Japan	A/Nagasaki/HA-60/2009(H1N1)
HA	CY058620	H1N1	10/27/2009	USA	A/Wisconsin/629-S1433/2009(H1N1)
HA	CY057063	H1N1	10/27/2009	United Ki	A/England/94780022/2009
HA	KC781079	H1N1	10/27/2009	USA	A/Maryland/30/2009
HA	CY060598	H1N1	10/27/2009	Canada	A/Ontario/305598/2009
HA	HQ228108	H1N1	10/27/2009	Finland	A/Finland/665/2009
HA	CY056052	H1N1	10/27/2009	USA	A/District of Columbia/INS19/2009(H

HA	CY060590	H1N1	10/27/2009	Canada	A/Ontario/305139/2009
HA	JN187133	H1N1	10/27/2009	Taiwan	A/Taiwan/6200/2009
HA	KT181119	H1N1	10/27/2009	Singapore	A/Singapore/DMS46/2009
HA	HQ228143	H1N1	10/27/2009	Finland	A/Finland/709/2009
HA	CY056116	H1N1	10/27/2009	USA	A/District of Columbia/INS31/2009(H
HA	KC780722	H1N1	10/27/2009	USA	A/Rhode Island/18/2009
HA	HM780483	H1N1	10/27/2009	China	A/Guangdong/0862/2009(H1N1)
HA	AB558405	H1N1	10/28/2009	Japan	A/Sendai/TU1005/2009
HA	AB558407	H1N1	10/28/2009	Japan	A/Sendai/TU1007/2009
HA	AB535741	H1N1	10/28/2009	Japan	A/Nagasaki/NU-2/2009(H1N1)
HA	CY066823	H1N1	10/28/2009	Belgium	A/Antwerp/INS221/2009(H1N1)
HA	JN171894	H1N1	10/28/2009	Canada	A/Quebec/QC153282/2009
HA	JN187134	H1N1	10/28/2009	Taiwan	A/Taiwan/7873/2009
HA	KC782276	H1N1	10/28/2009	USA	A/Hawaii/34/2009
HA	KP019831	H1N1	10/28/2009	China	A/FuZhou/SWL11782/2009
HA	CY056931	H1N1	10/28/2009	USA	A/San Diego/INS65/2009(H1N1)
HA	CY075487	H1N1	10/28/2009	Russia	A/Saint-Petersburg/RII82/2009(H1N1)
HA	KY925530	H1N1	10/28/2009	Brazil	A/Porto Alegre/LACENRS-3434/2009
HA	CY071426	H1N1	10/28/2009	Turkmenis	A/Ashgabat/WR0865N/2009(H1N1)
HA	AB558362	H1N1	10/28/2009	Japan	A/Sendai/TU483/2009
HA	KU310627	H1N1	10/28/2009	India	A/Assam/2257/2009
HA	HM568080	H1N1	10/28/2009	United Ki	A/England/1213/2009
HA	CY056044	H1N1	10/28/2009	USA	A/District of Columbia/INS18/2009(H
HA	JX625552	H1N1	10/28/2009	United Ki	A/England/1397/2009
HA	KC782164	H1N1	10/28/2009	USA	A/Hawaii/29/2009
HA	CY070410	H1N1	10/28/2009	United Ki	A/England/716/2009
HA	CY060606	H1N1	10/28/2009	Canada	A/Ontario/308054/2009
HA	JN171934	H1N1	10/28/2009	Canada	A/Montreal/MTL256259/2009
HA	CY066815	H1N1	10/28/2009	USA	A/San Diego/INS218/2009
HA	AB558404	H1N1	10/29/2009	Japan	A/Sendai/TU1004/2009
HA	JX875019	H1N1	10/29/2009	USA	A/Kentucky/99/2009
HA	CY060614	H1N1	10/29/2009	Canada	A/Ontario/309862/2009
HA	AB704462	H1N1	10/29/2009	Japan	A/Tochigi/350/2009
HA	KU310626	H1N1	10/29/2009	India	A/Assam/2264/2009
HA	CY075516	H1N1	10/29/2009	USA	A/Boston/602/2009(H1N1)
HA	CY063123	H1N1	10/29/2009	USA	A/California/VRDL96/2009(H1N1)
HA	CY057067	H1N1	10/29/2009	United Ki	A/England/94780029/2009
HA	CY063131	H1N1	10/29/2009	USA	A/California/VRDL97/2009(H1N1)
HA	KC780961	H1N1	10/29/2009	USA	A/Utah/58/2009
HA	CY128307	H1N1	10/29/2009	Viet Nam	A/Viet Nam/13032079/2009
HA	CY050845	H1N1	10/29/2009	Mongolia	A/Ulaanbaatar/6266/2009(H1N1)
HA	CY050846	H1N1	10/29/2009	Mongolia	A/Ulaanbaatar/6133/2009(H1N1)
HA	AB535748	H1N1	10/29/2009	Japan	A/Nagasaki/HA-63/2009(H1N1)
HA	AB536770	H1N1	10/29/2009	Japan	A/Nagasaki/HA-62/2009(H1N1)
HA	CY063139	H1N1	10/29/2009	USA	A/California/VRDL98/2009(H1N1)
HA	CY057066	H1N1	10/29/2009	United Ki	A/England/94780026/2009
HA	CY061267	H1N1	10/29/2009	Belgium	A/Brussels/INS106/2009(H1N1)
HA	CY066191	H1N1	10/29/2009	USA	A/California/VRDL94/2009(H1N1)
HA	CY075488	H1N1	10/29/2009	Russia	A/Saint-Petersburg/RII96/2009(H1N1)
HA	AB558406	H1N1	10/29/2009	Japan	A/Sendai/TU1006/2009
HA	AB558369	H1N1	10/29/2009	Japan	A/Sendai/TU499/2009
HA	AB558370	H1N1	10/29/2009	Japan	A/Sendai/TU502/2009
HA	CY057050	H1N1	10/29/2009	United Ki	A/England/94640079/2009
HA	JN171895	H1N1	10/29/2009	Canada	A/Quebec/QC153408/2009

HA	CY083647	H1N1	10/29/2009	USA	A/District of Columbia/INS22/2009(H
HA	KC781083	H1N1	10/30/2009	USA	A/District Of Columbia/10/2009
HA	JN207837	H1N1	10/30/2009	China	A/Zhejiang/89w/2009(H1N1)
HA	HM072419	H1N1	10/30/2009	Taiwan	A/Taiwan/7878/2009
HA	CY056068	H1N1	10/30/2009	USA	A/District of Columbia/INS21/2009(H
HA	AB558380	H1N1	10/30/2009	Japan	A/Sendai/TU538/2009
HA	CY057048	H1N1	10/30/2009	United Ki	A/England/94640077/2009
HA	JN171896	H1N1	10/30/2009	Canada	A/Quebec/QC153705/2009
HA	AB558378	H1N1	10/30/2009	Japan	A/Sendai/TU531/2009
HA	CY056939	H1N1	10/30/2009	Belgium	A/Brussels/INS71/2009(H1N1)
HA	CY066727	H1N1	10/30/2009	Belgium	A/Brussels/INS206/2009(H1N1)
HA	CY083846	H1N1	10/30/2009	USA	A/District of Columbia/INS23/2009(H
HA	CY083753	H1N1	10/30/2009	Belgium	A/Brussels/INS205/2009(H1N1)
HA	CY070536	H1N1	10/30/2009	United Ki	A/England/94540029/2009
HA	JQ173103	H1N1	10/30/2009	China	A/Nanjing/NJU-108/2009
HA	KC781334	H1N1	10/30/2009	USA	A/Kentucky/25/2009
HA	CY060622	H1N1	10/30/2009	Canada	A/Ontario/313762/2009
HA	CY128315	H1N1	10/31/2009	Viet Nam	A/Viet Nam/13032088/2009
HA	CY058652	H1N1	10/31/2009	USA	A/Wisconsin/629-D02002/2009(H1N1)
HA	CY073118	H1N1	10/31/2009	Russia	A/Moscow/WRAIR1632N/2009(H1N1)
HA	CY058660	H1N1	10/31/2009	USA	A/Wisconsin/629-D01913/2009(H1N1)
HA	CY058644	H1N1	10/31/2009	USA	A/Wisconsin/629-D02329/2009(H1N1)
HA	JN171935	H1N1	10/31/2009	Canada	A/Montreal/MTL260764/2009
HA	CY065808	H1N1	10/31/2009	Netherlar	A/Netherlands/1715b/2009
HA	JN171897	H1N1	10/31/2009	Canada	A/Quebec/QC153774/2009
HA	HM189414	H1N1	10/31/2009	Russia	A/Ulyanovsk/CRIE-SHTA/2009
HA	AB745194	H1N1	11/2009	Japan	A/Tottori/ST331/2009
HA	AB745181	H1N1	11/2009	Japan	A/Tottori/ST317/2009
HA	AB745184	H1N1	11/2009	Japan	A/Tottori/ST321/2009
HA	AB745157	H1N1	11/2009	Japan	A/Tottori/ST284/2009
HA	AB745174	H1N1	11/2009	Japan	A/Tottori/ST307/2009
HA	AB745163	H1N1	11/2009	Japan	A/Tottori/ST291/2009
HA	CY095912	H1N1	11/2009	China	A/Zhejiang/80/2009
HA	CY095920	H1N1	11/2009	China	A/Zhejiang/81/2009
HA	AB745160	H1N1	11/2009	Japan	A/Tottori/ST287/2009
HA	AB745188	H1N1	11/2009	Japan	A/Tottori/ST325/2009
HA	CY095891	H1N1	11/2009	China	A/Zhejiang/78/2009
HA	JQ364996	H1N1	11/2009	China	A/Hangzhou/1718/2009
HA	HM189411	H1N1	11/2009	Russia	A/Moscow oblast/CRIE-DEI/2009
HA	CY067622	H1N1	11/2009	China	A/Qingdao/1631/2009
HA	HM189419	H1N1	11/2009	Russia	A/Kaluga/CRIE-INV/2009
HA	CY060457	H1N1	11/2009	South Koi	A/Seoul/1785/2009(H1N1)
HA	JN381242	H1N1	11/2009	Taiwan	A/Taiwan/99542/2009
HA	AB745147	H1N1	11/2009	Japan	A/Tottori/ST270/2009
HA	AB745151	H1N1	11/2009	Japan	A/Tottori/ST276/2009
HA	JQ364997	H1N1	11/2009	China	A/Hangzhou/1719/2009
HA	AB745158	H1N1	11/2009	Japan	A/Tottori/ST285/2009
HA	AB745193	H1N1	11/2009	Japan	A/Tottori/ST330/2009
HA	AB745141	H1N1	11/2009	Japan	A/Tottori/ST262/2009
HA	AB745143	H1N1	11/2009	Japan	A/Tottori/ST264/2009
HA	AB745167	H1N1	11/2009	Japan	A/Tottori/ST298/2009
HA	AB745179	H1N1	11/2009	Japan	A/Tottori/ST315/2009
HA	AB745176	H1N1	11/2009	Japan	A/Tottori/ST310/2009
HA	HM189422	H1N1	11/2009	Russia	A/Ivanovo/CRIE-AMV/2009

HA	AB745166	H1N1	11/2009	Japan	A/Tottori/ST297/2009
HA	AB745162	H1N1	11/2009	Japan	A/Tottori/ST290/2009
HA	AB745189	H1N1	11/2009	Japan	A/Tottori/ST326/2009
HA	JN381247	H1N1	11/2009	Taiwan	A/Taiwan/99902/2009
HA	CY062366	H1N1	11/2009	Egypt	A/Egypt/N14645/2009(H1N1)
HA	CY067618	H1N1	11/2009	China	A/Qingdao/1609/2009
HA	GU968917	H1N1	11/2009	China	A/Beijing/SE2577/2009(H1N1)
HA	AB745164	H1N1	11/2009	Japan	A/Tottori/ST293/2009
HA	AB745192	H1N1	11/2009	Japan	A/Tottori/ST329/2009
HA	AB745177	H1N1	11/2009	Japan	A/Tottori/ST311/2009
HA	JN381246	H1N1	11/2009	Taiwan	A/Taiwan/99867/2009
HA	JN381244	H1N1	11/2009	Taiwan	A/Taiwan/99721/2009
HA	JN381245	H1N1	11/2009	Taiwan	A/Taiwan/99840/2009
HA	JQ364995	H1N1	11/2009	China	A/Hangzhou/1717/2009
HA	AB745139	H1N1	11/2009	Japan	A/Tottori/ST260/2009
HA	AB745145	H1N1	11/2009	Japan	A/Tottori/ST267/2009
HA	AB745148	H1N1	11/2009	Japan	A/Tottori/ST272/2009
HA	AB745149	H1N1	11/2009	Japan	A/Tottori/ST274/2009
HA	AB745152	H1N1	11/2009	Japan	A/Tottori/ST277/2009
HA	AB745159	H1N1	11/2009	Japan	A/Tottori/ST286/2009
HA	AB745161	H1N1	11/2009	Japan	A/Tottori/ST288/2009
HA	AB745155	H1N1	11/2009	Japan	A/Tottori/ST282/2009
HA	AB745156	H1N1	11/2009	Japan	A/Tottori/ST283/2009
HA	AB745168	H1N1	11/2009	Japan	A/Tottori/ST301/2009
HA	AB745170	H1N1	11/2009	Japan	A/Tottori/ST303/2009
HA	AB745172	H1N1	11/2009	Japan	A/Tottori/ST305/2009
HA	AB745173	H1N1	11/2009	Japan	A/Tottori/ST306/2009
HA	AB745175	H1N1	11/2009	Japan	A/Tottori/ST309/2009
HA	AB745182	H1N1	11/2009	Japan	A/Tottori/ST319/2009
HA	AB745183	H1N1	11/2009	Japan	A/Tottori/ST320/2009
HA	AB745187	H1N1	11/2009	Japan	A/Tottori/ST324/2009
HA	AB745191	H1N1	11/2009	Japan	A/Tottori/ST328/2009
HA	AB745171	H1N1	11/2009	Japan	A/Tottori/ST304/2009
HA	AB745180	H1N1	11/2009	Japan	A/Tottori/ST316/2009
HA	AB745185	H1N1	11/2009	Japan	A/Tottori/ST322/2009
HA	AB745186	H1N1	11/2009	Japan	A/Tottori/ST323/2009
HA	AB745190	H1N1	11/2009	Japan	A/Tottori/ST327/2009
HA	AB745195	H1N1	11/2009	Japan	A/Tottori/ST332/2009
HA	AB745138	H1N1	11/2009	Japan	A/Tottori/ST257/2009
HA	AB745165	H1N1	11/2009	Japan	A/Tottori/ST294/2009
HA	AB745196	H1N1	11/2009	Japan	A/Tottori/ST335/2009
HA	JN381243	H1N1	11/2009	Taiwan	A/Taiwan/99666/2009
HA	GU968919	H1N1	11/2009	China	A/Beijing/SE2733/2009(H1N1)
HA	CY053353	H1N1	11/2009	China	A/Beijing/721/2009(H1N1)
HA	JN381248	H1N1	11/2009	Taiwan	A/Taiwan/97080/2009
HA	JN381249	H1N1	11/2009	Taiwan	A/Taiwan/97138/2009
HA	AB745169	H1N1	11/2009	Japan	A/Tottori/ST302/2009
HA	AB745178	H1N1	11/2009	Japan	A/Tottori/ST312/2009
HA	AB745142	H1N1	11/2009	Japan	A/Tottori/ST263/2009
HA	AB745146	H1N1	11/2009	Japan	A/Tottori/ST269/2009
HA	CY053338	H1N1	11/2009	China	A/Beijing/718/2009(H1N1)
HA	CY053348	H1N1	11/2009	China	A/Beijing/720/2009(H1N1)
HA	CY095885	H1N1	11/2009	China	A/Zhejiang/77/2009
HA	CY095862	H1N1	11/2009	China	A/Hubei/74/2009

HA	CY062367	H1N1	11/2009	Egypt	A/Egypt/N14648/2009(H1N1)
HA	GU968921	H1N1	11/2009	China	A/Beijing/SE3182/2009(H1N1)
HA	GU968918	H1N1	11/2009	China	A/Beijing/SE2649/2009(H1N1)
HA	GU968920	H1N1	11/2009	China	A/Beijing/SE2849/2009(H1N1)
HA	JQ364998	H1N1	11/2009	China	A/Hangzhou/1724/2009
HA	CY067621	H1N1	11/2009	China	A/Qingdao/1629/2009
HA	CY067620	H1N1	11/2009	China	A/Qingdao/1626/2009
HA	CY067623	H1N1	11/2009	China	A/Qingdao/1642/2009
HA	CY067619	H1N1	11/2009	China	A/Qingdao/1610/2009
HA	CY067624	H1N1	11/2009	China	A/Qingdao/1669/2009
HA	CY067616	H1N1	11/2009	China	A/Qingdao/1568/2009
HA	CY067617	H1N1	11/2009	China	A/Qingdao/1577/2009
HA	AB745140	H1N1	11/2009	Japan	A/Tottori/ST261/2009
HA	AB745144	H1N1	11/2009	Japan	A/Tottori/ST265/2009
HA	AB745150	H1N1	11/2009	Japan	A/Tottori/ST275/2009
HA	AB745153	H1N1	11/2009	Japan	A/Tottori/ST279/2009
HA	AB745154	H1N1	11/2009	Japan	A/Tottori/ST281/2009
HA	CY095854	H1N1	11/2009	China	A/Hubei/73/2009
HA	CY095928	H1N1	11/2009	China	A/Zhejiang/82/2009
HA	CY095870	H1N1	11/2009	China	A/Hubei/75/2009
HA	CY095899	H1N1	11/2009	China	A/Zhejiang/79/2009
HA	CY095878	H1N1	11/2009	China	A/Hubei/76/2009
HA	CY053728	H1N1	11/01/2009	Russia	A/Russia/74/2009(H1N1)
HA	JX625576	H1N1	11/01/2009	United Ki	A/England/94580011/2009
HA	CY107732	H1N1	11/01/2009	United Ki	A/Scotland/Glasgow_445236/2009
HA	CY054654	H1N1	11/01/2009	Russia	A/Russia/61/2009(H1N1)
HA	CY054662	H1N1	11/01/2009	Russia	A/Russia/100/2009(H1N1)
HA	CY107705	H1N1	11/01/2009	United Ki	A/Scotland/Glasgow_443734/2009
HA	KC780179	H1N1	11/01/2009	USA	A/Texas/69/2009
HA	KC780652	H1N1	11/01/2009	USA	A/Utah/52/2009
HA	CY107712	H1N1	11/01/2009	United Ki	A/Scotland/Glasgow_444125/2009
HA	CY107720	H1N1	11/01/2009	United Ki	A/Scotland/Glasgow_444590/2009
HA	KC780909	H1N1	11/01/2009	USA	A/New York/104/2009
HA	CY070557	H1N1	11/01/2009	United Ki	A/England/819/2009
HA	CY058668	H1N1	11/01/2009	USA	A/Wisconsin/629-D00193/2009(H1N1)
HA	CY054646	H1N1	11/01/2009	Russia	A/Russia/19/2009(H1N1)
HA	KT181120	H1N1	11/01/2009	Singapore	A/Singapore/DMS47/2009
HA	KC781770	H1N1	11/01/2009	USA	A/Nevada/19/2009
HA	CY053752	H1N1	11/01/2009	Russia	A/Russia/200/2009(H1N1)
HA	CY070535	H1N1	11/01/2009	United Ki	A/England/794/2009
HA	CY054638	H1N1	11/01/2009	Russia	A/Russia/12/2009(H1N1)
HA	KM213258	H1N1	11/01/2009	Oman	A/Oman/SQUH-6/2009
HA	CY053744	H1N1	11/01/2009	Russia	A/Russia/190/2009(H1N1)
HA	CY053736	H1N1	11/01/2009	Russia	A/Russia/178/2009(H1N1)
HA	CY062365	H1N1	11/01/2009	Egypt	A/Egypt/N14644/2009(H1N1)
HA	CY053416	H1N1	11/01/2009	Russia	A/Russia/149/2009(H1N1)
HA	CY054630	H1N1	11/01/2009	Russia	A/Russia/4/2009(H1N1)
HA	CY053689	H1N1	11/01/2009	Russia	A/Russia/191/2009(H1N1)
HA	CY053681	H1N1	11/01/2009	Russia	A/Russia/14/2009(H1N1)
HA	HM189410	H1N1	11/02/2009	Russia	A/Moscow oblast/CRIE-OAM/2009
HA	CY056619	H1N1	11/02/2009	USA	A/New York/5988/2009(H1N1)
HA	HM189312	H1N1	11/02/2009	Russia	A/Chita/CRIE-8/2009
HA	HQ228139	H1N1	11/02/2009	Finland	A/Finland/700/2009
HA	AB558372	H1N1	11/02/2009	Japan	A/Sendai/TU514/2009

HA	CY066567	H1N1	11/02/2009	USA	A/San Diego/INS201/2009(H1N1)
HA	KC781129	H1N1	11/02/2009	USA	A/Alabama/04/2009
HA	CY060670	H1N1	11/02/2009	Canada	A/Ontario/315047/2009
HA	KC781773	H1N1	11/02/2009	USA	A/Hawaii/30/2009
HA	CY107382	H1N1	11/02/2009	United Ki	A/Scotland/Dundee_09V500624/2009
HA	KC780951	H1N1	11/02/2009	USA	A/Delaware/14/2009
HA	KC780857	H1N1	11/02/2009	USA	A/Wyoming/16/2009
HA	JF714142	H1N1	11/02/2009	South Kor	A/Korea/NAP-1/2009(H1N1)
HA	CY060630	H1N1	11/02/2009	Canada	A/Ontario/314095/2009
HA	CY070556	H1N1	11/02/2009	United Ki	A/England/826/2009
HA	CY083661	H1N1	11/02/2009	USA	A/San Diego/INS41/2009(H1N1)
HA	JN171936	H1N1	11/02/2009	Canada	A/Montreal/MTL260102/2009
HA	CY075524	H1N1	11/02/2009	USA	A/Boston/606/2009(H1N1)
HA	CY060646	H1N1	11/02/2009	Canada	A/Ontario/314603/2009
HA	CY057046	H1N1	11/02/2009	United Ki	A/England/94600038/2009
HA	CY057065	H1N1	11/02/2009	United Ki	A/England/94780024/2009
HA	AB558373	H1N1	11/02/2009	Japan	A/Sendai/TU524/2009
HA	CY075475	H1N1	11/02/2009	Russia	A/Saint-Petersburg/RII48/2009(H1N1)
HA	CY060843	H1N1	11/02/2009	USA	A/Texas/JMS356/2009(H1N1)
HA	CY058676	H1N1	11/02/2009	USA	A/Wisconsin/629-D00938/2009(H1N1)
HA	AB558379	H1N1	11/02/2009	Japan	A/Sendai/TU533/2009
HA	CY058268	H1N1	11/02/2009	USA	A/New York/5620/2009(H1N1)
HA	CY063275	H1N1	11/02/2009	USA	A/Wisconsin/629-D00888/2009(H1N1)
HA	CY123238	H1N1	11/02/2009	Singapore	A/Singapore/GP4218/2009
HA	CY070534	H1N1	11/02/2009	United Ki	A/England/801/2009
HA	CY066879	H1N1	11/02/2009	USA	A/District of Columbia/INS229/2009(I
HA	CY075532	H1N1	11/02/2009	USA	A/Boston/612/2009(H1N1)
HA	KC190041	H1N1	11/02/2009	Sri Lanka	A/Sri Lanka/2001/2009
HA	GU931802	H1N1	11/03/2009	Hong Kong	A/Hong Kong/415742M/2009(H1N1)
HA	CY056188	H1N1	11/03/2009	USA	A/District of Columbia/INS46/2009(H
HA	GU323343	H1N1	11/03/2009	China	A/Lishui/02w/2009(H1N1)
HA	CY062643	H1N1	11/03/2009	USA	A/District of Columbia/INS45/2009(H
HA	CY071778	H1N1	11/03/2009	Mexico	A/Mexico City/WR1762T/2009(H1N1)
HA	CY063811	H1N1	11/03/2009	Singapore	A/Singapore/ON2271/2009
HA	CY075477	H1N1	11/03/2009	Russia	A/Saint-Petersburg/RII35/2009(H1N1)
HA	CY056867	H1N1	11/03/2009	USA	A/District of Columbia/INS44/2009(H
HA	HM100229	H1N1	11/03/2009	Hong Kong	A/Hong Kong/415742Md/2009(H1N1)
HA	CY066735	H1N1	11/03/2009	Belgium	A/Brussels/INS208/2009(H1N1)
HA	CY070542	H1N1	11/03/2009	United Ki	A/England/825/2009
HA	CY070544	H1N1	11/03/2009	United Ki	A/England/827/2009
HA	HM072421	H1N1	11/03/2009	Taiwan	A/Taiwan/2202/2009
HA	CY083248	H1N1	11/03/2009	Russia	A/Moscow/WRAIR1627T/2009(H1N1)
HA	CY070545	H1N1	11/03/2009	United Ki	A/England/836/2009
HA	CY070538	H1N1	11/03/2009	United Ki	A/England/809/2009
HA	CY070539	H1N1	11/03/2009	United Ki	A/England/795/2009
HA	CY070543	H1N1	11/03/2009	United Ki	A/England/829/2009
HA	CY089211	H1N1	11/03/2009	USA	A/Boston/618/2009
HA	CY060654	H1N1	11/03/2009	Canada	A/Ontario/315003/2009
HA	CY070547	H1N1	11/03/2009	United Ki	A/England/813/2009
HA	CY070553	H1N1	11/03/2009	United Ki	A/England/837/2009
HA	KC780804	H1N1	11/03/2009	USA	A/Kansas/26/2009
HA	CY060702	H1N1	11/03/2009	Canada	A/Ontario/315613/2009
HA	CY056220	H1N1	11/03/2009	USA	A/San Diego/INS68/2009(H1N1)
HA	CY070551	H1N1	11/03/2009	United Ki	A/England/839/2009

HA	CY064397	H1N1	11/03/2009	China	A/Hualong/SWL1313/2009(H1N1)
HA	CY111050	H1N1	11/03/2009	Taiwan	A/Taiwan/NTU8536/2009
HA	CY066831	H1N1	11/03/2009	Spain	A/Madrid/INS222/2009
HA	KC781268	H1N1	11/03/2009	USA	A/Vermont/23/2009
HA	CY070552	H1N1	11/03/2009	United Ki	A/England/832/2009
HA	CY070562	H1N1	11/03/2009	United Ki	A/England/840/2009
HA	CY070541	H1N1	11/03/2009	United Ki	A/England/822/2009
HA	CY100472	H1N1	11/03/2009	Mexico	A/Mexico City/INER12/2009
HA	CY070549	H1N1	11/03/2009	United Ki	A/England/810/2009
HA	CY060686	H1N1	11/03/2009	Canada	A/Ontario/315181/2009
HA	CY060638	H1N1	11/03/2009	Canada	A/Ontario/314137/2009
HA	CY056579	H1N1	11/03/2009	USA	A/New York/5782/2009(H1N1)
HA	CY070550	H1N1	11/03/2009	United Ki	A/England/838/2009
HA	CY070540	H1N1	11/03/2009	United Ki	A/England/821/2009
HA	CY070554	H1N1	11/03/2009	United Ki	A/England/834/2009
HA	CY070546	H1N1	11/03/2009	United Ki	A/England/814/2009
HA	CY070548	H1N1	11/03/2009	United Ki	A/England/812/2009
HA	CY060694	H1N1	11/03/2009	Canada	A/Ontario/315187/2009
HA	KJ690526	H1N1	11/03/2009	Uganda	A/Uganda/MUWRP-137/2009
HA	CY075652	H1N1	11/03/2009	USA	A/Boston/617/2009(H1N1)
HA	CY103937	H1N1	11/04/2009	Brazil	A/Pernambuco/688/2009
HA	CY066583	H1N1	11/04/2009	USA	A/San Diego/INS203/2009(H1N1)
HA	KC780469	H1N1	11/04/2009	USA	A/Florida/25/2009
HA	CY123243	H1N1	11/04/2009	Singapore	A/Singapore/GP4228/2009
HA	HM189413	H1N1	11/04/2009	Russia	A/Moscow oblast/CRIE-LVS/2009
HA	CY056156	H1N1	11/04/2009	USA	A/Pensacola/INS38/2009(H1N1)
HA	CY056627	H1N1	11/04/2009	USA	A/New York/6018/2009(H1N1)
HA	CY056148	H1N1	11/04/2009	USA	A/Pensacola/INS37/2009(H1N1)
HA	CY056196	H1N1	11/04/2009	USA	A/San Diego/INS48/2009(H1N1)
HA	CY061259	H1N1	11/04/2009	USA	A/San Diego/INS105/2009(H1N1)
HA	AB558371	H1N1	11/04/2009	Japan	A/Sendai/TU512/2009
HA	CY070563	H1N1	11/04/2009	United Ki	A/England/94620043/2009
HA	CY066743	H1N1	11/04/2009	Belgium	A/Brussels/INS209/2009(H1N1)
HA	KM213259	H1N1	11/04/2009	Oman	A/Oman/SQUH-7/2009
HA	KT181121	H1N1	11/04/2009	Singapore	A/Singapore/DMS48/2009
HA	CY060662	H1N1	11/04/2009	Canada	A/Ontario/315015/2009
HA	CY056172	H1N1	11/04/2009	USA	A/San Diego/INS42/2009(H1N1)
HA	CY066575	H1N1	11/04/2009	USA	A/San Diego/INS202/2009(H1N1)
HA	KC780729	H1N1	11/04/2009	USA	A/South Carolina/41/2009
HA	KC781576	H1N1	11/04/2009	USA	A/Washington/65/2009
HA	JX875047	H1N1	11/04/2009	USA	A/Kentucky/104/2009
HA	JX875054	H1N1	11/04/2009	USA	A/Kentucky/110/2009
HA	CY060710	H1N1	11/04/2009	Canada	A/Ontario/315637/2009
HA	CY070565	H1N1	11/04/2009	United Ki	A/England/841/2009
HA	CY070537	H1N1	11/04/2009	United Ki	A/England/808/2009
HA	CY070555	H1N1	11/04/2009	United Ki	A/England/94560148/2009
HA	CY060678	H1N1	11/04/2009	Canada	A/Ontario/315107/2009
HA	JN171898	H1N1	11/04/2009	Canada	A/Quebec/QC154661/2009
HA	AB558374	H1N1	11/04/2009	Japan	A/Sendai/TU526/2009
HA	AB558377	H1N1	11/04/2009	Japan	A/Sendai/TU530/2009
HA	AB558375	H1N1	11/04/2009	Japan	A/Sendai/TU527/2009
HA	AB558391	H1N1	11/04/2009	Japan	A/Sendai/TU529/2009
HA	AB558376	H1N1	11/04/2009	Japan	A/Sendai/TU528/2009
HA	AB558408	H1N1	11/04/2009	Japan	A/Sendai/TU1015/2009

HA	CY062683	H1N1	11/04/2009	USA	A/District of Columbia/INS114/2009(I
HA	CY060851	H1N1	11/04/2009	USA	A/Texas/JMS358/2009(H1N1)
HA	CY057051	H1N1	11/04/2009	United Ki	A/England/94640080/2009
HA	CY064391	H1N1	11/04/2009	China	A/Qingfeng/SWL43/2009(H1N1)
HA	HM189357	H1N1	11/05/2009	Russia	A/Orel/CRIE-KAI1/2009
HA	CY057726	H1N1	11/05/2009	USA	A/Wisconsin/629-D01915/2009(H1N1)
HA	JN171937	H1N1	11/05/2009	Canada	A/Montreal/MTL263979/2009
HA	KC781085	H1N1	11/05/2009	USA	A/Minnesota/18/2009
HA	HQ263267	H1N1	11/05/2009	USA	A/Bethesda/NIH106-D14/2009
HA	CY070558	H1N1	11/05/2009	United Ki	A/Scotland/51/2009
HA	AB558382	H1N1	11/05/2009	Japan	A/Sendai/TU563/2009
HA	JN171899	H1N1	11/05/2009	Canada	A/Quebec/QC000026/2009
HA	CY065816	H1N1	11/05/2009	Netherlar	A/Netherlands/2442b/2009
HA	CY057734	H1N1	11/05/2009	USA	A/Wisconsin/629-D01140/2009(H1N1)
HA	CY083854	H1N1	11/05/2009	USA	A/Pensacola/INS39/2009(H1N1)
HA	CY083870	H1N1	11/05/2009	USA	A/San Diego/INS49/2009(H1N1)
HA	CY057750	H1N1	11/05/2009	USA	A/Wisconsin/629-D01570/2009(H1N1)
HA	CY057758	H1N1	11/05/2009	USA	A/Wisconsin/629-D02370/2009(H1N1)
HA	CY063147	H1N1	11/05/2009	USA	A/California/VRDL99/2009(H1N1)
HA	CY063171	H1N1	11/05/2009	USA	A/California/VRDL102/2009(H1N1)
HA	CY063155	H1N1	11/05/2009	USA	A/California/VRDL100/2009(H1N1)
HA	CY066895	H1N1	11/05/2009	USA	A/District of Columbia/INS231/2009
HA	CY066199	H1N1	11/05/2009	USA	A/California/VRDL103/2009(H1N1)
HA	CY057718	H1N1	11/05/2009	USA	A/Wisconsin/629-D02317/2009(H1N1)
HA	CY057049	H1N1	11/05/2009	United Ki	A/England/94640078/2009
HA	CY063163	H1N1	11/05/2009	USA	A/California/VRDL101/2009(H1N1)
HA	CY057742	H1N1	11/05/2009	USA	A/Wisconsin/629-D00151/2009(H1N1)
HA	CY070560	H1N1	11/05/2009	United Ki	A/Scotland/55/2009
HA	CY057766	H1N1	11/05/2009	USA	A/Wisconsin/629-D01346/2009(H1N1)
HA	HM072420	H1N1	11/05/2009	Taiwan	A/Taiwan/7336/2009
HA	CY083721	H1N1	11/05/2009	USA	A/District of Columbia/INS115/2009(I
HA	KC780480	H1N1	11/05/2009	USA	A/Montana/19/2009
HA	KC780477	H1N1	11/05/2009	USA	A/North Dakota/15/2009
HA	HQ228138	H1N1	11/05/2009	Finland	A/Finland/699/2009
HA	CY054678	H1N1	11/05/2009	Russia	A/Russia/180/2009(H1N1)
HA	CY056236	H1N1	11/05/2009	USA	A/San Diego/INS70/2009(H1N1)
HA	CY066591	H1N1	11/05/2009	USA	A/San Diego/INS204/2009(H1N1)
HA	CY056228	H1N1	11/05/2009	USA	A/San Diego/INS69/2009(H1N1)
HA	KC781988	H1N1	11/05/2009	USA	A/Iowa/17/2009
HA	CY075660	H1N1	11/05/2009	USA	A/Boston/628/2009(H1N1)
HA	HQ228137	H1N1	11/05/2009	Finland	A/Finland/698/2009
HA	CY128435	H1N1	11/05/2009	Viet Nam	A/Viet Nam/001-1424/2009
HA	CY070559	H1N1	11/05/2009	United Ki	A/Scotland/53/2009
HA	KC780800	H1N1	11/06/2009	USA	A/District Of Columbia/13/2009
HA	KC781631	H1N1	11/06/2009	USA	A/Texas/72/2009
HA	CY070566	H1N1	11/06/2009	United Ki	A/Scotland/61/2009
HA	CY070561	H1N1	11/06/2009	United Ki	A/Scotland/56/2009
HA	HQ228115	H1N1	11/06/2009	Finland	A/Finland/673/2009
HA	KC261319	H1N1	11/06/2009	Mexico	A/Mexico City/INERPHAC2/2009(H1N1)
HA	CY083862	H1N1	11/06/2009	USA	A/District of Columbia/INS47/2009(H
HA	CY063763	H1N1	11/06/2009	Singapore	A/Singapore/GP4247/2009(H1N1)
HA	CY064398	H1N1	11/06/2009	China	A/Hualong/SWL1324/2009(H1N1)
HA	HM245793	H1N1	11/06/2009	China	A/Lishui/01p/2009(H1N1)
HA	KT181122	H1N1	11/06/2009	Singapore	A/Singapore/DMS49/2009

HA	KP317216	H1N1	11/06/2009	India	A/Tamil Naidu/012/2009
HA	CY063809	H1N1	11/06/2009	Singapore	A/Singapore/ON2269/2009
HA	AB558381	H1N1	11/06/2009	Japan	A/Sendai/TU555/2009
HA	CY056595	H1N1	11/06/2009	USA	A/New York/5916/2009(H1N1)
HA	CY070611	H1N1	11/06/2009	United Ki	A/England/881/2009
HA	KC780748	H1N1	11/06/2009	USA	A/Illinois/15/2009
HA	HQ228136	H1N1	11/06/2009	Finland	A/Finland/697/2009
HA	CY073033	H1N1	11/06/2009	El Salvad	A/San Salvador/WRAIR1109N/2009(H1N1)
HA	CY057058	H1N1	11/06/2009	United Ki	A/England/94760027/2009
HA	KC781937	H1N1	11/07/2009	USA	A/Michigan/26/2009
HA	KC780196	H1N1	11/07/2009	USA	A/North Carolina/59/2009
HA	CY060867	H1N1	11/07/2009	USA	A/Texas/JMS361/2009(H1N1)
HA	CY057037	H1N1	11/07/2009	United Ki	A/England/00380009/2009
HA	KC782171	H1N1	11/07/2009	USA	A/New Mexico/16/2009
HA	CY089219	H1N1	11/07/2009	USA	A/Boston/630/2009
HA	CY087200	H1N1	11/07/2009	USA	A/Texas/JMS360/2009
HA	CY060859	H1N1	11/07/2009	USA	A/Texas/JMS359/2009(H1N1)
HA	CY057054	H1N1	11/07/2009	United Ki	A/England/94740137/2009
HA	CY057774	H1N1	11/07/2009	USA	A/Wisconsin/629-D00802/2009(H1N1)
HA	CY065832	H1N1	11/07/2009	Netherlar	A/Netherlands/2457b/2009
HA	CY060875	H1N1	11/07/2009	USA	A/Texas/JMS362/2009(H1N1)
HA	AB558383	H1N1	11/07/2009	Japan	A/Sendai/TU556/2009
HA	CY070567	H1N1	11/07/2009	United Ki	A/Scotland/63/2009
HA	CY089227	H1N1	11/07/2009	USA	A/Boston/632/2009
HA	KC781007	H1N1	11/07/2009	USA	A/Massachusetts/32/2009
HA	KC780819	H1N1	11/07/2009	USA	A/Nebraska/22/2009
HA	KC780648	H1N1	11/08/2009	USA	A/Connecticut/15/2009
HA	HQ228134	H1N1	11/08/2009	Finland	A/Finland/695/2009
HA	CY060883	H1N1	11/08/2009	USA	A/Texas/JMS363/2009(H1N1)
HA	CY060899	H1N1	11/08/2009	USA	A/Texas/JMS365/2009(H1N1)
HA	CY060891	H1N1	11/08/2009	USA	A/Texas/JMS364/2009(H1N1)
HA	KC782188	H1N1	11/08/2009	USA	A/New Mexico/15/2009
HA	CY057806	H1N1	11/08/2009	USA	A/Wisconsin/629-D02392/2009(H1N1)
HA	CY089251	H1N1	11/08/2009	USA	A/Boston/637/2009
HA	CY070568	H1N1	11/08/2009	United Ki	A/England/842/2009
HA	CY061554	H1N1	11/08/2009	USA	A/Texas/JMS366/2009(H1N1)
HA	KC781591	H1N1	11/08/2009	USA	A/Arizona/19/2009
HA	KC782060	H1N1	11/08/2009	USA	A/Wisconsin/55/2009
HA	JN171938	H1N1	11/08/2009	Canada	A/Montreal/MTL266917/2009
HA	CY057798	H1N1	11/08/2009	USA	A/Wisconsin/629-D01466/2009(H1N1)
HA	CY057790	H1N1	11/08/2009	USA	A/Wisconsin/629-D01369/2009(H1N1)
HA	KC782150	H1N1	11/08/2009	USA	A/New Mexico/09/2009
HA	CY057782	H1N1	11/08/2009	USA	A/Wisconsin/629-D01014/2009(H1N1)
HA	CY060718	H1N1	11/08/2009	Canada	A/Ontario/320266/2009
HA	CY056651	H1N1	11/08/2009	USA	A/New York/6110/2009(H1N1)
HA	CY123245	H1N1	11/09/2009	Singapore	A/Singapore/GP4275/2009
HA	CY056611	H1N1	11/09/2009	USA	A/New York/5976/2009(H1N1)
HA	CY070569	H1N1	11/09/2009	United Ki	A/Scotland/64/2009
HA	CY057053	H1N1	11/09/2009	United Ki	A/England/94740054/2009
HA	CY056635	H1N1	11/09/2009	USA	A/New York/6019/2009(H1N1)
HA	CY075014	H1N1	11/09/2009	Thailand	A/Thailand/CU-H910/2009
HA	CY066783	H1N1	11/09/2009	USA	A/San Diego/INS214/2009
HA	CY056587	H1N1	11/09/2009	USA	A/New York/5886/2009(H1N1)
HA	CY083886	H1N1	11/09/2009	USA	A/San Diego/INS72/2009(H1N1)

HA	CY083894	H1N1	11/09/2009	USA	A/San Diego/INS73/2009(H1N1)
HA	CY089235	H1N1	11/09/2009	USA	A/Boston/633/2009
HA	CY070572	H1N1	11/09/2009	United Ki	A/Scotland/71/2009
HA	KC781382	H1N1	11/09/2009	USA	A/Maryland/38/2009
HA	KC780381	H1N1	11/09/2009	USA	A/Washington/62/2009
HA	KC780821	H1N1	11/09/2009	USA	A/Rhode Island/20/2009
HA	KC780201	H1N1	11/09/2009	USA	A/Maine/13/2009
HA	KC781842	H1N1	11/09/2009	USA	A/Texas/73/2009
HA	KC781621	H1N1	11/09/2009	USA	A/Idaho/14/2009
HA	CY123836	H1N1	11/09/2009	Singapore	A/Singapore/ON2274/2009
HA	CY089243	H1N1	11/09/2009	USA	A/Boston/635/2009
HA	KC780286	H1N1	11/09/2009	USA	A/New Jersey/09/2009
HA	HM189386	H1N1	11/09/2009	Russia	A/Moscow oblast/CRIE-STV/2009
HA	CY064399	H1N1	11/09/2009	China	A/Hualong/SWL1334/2009(H1N1)
HA	CY056164	H1N1	11/09/2009	USA	A/Pensacola/INS40/2009(H1N1)
HA	CY075540	H1N1	11/09/2009	USA	A/Boston/634/2009(H1N1)
HA	CY066983	H1N1	11/09/2009	Belgium	A/Brussels/INS243/2009(H1N1)
HA	CY071770	H1N1	11/09/2009	Mexico	A/Mexico City/WR1747N/2009(H1N1)
HA	CY056603	H1N1	11/09/2009	USA	A/New York/5931/2009(H1N1)
HA	CY057326	H1N1	11/09/2009	USA	A/New York/6072/2009(H1N1)
HA	JN171900	H1N1	11/09/2009	Canada	A/Quebec/QC000462/2009
HA	HQ228135	H1N1	11/09/2009	Finland	A/Finland/696/2009
HA	KC780984	H1N1	11/09/2009	USA	A/Michigan/27/2009
HA	KC782241	H1N1	11/09/2009	USA	A/Minnesota/40/2009
HA	KC781424	H1N1	11/09/2009	USA	A/Oklahoma/10/2009
HA	KC780794	H1N1	11/09/2009	USA	A/Maryland/31/2009
HA	AB704494	H1N1	11/09/2009	Japan	A/Yamagata/473/2009
HA	CY083878	H1N1	11/09/2009	USA	A/San Diego/INS50/2009(H1N1)
HA	CY057056	H1N1	11/10/2009	United Ki	A/England/94740139/2009
HA	CY057052	H1N1	11/10/2009	United Ki	A/England/94740049/2009
HA	CY066791	H1N1	11/10/2009	USA	A/San Diego/INS215/2009
HA	CY066751	H1N1	11/10/2009	USA	A/Pensacola/INS210/2009
HA	CY052366	H1N1	11/10/2009	Mongolia	A/Zavkhan/8299/2009(H1N1)
HA	KC780249	H1N1	11/10/2009	USA	A/Washington/63/2009
HA	HQ228119	H1N1	11/10/2009	Finland	A/Finland/680/2009
HA	CY073142	H1N1	11/10/2009	Mexico	A/Mexico City/WRAIR1781T/2009(H1N1)
HA	CY066759	H1N1	11/10/2009	USA	A/Pensacola/INS211/2009
HA	KC780866	H1N1	11/10/2009	USA	A/Maine/09/2009
HA	CY107218	H1N1	11/10/2009	United Ki	A/Scotland/Inverness_10/2009
HA	HM189360	H1N1	11/10/2009	Russia	A/Yaroslavl/CRIE-CHMV/2009
HA	JN588783	H1N1	11/10/2009	Cambodia	A/Cambodia/T234/2009
HA	KP019832	H1N1	11/10/2009	China	A/FuZhou/SWL11911/2009
HA	CY083551	H1N1	11/10/2009	Hungary	A/Budapest/WRAIR2393T/2009
HA	CY070571	H1N1	11/10/2009	United Ki	A/Scotland/66/2009
HA	KC780733	H1N1	11/10/2009	USA	A/Kentucky/22/2009
HA	CY063813	H1N1	11/10/2009	Singapore	A/Singapore/ON2286/2009
HA	HM189352	H1N1	11/10/2009	Russia	A/Lipetsk/CRIE-BVV/2009
HA	CY063582	H1N1	11/10/2009	Norway	A/Oslo/INS110/2009(H1N1)
HA	KC781688	H1N1	11/10/2009	USA	A/Alaska/43/2009
HA	HQ652611	H1N1	11/10/2009	China	A/Jiangsu/S62/2009
HA	HQ652610	H1N1	11/10/2009	China	A/Jiangsu/S61/2009
HA	CY075479	H1N1	11/10/2009	Russia	A/Voronezh/RII1/2009(H1N1)
HA	KC781010	H1N1	11/10/2009	USA	A/New Jersey/11/2009
HA	KC780636	H1N1	11/10/2009	USA	A/Illinois/19/2009

HA	KC782225	H1N1	11/10/2009	USA	A/Arkansas/10/2009
HA	CY128323	H1N1	11/10/2009	Viet Nam	A/Viet Nam/13032093/2009
HA	CY107314	H1N1	11/10/2009	United Ki	A/Scotland/Aberdeen_8/2009
HA	CY107211	H1N1	11/10/2009	United Ki	A/Scotland/Aberdeen_1/2009
HA	CY107411	H1N1	11/10/2009	United Ki	A/Scotland/Perth_09V501016/2009
HA	CY053627	H1N1	11/10/2009	Russia	A/Russia/165/2009(H1N1)
HA	CY062787	H1N1	11/10/2009	Denmark	A/Hvidovre/INS139/2009(H1N1)
HA	CY065848	H1N1	11/10/2009	Netherlar	A/Netherlands/1324/2009(H1N1)
HA	CY083543	H1N1	11/10/2009	Hungary	A/Budapest/WRAIR2393N/2009
HA	CY057035	H1N1	11/10/2009	United Ki	A/England/00380004/2009
HA	CY066767	H1N1	11/10/2009	USA	A/Pensacola/INS212/2009
HA	JQ065196	H1N1	11/11/2009	China	A/Shenzhen/1391/2009
HA	HQ228111	H1N1	11/11/2009	Finland	A/Finland/668/2009
HA	CY107274	H1N1	11/11/2009	United Ki	A/Scotland/Aberdeen_2/2009
HA	JQ065198	H1N1	11/11/2009	China	A/Shenzhen/90/2009
HA	JQ065197	H1N1	11/11/2009	China	A/Shenzhen/1430/2009
HA	JQ065226	H1N1	11/11/2009	China	A/Shenzhen/A3/2009
HA	CY107281	H1N1	11/11/2009	United Ki	A/Scotland/Aberdeen_3/2009
HA	KP019833	H1N1	11/11/2009	China	A/FuZhou/SWL11916/2009
HA	JQ065195	H1N1	11/11/2009	China	A/Shenzhen/1387/2009
HA	JQ065229	H1N1	11/11/2009	China	A/Shenzhen/A8/2009
HA	JQ065191	H1N1	11/11/2009	China	A/Shenzhen/1282/2009
HA	JQ065227	H1N1	11/11/2009	China	A/Shenzhen/A4/2009
HA	JQ065189	H1N1	11/11/2009	China	A/Shenzhen/80/2009
HA	JQ065192	H1N1	11/11/2009	China	A/Shenzhen/1323/2009
HA	JQ065188	H1N1	11/11/2009	China	A/Shenzhen/79/2009
HA	JQ065194	H1N1	11/11/2009	China	A/Shenzhen/1347/2009
HA	JQ065190	H1N1	11/11/2009	China	A/Shenzhen/1266/2009
HA	JQ065186	H1N1	11/11/2009	China	A/Shenzhen/75/2009
HA	CY070603	H1N1	11/11/2009	United Ki	A/Scotland/80/2009
HA	GU369671	H1N1	11/11/2009	Turkey	A/Ankara/27/2009
HA	CY062811	H1N1	11/11/2009	Denmark	A/Odense/INS143/2009(H1N1)
HA	CY070570	H1N1	11/11/2009	United Ki	A/Scotland/65/2009
HA	CY070605	H1N1	11/11/2009	United Ki	A/Scotland/75/2009
HA	JQ065193	H1N1	11/11/2009	China	A/Shenzhen/1333/2009
HA	JN032406	H1N1	11/11/2009	China	A/Changchun/01/2009
HA	KC780634	H1N1	11/11/2009	USA	A/Mississippi/07/2009
HA	CY107225	H1N1	11/11/2009	United Ki	A/Scotland/Aberdeen_11/2009
HA	CY107308	H1N1	11/11/2009	United Ki	A/Scotland/Aberdeen_7/2009
HA	JQ065187	H1N1	11/11/2009	China	A/Shenzhen/78/2009
HA	JQ065185	H1N1	11/11/2009	China	A/Shenzhen/74/2009
HA	CY070602	H1N1	11/11/2009	United Ki	A/Scotland/79/2009
HA	GU371263	H1N1	11/11/2009	Russia	A/Bryansk/IIV2971/2009(H1N1)
HA	CY066839	H1N1	11/11/2009	Spain	A/Madrid/INS223/2009
HA	CY063283	H1N1	11/11/2009	USA	A/Wisconsin/629-D02008/2009(H1N1)
HA	CY084438	H1N1	11/11/2009	USA	A/New York/6064/2009
HA	CY057036	H1N1	11/11/2009	United Ki	A/England/00380005/2009
HA	CY107288	H1N1	11/11/2009	United Ki	A/Scotland/Aberdeen_4/2009
HA	KC781077	H1N1	11/12/2009	USA	A/Oklahoma/05/2009
HA	HQ228121	H1N1	11/12/2009	Finland	A/Finland/682/2009
HA	CY066807	H1N1	11/12/2009	USA	A/San Diego/INS217/2009
HA	CY066855	H1N1	11/12/2009	USA	A/District of Columbia/INS226/2009(I
HA	CY067643	H1N1	11/12/2009	Spain	A/Madrid/INS224/2009(H1N1)
HA	CY061562	H1N1	11/12/2009	USA	A/Texas/JMS367/2009(H1N1)

HA	CY057342	H1N1	11/12/2009	USA	A/San Diego/INS75/2009(H1N1)
HA	HM189423	H1N1	11/12/2009	Russia	A/Moscow oblast/CRIE-BGI/2009
HA	CY066863	H1N1	11/12/2009	USA	A/District of Columbia/INS227/2009(I
HA	CY064402	H1N1	11/12/2009	China	A/Xigong/SWL11045/2009(H1N1)
HA	CY107253	H1N1	11/12/2009	United Ki	A/Scotland/Inverness_15/2009
HA	KT181123	H1N1	11/12/2009	Singapore	A/Singapore/DMS50/2009
HA	JQ714163	H1N1	11/12/2009	China	A/Tianjinheping/SWL313/2009
HA	CY062651	H1N1	11/12/2009	USA	A/San Diego/INS74/2009(H1N1)
HA	HQ228122	H1N1	11/12/2009	Finland	A/Finland/683/2009
HA	CY057038	H1N1	11/12/2009	United Ki	A/England/00380013/2009
HA	CY070606	H1N1	11/12/2009	United Ki	A/Scotland/76/2009
HA	CY061275	H1N1	11/12/2009	USA	A/Pensacola/INS107/2009(H1N1)
HA	CY107267	H1N1	11/12/2009	United Ki	A/Scotland/Inverness_18/2009
HA	CY064404	H1N1	11/12/2009	China	A/Weidu/SWL1353/2009(H1N1)
HA	CY063765	H1N1	11/12/2009	Singapore	A/Singapore/GP4309/2009(H1N1)
HA	CY054670	H1N1	11/12/2009	Russia	A/Russia/171/2009(H1N1)
HA	HQ228088	H1N1	11/12/2009	Finland	A/Finland/643/2009
HA	CY070577	H1N1	11/12/2009	United Ki	A/England/94700035/2009
HA	JN171939	H1N1	11/12/2009	Canada	A/Montreal/MTL272178/2009
HA	CY123841	H1N1	11/12/2009	Singapore	A/Singapore/ON2291/2009
HA	CY107232	H1N1	11/12/2009	United Ki	A/Scotland/Aberdeen_12/2009
HA	CY107239	H1N1	11/12/2009	United Ki	A/Scotland/Aberdeen_13/2009
HA	CY107260	H1N1	11/12/2009	United Ki	A/Scotland/Aberdeen_16/2009
HA	GU369672	H1N1	11/12/2009	Turkey	A/Ankara/28/2009(H1N1)
HA	CY075548	H1N1	11/12/2009	USA	A/Boston/648/2009(H1N1)
HA	CY066799	H1N1	11/12/2009	USA	A/San Diego/INS216/2009
HA	CY063291	H1N1	11/12/2009	USA	A/Wisconsin/629-D00292/2009(H1N1)
HA	HM173599	H1N1	11/12/2009	Russia	A/Blagovechensk/01/2009(H1N1)
HA	AB558384	H1N1	11/12/2009	Japan	A/Sendai/TU565/2009
HA	HQ228120	H1N1	11/12/2009	Finland	A/Finland/681/2009
HA	CY089259	H1N1	11/12/2009	USA	A/Boston/650/2009
HA	CY073481	H1N1	11/13/2009	Germany	A/Bochum/INS248/2009(H1N1)
HA	CY066847	H1N1	11/13/2009	Spain	A/Madrid/INS225/2009
HA	CY067015	H1N1	11/13/2009	Germany	A/Bochum/INS249/2009(H1N1)
HA	CY067023	H1N1	11/13/2009	Germany	A/Bochum/INS250/2009(H1N1)
HA	CY070575	H1N1	11/13/2009	United Ki	A/England/94700028/2009
HA	CY075572	H1N1	11/13/2009	USA	A/Boston/658/2009(H1N1)
HA	CY071802	H1N1	11/13/2009	Haiti	A/Port Au Prince/WR2294T/2009(H1N1)
HA	HM072422	H1N1	11/13/2009	Taiwan	A/Taiwan/2203/2009
HA	CY062675	H1N1	11/13/2009	Spain	A/Madrid/INS113/2009(H1N1)
HA	HM189421	H1N1	11/13/2009	Russia	A/Ulyanovsk/CRIE-KLA/2009
HA	KC780255	H1N1	11/13/2009	USA	A/Minnesota/20/2009
HA	CY060907	H1N1	11/13/2009	USA	A/Texas/JMS369/2009(H1N1)
HA	CY107246	H1N1	11/13/2009	United Ki	A/Scotland/Aberdeen_14/2009
HA	HQ228125	H1N1	11/13/2009	Finland	A/Finland/686/2009
HA	HM773210	H1N1	11/13/2009	China	A/Jiangsu/2/2009(H1N1)
HA	CY075482	H1N1	11/13/2009	Russia	A/Nizhny Novgorod/RII2/2009(H1N1)
HA	CY073088	H1N1	11/13/2009	Turkey	A/Ankara/WRAIR1435T/2009
HA	JN631054	H1N1	11/13/2009	China	A/Shanghai/1744T/2009
HA	CY070581	H1N1	11/13/2009	United Ki	A/England/94720080/2009
HA	CY070609	H1N1	11/13/2009	United Ki	A/England/887/2009
HA	CY070626	H1N1	11/13/2009	United Ki	A/England/906/2009
HA	CY070573	H1N1	11/13/2009	United Ki	A/England/94700021/2009
HA	CY070576	H1N1	11/13/2009	United Ki	A/England/94700033/2009

HA	CY070580	H1N1	11/13/2009	United Ki	A/England/94720050/2009
HA	CY070584	H1N1	11/13/2009	United Ki	A/Scotland/70/2009
HA	CY070582	H1N1	11/13/2009	United Ki	A/England/94720081/2009
HA	KC780770	H1N1	11/13/2009	USA	A/Arkansas/09/2009
HA	CY128371	H1N1	11/13/2009	Viet Nam	A/Viet Nam/11032001/2009
HA	KC781104	H1N1	11/13/2009	USA	A/Rhode Island/22/2009
HA	CY070578	H1N1	11/13/2009	United Ki	A/England/94720002/2009
HA	CY107295	H1N1	11/13/2009	United Ki	A/Scotland/Aberdeen_5/2009
HA	CY064403	H1N1	11/13/2009	China	A/Hualong/SWL1347/2009(H1N1)
HA	CY087208	H1N1	11/13/2009	USA	A/Texas/JMS368/2009
HA	CY075564	H1N1	11/13/2009	USA	A/Boston/655/2009(H1N1)
HA	CY075556	H1N1	11/13/2009	USA	A/Boston/653/2009(H1N1)
HA	CY066871	H1N1	11/13/2009	USA	A/District of Columbia/INS228/2009(I
HA	CY063767	H1N1	11/13/2009	Singapore	A/Singapore/GP4326/2009(H1N1)
HA	CY070574	H1N1	11/13/2009	United Ki	A/England/94700025/2009
HA	KF860845	H1N1	11/14/2009	Spain	A/Castilla La Mancha/5911/2009
HA	HQ228112	H1N1	11/14/2009	Finland	A/Finland/669/2009
HA	HQ228114	H1N1	11/14/2009	Finland	A/Finland/672/2009
HA	CY128339	H1N1	11/14/2009	Viet Nam	A/Viet Nam/13032097/2009
HA	CY075483	H1N1	11/14/2009	Russia	A/Nizhny Novgorod/RII1/2009(H1N1)
HA	GU451254	H1N1	11/14/2009	Russia	A/Tver/IIV2969/2009(H1N1)
HA	CY070608	H1N1	11/14/2009	United Ki	A/England/886/2009
HA	CY070585	H1N1	11/14/2009	United Ki	A/Scotland/73/2009
HA	CY070586	H1N1	11/14/2009	United Ki	A/Scotland/74/2009
HA	CY089267	H1N1	11/14/2009	USA	A/Boston/657/2009
HA	CY128419	H1N1	11/14/2009	Viet Nam	A/Viet Nam/12032001/2009
HA	CY128331	H1N1	11/14/2009	Viet Nam	A/Viet Nam/13032096/2009
HA	CY123255	H1N1	11/14/2009	Singapore	A/Singapore/GP4348/2009
HA	CY057822	H1N1	11/14/2009	USA	A/Wisconsin/629-D00965/2009(H1N1)
HA	CY057814	H1N1	11/14/2009	USA	A/Wisconsin/629-D01826/2009(H1N1)
HA	CY060915	H1N1	11/14/2009	USA	A/Texas/JMS370/2009(H1N1)
HA	CY060923	H1N1	11/14/2009	USA	A/Texas/JMS371/2009(H1N1)
HA	CY073064	H1N1	11/15/2009	Turkey	A/Ankara/WRAIR1425T/2009(H1N1)
HA	CY065115	H1N1	11/15/2009	Netherlar	A/Netherlands/2464b/2009
HA	CY075596	H1N1	11/15/2009	USA	A/Boston/672/2009(H1N1)
HA	HM581923	H1N1	11/15/2009	Iran	A/Iran/14068/2009(H1N1)
HA	CY128347	H1N1	11/15/2009	Viet Nam	A/Viet Nam/13032100/2009
HA	CY060931	H1N1	11/15/2009	USA	A/Texas/JMS372/2009(H1N1)
HA	KT181124	H1N1	11/15/2009	Singapore	A/Singapore/DMS51/2009
HA	CY128467	H1N1	11/15/2009	Viet Nam	A/Viet Nam/001-1020/2009
HA	CY070583	H1N1	11/15/2009	United Ki	A/Scotland/68/2009
HA	CY057854	H1N1	11/15/2009	USA	A/Wisconsin/629-D00295/2009(H1N1)
HA	JN171901	H1N1	11/15/2009	Canada	A/Quebec/QC001496/2009
HA	CY056683	H1N1	11/15/2009	USA	A/New York/6257/2009(H1N1)
HA	CY070636	H1N1	11/15/2009	United Ki	A/England/925/2009
HA	CY066215	H1N1	11/15/2009	USA	A/California/VRDL105/2009(H1N1)
HA	HQ228117	H1N1	11/15/2009	Finland	A/Finland/675/2009
HA	CY057830	H1N1	11/15/2009	USA	A/Wisconsin/629-D00911/2009(H1N1)
HA	HM581921	H1N1	11/15/2009	Iran	A/Iran/14089/2009(H1N1)
HA	CY057838	H1N1	11/15/2009	USA	A/Wisconsin/629-D00128/2009(H1N1)
HA	CY057846	H1N1	11/15/2009	USA	A/Wisconsin/629-D02060/2009(H1N1)
HA	CY057862	H1N1	11/15/2009	USA	A/Wisconsin/629-D00936/2009(H1N1)
HA	CY066207	H1N1	11/15/2009	USA	A/California/VRDL104/2009(H1N1)
HA	CY066239	H1N1	11/15/2009	USA	A/California/VRDL108/2009(H1N1)

HA	CY066223	H1N1	11/15/2009	USA	A/California/VRDL106/2009(H1N1)
HA	CY066231	H1N1	11/15/2009	USA	A/California/VRDL107/2009(H1N1)
HA	JN171940	H1N1	11/15/2009	Canada	A/Montreal/MTL274373/2009
HA	CY089299	H1N1	11/15/2009	USA	A/Boston/671/2009
HA	CY123253	H1N1	11/16/2009	Singapore	A/Singapore/GP4332/2009
HA	CY056659	H1N1	11/16/2009	USA	A/New York/6182/2009(H1N1)
HA	CY089307	H1N1	11/16/2009	USA	A/Boston/673/2009
HA	CY089275	H1N1	11/16/2009	USA	A/Boston/665/2009
HA	CY075588	H1N1	11/16/2009	USA	A/Boston/666/2009(H1N1)
HA	CY058332	H1N1	11/16/2009	USA	A/Wisconsin/629-D01347/2009(H1N1)
HA	HQ698627	H1N1	11/16/2009	China	A/Beijing/16/2009
HA	CY056667	H1N1	11/16/2009	USA	A/New York/6213/2009(H1N1)
HA	CY056699	H1N1	11/16/2009	USA	A/New York/6293/2009(H1N1)
HA	HQ228092	H1N1	11/16/2009	Finland	A/Finland/647/2009
HA	CY089291	H1N1	11/16/2009	USA	A/Boston/670/2009
HA	CY089283	H1N1	11/16/2009	USA	A/Boston/667/2009
HA	CY063815	H1N1	11/16/2009	Singapore	A/Singapore/ON2308/2009
HA	KP637406	H1N1	11/16/2009	Thailand	A/Thailand/MR08303/2009
HA	CY057055	H1N1	11/16/2009	United Ki	A/England/94740138/2009
HA	CY065131	H1N1	11/16/2009	Netherlar	A/Netherlands/2143/2009
HA	GU371256	H1N1	11/16/2009	Russia	A/Orenburg/IIV2974/2009(H1N1)
HA	CY075580	H1N1	11/16/2009	USA	A/Boston/663/2009(H1N1)
HA	CY070579	H1N1	11/16/2009	United Ki	A/England/94720043/2009
HA	CY070590	H1N1	11/16/2009	United Ki	A/England/799/2009
HA	KC781108	H1N1	11/16/2009	USA	A/New Hampshire/21/2009
HA	KC781144	H1N1	11/16/2009	USA	A/Utah/59/2009
HA	CY123846	H1N1	11/16/2009	Singapore	A/Singapore/ON2299/2009
HA	AB704430	H1N1	11/16/2009	Japan	A/Gunma/263/2009
HA	CY057870	H1N1	11/16/2009	USA	A/Wisconsin/629-D01661/2009(H1N1)
HA	CY057057	H1N1	11/16/2009	United Ki	A/England/94740140/2009
HA	CY056675	H1N1	11/16/2009	USA	A/New York/6214/2009(H1N1)
HA	CY066999	H1N1	11/16/2009	Germany	A/Munich/INS246/2009
HA	CY064401	H1N1	11/16/2009	China	A/Zhongyuan/SWL1643/2009(H1N1)
HA	CY089331	H1N1	11/17/2009	USA	A/Boston/683/2009
HA	AB558390	H1N1	11/17/2009	Japan	A/Sendai/TU587/2009
HA	CY057039	H1N1	11/17/2009	United Ki	A/England/00380015/2009
HA	CY089323	H1N1	11/17/2009	USA	A/Boston/680/2009
HA	CY070588	H1N1	11/17/2009	United Ki	A/England/94760188/2009
HA	HQ228123	H1N1	11/17/2009	Finland	A/Finland/684/2009
HA	CY062667	H1N1	11/17/2009	USA	A/Pensacola/INS109/2009(H1N1)
HA	AB558388	H1N1	11/17/2009	Japan	A/Sendai/TU582/2009
HA	AB558387	H1N1	11/17/2009	Japan	A/Sendai/TU586/2009
HA	KC781331	H1N1	11/17/2009	USA	A/Tennessee/21/2009
HA	CY066719	H1N1	11/17/2009	Spain	A/Madrid/INS295/2009(H1N1)
HA	CY069114	H1N1	11/17/2009	Spain	A/Madrid/INS296/2009(H1N1)
HA	CY061283	H1N1	11/17/2009	USA	A/Pensacola/INS108/2009(H1N1)
HA	CY089315	H1N1	11/17/2009	USA	A/Boston/678/2009
HA	KC781934	H1N1	11/17/2009	USA	A/Utah/53/2009
HA	KP637806	H1N1	11/17/2009	Thailand	A/Thailand/SS08207/2009
HA	CY070591	H1N1	11/17/2009	United Ki	A/England/94760209/2009
HA	CY070592	H1N1	11/17/2009	United Ki	A/England/94760211/2009
HA	CY075604	H1N1	11/17/2009	USA	A/Boston/681/2009(H1N1)
HA	AB558385	H1N1	11/17/2009	Japan	A/Sendai/TU579/2009
HA	JF429394	H1N1	11/17/2009	France	A/Lyon/47.431/2009

HA	JN631053	H1N1	11/17/2009	China	A/Shanghai/1783T/2009
HA	CY070587	H1N1	11/17/2009	United Ki	A/England/849/2009
HA	KC780807	H1N1	11/17/2009	USA	A/Indiana/19/2009
HA	KC780346	H1N1	11/17/2009	USA	A/Alaska/44/2009
HA	CY128049	H1N1	11/17/2009	Viet Nam	A/Viet Nam/11032002/2009
HA	CY060726	H1N1	11/17/2009	Canada	A/Ontario/328474/2009
HA	CY123854	H1N1	11/17/2009	Singapore	A/Singapore/ON2318/2009
HA	CY064430	H1N1	11/17/2009	China	A/Guangdong/1078/2009(H1N1)
HA	CY070604	H1N1	11/17/2009	United Ki	A/Scotland/81/2009
HA	CY063817	H1N1	11/17/2009	Singapore	A/Singapore/ON2313/2009
HA	CY065824	H1N1	11/17/2009	Netherlan	A/Netherlands/2243/2009
HA	CY058348	H1N1	11/17/2009	USA	A/Wisconsin/629-D01582/2009(H1N1)
HA	CY058340	H1N1	11/17/2009	USA	A/Wisconsin/629-D00503/2009(H1N1)
HA	CY064394	H1N1	11/17/2009	China	A/Ruyang/SWL1606/2009(H1N1)
HA	CY056707	H1N1	11/17/2009	USA	A/New York/6300/2009(H1N1)
HA	CY075612	H1N1	11/17/2009	USA	A/Boston/682/2009(H1N1)
HA	CY066775	H1N1	11/17/2009	USA	A/Pensacola/INS213/2009
HA	AB558386	H1N1	11/17/2009	Japan	A/Sendai/TU589/2009
HA	AB558389	H1N1	11/17/2009	Japan	A/Sendai/TU588/2009
HA	CY057061	H1N1	11/17/2009	United Ki	A/England/94780019/2009
HA	CY056691	H1N1	11/17/2009	USA	A/New York/6292/2009(H1N1)
HA	CY056715	H1N1	11/18/2009	USA	A/New York/6346/2009(H1N1)
HA	CY064395	H1N1	11/18/2009	China	A/Xuchang/SWL48/2009(H1N1)
HA	GU369673	H1N1	11/18/2009	Turkey	A/Ankara/29/2009
HA	CY060460	H1N1	11/18/2009	South Kor	A/Gangwon/1805/2009(H1N1)
HA	CY064407	H1N1	11/18/2009	China	A/Weidu/SWL1379/2009(H1N1)
HA	CY073190	H1N1	11/18/2009	Serbia	A/Belgrade/WRAIR2368N/2009(H1N1)
HA	CY073174	H1N1	11/18/2009	Nigeria	A/Lagos/WRAIR1984T/2009(H1N1)
HA	CY067651	H1N1	11/18/2009	Belgium	A/Brussels/INS244/2009(H1N1)
HA	CY066991	H1N1	11/18/2009	Belgium	A/Brussels/INS245/2009(H1N1)
HA	CY089339	H1N1	11/18/2009	USA	A/Boston/685/2009
HA	CY057062	H1N1	11/18/2009	United Ki	A/England/94780021/2009
HA	HQ228113	H1N1	11/18/2009	Finland	A/Finland/671/2009
HA	CY070594	H1N1	11/18/2009	United Ki	A/England/94780297/2009
HA	CY070593	H1N1	11/18/2009	United Ki	A/England/94780281/2009
HA	JN171902	H1N1	11/18/2009	Canada	A/Quebec/QC001957/2009
HA	CY128355	H1N1	11/18/2009	Viet Nam	A/Viet Nam/15032003/2009
HA	CY063769	H1N1	11/18/2009	Singapore	A/Singapore/GP4381/2009
HA	HM567984	H1N1	11/18/2009	United Ki	A/England/869/2009
HA	JQ714177	H1N1	11/18/2009	China	A/Tianjinheping/SWL299/2009
HA	CY073166	H1N1	11/18/2009	Nigeria	A/Lagos/WRAIR1984N/2009(H1N1)
HA	CY073198	H1N1	11/18/2009	Serbia	A/Belgrade/WRAIR2368T/2009(H1N1)
HA	CY088729	H1N1	11/18/2009	Netherlan	A/Netherlands/2223b/2009
HA	CY064392	H1N1	11/18/2009	China	A/Jinshui/SWL54/2009(H1N1)
HA	CY064426	H1N1	11/18/2009	China	A/Guangdong/1071/2009(H1N1)
HA	CY064396	H1N1	11/18/2009	China	A/Xuchang/SWL49/2009(H1N1)
HA	KC780812	H1N1	11/18/2009	USA	A/Utah/57/2009
HA	CY070601	H1N1	11/18/2009	United Ki	A/England/852/2009
HA	JN171941	H1N1	11/18/2009	Canada	A/Montreal/MTL278270/2009
HA	HM189417	H1N1	11/18/2009	Russia	A/Ivanovo/CRIE-BOV/2009
HA	CY070589	H1N1	11/18/2009	United Ki	A/England/94760192/2009
HA	CY073741	H1N1	11/18/2009	Denmark	A/Copenhagen/INS433/2009(H1N1)
HA	CY070595	H1N1	11/18/2009	United Ki	A/England/94780318/2009
HA	CY089347	H1N1	11/18/2009	USA	A/Boston/686/2009

HA	GU560008	H1N1	11/18/2009	Russia	A/Tomsk/05/2009(H1N1)
HA	HM189403	H1N1	11/18/2009	Russia	A/Stavropol/CRIE-BLV/2009
HA	CY058830	H1N1	11/18/2009	China	A/Guangdong/2221/2009(H1N1)
HA	HM189358	H1N1	11/18/2009	Russia	A/Yakutsk/CRIE-EAV/2009
HA	CY058356	H1N1	11/18/2009	USA	A/Wisconsin/629-D02489/2009(H1N1)
HA	HM189418	H1N1	11/18/2009	Russia	A/Ivanovo/CRIE-AIV/2009
HA	CY065856	H1N1	11/19/2009	Netherlar	A/Netherlands/2229/2009
HA	HM189378	H1N1	11/19/2009	Russia	A/St.Petersburg/CRIE-VMN/2009
HA	CY064436	H1N1	11/19/2009	China	A/Guangdong/1241/2009(H1N1)
HA	CY064452	H1N1	11/19/2009	China	A/Guangdong/1271/2009(H1N1)
HA	CY064442	H1N1	11/19/2009	China	A/Guangdong/1252/2009(H1N1)
HA	CY064434	H1N1	11/19/2009	China	A/Guangdong/1240/2009(H1N1)
HA	CY064428	H1N1	11/19/2009	China	A/Guangdong/1075/2009(H1N1)
HA	CY063307	H1N1	11/19/2009	USA	A/Wisconsin/629-D01572/2009(H1N1)
HA	CY063299	H1N1	11/19/2009	USA	A/Wisconsin/629-D00859/2009(H1N1)
HA	CY057069	H1N1	11/19/2009	United Ki	A/England/94840152/2009
HA	CY083144	H1N1	11/19/2009	Kyrgyzsta	A/Bishkek/WRAIR0883N/2009
HA	HQ228118	H1N1	11/19/2009	Finland	A/Finland/677/2009
HA	HQ228126	H1N1	11/19/2009	Finland	A/Finland/687/2009
HA	HQ228093	H1N1	11/19/2009	Finland	A/Finland/648/2009
HA	CY123263	H1N1	11/19/2009	Singapore	A/Singapore/GP4407/2009
HA	AB704422	H1N1	11/19/2009	Japan	A/Gunma/262/2009
HA	JQ714164	H1N1	11/19/2009	China	A/Tianjintanggu/SWL432/2009
HA	CY071223	H1N1	11/19/2009	Norway	A/Oslo/INS404/2009(H1N1)
HA	CY066703	H1N1	11/19/2009	Austria	A/Vienna/INS291/2009(H1N1)
HA	KC780210	H1N1	11/19/2009	USA	A/Oregon/37/2009
HA	CY057040	H1N1	11/19/2009	United Ki	A/England/00380018/2009
HA	CY066631	H1N1	11/19/2009	Germany	A/Bonn/INS277/2009(H1N1)
HA	CY064390	H1N1	11/19/2009	China	A/Weidu/SWL51/2009(H1N1)
HA	CY071786	H1N1	11/19/2009	Mexico	A/Mexico City/WR1765N/2009(H1N1)
HA	CY066935	H1N1	11/19/2009	Denmark	A/Aarhus/INS236/2009(H1N1)
HA	CY070597	H1N1	11/19/2009	United Ki	A/England/94800096/2009
HA	CY070741	H1N1	11/19/2009	United Ki	A/England/891/2009
HA	CY071586	H1N1	11/19/2009	Jordan	A/Amman/WR1335T/2009(H1N1)
HA	CY062747	H1N1	11/19/2009	Spain	A/Madrid/INS129/2009(H1N1)
HA	CY100488	H1N1	11/19/2009	Mexico	A/Mexico City/INER14/2009
HA	CY062739	H1N1	11/19/2009	Germany	A/Bonn/INS127/2009(H1N1)
HA	CY064400	H1N1	11/19/2009	China	A/Baofeng/SWL537/2009(H1N1)
HA	CY120919	H1N1	11/20/2009	China	A/Guangdong/1103/2009
HA	CY066679	H1N1	11/20/2009	Denmark	A/Hvidovre/INS288/2009(H1N1)
HA	CY064393	H1N1	11/20/2009	China	A/Dongcheng/SWL45/2009(H1N1)
HA	HM754224	H1N1	11/20/2009	China	A/Jiangsu/1/2009(H1N1)
HA	CY070634	H1N1	11/20/2009	United Ki	A/Scotland/84/2009
HA	HQ420235	H1N1	11/20/2009	Poland	A/Gdansk/037/2009(H1N1)
HA	CY066647	H1N1	11/20/2009	Germany	A/Bonn/INS279/2009(H1N1)
HA	CY057070	H1N1	11/20/2009	United Ki	A/England/94840153/2009
HA	CY064444	H1N1	11/20/2009	China	A/Guangdong/1260/2009(H1N1)
HA	CY089355	H1N1	11/20/2009	USA	A/Boston/690/2009
HA	CY056947	H1N1	11/20/2009	Denmark	A/Aarhus/INS82/2009(H1N1)
HA	CY066639	H1N1	11/20/2009	Germany	A/Bonn/INS278/2009(H1N1)
HA	HM230693	H1N1	11/20/2009	China	A/Zhoushan/1/2009(H1N1)
HA	HQ228091	H1N1	11/20/2009	Finland	A/Finland/646/2009
HA	HM567968	H1N1	11/20/2009	United Ki	A/England/876/2009
HA	HM189406	H1N1	11/20/2009	Russia	A/Orel/CRIE-AAP/2009

HA	CY062795	H1N1	11/20/2009	Denmark	A/Hvidovre/INS141/2009(H1N1)
HA	CY083737	H1N1	11/20/2009	Spain	A/Madrid/INS130/2009(H1N1)
HA	HM072423	H1N1	11/20/2009	Taiwan	A/Taiwan/2253/2009
HA	CY057041	H1N1	11/20/2009	United Ki	A/England/00380020/2009
HA	HQ228129	H1N1	11/20/2009	Finland	A/Finland/690/2009
HA	HQ228128	H1N1	11/20/2009	Finland	A/Finland/689/2009
HA	CY070625	H1N1	11/20/2009	United Ki	A/England/94840810/2009
HA	CY070596	H1N1	11/20/2009	United Ki	A/England/94800066/2009
HA	HM568032	H1N1	11/20/2009	United Ki	A/England/902/2009
HA	CY064438	H1N1	11/20/2009	China	A/Guangdong/1249/2009(H1N1)
HA	CY064422	H1N1	11/20/2009	China	A/Guangdong/1069/2009(H1N1)
HA	CY064440	H1N1	11/20/2009	China	A/Guangdong/1250/2009(H1N1)
HA	CY064454	H1N1	11/20/2009	China	A/Guangdong/1275/2009(H1N1)
HA	HM568016	H1N1	11/20/2009	United Ki	A/England/872/2009
HA	CY063598	H1N1	11/20/2009	Germany	A/Bonn/INS128/2009(H1N1)
HA	CY070598	H1N1	11/20/2009	United Ki	A/England/94800108/2009
HA	CY123273	H1N1	11/21/2009	Singapore	A/Singapore/GP4425/2009
HA	CY067039	H1N1	11/21/2009	Denmark	A/Aarhus/INS252/2009(H1N1)
HA	CY064424	H1N1	11/21/2009	China	A/Guangdong/1070/2009(H1N1)
HA	JF429392	H1N1	11/21/2009	France	A/Lyon/47.942/2009
HA	CY064450	H1N1	11/21/2009	China	A/Guangdong/1270/2009(H1N1)
HA	CY060463	H1N1	11/21/2009	South Ko	A/Gyeongnam/1819/2009(H1N1)
HA	CY123275	H1N1	11/21/2009	Singapore	A/Singapore/GP4428/2009
HA	KM213257	H1N1	11/21/2009	Oman	A/Oman/SQUH-4/2009
HA	HM189397	H1N1	11/21/2009	Russia	A/Ulyanovsk/CRIE-OAA/2009
HA	HM189387	H1N1	11/21/2009	Russia	A/Ivanovo/CRIE-STV1/2009
HA	JN171942	H1N1	11/21/2009	Canada	A/Montreal/MTL281027/2009
HA	CY120920	H1N1	11/21/2009	China	A/Guangdong/1104/2009
HA	CY088585	H1N1	11/21/2009	USA	A/Boston/694/2009
HA	CY089363	H1N1	11/21/2009	USA	A/Boston/691/2009
HA	HQ228090	H1N1	11/21/2009	Finland	A/Finland/645/2009
HA	CY070600	H1N1	11/21/2009	United Ki	A/England/851/2009
HA	AB704510	H1N1	11/21/2009	Japan	A/Yamaguchi/217/2009
HA	HM581919	H1N1	11/21/2009	Iran	A/Iran/15583/2009(H1N1)
HA	CY067031	H1N1	11/21/2009	Denmark	A/Aarhus/INS251/2009
HA	GU371270	H1N1	11/21/2009	China	A/Hunan Kaifu/SWL4142/2009(H1N1)
HA	CY060939	H1N1	11/21/2009	USA	A/Texas/JMS373/2009(H1N1)
HA	CY058826	H1N1	11/21/2009	China	A/Guangdong/1782/2009(H1N1)
HA	CY123277	H1N1	11/21/2009	Singapore	A/Singapore/GP4436/2009
HA	CY056723	H1N1	11/22/2009	USA	A/New York/6473/2009(H1N1)
HA	CY057886	H1N1	11/22/2009	USA	A/Wisconsin/629-D00533/2009(H1N1)
HA	CY070607	H1N1	11/22/2009	United Ki	A/England/885/2009
HA	CY067047	H1N1	11/22/2009	Denmark	A/Aarhus/INS253/2009(H1N1)
HA	HM581915	H1N1	11/22/2009	Iran	A/Iran/16273/2009(H1N1)
HA	CY058820	H1N1	11/22/2009	China	A/Guangdong/1331/2009(H1N1)
HA	CY064446	H1N1	11/22/2009	China	A/Guangdong/1262/2009(H1N1)
HA	KM213261	H1N1	11/22/2009	Oman	A/Oman/SQUH-10/2009
HA	CY128363	H1N1	11/22/2009	Viet Nam	A/Viet Nam/15032004/2009
HA	JN171903	H1N1	11/22/2009	Canada	A/Quebec/QC002211/2009
HA	CY084454	H1N1	11/22/2009	USA	A/New York/6418/2009
HA	CY120924	H1N1	11/22/2009	China	A/Guangdong/1105/2009
HA	HM581917	H1N1	11/22/2009	Iran	A/Iran/16245/2009(H1N1)
HA	CY064448	H1N1	11/22/2009	China	A/Guangdong/1266/2009(H1N1)
HA	CY070635	H1N1	11/22/2009	United Ki	A/Scotland/83/2009

HA	CY057071	H1N1	11/22/2009	United Ki	A/England/94920013/2009
HA	CY060466	H1N1	11/22/2009	South Ko	A/Gyeongnam/1820/2009(H1N1)
HA	CY057894	H1N1	11/22/2009	USA	A/Wisconsin/629-D00908/2009(H1N1)
HA	GU560016	H1N1	11/22/2009	Russia	A/Tomsk/06/2009(H1N1)
HA	CY123279	H1N1	11/23/2009	Singapore	A/Singapore/GP4440/2009
HA	CY073158	H1N1	11/23/2009	Nigeria	A/Lagos/WRAIR1982N/2009(H1N1)
HA	CY056731	H1N1	11/23/2009	USA	A/New York/6546/2009(H1N1)
HA	JF429395	H1N1	11/23/2009	France	A/Lyon/48.49/2009
HA	CY065800	H1N1	11/23/2009	Netherlar	A/Netherlands/2445b/2009
HA	CY070610	H1N1	11/23/2009	United Ki	A/England/880/2009
HA	CY075620	H1N1	11/23/2009	USA	A/Boston/698/2009(H1N1)
HA	HM189391	H1N1	11/23/2009	Russia	A/Murmansk/CRIE-SLV/2009
HA	HM189405	H1N1	11/23/2009	Russia	A/Ulyanovsk/CRIE-BAA/2009
HA	HM189396	H1N1	11/23/2009	Russia	A/Moscow/CRIE-CHSN/2009
HA	CY066903	H1N1	11/23/2009	USA	A/District of Columbia/INS232/2009
HA	CY120921	H1N1	11/23/2009	China	A/Guangdong/1106/2009
HA	KP019834	H1N1	11/23/2009	China	A/FuZhou/SWL12025/2009
HA	CY066943	H1N1	11/23/2009	Denmark	A/Aarhus/INS237/2009(H1N1)
HA	CY070615	H1N1	11/23/2009	United Ki	A/England/94840760/2009
HA	CY070624	H1N1	11/23/2009	United Ki	A/England/901/2009
HA	CY070614	H1N1	11/23/2009	United Ki	A/England/94840759/2009
HA	CY058834	H1N1	11/23/2009	China	A/Guangdong/2282/2009(H1N1)
HA	CY070621	H1N1	11/23/2009	United Ki	A/England/94840792/2009
HA	CY072246	H1N1	11/23/2009	Denmark	A/Odense/INS307/2009(H1N1)
HA	CY123862	H1N1	11/23/2009	Singapore	A/Singapore/ON2344/2009
HA	HM568008	H1N1	11/23/2009	United Ki	A/England/854/2009
HA	HM568024	H1N1	11/23/2009	United Ki	A/England/862/2009
HA	HM189398	H1N1	11/23/2009	Russia	A/Moscow oblast/CRIE-KMA/2009
HA	HM189408	H1N1	11/23/2009	Russia	A/Moscow/CRIE-TYD/2009
HA	CY070620	H1N1	11/23/2009	United Ki	A/England/860/2009
HA	CY069146	H1N1	11/23/2009	Germany	A/Frankfurt/INS301/2009(H1N1)
HA	CY056843	H1N1	11/23/2009	USA	A/New York/6868/2009(H1N1)
HA	CY083910	H1N1	11/23/2009	Denmark	A/Aalborg/INS132/2009(H1N1)
HA	HM072424	H1N1	11/23/2009	Taiwan	A/Taiwan/7949/2009
HA	HM567960	H1N1	11/23/2009	United Ki	A/England/865/2009
HA	CY070617	H1N1	11/23/2009	United Ki	A/England/94840777/2009
HA	CY070630	H1N1	11/23/2009	United Ki	A/England/94860685/2009
HA	CY070599	H1N1	11/23/2009	United Ki	A/England/879/2009
HA	CY070613	H1N1	11/23/2009	United Ki	A/England/94840077/2009
HA	CY123268	H1N1	11/23/2009	Singapore	A/Singapore/GP4419/2009
HA	CY123859	H1N1	11/23/2009	Singapore	A/Singapore/ON2326/2009
HA	CY056755	H1N1	11/23/2009	USA	A/New York/6663/2009(H1N1)
HA	CY058716	H1N1	11/23/2009	USA	A/Texas/JMS375/2009(H1N1)
HA	CY058822	H1N1	11/23/2009	China	A/Guangdong/1748/2009(H1N1)
HA	CY057910	H1N1	11/23/2009	USA	A/Wisconsin/629-D00134/2009(H1N1)
HA	CY057918	H1N1	11/23/2009	USA	A/Wisconsin/629-D01330/2009(H1N1)
HA	CY057902	H1N1	11/23/2009	USA	A/Wisconsin/629-D02448/2009(H1N1)
HA	CY066887	H1N1	11/23/2009	USA	A/District of Columbia/INS230/2009(I
HA	CY123281	H1N1	11/23/2009	Singapore	A/Singapore/GP4451/2009
HA	AB704470	H1N1	11/24/2009	Japan	A/Tochigi/445/2009
HA	CY070618	H1N1	11/24/2009	United Ki	A/England/863/2009
HA	CY069122	H1N1	11/24/2009	Spain	A/Madrid/INS298/2009(H1N1)
HA	CY066975	H1N1	11/24/2009	Denmark	A/Aarhus/INS242/2009(H1N1)
HA	CY066655	H1N1	11/24/2009	Spain	A/Madrid/INS281/2009(H1N1)

HA	CY066271	H1N1	11/24/2009	USA	A/California/VRDL112/2009(H1N1)
HA	HQ263288	H1N1	11/24/2009	USA	A/Bethesda/NIH107-D31/2009
HA	CY066255	H1N1	11/24/2009	USA	A/California/VRDL110/2009(H1N1)
HA	HM189376	H1N1	11/24/2009	Russia	A/Ivanovo/CRIE-RNA/2009
HA	CY123868	H1N1	11/24/2009	Singapore	A/Singapore/ON2370/2009
HA	HM568000	H1N1	11/24/2009	United Ki	A/England/900/2009
HA	HM581911	H1N1	11/24/2009	Iran	A/Iran/16743/2009(H1N1)
HA	KT181126	H1N1	11/24/2009	Singapore	A/Singapore/DMS53/2009
HA	CY066263	H1N1	11/24/2009	USA	A/California/VRDL111/2009(H1N1)
HA	KC780816	H1N1	11/24/2009	USA	A/Colorado/15/2009
HA	CY070632	H1N1	11/24/2009	United Ki	A/England/94860699/2009
HA	CY070641	H1N1	11/24/2009	United Ki	A/England/920/2009
HA	GU371271	H1N1	11/24/2009	China	A/Hunan Changsha/SWL4346/2009(H1N1)
HA	KC780707	H1N1	11/24/2009	USA	A/California/41/2009
HA	HM189375	H1N1	11/24/2009	Russia	A/Moscow oblast/CRIE-ZNF/2009
HA	HM189383	H1N1	11/24/2009	Russia	A/Tambov/CRIE-SPN/2009
HA	HM189374	H1N1	11/24/2009	Russia	A/Astrakhan/CRIE-CHRM/2009
HA	CY070736	H1N1	11/24/2009	United Ki	A/England/909/2009
HA	CY066279	H1N1	11/24/2009	USA	A/California/VRDL113/2009(H1N1)
HA	CY057934	H1N1	11/24/2009	USA	A/Wisconsin/629-D00968/2009(H1N1)
HA	CY066247	H1N1	11/24/2009	USA	A/California/VRDL109/2009(H1N1)
HA	CY062691	H1N1	11/24/2009	Denmark	A/Aarhus/INS116/2009(H1N1)
HA	CY084462	H1N1	11/24/2009	USA	A/New York/6537/2009
HA	HQ228089	H1N1	11/24/2009	Finland	A/Finland/644/2009
HA	CY070619	H1N1	11/24/2009	United Ki	A/England/864/2009
HA	CY070616	H1N1	11/24/2009	United Ki	A/England/94840765/2009
HA	CY070622	H1N1	11/24/2009	United Ki	A/England/861/2009
HA	CY070640	H1N1	11/24/2009	United Ki	A/England/94880537/2009
HA	CY070623	H1N1	11/24/2009	United Ki	A/England/94840801/2009
HA	CY070633	H1N1	11/24/2009	United Ki	A/England/899/2009
HA	CY092438	H1N1	11/24/2009	Australia	A/Sydney/DD3-02/2009(H1N1)
HA	JN171943	H1N1	11/24/2009	Canada	A/Montreal/MTL283502/2009
HA	CY056779	H1N1	11/24/2009	USA	A/New York/6675/2009(H1N1)
HA	CY056747	H1N1	11/24/2009	USA	A/New York/6607/2009(H1N1)
HA	HM581913	H1N1	11/24/2009	Iran	A/Iran/16677/2009(H1N1)
HA	CY064432	H1N1	11/24/2009	China	A/Guangdong/1083/2009(H1N1)
HA	CY066951	H1N1	11/24/2009	Denmark	A/Aarhus/INS238/2009(H1N1)
HA	HQ228131	H1N1	11/24/2009	Finland	A/Finland/692/2009
HA	CY066711	H1N1	11/24/2009	Austria	A/Vienna/INS293/2009(H1N1)
HA	CY058828	H1N1	11/24/2009	China	A/Guangdong/1998/2009(H1N1)
HA	CY058724	H1N1	11/24/2009	USA	A/Texas/JMS376/2009(H1N1)
HA	CY057926	H1N1	11/24/2009	USA	A/Wisconsin/629-D01190/2009(H1N1)
HA	HQ228130	H1N1	11/24/2009	Finland	A/Finland/691/2009
HA	CY072254	H1N1	11/24/2009	Denmark	A/Odense/INS308/2009(H1N1)
HA	CY066911	H1N1	11/24/2009	USA	A/Pensacola/INS233/2009
HA	CY071247	H1N1	11/24/2009	Spain	A/Madrid/INS407/2009(H1N1)
HA	JQ173164	H1N1	11/25/2009	Finland	A/Helsinki/P18/2009
HA	HQ168410	H1N1	11/25/2009	China	A/Jinhua/01/2009(H1N1)
HA	CY056819	H1N1	11/25/2009	USA	A/New York/6810/2009(H1N1)
HA	CY056827	H1N1	11/25/2009	USA	A/New York/6846/2009(H1N1)
HA	CY067007	H1N1	11/25/2009	Germany	A/Munich/INS247/2009
HA	CY070631	H1N1	11/25/2009	United Ki	A/England/868/2009
HA	CY070642	H1N1	11/25/2009	United Ki	A/England/921/2009
HA	CY070627	H1N1	11/25/2009	United Ki	A/England/867/2009

HA	CY070628	H1N1	11/25/2009	United Ki	A/England/94860341/2009
HA	JN171904	H1N1	11/25/2009	Canada	A/Quebec/QC002461/2009
HA	KC780943	H1N1	11/25/2009	USA	A/Washington/72/2009
HA	CY058824	H1N1	11/25/2009	China	A/Guangdong/1759/2009(H1N1)
HA	AB704438	H1N1	11/25/2009	Japan	A/Gunma/267/2009
HA	CY123864	H1N1	11/25/2009	Singapore	A/Singapore/ON2349/2009
HA	HM567952	H1N1	11/25/2009	United Ki	A/England/857/2009
HA	CY072222	H1N1	11/25/2009	Germany	A/Frankfurt/INS302/2009(H1N1)
HA	AB704446	H1N1	11/25/2009	Japan	A/Gunma/287/2009
HA	HQ228132	H1N1	11/25/2009	Finland	A/Finland/693/2009
HA	HM568040	H1N1	11/25/2009	United Ki	A/England/898/2009
HA	HM189404	H1N1	11/25/2009	Russia	A/Ivanovo/CRIE-KOE/2009
HA	HM189320	H1N1	11/25/2009	Russia	A/Arkhangelsk/CRIE-GNY/2009
HA	HM101145	H1N1	11/25/2009	Russia	A/Tver/IIV-183/2009(H1N1)
HA	JF429393	H1N1	11/25/2009	France	A/Lyon/48.425/2009
HA	CY083776	H1N1	11/25/2009	Denmark	A/Aalborg/INS282/2009
HA	CY083761	H1N1	11/25/2009	Denmark	A/Aarhus/INS239/2009
HA	CY057072	H1N1	11/25/2009	United Ki	A/England/94920015/2009
HA	CY067183	H1N1	11/25/2009	Austria	A/Vienna/INS294/2009
HA	CY070629	H1N1	11/25/2009	United Ki	A/England/94860684/2009
HA	CY070738	H1N1	11/25/2009	United Ki	A/England/892/2009
HA	HM567992	H1N1	11/25/2009	United Ki	A/England/859/2009
HA	GU562450	H1N1	11/25/2009	Russia	A/Perm/01/2009(H1N1)
HA	CY066687	H1N1	11/25/2009	Denmark	A/Hvidovre/INS289/2009(H1N1)
HA	CY058836	H1N1	11/25/2009	China	A/Guangdong/2361/2009(H1N1)
HA	CY123286	H1N1	11/25/2009	Singapore	A/Singapore/GP4539/2009
HA	CY072230	H1N1	11/26/2009	Germany	A/Frankfurt/INS303/2009(H1N1)
HA	CY073749	H1N1	11/26/2009	Denmark	A/Copenhagen/INS434/2009(H1N1)
HA	CY070639	H1N1	11/26/2009	United Ki	A/England/919/2009
HA	CY070739	H1N1	11/26/2009	United Ki	A/England/888/2009
HA	CY058740	H1N1	11/26/2009	USA	A/Texas/JMS378/2009(H1N1)
HA	CY060472	H1N1	11/26/2009	South Ko	A/Seoul/1829/2009(H1N1)
HA	KP317211	H1N1	11/26/2009	India	A/Punjab/007/2009
HA	KP317213	H1N1	11/26/2009	India	A/Rajasthan/009/2009
HA	KT181127	H1N1	11/26/2009	Singapore	A/Singapore/DMS54/2009
HA	CY083729	H1N1	11/26/2009	Germany	A/Bochum/INS120/2009(H1N1)
HA	GU371272	H1N1	11/26/2009	China	A/Hunan Furong/SWL4224/2009(H1N1)
HA	HM189361	H1N1	11/26/2009	Russia	A/Volgograd/CRIE-VAA/2009
HA	HM189368	H1N1	11/26/2009	Russia	A/Tver/CRIE-ZC/2009
HA	KC190043	H1N1	11/26/2009	Sri Lanka	A/Sri Lanka/24206/2009
HA	CY056739	H1N1	11/26/2009	USA	A/New York/6571/2009(H1N1)
HA	CY066967	H1N1	11/26/2009	Denmark	A/Aarhus/INS241/2009(H1N1)
HA	CY070637	H1N1	11/26/2009	United Ki	A/England/922/2009
HA	CY070737	H1N1	11/26/2009	United Ki	A/England/908/2009
HA	CY070638	H1N1	11/26/2009	United Ki	A/England/918/2009
HA	CY088613	H1N1	11/26/2009	Germany	A/Frankfurt/INS93/2009
HA	CY057942	H1N1	11/26/2009	USA	A/Wisconsin/629-D00629/2009(H1N1)
HA	CY066959	H1N1	11/26/2009	Denmark	A/Aarhus/INS240/2009(H1N1)
HA	CY062803	H1N1	11/26/2009	Austria	A/Vienna/INS142/2009(H1N1)
HA	CY060947	H1N1	11/26/2009	USA	A/Texas/JMS379/2009(H1N1)
HA	CY062755	H1N1	11/26/2009	Spain	A/Madrid/INS131/2009(H1N1)
HA	CY058732	H1N1	11/26/2009	USA	A/Texas/JMS377/2009(H1N1)
HA	CY069130	H1N1	11/26/2009	Spain	A/Madrid/INS299/2009(H1N1)
HA	AB704502	H1N1	11/27/2009	Japan	A/Yamagata/674/2009

HA	CY075628	H1N1	11/27/2009	USA	A/Boston/701/2009(H1N1)
HA	CY057950	H1N1	11/27/2009	USA	A/Wisconsin/629-D00050/2009(H1N1)
HA	CY060955	H1N1	11/27/2009	USA	A/Texas/JMS380/2009(H1N1)
HA	CY054546	H1N1	11/27/2009	Mongolia	A/Unmugobi/9586/2009(H1N1)
HA	AB558535	H1N1	11/27/2009	Japan	A/Nagasaki/HA-64/2009(H1N1)
HA	CY062699	H1N1	11/27/2009	Denmark	A/Aarhus/INS118/2009(H1N1)
HA	JN171944	H1N1	11/27/2009	Canada	A/Montreal/MTL287409/2009
HA	KC190042	H1N1	11/27/2009	Sri Lanka	A/Sri Lanka/2005/2009
HA	CY058832	H1N1	11/27/2009	China	A/Guangdong/2281/2009(H1N1)
HA	JN171905	H1N1	11/27/2009	Canada	A/Quebec/QC002598/2009
HA	KC780297	H1N1	11/27/2009	USA	A/Kansas/27/2009
HA	CY064456	H1N1	11/27/2009	China	A/Guangdong/1738/2009(H1N1)
HA	CY057958	H1N1	11/27/2009	USA	A/Wisconsin/629-D00970/2009(H1N1)
HA	CY066695	H1N1	11/27/2009	Denmark	A/Hvidovre/INS290/2009(H1N1)
HA	CY064406	H1N1	11/27/2009	China	A/Weidong/SWL5165/2009(H1N1)
HA	KC781064	H1N1	11/27/2009	USA	A/Virginia/25/2009
HA	GU592897	H1N1	11/28/2009	Kyrgyzsta	A/Bishkek/03/2009(H1N1)
HA	CY060971	H1N1	11/28/2009	USA	A/Texas/JMS382/2009(H1N1)
HA	KR861657	H1N1	11/28/2009	India	A/Bangalore/10519-13/2009
HA	CY060963	H1N1	11/28/2009	USA	A/Texas/JMS381/2009(H1N1)
HA	KC781103	H1N1	11/28/2009	USA	A/Washington/69/2009
HA	CY067055	H1N1	11/28/2009	Denmark	A/Aarhus/INS254/2009(H1N1)
HA	CY070740	H1N1	11/28/2009	United Ki	A/England/912/2009
HA	HQ228094	H1N1	11/28/2009	Finland	A/Finland/649/2009
HA	CY120917	H1N1	11/29/2009	China	A/Guangdong/1101/2009
HA	CY056803	H1N1	11/29/2009	USA	A/New York/6808/2009(H1N1)
HA	CY060979	H1N1	11/29/2009	USA	A/Texas/JMS383/2009(H1N1)
HA	CY056851	H1N1	11/29/2009	USA	A/New York/6909/2009(H1N1)
HA	CY120918	H1N1	11/29/2009	China	A/Guangdong/1102/2009
HA	CY073238	H1N1	11/29/2009	Serbia	A/Belgrade/WRAIR2385N/2009(H1N1)
HA	CY088593	H1N1	11/29/2009	USA	A/Boston/702/2009
HA	CY057974	H1N1	11/29/2009	USA	A/Wisconsin/629-D01434/2009(H1N1)
HA	CY057966	H1N1	11/29/2009	USA	A/Wisconsin/629-D01528/2009(H1N1)
HA	CY064405	H1N1	11/29/2009	China	A/Ruzhou/SWL564/2009(H1N1)
HA	CY073246	H1N1	11/29/2009	Serbia	A/Belgrade/WRAIR2385T/2009(H1N1)
HA	JN171945	H1N1	11/29/2009	Canada	A/Montreal/MTL287826/2009
HA	CY065776	H1N1	11/30/2009	Netherlar	A/Netherlands/2635b/2009
HA	CY069218	H1N1	11/30/2009	Germany	A/Munich/INS363/2009(H1N1)
HA	CY072238	H1N1	11/30/2009	Germany	A/Frankfurt/INS305/2009(H1N1)
HA	CY069138	H1N1	11/30/2009	Spain	A/Madrid/INS300/2009(H1N1)
HA	CY070612	H1N1	11/30/2009	United Ki	A/England/903/2009
HA	KT241013	H1N1	11/30/2009	India	A/Indore/10/2009
HA	CY123873	H1N1	11/30/2009	Singapore	A/Singapore/ON2378/2009
HA	KJ856074	H1N1	11/30/2009	Sri Lanka	A/Sri Lanka/2008/2009
HA	KT181129	H1N1	11/30/2009	Singapore	A/Singapore/DMS55/2009
HA	CY083787	H1N1	11/30/2009	Denmark	A/Odense/INS309/2009(H1N1)
HA	CY063614	H1N1	11/30/2009	Germany	A/Cologne/INS136/2009(H1N1)
HA	CY123866	H1N1	11/30/2009	Singapore	A/Singapore/ON2365/2009
HA	HM189390	H1N1	11/30/2009	Russia	A/Perm/CRIE-ZTS/2009
HA	HM189409	H1N1	11/30/2009	Russia	A/Tambov/CRIE-LLA/2009
HA	CY083690	H1N1	11/30/2009	Spain	A/Terrassa/INS94/2009(H1N1)
HA	CY066663	H1N1	11/30/2009	Germany	A/Cologne/INS286/2009(H1N1)
HA	CY056811	H1N1	11/30/2009	USA	A/New York/6809/2009(H1N1)
HA	CY056763	H1N1	11/30/2009	USA	A/New York/6668/2009(H1N1)

HA	CY060987	H1N1	11/30/2009	USA	A/Texas/JMS384/2009(H1N1)
HA	JQ365001	H1N1	12/2009	China	A/Hangzhou/1748/2009
HA	HM204577	H1N1	12/2009	India	A/Pune/NIV20007/2009
HA	HM204579	H1N1	12/2009	India	A/Pune/NIV21123/2009
HA	HM204578	H1N1	12/2009	India	A/Pune/NIV21115/2009
HA	CY067629	H1N1	12/2009	China	A/Qingdao/1780/2009
HA	CY067626	H1N1	12/2009	China	A/Qingdao/1775/2009
HA	CY067628	H1N1	12/2009	China	A/Qingdao/1779/2009
HA	AB745241	H1N1	12/2009	Japan	A/Tottori/ST442/2009
HA	AB745243	H1N1	12/2009	Japan	A/Tottori/ST450/2009
HA	AB745242	H1N1	12/2009	Japan	A/Tottori/ST444/2009
HA	AB745234	H1N1	12/2009	Japan	A/Tottori/ST422/2009
HA	AB745233	H1N1	12/2009	Japan	A/Tottori/ST417/2009
HA	CY071946	H1N1	12/2009	Egypt	A/Egypt/N14328/2009(H1N1)
HA	CY071944	H1N1	12/2009	Egypt	A/Egypt/N14315/2009(H1N1)
HA	AB745245	H1N1	12/2009	Japan	A/Tottori/ST455/2009
HA	GU968908	H1N1	12/2009	China	A/Beijing/22811/2009(H1N1)
HA	HM138491	H1N1	12/2009	Germany	A/Germany-MV/HGW4/2009(H1N1)
HA	AB745200	H1N1	12/2009	Japan	A/Tottori/ST342/2009
HA	AB745216	H1N1	12/2009	Japan	A/Tottori/ST380/2009
HA	AB745235	H1N1	12/2009	Japan	A/Tottori/ST426/2009
HA	CY067625	H1N1	12/2009	China	A/Qingdao/1773/2009
HA	CY067627	H1N1	12/2009	China	A/Qingdao/1776/2009
HA	GU968907	H1N1	12/2009	China	A/Beijing/22808/2009(H1N1)
HA	AB745198	H1N1	12/2009	Japan	A/Tottori/ST339/2009
HA	AB745199	H1N1	12/2009	Japan	A/Tottori/ST340/2009
HA	CY071945	H1N1	12/2009	Egypt	A/Egypt/N14325/2009(H1N1)
HA	AB745215	H1N1	12/2009	Japan	A/Tottori/ST379/2009
HA	AB745223	H1N1	12/2009	Japan	A/Tottori/ST397/2009
HA	AB745240	H1N1	12/2009	Japan	A/Tottori/ST438/2009
HA	CY071942	H1N1	12/2009	Egypt	A/Egypt/N14313/2009(H1N1)
HA	AB745205	H1N1	12/2009	Japan	A/Tottori/ST354/2009
HA	AB745210	H1N1	12/2009	Japan	A/Tottori/ST369/2009
HA	AB745238	H1N1	12/2009	Japan	A/Tottori/ST430/2009
HA	AB745211	H1N1	12/2009	Japan	A/Tottori/ST370/2009
HA	AB745214	H1N1	12/2009	Japan	A/Tottori/ST378/2009
HA	AB745224	H1N1	12/2009	Japan	A/Tottori/ST402/2009
HA	CY071943	H1N1	12/2009	Egypt	A/Egypt/N14314/2009(H1N1)
HA	GU560024	H1N1	12/2009	Kazakhsta	A/Taraz/01/2009(H1N1)
HA	HM138493	H1N1	12/2009	Germany	A/Germany-MV/HGW6/2009(H1N1)
HA	AB745222	H1N1	12/2009	Japan	A/Tottori/ST393/2009
HA	AB745227	H1N1	12/2009	Japan	A/Tottori/ST408/2009
HA	AB745228	H1N1	12/2009	Japan	A/Tottori/ST409/2009
HA	JQ364999	H1N1	12/2009	China	A/Hangzhou/1732/2009
HA	JQ365000	H1N1	12/2009	China	A/Hangzhou/1733/2009
HA	AB539048	H1N1	12/2009	Japan	A/Nagasaki/NU-3/2009(H1N1)
HA	AB745244	H1N1	12/2009	Japan	A/Tottori/ST452/2009
HA	AB745207	H1N1	12/2009	Japan	A/Tottori/ST359/2009
HA	JN381257	H1N1	12/2009	Taiwan	A/Taiwan/97487/2009
HA	AB745239	H1N1	12/2009	Japan	A/Tottori/ST431/2009
HA	AB745225	H1N1	12/2009	Japan	A/Tottori/ST405/2009
HA	AB745226	H1N1	12/2009	Japan	A/Tottori/ST407/2009
HA	AB745236	H1N1	12/2009	Japan	A/Tottori/ST428/2009
HA	AB745237	H1N1	12/2009	Japan	A/Tottori/ST429/2009

HA	AB745201	H1N1	12/2009	Japan	A/Tottori/ST344/2009
HA	AB745206	H1N1	12/2009	Japan	A/Tottori/ST357/2009
HA	AB745209	H1N1	12/2009	Japan	A/Tottori/ST365/2009
HA	AB745213	H1N1	12/2009	Japan	A/Tottori/ST375/2009
HA	AB745218	H1N1	12/2009	Japan	A/Tottori/ST383/2009
HA	AB745221	H1N1	12/2009	Japan	A/Tottori/ST390/2009
HA	AB745229	H1N1	12/2009	Japan	A/Tottori/ST410/2009
HA	AB745230	H1N1	12/2009	Japan	A/Tottori/ST412/2009
HA	AB745231	H1N1	12/2009	Japan	A/Tottori/ST413/2009
HA	AB745232	H1N1	12/2009	Japan	A/Tottori/ST416/2009
HA	AB745197	H1N1	12/2009	Japan	A/Tottori/ST338/2009
HA	AB745202	H1N1	12/2009	Japan	A/Tottori/ST345/2009
HA	AB745203	H1N1	12/2009	Japan	A/Tottori/ST350/2009
HA	AB745208	H1N1	12/2009	Japan	A/Tottori/ST363/2009
HA	AB745212	H1N1	12/2009	Japan	A/Tottori/ST374/2009
HA	AB745217	H1N1	12/2009	Japan	A/Tottori/ST381/2009
HA	AB745219	H1N1	12/2009	Japan	A/Tottori/ST386/2009
HA	AB745220	H1N1	12/2009	Japan	A/Tottori/ST388/2009
HA	JQ365002	H1N1	12/2009	China	A/Hangzhou/1756/2009
HA	JN381250	H1N1	12/2009	Taiwan	A/Taiwan/97184/2009
HA	JN381258	H1N1	12/2009	Taiwan	A/Taiwan/97553/2009
HA	JN381251	H1N1	12/2009	Taiwan	A/Taiwan/97199/2009
HA	JN381253	H1N1	12/2009	Taiwan	A/Taiwan/97341/2009
HA	JN381252	H1N1	12/2009	Taiwan	A/Taiwan/97241/2009
HA	AB745204	H1N1	12/2009	Japan	A/Tottori/ST352/2009
HA	JN381256	H1N1	12/2009	Taiwan	A/Taiwan/97394/2009
HA	JN381255	H1N1	12/2009	Taiwan	A/Taiwan/97382/2009
HA	JN381254	H1N1	12/2009	Taiwan	A/Taiwan/97351/2009
HA	CY073134	H1N1	12/01/2009	Mexico	A/Mexico City/WRAIR1779T/2009(H1N1)
HA	CY055169	H1N1	12/01/2009	Mongolia	A/Dundgobi/9746/2009(H1N1)
HA	CY071826	H1N1	12/01/2009	Ethiopia	A/Addis Ababa/WR2848T/2009(H1N1)
HA	CY073150	H1N1	12/01/2009	Mexico	A/Mexico City/WRAIR1786T/2009(H1N1)
HA	KC780489	H1N1	12/01/2009	USA	A/Illinois/25/2009
HA	KC781111	H1N1	12/01/2009	USA	A/New Hampshire/27/2009
HA	HM189371	H1N1	12/01/2009	Russia	A/Tambov/CRIE-FOS/2009
HA	CY056771	H1N1	12/01/2009	USA	A/New York/6669/2009(H1N1)
HA	KC780182	H1N1	12/01/2009	USA	A/Montana/22/2009
HA	CY071818	H1N1	12/01/2009	Ethiopia	A/Addis Ababa/WR2848N/2009(H1N1)
HA	KC190044	H1N1	12/01/2009	Sri Lanka	A/Sri Lanka/24588/2009
HA	KM203130	H1N1	12/01/2009	India	A/Kerala/RGCBC291/2009
HA	KJ856082	H1N1	12/01/2009	Sri Lanka	A/Sri Lanka/2009/2009
HA	CY063771	H1N1	12/01/2009	Singapore	A/Singapore/GP4556/2009(H1N1)
HA	CY070744	H1N1	12/01/2009	United Ki	A/England/895/2009
HA	CY060481	H1N1	12/01/2009	South Kor	A/Daegu/1853/2009(H1N1)
HA	CY060475	H1N1	12/01/2009	South Kor	A/Daejeon/1840/2009(H1N1)
HA	CY065840	H1N1	12/01/2009	Netherlan	A/Netherlands/2636/2009
HA	CY070645	H1N1	12/01/2009	United Ki	A/Scotland/99/2009
HA	HQ228141	H1N1	12/01/2009	Finland	A/Finland/705/2009
HA	CY057982	H1N1	12/01/2009	USA	A/Wisconsin/629-D01412/2009(H1N1)
HA	CY070742	H1N1	12/01/2009	United Ki	A/England/911/2009
HA	CY070743	H1N1	12/01/2009	United Ki	A/England/894/2009
HA	CY073497	H1N1	12/01/2009	Germany	A/Frankfurt/INS304/2009(H1N1)
HA	CY070746	H1N1	12/02/2009	United Ki	A/England/896/2009
HA	KC780460	H1N1	12/02/2009	USA	A/North Dakota/16/2009

HA	CY071079	H1N1	12/02/2009	Germany	A/Bochum/INS375/2009(H1N1)
HA	CY056979	H1N1	12/02/2009	Denmark	A/Copenhagen/INS98/2009(H1N1)
HA	CY065896	H1N1	12/02/2009	Netherlar	A/Netherlands/2822/2009
HA	CY060469	H1N1	12/02/2009	South Kor	A/Seoul/2883/2009(H1N1)
HA	CY071087	H1N1	12/02/2009	Germany	A/Bochum/INS376/2009(H1N1)
HA	CY057990	H1N1	12/02/2009	USA	A/Wisconsin/629-D00935/2009(H1N1)
HA	KC780685	H1N1	12/02/2009	USA	A/California/42/2009
HA	CY128427	H1N1	12/02/2009	Viet Nam	A/Viet Nam/12032005/2009
HA	CY070644	H1N1	12/02/2009	United Ki	A/England/1048/2009
HA	CY071434	H1N1	12/02/2009	Kyrgyzsta	A/Bishkek/WR0881T/2009(H1N1)
HA	KP019835	H1N1	12/02/2009	China	A/FuZhou/SWL12139/2009
HA	CY056835	H1N1	12/02/2009	USA	A/New York/6864/2009(H1N1)
HA	CY071191	H1N1	12/02/2009	Germany	A/Frankfurt/INS399/2009(H1N1)
HA	AB704518	H1N1	12/02/2009	Japan	A/Yamaguchi/247/2009
HA	CY063606	H1N1	12/02/2009	Denmark	A/Aalborg/INS133/2009(H1N1)
HA	CY057998	H1N1	12/02/2009	USA	A/Wisconsin/629-D02299/2009(H1N1)
HA	CY063773	H1N1	12/02/2009	Singapore	A/Singapore/GP4581/2009(H1N1)
HA	CY072270	H1N1	12/02/2009	Poland	A/Warsaw/INS311/2009(H1N1)
HA	CY062763	H1N1	12/02/2009	Spain	A/Madrid/INS134/2009(H1N1)
HA	JN171906	H1N1	12/02/2009	Canada	A/Quebec/QC002766/2009
HA	HM568120	H1N1	12/02/2009	United Ki	A/England/941/2009
HA	CY072262	H1N1	12/02/2009	Poland	A/Warsaw/INS310/2009(H1N1)
HA	CY075636	H1N1	12/02/2009	USA	A/Boston/703/2009(H1N1)
HA	CY073104	H1N1	12/02/2009	Taiwan	A/Taipei/WRAIR1470T/2009(H1N1)
HA	CY070745	H1N1	12/02/2009	United Ki	A/England/897/2009
HA	KR861659	H1N1	12/03/2009	India	A/Bangalore/10698-02/2009
HA	KC781977	H1N1	12/03/2009	USA	A/Nevada/20/2009
HA	CY072278	H1N1	12/03/2009	Poland	A/Warsaw/INS312/2009(H1N1)
HA	CY083825	H1N1	12/03/2009	Poland	A/Wroclaw/INS435/2009
HA	KC782229	H1N1	12/03/2009	USA	A/Illinois/33/2009
HA	KC780789	H1N1	12/03/2009	USA	A/Utah/55/2009
HA	CY123879	H1N1	12/03/2009	Singapore	A/Singapore/ON2383/2009
HA	KR861658	H1N1	12/03/2009	India	A/Bangalore/10695-10/2009
HA	CY073765	H1N1	12/03/2009	Spain	A/Barcelona/INS378/2009(H1N1)
HA	KT181130	H1N1	12/03/2009	Singapore	A/Singapore/DMS56/2009
HA	AB558536	H1N1	12/03/2009	Japan	A/Nagasaki/HA-65/2009(H1N1)
HA	CY072286	H1N1	12/03/2009	Poland	A/Warsaw/INS313/2009(H1N1)
HA	CY083918	H1N1	12/03/2009	Poland	A/Warsaw/INS147/2009(H1N1)
HA	AB601604	H1N1	12/03/2009	Japan	A/Yamagata/752/2009
HA	CY075644	H1N1	12/03/2009	USA	A/Boston/704/2009(H1N1)
HA	CY062827	H1N1	12/03/2009	Poland	A/Warsaw/INS146/2009(H1N1)
HA	CY060995	H1N1	12/04/2009	USA	A/Texas/JMS385/2009(H1N1)
HA	CY069226	H1N1	12/04/2009	Germany	A/Munich/INS364/2009(H1N1)
HA	CY083559	H1N1	12/04/2009	Hungary	A/Budapest/WRAIR2411N/2009
HA	GU727825	H1N1	12/04/2009	Russia	A/IIV-Anadyr/177/2009(H1N1)
HA	JN187135	H1N1	12/04/2009	Taiwan	A/Taiwan/6341/2009
HA	CY066303	H1N1	12/04/2009	USA	A/California/VRDL116/2009(H1N1)
HA	KC780614	H1N1	12/04/2009	USA	A/Alabama/08/2009
HA	KC780303	H1N1	12/04/2009	USA	A/Pennsylvania/31/2009
HA	KP317217	H1N1	12/04/2009	India	A/Jammu & Kashmir/013/2009
HA	CY065784	H1N1	12/04/2009	Netherlar	A/Netherlands/2629/2009
HA	MF535109	H1N1	12/04/2009	India	A/Jodhpur/526/2009
HA	KC780781	H1N1	12/04/2009	USA	A/Alaska/47/2009
HA	CY062771	H1N1	12/04/2009	Spain	A/Madrid/INS135/2009(H1N1)

HA	CY057350	H1N1	12/04/2009	Germany	A/Hamburg/INS92/2009(H1N1)
HA	CY056795	H1N1	12/04/2009	USA	A/New York/6776/2009(H1N1)
HA	CY066311	H1N1	12/04/2009	USA	A/California/VRDL117/2009(H1N1)
HA	CY066319	H1N1	12/04/2009	USA	A/California/VRDL118/2009(H1N1)
HA	CY066287	H1N1	12/04/2009	USA	A/California/VRDL114/2009(H1N1)
HA	HM157254	H1N1	12/04/2009	Russia	A/Yaroslavl/IIV-196/2009(H1N1)
HA	CY070646	H1N1	12/04/2009	United Ki	A/England/1050/2009
HA	CY088601	H1N1	12/04/2009	USA	A/Boston/705/2009
HA	CY066295	H1N1	12/04/2009	USA	A/California/VRDL115/2009(H1N1)
HA	JN171907	H1N1	12/05/2009	Canada	A/Quebec/QC002857/2009
HA	CY056787	H1N1	12/05/2009	USA	A/New York/6775/2009(H1N1)
HA	AB704526	H1N1	12/05/2009	Japan	A/Yamaguchi/248/2009
HA	HM104477	H1N1	12/05/2009	Russia	A/Yaroslavl/IIV-198/2009(H1N1)
HA	CY189541	H1N1	12/05/2009	Japan	A/Yokohama/UT-K1205T/2009
HA	HM072425	H1N1	12/05/2009	Taiwan	A/Taiwan/2342/2009
HA	CY075902	H1N1	12/05/2009	India	A/Pune/NIV19609/2009
HA	JQ714165	H1N1	12/05/2009	China	A/Tianjinninghe/SWL49/2009
HA	KC780370	H1N1	12/06/2009	USA	A/Florida/30/2009
HA	KC781018	H1N1	12/06/2009	USA	A/Arizona/20/2009
HA	KC780599	H1N1	12/06/2009	USA	A/Arizona/20/2009
HA	CY123884	H1N1	12/06/2009	Singapore	A/Singapore/ON2394/2009
HA	KC780188	H1N1	12/06/2009	USA	A/Maine/15/2009
HA	CY084446	H1N1	12/06/2009	USA	A/New York/6902/2009
HA	KP317208	H1N1	12/06/2009	India	A/West Bengal/004/2009
HA	CY061003	H1N1	12/06/2009	USA	A/Texas/JMS386/2009(H1N1)
HA	CY061938	H1N1	12/06/2009	USA	A/New York/6941/2009(H1N1)
HA	HQ228142	H1N1	12/06/2009	Finland	A/Finland/708/2009
HA	CY065075	H1N1	12/06/2009	USA	A/New York/7421/2009(H1N1)
HA	CY072294	H1N1	12/07/2009	Poland	A/Warsaw/INS314/2009(H1N1)
HA	KC780401	H1N1	12/07/2009	USA	A/North Carolina/56/2009
HA	CY058396	H1N1	12/07/2009	USA	A/Wisconsin/629-D01591/2009(H1N1)
HA	CY061211	H1N1	12/07/2009	USA	A/New York/6771/2009(H1N1)
HA	CY062819	H1N1	12/07/2009	Denmark	A/Copenhagen/INS144/2009(H1N1)
HA	KC781093	H1N1	12/07/2009	USA	A/Iowa/14/2009
HA	KC780592	H1N1	12/07/2009	USA	A/Texas/75/2009
HA	KC780336	H1N1	12/07/2009	USA	A/Wisconsin/59/2009
HA	CY060484	H1N1	12/07/2009	South Ko	A/Daegu/1854/2009(H1N1)
HA	KT181132	H1N1	12/07/2009	Singapore	A/Singapore/DMS57/2009
HA	JN171946	H1N1	12/07/2009	Canada	A/Montreal/MTL295494/2009
HA	KC782008	H1N1	12/07/2009	USA	A/New Jersey/13/2009
HA	CY080247	H1N1	12/07/2009	China	A/Nanchang/8014/2009
HA	CY063622	H1N1	12/07/2009	USA	A/New York/INS150/2009(H1N1)
HA	CY065888	H1N1	12/07/2009	Netherlar	A/Netherlands/2815/2009
HA	CY056987	H1N1	12/07/2009	Poland	A/Warsaw/INS100/2009(H1N1)
HA	CY061594	H1N1	12/07/2009	USA	A/New York/6806/2009(H1N1)
HA	CY061219	H1N1	12/07/2009	USA	A/New York/6772/2009(H1N1)
HA	CY058006	H1N1	12/07/2009	USA	A/Wisconsin/629-D01473/2009(H1N1)
HA	CY056963	H1N1	12/07/2009	Denmark	A/Copenhagen/INS95/2009(H1N1)
HA	CY061946	H1N1	12/07/2009	USA	A/New York/6943/2009(H1N1)
HA	CY070649	H1N1	12/07/2009	United Ki	A/England/95100002/2009
HA	AB558398	H1N1	12/07/2009	Japan	A/Sendai/TU619/2009
HA	CY073725	H1N1	12/07/2009	Denmark	A/Aalborg/INS283/2009(H1N1)
HA	CY072302	H1N1	12/07/2009	Poland	A/Warsaw/INS315/2009(H1N1)
HA	CY072310	H1N1	12/08/2009	Poland	A/Warsaw/INS316/2009(H1N1)

HA	AB558393	H1N1	12/08/2009	Japan	A/Sendai/TU613/2009
HA	LN846550	H1N1	12/08/2009	United Ki	A/England/9/2009
HA	CY067167	H1N1	12/08/2009	Germany	A/Bonn/INS280/2009(H1N1)
HA	CY066927	H1N1	12/08/2009	USA	A/Pensacola/INS235/2009
HA	CY070651	H1N1	12/08/2009	United Ki	A/England/95100065/2009
HA	KP317218	H1N1	12/08/2009	India	A/Uttrakhand/014/2009
HA	CY058022	H1N1	12/08/2009	USA	A/Wisconsin/629-D00401/2009(H1N1)
HA	CY058014	H1N1	12/08/2009	USA	A/Wisconsin/629-D00672/2009(H1N1)
HA	KC780838	H1N1	12/08/2009	USA	A/Oregon/42/2009
HA	KC780212	H1N1	12/08/2009	USA	A/Indiana/20/2009
HA	CY080251	H1N1	12/08/2009	China	A/Nanchang/8021/2009
HA	JN171908	H1N1	12/08/2009	Canada	A/Quebec/QC002941/2009
HA	LN845782	H1N1	12/08/2009	United Ki	A/England/8/2009
HA	CY070647	H1N1	12/08/2009	United Ki	A/England/940/2009
HA	CY062432	H1N1	12/08/2009	Djibouti	A/Djibouti/N13142/2009(H1N1)
HA	CY061011	H1N1	12/08/2009	USA	A/Texas/JMS387/2009(H1N1)
HA	CY065099	H1N1	12/08/2009	USA	A/New York/7426/2009(H1N1)
HA	CY058380	H1N1	12/08/2009	USA	A/Wisconsin/629-D00780/2009(H1N1)
HA	CY061914	H1N1	12/08/2009	USA	A/New York/6907/2009(H1N1)
HA	CY066919	H1N1	12/08/2009	USA	A/Pensacola/INS234/2009
HA	AB558392	H1N1	12/08/2009	Japan	A/Sendai/TU612/2009
HA	CY063590	H1N1	12/09/2009	Greece	A/Athens/INS122/2009(H1N1)
HA	CY058364	H1N1	12/09/2009	USA	A/Wisconsin/629-D00689/2009(H1N1)
HA	CY069234	H1N1	12/09/2009	Germany	A/Munich/INS365/2009(H1N1)
HA	CY128379	H1N1	12/09/2009	Viet Nam	A/Viet Nam/11032006/2009
HA	CY058388	H1N1	12/09/2009	USA	A/Wisconsin/629-D00147/2009(H1N1)
HA	CY060487	H1N1	12/09/2009	South Ko	A/Daegu/1855/2009(H1N1)
HA	CY070648	H1N1	12/09/2009	United Ki	A/England/95060207/2009
HA	CY072542	H1N1	12/09/2009	Poland	A/Wroclaw/INS436/2009(H1N1)
HA	HM189379	H1N1	12/09/2009	Russia	A/Astrakhan/CRIE-PIV/2009
HA	KC780199	H1N1	12/09/2009	USA	A/Colorado/17/2009
HA	CY089608	H1N1	12/09/2009	China	A/Nanchang/8002/2009
HA	AB558399	H1N1	12/09/2009	Japan	A/Sendai/TU620/2009
HA	AB558395	H1N1	12/09/2009	Japan	A/Sendai/TU616/2009
HA	CY072550	H1N1	12/09/2009	Poland	A/Wroclaw/INS437/2009(H1N1)
HA	CY069210	H1N1	12/09/2009	Germany	A/Berlin/INS361/2009(H1N1)
HA	CY067175	H1N1	12/09/2009	Germany	A/Bonn/INS284/2009(H1N1)
HA	CY070652	H1N1	12/09/2009	United Ki	A/England/1049/2009
HA	AB558394	H1N1	12/09/2009	Japan	A/Sendai/TU615/2009
HA	AB558397	H1N1	12/09/2009	Japan	A/Sendai/TU618/2009
HA	JN863077	H1N1	12/09/2009	China	A/Taizhou/09/2009
HA	HQ228133	H1N1	12/09/2009	Finland	A/Finland/694/2009
HA	CY062779	H1N1	12/10/2009	Belgium	A/Tessenderlo/INS138/2009(H1N1)
HA	CY063775	H1N1	12/10/2009	Singapore	A/Singapore/GP4697/2009(H1N1)
HA	CY083768	H1N1	12/10/2009	Greece	A/Athens/INS256/2009(H1N1)
HA	CY063043	H1N1	12/10/2009	Germany	A/Bochum/INS187/2009(H1N1)
HA	CY065995	H1N1	12/10/2009	Mongolia	A/Ulgii/9911/2009(H1N1)
HA	CY073230	H1N1	12/10/2009	Serbia	A/Belgrade/WRAIR2384T/2009(H1N1)
HA	CY056971	H1N1	12/10/2009	Denmark	A/Copenhagen/INS96/2009(H1N1)
HA	CY058030	H1N1	12/10/2009	USA	A/Wisconsin/629-D00636/2009(H1N1)
HA	CY066671	H1N1	12/10/2009	Belgium	A/Tessenderlo/INS287/2009(H1N1)
HA	KT181133	H1N1	12/10/2009	Singapore	A/Singapore/DMS58/2009
HA	KC780405	H1N1	12/10/2009	USA	A/Indiana/21/2009
HA	KC780222	H1N1	12/10/2009	USA	A/Florida/31/2009

HA	CY061906	H1N1	12/10/2009	USA	A/New York/6903/2009(H1N1)
HA	KP019836	H1N1	12/10/2009	China	A/FuZhou/SWL12273/2009
HA	KM213263	H1N1	12/10/2009	Oman	A/Oman/SQUH-20/2009
HA	CY073222	H1N1	12/10/2009	Serbia	A/Belgrade/WRAIR2384N/2009(H1N1)
HA	AB558400	H1N1	12/10/2009	Japan	A/Sendai/TU622/2009
HA	CY075903	H1N1	12/10/2009	India	A/Pune/NIV19934/2009
HA	CY070656	H1N1	12/10/2009	United Ki	A/England/1054/2009
HA	CY120923	H1N1	12/11/2009	China	A/Guangdong/1202/2009
HA	CY061930	H1N1	12/11/2009	USA	A/New York/6939/2009(H1N1)
HA	CY083934	H1N1	12/11/2009	Poland	A/Warsaw/INS149/2009(H1N1)
HA	CY071095	H1N1	12/11/2009	Spain	A/Barcelona/INS379/2009(H1N1)
HA	CY061978	H1N1	12/11/2009	USA	A/New York/6977/2009(H1N1)
HA	CY063819	H1N1	12/11/2009	Singapore	A/Singapore/ON2400/2009
HA	CY083926	H1N1	12/11/2009	Poland	A/Warsaw/INS148/2009(H1N1)
HA	CY083745	H1N1	12/11/2009	Spain	A/Barcelona/INS189/2009(H1N1)
HA	CY070653	H1N1	12/11/2009	United Ki	A/Scotland/100/2009
HA	CY075904	H1N1	12/11/2009	India	A/Pune/NIV20069/2009
HA	CY099330	H1N1	12/11/2009	USA	A/Kentucky/136/2009
HA	CY070650	H1N1	12/11/2009	United Ki	A/England/95100060/2009
HA	CY063630	H1N1	12/11/2009	USA	A/New York/INS151/2009(H1N1)
HA	CY064772	H1N1	12/11/2009	Germany	A/Berlin/INS170/2009(H1N1)
HA	CY061019	H1N1	12/11/2009	USA	A/Texas/JMS388/2009(H1N1)
HA	CY061922	H1N1	12/11/2009	USA	A/New York/6937/2009(H1N1)
HA	CY061954	H1N1	12/11/2009	USA	A/New York/6945/2009(H1N1)
HA	KC780629	H1N1	12/11/2009	USA	A/Vermont/29/2009
HA	CY058372	H1N1	12/12/2009	USA	A/Wisconsin/629-D01244/2009(H1N1)
HA	CY070666	H1N1	12/12/2009	United Ki	A/England/935/2009
HA	KC780699	H1N1	12/12/2009	USA	A/Vermont/28/2009
HA	CY123303	H1N1	12/12/2009	Singapore	A/Singapore/GP4728/2009
HA	KM213264	H1N1	12/12/2009	Oman	A/Oman/SQUH-22/2009
HA	CY080249	H1N1	12/12/2009	China	A/Nanchang/8015/2009
HA	CY083797	H1N1	12/12/2009	Germany	A/Berlin/INS362/2009(H1N1)
HA	CY080253	H1N1	12/13/2009	China	A/Shantou/8027/2009
HA	CY056955	H1N1	12/13/2009	Greece	A/Athens/INS85/2009(H1N1)
HA	CY072342	H1N1	12/13/2009	Greece	A/Athens/INS320/2009(H1N1)
HA	CY067111	H1N1	12/13/2009	Greece	A/Athens/INS264/2009(H1N1)
HA	CY067103	H1N1	12/13/2009	Greece	A/Athens/INS263/2009(H1N1)
HA	CY083902	H1N1	12/13/2009	Greece	A/Athens/INS86/2009(H1N1)
HA	CY070664	H1N1	12/13/2009	United Ki	A/Scotland/103/2009
HA	CY062715	H1N1	12/13/2009	Greece	A/Athens/INS124/2009(H1N1)
HA	CY067087	H1N1	12/13/2009	Greece	A/Athens/INS261/2009(H1N1)
HA	CY067063	H1N1	12/13/2009	Greece	A/Athens/INS257/2009(H1N1)
HA	CY067079	H1N1	12/13/2009	Greece	A/Athens/INS260/2009(H1N1)
HA	KC780391	H1N1	12/13/2009	USA	A/Maryland/36/2009
HA	CY062723	H1N1	12/13/2009	Greece	A/Athens/INS125/2009(H1N1)
HA	CY062707	H1N1	12/13/2009	Greece	A/Athens/INS123/2009(H1N1)
HA	CY063821	H1N1	12/13/2009	Singapore	A/Singapore/ON2407/2009
HA	KP317221	H1N1	12/13/2009	India	A/Delhi/017/2009
HA	KM213265	H1N1	12/13/2009	Oman	A/Oman/SQUH-31/2009
HA	CY072350	H1N1	12/13/2009	Greece	A/Athens/INS322/2009(H1N1)
HA	CY067119	H1N1	12/13/2009	Greece	A/Athens/INS266/2009(H1N1)
HA	CY067095	H1N1	12/13/2009	Greece	A/Athens/INS262/2009(H1N1)
HA	CY067071	H1N1	12/13/2009	Greece	A/Athens/INS259/2009(H1N1)
HA	CY061962	H1N1	12/14/2009	USA	A/New York/6949/2009(H1N1)

HA	CY061994	H1N1	12/14/2009	USA	A/New York/7020/2009(H1N1)
HA	GU592905	H1N1	12/14/2009	Russia	A/Magadan/02/2009(H1N1)
HA	CY054548	H1N1	12/14/2009	Mongolia	A/Bayanulgii/9912/2009(H1N1)
HA	CY058452	H1N1	12/14/2009	USA	A/Wisconsin/629-D01846/2009(H1N1)
HA	CY058420	H1N1	12/14/2009	USA	A/Wisconsin/629-D01904/2009(H1N1)
HA	KC683495	H1N1	12/14/2009	China	A/Liaoning/14/2009
HA	CY073214	H1N1	12/14/2009	Serbia	A/Belgrade/WRAIR2375T/2009(H1N1)
HA	KC780939	H1N1	12/14/2009	USA	A/Washington/73/2009
HA	KT181134	H1N1	12/14/2009	Singapore	A/Singapore/DMS59/2009
HA	CY070654	H1N1	12/14/2009	United Ki	A/England/1051/2009
HA	CY061970	H1N1	12/14/2009	USA	A/New York/6976/2009(H1N1)
HA	KP317220	H1N1	12/14/2009	India	A/Delhi/016/2009
HA	CY073206	H1N1	12/14/2009	Serbia	A/Belgrade/WRAIR2375N/2009(H1N1)
HA	CY062659	H1N1	12/15/2009	Greece	A/Athens/INS87/2009(H1N1)
HA	AB704454	H1N1	12/15/2009	Japan	A/Gunma/293/2009
HA	CY063019	H1N1	12/15/2009	Spain	A/Madrid/INS184/2009(H1N1)
HA	CY070655	H1N1	12/15/2009	United Ki	A/England/933/2009
HA	CY073877	H1N1	12/15/2009	Greece	A/Athens/INS323/2009(H1N1)
HA	CY066607	H1N1	12/15/2009	Greece	A/Athens/INS273/2009(H1N1)
HA	CY066615	H1N1	12/15/2009	Greece	A/Athens/INS274/2009(H1N1)
HA	CY063051	H1N1	12/15/2009	Belgium	A/Tessenderlo/INS191/2009(H1N1)
HA	CY070662	H1N1	12/15/2009	United Ki	A/Scotland/101/2009
HA	CY070657	H1N1	12/15/2009	United Ki	A/England/1052/2009
HA	CY066335	H1N1	12/15/2009	USA	A/California/VRDL120/2009(H1N1)
HA	CY066351	H1N1	12/15/2009	USA	A/California/VRDL122/2009(H1N1)
HA	KM213256	H1N1	12/15/2009	Oman	A/Oman/SQUH-1/2009
HA	CY067135	H1N1	12/15/2009	Greece	A/Athens/INS268/2009(H1N1)
HA	CY067151	H1N1	12/15/2009	Greece	A/Athens/INS270/2009(H1N1)
HA	KR861660	H1N1	12/15/2009	India	A/Bangalore/11210-01/2009
HA	CY123305	H1N1	12/15/2009	Singapore	A/Singapore/GP4757/2009
HA	CY090097	H1N1	12/15/2009	USA	A/Boston/706/2009
HA	CY062731	H1N1	12/15/2009	Greece	A/Athens/INS126/2009(H1N1)
HA	CY066623	H1N1	12/15/2009	Greece	A/Athens/INS275/2009(H1N1)
HA	CY066599	H1N1	12/15/2009	Greece	A/Athens/INS272/2009(H1N1)
HA	CY060493	H1N1	12/15/2009	South Kor	A/Daejeon/1871/2009(H1N1)
HA	CY058428	H1N1	12/15/2009	USA	A/Wisconsin/629-D01752/2009(H1N1)
HA	CY067143	H1N1	12/15/2009	Greece	A/Athens/INS269/2009(H1N1)
HA	CY066327	H1N1	12/15/2009	USA	A/California/VRDL119/2009(H1N1)
HA	CY066359	H1N1	12/15/2009	USA	A/California/VRDL123/2009(H1N1)
HA	CY066343	H1N1	12/15/2009	USA	A/California/VRDL121/2009(H1N1)
HA	CY063823	H1N1	12/15/2009	Singapore	A/Singapore/ON2416/2009
HA	CY058468	H1N1	12/15/2009	USA	A/Wisconsin/629-D00337/2009(H1N1)
HA	CY070663	H1N1	12/15/2009	United Ki	A/Scotland/102/2009
HA	CY070658	H1N1	12/15/2009	United Ki	A/England/1055/2009
HA	CY073489	H1N1	12/15/2009	Greece	A/Athens/INS271/2009(H1N1)
HA	CY073182	H1N1	12/15/2009	Serbia	A/Belgrade/WRAIR2366N/2009(H1N1)
HA	CY072326	H1N1	12/15/2009	USA	A/Newark/INS318/2009(H1N1)
HA	CY058476	H1N1	12/15/2009	USA	A/Wisconsin/629-D02424/2009(H1N1)
HA	KC780463	H1N1	12/16/2009	USA	A/Mississippi/09/2009
HA	CY061986	H1N1	12/16/2009	USA	A/New York/7019/2009(H1N1)
HA	CY071103	H1N1	12/16/2009	Belgium	A/Tessenderlo/INS380/2009(H1N1)
HA	CY058404	H1N1	12/16/2009	USA	A/Wisconsin/629-D01038/2009(H1N1)
HA	KM885027	H1N1	12/16/2009	India	A/Indore/59/2009
HA	KC780487	H1N1	12/16/2009	USA	A/Tennessee/19/2009

HA	KT181135	H1N1	12/16/2009	Singapore	A/Singapore/DMS60/2009
HA	CY062002	H1N1	12/16/2009	USA	A/New York/7036/2009(H1N1)
HA	CY060496	H1N1	12/16/2009	South Kor	A/Daegu/1873/2009(H1N1)
HA	CY062939	H1N1	12/16/2009	Spain	A/Terrassa/INS172/2009(H1N1)
HA	KP317219	H1N1	12/16/2009	India	A/Haryana/015/2009
HA	CY058444	H1N1	12/16/2009	USA	A/Wisconsin/629-D02073/2009(H1N1)
HA	KC780644	H1N1	12/16/2009	USA	A/Connecticut/18/2009
HA	CY058460	H1N1	12/16/2009	USA	A/Wisconsin/629-D00996/2009(H1N1)
HA	CY067127	H1N1	12/16/2009	Greece	A/Athens/INS267/2009(H1N1)
HA	CY080257	H1N1	12/16/2009	China	A/Nanchang/8029/2009
HA	KC782235	H1N1	12/16/2009	USA	A/Michigan/29/2009
HA	HQ228127	H1N1	12/16/2009	Finland	A/Finland/688/2009
HA	CY070661	H1N1	12/16/2009	United Ki	A/England/95200063/2009
HA	CY061027	H1N1	12/16/2009	USA	A/Texas/JMS389/2009(H1N1)
HA	CY071255	H1N1	12/16/2009	Spain	A/Madrid/INS408/2009(H1N1)
HA	CY123307	H1N1	12/16/2009	Singapore	A/Singapore/GP4778/2009
HA	CY058436	H1N1	12/17/2009	USA	A/Wisconsin/629-D01556/2009(H1N1)
HA	CY072318	H1N1	12/17/2009	USA	A/New York/INS317/2009(H1N1)
HA	CY062851	H1N1	12/17/2009	Greece	A/Athens/INS158/2009(H1N1)
HA	CY072462	H1N1	12/17/2009	Greece	A/Athens/INS341/2009(H1N1)
HA	KC780429	H1N1	12/17/2009	USA	A/Virginia/27/2009
HA	CY070660	H1N1	12/17/2009	United Ki	A/England/95200044/2009
HA	CY128054	H1N1	12/17/2009	Viet Nam	A/Viet Nam/11032011/2009
HA	KC780446	H1N1	12/17/2009	USA	A/Virginia/27/2009
HA	CY128387	H1N1	12/17/2009	Viet Nam	A/Viet Nam/11032010/2009
HA	KC782331	H1N1	12/17/2009	USA	A/Kansas/25/2009
HA	AB558401	H1N1	12/17/2009	Japan	A/Sendai/TU628/2009
HA	CY083805	H1N1	12/17/2009	Spain	A/Madrid/INS377/2009(H1N1)
HA	CY062947	H1N1	12/17/2009	Spain	A/Terrassa/INS173/2009(H1N1)
HA	CY067159	H1N1	12/17/2009	Greece	A/Athens/INS276/2009(H1N1)
HA	CY072454	H1N1	12/17/2009	Greece	A/Athens/INS340/2009(H1N1)
HA	CY072470	H1N1	12/17/2009	Greece	A/Athens/INS342/2009(H1N1)
HA	CY070665	H1N1	12/18/2009	United Ki	A/England/1056/2009
HA	AB704534	H1N1	12/18/2009	Japan	A/Yamaguchi/273/2009
HA	KC781232	H1N1	12/18/2009	USA	A/Maine/14/2009
HA	CY058412	H1N1	12/18/2009	USA	A/Wisconsin/629-D00459/2009(H1N1)
HA	CY062931	H1N1	12/18/2009	Germany	A/Berlin/INS171/2009(H1N1)
HA	CY062867	H1N1	12/18/2009	Greece	A/Athens/INS160/2009(H1N1)
HA	CY072486	H1N1	12/18/2009	Greece	A/Athens/INS345/2009(H1N1)
HA	GU592889	H1N1	12/18/2009	Russia	A/Barnaul/04/2009(H1N1)
HA	CY072478	H1N1	12/18/2009	Greece	A/Athens/INS344/2009(H1N1)
HA	CY063654	H1N1	12/18/2009	Greece	A/Athens/INS157/2009(H1N1)
HA	CY060490	H1N1	12/18/2009	South Kor	A/Seoul/1870/2009(H1N1)
HA	CY062859	H1N1	12/18/2009	Greece	A/Athens/INS159/2009(H1N1)
HA	JN187136	H1N1	12/18/2009	Taiwan	A/Taiwan/11706/2009
HA	CY080259	H1N1	12/18/2009	China	A/Nanchang/8009/2009
HA	HM072426	H1N1	12/19/2009	Taiwan	A/Taiwan/2343/2009
HA	CY070659	H1N1	12/19/2009	United Ki	A/England/1053/2009
HA	HM230701	H1N1	12/19/2009	China	A/Zhoushan/52/2009(H1N1)
HA	CY089616	H1N1	12/19/2009	China	A/Nanchang/8008/2009
HA	CY062875	H1N1	12/19/2009	Greece	A/Athens/INS161/2009(H1N1)
HA	KR861661	H1N1	12/19/2009	India	A/Bangalore/11406-11/2009
HA	KC780572	H1N1	12/19/2009	USA	A/Delaware/18/2009
HA	CY062891	H1N1	12/20/2009	Greece	A/Athens/INS163/2009(H1N1)

HA	CY072510	H1N1	12/20/2009	Greece	A/Athens/INS348/2009(H1N1)
HA	CY065091	H1N1	12/20/2009	USA	A/New York/7425/2009(H1N1)
HA	CY072358	H1N1	12/20/2009	Greece	A/Athens/INS325/2009(H1N1)
HA	CY072518	H1N1	12/20/2009	Greece	A/Athens/INS349/2009(H1N1)
HA	CY072494	H1N1	12/20/2009	Greece	A/Athens/INS346/2009(H1N1)
HA	CY069154	H1N1	12/20/2009	Greece	A/Athens/INS352/2009(H1N1)
HA	CY072502	H1N1	12/20/2009	Greece	A/Athens/INS347/2009(H1N1)
HA	CY062883	H1N1	12/20/2009	Greece	A/Athens/INS162/2009(H1N1)
HA	CY061035	H1N1	12/20/2009	USA	A/Texas/JMS390/2009(H1N1)
HA	CY062899	H1N1	12/20/2009	Greece	A/Athens/INS164/2009(H1N1)
HA	CY073505	H1N1	12/20/2009	Greece	A/Athens/INS350/2009(H1N1)
HA	CY072366	H1N1	12/20/2009	Greece	A/Athens/INS327/2009(H1N1)
HA	JN171947	H1N1	12/21/2009	Canada	A/Montreal/MTL307782/2009
HA	CY088621	H1N1	12/21/2009	Denmark	A/Odense/INS176/2009
HA	CY070668	H1N1	12/21/2009	United Ki	A/England/1057/2009
HA	KT181136	H1N1	12/21/2009	Singapor	A/Singapore/DMS61/2009
HA	CY070667	H1N1	12/21/2009	United Ki	A/England/936/2009
HA	CY062907	H1N1	12/21/2009	Greece	A/Athens/INS165/2009(H1N1)
HA	CY062995	H1N1	12/21/2009	Estonia	A/Tallinn/INS181/2009(H1N1)
HA	KC781271	H1N1	12/21/2009	USA	A/New York/109/2009
HA	CY069162	H1N1	12/21/2009	Greece	A/Athens/INS353/2009(H1N1)
HA	CY062018	H1N1	12/21/2009	USA	A/New York/7216/2009(H1N1)
HA	CY069170	H1N1	12/21/2009	Greece	A/Athens/INS354/2009(H1N1)
HA	CY075888	H1N1	12/21/2009	India	A/Pune/NIV0953725/2009(H1N1)
HA	CY066399	H1N1	12/22/2009	USA	A/California/VRDL128/2009(H1N1)
HA	CY069178	H1N1	12/22/2009	Greece	A/Athens/INS355/2009(H1N1)
HA	CY071111	H1N1	12/22/2009	Germany	A/Wurzburg/INS381/2009(H1N1)
HA	CY066391	H1N1	12/22/2009	USA	A/California/VRDL127/2009(H1N1)
HA	CY066375	H1N1	12/22/2009	USA	A/California/VRDL125/2009(H1N1)
HA	CY069242	H1N1	12/22/2009	Austria	A/Vienna/INS366/2009(H1N1)
HA	CY070669	H1N1	12/22/2009	United Ki	A/England/95260539/2009
HA	CY089624	H1N1	12/22/2009	China	A/Nanchang/8011/2009
HA	CY066367	H1N1	12/22/2009	USA	A/California/VRDL124/2009(H1N1)
HA	KC780293	H1N1	12/22/2009	USA	A/Vermont/30/2009
HA	CY066383	H1N1	12/22/2009	USA	A/California/VRDL126/2009(H1N1)
HA	KC780330	H1N1	12/23/2009	USA	A/South Carolina/46/2009
HA	CY071063	H1N1	12/23/2009	Estonia	A/Tallinn/INS372/2009(H1N1)
HA	CY071199	H1N1	12/23/2009	Germany	A/Frankfurt/INS401/2009(H1N1)
HA	CY073254	H1N1	12/23/2009	Greece	A/Athens/WRAIR2962N/2009(H1N1)
HA	LN867652	H1N1	12/23/2009	United Ki	A/England/01220639/2009
HA	CY107439	H1N1	12/23/2009	United Ki	A/Scotland/Perth_09V502475/2009
HA	CY128395	H1N1	12/23/2009	Viet Nam	A/Viet Nam/11032016/2009
HA	CY062010	H1N1	12/23/2009	USA	A/New York/7107/2009(H1N1)
HA	CY061043	H1N1	12/23/2009	USA	A/Texas/JMS391/2009(H1N1)
HA	CY065083	H1N1	12/23/2009	USA	A/New York/7424/2009(H1N1)
HA	HM581925	H1N1	12/23/2009	Iran	A/Iran/1519/2009(H1N1)
HA	CY075905	H1N1	12/23/2009	India	A/Pune/NIV21090/2009
HA	CY070643	H1N1	12/23/2009	United Ki	A/Scotland/98/2009
HA	CY062963	H1N1	12/23/2009	Spain	A/Terrassa/INS175/2009(H1N1)
HA	CY075906	H1N1	12/23/2009	India	A/Pune/NIV21139/2009
HA	CY128403	H1N1	12/23/2009	Viet Nam	A/Viet Nam/11032017/2009
HA	CY075911	H1N1	12/24/2009	India	A/Pune/NIV21259/2009
HA	CY075910	H1N1	12/24/2009	India	A/Pune/NIV21218/2009
HA	CY062955	H1N1	12/24/2009	Spain	A/Terrassa/INS174/2009(H1N1)

HA	CY075907	H1N1	12/24/2009	India	A/Pune/NIV21159/2009
HA	CY075908	H1N1	12/24/2009	India	A/Pune/NIV21163/2009
HA	HQ228140	H1N1	12/24/2009	Finland	A/Finland/701/2009
HA	CY072374	H1N1	12/25/2009	Greece	A/Athens/INS329/2009(H1N1)
HA	CY071119	H1N1	12/25/2009	Germany	A/Wurzburg/INS382/2009(H1N1)
HA	KC780530	H1N1	12/25/2009	USA	A/Maryland/37/2009
HA	CY061051	H1N1	12/25/2009	USA	A/Texas/JMS392/2009(H1N1)
HA	KT181139	H1N1	12/25/2009	Singapore	A/Singapore/DMS62/2009
HA	CY075909	H1N1	12/25/2009	India	A/Pune/NIV21193/2009
HA	CY069194	H1N1	12/26/2009	Greece	A/Athens/INS358/2009(H1N1)
HA	CY069186	H1N1	12/26/2009	Greece	A/Athens/INS357/2009(H1N1)
HA	CY072382	H1N1	12/26/2009	Greece	A/Athens/INS331/2009(H1N1)
HA	KR861662	H1N1	12/26/2009	India	A/Bangalore/11658-15/2009
HA	CY072390	H1N1	12/26/2009	Greece	A/Athens/INS332/2009(H1N1)
HA	CY069202	H1N1	12/26/2009	Greece	A/Athens/INS359/2009(H1N1)
HA	CY073006	H1N1	12/26/2009	Greece	A/Athens/INS330/2009(H1N1)
HA	KF886294	H1N1	12/26/2009	India	A/Jabalpur/112/2009
HA	CY062034	H1N1	12/26/2009	USA	A/New York/0259/2009(H1N1)
HA	CY062915	H1N1	12/26/2009	Greece	A/Athens/INS166/2009(H1N1)
HA	CY062923	H1N1	12/26/2009	Greece	A/Athens/INS167/2009(H1N1)
HA	CY061059	H1N1	12/27/2009	USA	A/Texas/JMS393/2009(H1N1)
HA	CY062042	H1N1	12/27/2009	USA	A/New York/0352/2009(H1N1)
HA	KC780232	H1N1	12/27/2009	USA	A/Montana/25/2009
HA	CY062026	H1N1	12/28/2009	USA	A/New York/7236/2009(H1N1)
HA	KC780322	H1N1	12/28/2009	USA	A/Georgia/25/2009
HA	CY072334	H1N1	12/28/2009	USA	A/Newark/INS319/2009(H1N1)
HA	CY072398	H1N1	12/28/2009	Greece	A/Athens/INS333/2009(H1N1)
HA	KT181141	H1N1	12/28/2009	Singapore	A/Singapore/DMS63/2009
HA	CY062971	H1N1	12/28/2009	Denmark	A/Odense/INS177/2009(H1N1)
HA	KC780484	H1N1	12/28/2009	USA	A/Michigan/28/2009
HA	CY064780	H1N1	12/29/2009	Spain	A/Barcelona/INS190/2009(H1N1)
HA	JN187137	H1N1	12/29/2009	Taiwan	A/Taiwan/1017/2009
HA	CY070670	H1N1	12/29/2009	United Ki	A/England/95340029/2009
HA	CY071239	H1N1	12/29/2009	Poland	A/Warsaw/INS406/2009(H1N1)
HA	CY072406	H1N1	12/29/2009	Greece	A/Athens/INS334/2009(H1N1)
HA	CY071231	H1N1	12/29/2009	Poland	A/Warsaw/INS405/2009(H1N1)
HA	CY063638	H1N1	12/29/2009	Greece	A/Athens/INS153/2009(H1N1)
HA	CY063646	H1N1	12/29/2009	Greece	A/Athens/INS154/2009(H1N1)
HA	CY070733	H1N1	12/29/2009	United Ki	A/Scotland/104/2009
HA	CY061091	H1N1	12/30/2009	USA	A/Texas/JMS398/2009(H1N1)
HA	CY072414	H1N1	12/30/2009	Greece	A/Athens/INS335/2009(H1N1)
HA	JQ065213	H1N1	12/30/2009	China	A/Shenzhen/1648/2009
HA	JQ065203	H1N1	12/30/2009	China	A/Shenzhen/1528/2009
HA	CY066439	H1N1	12/30/2009	USA	A/California/VRDL133/2009(H1N1)
HA	CY128411	H1N1	12/30/2009	Viet Nam	A/Viet Nam/11032021/2009
HA	KC780632	H1N1	12/30/2009	USA	A/Rhode Island/25/2009
HA	CY066423	H1N1	12/30/2009	USA	A/California/VRDL131/2009(H1N1)
HA	CY062058	H1N1	12/30/2009	USA	A/New York/0461/2009(H1N1)
HA	JQ065214	H1N1	12/30/2009	China	A/Shenzhen/1658/2009
HA	JQ065207	H1N1	12/30/2009	China	A/Shenzhen/1604/2009
HA	JQ065217	H1N1	12/30/2009	China	A/Shenzhen/1664/2009
HA	CY061075	H1N1	12/30/2009	USA	A/Texas/JMS395/2009(H1N1)
HA	JQ065200	H1N1	12/30/2009	China	A/Shenzhen/1447/2009
HA	JQ065206	H1N1	12/30/2009	China	A/Shenzhen/1594/2009

HA	JQ065215	H1N1	12/30/2009	China	A/Shenzhen/1663/2009
HA	JQ065209	H1N1	12/30/2009	China	A/Shenzhen/1617/2009
HA	JQ065204	H1N1	12/30/2009	China	A/Shenzhen/1548/2009
HA	JQ065211	H1N1	12/30/2009	China	A/Shenzhen/1640/2009
HA	CY062843	H1N1	12/30/2009	Greece	A/Athens/INS156/2009(H1N1)
HA	KC780671	H1N1	12/30/2009	USA	A/Nebraska/23/2009
HA	CY061067	H1N1	12/30/2009	USA	A/Texas/JMS394/2009(H1N1)
HA	HM568128	H1N1	12/30/2009	United Ki	A/England/938/2009
HA	CY062835	H1N1	12/30/2009	Greece	A/Athens/INS155/2009(H1N1)
HA	CY063027	H1N1	12/30/2009	Spain	A/Madrid/INS185/2009(H1N1)
HA	CY066415	H1N1	12/30/2009	USA	A/California/VRDL130/2009(H1N1)
HA	CY066407	H1N1	12/30/2009	USA	A/California/VRDL129/2009(H1N1)
HA	LN846367	H1N1	12/30/2009	United Ki	A/England/WTSI1778/2009
HA	JQ714166	H1N1	12/30/2009	China	A/Tianjinjinnan/SWL4141/2009
HA	CY061083	H1N1	12/30/2009	USA	A/Texas/JMS397/2009(H1N1)
HA	CY066431	H1N1	12/30/2009	USA	A/California/VRDL132/2009(H1N1)
HA	JQ065201	H1N1	12/30/2009	China	A/Shenzhen/1500/2009
HA	JQ065202	H1N1	12/30/2009	China	A/Shenzhen/1511/2009
HA	CY072422	H1N1	12/30/2009	Greece	A/Athens/INS336/2009(H1N1)
HA	CY072438	H1N1	12/30/2009	Greece	A/Athens/INS338/2009(H1N1)
HA	CY062050	H1N1	12/30/2009	USA	A/New York/0357/2009(H1N1)
HA	JQ065208	H1N1	12/30/2009	China	A/Shenzhen/1605/2009
HA	CY063059	H1N1	12/30/2009	Belgium	A/Tessenderlo/INS192/2009(H1N1)
HA	JQ065205	H1N1	12/30/2009	China	A/Shenzhen/1565/2009
HA	JQ065212	H1N1	12/30/2009	China	A/Shenzhen/1646/2009
HA	CY063035	H1N1	12/30/2009	Spain	A/Madrid/INS186/2009(H1N1)
HA	JQ065210	H1N1	12/30/2009	China	A/Shenzhen/1627/2009
HA	JQ065199	H1N1	12/30/2009	China	A/Shenzhen/1441/2009
HA	CY072446	H1N1	12/30/2009	Greece	A/Athens/INS339/2009(H1N1)
HA	CY072430	H1N1	12/30/2009	Greece	A/Athens/INS337/2009(H1N1)
HA	CY123312	H1N1	12/30/2009	Singapore	A/Singapore/GP4895/2009
HA	CY123322	H1N1	12/31/2009	Singapore	A/Singapore/GP4915/2009
HA	CY070735	H1N1	12/31/2009	United Ki	A/England/937/2009
HA	KT181143	H1N1	12/31/2009	Singapore	A/Singapore/DMS64/2009
HA	CY071127	H1N1	12/31/2009	Greece	A/Athens/INS383/2009(H1N1)
HA	CY061099	H1N1	12/31/2009	USA	A/Texas/JMS399/2009(H1N1)
HA	CY061570	H1N1	12/31/2009	USA	A/Texas/JMS400/2009(H1N1)
HA	KR861663	H1N1	12/31/2009	India	A/Bangalore/12220-18/2009
HA	KP019837	H1N1	12/31/2009	China	A/FuZhou/SWL12496/2009
HA	CY123317	H1N1	12/31/2009	Singapore	A/Singapore/GP4907/2009
HA	KC780867	H1N1	12/31/2009	USA	A/Idaho/17/2009
HA	JN171948	H1N1	12/31/2009	Canada	A/Montreal/MTL007311/2009
HA	CY070734	H1N1	12/31/2009	United Ki	A/Scotland/87/2009
HA	GU576540	H1N1	12/31/2009	Italy	A/Ancona/508/2009(H1N1)
HA	KC780664	H1N1	12/31/2009	USA	A/Minnesota/38/2009
HA	AB745253	H1N1	01/2010	Japan	A/Tottori/ST484/2010
HA	AB745256	H1N1	01/2010	Japan	A/Tottori/ST487/2010
HA	CY067634	H1N1	01/2010	China	A/Qingdao/94/2010
HA	CY067611	H1N1	01/2010	China	A/Qingdao/101/2010
HA	CY071947	H1N1	01/2010	Egypt	A/Egypt/N00124/2010(H1N1)
HA	CY071961	H1N1	01/2010	Egypt	A/Egypt/N00283/2010(H1N1)
HA	CY071959	H1N1	01/2010	Egypt	A/Egypt/N00278/2010(H1N1)
HA	CY071957	H1N1	01/2010	Egypt	A/Egypt/N00275/2010(H1N1)
HA	JN381262	H1N1	01/2010	Taiwan	A/Taiwan/65078/2010

HA	JN381263	H1N1	01/2010	Taiwan	A/Taiwan/90019/2010
HA	JQ365003	H1N1	01/2010	China	A/Hangzhou/15/2010
HA	AB745255	H1N1	01/2010	Japan	A/Tottori/ST486/2010
HA	CY071955	H1N1	01/2010	Egypt	A/Egypt/N00267/2010(H1N1)
HA	AB745247	H1N1	01/2010	Japan	A/Tottori/ST469/2010
HA	AB745251	H1N1	01/2010	Japan	A/Tottori/ST479/2010
HA	AB745257	H1N1	01/2010	Japan	A/Tottori/ST489/2010
HA	JN381264	H1N1	01/2010	Taiwan	A/Taiwan/65211/2010
HA	JQ365006	H1N1	01/2010	China	A/Hangzhou/115/2010
HA	CY071949	H1N1	01/2010	Egypt	A/Egypt/N00128/2010(H1N1)
HA	AB745248	H1N1	01/2010	Japan	A/Tottori/ST472/2010
HA	AB745254	H1N1	01/2010	Japan	A/Tottori/ST485/2010
HA	AB745258	H1N1	01/2010	Japan	A/Tottori/ST490/2010
HA	AB745259	H1N1	01/2010	Japan	A/Tottori/ST494/2010
HA	JQ365004	H1N1	01/2010	China	A/Hangzhou/110/2010
HA	JQ365005	H1N1	01/2010	China	A/Hangzhou/111/2010
HA	CY120024	H1N1	01/2010	Mexico	A/Mexico/InDRECTRLA/2010
HA	JN381265	H1N1	01/2010	Taiwan	A/Taiwan/65229/2010
HA	JN381260	H1N1	01/2010	Taiwan	A/Taiwan/65048/2010
HA	JN381266	H1N1	01/2010	Taiwan	A/Taiwan/90027/2010
HA	JN381261	H1N1	01/2010	Taiwan	A/Taiwan/65052/2010
HA	JN381259	H1N1	01/2010	Taiwan	A/Taiwan/90001/2010
HA	AB745246	H1N1	01/2010	Japan	A/Tottori/ST462/2010
HA	AB745249	H1N1	01/2010	Japan	A/Tottori/ST474/2010
HA	AB745250	H1N1	01/2010	Japan	A/Tottori/ST477/2010
HA	AB745252	H1N1	01/2010	Japan	A/Tottori/ST482/2010
HA	AB745260	H1N1	01/2010	Japan	A/Tottori/ST499/2010
HA	CY071956	H1N1	01/2010	Egypt	A/Egypt/N00273/2010(H1N1)
HA	CY071958	H1N1	01/2010	Egypt	A/Egypt/N00277/2010(H1N1)
HA	CY067633	H1N1	01/2010	China	A/Qingdao/70/2010
HA	CY067631	H1N1	01/2010	China	A/Qingdao/63/2010
HA	CY067632	H1N1	01/2010	China	A/Qingdao/66/2010
HA	CY060454	H1N1	01/2010	South Ko	A/Seoul/7/2010(H1N1)
HA	CY071951	H1N1	01/2010	Egypt	A/Egypt/N00164/2010(H1N1)
HA	CY071960	H1N1	01/2010	Egypt	A/Egypt/N00281/2010(H1N1)
HA	CY071952	H1N1	01/2010	Egypt	A/Egypt/N00231/2010(H1N1)
HA	CY071950	H1N1	01/2010	Egypt	A/Egypt/N00145/2010(H1N1)
HA	CY071954	H1N1	01/2010	Egypt	A/Egypt/N00238/2010(H1N1)
HA	CY071953	H1N1	01/2010	Egypt	A/Egypt/N00236/2010(H1N1)
HA	CY071948	H1N1	01/2010	Egypt	A/Egypt/N00126/2010(H1N1)
HA	KC780547	H1N1	01/01/2010	USA	A/New Jersey/01/2010
HA	KC780307	H1N1	01/01/2010	USA	A/Minnesota/01/2010
HA	KC780408	H1N1	01/01/2010	USA	A/Utah/01/2010
HA	CY065067	H1N1	01/02/2010	USA	A/New York/7420/2010(H1N1)
HA	CY080265	H1N1	01/02/2010	China	A/Shantou/8064/2010
HA	JQ714167	H1N1	01/02/2010	China	A/Tianjinnankai/SWL49/2010
HA	KC780528	H1N1	01/02/2010	USA	A/Kansas/01/2010
HA	KC781359	H1N1	01/02/2010	USA	A/Hawaii/02/2010
HA	KM213266	H1N1	01/02/2010	Oman	A/Oman/SQUH-32/2010
HA	KM213272	H1N1	01/02/2010	Oman	A/Oman/SQUH-40/2010
HA	KC781435	H1N1	01/02/2010	USA	A/Hawaii/02/2010
HA	KM213268	H1N1	01/02/2010	Oman	A/Oman/SQUH-34/2010
HA	KM213267	H1N1	01/03/2010	Oman	A/Oman/SQUH-33/2010
HA	KC781238	H1N1	01/03/2010	USA	A/Hawaii/01/2010

HA	KC781179	H1N1	01/03/2010	USA	A/Hawaii/01/2010
HA	CY061578	H1N1	01/03/2010	USA	A/Texas/JMS401/2010(H1N1)
HA	KC780574	H1N1	01/03/2010	USA	A/New Hampshire/01/2010
HA	KT181144	H1N1	01/04/2010	Singapore	A/Singapore/DMS65/2010
HA	JX308949	H1N1	01/04/2010	Singapore	A/Singapore/GP10/2010
HA	GU562458	H1N1	01/04/2010	Russia	A/Karasuk/01/2010(H1N1)
HA	AB551871	H1N1	01/04/2010	Japan	A/Nagasaki/HA-10-1/2010(H1N1)
HA	AB551872	H1N1	01/04/2010	Japan	A/Nagasaki/HA-10-2/2010(H1N1)
HA	AB551874	H1N1	01/04/2010	Japan	A/Nagasaki/HA-10-4/2010(H1N1)
HA	JN588789	H1N1	01/04/2010	Cambodia	A/Cambodia/U094/2010
HA	CY061107	H1N1	01/04/2010	USA	A/Texas/JMS402/2010(H1N1)
HA	CY065872	H1N1	01/04/2010	Netherlar	A/Netherlands/2631b/2010
HA	CY061586	H1N1	01/04/2010	USA	A/Texas/JMS403/2010(H1N1)
HA	HM165185	H1N1	01/04/2010	China	A/Haishu/SWL110/2010(H1N1)
HA	CY096586	H1N1	01/04/2010	USA	A/District of Columbia/INS525/2010(I
HA	KC781903	H1N1	01/04/2010	USA	A/Alabama/01/2010
HA	KC780620	H1N1	01/04/2010	USA	A/Alabama/01/2010
HA	CY063003	H1N1	01/04/2010	Estonia	A/Tallinn/INS182/2010(H1N1)
HA	AB551873	H1N1	01/04/2010	Japan	A/Nagasaki/HA-10-3/2010(H1N1)
HA	CY062979	H1N1	01/05/2010	Germany	A/Bonn/INS178/2010(H1N1)
HA	KC782014	H1N1	01/05/2010	USA	A/Iowa/01/2010
HA	CY073773	H1N1	01/05/2010	Austria	A/Vienna/INS369/2010(H1N1)
HA	KC781020	H1N1	01/05/2010	USA	A/New Mexico/01/2010
HA	CY071055	H1N1	01/05/2010	Austria	A/Vienna/INS368/2010(H1N1)
HA	JX308957	H1N1	01/05/2010	Singapore	A/Singapore/GP33/2010
HA	JX309122	H1N1	01/05/2010	Singapore	A/Singapore/KK04/2010
HA	JX308986	H1N1	01/05/2010	Singapore	A/Singapore/TT01/2010
HA	CY080261	H1N1	01/05/2010	China	A/Shantou/8049/2010
HA	CY080255	H1N1	01/05/2010	China	A/Shantou/8028/2010
HA	KC780224	H1N1	01/05/2010	USA	A/Colorado/02/2010
HA	CY062987	H1N1	01/05/2010	Austria	A/Vienna/INS179/2010(H1N1)
HA	JX309082	H1N1	01/06/2010	Singapore	A/Singapore/TT10/2010
HA	JX308965	H1N1	01/06/2010	Singapore	A/Singapore/GP64/2010
HA	CY075067	H1N1	01/06/2010	Greece	A/Athens/INS393/2010(H1N1)
HA	CY075059	H1N1	01/06/2010	Greece	A/Athens/INS392/2010(H1N1)
HA	CY080263	H1N1	01/06/2010	China	A/Shantou/8012/2010
HA	JX309078	H1N1	01/06/2010	Singapore	A/Singapore/EN008/2010
HA	JX309126	H1N1	01/06/2010	Singapore	A/Singapore/KK12/2010
HA	KC780411	H1N1	01/06/2010	USA	A/Florida/01/2010
HA	CY063011	H1N1	01/07/2010	Estonia	A/Tallinn/INS183/2010(H1N1)
HA	CY072526	H1N1	01/07/2010	Estonia	A/Tallinn/INS374/2010(H1N1)
HA	JX309090	H1N1	01/07/2010	Singapore	A/Singapore/GP75/2010
HA	JX309094	H1N1	01/07/2010	Singapore	A/Singapore/GP95/2010
HA	KT181146	H1N1	01/07/2010	Singapore	A/Singapore/DMS66/2010
HA	CY071071	H1N1	01/07/2010	Estonia	A/Tallinn/INS373/2010(H1N1)
HA	CY056294	H1N1	01/08/2010	China	A/Henan/1/2010(H1N1)
HA	KP019838	H1N1	01/08/2010	China	A/FuZhou/SWL172/2010
HA	CY063855	H1N1	01/08/2010	Singapore	A/Singapore/TT017/2010(H1N1)
HA	CY071207	H1N1	01/08/2010	Germany	A/Frankfurt/INS402/2010(H1N1)
HA	CY061115	H1N1	01/08/2010	USA	A/Texas/JMS404/2010(H1N1)
HA	KM213269	H1N1	01/09/2010	Oman	A/Oman/SQUH-36/2010
HA	GU576542	H1N1	01/09/2010	Italy	A/Ancona/01/2010(H1N1)
HA	CY066447	H1N1	01/09/2010	USA	A/California/VRDL1/2010(H1N1)
HA	CY061123	H1N1	01/09/2010	USA	A/Texas/JMS405/2010(H1N1)

HA	KC780617	H1N1	01/09/2010	USA	A/North Carolina/02/2010
HA	KP317223	H1N1	01/10/2010	India	A/Rajasthan/019/2010
HA	CY061131	H1N1	01/10/2010	USA	A/Texas/JMS406/2010(H1N1)
HA	CY071159	H1N1	01/11/2010	Greece	A/Athens/INS394/2010(H1N1)
HA	CY066455	H1N1	01/11/2010	USA	A/California/VRDL2/2010(H1N1)
HA	KT181148	H1N1	01/11/2010	Singapore	A/Singapore/DMS67/2010
HA	CY071215	H1N1	01/11/2010	Germany	A/Frankfurt/INS403/2010(H1N1)
HA	JX308973	H1N1	01/11/2010	Singapore	A/Singapore/GP132/2010
HA	CY061139	H1N1	01/11/2010	USA	A/Texas/JMS407/2010(H1N1)
HA	HM752477	H1N1	01/11/2010	Thailand	A/Thailand/CU-MV10/2010
HA	CY100514	H1N1	01/11/2010	Mexico	A/Mexico City/INER18/2010
HA	JX308978	H1N1	01/11/2010	Singapore	A/Singapore/GP155/2010
HA	KM213270	H1N1	01/11/2010	Oman	A/Oman/SQUH-37/2010
HA	AB551880	H1N1	01/12/2010	Japan	A/Nagasaki/HA-10-11/2010(H1N1)
HA	AB551877	H1N1	01/12/2010	Japan	A/Nagasaki/HA-10-7/2010(H1N1)
HA	CY061147	H1N1	01/12/2010	USA	A/Texas/JMS408/2010(H1N1)
HA	AB704486	H1N1	01/12/2010	Japan	A/Tochigi/10/2010
HA	HQ228144	H1N1	01/12/2010	Finland	A/Finland/2/2010
HA	KC780310	H1N1	01/12/2010	USA	A/New York/02/2010
HA	KC781895	H1N1	01/12/2010	USA	A/North Carolina/01/2010
HA	JQ612507	H1N1	01/12/2010	Hungary	A/Hungary/2/2010
HA	KC780888	H1N1	01/12/2010	USA	A/Nevada/01/2010
HA	JX309130	H1N1	01/12/2010	Singapore	A/Singapore/KK25/2010
HA	AB551876	H1N1	01/12/2010	Japan	A/Nagasaki/HA-10-6/2010(H1N1)
HA	CY062066	H1N1	01/12/2010	USA	A/New York/1680/2010(H1N1)
HA	AB551875	H1N1	01/12/2010	Japan	A/Nagasaki/HA-10-5/2010(H1N1)
HA	KP637670	H1N1	01/12/2010	Thailand	A/Thailand/SP08358/2010
HA	JX309098	H1N1	01/12/2010	Singapore	A/Singapore/GP166/2010
HA	JX309086	H1N1	01/12/2010	Singapore	A/Singapore/TT31/2010
HA	JX308982	H1N1	01/12/2010	Singapore	A/Singapore/GP174/2010
HA	JX309102	H1N1	01/13/2010	Singapore	A/Singapore/GP189/2010
HA	CY074982	H1N1	01/13/2010	Thailand	A/Thailand/CU-H1222/2010(H1N1)
HA	CY075912	H1N1	01/13/2010	India	A/Ngp/NIV22704/2010(H1N1)
HA	AB704478	H1N1	01/13/2010	Japan	A/Tochigi/2/2010
HA	CY071135	H1N1	01/13/2010	Greece	A/Athens/INS389/2010(H1N1)
HA	KC780947	H1N1	01/13/2010	USA	A/Texas/01/2010
HA	JX309106	H1N1	01/13/2010	Singapore	A/Singapore/GP205/2010
HA	KC780646	H1N1	01/13/2010	USA	A/Maryland/03/2010
HA	HQ228145	H1N1	01/13/2010	Finland	A/Finland/3/2010
HA	JQ065238	H1N1	01/14/2010	China	A/Shenzhen/57/2010
HA	JQ065231	H1N1	01/14/2010	China	A/Shenzhen/7/2010
HA	JQ065232	H1N1	01/14/2010	China	A/Shenzhen/8/2010
HA	JQ065236	H1N1	01/14/2010	China	A/Shenzhen/37/2010
HA	JQ065234	H1N1	01/14/2010	China	A/Shenzhen/27/2010
HA	JQ065235	H1N1	01/14/2010	China	A/Shenzhen/31/2010
HA	JX309001	H1N1	01/14/2010	Singapore	A/Singapore/GP220/2010
HA	JX120566	H1N1	01/14/2010	China	A/Anhui/1225/2010
HA	CY103938	H1N1	01/14/2010	Brazil	A/Pernambuco/732/2010
HA	KM213271	H1N1	01/14/2010	Oman	A/Oman/SQUH-38/2010
HA	KT181150	H1N1	01/14/2010	Singapore	A/Singapore/DMS68/2010
HA	LN867804	H1N1	01/14/2010	United Ki	A/England/01220740/2010
HA	KC780419	H1N1	01/14/2010	USA	A/Indiana/01/2010
HA	CY071143	H1N1	01/14/2010	Greece	A/Athens/INS390/2010(H1N1)
HA	CY072534	H1N1	01/14/2010	USA	A/New York/INS432/2010(H1N1)

HA	JQ065233	H1N1	01/14/2010	China	A/Shenzhen/9/2010
HA	JX309134	H1N1	01/14/2010	Singapore	A/Singapore/TT37/2010
HA	JQ065237	H1N1	01/14/2010	China	A/Shenzhen/50/2010
HA	KC780235	H1N1	01/14/2010	USA	A/Kentucky/01/2010
HA	JX309017	H1N1	01/15/2010	Singapore	A/Singapore/GP256/2010
HA	KP317226	H1N1	01/15/2010	India	A/Jammu & Kashmir/022/2010
HA	CY062074	H1N1	01/15/2010	USA	A/New York/1796/2010(H1N1)
HA	KC781920	H1N1	01/15/2010	USA	A/Delaware/01/2010
HA	JN631052	H1N1	01/15/2010	China	A/Shanghai/2167T/2010
HA	CY061326	H1N1	01/15/2010	Argentina	A/Argentina/HNRG102/2010
HA	JX309009	H1N1	01/15/2010	Singapore	A/Singapore/GP241/2010
HA	KC782005	H1N1	01/16/2010	USA	A/Connecticut/01/2010
HA	KC781245	H1N1	01/16/2010	USA	A/Arizona/01/2010
HA	KT181151	H1N1	01/16/2010	Singapore	A/Singapore/DMS69/2010
HA	KC780365	H1N1	01/17/2010	USA	A/Pennsylvania/01/2010
HA	AB551878	H1N1	01/18/2010	Japan	A/Nagasaki/HA-10-9/2010(H1N1)
HA	AB551882	H1N1	01/18/2010	Japan	A/Nagasaki/HA-10-13/2010(H1N1)
HA	CY080315	H1N1	01/18/2010	Thailand	A/Thailand/H1255/2010
HA	CY129499	H1N1	01/18/2010	Germany	A/Wurzburg/INS578/2010
HA	HM752473	H1N1	01/18/2010	Thailand	A/Thailand/CU-MV6/2010
HA	AB551881	H1N1	01/18/2010	Japan	A/Nagasaki/HA-10-12/2010(H1N1)
HA	AB551879	H1N1	01/18/2010	Japan	A/Nagasaki/HA-10-10/2010(H1N1)
HA	CY100522	H1N1	01/18/2010	Mexico	A/Mexico City/INER9/2010
HA	LN846438	H1N1	01/18/2010	United Ki	A/England/32/2010
HA	CY062090	H1N1	01/18/2010	USA	A/New York/1999/2010(H1N1)
HA	CY062082	H1N1	01/18/2010	USA	A/New York/1998/2010(H1N1)
HA	JX309110	H1N1	01/18/2010	Singapore	A/Singapore/GP266/2010
HA	KC780569	H1N1	01/18/2010	USA	A/Georgia/03/2010
HA	CY071295	H1N1	01/18/2010	Greece	A/Athens/INS417/2010(H1N1)
HA	CY167772	H1N1	01/19/2010	USA	A/Tennessee/F1035/2010
HA	JX309114	H1N1	01/19/2010	Singapore	A/Singapore/GP293/2010
HA	CY066463	H1N1	01/19/2010	USA	A/California/VRDL3/2010(H1N1)
HA	JX309118	H1N1	01/19/2010	Singapore	A/Singapore/GP311/2010
HA	KC782067	H1N1	01/19/2010	USA	A/California/01/2010
HA	KC782182	H1N1	01/19/2010	USA	A/Mississippi/01/2010
HA	CY062098	H1N1	01/19/2010	USA	A/New York/2175/2010(H1N1)
HA	CY062114	H1N1	01/20/2010	USA	A/New York/2598/2010(H1N1)
HA	AB551883	H1N1	01/20/2010	Japan	A/Nagasaki/HA-10-69/2010(H1N1)
HA	CY062130	H1N1	01/20/2010	USA	A/New York/2876/2010(H1N1)
HA	CY061155	H1N1	01/20/2010	USA	A/Texas/JMS409/2010(H1N1)
HA	JX308993	H1N1	01/20/2010	Singapore	A/Singapore/GP329/2010
HA	KC780334	H1N1	01/20/2010	USA	A/North Carolina/03/2010
HA	CY062106	H1N1	01/20/2010	USA	A/New York/2372/2010(H1N1)
HA	KF849774	H1N1	01/21/2010	Thailand	A/Thailand/SirirajICRC_SEA-002(33)/2
HA	KP019839	H1N1	01/21/2010	China	A/FuZhou/SWL1223/2010
HA	KM885028	H1N1	01/21/2010	India	A/Khargone/293/2010
HA	KF849776	H1N1	01/21/2010	Thailand	A/Thailand/SirirajICRC_SEA-001(34)/2
HA	CY071167	H1N1	01/21/2010	Greece	A/Athens/INS396/2010(H1N1)
HA	KC780583	H1N1	01/21/2010	USA	A/Virginia/01/2010
HA	HM752474	H1N1	01/21/2010	Thailand	A/Thailand/CU-MV7/2010
HA	JX309025	H1N1	01/21/2010	Singapore	A/Singapore/GP368/2010
HA	HM752475	H1N1	01/22/2010	Thailand	A/Thailand/CU-MV8/2010
HA	CY062146	H1N1	01/22/2010	USA	A/New York/2963/2010(H1N1)
HA	CY062122	H1N1	01/22/2010	USA	A/New York/2648/2010(H1N1)

HA	CY061163	H1N1	01/22/2010	USA	A/Texas/JMS410/2010(H1N1)
HA	KT181153	H1N1	01/22/2010	Singapore	A/Singapore/DMS71/2010
HA	KC781419	H1N1	01/23/2010	USA	A/Kansas/02/2010
HA	KC780353	H1N1	01/23/2010	USA	A/Alabama/02/2010
HA	CY062154	H1N1	01/23/2010	USA	A/New York/2971/2010(H1N1)
HA	CY071175	H1N1	01/24/2010	Greece	A/Athens/INS397/2010(H1N1)
HA	KC780464	H1N1	01/24/2010	USA	A/Wisconsin/01/2010
HA	KC780442	H1N1	01/24/2010	USA	A/Georgia/02/2010
HA	CY071183	H1N1	01/24/2010	Greece	A/Athens/INS398/2010(H1N1)
HA	KC780498	H1N1	01/24/2010	USA	A/Wisconsin/01/2010
HA	KC780426	H1N1	01/25/2010	USA	A/Kentucky/02/2010
HA	JX309057	H1N1	01/25/2010	Singapore	A/Singapore/GP437/2010(H1N1)
HA	CY063203	H1N1	01/25/2010	USA	A/California/VRDL13/2010(H1N1)
HA	JX309041	H1N1	01/25/2010	Singapore	A/Singapore/GP398/2010
HA	CY061171	H1N1	01/25/2010	USA	A/Texas/JMS411/2010(H1N1)
HA	CY071303	H1N1	01/25/2010	Greece	A/Athens/INS419/2010(H1N1)
HA	KT181154	H1N1	01/25/2010	Singapore	A/Singapore/DMS72/2010
HA	JF340083	H1N1	01/25/2010	Russia	A/St.Petersburg/14/2010
HA	CY062162	H1N1	01/25/2010	USA	A/New York/3230/2010(H1N1)
HA	CY063211	H1N1	01/25/2010	USA	A/California/VRDL14/2010(H1N1)
HA	JX309033	H1N1	01/25/2010	Singapore	A/Singapore/GP413/2010
HA	CY083016	H1N1	01/25/2010	Senegal	A/Dakar/WRAIR0018N/2010
HA	CY073126	H1N1	01/25/2010	Mexico	A/Mexico City/WRAIR1773T/2010(H1N1)
HA	CY083535	H1N1	01/26/2010	Mexico	A/Mexico City/WRAIR1752N/2010(H1N1)
HA	CY083032	H1N1	01/26/2010	Senegal	A/Dakar/WRAIR0019T/2010
HA	KC780871	H1N1	01/26/2010	USA	A/California/03/2010
HA	CY062186	H1N1	01/26/2010	USA	A/New York/3251/2010(H1N1)
HA	CY063187	H1N1	01/26/2010	USA	A/California/VRDL11/2010(H1N1)
HA	HQ228147	H1N1	01/26/2010	Finland	A/Finland/5/2010
HA	CY062170	H1N1	01/26/2010	USA	A/New York/3236/2010(H1N1)
HA	CY062138	H1N1	01/26/2010	USA	A/New York/2960/2010(H1N1)
HA	CY080307	H1N1	01/26/2010	Thailand	A/Thailand/C602/2010(H1N1)
HA	JX309049	H1N1	01/26/2010	Singapore	A/Singapore/GP449/2010(H1N1)
HA	CY071311	H1N1	01/27/2010	Greece	A/Athens/INS420/2010(H1N1)
HA	CY063195	H1N1	01/27/2010	USA	A/California/VRDL12/2010(H1N1)
HA	JX308944	H1N1	01/27/2010	Singapore	A/Singapore/GP479/2010
HA	CY065019	H1N1	01/27/2010	USA	A/New York/3834/2010(H1N1)
HA	CY062178	H1N1	01/27/2010	USA	A/New York/3250/2010(H1N1)
HA	CY092638	H1N1	01/27/2010	Australia	A/Sydney/DD3-37/2010
HA	CY065027	H1N1	01/27/2010	USA	A/New York/3835/2010(H1N1)
HA	KP019840	H1N1	01/27/2010	China	A/FuZhou/SWL1286/2010
HA	CY063848	H1N1	01/27/2010	Singapore	A/Singapore/KK066/2010(H1N1)
HA	KC781448	H1N1	01/27/2010	USA	A/Michigan/01/2010
HA	JX309065	H1N1	01/27/2010	Singapore	A/Singapore/GP489/2010(H1N1)
HA	KC780661	H1N1	01/27/2010	USA	A/South Carolina/01/2010
HA	KP317228	H1N1	01/28/2010	India	A/Madhya Pradesh/024/2010
HA	KF849777	H1N1	01/28/2010	Thailand	A/Thailand/SirirajICRC_SEA-003(33)/2
HA	KP317224	H1N1	01/28/2010	India	A/Assam/020/2010
HA	JX309073	H1N1	01/28/2010	Singapore	A/Singapore/GP512/2010(H1N1)
HA	KC780447	H1N1	01/28/2010	USA	A/Nebraska/01/2010
HA	CY065035	H1N1	01/29/2010	USA	A/New York/3866/2010(H1N1)
HA	HM601436	H1N1	01/29/2010	Brazil	A/Goias/16651/2010
HA	KF860846	H1N1	01/29/2010	Spain	A/Extremadura/6530/2010
HA	CY071263	H1N1	01/30/2010	Greece	A/Athens/INS411/2010(H1N1)

HA	KT181157	H1N1	01/31/2010	Singapore	A/Singapore/DMS74/2010
HA	AB745261	H1N1	02/2010	Japan	A/Tottori/ST502/2010
HA	HQ419004	H1N1	02/2010	Iran	A/Shiraz/1/2010(H1N1)
HA	HQ419011	H1N1	02/2010	Iran	A/Shiraz/8/2010(H1N1)
HA	HQ419005	H1N1	02/2010	Iran	A/Shiraz/2/2010(H1N1)
HA	HQ419010	H1N1	02/2010	Iran	A/Shiraz/7/2010(H1N1)
HA	JN381268	H1N1	02/2010	Taiwan	A/Taiwan/65337/2010
HA	JQ365007	H1N1	02/2010	China	A/Hangzhou/117/2010
HA	JQ365008	H1N1	02/2010	China	A/Hangzhou/124/2010
HA	AB745263	H1N1	02/2010	Japan	A/Tottori/ST505/2010
HA	HQ419006	H1N1	02/2010	Iran	A/Shiraz/3/2010(H1N1)
HA	CY096805	H1N1	02/2010	Thailand	A/Thailand/ICRC-BKK34002/2010
HA	CY096806	H1N1	02/2010	Thailand	A/Thailand/ICRC-BKK34004/2010
HA	JQ365010	H1N1	02/2010	China	A/Hangzhou/129/2010
HA	JN381267	H1N1	02/2010	Taiwan	A/Taiwan/65299/2010
HA	AB745262	H1N1	02/2010	Japan	A/Tottori/ST503/2010
HA	HQ419007	H1N1	02/2010	Iran	A/Shiraz/4/2010(H1N1)
HA	HQ419009	H1N1	02/2010	Iran	A/Shiraz/6/2010(H1N1)
HA	HQ419008	H1N1	02/2010	Iran	A/Shiraz/5/2010(H1N1)
HA	JQ365009	H1N1	02/2010	China	A/Hangzhou/125/2010
HA	CY067612	H1N1	02/2010	China	A/Qingdao/123/2010
HA	AB558537	H1N1	02/01/2010	Japan	A/Nagasaki/HA-10-14/2010(H1N1)
HA	CY061179	H1N1	02/01/2010	USA	A/Texas/JMS412/2010(H1N1)
HA	CY065003	H1N1	02/01/2010	USA	A/New York/3682/2010(H1N1)
HA	KC780949	H1N1	02/01/2010	USA	A/New Mexico/02/2010
HA	KC780601	H1N1	02/01/2010	USA	A/California/02/2010
HA	KC780315	H1N1	02/01/2010	USA	A/Massachusetts/01/2010
HA	CY064995	H1N1	02/01/2010	USA	A/New York/3681/2010(H1N1)
HA	CY065011	H1N1	02/01/2010	USA	A/New York/3683/2010(H1N1)
HA	CY066487	H1N1	02/01/2010	USA	A/California/VRDL7/2010(H1N1)
HA	KC780290	H1N1	02/01/2010	USA	A/Colorado/03/2010
HA	JX309211	H1N1	02/01/2010	Singapore	A/Singapore/KK70/2010
HA	AB558538	H1N1	02/01/2010	Japan	A/Nagasaki/HA-10-15/2010(H1N1)
HA	CY063830	H1N1	02/02/2010	Singapore	A/Singapore/GP562/2010(H1N1)
HA	HQ228146	H1N1	02/02/2010	Finland	A/Finland/4/2010
HA	KT181166	H1N1	02/02/2010	Singapore	A/Singapore/DMS82/2010
HA	KC780537	H1N1	02/02/2010	USA	A/Florida/02/2010
HA	CY071271	H1N1	02/02/2010	Greece	A/Athens/INS412/2010(H1N1)
HA	KC780389	H1N1	02/02/2010	USA	A/North Carolina/04/2010
HA	JX309156	H1N1	02/02/2010	Singapore	A/Singapore/GP567/2010
HA	KF849773	H1N1	02/02/2010	Thailand	A/Thailand/SirirajICRC_SEA-002(34)/2
HA	KP019841	H1N1	02/02/2010	China	A/FuZhou/SWL1338/2010
HA	KC781411	H1N1	02/02/2010	USA	A/Georgia/09/2010
HA	KC782071	H1N1	02/02/2010	USA	A/Arizona/02/2010
HA	CY064987	H1N1	02/02/2010	USA	A/California/VRDL6/2010(H1N1)
HA	CY061187	H1N1	02/03/2010	USA	A/Texas/JMS413/2010(H1N1)
HA	KC780695	H1N1	02/03/2010	USA	A/Indiana/03/2010
HA	KC781452	H1N1	02/03/2010	USA	A/Louisiana/01/2010
HA	CY065051	H1N1	02/03/2010	USA	A/New York/4662/2010(H1N1)
HA	KT181158	H1N1	02/03/2010	Singapore	A/Singapore/DMS75/2010
HA	CY071319	H1N1	02/03/2010	Greece	A/Athens/INS421/2010(H1N1)
HA	CY072998	H1N1	02/03/2010	Nicaragua	A/Managua/07193_09/2010(H1N1)
HA	JF929780	H1N1	02/04/2010	China	A/Guangdong/2315/2010
HA	LN846494	H1N1	02/04/2010	United Ki	A/England/01220736/2010

HA	LN850030	H1N1	02/04/2010	United Ki	A/England/29/2010
HA	KC782022	H1N1	02/04/2010	USA	A/Vermont/01/2010
HA	CY061195	H1N1	02/04/2010	USA	A/Texas/JMS414/2010(H1N1)
HA	CY065043	H1N1	02/04/2010	USA	A/New York/4294/2010(H1N1)
HA	CY066479	H1N1	02/04/2010	USA	A/California/VRDL5/2010(H1N1)
HA	CY167324	H1N1	02/04/2010	USA	A/Tennessee/F1042A/2010
HA	JX309178	H1N1	02/05/2010	Singapore	A/Singapore/GP643/2010
HA	CY073733	H1N1	02/05/2010	Estonia	A/Tallinn/INS431/2010(H1N1)
HA	CY071375	H1N1	02/05/2010	Germany	A/Berlin/INS430/2010(H1N1)
HA	CY083817	H1N1	02/05/2010	USA	A/Los Angeles/INS423/2010(H1N1)
HA	KT181160	H1N1	02/05/2010	Singapore	A/Singapore/DMS76/2010
HA	CY071279	H1N1	02/05/2010	Greece	A/Athens/INS413/2010(H1N1)
HA	KF849775	H1N1	02/05/2010	Thailand	A/Thailand/SirirajICRC_SEA-003(34)/2
HA	KC781300	H1N1	02/05/2010	USA	A/Georgia/05/2010
HA	KC781255	H1N1	02/05/2010	USA	A/Georgia/06/2010
HA	CY071287	H1N1	02/05/2010	Greece	A/Athens/INS414/2010(H1N1)
HA	CY061203	H1N1	02/06/2010	USA	A/Texas/JMS415/2010(H1N1)
HA	KC781263	H1N1	02/06/2010	USA	A/Georgia/04/2010
HA	KC781284	H1N1	02/06/2010	USA	A/Georgia/07/2010
HA	KC780239	H1N1	02/06/2010	USA	A/Georgia/01/2010
HA	KR030166	H1N1	02/06/2010	China	A/Guangdong/501/2010
HA	KP637286	H1N1	02/07/2010	Thailand	A/Thailand/IY07115/2010
HA	KP317229	H1N1	02/07/2010	India	A/Chattisgarh/025/2010
HA	JX309181	H1N1	02/07/2010	Singapore	A/Singapore/KK77/2010
HA	KC781389	H1N1	02/07/2010	USA	A/Alaska/01/2010
HA	AB558539	H1N1	02/08/2010	Japan	A/Nagasaki/HA-10-17/2010(H1N1)
HA	JX309161	H1N1	02/08/2010	Singapore	A/Singapore/GP661/2010
HA	KC780586	H1N1	02/08/2010	USA	A/Maryland/04/2010
HA	KC781181	H1N1	02/08/2010	USA	A/Wyoming/01/2010
HA	JX309167	H1N1	02/08/2010	Singapore	A/Singapore/TT106/2010
HA	CY066471	H1N1	02/08/2010	USA	A/California/VRDL4/2010(H1N1)
HA	KC780724	H1N1	02/08/2010	USA	A/Hawaii/03/2010
HA	KT181161	H1N1	02/08/2010	Singapore	A/Singapore/DMS77/2010
HA	KP637838	H1N1	02/09/2010	Thailand	A/Thailand/IY07144/2010
HA	CY090861	H1N1	02/09/2010	Netherlar	A/Netherlands/2631c/2010(H1N1)
HA	KT241014	H1N1	02/09/2010	India	A/Indore/379/2010
HA	CY100538	H1N1	02/09/2010	Mexico	A/Mexico City/INER11/2010
HA	CY066503	H1N1	02/09/2010	USA	A/California/VRDL9/2010(H1N1)
HA	JX309202	H1N1	02/09/2010	Singapore	A/Singapore/GP680/2010
HA	KC261320	H1N1	02/09/2010	Mexico	A/Mexico City/INERPHAC3/2010(H1N1)
HA	KC780453	H1N1	02/09/2010	USA	A/New York/03/2010
HA	JN588793	H1N1	02/09/2010	Cambodia	A/Cambodia/U216/2010
HA	CY167332	H1N1	02/09/2010	USA	A/Tennessee/F1052A/2010
HA	CY100530	H1N1	02/09/2010	Mexico	A/Mexico City/INER10/2010
HA	KT181162	H1N1	02/10/2010	Singapore	A/Singapore/DMS78/2010
HA	KC781428	H1N1	02/10/2010	USA	A/Maine/01/2010
HA	KC780502	H1N1	02/10/2010	USA	A/Utah/02/2010
HA	JX309205	H1N1	02/10/2010	Singapore	A/Singapore/GP690/2010
HA	KP019842	H1N1	02/10/2010	China	A/FuZhou/SWL1395/2010
HA	JX309187	H1N1	02/11/2010	Singapore	A/Singapore/GP720/2010
HA	CY063179	H1N1	02/11/2010	USA	A/California/VRDL10/2010(H1N1)
HA	KC781033	H1N1	02/11/2010	USA	A/California/04/2010
HA	KC780625	H1N1	02/11/2010	USA	A/Indiana/02/2010
HA	JN588794	H1N1	02/11/2010	Cambodia	A/Cambodia/U219/2010

HA	KC780350	H1N1	02/11/2010	USA	A/Delaware/02/2010
HA	CY063835	H1N1	02/12/2010	Singapore	A/Singapore/GP737/2010(H1N1)
HA	CY075915	H1N1	02/12/2010	India	A/Lur/NIV24770/2010
HA	JX309172	H1N1	02/12/2010	Singapore	A/Singapore/GP735/2010
HA	JF906183	H1N1	02/12/2010	Netherlar	A/Netherlands/2631_1202/2010
HA	CY063853	H1N1	02/12/2010	Singapore	A/Singapore/KK081/2010(H1N1)
HA	KJ856114	H1N1	02/12/2010	Sri Lanka	A/Sri Lanka/20887/2010
HA	JX309208	H1N1	02/12/2010	Singapore	A/Singapore/KK80/2010
HA	CY066495	H1N1	02/13/2010	USA	A/California/VRDL8/2010(H1N1)
HA	KC780367	H1N1	02/15/2010	USA	A/Iowa/03/2010
HA	AB558541	H1N1	02/15/2010	Japan	A/Nagasaki/HA-10-19/2010(H1N1)
HA	AB558540	H1N1	02/15/2010	Japan	A/Nagasaki/HA-10-18/2010(H1N1)
HA	JN588795	H1N1	02/16/2010	Cambodia	A/Cambodia/U221/2010
HA	CY083040	H1N1	02/16/2010	Senegal	A/Dakar/WRAIR0020T/2010
HA	CY097789	H1N1	02/16/2010	Senegal	A/Dakar/WRAIR0020T/2010
HA	KC782004	H1N1	02/17/2010	USA	A/Minnesota/02/2010
HA	CY075075	H1N1	02/17/2010	Greece	A/Athens/INS416/2010(H1N1)
HA	JX309184	H1N1	02/17/2010	Singapore	A/Singapore/KK87/2010
HA	KC780525	H1N1	02/17/2010	USA	A/Wisconsin/02/2010
HA	KP637862	H1N1	02/18/2010	Thailand	A/Thailand/SS08248/2010
HA	CY167340	H1N1	02/18/2010	USA	A/Tennessee/F1057c56/2010
HA	CY167348	H1N1	02/18/2010	USA	A/Tennessee/F1060/2010
HA	CY072088	H1N1	02/18/2010	Brazil	A/Rio de Janeiro/491/2010(H1N1)
HA	KC781923	H1N1	02/19/2010	USA	A/New Hampshire/02/2010
HA	KC781197	H1N1	02/19/2010	USA	A/Kentucky/03/2010
HA	KC780069	H1N1	02/19/2010	USA	A/New Hampshire/02/2010
HA	LN867349	H1N1	02/20/2010	United Ki	A/England/01220741/2010
HA	KT181163	H1N1	02/20/2010	Singapore	A/Singapore/DMS80/2010
HA	CY065059	H1N1	02/21/2010	USA	A/New York/6530/2010(H1N1)
HA	AB558543	H1N1	02/22/2010	Japan	A/Nagasaki/HA-10-21/2010(H1N1)
HA	KC781368	H1N1	02/22/2010	USA	A/Wisconsin/04/2010
HA	KC781163	H1N1	02/22/2010	USA	A/Kentucky/04/2010
HA	JX309175	H1N1	02/22/2010	Singapore	A/Singapore/GP758/2010
HA	AB558542	H1N1	02/22/2010	Japan	A/Nagasaki/HA-10-20/2010(H1N1)
HA	CY096594	H1N1	02/23/2010	USA	A/District of Columbia/INS527/2010(
HA	CY071327	H1N1	02/23/2010	Thailand	A/Bangkok/INS424/2010(H1N1)
HA	KC782140	H1N1	02/23/2010	USA	A/Tennessee/01/2010
HA	KC780361	H1N1	02/24/2010	USA	A/North Carolina/05/2010
HA	JN588791	H1N1	02/24/2010	Cambodia	A/Cambodia/U127/2010
HA	KT181165	H1N1	02/24/2010	Singapore	A/Singapore/DMS81/2010
HA	JN588796	H1N1	02/24/2010	Cambodia	A/Cambodia/U223/2010
HA	KC781258	H1N1	02/24/2010	USA	A/Michigan/02/2010
HA	CY075916	H1N1	02/24/2010	India	A/Awb/NIV25611/2010
HA	CY129694	H1N1	02/24/2010	Greece	A/Athens/INS555/2010
HA	JX309196	H1N1	02/24/2010	Singapore	A/Singapore/GP784/2010
HA	JX309190	H1N1	02/25/2010	Singapore	A/Singapore/TT114/2010
HA	KC781915	H1N1	02/25/2010	USA	A/New York/04/2010
HA	CY071343	H1N1	02/25/2010	Thailand	A/Bangkok/INS426/2010(H1N1)
HA	KP019843	H1N1	02/25/2010	China	A/FuZhou/SWL1494/2010
HA	CY071335	H1N1	02/25/2010	Thailand	A/Bangkok/INS425/2010(H1N1)
HA	CY167364	H1N1	02/26/2010	USA	A/Tennessee/F1066c65/2010
HA	JX309193	H1N1	02/26/2010	Singapore	A/Singapore/EN35/2010
HA	CY167356	H1N1	02/26/2010	USA	A/Tennessee/F1063A/2010
HA	CY063840	H1N1	02/26/2010	Singapore	A/Singapore/GP999/2010(H1N1)

HA	CY167372	H1N1	02/26/2010	USA	A/Tennessee/F1067/2010
HA	JX309199	H1N1	02/26/2010	Singapore	A/Singapore/GP811/2010
HA	CY096809	H1N1	03/2010	Thailand	A/Thailand/ICRC-NMA1/2010
HA	JQ365014	H1N1	03/2010	China	A/Hangzhou/178/2010
HA	CY067630	H1N1	03/2010	China	A/Qingdao/220/2010
HA	AB745264	H1N1	03/2010	Japan	A/Tottori/ST509/2010
HA	JQ365013	H1N1	03/2010	China	A/Hangzhou/157/2010
HA	JQ365011	H1N1	03/2010	China	A/Hangzhou/141/2010
HA	JQ365012	H1N1	03/2010	China	A/Hangzhou/156/2010
HA	KC781169	H1N1	03/01/2010	USA	A/Alabama/03/2010
HA	JX309280	H1N1	03/01/2010	Singapore	A/Singapore/GP820/2010
HA	JX309259	H1N1	03/01/2010	Singapore	A/Singapore/EN39/2010
HA	CY065107	H1N1	03/01/2010	USA	A/New York/7480/2010(H1N1)
HA	AB558544	H1N1	03/01/2010	Japan	A/Nagasaki/HA-10-22/2010(H1N1)
HA	KT181167	H1N1	03/01/2010	Singapore	A/Singapore/DMS83/2010
HA	CY167404	H1N1	03/02/2010	USA	A/Tennessee/F1072c71/2010
HA	JX309271	H1N1	03/02/2010	Singapore	A/Singapore/KK90/2010
HA	HM569740	H1N1	03/02/2010	Russia	A/Orenburg/IIV-13/2010(H1N1)
HA	KC781911	H1N1	03/02/2010	USA	A/Massachusetts/02/2010
HA	CY072082	H1N1	03/02/2010	Brazil	A/Alagoas/96/2010(H1N1)
HA	KC781413	H1N1	03/02/2010	USA	A/Pennsylvania/03/2010
HA	CY167380	H1N1	03/02/2010	USA	A/Tennessee/F1068/2010
HA	CY167388	H1N1	03/02/2010	USA	A/Tennessee/F1071/2010
HA	HM124383	H1N1	03/02/2010	Russia	A/Orenburg/IIV-13/2010(H1N1)
HA	KC781355	H1N1	03/03/2010	USA	A/Iowa/04/2010
HA	CY071351	H1N1	03/04/2010	Thailand	A/Bangkok/INS427/2010(H1N1)
HA	CY071359	H1N1	03/04/2010	Thailand	A/Bangkok/INS428/2010(H1N1)
HA	KC780738	H1N1	03/04/2010	USA	A/California/05/2010
HA	CY075917	H1N1	03/04/2010	India	A/Hnl/NIV26271/2010
HA	CY072089	H1N1	03/04/2010	Brazil	A/Rio de Janeiro/88/2010(H1N1)
HA	CY167396	H1N1	03/05/2010	USA	A/Tennessee/F1071B/2010
HA	CY072074	H1N1	03/05/2010	Brazil	A/Alagoas/115/2010(H1N1)
HA	CY071367	H1N1	03/05/2010	USA	A/Newark/INS429/2010(H1N1)
HA	KC781372	H1N1	03/05/2010	USA	A/South Carolina/02/2010
HA	KC780610	H1N1	03/06/2010	USA	A/Arizona/03/2010
HA	CY103939	H1N1	03/06/2010	Brazil	A/Pernambuco/744/2010
HA	KC781343	H1N1	03/07/2010	USA	A/Kansas/03/2010
HA	JF929781	H1N1	03/07/2010	China	A/Guangdong/2989/2010
HA	KC780726	H1N1	03/07/2010	USA	A/New Mexico/03/2010
HA	HM624015	H1N1	03/08/2010	Brazil	A/Sao Paulo/15463/2010
HA	KP019844	H1N1	03/08/2010	China	A/FuZhou/SWL1549/2010
HA	KP019845	H1N1	03/08/2010	China	A/FuZhou/SWL1565/2010
HA	KC781406	H1N1	03/08/2010	USA	A/Utah/03/2010
HA	KC780587	H1N1	03/08/2010	USA	A/Hawaii/04/2010
HA	JX309268	H1N1	03/08/2010	Singapore	A/Singapore/GP866/2010
HA	JX309256	H1N1	03/08/2010	Singapore	A/Singapore/KK98/2010
HA	AB558545	H1N1	03/08/2010	Japan	A/Nagasaki/HA-10-24/2010(H1N1)
HA	KC781863	H1N1	03/08/2010	USA	A/Mississippi/02/2010
HA	AB558546	H1N1	03/08/2010	Japan	A/Nagasaki/HA-10-25/2010(H1N1)
HA	KM203132	H1N1	03/09/2010	India	A/Kerala/RGCBC700/2010
HA	CY075918	H1N1	03/09/2010	India	A/Pune/NIV26410/2010
HA	KC781173	H1N1	03/09/2010	USA	A/North Carolina/06/2010
HA	KM203131	H1N1	03/09/2010	India	A/Kerala/RGCBC699/2010
HA	JX309234	H1N1	03/10/2010	Singapore	A/Singapore/KK100/2010(H1N1)

HA	CY072075	H1N1	03/10/2010	Brazil	A/Alagoas/119/2010(H1N1)
HA	KP019846	H1N1	03/10/2010	China	A/FuZhou/SWL1563/2010
HA	KC781399	H1N1	03/11/2010	USA	A/Nebraska/02/2010
HA	KC781335	H1N1	03/12/2010	USA	A/Minnesota/03/2010
HA	KC781210	H1N1	03/12/2010	USA	A/Wisconsin/03/2010
HA	KT181170	H1N1	03/12/2010	Singapore	A/Singapore/DMS86/2010
HA	JX309274	H1N1	03/12/2010	Singapore	A/Singapore/GP903/2010
HA	KC781158	H1N1	03/12/2010	USA	A/Tennessee/02/2010
HA	CY072077	H1N1	03/13/2010	Brazil	A/Alagoas/143/2010(H1N1)
HA	JX309245	H1N1	03/15/2010	Singapore	A/Singapore/TT123/2010
HA	AB558548	H1N1	03/15/2010	Japan	A/Nagasaki/HA-10-27/2010(H1N1)
HA	CY072076	H1N1	03/15/2010	Brazil	A/Alagoas/140/2010(H1N1)
HA	JN588790	H1N1	03/15/2010	Cambodia	A/Cambodia/U099/2010
HA	CY167412	H1N1	03/15/2010	USA	A/Tennessee/F1074/2010
HA	KC781175	H1N1	03/15/2010	USA	A/Alabama/04/2010
HA	AB558547	H1N1	03/15/2010	Japan	A/Nagasaki/HA-10-26/2010(H1N1)
HA	KT181173	H1N1	03/16/2010	Singapore	A/Singapore/DMS88/2010
HA	KC781142	H1N1	03/16/2010	USA	A/Florida/05/2010
HA	JN588792	H1N1	03/16/2010	Cambodia	A/Cambodia/U132/2010
HA	CY167420	H1N1	03/16/2010	USA	A/Tennessee/F1076/2010
HA	CY167436	H1N1	03/16/2010	USA	A/Tennessee/F1078/2010
HA	LN850125	H1N1	03/18/2010	United Ki	A/England/11/2010
HA	KC781432	H1N1	03/18/2010	USA	A/Florida/04/2010
HA	LN867676	H1N1	03/18/2010	United Ki	A/England/WTSI2067/2010
HA	LN867423	H1N1	03/18/2010	United Ki	A/England/01180015/2010
HA	LN867486	H1N1	03/18/2010	United Ki	A/England/01180017/2010
HA	LN867397	H1N1	03/18/2010	United Ki	A/England/WTSI1783/2010
HA	LN850421	H1N1	03/18/2010	United Ki	A/England/01180007/2010
HA	LN850102	H1N1	03/18/2010	United Ki	A/England/WTSI1807/2010
HA	LN850345	H1N1	03/18/2010	United Ki	A/England/01180018/2010
HA	LN850078	H1N1	03/18/2010	United Ki	A/England/00380710/2010
HA	LN846787	H1N1	03/18/2010	United Ki	A/England/10/2010
HA	KC781275	H1N1	03/18/2010	USA	A/Florida/04/2010
HA	LN846534	H1N1	03/18/2010	United Ki	A/England/00380719/2010
HA	LN846590	H1N1	03/18/2010	United Ki	A/England/00380718/2010
HA	LN850337	H1N1	03/18/2010	United Ki	A/England/00380709/2010
HA	LN867780	H1N1	03/18/2010	United Ki	A/England/01180011/2010
HA	LN846510	H1N1	03/18/2010	United Ki	A/England/01180016/2010
HA	LN867820	H1N1	03/18/2010	United Ki	A/England/WTSI2076/2010
HA	LN867373	H1N1	03/18/2010	United Ki	A/England/WTSI1774/2010
HA	LN845774	H1N1	03/18/2010	United Ki	A/England/12/2010
HA	CY167780	H1N1	03/19/2010	USA	A/Tennessee/F1080/2010
HA	CY167428	H1N1	03/19/2010	USA	A/Tennessee/F1076A/2010
HA	KC781138	H1N1	03/19/2010	USA	A/Virginia/02/2010
HA	KC781240	H1N1	03/19/2010	USA	A/Georgia/11/2010
HA	CY089371	H1N1	03/19/2010	Nicaragua	A/Managua/5708.01/2010(H1N1)
HA	JX309250	H1N1	03/19/2010	Singapore	A/Singapore/KK103/2010
HA	CY072086	H1N1	03/19/2010	Brazil	A/Bahia/162/2010(H1N1)
HA	JX309253	H1N1	03/19/2010	Singapore	A/Singapore/GP937/2010
HA	CY072084	H1N1	03/19/2010	Brazil	A/Bahia/160/2010(H1N1)
HA	CY167444	H1N1	03/19/2010	USA	A/Tennessee/F1083c80/2010
HA	CY072085	H1N1	03/19/2010	Brazil	A/Bahia/161/2010(H1N1)
HA	KT181174	H1N1	03/20/2010	Singapore	A/Singapore/DMS89/2010
HA	CY080323	H1N1	03/20/2010	Thailand	A/Thailand/CU-H1786/2010(H1N1)

HA	CY096602	H1N1	03/21/2010	USA	A/Cambridge/INS528/2010(H1N1)
HA	KC782128	H1N1	03/22/2010	USA	A/Minnesota/04/2010
HA	JF293316	H1N1	03/22/2010	India	A/India/Blore/2010
HA	CY072078	H1N1	03/22/2010	Brazil	A/Alagoas/166/2010(H1N1)
HA	KM013732	H1N1	03/23/2010	Thailand	A/Bangkok/SIMI508/2010
HA	KT181175	H1N1	03/23/2010	Singapore	A/Singapore/DMS90/2010
HA	AB558549	H1N1	03/23/2010	Japan	A/Nagasaki/HA-10-28/2010(H1N1)
HA	CY090837	H1N1	03/23/2010	Nicaragua	A/Managua/0305_10/2010(H1N1)
HA	JX309262	H1N1	03/24/2010	Singapore	A/Singapore/GP965/2010
HA	CY167452	H1N1	03/24/2010	USA	A/Tennessee/F1089/2010
HA	JF929754	H1N1	03/24/2010	China	A/Guangdong/378/2010
HA	CY072079	H1N1	03/24/2010	Brazil	A/Alagoas/171/2010(H1N1)
HA	KC261321	H1N1	03/25/2010	Mexico	A/Mexico City/INERPHAC4/2010(H1N1)
HA	HM624014	H1N1	03/25/2010	Brazil	A/Piaui/21394/2010
HA	KT181177	H1N1	03/25/2010	Singapore	A/Singapore/DMS91/2010
HA	JX309265	H1N1	03/25/2010	Singapore	A/Singapore/GP982/2010
HA	CY089379	H1N1	03/25/2010	Nicaragua	A/Managua/3246.01/2010(H1N1)
HA	JX309214	H1N1	03/26/2010	Singapore	A/Singapore/GP1003/2010
HA	KC781422	H1N1	03/26/2010	USA	A/Delaware/03/2010
HA	CY079530	H1N1	03/26/2010	Brazil	A/Parana/914/2010
HA	LN867389	H1N1	03/26/2010	United Ki	A/England/01580005/2010
HA	CY072080	H1N1	03/26/2010	Brazil	A/Alagoas/201/2010(H1N1)
HA	HQ595290	H1N1	03/27/2010	Brazil	A/Sao Paulo/22205/2010
HA	KC781136	H1N1	03/27/2010	USA	A/West Virginia/01/2010
HA	JX309217	H1N1	03/29/2010	Singapore	A/Singapore/GP1012/2010
HA	AB558550	H1N1	03/29/2010	Japan	A/Nagasaki/HA-10-30/2010(H1N1)
HA	KC780911	H1N1	03/29/2010	USA	A/Oregon/01/2010
HA	KT181179	H1N1	03/29/2010	Singapore	A/Singapore/DMS92/2010
HA	CY080331	H1N1	03/29/2010	Thailand	A/Thailand/H1818/2010
HA	JX309277	H1N1	03/30/2010	Singapore	A/Singapore/TT128/2010
HA	KC780257	H1N1	03/30/2010	USA	A/Hawaii/05/2010
HA	JX309223	H1N1	03/30/2010	Singapore	A/Singapore/GP1035/2010
HA	KC781118	H1N1	03/31/2010	USA	A/Maryland/05/2010
HA	KC781226	H1N1	03/31/2010	USA	A/Michigan/03/2010
HA	JX309228	H1N1	03/31/2010	Singapore	A/Singapore/GP1040/2010
HA	CY072081	H1N1	03/31/2010	Brazil	A/Alagoas/266/2010(H1N1)
HA	CY080339	H1N1	03/31/2010	Thailand	A/Thailand/CU-H1821/2010(H1N1)
HA	JX309239	H1N1	03/31/2010	Singapore	A/Singapore/KK111/2010
HA	HQ595291	H1N1	03/31/2010	Brazil	A/Piaui/23931/2010

)

)

)

1N1)
H1N1)
1N1)

H1N1)

H1N1)

H1N1)

-1649/2009

)9

2009

N1)

1N1)

1)

1N1)

N1)
N1)

N1)

1N1)

1N1)

1N1)

1N1)
1N1)
1N1)
1N1)

1N1)

1N1)

1N1)

1N1)

1N1)

1N1)

1N1)

H1N1)

1N1)

1N1)

1N1)

H1N1)

H1N1)

1N1)

)

H1N1)

H1N1)

H1N1)

H1N1)

H1N1)

2010

2010

2010

2010

2010

H1N1)

Name	Sequence A	Subtype	Collection Da	Country	Strain Name
NA	FJ969517	H1N1	04/01/2009	USA	A/California/04/2009(H1N1)
NA	FJ966084	H1N1	04/01/2009	USA	A/California/04/2009(H1N1)
NA	CY080612	H1N1	04/01/2009	USA	A/Boston/95/2009
NA	CY100846	H1N1	04/01/2009	USA	A/Alabama/WRAIR1235P/2009(H1N1)
NA	GQ162193	H1N1	04/02/2009	Mexico	A/Mexico/3955/2009(H1N1)
NA	GQ149666	H1N1	04/03/2009	Mexico	A/Mexico/4108/2009(H1N1)
NA	GQ149650	H1N1	04/03/2009	Mexico	A/Mexico/4108/2009(H1N1)
NA	GQ149656	H1N1	04/03/2009	Mexico	A/Mexico/4108/2009(H1N1)
NA	GQ149659	H1N1	04/03/2009	Mexico	A/Mexico/4108/2009(H1N1)
NA	GQ149688	H1N1	04/03/2009	Mexico	A/Mexico/4108/2009(H1N1)
NA	GQ162169	H1N1	04/03/2009	Mexico	A/Mexico/4108/2009(H1N1)
NA	GQ223113	H1N1	04/03/2009	Mexico	A/Mexico/4108/2009(H1N1)
NA	CY077597	H1N1	04/03/2009	Mexico	A/Mexico/LaGloria-3/2009(H1N1)
NA	GQ149691	H1N1	04/07/2009	Mexico	A/Mexico/4115/2009(H1N1)
NA	GQ476024	H1N1	04/08/2009	USA	A/Michigan/07/2009(H1N1)
NA	KU933487	H1N1	04/09/2009	USA	A/California/07/2009
NA	KC781784	H1N1	04/09/2009	USA	A/California/07/2009
NA	KF009555	H1N1	04/09/2009	USA	A/California/07/2009
NA	CY266193	H1N1	04/09/2009	USA	A/California/07/2009
NA	GQ377079	H1N1	04/09/2009	USA	A/California/08/2009(H1N1)
NA	GQ377078	H1N1	04/09/2009	USA	A/California/07/2009(H1N1)
NA	CY181395	H1N1	04/09/2009	USA	A/California/07-JR002/2009
NA	CY181403	H1N1	04/09/2009	USA	A/California/07-JR003/2009
NA	CY181427	H1N1	04/09/2009	USA	A/California/07-JR010/2009
NA	CY181411	H1N1	04/09/2009	USA	A/California/07-JR008/2009
NA	CY181419	H1N1	04/09/2009	USA	A/California/07-JR009/2009
NA	FJ984386	H1N1	04/09/2009	USA	A/California/07/2009
NA	GQ476149	H1N1	04/09/2009	USA	A/New York/64/2009(H1N1)
NA	HM138502	H1N1	04/09/2009	USA	A/California/07/2009(H1N1)
NA	GQ377050	H1N1	04/09/2009	USA	A/California/08/2009(H1N1)
NA	KF527483	H1N1	04/09/2009	USA	A/California/07/2009(H1N1)
NA	NC_026434	H1N1	04/09/2009	USA	A/California/07/2009
NA	KC781678	H1N1	04/10/2009	USA	A/Michigan/30/2009
NA	GQ476079	H1N1	04/13/2009	USA	A/Hawaii/17/2009(H1N1)
NA	GQ162181	H1N1	04/13/2009	Mexico	A/Mexico/4176/2009(H1N1)
NA	GQ377071	H1N1	04/14/2009	USA	A/Texas/04/2009(H1N1)
NA	GQ149672	H1N1	04/14/2009	Mexico	A/Mexico/4482/2009(H1N1)
NA	CY044237	H1N1	04/14/2009	USA	A/San Antonio/PR921/2009(H1N1)
NA	CY147773	H1N1	04/14/2009	Mexico	A/Mexico/24033/2009
NA	GQ149644	H1N1	04/14/2009	Mexico	A/Mexico/4486/2009(H1N1)
NA	GQ149670	H1N1	04/14/2009	Mexico	A/Mexico/4482/2009(H1N1)
NA	FJ966981	H1N1	04/14/2009	USA	A/Texas/04/2009(H1N1)
NA	GQ379820	H1N1	04/14/2009	Mexico	A/Mexico/4482/2009(H1N1)
NA	GQ162201	H1N1	04/14/2009	Mexico	A/Mexico/4486/2009(H1N1)
NA	GQ149664	H1N1	04/14/2009	Mexico	A/Mexico/4486/2009(H1N1)
NA	GQ149622	H1N1	04/14/2009	Mexico	A/Mexico/4486/2009(H1N1)
NA	GQ162189	H1N1	04/14/2009	Mexico	A/Mexico/4482/2009(H1N1)
NA	GQ162187	H1N1	04/14/2009	Mexico	A/Mexico/4486/2009(H1N1)
NA	FJ981614	H1N1	04/14/2009	USA	A/Texas/04/2009(H1N1)
NA	FJ998214	H1N1	04/14/2009	Mexico	A/Mexico/InDRE4487/2009(H1N1)
NA	GQ221799	H1N1	04/14/2009	USA	A/Texas/04/2009(H1N1)
NA	GQ323512	H1N1	04/15/2009	USA	A/Texas/05/2009(H1N1)
NA	CY044245	H1N1	04/15/2009	USA	A/San Antonio/PR922/2009(H1N1)

NA	GQ457486	H1N1	04/15/2009	USA	A/Texas/05/2009(H1N1)
NA	FJ966973	H1N1	04/15/2009	USA	A/California/09/2009(H1N1)
NA	FJ966969	H1N1	04/15/2009	USA	A/Texas/05/2009(H1N1)
NA	GQ162171	H1N1	04/15/2009	Mexico	A/Mexico/4269/2009(H1N1)
NA	GQ122096	H1N1	04/15/2009	USA	A/Texas/15/2009(H1N1)
NA	GQ402234	H1N1	04/16/2009	Canada	A/Canada-QC/RV1595/2009(H1N1)
NA	KC782252	H1N1	04/16/2009	USA	A/Hawaii/13/2009
NA	FJ971075	H1N1	04/16/2009	USA	A/California/06/2009(H1N1)
NA	KC781721	H1N1	04/16/2009	USA	A/California/06/2009
NA	GQ476035	H1N1	04/16/2009	USA	A/Hawaii/13/2009(H1N1)
NA	CY147765	H1N1	04/17/2009	Mexico	A/Mexico/24032/2009
NA	CY147821	H1N1	04/17/2009	Mexico	A/Mexico/24043/2009
NA	GQ149631	H1N1	04/19/2009	Mexico	A/Mexico/4604/2009(H1N1)
NA	GQ149640	H1N1	04/19/2009	Mexico	A/Mexico/4603/2009(H1N1)
NA	GQ149681	H1N1	04/19/2009	Mexico	A/Mexico/4603/2009(H1N1)
NA	GQ894939	H1N1	04/20/2009	Mexico	A/Mexico/4627/2009(H1N1)
NA	GQ397120	H1N1	04/20/2009	Mexico	A/Mexico/4646/2009(H1N1)
NA	GQ162173	H1N1	04/20/2009	Mexico	A/Mexico/4593/2009(H1N1)
NA	GQ162196	H1N1	04/20/2009	Mexico	A/Mexico/4575/2009(H1N1)
NA	CY147837	H1N1	04/20/2009	Mexico	A/Mexico/24045/2009
NA	CY147829	H1N1	04/20/2009	Mexico	A/Mexico/24044/2009
NA	GQ397116	H1N1	04/20/2009	Mexico	A/Mexico/4595/2009(H1N1)
NA	GQ397117	H1N1	04/20/2009	Mexico	A/Mexico/4635/2009(H1N1)
NA	GQ323523	H1N1	04/21/2009	USA	A/California/13/2009(H1N1)
NA	CY043096	H1N1	04/21/2009	USA	A/Brawley/40082/2009(H1N1)
NA	GQ377092	H1N1	04/22/2009	USA	A/California/12/2009(H1N1)
NA	CY043088	H1N1	04/22/2009	USA	A/Brawley/40081/2009(H1N1)
NA	GQ117094	H1N1	04/22/2009	USA	A/Indiana/09/2009(H1N1)
NA	GQ117064	H1N1	04/22/2009	USA	A/Arizona/01/2009(H1N1)
NA	CY147613	H1N1	04/23/2009	Mexico	A/Mexico/24007/2009
NA	FJ984383	H1N1	04/23/2009	USA	A/Texas/06/2009(H1N1)
NA	CY044253	H1N1	04/23/2009	USA	A/San Antonio/PR923/2009(H1N1)
NA	GQ323513	H1N1	04/23/2009	USA	A/Texas/10/2009(H1N1)
NA	CY147701	H1N1	04/23/2009	Mexico	A/Mexico/24021/2009
NA	GQ323573	H1N1	04/23/2009	USA	A/Texas/11/2009(H1N1)
NA	FJ969520	H1N1	04/24/2009	USA	A/Ohio/07/2009(H1N1)
NA	FJ969534	H1N1	04/24/2009	USA	A/Ohio/07/2009(H1N1)
NA	CY041551	H1N1	04/24/2009	USA	A/New York/3210/2009(H1N1)
NA	FJ984357	H1N1	04/24/2009	USA	A/New York/31/2009(H1N1)
NA	FJ984362	H1N1	04/24/2009	USA	A/New York/23/2009(H1N1)
NA	GQ232025	H1N1	04/24/2009	USA	A/Illinois/01/2009(H1N1)
NA	GQ396745	H1N1	04/24/2009	USA	A/New York/25/2009(H1N1)
NA	GQ323567	H1N1	04/24/2009	USA	A/Ohio/07/2009(H1N1)
NA	GQ168669	H1N1	04/24/2009	USA	A/New York/24/2009(H1N1)
NA	GQ168660	H1N1	04/24/2009	USA	A/New York/31/2009(H1N1)
NA	GQ168632	H1N1	04/24/2009	USA	A/Texas/08/2009(H1N1)
NA	GQ117048	H1N1	04/24/2009	USA	A/Texas/08/2009(H1N1)
NA	GQ168627	H1N1	04/24/2009	USA	A/New York/23/2009(H1N1)
NA	GQ117022	H1N1	04/24/2009	USA	A/New York/22/2009(H1N1)
NA	GQ168643	H1N1	04/24/2009	USA	A/Kansas/03/2009(H1N1)
NA	GQ200251	H1N1	04/24/2009	USA	A/New York/21/2009(H1N1)
NA	GQ221819	H1N1	04/24/2009	USA	A/California/11/2009(H1N1)
NA	GQ221815	H1N1	04/24/2009	USA	A/New York/16/2009(H1N1)
NA	GQ117058	H1N1	04/24/2009	USA	A/Kansas/02/2009(H1N1)

NA	CY065152	H1N1	04/24/2009	United Ki	A/Scotland/91800799/2009
NA	FJ998215	H1N1	04/24/2009	Canada	A/Canada-ON/RV1527/2009(H1N1)
NA	FJ998213	H1N1	04/24/2009	Canada	A/Canada-NS/RV1535/2009(H1N1)
NA	GQ396740	H1N1	04/24/2009	USA	A/Iowa/04/2009(H1N1)
NA	GQ373263	H1N1	04/24/2009	Canada	A/Toronto/3141/2009(H1N1)
NA	GQ894876	H1N1	04/24/2009	USA	A/New York/24/2009(H1N1)
NA	GQ323526	H1N1	04/24/2009	USA	A/Texas/17/2009(H1N1)
NA	GQ323479	H1N1	04/24/2009	USA	A/Ohio/07/2009(H1N1)
NA	GQ323478	H1N1	04/24/2009	USA	A/Kansas/03/2009(H1N1)
NA	GQ117099	H1N1	04/24/2009	USA	A/Ohio/07/2009(H1N1)
NA	GQ117081	H1N1	04/25/2009	USA	A/New York/20/2009(H1N1)
NA	GQ117084	H1N1	04/25/2009	USA	A/New York/20/2009(H1N1)
NA	FJ984371	H1N1	04/25/2009	USA	A/New York/10/2009(H1N1)
NA	GQ117102	H1N1	04/25/2009	USA	A/Massachusetts/07/2009(H1N1)
NA	FJ984390	H1N1	04/25/2009	USA	A/New York/19/2009(H1N1)
NA	GQ117114	H1N1	04/25/2009	USA	A/New York/13/2009(H1N1)
NA	GQ323481	H1N1	04/25/2009	USA	A/New York/34/2009(H1N1)
NA	GQ323482	H1N1	04/25/2009	USA	A/New York/37/2009(H1N1)
NA	GQ323525	H1N1	04/25/2009	USA	A/New York/39/2009(H1N1)
NA	GQ323542	H1N1	04/25/2009	USA	A/Texas/07/2009(H1N1)
NA	CY065144	H1N1	04/25/2009	United Ki	A/Scotland/91800797/2009
NA	CY041132	H1N1	04/25/2009	USA	A/New York/3215/2009(H1N1)
NA	GQ377102	H1N1	04/25/2009	USA	A/New York/45/2009(H1N1)
NA	CY086979	H1N1	04/25/2009	USA	A/New York/3217/2009(H1N1)
NA	CY074389	H1N1	04/25/2009	USA	A/California/VRDL264/2009(H1N1)
NA	CY064873	H1N1	04/25/2009	USA	A/California/VRDL146/2009(H1N1)
NA	CY097878	H1N1	04/25/2009	Mexico	A/Mexico/47N/2009
NA	GQ221787	H1N1	04/25/2009	USA	A/New York/19/2009(H1N1)
NA	GQ117028	H1N1	04/25/2009	USA	A/Texas/09/2009(H1N1)
NA	GQ117036	H1N1	04/25/2009	USA	A/California/14/2009(H1N1)
NA	GQ168648	H1N1	04/25/2009	USA	A/New York/08/2009(H1N1)
NA	GQ168651	H1N1	04/25/2009	USA	A/New York/11/2009(H1N1)
NA	GQ168654	H1N1	04/25/2009	USA	A/New York/09/2009(H1N1)
NA	GQ168666	H1N1	04/25/2009	USA	A/New York/13/2009(H1N1)
NA	GQ168670	H1N1	04/25/2009	USA	A/Texas/09/2009(H1N1)
NA	GQ168672	H1N1	04/25/2009	USA	A/New York/12/2009(H1N1)
NA	FJ969541	H1N1	04/25/2009	USA	A/New York/20/2009(H1N1)
NA	FJ973552	H1N1	04/25/2009	New Zeala	A/Auckland/3/2009(H1N1)
NA	FJ973555	H1N1	04/25/2009	New Zeala	A/Auckland/1/2009(H1N1)
NA	GQ205439	H1N1	04/25/2009	New Zeala	A/Auckland/3/2009(H1N1)
NA	GQ200277	H1N1	04/25/2009	Colombia	A/Colombia/08/2009(H1N1)
NA	GQ205435	H1N1	04/25/2009	New Zeala	A/Auckland/1/2009(H1N1)
NA	GQ205437	H1N1	04/25/2009	New Zeala	A/Auckland/3/2009(H1N1)
NA	GQ160612	H1N1	04/25/2009	New Zeala	A/Auckland/1/2009(H1N1)
NA	CY041543	H1N1	04/25/2009	USA	A/New York/3209/2009(H1N1)
NA	CY040890	H1N1	04/25/2009	Mexico	A/Mexico/47N/2009(H1N1)
NA	FJ984350	H1N1	04/25/2009	USA	A/New York/18/2009(H1N1)
NA	FJ984340	H1N1	04/25/2009	USA	A/New York/06/2009(H1N1)
NA	CY040639	H1N1	04/25/2009	USA	A/New York/3191/2009(H1N1)
NA	FJ984335	H1N1	04/25/2009	USA	A/New York/12/2009(H1N1)
NA	FJ984344	H1N1	04/25/2009	USA	A/New York/11/2009(H1N1)
NA	CY041124	H1N1	04/25/2009	USA	A/New York/3214/2009(H1N1)
NA	CY041615	H1N1	04/25/2009	USA	A/New York/3002/2009(H1N1)
NA	GQ323555	H1N1	04/25/2009	USA	A/California/17/2009(H1N1)

NA	GQ323460	H1N1	04/25/2009	USA	A/New York/35/2009(H1N1)
NA	GQ323565	H1N1	04/25/2009	USA	A/New York/09/2009(H1N1)
NA	CY147677	H1N1	04/25/2009	Mexico	A/Mexico/24015/2009
NA	FJ984378	H1N1	04/25/2009	USA	A/New York/15/2009(H1N1)
NA	CY044165	H1N1	04/25/2009	Mexico	A/Mexico/48N/2009(H1N1)
NA	GQ377060	H1N1	04/25/2009	USA	A/Texas/20/2009(H1N1)
NA	GQ457483	H1N1	04/25/2009	USA	A/New York/15/2009(H1N1)
NA	GQ338348	H1N1	04/25/2009	USA	A/New York/33/2009(H1N1)
NA	GQ160571	H1N1	04/26/2009	USA	A/Texas/22/2009(H1N1)
NA	GQ457481	H1N1	04/26/2009	USA	A/Nevada/03/2009(H1N1)
NA	CY041736	H1N1	04/26/2009	USA	A/New York/3206/2009(H1N1)
NA	GQ338411	H1N1	04/26/2009	USA	A/Texas/37/2009(H1N1)
NA	CY092095	H1N1	04/26/2009	USA	A/California/VRDL372/2009
NA	GQ465700	H1N1	04/26/2009	Canada	A/Canada-PQ/RV1586/2009(H1N1)
NA	CY074613	H1N1	04/26/2009	USA	A/California/VRDL366/2009(H1N1)
NA	CY074629	H1N1	04/26/2009	USA	A/California/VRDL371/2009(H1N1)
NA	CY074645	H1N1	04/26/2009	USA	A/California/VRDL378/2009(H1N1)
NA	CY039895	H1N1	04/26/2009	USA	A/New York/1669/2009(H1N1)
NA	GQ160536	H1N1	04/26/2009	USA	A/New Mexico/04/2009(H1N1)
NA	GQ221789	H1N1	04/26/2009	USA	A/Arizona/02/2009(H1N1)
NA	GQ221795	H1N1	04/26/2009	USA	A/South Carolina/09/2009(H1N1)
NA	GQ221806	H1N1	04/26/2009	USA	A/Arizona/03/2009(H1N1)
NA	GQ221821	H1N1	04/26/2009	USA	A/Colorado/04/2009(H1N1)
NA	GQ117042	H1N1	04/26/2009	USA	A/Massachusetts/06/2009(H1N1)
NA	GQ117053	H1N1	04/26/2009	USA	A/South Carolina/09/2009(H1N1)
NA	HQ239839	H1N1	04/26/2009	Canada	A/Canada-ON/RV1590/2009
NA	FJ985754	H1N1	04/26/2009	Spain	A/Castilla-La Mancha/GP13/2009(H1N1
NA	GQ168888	H1N1	04/26/2009	Spain	A/Catalonia/3/2009(H1N1)
NA	GQ168889	H1N1	04/26/2009	Spain	A/Catalonia/4/2009(H1N1)
NA	GQ168890	H1N1	04/26/2009	Spain	A/Catalonia/14/2009(H1N1)
NA	GQ183608	H1N1	04/26/2009	Spain	A/Catalonia/8/2009(H1N1)
NA	GQ183611	H1N1	04/26/2009	Spain	A/Catalonia/10/2009(H1N1)
NA	FJ985759	H1N1	04/26/2009	Spain	A/Valencia/GP4/2009(H1N1)
NA	FJ985764	H1N1	04/26/2009	Spain	A/Pais Vasco/GP20/2009(H1N1)
NA	FJ985769	H1N1	04/26/2009	Spain	A/Castilla-La Mancha/GP9/2009(H1N1)
NA	CY041607	H1N1	04/26/2009	USA	A/New York/3207/2009(H1N1)
NA	CY041060	H1N1	04/26/2009	USA	A/New York/3168/2009(H1N1)
NA	CY041068	H1N1	04/26/2009	USA	A/New York/3169/2009(H1N1)
NA	CY041100	H1N1	04/26/2009	USA	A/New York/3176/2009(H1N1)
NA	CY041532	H1N1	04/26/2009	USA	A/New York/3205/2009(H1N1)
NA	GQ231980	H1N1	04/26/2009	USA	A/South Carolina/10/2009(H1N1)
NA	CY041172	H1N1	04/26/2009	USA	A/New York/3186/2009(H1N1)
NA	GQ117077	H1N1	04/26/2009	USA	A/Arizona/02/2009(H1N1)
NA	GQ377057	H1N1	04/26/2009	USA	A/Arizona/05/2009(H1N1)
NA	CY041052	H1N1	04/26/2009	USA	A/New York/3166/2009(H1N1)
NA	GQ117108	H1N1	04/26/2009	USA	A/Michigan/02/2009(H1N1)
NA	GQ183607	H1N1	04/27/2009	Spain	A/Catalonia/33/2009(H1N1)
NA	CY050614	H1N1	04/27/2009	USA	A/New York/3052/2009(H1N1)
NA	HQ239830	H1N1	04/27/2009	Canada	A/Canada-ON/RV1545/2009
NA	HQ239837	H1N1	04/27/2009	Canada	A/Canada-NS/RV1572/2009
NA	HQ239843	H1N1	04/27/2009	Canada	A/Canada-BC/RV1719/2009
NA	HQ239844	H1N1	04/27/2009	Canada	A/Canada-BC/RV1720/2009
NA	HQ239842	H1N1	04/27/2009	Canada	A/Canada-BC/RV1718/2009
NA	HQ239838	H1N1	04/27/2009	Canada	A/Canada-NS/RV1582/2009

NA	GQ200274	H1N1	04/27/2009	Colombia	A/Bogota/176/2009(H1N1)
NA	GQ168891	H1N1	04/27/2009	Spain	A/Catalonia/25/2009(H1N1)
NA	GQ168892	H1N1	04/27/2009	Spain	A/Catalonia/43/2009(H1N1)
NA	GQ168893	H1N1	04/27/2009	Spain	A/Catalonia/44/2009(H1N1)
NA	GQ168894	H1N1	04/27/2009	Spain	A/Catalonia/49/2009(H1N1)
NA	GQ168895	H1N1	04/27/2009	Spain	A/Catalonia/50/2009(H1N1)
NA	FJ984953	H1N1	04/27/2009	Germany	A/Regensburg/Germany/01/2009
NA	GQ396514	H1N1	04/27/2009	Spain	A/Murcia/GP78/2009(H1N1)
NA	CY041567	H1N1	04/27/2009	USA	A/New York/3225/2009(H1N1)
NA	CY041084	H1N1	04/27/2009	USA	A/New York/3172/2009(H1N1)
NA	CY040840	H1N1	04/27/2009	USA	A/New York/3262/2009(H1N1)
NA	CY041524	H1N1	04/27/2009	USA	A/New York/3204/2009(H1N1)
NA	CY041516	H1N1	04/27/2009	USA	A/New York/3203/2009(H1N1)
NA	CY065192	H1N1	04/27/2009	United Ki	A/England/202/2009
NA	GQ338413	H1N1	04/27/2009	USA	A/Tennessee/06/2009(H1N1)
NA	GQ323566	H1N1	04/27/2009	USA	A/Nebraska/03/2009(H1N1)
NA	GQ476064	H1N1	04/27/2009	USA	A/California/26/2009(H1N1)
NA	CY147709	H1N1	04/27/2009	Mexico	A/Mexico/24022/2009
NA	CY147717	H1N1	04/27/2009	Mexico	A/Mexico/24024/2009
NA	CY147885	H1N1	04/27/2009	Mexico	A/Mexico/24052/2009
NA	CY147781	H1N1	04/27/2009	Mexico	A/Mexico/24036/2009
NA	CY147869	H1N1	04/27/2009	Mexico	A/Mexico/24050/2009
NA	CY147877	H1N1	04/27/2009	Mexico	A/Mexico/24051/2009
NA	CY147893	H1N1	04/27/2009	Mexico	A/Mexico/24053/2009
NA	CY147901	H1N1	04/27/2009	Mexico	A/Mexico/24054/2009
NA	CY147845	H1N1	04/27/2009	Mexico	A/Mexico/24047/2009
NA	CY147861	H1N1	04/27/2009	Mexico	A/Mexico/24049/2009
NA	CY147853	H1N1	04/27/2009	Mexico	A/Mexico/24048/2009
NA	GQ221810	H1N1	04/27/2009	USA	A/Arizona/04/2009(H1N1)
NA	GQ338393	H1N1	04/27/2009	USA	A/Washington/08/2009(H1N1)
NA	GQ475937	H1N1	04/27/2009	USA	A/California/26/2009(H1N1)
NA	CY074669	H1N1	04/27/2009	USA	A/California/VRDL382/2009(H1N1)
NA	CY074637	H1N1	04/27/2009	USA	A/California/VRDL377/2009(H1N1)
NA	GQ323480	H1N1	04/27/2009	USA	A/Alabama/02/2009(H1N1)
NA	GQ338415	H1N1	04/27/2009	USA	A/Wisconsin/07/2009(H1N1)
NA	CY040824	H1N1	04/27/2009	USA	A/New York/3260/2009(H1N1)
NA	GQ338370	H1N1	04/27/2009	USA	A/Ohio/08/2009(H1N1)
NA	GQ323545	H1N1	04/27/2009	USA	A/District of Columbia/01/2009(H1N1
NA	GQ323441	H1N1	04/27/2009	USA	A/Missouri/01/2009(H1N1)
NA	CY074125	H1N1	04/27/2009	USA	A/California/VRDL194/2009(H1N1)
NA	CY070905	H1N1	04/27/2009	USA	A/California/VRDL150/2009(H1N1)
NA	CY070897	H1N1	04/27/2009	USA	A/California/VRDL149/2009(H1N1)
NA	GQ465701	H1N1	04/27/2009	Canada	A/Canada-ON/RV1589/2009(H1N1)
NA	GQ377059	H1N1	04/27/2009	USA	A/Illinois/03/2009(H1N1)
NA	GQ402231	H1N1	04/27/2009	Mexico	A/Mexico/InDRE13547/2009(H1N1)
NA	CY065224	H1N1	04/27/2009	United Ki	A/England/207/2009
NA	CY065216	H1N1	04/27/2009	United Ki	A/England/205/2009
NA	CY065248	H1N1	04/27/2009	United Ki	A/England/XFL00071/2009
NA	CY074397	H1N1	04/27/2009	USA	A/California/VRDL267/2009(H1N1)
NA	CY074605	H1N1	04/27/2009	USA	A/California/VRDL365/2009(H1N1)
NA	CY074653	H1N1	04/27/2009	USA	A/California/VRDL379/2009(H1N1)
NA	CY074661	H1N1	04/27/2009	USA	A/California/VRDL380/2009(H1N1)
NA	CY073831	H1N1	04/27/2009	USA	A/California/VRDL155/2009(H1N1)
NA	CY073807	H1N1	04/27/2009	USA	A/California/VRDL152/2009(H1N1)

NA	CY073815	H1N1	04/27/2009	USA	A/California/VRDL153/2009(H1N1)
NA	CY074621	H1N1	04/27/2009	USA	A/California/VRDL369/2009(H1N1)
NA	CY040009	H1N1	04/27/2009	USA	A/New York/3012/2009(H1N1)
NA	CY040017	H1N1	04/27/2009	USA	A/New York/3014/2009(H1N1)
NA	CY040025	H1N1	04/27/2009	USA	A/New York/3049/2009(H1N1)
NA	CY040044	H1N1	04/27/2009	USA	A/New York/3007/2009(H1N1)
NA	CY039903	H1N1	04/27/2009	USA	A/New York/1682/2009(H1N1)
NA	GQ160524	H1N1	04/27/2009	USA	A/Florida/04/2009(H1N1)
NA	GQ160589	H1N1	04/27/2009	USA	A/Virginia/04/2009(H1N1)
NA	CY040001	H1N1	04/27/2009	USA	A/New York/3008/2009(H1N1)
NA	CY064801	H1N1	04/27/2009	USA	A/California/VRDL134/2009(H1N1)
NA	CY064809	H1N1	04/27/2009	USA	A/California/VRDL135/2009(H1N1)
NA	CY064833	H1N1	04/27/2009	USA	A/California/VRDL140/2009(H1N1)
NA	CY050662	H1N1	04/27/2009	USA	A/New York/3150/2009
NA	CY069775	H1N1	04/27/2009	United Ki	A/England/209/2009
NA	CY069767	H1N1	04/27/2009	United Ki	A/England/196/2009
NA	CY065240	H1N1	04/27/2009	United Ki	A/England/551/2009
NA	CY080620	H1N1	04/27/2009	USA	A/Boston/100/2009
NA	CY087145	H1N1	04/27/2009	USA	A/California/VRDL137/2009
NA	CY087153	H1N1	04/27/2009	USA	A/California/VRDL148/2009
NA	CY065200	H1N1	04/27/2009	United Ki	A/England/197/2009
NA	CY050478	H1N1	04/27/2009	USA	A/New York/1692/2009(H1N1)
NA	GQ200238	H1N1	04/27/2009	USA	A/Georgia/01/2009(H1N1)
NA	GQ221813	H1N1	04/27/2009	USA	A/Colorado/03/2009(H1N1)
NA	GQ117071	H1N1	04/27/2009	USA	A/Minnesota/02/2009(H1N1)
NA	GQ160547	H1N1	04/27/2009	USA	A/Texas/23/2009(H1N1)
NA	GQ117118	H1N1	04/27/2009	USA	A/Colorado/03/2009(H1N1)
NA	CY041148	H1N1	04/28/2009	USA	A/New York/3181/2009(H1N1)
NA	CY041156	H1N1	04/28/2009	USA	A/New York/3183/2009(H1N1)
NA	GQ338333	H1N1	04/28/2009	USA	A/Louisiana/01/2009(H1N1)
NA	GQ323554	H1N1	04/28/2009	USA	A/Arizona/06/2009(H1N1)
NA	CY147733	H1N1	04/28/2009	Mexico	A/Mexico/24026/2009
NA	CY147741	H1N1	04/28/2009	Mexico	A/Mexico/24027/2009
NA	CY147749	H1N1	04/28/2009	Mexico	A/Mexico/24028/2009
NA	CY147917	H1N1	04/28/2009	Mexico	A/Mexico/24056/2009
NA	CY147925	H1N1	04/28/2009	Mexico	A/Mexico/24057/2009
NA	CY147933	H1N1	04/28/2009	Mexico	A/Mexico/24058/2009
NA	CY147941	H1N1	04/28/2009	Mexico	A/Mexico/24060/2009
NA	CY074109	H1N1	04/28/2009	USA	A/California/VRDL192/2009(H1N1)
NA	GQ160529	H1N1	04/28/2009	USA	A/Tennessee/05/2009(H1N1)
NA	GQ160540	H1N1	04/28/2009	USA	A/Delaware/03/2009(H1N1)
NA	GQ160581	H1N1	04/28/2009	USA	A/New Hampshire/02/2009(H1N1)
NA	GQ160585	H1N1	04/28/2009	USA	A/North Carolina/05/2009(H1N1)
NA	CY065208	H1N1	04/28/2009	United Ki	A/England/204/2009
NA	CY053073	H1N1	04/28/2009	USA	A/Houston/130S/2009(H1N1)
NA	CY092347	H1N1	04/28/2009	USA	A/California/VRDL191/2009
NA	CY065256	H1N1	04/28/2009	United Ki	A/England/211/2009
NA	CY050069	H1N1	04/28/2009	USA	A/Cherry Point/WR1355/2009(H1N1)
NA	CY040719	H1N1	04/28/2009	USA	A/New York/3238/2009(H1N1)
NA	GQ221824	H1N1	04/28/2009	USA	A/Delaware/05/2009(H1N1)
NA	GQ221827	H1N1	04/28/2009	USA	A/Idaho/02/2009(H1N1)
NA	CY040631	H1N1	04/28/2009	USA	A/New York/3234/2009(H1N1)
NA	CY147909	H1N1	04/28/2009	Mexico	A/Mexico/24055/2009
NA	CY147789	H1N1	04/28/2009	Mexico	A/Mexico/24038/2009

NA	CY046333	H1N1	04/28/2009	USA	A/Wisconsin/629-D01505/2009(H1N1)
NA	CY041559	H1N1	04/28/2009	USA	A/New York/3211/2009(H1N1)
NA	GQ402232	H1N1	04/28/2009	Mexico	A/Mexico/InDRE13551/2009(H1N1)
NA	GQ166659	H1N1	04/28/2009	United Ki	A/England/195/2009
NA	CY147949	H1N1	04/28/2009	Mexico	A/Mexico/24061/2009
NA	CY041591	H1N1	04/28/2009	USA	A/New York/3236/2009(H1N1)
NA	GQ323568	H1N1	04/28/2009	USA	A/Delaware/08/2009(H1N1)
NA	GQ338357	H1N1	04/28/2009	USA	A/Delaware/07/2009(H1N1)
NA	GQ338371	H1N1	04/28/2009	USA	A/Washington/10/2009(H1N1)
NA	GQ323543	H1N1	04/28/2009	USA	A/Washington/09/2009(H1N1)
NA	GQ338396	H1N1	04/28/2009	USA	A/Delaware/06/2009(H1N1)
NA	GQ338401	H1N1	04/28/2009	USA	A/Pennsylvania/10/2009(H1N1)
NA	CY073396	H1N1	04/28/2009	USA	A/Brawley/WR1635P/2009(H1N1)
NA	CY074085	H1N1	04/28/2009	USA	A/California/VRDL180/2009(H1N1)
NA	CY064841	H1N1	04/28/2009	USA	A/California/VRDL141/2009(H1N1)
NA	CY070913	H1N1	04/28/2009	USA	A/California/VRDL151/2009(H1N1)
NA	CY047320	H1N1	04/28/2009	USA	A/New York/3124/2009(H1N1)
NA	CY047328	H1N1	04/28/2009	USA	A/New York/3167/2009(H1N1)
NA	CY083306	H1N1	04/28/2009	USA	A/San Diego/WRAIR1648P/2009
NA	CY065232	H1N1	04/28/2009	United Ki	A/England/200/2009
NA	CY074149	H1N1	04/28/2009	USA	A/California/VRDL206/2009(H1N1)
NA	CY074173	H1N1	04/28/2009	USA	A/California/VRDL215/2009(H1N1)
NA	CY073823	H1N1	04/28/2009	USA	A/California/VRDL154/2009(H1N1)
NA	CY073847	H1N1	04/28/2009	USA	A/California/VRDL167/2009(H1N1)
NA	CY073855	H1N1	04/28/2009	USA	A/California/VRDL175/2009(H1N1)
NA	GQ396575	H1N1	04/28/2009	Spain	A/Valencia/GP154/2009(H1N1)
NA	HQ239840	H1N1	04/28/2009	Canada	A/Canada-PQ/RV1602/2009
NA	GQ168897	H1N1	04/28/2009	Spain	A/Catalonia/63/2009(H1N1)
NA	GQ168896	H1N1	04/28/2009	Spain	A/Catalonia/61/2009(H1N1)
NA	GQ396571	H1N1	04/28/2009	Spain	A/Valencia/GP153/2009(H1N1)
NA	GQ396536	H1N1	04/28/2009	Spain	A/Andalucia/GP151/2009(H1N1)
NA	GQ396538	H1N1	04/28/2009	Spain	A/Madrid/GP193/2009(H1N1)
NA	GQ465697	H1N1	04/28/2009	Canada	A/Canada-AB/RV1531/2009(H1N1)
NA	GQ160593	H1N1	04/28/2009	USA	A/Delaware/02/2009(H1N1)
NA	CY041575	H1N1	04/28/2009	USA	A/New York/3227/2009(H1N1)
NA	CY041583	H1N1	04/28/2009	USA	A/New York/3230/2009(H1N1)
NA	CY041832	H1N1	04/28/2009	USA	A/New York/3348/2009(H1N1)
NA	CY041092	H1N1	04/28/2009	USA	A/New York/3173/2009(H1N1)
NA	CY041108	H1N1	04/28/2009	USA	A/New York/3178/2009(H1N1)
NA	CY041116	H1N1	04/28/2009	USA	A/New York/3193/2009(H1N1)
NA	CY040703	H1N1	04/28/2009	USA	A/New York/3235/2009(H1N1)
NA	CY040711	H1N1	04/28/2009	USA	A/New York/3237/2009(H1N1)
NA	GQ232041	H1N1	04/28/2009	USA	A/Rhode Island/02/2009(H1N1)
NA	CY041492	H1N1	04/28/2009	USA	A/New York/3184/2009(H1N1)
NA	CY041188	H1N1	04/28/2009	USA	A/New York/3188/2009(H1N1)
NA	CY041500	H1N1	04/28/2009	USA	A/New York/3195/2009(H1N1)
NA	CY041204	H1N1	04/28/2009	USA	A/New York/3199/2009(H1N1)
NA	CY041508	H1N1	04/28/2009	USA	A/New York/3196/2009(H1N1)
NA	CY074317	H1N1	04/29/2009	USA	A/California/VRDL239/2009(H1N1)
NA	CY074349	H1N1	04/29/2009	USA	A/California/VRDL251/2009(H1N1)
NA	CY074117	H1N1	04/29/2009	USA	A/California/VRDL193/2009(H1N1)
NA	CY074157	H1N1	04/29/2009	USA	A/California/VRDL209/2009(H1N1)
NA	CY074205	H1N1	04/29/2009	USA	A/California/VRDL220/2009(H1N1)
NA	CY074213	H1N1	04/29/2009	USA	A/California/VRDL221/2009(H1N1)

NA	CY074229	H1N1	04/29/2009	USA	A/California/VRDL223/2009(H1N1)
NA	CY074253	H1N1	04/29/2009	USA	A/California/VRDL226/2009(H1N1)
NA	CY074285	H1N1	04/29/2009	USA	A/California/VRDL235/2009(H1N1)
NA	CY074293	H1N1	04/29/2009	USA	A/California/VRDL236/2009(H1N1)
NA	CY074221	H1N1	04/29/2009	USA	A/California/VRDL222/2009(H1N1)
NA	CY074357	H1N1	04/29/2009	USA	A/California/VRDL252/2009(H1N1)
NA	CY040033	H1N1	04/29/2009	USA	A/New York/3099/2009(H1N1)
NA	CY039528	H1N1	04/29/2009	Netherlar	A/Netherlands/602/2009
NA	CY092339	H1N1	04/29/2009	USA	A/California/VRDL190/2009
NA	CY065264	H1N1	04/29/2009	United Ki	A/England/XFL00137/2009
NA	CY065344	H1N1	04/29/2009	United Ki	A/England/223/2009
NA	CY050766	H1N1	04/29/2009	USA	A/New York/3315/2009(H1N1)
NA	CY050550	H1N1	04/29/2009	USA	A/New York/3095/2009(H1N1)
NA	GQ214152	H1N1	04/29/2009	France	A/Paris/2573/2009(H1N1)
NA	CY147805	H1N1	04/29/2009	Mexico	A/Mexico/24040/2009
NA	GQ338341	H1N1	04/29/2009	USA	A/Louisiana/02/2009(H1N1)
NA	CY041196	H1N1	04/29/2009	USA	A/New York/3190/2009(H1N1)
NA	GQ465699	H1N1	04/29/2009	Canada	A/Canada-NS/RV1554/2009(H1N1)
NA	GQ402233	H1N1	04/29/2009	Mexico	A/Mexico/InDRE13555/2009(H1N1)
NA	GQ465698	H1N1	04/29/2009	Canada	A/Canada-NS/RV1551/2009(H1N1)
NA	CY074365	H1N1	04/29/2009	USA	A/California/VRDL254/2009(H1N1)
NA	CY074261	H1N1	04/29/2009	USA	A/California/VRDL227/2009(H1N1)
NA	GQ323521	H1N1	04/29/2009	USA	A/Maryland/05/2009(H1N1)
NA	GQ323524	H1N1	04/29/2009	USA	A/Texas/28/2009(H1N1)
NA	GQ338374	H1N1	04/29/2009	USA	A/Nevada/04/2009(H1N1)
NA	GQ323544	H1N1	04/29/2009	USA	A/Pennsylvania/06/2009(H1N1)
NA	CY074141	H1N1	04/29/2009	USA	A/California/VRDL202/2009(H1N1)
NA	GQ368663	H1N1	04/29/2009	Brazil	A/Sao Paulo/1454/2009
NA	CY046301	H1N1	04/29/2009	USA	A/Wisconsin/629-D01486/2009(H1N1)
NA	CY046309	H1N1	04/29/2009	USA	A/Wisconsin/629-D01642/2009(H1N1)
NA	CY073404	H1N1	04/29/2009	USA	A/Brawley/WR1636P/2009(H1N1)
NA	GQ377086	H1N1	04/29/2009	USA	A/Oregon/07/2009(H1N1)
NA	CY047312	H1N1	04/29/2009	USA	A/New York/3074/2009(H1N1)
NA	GQ402230	H1N1	04/29/2009	Mexico	A/Mexico/InDRE13495/2009(H1N1)
NA	GQ402229	H1N1	04/29/2009	Mexico	A/Mexico/InDRE13494/2009(H1N1)
NA	CY074165	H1N1	04/29/2009	USA	A/California/VRDL213/2009(H1N1)
NA	CY074181	H1N1	04/29/2009	USA	A/California/VRDL217/2009(H1N1)
NA	CY074197	H1N1	04/29/2009	USA	A/California/VRDL219/2009(H1N1)
NA	CY086971	H1N1	04/29/2009	USA	A/New York/3198/2009(H1N1)
NA	CY074309	H1N1	04/29/2009	USA	A/California/VRDL238/2009(H1N1)
NA	CY074237	H1N1	04/29/2009	USA	A/California/VRDL224/2009(H1N1)
NA	CY074333	H1N1	04/29/2009	USA	A/California/VRDL245/2009(H1N1)
NA	CY074373	H1N1	04/29/2009	USA	A/California/VRDL256/2009(H1N1)
NA	CY074341	H1N1	04/29/2009	USA	A/California/VRDL249/2009(H1N1)
NA	CY074269	H1N1	04/29/2009	USA	A/California/VRDL228/2009(H1N1)
NA	CY074093	H1N1	04/29/2009	USA	A/California/VRDL186/2009(H1N1)
NA	CY074101	H1N1	04/29/2009	USA	A/California/VRDL189/2009(H1N1)
NA	CY074301	H1N1	04/29/2009	USA	A/California/VRDL237/2009(H1N1)
NA	CY073863	H1N1	04/29/2009	USA	A/California/VRDL229/2009(H1N1)
NA	CY073839	H1N1	04/29/2009	USA	A/California/VRDL163/2009
NA	CY074189	H1N1	04/29/2009	USA	A/California/VRDL218/2009(H1N1)
NA	GQ396543	H1N1	04/29/2009	Spain	A/CastillaLaMancha/GP248/2009(H1N1)
NA	KF527484	H1N1	04/29/2009	Netherlar	A/Netherlands/602/2009(H1N1)
NA	GQ396521	H1N1	04/29/2009	Spain	A/Galicia/GP224/2009(H1N1)

NA	CY041484	H1N1	04/29/2009	USA	A/New York/3189/2009(H1N1)
NA	HQ239832	H1N1	04/29/2009	Canada	A/Canada-NS/RV1552/2009
NA	HM567554	H1N1	04/29/2009	United Ki	A/England/213/2009
NA	HQ239831	H1N1	04/29/2009	Canada	A/Canada-NB/RV1546/2009
NA	GQ168898	H1N1	04/29/2009	Spain	A/Catalonia/67/2009(H1N1)
NA	GQ183609	H1N1	04/29/2009	Spain	A/Catalonia/79/2009(H1N1)
NA	GQ183610	H1N1	04/29/2009	Spain	A/Catalonia/82/2009(H1N1)
NA	GQ150333	H1N1	04/29/2009	New Zeala	A/Christchurch/2/2009(H1N1)
NA	GQ122098	H1N1	04/29/2009	New Zeala	A/Christchurch/2/2009(H1N1)
NA	FJ982431	H1N1	04/29/2009	Denmark	A/Denmark/513/2009(H1N1)
NA	CY117607	H1N1	04/29/2009	Mexico	A/Mexico/UASLP-004/2009
NA	GQ365660	H1N1	04/29/2009	Germany	A/Bayern/62/2009(H1N1)
NA	GQ396559	H1N1	04/29/2009	Spain	A/Andalucia/GP286/2009(H1N1)
NA	GQ396562	H1N1	04/29/2009	Spain	A/Valencia/GP272/2009(H1N1)
NA	GQ396591	H1N1	04/29/2009	Spain	A/Andalucia/GP251/2009(H1N1)
NA	GQ396580	H1N1	04/29/2009	Spain	A/Andalucia/GP230/2009(H1N1)
NA	GQ396566	H1N1	04/29/2009	Spain	A/Valencia/GP278/2009(H1N1)
NA	GQ338377	H1N1	04/29/2009	USA	A/Texas/36/2009(H1N1)
NA	CY041728	H1N1	04/29/2009	USA	A/New York/3247/2009(H1N1)
NA	CY040832	H1N1	04/29/2009	USA	A/New York/3261/2009(H1N1)
NA	CY040856	H1N1	04/29/2009	USA	A/New York/3264/2009(H1N1)
NA	CY040864	H1N1	04/29/2009	USA	A/New York/3265/2009(H1N1)
NA	CY041164	H1N1	04/29/2009	USA	A/New York/3185/2009(H1N1)
NA	GQ338418	H1N1	04/29/2009	USA	A/Indiana/12/2009(H1N1)
NA	GQ323461	H1N1	04/29/2009	USA	A/Florida/08/2009(H1N1)
NA	CY147757	H1N1	04/29/2009	Mexico	A/Mexico/24030/2009
NA	CY147797	H1N1	04/29/2009	Mexico	A/Mexico/24039/2009
NA	GQ214140	H1N1	04/30/2009	France	A/Paris/2590/2009(H1N1)
NA	GQ214146	H1N1	04/30/2009	France	A/Paris/2580/2009(H1N1)
NA	HQ239841	H1N1	04/30/2009	Canada	A/Canada-SK/RV1683/2009
NA	HQ239835	H1N1	04/30/2009	Canada	A/Canada-NS/RV1561/2009
NA	HQ239834	H1N1	04/30/2009	Canada	A/Canada-NS/RV1560/2009
NA	HQ239836	H1N1	04/30/2009	Canada	A/Canada-NS/RV1562/2009
NA	HQ239833	H1N1	04/30/2009	Canada	A/Canada-NS/RV1559/2009
NA	GQ144461	H1N1	04/30/2009	France	A/Paris/2590/2009(H1N1)
NA	CY147553	H1N1	04/30/2009	Mexico	A/Mexico/24029/2009
NA	GQ122104	H1N1	04/30/2009	Sweden	A/Stockholm/28/2009(H1N1)
NA	GQ166189	H1N1	04/30/2009	Spain	A/Catalonia/88/2009(H1N1)
NA	CY040647	H1N1	04/30/2009	USA	A/New York/3194/2009(H1N1)
NA	CY040695	H1N1	04/30/2009	USA	A/New York/3232/2009(H1N1)
NA	CY041623	H1N1	04/30/2009	USA	A/New York/3100/2009(H1N1)
NA	HM567562	H1N1	04/30/2009	United Ki	A/England/217/2009
NA	GQ323556	H1N1	04/30/2009	USA	A/Ohio/10/2009(H1N1)
NA	GQ323557	H1N1	04/30/2009	USA	A/South Dakota/05/2009(H1N1)
NA	GQ323442	H1N1	04/30/2009	USA	A/Florida/11/2009(H1N1)
NA	GQ323450	H1N1	04/30/2009	USA	A/Rhode Island/03/2009(H1N1)
NA	GQ323562	H1N1	04/30/2009	USA	A/Florida/10/2009(H1N1)
NA	GQ377074	H1N1	04/30/2009	USA	A/Washington/18/2009(H1N1)
NA	GQ476146	H1N1	04/30/2009	USA	A/Minnesota/04/2009(H1N1)
NA	CY074421	H1N1	04/30/2009	USA	A/California/VRDL275/2009(H1N1)
NA	GU931805	H1N1	04/30/2009	Hong Kong	A/Hong Kong/415742/2009(H1N1)
NA	CY074501	H1N1	04/30/2009	USA	A/California/VRDL297/2009(H1N1)
NA	CY074405	H1N1	04/30/2009	USA	A/California/VRDL270/2009(H1N1)
NA	CY074437	H1N1	04/30/2009	USA	A/California/VRDL280/2009(H1N1)

NA	GQ323515	H1N1	04/30/2009	USA	A/Kentucky/05/2009(H1N1)
NA	GQ338360	H1N1	04/30/2009	USA	A/Hawaii/09/2009(H1N1)
NA	GQ338404	H1N1	04/30/2009	USA	A/Pennsylvania/09/2009(H1N1)
NA	GQ323547	H1N1	04/30/2009	USA	A/Louisiana/04/2009(H1N1)
NA	CY074133	H1N1	04/30/2009	USA	A/California/VRDL200/2009(H1N1)
NA	CY074325	H1N1	04/30/2009	USA	A/California/VRDL244/2009(H1N1)
NA	CY046413	H1N1	04/30/2009	USA	A/Wisconsin/629-D00447/2009(H1N1)
NA	CY046261	H1N1	04/30/2009	USA	A/Wisconsin/629-D01773/2009(H1N1)
NA	CY046421	H1N1	04/30/2009	USA	A/Wisconsin/629-D00767/2009(H1N1)
NA	CY046453	H1N1	04/30/2009	USA	A/Wisconsin/629-D01558/2009(H1N1)
NA	CY046477	H1N1	04/30/2009	USA	A/Wisconsin/629-D00015/2009(H1N1)
NA	CY073352	H1N1	04/30/2009	USA	A/San Diego/WR1629P/2009(H1N1)
NA	CY046357	H1N1	04/30/2009	USA	A/Wisconsin/629-D01529/2009(H1N1)
NA	CY046517	H1N1	04/30/2009	USA	A/Wisconsin/629-D02141/2009(H1N1)
NA	CY046845	H1N1	04/30/2009	USA	A/Wisconsin/629-D02361/2009(H1N1)
NA	CY046389	H1N1	04/30/2009	USA	A/Wisconsin/629-D00750/2009(H1N1)
NA	CY073368	H1N1	04/30/2009	USA	A/San Diego/WR1631P/2009(H1N1)
NA	GQ377100	H1N1	04/30/2009	USA	A/California/19/2009(H1N1)
NA	CY047352	H1N1	04/30/2009	USA	A/New York/3365/2009(H1N1)
NA	GQ377036	H1N1	04/30/2009	USA	A/Illinois/02/2009(H1N1)
NA	GQ465707	H1N1	04/30/2009	Canada	A/Canada-NS/RV1565/2009(H1N1)
NA	GQ377044	H1N1	04/30/2009	USA	A/Tennessee/07/2009(H1N1)
NA	GQ465704	H1N1	04/30/2009	Canada	A/Canada-SK/RV1788/2009(H1N1)
NA	CY065280	H1N1	04/30/2009	United Ki	A/England/215/2009
NA	CY074477	H1N1	04/30/2009	USA	A/California/VRDL290/2009(H1N1)
NA	CY074493	H1N1	04/30/2009	USA	A/California/VRDL295/2009(H1N1)
NA	CY074509	H1N1	04/30/2009	USA	A/California/VRDL298/2009(H1N1)
NA	CY074453	H1N1	04/30/2009	USA	A/California/VRDL283/2009(H1N1)
NA	CY074533	H1N1	04/30/2009	USA	A/California/VRDL301/2009(H1N1)
NA	CY074413	H1N1	04/30/2009	USA	A/California/VRDL273/2009(H1N1)
NA	CY074525	H1N1	04/30/2009	USA	A/California/VRDL300/2009(H1N1)
NA	CY074429	H1N1	04/30/2009	USA	A/California/VRDL276/2009(H1N1)
NA	CY074485	H1N1	04/30/2009	USA	A/California/VRDL292/2009(H1N1)
NA	CY064742	H1N1	04/30/2009	Mexico	A/Mexico City/024/2009(H1N1)
NA	CY064710	H1N1	04/30/2009	Mexico	A/Mexico City/020/2009
NA	CY064702	H1N1	04/30/2009	Mexico	A/Mexico City/019/2009
NA	CY065272	H1N1	04/30/2009	United Ki	A/England/214/2009
NA	CY065288	H1N1	04/30/2009	United Ki	A/England/216/2009
NA	CY049830	H1N1	04/30/2009	USA	A/Cherry Point/WR0080/2009(H1N1)
NA	CY107187	H1N1	05/2009	United Ki	A/Scotland/Paisley_409499/2009
NA	CY053655	H1N1	05/2009	Russia	A/Novosibirsk/151/2009(H1N1)
NA	CY039988	H1N1	05/2009	Thailand	A/Nonthaburi/102/2009
NA	GQ377033	H1N1	05/2009	USA	A/Alaska/04/2009(H1N1)
NA	CY107560	H1N1	05/2009	United Ki	A/Scotland/Glasgow_412118/2009
NA	GU292383	H1N1	05/2009	India	A/Hyd/NIV51/2009(H1N1)
NA	GQ144464	H1N1	05/2009	Spain	A/Catalonia/186/2009(H1N1)
NA	GQ152373	H1N1	05/2009	Spain	A/Catalonia/53/2009(H1N1)
NA	GU931685	H1N1	05/2009	Singapore	A/Singapore/DS020090018/2009(H1N1)
NA	GU931686	H1N1	05/2009	Singapore	A/Singapore/DS020090024/2009(H1N1)
NA	AB568134	H1N1	05/2009	Japan	A/Kobe/9154/2009
NA	CY073786	H1N1	05/2009	Canada	A/Vancouver/01/2009(H1N1)
NA	CY087289	H1N1	05/2009	Hong Kong	A/Hong Kong/29992/2009
NA	CY064361	H1N1	05/2009	China	A/Guangdong/491/2009
NA	CY064322	H1N1	05/2009	China	A/Guangdong/357/2009

NA	CY064349	H1N1	05/2009	China	A/Guangdong/448/2009
NA	CY064352	H1N1	05/2009	China	A/Guangdong/455/2009
NA	CY064337	H1N1	05/2009	China	A/Guangdong/370/2009
NA	CY064343	H1N1	05/2009	China	A/Guangdong/383/2009
NA	CY064340	H1N1	05/2009	China	A/Guangdong/371/2009
NA	CY064313	H1N1	05/2009	China	A/Guangdong/336/2009
NA	CY064316	H1N1	05/2009	China	A/Guangdong/348/2009
NA	HM598346	H1N1	05/2009	Thailand	A/Thailand/CU-MV42/2009
NA	AB568129	H1N1	05/2009	Japan	A/Kobe/9109/2009
NA	AB568154	H1N1	05/2009	Japan	A/Kobe/91248/2009
NA	AB568149	H1N1	05/2009	Japan	A/Kobe/9358/2009
NA	AB568144	H1N1	05/2009	Japan	A/Kobe/9336/2009
NA	CY064319	H1N1	05/2009	China	A/Guangdong/356/2009
NA	CY064346	H1N1	05/2009	China	A/Guangdong/387/2009
NA	AB568139	H1N1	05/2009	Japan	A/Kobe/9185/2009
NA	AB568124	H1N1	05/2009	Japan	A/Kobe/9015/2009
NA	GQ258463	H1N1	05/2009	Australia	A/South Australia/2001/2009(H1N1)
NA	GQ292879	H1N1	05/01/2009	Mexico	A/Mexico City/2/2009(H1N1)
NA	GQ292889	H1N1	05/01/2009	Mexico	A/Mexico City/15/2009(H1N1)
NA	GQ292878	H1N1	05/01/2009	Mexico	A/Mexico City/1/2009(H1N1)
NA	GQ292882	H1N1	05/01/2009	Mexico	A/Mexico City/6/2009(H1N1)
NA	GQ292890	H1N1	05/01/2009	Mexico	A/Mexico City/16/2009(H1N1)
NA	GQ292892	H1N1	05/01/2009	Mexico	A/Mexico City/18/2009(H1N1)
NA	CY040458	H1N1	05/01/2009	USA	A/Tennessee/1-560/2009(H1N1)
NA	GQ396547	H1N1	05/01/2009	Spain	A/Andalucia/GP327/2009(H1N1)
NA	GQ396531	H1N1	05/01/2009	Spain	A/CastillaLaMancha/GP369/2009(H1N1)
NA	GQ396583	H1N1	05/01/2009	Spain	A/Extremadura/GP360/2009(H1N1)
NA	GQ292891	H1N1	05/01/2009	Mexico	A/Mexico City/17/2009(H1N1)
NA	GQ292893	H1N1	05/01/2009	Mexico	A/Mexico City/20/2009(H1N1)
NA	GQ169710	H1N1	05/01/2009	Ireland	A/Dublin/01/2009(H1N1)
NA	GQ292888	H1N1	05/01/2009	Mexico	A/Mexico City/14/2009(H1N1)
NA	GQ292880	H1N1	05/01/2009	Mexico	A/Mexico City/4/2009(H1N1)
NA	GQ292881	H1N1	05/01/2009	Mexico	A/Mexico City/5/2009(H1N1)
NA	GQ292883	H1N1	05/01/2009	Mexico	A/Mexico City/7/2009(H1N1)
NA	GQ292884	H1N1	05/01/2009	Mexico	A/Mexico City/9/2009(H1N1)
NA	GQ292885	H1N1	05/01/2009	Mexico	A/Mexico City/11/2009(H1N1)
NA	GQ292886	H1N1	05/01/2009	Mexico	A/Mexico City/12/2009(H1N1)
NA	GQ292887	H1N1	05/01/2009	Mexico	A/Mexico City/13/2009(H1N1)
NA	CY041180	H1N1	05/01/2009	USA	A/New York/3187/2009(H1N1)
NA	GQ144457	H1N1	05/01/2009	France	A/Paris/2592/2009(H1N1)
NA	GQ166190	H1N1	05/01/2009	Spain	A/Catalonia/117/2009(H1N1)
NA	GQ166191	H1N1	05/01/2009	Spain	A/Catalonia/122/2009(H1N1)
NA	GQ166192	H1N1	05/01/2009	Spain	A/Catalonia/127/2009(H1N1)
NA	GQ166193	H1N1	05/01/2009	Spain	A/Catalonia/128/2009(H1N1)
NA	GQ144459	H1N1	05/01/2009	France	A/Paris/2591/2009(H1N1)
NA	GQ338330	H1N1	05/01/2009	USA	A/Missouri/02/2009(H1N1)
NA	GQ323559	H1N1	05/01/2009	USA	A/Florida/09/2009(H1N1)
NA	CY046189	H1N1	05/01/2009	USA	A/Wisconsin/629-D01793/2009(H1N1)
NA	CY041639	H1N1	05/01/2009	USA	A/New York/3307/2009(H1N1)
NA	CY064750	H1N1	05/01/2009	Mexico	A/Mexico City/025/2009(H1N1)
NA	GQ254710	H1N1	05/01/2009	France	A/Paris/2591/2009(H1N1)
NA	GQ323472	H1N1	05/01/2009	USA	A/Louisiana/05/2009(H1N1)
NA	GQ323570	H1N1	05/01/2009	USA	A/Louisiana/03/2009(H1N1)
NA	GQ338380	H1N1	05/01/2009	USA	A/Oklahoma/03/2009(H1N1)

NA	GQ457516	H1N1	05/01/2009	USA	A/California/27/2009(H1N1)
NA	CY047344	H1N1	05/01/2009	USA	A/New York/3345/2009(H1N1)
NA	GQ465702	H1N1	05/01/2009	Canada	A/Canada-AB/RV1644/2009(H1N1)
NA	GQ377077	H1N1	05/01/2009	USA	A/South Carolina/13/2009(H1N1)
NA	GQ377084	H1N1	05/01/2009	USA	A/California/20/2009(H1N1)
NA	CY046461	H1N1	05/01/2009	USA	A/Wisconsin/629-D01861/2009(H1N1)
NA	CY046317	H1N1	05/01/2009	USA	A/Wisconsin/629-D01308/2009(H1N1)
NA	CY046973	H1N1	05/01/2009	USA	A/New York/3443/2009(H1N1)
NA	CY046493	H1N1	05/01/2009	USA	A/Wisconsin/629-D02028/2009(H1N1)
NA	CY074381	H1N1	05/01/2009	USA	A/California/VRDL258/2009(H1N1)
NA	CY074517	H1N1	05/01/2009	USA	A/California/VRDL299/2009(H1N1)
NA	CY074469	H1N1	05/01/2009	USA	A/California/VRDL289/2009(H1N1)
NA	CY064734	H1N1	05/01/2009	Mexico	A/Mexico City/023/2009(H1N1)
NA	CY046429	H1N1	05/01/2009	USA	A/Wisconsin/629-D00677/2009(H1N1)
NA	CY046293	H1N1	05/01/2009	USA	A/Wisconsin/629-D01817/2009(H1N1)
NA	CY053097	H1N1	05/01/2009	USA	A/Houston/150S/2009(H1N1)
NA	CY053081	H1N1	05/01/2009	USA	A/Houston/140S/2009H1N1)
NA	CY053105	H1N1	05/01/2009	USA	A/Houston/15H/2009(H1N1)
NA	CY053089	H1N1	05/01/2009	USA	A/Houston/14H/2009(H1N1)
NA	GQ222423	H1N1	05/01/2009	France	A/Paris/2592/2009(H1N1)
NA	CY064718	H1N1	05/01/2009	Mexico	A/Mexico City/021/2009
NA	GQ292896	H1N1	05/02/2009	Mexico	A/Mexico City/24/2009(H1N1)
NA	GQ292907	H1N1	05/02/2009	Mexico	A/Mexico City/36/2009(H1N1)
NA	GQ292894	H1N1	05/02/2009	Mexico	A/Mexico City/21/2009(H1N1)
NA	GQ292898	H1N1	05/02/2009	Mexico	A/Mexico City/26/2009(H1N1)
NA	GQ292899	H1N1	05/02/2009	Mexico	A/Mexico City/27/2009(H1N1)
NA	GQ292905	H1N1	05/02/2009	Mexico	A/Mexico City/34/2009(H1N1)
NA	GQ292908	H1N1	05/02/2009	Mexico	A/Mexico City/39/2009(H1N1)
NA	GQ292909	H1N1	05/02/2009	Mexico	A/Mexico City/40/2009(H1N1)
NA	GQ292900	H1N1	05/02/2009	Mexico	A/Mexico City/28/2009(H1N1)
NA	GQ292902	H1N1	05/02/2009	Mexico	A/Mexico City/31/2009(H1N1)
NA	GQ292903	H1N1	05/02/2009	Mexico	A/Mexico City/32/2009(H1N1)
NA	GQ292897	H1N1	05/02/2009	Mexico	A/Mexico City/25/2009(H1N1)
NA	GQ365668	H1N1	05/02/2009	Germany	A/Brandenburg/19/2009(H1N1)
NA	GQ292901	H1N1	05/02/2009	Mexico	A/Mexico City/30/2009(H1N1)
NA	GQ292906	H1N1	05/02/2009	Mexico	A/Mexico City/35/2009(H1N1)
NA	GQ292895	H1N1	05/02/2009	Mexico	A/Mexico City/22/2009(H1N1)
NA	GQ292904	H1N1	05/02/2009	Mexico	A/Mexico City/33/2009(H1N1)
NA	CY041076	H1N1	05/02/2009	USA	A/New York/3170/2009(H1N1)
NA	HM567570	H1N1	05/02/2009	United Ki	A/England/221/2009
NA	CY065328	H1N1	05/02/2009	United Ki	A/England/220/2009
NA	CY049869	H1N1	05/02/2009	USA	A/Cherry Point/WR0101/2009(H1N1)
NA	CY046205	H1N1	05/02/2009	USA	A/Wisconsin/629-D02408/2009(H1N1)
NA	CY064726	H1N1	05/02/2009	Mexico	A/Mexico City/022/2009
NA	CY064758	H1N1	05/02/2009	Mexico	A/Mexico City/026/2009(H1N1)
NA	CY041599	H1N1	05/02/2009	USA	A/New York/3177/2009(H1N1)
NA	CY041808	H1N1	05/02/2009	USA	A/New York/3408/2009(H1N1)
NA	CY065312	H1N1	05/02/2009	United Ki	A/England/246/2009
NA	GQ338351	H1N1	05/02/2009	USA	A/Pennsylvania/08/2009(H1N1)
NA	GQ338342	H1N1	05/02/2009	USA	A/Texas/29/2009(H1N1)
NA	CY073388	H1N1	05/02/2009	USA	A/San Diego/WR1634P/2009(H1N1)
NA	CY073428	H1N1	05/02/2009	USA	A/San Diego/WR1641P/2009(H1N1)
NA	CY046341	H1N1	05/02/2009	USA	A/Wisconsin/629-D02455/2009(H1N1)
NA	CY046197	H1N1	05/02/2009	USA	A/Wisconsin/629-D02312/2009(H1N1)

NA	CY046853	H1N1	05/02/2009	USA	A/Wisconsin/629-D00244/2009(H1N1)
NA	CY046381	H1N1	05/02/2009	USA	A/Wisconsin/629-D01980/2009(H1N1)
NA	CY046237	H1N1	05/02/2009	USA	A/Wisconsin/629-D01735/2009(H1N1)
NA	CY046397	H1N1	05/02/2009	USA	A/Wisconsin/629-D01189/2009(H1N1)
NA	CY074461	H1N1	05/02/2009	USA	A/California/VRDL284/2009(H1N1)
NA	CY074541	H1N1	05/02/2009	USA	A/California/VRDL311/2009(H1N1)
NA	GQ132185	H1N1	05/02/2009	South Ko	A/Korea/01/2009
NA	CY046269	H1N1	05/02/2009	USA	A/Wisconsin/629-D00933/2009(H1N1)
NA	CY046437	H1N1	05/02/2009	USA	A/Wisconsin/629-D02262/2009(H1N1)
NA	CY065320	H1N1	05/02/2009	United Ki	A/England/219/2009
NA	CY064419	H1N1	05/02/2009	Mexico	A/Mexico city/CIA11/2009(H1N1)
NA	CY049861	H1N1	05/02/2009	USA	A/Cherry Point/WR0100/2009(H1N1)
NA	CY049877	H1N1	05/02/2009	USA	A/Cherry Point/WR0102/2009(H1N1)
NA	CY069783	H1N1	05/02/2009	United Ki	A/England/221/2009
NA	CY065304	H1N1	05/02/2009	United Ki	A/England/218/2009
NA	CY065336	H1N1	05/02/2009	United Ki	A/England/201/2009
NA	CY054297	H1N1	05/02/2009	Mexico	A/Mexico City/MCIG01/2009(H1N1)
NA	GQ292921	H1N1	05/03/2009	Mexico	A/Mexico City/56/2009(H1N1)
NA	GQ292925	H1N1	05/03/2009	Mexico	A/Mexico City/61/2009(H1N1)
NA	GQ292927	H1N1	05/03/2009	Mexico	A/Mexico City/64/2009(H1N1)
NA	GQ292918	H1N1	05/03/2009	Mexico	A/Mexico City/53/2009(H1N1)
NA	GQ292913	H1N1	05/03/2009	Mexico	A/Mexico City/47/2009(H1N1)
NA	GQ292922	H1N1	05/03/2009	Mexico	A/Mexico City/57/2009(H1N1)
NA	GQ292910	H1N1	05/03/2009	Mexico	A/Mexico City/44/2009(H1N1)
NA	GQ292920	H1N1	05/03/2009	Mexico	A/Mexico City/55/2009(H1N1)
NA	GQ292924	H1N1	05/03/2009	Mexico	A/Mexico City/59/2009(H1N1)
NA	GQ292926	H1N1	05/03/2009	Mexico	A/Mexico City/62/2009(H1N1)
NA	GQ292914	H1N1	05/03/2009	Mexico	A/Mexico City/48/2009(H1N1)
NA	GQ292915	H1N1	05/03/2009	Mexico	A/Mexico City/49/2009(H1N1)
NA	GQ292916	H1N1	05/03/2009	Mexico	A/Mexico City/51/2009(H1N1)
NA	GQ292919	H1N1	05/03/2009	Mexico	A/Mexico City/54/2009(H1N1)
NA	GQ292917	H1N1	05/03/2009	Mexico	A/Mexico City/52/2009(H1N1)
NA	GQ292911	H1N1	05/03/2009	Mexico	A/Mexico City/45/2009(H1N1)
NA	GQ292912	H1N1	05/03/2009	Mexico	A/Mexico City/46/2009(H1N1)
NA	GQ292923	H1N1	05/03/2009	Mexico	A/Mexico City/58/2009(H1N1)
NA	GQ251037	H1N1	05/03/2009	Italy	A/Italy/05/2009(H1N1)
NA	GQ323532	H1N1	05/03/2009	USA	A/Massachusetts/10/2009(H1N1)
NA	CY046349	H1N1	05/03/2009	USA	A/Wisconsin/629-D01017/2009(H1N1)
NA	CY046213	H1N1	05/03/2009	USA	A/Wisconsin/629-D00869/2009(H1N1)
NA	CY046373	H1N1	05/03/2009	USA	A/Wisconsin/629-D01705/2009(H1N1)
NA	CY046229	H1N1	05/03/2009	USA	A/Wisconsin/629-D01296/2009(H1N1)
NA	CY046245	H1N1	05/03/2009	USA	A/Wisconsin/629-D01606/2009(H1N1)
NA	CY046885	H1N1	05/03/2009	USA	A/Wisconsin/629-D00498/2009(H1N1)
NA	CY046277	H1N1	05/03/2009	USA	A/Wisconsin/629-D01092/2009(H1N1)
NA	CY046285	H1N1	05/03/2009	USA	A/Wisconsin/629-D02473/2009(H1N1)
NA	CY069791	H1N1	05/03/2009	United Ki	A/England/222/2009
NA	CY065296	H1N1	05/03/2009	United Ki	A/Scotland/4/2009
NA	CY065352	H1N1	05/03/2009	United Ki	A/England/247/2009
NA	CY064412	H1N1	05/03/2009	Mexico	A/Mexico city/CIA10/2009(H1N1)
NA	GQ292937	H1N1	05/04/2009	Mexico	A/Mexico City/103/2009(H1N1)
NA	GQ292932	H1N1	05/04/2009	Mexico	A/Mexico City/69/2009(H1N1)
NA	GQ292938	H1N1	05/04/2009	Mexico	A/Mexico City/104/2009(H1N1)
NA	GQ292939	H1N1	05/04/2009	Mexico	A/Mexico City/105/2009(H1N1)
NA	GQ292934	H1N1	05/04/2009	Mexico	A/Mexico City/72/2009(H1N1)

NA	GQ292935	H1N1	05/04/2009	Mexico	A/Mexico City/73/2009(H1N1)
NA	GQ365676	H1N1	05/04/2009	Germany	A/Brandenburg/20/2009(H1N1)
NA	GQ292933	H1N1	05/04/2009	Mexico	A/Mexico City/70/2009(H1N1)
NA	GQ232005	H1N1	05/04/2009	USA	A/Vermont/03/2009(H1N1)
NA	GQ323548	H1N1	05/04/2009	USA	A/Wisconsin/11/2009(H1N1)
NA	GQ323549	H1N1	05/04/2009	USA	A/Georgia/02/2009(H1N1)
NA	GQ377040	H1N1	05/04/2009	USA	A/Montana/07/2009(H1N1)
NA	CY046221	H1N1	05/04/2009	USA	A/Wisconsin/629-D01251/2009(H1N1)
NA	CY041784	H1N1	05/04/2009	USA	A/New York/3337/2009(H1N1)
NA	CY041647	H1N1	05/04/2009	USA	A/New York/3313/2009(H1N1)
NA	GQ323468	H1N1	05/04/2009	USA	A/Kentucky/07/2009(H1N1)
NA	GQ323571	H1N1	05/04/2009	USA	A/Missouri/03/2009(H1N1)
NA	GQ323572	H1N1	05/04/2009	USA	A/Wisconsin/10/2009(H1N1)
NA	GQ323497	H1N1	05/04/2009	USA	A/Georgia/03/2009(H1N1)
NA	GQ402236	H1N1	05/04/2009	Canada	A/Canada-SK/RV1767/2009(H1N1)
NA	GQ377048	H1N1	05/04/2009	USA	A/Washington/17/2009(H1N1)
NA	CY046469	H1N1	05/04/2009	USA	A/Wisconsin/629-D01962/2009(H1N1)
NA	CY046325	H1N1	05/04/2009	USA	A/Wisconsin/629-D01810/2009(H1N1)
NA	CY046485	H1N1	05/04/2009	USA	A/Wisconsin/629-D00349/2009(H1N1)
NA	CY045484	H1N1	05/04/2009	Germany	A/Sachsen-Anhalt/101/2009(H1N1)
NA	CY046501	H1N1	05/04/2009	USA	A/Wisconsin/629-D00378/2009(H1N1)
NA	CY046509	H1N1	05/04/2009	USA	A/Wisconsin/629-D02425/2009(H1N1)
NA	CY046365	H1N1	05/04/2009	USA	A/Wisconsin/629-D00790/2009(H1N1)
NA	CY046525	H1N1	05/04/2009	USA	A/Wisconsin/629-D00183/2009(H1N1)
NA	CY046533	H1N1	05/04/2009	USA	A/Wisconsin/629-D00714/2009(H1N1)
NA	CY046861	H1N1	05/04/2009	USA	A/Wisconsin/629-D01415/2009(H1N1)
NA	CY046869	H1N1	05/04/2009	USA	A/Wisconsin/629-D02272/2009(H1N1)
NA	CY046405	H1N1	05/04/2009	USA	A/Wisconsin/629-D00807/2009(H1N1)
NA	CY046877	H1N1	05/04/2009	USA	A/Wisconsin/629-D00396/2009(H1N1)
NA	CY074557	H1N1	05/04/2009	USA	A/California/VRDL324/2009(H1N1)
NA	CY046253	H1N1	05/04/2009	USA	A/Wisconsin/629-D02432/2009(H1N1)
NA	CY046893	H1N1	05/04/2009	USA	A/Wisconsin/629-D01880/2009(H1N1)
NA	CY046445	H1N1	05/04/2009	USA	A/Wisconsin/629-D01226/2009(H1N1)
NA	CY046917	H1N1	05/04/2009	USA	A/Wisconsin/629-D01764/2009(H1N1)
NA	CY065360	H1N1	05/04/2009	United Ki	A/England/XFL00200/2009
NA	CY049845	H1N1	05/04/2009	USA	A/Craven/WR0093/2009(H1N1)
NA	CY050144	H1N1	05/04/2009	USA	A/Wisconsin/629-D01058/2009(H1N1)
NA	CY053144	H1N1	05/04/2009	USA	A/Houston/220S/2009(H1N1)
NA	CY069807	H1N1	05/04/2009	United Ki	A/England/XFL00230/2009
NA	CY069823	H1N1	05/04/2009	United Ki	A/England/250/2009
NA	CY043173	H1N1	05/04/2009	USA	A/New York/3460/2009(H1N1)
NA	CY053152	H1N1	05/04/2009	USA	A/Houston/22H/2009(H1N1)
NA	KC781881	H1N1	05/04/2009	USA	A/Texas/45/2009
NA	CY043205	H1N1	05/04/2009	USA	A/New York/3338/2009(H1N1)
NA	GQ292936	H1N1	05/05/2009	Mexico	A/Mexico City/75/2009(H1N1)
NA	GQ292929	H1N1	05/05/2009	Mexico	A/Mexico City/66/2009(H1N1)
NA	GQ292930	H1N1	05/05/2009	Mexico	A/Mexico City/67/2009(H1N1)
NA	GQ292931	H1N1	05/05/2009	Mexico	A/Mexico City/68/2009(H1N1)
NA	GQ292940	H1N1	05/05/2009	Mexico	A/Mexico City/106/2009(H1N1)
NA	GQ292928	H1N1	05/05/2009	Mexico	A/Mexico City/65/2009(H1N1)
NA	GQ144455	H1N1	05/05/2009	France	A/Paris/2604/2009(H1N1)
NA	GQ219779	H1N1	05/05/2009	Poland	A/Poland/37/2009(H1N1)
NA	GQ323458	H1N1	05/05/2009	USA	A/South Dakota/04/2009(H1N1)
NA	GQ323459	H1N1	05/05/2009	USA	A/Ohio/09/2009(H1N1)

NA	GQ323553	H1N1	05/05/2009	USA	A/Texas/30/2009(H1N1)
NA	GQ476009	H1N1	05/05/2009	USA	A/Virginia/06/2009(H1N1)
NA	GQ323519	H1N1	05/05/2009	USA	A/Michigan/05/2009(H1N1)
NA	CY074445	H1N1	05/05/2009	USA	A/California/VRDL281/2009(H1N1)
NA	GQ457510	H1N1	05/05/2009	USA	A/Montana/06/2009(H1N1)
NA	CY065384	H1N1	05/05/2009	United Ki	A/England/XFL00304/2009
NA	CY065416	H1N1	05/05/2009	United Ki	A/England/254/2009
NA	CY049822	H1N1	05/05/2009	USA	A/Craven/WR0019/2009(H1N1)
NA	CY069815	H1N1	05/05/2009	United Ki	A/England/249/2009
NA	HM567578	H1N1	05/05/2009	United Ki	A/England/249/2009
NA	CY065368	H1N1	05/05/2009	United Ki	A/England/XFL00275/2009
NA	CY069831	H1N1	05/05/2009	United Ki	A/England/XFL00303/2009
NA	CY069799	H1N1	05/05/2009	United Ki	A/England/XFL00228/2009
NA	CY045492	H1N1	05/06/2009	Germany	A/Sachsen-Anhalt/97/2009(H1N1)
NA	GQ200278	H1N1	05/06/2009	Colombia	A/Colombia/450/2009(H1N1)
NA	GQ179932	H1N1	05/06/2009	Thailand	A/Nonthaburi/102/2009(H1N1)
NA	GQ329090	H1N1	05/06/2009	France	A/Strasbourg/2611/2009(H1N1)
NA	CY041824	H1N1	05/06/2009	USA	A/New York/3352/2009(H1N1)
NA	CY041816	H1N1	05/06/2009	USA	A/New York/3351/2009(H1N1)
NA	CY041752	H1N1	05/06/2009	USA	A/New York/3323/2009(H1N1)
NA	GQ329108	H1N1	05/06/2009	France	A/Strasbourg/2609/2009(H1N1)
NA	GQ377063	H1N1	05/06/2009	USA	A/Virginia/05/2009(H1N1)
NA	CY047336	H1N1	05/06/2009	USA	A/New York/3364/2009(H1N1)
NA	GQ402238	H1N1	05/06/2009	Canada	A/Canada-SK/RV1798/2009(H1N1)
NA	CY046557	H1N1	05/06/2009	USA	A/Wisconsin/629-D00228/2009(H1N1)
NA	GQ169381	H1N1	05/06/2009	Thailand	A/Thailand/104/2009(H1N1)
NA	CY043197	H1N1	05/06/2009	USA	A/New York/3324/2009(H1N1)
NA	MG856198	H1N1	05/07/2009	Mexico	A/Mexico/IBT25/2009
NA	GQ323507	H1N1	05/07/2009	USA	A/Washington/14/2009(H1N1)
NA	GQ160610	H1N1	05/07/2009	Australia	A/Brisbane/17/2009(H1N1)
NA	GQ323493	H1N1	05/07/2009	USA	A/Arkansas/03/2009(H1N1)
NA	GQ338362	H1N1	05/07/2009	USA	A/Wisconsin/12/2009(H1N1)
NA	GQ323546	H1N1	05/07/2009	USA	A/Arizona/07/2009(H1N1)
NA	GQ402235	H1N1	05/07/2009	Canada	A/Canada-QC/RV1759/2009(H1N1)
NA	GQ465705	H1N1	05/07/2009	Canada	A/Canada-SK/RV1794/2009(H1N1)
NA	GQ465706	H1N1	05/07/2009	Canada	A/Canada-SK/RV1797/2009(H1N1)
NA	CY074549	H1N1	05/07/2009	USA	A/California/VRDL320/2009(H1N1)
NA	CY046605	H1N1	05/07/2009	USA	A/Wisconsin/629-D00487/2009(H1N1)
NA	CY065400	H1N1	05/07/2009	United Ki	A/England/XFL00311/2009
NA	CY065376	H1N1	05/07/2009	United Ki	A/England/XFL00300/2009
NA	CY050200	H1N1	05/07/2009	Mexico	A/Mexico City/001/2009
NA	CY100450	H1N1	05/07/2009	Mexico	A/Mexico City/IBT22/2009
NA	CY065408	H1N1	05/07/2009	United Ki	A/England/253/2009
NA	CY040874	H1N1	05/07/2009	Norway	A/Norway/1168/2009(H1N1)
NA	KC782032	H1N1	05/07/2009	USA	A/Washington/51/2009
NA	CY100458	H1N1	05/07/2009	Mexico	A/Mexico City/IBT23/2009
NA	HQ239845	H1N1	05/08/2009	Canada	A/Canada-MB/RV1765/2009
NA	GQ338406	H1N1	05/08/2009	USA	A/New York/57/2009(H1N1)
NA	CY050208	H1N1	05/08/2009	Mexico	A/Mexico City/002/2009(H1N1)
NA	GQ402237	H1N1	05/08/2009	Canada	A/Canada-SK/RV1793/2009(H1N1)
NA	GQ323540	H1N1	05/08/2009	USA	A/Texas/35/2009(H1N1)
NA	CY041768	H1N1	05/08/2009	USA	A/New York/3354/2009(H1N1)
NA	GQ465703	H1N1	05/08/2009	Canada	A/Canada-PQ/RV1758/2009(H1N1)
NA	CY073360	H1N1	05/08/2009	USA	A/San Diego/WR1630P/2009(H1N1)

NA	CY046541	H1N1	05/08/2009	USA	A/Wisconsin/629-D01313/2009(H1N1)
NA	CY050224	H1N1	05/08/2009	Mexico	A/Mexico City/004/2009(H1N1)
NA	CY074581	H1N1	05/08/2009	USA	A/California/VRDL346/2009(H1N1)
NA	CY074589	H1N1	05/08/2009	USA	A/California/VRDL348/2009(H1N1)
NA	CY046573	H1N1	05/08/2009	USA	A/Wisconsin/629-D00905/2009(H1N1)
NA	CY046581	H1N1	05/08/2009	USA	A/Wisconsin/629-D01909/2009(H1N1)
NA	CY046589	H1N1	05/08/2009	USA	A/Wisconsin/629-D02452/2009(H1N1)
NA	CY065432	H1N1	05/08/2009	United Ki	A/England/256/2009
NA	CY050881	H1N1	05/08/2009	Mexico	A/Mexico City/005/2009(H1N1)
NA	CY050216	H1N1	05/08/2009	Mexico	A/Mexico City/003/2009(H1N1)
NA	CY069839	H1N1	05/08/2009	United Ki	A/England/XFL00364/2009
NA	CY065392	H1N1	05/08/2009	United Ki	A/England/252/2009
NA	GQ166204	H1N1	05/08/2009	Japan	A/Narita/1/2009
NA	GQ166205	H1N1	05/08/2009	Japan	A/Narita/1/2009
NA	CY040882	H1N1	05/08/2009	Norway	A/Norway/1177/2009(H1N1)
NA	CY043125	H1N1	05/08/2009	USA	A/New York/3389/2009(H1N1)
NA	GQ227546	H1N1	05/08/2009	Sweden	A/Stockholm/29/2009(H1N1)
NA	CY050849	H1N1	05/09/2009	Mexico	A/Mexico City/007/2009(H1N1)
NA	CY069847	H1N1	05/09/2009	United Ki	A/England/XFL00376/2009
NA	GQ323445	H1N1	05/09/2009	USA	A/Maine/03/2009(H1N1)
NA	CY046565	H1N1	05/09/2009	USA	A/Wisconsin/629-D01919/2009(H1N1)
NA	CY050774	H1N1	05/09/2009	USA	A/New York/3442/2009(H1N1)
NA	CY050232	H1N1	05/09/2009	Mexico	A/Mexico City/006/2009(H1N1)
NA	CY046613	H1N1	05/09/2009	USA	A/Wisconsin/629-D01055/2009(H1N1)
NA	CY065440	H1N1	05/09/2009	United Ki	A/England/257/2009
NA	CY050256	H1N1	05/09/2009	Mexico	A/Mexico City/012/2009
NA	CY050176	H1N1	05/09/2009	Mexico	A/Mexico City/015/2009
NA	CY062637	H1N1	05/09/2009	Mexico	A/Mexico City/014/2009
NA	CY092103	H1N1	05/09/2009	Mexico	A/Mexico City/016/2009
NA	CY050873	H1N1	05/09/2009	Mexico	A/Mexico City/017/2009
NA	CY050240	H1N1	05/09/2009	Mexico	A/Mexico City/010/2009
NA	CY050248	H1N1	05/09/2009	Mexico	A/Mexico City/011/2009
NA	CY050865	H1N1	05/09/2009	Mexico	A/Mexico City/013/2009
NA	CY045948	H1N1	05/09/2009	Canada	A/Toronto/C2781/2009(H1N1)
NA	CY046597	H1N1	05/10/2009	USA	A/Wisconsin/629-D01482/2009(H1N1)
NA	CY050446	H1N1	05/10/2009	USA	A/Wisconsin/629-D01482/2009(H1N1)
NA	GQ200288	H1N1	05/10/2009	China	A/Shandong/1/2009(H1N1)
NA	GQ323475	H1N1	05/10/2009	USA	A/Massachusetts/09/2009(H1N1)
NA	GQ179931	H1N1	05/10/2009	Thailand	A/Nonthaburi/104/2009(H1N1)
NA	GQ166224	H1N1	05/10/2009	China	A/Sichuan/1/2009(H1N1)
NA	CY074565	H1N1	05/10/2009	USA	A/California/VRDL326/2009(H1N1)
NA	GQ183634	H1N1	05/10/2009	Finland	A/Finland/553/2009(H1N1)
NA	CY050889	H1N1	05/10/2009	Mexico	A/Mexico City/018/2009
NA	CY065424	H1N1	05/10/2009	United Ki	A/England/XFL00354/2009
NA	CY050168	H1N1	05/10/2009	Mexico	A/Mexico City/008/2009(H1N1)
NA	CY046549	H1N1	05/10/2009	USA	A/Wisconsin/629-D00179/2009(H1N1)
NA	JQ173158	H1N1	05/10/2009	Finland	A/Helsinki/P15/2009(H1N1)
NA	CY043221	H1N1	05/11/2009	USA	A/New York/3455/2009(H1N1)
NA	CY043165	H1N1	05/11/2009	USA	A/New York/3454/2009(H1N1)
NA	CY065456	H1N1	05/11/2009	United Ki	A/England/XFL00418/2009
NA	CY041760	H1N1	05/11/2009	USA	A/New York/3413/2009(H1N1)
NA	GQ323448	H1N1	05/11/2009	USA	A/Massachusetts/11/2009(H1N1)
NA	GQ323569	H1N1	05/11/2009	USA	A/North Dakota/04/2009(H1N1)
NA	GQ254714	H1N1	05/11/2009	France	A/Paris/2629/2009(H1N1)

NA	GQ323456	H1N1	05/11/2009	USA	A/Texas/32/2009(H1N1)
NA	CY065448	H1N1	05/11/2009	United Ki	A/England/259/2009
NA	CY069855	H1N1	05/11/2009	United Ki	A/England/260/2009
NA	CY065488	H1N1	05/12/2009	United Ki	A/England/264/2009
NA	CY065472	H1N1	05/12/2009	United Ki	A/England/XFL00473/2009
NA	CY047718	H1N1	05/12/2009	Panama	A/Panama/303053/2009
NA	CY043181	H1N1	05/12/2009	USA	A/New York/3488/2009(H1N1)
NA	CY043301	H1N1	05/12/2009	USA	A/New York/3498/2009(H1N1)
NA	CY043157	H1N1	05/12/2009	USA	A/New York/3418/2009(H1N1)
NA	HE804115	H1N1	05/12/2009	Uruguay	A/Uruguay/878/2009
NA	GQ323453	H1N1	05/12/2009	USA	A/Texas/33/2009(H1N1)
NA	GQ323575	H1N1	05/12/2009	USA	A/Texas/34/2009(H1N1)
NA	CY065496	H1N1	05/12/2009	United Ki	A/England/265/2009
NA	CY065464	H1N1	05/12/2009	United Ki	A/England/XFL00419/2009
NA	CY069863	H1N1	05/12/2009	United Ki	A/England/XFL00421/2009
NA	GQ475928	H1N1	05/12/2009	USA	A/Kentucky/08/2009(H1N1)
NA	GQ475871	H1N1	05/12/2009	USA	A/Kentucky/08/2009(H1N1)
NA	CY050857	H1N1	05/12/2009	Mexico	A/Mexico City/009/2009(H1N1)
NA	CY050330	H1N1	05/12/2009	USA	A/Wisconsin/629-D01851/2009(H1N1)
NA	CY065480	H1N1	05/12/2009	United Ki	A/England/263/2009
NA	CY047716	H1N1	05/12/2009	Panama	A/Panama/303052/2009
NA	CY065504	H1N1	05/13/2009	United Ki	A/England/XFL00481/2009
NA	KC781315	H1N1	05/13/2009	USA	A/Mississippi/01/2009
NA	CY043213	H1N1	05/13/2009	USA	A/New York/3420/2009(H1N1)
NA	CY046677	H1N1	05/13/2009	USA	A/Wisconsin/629-D01305/2009(H1N1)
NA	CY065512	H1N1	05/13/2009	United Ki	A/England/XFL00482/2009
NA	CY050374	H1N1	05/13/2009	USA	A/Wisconsin/629-D01154/2009(H1N1)
NA	CY051905	H1N1	05/13/2009	USA	A/Houston/10S/2009(H1N1)
NA	CY053057	H1N1	05/13/2009	USA	A/Houston/100S/2009(H1N1)
NA	CY053065	H1N1	05/13/2009	USA	A/Houston/10H/2009(H1N1)
NA	CY052961	H1N1	05/13/2009	USA	A/Houston/1H/2009(H1N1)
NA	CY047720	H1N1	05/13/2009	Panama	A/Panama/303066/2009
NA	CY050758	H1N1	05/13/2009	USA	A/New York/3467/2009(H1N1)
NA	GQ396525	H1N1	05/13/2009	Spain	A/Madrid/GP523/2009(H1N1)
NA	CY043285	H1N1	05/13/2009	USA	A/New York/3449/2009(H1N1)
NA	CY043133	H1N1	05/14/2009	USA	A/New York/3514/2009(H1N1)
NA	GQ896364	H1N1	05/14/2009	USA	A/Wisconsin/13/2009(H1N1)
NA	GQ476115	H1N1	05/14/2009	USA	A/Wisconsin/13/2009(H1N1)
NA	CY046653	H1N1	05/14/2009	USA	A/Wisconsin/629-D01837/2009(H1N1)
NA	CY046661	H1N1	05/14/2009	USA	A/Wisconsin/629-D00997/2009(H1N1)
NA	CY041792	H1N1	05/14/2009	USA	A/New York/3463/2009(H1N1)
NA	CY046685	H1N1	05/14/2009	USA	A/Wisconsin/629-D00978/2009(H1N1)
NA	CY047728	H1N1	05/14/2009	Panama	A/Panama/303306/2009
NA	GQ200599	H1N1	05/14/2009	Turkey	A/Istanbul/30/2009(H1N1)
NA	GQ476112	H1N1	05/14/2009	USA	A/Wisconsin/13/2009(H1N1)
NA	CY046669	H1N1	05/14/2009	USA	A/Wisconsin/629-D00064/2009(H1N1)
NA	CY065528	H1N1	05/15/2009	United Ki	A/England/XFL00532/2009
NA	CY046629	H1N1	05/15/2009	USA	A/Wisconsin/629-D01839/2009(H1N1)
NA	CY050390	H1N1	05/15/2009	USA	A/Wisconsin/629-D01839/2009(H1N1)
NA	CY050382	H1N1	05/15/2009	USA	A/Wisconsin/629-D01295/2009(H1N1)
NA	CY083298	H1N1	05/15/2009	USA	A/San Diego/WRAIR1647P/2009
NA	CY048927	H1N1	05/15/2009	Malaysia	A/Malaysia/820/2009(H1N1)
NA	GQ323477	H1N1	05/15/2009	USA	A/Utah/03/2009(H1N1)
NA	CY047722	H1N1	05/15/2009	Panama	A/Panama/303238/2009

NA	CY047726	H1N1	05/15/2009	Panama	A/Panama/303270/2009
NA	HQ239847	H1N1	05/15/2009	Canada	A/Canada-MB/RV1927/2009
NA	GQ225375	H1N1	05/15/2009	China	A/Beijing/01/2009(H1N1)
NA	CY044975	H1N1	05/15/2009	USA	A/New York/3744/2009(H1N1)
NA	CY065520	H1N1	05/15/2009	United Ki	A/England/266/2009
NA	CY047724	H1N1	05/15/2009	Panama	A/Panama/303246/2009
NA	CY043189	H1N1	05/16/2009	USA	A/New York/3499/2009(H1N1)
NA	CY041776	H1N1	05/16/2009	USA	A/New York/3532/2009(H1N1)
NA	GQ220736	H1N1	05/16/2009	Japan	A/Osaka-C/1/2009(H1N1)
NA	CY043293	H1N1	05/16/2009	USA	A/New York/3469/2009(H1N1)
NA	CY043237	H1N1	05/16/2009	USA	A/New York/3502/2009(H1N1)
NA	CY043253	H1N1	05/16/2009	USA	A/New York/3545/2009(H1N1)
NA	CY043245	H1N1	05/16/2009	USA	A/New York/3509/2009(H1N1)
NA	CY048935	H1N1	05/16/2009	Malaysia	A/Malaysia/854/2009(H1N1)
NA	GQ183619	H1N1	05/16/2009	China	A/Beijing/01/2009(H1N1)
NA	GQ183627	H1N1	05/16/2009	China	A/Beijing/02/2009(H1N1)
NA	CY046621	H1N1	05/16/2009	USA	A/Wisconsin/629-D00575/2009(H1N1)
NA	CY046637	H1N1	05/16/2009	USA	A/Wisconsin/629-D00967/2009(H1N1)
NA	CY046645	H1N1	05/16/2009	USA	A/Wisconsin/629-D02227/2009(H1N1)
NA	GQ220734	H1N1	05/16/2009	Japan	A/Osaka/1/2009(H1N1)
NA	CY046693	H1N1	05/16/2009	USA	A/Wisconsin/629-D00434/2009(H1N1)
NA	CY062542	H1N1	05/16/2009	Mexico	A/Mexico city/CIA9/2009(H1N1)
NA	CY044983	H1N1	05/16/2009	USA	A/New York/3747/2009(H1N1)
NA	GQ220735	H1N1	05/16/2009	Japan	A/Osaka/2/2009(H1N1)
NA	GQ220731	H1N1	05/17/2009	Japan	A/Hyogo/1/2009(H1N1)
NA	GQ221696	H1N1	05/17/2009	China	A/Guangdong/1/2009(H1N1)
NA	CY043229	H1N1	05/17/2009	USA	A/New York/3501/2009(H1N1)
NA	GQ402239	H1N1	05/17/2009	Canada	A/Canada-QC/RV1954/2009(H1N1)
NA	CY044130	H1N1	05/17/2009	USA	A/New York/3516/2009(H1N1)
NA	CY083290	H1N1	05/17/2009	USA	A/San Diego/WRAIR1646P/2009
NA	CY074573	H1N1	05/17/2009	USA	A/California/VRDL341/2009(H1N1)
NA	CY050398	H1N1	05/17/2009	USA	A/Wisconsin/629-D00989/2009(H1N1)
NA	CY069871	H1N1	05/17/2009	United Ki	A/England/271/2009
NA	GQ200281	H1N1	05/17/2009	Chile	A/Chile/8947/2009(H1N1)
NA	CY047734	H1N1	05/17/2009	Panama	A/Panama/303338/2009
NA	CY047733	H1N1	05/17/2009	Panama	A/Panama/303333/2009
NA	CY047731	H1N1	05/17/2009	Panama	A/Panama/303328/2009
NA	CY043309	H1N1	05/17/2009	USA	A/New York/3534/2009(H1N1)
NA	GQ220732	H1N1	05/17/2009	Japan	A/Hyogo/2/2009(H1N1)
NA	CY047735	H1N1	05/17/2009	Panama	A/Panama/303345/2009
NA	GQ329102	H1N1	05/17/2009	France	A/Paris/2650/2009(H1N1)
NA	CY045087	H1N1	05/17/2009	USA	A/New York/3574/2009(H1N1)
NA	CY041800	H1N1	05/17/2009	USA	A/New York/3468/2009(H1N1)
NA	GQ220737	H1N1	05/17/2009	Japan	A/Osaka-C/2/2009(H1N1)
NA	CY052993	H1N1	05/18/2009	USA	A/Houston/3H/2009(H1N1)
NA	GQ223445	H1N1	05/18/2009	China	A/Guangdong/1/2009(H1N1)
NA	GQ896361	H1N1	05/18/2009	USA	A/Utah/12/2009(H1N1)
NA	GQ896371	H1N1	05/18/2009	USA	A/Utah/12/2009(H1N1)
NA	GQ377067	H1N1	05/18/2009	USA	A/New York/59/2009(H1N1)
NA	GQ261273	H1N1	05/18/2009	Japan	A/Himeji/1/2009(H1N1)
NA	CY044911	H1N1	05/18/2009	USA	A/New York/3612/2009(H1N1)
NA	HQ011412	H1N1	05/18/2009	China	A/Guangdong/01/2009(H1N1)
NA	CY051913	H1N1	05/18/2009	USA	A/Houston/2H/2009(H1N1)
NA	CY052985	H1N1	05/18/2009	USA	A/Houston/30S/2009(H1N1)

NA	CY052969	H1N1	05/18/2009	USA	A/Houston/20S/2009(H1N1)
NA	CY047740	H1N1	05/18/2009	Panama	A/Panama/303378/2009
NA	CY047737	H1N1	05/18/2009	Panama	A/Panama/303360/2009
NA	GQ200279	H1N1	05/18/2009	Chile	A/Chile/8886/2009(H1N1)
NA	GQ200283	H1N1	05/18/2009	Chile	A/Chile/8949/2009(H1N1)
NA	CY044090	H1N1	05/18/2009	USA	A/New York/3740/2009(H1N1)
NA	HM569707	H1N1	05/18/2009	Argentina	A/Argentina/19659/2009(H1N1)
NA	CY043141	H1N1	05/19/2009	USA	A/New York/3529/2009(H1N1)
NA	GQ323488	H1N1	05/19/2009	USA	A/Arizona/09/2009(H1N1)
NA	CY053009	H1N1	05/19/2009	USA	A/Houston/5H/2009(H1N1)
NA	CY043269	H1N1	05/19/2009	USA	A/New York/3563/2009(H1N1)
NA	CY047360	H1N1	05/19/2009	USA	A/New York/3741/2009(H1N1)
NA	GQ287622	H1N1	05/19/2009	Japan	A/Shiga/2/2009(H1N1)
NA	CY044138	H1N1	05/19/2009	USA	A/New York/3528/2009(H1N1)
NA	CY044887	H1N1	05/19/2009	USA	A/New York/3580/2009(H1N1)
NA	CY046773	H1N1	05/19/2009	USA	A/Wisconsin/629-D00722/2009(H1N1)
NA	CY046717	H1N1	05/19/2009	USA	A/Wisconsin/629-D00752/2009(H1N1)
NA	CY045145	H1N1	05/19/2009	USA	A/New York/3577/2009(H1N1)
NA	CY046741	H1N1	05/19/2009	USA	A/Wisconsin/629-D00084/2009(H1N1)
NA	CY046757	H1N1	05/19/2009	USA	A/Wisconsin/629-D00160/2009(H1N1)
NA	CY050414	H1N1	05/19/2009	USA	A/Wisconsin/629-D00752/2009(H1N1)
NA	CY083314	H1N1	05/19/2009	USA	A/San Diego/WRAIR1649P/2009
NA	GQ243752	H1N1	05/19/2009	Australia	A/Victoria/2001/2009(H1N1)
NA	GQ396556	H1N1	05/19/2009	Spain	A/Madrid/GP575/2009(H1N1)
NA	HQ239849	H1N1	05/19/2009	Canada	A/Canada-MB/RV1968/2009
NA	HQ239846	H1N1	05/19/2009	Canada	A/Canada-MB/RV1923/2009
NA	GQ200285	H1N1	05/19/2009	Chile	A/Chile/9113/2009(H1N1)
NA	CY044919	H1N1	05/19/2009	USA	A/New York/3613/2009(H1N1)
NA	CY044198	H1N1	05/19/2009	Taiwan	A/Taiwan/T0724/2009(H1N1)
NA	CY044066	H1N1	05/19/2009	USA	A/New York/3568/2009(H1N1)
NA	CY053001	H1N1	05/19/2009	USA	A/Houston/50S/2009(H1N1)
NA	CY043261	H1N1	05/20/2009	USA	A/New York/3552/2009(H1N1)
NA	CY044935	H1N1	05/20/2009	USA	A/New York/3623/2009(H1N1)
NA	CY053176	H1N1	05/20/2009	USA	A/Brownsville/260S/2009(H1N1)
NA	CY053184	H1N1	05/20/2009	USA	A/Brownsville/270S/2009(H1N1)
NA	CY053017	H1N1	05/20/2009	USA	A/Houston/60S/2009(H1N1)
NA	CY053025	H1N1	05/20/2009	USA	A/Houston/6H/2009(H1N1)
NA	CY044058	H1N1	05/20/2009	USA	A/New York/3567/2009(H1N1)
NA	GQ290107	H1N1	05/20/2009	China	A/Beijing/502/2009(H1N1)
NA	GQ402240	H1N1	05/20/2009	Canada	A/Canada-MB/RV1964/2009(H1N1)
NA	CY043149	H1N1	05/20/2009	USA	A/New York/3551/2009(H1N1)
NA	CY044050	H1N1	05/20/2009	USA	A/New York/3566/2009(H1N1)
NA	CY044871	H1N1	05/20/2009	USA	A/New York/3571/2009(H1N1)
NA	CY044895	H1N1	05/20/2009	USA	A/New York/3582/2009(H1N1)
NA	CY046709	H1N1	05/20/2009	USA	A/Wisconsin/629-D01787/2009(H1N1)
NA	GQ243754	H1N1	05/20/2009	Philippin	A/Philippines/2001/2009(H1N1)
NA	CY064903	H1N1	05/20/2009	Italy	A/Rome/688/2009(H1N1)
NA	GQ225383	H1N1	05/20/2009	China	A/Beijing/3/2009(H1N1)
NA	HQ239851	H1N1	05/20/2009	Canada	A/Canada-MB/RV1973/2009
NA	HQ239848	H1N1	05/20/2009	Canada	A/Canada-MB/RV1967/2009
NA	CY046733	H1N1	05/20/2009	USA	A/Wisconsin/629-D01521/2009(H1N1)
NA	CY046725	H1N1	05/20/2009	USA	A/Wisconsin/629-D02427/2009(H1N1)
NA	CY053049	H1N1	05/21/2009	USA	A/Houston/9H/2009(H1N1)
NA	CY051921	H1N1	05/21/2009	USA	A/Houston/90S/2009(H1N1)

NA	CY087010	H1N1	05/21/2009	USA	A/New York/3565/2009(H1N1)
NA	CY053033	H1N1	05/21/2009	USA	A/Houston/70S/2009(H1N1)
NA	CY052977	H1N1	05/21/2009	USA	A/Houston/20S/2009(H1N1)
NA	CY053200	H1N1	05/21/2009	USA	A/Brownsville/300S/2009(H1N1)
NA	CY053192	H1N1	05/21/2009	USA	A/Brownsville/290S/2009(H1N1)
NA	CY053041	H1N1	05/21/2009	USA	A/Houston/7H/2009(H1N1)
NA	GQ402243	H1N1	05/21/2009	Canada	A/Canada-MB/RV1982/2009(H1N1)
NA	GQ261274	H1N1	05/21/2009	Japan	A/Sakai/1/2009(H1N1)
NA	AB514228	H1N1	05/21/2009	Japan	A/Saitama/43/2009(H1N1)
NA	CY044879	H1N1	05/21/2009	USA	A/New York/3573/2009(H1N1)
NA	CY044903	H1N1	05/21/2009	USA	A/New York/3611/2009(H1N1)
NA	GQ219585	H1N1	05/21/2009	Russia	A/Moscow/IIV01/2009(H1N1)
NA	CY046965	H1N1	05/21/2009	USA	A/New York/3576/2009(H1N1)
NA	GQ243762	H1N1	05/21/2009	Australia	A/Victoria/2004/2009(H1N1)
NA	CY089703	H1N1	05/21/2009	USA	A/New York/3750/2009
NA	HQ239850	H1N1	05/21/2009	Canada	A/Canada-MB/RV1971/2009
NA	GQ184628	H1N1	05/21/2009	Russia	A/Moscow/01/2009(H1N1)
NA	GQ261276	H1N1	05/21/2009	Japan	A/Sakai/2/2009(H1N1)
NA	CY089874	H1N1	05/21/2009	USA	A/Wisconsin/629-D02275/2009(H1N1)
NA	CY044967	H1N1	05/21/2009	USA	A/New York/3653/2009(H1N1)
NA	CY046749	H1N1	05/22/2009	USA	A/Wisconsin/629-D02064/2009(H1N1)
NA	CY044951	H1N1	05/22/2009	USA	A/New York/3627/2009(H1N1)
NA	CY053216	H1N1	05/22/2009	USA	A/Brownsville/31H/2009(H1N1)
NA	GQ225367	H1N1	05/22/2009	China	A/Fujian/1/2009(H1N1)
NA	CY053208	H1N1	05/22/2009	USA	A/Brownsville/310S/2009(H1N1)
NA	GQ402242	H1N1	05/22/2009	Canada	A/Canada-MB/RV1977/2009(H1N1)
NA	GQ329084	H1N1	05/22/2009	France	A/Paris/2670/2009(H1N1)
NA	GQ261278	H1N1	05/22/2009	Japan	A/Shiga/1/2009(H1N1)
NA	GQ896367	H1N1	05/22/2009	USA	A/Wisconsin/14/2009(H1N1)
NA	GQ896377	H1N1	05/22/2009	USA	A/Wisconsin/14/2009(H1N1)
NA	CY044214	H1N1	05/22/2009	Taiwan	A/Taiwan/T1339/2009(H1N1)
NA	GQ377046	H1N1	05/22/2009	USA	A/Texas/39/2009(H1N1)
NA	CY044206	H1N1	05/22/2009	Taiwan	A/Taiwan/T1338/2009(H1N1)
NA	CY046765	H1N1	05/22/2009	USA	A/Wisconsin/629-D02298/2009(H1N1)
NA	HQ239853	H1N1	05/22/2009	Canada	A/Canada-MB/RV1983/2009
NA	HQ239852	H1N1	05/22/2009	Canada	A/Canada-MB/RV1976/2009
NA	CY045095	H1N1	05/22/2009	USA	A/New York/3614/2009(H1N1)
NA	GQ255898	H1N1	05/22/2009	Russia	A/Kaluga/01/2009(H1N1)
NA	CY089181	H1N1	05/22/2009	Chile	A/Chile/19/2009
NA	CY075085	H1N1	05/23/2009	Chile	A/Chile/28/2009(H1N1)
NA	GQ232095	H1N1	05/23/2009	China	A/Beijing/4/2009(H1N1)
NA	CY049917	H1N1	05/23/2009	Dominican	A/Santo Domingo/0574T/2009(H1N1)
NA	CY050406	H1N1	05/23/2009	USA	A/Wisconsin/629-D00451/2009(H1N1)
NA	CY050422	H1N1	05/23/2009	USA	A/Wisconsin/629-D02206/2009(H1N1)
NA	CY053129	H1N1	05/23/2009	USA	A/Houston/200S/2009(H1N1)
NA	CY053224	H1N1	05/23/2009	USA	A/Brownsville/340S/2009(H1N1)
NA	CY053232	H1N1	05/23/2009	USA	A/Brownsville/34H/2009(H1N1)
NA	CY053137	H1N1	05/23/2009	USA	A/Houston/20H/2009(H1N1)
NA	GQ225359	H1N1	05/23/2009	China	A/Shanghai/1/2009(H1N1)
NA	GQ225351	H1N1	05/23/2009	China	A/Zhejiang/1/2009(H1N1)
NA	GQ323529	H1N1	05/23/2009	USA	A/Illinois/04/2009(H1N1)
NA	GQ243750	H1N1	05/23/2009	Philippin	A/Philippines/2009/2009(H1N1)
NA	GQ338336	H1N1	05/23/2009	USA	A/New York/58/2009(H1N1)
NA	CY049909	H1N1	05/23/2009	Dominican	A/Santo Domingo/0574/2009(H1N1)

NA	CY045940	H1N1	05/24/2009	Canada	A/Toronto/C0270/2009(H1N1)
NA	CY087297	H1N1	05/24/2009	Hong Kong	A/Hong Kong/419305/2009
NA	CY087295	H1N1	05/24/2009	Hong Kong	A/Hong Kong/419301/2009
NA	HM567594	H1N1	05/24/2009	Ireland	A/Ireland/1/2009
NA	GQ402241	H1N1	05/24/2009	Canada	A/Canada-MB/RV1975/2009(H1N1)
NA	CY044959	H1N1	05/24/2009	USA	A/New York/3629/2009(H1N1)
NA	CY041985	H1N1	05/24/2009	Dominican	A/Santo Domingo/0573N/2009(H1N1)
NA	CY046701	H1N1	05/24/2009	USA	A/Wisconsin/629-D01445/2009(H1N1)
NA	CY052070	H1N1	05/24/2009	Italy	A/Italy/211/2009(H1N1)
NA	CY041962	H1N1	05/24/2009	Dominican	A/Santo Domingo/572N/2009(H1N1)
NA	GQ253495	H1N1	05/24/2009	China	A/Shanghai/37T/2009(H1N1)
NA	GQ402227	H1N1	05/24/2009	Canada	A/Canada-MB/RV2023/2009(H1N1)
NA	CY075109	H1N1	05/25/2009	Chile	A/Chile/36/2009(H1N1)
NA	CY075117	H1N1	05/25/2009	Chile	A/Chile/40/2009(H1N1)
NA	CY075101	H1N1	05/25/2009	Chile	A/Chile/32/2009(H1N1)
NA	JQ409128	H1N1	05/25/2009	Finland	A/Helsinki/Vi1/2009
NA	CY050430	H1N1	05/25/2009	USA	A/Wisconsin/629-D02324/2009(H1N1)
NA	CY075093	H1N1	05/25/2009	Chile	A/Chile/31/2009
NA	CY044927	H1N1	05/25/2009	USA	A/New York/3617/2009(H1N1)
NA	GQ303551	H1N1	05/25/2009	Spain	A/Catalonia/308/2009(H1N1)
NA	HE804126	H1N1	05/25/2009	Uruguay	A/Uruguay/DR/2009
NA	HE804101	H1N1	05/25/2009	Uruguay	A/Uruguay/PF/2009
NA	GQ214167	H1N1	05/25/2009	Ireland	A/Dublin/02/2009(H1N1)
NA	CY043277	H1N1	05/25/2009	USA	A/New York/3651/2009(H1N1)
NA	CY075125	H1N1	05/25/2009	Chile	A/Chile/42/2009(H1N1)
NA	GQ283487	H1N1	05/26/2009	Finland	A/Finland/554/2009(H1N1)
NA	CY044074	H1N1	05/26/2009	USA	A/New York/3702/2009(H1N1)
NA	HM567586	H1N1	05/26/2009	United Ki	A/England/303/2009
NA	HM567602	H1N1	05/26/2009	United Ki	A/England/313/2009
NA	HM567810	H1N1	05/26/2009	Ireland	A/Ireland/2/2009
NA	HM567834	H1N1	05/26/2009	Ireland	A/Ireland/3/2009
NA	CY051249	H1N1	05/26/2009	USA	A/Wisconsin/629-S0035/2009(H1N1)
NA	GQ385302	H1N1	05/26/2009	Canada	A/Toronto/0462/2009(H1N1)
NA	GQ359769	H1N1	05/26/2009	Sweden	A/Stockholm/30/2009(H1N1)
NA	CY043112	H1N1	05/26/2009	Dominican	A/Santo Domingo/568T/2009(H1N1)
NA	GU065289	H1N1	05/26/2009	Poland	A/Poland/169/2009(H1N1)
NA	GQ375286	H1N1	05/26/2009	Russia	A/Moscow/03/2009(H1N1)
NA	CY043104	H1N1	05/26/2009	Dominican	A/Santo Domingo/565T/2009(H1N1)
NA	CY046781	H1N1	05/26/2009	USA	A/Wisconsin/629-D01894/2009(H1N1)
NA	GQ247727	H1N1	05/26/2009	Russia	A/Moscow/IIV02/2009(H1N1)
NA	GU014805	H1N1	05/26/2009	Japan	A/Shizuoka-C/97/2009(H1N1)
NA	CY046957	H1N1	05/26/2009	USA	A/New York/3654/2009(H1N1)
NA	CY075133	H1N1	05/26/2009	Chile	A/Chile/56/2009(H1N1)
NA	GQ281077	H1N1	05/26/2009	Ireland	A/Dublin/03/2009(H1N1)
NA	GQ243756	H1N1	05/26/2009	Australia	A/Perth/29/2009(H1N1)
NA	CY052071	H1N1	05/26/2009	Italy	A/Italy/212/2009(H1N1)
NA	CY050750	H1N1	05/26/2009	USA	A/New York/3768/2009(H1N1)
NA	HQ239854	H1N1	05/26/2009	Canada	A/Canada-MB/RV1985/2009
NA	HQ239859	H1N1	05/26/2009	Canada	A/Canada-MB/RV2004/2009
NA	CY044943	H1N1	05/26/2009	USA	A/New York/3625/2009(H1N1)
NA	CY045153	H1N1	05/26/2009	USA	A/New York/3655/2009(H1N1)
NA	CY075149	H1N1	05/27/2009	Chile	A/Chile/95/2009(H1N1)
NA	GQ283481	H1N1	05/27/2009	Italy	A/Italy/49/2009(H1N1)
NA	CY076757	H1N1	05/27/2009	Canada	A/Ontario/142358/2009(H1N1)

NA	CY053902	H1N1	05/27/2009	Argentina	A/Argentina/HNRG14/2009(H1N1)
NA	CY049933	H1N1	05/27/2009	USA	A/Craven/WR1001/2009(H1N1)
NA	CY050897	H1N1	05/27/2009	USA	A/Wisconsin/629-D00949/2009(H1N1)
NA	CY050438	H1N1	05/27/2009	USA	A/Wisconsin/629-D01886/2009(H1N1)
NA	CY053248	H1N1	05/27/2009	USA	A/Brownsville/35H/2009(H1N1)
NA	CY053256	H1N1	05/27/2009	USA	A/Brownsville/36H/2009(H1N1)
NA	CY046789	H1N1	05/27/2009	USA	A/Wisconsin/629-D02276/2009(H1N1)
NA	CY046813	H1N1	05/27/2009	USA	A/Wisconsin/629-D00698/2009(H1N1)
NA	CY046837	H1N1	05/27/2009	USA	A/Wisconsin/629-D01883/2009(H1N1)
NA	CY061307	H1N1	05/27/2009	Argentina	A/Argentina/HNRG1001/2009
NA	GQ232087	H1N1	05/27/2009	China	A/Guangdong/02/2009(H1N1)
NA	HQ239861	H1N1	05/27/2009	Canada	A/Canada-MB/RV2005/2009
NA	HQ239857	H1N1	05/27/2009	Canada	A/Canada-MB/RV2003/2009
NA	HQ239855	H1N1	05/27/2009	Canada	A/Canada-MB/RV1989/2009
NA	HQ239896	H1N1	05/27/2009	Canada	A/Canada-MB/RV2043/2009
NA	HM567690	H1N1	05/27/2009	United Ki	A/England/350/2009
NA	HQ239871	H1N1	05/27/2009	Canada	A/Canada-MB/RV2015/2009
NA	HQ239856	H1N1	05/27/2009	Canada	A/Canada-MB/RV2001/2009
NA	CY053240	H1N1	05/27/2009	USA	A/Brownsville/35OS/2009(H1N1)
NA	CY069879	H1N1	05/27/2009	United Ki	A/England/350/2009
NA	CY075141	H1N1	05/27/2009	Chile	A/Chile/88/2009(H1N1)
NA	GQ283492	H1N1	05/28/2009	Finland	A/Finland/555/2009(H1N1)
NA	GQ288374	H1N1	05/28/2009	China	A/Fuzhou/01/2009(H1N1)
NA	CY046901	H1N1	05/28/2009	USA	A/Wisconsin/629-D00829/2009(H1N1)
NA	CY046909	H1N1	05/28/2009	USA	A/Wisconsin/629-D01083/2009(H1N1)
NA	CY050905	H1N1	05/28/2009	USA	A/Wisconsin/629-D01779/2009(H1N1)
NA	CY050913	H1N1	05/28/2009	USA	A/Wisconsin/629-D01262/2009(H1N1)
NA	CY050152	H1N1	05/28/2009	USA	A/Wisconsin/629-D00117/2009(H1N1)
NA	CY053311	H1N1	05/28/2009	USA	A/Brownsville/37OS/2009(H1N1)
NA	CY053263	H1N1	05/28/2009	USA	A/Brownsville/37H/2009(H1N1)
NA	CY045007	H1N1	05/28/2009	USA	A/New York/3709/2009(H1N1)
NA	GQ402244	H1N1	05/28/2009	Canada	A/Canada-MB/RV2013/2009(H1N1)
NA	HM567610	H1N1	05/28/2009	United Ki	A/England/328/2009
NA	CY044222	H1N1	05/28/2009	Taiwan	A/Taiwan/T1773/2009(H1N1)
NA	CY046797	H1N1	05/28/2009	USA	A/Wisconsin/629-D01026/2009(H1N1)
NA	CY046829	H1N1	05/28/2009	USA	A/Wisconsin/629-D02144/2009(H1N1)
NA	HQ011420	H1N1	05/28/2009	China	A/Guangdong/45/2009(H1N1)
NA	CY064793	H1N1	05/28/2009	China	A/Guangdong/SB1/2009(H1N1)
NA	CY075157	H1N1	05/28/2009	Chile	A/Chile/158/2009(H1N1)
NA	CY123460	H1N1	05/28/2009	Singapore	A/Singapore/ON129/2009
NA	GQ387438	H1N1	05/28/2009	Spain	A/Catalonia/317/2009(H1N1)
NA	HQ239891	H1N1	05/28/2009	Canada	A/Canada-MB/RV2041/2009
NA	HQ239865	H1N1	05/28/2009	Canada	A/Canada-MB/RV2010/2009
NA	CY049070	H1N1	05/28/2009	Singapore	A/Singapore/ON129/2009(H1N1)
NA	GQ368666	H1N1	05/28/2009	Brazil	A/Mato Grosso/2329/2009
NA	CY075165	H1N1	05/29/2009	Chile	A/Chile/180/2009(H1N1)
NA	GQ365366	H1N1	05/29/2009	Sweden	A/Stockholm/36/2009(H1N1)
NA	CY050921	H1N1	05/29/2009	USA	A/Wisconsin/629-D02213/2009(H1N1)
NA	HM567634	H1N1	05/29/2009	United Ki	A/England/342/2009
NA	GQ223436	H1N1	05/29/2009	China	A/Guangdong/05/2009(H1N1)
NA	CY051257	H1N1	05/29/2009	USA	A/Wisconsin/629-S0086/2009(H1N1)
NA	GQ402245	H1N1	05/29/2009	Canada	A/Canada-MB/RV2018/2009(H1N1)
NA	CY071033	H1N1	05/29/2009	USA	A/New York/NHRC0002/2009(H1N1)
NA	HM567842	H1N1	05/29/2009	Ireland	A/Ireland/4/2009

NA	GQ359766	H1N1	05/29/2009	Sweden	A/Stockholm/31/2009(H1N1)
NA	CY046947	H1N1	05/29/2009	Norway	A/Norway/1613/2009(H1N1)
NA	HM780472	H1N1	05/29/2009	China	A/Guangdong/06/2009(H1N1)
NA	CY044082	H1N1	05/29/2009	USA	A/New York/3715/2009(H1N1)
NA	CY050937	H1N1	05/29/2009	USA	A/Wisconsin/629-D01847/2009(H1N1)
NA	GQ281078	H1N1	05/29/2009	Ireland	A/Dublin/04/2009(H1N1)
NA	GQ243758	H1N1	05/29/2009	Australia	A/Darwin/2001/2009(H1N1)
NA	CY052072	H1N1	05/29/2009	Italy	A/Italy/213/2009(H1N1)
NA	GQ250162	H1N1	05/29/2009	China	A/Guangdong/03/2009(H1N1)
NA	HQ239915	H1N1	05/29/2009	Canada	A/Canada-MB/RV2069/2009
NA	CY050929	H1N1	05/29/2009	USA	A/Wisconsin/629-D00426/2009(H1N1)
NA	CY044230	H1N1	05/30/2009	Taiwan	A/Taiwan/T1821/2009(H1N1)
NA	HM567626	H1N1	05/30/2009	United Ki	A/England/341/2009
NA	HM567682	H1N1	05/30/2009	United Ki	A/England/349/2009
NA	HM567666	H1N1	05/30/2009	United Ki	A/England/347/2009
NA	CY053271	H1N1	05/30/2009	USA	A/Brownsville/390S/2009(H1N1)
NA	CY053279	H1N1	05/30/2009	USA	A/Brownsville/39H/2009(H1N1)
NA	HM567642	H1N1	05/30/2009	United Ki	A/England/343/2009
NA	HM569661	H1N1	05/30/2009	Argentina	A/Argentina/07-09GP/2009(H1N1)
NA	CY055305	H1N1	05/30/2009	Singapore	A/Singapore/GN285/2009(H1N1)
NA	HM567658	H1N1	05/30/2009	United Ki	A/England/346/2009
NA	HM567674	H1N1	05/30/2009	United Ki	A/England/348/2009
NA	HM567618	H1N1	05/30/2009	United Ki	A/England/337/2009
NA	CY045964	H1N1	05/30/2009	Canada	A/Toronto/T5294/2009(H1N1)
NA	CY089882	H1N1	05/30/2009	USA	A/Wisconsin/629-D01829/2009(H1N1)
NA	CY046821	H1N1	05/30/2009	USA	A/Wisconsin/629-D02013/2009(H1N1)
NA	CY046805	H1N1	05/30/2009	USA	A/Wisconsin/629-D00223/2009(H1N1)
NA	HM567650	H1N1	05/30/2009	United Ki	A/England/345/2009
NA	GQ402246	H1N1	05/30/2009	Canada	A/Canada-MB/RV2020/2009(H1N1)
NA	GQ360064	H1N1	05/30/2009	Sweden	A/Stockholm/32/2009(H1N1)
NA	GQ360062	H1N1	05/30/2009	Sweden	A/Stockholm/33/2009(H1N1)
NA	HQ239889	H1N1	05/30/2009	Canada	A/Canada-MB/RV2040/2009
NA	HQ239906	H1N1	05/30/2009	Canada	A/Canada-MB/RV2054/2009
NA	HE804107	H1N1	05/30/2009	Uruguay	A/Uruguay/61/2009
NA	CY123466	H1N1	05/30/2009	Singapore	A/Singapore/ON132/2009
NA	CY052073	H1N1	05/30/2009	Italy	A/Italy/214/2009(H1N1)
NA	CY052074	H1N1	05/30/2009	Italy	A/Italy/215/2009(H1N1)
NA	CY046950	H1N1	05/30/2009	Norway	A/Norway/1623/2009(H1N1)
NA	HQ239901	H1N1	05/30/2009	Canada	A/Canada-MB/RV2046/2009
NA	HQ239907	H1N1	05/30/2009	Canada	A/Canada-MB/RV2055/2009
NA	GQ253504	H1N1	05/31/2009	China	A/Shanghai/71T/2009(H1N1)
NA	GQ281079	H1N1	05/31/2009	Ireland	A/Dublin/05/2009(H1N1)
NA	HM567698	H1N1	05/31/2009	United Ki	A/England/351/2009
NA	HM567850	H1N1	05/31/2009	Ireland	A/Ireland/5/2009
NA	CY071025	H1N1	05/31/2009	USA	A/New York/NHRC0001/2009(H1N1)
NA	HQ239917	H1N1	05/31/2009	Canada	A/Canada-MB/RV2076p/2009
NA	HM567714	H1N1	05/31/2009	United Ki	A/Scotland/8/2009
NA	HQ239913	H1N1	05/31/2009	Canada	A/Canada-MB/RV2066p/2009
NA	HQ239903	H1N1	05/31/2009	Canada	A/Canada-MB/RV2049/2009
NA	HQ239916	H1N1	05/31/2009	Canada	A/Canada-MB/RV2076i/2009
NA	HM567738	H1N1	05/31/2009	United Ki	A/England/366/2009
NA	HQ239912	H1N1	05/31/2009	Canada	A/Canada-MB/RV2066i/2009
NA	HM567730	H1N1	05/31/2009	United Ki	A/England/365/2009
NA	HQ239905	H1N1	05/31/2009	Canada	A/Canada-MB/RV2053/2009

NA	CY044863	H1N1	05/31/2009	USA	A/New York/3726/2009(H1N1)
NA	GQ377105	H1N1	05/31/2009	USA	A/Utah/04/2009(H1N1)
NA	CY065536	H1N1	05/31/2009	United Ki	A/England/359/2009
NA	HM567722	H1N1	05/31/2009	United Ki	A/England/364/2009
NA	CY045185	H1N1	05/31/2009	USA	A/New York/3960/2009(H1N1)
NA	CY045031	H1N1	05/31/2009	USA	A/New York/3812/2009(H1N1)
NA	HE804103	H1N1	05/31/2009	Uruguay	A/Uruguay/70/2009
NA	GQ293078	H1N1	05/31/2009	China	A/Zhejiang/2/2009(H1N1)
NA	JN381348	H1N1	06/2009	Taiwan	A/Taiwan/1775/2009
NA	JN381346	H1N1	06/2009	Taiwan	A/Taiwan/1767/2009
NA	JN381351	H1N1	06/2009	Taiwan	A/Taiwan/97264/2009
NA	JN381343	H1N1	06/2009	Taiwan	A/Taiwan/1753/2009
NA	JN381344	H1N1	06/2009	Taiwan	A/Taiwan/1765/2009
NA	JN381349	H1N1	06/2009	Taiwan	A/Taiwan/1776/2009
NA	JN381352	H1N1	06/2009	Taiwan	A/Taiwan/97291/2009
NA	JN381345	H1N1	06/2009	Taiwan	A/Taiwan/1766/2009
NA	JN381350	H1N1	06/2009	Taiwan	A/Taiwan/97196/2009
NA	CY045236	H1N1	06/2009	Taiwan	A/Taiwan/126/2009
NA	CY045244	H1N1	06/2009	Taiwan	A/Taiwan/137/2009
NA	JN381347	H1N1	06/2009	Taiwan	A/Taiwan/1768/2009
NA	CY047789	H1N1	06/2009	Argentina	A/Argentina/7785/2009
NA	CY047769	H1N1	06/2009	Argentina	A/Argentina/14332/2009
NA	JF429403	H1N1	06/2009	France	A/Lyon/969/2009
NA	CY045228	H1N1	06/2009	Taiwan	A/Taiwan/115/2009
NA	HQ104927	H1N1	06/2009	Germany	A/Hamburg/NY1580/2009
NA	CY069643	H1N1	06/2009	Singapore	A/Singapore/527/2009(H1N1)
NA	CY069636	H1N1	06/2009	Singapore	A/Singapore/478/2009(H1N1)
NA	CY069628	H1N1	06/2009	Singapore	A/Singapore/471/2009(H1N1)
NA	CY069621	H1N1	06/2009	Singapore	A/Singapore/276/2009(H1N1)
NA	HQ695922	H1N1	06/2009	Taiwan	A/Taiwan/14/2009
NA	GQ359760	H1N1	06/2009	China	A/Zhejiang/DTID-ZJU01/2009(H1N1)
NA	CY047775	H1N1	06/2009	Argentina	A/Argentina/18426/2009
NA	GU292381	H1N1	06/2009	India	A/Blore/NIV236/2009(H1N1)
NA	CY073794	H1N1	06/2009	USA	A/San Francisco/01/2009(H1N1)
NA	HM598318	H1N1	06/2009	Thailand	A/Thailand/CU-MV5/2009
NA	HM598338	H1N1	06/2009	Thailand	A/Thailand/CU-MV31/2009
NA	HM598339	H1N1	06/2009	Thailand	A/Thailand/CU-MV32/2009
NA	CY087291	H1N1	06/2009	Hong Kong	A/Hong Kong/33230/2009
NA	CY064370	H1N1	06/2009	China	A/Guangdong/860/2009
NA	CY064358	H1N1	06/2009	China	A/Guangdong/486/2009
NA	CY064367	H1N1	06/2009	China	A/Guangdong/824/2009
NA	CY064364	H1N1	06/2009	China	A/Guangdong/499/2009
NA	HM598316	H1N1	06/2009	Thailand	A/Thailand/CU-MV3/2009
NA	HM598317	H1N1	06/2009	Thailand	A/Thailand/CU-MV4/2009
NA	HM598341	H1N1	06/2009	Thailand	A/Thailand/CU-MV34/2009
NA	HM598342	H1N1	06/2009	Thailand	A/Thailand/CU-MV35/2009
NA	HM598314	H1N1	06/2009	Thailand	A/Thailand/CU-MV1/2009
NA	HM598315	H1N1	06/2009	Thailand	A/Thailand/CU-MV2/2009
NA	AB568164	H1N1	06/2009	Japan	A/Kobe/91524/2009
NA	AB568159	H1N1	06/2009	Japan	A/Kobe/91508/2009
NA	HM598340	H1N1	06/2009	Thailand	A/Thailand/CU-MV33/2009
NA	CY064355	H1N1	06/2009	China	A/Guangdong/475/2009
NA	CY054693	H1N1	06/01/2009	Nicaragua	A/Managua/4467.05/2009(H1N1)
NA	GQ338384	H1N1	06/01/2009	USA	A/Rhode Island/04/2009(H1N1)

NA	CY056389	H1N1	06/01/2009	Nicaragua	A/Managua/3119.01/2009(H1N1)
NA	CY065560	H1N1	06/01/2009	United Ki	A/England/382/2009
NA	CY065648	H1N1	06/01/2009	United Ki	A/England/402/2009
NA	CY050953	H1N1	06/01/2009	USA	A/Wisconsin/629-D00724/2009(H1N1)
NA	CY050961	H1N1	06/01/2009	USA	A/Wisconsin/629-D00941/2009(H1N1)
NA	HM567746	H1N1	06/01/2009	United Ki	A/England/374/2009
NA	CY053287	H1N1	06/01/2009	USA	A/Brownsville/400S/2009(H1N1)
NA	CY065584	H1N1	06/01/2009	United Ki	A/England/386/2009
NA	CY065624	H1N1	06/01/2009	United Ki	A/England/395/2009
NA	CY065568	H1N1	06/01/2009	United Ki	A/England/384/2009
NA	CY069887	H1N1	06/01/2009	United Ki	A/England/383/2009
NA	HM567818	H1N1	06/01/2009	Ireland	A/Ireland/7/2009
NA	CY065576	H1N1	06/01/2009	United Ki	A/England/385/2009
NA	CY050945	H1N1	06/01/2009	USA	A/Wisconsin/629-D00374/2009(H1N1)
NA	CY050969	H1N1	06/01/2009	USA	A/Wisconsin/629-D01725/2009(H1N1)
NA	CY071041	H1N1	06/01/2009	USA	A/New York/NHRC0003/2009(H1N1)
NA	CY053911	H1N1	06/01/2009	Argentina	A/Argentina/HNRG17/2009
NA	HM567706	H1N1	06/01/2009	United Ki	A/England/360/2009
NA	CY100814	H1N1	06/01/2009	USA	A/Texas/WRAIR1118P/2009(H1N1)
NA	CY065544	H1N1	06/01/2009	United Ki	A/England/361/2009
NA	CY065552	H1N1	06/01/2009	United Ki	A/England/381/2009
NA	HM567770	H1N1	06/01/2009	United Ki	A/England/383/2009
NA	CY047376	H1N1	06/01/2009	USA	A/New York/3753/2009(H1N1)
NA	CY045177	H1N1	06/01/2009	USA	A/New York/3959/2009(H1N1)
NA	CY044991	H1N1	06/01/2009	USA	A/New York/3751/2009(H1N1)
NA	CY047368	H1N1	06/01/2009	USA	A/New York/3795/2009(H1N1)
NA	CY076765	H1N1	06/01/2009	Canada	A/Ontario/147265/2009(H1N1)
NA	CY089890	H1N1	06/01/2009	USA	A/Wisconsin/629-D00881/2009(H1N1)
NA	FN423714	H1N1	06/01/2009	Luxembour	A/Luxembourg/43/2009
NA	AB510921	H1N1	06/01/2009	Japan	A/Aichi/197/2009(H1N1)
NA	AB510920	H1N1	06/01/2009	Japan	A/Aichi/196/2009(H1N1)
NA	GQ365370	H1N1	06/01/2009	Sweden	A/Stockholm/35/2009(H1N1)
NA	GQ281080	H1N1	06/01/2009	Ireland	A/Dublin/06/2009(H1N1)
NA	GQ281081	H1N1	06/01/2009	Ireland	A/Dublin/07/2009(H1N1)
NA	AB510919	H1N1	06/01/2009	Japan	A/Aichi/195/2009(H1N1)
NA	CY052075	H1N1	06/01/2009	Italy	A/Italy/216/2009(H1N1)
NA	CY052076	H1N1	06/01/2009	Italy	A/Italy/217/2009(H1N1)
NA	HQ239904	H1N1	06/01/2009	Canada	A/Canada-MB/RV2051/2009
NA	HQ239893	H1N1	06/01/2009	Canada	A/Canada-MB/RV2042i/2009
NA	HQ239911	H1N1	06/01/2009	Canada	A/Canada-MB/RV2064/2009
NA	HQ239908	H1N1	06/01/2009	Canada	A/Canada-MB/RV2058/2009
NA	HQ239909	H1N1	06/01/2009	Canada	A/Canada-MB/RV2060/2009
NA	HQ239894	H1N1	06/01/2009	Canada	A/Canada-MB/RV2042p/2009
NA	HQ239914	H1N1	06/01/2009	Canada	A/Canada-MB/RV2067/2009
NA	GQ367490	H1N1	06/01/2009	Brazil	A/Sao Paulo/2303/2009(H1N1)
NA	KC782254	H1N1	06/01/2009	USA	A/Michigan/14/2009
NA	HM567754	H1N1	06/02/2009	United Ki	A/England/377/2009
NA	HQ239898	H1N1	06/02/2009	Canada	A/Canada-MB/RV2044/2009
NA	CY053303	H1N1	06/02/2009	USA	A/Brownsville/43H/2009(H1N1)
NA	CY051265	H1N1	06/02/2009	USA	A/Wisconsin/629-S0143/2009(H1N1)
NA	CY069903	H1N1	06/02/2009	United Ki	A/England/393/2009
NA	CY069895	H1N1	06/02/2009	United Ki	A/England/391/2009
NA	CY065616	H1N1	06/02/2009	United Ki	A/England/394/2009
NA	CY051225	H1N1	06/02/2009	USA	A/Wisconsin/629-D00022/2009(H1N1)

NA	CY050977	H1N1	06/02/2009	USA	A/Wisconsin/629-D00367/2009(H1N1)
NA	CY050985	H1N1	06/02/2009	USA	A/Wisconsin/629-D00592/2009(H1N1)
NA	CY051281	H1N1	06/02/2009	USA	A/Wisconsin/629-S0148/2009(H1N1)
NA	HM567866	H1N1	06/02/2009	United Ki	A/England/416/2009
NA	HM567778	H1N1	06/02/2009	United Ki	A/England/391/2009
NA	CY065592	H1N1	06/02/2009	United Ki	A/England/388/2009
NA	HM567762	H1N1	06/02/2009	United Ki	A/England/380/2009
NA	CY051273	H1N1	06/02/2009	USA	A/Wisconsin/629-S0146/2009(H1N1)
NA	GQ329072	H1N1	06/02/2009	France	A/Caen/2716/2009(H1N1)
NA	GQ287626	H1N1	06/02/2009	Japan	A/Tokushima/1/2009(H1N1)
NA	GQ475991	H1N1	06/02/2009	USA	A/Pennsylvania/11/2009(H1N1)
NA	CY044999	H1N1	06/02/2009	USA	A/New York/3828/2009(H1N1)
NA	CY045015	H1N1	06/02/2009	USA	A/New York/3796/2009(H1N1)
NA	CY045023	H1N1	06/02/2009	USA	A/New York/3803/2009(H1N1)
NA	GQ334348	H1N1	06/02/2009	Japan	A/Shizuoka/759/2009(H1N1)
NA	GU014777	H1N1	06/02/2009	Japan	A/Chiba-C/48/2009(H1N1)
NA	CY076773	H1N1	06/02/2009	Canada	A/Ontario/147723/2009(H1N1)
NA	CY045161	H1N1	06/02/2009	USA	A/New York/3818/2009(H1N1)
NA	CY045169	H1N1	06/02/2009	USA	A/New York/3819/2009(H1N1)
NA	HM567786	H1N1	06/02/2009	United Ki	A/England/393/2009
NA	GQ281083	H1N1	06/02/2009	Ireland	A/Dublin/09/2009(H1N1)
NA	CY052077	H1N1	06/02/2009	Italy	A/Italy/218/2009(H1N1)
NA	CY052078	H1N1	06/02/2009	Italy	A/Italy/219/2009(H1N1)
NA	CY049078	H1N1	06/02/2009	Singapore	A/Singapore/ON141/2009(H1N1)
NA	CY053295	H1N1	06/02/2009	USA	A/Brownsville/430S/2009(H1N1)
NA	CY054685	H1N1	06/03/2009	USA	A/Wisconsin/629-D00589/2009(H1N1)
NA	HQ239910	H1N1	06/03/2009	Canada	A/Canada-MB/RV2063/2009
NA	KC781438	H1N1	06/03/2009	USA	A/Utah/27/2009
NA	CY107549	H1N1	06/03/2009	United Ki	A/Scotland/Livingston_412757/2009
NA	CY107601	H1N1	06/03/2009	United Ki	A/Scotland/Glasgow_413364/2009
NA	CY107629	H1N1	06/03/2009	United Ki	A/Scotland/Paisley_413869/2009
NA	CY107594	H1N1	06/03/2009	United Ki	A/Scotland/EastKilbride_412770/2009
NA	CY107611	H1N1	06/03/2009	United Ki	A/Scotland/EastKilbride_413652/2009
NA	CY107616	H1N1	06/03/2009	United Ki	A/Scotland/Paisley_413739/2009
NA	CY107587	H1N1	06/03/2009	United Ki	A/Scotland/Paisley_412666/2009
NA	CY107623	H1N1	06/03/2009	United Ki	A/Scotland/Glasgow_413848/2009
NA	CY107753	H1N1	06/03/2009	United Ki	A/Scotland/Paisley_413960/2009
NA	CY107637	H1N1	06/03/2009	United Ki	A/Scotland/Glasgow_413886/2009
NA	CY107580	H1N1	06/03/2009	United Ki	A/Scotland/Glasgow_412313/2009
NA	CY107572	H1N1	06/03/2009	United Ki	A/Scotland/Glasgow_412311/2009
NA	CY107567	H1N1	06/03/2009	United Ki	A/Scotland/Glasgow_412286/2009
NA	CY123474	H1N1	06/03/2009	Singapore	A/Singapore/ON141/2009
NA	CY069934	H1N1	06/03/2009	United Ki	A/England/425/2009
NA	CY051233	H1N1	06/03/2009	USA	A/Wisconsin/629-D01664/2009(H1N1)
NA	CY071049	H1N1	06/03/2009	USA	A/New York/NHRC0004/2009(H1N1)
NA	HM567794	H1N1	06/03/2009	United Ki	A/England/399/2009
NA	CY065608	H1N1	06/03/2009	United Ki	A/England/392/2009
NA	HM567802	H1N1	06/03/2009	United Ki	A/England/401/2009
NA	CY065600	H1N1	06/03/2009	United Ki	A/England/389/2009
NA	CY065632	H1N1	06/03/2009	United Ki	A/England/398/2009
NA	GQ330646	H1N1	06/03/2009	Russia	A/Moscow/IIV03/2009(H1N1)
NA	GQ396735	H1N1	06/03/2009	USA	A/Michigan/10/2009(H1N1)
NA	AB514229	H1N1	06/03/2009	Japan	A/Saitama/55/2009(H1N1)
NA	CY044098	H1N1	06/03/2009	USA	A/New York/3895/2009(H1N1)

NA	CY083274	H1N1	06/03/2009	USA	A/San Diego/WRAIR1644P/2009(H1N1)
NA	CY107608	H1N1	06/03/2009	United Ki	A/Scotland/Glasgow_413608/2009
NA	GU014809	H1N1	06/03/2009	Japan	A/Yamaguchi/21/2009(H1N1)
NA	CY045497	H1N1	06/03/2009	Germany	A/Baden-Wurttemberg/448/2009(H1N1)
NA	GQ411899	H1N1	06/03/2009	Canada	A/Toronto/T5308/2009(H1N1)
NA	GQ281082	H1N1	06/03/2009	Ireland	A/Dublin/08/2009(H1N1)
NA	GQ387439	H1N1	06/03/2009	Spain	A/Catalonia/331/2009(H1N1)
NA	CY055417	H1N1	06/03/2009	USA	A/Wisconsin/629-D00809/2009
NA	KC781926	H1N1	06/03/2009	USA	A/Virginia/24/2009
NA	CY065696	H1N1	06/04/2009	United Ki	A/England/428/2009
NA	CY043344	H1N1	06/04/2009	Denmark	A/Denmark/524/2009(H1N1)
NA	CY051025	H1N1	06/04/2009	USA	A/Wisconsin/629-D00017/2009(H1N1)
NA	CY051017	H1N1	06/04/2009	USA	A/Wisconsin/629-D00453/2009(H1N1)
NA	CY069918	H1N1	06/04/2009	United Ki	A/England/413/2009
NA	CY069911	H1N1	06/04/2009	United Ki	A/England/412/2009
NA	CY051033	H1N1	06/04/2009	USA	A/Wisconsin/629-D00658/2009(H1N1)
NA	CY051041	H1N1	06/04/2009	USA	A/Wisconsin/629-D00725/2009(H1N1)
NA	CY051049	H1N1	06/04/2009	USA	A/Wisconsin/629-D00008/2009(H1N1)
NA	CY051289	H1N1	06/04/2009	USA	A/Wisconsin/629-S0173/2009(H1N1)
NA	CY083266	H1N1	06/04/2009	USA	A/San Diego/WRAIR1643P/2009(H1N1)
NA	HM567858	H1N1	06/04/2009	United Ki	A/England/415/2009
NA	CY065664	H1N1	06/04/2009	United Ki	A/England/414/2009
NA	CY065688	H1N1	06/04/2009	United Ki	A/England/423/2009
NA	CY076781	H1N1	06/04/2009	Canada	A/Ontario/152439/2009(H1N1)
NA	CY076789	H1N1	06/04/2009	Canada	A/Ontario/152846/2009(H1N1)
NA	CY089898	H1N1	06/04/2009	USA	A/Wisconsin/629-D01420/2009(H1N1)
NA	HM567882	H1N1	06/04/2009	United Ki	A/England/424/2009
NA	GQ281084	H1N1	06/04/2009	Ireland	A/Dublin/10/2009(H1N1)
NA	AB520644	H1N1	06/04/2009	Japan	A/Aichi/267/2009(H1N1)
NA	HE804119	H1N1	06/04/2009	Uruguay	A/Uruguay/123/2009
NA	HQ239928	H1N1	06/04/2009	Canada	A/Canada-MB/RV2152/2009
NA	CY043336	H1N1	06/04/2009	Denmark	A/Denmark/523/2009(H1N1)
NA	CY065656	H1N1	06/04/2009	United Ki	A/England/411/2009
NA	CY058166	H1N1	06/05/2009	Nicaragua	A/Managua/4016.02/2009(H1N1)
NA	HQ239920	H1N1	06/05/2009	Canada	A/Canada-MB/RV2107/2009
NA	CY050993	H1N1	06/05/2009	USA	A/Wisconsin/629-D01903/2009(H1N1)
NA	CY052140	H1N1	06/05/2009	USA	A/New York/3980/2009(H1N1)
NA	CY069926	H1N1	06/05/2009	United Ki	A/England/417/2009
NA	HM567922	H1N1	06/05/2009	United Ki	A/England/443/2009
NA	CY065712	H1N1	06/05/2009	United Ki	A/England/431/2009
NA	CY065672	H1N1	06/05/2009	United Ki	A/England/418/2009
NA	CY072968	H1N1	06/05/2009	Nicaragua	A/Managua/2750.01/2009(H1N1)
NA	HM567826	H1N1	06/05/2009	Ireland	A/Ireland/10/2009
NA	CY065720	H1N1	06/05/2009	United Ki	A/England/433/2009
NA	HM567898	H1N1	06/05/2009	United Ki	A/England/432/2009
NA	HM567914	H1N1	06/05/2009	United Ki	A/England/436/2009
NA	CY065680	H1N1	06/05/2009	United Ki	A/England/419/2009
NA	CY065640	H1N1	06/05/2009	United Ki	A/England/400/2009
NA	GQ396731	H1N1	06/05/2009	USA	A/Utah/10/2009(H1N1)
NA	CY065704	H1N1	06/05/2009	United Ki	A/England/430/2009
NA	CY044106	H1N1	06/05/2009	USA	A/New York/3898/2009(H1N1)
NA	CY053898	H1N1	06/05/2009	Argentina	A/Argentina/HNRG13/2009(H1N1)
NA	GQ368668	H1N1	06/05/2009	Brazil	A/Distrito Federal/2611/2009(H1N1)
NA	CY072912	H1N1	06/05/2009	Nicaragua	A/Managua/2652.01/2009(H1N1)

NA	CY061310	H1N1	06/05/2009	Argentina	A/Argentina/HNRG1004/2009
NA	CY061308	H1N1	06/05/2009	Argentina	A/Argentina/HNRG1002/2009
NA	CY061309	H1N1	06/05/2009	Argentina	A/Argentina/HNRG1003/2009
NA	CY052079	H1N1	06/05/2009	Italy	A/Italy/220/2009(H1N1)
NA	CY052080	H1N1	06/05/2009	Italy	A/Italy/221/2009(H1N1)
NA	HM567546	H1N1	06/05/2009	United Ki	A/England/456/2009
NA	CY058526	H1N1	06/05/2009	Nicaragua	A/Managua/4905.02/2009(H1N1)
NA	JX625984	H1N1	06/06/2009	United Ki	A/England/421/2009
NA	CY058086	H1N1	06/06/2009	Nicaragua	A/Managua/1455.01/2009(H1N1)
NA	HM567874	H1N1	06/06/2009	United Ki	A/England/421/2009
NA	CY083601	H1N1	06/06/2009	Nicaragua	A/Managua/WRAIR8964F/2009(H1N1)
NA	CY083258	H1N1	06/06/2009	USA	A/San Diego/WRAIR1642P/2009(H1N1)
NA	GQ411905	H1N1	06/06/2009	China	A/Shanghai/143T/2009(H1N1)
NA	GQ365363	H1N1	06/06/2009	Sweden	A/Stockholm/42/2009(H1N1)
NA	GQ287624	H1N1	06/06/2009	Japan	A/Shiga/3/2009(H1N1)
NA	CY083473	H1N1	06/06/2009	Nicaragua	A/Managua/WRAIR1689P/2009(H1N1)
NA	GQ369275	H1N1	06/06/2009	Sweden	A/Stockholm/37/2009(H1N1)
NA	CY065728	H1N1	06/06/2009	United Ki	A/England/434/2009
NA	HM567906	H1N1	06/06/2009	United Ki	A/England/435/2009
NA	GQ351320	H1N1	06/06/2009	Italy	A/Firenze/10/2009(H1N1)
NA	GU012634	H1N1	06/06/2009	Poland	A/Poland/192/2009(H1N1)
NA	CY058078	H1N1	06/07/2009	Nicaragua	A/Managua/171.01/2009(H1N1)
NA	HM567938	H1N1	06/07/2009	United Ki	A/England/450/2009
NA	CY069942	H1N1	06/07/2009	United Ki	A/England/438/2009
NA	CY074597	H1N1	06/07/2009	USA	A/California/VRDL358/2009(H1N1)
NA	CY089914	H1N1	06/07/2009	USA	A/Wisconsin/629-D00112/2009(H1N1)
NA	HM567946	H1N1	06/07/2009	United Ki	A/England/451/2009
NA	GQ334340	H1N1	06/07/2009	Japan	A/Fukuoka-C/3/2009(H1N1)
NA	GQ287620	H1N1	06/07/2009	Japan	A/Fukuoka-C/1/2009(H1N1)
NA	GQ334332	H1N1	06/07/2009	Japan	A/Fukuoka-C/2/2009(H1N1)
NA	CY053121	H1N1	06/07/2009	USA	A/Houston/170S/2009(H1N1)
NA	KC781796	H1N1	06/08/2009	USA	A/Wisconsin/26/2009
NA	CY058134	H1N1	06/08/2009	Nicaragua	A/Managua/516.01/2009(H1N1)
NA	CY052132	H1N1	06/08/2009	USA	A/New York/3976/2009(H1N1)
NA	CY051329	H1N1	06/08/2009	USA	A/Wisconsin/629-S0203/2009(H1N1)
NA	CY051065	H1N1	06/08/2009	USA	A/Wisconsin/629-D00101/2009(H1N1)
NA	CY051073	H1N1	06/08/2009	USA	A/Wisconsin/629-D00579/2009(H1N1)
NA	CY051305	H1N1	06/08/2009	USA	A/Wisconsin/629-S0197/2009(H1N1)
NA	CY051321	H1N1	06/08/2009	USA	A/Wisconsin/629-S0202/2009(H1N1)
NA	CY051497	H1N1	06/08/2009	USA	A/Wisconsin/629-S0189/2009(H1N1)
NA	CY051297	H1N1	06/08/2009	USA	A/Wisconsin/629-S0186/2009(H1N1)
NA	CY051313	H1N1	06/08/2009	USA	A/Wisconsin/629-S0201/2009(H1N1)
NA	CY083154	H1N1	06/08/2009	Dominican	A/Santo Domingo/WRAIR1044N/2009
NA	HM567890	H1N1	06/08/2009	United Ki	A/England/429/2009
NA	CY051057	H1N1	06/08/2009	USA	A/Wisconsin/629-D02068/2009(H1N1)
NA	CY051505	H1N1	06/08/2009	USA	A/Wisconsin/629-S0206/2009(H1N1)
NA	GU012635	H1N1	06/08/2009	Poland	A/Poland/193/2009(H1N1)
NA	CY076797	H1N1	06/08/2009	Canada	A/Ontario/156785/2009(H1N1)
NA	CY045209	H1N1	06/08/2009	USA	A/New York/4057/2009(H1N1)
NA	CY071548	H1N1	06/08/2009	USA	A/California/WR1323P/2009(H1N1)
NA	GU049677	H1N1	06/08/2009	West Bank	A/Bethlehem/CBH-70G6/2009(H1N1)
NA	CY089986	H1N1	06/08/2009	USA	A/Wisconsin/629-D01822/2009(H1N1)
NA	CY061311	H1N1	06/08/2009	Argentina	A/Argentina/HNRG1005/2009
NA	CY052081	H1N1	06/08/2009	Italy	A/Italy/222/2009(H1N1)

NA	CY052082	H1N1	06/08/2009	Italy	A/Italy/223/2009(H1N1)
NA	HQ239879	H1N1	06/08/2009	Canada	A/Canada-MB/RV2029-10/2009
NA	HQ239918	H1N1	06/08/2009	Canada	A/Canada-MB/RV2103/2009
NA	HQ239921	H1N1	06/08/2009	Canada	A/Canada-MB/RV2110/2009
NA	HQ239923	H1N1	06/08/2009	Canada	A/Canada-MB/RV2123/2009
NA	HQ239877	H1N1	06/08/2009	Canada	A/Canada-MB/RV2027-10/2009
NA	HQ239876	H1N1	06/08/2009	Canada	A/Canada-MB/RV2026-10/2009
NA	HQ239878	H1N1	06/08/2009	Canada	A/Canada-MB/RV2028-10/2009
NA	GQ365685	H1N1	06/08/2009	Sweden	A/Stockholm/44/2009(H1N1)
NA	CY069950	H1N1	06/09/2009	United Ki	A/England/444/2009
NA	GQ367304	H1N1	06/09/2009	Sweden	A/Stockholm/43/2009(H1N1)
NA	CY095849	H1N1	06/09/2009	China	A/Shenzhen/71/2009
NA	CY051513	H1N1	06/09/2009	USA	A/Wisconsin/629-S0208/2009(H1N1)
NA	CY058102	H1N1	06/09/2009	Nicaragua	A/Managua/168.02/2009(H1N1)
NA	CY065736	H1N1	06/09/2009	United Ki	A/England/445/2009
NA	CY049885	H1N1	06/09/2009	El Salvac	A/San Salvador/WR0167N/2009(H1N1)
NA	CY095794	H1N1	06/09/2009	China	A/Shenzhen/42/2009
NA	CY095787	H1N1	06/09/2009	China	A/Shenzhen/40/2009
NA	CY095833	H1N1	06/09/2009	China	A/Shenzhen/63/2009
NA	CY095744	H1N1	06/09/2009	China	A/Shenzhen/17/2009
NA	CY095822	H1N1	06/09/2009	China	A/Shenzhen/51/2009
NA	CY095830	H1N1	06/09/2009	China	A/Shenzhen/62/2009
NA	CY095802	H1N1	06/09/2009	China	A/Shenzhen/45/2009
NA	CY095758	H1N1	06/09/2009	China	A/Shenzhen/25/2009
NA	CY095753	H1N1	06/09/2009	China	A/Shenzhen/22/2009
NA	CY095772	H1N1	06/09/2009	China	A/Shenzhen/37/2009
NA	CY095769	H1N1	06/09/2009	China	A/Shenzhen/32/2009
NA	CY052124	H1N1	06/09/2009	USA	A/New York/3969/2009(H1N1)
NA	CY051337	H1N1	06/09/2009	USA	A/Wisconsin/629-S0220/2009(H1N1)
NA	CY051345	H1N1	06/09/2009	USA	A/Wisconsin/629-S0222/2009(H1N1)
NA	CY051353	H1N1	06/09/2009	USA	A/Wisconsin/629-S0226/2009(H1N1)
NA	CY095798	H1N1	06/09/2009	China	A/Shenzhen/44/2009
NA	CY095806	H1N1	06/09/2009	China	A/Shenzhen/47/2009
NA	KF849742	H1N1	06/09/2009	Thailand	A/Thailand/SirirajICRC_CBI_8/2009
NA	KF849743	H1N1	06/09/2009	Thailand	A/Thailand/SirirajICRC_CBI_9/2009
NA	CY095836	H1N1	06/09/2009	China	A/Shenzhen/67/2009
NA	CY051241	H1N1	06/09/2009	USA	A/Wisconsin/629-D01199/2009(H1N1)
NA	HM567930	H1N1	06/09/2009	United Ki	A/England/446/2009
NA	CY043352	H1N1	06/09/2009	Denmark	A/Denmark/528/2009(H1N1)
NA	GQ365680	H1N1	06/09/2009	Sweden	A/Stockholm/38/2009(H1N1)
NA	GQ365682	H1N1	06/09/2009	Sweden	A/Stockholm/40/2009(H1N1)
NA	GQ287628	H1N1	06/09/2009	Japan	A/Yokohama/1/2009(H1N1)
NA	CY045039	H1N1	06/09/2009	USA	A/New York/3967/2009(H1N1)
NA	CY045055	H1N1	06/09/2009	USA	A/New York/4052/2009(H1N1)
NA	GQ365427	H1N1	06/09/2009	Japan	A/Iwate/1/2009(H1N1)
NA	GU012636	H1N1	06/09/2009	Poland	A/Poland/195/2009(H1N1)
NA	CY045972	H1N1	06/09/2009	Canada	A/Toronto/T5362/2009(H1N1)
NA	CY095825	H1N1	06/09/2009	China	A/Shenzhen/54/2009
NA	GQ365688	H1N1	06/09/2009	Sweden	A/Stockholm/39/2009(H1N1)
NA	CY061312	H1N1	06/09/2009	Argentina	A/Argentina/HNRG1006/2009
NA	CY052083	H1N1	06/09/2009	Italy	A/Italy/224/2009(H1N1)
NA	CY095810	H1N1	06/09/2009	China	A/Shenzhen/48/2009
NA	HQ239881	H1N1	06/09/2009	Canada	A/Canada-MB/RV2031-10/2009
NA	HQ239925	H1N1	06/09/2009	Canada	A/Canada-MB/RV2138/2009

NA	HQ239919	H1N1	06/09/2009	Canada	A/Canada-MB/RV2105/2009
NA	CY052292	H1N1	06/09/2009	USA	A/Texas/42091791/2009(H1N1)
NA	CY045217	H1N1	06/10/2009	USA	A/New York/4074/2009(H1N1)
NA	CY045201	H1N1	06/10/2009	USA	A/New York/4048/2009(H1N1)
NA	CY071452	H1N1	06/10/2009	Dominican	A/Santo Domingo/WR1072T/2009(H1N1)
NA	CY069311	H1N1	06/10/2009	Colombia	A/Bogota/WRAIR0088N/2009(H1N1)
NA	CY045193	H1N1	06/10/2009	USA	A/New York/4014/2009(H1N1)
NA	CY073344	H1N1	06/10/2009	USA	A/San Diego/WR1628P/2009(H1N1)
NA	HQ247611	H1N1	06/10/2009	Finland	A/Finland/572/2009
NA	CY044114	H1N1	06/10/2009	USA	A/New York/3992/2009(H1N1)
NA	CY051393	H1N1	06/10/2009	USA	A/Wisconsin/629-S0255/2009(H1N1)
NA	CY051385	H1N1	06/10/2009	USA	A/Wisconsin/629-S0254/2009(H1N1)
NA	CY045047	H1N1	06/10/2009	USA	A/New York/4006/2009(H1N1)
NA	CY051401	H1N1	06/10/2009	USA	A/Wisconsin/629-S0252/2009(H1N1)
NA	CY107656	H1N1	06/10/2009	United Ki	A/Scotland/Glasgow_414473/2009
NA	HQ239922	H1N1	06/10/2009	Canada	A/Canada-MB/RV2121/2009
NA	HQ239926	H1N1	06/10/2009	Canada	A/Canada-MB/RV2146/2009
NA	HQ239927	H1N1	06/10/2009	Canada	A/Canada-MB/RV2147/2009
NA	HQ239924	H1N1	06/10/2009	Canada	A/Canada-MB/RV2132/2009
NA	HQ239885	H1N1	06/10/2009	Canada	A/Canada-MB/RV2036-10/2009
NA	HQ239884	H1N1	06/10/2009	Canada	A/Canada-MB/RV2034-10/2009
NA	HQ239882	H1N1	06/10/2009	Canada	A/Canada-MB/RV2032-10/2009
NA	CY090002	H1N1	06/10/2009	USA	A/Wisconsin/629-S0235/2009(H1N1)
NA	CY061313	H1N1	06/10/2009	Argentina	A/Argentina/HNRG1007/2009
NA	CY051369	H1N1	06/10/2009	USA	A/Wisconsin/629-S0248/2009(H1N1)
NA	CY051377	H1N1	06/10/2009	USA	A/Wisconsin/629-S0247/2009(H1N1)
NA	CY051361	H1N1	06/10/2009	USA	A/Wisconsin/629-S0245/2009(H1N1)
NA	CY107650	H1N1	06/10/2009	United Ki	A/Scotland/Glasgow_414159/2009
NA	CY107553	H1N1	06/10/2009	United Ki	A/Scotland/Livingston_416138/2009
NA	CY107644	H1N1	06/10/2009	United Ki	A/Scotland/Glasgow_413941/2009
NA	CY107740	H1N1	06/10/2009	United Ki	A/Scotland/Aberdeen_416064/2009
NA	CY107759	H1N1	06/10/2009	United Ki	A/Scotland/Paisley_415110/2009
NA	CY107474	H1N1	06/10/2009	United Ki	A/Scotland/Edinburgh_17343/2009
NA	CY052268	H1N1	06/10/2009	USA	A/Texas/42102708/2009(H1N1)
NA	CY052300	H1N1	06/10/2009	USA	A/Texas/42103399/2009(H1N1)
NA	KC780422	H1N1	06/10/2009	USA	A/Minnesota/22/2009
NA	GQ369279	H1N1	06/11/2009	Sweden	A/Stockholm/41/2009(H1N1)
NA	CY051081	H1N1	06/11/2009	USA	A/Wisconsin/629-D01326/2009(H1N1)
NA	CY051881	H1N1	06/11/2009	USA	A/Texas/42113095/2009(H1N1)
NA	CY089994	H1N1	06/11/2009	USA	A/Wisconsin/629-D01183/2009(H1N1)
NA	HM569708	H1N1	06/11/2009	Argentina	A/Argentina/19464/2009(H1N1)
NA	CY057208	H1N1	06/11/2009	Nicaragua	A/Managua/167.01/2009(H1N1)
NA	CY063488	H1N1	06/11/2009	USA	A/Boston/110/2009(H1N1)
NA	HQ239883	H1N1	06/11/2009	Canada	A/Canada-MB/RV2033-10/2009
NA	GQ365412	H1N1	06/11/2009	Japan	A/Akita/1/2009(H1N1)
NA	HQ239880	H1N1	06/11/2009	Canada	A/Canada-MB/RV2030-10/2009
NA	CY053975	H1N1	06/11/2009	Argentina	A/Argentina/HNRG8/2009
NA	GQ365448	H1N1	06/11/2009	Japan	A/Sapporo/1/2009(H1N1)
NA	CY051409	H1N1	06/11/2009	USA	A/Wisconsin/629-S0269/2009(H1N1)
NA	CY065744	H1N1	06/11/2009	United Ki	A/Scotland/10/2009
NA	HQ247607	H1N1	06/11/2009	Finland	A/Finland/568/2009
NA	CY053113	H1N1	06/11/2009	USA	A/Houston/160S/2009(H1N1)
NA	JX556111	H1N1	06/11/2009	Cuba	A/Habana/289/2009
NA	GQ351316	H1N1	06/11/2009	Hong Kong	A/Hong Kong/2369/2009(H1N1)

NA	CY051889	H1N1	06/11/2009	USA	A/Texas/42114261/2009(H1N1)
NA	CY052196	H1N1	06/12/2009	USA	A/Texas/42121926/2009(H1N1)
NA	CY073320	H1N1	06/12/2009	USA	A/San Diego/WR1625P/2009(H1N1)
NA	CY073328	H1N1	06/12/2009	USA	A/San Diego/WR1626P/2009(H1N1)
NA	HM569685	H1N1	06/12/2009	Argentina	A/Argentina/19618/2009(H1N1)
NA	HQ247605	H1N1	06/12/2009	Finland	A/Finland/566/2009
NA	HQ247604	H1N1	06/12/2009	Finland	A/Finland/565/2009
NA	CY051113	H1N1	06/12/2009	USA	A/Wisconsin/629-D02263/2009(H1N1)
NA	CY050160	H1N1	06/12/2009	USA	A/New York/4095/2009(H1N1)
NA	CY049893	H1N1	06/12/2009	El Salvac	A/San Salvador/0169T/2009(H1N1)
NA	CY051841	H1N1	06/12/2009	USA	A/Texas/42123701/2009(H1N1)
NA	CY051857	H1N1	06/12/2009	USA	A/Texas/42122969/2009(H1N1)
NA	CY058214	H1N1	06/12/2009	Nicaragua	A/Managua/578.01/2009(H1N1)
NA	KT181405	H1N1	06/12/2009	Singapore	A/Singapore/DMS1/2009
NA	CY058094	H1N1	06/12/2009	Nicaragua	A/Managua/655.02/2009(H1N1)
NA	CY061314	H1N1	06/12/2009	Argentina	A/Argentina/HNRG1008/2009
NA	CY061315	H1N1	06/12/2009	Argentina	A/Argentina/HNRG1009/2009
NA	CY061316	H1N1	06/12/2009	Argentina	A/Argentina/HNRG1010/2009
NA	CY052084	H1N1	06/12/2009	Italy	A/Italy/225/2009(H1N1)
NA	HQ239951	H1N1	06/12/2009	Canada	A/Canada-NFL/RV2513/2009
NA	CY045079	H1N1	06/12/2009	USA	A/New York/4072/2009(H1N1)
NA	GU014793	H1N1	06/12/2009	Japan	A/Kanagawa/137/2009(H1N1)
NA	CY123693	H1N1	06/13/2009	Singapore	A/Singapore/ON199/2009
NA	HQ247606	H1N1	06/13/2009	Finland	A/Finland/567/2009
NA	CY073312	H1N1	06/13/2009	USA	A/San Diego/WR1624P/2009(H1N1)
NA	CY054749	H1N1	06/13/2009	USA	A/California/VRDL9/2009(H1N1)
NA	CY073336	H1N1	06/13/2009	USA	A/San Diego/WR1627P/2009(H1N1)
NA	GQ866953	H1N1	06/13/2009	Thailand	A/Thailand/CU-B5/2009
NA	CY051001	H1N1	06/13/2009	USA	A/Wisconsin/629-D00665/2009(H1N1)
NA	GU014781	H1N1	06/13/2009	Japan	A/Hiroshima/200/2009(H1N1)
NA	CY047384	H1N1	06/13/2009	USA	A/New York/4100/2009(H1N1)
NA	CY051105	H1N1	06/13/2009	USA	A/Wisconsin/629-D01618/2009(H1N1)
NA	JQ409136	H1N1	06/13/2009	Finland	A/Helsinki/Vi2/2009
NA	GQ894817	H1N1	06/13/2009	USA	A/Illinois/07/2009(H1N1)
NA	CY083609	H1N1	06/13/2009	Nicaragua	A/Managua/WRAIR8997F/2009(H1N1)
NA	GQ365438	H1N1	06/13/2009	Japan	A/Kagoshima/1/2009(H1N1)
NA	GQ463202	H1N1	06/13/2009	China	A/Hunan/SWL3/2009(H1N1)
NA	CY045063	H1N1	06/13/2009	USA	A/New York/4099/2009(H1N1)
NA	CY057216	H1N1	06/13/2009	Nicaragua	A/Managua/109.01/2009(H1N1)
NA	CY058222	H1N1	06/13/2009	Nicaragua	A/Managua/5221.03/2009(H1N1)
NA	CY058230	H1N1	06/13/2009	Nicaragua	A/Managua/5363.02/2009(H1N1)
NA	CY064462	H1N1	06/13/2009	USA	A/Boston/96/2009(H1N1)
NA	CY051097	H1N1	06/13/2009	USA	A/Wisconsin/629-D00196/2009(H1N1)
NA	CY064502	H1N1	06/13/2009	USA	A/Boston/115/2009(H1N1)
NA	CY052316	H1N1	06/13/2009	USA	A/Texas/42132413/2009(H1N1)
NA	CY058174	H1N1	06/13/2009	Nicaragua	A/Managua/792.01/2009(H1N1)
NA	KC782100	H1N1	06/13/2009	USA	A/Rhode Island/15/2009
NA	KC781651	H1N1	06/13/2009	USA	A/Maryland/32/2009
NA	CY054725	H1N1	06/13/2009	USA	A/California/VRDL6/2009(H1N1)
NA	CY058110	H1N1	06/13/2009	Nicaragua	A/Managua/2152.01/2009(H1N1)
NA	CY058150	H1N1	06/13/2009	Nicaragua	A/Managua/4085.02/2009(H1N1)
NA	CY058118	H1N1	06/13/2009	Nicaragua	A/Managua/1637.01/2009(H1N1)
NA	CY075173	H1N1	06/13/2009	Chile	A/Chile/1586/2009(H1N1)
NA	CY083505	H1N1	06/13/2009	Nicaragua	A/Managua/WRAIR1693P/2009(H1N1)

NA	HQ247612	H1N1	06/13/2009	Finland	A/Finland/573/2009
NA	HQ247608	H1N1	06/13/2009	Finland	A/Finland/569/2009
NA	CY063496	H1N1	06/13/2009	USA	A/Boston/112/2009(H1N1)
NA	JQ409144	H1N1	06/13/2009	Finland	A/Helsinki/Vi3/2009(H1N1)
NA	GQ457469	H1N1	06/14/2009	USA	A/Utah/11/2009(H1N1)
NA	KJ130190	H1N1	06/14/2009	New Zeala	A/New Zealand/1212f/2009
NA	KJ130182	H1N1	06/14/2009	New Zeala	A/New Zealand/1212d/2009
NA	KJ130206	H1N1	06/14/2009	New Zeala	A/New Zealand/1212e/2009
NA	KJ130166	H1N1	06/14/2009	New Zeala	A/New Zealand/1212b/2009
NA	KJ130150	H1N1	06/14/2009	New Zeala	A/New Zealand/1212c/2009
NA	KJ130174	H1N1	06/14/2009	New Zeala	A/New Zealand/1212a/2009(H1N1)
NA	CY062523	H1N1	06/14/2009	Mexico	A/Mexico city/CIA6/2009(H1N1)
NA	CY049925	H1N1	06/14/2009	USA	A/New Bern/WR0670/2009(H1N1)
NA	GQ457464	H1N1	06/14/2009	USA	A/Utah/05/2009(H1N1)
NA	GQ351292	H1N1	06/14/2009	Italy	A/Italy/85/2009(H1N1)
NA	CY049126	H1N1	06/14/2009	Singapore	A/Singapore/ON193/2009(H1N1)
NA	JX556112	H1N1	06/14/2009	Cuba	A/Habana/353/2009
NA	CY054709	H1N1	06/14/2009	USA	A/California/VRDL4/2009(H1N1)
NA	CY052393	H1N1	06/14/2009	USA	A/Texas/42142537/2009(H1N1)
NA	CY123719	H1N1	06/14/2009	Singapore	A/Singapore/ON202/2009
NA	CY123651	H1N1	06/14/2009	Singapore	A/Singapore/ON193/2009
NA	CY051153	H1N1	06/14/2009	USA	A/Wisconsin/629-D01147/2009(H1N1)
NA	CY051129	H1N1	06/14/2009	USA	A/Wisconsin/629-D00119/2009(H1N1)
NA	CY051121	H1N1	06/14/2009	USA	A/Wisconsin/629-D00916/2009(H1N1)
NA	CY054741	H1N1	06/14/2009	USA	A/California/VRDL8/2009(H1N1)
NA	AB520649	H1N1	06/15/2009	Japan	A/Aichi/275/2009(H1N1)
NA	AB510922	H1N1	06/15/2009	Japan	A/Aichi/198/2009(H1N1)
NA	CY089922	H1N1	06/15/2009	USA	A/Wisconsin/629-D01717/2009(H1N1)
NA	GQ303552	H1N1	06/15/2009	Spain	A/Catalonia/360/2009(H1N1)
NA	CY047108	H1N1	06/15/2009	Spain	A/Catalonia/S1045/2009(H1N1)
NA	AB520648	H1N1	06/15/2009	Japan	A/Aichi/274/2009(H1N1)
NA	AB510923	H1N1	06/15/2009	Japan	A/Aichi/199/2009(H1N1)
NA	CY061320	H1N1	06/15/2009	Argentina	A/Argentina/HNRG1014/2009
NA	CY061318	H1N1	06/15/2009	Argentina	A/Argentina/HNRG1012/2009
NA	CY061317	H1N1	06/15/2009	Argentina	A/Argentina/HNRG1011/2009
NA	CY061321	H1N1	06/15/2009	Argentina	A/Argentina/HNRG1015/2009
NA	CY061319	H1N1	06/15/2009	Argentina	A/Argentina/HNRG1013/2009
NA	CY061322	H1N1	06/15/2009	Argentina	A/Argentina/HNRG1016/2009
NA	CY049134	H1N1	06/15/2009	Singapore	A/Singapore/ON226/2009(H1N1)
NA	CY089930	H1N1	06/15/2009	USA	A/Wisconsin/629-D00889/2009(H1N1)
NA	CY060752	H1N1	06/15/2009	Canada	A/Ontario/9739/2009(H1N1)
NA	HQ239947	H1N1	06/15/2009	Canada	A/Canada-NFL/RV2507/2009
NA	HQ239950	H1N1	06/15/2009	Canada	A/Canada-NFL/RV2512/2009
NA	HQ239948	H1N1	06/15/2009	Canada	A/Canada-NFL/RV2509/2009
NA	GQ328767	H1N1	06/15/2009	Greece	A/Athens/968/2009(H1N1)
NA	GQ433898	H1N1	06/15/2009	China	A/Jiangsu/1/2009(H1N1)
NA	CY063504	H1N1	06/15/2009	USA	A/Boston/113/2009(H1N1)
NA	CY060744	H1N1	06/15/2009	Canada	A/Ontario/9698/2009(H1N1)
NA	CY089719	H1N1	06/15/2009	USA	A/Boston/107/2009
NA	CY075197	H1N1	06/15/2009	Chile	A/Chile/1600/2009(H1N1)
NA	GU014799	H1N1	06/15/2009	Japan	A/Niigata/690/2009(H1N1)
NA	GU014803	H1N1	06/15/2009	Japan	A/Shizuoka/793/2009(H1N1)
NA	HM569677	H1N1	06/15/2009	Argentina	A/Argentina/19527/2009(H1N1)
NA	GU014773	H1N1	06/15/2009	Japan	A/Aichi/198/2009(H1N1)

NA	CY083338	H1N1	06/15/2009	USA	A/San Diego/WRAIR1654P/2009
NA	CY075181	H1N1	06/15/2009	Chile	A/Chile/1598/2009(H1N1)
NA	CY075213	H1N1	06/15/2009	Chile	A/Chile/1624/2009(H1N1)
NA	CY075189	H1N1	06/15/2009	Chile	A/Chile/1599/2009(H1N1)
NA	CY075205	H1N1	06/15/2009	Chile	A/Chile/1603/2009(H1N1)
NA	CY051009	H1N1	06/15/2009	USA	A/Wisconsin/629-D01619/2009(H1N1)
NA	CY051089	H1N1	06/15/2009	USA	A/Wisconsin/629-D01844/2009(H1N1)
NA	CY051137	H1N1	06/15/2009	USA	A/Wisconsin/629-D01152/2009(H1N1)
NA	CY051417	H1N1	06/15/2009	USA	A/Wisconsin/629-S0280/2009(H1N1)
NA	CY051433	H1N1	06/15/2009	USA	A/Wisconsin/629-S0293/2009(H1N1)
NA	CY052108	H1N1	06/15/2009	USA	A/Wisconsin/629-D00739/2009(H1N1)
NA	HQ247609	H1N1	06/15/2009	Finland	A/Finland/570/2009
NA	HQ247610	H1N1	06/15/2009	Finland	A/Finland/571/2009
NA	HM569706	H1N1	06/15/2009	Argentina	A/Argentina/19531/2009(H1N1)
NA	CY051425	H1N1	06/15/2009	USA	A/Wisconsin/629-S0279/2009(H1N1)
NA	CY123711	H1N1	06/15/2009	Singapore	A/Singapore/ON201/2009
NA	CY064494	H1N1	06/15/2009	USA	A/Boston/109/2009(H1N1)
NA	CY123827	H1N1	06/15/2009	Singapore	A/Singapore/ON226/2009
NA	CY051817	H1N1	06/15/2009	USA	A/Texas/42152486/2009(H1N1)
NA	CY052276	H1N1	06/15/2009	USA	A/Texas/42151049/2009(H1N1)
NA	CY054717	H1N1	06/15/2009	USA	A/California/VRDL5/2009(H1N1)
NA	CY055457	H1N1	06/15/2009	USA	A/California/VRDL12/2009(H1N1)
NA	KC781701	H1N1	06/15/2009	USA	A/Oregon/10/2009
NA	KC781552	H1N1	06/15/2009	USA	A/Massachusetts/16/2009
NA	CY058550	H1N1	06/15/2009	Nicaragua	A/Managua/4935.03/2009(H1N1)
NA	CY058126	H1N1	06/15/2009	Nicaragua	A/Managua/5453.01/2009(H1N1)
NA	CY064470	H1N1	06/15/2009	USA	A/Boston/103/2009(H1N1)
NA	CY064478	H1N1	06/15/2009	USA	A/Boston/106/2009(H1N1)
NA	CY050088	H1N1	06/16/2009	Colombia	A/Bogota/WR0090N/2009(H1N1)
NA	GQ328768	H1N1	06/16/2009	Greece	A/Athens/992/2009(H1N1)
NA	CY052212	H1N1	06/16/2009	USA	A/Texas/42163291/2009(H1N1)
NA	CY052385	H1N1	06/16/2009	USA	A/Texas/42163295/2009(H1N1)
NA	GQ902803	H1N1	06/16/2009	Thailand	A/Thailand/CU-B42/2009(H1N1)
NA	CY044173	H1N1	06/16/2009	USA	A/Bethesda/SP506/2009(H1N1)
NA	GQ334357	H1N1	06/16/2009	Japan	A/Utsunomiya/1/2009(H1N1)
NA	CY058158	H1N1	06/16/2009	Nicaragua	A/Managua/2330.02/2009(H1N1)
NA	GU014775	H1N1	06/16/2009	Japan	A/Aichi/202/2009(H1N1)
NA	GQ365457	H1N1	06/16/2009	Japan	A/Utsunomiya/2/2009(H1N1)
NA	CY074046	H1N1	06/16/2009	Argentina	A/Argentina/HNRG49/2009
NA	CY053966	H1N1	06/16/2009	Argentina	A/Argentina/HNRG45/2009(H1N1)
NA	CY064486	H1N1	06/16/2009	USA	A/Boston/108/2009(H1N1)
NA	CY051161	H1N1	06/16/2009	USA	A/Wisconsin/629-D00541/2009(H1N1)
NA	CY051465	H1N1	06/16/2009	USA	A/Wisconsin/629-S0314/2009(H1N1)
NA	HQ247613	H1N1	06/16/2009	Finland	A/Finland/574/2009
NA	AB510924	H1N1	06/16/2009	Japan	A/Aichi/200/2009(H1N1)
NA	JX556113	H1N1	06/16/2009	Cuba	A/Habana/406/2009
NA	GQ303553	H1N1	06/16/2009	Spain	A/Catalonia/362/2009(H1N1)
NA	AB510925	H1N1	06/16/2009	Japan	A/Aichi/201/2009(H1N1)
NA	AB510926	H1N1	06/16/2009	Japan	A/Aichi/202/2009(H1N1)
NA	CY061323	H1N1	06/16/2009	Argentina	A/Argentina/HNRG1017/2009
NA	CY061324	H1N1	06/16/2009	Argentina	A/Argentina/HNRG1018/2009
NA	CY061325	H1N1	06/16/2009	Argentina	A/Argentina/HNRG1019/2009
NA	CY061329	H1N1	06/16/2009	Argentina	A/Argentina/HNRG1022/2009
NA	CY061327	H1N1	06/16/2009	Argentina	A/Argentina/HNRG1020/2009

NA	CY061328	H1N1	06/16/2009	Argentina	A/Argentina/HNRG1021/2009
NA	CY089938	H1N1	06/16/2009	USA	A/Wisconsin/629-D00744/2009(H1N1)
NA	HQ239949	H1N1	06/16/2009	Canada	A/Canada-NFL/RV2510/2009
NA	HQ239930	H1N1	06/16/2009	Canada	A/Canada-MB/RV2291/2009
NA	CY051145	H1N1	06/16/2009	USA	A/Wisconsin/629-D01734/2009(H1N1)
NA	CY047813	H1N1	06/17/2009	Argentina	A/Argentina/7967/2009
NA	CY107200	H1N1	06/17/2009	United Ki	A/Scotland/Paisley_419540/2009
NA	CY051441	H1N1	06/17/2009	USA	A/Wisconsin/629-S0319/2009(H1N1)
NA	CY107774	H1N1	06/17/2009	United Ki	A/Scotland/EastKilbride_419809/2009
NA	CY107195	H1N1	06/17/2009	United Ki	A/Scotland/Paisley_417557/2009
NA	CY107764	H1N1	06/17/2009	United Ki	A/Scotland/Inverness_418366/2009
NA	CY107677	H1N1	06/17/2009	United Ki	A/Scotland/Glasgow_419977/2009
NA	CY107663	H1N1	06/17/2009	United Ki	A/Scotland/Glasgow_419707/2009
NA	CY107670	H1N1	06/17/2009	United Ki	A/Scotland/Glasgow_419865/2009
NA	CY054845	H1N1	06/17/2009	USA	A/California/VRDL31/2009(H1N1)
NA	CY051873	H1N1	06/17/2009	USA	A/Texas/42173957/2009(H1N1)
NA	CY053934	H1N1	06/17/2009	Argentina	A/Argentina/HNRG30/2009
NA	CY055449	H1N1	06/17/2009	USA	A/California/VRDL11/2009(H1N1)
NA	CY054733	H1N1	06/17/2009	USA	A/California/VRDL7/2009(H1N1)
NA	CY064590	H1N1	06/17/2009	USA	A/Boston/127/2009(H1N1)
NA	CY047781	H1N1	06/17/2009	Argentina	A/Argentina/7649/2009
NA	CY089727	H1N1	06/17/2009	USA	A/Boston/139/2009
NA	CY044122	H1N1	06/17/2009	USA	A/New York/4197/2009(H1N1)
NA	CY047821	H1N1	06/17/2009	Argentina	A/Argentina/7980/2009
NA	GQ392031	H1N1	06/17/2009	Italy	A/Italy/127/2009(H1N1)
NA	GU014751	H1N1	06/17/2009	Japan	A/Hiroshima/201/2009(H1N1)
NA	CY067802	H1N1	06/17/2009	Portugal	A/Lisboa/29/2009(H1N1)
NA	GQ455034	H1N1	06/17/2009	China	A/Nanjing/2/2009(H1N1)
NA	CY074073	H1N1	06/17/2009	Argentina	A/Argentina/HNRG83/2009
NA	CY053937	H1N1	06/17/2009	Argentina	A/Argentina/HNRG31/2009
NA	CY045980	H1N1	06/17/2009	Canada	A/Toronto/T9842/2009(H1N1)
NA	CY064574	H1N1	06/17/2009	USA	A/Boston/125/2009(H1N1)
NA	CY049973	H1N1	06/17/2009	Dominican	A/Santo Domingo/1066N/2009(H1N1)
NA	CY051545	H1N1	06/17/2009	USA	A/New York/4403/2009(H1N1)
NA	AB520639	H1N1	06/17/2009	Japan	A/Aichi/257/2009(H1N1)
NA	AB520640	H1N1	06/17/2009	Japan	A/Aichi/258/2009(H1N1)
NA	AB520641	H1N1	06/17/2009	Japan	A/Aichi/260/2009(H1N1)
NA	CY061330	H1N1	06/17/2009	Argentina	A/Argentina/HNRG1023/2009
NA	CY061332	H1N1	06/17/2009	Argentina	A/Argentina/HNRG1025/2009
NA	CY061331	H1N1	06/17/2009	Argentina	A/Argentina/HNRG1024/2009
NA	CY053466	H1N1	06/17/2009	Argentina	A/Argentina/16304/2009
NA	HQ239929	H1N1	06/17/2009	Canada	A/Canada-NB/RV2276/2009
NA	HQ239886	H1N1	06/17/2009	Canada	A/Canada-MB/RV2037-10/2009
NA	GQ866961	H1N1	06/17/2009	Thailand	A/Thailand/CU-H9/2009
NA	CY052100	H1N1	06/17/2009	USA	A/New York/4238/2009(H1N1)
NA	HQ247622	H1N1	06/18/2009	Finland	A/Finland/583/2009
NA	CY051169	H1N1	06/18/2009	USA	A/Wisconsin/629-D02063/2009(H1N1)
NA	HQ247623	H1N1	06/18/2009	Finland	A/Finland/584/2009
NA	CY047829	H1N1	06/18/2009	Argentina	A/Argentina/8019/2009
NA	HM569693	H1N1	06/18/2009	Argentina	A/Argentina/19656/2009(H1N1)
NA	CY054805	H1N1	06/18/2009	USA	A/California/VRDL26/2009(H1N1)
NA	CY064662	H1N1	06/18/2009	USA	A/Boston/138/2009(H1N1)
NA	CY051457	H1N1	06/18/2009	USA	A/Wisconsin/629-S0339/2009(H1N1)
NA	KC781804	H1N1	06/18/2009	USA	A/Oregon/11/2009

NA	KC781846	H1N1	06/18/2009	USA	A/Oregon/13/2009
NA	CY064630	H1N1	06/18/2009	USA	A/Boston/133/2009(H1N1)
NA	CY044181	H1N1	06/18/2009	USA	A/Silver Spring/SP509/2009(H1N1)
NA	GQ902835	H1N1	06/18/2009	Thailand	A/Thailand/CU-H17/2009(H1N1)
NA	GQ504753	H1N1	06/18/2009	China	A/Nanjing/1/2009(H1N1)
NA	CY044189	H1N1	06/18/2009	USA	A/Silver Spring/SP510/2009(H1N1)
NA	CY073296	H1N1	06/18/2009	USA	A/Silver Spring/WRAIRSP510P20/2009(I
NA	GQ457554	H1N1	06/18/2009	Canada	A/Saskatchewan/5350/2009
NA	GQ457555	H1N1	06/18/2009	Canada	A/Saskatchewan/5351/2009
NA	GQ365430	H1N1	06/18/2009	Japan	A/Iwate/2/2009(H1N1)
NA	GU014779	H1N1	06/18/2009	Japan	A/Chiba-C/51/2009(H1N1)
NA	GU014795	H1N1	06/18/2009	Japan	A/Kanagawa/140/2009(H1N1)
NA	CY043120	H1N1	06/18/2009	USA	A/Bethesda/SP508/2009(H1N1)
NA	CY053930	H1N1	06/18/2009	Argentina	A/Argentina/HNRG3/2009(H1N1)
NA	CY089906	H1N1	06/18/2009	USA	A/Wisconsin/629-D01115/2009(H1N1)
NA	CY073272	H1N1	06/18/2009	USA	A/Silver Spring/WRAIRSP510P12/2009(I
NA	CY073288	H1N1	06/18/2009	USA	A/Silver Spring/WRAIRSP510P2/2009(H
NA	CY073264	H1N1	06/18/2009	USA	A/Silver Spring/WRAIRSP510P10/2009(I
NA	CY089946	H1N1	06/18/2009	USA	A/Wisconsin/629-D01875/2009(H1N1)
NA	CY064646	H1N1	06/18/2009	USA	A/Boston/135/2009(H1N1)
NA	CY073280	H1N1	06/18/2009	USA	A/Silver Spring/WRAIRSP510P18/2009(I
NA	CY073304	H1N1	06/18/2009	USA	A/Silver Spring/WRAIRSP510P3/2009(H
NA	CY075221	H1N1	06/18/2009	Chile	A/Chile/2009/2009(H1N1)
NA	CY051449	H1N1	06/18/2009	USA	A/Wisconsin/629-S0345/2009(H1N1)
NA	HQ247624	H1N1	06/18/2009	Finland	A/Finland/585/2009
NA	HQ247615	H1N1	06/18/2009	Finland	A/Finland/576/2009
NA	HQ247621	H1N1	06/18/2009	Finland	A/Finland/582/2009
NA	GQ303554	H1N1	06/18/2009	Spain	A/Catalonia/366/2009(H1N1)
NA	CY089954	H1N1	06/18/2009	USA	A/Wisconsin/629-D01039/2009(H1N1)
NA	CY046924	H1N1	06/18/2009	Chile	A/Santiago/2009/2009(H1N1)
NA	CY061343	H1N1	06/18/2009	Argentina	A/Argentina/HNRG1036/2009
NA	CY061344	H1N1	06/18/2009	Argentina	A/Argentina/HNRG1037/2009
NA	CY061333	H1N1	06/18/2009	Argentina	A/Argentina/HNRG1026/2009
NA	CY061337	H1N1	06/18/2009	Argentina	A/Argentina/HNRG1030/2009
NA	CY061334	H1N1	06/18/2009	Argentina	A/Argentina/HNRG1027/2009
NA	CY061335	H1N1	06/18/2009	Argentina	A/Argentina/HNRG1028/2009
NA	CY061336	H1N1	06/18/2009	Argentina	A/Argentina/HNRG1029/2009
NA	CY056256	H1N1	06/18/2009	Argentina	A/Buenos Aires/HIFJM239057/2009(H1N
NA	CY061342	H1N1	06/18/2009	Argentina	A/Argentina/HNRG1035/2009
NA	CY047797	H1N1	06/18/2009	Argentina	A/Argentina/7937/2009
NA	CY061338	H1N1	06/18/2009	Argentina	A/Argentina/HNRG1031/2009
NA	CY061340	H1N1	06/18/2009	Argentina	A/Argentina/HNRG1033/2009
NA	CY064558	H1N1	06/18/2009	USA	A/Boston/123/2009(H1N1)
NA	HQ239959	H1N1	06/18/2009	Canada	A/Canada-AB/RV2811/2009
NA	CY061339	H1N1	06/18/2009	Argentina	A/Argentina/HNRG1032/2009
NA	CY061341	H1N1	06/18/2009	Argentina	A/Argentina/HNRG1034/2009
NA	HQ247617	H1N1	06/18/2009	Finland	A/Finland/578/2009
NA	HQ247619	H1N1	06/19/2009	Finland	A/Finland/580/2009
NA	HQ247620	H1N1	06/19/2009	Finland	A/Finland/581/2009
NA	HQ247616	H1N1	06/19/2009	Finland	A/Finland/577/2009
NA	CY087300	H1N1	06/19/2009	Hong Kong	A/Hong Kong/423432/2009
NA	CY083090	H1N1	06/19/2009	Colombia	A/Bogota/WRAIR0442T/2009
NA	CY049989	H1N1	06/19/2009	Mexico	A/Mexico City/WR1087T/2009(H1N1)
NA	CY047853	H1N1	06/19/2009	Argentina	A/Argentina/8673/2009

NA	CY047805	H1N1	06/19/2009	Argentina	A/Argentina/7953/2009
NA	CY052085	H1N1	06/19/2009	Italy	A/Italy/226/2009(H1N1)
NA	CY123898	H1N1	06/19/2009	Singapore	A/Singapore/ON276/2009
NA	CY123899	H1N1	06/19/2009	Singapore	A/Singapore/ON278/2009
NA	CY123901	H1N1	06/19/2009	Singapore	A/Singapore/ON279/2009
NA	CY123904	H1N1	06/19/2009	Singapore	A/Singapore/ON288/2009
NA	CY123917	H1N1	06/19/2009	Singapore	A/Singapore/ON312/2009
NA	CY052236	H1N1	06/19/2009	USA	A/Texas/42191647/2009(H1N1)
NA	CY052417	H1N1	06/19/2009	USA	A/Texas/42192947/2009(H1N1)
NA	CY051849	H1N1	06/19/2009	USA	A/Texas/42191653/2009(H1N1)
NA	CY064542	H1N1	06/19/2009	USA	A/Boston/121/2009(H1N1)
NA	CY049366	H1N1	06/19/2009	Singapore	A/Singapore/ON313/2009(H1N1)
NA	CY045071	H1N1	06/19/2009	USA	A/New York/4290/2009(H1N1)
NA	CY075229	H1N1	06/19/2009	Chile	A/Chile/2239/2009(H1N1)
NA	CY064622	H1N1	06/19/2009	USA	A/Boston/132/2009(H1N1)
NA	GQ457553	H1N1	06/19/2009	Canada	A/Saskatchewan/5131/2009
NA	CY067805	H1N1	06/19/2009	Portugal	A/Lisboa/31/2009(H1N1)
NA	CY073976	H1N1	06/19/2009	Argentina	A/Argentina/HNRG104/2009
NA	CY074056	H1N1	06/19/2009	Argentina	A/Argentina/HNRG72/2009
NA	CY053917	H1N1	06/19/2009	Argentina	A/Argentina/HNRG20/2009
NA	CY051561	H1N1	06/19/2009	USA	A/New York/4459/2009(H1N1)
NA	HQ247618	H1N1	06/19/2009	Finland	A/Finland/579/2009
NA	CY089962	H1N1	06/19/2009	USA	A/Wisconsin/629-D02404/2009(H1N1)
NA	CY061346	H1N1	06/19/2009	Argentina	A/Argentina/HNRG1039/2009
NA	AB520642	H1N1	06/19/2009	Japan	A/Aichi/265/2009(H1N1)
NA	CY061350	H1N1	06/19/2009	Argentina	A/Argentina/HNRG1042/2009
NA	CY061351	H1N1	06/19/2009	Argentina	A/Argentina/HNRG1043/2009
NA	CY061353	H1N1	06/19/2009	Argentina	A/Argentina/HNRG1045/2009
NA	CY061345	H1N1	06/19/2009	Argentina	A/Argentina/HNRG1038/2009
NA	CY061348	H1N1	06/19/2009	Argentina	A/Argentina/HNRG1040/2009
NA	CY061352	H1N1	06/19/2009	Argentina	A/Argentina/HNRG1044/2009
NA	CY061354	H1N1	06/19/2009	Argentina	A/Argentina/HNRG1046/2009
NA	CY061349	H1N1	06/19/2009	Argentina	A/Argentina/HNRG1041/2009
NA	CY049086	H1N1	06/19/2009	Singapore	A/Singapore/ON279/2009(H1N1)
NA	CY064904	H1N1	06/19/2009	Italy	A/Rome/689/2009(H1N1)
NA	KC117387	H1N1	06/19/2009	New Zeala	A/Wellington/31/2009
NA	CY061356	H1N1	06/19/2009	Argentina	A/Argentina/HNRG1048/2009
NA	CY061355	H1N1	06/19/2009	Argentina	A/Argentina/HNRG1047/2009
NA	CY064534	H1N1	06/19/2009	USA	A/Boston/120/2009(H1N1)
NA	CY123923	H1N1	06/19/2009	Singapore	A/Singapore/ON313/2009
NA	CY051865	H1N1	06/20/2009	USA	A/Texas/42201824/2009(H1N1)
NA	GQ409638	H1N1	06/20/2009	Greece	A/Athens/1079/2009(H1N1)
NA	CY049142	H1N1	06/20/2009	Singapore	A/Singapore/ON304/2009(H1N1)
NA	CY049062	H1N1	06/20/2009	Singapore	A/Singapore/ON305/2009(H1N1)
NA	CY054765	H1N1	06/20/2009	USA	A/California/VRDL20/2009(H1N1)
NA	HQ247614	H1N1	06/20/2009	Finland	A/Finland/575/2009
NA	CY064526	H1N1	06/20/2009	USA	A/Boston/118/2009(H1N1)
NA	CY051193	H1N1	06/20/2009	USA	A/Wisconsin/629-D00496/2009(H1N1)
NA	CY051177	H1N1	06/20/2009	USA	A/Wisconsin/629-D00428/2009(H1N1)
NA	KT181540	H1N1	06/20/2009	Singapore	A/Singapore/DMS385/2009
NA	CY123910	H1N1	06/20/2009	Singapore	A/Singapore/ON304/2009
NA	CY072984	H1N1	06/20/2009	Nicaragua	A/Managua/5273.02/2009(H1N1)
NA	CY075237	H1N1	06/20/2009	Chile	A/Chile/2851/2009(H1N1)
NA	CY061360	H1N1	06/20/2009	Argentina	A/Argentina/HNRG1051/2009

NA	CY123914	H1N1	06/20/2009	Singapore	A/Singapore/ON305/2009
NA	GQ494353	H1N1	06/20/2009	Russia	A/Moscow/IIV05/2009(H1N1)
NA	CY052324	H1N1	06/20/2009	USA	A/Texas/42202026/2009(H1N1)
NA	CY052244	H1N1	06/20/2009	USA	A/Texas/42201798/2009(H1N1)
NA	CY064654	H1N1	06/20/2009	USA	A/Boston/137/2009(H1N1)
NA	CY047877	H1N1	06/20/2009	Argentina	A/Argentina/9004/2009
NA	CY061357	H1N1	06/20/2009	Argentina	A/Argentina/HNRG1049/2009
NA	CY061359	H1N1	06/20/2009	Argentina	A/Argentina/HNRG1050/2009
NA	AB520643	H1N1	06/20/2009	Japan	A/Aichi/266/2009(H1N1)
NA	GQ409645	H1N1	06/20/2009	Greece	A/Athens/1087/2009(H1N1)
NA	CY064582	H1N1	06/20/2009	USA	A/Boston/126/2009(H1N1)
NA	CY123935	H1N1	06/21/2009	Singapore	A/Singapore/ON360/2009
NA	GQ409639	H1N1	06/21/2009	Greece	A/Lamia/1096/2009(H1N1)
NA	CY058534	H1N1	06/21/2009	Nicaragua	A/Managua/4514.03/2009(H1N1)
NA	CY064606	H1N1	06/21/2009	USA	A/Boston/130/2009(H1N1)
NA	CY047845	H1N1	06/21/2009	Argentina	A/Argentina/8574/2009
NA	CY051185	H1N1	06/21/2009	USA	A/Wisconsin/629-D02220/2009(H1N1)
NA	CY047861	H1N1	06/21/2009	Argentina	A/Argentina/8989/2009
NA	CY123943	H1N1	06/21/2009	Singapore	A/Singapore/ON362/2009
NA	CY047837	H1N1	06/21/2009	Argentina	A/Argentina/8551/2009
NA	CY054278	H1N1	06/21/2009	Argentina	A/Argentina/HNRG36/2009(H1N1)
NA	CY051537	H1N1	06/21/2009	USA	A/New York/4402/2009(H1N1)
NA	CY061361	H1N1	06/21/2009	Argentina	A/Argentina/HNRG1052/2009
NA	CY052332	H1N1	06/21/2009	USA	A/Texas/42211898/2009(H1N1)
NA	CY061362	H1N1	06/21/2009	Argentina	A/Argentina/HNRG1053/2009
NA	CY089970	H1N1	06/21/2009	USA	A/Wisconsin/629-D02294/2009(H1N1)
NA	GQ409636	H1N1	06/21/2009	Greece	A/Athens/1097/2009(H1N1)
NA	GQ409637	H1N1	06/21/2009	Greece	A/Athens/1085/2009(H1N1)
NA	CY123939	H1N1	06/21/2009	Singapore	A/Singapore/ON361/2009
NA	CY123931	H1N1	06/22/2009	Singapore	A/Singapore/ON355/2009(H1N1)
NA	CY061376	H1N1	06/22/2009	Argentina	A/Argentina/HNRG1066/2009
NA	CY123947	H1N1	06/22/2009	Singapore	A/Singapore/ON368/2009
NA	CY123348	H1N1	06/22/2009	Singapore	A/Singapore/GP687/2009
NA	CY064550	H1N1	06/22/2009	USA	A/Boston/122/2009(H1N1)
NA	CY052260	H1N1	06/22/2009	USA	A/Texas/42221280/2009(H1N1)
NA	KC781888	H1N1	06/22/2009	USA	A/Utah/21/2009
NA	KC781713	H1N1	06/22/2009	USA	A/New York/98/2009
NA	CY064598	H1N1	06/22/2009	USA	A/Boston/129/2009(H1N1)
NA	CY064670	H1N1	06/22/2009	USA	A/Boston/140/2009(H1N1)
NA	CY058142	H1N1	06/22/2009	Nicaragua	A/Managua/5665.01/2009(H1N1)
NA	CY054821	H1N1	06/22/2009	USA	A/California/VRDL28/2009(H1N1)
NA	JN256858	H1N1	06/22/2009	Hong Kong	A/Hong Kong/H090-661-V1(0)/2009
NA	CY051201	H1N1	06/22/2009	USA	A/Wisconsin/629-D02024/2009(H1N1)
NA	CY049853	H1N1	06/22/2009	Bolivia	A/La Paz/WR0096T/2009(H1N1)
NA	CY111613	H1N1	06/22/2009	Hong Kong	A/Hong Kong/H090-661-V10/2009
NA	KT181406	H1N1	06/22/2009	Singapore	A/Singapore/DMS4/2009
NA	KT181559	H1N1	06/22/2009	Singapore	A/Singapore/DMS562/2009
NA	KT181553	H1N1	06/22/2009	Singapore	A/Singapore/DMS516/2009
NA	CY120495	H1N1	06/22/2009	Hong Kong	A/Hong Kong/H090-661-V10/2009
NA	CY047909	H1N1	06/22/2009	Argentina	A/Argentina/9452/2009
NA	GU271952	H1N1	06/22/2009	Thailand	A/Thailand/CU-B97/2009(H1N1)
NA	GU014811	H1N1	06/22/2009	Japan	A/Yamaguchi/23/2009(H1N1)
NA	GU014765	H1N1	06/22/2009	Japan	A/Niigata/700/2009(H1N1)
NA	CY053948	H1N1	06/22/2009	Argentina	A/Argentina/HNRG37/2009

NA	CY053940	H1N1	06/22/2009	Argentina	A/Argentina/HNRG32/2009(H1N1)
NA	CY047869	H1N1	06/22/2009	Argentina	A/Argentina/8994/2009
NA	CY075245	H1N1	06/22/2009	Chile	A/Chile/2362/2009(H1N1)
NA	CY123970	H1N1	06/22/2009	Singapore	A/Singapore/ON406/2009
NA	CY061366	H1N1	06/22/2009	Argentina	A/Argentina/HNRG1057/2009
NA	CY061371	H1N1	06/22/2009	Argentina	A/Argentina/HNRG1061/2009
NA	CY061363	H1N1	06/22/2009	Argentina	A/Argentina/HNRG1054/2009
NA	CY061364	H1N1	06/22/2009	Argentina	A/Argentina/HNRG1055/2009
NA	CY061367	H1N1	06/22/2009	Argentina	A/Argentina/HNRG1058/2009
NA	CY049374	H1N1	06/22/2009	Singapore	A/Singapore/ON355/2009(H1N1)
NA	CY049094	H1N1	06/22/2009	Singapore	A/Singapore/ON354/2009(H1N1)
NA	CY053972	H1N1	06/22/2009	Argentina	A/Argentina/HNRG5/2009(H1N1)
NA	CY055473	H1N1	06/22/2009	USA	A/California/VRDL18/2009(H1N1)
NA	CY061373	H1N1	06/22/2009	Argentina	A/Argentina/HNRG1063/2009
NA	CY061374	H1N1	06/22/2009	Argentina	A/Argentina/HNRG1064/2009
NA	CY061370	H1N1	06/22/2009	Argentina	A/Argentina/HNRG1060/2009
NA	CY061372	H1N1	06/22/2009	Argentina	A/Argentina/HNRG1062/2009
NA	CY061365	H1N1	06/22/2009	Argentina	A/Argentina/HNRG1056/2009
NA	CY061368	H1N1	06/22/2009	Argentina	A/Argentina/HNRG1059/2009
NA	CY049358	H1N1	06/22/2009	Singapore	A/Singapore/ON368/2009(H1N1)
NA	AB520647	H1N1	06/22/2009	Japan	A/Aichi/272/2009(H1N1)
NA	AB520645	H1N1	06/22/2009	Japan	A/Aichi/269/2009(H1N1)
NA	AB520646	H1N1	06/22/2009	Japan	A/Aichi/270/2009(H1N1)
NA	CY061375	H1N1	06/22/2009	Argentina	A/Argentina/HNRG1065/2009
NA	GQ409641	H1N1	06/22/2009	Greece	A/Athens/1106/2009(H1N1)
NA	CY061377	H1N1	06/22/2009	Argentina	A/Argentina/HNRG1067/2009
NA	CY054773	H1N1	06/22/2009	USA	A/California/VRDL21/2009(H1N1)
NA	CY123340	H1N1	06/23/2009	Singapore	A/Singapore/GP674/2009
NA	CY061389	H1N1	06/23/2009	Argentina	A/Argentina/HNRG1078/2009
NA	JX625413	H1N1	06/23/2009	United Ki	A/England/92880002/2009
NA	HQ256629	H1N1	06/23/2009	Myanmar	A/Myanmar/M286/2009(H1N1)
NA	HQ247625	H1N1	06/23/2009	Finland	A/Finland/592/2009
NA	CY047893	H1N1	06/23/2009	Argentina	A/Argentina/9333/2009
NA	CY065160	H1N1	06/23/2009	United Ki	A/England/541/2009
NA	KT181556	H1N1	06/23/2009	Singapore	A/Singapore/DMS547/2009
NA	CY051481	H1N1	06/23/2009	USA	A/Wisconsin/629-S0410/2009(H1N1)
NA	CY123955	H1N1	06/23/2009	Singapore	A/Singapore/ON392/2009(H1N1)
NA	CY047885	H1N1	06/23/2009	Argentina	A/Argentina/9180/2009
NA	CY047973	H1N1	06/23/2009	Argentina	A/Argentina/9721/2009
NA	CY047901	H1N1	06/23/2009	Argentina	A/Argentina/9384/2009
NA	GU014801	H1N1	06/23/2009	Japan	A/Niigata/717/2009(H1N1)
NA	GQ906584	H1N1	06/23/2009	Sweden	A/Stockholm/49/2009(H1N1)
NA	GQ365420	H1N1	06/23/2009	Japan	A/Fukushima/1/2009(H1N1)
NA	CY053951	H1N1	06/23/2009	Argentina	A/Argentina/HNRG39/2009
NA	KF849741	H1N1	06/23/2009	Thailand	A/Thailand/SirirajICRC_CBI_7/2009
NA	KF849738	H1N1	06/23/2009	Thailand	A/Thailand/SirirajICRC_CBI_2/2009
NA	KF849740	H1N1	06/23/2009	Thailand	A/Thailand/SirirajICRC_CBI_4/2009
NA	KF849739	H1N1	06/23/2009	Thailand	A/Thailand/SirirajICRC_CBI_3/2009
NA	CY051553	H1N1	06/23/2009	USA	A/New York/4434/2009(H1N1)
NA	HQ256631	H1N1	06/23/2009	Myanmar	A/Myanmar/M294/2009(H1N1)
NA	HE804123	H1N1	06/23/2009	Uruguay	A/Uruguay/319/2009
NA	HE804124	H1N1	06/23/2009	Uruguay	A/Uruguay/323/2009
NA	CY049294	H1N1	06/23/2009	Singapore	A/Singapore/ON380/2009(H1N1)
NA	CY049302	H1N1	06/23/2009	Singapore	A/Singapore/ON384/2009(H1N1)

NA	CY055465	H1N1	06/23/2009	USA	A/California/VRDL14/2009(H1N1)
NA	CY047965	H1N1	06/23/2009	Argentina	A/Argentina/9711/2009
NA	CY061383	H1N1	06/23/2009	Argentina	A/Argentina/HNRG1072/2009
NA	CY061391	H1N1	06/23/2009	Argentina	A/Argentina/HNRG1080/2009
NA	CY061379	H1N1	06/23/2009	Argentina	A/Argentina/HNRG1069/2009
NA	CY061390	H1N1	06/23/2009	Argentina	A/Argentina/HNRG1079/2009
NA	CY061381	H1N1	06/23/2009	Argentina	A/Argentina/HNRG1070/2009
NA	CY061382	H1N1	06/23/2009	Argentina	A/Argentina/HNRG1071/2009
NA	CY061385	H1N1	06/23/2009	Argentina	A/Argentina/HNRG1074/2009
NA	CY061388	H1N1	06/23/2009	Argentina	A/Argentina/HNRG1077/2009
NA	CY061392	H1N1	06/23/2009	Argentina	A/Argentina/HNRG1081/2009
NA	CY061386	H1N1	06/23/2009	Argentina	A/Argentina/HNRG1075/2009
NA	CY061387	H1N1	06/23/2009	Argentina	A/Argentina/HNRG1076/2009
NA	GQ387440	H1N1	06/23/2009	Spain	A/Catalonia/378/2009(H1N1)
NA	GQ387441	H1N1	06/23/2009	Spain	A/Catalonia/382/2009(H1N1)
NA	AB520650	H1N1	06/23/2009	Japan	A/Aichi/277/2009(H1N1)
NA	CY061378	H1N1	06/23/2009	Argentina	A/Argentina/HNRG1068/2009
NA	CY061384	H1N1	06/23/2009	Argentina	A/Argentina/HNRG1073/2009
NA	CY089978	H1N1	06/23/2009	USA	A/Wisconsin/629-D00466/2009(H1N1)
NA	AB520652	H1N1	06/23/2009	Japan	A/Aichi/280/2009(H1N1)
NA	AB520651	H1N1	06/23/2009	Japan	A/Aichi/278/2009(H1N1)
NA	AB520653	H1N1	06/23/2009	Japan	A/Aichi/281/2009(H1N1)
NA	CY123332	H1N1	06/23/2009	Singapore	A/Singapore/GP669/2009
NA	CY123966	H1N1	06/24/2009	Singapore	A/Singapore/ON402/2009(H1N1)
NA	CY054781	H1N1	06/24/2009	USA	A/California/VRDL22/2009(H1N1)
NA	HM569705	H1N1	06/24/2009	Argentina	A/Argentina/19911/2009(H1N1)
NA	CY054829	H1N1	06/24/2009	USA	A/California/VRDL29/2009(H1N1)
NA	CY051473	H1N1	06/24/2009	USA	A/Wisconsin/629-S0435/2009(H1N1)
NA	CY047925	H1N1	06/24/2009	Argentina	A/Argentina/9579/2009
NA	CY047957	H1N1	06/24/2009	Argentina	A/Argentina/9705/2009
NA	CY087218	H1N1	06/24/2009	USA	A/Houston/210S/2009
NA	CY083625	H1N1	06/24/2009	Peru	A/Trujillo/WRAIR9309F/2009
NA	KT181407	H1N1	06/24/2009	Singapore	A/Singapore/DMS5/2009
NA	KT181527	H1N1	06/24/2009	Singapore	A/Singapore/DMS223/2009
NA	CY052148	H1N1	06/24/2009	USA	A/New York/4398/2009(H1N1)
NA	CY107785	H1N1	06/24/2009	United Ki	A/Scotland/EastKilbride_420874/2009
NA	CY107799	H1N1	06/24/2009	United Ki	A/Scotland/EastKilbride_420880/2009
NA	CY107806	H1N1	06/24/2009	United Ki	A/Scotland/EastKilbride_420881/2009
NA	CY107821	H1N1	06/24/2009	United Ki	A/Scotland/StAndrews_17693/2009
NA	CY107542	H1N1	06/24/2009	United Ki	A/Scotland/Fife_17690/2009
NA	CY107684	H1N1	06/24/2009	United Ki	A/Scotland/Glasgow_420755/2009
NA	CY107695	H1N1	06/24/2009	United Ki	A/Scotland/Glasgow_422823/2009
NA	CY107781	H1N1	06/24/2009	United Ki	A/Scotland/EastKilbride_420703/2009
NA	CY107792	H1N1	06/24/2009	United Ki	A/Scotland/EastKilbride_420875/2009
NA	CY107688	H1N1	06/24/2009	United Ki	A/Scotland/Glasgow_420768/2009
NA	CY107481	H1N1	06/24/2009	United Ki	A/Scotland/Edinburgh_17645/2009
NA	CY107328	H1N1	06/24/2009	United Ki	A/Scotland/Edinburgh_17715/2009
NA	CY123974	H1N1	06/24/2009	Singapore	A/Singapore/ON457/2009
NA	CY123959	H1N1	06/24/2009	Singapore	A/Singapore/ON396/2009
NA	CY047941	H1N1	06/24/2009	Argentina	A/Argentina/9588/2009
NA	CY047949	H1N1	06/24/2009	Argentina	A/Argentina/9597/2009
NA	CY047933	H1N1	06/24/2009	Argentina	A/Argentina/9583/2009
NA	CY107467	H1N1	06/24/2009	United Ki	A/Scotland/Fife_17712/2009
NA	CY053954	H1N1	06/24/2009	Argentina	A/Argentina/HNRG40/2009(H1N1)

NA	CY053943	H1N1	06/24/2009	Argentina	A/Argentina/HNRG33/2009(H1N1)
NA	CY073983	H1N1	06/24/2009	Argentina	A/Argentina/HNRG105/2009
NA	KF612133	H1N1	06/24/2009	Bolivia	A/Santa Cruz/46631/2009
NA	CY049334	H1N1	06/24/2009	Singapore	A/Singapore/ON479/2009(H1N1)
NA	CY107489	H1N1	06/24/2009	United Ki	A/Scotland/Edinburgh_17714/2009
NA	CY049310	H1N1	06/24/2009	Singapore	A/Singapore/ON402/2009
NA	CY064614	H1N1	06/24/2009	USA	A/Boston/131/2009(H1N1)
NA	CY061400	H1N1	06/24/2009	Argentina	A/Argentina/HNRG1089/2009
NA	CY061402	H1N1	06/24/2009	Argentina	A/Argentina/HNRG1091/2009
NA	CY061396	H1N1	06/24/2009	Argentina	A/Argentina/HNRG1085/2009
NA	CY061394	H1N1	06/24/2009	Argentina	A/Argentina/HNRG1083/2009
NA	CY061398	H1N1	06/24/2009	Argentina	A/Argentina/HNRG1087/2009
NA	CY061401	H1N1	06/24/2009	Argentina	A/Argentina/HNRG1090/2009
NA	CY061404	H1N1	06/24/2009	Argentina	A/Argentina/HNRG1093/2009
NA	AB520654	H1N1	06/24/2009	Japan	A/Aichi/282/2009(H1N1)
NA	CY061403	H1N1	06/24/2009	Argentina	A/Argentina/HNRG1092/2009
NA	CY046926	H1N1	06/24/2009	Chile	A/Santiago/2696/2009(H1N1)
NA	CY061397	H1N1	06/24/2009	Argentina	A/Argentina/HNRG1086/2009
NA	CY047917	H1N1	06/24/2009	Argentina	A/Argentina/9576/2009
NA	CY061393	H1N1	06/24/2009	Argentina	A/Argentina/HNRG1082/2009
NA	CY061399	H1N1	06/24/2009	Argentina	A/Argentina/HNRG1088/2009
NA	CY061395	H1N1	06/24/2009	Argentina	A/Argentina/HNRG1084/2009
NA	CY052308	H1N1	06/25/2009	USA	A/Texas/42254309/2009(H1N1)
NA	CY061430	H1N1	06/25/2009	Argentina	A/Argentina/HNRG1120/2009
NA	CY057224	H1N1	06/25/2009	Nicaragua	A/Managua/611.01/2009(H1N1)
NA	CY075261	H1N1	06/25/2009	Chile	A/Chile/2911/2009(H1N1)
NA	CY050184	H1N1	06/25/2009	USA	A/New York/4438/2009(H1N1)
NA	CY083577	H1N1	06/25/2009	Peru	A/Lima/WRAIR8648F/2009
NA	CY049981	H1N1	06/25/2009	Dominican	A/Santo Domingo/WR1068N/2009(H1N1)
NA	CY083529	H1N1	06/25/2009	Peru	A/Lima/WRAIR1696P/2009
NA	CY052156	H1N1	06/25/2009	USA	A/New York/4401/2009(H1N1)
NA	CY058206	H1N1	06/25/2009	Nicaragua	A/Managua/791.03/2009(H1N1)
NA	CY075253	H1N1	06/25/2009	Chile	A/Chile/2909/2009(H1N1)
NA	CY044149	H1N1	06/25/2009	Colombia	A/Bogota/0466N/2009(H1N1)
NA	GU014789	H1N1	06/25/2009	Japan	A/Hiroshima/230/2009(H1N1)
NA	KJ130198	H1N1	06/25/2009	New Zeala	A/New Zealand/2047/2009
NA	CY053969	H1N1	06/25/2009	Argentina	A/Argentina/HNRG48/2009
NA	CY083569	H1N1	06/25/2009	Peru	A/Lima/WRAIR8511F/2009
NA	CY051529	H1N1	06/25/2009	USA	A/New York/4400/2009(H1N1)
NA	CY049382	H1N1	06/25/2009	Singapore	A/Singapore/ON442/2009(H1N1)
NA	CY049390	H1N1	06/25/2009	Singapore	A/Singapore/ON496/2009(H1N1)
NA	CY049398	H1N1	06/25/2009	Singapore	A/Singapore/ON506/2009(H1N1)
NA	HE804121	H1N1	06/25/2009	Uruguay	A/Uruguay/345/2009
NA	CY053467	H1N1	06/25/2009	Argentina	A/Argentina/16919/2009
NA	CY052086	H1N1	06/25/2009	Italy	A/Italy/227/2009(H1N1)
NA	CY061422	H1N1	06/25/2009	Argentina	A/Argentina/HNRG1112/2009
NA	CY049150	H1N1	06/25/2009	Singapore	A/Singapore/ON504/2009(H1N1)
NA	CY049102	H1N1	06/25/2009	Singapore	A/Singapore/ON511/2009(H1N1)
NA	HQ239931	H1N1	06/25/2009	Canada	A/Canada-NB/RV2431/2009
NA	HQ239932	H1N1	06/25/2009	Canada	A/Canada-NB/RV2456/2009
NA	CY049158	H1N1	06/25/2009	Singapore	A/Singapore/ON507/2009(H1N1)
NA	CY049318	H1N1	06/25/2009	Singapore	A/Singapore/ON439/2009(H1N1)
NA	CY049326	H1N1	06/25/2009	Singapore	A/Singapore/ON478/2009(H1N1)
NA	CY049941	H1N1	06/25/2009	Dominican	A/Santo Domingo/WR1056T/2009(H1N1)

NA	CY064678	H1N1	06/25/2009	USA	A/Boston/141/2009(H1N1)
NA	CY061418	H1N1	06/25/2009	Argentina	A/Argentina/HNRG1108/2009
NA	CY061421	H1N1	06/25/2009	Argentina	A/Argentina/HNRG1111/2009
NA	CY061424	H1N1	06/25/2009	Argentina	A/Argentina/HNRG1114/2009
NA	CY061428	H1N1	06/25/2009	Argentina	A/Argentina/HNRG1118/2009
NA	CY061407	H1N1	06/25/2009	Argentina	A/Argentina/HNRG1096/2009
NA	CY061408	H1N1	06/25/2009	Argentina	A/Argentina/HNRG1097/2009
NA	CY061417	H1N1	06/25/2009	Argentina	A/Argentina/HNRG1107/2009
NA	CY061419	H1N1	06/25/2009	Argentina	A/Argentina/HNRG1109/2009
NA	CY061423	H1N1	06/25/2009	Argentina	A/Argentina/HNRG1113/2009
NA	CY061427	H1N1	06/25/2009	Argentina	A/Argentina/HNRG1117/2009
NA	CY061410	H1N1	06/25/2009	Argentina	A/Argentina/HNRG1099/2009
NA	CY061411	H1N1	06/25/2009	Argentina	A/Argentina/HNRG1100/2009
NA	CY061412	H1N1	06/25/2009	Argentina	A/Argentina/HNRG1101/2009
NA	CY061415	H1N1	06/25/2009	Argentina	A/Argentina/HNRG1105/2009
NA	CY061426	H1N1	06/25/2009	Argentina	A/Argentina/HNRG1116/2009
NA	CY056257	H1N1	06/25/2009	Argentina	A/Buenos Aires/HIFJM239548/2009(H1N
NA	CY061405	H1N1	06/25/2009	Argentina	A/Argentina/HNRG1094/2009
NA	CY061413	H1N1	06/25/2009	Argentina	A/Argentina/HNRG1103/2009
NA	CY061420	H1N1	06/25/2009	Argentina	A/Argentina/HNRG1110/2009
NA	CY061416	H1N1	06/25/2009	Argentina	A/Argentina/HNRG1106/2009
NA	CY061414	H1N1	06/25/2009	Argentina	A/Argentina/HNRG1104/2009
NA	HE804104	H1N1	06/25/2009	Uruguay	A/Uruguay/39/2009
NA	GQ355295	H1N1	06/25/2009	Spain	A/Catalonia/385/2009(H1N1)
NA	CY061406	H1N1	06/25/2009	Argentina	A/Argentina/HNRG1095/2009
NA	CY061409	H1N1	06/25/2009	Argentina	A/Argentina/HNRG1098/2009
NA	CY061425	H1N1	06/25/2009	Argentina	A/Argentina/HNRG1115/2009
NA	CY061429	H1N1	06/25/2009	Argentina	A/Argentina/HNRG1119/2009
NA	KC781503	H1N1	06/25/2009	USA	A/Utah/22/2009
NA	CY123998	H1N1	06/26/2009	Singapore	A/Singapore/ON496/2009
NA	CY061436	H1N1	06/26/2009	Argentina	A/Argentina/HNRG1126/2009
NA	JX625389	H1N1	06/26/2009	United Ki	A/England/506/2009
NA	CY052116	H1N1	06/26/2009	USA	A/Wisconsin/629-D01973/2009(H1N1)
NA	CY053168	H1N1	06/26/2009	USA	A/Houston/240S/2009(H1N1)
NA	KC782289	H1N1	06/26/2009	USA	A/Texas/48/2009
NA	CY049342	H1N1	06/26/2009	Singapore	A/Singapore/ON582/2009(H1N1)
NA	CY058542	H1N1	06/26/2009	Nicaragua	A/Managua/462.01/2009(H1N1)
NA	CY049110	H1N1	06/26/2009	Singapore	A/Singapore/ON581/2009(H1N1)
NA	CY075277	H1N1	06/26/2009	Chile	A/Chile/3019/2009(H1N1)
NA	CY124006	H1N1	06/26/2009	Singapore	A/Singapore/ON506/2009
NA	CY124014	H1N1	06/26/2009	Singapore	A/Singapore/ON520/2009
NA	CY123990	H1N1	06/26/2009	Singapore	A/Singapore/ON479/2009
NA	CY123982	H1N1	06/26/2009	Singapore	A/Singapore/ON477/2009
NA	CY124022	H1N1	06/26/2009	Singapore	A/Singapore/ON547/2009
NA	CY044157	H1N1	06/26/2009	Nicaragua	A/Managua/0536N/2009(H1N1)
NA	CY064897	H1N1	06/26/2009	USA	A/Boston/136/2009(H1N1)
NA	CY067877	H1N1	06/26/2009	Portugal	A/Lisboa/75/2009(H1N1)
NA	CY075285	H1N1	06/26/2009	Chile	A/Chile/3056/2009
NA	CY053957	H1N1	06/26/2009	Argentina	A/Argentina/HNRG41/2009
NA	KF612140	H1N1	06/26/2009	Bolivia	A/Santa Cruz/97831/2009
NA	CY075269	H1N1	06/26/2009	Chile	A/Chile/2994/2009(H1N1)
NA	CY075293	H1N1	06/26/2009	Chile	A/Chile/3123/2009(H1N1)
NA	CY049190	H1N1	06/26/2009	Singapore	A/Singapore/ON594/2009(H1N1)
NA	CY049350	H1N1	06/26/2009	Singapore	A/Singapore/ON593/2009(H1N1)

NA	CY049406	H1N1	06/26/2009	Singapore	A/Singapore/ON575/2009(H1N1)
NA	HE804117	H1N1	06/26/2009	Uruguay	A/Uruguay/5/2009
NA	HQ239954	H1N1	06/26/2009	Canada	A/Canada-AB/RV2621/2009
NA	CY049166	H1N1	06/26/2009	Singapore	A/Singapore/ON584/2009(H1N1)
NA	CY049174	H1N1	06/26/2009	Singapore	A/Singapore/ON588/2009(H1N1)
NA	CY064638	H1N1	06/26/2009	USA	A/Boston/134/2009(H1N1)
NA	CY064518	H1N1	06/26/2009	USA	A/Boston/117/2009(H1N1)
NA	CY064566	H1N1	06/26/2009	USA	A/Boston/124/2009(H1N1)
NA	CY061445	H1N1	06/26/2009	Argentina	A/Argentina/HNRG1135/2009
NA	CY061438	H1N1	06/26/2009	Argentina	A/Argentina/HNRG1128/2009
NA	CY061444	H1N1	06/26/2009	Argentina	A/Argentina/HNRG1134/2009
NA	CY061446	H1N1	06/26/2009	Argentina	A/Argentina/HNRG1136/2009
NA	CY061431	H1N1	06/26/2009	Argentina	A/Argentina/HNRG1121/2009
NA	CY061435	H1N1	06/26/2009	Argentina	A/Argentina/HNRG1125/2009
NA	CY061433	H1N1	06/26/2009	Argentina	A/Argentina/HNRG1123/2009
NA	CY061439	H1N1	06/26/2009	Argentina	A/Argentina/HNRG1129/2009
NA	CY061442	H1N1	06/26/2009	Argentina	A/Argentina/HNRG1132/2009
NA	CY061443	H1N1	06/26/2009	Argentina	A/Argentina/HNRG1133/2009
NA	CY049182	H1N1	06/26/2009	Singapore	A/Singapore/ON590/2009(H1N1)
NA	CY061437	H1N1	06/26/2009	Argentina	A/Argentina/HNRG1127/2009
NA	CY061441	H1N1	06/26/2009	Argentina	A/Argentina/HNRG1131/2009
NA	CY061447	H1N1	06/26/2009	Argentina	A/Argentina/HNRG1137/2009
NA	CY061434	H1N1	06/26/2009	Argentina	A/Argentina/HNRG1124/2009
NA	CY061440	H1N1	06/26/2009	Argentina	A/Argentina/HNRG1130/2009
NA	CY046929	H1N1	06/26/2009	Chile	A/Santiago/2968/2009(H1N1)
NA	GQ355296	H1N1	06/26/2009	Spain	A/Catalonia/386/2009(H1N1)
NA	GQ355297	H1N1	06/26/2009	Spain	A/Catalonia/387/2009(H1N1)
NA	CY061432	H1N1	06/26/2009	Argentina	A/Argentina/HNRG1122/2009
NA	CY054757	H1N1	06/26/2009	USA	A/California/VRDL19/2009(H1N1)
NA	CY124037	H1N1	06/27/2009	Singapore	A/Singapore/ON580/2009
NA	CY061452	H1N1	06/27/2009	Argentina	A/Argentina/HNRG1142/2009
NA	CY051209	H1N1	06/27/2009	USA	A/Wisconsin/629-D01414/2009(H1N1)
NA	CY083457	H1N1	06/27/2009	Peru	A/Lima/WRAIR1687P/2009
NA	CY124030	H1N1	06/27/2009	Singapore	A/Singapore/ON575/2009
NA	CY053960	H1N1	06/27/2009	Argentina	A/Argentina/HNRG42/2009
NA	CY073990	H1N1	06/27/2009	Argentina	A/Argentina/HNRG106/2009
NA	CY083585	H1N1	06/27/2009	Peru	A/Lima/WRAIR8689F/2009(H1N1)
NA	HQ256609	H1N1	06/27/2009	Myanmar	A/Myanmar/M061/2009(H1N1)
NA	CY049198	H1N1	06/27/2009	Singapore	A/Singapore/ON621/2009(H1N1)
NA	CY049206	H1N1	06/27/2009	Singapore	A/Singapore/ON628/2009(H1N1)
NA	CY049214	H1N1	06/27/2009	Singapore	A/Singapore/ON635/2009(H1N1)
NA	HQ239946	H1N1	06/27/2009	Canada	A/Canada-NFL/RV2506/2009
NA	GU290049	H1N1	06/27/2009	Czech Rep	A/Prague/197-81395/2009(H1N1)
NA	CY061448	H1N1	06/27/2009	Argentina	A/Argentina/HNRG1138/2009
NA	CY061449	H1N1	06/27/2009	Argentina	A/Argentina/HNRG1139/2009
NA	CY061450	H1N1	06/27/2009	Argentina	A/Argentina/HNRG1140/2009
NA	CY061451	H1N1	06/27/2009	Argentina	A/Argentina/HNRG1141/2009
NA	CY049222	H1N1	06/27/2009	Singapore	A/Singapore/ON639/2009(H1N1)
NA	CY054813	H1N1	06/27/2009	USA	A/California/VRDL27/2009(H1N1)
NA	CY054837	H1N1	06/28/2009	USA	A/California/VRDL30/2009(H1N1)
NA	HQ247626	H1N1	06/28/2009	Finland	A/Finland/593/2009
NA	KT181408	H1N1	06/28/2009	Singapore	A/Singapore/DMS7/2009
NA	CY061453	H1N1	06/28/2009	Argentina	A/Argentina/HNRG1143/2009
NA	CY046953	H1N1	06/28/2009	Norway	A/Norway/2010/2009(H1N1)

NA	CY052188	H1N1	06/28/2009	USA	A/Texas/42281289/2009(H1N1)
NA	CY052087	H1N1	06/28/2009	Italy	A/Italy/228/2009(H1N1)
NA	CY123356	H1N1	06/29/2009	Singapore	A/Singapore/GP835/2009
NA	GQ355302	H1N1	06/29/2009	Spain	A/Catalonia/399/2009(H1N1)
NA	CY049997	H1N1	06/29/2009	Mexico	A/Mexico City/WR1100N/2009(H1N1)
NA	CY058182	H1N1	06/29/2009	Nicaragua	A/Managua/3189.01/2009(H1N1)
NA	CY052401	H1N1	06/29/2009	USA	A/Texas/42291877/2009(H1N1)
NA	CY058198	H1N1	06/29/2009	Nicaragua	A/Managua/1127.02/2009(H1N1)
NA	GQ463958	H1N1	06/29/2009	China	A/Changsha/78/2009(H1N1)
NA	CY063560	H1N1	06/29/2009	USA	A/Boston/152/2009(H1N1)
NA	GU014769	H1N1	06/29/2009	Japan	A/Saitama/85/2009(H1N1)
NA	CY067808	H1N1	06/29/2009	Portugal	A/Lisboa/34/2009(H1N1)
NA	CY067871	H1N1	06/29/2009	Portugal	A/Lisboa/73/2009(H1N1)
NA	CY043080	H1N1	06/29/2009	Japan	A/Japan/1070/2009(H1N1)
NA	CY053908	H1N1	06/29/2009	Argentina	A/Argentina/HNRG16/2009
NA	HE804129	H1N1	06/29/2009	Uruguay	A/Uruguay/369/2009
NA	CY052088	H1N1	06/29/2009	Italy	A/Italy/229/2009(H1N1)
NA	CY064510	H1N1	06/29/2009	USA	A/Boston/116/2009(H1N1)
NA	CY061454	H1N1	06/29/2009	Argentina	A/Argentina/HNRG1144/2009
NA	CY061455	H1N1	06/29/2009	Argentina	A/Argentina/HNRG1145/2009
NA	CY061456	H1N1	06/29/2009	Argentina	A/Argentina/HNRG1146/2009
NA	CY061457	H1N1	06/29/2009	Argentina	A/Argentina/HNRG1147/2009
NA	CY061458	H1N1	06/29/2009	Argentina	A/Argentina/HNRG1148/2009
NA	CY061459	H1N1	06/29/2009	Argentina	A/Argentina/HNRG1149/2009
NA	GQ375278	H1N1	06/29/2009	Ireland	A/Dublin/11/2009(H1N1)
NA	GQ355298	H1N1	06/29/2009	Spain	A/Catalonia/390/2009(H1N1)
NA	GQ355299	H1N1	06/29/2009	Spain	A/Catalonia/394/2009(H1N1)
NA	GQ355300	H1N1	06/29/2009	Spain	A/Catalonia/397/2009(H1N1)
NA	GQ355301	H1N1	06/29/2009	Spain	A/Catalonia/398/2009(H1N1)
NA	CY054789	H1N1	06/29/2009	USA	A/California/VRDL23/2009(H1N1)
NA	CY049413	H1N1	06/30/2009	Singapore	A/Singapore/GP875/2009(H1N1)
NA	CY054869	H1N1	06/30/2009	USA	A/California/VRDL34/2009(H1N1)
NA	CY061465	H1N1	06/30/2009	Argentina	A/Argentina/HNRG1155/2009
NA	CY061463	H1N1	06/30/2009	Argentina	A/Argentina/HNRG1153/2009
NA	GQ379825	H1N1	06/30/2009	Spain	A/Catalonia/403/2009(H1N1)
NA	GQ379826	H1N1	06/30/2009	Spain	A/Catalonia/405/2009(H1N1)
NA	CY046932	H1N1	06/30/2009	Chile	A/Santiago/3221/2009(H1N1)
NA	GQ375279	H1N1	06/30/2009	Ireland	A/Dublin/12/2009(H1N1)
NA	CY061464	H1N1	06/30/2009	Argentina	A/Argentina/HNRG1154/2009
NA	CY075828	H1N1	06/30/2009	Thailand	A/Thailand/CU-H75/2009(H1N1)
NA	CY061470	H1N1	06/30/2009	Argentina	A/Argentina/HNRG1160/2009
NA	CY061472	H1N1	06/30/2009	Argentina	A/Argentina/HNRG1162/2009
NA	CY061475	H1N1	06/30/2009	Argentina	A/Argentina/HNRG1165/2009
NA	CY061474	H1N1	06/30/2009	Argentina	A/Argentina/HNRG1164/2009
NA	CY061478	H1N1	06/30/2009	Argentina	A/Argentina/HNRG1168/2009
NA	CY061467	H1N1	06/30/2009	Argentina	A/Argentina/HNRG1157/2009
NA	CY061461	H1N1	06/30/2009	Argentina	A/Argentina/HNRG1151/2009
NA	CY061477	H1N1	06/30/2009	Argentina	A/Argentina/HNRG1167/2009
NA	CY061476	H1N1	06/30/2009	Argentina	A/Argentina/HNRG1166/2009
NA	CY061480	H1N1	06/30/2009	Argentina	A/Argentina/HNRG1170/2009
NA	CY061479	H1N1	06/30/2009	Argentina	A/Argentina/HNRG1169/2009
NA	CY061469	H1N1	06/30/2009	Argentina	A/Argentina/HNRG1159/2009
NA	CY061471	H1N1	06/30/2009	Argentina	A/Argentina/HNRG1161/2009
NA	CY061473	H1N1	06/30/2009	Argentina	A/Argentina/HNRG1163/2009

NA	CY061466	H1N1	06/30/2009	Argentina	A/Argentina/HNRG1156/2009
NA	CY061468	H1N1	06/30/2009	Argentina	A/Argentina/HNRG1158/2009
NA	CY056258	H1N1	06/30/2009	Argentina	A/Buenos Aires/HIFJM239859/2009(H1N
NA	CY061460	H1N1	06/30/2009	Argentina	A/Argentina/HNRG1150/2009
NA	CY064686	H1N1	06/30/2009	USA	A/Boston/142/2009(H1N1)
NA	CY058190	H1N1	06/30/2009	Nicaragua	A/Managua/5586.01/2009(H1N1)
NA	CY073420	H1N1	06/30/2009	USA	A/San Diego/WR1640P/2009(H1N1)
NA	GU065291	H1N1	06/30/2009	Poland	A/Poland/253/2009(H1N1)
NA	GU014785	H1N1	06/30/2009	Japan	A/Hiroshima/216/2009(H1N1)
NA	CY053963	H1N1	06/30/2009	Argentina	A/Argentina/HNRG44/2009
NA	CY075301	H1N1	06/30/2009	Chile	A/Chile/3220/2009(H1N1)
NA	CY075317	H1N1	06/30/2009	Chile	A/Chile/3244/2009(H1N1)
NA	CY075325	H1N1	06/30/2009	Chile	A/Chile/3295/2009(H1N1)
NA	CY075309	H1N1	06/30/2009	Chile	A/Chile/3242/2009(H1N1)
NA	CY051489	H1N1	06/30/2009	USA	A/Wisconsin/629-S0464/2009(H1N1)
NA	CY047755	H1N1	06/30/2009	Argentina	A/Argentina/12508/2009
NA	JN256859	H1N1	06/30/2009	Hong Kong	A/Hong Kong/H090-663-V1(0)/2009
NA	CY051217	H1N1	06/30/2009	USA	A/Wisconsin/629-D02292/2009(H1N1)
NA	CY051649	H1N1	06/30/2009	USA	A/New York/4691/2009(H1N1)
NA	CY047753	H1N1	06/30/2009	Argentina	A/Argentina/12295/2009
NA	CY047761	H1N1	06/30/2009	Argentina	A/Argentina/13777/2009
NA	CY111621	H1N1	06/30/2009	Hong Kong	A/Hong Kong/H090-663-V10/2009
NA	CY120103	H1N1	06/30/2009	Hong Kong	A/Hong Kong/H090-663-V10/2009
NA	CY123364	H1N1	06/30/2009	Singapore	A/Singapore/GP875/2009
NA	CY054797	H1N1	06/30/2009	USA	A/California/VRDL25/2009(H1N1)
NA	CY069751	H1N1	06/30/2009	United Ki	A/England/527/2009
NA	CY069759	H1N1	06/30/2009	United Ki	A/England/633/2009
NA	JX625397	H1N1	06/30/2009	United Ki	A/England/527/2009
NA	CY052204	H1N1	06/30/2009	USA	A/Texas/42303371/2009(H1N1)
NA	CY061462	H1N1	06/30/2009	Argentina	A/Argentina/HNRG1152/2009
NA	GU134729	H1N1	07/2009	Italy	A/Italy/176/2009(H1N1)
NA	GU134730	H1N1	07/2009	Italy	A/Italy/177/2009(H1N1)
NA	CY064385	H1N1	07/2009	China	A/Guangdong/936/2009
NA	CY064379	H1N1	07/2009	China	A/Guangdong/912/2009
NA	HM598324	H1N1	07/2009	Thailand	A/Thailand/CU-MV11/2009
NA	GU123923	H1N1	07/2009	Italy	A/Italy/156/2009(H1N1)
NA	GU123924	H1N1	07/2009	Italy	A/Italy/157/2009(H1N1)
NA	GU123902	H1N1	07/2009	Italy	A/Italy/135/2009(H1N1)
NA	GU123903	H1N1	07/2009	Italy	A/Italy/136/2009(H1N1)
NA	GU123904	H1N1	07/2009	Italy	A/Italy/137/2009(H1N1)
NA	GU123905	H1N1	07/2009	Italy	A/Italy/138/2009(H1N1)
NA	GU123906	H1N1	07/2009	Italy	A/Italy/139/2009(H1N1)
NA	GU123907	H1N1	07/2009	Italy	A/Italy/140/2009(H1N1)
NA	GU123912	H1N1	07/2009	Italy	A/Italy/158/2009(H1N1)
NA	GU123913	H1N1	07/2009	Italy	A/Italy/146/2009(H1N1)
NA	GU123916	H1N1	07/2009	Italy	A/Italy/149/2009(H1N1)
NA	GU123917	H1N1	07/2009	Italy	A/Italy/150/2009(H1N1)
NA	GU123918	H1N1	07/2009	Italy	A/Italy/151/2009(H1N1)
NA	GU123920	H1N1	07/2009	Italy	A/Italy/153/2009(H1N1)
NA	GU123921	H1N1	07/2009	Italy	A/Italy/154/2009(H1N1)
NA	GU123922	H1N1	07/2009	Italy	A/Italy/155/2009(H1N1)
NA	AB568179	H1N1	07/2009	Japan	A/Kobe/91559/2009
NA	GU983244	H1N1	07/2009	India	A/Eastern India/66/2009
NA	AB568174	H1N1	07/2009	Japan	A/Kobe/91537/2009

NA	GU134724	H1N1	07/2009	Italy	A/Italy/171/2009(H1N1)
NA	JN381356	H1N1	07/2009	Taiwan	A/Taiwan/97338/2009
NA	JN381353	H1N1	07/2009	Taiwan	A/Taiwan/71354/2009
NA	CY047746	H1N1	07/2009	Taiwan	A/Taiwan/526/2009
NA	JN381355	H1N1	07/2009	Taiwan	A/Taiwan/71526/2009
NA	GU134716	H1N1	07/2009	Italy	A/Italy/163/2009(H1N1)
NA	GU134717	H1N1	07/2009	Italy	A/Italy/164/2009(H1N1)
NA	GU134718	H1N1	07/2009	Italy	A/Italy/165/2009(H1N1)
NA	GU134720	H1N1	07/2009	Italy	A/Italy/167/2009(H1N1)
NA	GU134721	H1N1	07/2009	Italy	A/Italy/168/2009(H1N1)
NA	GU123901	H1N1	07/2009	Italy	A/Italy/134/2009(H1N1)
NA	GU123908	H1N1	07/2009	Italy	A/Italy/141/2009(H1N1)
NA	GU123911	H1N1	07/2009	Italy	A/Italy/144/2009(H1N1)
NA	GU123914	H1N1	07/2009	Italy	A/Italy/147/2009(H1N1)
NA	GU123915	H1N1	07/2009	Italy	A/Italy/148/2009(H1N1)
NA	GU123919	H1N1	07/2009	Italy	A/Italy/152/2009(H1N1)
NA	AB572353	H1N1	07/2009	Zambia	A/Zambia/CZC1/2009(H1N1)
NA	JN381354	H1N1	07/2009	Taiwan	A/Taiwan/71433/2009
NA	GU292382	H1N1	07/2009	India	A/Blore/NIV310/2009(H1N1)
NA	GU123909	H1N1	07/2009	Italy	A/Italy/142/2009(H1N1)
NA	GU123910	H1N1	07/2009	Italy	A/Italy/143/2009(H1N1)
NA	CY050365	H1N1	07/2009	Singapore	A/Singapore/279M/2009(H1N1)
NA	GQ866936	H1N1	07/2009	Thailand	A/Thailand/CU-H106/2009
NA	GQ866937	H1N1	07/2009	Thailand	A/Thailand/CU-H276/2009
NA	CY064373	H1N1	07/2009	China	A/Guangdong/863/2009
NA	GU983245	H1N1	07/2009	India	A/Eastern India/67/2009
NA	CY064382	H1N1	07/2009	China	A/Guangdong/933/2009
NA	CY064376	H1N1	07/2009	China	A/Guangdong/898/2009
NA	HM598345	H1N1	07/2009	Thailand	A/Thailand/CU-MV40/2009
NA	HM598343	H1N1	07/2009	Thailand	A/Thailand/CU-MV36/2009
NA	HM598326	H1N1	07/2009	Thailand	A/Thailand/CU-MV14/2009
NA	HM598325	H1N1	07/2009	Thailand	A/Thailand/CU-MV12/2009
NA	HM598344	H1N1	07/2009	Thailand	A/Thailand/CU-MV38/2009
NA	AB568194	H1N1	07/2009	Japan	A/Kobe/91585/2009
NA	AB568209	H1N1	07/2009	Japan	A/Kobe/91660/2009
NA	AB568169	H1N1	07/2009	Japan	A/Kobe/91533/2009
NA	AB568199	H1N1	07/2009	Japan	A/Kobe/91586/2009
NA	AB568204	H1N1	07/2009	Japan	A/Kobe/91615/2009
NA	AB568189	H1N1	07/2009	Japan	A/Kobe/91571/2009
NA	AB568184	H1N1	07/2009	Japan	A/Kobe/91569/2009
NA	AB568214	H1N1	07/2009	Japan	A/Kobe/91709/2009
NA	KM035426	H1N1	07/2009	Brazil	A/Rio Grande do Sul/1829/2009
NA	KM035440	H1N1	07/2009	Brazil	A/Rio Grande do Sul/3017/2009
NA	KM035446	H1N1	07/2009	Brazil	A/Rio Grande do Sul/3570/2009
NA	KM035448	H1N1	07/2009	Brazil	A/Rio Grande do Sul/3908/2009
NA	KM035428	H1N1	07/2009	Brazil	A/Rio Grande do Sul/1865/2009
NA	KM035445	H1N1	07/2009	Brazil	A/Rio Grande do Sul/3554/2009
NA	KM035427	H1N1	07/2009	Brazil	A/Rio Grande do Sul/1860/2009
NA	KM035429	H1N1	07/2009	Brazil	A/Rio Grande do Sul/2014/2009
NA	KM035432	H1N1	07/2009	Brazil	A/Rio Grande do Sul/2684/2009
NA	KM035441	H1N1	07/2009	Brazil	A/Rio Grande do Sul/3077/2009
NA	KM035453	H1N1	07/2009	Brazil	A/Rio Grande do Sul/5516/2009
NA	KM035425	H1N1	07/2009	Brazil	A/Rio Grande do Sul/1759/2009
NA	HM598327	H1N1	07/2009	Thailand	A/Thailand/CU-MV15/2009

NA	GU134712	H1N1	07/2009	Italy	A/Italy/159/2009(H1N1)
NA	GU134713	H1N1	07/2009	Italy	A/Italy/160/2009(H1N1)
NA	GU134714	H1N1	07/2009	Italy	A/Italy/161/2009(H1N1)
NA	GU134715	H1N1	07/2009	Italy	A/Italy/162/2009(H1N1)
NA	GU134719	H1N1	07/2009	Italy	A/Italy/166/2009(H1N1)
NA	GU134722	H1N1	07/2009	Italy	A/Italy/169/2009(H1N1)
NA	GU134723	H1N1	07/2009	Italy	A/Italy/170/2009(H1N1)
NA	GU134725	H1N1	07/2009	Italy	A/Italy/182/2009(H1N1)
NA	GU134726	H1N1	07/2009	Italy	A/Italy/173/2009(H1N1)
NA	GU134727	H1N1	07/2009	Italy	A/Italy/174/2009(H1N1)
NA	GU134728	H1N1	07/2009	Italy	A/Italy/175/2009(H1N1)
NA	GQ379837	H1N1	07/01/2009	Spain	A/Catalonia/467/2009(H1N1)
NA	GQ379838	H1N1	07/01/2009	Spain	A/Catalonia/470/2009(H1N1)
NA	CY065176	H1N1	07/01/2009	United Ki	A/England/634/2009
NA	AB649906	H1N1	07/01/2009	Japan	A/Toyama/248/2009
NA	CY107813	H1N1	07/01/2009	United Ki	A/Scotland/EastKilbride_425383/2009
NA	JX625421	H1N1	07/01/2009	United Ki	A/England/667/2009
NA	JX625405	H1N1	07/01/2009	United Ki	A/England/685/2009
NA	JX625429	H1N1	07/01/2009	United Ki	A/England/92920018/2009
NA	KP637528	H1N1	07/01/2009	Thailand	A/Thailand/SN08270/2009
NA	CY051833	H1N1	07/01/2009	USA	A/Texas/43011033/2009(H1N1)
NA	CY054853	H1N1	07/01/2009	USA	A/California/VRDL32/2009(H1N1)
NA	CY054949	H1N1	07/01/2009	USA	A/California/VRDL50/2009(H1N1)
NA	CY054933	H1N1	07/01/2009	USA	A/California/VRDL45/2009(H1N1)
NA	CY054909	H1N1	07/01/2009	USA	A/California/VRDL40/2009(H1N1)
NA	CY075349	H1N1	07/01/2009	Chile	A/Chile/3369/2009(H1N1)
NA	CY053914	H1N1	07/01/2009	Argentina	A/Argentina/HNRG18/2009
NA	CY051577	H1N1	07/01/2009	USA	A/New York/4500/2009(H1N1)
NA	CY051569	H1N1	07/01/2009	USA	A/New York/4476/2009(H1N1)
NA	GQ421202	H1N1	07/01/2009	Italy	A/Ancona/04/2009(H1N1)
NA	CY047765	H1N1	07/01/2009	Argentina	A/Argentina/14044/2009
NA	CY047767	H1N1	07/01/2009	Argentina	A/Argentina/14192/2009
NA	HQ240004	H1N1	07/01/2009	Canada	A/Canada-BC/RV2933/2009
NA	GU014783	H1N1	07/01/2009	Japan	A/Hiroshima/207/2009(H1N1)
NA	CY047773	H1N1	07/01/2009	Argentina	A/Argentina/15765/2009
NA	CY053920	H1N1	07/01/2009	Argentina	A/Argentina/HNRG21/2009(H1N1)
NA	KF612132	H1N1	07/01/2009	Bolivia	A/Santa Cruz/12541/2009
NA	CY073412	H1N1	07/01/2009	USA	A/San Diego/WR1639P/2009(H1N1)
NA	CY063782	H1N1	07/01/2009	Singapore	A/Singapore/ON0801/2009(H1N1)
NA	GU065292	H1N1	07/01/2009	Poland	A/Poland/266/2009(H1N1)
NA	CY083593	H1N1	07/01/2009	Peru	A/Lima/WRAIR8893F/2009(H1N1)
NA	KF612136	H1N1	07/01/2009	Bolivia	A/Santa Cruz/21641/2009
NA	CY075333	H1N1	07/01/2009	Chile	A/Chile/3349/2009(H1N1)
NA	CY075341	H1N1	07/01/2009	Chile	A/Chile/3361/2009(H1N1)
NA	CY061482	H1N1	07/01/2009	Argentina	A/Argentina/HNRG1172/2009
NA	CY061490	H1N1	07/01/2009	Argentina	A/Argentina/HNRG1180/2009
NA	CY056259	H1N1	07/01/2009	Argentina	A/Buenos Aires/HIFJM240046/2009(H1N
NA	CY075357	H1N1	07/01/2009	Chile	A/Chile/3375/2009(H1N1)
NA	CY107460	H1N1	07/01/2009	United Ki	A/Scotland/Dumfries_425403/2009
NA	CY063544	H1N1	07/01/2009	USA	A/Boston/149/2009(H1N1)
NA	CY063520	H1N1	07/01/2009	USA	A/Boston/146/2009(H1N1)
NA	GQ465696	H1N1	07/01/2009	Canada	A/Canada-SK/RV2486/2009(H1N1)
NA	CY047771	H1N1	07/01/2009	Argentina	A/Argentina/14591/2009
NA	CY061481	H1N1	07/01/2009	Argentina	A/Argentina/HNRG1171/2009

NA	CY061489	H1N1	07/01/2009	Argentina	A/Argentina/HNRG1179/2009
NA	CY061486	H1N1	07/01/2009	Argentina	A/Argentina/HNRG1176/2009
NA	CY047111	H1N1	07/01/2009	Spain	A/Catalonia/S1068/2009(H1N1)
NA	GQ379827	H1N1	07/01/2009	Spain	A/Catalonia/408/2009(H1N1)
NA	GQ379828	H1N1	07/01/2009	Spain	A/Catalonia/410/2009(H1N1)
NA	CY075840	H1N1	07/01/2009	Thailand	A/Thailand/CU-H88/2009
NA	CY061483	H1N1	07/01/2009	Argentina	A/Argentina/HNRG1173/2009
NA	CY061484	H1N1	07/01/2009	Argentina	A/Argentina/HNRG1174/2009
NA	CY061485	H1N1	07/01/2009	Argentina	A/Argentina/HNRG1175/2009
NA	CY061488	H1N1	07/01/2009	Argentina	A/Argentina/HNRG1178/2009
NA	GQ387385	H1N1	07/01/2009	Spain	A/Catalonia/432/2009(H1N1)
NA	GQ387386	H1N1	07/01/2009	Spain	A/Catalonia/434/2009(H1N1)
NA	GQ387387	H1N1	07/01/2009	Spain	A/Catalonia/446/2009(H1N1)
NA	CY075834	H1N1	07/01/2009	Thailand	A/Thailand/CU-H85/2009(H1N1)
NA	CY061487	H1N1	07/01/2009	Argentina	A/Argentina/HNRG1177/2009
NA	GQ379829	H1N1	07/01/2009	Spain	A/Catalonia/412/2009(H1N1)
NA	GQ379830	H1N1	07/01/2009	Spain	A/Catalonia/414/2009(H1N1)
NA	GQ379831	H1N1	07/01/2009	Spain	A/Catalonia/417/2009(H1N1)
NA	GQ379832	H1N1	07/01/2009	Spain	A/Catalonia/423/2009(H1N1)
NA	GQ379833	H1N1	07/01/2009	Spain	A/Catalonia/442/2009(H1N1)
NA	GQ379834	H1N1	07/01/2009	Spain	A/Catalonia/443/2009(H1N1)
NA	GQ379835	H1N1	07/01/2009	Spain	A/Catalonia/456/2009(H1N1)
NA	GQ379836	H1N1	07/01/2009	Spain	A/Catalonia/458/2009(H1N1)
NA	CY111629	H1N1	07/02/2009	Hong Kong	A/Hong Kong/H090-665-V10/2009
NA	GQ379843	H1N1	07/02/2009	Spain	A/Catalonia/480/2009(H1N1)
NA	CY120119	H1N1	07/02/2009	Hong Kong	A/Hong Kong/H090-667-V10/2009
NA	CY124089	H1N1	07/02/2009	Singapore	A/Singapore/ON806/2009(H1N1)
NA	CY124069	H1N1	07/02/2009	Singapore	A/Singapore/ON803/2009(H1N1)
NA	CY124061	H1N1	07/02/2009	Singapore	A/Singapore/ON802/2009(H1N1)
NA	CY124125	H1N1	07/02/2009	Singapore	A/Singapore/ON812/2009
NA	CY124109	H1N1	07/02/2009	Singapore	A/Singapore/ON809/2009
NA	CY124073	H1N1	07/02/2009	Singapore	A/Singapore/ON804/2009
NA	CY124101	H1N1	07/02/2009	Singapore	A/Singapore/ON808/2009
NA	CY124117	H1N1	07/02/2009	Singapore	A/Singapore/ON811/2009
NA	CY124045	H1N1	07/02/2009	Singapore	A/Singapore/ON799/2009
NA	CY124053	H1N1	07/02/2009	Singapore	A/Singapore/ON800/2009
NA	KP637792	H1N1	07/02/2009	Thailand	A/Thailand/SS08191/2009
NA	CY054893	H1N1	07/02/2009	USA	A/California/VRDL38/2009(H1N1)
NA	CY124081	H1N1	07/02/2009	Singapore	A/Singapore/ON805/2009(H1N1)
NA	CY124097	H1N1	07/02/2009	Singapore	A/Singapore/ON807/2009(H1N1)
NA	CY075365	H1N1	07/02/2009	Chile	A/Chile/3467/2009(H1N1)
NA	CY075373	H1N1	07/02/2009	Chile	A/Chile/3553/2009(H1N1)
NA	CY073997	H1N1	07/02/2009	Argentina	A/Argentina/HNRG107/2009
NA	CY049957	H1N1	07/02/2009	Dominican	A/Santo Domingo/WR1058N/2009(H1N1)
NA	JN256861	H1N1	07/02/2009	Hong Kong	A/Hong Kong/H090-667-V1(0)/2009
NA	JN256860	H1N1	07/02/2009	Hong Kong	A/Hong Kong/H090-665-V1(0)/2009
NA	JN256862	H1N1	07/02/2009	Hong Kong	A/Hong Kong/H090-667-V2(3)/2009
NA	CY111645	H1N1	07/02/2009	Hong Kong	A/Hong Kong/H090-667-V23/2009
NA	HQ239938	H1N1	07/02/2009	Canada	A/Canada-NB/RV2478/2009
NA	CY067874	H1N1	07/02/2009	Portugal	A/Lisboa/74/2009(H1N1)
NA	CY067811	H1N1	07/02/2009	Portugal	A/Lisboa/35/2009(H1N1)
NA	CY111637	H1N1	07/02/2009	Hong Kong	A/Hong Kong/H090-667-V10/2009
NA	GU271960	H1N1	07/02/2009	Thailand	A/Thailand/CU-B267/2009(H1N1)
NA	CY055197	H1N1	07/02/2009	Malaysia	A/Malaysia/3758/2009

NA	CY072760	H1N1	07/02/2009	Nicaragua	A/Managua/435.01/2009(H1N1)
NA	KF612134	H1N1	07/02/2009	Bolivia	A/Santa Cruz/94841/2009
NA	CY072776	H1N1	07/02/2009	Nicaragua	A/Managua/5235.03/2009(H1N1)
NA	CY061496	H1N1	07/02/2009	Argentina	A/Argentina/HNRG1186/2009
NA	CY061498	H1N1	07/02/2009	Argentina	A/Argentina/HNRG1188/2009
NA	HQ239935	H1N1	07/02/2009	Canada	A/Canada-NB/RV2474/2009
NA	CY056260	H1N1	07/02/2009	Argentina	A/Buenos Aires/HIFJM240104/2009(H1N
NA	CY056261	H1N1	07/02/2009	Argentina	A/Buenos Aires/HIFJM240169/2009(H1N
NA	CY049949	H1N1	07/02/2009	Dominican	A/Santo Domingo/WR1057N/2009(H1N1)
NA	CY054861	H1N1	07/02/2009	USA	A/California/VRDL33/2009(H1N1)
NA	CY061493	H1N1	07/02/2009	Argentina	A/Argentina/HNRG1183/2009
NA	CY061497	H1N1	07/02/2009	Argentina	A/Argentina/HNRG1187/2009
NA	CY061499	H1N1	07/02/2009	Argentina	A/Argentina/HNRG1189/2009
NA	CY075846	H1N1	07/02/2009	Thailand	A/Thailand/CU-H94/2009(H1N1)
NA	CY075673	H1N1	07/02/2009	Thailand	A/Thailand/CU-H104/2009(H1N1)
NA	CY061491	H1N1	07/02/2009	Argentina	A/Argentina/HNRG1181/2009
NA	CY061492	H1N1	07/02/2009	Argentina	A/Argentina/HNRG1182/2009
NA	CY061494	H1N1	07/02/2009	Argentina	A/Argentina/HNRG1184/2009
NA	CY061495	H1N1	07/02/2009	Argentina	A/Argentina/HNRG1185/2009
NA	GQ379839	H1N1	07/02/2009	Spain	A/Catalonia/475/2009(H1N1)
NA	GQ379840	H1N1	07/02/2009	Spain	A/Catalonia/476/2009(H1N1)
NA	GQ379841	H1N1	07/02/2009	Spain	A/Catalonia/478/2009(H1N1)
NA	GQ379842	H1N1	07/02/2009	Spain	A/Catalonia/479/2009(H1N1)
NA	CY120111	H1N1	07/02/2009	Hong Kong	A/Hong Kong/H090-665-V10/2009
NA	CY124133	H1N1	07/03/2009	Singapore	A/Singapore/ON816/2009
NA	GQ379849	H1N1	07/03/2009	Spain	A/Catalonia/497/2009(H1N1)
NA	HQ247627	H1N1	07/03/2009	Finland	A/Finland/594/2009
NA	HQ247630	H1N1	07/03/2009	Finland	A/Finland/597/2009
NA	CY083617	H1N1	07/03/2009	Peru	A/Lima/WRAIR9202F/2009(H1N1)
NA	CY083465	H1N1	07/03/2009	Peru	A/Lima/WRAIR1688P/2009
NA	CY122557	H1N1	07/03/2009	Singapore	A/Singapore/GP1020/2009
NA	CY124148	H1N1	07/03/2009	Singapore	A/Singapore/ON822/2009
NA	HQ240005	H1N1	07/03/2009	Canada	A/Canada-BC/RV2934/2009
NA	HQ239933	H1N1	07/03/2009	Canada	A/Canada-NB/RV2457/2009
NA	HQ239934	H1N1	07/03/2009	Canada	A/Canada-NB/RV2458/2009
NA	GU014787	H1N1	07/03/2009	Japan	A/Hiroshima/220/2009(H1N1)
NA	CY087026	H1N1	07/03/2009	USA	A/New York/4557/2009(H1N1)
NA	CY061500	H1N1	07/03/2009	Argentina	A/Argentina/HNRG1190/2009
NA	CY061501	H1N1	07/03/2009	Argentina	A/Argentina/HNRG1191/2009
NA	CY061505	H1N1	07/03/2009	Argentina	A/Argentina/HNRG1195/2009
NA	CY056262	H1N1	07/03/2009	Argentina	A/Buenos Aires/HIFJM240220/2009(H1N
NA	GQ502908	H1N1	07/03/2009	Canada	A/Toronto/R8557/2009(H1N1)
NA	GQ379850	H1N1	07/03/2009	Spain	A/Catalonia/498/2009(H1N1)
NA	GQ379851	H1N1	07/03/2009	Spain	A/Catalonia/500/2009(H1N1)
NA	GQ379852	H1N1	07/03/2009	Spain	A/Catalonia/502/2009(H1N1)
NA	GQ379853	H1N1	07/03/2009	Spain	A/Catalonia/503/2009(H1N1)
NA	GQ379854	H1N1	07/03/2009	Spain	A/Catalonia/508/2009(H1N1)
NA	GQ379855	H1N1	07/03/2009	Spain	A/Catalonia/510/2009(H1N1)
NA	CY061502	H1N1	07/03/2009	Argentina	A/Argentina/HNRG1192/2009
NA	CY061503	H1N1	07/03/2009	Argentina	A/Argentina/HNRG1193/2009
NA	CY061504	H1N1	07/03/2009	Argentina	A/Argentina/HNRG1194/2009
NA	GQ379858	H1N1	07/03/2009	Spain	A/Catalonia/519/2009(H1N1)
NA	GQ379859	H1N1	07/03/2009	Spain	A/Catalonia/521/2009(H1N1)
NA	GQ379860	H1N1	07/03/2009	Spain	A/Catalonia/522/2009(H1N1)

NA	HE804118	H1N1	07/03/2009	Uruguay	A/Uruguay/18/2009
NA	CY075679	H1N1	07/03/2009	Thailand	A/Thailand/CU-H109/2009(H1N1)
NA	GQ379856	H1N1	07/03/2009	Spain	A/Catalonia/517/2009(H1N1)
NA	GQ379857	H1N1	07/03/2009	Spain	A/Catalonia/518/2009(H1N1)
NA	CY048995	H1N1	07/03/2009	Spain	A/Catalonia/NS508/2009(H1N1)
NA	GQ379861	H1N1	07/03/2009	Spain	A/Catalonia/523/2009(H1N1)
NA	GQ379862	H1N1	07/03/2009	Spain	A/Catalonia/551/2009(H1N1)
NA	GQ379844	H1N1	07/03/2009	Spain	A/Catalonia/481/2009(H1N1)
NA	GQ379845	H1N1	07/03/2009	Spain	A/Catalonia/482/2009(H1N1)
NA	GQ379846	H1N1	07/03/2009	Spain	A/Catalonia/483/2009(H1N1)
NA	GQ379847	H1N1	07/03/2009	Spain	A/Catalonia/490/2009(H1N1)
NA	GQ379848	H1N1	07/03/2009	Spain	A/Catalonia/496/2009(H1N1)
NA	CY075381	H1N1	07/03/2009	Chile	A/Chile/3586/2009(H1N1)
NA	CY062518	H1N1	07/04/2009	Mexico	A/Mexico city/CIA5/2009
NA	GQ387391	H1N1	07/04/2009	Spain	A/Catalonia/565/2009(H1N1)
NA	HQ247629	H1N1	07/04/2009	Finland	A/Finland/596/2009
NA	HQ247628	H1N1	07/04/2009	Finland	A/Finland/595/2009
NA	GU112751	H1N1	07/04/2009	Poland	A/Poland/274/2009(H1N1)
NA	CY055201	H1N1	07/04/2009	Malaysia	A/Malaysia/4039/2009
NA	HQ256613	H1N1	07/04/2009	Myanmar	A/Myanmar/M121/2009(H1N1)
NA	CY061508	H1N1	07/04/2009	Argentina	A/Argentina/HNRG1198/2009
NA	CY061506	H1N1	07/04/2009	Argentina	A/Argentina/HNRG1196/2009
NA	HQ239936	H1N1	07/04/2009	Canada	A/Canada-NB/RV2475/2009
NA	HQ239944	H1N1	07/04/2009	Canada	A/Canada-NFL/RV2504/2009
NA	CY052352	H1N1	07/04/2009	United Ar	A/UAE/B06/2009(H1N1)
NA	CY049421	H1N1	07/04/2009	Singapore	A/Singapore/ON903/2009(H1N1)
NA	CY049230	H1N1	07/04/2009	Singapore	A/Singapore/ON894/2009(H1N1)
NA	CY049238	H1N1	07/04/2009	Singapore	A/Singapore/ON896/2009(H1N1)
NA	CY049246	H1N1	07/04/2009	Singapore	A/Singapore/ON899/2009(H1N1)
NA	CY049437	H1N1	07/04/2009	Singapore	A/Singapore/ON907/2009(H1N1)
NA	CY049445	H1N1	07/04/2009	Singapore	A/Singapore/ON912/2009(H1N1)
NA	HE804130	H1N1	07/04/2009	Uruguay	A/Uruguay/479/2009
NA	CY055199	H1N1	07/04/2009	Malaysia	A/Malaysia/4038/2009
NA	CY062492	H1N1	07/04/2009	Mexico	A/Mexico city/CIA1/2009(H1N1)
NA	CY061507	H1N1	07/04/2009	Argentina	A/Argentina/HNRG1197/2009
NA	HE804131	H1N1	07/04/2009	Uruguay	A/Uruguay/495/2009
NA	CY075685	H1N1	07/04/2009	Thailand	A/Thailand/CU-H119/2009(H1N1)
NA	GQ387390	H1N1	07/04/2009	Spain	A/Catalonia/563/2009(H1N1)
NA	GQ387392	H1N1	07/04/2009	Spain	A/Catalonia/569/2009(H1N1)
NA	GQ387393	H1N1	07/04/2009	Spain	A/Catalonia/570/2009(H1N1)
NA	GQ387394	H1N1	07/04/2009	Spain	A/Catalonia/571/2009(H1N1)
NA	GQ387395	H1N1	07/04/2009	Spain	A/Catalonia/572/2009(H1N1)
NA	GQ387388	H1N1	07/04/2009	Spain	A/Catalonia/554/2009(H1N1)
NA	GQ387389	H1N1	07/04/2009	Spain	A/Catalonia/562/2009(H1N1)
NA	CY075691	H1N1	07/04/2009	Thailand	A/Thailand/CU-H122/2009(H1N1)
NA	CY049429	H1N1	07/04/2009	Singapore	A/Singapore/ON905/2009(H1N1)
NA	JX556110	H1N1	07/04/2009	Cuba	A/Habana/1156/2009
NA	CY049254	H1N1	07/04/2009	Singapore	A/Singapore/ON901/2009(H1N1)
NA	CY049118	H1N1	07/05/2009	Singapore	A/Singapore/ON923/2009(H1N1)
NA	KT181409	H1N1	07/05/2009	Singapore	A/Singapore/DMS10/2009
NA	JX625453	H1N1	07/05/2009	United Ki	A/England/671/2009
NA	CY054877	H1N1	07/05/2009	USA	A/California/VRDL35/2009(H1N1)
NA	CY122565	H1N1	07/05/2009	Singapore	A/Singapore/GP1022/2009
NA	CY061510	H1N1	07/05/2009	Argentina	A/Argentina/HNRG1201/2009

NA	CY061509	H1N1	07/05/2009	Argentina	A/Argentina/HNRG1199/2009
NA	CY061511	H1N1	07/05/2009	Argentina	A/Argentina/HNRG1201/2009
NA	HQ239952	H1N1	07/05/2009	Canada	A/Canada-NS/RV2598/2009
NA	HQ239937	H1N1	07/05/2009	Canada	A/Canada-NB/RV2476/2009
NA	CY049461	H1N1	07/05/2009	Singapore	A/Singapore/ON925/2009(H1N1)
NA	CY049453	H1N1	07/05/2009	Singapore	A/Singapore/ON921/2009(H1N1)
NA	HE804122	H1N1	07/05/2009	Uruguay	A/Uruguay/518/2009
NA	CY061541	H1N1	07/05/2009	Argentina	A/Argentina/HNRG84/2009
NA	GQ387396	H1N1	07/05/2009	Spain	A/Catalonia/580/2009(H1N1)
NA	GQ387397	H1N1	07/05/2009	Spain	A/Catalonia/586/2009(H1N1)
NA	CY061543	H1N1	07/05/2009	Argentina	A/Argentina/HNRG91/2009
NA	HM104437	H1N1	07/05/2009	Chile	A/Santiago/21579/2009(H1N1)
NA	CY049286	H1N1	07/06/2009	Singapore	A/Singapore/ON981/2009(H1N1)
NA	GQ387406	H1N1	07/06/2009	Spain	A/Catalonia/594/2009(H1N1)
NA	KC781608	H1N1	07/06/2009	USA	A/Arizona/14/2009
NA	CY122613	H1N1	07/06/2009	Singapore	A/Singapore/GP1099/2009
NA	CY054925	H1N1	07/06/2009	USA	A/California/VRDL43/2009(H1N1)
NA	CY122689	H1N1	07/06/2009	Singapore	A/Singapore/GP1137/2009
NA	CY122589	H1N1	07/06/2009	Singapore	A/Singapore/GP1092/2009
NA	CY075389	H1N1	07/06/2009	Chile	A/Chile/3760/2009(H1N1)
NA	CY075405	H1N1	07/06/2009	Chile	A/Chile/3819/2009(H1N1)
NA	CY075397	H1N1	07/06/2009	Chile	A/Chile/3766/2009(H1N1)
NA	CY122597	H1N1	07/06/2009	Singapore	A/Singapore/GP1094/2009
NA	CY122605	H1N1	07/06/2009	Singapore	A/Singapore/GP1097/2009
NA	CY083633	H1N1	07/06/2009	Peru	A/Arequipa/WRAIR9939F/2009
NA	HQ239945	H1N1	07/06/2009	Canada	A/Canada-NFL/RV2505/2009
NA	KF687911	H1N1	07/06/2009	Cyprus	A/Cyprus/050/2009
NA	KF687910	H1N1	07/06/2009	Cyprus	A/Cyprus/045/2009
NA	KF687912	H1N1	07/06/2009	Cyprus	A/Cyprus/059/2009
NA	KF687914	H1N1	07/06/2009	Cyprus	A/Cyprus/062/2009
NA	KF695073	H1N1	07/06/2009	Singapore	A/Singapore/376Y/2009
NA	CY062499	H1N1	07/06/2009	Mexico	A/Mexico city/CIA2/2009(H1N1)
NA	CY045513	H1N1	07/06/2009	Canada	A/Toronto/R8564/2009(H1N1)
NA	CY069252	H1N1	07/06/2009	Nicaragua	A/Managua/4943.09/2009(H1N1)
NA	CY072568	H1N1	07/06/2009	Nicaragua	A/Managua/4197.01/2009(H1N1)
NA	CY083346	H1N1	07/06/2009	USA	A/San Diego/WRAIR1656P/2009
NA	CY065168	H1N1	07/06/2009	United Ki	A/England/92860032/2009
NA	CY061512	H1N1	07/06/2009	Argentina	A/Argentina/HNRG1202/2009
NA	CY049262	H1N1	07/06/2009	Singapore	A/Singapore/ON975/2009(H1N1)
NA	CY049270	H1N1	07/06/2009	Singapore	A/Singapore/ON978/2009(H1N1)
NA	HE804120	H1N1	07/06/2009	Uruguay	A/Uruguay/549/2009
NA	CY063552	H1N1	07/06/2009	USA	A/Boston/151/2009(H1N1)
NA	CY062506	H1N1	07/06/2009	Mexico	A/Mexico city/CIA3/2009(H1N1)
NA	CY049278	H1N1	07/06/2009	Singapore	A/Singapore/ON980/2009(H1N1)
NA	CY061513	H1N1	07/06/2009	Argentina	A/Argentina/HNRG1203/2009
NA	CY075709	H1N1	07/06/2009	Thailand	A/Thailand/CU-H130/2009(H1N1)
NA	CY075697	H1N1	07/06/2009	Thailand	A/Thailand/CU-H126/2009(H1N1)
NA	CY075715	H1N1	07/06/2009	Thailand	A/Thailand/CU-H132/2009(H1N1)
NA	CY075721	H1N1	07/06/2009	Thailand	A/Thailand/CU-H133/2009(H1N1)
NA	GQ387398	H1N1	07/06/2009	Spain	A/Catalonia/596/2009(H1N1)
NA	GQ387399	H1N1	07/06/2009	Spain	A/Catalonia/597/2009(H1N1)
NA	GQ387400	H1N1	07/06/2009	Spain	A/Catalonia/598/2009(H1N1)
NA	GQ387402	H1N1	07/06/2009	Spain	A/Catalonia/604/2009(H1N1)
NA	GQ387403	H1N1	07/06/2009	Spain	A/Catalonia/607/2009(H1N1)

NA	CY075703	H1N1	07/06/2009	Thailand	A/Thailand/CU-H128/2009(H1N1)
NA	GQ387401	H1N1	07/06/2009	Spain	A/Catalonia/600/2009(H1N1)
NA	KC781693	H1N1	07/06/2009	USA	A/Georgia/08/2009
NA	CY053160	H1N1	07/07/2009	USA	A/Houston/23H/2009(H1N1)
NA	CY046939	H1N1	07/07/2009	Chile	A/Santiago/3837/2009(H1N1)
NA	CY120127	H1N1	07/07/2009	Hong Kong	A/Hong Kong/H090-678-V10/2009
NA	CY122649	H1N1	07/07/2009	Singapore	A/Singapore/GP1122/2009
NA	CY111653	H1N1	07/07/2009	Hong Kong	A/Hong Kong/H090-678-V10/2009
NA	CY075421	H1N1	07/07/2009	Chile	A/Chile/3935/2009(H1N1)
NA	HQ256611	H1N1	07/07/2009	Myanmar	A/Myanmar/M097/2009(H1N1)
NA	HQ247631	H1N1	07/07/2009	Finland	A/Finland/598/2009
NA	CY088682	H1N1	07/07/2009	India	A/Mum/NIV398/2009(H1N1)
NA	JN256863	H1N1	07/07/2009	Hong Kong	A/Hong Kong/H090-678-V1(0)/2009
NA	CY122573	H1N1	07/07/2009	Singapore	A/Singapore/GP1087/2009
NA	CY122621	H1N1	07/07/2009	Singapore	A/Singapore/GP1102/2009
NA	CY122581	H1N1	07/07/2009	Singapore	A/Singapore/GP1090/2009
NA	CY122657	H1N1	07/07/2009	Singapore	A/Singapore/GP1124/2009
NA	CY122681	H1N1	07/07/2009	Singapore	A/Singapore/GP1136/2009
NA	GU014767	H1N1	07/07/2009	Japan	A/Niigata/749/2009(H1N1)
NA	KF687913	H1N1	07/07/2009	Cyprus	A/Cyprus/061/2009
NA	CY045956	H1N1	07/07/2009	Canada	A/Toronto/T0106/2009(H1N1)
NA	GU014749	H1N1	07/07/2009	Japan	A/Gifu-C/67/2009(H1N1)
NA	GQ497278	H1N1	07/07/2009	Poland	A/Poland/282/2009(H1N1)
NA	CY073887	H1N1	07/07/2009	Nicaragua	A/Managua/2729.02/2009(H1N1)
NA	CY052353	H1N1	07/07/2009	United Ar	A/UAE/C03/2009(H1N1)
NA	CY075413	H1N1	07/07/2009	Chile	A/Chile/3905/2009(H1N1)
NA	CY063536	H1N1	07/07/2009	USA	A/Boston/148/2009(H1N1)
NA	CY061519	H1N1	07/07/2009	Argentina	A/Argentina/HNRG1009/2009
NA	CY061517	H1N1	07/07/2009	Argentina	A/Argentina/HNRG1207/2009
NA	CY061515	H1N1	07/07/2009	Argentina	A/Argentina/HNRG1205/2009
NA	CY061516	H1N1	07/07/2009	Argentina	A/Argentina/HNRG1206/2009
NA	CY061518	H1N1	07/07/2009	Argentina	A/Argentina/HNRG1208/2009
NA	CY061514	H1N1	07/07/2009	Argentina	A/Argentina/HNRG1204/2009
NA	CY047091	H1N1	07/07/2009	Spain	A/Catalonia/NS658/2009(H1N1)
NA	HE804125	H1N1	07/07/2009	Uruguay	A/Uruguay/605/2009
NA	CY075745	H1N1	07/07/2009	Thailand	A/Thailand/CU-H140/2009(H1N1)
NA	CY075733	H1N1	07/07/2009	Thailand	A/Thailand/CU-H137/2009(H1N1)
NA	CY075739	H1N1	07/07/2009	Thailand	A/Thailand/CU-H139/2009(H1N1)
NA	CY075727	H1N1	07/07/2009	Thailand	A/Thailand/CU-H134/2009(H1N1)
NA	GQ387404	H1N1	07/07/2009	Spain	A/Catalonia/627/2009(H1N1)
NA	GQ387405	H1N1	07/07/2009	Spain	A/Catalonia/635/2009(H1N1)
NA	CY056263	H1N1	07/07/2009	Argentina	A/Buenos Aires/HIFJM240482/2009(H1N
NA	CY075751	H1N1	07/07/2009	Thailand	A/Thailand/CU-H143/2009(H1N1)
NA	JX556117	H1N1	07/07/2009	Cuba	A/Habana/2177/2009
NA	JX556114	H1N1	07/07/2009	Cuba	A/Habana/1235/2009
NA	CY046935	H1N1	07/07/2009	Chile	A/Santiago/3827/2009(H1N1)
NA	CY046937	H1N1	07/07/2009	Chile	A/Santiago/3832/2009(H1N1)
NA	KC781860	H1N1	07/07/2009	USA	A/Washington/52/2009
NA	KC782221	H1N1	07/08/2009	USA	A/Alaska/30/2009
NA	CY047103	H1N1	07/08/2009	Spain	A/Catalonia/NS686/2009(H1N1)
NA	KC780276	H1N1	07/08/2009	USA	A/Utah/40/2009
NA	CY064694	H1N1	07/08/2009	USA	A/Boston/143/2009(H1N1)
NA	CY120135	H1N1	07/08/2009	Hong Kong	A/Hong Kong/H090-681-V10/2009
NA	CY122717	H1N1	07/08/2009	Singapore	A/Singapore/GP1156/2009

NA	CY122757	H1N1	07/08/2009	Singapore	A/Singapore/GP1169/2009
NA	CY122633	H1N1	07/08/2009	Singapore	A/Singapore/GP1114/2009
NA	CY122741	H1N1	07/08/2009	Singapore	A/Singapore/GP1164/2009
NA	CY122733	H1N1	07/08/2009	Singapore	A/Singapore/GP1163/2009
NA	CY122773	H1N1	07/08/2009	Singapore	A/Singapore/GP1178/2009
NA	CY122721	H1N1	07/08/2009	Singapore	A/Singapore/GP1160/2009
NA	CY122697	H1N1	07/08/2009	Singapore	A/Singapore/GP1142/2009
NA	CY122725	H1N1	07/08/2009	Singapore	A/Singapore/GP1162/2009
NA	CY122673	H1N1	07/08/2009	Singapore	A/Singapore/GP1132/2009
NA	CY051609	H1N1	07/08/2009	USA	A/New York/4567/2009(H1N1)
NA	CY050192	H1N1	07/08/2009	USA	A/New York/4576/2009(H1N1)
NA	CY083098	H1N1	07/08/2009	Peru	A/Lima/WRAIR0519F/2009
NA	KT181417	H1N1	07/08/2009	Singapore	A/Singapore/DMS11/2009
NA	CY122749	H1N1	07/08/2009	Singapore	A/Singapore/GP1167/2009
NA	CY122625	H1N1	07/08/2009	Singapore	A/Singapore/GP1107/2009
NA	CY122665	H1N1	07/08/2009	Singapore	A/Singapore/GP1127/2009
NA	CY122713	H1N1	07/08/2009	Singapore	A/Singapore/GP1147/2009
NA	JN256685	H1N1	07/08/2009	Hong Kong	A/Hong Kong/H090-682-V1(0)/2009
NA	JN256864	H1N1	07/08/2009	Hong Kong	A/Hong Kong/H090-681-V1(0)/2009
NA	CY122765	H1N1	07/08/2009	Singapore	A/Singapore/GP1174/2009
NA	CY122705	H1N1	07/08/2009	Singapore	A/Singapore/GP1146/2009
NA	HQ239939	H1N1	07/08/2009	Canada	A/Canada-NB/RV2479/2009
NA	KF687915	H1N1	07/08/2009	Cyprus	A/Cyprus/113/2009
NA	KF687916	H1N1	07/08/2009	Cyprus	A/Cyprus/116/2009
NA	KF687917	H1N1	07/08/2009	Cyprus	A/Cyprus/119/2009
NA	CY071524	H1N1	07/08/2009	USA	A/California/WR1319P/2009(H1N1)
NA	GU014757	H1N1	07/08/2009	Myanmar	A/Myanmar/JP90/2009(H1N1)
NA	CY111661	H1N1	07/08/2009	Hong Kong	A/Hong Kong/H090-681-V10/2009
NA	CY051593	H1N1	07/08/2009	USA	A/New York/4565/2009(H1N1)
NA	CY051601	H1N1	07/08/2009	USA	A/New York/4566/2009(H1N1)
NA	CY071476	H1N1	07/08/2009	USA	A/California/WR1313P/2009(H1N1)
NA	HQ239941	H1N1	07/08/2009	Canada	A/Canada-NB/RV2490/2009
NA	HQ240007	H1N1	07/08/2009	Canada	A/Canada-BC/RV2936/2009
NA	CY122641	H1N1	07/08/2009	Singapore	A/Singapore/GP1120/2009
NA	CY061521	H1N1	07/08/2009	Argentina	A/Argentina/HNRG1211/2009
NA	CY061520	H1N1	07/08/2009	Argentina	A/Argentina/HNRG1210/2009
NA	CY047093	H1N1	07/08/2009	Spain	A/Catalonia/NS670/2009(H1N1)
NA	CY047095	H1N1	07/08/2009	Spain	A/Catalonia/NS672/2009(H1N1)
NA	CY047097	H1N1	07/08/2009	Spain	A/Catalonia/NS674/2009(H1N1)
NA	CY047099	H1N1	07/08/2009	Spain	A/Catalonia/NS675/2009(H1N1)
NA	CY047101	H1N1	07/08/2009	Spain	A/Catalonia/NS677/2009(H1N1)
NA	KC780217	H1N1	07/08/2009	USA	A/Utah/33/2009
NA	KC780622	H1N1	07/09/2009	USA	A/Utah/35/2009
NA	CY049493	H1N1	07/09/2009	Singapore	A/Singapore/ON1072/2009(H1N1)
NA	CY095908	H1N1	07/09/2009	China	A/Zhejiang/8/2009
NA	CY095742	H1N1	07/09/2009	China	A/Zhejiang/11/2009
NA	CY095730	H1N1	07/09/2009	China	A/Zhejiang/10/2009
NA	CY095765	H1N1	07/09/2009	China	A/Zhejiang/3/2009
NA	CY120167	H1N1	07/09/2009	Hong Kong	A/Hong Kong/H090-684-V10/2009
NA	KP637800	H1N1	07/09/2009	Thailand	A/Thailand/SS08193/2009
NA	KP637720	H1N1	07/09/2009	Thailand	A/Thailand/SR08021/2009
NA	CY054901	H1N1	07/09/2009	USA	A/California/VRDL39/2009(H1N1)
NA	CY123400	H1N1	07/09/2009	Singapore	A/Singapore/ON1074/2009
NA	CY122781	H1N1	07/09/2009	Singapore	A/Singapore/GP1182/2009

NA	CY095819	H1N1	07/09/2009	China	A/Zhejiang/5/2009
NA	HQ256633	H1N1	07/09/2009	Myanmar	A/Myanmar/M306/2009(H1N1)
NA	CY075437	H1N1	07/09/2009	Chile	A/Chile/4181/2009(H1N1)
NA	CY075429	H1N1	07/09/2009	Chile	A/Chile/4064/2009(H1N1)
NA	CY051585	H1N1	07/09/2009	USA	A/New York/4556/2009(H1N1)
NA	CY051617	H1N1	07/09/2009	USA	A/New York/4568/2009(H1N1)
NA	HQ247644	H1N1	07/09/2009	Finland	A/Finland/611/2009
NA	HQ247632	H1N1	07/09/2009	Finland	A/Finland/599/2009
NA	HQ247633	H1N1	07/09/2009	Finland	A/Finland/600/2009
NA	JN256869	H1N1	07/09/2009	Hong Kong	A/Hong Kong/H090-684-V1(0)/2009
NA	CY095843	H1N1	07/09/2009	China	A/Zhejiang/7/2009
NA	CY095779	H1N1	07/09/2009	China	A/Zhejiang/4/2009
NA	HQ239940	H1N1	07/09/2009	Canada	A/Canada-NB/RV2488/2009
NA	HQ239942	H1N1	07/09/2009	Canada	A/Canada-NFL/RV2502/2009
NA	CY066185	H1N1	07/09/2009	USA	A/Boston/144/2009(H1N1)
NA	CY055203	H1N1	07/09/2009	Malaysia	A/Malaysia/4737/2009(H1N1)
NA	CY095750	H1N1	07/09/2009	China	A/Zhejiang/X2/2009
NA	GU112752	H1N1	07/09/2009	Poland	A/Poland/295/2009(H1N1)
NA	GQ392023	H1N1	07/09/2009	Russia	A/Moscow/IIV04/2009(H1N1)
NA	HQ239968	H1N1	07/09/2009	Canada	A/Canada-AB/RV2820/2009
NA	CY049541	H1N1	07/09/2009	Singapore	A/Singapore/ON1118/2009(H1N1)
NA	CY049485	H1N1	07/09/2009	Singapore	A/Singapore/ON1067/2009(H1N1)
NA	CY095723	H1N1	07/09/2009	China	A/Zhejiang/X1/2009
NA	CY063512	H1N1	07/09/2009	USA	A/Boston/145/2009(H1N1)
NA	CY063528	H1N1	07/09/2009	USA	A/Boston/147/2009(H1N1)
NA	CY055205	H1N1	07/09/2009	Malaysia	A/Malaysia/4847/2009(H1N1)
NA	CY095981	H1N1	07/09/2009	China	A/Zhejiang/9/2009
NA	CY047113	H1N1	07/09/2009	Spain	A/Catalonia/S1084/2009(H1N1)
NA	CY111701	H1N1	07/09/2009	Hong Kong	A/Hong Kong/H090-684-V10/2009
NA	CY111717	H1N1	07/10/2009	Hong Kong	A/Hong Kong/H090-693-V10/2009
NA	CY047115	H1N1	07/10/2009	Spain	A/Catalonia/S1087/2009(H1N1)
NA	GQ497279	H1N1	07/10/2009	Poland	A/Poland/303/2009(H1N1)
NA	GU183801	H1N1	07/10/2009	Thailand	A/Thailand/CU-B589/2009(H1N1)
NA	CY049533	H1N1	07/10/2009	Singapore	A/Singapore/ON1113/2009(H1N1)
NA	CY075445	H1N1	07/10/2009	Chile	A/Chile/4182/2009(H1N1)
NA	HQ239953	H1N1	07/10/2009	Canada	A/Canada-NS/RV2599/2009
NA	CY074054	H1N1	07/10/2009	Argentina	A/Argentina/HNRG66/2009
NA	HE804102	H1N1	07/10/2009	Uruguay	A/Uruguay/11/2009
NA	HE804116	H1N1	07/10/2009	Uruguay	A/Uruguay/57/2009
NA	CY050085	H1N1	07/10/2009	Taiwan	A/Taiwan/T0826/2009(H1N1)
NA	CY120183	H1N1	07/10/2009	Hong Kong	A/Hong Kong/H090-693-V10/2009
NA	CY111669	H1N1	07/10/2009	Hong Kong	A/Hong Kong/H090-681-V22/2009
NA	JN256865	H1N1	07/10/2009	Hong Kong	A/Hong Kong/H090-681-V2(2)/2009
NA	KC780171	H1N1	07/10/2009	USA	A/Utah/46/2009
NA	JN256871	H1N1	07/10/2009	Hong Kong	A/Hong Kong/H090-693-V1(0)/2009
NA	CY054941	H1N1	07/10/2009	USA	A/California/VRDL48/2009(H1N1)
NA	JX556115	H1N1	07/10/2009	Cuba	A/Habana/1383/2009
NA	CY047116	H1N1	07/10/2009	Spain	A/Catalonia/S1090/2009(H1N1)
NA	CY061524	H1N1	07/10/2009	Argentina	A/Argentina/HNRG1014/2009
NA	CY061525	H1N1	07/10/2009	Argentina	A/Argentina/HNRG1215/2009
NA	CY061522	H1N1	07/10/2009	Argentina	A/Argentina/HNRG1212/2009
NA	CY061523	H1N1	07/10/2009	Argentina	A/Argentina/HNRG1213/2009
NA	CY061526	H1N1	07/10/2009	Argentina	A/Argentina/HNRG1216/2009
NA	CY065184	H1N1	07/10/2009	United Ki	A/England/675/2009

NA	CY062262	H1N1	07/10/2009	Malaysia	A/Malaysia/4969/2009
NA	CY056264	H1N1	07/11/2009	Argentina	A/Buenos Aires/HIFJM240848/2009(H1N
NA	KC782132	H1N1	07/11/2009	USA	A/Utah/34/2009
NA	CY054957	H1N1	07/11/2009	USA	A/California/VRDL51/2009(H1N1)
NA	CY054917	H1N1	07/11/2009	USA	A/California/VRDL41/2009(H1N1)
NA	CY055213	H1N1	07/11/2009	Malaysia	A/Malaysia/5112/2009(H1N1)
NA	CY055209	H1N1	07/11/2009	Malaysia	A/Malaysia/5057/2009(H1N1)
NA	CY055207	H1N1	07/11/2009	Malaysia	A/Malaysia/5027/2009(H1N1)
NA	JX625445	H1N1	07/11/2009	United Ki	A/England/687/2009
NA	CY120151	H1N1	07/11/2009	Hong Kong	A/Hong Kong/H090-683-V10/2009
NA	CY106570	H1N1	07/11/2009	Mexico	A/Merida/2189-CIR/2009
NA	CY111685	H1N1	07/11/2009	Hong Kong	A/Hong Kong/H090-683-V10/2009
NA	JN256867	H1N1	07/11/2009	Hong Kong	A/Hong Kong/H090-683-V1(0)/2009
NA	KT181455	H1N1	07/11/2009	Singapore	A/Singapore/DMS12/2009
NA	GU014753	H1N1	07/11/2009	Japan	A/Mie/41/2009(H1N1)
NA	KF687918	H1N1	07/11/2009	Cyprus	A/Cyprus/142/2009
NA	GU065290	H1N1	07/11/2009	Poland	A/Poland/304/2009(H1N1)
NA	CY049469	H1N1	07/12/2009	Singapore	A/Singapore/ON1050/2009(H1N1)
NA	CY056265	H1N1	07/12/2009	Argentina	A/Buenos Aires/HIFJM240866/2009(H1N
NA	CY049549	H1N1	07/12/2009	Singapore	A/Singapore/ON1126/2009(H1N1)
NA	CY072880	H1N1	07/12/2009	Nicaragua	A/Managua/4606.06/2009(H1N1)
NA	CY072960	H1N1	07/12/2009	Nicaragua	A/Managua/585.01/2009(H1N1)
NA	GQ421204	H1N1	07/12/2009	Italy	A/Ancona/05/2009(H1N1)
NA	HQ239943	H1N1	07/12/2009	Canada	A/Canada-NFL/RV2503/2009
NA	HQ240008	H1N1	07/12/2009	Canada	A/Canada-BC/RV2937/2009
NA	CY047106	H1N1	07/12/2009	Spain	A/Catalonia/NS867/2009(H1N1)
NA	CY055211	H1N1	07/12/2009	Malaysia	A/Malaysia/5103/2009(H1N1)
NA	KF687919	H1N1	07/12/2009	Cyprus	A/Cyprus/147/2009
NA	CY052425	H1N1	07/12/2009	USA	A/Texas/43122467/2009(H1N1)
NA	HQ247634	H1N1	07/12/2009	Finland	A/Finland/601/2009
NA	CY049477	H1N1	07/12/2009	Singapore	A/Singapore/ON1060/2009(H1N1)
NA	CY056266	H1N1	07/12/2009	Argentina	A/Buenos Aires/HIFJM240867/2009(H1N
NA	GU183809	H1N1	07/12/2009	Thailand	A/Thailand/CU-B685/2009(H1N1)
NA	JN256875	H1N1	07/13/2009	Hong Kong	A/Hong Kong/H090-701-V1(0)/2009
NA	JN256876	H1N1	07/13/2009	Hong Kong	A/Hong Kong/H090-704-V1(0)/2009
NA	CY111677	H1N1	07/13/2009	Hong Kong	A/Hong Kong/H090-681-V32/2009
NA	CY111749	H1N1	07/13/2009	Hong Kong	A/Hong Kong/H090-701-V10/2009
NA	CY111733	H1N1	07/13/2009	Hong Kong	A/Hong Kong/H090-698-V10/2009
NA	CY120078	H1N1	07/13/2009	Hong Kong	A/Hong Kong/H090-698-V10/2009
NA	CY120143	H1N1	07/13/2009	Hong Kong	A/Hong Kong/H090-681-V32/2009
NA	CY111725	H1N1	07/13/2009	Hong Kong	A/Hong Kong/H090-693-V20/2009
NA	CY120070	H1N1	07/13/2009	Hong Kong	A/Hong Kong/H090-693-V20/2009
NA	CY120199	H1N1	07/13/2009	Hong Kong	A/Hong Kong/H090-701-V10/2009
NA	CY120207	H1N1	07/13/2009	Hong Kong	A/Hong Kong/H090-704-V10/2009
NA	CY123384	H1N1	07/13/2009	Singapore	A/Singapore/ON1063/2009
NA	JX625461	H1N1	07/13/2009	United Ki	A/England/688/2009
NA	KP637656	H1N1	07/13/2009	Thailand	A/Thailand/SP08279/2009
NA	CY052409	H1N1	07/13/2009	USA	A/Texas/43132503/2009(H1N1)
NA	CY111757	H1N1	07/13/2009	Hong Kong	A/Hong Kong/H090-704-V10/2009
NA	CY055215	H1N1	07/13/2009	Malaysia	A/Malaysia/5162/2009(H1N1)
NA	CY055217	H1N1	07/13/2009	Malaysia	A/Malaysia/5163/2009(H1N1)
NA	CY055219	H1N1	07/13/2009	Malaysia	A/Malaysia/5175/2009(H1N1)
NA	CY123368	H1N1	07/13/2009	Singapore	A/Singapore/ON1048/2009
NA	CY123376	H1N1	07/13/2009	Singapore	A/Singapore/ON1058/2009

NA	KC780128	H1N1	07/13/2009	USA	A/Utah/36/2009
NA	KC781488	H1N1	07/13/2009	USA	A/Minnesota/12/2009
NA	CY049557	H1N1	07/13/2009	Singapore	A/Singapore/ON1138/2009(H1N1)
NA	CY049565	H1N1	07/13/2009	Singapore	A/Singapore/ON1144/2009(H1N1)
NA	CY049501	H1N1	07/13/2009	Singapore	A/Singapore/ON1087/2009(H1N1)
NA	CY049509	H1N1	07/13/2009	Singapore	A/Singapore/ON1093/2009(H1N1)
NA	CY049517	H1N1	07/13/2009	Singapore	A/Singapore/ON1099/2009(H1N1)
NA	CY061528	H1N1	07/13/2009	Argentina	A/Argentina/HNRG1018/2009
NA	CY061527	H1N1	07/13/2009	Argentina	A/Argentina/HNRG1217/2009
NA	CY075757	H1N1	07/13/2009	Thailand	A/Thailand/CU-H199/2009(H1N1)
NA	CY075762	H1N1	07/13/2009	Thailand	A/Thailand/CU-H203/2009(H1N1)
NA	CY049525	H1N1	07/13/2009	Singapore	A/Singapore/ON1105/2009(H1N1)
NA	JN256873	H1N1	07/13/2009	Hong Kong	A/Hong Kong/H090-698-V1(0)/2009
NA	HQ240011	H1N1	07/13/2009	Canada	A/Canada-BC/RV2940/2009
NA	GU014771	H1N1	07/13/2009	Japan	A/Wakayama/57/2009(H1N1)
NA	KF687920	H1N1	07/13/2009	Cyprus	A/Cyprus/183/2009
NA	CY075453	H1N1	07/13/2009	Chile	A/Chile/4257/2009(H1N1)
NA	HQ256635	H1N1	07/13/2009	Myanmar	A/Myanmar/M314/2009(H1N1)
NA	HQ247635	H1N1	07/13/2009	Finland	A/Finland/602/2009
NA	CY083513	H1N1	07/13/2009	Peru	A/Piura/WRAIR1694P/2009(H1N1)
NA	CY083106	H1N1	07/13/2009	Peru	A/Piura/WRAIR0603F/2009(H1N1)
NA	CY083322	H1N1	07/13/2009	USA	A/Great Lakes/WRAIR1652P/2009
NA	CY123392	H1N1	07/13/2009	Singapore	A/Singapore/ON1069/2009
NA	JN256866	H1N1	07/13/2009	Hong Kong	A/Hong Kong/H090-681-V3(2)/2009
NA	JN256872	H1N1	07/13/2009	Hong Kong	A/Hong Kong/H090-693-V2(0)/2009
NA	CY049573	H1N1	07/14/2009	Singapore	A/Singapore/ON1156/2009(H1N1)
NA	CY049581	H1N1	07/14/2009	Singapore	A/Singapore/ON1163/2009(H1N1)
NA	CY047119	H1N1	07/14/2009	Spain	A/Catalonia/S1096/2009(H1N1)
NA	CY052089	H1N1	07/14/2009	Italy	A/Italy/230/2009(H1N1)
NA	HQ240012	H1N1	07/14/2009	Canada	A/Canada-BC/RV2941/2009
NA	HQ239887	H1N1	07/14/2009	Canada	A/Canada-MB/RV2038-10/2009
NA	HQ239960	H1N1	07/14/2009	Canada	A/Canada-AB/RV2812/2009
NA	CY049597	H1N1	07/14/2009	Singapore	A/Singapore/ON1177/2009(H1N1)
NA	HE804127	H1N1	07/14/2009	Uruguay	A/Uruguay/58/2009
NA	GQ499337	H1N1	07/14/2009	USA	A/Washington/28/2009(H1N1)
NA	CY049589	H1N1	07/14/2009	Singapore	A/Singapore/ON1168/2009(H1N1)
NA	KF687921	H1N1	07/14/2009	Cyprus	A/Cyprus/207/2009
NA	CY073699	H1N1	07/14/2009	Nicaragua	A/Managua/3490.03/2009(H1N1)
NA	GU014759	H1N1	07/14/2009	Myanmar	A/Myanmar/JP101/2009(H1N1)
NA	CY051633	H1N1	07/14/2009	USA	A/New York/4620/2009(H1N1)
NA	CY051625	H1N1	07/14/2009	USA	A/New York/4607/2009(H1N1)
NA	HQ247637	H1N1	07/14/2009	Finland	A/Finland/604/2009
NA	HQ247636	H1N1	07/14/2009	Finland	A/Finland/603/2009
NA	KT181499	H1N1	07/14/2009	Singapore	A/Singapore/DMS13/2009
NA	CY123448	H1N1	07/14/2009	Singapore	A/Singapore/ON1114/2009
NA	JN256868	H1N1	07/14/2009	Hong Kong	A/Hong Kong/H090-683-V2(0)/2009
NA	JN256870	H1N1	07/14/2009	Hong Kong	A/Hong Kong/H090-684-V2(3)/2009
NA	JN256877	H1N1	07/14/2009	Hong Kong	A/Hong Kong/H090-708-V1(0)/2009
NA	CY123408	H1N1	07/14/2009	Singapore	A/Singapore/ON1079/2009
NA	CY123424	H1N1	07/14/2009	Singapore	A/Singapore/ON1095/2009
NA	CY111693	H1N1	07/14/2009	Hong Kong	A/Hong Kong/H090-683-V20/2009
NA	CY111709	H1N1	07/14/2009	Hong Kong	A/Hong Kong/H090-684-V23/2009
NA	CY111765	H1N1	07/14/2009	Hong Kong	A/Hong Kong/H090-708-V10/2009
NA	CY120215	H1N1	07/14/2009	Hong Kong	A/Hong Kong/H090-708-V10/2009

NA	CY120159	H1N1	07/14/2009	Hong Kong	A/Hong Kong/H090-683-V20/2009
NA	CY122789	H1N1	07/14/2009	Singapore	A/Singapore/GP1358/2009
NA	CY123456	H1N1	07/14/2009	Singapore	A/Singapore/ON1121/2009
NA	CY052252	H1N1	07/14/2009	USA	A/Texas/43143450/2009(H1N1)
NA	HM992567	H1N1	07/14/2009	Italy	A/Rome/709/2009
NA	CY120175	H1N1	07/14/2009	Hong Kong	A/Hong Kong/H090-684-V23/2009
NA	CY054965	H1N1	07/14/2009	USA	A/California/VRDL52/2009(H1N1)
NA	CY054981	H1N1	07/14/2009	USA	A/California/VRDL55/2009(H1N1)
NA	CY123440	H1N1	07/14/2009	Singapore	A/Singapore/ON1107/2009
NA	CY123416	H1N1	07/14/2009	Singapore	A/Singapore/ON1091/2009
NA	CY123432	H1N1	07/14/2009	Singapore	A/Singapore/ON1102/2009
NA	KC782051	H1N1	07/14/2009	USA	A/Maryland/33/2009
NA	JN256879	H1N1	07/15/2009	Hong Kong	A/Hong Kong/H090-712-V1(4)/2009
NA	JN256686	H1N1	07/15/2009	Hong Kong	A/Hong Kong/H090-718-V1(0)/2009
NA	CY111773	H1N1	07/15/2009	Hong Kong	A/Hong Kong/H090-712-V10/2009
NA	CY111781	H1N1	07/15/2009	Hong Kong	A/Hong Kong/H090-712-V1_4/2009
NA	CY120231	H1N1	07/15/2009	Hong Kong	A/Hong Kong/H090-712-V14/2009
NA	CY120223	H1N1	07/15/2009	Hong Kong	A/Hong Kong/H090-712-V10/2009
NA	JX625437	H1N1	07/15/2009	United Ki	A/England/92960004/2009
NA	CY055221	H1N1	07/15/2009	Malaysia	A/Malaysia/5192/2009(H1N1)
NA	CY055223	H1N1	07/15/2009	Malaysia	A/Malaysia/5193/2009(H1N1)
NA	CY049605	H1N1	07/15/2009	Singapore	A/Singapore/ON1187/2009(H1N1)
NA	CY049613	H1N1	07/15/2009	Singapore	A/Singapore/ON1194/2009(H1N1)
NA	CY047121	H1N1	07/15/2009	Spain	A/Catalonia/S1099/2009(H1N1)
NA	CY047126	H1N1	07/15/2009	Spain	A/Catalonia/S1102/2009(H1N1)
NA	CY047129	H1N1	07/15/2009	Spain	A/Catalonia/S1107/2009(H1N1)
NA	CY061530	H1N1	07/15/2009	Argentina	A/Argentina/HNRG1220/2009
NA	CY061531	H1N1	07/15/2009	Argentina	A/Argentina/HNRG1221/2009
NA	CY061532	H1N1	07/15/2009	Argentina	A/Argentina/HNRG1222/2009
NA	CY061529	H1N1	07/15/2009	Argentina	A/Argentina/HNRG1219/2009
NA	HE804110	H1N1	07/15/2009	Uruguay	A/Uruguay/689/2009
NA	CY075768	H1N1	07/15/2009	Thailand	A/Thailand/CU-H216/2009(H1N1)
NA	HQ239888	H1N1	07/15/2009	Canada	A/Canada-MB/RV2039-10/2009
NA	HQ240009	H1N1	07/15/2009	Canada	A/Canada-BC/RV2938/2009
NA	CY056267	H1N1	07/15/2009	Argentina	A/Buenos Aires/HIFJM241261/2009(H1N
NA	CY063568	H1N1	07/15/2009	USA	A/Boston/153/2009(H1N1)
NA	HQ240010	H1N1	07/15/2009	Canada	A/Canada-BC/RV2939/2009
NA	HM569704	H1N1	07/15/2009	Argentina	A/Argentina/07AR/2009(H1N1)
NA	CY087035	H1N1	07/15/2009	USA	A/New York/4631/2009(H1N1)
NA	HQ247638	H1N1	07/15/2009	Finland	A/Finland/605/2009
NA	HQ247639	H1N1	07/15/2009	Finland	A/Finland/606/2009
NA	CY083050	H1N1	07/15/2009	Peru	A/Lima/WRAIR0285F/2009
NA	HM189330	H1N1	07/15/2009	Russia	A/Moscow oblast/CRIE-4142/2009
NA	HM189331	H1N1	07/15/2009	Russia	A/Moscow oblast/CRIE-4145/2009
NA	AB603613	H1N1	07/15/2009	Lebanon	A/Lebanon/09L-01/2009
NA	GU290057	H1N1	07/15/2009	Czech Rep	A/Prague/196-81511/2009(H1N1)
NA	JN256878	H1N1	07/15/2009	Hong Kong	A/Hong Kong/H090-712-V1(0)/2009
NA	JN256874	H1N1	07/16/2009	Hong Kong	A/Hong Kong/H090-698-V2(0)/2009
NA	HM189337	H1N1	07/16/2009	Russia	A/Moscow/CRIE-ION/2009
NA	CY111805	H1N1	07/16/2009	Hong Kong	A/Hong Kong/H090-715-V10/2009
NA	AB649907	H1N1	07/16/2009	Japan	A/Toyama/251/2009
NA	CY111741	H1N1	07/16/2009	Hong Kong	A/Hong Kong/H090-698-V20/2009
NA	CY055528	H1N1	07/16/2009	Australia	A/Australia/1/2009(H1N1)
NA	CY120191	H1N1	07/16/2009	Hong Kong	A/Hong Kong/H090-698-V20/2009

NA	CY055225	H1N1	07/16/2009	Malaysia	A/Malaysia/5218/2009(H1N1)
NA	KC780280	H1N1	07/16/2009	USA	A/Utah/41/2009
NA	CY047135	H1N1	07/16/2009	Spain	A/Catalonia/S1118/2009(H1N1)
NA	CY047132	H1N1	07/16/2009	Spain	A/Catalonia/S1113/2009(H1N1)
NA	HQ240013	H1N1	07/16/2009	Canada	A/Canada-BC/RV2942/2009
NA	HQ239890	H1N1	07/16/2009	Canada	A/Canada-MB/RV2040-10/2009
NA	GU183817	H1N1	07/16/2009	Thailand	A/Thailand/CU-H223/2009(H1N1)
NA	CY072840	H1N1	07/16/2009	Nicaragua	A/Managua/265.01/2009(H1N1)
NA	CY072896	H1N1	07/16/2009	Nicaragua	A/Managua/3214.04/2009(H1N1)
NA	CY083361	H1N1	07/16/2009	USA	A/San Diego/WRAIR1658P/2009
NA	HQ256619	H1N1	07/16/2009	Myanmar	A/Myanmar/M256/2009(H1N1)
NA	JN256882	H1N1	07/16/2009	Hong Kong	A/Hong Kong/H090-715-V1(0)/2009
NA	CY051641	H1N1	07/16/2009	USA	A/New York/4677/2009(H1N1)
NA	HQ247660	H1N1	07/16/2009	Finland	A/Finland/627/2009
NA	JX625469	H1N1	07/17/2009	United Ki	A/England/92980479/2009
NA	HQ247661	H1N1	07/17/2009	Finland	A/Finland/628/2009
NA	CY111813	H1N1	07/17/2009	Hong Kong	A/Hong Kong/H090-721-V10/2009
NA	HQ239963	H1N1	07/17/2009	Canada	A/Canada-AB/RV2815/2009
NA	HQ240015	H1N1	07/17/2009	Canada	A/Canada-BC/RV2944/2009
NA	JN256883	H1N1	07/17/2009	Hong Kong	A/Hong Kong/H090-721-V1(0)/2009
NA	CY075774	H1N1	07/17/2009	Thailand	A/Thailand/CU-H243/2009(H1N1)
NA	CY120255	H1N1	07/17/2009	Hong Kong	A/Hong Kong/H090-721-V10/2009
NA	CY083282	H1N1	07/17/2009	USA	A/South Carolina/WRAIR1645P/2009
NA	CY075461	H1N1	07/17/2009	Chile	A/Chile/4406/2009(H1N1)
NA	JN256884	H1N1	07/17/2009	Hong Kong	A/Hong Kong/H090-722-V1(0)/2009
NA	KT181508	H1N1	07/17/2009	Singapore	A/Singapore/DMS14/2009
NA	CY097886	H1N1	07/17/2009	USA	A/South Carolina/WRAIR1645P/2009
NA	CY120263	H1N1	07/17/2009	Hong Kong	A/Hong Kong/H090-722-V10/2009
NA	GU576503	H1N1	07/17/2009	Italy	A/Ancona/15/2009(H1N1)
NA	GU576505	H1N1	07/17/2009	Italy	A/Ancona/16/2009(H1N1)
NA	GU576507	H1N1	07/17/2009	Italy	A/Ancona/17/2009(H1N1)
NA	CY055061	H1N1	07/17/2009	Australia	A/Australia/3/2009(H1N1)
NA	CY055069	H1N1	07/17/2009	Australia	A/Australia/4/2009(H1N1)
NA	CY055227	H1N1	07/17/2009	Malaysia	A/Malaysia/5225/2009(H1N1)
NA	CY083138	H1N1	07/17/2009	Peru	A/Lima/WRAIR0681F/2009
NA	AB603614	H1N1	07/17/2009	Lebanon	A/Lebanon/09L-02/2009
NA	KC781467	H1N1	07/17/2009	USA	A/Idaho/08/2009
NA	HQ247659	H1N1	07/17/2009	Finland	A/Finland/626/2009
NA	CY111821	H1N1	07/17/2009	Hong Kong	A/Hong Kong/H090-722-V10/2009(H1N1)
NA	CY055536	H1N1	07/18/2009	Australia	A/Australia/7/2009(H1N1)
NA	HQ240014	H1N1	07/18/2009	Canada	A/Canada-BC/RV2943/2009
NA	CY064905	H1N1	07/18/2009	Italy	A/Rome/690/2009(H1N1)
NA	KC780326	H1N1	07/18/2009	USA	A/Utah/37/2009
NA	CY111797	H1N1	07/18/2009	Hong Kong	A/Hong Kong/H090-712-V24/2009
NA	CY055077	H1N1	07/18/2009	Australia	A/Australia/6/2009(H1N1)
NA	CY120239	H1N1	07/18/2009	Hong Kong	A/Hong Kong/H090-712-V20/2009
NA	CY120247	H1N1	07/18/2009	Hong Kong	A/Hong Kong/H090-712-V24/2009
NA	CY111789	H1N1	07/18/2009	Hong Kong	A/Hong Kong/H090-712-V20/2009
NA	AB603615	H1N1	07/18/2009	Lebanon	A/Lebanon/09L-03/2009
NA	JN256881	H1N1	07/18/2009	Hong Kong	A/Hong Kong/H090-712-V2(4)/2009
NA	JN256880	H1N1	07/18/2009	Hong Kong	A/Hong Kong/H090-712-V2(0)/2009
NA	CY083353	H1N1	07/18/2009	USA	A/San Diego/WRAIR1657P/2009
NA	GU014807	H1N1	07/18/2009	Japan	A/Tokushima/2/2009(H1N1)
NA	HQ240018	H1N1	07/18/2009	Canada	A/Canada-BC/RV2947/2009

NA	GU433027	H1N1	07/19/2009	Russia	A/Tomsk/03/2009(H1N1)
NA	KC780158	H1N1	07/19/2009	USA	A/Utah/47/2009
NA	CY083130	H1N1	07/19/2009	Peru	A/Lima/WRAIR0672F/2009
NA	GQ409973	H1N1	07/19/2009	Greece	A/Crete/2331/2009(H1N1)
NA	HQ240019	H1N1	07/19/2009	Canada	A/Canada-BC/RV2948/2009
NA	GQ409643	H1N1	07/19/2009	Greece	A/Crete/2307/2009(H1N1)
NA	GQ409642	H1N1	07/19/2009	Greece	A/Athens/2277/2009(H1N1)
NA	GQ409640	H1N1	07/19/2009	Greece	A/Athens/2275/2009(H1N1)
NA	KC781036	H1N1	07/19/2009	USA	A/Washington/74/2009
NA	GQ409644	H1N1	07/19/2009	Greece	A/Crete/2305/2009(H1N1)
NA	KP637400	H1N1	07/20/2009	Thailand	A/Thailand/MR08208/2009
NA	HQ239965	H1N1	07/20/2009	Canada	A/Canada-AB/RV2817/2009
NA	CY055583	H1N1	07/20/2009	Australia	A/Australia/14/2009(H1N1)
NA	CY055552	H1N1	07/20/2009	Australia	A/Australia/10/2009(H1N1)
NA	CY055567	H1N1	07/20/2009	Australia	A/Australia/12/2009(H1N1)
NA	CY056245	H1N1	07/20/2009	Australia	A/Australia/11/2009(H1N1)
NA	CY055591	H1N1	07/20/2009	Australia	A/Australia/15/2009(H1N1)
NA	CY052377	H1N1	07/20/2009	USA	A/Texas/43200999/2009(H1N1)
NA	HM241698	H1N1	07/20/2009	India	A/Che/NIV658/2009
NA	CY071001	H1N1	07/20/2009	Guam	A/Guam/NHRC0030/2009(H1N1)
NA	HQ256615	H1N1	07/20/2009	Myanmar	A/Myanmar/M218/2009(H1N1)
NA	CY075469	H1N1	07/20/2009	Chile	A/Chile/4438/2009(H1N1)
NA	HQ247658	H1N1	07/20/2009	Finland	A/Finland/625/2009
NA	HQ247662	H1N1	07/20/2009	Finland	A/Finland/629/2009
NA	HQ247663	H1N1	07/20/2009	Finland	A/Finland/630/2009
NA	CY055544	H1N1	07/20/2009	Australia	A/Australia/9/2009(H1N1)
NA	HQ240017	H1N1	07/20/2009	Canada	A/Canada-BC/RV2946/2009
NA	HQ239961	H1N1	07/20/2009	Canada	A/Canada-AB/RV2813/2009
NA	KJ130158	H1N1	07/20/2009	New Zeala	A/New Zealand/3958/2009
NA	CY055229	H1N1	07/20/2009	Malaysia	A/Malaysia/5234/2009(H1N1)
NA	CY055231	H1N1	07/20/2009	Malaysia	A/Malaysia/5237/2009(H1N1)
NA	CY053905	H1N1	07/20/2009	Argentina	A/Argentina/HNRG15/2009(H1N1)
NA	CY052090	H1N1	07/20/2009	Italy	A/Italy/231/2009(H1N1)
NA	CY047138	H1N1	07/20/2009	Spain	A/Catalonia/S1120/2009(H1N1)
NA	CY047144	H1N1	07/20/2009	Spain	A/Catalonia/S1127/2009(H1N1)
NA	CY047146	H1N1	07/20/2009	Spain	A/Catalonia/S1128/2009(H1N1)
NA	GQ409646	H1N1	07/20/2009	Greece	A/Athens/2294/2009(H1N1)
NA	CY064907	H1N1	07/20/2009	Italy	A/Rome/692/2009(H1N1)
NA	CY053468	H1N1	07/20/2009	Argentina	A/Argentina/18417/2009
NA	CY064906	H1N1	07/20/2009	Italy	A/Rome/691/2009(H1N1)
NA	HQ240016	H1N1	07/20/2009	Canada	A/Canada-BC/RV2945/2009
NA	HQ239966	H1N1	07/20/2009	Canada	A/Canada-AB/RV2818/2009
NA	HQ239964	H1N1	07/20/2009	Canada	A/Canada-AB/RV2816/2009
NA	CY055575	H1N1	07/20/2009	Australia	A/Australia/13/2009(H1N1)
NA	CY055631	H1N1	07/21/2009	Australia	A/Australia/24/2009(H1N1)
NA	CY055297	H1N1	07/21/2009	Malaysia	A/Malaysia/5259/2009(H1N1)
NA	KC782034	H1N1	07/21/2009	USA	A/Washington/47/2009
NA	KC782040	H1N1	07/21/2009	USA	A/West Virginia/06/2009
NA	KC781563	H1N1	07/21/2009	USA	A/Virginia/17/2009
NA	GU433035	H1N1	07/21/2009	Russia	A/Novosibirsk/02/2009(H1N1)
NA	CY055615	H1N1	07/21/2009	Australia	A/Australia/21/2009(H1N1)
NA	CY055599	H1N1	07/21/2009	Australia	A/Australia/16/2009(H1N1)
NA	CY055233	H1N1	07/21/2009	Malaysia	A/Malaysia/5258/2009(H1N1)
NA	CY055607	H1N1	07/21/2009	Australia	A/Australia/20/2009(H1N1)

NA	CY054989	H1N1	07/21/2009	USA	A/California/VRDL58/2009(H1N1)
NA	HM569669	H1N1	07/21/2009	Argentina	A/Argentina/08AR/2009(H1N1)
NA	CY054973	H1N1	07/21/2009	USA	A/California/VRDL53/2009(H1N1)
NA	CY055359	H1N1	07/21/2009	Italy	A/Rome/641/2009(H1N1)
NA	CY055289	H1N1	07/21/2009	Malaysia	A/Malaysia/5259/2009(H1N1)
NA	CY072856	H1N1	07/21/2009	Nicaragua	A/Managua/3438.02/2009(H1N1)
NA	KC620388	H1N1	07/21/2009	Germany	A/Giessen/6/2009
NA	HQ247664	H1N1	07/21/2009	Finland	A/Finland/631/2009
NA	HQ247645	H1N1	07/21/2009	Finland	A/Finland/612/2009
NA	CY083178	H1N1	07/21/2009	USA	A/South Carolina/WRAIR1500P/2009
NA	CY083210	H1N1	07/21/2009	USA	A/New Jersey/WRAIR1504P/2009(H1N1)
NA	JN256885	H1N1	07/21/2009	Hong Kong	A/Hong Kong/H090-722-V2(0)/2009
NA	AB649908	H1N1	07/21/2009	Japan	A/Toyama/254/2009
NA	JN631043	H1N1	07/21/2009	China	A/Shanghai/1195T/2009
NA	CY111829	H1N1	07/21/2009	Hong Kong	A/Hong Kong/H090-722-V20/2009(H1N1)
NA	CY073444	H1N1	07/21/2009	USA	A/San Diego/WR1651P/2009(H1N1)
NA	CY069036	H1N1	07/21/2009	Guam	A/Guam/NHRC0019/2009(H1N1)
NA	CY073436	H1N1	07/21/2009	USA	A/San Diego/WR1650P/2009(H1N1)
NA	CY069260	H1N1	07/21/2009	Nicaragua	A/Managua/5364.01/2009(H1N1)
NA	CY069092	H1N1	07/21/2009	Guam	A/Guam/NHRC0026/2009(H1N1)
NA	CY068964	H1N1	07/21/2009	Guam	A/Guam/NHRC0010/2009(H1N1)
NA	CY068972	H1N1	07/21/2009	Guam	A/Guam/NHRC0011/2009(H1N1)
NA	GU014755	H1N1	07/21/2009	Japan	A/Mie/52/2009(H1N1)
NA	CY092187	H1N1	07/21/2009	Australia	A/Australia/17/2009
NA	CY047148	H1N1	07/21/2009	Spain	A/Catalonia/S1137/2009(H1N1)
NA	CY047151	H1N1	07/21/2009	Spain	A/Catalonia/S1138/2009(H1N1)
NA	GQ409647	H1N1	07/21/2009	Greece	A/Athens/2385/2009(H1N1)
NA	CY061533	H1N1	07/21/2009	Argentina	A/Argentina/HNRG1224/2009
NA	JN034587	H1N1	07/21/2009	Philippir	A/Philippines/TMC120/2009
NA	HQ239967	H1N1	07/21/2009	Canada	A/Canada-AB/RV2819/2009
NA	HQ239969	H1N1	07/21/2009	Canada	A/Canada-AB/RV2821/2009
NA	CY069052	H1N1	07/21/2009	Guam	A/Guam/NHRC0021/2009(H1N1)
NA	CY055623	H1N1	07/22/2009	Australia	A/Australia/23/2009(H1N1)
NA	HQ239971	H1N1	07/22/2009	Canada	A/Canada-AB/RV2824/2009
NA	CY055647	H1N1	07/22/2009	Australia	A/Australia/26/2009(H1N1)
NA	CY055655	H1N1	07/22/2009	Australia	A/Australia/27/2009(H1N1)
NA	CY055235	H1N1	07/22/2009	Malaysia	A/Malaysia/5262/2009(H1N1)
NA	CY055237	H1N1	07/22/2009	Malaysia	A/Malaysia/5270/2009(H1N1)
NA	CY055239	H1N1	07/22/2009	Malaysia	A/Malaysia/5272/2009(H1N1)
NA	CY055241	H1N1	07/22/2009	Malaysia	A/Malaysia/5277/2009(H1N1)
NA	CY073643	H1N1	07/22/2009	Nicaragua	A/Managua/1973.02/2009(H1N1)
NA	CY073651	H1N1	07/22/2009	Nicaragua	A/Managua/4590.03/2009(H1N1)
NA	HQ256637	H1N1	07/22/2009	Myanmar	A/Myanmar/M353/2009(H1N1)
NA	HQ247646	H1N1	07/22/2009	Finland	A/Finland/613/2009
NA	CY083186	H1N1	07/22/2009	USA	A/South Carolina/WRAIR1501P/2009
NA	CY083202	H1N1	07/22/2009	USA	A/South Carolina/WRAIR1503P/2009
NA	KX013007	H1N1	07/22/2009	Germany	A/Jena/MSV-VI5258/2009
NA	KC222638	H1N1	07/22/2009	Germany	A/Jena/VI5258/2009(H1N1)
NA	KF695074	H1N1	07/22/2009	Singapore	A/Singapore/384P/2009
NA	GQ496143	H1N1	07/22/2009	Russia	A/Vladivostok/IIV17/2009(H1N1)
NA	KJ690426	H1N1	07/22/2009	Uganda	A/Uganda/MUWRP-059/2009
NA	KJ549780	H1N1	07/22/2009	Germany	A/Jena/5258/2009
NA	CY068940	H1N1	07/22/2009	Guam	A/Guam/NHRC0008/2009(H1N1)
NA	CY069028	H1N1	07/22/2009	Guam	A/Guam/NHRC0018/2009(H1N1)

NA	CY073619	H1N1	07/22/2009	Nicaragua	A/Managua/4747.01/2009(H1N1)
NA	GU014761	H1N1	07/22/2009	Myanmar	A/Myanmar/JP131/2009(H1N1)
NA	GU014763	H1N1	07/22/2009	Myanmar	A/Myanmar/JP133/2009(H1N1)
NA	CY083369	H1N1	07/22/2009	USA	A/South Carolina/WRAIR1660P/2009
NA	CY061538	H1N1	07/22/2009	Argentina	A/Argentina/HNRG82/2009
NA	CY047141	H1N1	07/22/2009	Spain	A/Catalonia/S1124/2009(H1N1)
NA	CY053469	H1N1	07/22/2009	Argentina	A/Argentina/18556/2009
NA	CY055639	H1N1	07/22/2009	Australia	A/Australia/25/2009(H1N1)
NA	CY055670	H1N1	07/23/2009	Australia	A/Australia/29/2009(H1N1)
NA	CY047153	H1N1	07/23/2009	Spain	A/Catalonia/S1148/2009(H1N1)
NA	CY055686	H1N1	07/23/2009	Australia	A/Australia/32/2009(H1N1)
NA	CY055663	H1N1	07/23/2009	Australia	A/Australia/28/2009(H1N1)
NA	KC781867	H1N1	07/23/2009	USA	A/Kansas/14/2009
NA	KC781871	H1N1	07/23/2009	USA	A/South Carolina/29/2009
NA	JX625500	H1N1	07/23/2009	United Ki	A/England/93760756/2009
NA	CY055243	H1N1	07/23/2009	Malaysia	A/Malaysia/5283/2009(H1N1)
NA	CY055245	H1N1	07/23/2009	Malaysia	A/Malaysia/5286/2009(H1N1)
NA	CY065954	H1N1	07/23/2009	Japan	A/Japan/NHRC0001/2009(H1N1)
NA	CY073595	H1N1	07/23/2009	Nicaragua	A/Managua/5295.02/2009(H1N1)
NA	CY065962	H1N1	07/23/2009	Japan	A/Japan/NHRC0002/2009(H1N1)
NA	KT181517	H1N1	07/23/2009	Singapore	A/Singapore/DMS16/2009
NA	CY119356	H1N1	07/23/2009	Malaysia	A/Malaysia/2050837/2009
NA	CY065938	H1N1	07/23/2009	Guam	A/Guam/NHRC0002/2009(H1N1)
NA	CY068980	H1N1	07/23/2009	Guam	A/Guam/NHRC0012/2009(H1N1)
NA	CY068916	H1N1	07/23/2009	Japan	A/Japan/NHRC0003/2009(H1N1)
NA	CY055678	H1N1	07/23/2009	Australia	A/Australia/31/2009(H1N1)
NA	CY055694	H1N1	07/24/2009	Australia	A/Australia/35/2009(H1N1)
NA	CY072576	H1N1	07/24/2009	Nicaragua	A/Managua/3231.02/2009(H1N1)
NA	CY055710	H1N1	07/24/2009	Australia	A/Australia/37/2009(H1N1)
NA	KC780097	H1N1	07/24/2009	USA	A/Utah/42/2009
NA	KC782282	H1N1	07/24/2009	USA	A/Utah/42/2009
NA	KC782284	H1N1	07/24/2009	USA	A/Utah/42/2009
NA	CY120279	H1N1	07/24/2009	Hong Kong	A/Hong Kong/H090-742-V10/2009
NA	CY120271	H1N1	07/24/2009	Hong Kong	A/Hong Kong/H090-738-V10/2009
NA	CY128085	H1N1	07/24/2009	Viet Nam	A/Viet Nam/811/2009
NA	CY128077	H1N1	07/24/2009	Viet Nam	A/Viet Nam/817/2009
NA	KP637456	H1N1	07/24/2009	Thailand	A/Thailand/PY08016/2009
NA	KP637664	H1N1	07/24/2009	Thailand	A/Thailand/SP08294/2009
NA	CY051825	H1N1	07/24/2009	USA	A/Texas/43242018/2009(H1N1)
NA	CY055718	H1N1	07/24/2009	Australia	A/Australia/39/2009(H1N1)
NA	CY055726	H1N1	07/24/2009	Australia	A/Australia/40/2009(H1N1)
NA	CY055368	H1N1	07/24/2009	Italy	A/Rome/648/2009(H1N1)
NA	CY092195	H1N1	07/24/2009	Australia	A/Australia/38/2009
NA	CY051657	H1N1	07/24/2009	USA	A/New York/4728/2009(H1N1)
NA	HQ247647	H1N1	07/24/2009	Finland	A/Finland/614/2009
NA	GU290191	H1N1	07/24/2009	USA	A/Utah/42/2009(H1N1)
NA	JN256886	H1N1	07/24/2009	Hong Kong	A/Hong Kong/H090-738-V1(0)/2009
NA	JN256887	H1N1	07/24/2009	Hong Kong	A/Hong Kong/H090-742-V1(0)/2009
NA	JN256687	H1N1	07/24/2009	Hong Kong	A/Hong Kong/H090-744-V1(0)/2009
NA	CY111837	H1N1	07/24/2009	Hong Kong	A/Hong Kong/H090-738-V10/2009
NA	CY069284	H1N1	07/24/2009	Nicaragua	A/Managua/3467.01/2009(H1N1)
NA	CY062530	H1N1	07/24/2009	Mexico	A/Mexico city/CIA7/2009(H1N1)
NA	GQ496148	H1N1	07/24/2009	Russia	A/Vladivostok/IIV18/2009(H1N1)
NA	CY069292	H1N1	07/24/2009	Nicaragua	A/Managua/4951.02/2009(H1N1)

NA	CY111845	H1N1	07/24/2009	Hong Kong	A/Hong Kong/H090-742-V10/2009(H1N1)
NA	CY047156	H1N1	07/24/2009	Spain	A/Catalonia/S1153/2009(H1N1)
NA	HM624082	H1N1	07/24/2009	Australia	A/Perth/265/2009
NA	CY055702	H1N1	07/24/2009	Australia	A/Australia/36/2009(H1N1)
NA	KC780094	H1N1	07/25/2009	USA	A/Utah/20/2009
NA	KC782198	H1N1	07/25/2009	USA	A/Utah/20/2009
NA	CY128093	H1N1	07/25/2009	Viet Nam	A/Viet Nam/818/2009
NA	CY055371	H1N1	07/25/2009	Italy	A/Rome/649/2009(H1N1)
NA	CY069268	H1N1	07/25/2009	Nicaragua	A/Managua/1264.03/2009(H1N1)
NA	CY088696	H1N1	07/25/2009	India	A/Pune/NIV759/2009
NA	CY055734	H1N1	07/25/2009	Australia	A/Australia/42/2009(H1N1)
NA	HQ239962	H1N1	07/25/2009	Canada	A/Canada-AB/RV2814/2009
NA	CY051665	H1N1	07/25/2009	USA	A/New York/4735/2009(H1N1)
NA	KC781461	H1N1	07/26/2009	USA	A/Hawaii/22/2009
NA	CY075780	H1N1	07/26/2009	Thailand	A/Thailand/CU-H300/2009(H1N1)
NA	CY055750	H1N1	07/26/2009	Australia	A/Australia/44/2009(H1N1)
NA	CY055742	H1N1	07/26/2009	Australia	A/Australia/43/2009(H1N1)
NA	CY055373	H1N1	07/26/2009	Italy	A/Rome/650/2009(H1N1)
NA	KT181521	H1N1	07/26/2009	Singapore	A/Singapore/DMS17/2009
NA	CY047072	H1N1	07/26/2009	Spain	A/Catalonia/NS1586/2009(H1N1)
NA	GU056997	H1N1	07/26/2009	China	A/Chengdu/03/2009(H1N1)
NA	GU056998	H1N1	07/26/2009	China	A/Chengdu/04/2009(H1N1)
NA	GU056999	H1N1	07/26/2009	China	A/Chengdu/06/2009(H1N1)
NA	GU057000	H1N1	07/26/2009	China	A/Chengdu/14/2009(H1N1)
NA	GU057001	H1N1	07/26/2009	China	A/Chengdu/17/2009(H1N1)
NA	GU057002	H1N1	07/26/2009	China	A/Chengdu/18/2009(H1N1)
NA	GU057003	H1N1	07/26/2009	China	A/Chengdu/21/2009(H1N1)
NA	GU057004	H1N1	07/26/2009	China	A/Chengdu/31/2009(H1N1)
NA	GU057005	H1N1	07/26/2009	China	A/Chengdu/33/2009(H1N1)
NA	GU057006	H1N1	07/26/2009	China	A/Chengdu/39/2009(H1N1)
NA	GU057007	H1N1	07/26/2009	China	A/Chengdu/40/2009(H1N1)
NA	GU057008	H1N1	07/26/2009	China	A/Chengdu/47/2009(H1N1)
NA	GU057009	H1N1	07/26/2009	China	A/Chengdu/54/2009(H1N1)
NA	CY128109	H1N1	07/26/2009	Viet Nam	A/Viet Nam/823/2009
NA	KC781837	H1N1	07/27/2009	USA	A/South Carolina/28/2009
NA	CY075786	H1N1	07/27/2009	Thailand	A/Thailand/CU-H302/2009(H1N1)
NA	CY122821	H1N1	07/27/2009	Singapore	A/Singapore/GP2224/2009
NA	CY128101	H1N1	07/27/2009	Viet Nam	A/Viet Nam/835/2009
NA	CY052369	H1N1	07/27/2009	USA	A/Texas/43272683/2009(H1N1)
NA	CY055758	H1N1	07/27/2009	Australia	A/Australia/45/2009(H1N1)
NA	CY054997	H1N1	07/27/2009	USA	A/California/VRDL61/2009(H1N1)
NA	CY055766	H1N1	07/27/2009	Australia	A/Australia/47/2009(H1N1)
NA	CY055774	H1N1	07/27/2009	Australia	A/Australia/48/2009(H1N1)
NA	CY122813	H1N1	07/27/2009	Singapore	A/Singapore/GP2223/2009
NA	CY055377	H1N1	07/27/2009	Italy	A/Rome/653/2009(H1N1)
NA	CY072624	H1N1	07/27/2009	Nicaragua	A/Managua/5339.04/2009(H1N1)
NA	CY073659	H1N1	07/27/2009	Nicaragua	A/Managua/3225.03/2009(H1N1)
NA	CY073531	H1N1	07/27/2009	Nicaragua	A/Managua/466.01/2009(H1N1)
NA	HQ256639	H1N1	07/27/2009	Myanmar	A/Myanmar/M374/2009(H1N1)
NA	JF789631	H1N1	07/27/2009	Kazakhstan	A/Astana/S2/2009
NA	CY053923	H1N1	07/27/2009	Argentina	A/Argentina/HNRG23/2009(H1N1)
NA	JF789630	H1N1	07/27/2009	Kazakhstan	A/Astana/S1/2009
NA	HQ247648	H1N1	07/27/2009	Finland	A/Finland/615/2009
NA	HQ247649	H1N1	07/27/2009	Finland	A/Finland/616/2009

NA	JN256889	H1N1	07/27/2009	Hong Kong	A/Hong Kong/H090-745-V1(0)/2009
NA	CY128125	H1N1	07/27/2009	Viet Nam	A/Viet Nam/841/2009
NA	GU071976	H1N1	07/27/2009	Iran	A/Lorestan/1599/2009(H1N1)
NA	CY065930	H1N1	07/27/2009	Guam	A/Guam/NHRC0001/2009(H1N1)
NA	KF695075	H1N1	07/27/2009	Singapore	A/Singapore/385K/2009
NA	KF695076	H1N1	07/27/2009	Singapore	A/Singapore/386T/2009
NA	CY111861	H1N1	07/27/2009	Hong Kong	A/Hong Kong/H090-745-V10/2009(H1N1)
NA	HM998847	H1N1	07/27/2009	China	A/Inner Mongolia/01/2009(H1N1)
NA	CY072728	H1N1	07/27/2009	Nicaragua	A/Managua/4407.02/2009(H1N1)
NA	JF789632	H1N1	07/27/2009	Kazakhsta	A/Astana/S3/2009
NA	JF789633	H1N1	07/27/2009	Kazakhsta	A/Astana/S4/2009
NA	JQ173150	H1N1	07/27/2009	Finland	A/Helsinki/P14/2009
NA	CY083330	H1N1	07/27/2009	USA	A/Cape May/WRAIR1653P/2009
NA	JN187183	H1N1	07/27/2009	Taiwan	A/Taiwan/2826/2009
NA	CY064908	H1N1	07/27/2009	Italy	A/Rome/693/2009(H1N1)
NA	CY053470	H1N1	07/27/2009	Argentina	A/Argentina/18739/2009
NA	CY120086	H1N1	07/27/2009	Hong Kong	A/Hong Kong/H090-745-V10/2009
NA	CY055782	H1N1	07/28/2009	Australia	A/Australia/51/2009(H1N1)
NA	HQ239957	H1N1	07/28/2009	Canada	A/Canada-AB/RV2809/2009
NA	KC781673	H1N1	07/28/2009	USA	A/Washington/29/2009
NA	CY120295	H1N1	07/28/2009	Hong Kong	A/Hong Kong/H090-746-V10/2009
NA	CY120287	H1N1	07/28/2009	Hong Kong	A/Hong Kong/H090-742-V33/2009
NA	CY128117	H1N1	07/28/2009	Viet Nam	A/Viet Nam/850/2009
NA	CY122805	H1N1	07/28/2009	Singapore	A/Singapore/GP2221/2009
NA	CY073635	H1N1	07/28/2009	Nicaragua	A/Managua/4960.01/2009(H1N1)
NA	CY051673	H1N1	07/28/2009	USA	A/New York/4738/2009(H1N1)
NA	JN256888	H1N1	07/28/2009	Hong Kong	A/Hong Kong/H090-742-V3(3)/2009
NA	JN256891	H1N1	07/28/2009	Hong Kong	A/Hong Kong/H090-746-V1(0)/2009
NA	CY122845	H1N1	07/28/2009	Singapore	A/Singapore/GP2252/2009
NA	CY122829	H1N1	07/28/2009	Singapore	A/Singapore/GP2238/2009
NA	CY123478	H1N1	07/28/2009	Singapore	A/Singapore/ON1576/2009
NA	CY111853	H1N1	07/28/2009	Hong Kong	A/Hong Kong/H090-742-V33/2009
NA	CY119532	H1N1	07/28/2009	Malaysia	A/Malaysia/2072040/2009
NA	CY055388	H1N1	07/28/2009	Italy	A/Rome/658/2009(H1N1)
NA	KF695077	H1N1	07/28/2009	Singapore	A/Singapore/390T/2009
NA	KF695078	H1N1	07/28/2009	Singapore	A/Singapore/392N/2009
NA	GQ499335	H1N1	07/28/2009	USA	A/Washington/29/2009(H1N1)
NA	CY111877	H1N1	07/28/2009	Hong Kong	A/Hong Kong/H090-746-V10/2009(H1N1)
NA	CY083242	H1N1	07/28/2009	USA	A/Virginia/WRAIR1511P/2009
NA	CY055386	H1N1	07/28/2009	Italy	A/Rome/657/2009(H1N1)
NA	CY047174	H1N1	07/28/2009	Spain	A/Catalonia/S1173/2009(H1N1)
NA	CY047159	H1N1	07/28/2009	Spain	A/Catalonia/S1161/2009(H1N1)
NA	CY047162	H1N1	07/28/2009	Spain	A/Catalonia/S1162/2009(H1N1)
NA	CY047168	H1N1	07/28/2009	Spain	A/Catalonia/S1169/2009(H1N1)
NA	CY047171	H1N1	07/28/2009	Spain	A/Catalonia/S1171/2009(H1N1)
NA	CY047186	H1N1	07/28/2009	Spain	A/Catalonia/S1183/2009(H1N1)
NA	CY064909	H1N1	07/28/2009	Italy	A/Rome/694/2009(H1N1)
NA	KC781636	H1N1	07/28/2009	USA	A/Washington/49/2009
NA	KC781918	H1N1	07/29/2009	USA	A/South Carolina/31/2009
NA	HQ239892	H1N1	07/29/2009	Canada	A/Canada-MB/RV2041-10/2009
NA	CY120303	H1N1	07/29/2009	Hong Kong	A/Hong Kong/H090-751-V10/2009
NA	CY122873	H1N1	07/29/2009	Singapore	A/Singapore/GP2297/2009
NA	CY128453	H1N1	07/29/2009	Viet Nam	A/Viet Nam/001-1880/2009
NA	KP637536	H1N1	07/29/2009	Thailand	A/Thailand/SN08294/2009

NA	CY052284	H1N1	07/29/2009	USA	A/Texas/43292238/2009(H1N1)
NA	CY073555	H1N1	07/29/2009	Nicaragua	A/Managua/2978.04/2009(H1N1)
NA	CY065946	H1N1	07/29/2009	Guam	A/Guam/NHRC0003/2009(H1N1)
NA	CY083226	H1N1	07/29/2009	USA	A/California/WRAIR1507P/2009
NA	JN256893	H1N1	07/29/2009	Hong Kong	A/Hong Kong/H090-751-V1(0)/2009
NA	CY122797	H1N1	07/29/2009	Singapore	A/Singapore/GP2215/2009
NA	CY122837	H1N1	07/29/2009	Singapore	A/Singapore/GP2242/2009
NA	CY111893	H1N1	07/29/2009	Hong Kong	A/Hong Kong/H090-751-V10/2009
NA	KT181522	H1N1	07/29/2009	Singapore	A/Singapore/DMS18/2009
NA	CY055790	H1N1	07/29/2009	Australia	A/Australia/53/2009(H1N1)
NA	KF695079	H1N1	07/29/2009	Singapore	A/Singapore/394T/2009
NA	KF695080	H1N1	07/29/2009	Singapore	A/Singapore/395T/2009
NA	GU071975	H1N1	07/29/2009	Iran	A/Khorasan/1583/2009(H1N1)
NA	KC781540	H1N1	07/29/2009	USA	A/South Carolina/16/2009
NA	CY047183	H1N1	07/29/2009	Spain	A/Catalonia/S1182/2009(H1N1)
NA	CY047075	H1N1	07/29/2009	Spain	A/Catalonia/NS1706/2009(H1N1)
NA	CY064910	H1N1	07/29/2009	Italy	A/Rome/695/2009(H1N1)
NA	CY064911	H1N1	07/29/2009	Italy	A/Rome/696/2009(H1N1)
NA	CY064912	H1N1	07/29/2009	Italy	A/Rome/697/2009(H1N1)
NA	CY052091	H1N1	07/29/2009	Italy	A/Italy/232/2009(H1N1)
NA	KP317313	H1N1	07/29/2009	India	A/Delhi/018/2009
NA	CY075792	H1N1	07/29/2009	Thailand	A/Thailand/CU-H313/2009(H1N1)
NA	KC781492	H1N1	07/29/2009	USA	A/Hawaii/25/2009
NA	CY120359	H1N1	07/30/2009	Hong Kong	A/Hong Kong/H090-758-V10/2009
NA	CY111957	H1N1	07/30/2009	Hong Kong	A/Hong Kong/H090-759-V10/2009
NA	CY122881	H1N1	07/30/2009	Singapore	A/Singapore/GP2315/2009
NA	CY122861	H1N1	07/30/2009	Singapore	A/Singapore/GP2290/2009
NA	KP637328	H1N1	07/30/2009	Thailand	A/Thailand/KS08277/2009
NA	KC781724	H1N1	07/30/2009	USA	A/Oregon/27/2009
NA	CY111885	H1N1	07/30/2009	Hong Kong	A/Hong Kong/H090-746-V20/2009
NA	CY123482	H1N1	07/30/2009	Singapore	A/Singapore/ON1616/2009
NA	CY123480	H1N1	07/30/2009	Singapore	A/Singapore/ON1609/2009
NA	CY055806	H1N1	07/30/2009	Australia	A/Australia/56/2009(H1N1)
NA	KC782295	H1N1	07/30/2009	USA	A/Kentucky/16/2009
NA	CY064913	H1N1	07/30/2009	Italy	A/Rome/698/2009(H1N1)
NA	CY047177	H1N1	07/30/2009	Spain	A/Catalonia/S1179/2009(H1N1)
NA	CY047165	H1N1	07/30/2009	Spain	A/Catalonia/S1165/2009(H1N1)
NA	CY047180	H1N1	07/30/2009	Spain	A/Catalonia/S1181/2009(H1N1)
NA	CY047189	H1N1	07/30/2009	Spain	A/Catalonia/S1187/2009(H1N1)
NA	HE804113	H1N1	07/30/2009	Uruguay	A/Uruguay/815/2009
NA	HQ239895	H1N1	07/30/2009	Canada	A/Canada-MB/RV2042-10/2009
NA	CY051681	H1N1	07/30/2009	USA	A/New York/4747/2009(H1N1)
NA	CY122889	H1N1	07/30/2009	Singapore	A/Singapore/GP2317/2009
NA	CY122853	H1N1	07/30/2009	Singapore	A/Singapore/GP2286/2009
NA	CY120367	H1N1	07/30/2009	Hong Kong	A/Hong Kong/H090-759-V10/2009
NA	KY925418	H1N1	07/30/2009	Brazil	A/Porto Alegre/LACENRS-1771/2009
NA	GU071974	H1N1	07/30/2009	Iran	A/Ghom/1550/2009(H1N1)
NA	CY072632	H1N1	07/30/2009	Nicaragua	A/Managua/1836.02/2009(H1N1)
NA	KY925507	H1N1	07/30/2009	Brazil	A/Tramandai/LACENRS-324/2009
NA	KM013714	H1N1	07/30/2009	Thailand	A/Bangkok/SIMI501/2009
NA	KM013720	H1N1	07/30/2009	Thailand	A/Bangkok/SIMI507/2009
NA	HQ256617	H1N1	07/30/2009	Myanmar	A/Myanmar/M253/2009(H1N1)
NA	CY066144	H1N1	07/30/2009	Japan	A/Nagasaki/09N019/2009
NA	JN256892	H1N1	07/30/2009	Hong Kong	A/Hong Kong/H090-746-V2(0)/2009

NA	JN256899	H1N1	07/30/2009	Hong Kong	A/Hong Kong/H090-757-V1(0)/2009
NA	JN256900	H1N1	07/30/2009	Hong Kong	A/Hong Kong/H090-758-V1(0)/2009
NA	JN256901	H1N1	07/30/2009	Hong Kong	A/Hong Kong/H090-759-V1(0)/2009
NA	CY122849	H1N1	07/30/2009	Singapore	A/Singapore/GP2266/2009
NA	CY122869	H1N1	07/30/2009	Singapore	A/Singapore/GP2293/2009
NA	CY111949	H1N1	07/30/2009	Hong Kong	A/Hong Kong/H090-758-V10/2009
NA	CY111941	H1N1	07/30/2009	Hong Kong	A/Hong Kong/H090-757-V10/2009
NA	CY120351	H1N1	07/30/2009	Hong Kong	A/Hong Kong/H090-757-V10/2009
NA	CY120311	H1N1	07/31/2009	Hong Kong	A/Hong Kong/H090-751-V20/2009
NA	CY119364	H1N1	07/31/2009	Malaysia	A/Malaysia/2068631/2009
NA	CY120383	H1N1	07/31/2009	Hong Kong	A/Hong Kong/H090-762-V10/2009
NA	CY120375	H1N1	07/31/2009	Hong Kong	A/Hong Kong/H090-761-V10/2009
NA	CY120319	H1N1	07/31/2009	Hong Kong	A/Hong Kong/H090-751-V23/2009
NA	CY111869	H1N1	07/31/2009	Hong Kong	A/Hong Kong/H090-745-V24/2009
NA	CY055247	H1N1	07/31/2009	Malaysia	A/Malaysia/5670/2009(H1N1)
NA	KC782110	H1N1	07/31/2009	USA	A/South Carolina/30/2009
NA	KC782298	H1N1	07/31/2009	USA	A/Illinois/10/2009
NA	CY120741	H1N1	07/31/2009	Brazil	A/Brazil/AVS07/2009
NA	CY047078	H1N1	07/31/2009	Spain	A/Catalonia/NS1875/2009(H1N1)
NA	CY049621	H1N1	07/31/2009	Singapore	A/Singapore/GP2312/2009(H1N1)
NA	CY049629	H1N1	07/31/2009	Singapore	A/Singapore/GP2316/2009(H1N1)
NA	CY056364	H1N1	07/31/2009	Spain	A/Madrid/RC5/2009(H1N1)
NA	JN256894	H1N1	07/31/2009	Hong Kong	A/Hong Kong/H090-751-V2(0)/2009
NA	KY925637	H1N1	07/31/2009	Brazil	A/Porto Alegre/LACENRS-1786/2009
NA	KF695083	H1N1	07/31/2009	Singapore	A/Singapore/405S/2009
NA	KF695085	H1N1	07/31/2009	Singapore	A/Singapore/410W/2009
NA	KF695088	H1N1	07/31/2009	Singapore	A/Singapore/418W/2009
NA	KF695089	H1N1	07/31/2009	Singapore	A/Singapore/419B/2009
NA	KF695081	H1N1	07/31/2009	Singapore	A/Singapore/401T/2009
NA	KF695082	H1N1	07/31/2009	Singapore	A/Singapore/404C/2009
NA	KF695084	H1N1	07/31/2009	Singapore	A/Singapore/406K/2009
NA	KF695086	H1N1	07/31/2009	Singapore	A/Singapore/413Y/2009
NA	KF695087	H1N1	07/31/2009	Singapore	A/Singapore/416C/2009
NA	CY068932	H1N1	07/31/2009	Guam	A/Guam/NHRC0005/2009(H1N1)
NA	CY071572	H1N1	07/31/2009	USA	A/South Carolina/WR1325P/2009(H1N1)
NA	CY071580	H1N1	07/31/2009	USA	A/South Carolina/WR1326P/2009(H1N1)
NA	CY070985	H1N1	07/31/2009	Guam	A/Guam/NHRC0007/2009(H1N1)
NA	CY111901	H1N1	07/31/2009	Hong Kong	A/Hong Kong/H090-751-V20/2009(H1N1)
NA	CY111917	H1N1	07/31/2009	Hong Kong	A/Hong Kong/H090-751-V24/2009(H1N1)
NA	KY924972	H1N1	07/31/2009	Brazil	A/Estrela/LACENRS-313/2009
NA	KY925572	H1N1	07/31/2009	Brazil	A/Igrejinha/LACENRS-1803/2009
NA	CY083218	H1N1	07/31/2009	USA	A/New Jersey/WRAIR1506P/2009(H1N1)
NA	JN256890	H1N1	07/31/2009	Hong Kong	A/Hong Kong/H090-745-V2(4)/2009
NA	JN256903	H1N1	07/31/2009	Hong Kong	A/Hong Kong/H090-762-V1(0)/2009
NA	JN256895	H1N1	07/31/2009	Hong Kong	A/Hong Kong/H090-751-V2(3)/2009
NA	JN256896	H1N1	07/31/2009	Hong Kong	A/Hong Kong/H090-751-V2(4)/2009
NA	JN256902	H1N1	07/31/2009	Hong Kong	A/Hong Kong/H090-761-V1(0)/2009
NA	JN256904	H1N1	07/31/2009	Hong Kong	A/Hong Kong/H090-765-V1(0)/2009
NA	CY120391	H1N1	07/31/2009	Hong Kong	A/Hong Kong/H090-765-V10/2009
NA	CY111965	H1N1	07/31/2009	Hong Kong	A/Hong Kong/H090-761-V10/2009
NA	CY111909	H1N1	07/31/2009	Hong Kong	A/Hong Kong/H090-751-V23/2009
NA	CY111981	H1N1	07/31/2009	Hong Kong	A/Hong Kong/H090-765-V10/2009
NA	CY111973	H1N1	07/31/2009	Hong Kong	A/Hong Kong/H090-762-V10/2009
NA	CY120327	H1N1	07/31/2009	Hong Kong	A/Hong Kong/H090-751-V24/2009

NA	GU134733	H1N1	08/2009	Italy	A/Italy/180/2009(H1N1)
NA	GU134734	H1N1	08/2009	Italy	A/Italy/181/2009(H1N1)
NA	GU983250	H1N1	08/2009	India	A/Eastern India/261/2009
NA	JN381361	H1N1	08/2009	Taiwan	A/Taiwan/97888/2009
NA	JN381357	H1N1	08/2009	Taiwan	A/Taiwan/90052/2009
NA	JN381360	H1N1	08/2009	Taiwan	A/Taiwan/97674/2009
NA	JN381358	H1N1	08/2009	Taiwan	A/Taiwan/90060/2009
NA	JN381362	H1N1	08/2009	Taiwan	A/Taiwan/98031/2009
NA	JN381363	H1N1	08/2009	Taiwan	A/Taiwan/98158/2009
NA	JN006471	H1N1	08/2009	China	A/Hangzhou/3/2009
NA	HM446344	H1N1	08/2009	Thailand	A/Thailand/CU-H88/2009
NA	JN381359	H1N1	08/2009	Taiwan	A/Taiwan/97565/2009
NA	GU292384	H1N1	08/2009	India	A/Pune/NIV6196/2009(H1N1)
NA	GU292385	H1N1	08/2009	India	A/Pune/NIV6447/2009(H1N1)
NA	GU292386	H1N1	08/2009	India	A/Pune/NIV8489/2009(H1N1)
NA	CY050341	H1N1	08/2009	Singapore	A/Singapore/9061364/2009(H1N1)
NA	CY050349	H1N1	08/2009	Singapore	A/Singapore/9061365/2009(H1N1)
NA	CY050357	H1N1	08/2009	Singapore	A/Singapore/9061366/2009(H1N1)
NA	CY052059	H1N1	08/2009	Belarus	A/Belarus/24/2009(H1N1)
NA	CY052061	H1N1	08/2009	Belarus	A/Belarus/25/2009(H1N1)
NA	CY052065	H1N1	08/2009	Belarus	A/Belarus/39/2009(H1N1)
NA	CY052067	H1N1	08/2009	Belarus	A/Belarus/4/2009(H1N1)
NA	CY052069	H1N1	08/2009	Belarus	A/Belarus/42/2009(H1N1)
NA	CY052057	H1N1	08/2009	Belarus	A/Belarus/23/2009(H1N1)
NA	HM598331	H1N1	08/2009	Thailand	A/Thailand/CU-MV20/2009
NA	HM598347	H1N1	08/2009	Thailand	A/Thailand/CU-MV44/2009
NA	GQ866939	H1N1	08/2009	Thailand	A/Thailand/CU-B938/2009
NA	GU983246	H1N1	08/2009	India	A/Eastern India/74/2009
NA	GU983249	H1N1	08/2009	India	A/Eastern India/176/2009
NA	GU983247	H1N1	08/2009	India	A/Eastern India/107/2009
NA	GU983252	H1N1	08/2009	India	A/Eastern India/327/2009
NA	GU983248	H1N1	08/2009	India	A/Eastern India/109/2009
NA	GU983251	H1N1	08/2009	India	A/Eastern India/312/2009
NA	HM598330	H1N1	08/2009	Thailand	A/Thailand/CU-MV18/2009
NA	HM598348	H1N1	08/2009	Thailand	A/Thailand/CU-MV45/2009
NA	HM598329	H1N1	08/2009	Thailand	A/Thailand/CU-MV17/2009
NA	HM598328	H1N1	08/2009	Thailand	A/Thailand/CU-MV16/2009
NA	GU983253	H1N1	08/2009	India	A/Eastern India/330/2009
NA	AB568229	H1N1	08/2009	Japan	A/Kobe/91799/2009
NA	AB568234	H1N1	08/2009	Japan	A/Kobe/91821/2009
NA	AB568244	H1N1	08/2009	Japan	A/Kobe/91825/2009
NA	AB568249	H1N1	08/2009	Japan	A/Kobe/91873/2009
NA	AB568254	H1N1	08/2009	Japan	A/Kobe/91890/2009
NA	AB568224	H1N1	08/2009	Japan	A/Kobe/91780/2009
NA	AB568219	H1N1	08/2009	Japan	A/Kobe/91738/2009
NA	AB568239	H1N1	08/2009	Japan	A/Kobe/91823/2009
NA	KM035436	H1N1	08/2009	Brazil	A/Rio Grande do Sul/2811/2009
NA	KM035437	H1N1	08/2009	Brazil	A/Rio Grande do Sul/2842/2009
NA	KM035443	H1N1	08/2009	Brazil	A/Rio Grande do Sul/3335/2009
NA	KM035447	H1N1	08/2009	Brazil	A/Rio Grande do Sul/3841/2009
NA	KM035434	H1N1	08/2009	Brazil	A/Rio Grande do Sul/2722/2009
NA	KM035438	H1N1	08/2009	Brazil	A/Rio Grande do Sul/2905/2009
NA	KM035444	H1N1	08/2009	Brazil	A/Rio Grande do Sul/3340/2009
NA	KM035431	H1N1	08/2009	Brazil	A/Rio Grande do Sul/2656/2009

NA	KM035433	H1N1	08/2009	Brazil	A/Rio Grande do Sul/2721/2009
NA	KM035439	H1N1	08/2009	Brazil	A/Rio Grande do Sul/2937/2009
NA	KM035449	H1N1	08/2009	Brazil	A/Rio Grande do Sul/4078/2009
NA	KM035452	H1N1	08/2009	Brazil	A/Rio Grande do Sul/5063/2009
NA	KM035430	H1N1	08/2009	Brazil	A/Rio Grande do Sul/2648/2009
NA	KM035435	H1N1	08/2009	Brazil	A/Rio Grande do Sul/2737/2009
NA	KM035442	H1N1	08/2009	Brazil	A/Rio Grande do Sul/3093/2009
NA	GQ866938	H1N1	08/2009	Thailand	A/Thailand/CU-H340/2009
NA	GU134731	H1N1	08/2009	Italy	A/Italy/178/2009(H1N1)
NA	CY064250	H1N1	08/2009	China	A/Guangdong/1001/2009
NA	CY064388	H1N1	08/2009	China	A/Guangdong/982/2009
NA	CY064253	H1N1	08/2009	China	A/Guangdong/1016/2009
NA	GU134732	H1N1	08/2009	Italy	A/Italy/179/2009(H1N1)
NA	KC781510	H1N1	08/01/2009	USA	A/Colorado/11/2009
NA	KC780433	H1N1	08/01/2009	USA	A/Texas/71/2009
NA	KT181523	H1N1	08/01/2009	Singapore	A/Singapore/DMS19/2009
NA	CY055397	H1N1	08/01/2009	Italy	A/Rome/662/2009(H1N1)
NA	CY055401	H1N1	08/01/2009	Italy	A/Rome/664/2009(H1N1)
NA	CY055399	H1N1	08/01/2009	Italy	A/Rome/663/2009(H1N1)
NA	KC781562	H1N1	08/01/2009	USA	A/Iowa/07/2009
NA	CY055814	H1N1	08/01/2009	Australia	A/Australia/57/2009(H1N1)
NA	CY055822	H1N1	08/01/2009	Australia	A/Australia/58/2009(H1N1)
NA	CY075822	H1N1	08/01/2009	Thailand	A/Thailand/CU-H491/2009(H1N1)
NA	CY055838	H1N1	08/02/2009	Australia	A/Australia/60/2009(H1N1)
NA	KC781854	H1N1	08/02/2009	USA	A/Nevada/10/2009
NA	KC781814	H1N1	08/02/2009	USA	A/Nevada/09/2009
NA	CY083170	H1N1	08/02/2009	USA	A/New Jersey/WRAIR1499P/2009(H1N1)
NA	CY111933	H1N1	08/02/2009	Hong Kong	A/Hong Kong/H090-751-V34/2009
NA	CY120335	H1N1	08/02/2009	Hong Kong	A/Hong Kong/H090-751-V31/2009
NA	JN703385	H1N1	08/02/2009	Philippin	A/Philippines/TMC09_245/2009
NA	CY118201	H1N1	08/02/2009	Malaysia	A/Malaysia/2076212/2009
NA	CY119372	H1N1	08/02/2009	Malaysia	A/Malaysia/2076204/2009
NA	CY055830	H1N1	08/02/2009	Australia	A/Australia/59/2009(H1N1)
NA	CY111925	H1N1	08/02/2009	Hong Kong	A/Hong Kong/H090-751-V31/2009(H1N1)
NA	JN256897	H1N1	08/02/2009	Hong Kong	A/Hong Kong/H090-751-V3(1)/2009
NA	JN256898	H1N1	08/02/2009	Hong Kong	A/Hong Kong/H090-751-V3(4)/2009
NA	CY120343	H1N1	08/02/2009	Hong Kong	A/Hong Kong/H090-751-V34/2009
NA	CY055846	H1N1	08/03/2009	Australia	A/Australia/61/2009(H1N1)
NA	CY055870	H1N1	08/03/2009	Australia	A/Australia/65/2009(H1N1)
NA	KC781766	H1N1	08/03/2009	USA	A/New Hampshire/17/2009
NA	JX625477	H1N1	08/03/2009	United Ki	A/England/646/2009
NA	CY119388	H1N1	08/03/2009	Malaysia	A/Malaysia/2077462/2009
NA	CY119380	H1N1	08/03/2009	Malaysia	A/Malaysia/2076039/2009
NA	CY120407	H1N1	08/03/2009	Hong Kong	A/Hong Kong/H090-771-V10/2009
NA	CY066146	H1N1	08/03/2009	Japan	A/Nagasaki/09N020/2009
NA	CY122961	H1N1	08/03/2009	Singapore	A/Singapore/GP2714/2009
NA	GU576509	H1N1	08/03/2009	Italy	A/Ancona/56/2009(H1N1)
NA	CY055405	H1N1	08/03/2009	Italy	A/Rome/666/2009(H1N1)
NA	CY055854	H1N1	08/03/2009	Australia	A/Australia/63/2009(H1N1)
NA	CY055862	H1N1	08/03/2009	Australia	A/Australia/64/2009(H1N1)
NA	KF695092	H1N1	08/03/2009	Singapore	A/Singapore/437Y/2009
NA	KF695093	H1N1	08/03/2009	Singapore	A/Singapore/440F/2009
NA	KF695090	H1N1	08/03/2009	Singapore	A/Singapore/420T/2009
NA	KF695091	H1N1	08/03/2009	Singapore	A/Singapore/426N/2009

NA	KF695094	H1N1	08/03/2009	Singapore	A/Singapore/441T/2009
NA	CY072560	H1N1	08/03/2009	Nicaragua	A/Managua/451.01/2009(H1N1)
NA	CY072680	H1N1	08/03/2009	Nicaragua	A/Managua/209.01/2009(H1N1)
NA	JN256688	H1N1	08/03/2009	Hong Kong	A/Hong Kong/H090-766-V1(0)/2009
NA	JN256906	H1N1	08/03/2009	Hong Kong	A/Hong Kong/H090-771-V1(0)/2009
NA	JX556116	H1N1	08/03/2009	Cuba	A/Habana/2030/2009
NA	CY047081	H1N1	08/03/2009	Spain	A/Catalonia/NS2001/2009(H1N1)
NA	CY052354	H1N1	08/03/2009	United Ar	A/UAE/D02/2009(H1N1)
NA	HE804105	H1N1	08/03/2009	Uruguay	A/Uruguay/835/2009
NA	HE804106	H1N1	08/03/2009	Uruguay	A/Uruguay/825/2009
NA	CY047084	H1N1	08/03/2009	Spain	A/Catalonia/NS2008/2009(H1N1)
NA	CY111997	H1N1	08/03/2009	Hong Kong	A/Hong Kong/H090-771-V10/2009
NA	CY106978	H1N1	08/03/2009	Hong Kong	A/Hong Kong/H090-770-V10/2009
NA	KC781825	H1N1	08/03/2009	USA	A/North Dakota/12/2009
NA	KC781496	H1N1	08/04/2009	USA	A/Montana/15/2009
NA	HQ239955	H1N1	08/04/2009	Canada	A/Canada-MB/RV2661/2009
NA	CY120423	H1N1	08/04/2009	Hong Kong	A/Hong Kong/H090-774-V10/2009
NA	CY119524	H1N1	08/04/2009	Malaysia	A/Malaysia/2076756/2009
NA	CY122897	H1N1	08/04/2009	Singapore	A/Singapore/GP2640/2009
NA	CY055407	H1N1	08/04/2009	Italy	A/Rome/667/2009(H1N1)
NA	CY123488	H1N1	08/04/2009	Singapore	A/Singapore/ON1666/2009
NA	CY122905	H1N1	08/04/2009	Singapore	A/Singapore/GP2649/2009
NA	CY073611	H1N1	08/04/2009	Nicaragua	A/Managua/1256.04/2009(H1N1)
NA	CY070977	H1N1	08/04/2009	Guam	A/Guam/NHRC0006/2009(H1N1)
NA	KF695095	H1N1	08/04/2009	Singapore	A/Singapore/443N/2009
NA	KF695096	H1N1	08/04/2009	Singapore	A/Singapore/444W/2009
NA	CY073603	H1N1	08/04/2009	Nicaragua	A/Managua/4451.02/2009(H1N1)
NA	CY068948	H1N1	08/04/2009	Japan	A/Japan/NHRC0004/2009(H1N1)
NA	JN703386	H1N1	08/04/2009	Philippin	A/Philippines/TMC09_262/2009
NA	JN256909	H1N1	08/04/2009	Hong Kong	A/Hong Kong/H090-774-V1(0)/2009
NA	CY047195	H1N1	08/04/2009	Spain	A/Catalonia/S1196/2009(H1N1)
NA	HQ239974	H1N1	08/04/2009	Canada	A/Canada-AB/RV2828/2009
NA	CY047087	H1N1	08/04/2009	Spain	A/Catalonia/NS2045/2009(H1N1)
NA	CY112021	H1N1	08/04/2009	Hong Kong	A/Hong Kong/H090-774-V10/2009
NA	CY055878	H1N1	08/04/2009	Australia	A/Australia/66/2009(H1N1)
NA	KT181524	H1N1	08/04/2009	Singapore	A/Singapore/DMS20/2009
NA	KC781906	H1N1	08/05/2009	USA	A/Colorado/12/2009
NA	CY049637	H1N1	08/05/2009	Singapore	A/Singapore/GP2641/2009(H1N1)
NA	KC781661	H1N1	08/05/2009	USA	A/Wisconsin/45/2009
NA	KC782045	H1N1	08/05/2009	USA	A/North Carolina/28/2009
NA	CY071500	H1N1	08/05/2009	USA	A/California/WR1316P/2009(H1N1)
NA	CY071492	H1N1	08/05/2009	USA	A/California/WR1315P/2009(H1N1)
NA	CY071484	H1N1	08/05/2009	USA	A/California/WR1314P/2009(H1N1)
NA	CY122937	H1N1	08/05/2009	Singapore	A/Singapore/GP2693/2009
NA	CY112029	H1N1	08/05/2009	Hong Kong	A/Hong Kong/H090-779-V10/2009
NA	CY112037	H1N1	08/05/2009	Hong Kong	A/Hong Kong/H090-779-V12/2009
NA	CY112013	H1N1	08/05/2009	Hong Kong	A/Hong Kong/H090-772-V10/2009
NA	CY112005	H1N1	08/05/2009	Hong Kong	A/Hong Kong/H090-771-V20/2009
NA	CY120439	H1N1	08/05/2009	Hong Kong	A/Hong Kong/H090-779-V12/2009
NA	CY122945	H1N1	08/05/2009	Singapore	A/Singapore/GP2697/2009
NA	CY122921	H1N1	08/05/2009	Singapore	A/Singapore/GP2672/2009
NA	CY120415	H1N1	08/05/2009	Hong Kong	A/Hong Kong/H090-772-V10/2009
NA	CY120431	H1N1	08/05/2009	Hong Kong	A/Hong Kong/H090-779-V10/2009
NA	CY055037	H1N1	08/05/2009	USA	A/California/VRDL69/2009(H1N1)

NA	HQ239897	H1N1	08/05/2009	Canada	A/Canada-MB/RV2043-10/2009
NA	KJ690505	H1N1	08/05/2009	Uganda	A/Uganda/MUWRP-067/2009
NA	GU471691	H1N1	08/05/2009	China	A/Guangdong/801/2009
NA	KY925341	H1N1	08/05/2009	Brazil	A/Teutonia/LACENRS-711/2009
NA	KY926379	H1N1	08/05/2009	Brazil	A/Nonoai/LACENRS-714/2009
NA	HQ256641	H1N1	08/05/2009	Myanmar	A/Myanmar/M411/2009(H1N1)
NA	KJ690410	H1N1	08/05/2009	Uganda	A/Uganda/MUWRP-066/2009
NA	CY072600	H1N1	08/05/2009	Nicaragua	A/Managua/1077.04/2009(H1N1)
NA	CY073571	H1N1	08/05/2009	Nicaragua	A/Managua/5230.01/2009(H1N1)
NA	JN256907	H1N1	08/05/2009	Hong Kong	A/Hong Kong/H090-771-V2(0)/2009
NA	JN256908	H1N1	08/05/2009	Hong Kong	A/Hong Kong/H090-772-V1(0)/2009
NA	JN256910	H1N1	08/05/2009	Hong Kong	A/Hong Kong/H090-779-V1(0)/2009
NA	JN256911	H1N1	08/05/2009	Hong Kong	A/Hong Kong/H090-779-V1(2)/2009
NA	CY047192	H1N1	08/05/2009	Spain	A/Catalonia/S1194/2009(H1N1)
NA	CY047204	H1N1	08/05/2009	Spain	A/Catalonia/S1205/2009(H1N1)
NA	CY047207	H1N1	08/05/2009	Spain	A/Catalonia/S1206/2009(H1N1)
NA	CY047198	H1N1	08/05/2009	Spain	A/Catalonia/S1198/2009(H1N1)
NA	HQ239899	H1N1	08/05/2009	Canada	A/Canada-MB/RV2044-10/2009
NA	CY049645	H1N1	08/05/2009	Singapore	A/Singapore/GP2651/2009(H1N1)
NA	HE804108	H1N1	08/05/2009	Uruguay	A/Uruguay/78/2009
NA	CY047216	H1N1	08/05/2009	Spain	A/Catalonia/S1209/2009(H1N1)
NA	GU576511	H1N1	08/05/2009	Italy	A/Ancona/60/2009(H1N1)
NA	KC781762	H1N1	08/05/2009	USA	A/Maryland/15/2009
NA	CY119396	H1N1	08/05/2009	Malaysia	A/Malaysia/2082543/2009
NA	CY122929	H1N1	08/05/2009	Singapore	A/Singapore/GP2680/2009
NA	CY055886	H1N1	08/05/2009	Australia	A/Australia/69/2009(H1N1)
NA	KC782103	H1N1	08/05/2009	USA	A/Massachusetts/29/2009
NA	KC781611	H1N1	08/06/2009	USA	A/California/33/2009
NA	KC781943	H1N1	08/06/2009	USA	A/Nevada/14/2009
NA	KC781473	H1N1	08/06/2009	USA	A/Nebraska/13/2009
NA	CY051689	H1N1	08/06/2009	USA	A/New York/4761/2009(H1N1)
NA	CY111989	H1N1	08/06/2009	Hong Kong	A/Hong Kong/H090-770-V22/2009
NA	JN596859	H1N1	08/06/2009	Russia	A/Orenburg/01/2009(H1N1)
NA	JX625485	H1N1	08/06/2009	United Ki	A/England/93320016/2009
NA	CY120399	H1N1	08/06/2009	Hong Kong	A/Hong Kong/H090-770-V22/2009
NA	CY122913	H1N1	08/06/2009	Singapore	A/Singapore/GP2658/2009
NA	GU576513	H1N1	08/06/2009	Italy	A/Ancona/61/2009(H1N1)
NA	HQ239972	H1N1	08/06/2009	Canada	A/Canada-AB/RV2825/2009
NA	CY068924	H1N1	08/06/2009	Guam	A/Guam/NHRC0004/2009(H1N1)
NA	JN256905	H1N1	08/06/2009	Hong Kong	A/Hong Kong/H090-770-V2(2)/2009
NA	CY047201	H1N1	08/06/2009	Spain	A/Catalonia/S1199/2009(H1N1)
NA	CY047210	H1N1	08/06/2009	Spain	A/Catalonia/S1207/2009(H1N1)
NA	CY049669	H1N1	08/06/2009	Singapore	A/Singapore/GP2687/2009(H1N1)
NA	CY049677	H1N1	08/06/2009	Singapore	A/Singapore/GP2695/2009(H1N1)
NA	CY049685	H1N1	08/06/2009	Singapore	A/Singapore/GP2703/2009(H1N1)
NA	CY049693	H1N1	08/06/2009	Singapore	A/Singapore/GP2711/2009(H1N1)
NA	CY049701	H1N1	08/06/2009	Singapore	A/Singapore/GP2715/2009(H1N1)
NA	CY049661	H1N1	08/06/2009	Singapore	A/Singapore/GP2686/2009(H1N1)
NA	HE804114	H1N1	08/06/2009	Uruguay	A/Uruguay/842/2009
NA	KP317296	H1N1	08/06/2009	India	A/Karnataka/001/2009
NA	CY047213	H1N1	08/06/2009	Spain	A/Catalonia/S1208/2009(H1N1)
NA	KF695097	H1N1	08/06/2009	Singapore	A/Singapore/448D/2009
NA	CY122953	H1N1	08/06/2009	Singapore	A/Singapore/GP2709/2009
NA	CY055894	H1N1	08/06/2009	Australia	A/Australia/70/2009(H1N1)

NA	HE804109	H1N1	08/07/2009	Uruguay	A/Uruguay/856/2009
NA	CY071532	H1N1	08/07/2009	USA	A/California/WR1320P/2009(H1N1)
NA	JQ666886	H1N1	08/07/2009	Brazil	A/Minas Gerais/21/2009
NA	KJ958952	H1N1	08/07/2009	India	A/Mumbai/162/2009
NA	CY047219	H1N1	08/07/2009	Spain	A/Catalonia/S1210/2009(H1N1)
NA	KJ958954	H1N1	08/07/2009	India	A/Mumbai/178/2009
NA	KJ958953	H1N1	08/07/2009	India	A/Mumbai/173/2009
NA	KJ958946	H1N1	08/07/2009	India	A/Mumbai/154/2009
NA	KJ958945	H1N1	08/07/2009	India	A/Mumbai/151/2009
NA	KJ958948	H1N1	08/07/2009	India	A/Mumbai/180/2009
NA	KJ958947	H1N1	08/07/2009	India	A/Mumbai/163/2009
NA	KJ958951	H1N1	08/07/2009	India	A/Mumbai/157/2009
NA	HQ239900	H1N1	08/07/2009	Canada	A/Canada-MB/RV2045-10/2009
NA	CY119404	H1N1	08/07/2009	Malaysia	A/Malaysia/2086505/2009
NA	HQ239958	H1N1	08/07/2009	Canada	A/Canada-AB/RV2810/2009
NA	KC781777	H1N1	08/07/2009	USA	A/Michigan/21/2009
NA	CY120463	H1N1	08/07/2009	Hong Kong	A/Hong Kong/H090-785-V10/2009
NA	KT181525	H1N1	08/07/2009	Singapore	A/Singapore/DMS21/2009
NA	CY072616	H1N1	08/07/2009	Nicaragua	A/Managua/2604.02/2009(H1N1)
NA	CY071540	H1N1	08/07/2009	USA	A/California/WR1321P/2009(H1N1)
NA	HM624081	H1N1	08/07/2009	Australia	A/Perth/267/2009
NA	CY112069	H1N1	08/07/2009	Hong Kong	A/Hong Kong/H090-785-V10/2009
NA	HQ239956	H1N1	08/07/2009	Canada	A/Canada-AB/RV2735/2009
NA	CY073523	H1N1	08/07/2009	Nicaragua	A/Managua/5399.04/2009(H1N1)
NA	KJ958961	H1N1	08/07/2009	India	A/Mumbai/183/2009
NA	KJ690465	H1N1	08/07/2009	Uganda	A/Uganda/MUWRP-069/2009
NA	JN256915	H1N1	08/07/2009	Hong Kong	A/Hong Kong/H090-785-V1(0)/2009
NA	JN256913	H1N1	08/08/2009	Hong Kong	A/Hong Kong/H090-779-V2(1)/2009
NA	CY073627	H1N1	08/08/2009	Nicaragua	A/Managua/5595.03/2009(H1N1)
NA	KJ958958	H1N1	08/08/2009	India	A/Mumbai/264/2009
NA	KJ958957	H1N1	08/08/2009	India	A/Mumbai/262/2009
NA	KJ958956	H1N1	08/08/2009	India	A/Mumbai/238/2009
NA	HQ256623	H1N1	08/08/2009	Myanmar	A/Myanmar/M280/2009(H1N1)
NA	CY073587	H1N1	08/08/2009	Nicaragua	A/Managua/4064.01/2009(H1N1)
NA	CY055409	H1N1	08/08/2009	Italy	A/Rome/668/2009(H1N1)
NA	CY055249	H1N1	08/08/2009	Malaysia	A/Malaysia/8860/2009(H1N1)
NA	CY120447	H1N1	08/08/2009	Hong Kong	A/Hong Kong/H090-779-V21/2009
NA	CY120455	H1N1	08/08/2009	Hong Kong	A/Hong Kong/H090-779-V22/2009
NA	CY120471	H1N1	08/08/2009	Hong Kong	A/Hong Kong/H090-787-V10/2009
NA	CY112045	H1N1	08/08/2009	Hong Kong	A/Hong Kong/H090-779-V20/2009
NA	CY112077	H1N1	08/08/2009	Hong Kong	A/Hong Kong/H090-787-V10/2009
NA	CY112061	H1N1	08/08/2009	Hong Kong	A/Hong Kong/H090-779-V22/2009
NA	CY112053	H1N1	08/08/2009	Hong Kong	A/Hong Kong/H090-779-V21/2009
NA	CY120094	H1N1	08/08/2009	Hong Kong	A/Hong Kong/H090-779-V20/2009
NA	CY087018	H1N1	08/08/2009	USA	A/New York/4764/2009(H1N1)
NA	JN256912	H1N1	08/08/2009	Hong Kong	A/Hong Kong/H090-779-V2(0)/2009
NA	KC781734	H1N1	08/08/2009	USA	A/California/37/2009
NA	KC781568	H1N1	08/08/2009	USA	A/Kansas/17/2009
NA	CY075798	H1N1	08/08/2009	Thailand	A/Thailand/CU-H354/2009(H1N1)
NA	HM051341	H1N1	08/08/2009	China	A/Guangdong/SWL28/2009(H1N1)
NA	CY052356	H1N1	08/08/2009	United Ar	A/UAE/D08/2009(H1N1)
NA	KP317303	H1N1	08/08/2009	India	A/Gujarat/008/2009
NA	CY052355	H1N1	08/08/2009	United Ar	A/UAE/D07/2009(H1N1)
NA	CY071468	H1N1	08/08/2009	USA	A/California/WR1312P/2009(H1N1)

NA	JN256916	H1N1	08/08/2009	Hong Kong	A/Hong Kong/H090-787-V1(0)/2009
NA	JN256914	H1N1	08/08/2009	Hong Kong	A/Hong Kong/H090-779-V2(2)/2009
NA	CY060544	H1N1	08/09/2009	Canada	A/Ontario/25389/2009(H1N1)
NA	KC781730	H1N1	08/09/2009	USA	A/Oregon/30/2009
NA	HQ239987	H1N1	08/09/2009	Canada	A/Canada-ON/RV2905/2009
NA	KJ958960	H1N1	08/09/2009	India	A/Mumbai/193/2009
NA	KJ958959	H1N1	08/09/2009	India	A/Mumbai/260/2009
NA	CY119420	H1N1	08/09/2009	Malaysia	A/Malaysia/2089888/2009
NA	KC781743	H1N1	08/09/2009	USA	A/Wyoming/08/2009
NA	CY119412	H1N1	08/09/2009	Malaysia	A/Malaysia/2089841/2009
NA	CY047228	H1N1	08/10/2009	Spain	A/Catalonia/S1222/2009(H1N1)
NA	KC781495	H1N1	08/10/2009	USA	A/Vermont/17/2009
NA	KC781627	H1N1	08/10/2009	USA	A/Delaware/10/2009
NA	KC781890	H1N1	08/10/2009	USA	A/Alaska/34/2009
NA	KC781660	H1N1	08/10/2009	USA	A/Florida/14/2009
NA	KC781539	H1N1	08/10/2009	USA	A/Florida/13/2009
NA	CY055005	H1N1	08/10/2009	USA	A/California/VRDL64/2009(H1N1)
NA	CY055013	H1N1	08/10/2009	USA	A/California/VRDL65/2009(H1N1)
NA	CY119444	H1N1	08/10/2009	Malaysia	A/Malaysia/2098053/2009
NA	CY119428	H1N1	08/10/2009	Malaysia	A/Malaysia/2098001/2009
NA	KT181526	H1N1	08/10/2009	Singapore	A/Singapore/DMS22/2009
NA	CY071516	H1N1	08/10/2009	USA	A/California/WR1318P/2009(H1N1)
NA	JX625244	H1N1	08/10/2009	Paraguay	A/Paraguay/86HCl/2009
NA	JX625243	H1N1	08/10/2009	Paraguay	A/Paraguay/85HCl/2009
NA	CY083194	H1N1	08/10/2009	USA	A/California/WRAIR1502P/2009
NA	CY119436	H1N1	08/10/2009	Malaysia	A/Malaysia/2098044/2009
NA	CY072688	H1N1	08/10/2009	Nicaragua	A/Managua/554.01/2009(H1N1)
NA	CY071508	H1N1	08/10/2009	USA	A/California/WR1317P/2009(H1N1)
NA	KY935482	H1N1	08/10/2009	Brazil	A/Rio Grande/LACENRS-1406/2009
NA	CY073547	H1N1	08/10/2009	Nicaragua	A/Managua/3083.01/2009(H1N1)
NA	CY055902	H1N1	08/10/2009	Australia	A/Australia/72/2009(H1N1)
NA	CY053786	H1N1	08/10/2009	Norway	A/Norway/2924/2009(H1N1)
NA	HM014326	H1N1	08/10/2009	China	A/Guangzhou/GIRD07/2009
NA	CY055496	H1N1	08/10/2009	USA	A/California/VRDL74/2009(H1N1)
NA	CY047231	H1N1	08/10/2009	Spain	A/Catalonia/S1227/2009(H1N1)
NA	KC781476	H1N1	08/11/2009	USA	A/Vermont/18/2009
NA	CY047225	H1N1	08/11/2009	Spain	A/Catalonia/S1221/2009(H1N1)
NA	CY055029	H1N1	08/11/2009	USA	A/California/VRDL68/2009(H1N1)
NA	CY123496	H1N1	08/11/2009	Singapore	A/Singapore/ON1747/2009
NA	CY123490	H1N1	08/11/2009	Singapore	A/Singapore/ON1746/2009
NA	CY123508	H1N1	08/11/2009	Singapore	A/Singapore/ON1760/2009
NA	CY115864	H1N1	08/11/2009	Netherlan	A/Netherlands/947b/2009
NA	CY072648	H1N1	08/11/2009	Nicaragua	A/Managua/2275.01/2009(H1N1)
NA	CY072592	H1N1	08/11/2009	Nicaragua	A/Managua/1356.03/2009(H1N1)
NA	KJ958962	H1N1	08/11/2009	India	A/Mumbai/925/2009
NA	HQ256643	H1N1	08/11/2009	Myanmar	A/Myanmar/M428/2009(H1N1)
NA	CY073563	H1N1	08/11/2009	Nicaragua	A/Managua/5119.06/2009(H1N1)
NA	KC781940	H1N1	08/11/2009	USA	A/Alaska/33/2009
NA	CY072704	H1N1	08/11/2009	Nicaragua	A/Managua/5036.01/2009(H1N1)
NA	CY072664	H1N1	08/11/2009	Nicaragua	A/Managua/2339.03/2009(H1N1)
NA	CY118221	H1N1	08/11/2009	Malaysia	A/Malaysia/2098038/2009
NA	CY069276	H1N1	08/11/2009	Nicaragua	A/Managua/5212.04/2009(H1N1)
NA	CY073539	H1N1	08/11/2009	Nicaragua	A/Managua/4012.01/2009(H1N1)
NA	CY119452	H1N1	08/11/2009	Malaysia	A/Malaysia/2097717/2009

NA	CY055261	H1N1	08/11/2009	Malaysia	A/Malaysia/9254/2009(H1N1)
NA	JQ431274	H1N1	08/11/2009	France	A/Reunion/2336-4-M1E/2009
NA	CY060520	H1N1	08/11/2009	Canada	A/Ontario/222656/2009(H1N1)
NA	JN631047	H1N1	08/11/2009	China	A/Shanghai/1300T/2009
NA	CY123502	H1N1	08/11/2009	Singapore	A/Singapore/ON1757/2009
NA	JX465465	H1N1	08/11/2009	South Ko	A/Gyeonggi/1580/2009
NA	CY123500	H1N1	08/11/2009	Singapore	A/Singapore/ON1754/2009
NA	KC781663	H1N1	08/11/2009	USA	A/Utah/30/2009
NA	KC782269	H1N1	08/11/2009	USA	A/Illinois/12/2009
NA	CY047222	H1N1	08/12/2009	Spain	A/Catalonia/S1215/2009(H1N1)
NA	HM624083	H1N1	08/12/2009	Australia	A/Perth/262/2009
NA	CY056254	H1N1	08/12/2009	Malaysia	A/Malaysia/9112/2009
NA	CY055910	H1N1	08/12/2009	Australia	A/Australia/73/2009(H1N1)
NA	CY055251	H1N1	08/12/2009	Malaysia	A/Malaysia/9117/2009(H1N1)
NA	CY055253	H1N1	08/12/2009	Malaysia	A/Malaysia/9118/2009(H1N1)
NA	CY055255	H1N1	08/12/2009	Malaysia	A/Malaysia/9131/2009(H1N1)
NA	CY072720	H1N1	08/12/2009	Nicaragua	A/Managua/3359.01/2009(H1N1)
NA	KY925984	H1N1	08/12/2009	Brazil	A/Alegrete/LACENRS-1453/2009
NA	CY062264	H1N1	08/12/2009	Malaysia	A/Malaysia/9538/2009
NA	CY067916	H1N1	08/12/2009	Portugal	A/Lisboa/88/2009(H1N1)
NA	KY925937	H1N1	08/12/2009	Brazil	A/Porto Alegre/LACENRS-1598/2009
NA	KJ958980	H1N1	08/12/2009	India	A/Mumbai/2381/2009
NA	CY072584	H1N1	08/12/2009	Nicaragua	A/Managua/4186.02/2009(H1N1)
NA	JN256917	H1N1	08/12/2009	Hong Kong	A/Hong Kong/H090-793-V1(0)/2009
NA	CY083234	H1N1	08/12/2009	Guam	A/Guam/WRAIR1510P/2009
NA	CY083393	H1N1	08/12/2009	USA	A/San Diego/WRAIR1663P/2009
NA	CY083385	H1N1	08/12/2009	USA	A/San Diego/WRAIR1662P/2009
NA	CY112085	H1N1	08/12/2009	Hong Kong	A/Hong Kong/H090-793-V10/2009
NA	CY120479	H1N1	08/12/2009	Hong Kong	A/Hong Kong/H090-793-V10/2009
NA	CY128133	H1N1	08/12/2009	Viet Nam	A/Viet Nam/13032001/2009
NA	CY128141	H1N1	08/12/2009	Viet Nam	A/Viet Nam/13032002/2009
NA	CY055267	H1N1	08/12/2009	Malaysia	A/Malaysia/9541/2009(H1N1)
NA	CY055257	H1N1	08/12/2009	Malaysia	A/Malaysia/9147/2009(H1N1)
NA	CY055259	H1N1	08/12/2009	Malaysia	A/Malaysia/9237/2009(H1N1)
NA	KC782265	H1N1	08/12/2009	USA	A/Texas/57/2009
NA	KC781857	H1N1	08/12/2009	USA	A/Oregon/31/2009
NA	CY120717	H1N1	08/12/2009	Brazil	A/Brazil/AVS03/2009
NA	KY925263	H1N1	08/13/2009	Brazil	A/Venancio Aires/LACENRS-1539/2009
NA	CY047233	H1N1	08/13/2009	Spain	A/Catalonia/S1228/2009(H1N1)
NA	CY062263	H1N1	08/13/2009	Malaysia	A/Malaysia/9407/2009
NA	KY926335	H1N1	08/13/2009	Brazil	A/Santo Angelo/LACENRS-1537/2009
NA	JQ431270	H1N1	08/13/2009	France	A/Reunion/1722-8-M1E/2009
NA	KY925701	H1N1	08/13/2009	Brazil	A/Vacaria/LACENRS-1720/2009
NA	JQ431269	H1N1	08/13/2009	France	A/Reunion/1722-7-M1E/2009
NA	CY073579	H1N1	08/13/2009	Nicaragua	A/Managua/921.02/2009(H1N1)
NA	CY123512	H1N1	08/13/2009	Singapore	A/Singapore/ON1767/2009
NA	CY056255	H1N1	08/13/2009	Malaysia	A/Malaysia/9368/2009
NA	CY060736	H1N1	08/13/2009	Canada	A/Ontario/35273/2009(H1N1)
NA	CY120725	H1N1	08/13/2009	Brazil	A/Brazil/AVS04/2009
NA	CY055265	H1N1	08/13/2009	Malaysia	A/Malaysia/9451/2009(H1N1)
NA	JX625245	H1N1	08/13/2009	Paraguay	A/Paraguay/98HCl/2009
NA	HE804128	H1N1	08/13/2009	Uruguay	A/Uruguay/76/2009
NA	CY047244	H1N1	08/13/2009	Spain	A/Catalonia/S1237/2009(H1N1)
NA	KF612138	H1N1	08/13/2009	Bolivia	A/Santa Cruz/87212/2009

NA	CY047241	H1N1	08/13/2009	Spain	A/Catalonia/S1236/2009(H1N1)
NA	CY047238	H1N1	08/13/2009	Spain	A/Catalonia/S1233/2009(H1N1)
NA	CY047236	H1N1	08/13/2009	Spain	A/Catalonia/S1230/2009(H1N1)
NA	HQ256625	H1N1	08/13/2009	Myanmar	A/Myanmar/M278/2009(H1N1)
NA	KT181528	H1N1	08/13/2009	Singapore	A/Singapore/DMS23/2009
NA	CY088675	H1N1	08/13/2009	India	A/Delhi/NIV3610/2009
NA	CY072640	H1N1	08/13/2009	Nicaragua	A/Managua/3642.04/2009(H1N1)
NA	CY072992	H1N1	08/13/2009	Nicaragua	A/Managua/527.04/2009(H1N1)
NA	CY055263	H1N1	08/13/2009	Malaysia	A/Malaysia/9343/2009(H1N1)
NA	CY055411	H1N1	08/13/2009	Italy	A/Rome/669/2009(H1N1)
NA	CY066148	H1N1	08/13/2009	Japan	A/Nagasaki/09N021/2009
NA	CY075481	H1N1	08/13/2009	Russia	A/Saint-Petersburg/RII5/2009(H1N1)
NA	CY067910	H1N1	08/14/2009	Portugal	A/Lisboa/86/2009(H1N1)
NA	KY925348	H1N1	08/14/2009	Brazil	A/Sao Gabriel/LACENRS-1626/2009
NA	KY925212	H1N1	08/14/2009	Brazil	A/Triunfo/LACENRS-1611/2009
NA	HQ239973	H1N1	08/14/2009	Canada	A/Canada-AB/RV2827/2009
NA	CY075804	H1N1	08/14/2009	Thailand	A/Thailand/CU-H393/2009(H1N1)
NA	KP317297	H1N1	08/14/2009	India	A/Kerala/002/2009
NA	CY047249	H1N1	08/14/2009	Spain	A/Catalonia/S1243/2009(H1N1)
NA	CY055413	H1N1	08/14/2009	Italy	A/Rome/670/2009(H1N1)
NA	CY122969	H1N1	08/14/2009	Singapore	A/Singapore/GP2982/2009
NA	CY120487	H1N1	08/14/2009	Hong Kong	A/Hong Kong/H090-793-V20/2009
NA	KP637832	H1N1	08/14/2009	Thailand	A/Thailand/IY07083/2009
NA	CY112093	H1N1	08/14/2009	Hong Kong	A/Hong Kong/H090-793-V20/2009
NA	CY122981	H1N1	08/14/2009	Singapore	A/Singapore/GP3009/2009
NA	CY123516	H1N1	08/14/2009	Singapore	A/Singapore/ON1782/2009
NA	CY123514	H1N1	08/14/2009	Singapore	A/Singapore/ON1774/2009
NA	CY051697	H1N1	08/14/2009	USA	A/New York/4777/2009(H1N1)
NA	JN256918	H1N1	08/14/2009	Hong Kong	A/Hong Kong/H090-793-V2(0)/2009
NA	KC782224	H1N1	08/14/2009	USA	A/California/34/2009
NA	CY055273	H1N1	08/14/2009	Malaysia	A/Malaysia/10226/2009(H1N1)
NA	KY926356	H1N1	08/14/2009	Brazil	A/Pelotas/LACENRS-1658/2009
NA	JQ431267	H1N1	08/14/2009	France	A/Reunion/1658-4-M1E/2009
NA	CY058246	H1N1	08/15/2009	Nicaragua	A/Managua/3275.01/2009(H1N1)
NA	CY058606	H1N1	08/15/2009	Nicaragua	A/Managua/5747.02/2009(H1N1)
NA	CY072712	H1N1	08/15/2009	Nicaragua	A/Managua/5307.01/2009(H1N1)
NA	CY052849	H1N1	08/15/2009	USA	A/Texas/44152535/2009(H1N1)
NA	CY052873	H1N1	08/15/2009	USA	A/Texas/44151841/2009(H1N1)
NA	HQ239979	H1N1	08/15/2009	Canada	A/Canada-NWT/RV2869/2009
NA	KY925364	H1N1	08/15/2009	Brazil	A/Santo Antonio da Patrulha/LACENRS-
NA	CY058238	H1N1	08/15/2009	Nicaragua	A/Managua/473.02/2009(H1N1)
NA	CY055798	H1N1	08/16/2009	Australia	A/Australia/54/2009(H1N1)
NA	CY072656	H1N1	08/16/2009	Nicaragua	A/Managua/4570.01/2009(H1N1)
NA	CY122989	H1N1	08/16/2009	Singapore	A/Singapore/GP3012/2009
NA	KT181529	H1N1	08/16/2009	Singapore	A/Singapore/DMS24/2009
NA	HM189338	H1N1	08/16/2009	Russia	A/Orenburg/CRIE-VNV/2009
NA	CY088689	H1N1	08/16/2009	India	A/Mum/NIV5442/2009
NA	CY122977	H1N1	08/16/2009	Singapore	A/Singapore/GP2987/2009
NA	GU057041	H1N1	08/16/2009	China	A/Jiangyin/01/2009(H1N1)
NA	GU057042	H1N1	08/16/2009	China	A/Jiangyin/02/2009(H1N1)
NA	GU057043	H1N1	08/16/2009	China	A/Jiangyin/15/2009(H1N1)
NA	GU057044	H1N1	08/16/2009	China	A/Jiangyin/16/2009(H1N1)
NA	GU057045	H1N1	08/16/2009	China	A/Jiangyin/17/2009(H1N1)
NA	GU057046	H1N1	08/16/2009	China	A/Jiangyin/34/2009(H1N1)

NA	GU057047	H1N1	08/16/2009	China	A/Jiangyin/47/2009(H1N1)
NA	GU057048	H1N1	08/16/2009	China	A/Jiangyin/48/2009(H1N1)
NA	GU057049	H1N1	08/16/2009	China	A/Jiangyin/68/2009(H1N1)
NA	GU057050	H1N1	08/16/2009	China	A/Jiangyin/69/2009(H1N1)
NA	CY072672	H1N1	08/16/2009	Nicaragua	A/Managua/1054.02/2009(H1N1)
NA	JQ431273	H1N1	08/17/2009	France	A/Reunion/2245-3-M1E/2009
NA	KY925722	H1N1	08/17/2009	Brazil	A/Passo Fundo/LACENRS-1972/2009
NA	CY058566	H1N1	08/17/2009	Nicaragua	A/Managua/4218.01/2009(H1N1)
NA	KF695099	H1N1	08/17/2009	Singapore	A/Singapore/464L/2009
NA	HQ256627	H1N1	08/17/2009	Myanmar	A/Myanmar/M279/2009(H1N1)
NA	CY072608	H1N1	08/17/2009	Nicaragua	A/Managua/2155.01/2009(H1N1)
NA	CY066138	H1N1	08/17/2009	Japan	A/Nagasaki/09N015/2009
NA	CY123005	H1N1	08/17/2009	Singapore	A/Singapore/GP3026/2009
NA	AB649909	H1N1	08/17/2009	Japan	A/Toyama/267/2009
NA	CY123518	H1N1	08/17/2009	Singapore	A/Singapore/ON1786/2009
NA	CY123132	H1N1	08/17/2009	Singapore	A/Singapore/GP3278/2009
NA	CY122997	H1N1	08/17/2009	Singapore	A/Singapore/GP3021/2009
NA	CY123021	H1N1	08/17/2009	Singapore	A/Singapore/GP3044/2009
NA	CY123520	H1N1	08/17/2009	Singapore	A/Singapore/ON1790/2009
NA	CY047246	H1N1	08/17/2009	Spain	A/Catalonia/S1242/2009(H1N1)
NA	CY047252	H1N1	08/17/2009	Spain	A/Catalonia/S1244/2009(H1N1)
NA	CY052357	H1N1	08/17/2009	United Ar	A/UAE/E01/2009(H1N1)
NA	HE804112	H1N1	08/17/2009	Uruguay	A/Uruguay/890/2009
NA	HM624084	H1N1	08/17/2009	Australia	A/Perth/268/2009
NA	KF695098	H1N1	08/17/2009	Singapore	A/Singapore/463B/2009
NA	CY060512	H1N1	08/17/2009	Canada	A/Ontario/10296/2009(H1N1)
NA	KY925401	H1N1	08/17/2009	Brazil	A/Santa Cruz do Sul/LACENRS-2013/20(
NA	KC781639	H1N1	08/17/2009	USA	A/Nevada/13/2009
NA	CY123037	H1N1	08/18/2009	Singapore	A/Singapore/GP3074/2009
NA	CY058574	H1N1	08/18/2009	Nicaragua	A/Managua/4757.02/2009(H1N1)
NA	KC781513	H1N1	08/18/2009	USA	A/Wisconsin/49/2009
NA	KC781464	H1N1	08/18/2009	USA	A/Texas/56/2009
NA	CY058582	H1N1	08/18/2009	Nicaragua	A/Managua/989.01/2009(H1N1)
NA	HQ256621	H1N1	08/18/2009	Myanmar	A/Myanmar/M277/2009(H1N1)
NA	CY123029	H1N1	08/18/2009	Singapore	A/Singapore/GP3069/2009
NA	CY051705	H1N1	08/18/2009	USA	A/New York/4780/2009(H1N1)
NA	CY123053	H1N1	08/18/2009	Singapore	A/Singapore/GP3093/2009
NA	JX625492	H1N1	08/18/2009	United Ki	A/England/93520038/2009
NA	CY058254	H1N1	08/18/2009	Nicaragua	A/Managua/3244.01/2009(H1N1)
NA	CY058262	H1N1	08/18/2009	Nicaragua	A/Managua/164.01/2009(H1N1)
NA	CY055021	H1N1	08/18/2009	USA	A/California/VRDL67/2009(H1N1)
NA	CY123013	H1N1	08/18/2009	Singapore	A/Singapore/GP3040/2009
NA	CY047255	H1N1	08/18/2009	Spain	A/Catalonia/S1248/2009(H1N1)
NA	CY047258	H1N1	08/18/2009	Spain	A/Catalonia/S1249/2009(H1N1)
NA	KF695100	H1N1	08/18/2009	Singapore	A/Singapore/468H/2009
NA	CY063756	H1N1	08/18/2009	Singapore	A/Singapore/GP3084/2009(H1N1)
NA	CY063576	H1N1	08/18/2009	USA	A/Boston/154/2009(H1N1)
NA	CY058614	H1N1	08/18/2009	Nicaragua	A/Managua/2323.02/2009(H1N1)
NA	CY058598	H1N1	08/18/2009	Nicaragua	A/Managua/3435.01/2009(H1N1)
NA	CY058558	H1N1	08/18/2009	Nicaragua	A/Managua/2093.01/2009(H1N1)
NA	JQ431266	H1N1	08/18/2009	France	A/Reunion/1632-5-M1E/2009
NA	JQ431260	H1N1	08/19/2009	France	A/Reunion/0148-4-M1E/2009
NA	CY067826	H1N1	08/19/2009	Portugal	A/Lisboa/58/2009(H1N1)
NA	CY072848	H1N1	08/19/2009	Nicaragua	A/Managua/4702.04/2009(H1N1)

NA	CY123550	H1N1	08/19/2009	Singapore	A/Singapore/ON1813/2009
NA	CY123045	H1N1	08/19/2009	Singapore	A/Singapore/GP3081/2009
NA	KR870126	H1N1	08/19/2009	India	A/Bangalore/2960-03/2009
NA	KT181530	H1N1	08/19/2009	Singapore	A/Singapore/DMS25/2009
NA	CY123526	H1N1	08/19/2009	Singapore	A/Singapore/ON1802/2009
NA	CY123534	H1N1	08/19/2009	Singapore	A/Singapore/ON1808/2009
NA	CY123542	H1N1	08/19/2009	Singapore	A/Singapore/ON1811/2009
NA	GU576518	H1N1	08/19/2009	Italy	A/Ancona/69/2009(H1N1)
NA	GU576516	H1N1	08/19/2009	Italy	A/Ancona/68/2009(H1N1)
NA	CY123061	H1N1	08/19/2009	Singapore	A/Singapore/GP3104/2009
NA	CY047261	H1N1	08/19/2009	Spain	A/Catalonia/S1250/2009(H1N1)
NA	CY047264	H1N1	08/19/2009	Spain	A/Catalonia/S1254/2009(H1N1)
NA	CY047267	H1N1	08/19/2009	Spain	A/Catalonia/S1260/2009(H1N1)
NA	CY075810	H1N1	08/19/2009	Thailand	A/Thailand/CU-H427/2009(H1N1)
NA	CY061534	H1N1	08/19/2009	Argentina	A/Argentina/HNRG1227/2009
NA	CY047294	H1N1	08/19/2009	Spain	A/Catalonia/S1276/2009(H1N1)
NA	CY067820	H1N1	08/19/2009	Portugal	A/Lisboa/51/2009(H1N1)
NA	KY924959	H1N1	08/19/2009	Brazil	A/Tenente Portela/LACENRS-2073/2009
NA	CY067823	H1N1	08/19/2009	Portugal	A/Lisboa/57/2009(H1N1)
NA	JQ431261	H1N1	08/19/2009	France	A/Reunion/0148-6-M1E/2009
NA	JQ431276	H1N1	08/20/2009	France	A/Reunion/2923-3-M1E/2009
NA	KF695101	H1N1	08/20/2009	Singapore	A/Singapore/480Y/2009
NA	CY128149	H1N1	08/20/2009	Viet Nam	A/Viet Nam/13032008/2009
NA	CY128173	H1N1	08/20/2009	Viet Nam	A/Viet Nam/13032015/2009
NA	CY128165	H1N1	08/20/2009	Viet Nam	A/Viet Nam/13032011/2009
NA	CY055281	H1N1	08/20/2009	Malaysia	A/Malaysia/12617/2009(H1N1)
NA	CY072768	H1N1	08/20/2009	Nicaragua	A/Managua/287.01/2009(H1N1)
NA	HQ256645	H1N1	08/20/2009	Myanmar	A/Myanmar/M470/2009(H1N1)
NA	CY051721	H1N1	08/20/2009	USA	A/New York/4789/2009(H1N1)
NA	JX625254	H1N1	08/20/2009	Paraguay	A/Paraguay/1CMI/2009
NA	JX625246	H1N1	08/20/2009	Paraguay	A/Paraguay/125HCl/2009
NA	CY123077	H1N1	08/20/2009	Singapore	A/Singapore/GP3178/2009
NA	CY128181	H1N1	08/20/2009	Viet Nam	A/Viet Nam/13032016/2009
NA	CY064915	H1N1	08/20/2009	Italy	A/Rome/700/2009(H1N1)
NA	CY064914	H1N1	08/20/2009	Italy	A/Rome/699/2009(H1N1)
NA	KP317298	H1N1	08/20/2009	India	A/Bihar/003/2009
NA	CY075816	H1N1	08/20/2009	Thailand	A/Thailand/CU-H429/2009(H1N1)
NA	CY055918	H1N1	08/20/2009	Australia	A/Australia/79/2009(H1N1)
NA	CY072872	H1N1	08/20/2009	Nicaragua	A/Managua/2999.03/2009(H1N1)
NA	KY924973	H1N1	08/20/2009	Brazil	A/Rosario do Sul/LACENRS-2110/2009
NA	KY925994	H1N1	08/20/2009	Brazil	A/Porto Alegre/LACENRS-2121/2009
NA	CY128157	H1N1	08/20/2009	Viet Nam	A/Viet Nam/13032009/2009
NA	GU576520	H1N1	08/21/2009	Italy	A/Ancona/75/2009(H1N1)
NA	CY060528	H1N1	08/21/2009	Canada	A/Ontario/235657/2009(H1N1)
NA	JQ431256	H1N1	08/21/2009	France	A/Reunion/2433-3-M1E/2009
NA	JQ431265	H1N1	08/21/2009	France	A/Reunion/1572-3-M1E/2009
NA	CY128189	H1N1	08/21/2009	Viet Nam	A/Viet Nam/13032019/2009
NA	CY051729	H1N1	08/21/2009	USA	A/New York/4790/2009(H1N1)
NA	CY051713	H1N1	08/21/2009	USA	A/New York/4787/2009(H1N1)
NA	JX625250	H1N1	08/21/2009	Paraguay	A/Paraguay/145HCl/2009
NA	KM985427	H1N1	08/21/2009	India	A/Trivandrum/MCVR261/2009
NA	CY051745	H1N1	08/21/2009	USA	A/New York/4810/2009(H1N1)
NA	CY123069	H1N1	08/21/2009	Singapore	A/Singapore/GP3175/2009
NA	CY052164	H1N1	08/21/2009	USA	A/New York/4788/2009(H1N1)

NA	HE804111	H1N1	08/21/2009	Uruguay	A/Uruguay/905/2009
NA	CY117614	H1N1	08/21/2009	Mexico	A/Mexico/UASLP-018/2009
NA	CY047285	H1N1	08/21/2009	Spain	A/Catalonia/S1271/2009(H1N1)
NA	KF695102	H1N1	08/21/2009	Singapore	A/Singapore/482H/2009
NA	KF695103	H1N1	08/21/2009	Singapore	A/Singapore/483A/2009
NA	CY123085	H1N1	08/21/2009	Singapore	A/Singapore/GP3179/2009
NA	JQ431268	H1N1	08/22/2009	France	A/Reunion/1709-4-M2E/2009
NA	KC781530	H1N1	08/22/2009	USA	A/Hawaii/26/2009
NA	KC781946	H1N1	08/22/2009	USA	A/California/36/2009
NA	CY072752	H1N1	08/22/2009	Nicaragua	A/Managua/5227.04/2009(H1N1)
NA	CY072864	H1N1	08/22/2009	Nicaragua	A/Managua/4147.01/2009(H1N1)
NA	CY072800	H1N1	08/22/2009	Nicaragua	A/Managua/432.01/2009(H1N1)
NA	JX625248	H1N1	08/22/2009	Paraguay	A/Paraguay/138HCl/2009
NA	KT181531	H1N1	08/22/2009	Singapore	A/Singapore/DMS26/2009
NA	CY056366	H1N1	08/22/2009	Spain	A/Madrid/RC1/2009(H1N1)
NA	JX556119	H1N1	08/22/2009	Cuba	A/Habana/2565/2009
NA	CY063790	H1N1	08/22/2009	Singapore	A/Singapore/ON1868/2009
NA	JX625247	H1N1	08/22/2009	Paraguay	A/Paraguay/136HCl/2009
NA	CY123116	H1N1	08/23/2009	Singapore	A/Singapore/GP3205/2009
NA	CY123108	H1N1	08/23/2009	Singapore	A/Singapore/GP3191/2009
NA	KY925980	H1N1	08/23/2009	Brazil	A/Ijui/LACENRS-2326/2009
NA	CY123124	H1N1	08/23/2009	Singapore	A/Singapore/GP3212/2009
NA	JX556118	H1N1	08/23/2009	Cuba	A/Habana/2558/2009
NA	JX625249	H1N1	08/23/2009	Paraguay	A/Paraguay/141HCl/2009
NA	JQ431272	H1N1	08/24/2009	France	A/Reunion/2133-6-M1E/2009
NA	CY060536	H1N1	08/24/2009	Canada	A/Ontario/237882/2009(H1N1)
NA	JQ431255	H1N1	08/24/2009	France	A/Reunion/2378-1-M1E/2009
NA	CY051737	H1N1	08/24/2009	USA	A/New York/4791/2009(H1N1)
NA	CY072816	H1N1	08/24/2009	Nicaragua	A/Managua/3421.02/2009(H1N1)
NA	CY123101	H1N1	08/24/2009	Singapore	A/Singapore/GP3187/2009
NA	KT181568	H1N1	08/24/2009	Singapore	A/Singapore/DMS623/2009
NA	CY119540	H1N1	08/24/2009	Malaysia	A/Malaysia/2126082/2009
NA	CY119460	H1N1	08/24/2009	Malaysia	A/Malaysia/2126061/2009
NA	CY123568	H1N1	08/24/2009	Singapore	A/Singapore/ON1858/2009
NA	CY123558	H1N1	08/24/2009	Singapore	A/Singapore/ON1852/2009
NA	CY123562	H1N1	08/24/2009	Singapore	A/Singapore/ON1855/2009
NA	CY047299	H1N1	08/24/2009	Spain	A/Catalonia/S1278/2009(H1N1)
NA	CY047270	H1N1	08/24/2009	Spain	A/Catalonia/S1265/2009(H1N1)
NA	JX556120	H1N1	08/24/2009	Cuba	A/Habana/2566/2009
NA	CY061535	H1N1	08/24/2009	Argentina	A/Argentina/HNRG1228/2009
NA	CY047273	H1N1	08/24/2009	Spain	A/Catalonia/S1267/2009(H1N1)
NA	CY047276	H1N1	08/24/2009	Spain	A/Catalonia/S1268/2009(H1N1)
NA	CY047279	H1N1	08/24/2009	Spain	A/Catalonia/S1269/2009(H1N1)
NA	CY047282	H1N1	08/24/2009	Spain	A/Catalonia/S1270/2009(H1N1)
NA	CY047297	H1N1	08/24/2009	Spain	A/Catalonia/S1277/2009(H1N1)
NA	CY060504	H1N1	08/24/2009	Canada	A/Ontario/10016/2009(H1N1)
NA	JQ431271	H1N1	08/24/2009	France	A/Reunion/2133-5-M1E/2009
NA	KC781596	H1N1	08/25/2009	USA	A/Idaho/11/2009
NA	KF695104	H1N1	08/25/2009	Singapore	A/Singapore/491H/2009
NA	KC781347	H1N1	08/25/2009	USA	A/Idaho/10/2009
NA	CY065794	H1N1	08/25/2009	Netherlar	A/Netherlands/1039/2009(H1N1)
NA	KT181566	H1N1	08/25/2009	Singapore	A/Singapore/DMS618/2009
NA	KT181570	H1N1	08/25/2009	Singapore	A/Singapore/DMS638/2009
NA	KT181565	H1N1	08/25/2009	Singapore	A/Singapore/DMS617/2009

NA	CY123136	H1N1	08/25/2009	Singapore	A/Singapore/GP3292/2009
NA	CY047302	H1N1	08/25/2009	Spain	A/Catalonia/S1279/2009(H1N1)
NA	CY047305	H1N1	08/25/2009	Spain	A/Catalonia/S1285/2009(H1N1)
NA	CY047308	H1N1	08/25/2009	Spain	A/Catalonia/S1286/2009(H1N1)
NA	CY047291	H1N1	08/25/2009	Spain	A/Catalonia/S1273/2009(H1N1)
NA	HQ239970	H1N1	08/25/2009	Canada	A/Canada-AB/RV2823/2009
NA	JQ283443	H1N1	08/25/2009	Brazil	A/Rio Grande do Sul/4144/2009
NA	CY047288	H1N1	08/25/2009	Spain	A/Catalonia/S1272/2009(H1N1)
NA	CY056397	H1N1	08/25/2009	USA	A/New York/4925/2009(H1N1)
NA	KC780676	H1N1	08/25/2009	USA	A/West Virginia/13/2009
NA	CY058590	H1N1	08/25/2009	Nicaragua	A/Managua/65.02/2009(H1N1)
NA	KC781522	H1N1	08/25/2009	USA	A/Utah/51/2009
NA	CY123572	H1N1	08/26/2009	Singapore	A/Singapore/ON1876/2009
NA	CY074976	H1N1	08/26/2009	Thailand	A/Thailand/CU-C161/2009(H1N1)
NA	JQ431252	H1N1	08/26/2009	France	A/Reunion/2224-3-M3E/2009
NA	JQ431262	H1N1	08/26/2009	France	A/Reunion/0159-3-M1E/2009
NA	HQ256647	H1N1	08/26/2009	Myanmar	A/Myanmar/M494/2009(H1N1)
NA	KJ958964	H1N1	08/26/2009	India	A/Mumbai/2244/2009
NA	KF695105	H1N1	08/26/2009	Singapore	A/Singapore/495W/2009
NA	CY073019	H1N1	08/26/2009	Hong Kong	A/Hong Kong/WRAIR0113N/2009(H1N1)
NA	JX625251	H1N1	08/26/2009	Paraguay	A/Paraguay/153HC1/2009
NA	KT181573	H1N1	08/26/2009	Singapore	A/Singapore/DMS653/2009
NA	CY048998	H1N1	08/26/2009	Spain	A/Catalonia/S1289/2009(H1N1)
NA	KF695106	H1N1	08/26/2009	Singapore	A/Singapore/496T/2009
NA	CY055926	H1N1	08/26/2009	Australia	A/Australia/80/2009(H1N1)
NA	KJ958963	H1N1	08/26/2009	India	A/Mumbai/2236/2009
NA	CY123594	H1N1	08/27/2009	Singapore	A/Singapore/ON1896/2009
NA	CY055045	H1N1	08/27/2009	USA	A/California/VRDL70/2009(H1N1)
NA	KC781965	H1N1	08/27/2009	USA	A/Mississippi/05/2009
NA	KC781648	H1N1	08/27/2009	USA	A/South Carolina/21/2009
NA	KC781580	H1N1	08/27/2009	USA	A/Kentucky/17/2009
NA	AB538391	H1N1	08/27/2009	Japan	A/Nagano/RC1/2009(H1N1)
NA	CY072904	H1N1	08/27/2009	Nicaragua	A/Managua/3434.02/2009(H1N1)
NA	JN187184	H1N1	08/27/2009	Taiwan	A/Taiwan/6078/2009
NA	CY123586	H1N1	08/27/2009	Singapore	A/Singapore/ON1892/2009
NA	CY123093	H1N1	08/27/2009	Singapore	A/Singapore/GP3183/2009
NA	KT181577	H1N1	08/27/2009	Singapore	A/Singapore/DMS676/2009
NA	KT181575	H1N1	08/27/2009	Singapore	A/Singapore/DMS665/2009
NA	CY119468	H1N1	08/27/2009	Malaysia	A/Malaysia/2135565/2009
NA	CY123578	H1N1	08/27/2009	Singapore	A/Singapore/ON1888/2009
NA	GU936490	H1N1	08/27/2009	Italy	A/Roma/ISS1897/2009(H1N1)
NA	GU936491	H1N1	08/27/2009	Italy	A/Roma/ISS1941/2009(H1N1)
NA	CY052358	H1N1	08/27/2009	United A	A/UAE/E02/2009(H1N1)
NA	KF695107	H1N1	08/27/2009	Singapore	A/Singapore/501L/2009
NA	KF695108	H1N1	08/27/2009	Singapore	A/Singapore/502J/2009
NA	KC781630	H1N1	08/27/2009	USA	A/Virginia/19/2009
NA	CY073027	H1N1	08/27/2009	Hong Kong	A/Hong Kong/WRAIR0114T/2009(H1N1)
NA	CY071396	H1N1	08/27/2009	Hong Kong	A/Hong Kong/WR0114N/2009(H1N1)
NA	CY052769	H1N1	08/28/2009	USA	A/Texas/44282651/2009(H1N1)
NA	KF695109	H1N1	08/28/2009	Singapore	A/Singapore/506T/2009
NA	CY055053	H1N1	08/28/2009	USA	A/California/VRDL71/2009(H1N1)
NA	CY072944	H1N1	08/28/2009	Nicaragua	A/Managua/1244.01/2009(H1N1)
NA	CY064916	H1N1	08/28/2009	Italy	A/Rome/704/2009(H1N1)
NA	HQ712182	H1N1	08/28/2009	Australia	A/Brisbane/2015/2009

NA	CY049004	H1N1	08/28/2009	Spain	A/Catalonia/S1304/2009(H1N1)
NA	CY049001	H1N1	08/28/2009	Spain	A/Catalonia/S1300/2009(H1N1)
NA	KC780561	H1N1	08/28/2009	USA	A/Minnesota/15/2009
NA	KC782313	H1N1	08/28/2009	USA	A/Minnesota/16/2009
NA	KC781500	H1N1	08/28/2009	USA	A/South Carolina/19/2009
NA	KJ958966	H1N1	08/28/2009	India	A/Mumbai/2877/2009
NA	CY073667	H1N1	08/28/2009	Nicaragua	A/Managua/4209.04/2009(H1N1)
NA	CY066150	H1N1	08/28/2009	Japan	A/Nagasaki/09N023/2009
NA	HM241712	H1N1	08/28/2009	India	A/Ytml/NIV9438/2009
NA	JX625252	H1N1	08/28/2009	Paraguay	A/Paraguay/165HCl/2009
NA	CY123602	H1N1	08/28/2009	Singapore	A/Singapore/ON1900/2009
NA	CY123618	H1N1	08/28/2009	Singapore	A/Singapore/ON1911/2009
NA	KT181580	H1N1	08/28/2009	Singapore	A/Singapore/DMS698/2009
NA	KT181532	H1N1	08/28/2009	Singapore	A/Singapore/DMS28/2009
NA	CY123610	H1N1	08/28/2009	Singapore	A/Singapore/ON1908/2009
NA	CY064917	H1N1	08/28/2009	Italy	A/Rome/703/2009(H1N1)
NA	CY049007	H1N1	08/28/2009	Spain	A/Catalonia/S1305/2009(H1N1)
NA	HQ239981	H1N1	08/29/2009	Canada	A/Canada-ON/RV2888/2009
NA	CY119476	H1N1	08/29/2009	Malaysia	A/Malaysia/2139971/2009
NA	KP637848	H1N1	08/29/2009	Thailand	A/Thailand/IY07086/2009
NA	KC781642	H1N1	08/29/2009	USA	A/California/35/2009
NA	CY088703	H1N1	08/29/2009	India	A/Pune/NIV9355/2009
NA	CY128197	H1N1	08/29/2009	Viet Nam	A/Viet Nam/13032030/2009
NA	KC781759	H1N1	08/30/2009	USA	A/South Carolina/32/2009
NA	HM241705	H1N1	08/30/2009	India	A/Jalna/NIV9436/2009
NA	KC781524	H1N1	08/30/2009	USA	A/South Carolina/20/2009
NA	CY052625	H1N1	08/30/2009	USA	A/Texas/44302167/2009(H1N1)
NA	CY052617	H1N1	08/30/2009	USA	A/Texas/44302533/2009(H1N1)
NA	CY072792	H1N1	08/30/2009	Nicaragua	A/Managua/1984.02/2009(H1N1)
NA	CY052228	H1N1	08/30/2009	USA	A/Texas/44302551/2009(H1N1)
NA	CY052220	H1N1	08/30/2009	USA	A/Texas/44301765/2009(H1N1)
NA	CY123646	H1N1	08/31/2009	Singapore	A/Singapore/ON1927/2009
NA	CY123634	H1N1	08/31/2009	Singapore	A/Singapore/ON1921/2009
NA	CY123642	H1N1	08/31/2009	Singapore	A/Singapore/ON1924/2009
NA	AB649896	H1N1	08/31/2009	Japan	A/Toyama/287/2009
NA	KT181583	H1N1	08/31/2009	Singapore	A/Singapore/DMS722/2009
NA	KT181533	H1N1	08/31/2009	Singapore	A/Singapore/DMS29/2009
NA	CY052673	H1N1	08/31/2009	USA	A/Texas/44313703/2009(H1N1)
NA	CY052340	H1N1	08/31/2009	USA	A/Texas/44312415/2009(H1N1)
NA	GU576523	H1N1	08/31/2009	Italy	A/Ancona/86/2009(H1N1)
NA	CY123626	H1N1	08/31/2009	Singapore	A/Singapore/ON1914/2009
NA	HQ247640	H1N1	08/31/2009	Finland	A/Finland/607/2009
NA	CY066140	H1N1	08/31/2009	Japan	A/Nagasaki/09N016/2009
NA	KJ958965	H1N1	08/31/2009	India	A/Mumbai/2867/2009
NA	CY073675	H1N1	08/31/2009	Nicaragua	A/Managua/5248.03/2009(H1N1)
NA	CY072976	H1N1	08/31/2009	Nicaragua	A/Managua/3507.01/2009(H1N1)
NA	KY925502	H1N1	08/31/2009	Brazil	A/Palmitinho/LACENRS-2595/2009
NA	CY063760	H1N1	08/31/2009	Singapore	A/Singapore/GP3441/2009(H1N1)
NA	CY049010	H1N1	08/31/2009	Spain	A/Catalonia/S1307/2009(H1N1)
NA	CY072928	H1N1	08/31/2009	Nicaragua	A/Managua/4063.01/2009(H1N1)
NA	JN381371	H1N1	09/2009	Taiwan	A/Taiwan/99006/2009
NA	JN381370	H1N1	09/2009	Taiwan	A/Taiwan/98928/2009
NA	JN381365	H1N1	09/2009	Taiwan	A/Taiwan/98584/2009
NA	JN381372	H1N1	09/2009	Taiwan	A/Taiwan/99011/2009

NA	JN381367	H1N1	09/2009	Taiwan	A/Taiwan/98706/2009
NA	JN381368	H1N1	09/2009	Taiwan	A/Taiwan/98812/2009
NA	JN381369	H1N1	09/2009	Taiwan	A/Taiwan/98911/2009
NA	JN381366	H1N1	09/2009	Taiwan	A/Taiwan/98677/2009
NA	JN381364	H1N1	09/2009	Taiwan	A/Taiwan/98229/2009
NA	JN006472	H1N1	09/2009	China	A/Hangzhou/4/2009
NA	CY189538	H1N1	09/2009	Norway	A/Norway/3568/2009
NA	HM189491	H1N1	09/2009	South Ko	A/Korea/CJ04/2009(H1N1)
NA	HM189493	H1N1	09/2009	South Ko	A/Korea/CJ15/2009(H1N1)
NA	HM189490	H1N1	09/2009	South Ko	A/Korea/CJ01/2009(H1N1)
NA	HM189495	H1N1	09/2009	South Ko	A/Korea/CJ24/2009(H1N1)
NA	HM189494	H1N1	09/2009	South Ko	A/Korea/CJ19/2009(H1N1)
NA	HM189492	H1N1	09/2009	South Ko	A/Korea/CJ09/2009(H1N1)
NA	GU983255	H1N1	09/2009	India	A/Eastern India/2493/2009
NA	GU983256	H1N1	09/2009	India	A/Eastern India/2498/2009
NA	GU983257	H1N1	09/2009	India	A/Eastern India/2499/2009
NA	GU983254	H1N1	09/2009	India	A/Eastern India/1909/2009
NA	GU983258	H1N1	09/2009	India	A/Eastern India/2503/2009
NA	KM035451	H1N1	09/2009	Brazil	A/Rio Grande do Sul/5014/2009
NA	KM035450	H1N1	09/2009	Brazil	A/Rio Grande do Sul/4523/2009
NA	CY049016	H1N1	09/01/2009	Spain	A/Catalonia/S1333/2009(H1N1)
NA	CY119340	H1N1	09/01/2009	Malaysia	A/Malaysia/2142299/2009(H1N1)
NA	KC782304	H1N1	09/01/2009	USA	A/Wisconsin/53/2009
NA	KC781446	H1N1	09/01/2009	USA	A/New York/99/2009
NA	KC782037	H1N1	09/01/2009	USA	A/Texas/58/2009
NA	CY056244	H1N1	09/01/2009	USA	A/California/VRDL72/2009(H1N1)
NA	CY055488	H1N1	09/01/2009	USA	A/California/VRDL73/2009(H1N1)
NA	CY128461	H1N1	09/01/2009	Viet Nam	A/Viet Nam/001-1889/2009
NA	CY128066	H1N1	09/01/2009	Viet Nam	A/Viet Nam/001-1890/2009
NA	CY128205	H1N1	09/01/2009	Viet Nam	A/Viet Nam/13032035/2009
NA	CY123659	H1N1	09/01/2009	Singapore	A/Singapore/ON1934/2009
NA	CY123663	H1N1	09/01/2009	Singapore	A/Singapore/ON1937/2009
NA	CY052681	H1N1	09/01/2009	USA	A/Texas/45010998/2009(H1N1)
NA	CY065125	H1N1	09/01/2009	Netherlar	A/Netherlands/1064b/2009(H1N1)
NA	JX625242	H1N1	09/01/2009	Paraguay	A/Paraguay/20HBO/2009
NA	HQ247641	H1N1	09/01/2009	Finland	A/Finland/608/2009
NA	KP019720	H1N1	09/01/2009	China	A/FuZhou/SWL11202/2009
NA	CY119332	H1N1	09/01/2009	Malaysia	A/Malaysia/2142295/2009(H1N1)
NA	CY052021	H1N1	09/01/2009	Norway	A/Norway/3206-3/2009(H1N1)
NA	CY063762	H1N1	09/01/2009	Singapore	A/Singapore/GP3491/2009(H1N1)
NA	CY073683	H1N1	09/01/2009	Nicaragua	A/Managua/4736.02/2009(H1N1)
NA	CY071412	H1N1	09/01/2009	Taiwan	A/Taipei/WR0194N/2009(H1N1)
NA	CY064919	H1N1	09/01/2009	Italy	A/Rome/706/2009(H1N1)
NA	CY064918	H1N1	09/01/2009	Italy	A/Rome/705/2009(H1N1)
NA	CY119348	H1N1	09/01/2009	Malaysia	A/Malaysia/2143696/2009(H1N1)
NA	CY052857	H1N1	09/02/2009	USA	A/Texas/45023717/2009(H1N1)
NA	CY052359	H1N1	09/02/2009	United A	A/UAE/E04/2009(H1N1)
NA	CY123669	H1N1	09/02/2009	Singapore	A/Singapore/ON1943/2009
NA	KC781306	H1N1	09/02/2009	USA	A/Kentucky/19/2009
NA	CY050021	H1N1	09/02/2009	Mexico	A/Mexico City/WR1306N/2009(H1N1)
NA	KT181584	H1N1	09/02/2009	Singapore	A/Singapore/DMS737/2009
NA	KT181587	H1N1	09/02/2009	Singapore	A/Singapore/DMS752/2009
NA	CY055934	H1N1	09/02/2009	Australia	A/Australia/81/2009(H1N1)
NA	CY055942	H1N1	09/02/2009	Australia	A/Australia/82/2009(H1N1)

NA	KC781973	H1N1	09/02/2009	USA	A/South Carolina/35/2009
NA	JX625253	H1N1	09/02/2009	Paraguay	A/Paraguay/171HCl/2009
NA	CY074992	H1N1	09/02/2009	Thailand	A/Thailand/CU-H567/2009
NA	CY052777	H1N1	09/02/2009	USA	A/Texas/45021632/2009(H1N1)
NA	HQ247650	H1N1	09/02/2009	Finland	A/Finland/617/2009
NA	CY051753	H1N1	09/02/2009	USA	A/New York/4820/2009(H1N1)
NA	KC781802	H1N1	09/02/2009	USA	A/South Carolina/37/2009
NA	KC781757	H1N1	09/02/2009	USA	A/South Carolina/36/2009
NA	CY123150	H1N1	09/02/2009	Singapore	A/Singapore/GP3515/2009
NA	KC781624	H1N1	09/02/2009	USA	A/Idaho/09/2009
NA	GU271968	H1N1	09/02/2009	Thailand	A/Thailand/CU-H565/2009(H1N1)
NA	CY050132	H1N1	09/02/2009	China	A/Qingdao/1004/2009(H1N1)
NA	CY050141	H1N1	09/02/2009	China	A/Qingdao/1008/2009(H1N1)
NA	KY925739	H1N1	09/02/2009	Brazil	A/Osorio/LACENRS-2638/2009
NA	CY069100	H1N1	09/02/2009	Guam	A/Guam/NHRC0027/2009(H1N1)
NA	JQ431279	H1N1	09/02/2009	France	A/Reunion/2154-4-M4E/2009
NA	JQ431275	H1N1	09/02/2009	France	A/Reunion/2692-2-M2E/2009
NA	CY072744	H1N1	09/02/2009	Nicaragua	A/Managua/2958.01/2009(H1N1)
NA	JQ431254	H1N1	09/02/2009	France	A/Reunion/2956-2-M1E/2009
NA	CY123146	H1N1	09/02/2009	Singapore	A/Singapore/GP3495/2009
NA	KC782084	H1N1	09/02/2009	USA	A/Kentucky/18/2009
NA	CY052881	H1N1	09/02/2009	USA	A/Texas/45024243/2009(H1N1)
NA	CY123138	H1N1	09/02/2009	Singapore	A/Singapore/GP3485/2009
NA	CY123148	H1N1	09/03/2009	Singapore	A/Singapore/GP3502/2009
NA	HQ239975	H1N1	09/03/2009	Canada	A/Canada-NU/RV2851/2009
NA	CY049013	H1N1	09/03/2009	Spain	A/Catalonia/S1331/2009(H1N1)
NA	KJ958949	H1N1	09/03/2009	India	A/Mumbai/3026/2009
NA	KJ958935	H1N1	09/03/2009	India	A/Mumbai/3011/2009
NA	HM006721	H1N1	09/03/2009	China	A/Xian/001/2009(H1N1)
NA	CY051769	H1N1	09/03/2009	USA	A/New York/4824/2009(H1N1)
NA	CY051777	H1N1	09/03/2009	USA	A/New York/4841/2009(H1N1)
NA	CY051761	H1N1	09/03/2009	USA	A/New York/4823/2009(H1N1)
NA	CY090083	H1N1	09/03/2009	Nicaragua	A/Managua/4032.03/2009
NA	CY052609	H1N1	09/03/2009	USA	A/Texas/45034157/2009(H1N1)
NA	CY052905	H1N1	09/03/2009	USA	A/Texas/45032708/2009(H1N1)
NA	CY123677	H1N1	09/03/2009	Singapore	A/Singapore/ON1955/2009
NA	CY087194	H1N1	09/03/2009	USA	A/Texas/45034240/2009
NA	KT181534	H1N1	09/03/2009	Singapore	A/Singapore/DMS30/2009
NA	CY050005	H1N1	09/03/2009	Mexico	A/Mexico City/WR1297N/2009(H1N1)
NA	CY088710	H1N1	09/03/2009	India	A/Mum/NIV9945/2009
NA	CY123685	H1N1	09/03/2009	Singapore	A/Singapore/ON1964/2009
NA	JQ431277	H1N1	09/03/2009	France	A/Reunion/2969-5-M1E/2009
NA	CY128213	H1N1	09/03/2009	Viet Nam	A/Viet Nam/13032036/2009
NA	CY052593	H1N1	09/03/2009	USA	A/Texas/45033774/2009(H1N1)
NA	CY052601	H1N1	09/03/2009	USA	A/Texas/45033567/2009(H1N1)
NA	KC781948	H1N1	09/03/2009	USA	A/Maryland/28/2009
NA	KC781654	H1N1	09/03/2009	USA	A/New Hampshire/18/2009
NA	KC781470	H1N1	09/03/2009	USA	A/New Mexico/05/2009
NA	CY075000	H1N1	09/03/2009	Thailand	A/Thailand/CU-H572/2009
NA	CY052937	H1N1	09/03/2009	USA	A/Texas/45032753/2009(H1N1)
NA	HQ256649	H1N1	09/04/2009	Myanmar	A/Myanmar/M533/2009(H1N1)
NA	CY070993	H1N1	09/04/2009	Guam	A/Guam/NHRC0029/2009(H1N1)
NA	CY123689	H1N1	09/04/2009	Singapore	A/Singapore/ON1972/2009
NA	KT181592	H1N1	09/04/2009	Singapore	A/Singapore/DMS804/2009

NA	CY052865	H1N1	09/04/2009	USA	A/Texas/45042604/2009(H1N1)
NA	CY066076	H1N1	09/04/2009	Japan	A/Kyoto/09K017/2009
NA	JQ431253	H1N1	09/04/2009	France	A/Reunion/2956-1-M2E/2009
NA	JQ431263	H1N1	09/04/2009	France	A/Reunion/0215-4-M1E/2009
NA	CY128221	H1N1	09/04/2009	Viet Nam	A/Viet Nam/13032037/2009
NA	CY052785	H1N1	09/04/2009	USA	A/Texas/45043852/2009(H1N1)
NA	KC781589	H1N1	09/04/2009	USA	A/Alaska/35/2009
NA	KC781595	H1N1	09/04/2009	USA	A/Missouri/08/2009
NA	CY064920	H1N1	09/04/2009	Italy	A/Rome/701/2009(H1N1)
NA	CY063794	H1N1	09/04/2009	Singapore	A/Singapore/ON2018/2009
NA	CY064921	H1N1	09/04/2009	Italy	A/Rome/702/2009(H1N1)
NA	KJ958936	H1N1	09/04/2009	India	A/Mumbai/3043/2009
NA	CY068988	H1N1	09/04/2009	Guam	A/Guam/NHRC0013/2009(H1N1)
NA	CY069012	H1N1	09/04/2009	Guam	A/Guam/NHRC0016/2009(H1N1)
NA	CY050099	H1N1	09/04/2009	China	A/Qingdao/1020/2009(H1N1)
NA	CY050101	H1N1	09/04/2009	China	A/Qingdao/1006/2009(H1N1)
NA	HM006722	H1N1	09/04/2009	China	A/Xian/002/2009(H1N1)
NA	HQ247665	H1N1	09/04/2009	Finland	A/Finland/632/2009
NA	HM072403	H1N1	09/04/2009	Taiwan	A/Taiwan/6662/2009
NA	HM072404	H1N1	09/04/2009	Taiwan	A/Taiwan/6663/2009
NA	HM072405	H1N1	09/04/2009	Taiwan	A/Taiwan/7717/2009
NA	CY065882	H1N1	09/04/2009	Netherlar	A/Netherlands/1093/2009(H1N1)
NA	HM006723	H1N1	09/05/2009	China	A/Xian/004/2009(H1N1)
NA	CY052921	H1N1	09/05/2009	USA	A/Texas/45052569/2009(H1N1)
NA	KT181596	H1N1	09/05/2009	Singapore	A/Singapore/DMS835/2009
NA	JQ431257	H1N1	09/05/2009	France	A/Reunion/0116-3-M1E/2009
NA	CY072808	H1N1	09/05/2009	Nicaragua	A/Managua/572.02/2009(H1N1)
NA	KC781584	H1N1	09/05/2009	USA	A/Alaska/36/2009
NA	LC093035	H1N1	09/05/2009	Saudi Ara	A/Riyadh/124/2009
NA	KJ958937	H1N1	09/05/2009	India	A/Mumbai/3049/2009
NA	CY071836	H1N1	09/05/2009	USA	A/South Carolina/WRSP520/2009(H1N1)
NA	JQ431278	H1N1	09/05/2009	France	A/Reunion/2154-2-M5E/2009
NA	CY051897	H1N1	09/06/2009	USA	A/Texas/45062633/2009(H1N1)
NA	KT181535	H1N1	09/06/2009	Singapore	A/Singapore/DMS31/2009
NA	CY123162	H1N1	09/06/2009	Singapore	A/Singapore/GP3583/2009
NA	CY052633	H1N1	09/06/2009	USA	A/Texas/45061670/2009(H1N1)
NA	CY052953	H1N1	09/06/2009	USA	A/Texas/45061755/2009(H1N1)
NA	CY052889	H1N1	09/06/2009	USA	A/Texas/45062346/2009(H1N1)
NA	CY051785	H1N1	09/06/2009	USA	A/New York/4844/2009(H1N1)
NA	KJ958939	H1N1	09/06/2009	India	A/Mumbai/3071/2009
NA	KC781820	H1N1	09/06/2009	USA	A/District Of Columbia/09/2009
NA	KC781951	H1N1	09/06/2009	USA	A/Delaware/12/2009
NA	HQ239978	H1N1	09/06/2009	Canada	A/Canada-AB/RV2854/2009
NA	GU108488	H1N1	09/06/2009	China	A/Zhejiang-Yiwu/11/2009(H1N1)
NA	HM006724	H1N1	09/06/2009	China	A/Xian/006/2009(H1N1)
NA	CY052641	H1N1	09/06/2009	USA	A/Texas/45062584/2009(H1N1)
NA	CY057880	H1N1	09/07/2009	USA	A/Wisconsin/629-D01987/2009(H1N1)
NA	HM241719	H1N1	09/07/2009	India	A/Nsk/NIV10348/2009(H1N1)
NA	CY119484	H1N1	09/07/2009	Malaysia	A/Malaysia/2156796/2009
NA	CY123160	H1N1	09/07/2009	Singapore	A/Singapore/GP3560/2009
NA	CY123164	H1N1	09/07/2009	Singapore	A/Singapore/GP3594/2009
NA	JQ431264	H1N1	09/07/2009	France	A/Reunion/0215-4-M2E/2009
NA	JQ431258	H1N1	09/07/2009	France	A/Reunion/0116-3-M2E/2009
NA	CY052793	H1N1	09/07/2009	USA	A/Texas/45072273/2009(H1N1)

NA	CY052809	H1N1	09/07/2009	USA	A/Texas/45071344/2009(H1N1)
NA	CY052817	H1N1	09/07/2009	USA	A/Texas/45072656/2009(H1N1)
NA	CY052825	H1N1	09/07/2009	USA	A/Texas/45072128/2009(H1N1)
NA	KC782190	H1N1	09/07/2009	USA	A/Kentucky/20/2009
NA	CY088640	H1N1	09/07/2009	India	A/Pune/NIV10278/2009
NA	CY060552	H1N1	09/07/2009	Canada	A/Ontario/25913/2009(H1N1)
NA	HQ239986	H1N1	09/07/2009	Canada	A/Canada-ON/RV2904/2009
NA	HQ239976	H1N1	09/07/2009	Canada	A/Canada-NWT/RV2852/2009
NA	LC093015	H1N1	09/07/2009	Saudi Ara	A/Riyadh/898/2009
NA	LC093021	H1N1	09/07/2009	Saudi Ara	A/Riyadh/891/2009
NA	LC093040	H1N1	09/07/2009	Saudi Ara	A/Riyadh/953/2009
NA	KJ958940	H1N1	09/07/2009	India	A/Mumbai/3072/2009
NA	KJ958938	H1N1	09/07/2009	India	A/Mumbai/3051/2009
NA	GU112092	H1N1	09/07/2009	China	A/Zhejiang/DTID-ZJU02/2009(H1N1)
NA	HM006725	H1N1	09/07/2009	China	A/Xian/009/2009(H1N1)
NA	GU189651	H1N1	09/07/2009	China	A/Zhejiang/DTID-ZJU03/2009(H1N1)
NA	CY066096	H1N1	09/07/2009	Japan	A/Hyogo/09K091/2009
NA	CY052689	H1N1	09/07/2009	USA	A/Texas/45071524/2009(H1N1)
NA	KC780074	H1N1	09/08/2009	USA	A/Oregon/32/2009
NA	KC781479	H1N1	09/08/2009	USA	A/Nebraska/18/2009
NA	CY073691	H1N1	09/08/2009	Nicaragua	A/Managua/5953.01/2009(H1N1)
NA	KJ958955	H1N1	09/08/2009	India	A/Mumbai/3086/2009
NA	CY123723	H1N1	09/08/2009	Singapore	A/Singapore/ON2020/2009
NA	KR870127	H1N1	09/08/2009	India	A/Bangalore/6053-14/2009
NA	KT181599	H1N1	09/08/2009	Singapore	A/Singapore/DMS869/2009
NA	KT181597	H1N1	09/08/2009	Singapore	A/Singapore/DMS847/2009
NA	CY123727	H1N1	09/08/2009	Singapore	A/Singapore/ON2028/2009
NA	CY123703	H1N1	09/08/2009	Singapore	A/Singapore/ON2009/2009
NA	CY123725	H1N1	09/08/2009	Singapore	A/Singapore/ON2024/2009
NA	CY123759	H1N1	09/08/2009	Singapore	A/Singapore/ON2111/2009
NA	CY123166	H1N1	09/08/2009	Singapore	A/Singapore/GP3621/2009
NA	KC780066	H1N1	09/08/2009	USA	A/Rhode Island/17/2009
NA	KC782106	H1N1	09/08/2009	USA	A/Iowa/08/2009
NA	KC781599	H1N1	09/08/2009	USA	A/Wyoming/14/2009
NA	HQ239977	H1N1	09/08/2009	Canada	A/Canada-AB/RV2853/2009
NA	CY052092	H1N1	09/08/2009	Italy	A/Italy/233/2009(H1N1)
NA	KJ958941	H1N1	09/08/2009	India	A/Mumbai/3087/2009
NA	CY057005	H1N1	09/08/2009	USA	A/Wisconsin/629-D02367/2009(H1N1)
NA	CY056997	H1N1	09/08/2009	USA	A/Wisconsin/629-D00734/2009(H1N1)
NA	HM006726	H1N1	09/08/2009	China	A/Xian/010/2009(H1N1)
NA	CY128229	H1N1	09/08/2009	Viet Nam	A/Viet Nam/13032040/2009
NA	CY052697	H1N1	09/08/2009	USA	A/Texas/45083819/2009(H1N1)
NA	HQ247642	H1N1	09/08/2009	Finland	A/Finland/609/2009
NA	HQ247643	H1N1	09/08/2009	Finland	A/Finland/610/2009
NA	CY052000	H1N1	09/08/2009	Norway	A/Norway/3364-2/2009(H1N1)
NA	KC781587	H1N1	09/09/2009	USA	A/Maine/08/2009
NA	CY051801	H1N1	09/09/2009	USA	A/New York/4866/2009(H1N1)
NA	CY052721	H1N1	09/09/2009	USA	A/Texas/45091402/2009(H1N1)
NA	CY052729	H1N1	09/09/2009	USA	A/Texas/45091405/2009(H1N1)
NA	KJ958977	H1N1	09/09/2009	India	A/Mumbai/3140/2009
NA	CY052705	H1N1	09/09/2009	USA	A/Texas/45093214/2009(H1N1)
NA	CY066070	H1N1	09/09/2009	Japan	A/Kyoto/09K002/2009
NA	KT181600	H1N1	09/09/2009	Singapore	A/Singapore/DMS870/2009
NA	KR870128	H1N1	09/09/2009	India	A/Bangalore/6206-12/2009

NA	CY056247	H1N1	09/09/2009	Malaysia	A/Malaysia/15110/2009
NA	CY052737	H1N1	09/09/2009	USA	A/Texas/45091417/2009(H1N1)
NA	CY123168	H1N1	09/09/2009	Singapore	A/Singapore/GP3624/2009
NA	CY123729	H1N1	09/09/2009	Singapore	A/Singapore/ON2035/2009
NA	CY052657	H1N1	09/09/2009	USA	A/Texas/45093846/2009(H1N1)
NA	CY052665	H1N1	09/09/2009	USA	A/Texas/45093670/2009(H1N1)
NA	KC781690	H1N1	09/09/2009	USA	A/Ohio/15/2009
NA	KC780191	H1N1	09/09/2009	USA	A/Texas/70/2009
NA	CY123731	H1N1	09/09/2009	Singapore	A/Singapore/ON2040/2009
NA	CY049019	H1N1	09/09/2009	Spain	A/Catalonia/S1350/2009(H1N1)
NA	CY049022	H1N1	09/09/2009	Spain	A/Catalonia/S1353/2009(H1N1)
NA	CY069084	H1N1	09/09/2009	Guam	A/Guam/NHRC0025/2009(H1N1)
NA	CY069108	H1N1	09/09/2009	Guam	A/Guam/NHRC0028/2009(H1N1)
NA	CY057021	H1N1	09/09/2009	USA	A/Wisconsin/629-D02133/2009(H1N1)
NA	HM006727	H1N1	09/09/2009	China	A/Xian/017/2009(H1N1)
NA	CY057013	H1N1	09/09/2009	USA	A/Wisconsin/629-D02041/2009(H1N1)
NA	HM568138	H1N1	09/09/2009	United Ki	A/England/H093780041/2009
NA	JN187185	H1N1	09/09/2009	Taiwan	A/Taiwan/937/2009
NA	CY050045	H1N1	09/09/2009	Mexico	A/Mexico City/WR1310N/2009(H1N1)
NA	CY051793	H1N1	09/09/2009	USA	A/New York/4856/2009(H1N1)
NA	CY052172	H1N1	09/09/2009	USA	A/New York/4855/2009(H1N1)
NA	HQ247651	H1N1	09/09/2009	Finland	A/Finland/618/2009
NA	CY083401	H1N1	09/09/2009	USA	A/Great Lakes/WRAIR1664P/2009
NA	CY083377	H1N1	09/09/2009	USA	A/Pendleton/WRAIR1661P/2009(H1N1)
NA	KC781572	H1N1	09/09/2009	USA	A/Kansas/18/2009
NA	GU576525	H1N1	09/10/2009	Italy	A/Ancona/97/2009(H1N1)
NA	CY052529	H1N1	09/10/2009	USA	A/Texas/45103998/2009(H1N1)
NA	CY118259	H1N1	09/10/2009	Malaysia	A/Malaysia/2089302/2009
NA	CY118258	H1N1	09/10/2009	Malaysia	A/Malaysia/2089302/2009
NA	CY123170	H1N1	09/10/2009	Singapore	A/Singapore/GP3668/2009
NA	CY123733	H1N1	09/10/2009	Singapore	A/Singapore/ON2054/2009
NA	KT181604	H1N1	09/10/2009	Singapore	A/Singapore/DMS905/2009
NA	HM241726	H1N1	09/10/2009	India	A/Pune/NIV10604/2009
NA	CY056248	H1N1	09/10/2009	Malaysia	A/Malaysia/15192/2009
NA	CY052753	H1N1	09/10/2009	USA	A/Texas/45103759/2009(H1N1)
NA	CY052745	H1N1	09/10/2009	USA	A/Texas/45101424/2009(H1N1)
NA	CY052761	H1N1	09/10/2009	USA	A/Texas/45101422/2009(H1N1)
NA	CY052897	H1N1	09/10/2009	USA	A/Texas/45102952/2009(H1N1)
NA	CY052649	H1N1	09/10/2009	USA	A/Texas/45103259/2009(H1N1)
NA	CY052505	H1N1	09/10/2009	USA	A/Texas/45104026/2009(H1N1)
NA	CY052513	H1N1	09/10/2009	USA	A/Texas/45103737/2009(H1N1)
NA	CY052521	H1N1	09/10/2009	USA	A/Texas/45104048/2009(H1N1)
NA	KC781811	H1N1	09/10/2009	USA	A/Nevada/15/2009
NA	KC781751	H1N1	09/10/2009	USA	A/North Carolina/37/2009
NA	KC780811	H1N1	09/10/2009	USA	A/Montana/17/2009
NA	KC781558	H1N1	09/10/2009	USA	A/Washington/57/2009
NA	CY123735	H1N1	09/10/2009	Singapore	A/Singapore/ON2058/2009
NA	CY072920	H1N1	09/10/2009	Nicaragua	A/Managua/4972.02/2009(H1N1)
NA	CY063796	H1N1	09/10/2009	Singapore	A/Singapore/ON2063/2009
NA	KY925041	H1N1	09/10/2009	Brazil	A/Campina das Missoes/LACENRS-2882/2
NA	HQ256671	H1N1	09/10/2009	Myanmar	A/Myanmar/M571/2009(H1N1)
NA	HQ256669	H1N1	09/10/2009	Myanmar	A/Myanmar/M570/2009(H1N1)
NA	CY057029	H1N1	09/10/2009	USA	A/Wisconsin/629-D00357/2009(H1N1)
NA	JN187186	H1N1	09/10/2009	Taiwan	A/Taiwan/940/2009

NA	CY050061	H1N1	09/10/2009	Mexico	A/Mexico City/WR1312N/2009(H1N1)
NA	HQ247666	H1N1	09/10/2009	Finland	A/Finland/633/2009
NA	HM568146	H1N1	09/10/2009	United Ki	A/England/H093780034/2009
NA	CY051809	H1N1	09/10/2009	USA	A/New York/4870/2009(H1N1)
NA	CY050053	H1N1	09/10/2009	Mexico	A/Mexico City/WR1311T/2009(H1N1)
NA	CY065906	H1N1	09/10/2009	Netherlar	A/Netherlands/1083/2009(H1N1)
NA	CY065914	H1N1	09/10/2009	Netherlar	A/Netherlands/1084/2009(H1N1)
NA	CY067722	H1N1	09/10/2009	United Ki	A/England/93960032/2009
NA	KJ958979	H1N1	09/10/2009	India	A/Mumbai/3133/2009
NA	KJ958950	H1N1	09/10/2009	India	A/Mumbai/3109/2009
NA	CY119516	H1N1	09/10/2009	Malaysia	A/Malaysia/2097960/2009
NA	KC781674	H1N1	09/11/2009	USA	A/Georgia/23/2009
NA	CY073515	H1N1	09/11/2009	Nicaragua	A/Managua/3014.02/2009(H1N1)
NA	CY052913	H1N1	09/11/2009	USA	A/Texas/45113911/2009(H1N1)
NA	CY052537	H1N1	09/11/2009	USA	A/Texas/45113882/2009(H1N1)
NA	CY052497	H1N1	09/11/2009	USA	A/Texas/45113371/2009(H1N1)
NA	CY123739	H1N1	09/11/2009	Singapore	A/Singapore/ON2067/2009
NA	CY123737	H1N1	09/11/2009	Singapore	A/Singapore/ON2064/2009
NA	CY123741	H1N1	09/11/2009	Singapore	A/Singapore/ON2075/2009
NA	KT181606	H1N1	09/11/2009	Singapore	A/Singapore/DMS913/2009
NA	CY128237	H1N1	09/11/2009	Viet Nam	A/Viet Nam/13032044/2009
NA	KC780108	H1N1	09/11/2009	USA	A/Alaska/38/2009
NA	CY056285	H1N1	09/11/2009	Malaysia	A/Malaysia/15333/2009
NA	LC093013	H1N1	09/11/2009	Saudi Ara	A/Riyadh/1058/2009
NA	LC093017	H1N1	09/11/2009	Saudi Ara	A/Riyadh/1059/2009
NA	KJ958978	H1N1	09/11/2009	India	A/Mumbai/3139/2009
NA	CY072824	H1N1	09/11/2009	Nicaragua	A/Managua/5115.01/2009(H1N1)
NA	KJ958942	H1N1	09/11/2009	India	A/Mumbai/3158/2009
NA	CY056250	H1N1	09/11/2009	Malaysia	A/Malaysia/15326/2009
NA	CY123156	H1N1	09/11/2009	Singapore	A/Singapore/GP3545/2009
NA	CY083066	H1N1	09/11/2009	Colombia	A/Bogota/WRAIR0435N/2009
NA	CY083074	H1N1	09/11/2009	Colombia	A/Bogota/WRAIR0435T/2009
NA	CY057360	H1N1	09/11/2009	USA	A/Wisconsin/629-D02485/2009(H1N1)
NA	CY056249	H1N1	09/12/2009	Malaysia	A/Malaysia/15294/2009
NA	CY052465	H1N1	09/12/2009	USA	A/Texas/45121606/2009(H1N1)
NA	CY052473	H1N1	09/12/2009	USA	A/Texas/45122033/2009(H1N1)
NA	CY052481	H1N1	09/12/2009	USA	A/Texas/45122282/2009(H1N1)
NA	CY052553	H1N1	09/12/2009	USA	A/Texas/45122774/2009(H1N1)
NA	CY052561	H1N1	09/12/2009	USA	A/Texas/45122722/2009(H1N1)
NA	CY052489	H1N1	09/12/2009	USA	A/Texas/45122036/2009(H1N1)
NA	CY052929	H1N1	09/12/2009	USA	A/Texas/45121004/2009(H1N1)
NA	CY050013	H1N1	09/12/2009	Mexico	A/Mexico City/WR1301N/2009(H1N1)
NA	KT181536	H1N1	09/12/2009	Singapore	A/Singapore/DMS33/2009
NA	CY052945	H1N1	09/12/2009	USA	A/Texas/45122886/2009(H1N1)
NA	CY052433	H1N1	09/12/2009	USA	A/Texas/45120922/2009(H1N1)
NA	CY052441	H1N1	09/12/2009	USA	A/Texas/45122538/2009(H1N1)
NA	KJ958943	H1N1	09/12/2009	India	A/Mumbai/3165/2009
NA	CY057376	H1N1	09/12/2009	USA	A/Wisconsin/629-D01850/2009(H1N1)
NA	CY056284	H1N1	09/12/2009	Malaysia	A/Malaysia/15263/2009
NA	CY056251	H1N1	09/12/2009	Malaysia	A/Malaysia/15332/2009
NA	KJ958944	H1N1	09/12/2009	India	A/Mumbai/3166/2009
NA	CY072736	H1N1	09/12/2009	Nicaragua	A/Managua/1419.02/2009(H1N1)
NA	CY072832	H1N1	09/12/2009	Nicaragua	A/Managua/1909.02/2009(H1N1)
NA	CY050114	H1N1	09/12/2009	China	A/Qingdao/1215/2009(H1N1)

NA	CY052180	H1N1	09/12/2009	USA	A/New York/4881/2009(H1N1)
NA	CY052457	H1N1	09/12/2009	USA	A/Texas/45122369/2009(H1N1)
NA	CY057368	H1N1	09/12/2009	USA	A/Wisconsin/629-D00643/2009(H1N1)
NA	KC780882	H1N1	09/13/2009	USA	A/Texas/67/2009
NA	CY052833	H1N1	09/13/2009	USA	A/Texas/45131305/2009(H1N1)
NA	CY052841	H1N1	09/13/2009	USA	A/Texas/45132202/2009(H1N1)
NA	CY052801	H1N1	09/13/2009	USA	A/Texas/45131576/2009(H1N1)
NA	CY052545	H1N1	09/13/2009	USA	A/Texas/45132214/2009(H1N1)
NA	CY052577	H1N1	09/13/2009	USA	A/Texas/45131774/2009(H1N1)
NA	CY052585	H1N1	09/13/2009	USA	A/Texas/45132647/2009(H1N1)
NA	CY052449	H1N1	09/13/2009	USA	A/Texas/45130742/2009(H1N1)
NA	KC781826	H1N1	09/13/2009	USA	A/Connecticut/13/2009
NA	KC780879	H1N1	09/13/2009	USA	A/Texas/67/2009
NA	CY049653	H1N1	09/13/2009	Singapore	A/Singapore/ON2081/2009(H1N1)
NA	LC093011	H1N1	09/13/2009	Saudi Ara	A/Riyadh/1327/2009
NA	LC093036	H1N1	09/13/2009	Saudi Ara	A/Riyadh/1120/2009
NA	CY063798	H1N1	09/13/2009	Singapore	A/Singapore/ON2083/2009
NA	CY069020	H1N1	09/13/2009	Guam	A/Guam/NHRC0017/2009(H1N1)
NA	CY052569	H1N1	09/13/2009	USA	A/Texas/45132788/2009(H1N1)
NA	JF429401	H1N1	09/14/2009	France	A/StEtienne/1691/2009
NA	CY052713	H1N1	09/14/2009	USA	A/Texas/45140902/2009(H1N1)
NA	CY123180	H1N1	09/14/2009	Singapore	A/Singapore/GP3733/2009
NA	CY123176	H1N1	09/14/2009	Singapore	A/Singapore/GP3710/2009
NA	JX625562	H1N1	09/14/2009	United Ki	A/England/94560024/2009
NA	AB649897	H1N1	09/14/2009	Japan	A/Toyama/291/2009
NA	CY056252	H1N1	09/14/2009	Malaysia	A/Malaysia/15369/2009
NA	CY056286	H1N1	09/14/2009	Malaysia	A/Malaysia/15395/2009
NA	CY055425	H1N1	09/14/2009	USA	A/New York/4887/2009(H1N1)
NA	KC780827	H1N1	09/14/2009	USA	A/Kansas/20/2009
NA	KC781780	H1N1	09/14/2009	USA	A/Wisconsin/50/2009
NA	KC781755	H1N1	09/14/2009	USA	A/Texas/60/2009
NA	KC780924	H1N1	09/14/2009	USA	A/Kansas/19/2009
NA	CY066078	H1N1	09/14/2009	Japan	A/Kyoto/09K033/2009
NA	HQ239988	H1N1	09/14/2009	Canada	A/Canada-ON/RV2906/2009
NA	CY069044	H1N1	09/14/2009	Guam	A/Guam/NHRC0020/2009(H1N1)
NA	CY072936	H1N1	09/14/2009	Nicaragua	A/Managua/5590.02/2009(H1N1)
NA	CY063800	H1N1	09/14/2009	Singapore	A/Singapore/ON2090/2009
NA	CY071017	H1N1	09/14/2009	Guam	A/Guam/NHRC0032/2009(H1N1)
NA	CY067832	H1N1	09/14/2009	Portugal	A/Lisboa/60/2009(H1N1)
NA	CY067835	H1N1	09/14/2009	Portugal	A/Lisboa/61/2009(H1N1)
NA	CY067838	H1N1	09/14/2009	Portugal	A/Lisboa/62/2009(H1N1)
NA	CY067841	H1N1	09/14/2009	Portugal	A/Lisboa/63/2009(H1N1)
NA	CY067844	H1N1	09/14/2009	Portugal	A/Lisboa/64/2009(H1N1)
NA	CY067847	H1N1	09/14/2009	Portugal	A/Lisboa/65/2009(H1N1)
NA	CY050029	H1N1	09/14/2009	Mexico	A/Mexico City/WR1307N/2009(H1N1)
NA	HQ247679	H1N1	09/14/2009	Finland	A/Finland/651/2009
NA	CY071009	H1N1	09/14/2009	Guam	A/Guam/NHRC0031/2009(H1N1)
NA	CY072888	H1N1	09/14/2009	Nicaragua	A/Managua/12.01/2009(H1N1)
NA	HQ256673	H1N1	09/14/2009	Myanmar	A/Myanmar/M574/2009(H1N1)
NA	KJ909524	H1N1	09/14/2009	India	A/Mumbai/3212/2009
NA	CY123747	H1N1	09/14/2009	Singapore	A/Singapore/ON2081/2009
NA	CY057384	H1N1	09/15/2009	USA	A/Wisconsin/629-D02192/2009(H1N1)
NA	CY056253	H1N1	09/15/2009	Malaysia	A/Malaysia/15431/2009
NA	KC782054	H1N1	09/15/2009	USA	A/South Carolina/24/2009

NA	KC781531	H1N1	09/15/2009	USA	A/Arizona/16/2009
NA	KC781046	H1N1	09/15/2009	USA	A/Massachusetts/31/2009
NA	KC781819	H1N1	09/15/2009	USA	A/Texas/59/2009
NA	KC781666	H1N1	09/15/2009	USA	A/South Carolina/26/2009
NA	CY057392	H1N1	09/15/2009	USA	A/Wisconsin/629-D00036/2009(H1N1)
NA	CY057400	H1N1	09/15/2009	USA	A/Wisconsin/629-D01469/2009(H1N1)
NA	CY123755	H1N1	09/15/2009	Singapore	A/Singapore/ON2095/2009
NA	CY119492	H1N1	09/15/2009	Malaysia	A/Malaysia/2173411/2009
NA	CY053476	H1N1	09/15/2009	Taiwan	A/Taiwan/143/2009
NA	CY066098	H1N1	09/15/2009	Japan	A/Hyogo/09K100/2009
NA	KT181610	H1N1	09/15/2009	Singapore	A/Singapore/DMS952/2009
NA	KT181537	H1N1	09/15/2009	Singapore	A/Singapore/DMS34/2009
NA	KT181609	H1N1	09/15/2009	Singapore	A/Singapore/DMS946/2009
NA	CY071732	H1N1	09/15/2009	Mexico	A/Mexico City/WR1697T/2009(H1N1)
NA	CY090091	H1N1	09/15/2009	Nicaragua	A/Managua/3755.03/2009
NA	CY069068	H1N1	09/15/2009	Guam	A/Guam/NHRC0023/2009(H1N1)
NA	CY050118	H1N1	09/15/2009	China	A/Qingdao/1261/2009(H1N1)
NA	CY067829	H1N1	09/15/2009	Portugal	A/Lisboa/59/2009(H1N1)
NA	CY067850	H1N1	09/15/2009	Portugal	A/Lisboa/66/2009(H1N1)
NA	CY067853	H1N1	09/15/2009	Portugal	A/Lisboa/67/2009(H1N1)
NA	CY067856	H1N1	09/15/2009	Portugal	A/Lisboa/68/2009(H1N1)
NA	CY067859	H1N1	09/15/2009	Portugal	A/Lisboa/69/2009(H1N1)
NA	CY067862	H1N1	09/15/2009	Portugal	A/Lisboa/70/2009(H1N1)
NA	CY067865	H1N1	09/15/2009	Portugal	A/Lisboa/71/2009(H1N1)
NA	CY067868	H1N1	09/15/2009	Portugal	A/Lisboa/72/2009(H1N1)
NA	CY057408	H1N1	09/15/2009	USA	A/Wisconsin/629-D00832/2009(H1N1)
NA	CY056288	H1N1	09/15/2009	Malaysia	A/Malaysia/15483/2009
NA	HQ286395	H1N1	09/15/2009	Japan	A/Kurume/6/2009
NA	HQ286397	H1N1	09/15/2009	Japan	A/Kurume/8/2009
NA	HQ286399	H1N1	09/15/2009	Japan	A/Kurume/10/2009
NA	HQ286391	H1N1	09/15/2009	Japan	A/Kurume/2/2009
NA	HQ286390	H1N1	09/15/2009	Japan	A/Kurume/1/2009
NA	HQ286396	H1N1	09/15/2009	Japan	A/Kurume/7/2009
NA	HQ286400	H1N1	09/15/2009	Japan	A/Kurume/11/2009
NA	HQ286398	H1N1	09/15/2009	Japan	A/Kurume/9/2009
NA	HQ286392	H1N1	09/15/2009	Japan	A/Kurume/3/2009
NA	CY049025	H1N1	09/15/2009	Spain	A/Catalonia/S1369/2009(H1N1)
NA	LC093005	H1N1	09/15/2009	Saudi Ara	A/Riyadh/1342/2009
NA	HQ286393	H1N1	09/15/2009	Japan	A/Kurume/4/2009
NA	HQ286394	H1N1	09/15/2009	Japan	A/Kurume/5/2009
NA	CY049028	H1N1	09/16/2009	Spain	A/Catalonia/S1379/2009(H1N1)
NA	KR870129	H1N1	09/16/2009	India	A/Bangalore/6974-08/2009
NA	CY050120	H1N1	09/16/2009	China	A/Qingdao/1268/2009(H1N1)
NA	KC781876	H1N1	09/16/2009	USA	A/Arizona/15/2009
NA	CY057416	H1N1	09/16/2009	USA	A/Wisconsin/629-D01802/2009(H1N1)
NA	CY057424	H1N1	09/16/2009	USA	A/Wisconsin/629-D00485/2009(H1N1)
NA	CY057432	H1N1	09/16/2009	USA	A/Wisconsin/629-D01935/2009(H1N1)
NA	KC782048	H1N1	09/16/2009	USA	A/South Carolina/17/2009
NA	KC781554	H1N1	09/16/2009	USA	A/South Carolina/18/2009
NA	KC781831	H1N1	09/16/2009	USA	A/Florida/20/2009
NA	CY056287	H1N1	09/16/2009	Malaysia	A/Malaysia/15473/2009
NA	KT181612	H1N1	09/16/2009	Singapore	A/Singapore/DMS962/2009
NA	KC782204	H1N1	09/16/2009	USA	A/South Carolina/18/2009
NA	KC780086	H1N1	09/16/2009	USA	A/South Carolina/18/2009

NA	CY120733	H1N1	09/16/2009	Brazil	A/Brazil/AVS06/2009
NA	KC780079	H1N1	09/16/2009	USA	A/South Carolina/18/2009
NA	KC782212	H1N1	09/16/2009	USA	A/South Carolina/18/2009
NA	KC781535	H1N1	09/16/2009	USA	A/South Carolina/22/2009
NA	KC780855	H1N1	09/16/2009	USA	A/Kansas/21/2009
NA	KC782107	H1N1	09/16/2009	USA	A/North Carolina/35/2009
NA	KC781308	H1N1	09/16/2009	USA	A/Wisconsin/66/2009
NA	CY050122	H1N1	09/16/2009	China	A/Qingdao/1269/2009(H1N1)
NA	CY067913	H1N1	09/16/2009	Portugal	A/Lisboa/87/2009(H1N1)
NA	KJ690473	H1N1	09/16/2009	Uganda	A/Uganda/MUWRP-094/2009
NA	HQ256665	H1N1	09/16/2009	Myanmar	A/Myanmar/M544/2009(H1N1)
NA	KJ958976	H1N1	09/16/2009	India	A/Mumbai/3264/2009
NA	HQ256667	H1N1	09/16/2009	Myanmar	A/Myanmar/M545/2009(H1N1)
NA	CY072784	H1N1	09/16/2009	Nicaragua	A/Managua/5401.01/2009(H1N1)
NA	CY066142	H1N1	09/16/2009	Japan	A/Nagasaki/09N018/2009
NA	HQ247652	H1N1	09/16/2009	Finland	A/Finland/619/2009
NA	CY088654	H1N1	09/16/2009	India	A/Rtng/NIV11650/2009
NA	CY123184	H1N1	09/16/2009	Singapore	A/Singapore/GP3804/2009
NA	CY049031	H1N1	09/17/2009	Spain	A/Catalonia/S1383/2009(H1N1)
NA	CY090067	H1N1	09/17/2009	Nicaragua	A/Managua/4877.02/2009
NA	CY071724	H1N1	09/17/2009	Mexico	A/Mexico City/WR1696T/2009(H1N1)
NA	KC781657	H1N1	09/17/2009	USA	A/South Carolina/23/2009
NA	KC781602	H1N1	09/17/2009	USA	A/South Carolina/38/2009
NA	KC781612	H1N1	09/17/2009	USA	A/South Carolina/25/2009
NA	KC781957	H1N1	09/17/2009	USA	A/Minnesota/13/2009
NA	CY123697	H1N1	09/17/2009	Singapore	A/Singapore/ON1999/2009
NA	CY123691	H1N1	09/17/2009	Singapore	A/Singapore/ON1982/2009
NA	CY128245	H1N1	09/17/2009	Viet Nam	A/Viet Nam/13032048/2009
NA	CY062258	H1N1	09/17/2009	Malaysia	A/Malaysia/15506/2009
NA	CY056289	H1N1	09/17/2009	Malaysia	A/Malaysia/15504/2009
NA	KC781955	H1N1	09/17/2009	USA	A/South Carolina/27/2009
NA	CY062259	H1N1	09/17/2009	Malaysia	A/Malaysia/15521/2009
NA	HQ239995	H1N1	09/17/2009	Canada	A/Canada-ON/RV2914/2009
NA	CY049034	H1N1	09/17/2009	Spain	A/Catalonia/S1384/2009(H1N1)
NA	LC093004	H1N1	09/17/2009	Saudi Ara	A/Riyadh/1363/2009
NA	CY053508	H1N1	09/18/2009	Taiwan	A/Taiwan/206/2009
NA	CY119500	H1N1	09/18/2009	Malaysia	A/Malaysia/2180812/2009
NA	KY925671	H1N1	09/18/2009	Brazil	A/Pelotas/LACENRS-3045/2009
NA	CY057440	H1N1	09/18/2009	USA	A/Wisconsin/629-D01522/2009(H1N1)
NA	HQ256675	H1N1	09/18/2009	Myanmar	A/Myanmar/M576/2009(H1N1)
NA	HQ240021	H1N1	09/18/2009	Canada	A/Canada-BC/RV2950/2009
NA	KP637544	H1N1	09/18/2009	Thailand	A/Thailand/SN08312/2009
NA	CY053484	H1N1	09/18/2009	Taiwan	A/Taiwan/156/2009
NA	KT181617	H1N1	09/18/2009	Singapore	A/Singapore/DMS982/2009
NA	CY053500	H1N1	09/18/2009	Taiwan	A/Taiwan/177/2009
NA	CY071756	H1N1	09/18/2009	Mexico	A/Mexico City/WR1706T/2009(H1N1)
NA	KC780957	H1N1	09/18/2009	USA	A/North Carolina/58/2009
NA	HQ247654	H1N1	09/18/2009	Finland	A/Finland/621/2009
NA	CY123182	H1N1	09/18/2009	Singapore	A/Singapore/GP3791/2009
NA	KC782184	H1N1	09/18/2009	USA	A/Texas/76/2009
NA	HM568154	H1N1	09/18/2009	United Ki	A/England/H093900113/2009
NA	CY050131	H1N1	09/18/2009	China	A/Qingdao/1364/2009(H1N1)
NA	CY053492	H1N1	09/18/2009	Taiwan	A/Taiwan/167/2009
NA	CY064937	H1N1	09/18/2009	Italy	A/Rome/707/2009(H1N1)

NA	KT181614	H1N1	09/18/2009	Singapore	A/Singapore/DMS978/2009
NA	KT181616	H1N1	09/18/2009	Singapore	A/Singapore/DMS980/2009
NA	KC780057	H1N1	09/18/2009	USA	A/Virginia/20/2009
NA	KT181538	H1N1	09/18/2009	Singapore	A/Singapore/DMS35/2009
NA	HQ247655	H1N1	09/18/2009	Finland	A/Finland/622/2009
NA	HQ256677	H1N1	09/18/2009	Myanmar	A/Myanmar/M577/2009(H1N1)
NA	KJ958973	H1N1	09/19/2009	India	A/Mumbai/3417/2009
NA	CY055504	H1N1	09/19/2009	USA	A/California/VRDL75/2009(H1N1)
NA	KJ958974	H1N1	09/19/2009	India	A/Mumbai/3413/2009
NA	CY056290	H1N1	09/19/2009	Malaysia	A/Malaysia/15547/2009
NA	KJ958975	H1N1	09/19/2009	India	A/Mumbai/3411/2009
NA	KC781616	H1N1	09/20/2009	USA	A/Delaware/11/2009
NA	KC781060	H1N1	09/20/2009	USA	A/North Carolina/44/2009
NA	CY068996	H1N1	09/20/2009	Guam	A/Guam/NHRC0014/2009(H1N1)
NA	LC093028	H1N1	09/20/2009	Saudi Ara	A/Riyadh/1422/2009
NA	JN187187	H1N1	09/20/2009	Taiwan	A/Taiwan/8575/2009
NA	KC781868	H1N1	09/20/2009	USA	A/Washington/59/2009
NA	KT181411	H1N1	09/21/2009	Singapore	A/Singapore/DMS1006/2009
NA	CY055512	H1N1	09/21/2009	USA	A/California/VRDL76/2009(H1N1)
NA	CY057448	H1N1	09/21/2009	USA	A/Wisconsin/629-D01355/2009(H1N1)
NA	KC781544	H1N1	09/21/2009	USA	A/Utah/31/2009
NA	KC780126	H1N1	09/21/2009	USA	A/Mississippi/02/2009
NA	CY057456	H1N1	09/21/2009	USA	A/Wisconsin/629-D00748/2009(H1N1)
NA	KF687922	H1N1	09/21/2009	Cyprus	A/Cyprus/392/2009
NA	CY049037	H1N1	09/21/2009	Spain	A/Catalonia/S1389/2009(H1N1)
NA	CY049046	H1N1	09/21/2009	Spain	A/Catalonia/S1414/2009(H1N1)
NA	AB539741	H1N1	09/21/2009	China	A/Shanghai/P1/2009(H1N1)
NA	HQ247653	H1N1	09/21/2009	Finland	A/Finland/620/2009
NA	CY083433	H1N1	09/21/2009	USA	A/San Diego/WRAIR1668P/2009
NA	KJ690520	H1N1	09/21/2009	Uganda	A/Uganda/MUWRP-092/2009
NA	CY057472	H1N1	09/22/2009	USA	A/Wisconsin/629-D00402/2009(H1N1)
NA	HQ247667	H1N1	09/22/2009	Finland	A/Finland/634/2009
NA	CY057464	H1N1	09/22/2009	USA	A/Wisconsin/629-D00839/2009(H1N1)
NA	HQ239982	H1N1	09/22/2009	Canada	A/Canada-ON/RV2889/2009
NA	KC781985	H1N1	09/22/2009	USA	A/Kansas/22/2009
NA	GU211229	H1N1	09/22/2009	Russia	A/Russia/01/2009(H1N1)
NA	CY057480	H1N1	09/22/2009	USA	A/Wisconsin/629-D01237/2009(H1N1)
NA	CY123763	H1N1	09/22/2009	Singapore	A/Singapore/ON2131/2009
NA	CY049040	H1N1	09/22/2009	Spain	A/Catalonia/S1402/2009(H1N1)
NA	CY071708	H1N1	09/22/2009	Mexico	A/Mexico City/WR1692T/2009(H1N1)
NA	KC781344	H1N1	09/22/2009	USA	A/Washington/58/2009
NA	CY128445	H1N1	09/22/2009	Viet Nam	A/Viet Nam/001-1716/2009
NA	CY123761	H1N1	09/22/2009	Singapore	A/Singapore/ON2125/2009
NA	CY055520	H1N1	09/22/2009	USA	A/California/VRDL78/2009(H1N1)
NA	KC781063	H1N1	09/23/2009	USA	A/Florida/23/2009
NA	CY056413	H1N1	09/23/2009	USA	A/New York/4945/2009(H1N1)
NA	CY123769	H1N1	09/23/2009	Singapore	A/Singapore/ON2135/2009
NA	KU296289	H1N1	09/23/2009	India	A/Kannur/MCVR739/2009
NA	CY123190	H1N1	09/23/2009	Singapore	A/Singapore/GP3839/2009
NA	CY123777	H1N1	09/23/2009	Singapore	A/Singapore/ON2136/2009
NA	CY056405	H1N1	09/23/2009	USA	A/New York/4944/2009(H1N1)
NA	CY056421	H1N1	09/23/2009	USA	A/New York/4947/2009(H1N1)
NA	KC781575	H1N1	09/23/2009	USA	A/Rhode Island/16/2009
NA	KC780975	H1N1	09/23/2009	USA	A/Kansas/23/2009

NA	CY057488	H1N1	09/23/2009	USA	A/Wisconsin/629-D00346/2009(H1N1)
NA	CY062260	H1N1	09/23/2009	Malaysia	A/Malaysia/15636/2009
NA	KJ690402	H1N1	09/23/2009	Uganda	A/Uganda/MUWRP-093/2009
NA	HM998848	H1N1	09/23/2009	China	A/Inner Mongolia/03/2009(H1N1)
NA	HM998849	H1N1	09/23/2009	China	A/Inner Mongolia/04/2009(H1N1)
NA	CY083489	H1N1	09/23/2009	Mexico	A/Mexico City/WRAIR1691N/2009(H1N1)
NA	CY083449	H1N1	09/23/2009	Mexico	A/Mexico City/WRAIR1679N/2009(H1N1)
NA	CY083409	H1N1	09/23/2009	USA	A/San Diego/WRAIR1665P/2009
NA	HQ247656	H1N1	09/23/2009	Finland	A/Finland/623/2009
NA	CY049043	H1N1	09/23/2009	Spain	A/Catalonia/S1411/2009(H1N1)
NA	HQ239996	H1N1	09/23/2009	Canada	A/Canada-SK/RV2924/2009
NA	CY071684	H1N1	09/23/2009	Mexico	A/Mexico City/WR1675T/2009(H1N1)
NA	CY057496	H1N1	09/23/2009	USA	A/Wisconsin/629-D00287/2009(H1N1)
NA	KT181539	H1N1	09/24/2009	Singapore	A/Singapore/DMS37/2009
NA	CY057504	H1N1	09/24/2009	USA	A/Wisconsin/629-D00565/2009(H1N1)
NA	KC781363	H1N1	09/24/2009	USA	A/Montana/18/2009
NA	KC782336	H1N1	09/24/2009	USA	A/Illinois/18/2009
NA	CY052360	H1N1	09/24/2009	United Ar	A/UAE/F01/2009(H1N1)
NA	CY057512	H1N1	09/24/2009	USA	A/Wisconsin/629-D00410/2009(H1N1)
NA	CY054885	H1N1	09/24/2009	USA	A/California/VRDL36/2009(H1N1)
NA	CY064938	H1N1	09/24/2009	Italy	A/Rome/708/2009(H1N1)
NA	CY119508	H1N1	09/24/2009	Malaysia	A/Malaysia/2190140/2009
NA	HQ239983	H1N1	09/24/2009	Canada	A/Canada-ON/RV2890/2009
NA	HQ239997	H1N1	09/24/2009	Canada	A/Canada-SK/RV2925/2009
NA	CY071740	H1N1	09/24/2009	Mexico	A/Mexico City/WR1701T/2009(H1N1)
NA	CY056291	H1N1	09/24/2009	Malaysia	A/Malaysia/15653/2009
NA	KC781834	H1N1	09/24/2009	USA	A/Vermont/19/2009
NA	CY066072	H1N1	09/25/2009	Japan	A/Kyoto/09K010/2009
NA	CY066100	H1N1	09/25/2009	Japan	A/Hyogo/09K106/2009
NA	KC780709	H1N1	09/25/2009	USA	A/Nebraska/19/2009
NA	CY050369	H1N1	09/25/2009	South Kor	A/Korea/S1/2009
NA	LC093003	H1N1	09/25/2009	Saudi Ara	A/Riyadh/1639/2009
NA	CY069060	H1N1	09/25/2009	Guam	A/Guam/NHRC0022/2009(H1N1)
NA	HQ240022	H1N1	09/25/2009	Canada	A/Canada-ON/RV2961/2009
NA	CY071748	H1N1	09/25/2009	Mexico	A/Mexico City/WR1704T/2009(H1N1)
NA	CY071692	H1N1	09/25/2009	Mexico	A/Mexico City/WR1683T/2009(H1N1)
NA	KC780263	H1N1	09/25/2009	USA	A/Minnesota/21/2009
NA	KC780154	H1N1	09/25/2009	USA	A/Mississippi/03/2009
NA	CY062261	H1N1	09/26/2009	Malaysia	A/Malaysia/15710/2009
NA	MG856208	H1N1	09/26/2009	Mexico	A/Mexico/IBT4/2009
NA	KC781323	H1N1	09/26/2009	USA	A/Michigan/24/2009
NA	CY057520	H1N1	09/26/2009	USA	A/Wisconsin/629-D00891/2009(H1N1)
NA	HQ239989	H1N1	09/26/2009	Canada	A/Canada-ON/RV2907/2009
NA	CY069076	H1N1	09/26/2009	Guam	A/Guam/NHRC0024/2009(H1N1)
NA	KC780905	H1N1	09/27/2009	USA	A/Wisconsin/51/2009
NA	CY066134	H1N1	09/27/2009	Japan	A/Nagasaki/09N005/2009
NA	HQ239985	H1N1	09/27/2009	Canada	A/Canada-ON/RV2892/2009
NA	JN187188	H1N1	09/28/2009	Taiwan	A/Taiwan/4909/2009
NA	JN187189	H1N1	09/28/2009	Taiwan	A/Taiwan/8542/2009
NA	KJ690481	H1N1	09/28/2009	Uganda	A/Uganda/MUWRP-098/2009
NA	CY057528	H1N1	09/28/2009	USA	A/Wisconsin/629-D01391/2009(H1N1)
NA	KC781908	H1N1	09/28/2009	USA	A/Texas/62/2009
NA	JQ431259	H1N1	09/28/2009	France	A/Reunion/1698-3-M1E/2009
NA	KP019721	H1N1	09/28/2009	China	A/FuZhou/SWL11479/2009

NA	CY049049	H1N1	09/28/2009	Spain	A/Catalonia/S1435/2009(H1N1)
NA	CY117621	H1N1	09/28/2009	Mexico	A/Mexico/UASLP-019/2009
NA	CY049052	H1N1	09/28/2009	Spain	A/Catalonia/S1436/2009(H1N1)
NA	CY123785	H1N1	09/28/2009	Singapore	A/Singapore/ON2145/2009
NA	KC782301	H1N1	09/28/2009	USA	A/California/38/2009
NA	KF687923	H1N1	09/28/2009	Cyprus	A/Cyprus/407/2009
NA	CY056292	H1N1	09/28/2009	Malaysia	A/Malaysia/15763/2009
NA	KF687924	H1N1	09/29/2009	Cyprus	A/Cyprus/412/2009
NA	CY060560	H1N1	09/29/2009	Canada	A/Ontario/26184/2009(H1N1)
NA	HQ239991	H1N1	09/29/2009	Canada	A/Canada-ON/RV2909/2009
NA	HQ239992	H1N1	09/29/2009	Canada	A/Canada-ON/RV2910/2009
NA	HQ239990	H1N1	09/29/2009	Canada	A/Canada-ON/RV2908/2009
NA	JX556121	H1N1	09/29/2009	Cuba	A/Habana/3469/2009
NA	CY123194	H1N1	09/29/2009	Singapore	A/Singapore/GP3869/2009
NA	KC781000	H1N1	09/29/2009	USA	A/Texas/66/2009
NA	KC780991	H1N1	09/29/2009	USA	A/Texas/66/2009
NA	CY058278	H1N1	09/29/2009	USA	A/Wisconsin/629-D01015/2009(H1N1)
NA	KC782075	H1N1	09/29/2009	USA	A/Pennsylvania/25/2009
NA	KC780898	H1N1	09/29/2009	USA	A/Alaska/39/2009
NA	CY072952	H1N1	09/29/2009	Nicaragua	A/Managua/5258.02/2009(H1N1)
NA	CY056293	H1N1	09/30/2009	Malaysia	A/Malaysia/15815/2009
NA	KF687926	H1N1	09/30/2009	Cyprus	A/Cyprus/418/2009
NA	CY066102	H1N1	09/30/2009	Japan	A/Hyogo/09K109/2009
NA	CY123200	H1N1	09/30/2009	Singapore	A/Singapore/GP3912/2009
NA	CY123789	H1N1	09/30/2009	Singapore	A/Singapore/ON2159/2009
NA	KC780071	H1N1	09/30/2009	USA	A/Oklahoma/04/2009
NA	KC780283	H1N1	09/30/2009	USA	A/South Dakota/11/2009
NA	KC780031	H1N1	09/30/2009	USA	A/Wisconsin/67/2009
NA	CY083441	H1N1	09/30/2009	USA	A/Ft.Benning/WRAIR1669P/2009
NA	HQ247669	H1N1	09/30/2009	Finland	A/Finland/636/2009
NA	KP019722	H1N1	09/30/2009	China	A/FuZhou/SWL11510/2009
NA	CY066152	H1N1	09/30/2009	Japan	A/Nagasaki/09N024/2009
NA	JX556122	H1N1	09/30/2009	Cuba	A/Habana/3528/2009
NA	KP317305	H1N1	09/30/2009	India	A/Chattisgarh/010/2009
NA	CY049055	H1N1	09/30/2009	Spain	A/Catalonia/S1453/2009(H1N1)
NA	JQ692309	H1N1	09/30/2009	India	A/Port Blair/PB_SF14/2009
NA	JX875008	H1N1	09/30/2009	USA	A/Kentucky/80/2009
NA	CY067724	H1N1	09/30/2009	United Ki	A/England/94280035/2009
NA	CY065922	H1N1	09/30/2009	Netherlan	A/Netherlands/2290/2009
NA	KF687927	H1N1	09/30/2009	Cyprus	A/Cyprus/421/2009
NA	KF687925	H1N1	09/30/2009	Cyprus	A/Cyprus/413/2009
NA	KT181541	H1N1	09/30/2009	Singapore	A/Singapore/DMS39/2009
NA	JN381379	H1N1	10/2009	Taiwan	A/Taiwan/99384/2009
NA	JN381374	H1N1	10/2009	Taiwan	A/Taiwan/90233/2009
NA	JN381375	H1N1	10/2009	Taiwan	A/Taiwan/99127/2009
NA	JN381373	H1N1	10/2009	Taiwan	A/Taiwan/90230/2009
NA	JN381377	H1N1	10/2009	Taiwan	A/Taiwan/99330/2009
NA	JN381381	H1N1	10/2009	Taiwan	A/Taiwan/99502/2009
NA	JN381380	H1N1	10/2009	Taiwan	A/Taiwan/99422/2009
NA	JN381376	H1N1	10/2009	Taiwan	A/Taiwan/99144/2009
NA	JN381378	H1N1	10/2009	Taiwan	A/Taiwan/99334/2009
NA	JN006473	H1N1	10/2009	China	A/Hangzhou/1693/2009
NA	JN006474	H1N1	10/2009	China	A/Hangzhou/1708/2009
NA	JN006475	H1N1	10/2009	China	A/Hangzhou/1709/2009

NA	JN006476	H1N1	10/2009	China	A/Hangzhou/1715/2009
NA	HM189326	H1N1	10/2009	Russia	A/Perm/CRIE-PMI/2009
NA	HQ247670	H1N1	10/2009	Finland	A/Finland/641/2009
NA	HM189499	H1N1	10/2009	South Ko	A/Korea/CJ109/2009(H1N1)
NA	HM189496	H1N1	10/2009	South Ko	A/Korea/CJ40/2009(H1N1)
NA	HM189497	H1N1	10/2009	South Ko	A/Korea/CJ63/2009(H1N1)
NA	HM189500	H1N1	10/2009	South Ko	A/Korea/CJ112/2009(H1N1)
NA	HQ247671	H1N1	10/2009	Finland	A/Finland/642/2009
NA	HM189498	H1N1	10/2009	South Ko	A/Korea/CJ68/2009(H1N1)
NA	GU265764	H1N1	10/2009	Germany	A/Berlin/113/2009(H1N1)
NA	GU265765	H1N1	10/2009	Germany	A/Berlin/45762/2009(H1N1)
NA	GU265766	H1N1	10/2009	Germany	A/Berlin/111/2009(H1N1)
NA	GU265767	H1N1	10/2009	Germany	A/Berlin/112/2009(H1N1)
NA	GU265768	H1N1	10/2009	Germany	A/Berlin/46030/2009(H1N1)
NA	GU265770	H1N1	10/2009	Germany	A/Berlin/114/2009(H1N1)
NA	GU265771	H1N1	10/2009	Germany	A/Berlin/46731/2009(H1N1)
NA	GU265761	H1N1	10/2009	Germany	A/Berlin/45623/2009(H1N1)
NA	GU265762	H1N1	10/2009	Germany	A/Berlin/109/2009(H1N1)
NA	GU265763	H1N1	10/2009	Germany	A/Berlin/110/2009(H1N1)
NA	GU265760	H1N1	10/2009	Germany	A/Berlin/45528/2009(H1N1)
NA	GU265759	H1N1	10/2009	Germany	A/Berlin/45527/2009(H1N1)
NA	CY048938	H1N1	10/2009	China	A/Beijing/337/2009(H1N1)
NA	CY048945	H1N1	10/2009	China	A/Beijing/342/2009(H1N1)
NA	CY048980	H1N1	10/2009	China	A/Beijing/370/2009
NA	CY052052	H1N1	10/2009	Belarus	A/Belarus/131/2009(H1N1)
NA	CY048976	H1N1	10/2009	China	A/Beijing/368/2009
NA	CY048953	H1N1	10/2009	China	A/Beijing/346/2009(H1N1)
NA	GU265769	H1N1	10/2009	Germany	A/Berlin/115/2009(H1N1)
NA	CY118269	H1N1	10/01/2009	Malaysia	A/Malaysia/2205822/2009
NA	CY069004	H1N1	10/01/2009	Guam	A/Guam/NHRC0015/2009(H1N1)
NA	JX625546	H1N1	10/01/2009	United Ki	A/Northern Ireland/94480417/2009
NA	CY128253	H1N1	10/01/2009	Viet Nam	A/Viet Nam/13032051/2009
NA	CY071716	H1N1	10/01/2009	Mexico	A/Mexico City/WR1695N/2009(H1N1)
NA	CY083417	H1N1	10/01/2009	USA	A/San Diego/WRAIR1666P/2009
NA	CY123791	H1N1	10/01/2009	Singapore	A/Singapore/ON2168/2009
NA	HQ239993	H1N1	10/01/2009	Canada	A/Canada-ON/RV2912/2009
NA	CY062212	H1N1	10/01/2009	USA	A/California/VRDL81/2009(H1N1)
NA	CY063221	H1N1	10/01/2009	USA	A/Wisconsin/629-D01351/2009(H1N1)
NA	CY062220	H1N1	10/01/2009	USA	A/California/VRDL82/2009(H1N1)
NA	CY062228	H1N1	10/01/2009	USA	A/California/VRDL83/2009(H1N1)
NA	KC780271	H1N1	10/01/2009	USA	A/Connecticut/14/2009
NA	KC782148	H1N1	10/01/2009	USA	A/Illinois/13/2009
NA	KC780965	H1N1	10/01/2009	USA	A/Alaska/40/2009
NA	KC782078	H1N1	10/01/2009	USA	A/Minnesota/14/2009
NA	KC780260	H1N1	10/01/2009	USA	A/Florida/21/2009
NA	CY068956	H1N1	10/01/2009	Guam	A/Guam/NHRC0009/2009(H1N1)
NA	CY063802	H1N1	10/01/2009	Singapore	A/Singapore/ON2172/2009
NA	CY056573	H1N1	10/01/2009	USA	A/New York/5755/2009(H1N1)
NA	CY062204	H1N1	10/01/2009	USA	A/California/VRDL80/2009(H1N1)
NA	CY062196	H1N1	10/01/2009	USA	A/California/VRDL79/2009(H1N1)
NA	KC781996	H1N1	10/01/2009	USA	A/North Carolina/45/2009
NA	KC780763	H1N1	10/02/2009	USA	A/Maryland/29/2009
NA	CY052361	H1N1	10/02/2009	United Ar	A/UAE/F06/2009(H1N1)
NA	KC780847	H1N1	10/02/2009	USA	A/Iowa/09/2009

NA	KT181542	H1N1	10/02/2009	Singapore	A/Singapore/DMS40/2009
NA	CY066074	H1N1	10/02/2009	Japan	A/Kyoto/09K015/2009
NA	CY057544	H1N1	10/02/2009	USA	A/Wisconsin/629-D00853/2009(H1N1)
NA	JX625507	H1N1	10/02/2009	United Ki	A/England/94200009/2009
NA	LC093026	H1N1	10/02/2009	Saudi Ara	A/Riyadh/1811/2009
NA	KC781366	H1N1	10/02/2009	USA	A/Nevada/16/2009
NA	CY083425	H1N1	10/02/2009	USA	A/San Diego/WRAIR1667P/2009
NA	CY057536	H1N1	10/02/2009	USA	A/Wisconsin/629-D00250/2009(H1N1)
NA	HQ239994	H1N1	10/02/2009	Canada	A/Canada-ON/RV2913/2009
NA	KC780140	H1N1	10/02/2009	USA	A/New Mexico/08/2009
NA	CY066080	H1N1	10/02/2009	Japan	A/Kyoto/09K046/2009
NA	CY057552	H1N1	10/03/2009	USA	A/Wisconsin/629-D02337/2009(H1N1)
NA	CY055433	H1N1	10/03/2009	USA	A/New York/4977/2009(H1N1)
NA	CY063687	H1N1	10/03/2009	China	A/Shandong-Rencheng/SWL312/2009(H1N
NA	CY063686	H1N1	10/03/2009	China	A/Shandong-Rencheng/SWL311/2009(H1N
NA	CY063688	H1N1	10/03/2009	China	A/Shandong-Rencheng/SWL313/2009(H1N
NA	CY056485	H1N1	10/04/2009	USA	A/New York/4995/2009(H1N1)
NA	CY057560	H1N1	10/04/2009	USA	A/Wisconsin/629-D00690/2009(H1N1)
NA	KC782121	H1N1	10/04/2009	USA	A/Alaska/37/2009
NA	KC780373	H1N1	10/04/2009	USA	A/New York/101/2009
NA	KC781328	H1N1	10/04/2009	USA	A/Texas/61/2009
NA	KP317300	H1N1	10/04/2009	India	A/Uttar Pradesh/005/2009
NA	CY123799	H1N1	10/05/2009	Singapore	A/Singapore/ON2180/2009
NA	CY056477	H1N1	10/05/2009	USA	A/New York/4990/2009(H1N1)
NA	JX625530	H1N1	10/05/2009	United Ki	A/Northern Ireland/94480397/2009
NA	HQ214279	H1N1	10/05/2009	Kenya	A/Kenya/0074/2009(H1N1)
NA	KJ690544	H1N1	10/05/2009	Uganda	A/Uganda/MUWRP-102/2009
NA	CY056445	H1N1	10/05/2009	USA	A/New York/4985/2009(H1N1)
NA	CY056453	H1N1	10/05/2009	USA	A/New York/4986/2009(H1N1)
NA	CY055441	H1N1	10/05/2009	USA	A/New York/4979/2009(H1N1)
NA	CY056429	H1N1	10/05/2009	USA	A/New York/4981/2009(H1N1)
NA	KC780641	H1N1	10/05/2009	USA	A/California/39/2009
NA	KC780175	H1N1	10/05/2009	USA	A/Minnesota/24/2009
NA	KC781374	H1N1	10/05/2009	USA	A/Oregon/35/2009
NA	CY057568	H1N1	10/05/2009	USA	A/Wisconsin/629-D00834/2009(H1N1)
NA	CY049705	H1N1	10/05/2009	Spain	A/Catalonia/S1478/2009(H1N1)
NA	CY049708	H1N1	10/05/2009	Spain	A/Catalonia/S1479/2009(H1N1)
NA	CY063685	H1N1	10/05/2009	China	A/Shandong-Weishan/SWL311/2009(H1N1
NA	LC093018	H1N1	10/05/2009	Saudi Ara	A/Riyadh/1880/2009
NA	LC093025	H1N1	10/05/2009	Saudi Ara	A/Riyadh/1829/2009
NA	CY063683	H1N1	10/05/2009	China	A/Shandong-Weishan/SWL36/2009(H1N1)
NA	CY063684	H1N1	10/05/2009	China	A/Shandong-Weishan/SWL39/2009(H1N1)
NA	KT181543	H1N1	10/05/2009	Singapore	A/Singapore/DMS41/2009
NA	AB603616	H1N1	10/05/2009	Lebanon	A/Lebanon/09L-04/2009
NA	HQ247668	H1N1	10/05/2009	Finland	A/Finland/635/2009
NA	CY066136	H1N1	10/05/2009	Japan	A/Nagasaki/09N012/2009
NA	HQ214305	H1N1	10/05/2009	Kenya	A/Kenya/0002/2009(H1N1)
NA	HQ214290	H1N1	10/06/2009	Kenya	A/Kenya/0005/2009(H1N1)
NA	CY057576	H1N1	10/06/2009	USA	A/Wisconsin/629-S1252/2009(H1N1)
NA	HM776032	H1N1	10/06/2009	South Kor	A/Seoul/Y-01/2009(H1N1)
NA	KY888157	H1N1	10/06/2009	India	A/Kerala/RGCBA923/2009
NA	CY056437	H1N1	10/06/2009	USA	A/New York/4984/2009(H1N1)
NA	KC780933	H1N1	10/06/2009	USA	A/Colorado/13/2009
NA	KC780928	H1N1	10/06/2009	USA	A/Colorado/13/2009

NA	KC782131	H1N1	10/06/2009	USA	A/Texas/77/2009
NA	KC780266	H1N1	10/06/2009	USA	A/Delaware/13/2009
NA	KC782092	H1N1	10/06/2009	USA	A/Washington/60/2009
NA	CY058286	H1N1	10/06/2009	USA	A/Wisconsin/629-D01102/2009(H1N1)
NA	CY056469	H1N1	10/06/2009	USA	A/New York/4988/2009(H1N1)
NA	CY056461	H1N1	10/06/2009	USA	A/New York/4987/2009(H1N1)
NA	CY052362	H1N1	10/06/2009	United Ar	A/UAE/G01/2009(H1N1)
NA	CY057192	H1N1	10/06/2009	Greece	A/Thessaloniki/1652/2009
NA	KP317301	H1N1	10/06/2009	India	A/Goa/006/2009
NA	JX465466	H1N1	10/06/2009	South Kor	A/Gyeonggi/4006/2009
NA	CY088661	H1N1	10/06/2009	India	A/Ngp/NIV14725/2009(H1N1)
NA	KM289090	H1N1	10/06/2009	Peru	A/Peru/2010731296/2009
NA	CY067723	H1N1	10/06/2009	United Ki	A/England/94280034/2009
NA	JN676131	H1N1	10/06/2009	Thailand	A/Thailand/Siriraj-3043/2009
NA	CY066104	H1N1	10/06/2009	Japan	A/Hyogo/09K112/2009
NA	CY083082	H1N1	10/06/2009	Colombia	A/Bogota/WRAIR0440T/2009
NA	CY083114	H1N1	10/07/2009	Ecuador	A/Quito/WRAIR0617N/2009
NA	HQ214306	H1N1	10/07/2009	Kenya	A/Kenya/0007/2009(H1N1)
NA	HQ214295	H1N1	10/07/2009	Kenya	A/Kenya/0008/2009(H1N1)
NA	CY058294	H1N1	10/07/2009	USA	A/Wisconsin/629-D02100/2009(H1N1)
NA	KC780894	H1N1	10/07/2009	USA	A/Idaho/13/2009
NA	KC780133	H1N1	10/07/2009	USA	A/Oregon/33/2009
NA	KC781030	H1N1	10/07/2009	USA	A/Wyoming/15/2009
NA	CY075494	H1N1	10/07/2009	USA	A/Boston/584/2009(H1N1)
NA	KC781050	H1N1	10/07/2009	USA	A/North Carolina/57/2009
NA	HQ239998	H1N1	10/07/2009	Canada	A/Canada-SK/RV2926/2009
NA	CY049711	H1N1	10/07/2009	Spain	A/Catalonia/S1490/2009(H1N1)
NA	KR870130	H1N1	10/07/2009	India	A/Bangalore/8330-09/2009
NA	CY083122	H1N1	10/07/2009	Ecuador	A/Quito/WRAIR0617T/2009(H1N1)
NA	CY128269	H1N1	10/08/2009	Viet Nam	A/Viet Nam/13032060/2009
NA	CY128261	H1N1	10/08/2009	Viet Nam	A/Viet Nam/13032059/2009
NA	CY063804	H1N1	10/08/2009	Singapore	A/Singapore/ON2193/2009
NA	KC781230	H1N1	10/08/2009	USA	A/South Carolina/39/2009
NA	KC780712	H1N1	10/08/2009	USA	A/Vermont/20/2009
NA	KC780439	H1N1	10/08/2009	USA	A/Tennessee/17/2009
NA	KC882399	H1N1	10/08/2009	USA	A/Texas/79/2009
NA	CY049714	H1N1	10/08/2009	Spain	A/Catalonia/S1501/2009(H1N1)
NA	KT181544	H1N1	10/08/2009	Singapore	A/Singapore/DMS42/2009
NA	JN187190	H1N1	10/08/2009	Taiwan	A/Taiwan/7418/2009
NA	HQ214280	H1N1	10/08/2009	Kenya	A/Kenya/0009/2009(H1N1)
NA	KC781684	H1N1	10/09/2009	USA	A/Hawaii/27/2009
NA	JX625538	H1N1	10/09/2009	United Ki	A/Northern Ireland/94480404/2009
NA	CY128277	H1N1	10/09/2009	Viet Nam	A/Viet Nam/13032061/2009
NA	JX625515	H1N1	10/09/2009	United Ki	A/England/1072/2009
NA	JX625608	H1N1	10/09/2009	United Ki	A/Northern Ireland/94940020/2009(H1N
NA	CY063069	H1N1	10/09/2009	USA	A/California/VRDL87/2009(H1N1)
NA	CY057584	H1N1	10/09/2009	USA	A/Wisconsin/629-D01749/2009(H1N1)
NA	CY057592	H1N1	10/09/2009	USA	A/Wisconsin/629-D02090/2009(H1N1)
NA	CY057600	H1N1	10/09/2009	USA	A/Wisconsin/629-D01080/2009(H1N1)
NA	KC781840	H1N1	10/09/2009	USA	A/Arkansas/07/2009
NA	KC781049	H1N1	10/09/2009	USA	A/Kentucky/24/2009
NA	KP637464	H1N1	10/09/2009	Thailand	A/Thailand/PY08063/2009
NA	CY062236	H1N1	10/09/2009	USA	A/California/VRDL84/2009(H1N1)
NA	CY063077	H1N1	10/09/2009	USA	A/California/VRDL88/2009(H1N1)

NA	CY062252	H1N1	10/09/2009	USA	A/California/VRDL86/2009(H1N1)
NA	CY062244	H1N1	10/09/2009	USA	A/California/VRDL85/2009(H1N1)
NA	CY071764	H1N1	10/09/2009	Mexico	A/Mexico City/WR1708T/2009(H1N1)
NA	HQ240023	H1N1	10/09/2009	Canada	A/Canada-ON/RV2962/2009
NA	HQ239999	H1N1	10/09/2009	Canada	A/Canada-SK/RV2927/2009
NA	CY123803	H1N1	10/09/2009	Singapore	A/Singapore/ON2195/2009
NA	HQ247682	H1N1	10/09/2009	Finland	A/Finland/654/2009
NA	HQ214302	H1N1	10/09/2009	Kenya	A/Kenya/0011/2009(H1N1)
NA	HQ214296	H1N1	10/09/2009	Kenya	A/Kenya/0012/2009(H1N1)
NA	KP317306	H1N1	10/10/2009	India	A/Madhya Pradesh/011/2009
NA	CY056368	H1N1	10/10/2009	Spain	A/Madrid/RC10/2009(H1N1)
NA	LC093008	H1N1	10/10/2009	Saudi Ara	A/Riyadh/2116/2009
NA	CY089189	H1N1	10/10/2009	USA	A/Boston/583/2009(H1N1)
NA	KC297151	H1N1	10/10/2009	Saudi Ara	A/Jeddah/1296/2009
NA	CY057608	H1N1	10/10/2009	USA	A/Wisconsin/629-D00557/2009(H1N1)
NA	KC782173	H1N1	10/10/2009	USA	A/Tennessee/18/2009
NA	KC780399	H1N1	10/10/2009	USA	A/New Mexico/06/2009
NA	KC780979	H1N1	10/10/2009	USA	A/Nebraska/20/2009
NA	KC782192	H1N1	10/10/2009	USA	A/Hawaii/28/2009
NA	KC297156	H1N1	10/10/2009	Saudi Ara	A/Jeddah/1365/2009
NA	HQ240024	H1N1	10/11/2009	Canada	A/Canada-ON/RV2963/2009
NA	CY095737	H1N1	10/11/2009	China	A/Hubei/100/2009
NA	CY095944	H1N1	10/11/2009	China	A/Zhejiang/84/2009
NA	CY095951	H1N1	10/11/2009	China	A/Zhejiang/85/2009
NA	CY096047	H1N1	10/11/2009	China	A/Hubei/99/2009
NA	CY095973	H1N1	10/11/2009	China	A/Zhejiang/88/2009
NA	CY095965	H1N1	10/11/2009	China	A/Zhejiang/87/2009
NA	CY095937	H1N1	10/11/2009	China	A/Zhejiang/83/2009
NA	CY123210	H1N1	10/11/2009	Singapore	A/Singapore/GP4000/2009
NA	CY083058	H1N1	10/11/2009	USA	A/Colorado/WRAIR0353S/2009
NA	CY072696	H1N1	10/11/2009	Nicaragua	A/Managua/3013.01/2009(H1N1)
NA	CY090059	H1N1	10/11/2009	Nicaragua	A/Managua/4606.07/2009
NA	KC782115	H1N1	10/11/2009	USA	A/Arkansas/08/2009
NA	CY095957	H1N1	10/11/2009	China	A/Zhejiang/86/2009
NA	CY055966	H1N1	10/12/2009	USA	A/San Diego/INS03/2009(H1N1)
NA	CY055974	H1N1	10/12/2009	USA	A/San Diego/INS04/2009(H1N1)
NA	CY055950	H1N1	10/12/2009	USA	A/San Diego/INS01/2009(H1N1)
NA	CY055982	H1N1	10/12/2009	USA	A/San Diego/INS05/2009(H1N1)
NA	KC781113	H1N1	10/12/2009	USA	A/Wisconsin/52/2009
NA	CY066529	H1N1	10/12/2009	USA	A/San Diego/INS193/2009(H1N1)
NA	KC781099	H1N1	10/12/2009	USA	A/Texas/65/2009
NA	KC780834	H1N1	10/12/2009	USA	A/Virginia/21/2009
NA	KC780289	H1N1	10/12/2009	USA	A/Pennsylvania/26/2009
NA	KC780766	H1N1	10/12/2009	USA	A/Washington/70/2009
NA	KC782089	H1N1	10/12/2009	USA	A/Texas/64/2009
NA	CY055990	H1N1	10/12/2009	USA	A/San Diego/INS06/2009(H1N1)
NA	CY055958	H1N1	10/12/2009	USA	A/San Diego/INS02/2009(H1N1)
NA	KC780954	H1N1	10/12/2009	USA	A/Wisconsin/60/2009
NA	KC781097	H1N1	10/12/2009	USA	A/Texas/63/2009
NA	CY056885	H1N1	10/12/2009	USA	A/San Diego/INS54/2009(H1N1)
NA	CY056877	H1N1	10/12/2009	USA	A/San Diego/INS51/2009(H1N1)
NA	CY100466	H1N1	10/12/2009	Mexico	A/Mexico City/INER2/2009
NA	KP637280	H1N1	10/12/2009	Thailand	A/Thailand/IY07093/2009
NA	KC261326	H1N1	10/12/2009	Mexico	A/Mexico City/INERPHAC1/2009(H1N1)

NA	CY056206	H1N1	10/12/2009	USA	A/San Diego/INS52/2009(H1N1)
NA	CY056214	H1N1	10/12/2009	USA	A/San Diego/INS53/2009(H1N1)
NA	CY057232	H1N1	10/12/2009	USA	A/New York/5045/2009(H1N1)
NA	CY096013	H1N1	10/12/2009	China	A/Zhejiang/94/2009
NA	CY096039	H1N1	10/12/2009	China	A/Zhejiang/98/2009
NA	CY096001	H1N1	10/12/2009	China	A/Zhejiang/92/2009
NA	CY096025	H1N1	10/12/2009	China	A/Zhejiang/96/2009
NA	CY096018	H1N1	10/12/2009	China	A/Zhejiang/95/2009
NA	CY096007	H1N1	10/12/2009	China	A/Zhejiang/93/2009
NA	CY096032	H1N1	10/12/2009	China	A/Zhejiang/97/2009
NA	CY095988	H1N1	10/12/2009	China	A/Zhejiang/90/2009
NA	CY123204	H1N1	10/12/2009	Singapore	A/Singapore/GP3991/2009
NA	KM289091	H1N1	10/12/2009	Peru	A/Peru/2010731297/2009
NA	KT181545	H1N1	10/12/2009	Singapore	A/Singapore/DMS43/2009
NA	CY095994	H1N1	10/12/2009	China	A/Zhejiang/91/2009
NA	HQ247702	H1N1	10/12/2009	Finland	A/Finland/674/2009
NA	CY083944	H1N1	10/12/2009	USA	A/San Diego/INS194/2009(H1N1)
NA	HQ247680	H1N1	10/12/2009	Finland	A/Finland/652/2009
NA	HQ214317	H1N1	10/12/2009	Kenya	A/Kenya/0015/2009(H1N1)
NA	HQ214283	H1N1	10/12/2009	Kenya	A/Kenya/0017/2009(H1N1)
NA	HM568074	H1N1	10/12/2009	United Ki	A/England/H094220579/2009
NA	HM568066	H1N1	10/12/2009	United Ki	A/England/H094240645/2009
NA	AB603617	H1N1	10/12/2009	Lebanon	A/Lebanon/09L-05/2009
NA	HQ214307	H1N1	10/12/2009	Kenya	A/Kenya/0016/2009(H1N1)
NA	CY067886	H1N1	10/12/2009	Portugal	A/Lisboa/78/2009(H1N1)
NA	CY057624	H1N1	10/12/2009	USA	A/Wisconsin/629-S1313/2009(H1N1)
NA	CY057616	H1N1	10/12/2009	USA	A/Wisconsin/629-S1309/2009(H1N1)
NA	CY063229	H1N1	10/12/2009	USA	A/Wisconsin/629-D00675/2009(H1N1)
NA	CY063237	H1N1	10/12/2009	USA	A/Wisconsin/629-D02442/2009(H1N1)
NA	CY063245	H1N1	10/12/2009	USA	A/Wisconsin/629-D00830/2009(H1N1)
NA	HQ240000	H1N1	10/12/2009	Canada	A/Canada-SK/RV2928/2009
NA	CY056493	H1N1	10/13/2009	USA	A/New York/5074/2009(H1N1)
NA	CY123214	H1N1	10/13/2009	Singapore	A/Singapore/GP4007/2009
NA	CY057320	H1N1	10/13/2009	USA	A/New York/5667/2009(H1N1)
NA	CY056501	H1N1	10/13/2009	USA	A/New York/5079/2009(H1N1)
NA	CY123805	H1N1	10/13/2009	Singapore	A/Singapore/ON2202/2009
NA	CY056525	H1N1	10/13/2009	USA	A/New York/5130/2009(H1N1)
NA	CY057336	H1N1	10/13/2009	USA	A/San Diego/INS08/2009(H1N1)
NA	CY056517	H1N1	10/13/2009	USA	A/New York/5086/2009(H1N1)
NA	CY055998	H1N1	10/13/2009	USA	A/San Diego/INS09/2009(H1N1)
NA	CY056006	H1N1	10/13/2009	USA	A/San Diego/INS10/2009(H1N1)
NA	HQ214271	H1N1	10/13/2009	Kenya	A/Kenya/0020/2009(H1N1)
NA	CY090075	H1N1	10/13/2009	Nicaragua	A/Managua/4076.03/2009
NA	CY083832	H1N1	10/13/2009	USA	A/San Diego/INS07/2009(H1N1)
NA	HQ247681	H1N1	10/13/2009	Finland	A/Finland/653/2009
NA	HQ214303	H1N1	10/13/2009	Kenya	A/Kenya/0018/2009(H1N1)
NA	HQ214297	H1N1	10/13/2009	Kenya	A/Kenya/0021/2009(H1N1)
NA	CY071612	H1N1	10/13/2009	USA	A/Ft Carson/WR1448P/2009(H1N1)
NA	CY049717	H1N1	10/13/2009	Spain	A/Catalonia/S1514/2009(H1N1)
NA	CY049720	H1N1	10/13/2009	Spain	A/Catalonia/S1515/2009(H1N1)
NA	CY049726	H1N1	10/13/2009	Spain	A/Catalonia/S1533/2009(H1N1)
NA	HQ240002	H1N1	10/13/2009	Canada	A/Canada-SK/RV2930/2009
NA	HQ240001	H1N1	10/13/2009	Canada	A/Canada-SK/RV2929/2009
NA	CY056509	H1N1	10/13/2009	USA	A/New York/5083/2009(H1N1)

NA	KC782072	H1N1	10/14/2009	USA	A/Washington/68/2009
NA	CY083497	H1N1	10/14/2009	USA	A/Chicago/WRAIR1691P/2009
NA	CY081512	H1N1	10/14/2009	Cambodia	A/Cambodia/NHRCC00004/2009(H1N1)
NA	CY081528	H1N1	10/14/2009	Cambodia	A/Cambodia/NHRCC00006/2009(H1N1)
NA	HQ247687	H1N1	10/14/2009	Finland	A/Finland/659/2009
NA	CY081544	H1N1	10/14/2009	Cambodia	A/Cambodia/NHRCC00008/2009(H1N1)
NA	CY056893	H1N1	10/14/2009	USA	A/San Diego/INS55/2009(H1N1)
NA	CY123220	H1N1	10/14/2009	Singapore	A/Singapore/GP4012/2009
NA	CY056022	H1N1	10/14/2009	USA	A/San Diego/INS12/2009(H1N1)
NA	KC780745	H1N1	10/14/2009	USA	A/Utah/32/2009
NA	KC780550	H1N1	10/14/2009	USA	A/Delaware/20/2009
NA	KC782082	H1N1	10/14/2009	USA	A/Kansas/24/2009
NA	KC780755	H1N1	10/14/2009	USA	A/Michigan/25/2009
NA	CY056014	H1N1	10/14/2009	USA	A/San Diego/INS11/2009(H1N1)
NA	AB576884	H1N1	10/14/2009	Japan	A/Hokkaido/256/2009(H1N1)
NA	CY050135	H1N1	10/14/2009	China	A/Qingdao/1424/2009(H1N1)
NA	CY067907	H1N1	10/14/2009	Portugal	A/Lisboa/85/2009(H1N1)
NA	CY067883	H1N1	10/14/2009	Portugal	A/Lisboa/77/2009(H1N1)
NA	CY067889	H1N1	10/14/2009	Portugal	A/Lisboa/79/2009(H1N1)
NA	CY090010	H1N1	10/14/2009	USA	A/Wisconsin/629-S1368/2009(H1N1)
NA	KJ958972	H1N1	10/14/2009	India	A/Mumbai/4066/2009
NA	CY057632	H1N1	10/14/2009	USA	A/Wisconsin/629-S1348/2009(H1N1)
NA	CY066106	H1N1	10/14/2009	Japan	A/Hyogo/09K115/2009
NA	CY066158	H1N1	10/14/2009	Japan	A/Nagasaki/09N036/2009
NA	CY081504	H1N1	10/14/2009	Cambodia	A/Cambodia/NHRCC00003/2009(H1N1)
NA	CY081536	H1N1	10/14/2009	Cambodia	A/Cambodia/NHRCC00007/2009(H1N1)
NA	CY081520	H1N1	10/14/2009	Cambodia	A/Cambodia/NHRCC00005/2009(H1N1)
NA	CY081488	H1N1	10/14/2009	Cambodia	A/Cambodia/NHRCC00001/2009(H1N1)
NA	HQ214291	H1N1	10/14/2009	Kenya	A/Kenya/0024/2009(H1N1)
NA	HQ214304	H1N1	10/14/2009	Kenya	A/Kenya/0023/2009(H1N1)
NA	AB603618	H1N1	10/14/2009	Lebanon	A/Lebanon/09L-06/2009
NA	CY083481	H1N1	10/14/2009	USA	A/Chicago/WRAIR1690P/2009
NA	CY066082	H1N1	10/14/2009	Japan	A/Kyoto/09K052/2009
NA	JN896301	H1N1	10/14/2009	Viet Nam	A/Hue/6/2009
NA	KT181546	H1N1	10/14/2009	Singapore	A/Singapore/DMS44/2009
NA	CY049723	H1N1	10/14/2009	Spain	A/Catalonia/S1525/2009(H1N1)
NA	CY063699	H1N1	10/14/2009	China	A/Shandong-Taishan/SWL4205/2009(H1N
NA	GU371269	H1N1	10/14/2009	Israel	A/Israel/6290/2009(H1N1)
NA	CY061237	H1N1	10/14/2009	USA	A/San Diego/INS102/2009(H1N1)
NA	HQ240029	H1N1	10/14/2009	Canada	A/Canada-ON/RV2983/2009
NA	HQ240026	H1N1	10/14/2009	Canada	A/Canada-ON/RV2965/2009
NA	CY061229	H1N1	10/14/2009	USA	A/San Diego/INS101/2009(H1N1)
NA	HQ240031	H1N1	10/14/2009	Canada	A/Canada-ON/RV2985/2009
NA	HQ240025	H1N1	10/14/2009	Canada	A/Canada-ON/RV2964/2009
NA	CY057640	H1N1	10/14/2009	USA	A/Wisconsin/629-S1350/2009(H1N1)
NA	CY067892	H1N1	10/14/2009	Portugal	A/Lisboa/80/2009(H1N1)
NA	CY067895	H1N1	10/14/2009	Portugal	A/Lisboa/81/2009(H1N1)
NA	CY067898	H1N1	10/14/2009	Portugal	A/Lisboa/82/2009(H1N1)
NA	CY067901	H1N1	10/14/2009	Portugal	A/Lisboa/83/2009(H1N1)
NA	CY067904	H1N1	10/14/2009	Portugal	A/Lisboa/84/2009(H1N1)
NA	CY050133	H1N1	10/14/2009	China	A/Qingdao/1418/2009(H1N1)
NA	CY050134	H1N1	10/14/2009	China	A/Qingdao/1422/2009(H1N1)
NA	CY081496	H1N1	10/14/2009	Cambodia	A/Cambodia/NHRCC00002/2009(H1N1)
NA	CY082986	H1N1	10/15/2009	Cambodia	A/Cambodia/NHRCC00009/2009

NA	CY080346	H1N1	10/15/2009	Cambodia	A/Cambodia/NHRCC00011/2009(H1N1)
NA	KC780247	H1N1	10/15/2009	USA	A/New York/100/2009
NA	CY056901	H1N1	10/15/2009	USA	A/San Diego/INS57/2009(H1N1)
NA	KC781146	H1N1	10/15/2009	USA	A/Washington/61/2009
NA	KC780344	H1N1	10/15/2009	USA	A/New York/102/2009
NA	JX625570	H1N1	10/15/2009	United Ki	A/Northern Ireland/1/2009
NA	CY107342	H1N1	10/15/2009	United Ki	A/Scotland/Dundee_09V500203/2009
NA	CY107335	H1N1	10/15/2009	United Ki	A/Scotland/Dundee_09V500199/2009
NA	CY066537	H1N1	10/15/2009	USA	A/San Diego/INS195/2009(H1N1)
NA	KC781968	H1N1	10/15/2009	USA	A/Mississippi/04/2009
NA	KC782057	H1N1	10/15/2009	USA	A/Arizona/17/2009
NA	KC780757	H1N1	10/15/2009	USA	A/Minnesota/17/2009
NA	CY056078	H1N1	10/15/2009	USA	A/District of Columbia/INS24/2009(H
NA	CY071388	H1N1	10/15/2009	Jordan	A/Amman/WR0060N/2009(H1N1)
NA	CY067880	H1N1	10/15/2009	Portugal	A/Lisboa/76/2009(H1N1)
NA	CY071604	H1N1	10/15/2009	USA	A/Ft Carson/WR1446P/2009(H1N1)
NA	CY092922	H1N1	10/15/2009	USA	A/Maryland/NHRC0002/2009
NA	HM072406	H1N1	10/15/2009	Taiwan	A/Taiwan/7855/2009
NA	KJ958934	H1N1	10/15/2009	India	A/Mumbai/4109/2009
NA	CY107321	H1N1	10/15/2009	United Ki	A/Scotland/Fife_20234/2009
NA	CY056370	H1N1	10/15/2009	Spain	A/Madrid/RC12/2009(H1N1)
NA	CY063253	H1N1	10/15/2009	USA	A/Wisconsin/629-D00692/2009(H1N1)
NA	HQ239980	H1N1	10/15/2009	Canada	A/Canada-ON/RV2887/2009
NA	CY052363	H1N1	10/15/2009	United Ar	A/UAE/G09/2009(H1N1)
NA	HQ239984	H1N1	10/15/2009	Canada	A/Canada-ON/RV2891/2009
NA	HQ240027	H1N1	10/15/2009	Canada	A/Canada-ON/RV2966/2009
NA	CY057240	H1N1	10/15/2009	USA	A/New York/5158/2009(H1N1)
NA	CY081552	H1N1	10/15/2009	Cambodia	A/Cambodia/NHRCC00010/2009(H1N1)
NA	CY066030	H1N1	10/16/2009	Japan	A/Fukushima/09FY002/2009
NA	CY123224	H1N1	10/16/2009	Singapore	A/Singapore/GP4049/2009
NA	KC780253	H1N1	10/16/2009	USA	A/North Carolina/49/2009
NA	CY056533	H1N1	10/16/2009	USA	A/New York/5141/2009(H1N1)
NA	CY056541	H1N1	10/16/2009	USA	A/New York/5150/2009(H1N1)
NA	AB603619	H1N1	10/16/2009	Lebanon	A/Lebanon/09L-07/2009
NA	KC781224	H1N1	10/16/2009	USA	A/New Hampshire/19/2009
NA	KC781680	H1N1	10/16/2009	USA	A/Arizona/18/2009
NA	KC781023	H1N1	10/16/2009	USA	A/North Carolina/43/2009
NA	KC780508	H1N1	10/16/2009	USA	A/North Carolina/46/2009
NA	HQ214272	H1N1	10/16/2009	Kenya	A/Kenya/0025/2009(H1N1)
NA	HQ214314	H1N1	10/16/2009	Kenya	A/Kenya/0026/2009(H1N1)
NA	CY073051	H1N1	10/16/2009	Dominican	A/Santo Domingo/WRAIR1414N/2009(H1N
NA	HQ247657	H1N1	10/16/2009	Finland	A/Finland/624/2009
NA	CY092930	H1N1	10/16/2009	USA	A/Maryland/NHRC0003/2009
NA	JN896303	H1N1	10/16/2009	Viet Nam	A/Hue/10/2009
NA	HQ240028	H1N1	10/16/2009	Canada	A/Canada-ON/RV2967/2009
NA	HQ239902	H1N1	10/16/2009	Canada	A/Canada-MB/RV2046-10/2009
NA	HQ240034	H1N1	10/16/2009	Canada	A/Canada-MB/RV3607/2009
NA	HQ240030	H1N1	10/16/2009	Canada	A/Canada-ON/RV2984/2009
NA	CY056645	H1N1	10/16/2009	USA	A/New York/6079/2009(H1N1)
NA	CY057248	H1N1	10/16/2009	USA	A/New York/5161/2009(H1N1)
NA	CY063261	H1N1	10/16/2009	USA	A/Wisconsin/629-D00740/2009(H1N1)
NA	KC782322	H1N1	10/16/2009	USA	A/North Carolina/46/2009
NA	KC781668	H1N1	10/17/2009	USA	A/Arkansas/06/2009
NA	KC781133	H1N1	10/17/2009	USA	A/Nevada/17/2009

NA	CY107356	H1N1	10/17/2009	United Ki	A/Scotland/Perth_09V500257/2009
NA	CY063109	H1N1	10/17/2009	USA	A/California/VRDL92/2009(H1N1)
NA	HM780478	H1N1	10/17/2009	China	A/Guangdong/0752/2009(H1N1)
NA	CY058040	H1N1	10/17/2009	USA	A/Texas/46172731/2009(H1N1)
NA	CY058048	H1N1	10/17/2009	USA	A/Texas/46172734/2009(H1N1)
NA	GU198203	H1N1	10/17/2009	China	A/Nanjing/3/2009(H1N1)
NA	CY107349	H1N1	10/17/2009	United Ki	A/Scotland/StAndrews_09V500252/2009
NA	CY107363	H1N1	10/17/2009	United Ki	A/Scotland/Perth_09V500259/2009
NA	CY063093	H1N1	10/17/2009	USA	A/California/VRDL90/2009(H1N1)
NA	CY067739	H1N1	10/17/2009	United Ki	A/England/94760028/2009
NA	CY063101	H1N1	10/17/2009	USA	A/California/VRDL91/2009(H1N1)
NA	CY063117	H1N1	10/17/2009	USA	A/California/VRDL93/2009(H1N1)
NA	CY056549	H1N1	10/17/2009	USA	A/New York/5173/2009(H1N1)
NA	CY063085	H1N1	10/17/2009	USA	A/California/VRDL89/2009(H1N1)
NA	HQ214315	H1N1	10/17/2009	Kenya	A/Kenya/0027/2009(H1N1)
NA	CY058056	H1N1	10/18/2009	USA	A/Texas/46181235/2009(H1N1)
NA	CY057648	H1N1	10/18/2009	USA	A/Wisconsin/629-D02342/2009(H1N1)
NA	CY058686	H1N1	10/18/2009	USA	A/Texas/46182018/2009(H1N1)
NA	CY057664	H1N1	10/18/2009	USA	A/Wisconsin/629-D00099/2009(H1N1)
NA	CY060568	H1N1	10/18/2009	Canada	A/Ontario/296008/2009(H1N1)
NA	CY057656	H1N1	10/18/2009	USA	A/Wisconsin/629-D00544/2009(H1N1)
NA	CY058064	H1N1	10/18/2009	USA	A/Texas/46181292/2009(H1N1)
NA	CY073043	H1N1	10/18/2009	Jordan	A/Amman/WRAIR1339N/2009(H1N1)
NA	CY071556	H1N1	10/18/2009	Jordan	A/Amman/WR1324N/2009(H1N1)
NA	CY075502	H1N1	10/18/2009	USA	A/Boston/591/2009(H1N1)
NA	KC781127	H1N1	10/18/2009	USA	A/North Carolina/53/2009
NA	KC780117	H1N1	10/18/2009	USA	A/New Mexico/07/2009
NA	CY128285	H1N1	10/18/2009	Viet Nam	A/Viet Nam/13032071/2009
NA	CY056062	H1N1	10/19/2009	USA	A/District of Columbia/INS20/2009(H
NA	CY056909	H1N1	10/19/2009	USA	A/San Diego/INS60/2009(H1N1)
NA	HQ214273	H1N1	10/19/2009	Kenya	A/Kenya/0029/2009(H1N1)
NA	KC780786	H1N1	10/19/2009	USA	A/Nevada/18/2009
NA	CY058710	H1N1	10/19/2009	USA	A/Texas/46193311/2009(H1N1)
NA	CY060757	H1N1	10/19/2009	USA	A/Texas/46193632/2009(H1N1)
NA	CY063806	H1N1	10/19/2009	Singapore	A/Singapore/ON2230/2009
NA	CY067744	H1N1	10/19/2009	United Ki	A/England/94780023/2009
NA	KJ690458	H1N1	10/19/2009	Uganda	A/Uganda/MUWRP-111/2009
NA	CY057672	H1N1	10/19/2009	USA	A/Wisconsin/629-D00132/2009(H1N1)
NA	CY071596	H1N1	10/19/2009	Turkey	A/Ankara/WR1429T/2009(H1N1)
NA	CY063269	H1N1	10/19/2009	USA	A/Wisconsin/629-D02082/2009(H1N1)
NA	CY083641	H1N1	10/19/2009	USA	A/San Diego/INS13/2009
NA	HQ214281	H1N1	10/19/2009	Kenya	A/Kenya/0030/2009(H1N1)
NA	HQ214308	H1N1	10/19/2009	Kenya	A/Kenya/0028/2009(H1N1)
NA	CY071644	H1N1	10/19/2009	Taiwan	A/Taipei/WR1489N/2009(H1N1)
NA	CY083840	H1N1	10/19/2009	USA	A/San Diego/INS14/2009(H1N1)
NA	CY049729	H1N1	10/19/2009	Spain	A/Catalonia/S1545/2009(H1N1)
NA	CY049731	H1N1	10/19/2009	Spain	A/Catalonia/S1547/2009(H1N1)
NA	CY063700	H1N1	10/19/2009	China	A/Shandong-Taian/SWL1311/2009(H1N1)
NA	CY058694	H1N1	10/19/2009	USA	A/Texas/461917783/2009(H1N1)
NA	KC780515	H1N1	10/19/2009	USA	A/Delaware/19/2009
NA	CY057256	H1N1	10/19/2009	USA	A/New York/5186/2009(H1N1)
NA	CY061245	H1N1	10/19/2009	USA	A/San Diego/INS103/2009(H1N1)
NA	AB603620	H1N1	10/19/2009	Lebanon	A/Lebanon/09L-08/2009
NA	CY060576	H1N1	10/19/2009	Canada	A/Ontario/29801/2009(H1N1)

NA	CY107496	H1N1	10/19/2009	United Ki	A/Scotland/Edinburgh_20203/2009
NA	CY107510	H1N1	10/19/2009	United Ki	A/Scotland/Edinburgh_20308/2009
NA	CY107370	H1N1	10/19/2009	United Ki	A/Scotland/Dundee_09V500266/2009
NA	CY107504	H1N1	10/19/2009	United Ki	A/Scotland/Livingston_20263/2009
NA	CY057680	H1N1	10/19/2009	USA	A/Wisconsin/629-S1384/2009(H1N1)
NA	CY057688	H1N1	10/19/2009	USA	A/Wisconsin/629-S1388/2009(H1N1)
NA	JX403980	H1N1	10/19/2009	China	A/Liaoning/1/2009(H1N1)
NA	CY058702	H1N1	10/19/2009	USA	A/Texas/46192760/2009(H1N1)
NA	CY049734	H1N1	10/20/2009	Spain	A/Catalonia/S1554/2009(H1N1)
NA	KC781069	H1N1	10/20/2009	USA	A/Minnesota/23/2009
NA	CY061253	H1N1	10/20/2009	USA	A/San Diego/INS104/2009(H1N1)
NA	CY064459	H1N1	10/20/2009	China	A/Guangdong/5024/2009(H1N1)
NA	CY060765	H1N1	10/20/2009	USA	A/Texas/46201823/2009(H1N1)
NA	CY056371	H1N1	10/20/2009	Spain	A/Madrid/RC13/2009(H1N1)
NA	CY056557	H1N1	10/20/2009	USA	A/New York/5199/2009(H1N1)
NA	CY056565	H1N1	10/20/2009	USA	A/New York/5227/2009(H1N1)
NA	HQ247705	H1N1	10/20/2009	Finland	A/Finland/678/2009
NA	CY057280	H1N1	10/20/2009	USA	A/New York/5221/2009(H1N1)
NA	CY073074	H1N1	10/20/2009	Turkey	A/Ankara/WRAIR1426N/2009(H1N1)
NA	CY056861	H1N1	10/20/2009	USA	A/San Diego/INS15/2009(H1N1)
NA	HQ247686	H1N1	10/20/2009	Finland	A/Finland/658/2009
NA	CY107377	H1N1	10/20/2009	United Ki	A/Scotland/Dundee_09V500321/2009
NA	KR870132	H1N1	10/20/2009	India	A/Bangalore/8975-19/2009
NA	KR870131	H1N1	10/20/2009	India	A/Bangalore/8973-04/2009
NA	AB603622	H1N1	10/20/2009	Lebanon	A/Lebanon/09L-10/2009
NA	AB603621	H1N1	10/20/2009	Lebanon	A/Lebanon/09L-09/2009
NA	HQ247684	H1N1	10/20/2009	Finland	A/Finland/656/2009
NA	HQ247683	H1N1	10/20/2009	Finland	A/Finland/655/2009
NA	HM568050	H1N1	10/20/2009	United Ki	A/England/950/2009
NA	CY066160	H1N1	10/20/2009	Japan	A/Nagasaki/09N040/2009
NA	GU562468	H1N1	10/20/2009	China	A/Guangdong/12012/2009
NA	CY123226	H1N1	10/20/2009	Singapore	A/Singapore/GP4078/2009
NA	CY107515	H1N1	10/20/2009	United Ki	A/Scotland/Edinburgh_20342/2009
NA	CY107522	H1N1	10/20/2009	United Ki	A/Scotland/Edinburgh_20665/2009
NA	CY123811	H1N1	10/20/2009	Singapore	A/Singapore/ON2224/2009
NA	JX625593	H1N1	10/20/2009	United Ki	A/Northern Ireland/94620068/2009(H1N
NA	JX625624	H1N1	10/20/2009	United Ki	A/Northern Ireland/94940033/2009(H1N
NA	JX625522	H1N1	10/20/2009	United Ki	A/England/94400003/2009
NA	CY056030	H1N1	10/20/2009	USA	A/San Diego/INS16/2009(H1N1)
NA	CY057272	H1N1	10/20/2009	USA	A/New York/5217/2009(H1N1)
NA	CY057264	H1N1	10/20/2009	USA	A/New York/5196/2009(H1N1)
NA	LC093029	H1N1	10/20/2009	Saudi Ara	A/Riyadh/2591/2009
NA	CY049743	H1N1	10/21/2009	Spain	A/Catalonia/S1592/2009(H1N1)
NA	CY056134	H1N1	10/21/2009	USA	A/San Diego/INS34/2009(H1N1)
NA	CY060773	H1N1	10/21/2009	USA	A/Texas/46214103/2009(H1N1)
NA	HQ240020	H1N1	10/21/2009	Canada	A/Canada-BC/RV2949/2009
NA	HQ240006	H1N1	10/21/2009	Canada	A/Canada-BC/RV2935/2009
NA	HQ240003	H1N1	10/21/2009	Canada	A/Canada-BC/RV2931/2009
NA	CY049740	H1N1	10/21/2009	Spain	A/Catalonia/S1589/2009(H1N1)
NA	CY063808	H1N1	10/21/2009	Singapore	A/Singapore/ON2241/2009
NA	KC782154	H1N1	10/21/2009	USA	A/New Mexico/10/2009
NA	CY073082	H1N1	10/21/2009	Turkey	A/Ankara/WRAIR1428T/2009(H1N1)
NA	AB603623	H1N1	10/21/2009	Lebanon	A/Lebanon/09L-11/2009
NA	HQ214298	H1N1	10/21/2009	Kenya	A/Kenya/0075/2009(H1N1)

NA	HQ214285	H1N1	10/21/2009	Kenya	A/Kenya/0033/2009(H1N1)
NA	HQ247685	H1N1	10/21/2009	Finland	A/Finland/657/2009
NA	HQ247688	H1N1	10/21/2009	Finland	A/Finland/660/2009
NA	HQ214282	H1N1	10/21/2009	Kenya	A/Kenya/0031/2009(H1N1)
NA	HQ214284	H1N1	10/21/2009	Kenya	A/Kenya/0032/2009(H1N1)
NA	HQ214299	H1N1	10/21/2009	Kenya	A/Kenya/0034/2009(H1N1)
NA	HM567978	H1N1	10/21/2009	United Ki	A/England/1111/2009
NA	HM568098	H1N1	10/21/2009	United Ki	A/England/766/2009
NA	CY071628	H1N1	10/21/2009	Taiwan	A/Taipei/WR1472T/2009(H1N1)
NA	CY071636	H1N1	10/21/2009	Taiwan	A/Taipei/WR1485T/2009(H1N1)
NA	CY071652	H1N1	10/21/2009	Ecuador	A/Quito/WR1589N/2009(H1N1)
NA	JN656969	H1N1	10/21/2009	Viet Nam	A/Hue/15/2009
NA	HM568058	H1N1	10/21/2009	United Ki	A/England/1116/2009
NA	CY083671	H1N1	10/21/2009	USA	A/San Diego/INS62/2009(H1N1)
NA	CY066088	H1N1	10/21/2009	Japan	A/Kyoto/09K063/2009
NA	HM568090	H1N1	10/21/2009	United Ki	A/England/H094380096/2009
NA	KC780938	H1N1	10/21/2009	USA	A/Louisiana/09/2009
NA	CY056917	H1N1	10/21/2009	USA	A/San Diego/INS61/2009(H1N1)
NA	KC780916	H1N1	10/21/2009	USA	A/New Mexico/12/2009
NA	CY057696	H1N1	10/21/2009	USA	A/Wisconsin/629-S1398/2009(H1N1)
NA	CY057704	H1N1	10/21/2009	USA	A/Wisconsin/629-S1397/2009(H1N1)
NA	JX625616	H1N1	10/21/2009	United Ki	A/Northern Ireland/94940023/2009(H1N
NA	KC780785	H1N1	10/21/2009	USA	A/South Dakota/13/2009
NA	CY057296	H1N1	10/21/2009	USA	A/New York/5276/2009(H1N1)
NA	KC780119	H1N1	10/21/2009	USA	A/Pennsylvania/27/2009
NA	CY056094	H1N1	10/21/2009	USA	A/District of Columbia/INS27/2009(H
NA	CY066545	H1N1	10/21/2009	USA	A/San Diego/INS197/2009(H1N1)
NA	CY066553	H1N1	10/21/2009	USA	A/San Diego/INS198/2009(H1N1)
NA	KC780884	H1N1	10/21/2009	USA	A/North Carolina/38/2009
NA	KC781002	H1N1	10/21/2009	USA	A/North Carolina/38/2009
NA	CY060805	H1N1	10/22/2009	USA	A/Texas/46223582/2009(H1N1)
NA	CY060797	H1N1	10/22/2009	USA	A/Texas/46223444/2009(H1N1)
NA	CY060781	H1N1	10/22/2009	USA	A/Texas/46221665/2009(H1N1)
NA	CY067740	H1N1	10/22/2009	United Ki	A/England/94780010/2009
NA	KC780681	H1N1	10/22/2009	USA	A/Ohio/16/2009
NA	AB742517	H1N1	10/22/2009	India	A/Patna-India/RMRI-VIRO-PMCH006/2009
NA	CY053412	H1N1	10/22/2009	United Ar	A/UAE/A01/2009(H1N1)
NA	CY060813	H1N1	10/22/2009	USA	A/Texas/46224042/2009(H1N1)
NA	GU571154	H1N1	10/22/2009	USA	A/Bethesda/NIH106-D0/2009(H1N1)
NA	CY066108	H1N1	10/22/2009	Japan	A/Hyogo/09K116/2009
NA	CY066032	H1N1	10/22/2009	Japan	A/Fukushima/09FY004/2009
NA	CY049737	H1N1	10/22/2009	Spain	A/Catalonia/S1587/2009(H1N1)
NA	HQ247712	H1N1	10/22/2009	Finland	A/Finland/685/2009
NA	HQ214301	H1N1	10/22/2009	Kenya	A/Kenya/0039/2009(H1N1)
NA	CY066561	H1N1	10/22/2009	USA	A/San Diego/INS199/2009(H1N1)
NA	KC780823	H1N1	10/22/2009	USA	A/New Mexico/13/2009
NA	HQ214292	H1N1	10/22/2009	Kenya	A/Kenya/0037/2009(H1N1)
NA	CY065989	H1N1	10/22/2009	Mongolia	A/Ulaanbaatar/6525/2009(H1N1)
NA	CY057712	H1N1	10/22/2009	USA	A/Wisconsin/629-S1407/2009(H1N1)
NA	CY057288	H1N1	10/22/2009	USA	A/New York/5271/2009(H1N1)
NA	HQ214300	H1N1	10/22/2009	Kenya	A/Kenya/0038/2009(H1N1)
NA	HQ214309	H1N1	10/22/2009	Kenya	A/Kenya/0035/2009(H1N1)
NA	HM568114	H1N1	10/22/2009	United Ki	A/England/798/2009
NA	CY057304	H1N1	10/22/2009	USA	A/New York/5297/2009(H1N1)

NA	KP019723	H1N1	10/22/2009	China	A/FuZhou/SWL11704/2009
NA	CY071460	H1N1	10/22/2009	Mexico	A/Mexico City/WR1090N/2009(H1N1)
NA	CY067748	H1N1	10/22/2009	United Ki	A/England/94780031/2009
NA	CY089197	H1N1	10/22/2009	USA	A/Boston/593/2009(H1N1)
NA	CY089205	H1N1	10/22/2009	USA	A/Boston/594/2009(H1N1)
NA	CY060789	H1N1	10/22/2009	USA	A/Texas/46222134/2009(H1N1)
NA	CY056373	H1N1	10/23/2009	Spain	A/Madrid/RC6/2009(H1N1)
NA	CY092938	H1N1	10/23/2009	USA	A/Maryland/NHRC0004/2009
NA	HQ214293	H1N1	10/23/2009	Kenya	A/Kenya/0046/2009(H1N1)
NA	HQ214275	H1N1	10/23/2009	Kenya	A/Kenya/0045/2009(H1N1)
NA	HQ214312	H1N1	10/23/2009	Kenya	A/Kenya/0048/2009(H1N1)
NA	HQ214310	H1N1	10/23/2009	Kenya	A/Kenya/0043/2009(H1N1)
NA	HQ214274	H1N1	10/23/2009	Kenya	A/Kenya/0044/2009(H1N1)
NA	AB603625	H1N1	10/23/2009	Lebanon	A/Lebanon/09L-13/2009
NA	KC780553	H1N1	10/23/2009	USA	A/West Virginia/12/2009
NA	CY056102	H1N1	10/23/2009	USA	A/District of Columbia/INS28/2009(H
NA	CY057312	H1N1	10/23/2009	USA	A/New York/5447/2009(H1N1)
NA	CY128293	H1N1	10/23/2009	Viet Nam	A/Viet Nam/13032074/2009
NA	AB603624	H1N1	10/23/2009	Lebanon	A/Lebanon/09L-12/2009
NA	KC781976	H1N1	10/23/2009	USA	A/New Mexico/14/2009
NA	CY065866	H1N1	10/23/2009	Netherlan	A/Netherlands/1493b/2009
NA	HQ214313	H1N1	10/23/2009	Kenya	A/Kenya/0013/2009(H1N1)
NA	CY058302	H1N1	10/23/2009	USA	A/Wisconsin/629-D02018/2009(H1N1)
NA	CY060821	H1N1	10/23/2009	USA	A/Texas/46233104/2009(H1N1)
NA	CY071620	H1N1	10/23/2009	USA	A/Ft Carson/WR1449P/2009(H1N1)
NA	CY128301	H1N1	10/24/2009	Viet Nam	A/Viet Nam/13032076/2009
NA	GU571152	H1N1	10/24/2009	USA	A/Bethesda/NIH107-D0/2009(H1N1)
NA	CY060829	H1N1	10/24/2009	USA	A/Texas/46240925/2009(H1N1)
NA	CY060584	H1N1	10/24/2009	Canada	A/Ontario/304434/2009(H1N1)
NA	HM189336	H1N1	10/24/2009	Russia	A/Moscow/CRIE-BVA/2009
NA	HQ121413	H1N1	10/24/2009	Spain	A/Madrid/1377206/2009
NA	CY075510	H1N1	10/24/2009	USA	A/Boston/595/2009(H1N1)
NA	CY060837	H1N1	10/24/2009	USA	A/Texas/46241654/2009(H1N1)
NA	CY092914	H1N1	10/25/2009	USA	A/Maryland/NHRC0001/2009
NA	HQ240032	H1N1	10/25/2009	Canada	A/Canada-NFL/RV3019/2009
NA	CY090018	H1N1	10/25/2009	USA	A/Wisconsin/629-D02330/2009(H1N1)
NA	AB603630	H1N1	10/26/2009	Lebanon	A/Lebanon/09L-18/2009
NA	HQ247691	H1N1	10/26/2009	Finland	A/Finland/663/2009
NA	HM568106	H1N1	10/26/2009	United Ki	A/England/797/2009
NA	KP019724	H1N1	10/26/2009	China	A/FuZhou/SWL11724/2009
NA	JQ612501	H1N1	10/26/2009	Hungary	A/Hungary/40/2009
NA	CY056925	H1N1	10/26/2009	USA	A/San Diego/INS63/2009(H1N1)
NA	KC780687	H1N1	10/26/2009	USA	A/Rhode Island/19/2009
NA	KC780982	H1N1	10/26/2009	USA	A/Iowa/10/2009
NA	CY056182	H1N1	10/26/2009	USA	A/District of Columbia/INS43/2009(H
NA	AB603626	H1N1	10/26/2009	Lebanon	A/Lebanon/09L-14/2009
NA	CY123819	H1N1	10/26/2009	Singapore	A/Singapore/ON2250/2009
NA	AB649910	H1N1	10/26/2009	Japan	A/Toyama/316/2009
NA	CY056110	H1N1	10/26/2009	USA	A/District of Columbia/INS29/2009(H
NA	CY056126	H1N1	10/26/2009	USA	A/District of Columbia/INS33/2009(H
NA	KC780968	H1N1	10/26/2009	USA	A/New Mexico/11/2009
NA	CY056086	H1N1	10/26/2009	USA	A/District of Columbia/INS25/2009(H
NA	KC780052	H1N1	10/26/2009	USA	A/Washington/75/2009
NA	HQ256653	H1N1	10/26/2009	Myanmar	A/Myanmar/M535/2009(H1N1)

NA	CY058326	H1N1	10/26/2009	USA	A/Wisconsin/629-S1416/2009(H1N1)
NA	CY058318	H1N1	10/26/2009	USA	A/Wisconsin/629-S1414/2009(H1N1)
NA	CY058310	H1N1	10/26/2009	USA	A/Wisconsin/629-S1417/2009(H1N1)
NA	HQ247689	H1N1	10/26/2009	Finland	A/Finland/661/2009
NA	HQ247690	H1N1	10/26/2009	Finland	A/Finland/662/2009
NA	HQ247695	H1N1	10/26/2009	Finland	A/Finland/667/2009
NA	HQ214286	H1N1	10/26/2009	Kenya	A/Kenya/0052/2009(H1N1)
NA	HQ214316	H1N1	10/26/2009	Kenya	A/Kenya/0055/2009(H1N1)
NA	HQ214287	H1N1	10/26/2009	Kenya	A/Kenya/0053/2009(H1N1)
NA	AB603629	H1N1	10/26/2009	Lebanon	A/Lebanon/09L-17/2009
NA	CY066084	H1N1	10/26/2009	Japan	A/Kyoto/09K053/2009
NA	CY066110	H1N1	10/26/2009	Japan	A/Hyogo/09K119/2009
NA	CY066154	H1N1	10/26/2009	Japan	A/Nagasaki/09N028/2009
NA	AB603628	H1N1	10/26/2009	Lebanon	A/Lebanon/09L-16/2009
NA	AB603627	H1N1	10/26/2009	Lebanon	A/Lebanon/09L-15/2009
NA	KR870133	H1N1	10/26/2009	India	A/Bangalore/9274-06/2009
NA	CY075486	H1N1	10/26/2009	Russia	A/Belgorod/RII4/2009(H1N1)
NA	CY050302	H1N1	10/26/2009	Spain	A/Catalonia/S1604/2009(H1N1)
NA	CY050305	H1N1	10/26/2009	Spain	A/Catalonia/S1605/2009(H1N1)
NA	CY050308	H1N1	10/26/2009	Spain	A/Catalonia/S1606/2009(H1N1)
NA	CY050299	H1N1	10/26/2009	Spain	A/Catalonia/S1600/2009(H1N1)
NA	CY050311	H1N1	10/26/2009	Spain	A/Catalonia/S1620/2009(H1N1)
NA	CY067725	H1N1	10/26/2009	United Ki	A/England/94600034/2009
NA	CY056038	H1N1	10/26/2009	USA	A/District of Columbia/INS17/2009(H
NA	CY064766	H1N1	10/26/2009	USA	A/District of Columbia/INS32/2009(H
NA	JX875036	H1N1	10/26/2009	USA	A/Kentucky/96/2009
NA	HQ256651	H1N1	10/26/2009	Myanmar	A/Myanmar/M534/2009(H1N1)
NA	CY075008	H1N1	10/26/2009	Thailand	A/Thailand/CU-H847/2009
NA	CY056142	H1N1	10/26/2009	USA	A/San Diego/INS35/2009(H1N1)
NA	HQ247692	H1N1	10/27/2009	Finland	A/Finland/664/2009
NA	CY060592	H1N1	10/27/2009	Canada	A/Ontario/305139/2009(H1N1)
NA	HQ247693	H1N1	10/27/2009	Finland	A/Finland/665/2009
NA	HQ247694	H1N1	10/27/2009	Finland	A/Finland/666/2009
NA	CY067637	H1N1	10/27/2009	Norway	A/Oslo/INS219/2009(H1N1)
NA	AB603634	H1N1	10/27/2009	Lebanon	A/Lebanon/09L-22/2009
NA	CY123232	H1N1	10/27/2009	Singapore	A/Singapore/GP4148/2009
NA	CY056118	H1N1	10/27/2009	USA	A/District of Columbia/INS31/2009(H
NA	AB537490	H1N1	10/27/2009	Japan	A/Nagasaki/HA-58/2009(H1N1)
NA	KC781078	H1N1	10/27/2009	USA	A/Maryland/30/2009
NA	KC780718	H1N1	10/27/2009	USA	A/Rhode Island/18/2009
NA	HM780484	H1N1	10/27/2009	China	A/Guangdong/0862/2009(H1N1)
NA	CY067743	H1N1	10/27/2009	United Ki	A/England/94780022/2009
NA	CY058638	H1N1	10/27/2009	USA	A/Wisconsin/629-S1425/2009(H1N1)
NA	CY058630	H1N1	10/27/2009	USA	A/Wisconsin/629-S1431/2009(H1N1)
NA	CY058622	H1N1	10/27/2009	USA	A/Wisconsin/629-S1433/2009(H1N1)
NA	HQ214311	H1N1	10/27/2009	Kenya	A/Kenya/0056/2009(H1N1)
NA	HQ214288	H1N1	10/27/2009	Kenya	A/Kenya/0057/2009(H1N1)
NA	KT181547	H1N1	10/27/2009	Singapore	A/Singapore/DMS46/2009
NA	AB603633	H1N1	10/27/2009	Lebanon	A/Lebanon/09L-21/2009
NA	AB603635	H1N1	10/27/2009	Lebanon	A/Lebanon/09L-23/2009
NA	AB603631	H1N1	10/27/2009	Lebanon	A/Lebanon/09L-19/2009
NA	AB603632	H1N1	10/27/2009	Lebanon	A/Lebanon/09L-20/2009
NA	CY067727	H1N1	10/27/2009	United Ki	A/England/94600039/2009
NA	CY056054	H1N1	10/27/2009	USA	A/District of Columbia/INS19/2009(H

NA	CY060600	H1N1	10/27/2009	Canada	A/Ontario/305598/2009(H1N1)
NA	CY052364	H1N1	10/27/2009	United A	A/UAE/H01/2009(H1N1)
NA	JN187191	H1N1	10/27/2009	Taiwan	A/Taiwan/6200/2009
NA	HQ247706	H1N1	10/28/2009	Finland	A/Finland/679/2009
NA	HM568082	H1N1	10/28/2009	United Ki	A/England/1213/2009
NA	KC782275	H1N1	10/28/2009	USA	A/Hawaii/34/2009
NA	JX625554	H1N1	10/28/2009	United Ki	A/England/1397/2009
NA	AB603637	H1N1	10/28/2009	Lebanon	A/Lebanon/09L-25/2009
NA	CY055323	H1N1	10/28/2009	Italy	A/Rome/623/2009(H1N1)
NA	CY055325	H1N1	10/28/2009	Italy	A/Rome/624/2009(H1N1)
NA	CY055328	H1N1	10/28/2009	Italy	A/Rome/625/2009(H1N1)
NA	CY055330	H1N1	10/28/2009	Italy	A/Rome/626/2009(H1N1)
NA	KC782162	H1N1	10/28/2009	USA	A/Hawaii/29/2009
NA	CY060608	H1N1	10/28/2009	Canada	A/Ontario/308054/2009(H1N1)
NA	CY066817	H1N1	10/28/2009	USA	A/San Diego/INS218/2009
NA	KY925343	H1N1	10/28/2009	Brazil	A/Porto Alegre/LACENRS-3434/2009
NA	CY071428	H1N1	10/28/2009	Turkmenis	A/Ashgabat/WR0865N/2009(H1N1)
NA	HQ214289	H1N1	10/28/2009	Kenya	A/Kenya/0061/2009(H1N1)
NA	AB603636	H1N1	10/28/2009	Lebanon	A/Lebanon/09L-24/2009
NA	JN187192	H1N1	10/28/2009	Taiwan	A/Taiwan/7873/2009
NA	AB603638	H1N1	10/28/2009	Lebanon	A/Lebanon/09L-26/2009
NA	KU310645	H1N1	10/28/2009	India	A/Assam/2257/2009
NA	CY050323	H1N1	10/28/2009	Spain	A/Catalonia/S1650/2009(H1N1)
NA	CY050314	H1N1	10/28/2009	Spain	A/Catalonia/S1632/2009(H1N1)
NA	CY050317	H1N1	10/28/2009	Spain	A/Catalonia/S1637/2009(H1N1)
NA	CY050320	H1N1	10/28/2009	Spain	A/Catalonia/S1641/2009(H1N1)
NA	CY056046	H1N1	10/28/2009	USA	A/District of Columbia/INS18/2009(H
NA	CY056933	H1N1	10/28/2009	USA	A/San Diego/INS65/2009(H1N1)
NA	CY066825	H1N1	10/28/2009	Belgium	A/Antwerp/INS221/2009(H1N1)
NA	KP019725	H1N1	10/28/2009	China	A/FuZhou/SWL11782/2009
NA	CY067747	H1N1	10/29/2009	United Ki	A/England/94780029/2009
NA	CY063141	H1N1	10/29/2009	USA	A/California/VRDL98/2009(H1N1)
NA	AB603639	H1N1	10/29/2009	Lebanon	A/Lebanon/09L-27/2009
NA	CY055332	H1N1	10/29/2009	Italy	A/Rome/627/2009(H1N1)
NA	CY055335	H1N1	10/29/2009	Italy	A/Rome/628/2009(H1N1)
NA	KC780960	H1N1	10/29/2009	USA	A/Utah/58/2009
NA	CY075518	H1N1	10/29/2009	USA	A/Boston/602/2009(H1N1)
NA	HQ256655	H1N1	10/29/2009	Myanmar	A/Myanmar/M536/2009(H1N1)
NA	CY067746	H1N1	10/29/2009	United Ki	A/England/94780026/2009
NA	CY083649	H1N1	10/29/2009	USA	A/District of Columbia/INS22/2009(H
NA	AB704464	H1N1	10/29/2009	Japan	A/Tochigi/350/2009
NA	KU310644	H1N1	10/29/2009	India	A/Assam/2264/2009
NA	MF196264	H1N1	10/29/2009	Iran	A/Tehran/88-8-30-1/2009
NA	CY075489	H1N1	10/29/2009	Russia	A/Saint-Petersburg/RII96/2009(H1N1)
NA	CY050326	H1N1	10/29/2009	Spain	A/Catalonia/S1651/2009(H1N1)
NA	CY067730	H1N1	10/29/2009	United Ki	A/England/94640079/2009
NA	CY060616	H1N1	10/29/2009	Canada	A/Ontario/309862/2009(H1N1)
NA	CY055337	H1N1	10/29/2009	Italy	A/Rome/629/2009(H1N1)
NA	CY061269	H1N1	10/29/2009	Belgium	A/Brussels/INS106/2009(H1N1)
NA	JX875016	H1N1	10/29/2009	USA	A/Kentucky/99/2009
NA	CY066193	H1N1	10/29/2009	USA	A/California/VRDL94/2009(H1N1)
NA	CY063125	H1N1	10/29/2009	USA	A/California/VRDL96/2009(H1N1)
NA	CY063133	H1N1	10/29/2009	USA	A/California/VRDL97/2009(H1N1)
NA	CY128309	H1N1	10/29/2009	Viet Nam	A/Viet Nam/13032079/2009

NA	CY056375	H1N1	10/30/2009	Spain	A/Madrid/RC7/2009(H1N1)
NA	KC781333	H1N1	10/30/2009	USA	A/Kentucky/25/2009
NA	CY056941	H1N1	10/30/2009	Belgium	A/Brussels/INS71/2009(H1N1)
NA	CY107529	H1N1	10/30/2009	United Ki	A/Scotland/Edinburgh_21027/2009
NA	JN207838	H1N1	10/30/2009	China	A/Zhejiang/89w/2009(H1N1)
NA	JQ173105	H1N1	10/30/2009	China	A/Nanjing/NJU-108/2009
NA	CY060624	H1N1	10/30/2009	Canada	A/Ontario/313762/2009(H1N1)
NA	HQ214294	H1N1	10/30/2009	Kenya	A/Kenya/0066/2009(H1N1)
NA	CY067728	H1N1	10/30/2009	United Ki	A/England/94640077/2009
NA	KC781081	H1N1	10/30/2009	USA	A/District Of Columbia/10/2009
NA	CY056070	H1N1	10/30/2009	USA	A/District of Columbia/INS21/2009(H
NA	KM985443	H1N1	10/30/2009	India	A/Thrissur/MCVR1400/2009
NA	CY066729	H1N1	10/30/2009	Belgium	A/Brussels/INS206/2009(H1N1)
NA	HM072407	H1N1	10/30/2009	Taiwan	A/Taiwan/7878/2009
NA	KM985444	H1N1	10/30/2009	India	A/Thrissur/MCVR1402/2009
NA	CY083848	H1N1	10/30/2009	USA	A/District of Columbia/INS23/2009(H
NA	HQ214277	H1N1	10/30/2009	Kenya	A/Kenya/0064/2009(H1N1)
NA	HQ214278	H1N1	10/30/2009	Kenya	A/Kenya/0065/2009(H1N1)
NA	HQ214276	H1N1	10/30/2009	Kenya	A/Kenya/0063/2009(H1N1)
NA	CY066034	H1N1	10/30/2009	Japan	A/Fukushima/09FY007/2009
NA	CY083755	H1N1	10/30/2009	Belgium	A/Brussels/INS205/2009(H1N1)
NA	CY128317	H1N1	10/31/2009	Viet Nam	A/Viet Nam/13032088/2009
NA	CY058654	H1N1	10/31/2009	USA	A/Wisconsin/629-D02002/2009(H1N1)
NA	CY058662	H1N1	10/31/2009	USA	A/Wisconsin/629-D01913/2009(H1N1)
NA	CY058646	H1N1	10/31/2009	USA	A/Wisconsin/629-D02329/2009(H1N1)
NA	CY065810	H1N1	10/31/2009	Netherlar	A/Netherlands/1715b/2009
NA	CY073120	H1N1	10/31/2009	Russia	A/Moscow/WRAIR1632N/2009(H1N1)
NA	CY052053	H1N1	11/2009	Belarus	A/Belarus/216/2009(H1N1)
NA	CY052055	H1N1	11/2009	Belarus	A/Belarus/220/2009(H1N1)
NA	CY053350	H1N1	11/2009	China	A/Beijing/720/2009(H1N1)
NA	CY053344	H1N1	11/2009	China	A/Beijing/719/2009(H1N1)
NA	CY052063	H1N1	11/2009	Belarus	A/Belarus/360/2009(H1N1)
NA	CY053340	H1N1	11/2009	China	A/Beijing/718/2009(H1N1)
NA	CY060458	H1N1	11/2009	South Kor	A/Seoul/1785/2009(H1N1)
NA	CY095893	H1N1	11/2009	China	A/Zhejiang/78/2009
NA	CY095872	H1N1	11/2009	China	A/Hubei/75/2009
NA	JN006478	H1N1	11/2009	China	A/Hangzhou/1718/2009
NA	CY095856	H1N1	11/2009	China	A/Hubei/73/2009
NA	JN006477	H1N1	11/2009	China	A/Hangzhou/1717/2009
NA	JN006480	H1N1	11/2009	China	A/Hangzhou/1724/2009
NA	JN006479	H1N1	11/2009	China	A/Hangzhou/1719/2009
NA	HM189328	H1N1	11/2009	Russia	A/Ivanovo/CRIE-AMV/2009
NA	HM189346	H1N1	11/2009	Russia	A/Moscow oblast/CRIE-DEI/2009
NA	JN381389	H1N1	11/2009	Taiwan	A/Taiwan/97138/2009
NA	CY095880	H1N1	11/2009	China	A/Hubei/76/2009
NA	CY095864	H1N1	11/2009	China	A/Hubei/74/2009
NA	CY095922	H1N1	11/2009	China	A/Zhejiang/81/2009
NA	CY095929	H1N1	11/2009	China	A/Zhejiang/82/2009
NA	CY095887	H1N1	11/2009	China	A/Zhejiang/77/2009
NA	HM189332	H1N1	11/2009	Russia	A/Kaluga/CRIE-INV/2009
NA	CY095914	H1N1	11/2009	China	A/Zhejiang/80/2009
NA	JN381386	H1N1	11/2009	Taiwan	A/Taiwan/99867/2009
NA	JN381385	H1N1	11/2009	Taiwan	A/Taiwan/99840/2009
NA	JN381388	H1N1	11/2009	Taiwan	A/Taiwan/97080/2009

NA	JN381383	H1N1	11/2009	Taiwan	A/Taiwan/99666/2009
NA	JN381384	H1N1	11/2009	Taiwan	A/Taiwan/99721/2009
NA	JN381387	H1N1	11/2009	Taiwan	A/Taiwan/99902/2009
NA	JN381382	H1N1	11/2009	Taiwan	A/Taiwan/99542/2009
NA	CY054640	H1N1	11/01/2009	Russia	A/Russia/12/2009(H1N1)
NA	CY054656	H1N1	11/01/2009	Russia	A/Russia/61/2009(H1N1)
NA	CY054664	H1N1	11/01/2009	Russia	A/Russia/100/2009(H1N1)
NA	CY053738	H1N1	11/01/2009	Russia	A/Russia/178/2009(H1N1)
NA	CY053746	H1N1	11/01/2009	Russia	A/Russia/190/2009(H1N1)
NA	CY053754	H1N1	11/01/2009	Russia	A/Russia/200/2009(H1N1)
NA	CY053730	H1N1	11/01/2009	Russia	A/Russia/74/2009(H1N1)
NA	KC781769	H1N1	11/01/2009	USA	A/Nevada/19/2009
NA	KC780910	H1N1	11/01/2009	USA	A/New York/104/2009
NA	KC780177	H1N1	11/01/2009	USA	A/Texas/69/2009
NA	KC780653	H1N1	11/01/2009	USA	A/Utah/52/2009
NA	CY053691	H1N1	11/01/2009	Russia	A/Russia/191/2009(H1N1)
NA	CY053683	H1N1	11/01/2009	Russia	A/Russia/14/2009(H1N1)
NA	CY107701	H1N1	11/01/2009	United Ki	A/Scotland/Glasgow_443726/2009
NA	CY107734	H1N1	11/01/2009	United Ki	A/Scotland/Glasgow_445236/2009
NA	CY107707	H1N1	11/01/2009	United Ki	A/Scotland/Glasgow_443734/2009
NA	CY107729	H1N1	11/01/2009	United Ki	A/Scotland/Glasgow_444809/2009
NA	CY107722	H1N1	11/01/2009	United Ki	A/Scotland/Glasgow_444590/2009
NA	JX625578	H1N1	11/01/2009	United Ki	A/England/94580011/2009
NA	CY054632	H1N1	11/01/2009	Russia	A/Russia/4/2009(H1N1)
NA	CY107714	H1N1	11/01/2009	United Ki	A/Scotland/Glasgow_444125/2009
NA	KM213297	H1N1	11/01/2009	Oman	A/Oman/SQUH-6/2009
NA	KT181548	H1N1	11/01/2009	Singapore	A/Singapore/DMS47/2009
NA	CY053418	H1N1	11/01/2009	Russia	A/Russia/149/2009(H1N1)
NA	CY054648	H1N1	11/01/2009	Russia	A/Russia/19/2009(H1N1)
NA	HM625647	H1N1	11/01/2009	Italy	A/Rome/PTV1/2009
NA	HM625648	H1N1	11/01/2009	Italy	A/Rome/PTV2/2009
NA	MF196265	H1N1	11/01/2009	Iran	A/Tehran/88-8-30-2/2009
NA	CY058670	H1N1	11/01/2009	USA	A/Wisconsin/629-D00193/2009(H1N1)
NA	KC780953	H1N1	11/02/2009	USA	A/Delaware/14/2009
NA	KC781772	H1N1	11/02/2009	USA	A/Hawaii/30/2009
NA	CY056621	H1N1	11/02/2009	USA	A/New York/5988/2009(H1N1)
NA	CY058270	H1N1	11/02/2009	USA	A/New York/5620/2009(H1N1)
NA	CY066569	H1N1	11/02/2009	USA	A/San Diego/INS201/2009(H1N1)
NA	CY067726	H1N1	11/02/2009	United Ki	A/England/94600038/2009
NA	JF714144	H1N1	11/02/2009	South Kor	A/Korea/NAP-1/2009(H1N1)
NA	CY123240	H1N1	11/02/2009	Singapore	A/Singapore/GP4218/2009
NA	HM189314	H1N1	11/02/2009	Russia	A/Chita/CRIE-8/2009
NA	CY107391	H1N1	11/02/2009	United Ki	A/Scotland/StAndrews_09V500630/2009
NA	KC781128	H1N1	11/02/2009	USA	A/Alabama/04/2009
NA	CY063277	H1N1	11/02/2009	USA	A/Wisconsin/629-D00888/2009(H1N1)
NA	HQ247726	H1N1	11/02/2009	Finland	A/Finland/700/2009
NA	CY083663	H1N1	11/02/2009	USA	A/San Diego/INS41/2009(H1N1)
NA	CY066156	H1N1	11/02/2009	Japan	A/Nagasaki/09N033/2009
NA	CY075526	H1N1	11/02/2009	USA	A/Boston/606/2009(H1N1)
NA	CY075534	H1N1	11/02/2009	USA	A/Boston/612/2009(H1N1)
NA	CY066881	H1N1	11/02/2009	USA	A/District of Columbia/INS229/2009(I
NA	HM189345	H1N1	11/02/2009	Russia	A/Moscow oblast/CRIE-OAM/2009
NA	CY060648	H1N1	11/02/2009	Canada	A/Ontario/314603/2009(H1N1)
NA	CY107384	H1N1	11/02/2009	United Ki	A/Scotland/Dundee_09V500624/2009

NA	KC780859	H1N1	11/02/2009	USA	A/Wyoming/16/2009
NA	CY060672	H1N1	11/02/2009	Canada	A/Ontario/315047/2009(H1N1)
NA	CY060632	H1N1	11/02/2009	Canada	A/Ontario/314095/2009(H1N1)
NA	CY071660	H1N1	11/02/2009	Russia	A/Moscow/WR1638T/2009(H1N1)
NA	CY073058	H1N1	11/02/2009	Turkey	A/Ankara/WRAIR1424T/2009(H1N1)
NA	CY067745	H1N1	11/02/2009	United Ki	A/England/94780024/2009
NA	CY087948	H1N1	11/02/2009	Thailand	A/Thailand/CU-H874/2009
NA	HM625649	H1N1	11/02/2009	Italy	A/Rome/PTV3/2009
NA	CY051944	H1N1	11/02/2009	Spain	A/Catalonia/S1676/2009(H1N1)
NA	CY051953	H1N1	11/02/2009	Spain	A/Catalonia/S1693/2009(H1N1)
NA	CY051959	H1N1	11/02/2009	Spain	A/Catalonia/S1702/2009(H1N1)
NA	CY051935	H1N1	11/02/2009	Spain	A/Catalonia/S1671/2009(H1N1)
NA	CY051938	H1N1	11/02/2009	Spain	A/Catalonia/S1672/2009(H1N1)
NA	CY051941	H1N1	11/02/2009	Spain	A/Catalonia/S1674/2009(H1N1)
NA	CY075476	H1N1	11/02/2009	Russia	A/Saint-Petersburg/RII48/2009(H1N1)
NA	CY051956	H1N1	11/02/2009	Spain	A/Catalonia/S1698/2009(H1N1)
NA	CY051932	H1N1	11/02/2009	Spain	A/Catalonia/S1668/2009(H1N1)
NA	CY060845	H1N1	11/02/2009	USA	A/Texas/JMS356/2009(H1N1)
NA	CY056382	H1N1	11/02/2009	Spain	A/Madrid/RC3/2009(H1N1)
NA	CY058678	H1N1	11/02/2009	USA	A/Wisconsin/629-D00938/2009(H1N1)
NA	HQ240033	H1N1	11/02/2009	Canada	A/Canada-PQ/RV3189/2009
NA	KC781269	H1N1	11/03/2009	USA	A/Vermont/23/2009
NA	CY100474	H1N1	11/03/2009	Mexico	A/Mexico City/INER12/2009
NA	CY056190	H1N1	11/03/2009	USA	A/District of Columbia/INS46/2009(H
NA	KC780805	H1N1	11/03/2009	USA	A/Kansas/26/2009
NA	AB603640	H1N1	11/03/2009	Lebanon	A/Lebanon/09L-28/2009
NA	CY056581	H1N1	11/03/2009	USA	A/New York/5782/2009(H1N1)
NA	CY056222	H1N1	11/03/2009	USA	A/San Diego/INS68/2009(H1N1)
NA	GU931806	H1N1	11/03/2009	Hong Kon	A/Hong Kong/415742M/2009(H1N1)
NA	HM100231	H1N1	11/03/2009	Hong Kon	A/Hong Kong/415742Md/2009(H1N1)
NA	HM072409	H1N1	11/03/2009	Taiwan	A/Taiwan/2202/2009
NA	CY083250	H1N1	11/03/2009	Russia	A/Moscow/WRAIR1627T/2009(H1N1)
NA	CY071780	H1N1	11/03/2009	Mexico	A/Mexico City/WR1762T/2009(H1N1)
NA	CY066833	H1N1	11/03/2009	Spain	A/Madrid/INS222/2009
NA	CY075654	H1N1	11/03/2009	USA	A/Boston/617/2009(H1N1)
NA	CY066737	H1N1	11/03/2009	Belgium	A/Brussels/INS208/2009(H1N1)
NA	CY056869	H1N1	11/03/2009	USA	A/District of Columbia/INS44/2009(H
NA	CY060688	H1N1	11/03/2009	Canada	A/Ontario/315181/2009(H1N1)
NA	CY060696	H1N1	11/03/2009	Canada	A/Ontario/315187/2009(H1N1)
NA	CY060704	H1N1	11/03/2009	Canada	A/Ontario/315613/2009(H1N1)
NA	CY062645	H1N1	11/03/2009	USA	A/District of Columbia/INS45/2009(H
NA	CY060656	H1N1	11/03/2009	Canada	A/Ontario/315003/2009(H1N1)
NA	CY089213	H1N1	11/03/2009	USA	A/Boston/618/2009
NA	CY063812	H1N1	11/03/2009	Singapore	A/Singapore/ON2271/2009
NA	KJ690528	H1N1	11/03/2009	Uganda	A/Uganda/MUWRP-137/2009
NA	CY051947	H1N1	11/03/2009	Spain	A/Catalonia/S1686/2009(H1N1)
NA	CY051950	H1N1	11/03/2009	Spain	A/Catalonia/S1687/2009(H1N1)
NA	CY075478	H1N1	11/03/2009	Russia	A/Saint-Petersburg/RII35/2009(H1N1)
NA	CY117629	H1N1	11/03/2009	Mexico	A/Mexico/UASLP-021/2009
NA	CY117636	H1N1	11/03/2009	Mexico	A/Mexico/UASLP-022/2009
NA	CY056377	H1N1	11/03/2009	Spain	A/Madrid/RC8/2009(H1N1)
NA	CY056379	H1N1	11/03/2009	Spain	A/Madrid/RC9/2009(H1N1)
NA	CY060640	H1N1	11/03/2009	Canada	A/Ontario/314137/2009(H1N1)
NA	KC781578	H1N1	11/04/2009	USA	A/Washington/65/2009

NA	CY056174	H1N1	11/04/2009	USA	A/San Diego/INS42/2009(H1N1)
NA	CY056629	H1N1	11/04/2009	USA	A/New York/6018/2009(H1N1)
NA	CY123244	H1N1	11/04/2009	Singapore	A/Singapore/GP4228/2009
NA	CY056198	H1N1	11/04/2009	USA	A/San Diego/INS48/2009(H1N1)
NA	CY066585	H1N1	11/04/2009	USA	A/San Diego/INS203/2009(H1N1)
NA	KC780470	H1N1	11/04/2009	USA	A/Florida/25/2009
NA	CY056158	H1N1	11/04/2009	USA	A/Pensacola/INS38/2009(H1N1)
NA	KC780731	H1N1	11/04/2009	USA	A/South Carolina/41/2009
NA	KM213298	H1N1	11/04/2009	Oman	A/Oman/SQUH-7/2009
NA	JX625585	H1N1	11/04/2009	United Ki	A/England/94620043/2009
NA	CY066577	H1N1	11/04/2009	USA	A/San Diego/INS202/2009(H1N1)
NA	CY060853	H1N1	11/04/2009	USA	A/Texas/JMS358/2009(H1N1)
NA	CY066036	H1N1	11/04/2009	Japan	A/Fukushima/09FY009/2009
NA	KT181549	H1N1	11/04/2009	Singapore	A/Singapore/DMS48/2009
NA	CY062685	H1N1	11/04/2009	USA	A/District of Columbia/INS114/2009(I
NA	CY056150	H1N1	11/04/2009	USA	A/Pensacola/INS37/2009(H1N1)
NA	CY060680	H1N1	11/04/2009	Canada	A/Ontario/315107/2009(H1N1)
NA	CY060664	H1N1	11/04/2009	Canada	A/Ontario/315015/2009(H1N1)
NA	CY061261	H1N1	11/04/2009	USA	A/San Diego/INS105/2009(H1N1)
NA	CY073098	H1N1	11/04/2009	Turkey	A/Ankara/WRAIR1440T/2009(H1N1)
NA	CY066745	H1N1	11/04/2009	Belgium	A/Brussels/INS209/2009(H1N1)
NA	CY067731	H1N1	11/04/2009	United Ki	A/England/94640080/2009
NA	HM625651	H1N1	11/04/2009	Italy	A/Rome/PTV5/2009
NA	HM625650	H1N1	11/04/2009	Italy	A/Rome/PTV4/2009
NA	HM625652	H1N1	11/04/2009	Italy	A/Rome/PTV6/2009
NA	CY051928	H1N1	11/04/2009	Spain	A/Catalonia/NS5408/2009(H1N1)
NA	CY060712	H1N1	11/04/2009	Canada	A/Ontario/315637/2009(H1N1)
NA	CY056381	H1N1	11/04/2009	Spain	A/Madrid/RC2/2009(H1N1)
NA	JX875052	H1N1	11/04/2009	USA	A/Kentucky/110/2009
NA	JX875044	H1N1	11/04/2009	USA	A/Kentucky/104/2009
NA	KC781084	H1N1	11/05/2009	USA	A/Minnesota/18/2009
NA	KC781990	H1N1	11/05/2009	USA	A/Iowa/17/2009
NA	CY054680	H1N1	11/05/2009	Russia	A/Russia/180/2009(H1N1)
NA	CY066897	H1N1	11/05/2009	USA	A/District of Columbia/INS231/2009
NA	CY056238	H1N1	11/05/2009	USA	A/San Diego/INS70/2009(H1N1)
NA	CY066593	H1N1	11/05/2009	USA	A/San Diego/INS204/2009(H1N1)
NA	CY056230	H1N1	11/05/2009	USA	A/San Diego/INS69/2009(H1N1)
NA	KC780479	H1N1	11/05/2009	USA	A/Montana/19/2009
NA	HQ247724	H1N1	11/05/2009	Finland	A/Finland/698/2009
NA	CY107398	H1N1	11/05/2009	United Ki	A/Scotland/Dundee_09V500795/2009
NA	CY107405	H1N1	11/05/2009	United Ki	A/Scotland/Dundee_09V500800/2009
NA	CY128437	H1N1	11/05/2009	Viet Nam	A/Viet Nam/001-1424/2009
NA	CY067729	H1N1	11/05/2009	United Ki	A/England/94640078/2009
NA	CY057744	H1N1	11/05/2009	USA	A/Wisconsin/629-D00151/2009(H1N1)
NA	CY063157	H1N1	11/05/2009	USA	A/California/VRDL100/2009(H1N1)
NA	CY063173	H1N1	11/05/2009	USA	A/California/VRDL102/2009(H1N1)
NA	CY057760	H1N1	11/05/2009	USA	A/Wisconsin/629-D02370/2009(H1N1)
NA	CY057768	H1N1	11/05/2009	USA	A/Wisconsin/629-D01346/2009(H1N1)
NA	CY057752	H1N1	11/05/2009	USA	A/Wisconsin/629-D01570/2009(H1N1)
NA	CY083856	H1N1	11/05/2009	USA	A/Pensacola/INS39/2009(H1N1)
NA	CY083872	H1N1	11/05/2009	USA	A/San Diego/INS49/2009(H1N1)
NA	HQ247725	H1N1	11/05/2009	Finland	A/Finland/699/2009
NA	HM072408	H1N1	11/05/2009	Taiwan	A/Taiwan/7336/2009
NA	CY075662	H1N1	11/05/2009	USA	A/Boston/628/2009(H1N1)

NA	AB603641	H1N1	11/05/2009	Lebanon	A/Lebanon/09L-29/2009
NA	CY063149	H1N1	11/05/2009	USA	A/California/VRDL99/2009(H1N1)
NA	CY063165	H1N1	11/05/2009	USA	A/California/VRDL101/2009(H1N1)
NA	CY057720	H1N1	11/05/2009	USA	A/Wisconsin/629-D02317/2009(H1N1)
NA	KC780475	H1N1	11/05/2009	USA	A/North Dakota/15/2009
NA	CY066018	H1N1	11/05/2009	Japan	A/Niigata/09F010/2009
NA	CY083723	H1N1	11/05/2009	USA	A/District of Columbia/INS115/2009(I
NA	GU571155	H1N1	11/05/2009	USA	A/Bethesda/NIH106-D14/2009(H1N1)
NA	CY057736	H1N1	11/05/2009	USA	A/Wisconsin/629-D01140/2009(H1N1)
NA	CY057728	H1N1	11/05/2009	USA	A/Wisconsin/629-D01915/2009(H1N1)
NA	CY066201	H1N1	11/05/2009	USA	A/California/VRDL103/2009(H1N1)
NA	CY117643	H1N1	11/05/2009	Mexico	A/Mexico/UASLP-023/2009
NA	CY065818	H1N1	11/05/2009	Netherlar	A/Netherlands/2442b/2009
NA	CY052365	H1N1	11/05/2009	United Ar	A/UAE/H05/2009(H1N1)
NA	KC780802	H1N1	11/06/2009	USA	A/District Of Columbia/13/2009
NA	KC780750	H1N1	11/06/2009	USA	A/Illinois/15/2009
NA	KC781633	H1N1	11/06/2009	USA	A/Texas/72/2009
NA	HQ247701	H1N1	11/06/2009	Finland	A/Finland/673/2009
NA	CY067738	H1N1	11/06/2009	United Ki	A/England/94760027/2009
NA	CY066086	H1N1	11/06/2009	Japan	A/Kyoto/09K059/2009
NA	CY063810	H1N1	11/06/2009	Singapore	A/Singapore/ON2269/2009
NA	CY056597	H1N1	11/06/2009	USA	A/New York/5916/2009(H1N1)
NA	CY083864	H1N1	11/06/2009	USA	A/District of Columbia/INS47/2009(H1
NA	KT181550	H1N1	11/06/2009	Singapore	A/Singapore/DMS49/2009
NA	KC261327	H1N1	11/06/2009	Mexico	A/Mexico City/INERPHAC2/2009(H1N1)
NA	CY073035	H1N1	11/06/2009	El Salvac	A/San Salvador/WRAIR1109N/2009(H1N1)
NA	CY063764	H1N1	11/06/2009	Singapore	A/Singapore/GP4247/2009(H1N1)
NA	LC093012	H1N1	11/06/2009	Saudi Ara	A/Riyadh/3728/2009
NA	HM625653	H1N1	11/06/2009	Italy	A/Rome/PTV7/2009
NA	KP317307	H1N1	11/06/2009	India	A/Tamil Naidu/012/2009
NA	CY117651	H1N1	11/06/2009	Mexico	A/Mexico/UASLP-024/2009
NA	HM051349	H1N1	11/06/2009	China	A/Sichuan-Wenjiang/SWL456/2009
NA	KU310646	H1N1	11/06/2009	India	A/Assam/2283/2009
NA	MF196263	H1N1	11/06/2009	Iran	A/Tehran/88-8-135/2009
NA	HQ247723	H1N1	11/06/2009	Finland	A/Finland/697/2009
NA	CY067734	H1N1	11/07/2009	United Ki	A/England/94740137/2009
NA	CY060869	H1N1	11/07/2009	USA	A/Texas/JMS361/2009(H1N1)
NA	HM625654	H1N1	11/07/2009	Italy	A/Rome/PTV8/2009
NA	CY128046	H1N1	11/07/2009	Viet Nam	A/Viet Nam/15032002/2009
NA	CY063701	H1N1	11/07/2009	China	A/Shandong-Taishan/SWL1406/2009(H1N
NA	CY060877	H1N1	11/07/2009	USA	A/Texas/JMS362/2009(H1N1)
NA	KC780194	H1N1	11/07/2009	USA	A/North Carolina/59/2009
NA	KC780817	H1N1	11/07/2009	USA	A/Nebraska/22/2009
NA	KC781938	H1N1	11/07/2009	USA	A/Michigan/26/2009
NA	CY065834	H1N1	11/07/2009	Netherlar	A/Netherlands/2457b/2009
NA	CY067717	H1N1	11/07/2009	United Ki	A/England/00380009/2009
NA	CY089221	H1N1	11/07/2009	USA	A/Boston/630/2009
NA	CY089229	H1N1	11/07/2009	USA	A/Boston/632/2009
NA	KC782170	H1N1	11/07/2009	USA	A/New Mexico/16/2009
NA	KC781005	H1N1	11/07/2009	USA	A/Massachusetts/32/2009
NA	CY087202	H1N1	11/07/2009	USA	A/Texas/JMS360/2009
NA	CY057776	H1N1	11/07/2009	USA	A/Wisconsin/629-D00802/2009(H1N1)
NA	CY060861	H1N1	11/07/2009	USA	A/Texas/JMS359/2009(H1N1)
NA	CY063702	H1N1	11/07/2009	China	A/Shandong-Taishan/SWL1436/2009(H1N

NA	CY057792	H1N1	11/08/2009	USA	A/Wisconsin/629-D01369/2009(H1N1)
NA	CY060720	H1N1	11/08/2009	Canada	A/Ontario/320266/2009(H1N1)
NA	CY061556	H1N1	11/08/2009	USA	A/Texas/JMS366/2009(H1N1)
NA	CY057800	H1N1	11/08/2009	USA	A/Wisconsin/629-D01466/2009(H1N1)
NA	CY057808	H1N1	11/08/2009	USA	A/Wisconsin/629-D02392/2009(H1N1)
NA	KC782187	H1N1	11/08/2009	USA	A/New Mexico/15/2009
NA	KC782151	H1N1	11/08/2009	USA	A/New Mexico/09/2009
NA	KC780649	H1N1	11/08/2009	USA	A/Connecticut/15/2009
NA	KC782061	H1N1	11/08/2009	USA	A/Wisconsin/55/2009
NA	HQ247721	H1N1	11/08/2009	Finland	A/Finland/695/2009
NA	CY089253	H1N1	11/08/2009	USA	A/Boston/637/2009
NA	KC781592	H1N1	11/08/2009	USA	A/Arizona/19/2009
NA	CY056653	H1N1	11/08/2009	USA	A/New York/6110/2009(H1N1)
NA	CY057784	H1N1	11/08/2009	USA	A/Wisconsin/629-D01014/2009(H1N1)
NA	CY060901	H1N1	11/08/2009	USA	A/Texas/JMS365/2009(H1N1)
NA	CY060885	H1N1	11/08/2009	USA	A/Texas/JMS363/2009(H1N1)
NA	CY060893	H1N1	11/08/2009	USA	A/Texas/JMS364/2009(H1N1)
NA	CY051980	H1N1	11/09/2009	Spain	A/Catalonia/S1760/2009(H1N1)
NA	CY067733	H1N1	11/09/2009	United Ki	A/England/94740054/2009
NA	CY051962	H1N1	11/09/2009	Spain	A/Catalonia/S1744/2009(H1N1)
NA	CY051965	H1N1	11/09/2009	Spain	A/Catalonia/S1748/2009(H1N1)
NA	CY051968	H1N1	11/09/2009	Spain	A/Catalonia/S1751/2009(H1N1)
NA	CY051983	H1N1	11/09/2009	Spain	A/Catalonia/S1761/2009(H1N1)
NA	CY051971	H1N1	11/09/2009	Spain	A/Catalonia/S1753/2009(H1N1)
NA	CY051974	H1N1	11/09/2009	Spain	A/Catalonia/S1756/2009(H1N1)
NA	CY051977	H1N1	11/09/2009	Spain	A/Catalonia/S1758/2009(H1N1)
NA	HQ239872	H1N1	11/09/2009	Canada	A/Canada-ON/RV2015-10/2009(H1N1)
NA	KC780285	H1N1	11/09/2009	USA	A/New Jersey/09/2009
NA	KC781384	H1N1	11/09/2009	USA	A/Maryland/38/2009
NA	KC782242	H1N1	11/09/2009	USA	A/Minnesota/40/2009
NA	KC781843	H1N1	11/09/2009	USA	A/Texas/73/2009
NA	CY075016	H1N1	11/09/2009	Thailand	A/Thailand/CU-H910/2009
NA	KC780820	H1N1	11/09/2009	USA	A/Rhode Island/20/2009
NA	KC780985	H1N1	11/09/2009	USA	A/Michigan/27/2009
NA	CY123246	H1N1	11/09/2009	Singapore	A/Singapore/GP4275/2009
NA	CY056637	H1N1	11/09/2009	USA	A/New York/6019/2009(H1N1)
NA	CY056613	H1N1	11/09/2009	USA	A/New York/5976/2009(H1N1)
NA	CY057328	H1N1	11/09/2009	USA	A/New York/6072/2009(H1N1)
NA	KC780796	H1N1	11/09/2009	USA	A/Maryland/31/2009
NA	CY056166	H1N1	11/09/2009	USA	A/Pensacola/INS40/2009(H1N1)
NA	KC780203	H1N1	11/09/2009	USA	A/Maine/13/2009
NA	KC780382	H1N1	11/09/2009	USA	A/Washington/62/2009
NA	KC781426	H1N1	11/09/2009	USA	A/Oklahoma/10/2009
NA	HQ247722	H1N1	11/09/2009	Finland	A/Finland/696/2009
NA	CY089237	H1N1	11/09/2009	USA	A/Boston/633/2009
NA	CY089245	H1N1	11/09/2009	USA	A/Boston/635/2009
NA	AB704496	H1N1	11/09/2009	Japan	A/Yamagata/473/2009
NA	CY066162	H1N1	11/09/2009	Japan	A/Nagasaki/09N057/2009
NA	CY123837	H1N1	11/09/2009	Singapore	A/Singapore/ON2274/2009
NA	HM189333	H1N1	11/09/2009	Russia	A/Moscow oblast/CRIE-STV/2009
NA	KC781619	H1N1	11/09/2009	USA	A/Idaho/14/2009
NA	CY056589	H1N1	11/09/2009	USA	A/New York/5886/2009(H1N1)
NA	CY056605	H1N1	11/09/2009	USA	A/New York/5931/2009(H1N1)
NA	CY083880	H1N1	11/09/2009	USA	A/San Diego/INS50/2009(H1N1)

NA	CY083896	H1N1	11/09/2009	USA	A/San Diego/INS73/2009(H1N1)
NA	CY083888	H1N1	11/09/2009	USA	A/San Diego/INS72/2009(H1N1)
NA	HQ256657	H1N1	11/09/2009	Myanmar	A/Myanmar/M538/2009(H1N1)
NA	CY075542	H1N1	11/09/2009	USA	A/Boston/634/2009(H1N1)
NA	CY071772	H1N1	11/09/2009	Mexico	A/Mexico City/WR1747N/2009(H1N1)
NA	CY066985	H1N1	11/09/2009	Belgium	A/Brussels/INS243/2009(H1N1)
NA	JN716359	H1N1	11/09/2009	China	A/Suzhou/J1151/2009
NA	CY066785	H1N1	11/09/2009	USA	A/San Diego/INS214/2009
NA	CY067769	H1N1	11/09/2009	Portugal	A/Lisboa/111/2009(H1N1)
NA	CY063703	H1N1	11/09/2009	China	A/Shandong-Taishan/SWL1462/2009(H1N
NA	KC780865	H1N1	11/10/2009	USA	A/Maine/09/2009
NA	JX465467	H1N1	11/10/2009	South Ko	A/Gyeonggi/4596/2009
NA	KC780732	H1N1	11/10/2009	USA	A/Kentucky/22/2009
NA	KC781686	H1N1	11/10/2009	USA	A/Alaska/43/2009
NA	KC782227	H1N1	11/10/2009	USA	A/Arkansas/10/2009
NA	KC780638	H1N1	11/10/2009	USA	A/Illinois/19/2009
NA	CY067736	H1N1	11/10/2009	United Ki	A/England/94740139/2009
NA	CY067732	H1N1	11/10/2009	United Ki	A/England/94740049/2009
NA	HQ652614	H1N1	11/10/2009	China	A/Jiangsu/S61/2009
NA	HQ652615	H1N1	11/10/2009	China	A/Jiangsu/S62/2009
NA	CY066769	H1N1	11/10/2009	USA	A/Pensacola/INS212/2009
NA	CY063584	H1N1	11/10/2009	Norway	A/Oslo/INS110/2009(H1N1)
NA	CY063814	H1N1	11/10/2009	Singapore	A/Singapore/ON2286/2009
NA	CY066793	H1N1	11/10/2009	USA	A/San Diego/INS215/2009
NA	CY062789	H1N1	11/10/2009	Denmark	A/Hvidovre/INS139/2009(H1N1)
NA	AB603642	H1N1	11/10/2009	Lebanon	A/Lebanon/09L-30/2009
NA	HM189334	H1N1	11/10/2009	Russia	A/Yaroslavl/CRIE-CHMV/2009
NA	CY066761	H1N1	11/10/2009	USA	A/Pensacola/INS211/2009
NA	HM189354	H1N1	11/10/2009	Russia	A/Lipetsk/CRIE-BVV/2009
NA	CY073144	H1N1	11/10/2009	Mexico	A/Mexico City/WRAIR1781T/2009(H1N1)
NA	KC780250	H1N1	11/10/2009	USA	A/Washington/63/2009
NA	HQ247730	H1N1	11/10/2009	Finland	A/Finland/704/2009
NA	HQ247707	H1N1	11/10/2009	Finland	A/Finland/680/2009
NA	KP019726	H1N1	11/10/2009	China	A/FuZhou/SWL11911/2009
NA	CY067715	H1N1	11/10/2009	United Ki	A/England/00380004/2009
NA	CY053629	H1N1	11/10/2009	Russia	A/Russia/165/2009(H1N1)
NA	CY107220	H1N1	11/10/2009	United Ki	A/Scotland/Inverness_10/2009
NA	CY107316	H1N1	11/10/2009	United Ki	A/Scotland/Aberdeen_8/2009
NA	CY107213	H1N1	11/10/2009	United Ki	A/Scotland/Aberdeen_1/2009
NA	CY100482	H1N1	11/10/2009	Mexico	A/Mexico City/INER13/2009
NA	CY128325	H1N1	11/10/2009	Viet Nam	A/Viet Nam/13032093/2009
NA	CY107413	H1N1	11/10/2009	United Ki	A/Scotland/Perth_09V501016/2009
NA	KC781008	H1N1	11/10/2009	USA	A/New Jersey/11/2009
NA	CY066753	H1N1	11/10/2009	USA	A/Pensacola/INS210/2009
NA	CY083553	H1N1	11/10/2009	Hungary	A/Budapest/WRAIR2393T/2009
NA	CY083545	H1N1	11/10/2009	Hungary	A/Budapest/WRAIR2393N/2009
NA	CY065850	H1N1	11/10/2009	Netherlar	A/Netherlands/1324/2009(H1N1)
NA	CY084440	H1N1	11/11/2009	USA	A/New York/6064/2009
NA	KC780633	H1N1	11/11/2009	USA	A/Mississippi/07/2009
NA	CY063285	H1N1	11/11/2009	USA	A/Wisconsin/629-D02008/2009(H1N1)
NA	CY107276	H1N1	11/11/2009	United Ki	A/Scotland/Aberdeen_2/2009
NA	CY107290	H1N1	11/11/2009	United Ki	A/Scotland/Aberdeen_4/2009
NA	CY107303	H1N1	11/11/2009	United Ki	A/Scotland/Aberdeen_6/2009
NA	CY107283	H1N1	11/11/2009	United Ki	A/Scotland/Aberdeen_3/2009

NA	CY066841	H1N1	11/11/2009	Spain	A/Madrid/INS223/2009
NA	CY107310	H1N1	11/11/2009	United Ki	A/Scotland/Aberdeen_7/2009
NA	KP019727	H1N1	11/11/2009	China	A/FuZhou/SWL11916/2009
NA	CY067716	H1N1	11/11/2009	United Ki	A/England/00380005/2009
NA	JN032408	H1N1	11/11/2009	China	A/Changchun/01/2009
NA	HQ247696	H1N1	11/11/2009	Finland	A/Finland/668/2009
NA	CY107227	H1N1	11/11/2009	United Ki	A/Scotland/Aberdeen_11/2009
NA	CY062813	H1N1	11/11/2009	Denmark	A/Odense/INS143/2009(H1N1)
NA	LC093014	H1N1	11/12/2009	Saudi Ara	A/Riyadh/3997/2009
NA	LC093031	H1N1	11/12/2009	Saudi Ara	A/Riyadh/3868/2009
NA	HM625655	H1N1	11/12/2009	Italy	A/Rome/PTV10/2009
NA	HM625656	H1N1	11/12/2009	Italy	A/Rome/PTV9/2009
NA	CY052033	H1N1	11/12/2009	Spain	A/Catalonia/S1828/2009(H1N1)
NA	CY052039	H1N1	11/12/2009	Spain	A/Catalonia/S1833/2009(H1N1)
NA	CY061277	H1N1	11/12/2009	USA	A/Pensacola/INS107/2009(H1N1)
NA	HQ247708	H1N1	11/12/2009	Finland	A/Finland/681/2009
NA	CY062653	H1N1	11/12/2009	USA	A/San Diego/INS74/2009(H1N1)
NA	HM173601	H1N1	11/12/2009	Russia	A/Blagovechensk/01/2009(H1N1)
NA	CY063293	H1N1	11/12/2009	USA	A/Wisconsin/629-D00292/2009(H1N1)
NA	CY061564	H1N1	11/12/2009	USA	A/Texas/JMS367/2009(H1N1)
NA	CY063766	H1N1	11/12/2009	Singapore	A/Singapore/GP4309/2009(H1N1)
NA	CY066809	H1N1	11/12/2009	USA	A/San Diego/INS217/2009
NA	CY067718	H1N1	11/12/2009	United Ki	A/England/00380013/2009
NA	CY067919	H1N1	11/12/2009	Portugal	A/Lisboa/89/2009(H1N1)
NA	CY107255	H1N1	11/12/2009	United Ki	A/Scotland/Inverness_15/2009
NA	JQ714186	H1N1	11/12/2009	China	A/Tianjinheping/SWL313/2009
NA	CY066060	H1N1	11/12/2009	Japan	A/Gunma/09G004/2009
NA	CY075550	H1N1	11/12/2009	USA	A/Boston/648/2009(H1N1)
NA	CY066865	H1N1	11/12/2009	USA	A/District of Columbia/INS227/2009(I
NA	CY066801	H1N1	11/12/2009	USA	A/San Diego/INS216/2009
NA	CY066857	H1N1	11/12/2009	USA	A/District of Columbia/INS226/2009(I
NA	CY067645	H1N1	11/12/2009	Spain	A/Madrid/INS224/2009(H1N1)
NA	KT181551	H1N1	11/12/2009	Singapore	A/Singapore/DMS50/2009
NA	HQ247709	H1N1	11/12/2009	Finland	A/Finland/682/2009
NA	HQ247710	H1N1	11/12/2009	Finland	A/Finland/683/2009
NA	HQ247672	H1N1	11/12/2009	Finland	A/Finland/643/2009
NA	CY089261	H1N1	11/12/2009	USA	A/Boston/650/2009
NA	CY107269	H1N1	11/12/2009	United Ki	A/Scotland/Inverness_18/2009
NA	CY107241	H1N1	11/12/2009	United Ki	A/Scotland/Aberdeen_13/2009
NA	CY107234	H1N1	11/12/2009	United Ki	A/Scotland/Aberdeen_12/2009
NA	CY066020	H1N1	11/12/2009	Japan	A/Niigata/09F027/2009
NA	CY107262	H1N1	11/12/2009	United Ki	A/Scotland/Aberdeen_16/2009
NA	CY057344	H1N1	11/12/2009	USA	A/San Diego/INS75/2009(H1N1)
NA	CY054672	H1N1	11/12/2009	Russia	A/Russia/171/2009(H1N1)
NA	GU576544	H1N1	11/12/2009	Italy	A/Ancona/254/2009(H1N1)
NA	CY123843	H1N1	11/12/2009	Singapore	A/Singapore/ON2291/2009
NA	KC781075	H1N1	11/12/2009	USA	A/Oklahoma/05/2009
NA	CY087954	H1N1	11/13/2009	Thailand	A/Thailand/CU-H935/2009
NA	HM625657	H1N1	11/13/2009	Italy	A/Rome/PTV11/2009
NA	HM625658	H1N1	11/13/2009	Italy	A/Rome/PTV12/2009
NA	HM625659	H1N1	11/13/2009	Italy	A/Rome/PTV13/2009
NA	CY052029	H1N1	11/13/2009	Spain	A/Catalonia/S1825/2009(H1N1)
NA	CY052031	H1N1	11/13/2009	Spain	A/Catalonia/S1827/2009(H1N1)
NA	CY083679	H1N1	11/13/2009	Spain	A/Madrid/INS77/2009

NA	CY117662	H1N1	11/13/2009	Mexico	A/Mexico/UASLP-026/2009
NA	KC457914	H1N1	11/13/2009	Japan	A/Japan/627/2009
NA	KC457917	H1N1	11/13/2009	Japan	A/Japan/633/2009
NA	KC457913	H1N1	11/13/2009	Japan	A/Japan/624/2009
NA	CY052035	H1N1	11/13/2009	Spain	A/Catalonia/S1829/2009(H1N1)
NA	CY052037	H1N1	11/13/2009	Spain	A/Catalonia/S1830/2009(H1N1)
NA	KC457922	H1N1	11/13/2009	Japan	A/Japan/643/2009
NA	KC457916	H1N1	11/13/2009	Japan	A/Japan/630/2009
NA	KC457918	H1N1	11/13/2009	Japan	A/Japan/636/2009
NA	HQ239858	H1N1	11/13/2009	Canada	A/Canada-MB/RV2003-10/2009
NA	CY056384	H1N1	11/13/2009	Spain	A/Madrid/RC4/2009(H1N1)
NA	KC457919	H1N1	11/13/2009	Japan	A/Japan/637/2009
NA	KC457920	H1N1	11/13/2009	Japan	A/Japan/639/2009
NA	KC457915	H1N1	11/13/2009	Japan	A/Japan/628/2009
NA	KC457921	H1N1	11/13/2009	Japan	A/Japan/642/2009
NA	CY067017	H1N1	11/13/2009	Germany	A/Bochum/INS249/2009(H1N1)
NA	CY063768	H1N1	11/13/2009	Singapore	A/Singapore/GP4326/2009(H1N1)
NA	CY060909	H1N1	11/13/2009	USA	A/Texas/JMS369/2009(H1N1)
NA	HM773212	H1N1	11/13/2009	China	A/Jiangsu/2/2009(H1N1)
NA	CY062677	H1N1	11/13/2009	Spain	A/Madrid/INS113/2009(H1N1)
NA	CY083715	H1N1	11/13/2009	Spain	A/Madrid/INS112/2009(H1N1)
NA	CY073483	H1N1	11/13/2009	Germany	A/Bochum/INS248/2009(H1N1)
NA	CY075574	H1N1	11/13/2009	USA	A/Boston/658/2009(H1N1)
NA	CY075566	H1N1	11/13/2009	USA	A/Boston/655/2009(H1N1)
NA	CY075558	H1N1	11/13/2009	USA	A/Boston/653/2009(H1N1)
NA	CY067025	H1N1	11/13/2009	Germany	A/Bochum/INS250/2009(H1N1)
NA	CY066849	H1N1	11/13/2009	Spain	A/Madrid/INS225/2009
NA	CY066873	H1N1	11/13/2009	USA	A/District of Columbia/INS228/2009(I
NA	CY071804	H1N1	11/13/2009	Haiti	A/Port Au Prince/WR2294T/2009(H1N1)
NA	CY087210	H1N1	11/13/2009	USA	A/Texas/JMS368/2009
NA	HQ247698	H1N1	11/13/2009	Finland	A/Finland/670/2009
NA	HQ247713	H1N1	11/13/2009	Finland	A/Finland/686/2009
NA	HM072410	H1N1	11/13/2009	Taiwan	A/Taiwan/2203/2009
NA	CY107248	H1N1	11/13/2009	United Ki	A/Scotland/Aberdeen_14/2009
NA	CY128373	H1N1	11/13/2009	Viet Nam	A/Viet Nam/11032001/2009
NA	JX625600	H1N1	11/13/2009	United Ki	A/England/94720002/2009
NA	CY073090	H1N1	11/13/2009	Turkey	A/Ankara/WRAIR1435T/2009
NA	JN631046	H1N1	11/13/2009	China	A/Shanghai/1744T/2009
NA	CY066090	H1N1	11/13/2009	Japan	A/Kyoto/09K076/2009
NA	HM189335	H1N1	11/13/2009	Russia	A/Ulyanovsk/CRIE-KLA/2009
NA	CY107297	H1N1	11/13/2009	United Ki	A/Scotland/Aberdeen_5/2009
NA	KC781106	H1N1	11/13/2009	USA	A/Rhode Island/22/2009
NA	GU576530	H1N1	11/13/2009	Italy	A/Ancona/274/2009(H1N1)
NA	GU576528	H1N1	11/13/2009	Italy	A/Ancona/263/2009(H1N1)
NA	KC780760	H1N1	11/13/2009	USA	A/West Virginia/11/2009
NA	KC780771	H1N1	11/13/2009	USA	A/Arkansas/09/2009
NA	KC780256	H1N1	11/13/2009	USA	A/Minnesota/20/2009
NA	CY075484	H1N1	11/14/2009	Russia	A/Nizhny Novgorod/RII1/2009(H1N1)
NA	CY060917	H1N1	11/14/2009	USA	A/Texas/JMS370/2009(H1N1)
NA	CY060925	H1N1	11/14/2009	USA	A/Texas/JMS371/2009(H1N1)
NA	CY089269	H1N1	11/14/2009	USA	A/Boston/657/2009
NA	CY123256	H1N1	11/14/2009	Singapore	A/Singapore/GP4348/2009
NA	HQ247700	H1N1	11/14/2009	Finland	A/Finland/672/2009
NA	HQ247697	H1N1	11/14/2009	Finland	A/Finland/669/2009

NA	CY128421	H1N1	11/14/2009	Viet Nam	A/Viet Nam/12032001/2009
NA	CY128341	H1N1	11/14/2009	Viet Nam	A/Viet Nam/13032097/2009
NA	CY128333	H1N1	11/14/2009	Viet Nam	A/Viet Nam/13032096/2009
NA	CY057816	H1N1	11/14/2009	USA	A/Wisconsin/629-D01826/2009(H1N1)
NA	GU451256	H1N1	11/14/2009	Russia	A/Tver/IIV2969/2009(H1N1)
NA	CY057824	H1N1	11/14/2009	USA	A/Wisconsin/629-D00965/2009(H1N1)
NA	HM625660	H1N1	11/15/2009	Italy	A/Rome/PTV14/2009
NA	CY060933	H1N1	11/15/2009	USA	A/Texas/JMS372/2009(H1N1)
NA	CY065117	H1N1	11/15/2009	Netherlar	A/Netherlands/2464b/2009
NA	CY066225	H1N1	11/15/2009	USA	A/California/VRDL106/2009(H1N1)
NA	CY066241	H1N1	11/15/2009	USA	A/California/VRDL108/2009(H1N1)
NA	CY066217	H1N1	11/15/2009	USA	A/California/VRDL105/2009(H1N1)
NA	CY066209	H1N1	11/15/2009	USA	A/California/VRDL104/2009(H1N1)
NA	CY056685	H1N1	11/15/2009	USA	A/New York/6257/2009(H1N1)
NA	CY128349	H1N1	11/15/2009	Viet Nam	A/Viet Nam/13032100/2009
NA	CY089301	H1N1	11/15/2009	USA	A/Boston/671/2009
NA	CY073066	H1N1	11/15/2009	Turkey	A/Ankara/WRAIR1425T/2009(H1N1)
NA	CY075598	H1N1	11/15/2009	USA	A/Boston/672/2009(H1N1)
NA	CY066233	H1N1	11/15/2009	USA	A/California/VRDL107/2009(H1N1)
NA	KT181552	H1N1	11/15/2009	Singapore	A/Singapore/DMS51/2009
NA	HM581922	H1N1	11/15/2009	Iran	A/Iran/14068/2009(H1N1)
NA	HQ247703	H1N1	11/15/2009	Finland	A/Finland/675/2009
NA	CY128469	H1N1	11/15/2009	Viet Nam	A/Viet Nam/001-1020/2009
NA	HM581920	H1N1	11/15/2009	Iran	A/Iran/14089/2009(H1N1)
NA	CY057848	H1N1	11/15/2009	USA	A/Wisconsin/629-D02060/2009(H1N1)
NA	CY057840	H1N1	11/15/2009	USA	A/Wisconsin/629-D00128/2009(H1N1)
NA	CY057856	H1N1	11/15/2009	USA	A/Wisconsin/629-D00295/2009(H1N1)
NA	CY057832	H1N1	11/15/2009	USA	A/Wisconsin/629-D00911/2009(H1N1)
NA	CY057864	H1N1	11/15/2009	USA	A/Wisconsin/629-D00936/2009(H1N1)
NA	JX482561	H1N1	11/15/2009	Mexico	A/Yucatan/2441/2009
NA	JX482562	H1N1	11/15/2009	Mexico	A/Yucatan/2440/2009
NA	JX482563	H1N1	11/15/2009	Mexico	A/Yucatan/2439/2009
NA	JX482564	H1N1	11/15/2009	Mexico	A/Yucatan/2438/2009
NA	KC457924	H1N1	11/16/2009	Japan	A/Japan/649/2009
NA	KC459814	H1N1	11/16/2009	Japan	A/Japan/714/2009
NA	KC459867	H1N1	11/16/2009	Japan	A/Japan/649/2009
NA	KC459863	H1N1	11/16/2009	Japan	A/Japan/649/2009
NA	KC459856	H1N1	11/16/2009	Japan	A/Japan/649/2009
NA	KC459854	H1N1	11/16/2009	Japan	A/Japan/649/2009
NA	KC457926	H1N1	11/16/2009	Japan	A/Japan/659/2009
NA	KC459824	H1N1	11/16/2009	Japan	A/Japan/714/2009
NA	KC459827	H1N1	11/16/2009	Japan	A/Japan/714/2009
NA	KC459833	H1N1	11/16/2009	Japan	A/Japan/714/2009
NA	CY067001	H1N1	11/16/2009	Germany	A/Munich/INS246/2009
NA	CY089285	H1N1	11/16/2009	USA	A/Boston/667/2009
NA	CY067735	H1N1	11/16/2009	United Ki	A/England/94740138/2009
NA	HQ256659	H1N1	11/16/2009	Myanmar	A/Myanmar/M541/2009(H1N1)
NA	CY063816	H1N1	11/16/2009	Singapore	A/Singapore/ON2308/2009
NA	CY056669	H1N1	11/16/2009	USA	A/New York/6213/2009(H1N1)
NA	AB704432	H1N1	11/16/2009	Japan	A/Gunma/263/2009
NA	CY056701	H1N1	11/16/2009	USA	A/New York/6293/2009(H1N1)
NA	CY066092	H1N1	11/16/2009	Japan	A/Kyoto/09K084/2009
NA	CY056661	H1N1	11/16/2009	USA	A/New York/6182/2009(H1N1)
NA	CY075582	H1N1	11/16/2009	USA	A/Boston/663/2009(H1N1)

NA	CY089293	H1N1	11/16/2009	USA	A/Boston/670/2009
NA	HQ698629	H1N1	11/16/2009	China	A/Beijing/16/2009
NA	CY075590	H1N1	11/16/2009	USA	A/Boston/666/2009(H1N1)
NA	HQ247676	H1N1	11/16/2009	Finland	A/Finland/647/2009
NA	CY067737	H1N1	11/16/2009	United Ki	A/England/94740140/2009
NA	CY089309	H1N1	11/16/2009	USA	A/Boston/673/2009
NA	CY089277	H1N1	11/16/2009	USA	A/Boston/665/2009
NA	CY123254	H1N1	11/16/2009	Singapore	A/Singapore/GP4332/2009
NA	CY057872	H1N1	11/16/2009	USA	A/Wisconsin/629-D01661/2009(H1N1)
NA	CY056677	H1N1	11/16/2009	USA	A/New York/6214/2009(H1N1)
NA	KP637408	H1N1	11/16/2009	Thailand	A/Thailand/MR08303/2009
NA	CY065133	H1N1	11/16/2009	Netherlar	A/Netherlands/2143/2009
NA	GU371257	H1N1	11/16/2009	Russia	A/Orenburg/IIV2974/2009(H1N1)
NA	GU576533	H1N1	11/16/2009	Italy	A/Ancona/310/2009(H1N1)
NA	CY123847	H1N1	11/16/2009	Singapore	A/Singapore/ON2299/2009
NA	CY058334	H1N1	11/16/2009	USA	A/Wisconsin/629-D01347/2009(H1N1)
NA	KC781145	H1N1	11/16/2009	USA	A/Utah/59/2009
NA	KC781109	H1N1	11/16/2009	USA	A/New Hampshire/21/2009
NA	LC093010	H1N1	11/16/2009	Saudi Ara	A/Riyadh/4136/2009
NA	LC093042	H1N1	11/16/2009	Saudi Ara	A/Riyadh/4160/2009
NA	KC459848	H1N1	11/16/2009	Japan	A/Japan/649/2009
NA	KC459825	H1N1	11/16/2009	Japan	A/Japan/714/2009
NA	KC459850	H1N1	11/16/2009	Japan	A/Japan/649/2009
NA	KC459818	H1N1	11/16/2009	Japan	A/Japan/714/2009
NA	KC459834	H1N1	11/16/2009	Japan	A/Japan/714/2009
NA	KC459821	H1N1	11/16/2009	Japan	A/Japan/714/2009
NA	KC459844	H1N1	11/16/2009	Japan	A/Japan/649/2009
NA	KC459832	H1N1	11/16/2009	Japan	A/Japan/714/2009
NA	CY052043	H1N1	11/16/2009	Spain	A/Catalonia/S1835/2009(H1N1)
NA	KC457930	H1N1	11/16/2009	Japan	A/Japan/697/2009
NA	KC459813	H1N1	11/16/2009	Japan	A/Japan/714/2009
NA	KC459859	H1N1	11/16/2009	Japan	A/Japan/649/2009
NA	KC457931	H1N1	11/16/2009	Japan	A/Japan/713/2009
NA	GU216651	H1N1	11/16/2009	Italy	A/Pavia/21/2009(H1N1)
NA	KC459838	H1N1	11/16/2009	Japan	A/Japan/649/2009
NA	KC459822	H1N1	11/16/2009	Japan	A/Japan/714/2009
NA	KC459864	H1N1	11/16/2009	Japan	A/Japan/649/2009
NA	KC459815	H1N1	11/16/2009	Japan	A/Japan/714/2009
NA	KC459817	H1N1	11/16/2009	Japan	A/Japan/714/2009
NA	KC459839	H1N1	11/16/2009	Japan	A/Japan/649/2009
NA	KC459837	H1N1	11/16/2009	Japan	A/Japan/714/2009
NA	KC457928	H1N1	11/16/2009	Japan	A/Japan/674/2009
NA	KC459826	H1N1	11/16/2009	Japan	A/Japan/714/2009
NA	KC459829	H1N1	11/16/2009	Japan	A/Japan/714/2009
NA	KC459830	H1N1	11/16/2009	Japan	A/Japan/714/2009
NA	KC459851	H1N1	11/16/2009	Japan	A/Japan/649/2009
NA	CY052041	H1N1	11/16/2009	Spain	A/Catalonia/S1834/2009(H1N1)
NA	KC459849	H1N1	11/16/2009	Japan	A/Japan/649/2009
NA	KC459857	H1N1	11/16/2009	Japan	A/Japan/649/2009
NA	KC459868	H1N1	11/16/2009	Japan	A/Japan/649/2009
NA	KC459853	H1N1	11/16/2009	Japan	A/Japan/649/2009
NA	KC459855	H1N1	11/16/2009	Japan	A/Japan/649/2009
NA	KC459842	H1N1	11/16/2009	Japan	A/Japan/649/2009
NA	KC459852	H1N1	11/16/2009	Japan	A/Japan/649/2009

NA	KC457927	H1N1	11/16/2009	Japan	A/Japan/662/2009
NA	KC457932	H1N1	11/16/2009	Japan	A/Japan/714/2009
NA	KC459861	H1N1	11/16/2009	Japan	A/Japan/649/2009
NA	KC459812	H1N1	11/16/2009	Japan	A/Japan/714/2009
NA	KC459835	H1N1	11/16/2009	Japan	A/Japan/714/2009
NA	KC459866	H1N1	11/16/2009	Japan	A/Japan/649/2009
NA	KC459823	H1N1	11/16/2009	Japan	A/Japan/714/2009
NA	KC459828	H1N1	11/16/2009	Japan	A/Japan/714/2009
NA	KC459831	H1N1	11/16/2009	Japan	A/Japan/714/2009
NA	KC457933	H1N1	11/16/2009	Japan	A/Japan/715/2009
NA	KC459843	H1N1	11/16/2009	Japan	A/Japan/649/2009
NA	KC459836	H1N1	11/16/2009	Japan	A/Japan/714/2009
NA	KC459858	H1N1	11/16/2009	Japan	A/Japan/649/2009
NA	KC457923	H1N1	11/16/2009	Japan	A/Japan/648/2009
NA	KC457925	H1N1	11/16/2009	Japan	A/Japan/653/2009
NA	KC459847	H1N1	11/16/2009	Japan	A/Japan/649/2009
NA	KC459865	H1N1	11/16/2009	Japan	A/Japan/649/2009
NA	KC459820	H1N1	11/16/2009	Japan	A/Japan/714/2009
NA	HQ239873	H1N1	11/16/2009	Canada	A/Canada-ON/RV2016-10/2009(H1N1)
NA	KC459846	H1N1	11/16/2009	Japan	A/Japan/649/2009
NA	KC459862	H1N1	11/16/2009	Japan	A/Japan/649/2009
NA	KC459816	H1N1	11/16/2009	Japan	A/Japan/714/2009
NA	KC457929	H1N1	11/16/2009	Japan	A/Japan/696/2009
NA	KC459840	H1N1	11/16/2009	Japan	A/Japan/649/2009
NA	KC459860	H1N1	11/16/2009	Japan	A/Japan/649/2009
NA	KC459845	H1N1	11/16/2009	Japan	A/Japan/649/2009
NA	KC459819	H1N1	11/16/2009	Japan	A/Japan/714/2009
NA	KC459841	H1N1	11/16/2009	Japan	A/Japan/649/2009
NA	KC780808	H1N1	11/17/2009	USA	A/Indiana/19/2009
NA	KC781330	H1N1	11/17/2009	USA	A/Tennessee/21/2009
NA	CY067262	H1N1	11/17/2009	Kazakhsta	A/Almaty/330/2009(H1N1)
NA	CY087960	H1N1	11/17/2009	Thailand	A/Thailand/CU-H952/2009
NA	KC457940	H1N1	11/17/2009	Japan	A/Japan/738/2009
NA	KC459811	H1N1	11/17/2009	Japan	A/Japan/736/2009
NA	KC459800	H1N1	11/17/2009	Japan	A/Japan/736/2009
NA	KC459799	H1N1	11/17/2009	Japan	A/Japan/736/2009
NA	KC459794	H1N1	11/17/2009	Japan	A/Japan/736/2009
NA	GU361110	H1N1	11/17/2009	Sweden	A/Stockholm/091120-2/2009(H1N1)
NA	CY064798	H1N1	11/17/2009	Spain	A/Valencia/HLF2/2009(H1N1)
NA	KC459807	H1N1	11/17/2009	Japan	A/Japan/736/2009
NA	KC459798	H1N1	11/17/2009	Japan	A/Japan/736/2009
NA	KC459795	H1N1	11/17/2009	Japan	A/Japan/736/2009
NA	KC459797	H1N1	11/17/2009	Japan	A/Japan/736/2009
NA	KC459804	H1N1	11/17/2009	Japan	A/Japan/736/2009
NA	KC459790	H1N1	11/17/2009	Japan	A/Japan/736/2009
NA	KC459801	H1N1	11/17/2009	Japan	A/Japan/736/2009
NA	KC459792	H1N1	11/17/2009	Japan	A/Japan/736/2009
NA	KC459793	H1N1	11/17/2009	Japan	A/Japan/736/2009
NA	KC457939	H1N1	11/17/2009	Japan	A/Japan/737/2009
NA	KC459796	H1N1	11/17/2009	Japan	A/Japan/736/2009
NA	KC459802	H1N1	11/17/2009	Japan	A/Japan/736/2009
NA	KC457934	H1N1	11/17/2009	Japan	A/Japan/731/2009
NA	CY061285	H1N1	11/17/2009	USA	A/Pensacola/INS108/2009(H1N1)
NA	KC457935	H1N1	11/17/2009	Japan	A/Japan/732/2009

NA	KC457941	H1N1	11/17/2009	Japan	A/Japan/740/2009
NA	KC459805	H1N1	11/17/2009	Japan	A/Japan/736/2009
NA	KC459803	H1N1	11/17/2009	Japan	A/Japan/736/2009
NA	KC459806	H1N1	11/17/2009	Japan	A/Japan/736/2009
NA	KC459808	H1N1	11/17/2009	Japan	A/Japan/736/2009
NA	KC457937	H1N1	11/17/2009	Japan	A/Japan/735/2009
NA	KC459810	H1N1	11/17/2009	Japan	A/Japan/736/2009
NA	KC457936	H1N1	11/17/2009	Japan	A/Japan/734/2009
NA	KC457938	H1N1	11/17/2009	Japan	A/Japan/736/2009
NA	KC459809	H1N1	11/17/2009	Japan	A/Japan/736/2009
NA	KC459791	H1N1	11/17/2009	Japan	A/Japan/736/2009
NA	CY062669	H1N1	11/17/2009	USA	A/Pensacola/INS109/2009(H1N1)
NA	GU576545	H1N1	11/17/2009	Italy	A/Ancona/337/2009(H1N1)
NA	CY067719	H1N1	11/17/2009	United Ki	A/England/00380015/2009
NA	CY067741	H1N1	11/17/2009	United Ki	A/England/94780019/2009
NA	CY060728	H1N1	11/17/2009	Canada	A/Ontario/328474/2009(H1N1)
NA	AB847955	H1N1	11/17/2009	Japan	A/Hokkaido/T64/2009
NA	CY063818	H1N1	11/17/2009	Singapore	A/Singapore/ON2313/2009
NA	CY056693	H1N1	11/17/2009	USA	A/New York/6292/2009(H1N1)
NA	CY066094	H1N1	11/17/2009	Japan	A/Kyoto/09K085/2009
NA	CY075606	H1N1	11/17/2009	USA	A/Boston/681/2009(H1N1)
NA	CY075614	H1N1	11/17/2009	USA	A/Boston/682/2009(H1N1)
NA	CY066777	H1N1	11/17/2009	USA	A/Pensacola/INS213/2009
NA	CY069116	H1N1	11/17/2009	Spain	A/Madrid/INS296/2009(H1N1)
NA	CY065826	H1N1	11/17/2009	Netherlar	A/Netherlands/2243/2009
NA	HQ247711	H1N1	11/17/2009	Finland	A/Finland/684/2009
NA	CY089325	H1N1	11/17/2009	USA	A/Boston/680/2009
NA	CY089333	H1N1	11/17/2009	USA	A/Boston/683/2009
NA	CY089317	H1N1	11/17/2009	USA	A/Boston/678/2009
NA	CY128050	H1N1	11/17/2009	Viet Nam	A/Viet Nam/11032002/2009
NA	JF429399	H1N1	11/17/2009	France	A/Lyon/47.431/2009
NA	CY123855	H1N1	11/17/2009	Singapore	A/Singapore/ON2318/2009
NA	KP637808	H1N1	11/17/2009	Thailand	A/Thailand/SS08207/2009
NA	CY064431	H1N1	11/17/2009	China	A/Guangdong/1078/2009(H1N1)
NA	CY056709	H1N1	11/17/2009	USA	A/New York/6300/2009(H1N1)
NA	KC780348	H1N1	11/17/2009	USA	A/Alaska/44/2009
NA	CY058350	H1N1	11/17/2009	USA	A/Wisconsin/629-D01582/2009(H1N1)
NA	CY058342	H1N1	11/17/2009	USA	A/Wisconsin/629-D00503/2009(H1N1)
NA	CY066721	H1N1	11/17/2009	Spain	A/Madrid/INS295/2009(H1N1)
NA	KC781935	H1N1	11/17/2009	USA	A/Utah/53/2009
NA	KC459785	H1N1	11/18/2009	Japan	A/Japan/751/2009
NA	KC459726	H1N1	11/18/2009	Japan	A/Japan/782/2009
NA	KC459749	H1N1	11/18/2009	Japan	A/Japan/753/2009
NA	KC458559	H1N1	11/18/2009	Japan	A/Japan/750/2009
NA	KC458556	H1N1	11/18/2009	Japan	A/Japan/750/2009
NA	KC457948	H1N1	11/18/2009	Japan	A/Japan/751/2009
NA	KC459751	H1N1	11/18/2009	Japan	A/Japan/753/2009
NA	HM625662	H1N1	11/18/2009	Italy	A/Rome/PTV16/2009
NA	HM625661	H1N1	11/18/2009	Italy	A/Rome/PTV15/2009
NA	CY117665	H1N1	11/18/2009	Mexico	A/Mexico/UASLP-027/2009
NA	KC457962	H1N1	11/18/2009	Japan	A/Japan/791/2009
NA	KC457946	H1N1	11/18/2009	Japan	A/Japan/749/2009
NA	KC459773	H1N1	11/18/2009	Japan	A/Japan/752/2009
NA	KC459738	H1N1	11/18/2009	Japan	A/Japan/782/2009

NA	KC459762	H1N1	11/18/2009	Japan	A/Japan/753/2009
NA	KC459765	H1N1	11/18/2009	Japan	A/Japan/753/2009
NA	KC459787	H1N1	11/18/2009	Japan	A/Japan/751/2009
NA	KC457969	H1N1	11/18/2009	Japan	A/Japan/805/2009
NA	KC457961	H1N1	11/18/2009	Japan	A/Japan/790/2009
NA	KC457970	H1N1	11/18/2009	Japan	A/Japan/806/2009
NA	KC457953	H1N1	11/18/2009	Japan	A/Japan/757/2009
NA	KC459734	H1N1	11/18/2009	Japan	A/Japan/782/2009
NA	KC459786	H1N1	11/18/2009	Japan	A/Japan/751/2009
NA	KC457974	H1N1	11/18/2009	Japan	A/Japan/812/2009
NA	KC457958	H1N1	11/18/2009	Japan	A/Japan/780/2009
NA	KC457973	H1N1	11/18/2009	Japan	A/Japan/811/2009
NA	KC459761	H1N1	11/18/2009	Japan	A/Japan/753/2009
NA	KC459767	H1N1	11/18/2009	Japan	A/Japan/752/2009
NA	KC459720	H1N1	11/18/2009	Japan	A/Japan/782/2009
NA	KC459770	H1N1	11/18/2009	Japan	A/Japan/752/2009
NA	KC458566	H1N1	11/18/2009	Japan	A/Japan/750/2009
NA	KC457956	H1N1	11/18/2009	Japan	A/Japan/768/2009
NA	KC457960	H1N1	11/18/2009	Japan	A/Japan/782/2009
NA	KC458562	H1N1	11/18/2009	Japan	A/Japan/750/2009
NA	KC459758	H1N1	11/18/2009	Japan	A/Japan/753/2009
NA	KC457950	H1N1	11/18/2009	Japan	A/Japan/753/2009
NA	KC457951	H1N1	11/18/2009	Japan	A/Japan/754/2009
NA	KC457971	H1N1	11/18/2009	Japan	A/Japan/808/2009
NA	KC457967	H1N1	11/18/2009	Japan	A/Japan/799/2009
NA	KC459778	H1N1	11/18/2009	Japan	A/Japan/752/2009
NA	KC459779	H1N1	11/18/2009	Japan	A/Japan/751/2009
NA	KC459788	H1N1	11/18/2009	Japan	A/Japan/751/2009
NA	KC459789	H1N1	11/18/2009	Japan	A/Japan/751/2009
NA	KC457972	H1N1	11/18/2009	Japan	A/Japan/809/2009
NA	KC458563	H1N1	11/18/2009	Japan	A/Japan/750/2009
NA	KC459769	H1N1	11/18/2009	Japan	A/Japan/752/2009
NA	KC459771	H1N1	11/18/2009	Japan	A/Japan/752/2009
NA	KC457968	H1N1	11/18/2009	Japan	A/Japan/800/2009
NA	KC457945	H1N1	11/18/2009	Japan	A/Japan/748/2009
NA	KC459764	H1N1	11/18/2009	Japan	A/Japan/753/2009
NA	KC457947	H1N1	11/18/2009	Japan	A/Japan/750/2009
NA	KC457943	H1N1	11/18/2009	Japan	A/Japan/746/2009
NA	KC459721	H1N1	11/18/2009	Japan	A/Japan/782/2009
NA	KC459759	H1N1	11/18/2009	Japan	A/Japan/753/2009
NA	KC457957	H1N1	11/18/2009	Japan	A/Japan/769/2009
NA	KC457942	H1N1	11/18/2009	Japan	A/Japan/744/2009
NA	KC457959	H1N1	11/18/2009	Japan	A/Japan/781/2009
NA	KC459719	H1N1	11/18/2009	Japan	A/Japan/782/2009
NA	KC459748	H1N1	11/18/2009	Japan	A/Japan/753/2009
NA	KC459755	H1N1	11/18/2009	Japan	A/Japan/753/2009
NA	KC459760	H1N1	11/18/2009	Japan	A/Japan/753/2009
NA	KC458557	H1N1	11/18/2009	Japan	A/Japan/750/2009
NA	KC458565	H1N1	11/18/2009	Japan	A/Japan/750/2009
NA	KC458558	H1N1	11/18/2009	Japan	A/Japan/750/2009
NA	KC457949	H1N1	11/18/2009	Japan	A/Japan/752/2009
NA	KC457955	H1N1	11/18/2009	Japan	A/Japan/760/2009
NA	KC457952	H1N1	11/18/2009	Japan	A/Japan/755/2009
NA	KC457964	H1N1	11/18/2009	Japan	A/Japan/794/2009

NA	KC459746	H1N1	11/18/2009	Japan	A/Japan/753/2009
NA	KC459747	H1N1	11/18/2009	Japan	A/Japan/753/2009
NA	KC459756	H1N1	11/18/2009	Japan	A/Japan/753/2009
NA	KC458561	H1N1	11/18/2009	Japan	A/Japan/750/2009
NA	KC459740	H1N1	11/18/2009	Japan	A/Japan/782/2009
NA	KC459781	H1N1	11/18/2009	Japan	A/Japan/751/2009
NA	KC459784	H1N1	11/18/2009	Japan	A/Japan/751/2009
NA	KC457954	H1N1	11/18/2009	Japan	A/Japan/758/2009
NA	KC457966	H1N1	11/18/2009	Japan	A/Japan/797/2009
NA	KC459723	H1N1	11/18/2009	Japan	A/Japan/782/2009
NA	KC458560	H1N1	11/18/2009	Japan	A/Japan/750/2009
NA	KC458564	H1N1	11/18/2009	Japan	A/Japan/750/2009
NA	KC459744	H1N1	11/18/2009	Japan	A/Japan/753/2009
NA	KC459745	H1N1	11/18/2009	Japan	A/Japan/753/2009
NA	KC459750	H1N1	11/18/2009	Japan	A/Japan/753/2009
NA	KC459774	H1N1	11/18/2009	Japan	A/Japan/752/2009
NA	KC459780	H1N1	11/18/2009	Japan	A/Japan/751/2009
NA	KC459724	H1N1	11/18/2009	Japan	A/Japan/782/2009
NA	KC459742	H1N1	11/18/2009	Japan	A/Japan/753/2009
NA	KC459728	H1N1	11/18/2009	Japan	A/Japan/782/2009
NA	KC459730	H1N1	11/18/2009	Japan	A/Japan/782/2009
NA	KC457944	H1N1	11/18/2009	Japan	A/Japan/747/2009
NA	KC459735	H1N1	11/18/2009	Japan	A/Japan/782/2009
NA	KC459752	H1N1	11/18/2009	Japan	A/Japan/753/2009
NA	KC459757	H1N1	11/18/2009	Japan	A/Japan/753/2009
NA	KC459776	H1N1	11/18/2009	Japan	A/Japan/752/2009
NA	KC459722	H1N1	11/18/2009	Japan	A/Japan/782/2009
NA	KC459733	H1N1	11/18/2009	Japan	A/Japan/782/2009
NA	KC459782	H1N1	11/18/2009	Japan	A/Japan/751/2009
NA	KC459727	H1N1	11/18/2009	Japan	A/Japan/782/2009
NA	KC459768	H1N1	11/18/2009	Japan	A/Japan/752/2009
NA	KC459772	H1N1	11/18/2009	Japan	A/Japan/752/2009
NA	KC459743	H1N1	11/18/2009	Japan	A/Japan/753/2009
NA	KC459753	H1N1	11/18/2009	Japan	A/Japan/753/2009
NA	KC459754	H1N1	11/18/2009	Japan	A/Japan/753/2009
NA	KC459731	H1N1	11/18/2009	Japan	A/Japan/782/2009
NA	KC459777	H1N1	11/18/2009	Japan	A/Japan/752/2009
NA	KC459783	H1N1	11/18/2009	Japan	A/Japan/751/2009
NA	KC458555	H1N1	11/18/2009	Japan	A/Japan/750/2009
NA	KC459737	H1N1	11/18/2009	Japan	A/Japan/782/2009
NA	KC459741	H1N1	11/18/2009	Japan	A/Japan/782/2009
NA	KC459766	H1N1	11/18/2009	Japan	A/Japan/752/2009
NA	KC459725	H1N1	11/18/2009	Japan	A/Japan/782/2009
NA	KC459739	H1N1	11/18/2009	Japan	A/Japan/782/2009
NA	KC457965	H1N1	11/18/2009	Japan	A/Japan/796/2009
NA	KC459736	H1N1	11/18/2009	Japan	A/Japan/782/2009
NA	KC459763	H1N1	11/18/2009	Japan	A/Japan/753/2009
NA	KC459775	H1N1	11/18/2009	Japan	A/Japan/752/2009
NA	KC459729	H1N1	11/18/2009	Japan	A/Japan/782/2009
NA	KC459732	H1N1	11/18/2009	Japan	A/Japan/782/2009
NA	KC457963	H1N1	11/18/2009	Japan	A/Japan/793/2009
NA	CY058831	H1N1	11/18/2009	China	A/Guangdong/2221/2009(H1N1)
NA	CY064427	H1N1	11/18/2009	China	A/Guangdong/1071/2009(H1N1)
NA	CY060461	H1N1	11/18/2009	South Ko	A/Gangwon/1805/2009(H1N1)

NA	CY073192	H1N1	11/18/2009	Serbia	A/Belgrade/WRAIR2368N/2009(H1N1)
NA	JN036573	H1N1	11/18/2009	China	A/Hualong/SWL1347/2009
NA	CY067653	H1N1	11/18/2009	Belgium	A/Brussels/INS244/2009(H1N1)
NA	CY073168	H1N1	11/18/2009	Nigeria	A/Lagos/WRAIR1984N/2009(H1N1)
NA	CY073200	H1N1	11/18/2009	Serbia	A/Belgrade/WRAIR2368T/2009(H1N1)
NA	CY073176	H1N1	11/18/2009	Nigeria	A/Lagos/WRAIR1984T/2009(H1N1)
NA	CY073743	H1N1	11/18/2009	Denmark	A/Copenhagen/INS433/2009(H1N1)
NA	CY067742	H1N1	11/18/2009	United Ki	A/England/94780021/2009
NA	CY066993	H1N1	11/18/2009	Belgium	A/Brussels/INS245/2009(H1N1)
NA	CY088731	H1N1	11/18/2009	Netherlar	A/Netherlands/2223b/2009
NA	CY089341	H1N1	11/18/2009	USA	A/Boston/685/2009
NA	CY089349	H1N1	11/18/2009	USA	A/Boston/686/2009
NA	CY066112	H1N1	11/18/2009	Japan	A/Hyogo/09K128/2009
NA	HQ247732	H1N1	11/18/2009	Finland	A/Finland/706/2009
NA	HQ247699	H1N1	11/18/2009	Finland	A/Finland/671/2009
NA	HM567986	H1N1	11/18/2009	United Ki	A/England/869/2009
NA	JN036566	H1N1	11/18/2009	China	A/Xuchang/SWL49/2009
NA	JN036565	H1N1	11/18/2009	China	A/Xuchang/SWL48/2009
NA	JN036574	H1N1	11/18/2009	China	A/Weidu/SWL1353/2009
NA	JN036575	H1N1	11/18/2009	China	A/Ruzhou/SWL564/2009
NA	JN036563	H1N1	11/18/2009	China	A/Dongcheng/SWL45/2009
NA	JN036572	H1N1	11/18/2009	China	A/Xigong/SWL11045/2009
NA	JN036571	H1N1	11/18/2009	China	A/Zhongyuan/SWL1643/2009
NA	JN036576	H1N1	11/18/2009	China	A/Weidong/SWL5165/2009
NA	JN036577	H1N1	11/18/2009	China	A/Weidu/SWL1379/2009
NA	JN036568	H1N1	11/18/2009	China	A/Hualong/SWL1324/2009
NA	JN036561	H1N1	11/18/2009	China	A/Qingfeng/SWL43/2009
NA	JN036569	H1N1	11/18/2009	China	A/Hualong/SWL1334/2009
NA	JN036567	H1N1	11/18/2009	China	A/Hualong/SWL1313/2009
NA	JN036564	H1N1	11/18/2009	China	A/Ruyang/SWL1606/2009
NA	JN036562	H1N1	11/18/2009	China	A/Jinshui/SWL54/2009
NA	JN036570	H1N1	11/18/2009	China	A/Baofeng/SWL537/2009
NA	HM189341	H1N1	11/18/2009	Russia	A/Stavropol/CRIE-BLV/2009
NA	CY066022	H1N1	11/18/2009	Japan	A/Niigata/09F047/2009
NA	CY066164	H1N1	11/18/2009	Japan	A/Nagasaki/09N074/2009
NA	CY063770	H1N1	11/18/2009	Singapore	A/Singapore/GP4381/2009
NA	CY128357	H1N1	11/18/2009	Viet Nam	A/Viet Nam/15032003/2009
NA	GU560010	H1N1	11/18/2009	Russia	A/Tomsk/05/2009(H1N1)
NA	GU576535	H1N1	11/18/2009	Italy	A/Ancona/348/2009(H1N1)
NA	CY056717	H1N1	11/18/2009	USA	A/New York/6346/2009(H1N1)
NA	CY058358	H1N1	11/18/2009	USA	A/Wisconsin/629-D02489/2009(H1N1)
NA	KC459717	H1N1	11/19/2009	Japan	A/Japan/826/2009
NA	KC459683	H1N1	11/19/2009	Japan	A/Japan/833/2009
NA	KC459685	H1N1	11/19/2009	Japan	A/Japan/833/2009
NA	KC457984	H1N1	11/19/2009	Japan	A/Japan/836/2009
NA	KC459632	H1N1	11/19/2009	Japan	A/Japan/889/2009
NA	KC459652	H1N1	11/19/2009	Japan	A/Japan/842/2009
NA	KC459629	H1N1	11/19/2009	Japan	A/Japan/889/2009
NA	KC457982	H1N1	11/19/2009	Japan	A/Japan/832/2009
NA	KC457988	H1N1	11/19/2009	Japan	A/Japan/859/2009
NA	KC459653	H1N1	11/19/2009	Japan	A/Japan/842/2009
NA	KC459674	H1N1	11/19/2009	Japan	A/Japan/833/2009
NA	KC457996	H1N1	11/19/2009	Japan	A/Japan/879/2009
NA	KC459677	H1N1	11/19/2009	Japan	A/Japan/833/2009

NA	KC457992	H1N1	11/19/2009	Japan	A/Japan/871/2009
NA	KC459662	H1N1	11/19/2009	Japan	A/Japan/842/2009
NA	KC459676	H1N1	11/19/2009	Japan	A/Japan/833/2009
NA	KC459617	H1N1	11/19/2009	Japan	A/Japan/889/2009
NA	KC459636	H1N1	11/19/2009	Japan	A/Japan/842/2009
NA	KC459681	H1N1	11/19/2009	Japan	A/Japan/833/2009
NA	KC459622	H1N1	11/19/2009	Japan	A/Japan/889/2009
NA	KC459643	H1N1	11/19/2009	Japan	A/Japan/842/2009
NA	KC459658	H1N1	11/19/2009	Japan	A/Japan/842/2009
NA	KC459625	H1N1	11/19/2009	Japan	A/Japan/889/2009
NA	KC457980	H1N1	11/19/2009	Japan	A/Japan/828/2009
NA	KC459716	H1N1	11/19/2009	Japan	A/Japan/826/2009
NA	KC459688	H1N1	11/19/2009	Japan	A/Japan/833/2009
NA	KC459626	H1N1	11/19/2009	Japan	A/Japan/889/2009
NA	KC459644	H1N1	11/19/2009	Japan	A/Japan/842/2009
NA	KC459692	H1N1	11/19/2009	Japan	A/Japan/832/2009
NA	KC459628	H1N1	11/19/2009	Japan	A/Japan/889/2009
NA	KC459604	H1N1	11/19/2009	Japan	A/Japan/889/2009
NA	KC457983	H1N1	11/19/2009	Japan	A/Japan/833/2009
NA	KC459690	H1N1	11/19/2009	Japan	A/Japan/832/2009
NA	KC457986	H1N1	11/19/2009	Japan	A/Japan/842/2009
NA	KC459691	H1N1	11/19/2009	Japan	A/Japan/832/2009
NA	KC459609	H1N1	11/19/2009	Japan	A/Japan/889/2009
NA	KC459706	H1N1	11/19/2009	Japan	A/Japan/826/2009
NA	KC459718	H1N1	11/19/2009	Japan	A/Japan/826/2009
NA	KC457979	H1N1	11/19/2009	Japan	A/Japan/827/2009
NA	KC459650	H1N1	11/19/2009	Japan	A/Japan/842/2009
NA	KC459634	H1N1	11/19/2009	Japan	A/Japan/889/2009
NA	KC457987	H1N1	11/19/2009	Japan	A/Japan/858/2009
NA	KC459670	H1N1	11/19/2009	Japan	A/Japan/833/2009
NA	KC459614	H1N1	11/19/2009	Japan	A/Japan/889/2009
NA	KC459619	H1N1	11/19/2009	Japan	A/Japan/889/2009
NA	CY067266	H1N1	11/19/2009	Kazakhsta	A/Almaty/347/2009(H1N1)
NA	CY067264	H1N1	11/19/2009	Kazakhsta	A/Almaty/347/2009(H1N1)
NA	KC459673	H1N1	11/19/2009	Japan	A/Japan/833/2009
NA	KC459686	H1N1	11/19/2009	Japan	A/Japan/833/2009
NA	KC457990	H1N1	11/19/2009	Japan	A/Japan/867/2009
NA	KC459684	H1N1	11/19/2009	Japan	A/Japan/833/2009
NA	KC459610	H1N1	11/19/2009	Japan	A/Japan/889/2009
NA	KC458000	H1N1	11/19/2009	Japan	A/Japan/888/2009
NA	KC457994	H1N1	11/19/2009	Japan	A/Japan/874/2009
NA	KC459603	H1N1	11/19/2009	Japan	A/Japan/889/2009
NA	KC459710	H1N1	11/19/2009	Japan	A/Japan/826/2009
NA	KC459714	H1N1	11/19/2009	Japan	A/Japan/826/2009
NA	KC459631	H1N1	11/19/2009	Japan	A/Japan/889/2009
NA	KC459669	H1N1	11/19/2009	Japan	A/Japan/833/2009
NA	KC459671	H1N1	11/19/2009	Japan	A/Japan/833/2009
NA	KC459657	H1N1	11/19/2009	Japan	A/Japan/842/2009
NA	KC459641	H1N1	11/19/2009	Japan	A/Japan/842/2009
NA	KC459649	H1N1	11/19/2009	Japan	A/Japan/842/2009
NA	KC459704	H1N1	11/19/2009	Japan	A/Japan/826/2009
NA	KC459707	H1N1	11/19/2009	Japan	A/Japan/826/2009
NA	KC459715	H1N1	11/19/2009	Japan	A/Japan/826/2009
NA	KC459615	H1N1	11/19/2009	Japan	A/Japan/889/2009

NA	KC459620	H1N1	11/19/2009	Japan	A/Japan/889/2009
NA	KC459621	H1N1	11/19/2009	Japan	A/Japan/889/2009
NA	KC459638	H1N1	11/19/2009	Japan	A/Japan/842/2009
NA	KC459639	H1N1	11/19/2009	Japan	A/Japan/842/2009
NA	KC459680	H1N1	11/19/2009	Japan	A/Japan/833/2009
NA	KC459698	H1N1	11/19/2009	Japan	A/Japan/832/2009
NA	KC459607	H1N1	11/19/2009	Japan	A/Japan/889/2009
NA	KC459633	H1N1	11/19/2009	Japan	A/Japan/889/2009
NA	KC459678	H1N1	11/19/2009	Japan	A/Japan/833/2009
NA	KC459694	H1N1	11/19/2009	Japan	A/Japan/832/2009
NA	KC459697	H1N1	11/19/2009	Japan	A/Japan/832/2009
NA	KC459627	H1N1	11/19/2009	Japan	A/Japan/889/2009
NA	KC459647	H1N1	11/19/2009	Japan	A/Japan/842/2009
NA	KC457975	H1N1	11/19/2009	Japan	A/Japan/821/2009
NA	KC459656	H1N1	11/19/2009	Japan	A/Japan/842/2009
NA	KC459672	H1N1	11/19/2009	Japan	A/Japan/833/2009
NA	KC459693	H1N1	11/19/2009	Japan	A/Japan/832/2009
NA	KC459695	H1N1	11/19/2009	Japan	A/Japan/832/2009
NA	KC457981	H1N1	11/19/2009	Japan	A/Japan/830/2009
NA	KC457991	H1N1	11/19/2009	Japan	A/Japan/870/2009
NA	KC457997	H1N1	11/19/2009	Japan	A/Japan/881/2009
NA	KC457995	H1N1	11/19/2009	Japan	A/Japan/876/2009
NA	KC459661	H1N1	11/19/2009	Japan	A/Japan/842/2009
NA	KC459667	H1N1	11/19/2009	Japan	A/Japan/833/2009
NA	KC459611	H1N1	11/19/2009	Japan	A/Japan/889/2009
NA	KC459613	H1N1	11/19/2009	Japan	A/Japan/889/2009
NA	KC459654	H1N1	11/19/2009	Japan	A/Japan/842/2009
NA	KC457999	H1N1	11/19/2009	Japan	A/Japan/887/2009
NA	KC457977	H1N1	11/19/2009	Japan	A/Japan/825/2009
NA	KC459618	H1N1	11/19/2009	Japan	A/Japan/889/2009
NA	KC459630	H1N1	11/19/2009	Japan	A/Japan/889/2009
NA	KC459702	H1N1	11/19/2009	Japan	A/Japan/832/2009
NA	KC459709	H1N1	11/19/2009	Japan	A/Japan/826/2009
NA	KC459664	H1N1	11/19/2009	Japan	A/Japan/842/2009
NA	KC459616	H1N1	11/19/2009	Japan	A/Japan/889/2009
NA	KC459645	H1N1	11/19/2009	Japan	A/Japan/842/2009
NA	KC457989	H1N1	11/19/2009	Japan	A/Japan/864/2009
NA	KC459640	H1N1	11/19/2009	Japan	A/Japan/842/2009
NA	KC459675	H1N1	11/19/2009	Japan	A/Japan/833/2009
NA	KC459711	H1N1	11/19/2009	Japan	A/Japan/826/2009
NA	KC459696	H1N1	11/19/2009	Japan	A/Japan/832/2009
NA	KC457985	H1N1	11/19/2009	Japan	A/Japan/841/2009
NA	KC457978	H1N1	11/19/2009	Japan	A/Japan/826/2009
NA	KC457993	H1N1	11/19/2009	Japan	A/Japan/873/2009
NA	CY064435	H1N1	11/19/2009	China	A/Guangdong/1240/2009(H1N1)
NA	KC780208	H1N1	11/19/2009	USA	A/Oregon/37/2009
NA	CY066705	H1N1	11/19/2009	Austria	A/Vienna/INS291/2009(H1N1)
NA	CY066633	H1N1	11/19/2009	Germany	A/Bonn/INS277/2009(H1N1)
NA	HQ247714	H1N1	11/19/2009	Finland	A/Finland/687/2009
NA	HQ247704	H1N1	11/19/2009	Finland	A/Finland/677/2009
NA	HQ247677	H1N1	11/19/2009	Finland	A/Finland/648/2009
NA	AB704424	H1N1	11/19/2009	Japan	A/Gunma/262/2009
NA	CY071788	H1N1	11/19/2009	Mexico	A/Mexico City/WR1765N/2009(H1N1)
NA	CY067749	H1N1	11/19/2009	United Ki	A/England/94840152/2009

NA	CY071225	H1N1	11/19/2009	Norway	A/Oslo/INS404/2009(H1N1)
NA	HQ214318	H1N1	11/19/2009	Kenya	A/Kenya/0071/2009(H1N1)
NA	CY083146	H1N1	11/19/2009	Kyrgyzsta	A/Bishkek/WRAIR0883N/2009
NA	CY066062	H1N1	11/19/2009	Japan	A/Gunma/09G015/2009
NA	CY063301	H1N1	11/19/2009	USA	A/Wisconsin/629-D00859/2009(H1N1)
NA	CY123264	H1N1	11/19/2009	Singapore	A/Singapore/GP4407/2009
NA	CY100490	H1N1	11/19/2009	Mexico	A/Mexico City/INER14/2009
NA	JQ714187	H1N1	11/19/2009	China	A/Tianjintanggu/SWL432/2009
NA	CY062749	H1N1	11/19/2009	Spain	A/Madrid/INS129/2009(H1N1)
NA	KF687928	H1N1	11/19/2009	Cyprus	A/Cyprus/447/2009
NA	CY066937	H1N1	11/19/2009	Denmark	A/Aarhus/INS236/2009(H1N1)
NA	CY062741	H1N1	11/19/2009	Germany	A/Bonn/INS127/2009(H1N1)
NA	CY071588	H1N1	11/19/2009	Jordan	A/Amman/WR1335T/2009(H1N1)
NA	KC459624	H1N1	11/19/2009	Japan	A/Japan/889/2009
NA	KC459668	H1N1	11/19/2009	Japan	A/Japan/833/2009
NA	KC459635	H1N1	11/19/2009	Japan	A/Japan/889/2009
NA	KC459642	H1N1	11/19/2009	Japan	A/Japan/842/2009
NA	KC459608	H1N1	11/19/2009	Japan	A/Japan/889/2009
NA	KC459700	H1N1	11/19/2009	Japan	A/Japan/832/2009
NA	KC459623	H1N1	11/19/2009	Japan	A/Japan/889/2009
NA	KC459655	H1N1	11/19/2009	Japan	A/Japan/842/2009
NA	KC459689	H1N1	11/19/2009	Japan	A/Japan/832/2009
NA	KC459648	H1N1	11/19/2009	Japan	A/Japan/842/2009
NA	KC459651	H1N1	11/19/2009	Japan	A/Japan/842/2009
NA	KC459687	H1N1	11/19/2009	Japan	A/Japan/833/2009
NA	KC459705	H1N1	11/19/2009	Japan	A/Japan/826/2009
NA	KC458001	H1N1	11/19/2009	Japan	A/Japan/889/2009
NA	KC459701	H1N1	11/19/2009	Japan	A/Japan/832/2009
NA	KC459637	H1N1	11/19/2009	Japan	A/Japan/842/2009
NA	KC459663	H1N1	11/19/2009	Japan	A/Japan/842/2009
NA	KC459703	H1N1	11/19/2009	Japan	A/Japan/826/2009
NA	KC459713	H1N1	11/19/2009	Japan	A/Japan/826/2009
NA	KC457976	H1N1	11/19/2009	Japan	A/Japan/824/2009
NA	KC459699	H1N1	11/19/2009	Japan	A/Japan/832/2009
NA	KC459666	H1N1	11/19/2009	Japan	A/Japan/833/2009
NA	KC459708	H1N1	11/19/2009	Japan	A/Japan/826/2009
NA	KC459605	H1N1	11/19/2009	Japan	A/Japan/889/2009
NA	KC459712	H1N1	11/19/2009	Japan	A/Japan/826/2009
NA	CY064429	H1N1	11/19/2009	China	A/Guangdong/1075/2009(H1N1)
NA	CY067720	H1N1	11/19/2009	United Ki	A/England/00380018/2009
NA	CY064443	H1N1	11/19/2009	China	A/Guangdong/1252/2009(H1N1)
NA	CY064437	H1N1	11/19/2009	China	A/Guangdong/1241/2009(H1N1)
NA	CY064453	H1N1	11/19/2009	China	A/Guangdong/1271/2009(H1N1)
NA	CY065858	H1N1	11/19/2009	Netherlar	A/Netherlands/2229/2009
NA	CY063309	H1N1	11/19/2009	USA	A/Wisconsin/629-D01572/2009(H1N1)
NA	KC459682	H1N1	11/19/2009	Japan	A/Japan/833/2009
NA	KC459679	H1N1	11/19/2009	Japan	A/Japan/833/2009
NA	KC457998	H1N1	11/19/2009	Japan	A/Japan/886/2009
NA	KC459660	H1N1	11/19/2009	Japan	A/Japan/842/2009
NA	KC459606	H1N1	11/19/2009	Japan	A/Japan/889/2009
NA	KC459612	H1N1	11/19/2009	Japan	A/Japan/889/2009
NA	KC459646	H1N1	11/19/2009	Japan	A/Japan/842/2009
NA	KC459659	H1N1	11/19/2009	Japan	A/Japan/842/2009
NA	KC459665	H1N1	11/19/2009	Japan	A/Japan/842/2009

NA	KC459597	H1N1	11/20/2009	Japan	A/Japan/896/2009
NA	KC459589	H1N1	11/20/2009	Japan	A/Japan/896/2009
NA	KC459539	H1N1	11/20/2009	Japan	A/Japan/921/2009
NA	CY064441	H1N1	11/20/2009	China	A/Guangdong/1250/2009(H1N1)
NA	CY064423	H1N1	11/20/2009	China	A/Guangdong/1069/2009(H1N1)
NA	HM230695	H1N1	11/20/2009	China	A/Zhoushan/1/2009(H1N1)
NA	KC459566	H1N1	11/20/2009	Japan	A/Japan/921/2009
NA	KC459576	H1N1	11/20/2009	Japan	A/Japan/896/2009
NA	KC459595	H1N1	11/20/2009	Japan	A/Japan/896/2009
NA	KC458008	H1N1	11/20/2009	Japan	A/Japan/908/2009
NA	KC459567	H1N1	11/20/2009	Japan	A/Japan/921/2009
NA	KC459580	H1N1	11/20/2009	Japan	A/Japan/896/2009
NA	KC459537	H1N1	11/20/2009	Japan	A/Japan/921/2009
NA	KC459541	H1N1	11/20/2009	Japan	A/Japan/921/2009
NA	KC459583	H1N1	11/20/2009	Japan	A/Japan/896/2009
NA	KC459593	H1N1	11/20/2009	Japan	A/Japan/896/2009
NA	KC459549	H1N1	11/20/2009	Japan	A/Japan/921/2009
NA	KC459558	H1N1	11/20/2009	Japan	A/Japan/921/2009
NA	KC459587	H1N1	11/20/2009	Japan	A/Japan/896/2009
NA	KC459554	H1N1	11/20/2009	Japan	A/Japan/921/2009
NA	KC459555	H1N1	11/20/2009	Japan	A/Japan/921/2009
NA	KC459600	H1N1	11/20/2009	Japan	A/Japan/896/2009
NA	KC459577	H1N1	11/20/2009	Japan	A/Japan/896/2009
NA	KC459584	H1N1	11/20/2009	Japan	A/Japan/896/2009
NA	KC459594	H1N1	11/20/2009	Japan	A/Japan/896/2009
NA	KC459601	H1N1	11/20/2009	Japan	A/Japan/896/2009
NA	KC458009	H1N1	11/20/2009	Japan	A/Japan/912/2009
NA	KC459582	H1N1	11/20/2009	Japan	A/Japan/896/2009
NA	KC459592	H1N1	11/20/2009	Japan	A/Japan/896/2009
NA	KC459545	H1N1	11/20/2009	Japan	A/Japan/921/2009
NA	CY066649	H1N1	11/20/2009	Germany	A/Bonn/INS279/2009(H1N1)
NA	CY066681	H1N1	11/20/2009	Denmark	A/Hvidovre/INS288/2009(H1N1)
NA	CY056949	H1N1	11/20/2009	Denmark	A/Aarhus/INS82/2009(H1N1)
NA	CY066641	H1N1	11/20/2009	Germany	A/Bonn/INS278/2009(H1N1)
NA	HQ247675	H1N1	11/20/2009	Finland	A/Finland/646/2009
NA	HM568034	H1N1	11/20/2009	United Ki	A/England/902/2009
NA	HM567970	H1N1	11/20/2009	United Ki	A/England/876/2009
NA	CY067721	H1N1	11/20/2009	United Ki	A/England/00380020/2009
NA	CY064455	H1N1	11/20/2009	China	A/Guangdong/1275/2009(H1N1)
NA	CY064439	H1N1	11/20/2009	China	A/Guangdong/1249/2009(H1N1)
NA	CY064445	H1N1	11/20/2009	China	A/Guangdong/1260/2009(H1N1)
NA	CY066038	H1N1	11/20/2009	Japan	A/Fukushima/09FY016/2009
NA	CY067750	H1N1	11/20/2009	United Ki	A/England/94840153/2009
NA	HM754223	H1N1	11/20/2009	China	A/Jiangsu/1/2009(H1N1)
NA	HM072411	H1N1	11/20/2009	Taiwan	A/Taiwan/2253/2009
NA	KC459581	H1N1	11/20/2009	Japan	A/Japan/896/2009
NA	KC459557	H1N1	11/20/2009	Japan	A/Japan/921/2009
NA	KC459564	H1N1	11/20/2009	Japan	A/Japan/921/2009
NA	KC459585	H1N1	11/20/2009	Japan	A/Japan/896/2009
NA	KC459553	H1N1	11/20/2009	Japan	A/Japan/921/2009
NA	KC458013	H1N1	11/20/2009	Japan	A/Japan/921/2009
NA	KC458003	H1N1	11/20/2009	Japan	A/Japan/895/2009
NA	KC458004	H1N1	11/20/2009	Japan	A/Japan/896/2009
NA	KC459534	H1N1	11/20/2009	Japan	A/Japan/921/2009

NA	KC459535	H1N1	11/20/2009	Japan	A/Japan/921/2009
NA	KC459543	H1N1	11/20/2009	Japan	A/Japan/921/2009
NA	KC459569	H1N1	11/20/2009	Japan	A/Japan/921/2009
NA	KC459571	H1N1	11/20/2009	Japan	A/Japan/921/2009
NA	KC459574	H1N1	11/20/2009	Japan	A/Japan/921/2009
NA	KC459596	H1N1	11/20/2009	Japan	A/Japan/896/2009
NA	KC459544	H1N1	11/20/2009	Japan	A/Japan/921/2009
NA	KC459560	H1N1	11/20/2009	Japan	A/Japan/921/2009
NA	KC458002	H1N1	11/20/2009	Japan	A/Japan/890/2009
NA	KC458010	H1N1	11/20/2009	Japan	A/Japan/917/2009
NA	KC459546	H1N1	11/20/2009	Japan	A/Japan/921/2009
NA	KC459552	H1N1	11/20/2009	Japan	A/Japan/921/2009
NA	KC459570	H1N1	11/20/2009	Japan	A/Japan/921/2009
NA	KC459586	H1N1	11/20/2009	Japan	A/Japan/896/2009
NA	KC459561	H1N1	11/20/2009	Japan	A/Japan/921/2009
NA	KC459559	H1N1	11/20/2009	Japan	A/Japan/921/2009
NA	KC458005	H1N1	11/20/2009	Japan	A/Japan/898/2009
NA	KC459563	H1N1	11/20/2009	Japan	A/Japan/921/2009
NA	KC458006	H1N1	11/20/2009	Japan	A/Japan/906/2009
NA	KC459568	H1N1	11/20/2009	Japan	A/Japan/921/2009
NA	KC459578	H1N1	11/20/2009	Japan	A/Japan/896/2009
NA	KC459551	H1N1	11/20/2009	Japan	A/Japan/921/2009
NA	KC459591	H1N1	11/20/2009	Japan	A/Japan/896/2009
NA	KC459565	H1N1	11/20/2009	Japan	A/Japan/921/2009
NA	KC459572	H1N1	11/20/2009	Japan	A/Japan/921/2009
NA	KC459556	H1N1	11/20/2009	Japan	A/Japan/921/2009
NA	KC458011	H1N1	11/20/2009	Japan	A/Japan/919/2009
NA	KC459573	H1N1	11/20/2009	Japan	A/Japan/921/2009
NA	KC459590	H1N1	11/20/2009	Japan	A/Japan/896/2009
NA	KC459538	H1N1	11/20/2009	Japan	A/Japan/921/2009
NA	KC459536	H1N1	11/20/2009	Japan	A/Japan/921/2009
NA	KC458012	H1N1	11/20/2009	Japan	A/Japan/920/2009
NA	KC459575	H1N1	11/20/2009	Japan	A/Japan/921/2009
NA	KC459579	H1N1	11/20/2009	Japan	A/Japan/896/2009
NA	HM625663	H1N1	11/20/2009	Italy	A/Rome/PTV17/2009
NA	KC458007	H1N1	11/20/2009	Japan	A/Japan/907/2009
NA	KC459540	H1N1	11/20/2009	Japan	A/Japan/921/2009
NA	KC459602	H1N1	11/20/2009	Japan	A/Japan/896/2009
NA	KC459547	H1N1	11/20/2009	Japan	A/Japan/921/2009
NA	KC459550	H1N1	11/20/2009	Japan	A/Japan/921/2009
NA	KC459548	H1N1	11/20/2009	Japan	A/Japan/921/2009
NA	LC093007	H1N1	11/20/2009	Saudi Ara	A/Riyadh/4216/2009
NA	HM568018	H1N1	11/20/2009	United Ki	A/England/872/2009
NA	CY089357	H1N1	11/20/2009	USA	A/Boston/690/2009
NA	HQ247717	H1N1	11/20/2009	Finland	A/Finland/690/2009
NA	CY083739	H1N1	11/20/2009	Spain	A/Madrid/INS130/2009(H1N1)
NA	CY063600	H1N1	11/20/2009	Germany	A/Bonn/INS128/2009(H1N1)
NA	HQ247716	H1N1	11/20/2009	Finland	A/Finland/689/2009
NA	CY062797	H1N1	11/20/2009	Denmark	A/Hvidovre/INS141/2009(H1N1)
NA	CY071444	H1N1	11/20/2009	Kyrgyzsta	A/Bishkek/WR0882N/2009(H1N1)
NA	KC459588	H1N1	11/20/2009	Japan	A/Japan/896/2009
NA	KC459598	H1N1	11/20/2009	Japan	A/Japan/896/2009
NA	KC459599	H1N1	11/20/2009	Japan	A/Japan/896/2009
NA	KC459542	H1N1	11/20/2009	Japan	A/Japan/921/2009

NA	KC459562	H1N1	11/20/2009	Japan	A/Japan/921/2009
NA	CY060464	H1N1	11/21/2009	South Ko	A/Gyeongnam/1819/2009(H1N1)
NA	CY067041	H1N1	11/21/2009	Denmark	A/Aarhus/INS252/2009(H1N1)
NA	CY089365	H1N1	11/21/2009	USA	A/Boston/691/2009
NA	CY058827	H1N1	11/21/2009	China	A/Guangdong/1782/2009(H1N1)
NA	JF429397	H1N1	11/21/2009	France	A/Lyon/47.942/2009
NA	CY060941	H1N1	11/21/2009	USA	A/Texas/JMS373/2009(H1N1)
NA	KC514522	H1N1	11/21/2009	Turkey	A/Istanbul/3720/2009
NA	CY064451	H1N1	11/21/2009	China	A/Guangdong/1270/2009(H1N1)
NA	CY064425	H1N1	11/21/2009	China	A/Guangdong/1070/2009(H1N1)
NA	CY123274	H1N1	11/21/2009	Singapore	A/Singapore/GP4425/2009
NA	AB704512	H1N1	11/21/2009	Japan	A/Yamaguchi/217/2009
NA	HQ247674	H1N1	11/21/2009	Finland	A/Finland/645/2009
NA	CY123276	H1N1	11/21/2009	Singapore	A/Singapore/GP4428/2009
NA	KM213296	H1N1	11/21/2009	Oman	A/Oman/SQUH-4/2009
NA	HM581908	H1N1	11/21/2009	Iran	A/Iran/20001/2009(H1N1)
NA	HM581918	H1N1	11/21/2009	Iran	A/Iran/15583/2009(H1N1)
NA	CY123278	H1N1	11/21/2009	Singapore	A/Singapore/GP4436/2009
NA	CY067033	H1N1	11/21/2009	Denmark	A/Aarhus/INS251/2009
NA	CY088587	H1N1	11/21/2009	USA	A/Boston/694/2009
NA	CY060467	H1N1	11/22/2009	South Ko	A/Gyeongnam/1820/2009(H1N1)
NA	CY084456	H1N1	11/22/2009	USA	A/New York/6418/2009
NA	CY067049	H1N1	11/22/2009	Denmark	A/Aarhus/INS253/2009(H1N1)
NA	CY058821	H1N1	11/22/2009	China	A/Guangdong/1331/2009(H1N1)
NA	HM581916	H1N1	11/22/2009	Iran	A/Iran/16245/2009(H1N1)
NA	HM581914	H1N1	11/22/2009	Iran	A/Iran/16273/2009(H1N1)
NA	CY128365	H1N1	11/22/2009	Viet Nam	A/Viet Nam/15032004/2009
NA	GU560018	H1N1	11/22/2009	Russia	A/Tomsk/06/2009(H1N1)
NA	CY056725	H1N1	11/22/2009	USA	A/New York/6473/2009(H1N1)
NA	CY057896	H1N1	11/22/2009	USA	A/Wisconsin/629-D00908/2009(H1N1)
NA	CY057888	H1N1	11/22/2009	USA	A/Wisconsin/629-D00533/2009(H1N1)
NA	CY055341	H1N1	11/22/2009	Italy	A/Rome/630/2009(H1N1)
NA	KM213300	H1N1	11/22/2009	Oman	A/Oman/SQUH-10/2009
NA	CY067751	H1N1	11/22/2009	United Ki	A/England/94920013/2009
NA	CY064447	H1N1	11/22/2009	China	A/Guangdong/1262/2009(H1N1)
NA	CY064449	H1N1	11/22/2009	China	A/Guangdong/1266/2009(H1N1)
NA	CY053380	H1N1	11/23/2009	Spain	A/Catalonia/S1938/2009(H1N1)
NA	CY072248	H1N1	11/23/2009	Denmark	A/Odense/INS307/2009(H1N1)
NA	CY053389	H1N1	11/23/2009	Spain	A/Catalonia/S1946/2009(H1N1)
NA	KX651746	H1N1	11/23/2009	USA	A/USA/525-1T/2009
NA	CY087966	H1N1	11/23/2009	Thailand	A/Thailand/CU-H982/2009
NA	HM625665	H1N1	11/23/2009	Italy	A/Rome/PTV19/2009
NA	HM625664	H1N1	11/23/2009	Italy	A/Rome/PTV18/2009
NA	CY067268	H1N1	11/23/2009	Kazakhsta	A/Almaty/352/2009(H1N1)
NA	CY053374	H1N1	11/23/2009	Spain	A/Catalonia/S1935/2009(H1N1)
NA	CY053383	H1N1	11/23/2009	Spain	A/Catalonia/S1943/2009(H1N1)
NA	CY053392	H1N1	11/23/2009	Spain	A/Catalonia/S1947/2009(H1N1)
NA	CY053377	H1N1	11/23/2009	Spain	A/Catalonia/S1937/2009(H1N1)
NA	HM072412	H1N1	11/23/2009	Taiwan	A/Taiwan/7949/2009
NA	CY083912	H1N1	11/23/2009	Denmark	A/Aalborg/INS132/2009(H1N1)
NA	CY056845	H1N1	11/23/2009	USA	A/New York/6868/2009(H1N1)
NA	CY056757	H1N1	11/23/2009	USA	A/New York/6663/2009(H1N1)
NA	CY057920	H1N1	11/23/2009	USA	A/Wisconsin/629-D01330/2009(H1N1)
NA	GU571156	H1N1	11/23/2009	USA	A/Bethesda/NIH106-D32/2009(H1N1)

NA	HQ256661	H1N1	11/23/2009	Myanmar	A/Myanmar/M542/2009(H1N1)
NA	CY073160	H1N1	11/23/2009	Nigeria	A/Lagos/WRAIR1982N/2009(H1N1)
NA	CY058835	H1N1	11/23/2009	China	A/Guangdong/2282/2009(H1N1)
NA	CY055347	H1N1	11/23/2009	Italy	A/Rome/633/2009(H1N1)
NA	CY058718	H1N1	11/23/2009	USA	A/Texas/JMS375/2009(H1N1)
NA	CY058823	H1N1	11/23/2009	China	A/Guangdong/1748/2009(H1N1)
NA	CY066905	H1N1	11/23/2009	USA	A/District of Columbia/INS232/2009
NA	CY056733	H1N1	11/23/2009	USA	A/New York/6546/2009(H1N1)
NA	CY057904	H1N1	11/23/2009	USA	A/Wisconsin/629-D02448/2009(H1N1)
NA	CY057912	H1N1	11/23/2009	USA	A/Wisconsin/629-D00134/2009(H1N1)
NA	CY055343	H1N1	11/23/2009	Italy	A/Rome/631/2009(H1N1)
NA	CY055345	H1N1	11/23/2009	Italy	A/Rome/632/2009(H1N1)
NA	HM568026	H1N1	11/23/2009	United Ki	A/England/862/2009
NA	HM567962	H1N1	11/23/2009	United Ki	A/England/865/2009
NA	KP019728	H1N1	11/23/2009	China	A/FuZhou/SWL12025/2009
NA	CY065802	H1N1	11/23/2009	Netherlar	A/Netherlands/2445b/2009
NA	CY123270	H1N1	11/23/2009	Singapore	A/Singapore/GP4419/2009
NA	JF429400	H1N1	11/23/2009	France	A/Lyon/48.49/2009
NA	CY123863	H1N1	11/23/2009	Singapore	A/Singapore/ON2344/2009
NA	CY123861	H1N1	11/23/2009	Singapore	A/Singapore/ON2326/2009
NA	CY123280	H1N1	11/23/2009	Singapore	A/Singapore/GP4440/2009
NA	CY123282	H1N1	11/23/2009	Singapore	A/Singapore/GP4451/2009
NA	HM189327	H1N1	11/23/2009	Russia	A/Moscow oblast/CRIE-KMA/2009
NA	HM189329	H1N1	11/23/2009	Russia	A/Ulyanovsk/CRIE-BAA/2009
NA	HM189339	H1N1	11/23/2009	Russia	A/Moscow/CRIE-CHSN/2009
NA	CY066945	H1N1	11/23/2009	Denmark	A/Aarhus/INS237/2009(H1N1)
NA	CY069148	H1N1	11/23/2009	Germany	A/Frankfurt/INS301/2009(H1N1)
NA	CY075622	H1N1	11/23/2009	USA	A/Boston/698/2009(H1N1)
NA	CY066889	H1N1	11/23/2009	USA	A/District of Columbia/INS230/2009(I
NA	HM568010	H1N1	11/23/2009	United Ki	A/England/854/2009
NA	CY053386	H1N1	11/23/2009	Spain	A/Catalonia/S1944/2009(H1N1)
NA	HM581912	H1N1	11/24/2009	Iran	A/Iran/16677/2009(H1N1)
NA	HM581909	H1N1	11/24/2009	Iran	A/Iran/16751/2009(H1N1)
NA	CY056749	H1N1	11/24/2009	USA	A/New York/6607/2009(H1N1)
NA	CY056781	H1N1	11/24/2009	USA	A/New York/6675/2009(H1N1)
NA	HM581910	H1N1	11/24/2009	Iran	A/Iran/16743/2009(H1N1)
NA	CY058726	H1N1	11/24/2009	USA	A/Texas/JMS376/2009(H1N1)
NA	CY066713	H1N1	11/24/2009	Austria	A/Vienna/INS293/2009(H1N1)
NA	CY066657	H1N1	11/24/2009	Spain	A/Madrid/INS281/2009(H1N1)
NA	KC780706	H1N1	11/24/2009	USA	A/California/41/2009
NA	HQ247673	H1N1	11/24/2009	Finland	A/Finland/644/2009
NA	HQ247719	H1N1	11/24/2009	Finland	A/Finland/692/2009
NA	HM568002	H1N1	11/24/2009	United Ki	A/England/900/2009
NA	KT181554	H1N1	11/24/2009	Singapore	A/Singapore/DMS53/2009
NA	HM189348	H1N1	11/24/2009	Russia	A/Moscow oblast/CRIE-ZNF/2009
NA	HM189342	H1N1	11/24/2009	Russia	A/Astrakhan/CRIE-CHRM/2009
NA	HM189344	H1N1	11/24/2009	Russia	A/Ivanovo/CRIE-RNA/2009
NA	CY123869	H1N1	11/24/2009	Singapore	A/Singapore/ON2370/2009
NA	CY066953	H1N1	11/24/2009	Denmark	A/Aarhus/INS238/2009(H1N1)
NA	CY066913	H1N1	11/24/2009	USA	A/Pensacola/INS233/2009
NA	CY092440	H1N1	11/24/2009	Australia	A/Sydney/DD3-02/2009(H1N1)
NA	CY071249	H1N1	11/24/2009	Spain	A/Madrid/INS407/2009(H1N1)
NA	CY084464	H1N1	11/24/2009	USA	A/New York/6537/2009
NA	AB704472	H1N1	11/24/2009	Japan	A/Tochigi/445/2009

NA	HQ247718	H1N1	11/24/2009	Finland	A/Finland/691/2009
NA	CY064433	H1N1	11/24/2009	China	A/Guangdong/1083/2009(H1N1)
NA	CY069124	H1N1	11/24/2009	Spain	A/Madrid/INS298/2009(H1N1)
NA	KC780814	H1N1	11/24/2009	USA	A/Colorado/15/2009
NA	CY062693	H1N1	11/24/2009	Denmark	A/Aarhus/INS116/2009(H1N1)
NA	GU571153	H1N1	11/24/2009	USA	A/Bethesda/NIH107-D31/2009(H1N1)
NA	CY057936	H1N1	11/24/2009	USA	A/Wisconsin/629-D00968/2009(H1N1)
NA	CY057928	H1N1	11/24/2009	USA	A/Wisconsin/629-D01190/2009(H1N1)
NA	CY066273	H1N1	11/24/2009	USA	A/California/VRDL112/2009(H1N1)
NA	CY066281	H1N1	11/24/2009	USA	A/California/VRDL113/2009(H1N1)
NA	CY066257	H1N1	11/24/2009	USA	A/California/VRDL110/2009(H1N1)
NA	CY072256	H1N1	11/24/2009	Denmark	A/Odense/INS308/2009(H1N1)
NA	CY066265	H1N1	11/24/2009	USA	A/California/VRDL111/2009(H1N1)
NA	CY066249	H1N1	11/24/2009	USA	A/California/VRDL109/2009(H1N1)
NA	CY058829	H1N1	11/24/2009	China	A/Guangdong/1998/2009(H1N1)
NA	CY066977	H1N1	11/24/2009	Denmark	A/Aarhus/INS242/2009(H1N1)
NA	LC093032	H1N1	11/24/2009	Saudi Ara	A/Riyadh/4309/2009
NA	LC093037	H1N1	11/24/2009	Saudi Ara	A/Riyadh/4312/2009
NA	HM625668	H1N1	11/24/2009	Italy	A/Rome/PTV22/2009
NA	HM625666	H1N1	11/24/2009	Italy	A/Rome/PTV20/2009
NA	HM625667	H1N1	11/24/2009	Italy	A/Rome/PTV21/2009
NA	CY053395	H1N1	11/24/2009	Spain	A/Catalonia/S1952/2009(H1N1)
NA	HM567954	H1N1	11/25/2009	United Ki	A/England/857/2009
NA	CY066024	H1N1	11/25/2009	Japan	A/Niigata/09F070/2009
NA	JQ173166	H1N1	11/25/2009	Finland	A/Helsinki/P18/2009(H1N1)
NA	CY056829	H1N1	11/25/2009	USA	A/New York/6846/2009(H1N1)
NA	CY067009	H1N1	11/25/2009	Germany	A/Munich/INS247/2009
NA	CY058825	H1N1	11/25/2009	China	A/Guangdong/1759/2009(H1N1)
NA	HM101150	H1N1	11/25/2009	Russia	A/Tver/IIV-183/2009(H1N1)
NA	KX651747	H1N1	11/25/2009	USA	A/USA/525-3T/2009
NA	HM625670	H1N1	11/25/2009	Italy	A/Rome/PTV24/2009
NA	HM625669	H1N1	11/25/2009	Italy	A/Rome/PTV23/2009
NA	JF513113	H1N1	11/25/2009	Italy	A/Roma/ISS9825/2009
NA	CY052025	H1N1	11/25/2009	Spain	A/Catalonia/NS7267/2009(H1N1)
NA	GU562452	H1N1	11/25/2009	Russia	A/Perm/01/2009(H1N1)
NA	CY100495	H1N1	11/25/2009	Mexico	A/Mexico City/INER15/2009
NA	CY123288	H1N1	11/25/2009	Singapore	A/Singapore/GP4539/2009
NA	AB704448	H1N1	11/25/2009	Japan	A/Gunma/287/2009
NA	CY056821	H1N1	11/25/2009	USA	A/New York/6810/2009(H1N1)
NA	KC780944	H1N1	11/25/2009	USA	A/Washington/72/2009
NA	CY066689	H1N1	11/25/2009	Denmark	A/Hvidovre/INS289/2009(H1N1)
NA	HQ247720	H1N1	11/25/2009	Finland	A/Finland/693/2009
NA	HM568042	H1N1	11/25/2009	United Ki	A/England/898/2009
NA	HM189340	H1N1	11/25/2009	Russia	A/Ivanovo/CRIE-KOE/2009
NA	HM189322	H1N1	11/25/2009	Russia	A/Arkhangelsk/CRIE-GNY/2009
NA	JF429398	H1N1	11/25/2009	France	A/Lyon/48.425/2009
NA	AB649911	H1N1	11/25/2009	Japan	A/Toyama/383/2009
NA	AB704440	H1N1	11/25/2009	Japan	A/Gunma/267/2009
NA	CY123865	H1N1	11/25/2009	Singapore	A/Singapore/ON2349/2009
NA	CY067752	H1N1	11/25/2009	United Ki	A/England/94920015/2009
NA	CY067185	H1N1	11/25/2009	Austria	A/Vienna/INS294/2009
NA	CY072224	H1N1	11/25/2009	Germany	A/Frankfurt/INS302/2009(H1N1)
NA	HM567994	H1N1	11/25/2009	United Ki	A/England/859/2009
NA	CY083763	H1N1	11/25/2009	Denmark	A/Aarhus/INS239/2009

NA	CY066124	H1N1	11/25/2009	Japan	A/Hyogo/09K300/2009
NA	HQ247729	H1N1	11/25/2009	Finland	A/Finland/703/2009
NA	CY066064	H1N1	11/25/2009	Japan	A/Gunma/09G024/2009
NA	CY058837	H1N1	11/25/2009	China	A/Guangdong/2361/2009(H1N1)
NA	CY069132	H1N1	11/26/2009	Spain	A/Madrid/INS299/2009(H1N1)
NA	CY066969	H1N1	11/26/2009	Denmark	A/Aarhus/INS241/2009(H1N1)
NA	CY057944	H1N1	11/26/2009	USA	A/Wisconsin/629-D00629/2009(H1N1)
NA	CY062805	H1N1	11/26/2009	Austria	A/Vienna/INS142/2009(H1N1)
NA	CY056741	H1N1	11/26/2009	USA	A/New York/6571/2009(H1N1)
NA	CY066961	H1N1	11/26/2009	Denmark	A/Aarhus/INS240/2009(H1N1)
NA	CY067790	H1N1	11/26/2009	Portugal	A/Lisboa/162/2009(H1N1)
NA	CY067754	H1N1	11/26/2009	Portugal	A/Lisboa/100/2009(H1N1)
NA	CY060949	H1N1	11/26/2009	USA	A/Texas/JMS379/2009(H1N1)
NA	CY052027	H1N1	11/26/2009	Spain	A/Catalonia/NS7362/2009(H1N1)
NA	MF196268	H1N1	11/26/2009	Iran	A/Tehran/88-9-26/2009
NA	CY060473	H1N1	11/26/2009	South Ko	A/Seoul/1829/2009(H1N1)
NA	KX651749	H1N1	11/26/2009	USA	A/USA/1459-1T/2009
NA	CY087851	H1N1	11/26/2009	Thailand	A/Thailand/CU-H1002/2009
NA	KP317302	H1N1	11/26/2009	India	A/Punjab/007/2009
NA	KP317304	H1N1	11/26/2009	India	A/Rajasthan/009/2009
NA	CY058734	H1N1	11/26/2009	USA	A/Texas/JMS377/2009(H1N1)
NA	CY058742	H1N1	11/26/2009	USA	A/Texas/JMS378/2009(H1N1)
NA	CY055352	H1N1	11/26/2009	Italy	A/Rome/636/2009(H1N1)
NA	CY055356	H1N1	11/26/2009	Italy	A/Rome/639/2009(H1N1)
NA	CY066114	H1N1	11/26/2009	Japan	A/Kyoto/09K158/2009
NA	KJ856232	H1N1	11/26/2009	Sri Lanka	A/Sri Lanka/24206/2009
NA	KT181555	H1N1	11/26/2009	Singapore	A/Singapore/DMS54/2009
NA	CY066040	H1N1	11/26/2009	Japan	A/Fukushima/09FY021/2009
NA	GU471693	H1N1	11/26/2009	China	A/Guangdong/1103/2009
NA	CY073751	H1N1	11/26/2009	Denmark	A/Copenhagen/INS434/2009(H1N1)
NA	GU471694	H1N1	11/26/2009	China	A/Guangdong/1106/2009
NA	GU471692	H1N1	11/26/2009	China	A/Guangdong/1104/2009
NA	GU471695	H1N1	11/26/2009	China	A/Guangdong/1105/2009
NA	CY072232	H1N1	11/26/2009	Germany	A/Frankfurt/INS303/2009(H1N1)
NA	CY083731	H1N1	11/26/2009	Germany	A/Bochum/INS120/2009(H1N1)
NA	CY088615	H1N1	11/26/2009	Germany	A/Frankfurt/INS93/2009
NA	CY062757	H1N1	11/26/2009	Spain	A/Madrid/INS131/2009(H1N1)
NA	CY062701	H1N1	11/27/2009	Denmark	A/Aarhus/INS118/2009(H1N1)
NA	CY058833	H1N1	11/27/2009	China	A/Guangdong/2281/2009(H1N1)
NA	HQ239875	H1N1	11/27/2009	Canada	A/Canada-MB/RV2018-10/2009
NA	CY060957	H1N1	11/27/2009	USA	A/Texas/JMS380/2009(H1N1)
NA	HQ286403	H1N1	11/27/2009	Japan	A/Kurume/14/2009
NA	HQ286405	H1N1	11/27/2009	Japan	A/Kurume/16/2009
NA	HQ286408	H1N1	11/27/2009	Japan	A/Kurume/19/2009
NA	HQ286402	H1N1	11/27/2009	Japan	A/Kurume/13/2009
NA	HQ286406	H1N1	11/27/2009	Japan	A/Kurume/17/2009
NA	HQ286409	H1N1	11/27/2009	Japan	A/Kurume/20/2009
NA	HQ286407	H1N1	11/27/2009	Japan	A/Kurume/18/2009
NA	HQ286410	H1N1	11/27/2009	Japan	A/Kurume/21/2009
NA	HQ286404	H1N1	11/27/2009	Japan	A/Kurume/15/2009
NA	HQ286401	H1N1	11/27/2009	Japan	A/Kurume/12/2009
NA	MF196259	H1N1	11/27/2009	Iran	A/Tehran/88-09-26-1/2009
NA	KX651748	H1N1	11/27/2009	USA	A/USA/525-5T/2009
NA	LC093038	H1N1	11/27/2009	Saudi Ara	A/Riyadh/4326/2009

NA	LC093041	H1N1	11/27/2009	Saudi Ara	A/Riyadh/4324/2009
NA	CY057952	H1N1	11/27/2009	USA	A/Wisconsin/629-D00050/2009(H1N1)
NA	CY057960	H1N1	11/27/2009	USA	A/Wisconsin/629-D00970/2009(H1N1)
NA	KC781065	H1N1	11/27/2009	USA	A/Virginia/25/2009
NA	KC780298	H1N1	11/27/2009	USA	A/Kansas/27/2009
NA	CY066697	H1N1	11/27/2009	Denmark	A/Hvidovre/INS290/2009(H1N1)
NA	KJ856068	H1N1	11/27/2009	Sri Lanka	A/Sri Lanka/2005/2009
NA	CY064457	H1N1	11/27/2009	China	A/Guangdong/1738/2009(H1N1)
NA	CY075630	H1N1	11/27/2009	USA	A/Boston/701/2009(H1N1)
NA	CY066166	H1N1	11/27/2009	Japan	A/Nagasaki/09N079/2009
NA	AB704504	H1N1	11/27/2009	Japan	A/Yamagata/674/2009
NA	GU592899	H1N1	11/28/2009	Kyrgyzsta	A/Bishkek/03/2009(H1N1)
NA	CY055363	H1N1	11/28/2009	Italy	A/Rome/644/2009(H1N1)
NA	CY067270	H1N1	11/28/2009	Kazakhsta	A/Almaty/373/2009(H1N1)
NA	CY060973	H1N1	11/28/2009	USA	A/Texas/JMS382/2009(H1N1)
NA	KX651639	H1N1	11/28/2009	France	A/France/1214-1T/2009
NA	CY060965	H1N1	11/28/2009	USA	A/Texas/JMS381/2009(H1N1)
NA	HQ247678	H1N1	11/28/2009	Finland	A/Finland/649/2009
NA	KR870134	H1N1	11/28/2009	India	A/Bangalore/10519-13/2009
NA	KC781102	H1N1	11/28/2009	USA	A/Washington/69/2009
NA	KX651750	H1N1	11/28/2009	USA	A/USA/1615-1T/2009
NA	CY067057	H1N1	11/28/2009	Denmark	A/Aarhus/INS254/2009(H1N1)
NA	CY057976	H1N1	11/29/2009	USA	A/Wisconsin/629-D01434/2009(H1N1)
NA	CY073248	H1N1	11/29/2009	Serbia	A/Belgrade/WRAIR2385T/2009(H1N1)
NA	CY090042	H1N1	11/29/2009	USA	A/Wisconsin/629-D01667/2009
NA	GU562466	H1N1	11/29/2009	China	A/Guangdong/1101/2009
NA	CY073240	H1N1	11/29/2009	Serbia	A/Belgrade/WRAIR2385N/2009(H1N1)
NA	CY088595	H1N1	11/29/2009	USA	A/Boston/702/2009
NA	CY057968	H1N1	11/29/2009	USA	A/Wisconsin/629-D01528/2009(H1N1)
NA	LC093027	H1N1	11/29/2009	Saudi Ara	A/Riyadh/4357/2009
NA	JX465468	H1N1	11/29/2009	South Ko	A/Gyeonggi/4873/2009
NA	CY117666	H1N1	11/29/2009	Mexico	A/Mexico/UASLP-028/2009
NA	CY060981	H1N1	11/29/2009	USA	A/Texas/JMS383/2009(H1N1)
NA	CY056853	H1N1	11/29/2009	USA	A/New York/6909/2009(H1N1)
NA	KF687932	H1N1	11/29/2009	Cyprus	A/Cyprus/504/2009
NA	KF687930	H1N1	11/29/2009	Cyprus	A/Cyprus/493/2009
NA	KF687931	H1N1	11/29/2009	Cyprus	A/Cyprus/495/2009
NA	KF687929	H1N1	11/29/2009	Cyprus	A/Cyprus/492/2009
NA	GU562467	H1N1	11/29/2009	China	A/Guangdong/1102/2009
NA	CY056805	H1N1	11/29/2009	USA	A/New York/6808/2009(H1N1)
NA	CY100503	H1N1	11/30/2009	Mexico	A/Mexico City/INER16/2009
NA	JF513114	H1N1	11/30/2009	Italy	A/Roma/ISS9998/2009
NA	CY066665	H1N1	11/30/2009	Germany	A/Cologne/INS286/2009(H1N1)
NA	CY055365	H1N1	11/30/2009	Italy	A/Rome/645/2009(H1N1)
NA	CY066170	H1N1	11/30/2009	Japan	A/Nagasaki/09N102/2009
NA	CY083692	H1N1	11/30/2009	Spain	A/Terrassa/INS94/2009(H1N1)
NA	CY066168	H1N1	11/30/2009	Japan	A/Nagasaki/09N083/2009
NA	KJ856076	H1N1	11/30/2009	Sri Lanka	A/Sri Lanka/2008/2009
NA	KT181557	H1N1	11/30/2009	Singapore	A/Singapore/DMS55/2009
NA	HM189347	H1N1	11/30/2009	Russia	A/Tambov/CRIE-LLA/2009
NA	HM189325	H1N1	11/30/2009	Russia	A/Perm/CRIE-ZTS/2009
NA	CY123875	H1N1	11/30/2009	Singapore	A/Singapore/ON2378/2009
NA	CY123867	H1N1	11/30/2009	Singapore	A/Singapore/ON2365/2009
NA	CY072240	H1N1	11/30/2009	Germany	A/Frankfurt/INS305/2009(H1N1)

NA	CY083789	H1N1	11/30/2009	Denmark	A/Odense/INS309/2009(H1N1)
NA	CY065778	H1N1	11/30/2009	Netherlar	A/Netherlands/2635b/2009
NA	CY069140	H1N1	11/30/2009	Spain	A/Madrid/INS300/2009(H1N1)
NA	CY069220	H1N1	11/30/2009	Germany	A/Munich/INS363/2009(H1N1)
NA	KF687933	H1N1	11/30/2009	Cyprus	A/Cyprus/514/2009
NA	CY056765	H1N1	11/30/2009	USA	A/New York/6668/2009(H1N1)
NA	CY067775	H1N1	11/30/2009	Portugal	A/Lisboa/140/2009(H1N1)
NA	CY067766	H1N1	11/30/2009	Portugal	A/Lisboa/108/2009(H1N1)
NA	CY063616	H1N1	11/30/2009	Germany	A/Cologne/INS136/2009(H1N1)
NA	CY060989	H1N1	11/30/2009	USA	A/Texas/JMS384/2009(H1N1)
NA	CY053398	H1N1	11/30/2009	Spain	A/Catalonia/S2006/2009(H1N1)
NA	MF196260	H1N1	11/30/2009	Iran	A/Tehran/88-09-26-2/2009
NA	KX651640	H1N1	11/30/2009	France	A/France/1214-3R/2009
NA	KX651641	H1N1	11/30/2009	France	A/France/1214-3T/2009
NA	KY631546	H1N1	11/30/2009	France	A/Lyon/48-307/2009
NA	CY053402	H1N1	11/30/2009	Spain	A/Catalonia/S2010/2009(H1N1)
NA	CY053410	H1N1	11/30/2009	Spain	A/Catalonia/S2019/2009(H1N1)
NA	CY056813	H1N1	11/30/2009	USA	A/New York/6809/2009(H1N1)
NA	HQ698877	H1N1	12/2009	Saudi Ara	A/Saudi Arabia/KSU-10/2009
NA	HQ698879	H1N1	12/2009	Saudi Ara	A/Saudi Arabia/KSU-12/2009
NA	HQ698881	H1N1	12/2009	Saudi Ara	A/Saudi Arabia/KSU-15/2009
NA	HQ698872	H1N1	12/2009	Saudi Ara	A/Saudi Arabia/KSU-4/2009
NA	HQ698880	H1N1	12/2009	Saudi Ara	A/Saudi Arabia/KSU-14/2009
NA	HQ698884	H1N1	12/2009	Saudi Ara	A/Saudi Arabia/KSU-102/2009
NA	CY078364	H1N1	12/2009	Romania	A/Bistrita-Nasaud/06/2009
NA	HM598332	H1N1	12/2009	Thailand	A/Thailand/CU-MV23/2009
NA	HQ698883	H1N1	12/2009	Saudi Ara	A/Saudi Arabia/KSU-101/2009
NA	HQ698875	H1N1	12/2009	Saudi Ara	A/Saudi Arabia/KSU-8/2009
NA	HQ698869	H1N1	12/2009	Saudi Ara	A/Saudi Arabia/KSU-1/2009
NA	HQ698882	H1N1	12/2009	Saudi Ara	A/Saudi Arabia/KSU-16/2009
NA	HQ698870	H1N1	12/2009	Saudi Ara	A/Saudi Arabia/KSU-2/2009
NA	HQ698874	H1N1	12/2009	Saudi Ara	A/Saudi Arabia/KSU-6/2009
NA	HQ698871	H1N1	12/2009	Saudi Ara	A/Saudi Arabia/KSU-3/2009
NA	HQ698873	H1N1	12/2009	Saudi Ara	A/Saudi Arabia/KSU-5/2009
NA	HQ698876	H1N1	12/2009	Saudi Ara	A/Saudi Arabia/KSU-9/2009
NA	HQ698878	H1N1	12/2009	Saudi Ara	A/Saudi Arabia/KSU-11/2009
NA	HM138490	H1N1	12/2009	Germany	A/Germany-MV/HGW4/2009(H1N1)
NA	HM138492	H1N1	12/2009	Germany	A/Germany-MV/HGW6/2009(H1N1)
NA	JN381398	H1N1	12/2009	Taiwan	A/Taiwan/97553/2009
NA	JN006481	H1N1	12/2009	China	A/Hangzhou/1732/2009
NA	JN006483	H1N1	12/2009	China	A/Hangzhou/1748/2009
NA	JN006484	H1N1	12/2009	China	A/Hangzhou/1756/2009
NA	JN006482	H1N1	12/2009	China	A/Hangzhou/1733/2009
NA	JN381390	H1N1	12/2009	Taiwan	A/Taiwan/97184/2009
NA	JN381395	H1N1	12/2009	Taiwan	A/Taiwan/97382/2009
NA	JN381391	H1N1	12/2009	Taiwan	A/Taiwan/97199/2009
NA	JN381394	H1N1	12/2009	Taiwan	A/Taiwan/97351/2009
NA	JN381393	H1N1	12/2009	Taiwan	A/Taiwan/97341/2009
NA	JN381397	H1N1	12/2009	Taiwan	A/Taiwan/97487/2009
NA	JN381396	H1N1	12/2009	Taiwan	A/Taiwan/97394/2009
NA	JN381392	H1N1	12/2009	Taiwan	A/Taiwan/97241/2009
NA	CY060476	H1N1	12/01/2009	South Kor	A/Daejeon/1840/2009(H1N1)
NA	KJ856084	H1N1	12/01/2009	Sri Lanka	A/Sri Lanka/2009/2009
NA	KX651754	H1N1	12/01/2009	USA	A/USA/2504-1T/2009

NA	CY063689	H1N1	12/01/2009	China	A/Shandong-Shizhong/SWL1518/2009(H1N
NA	CY063772	H1N1	12/01/2009	Singapore	A/Singapore/GP4556/2009(H1N1)
NA	KC780490	H1N1	12/01/2009	USA	A/Illinois/25/2009
NA	KJ856239	H1N1	12/01/2009	Sri Lanka	A/Sri Lanka/24588/2009
NA	CY057984	H1N1	12/01/2009	USA	A/Wisconsin/629-D01412/2009(H1N1)
NA	HQ247728	H1N1	12/01/2009	Finland	A/Finland/702/2009
NA	LC093043	H1N1	12/01/2009	Saudi Ara	A/Riyadh/4439/2009
NA	CY063690	H1N1	12/01/2009	China	A/Shandong-Shizhong/SWL1604/2009(H1N
NA	CY087857	H1N1	12/01/2009	Thailand	A/Thailand/CU-H1039/2009
NA	CY053406	H1N1	12/01/2009	Spain	A/Catalonia/S2016/2009(H1N1)
NA	HM189343	H1N1	12/01/2009	Russia	A/Tambov/CRIE-FOS/2009
NA	AB649898	H1N1	12/01/2009	Japan	A/Toyama/402/2009
NA	CY073152	H1N1	12/01/2009	Mexico	A/Mexico City/WRAIR1786T/2009(H1N1)
NA	CY073499	H1N1	12/01/2009	Germany	A/Frankfurt/INS304/2009(H1N1)
NA	CY071820	H1N1	12/01/2009	Ethiopia	A/Addis Ababa/WR2848N/2009(H1N1)
NA	CY073136	H1N1	12/01/2009	Mexico	A/Mexico City/WRAIR1779T/2009(H1N1)
NA	CY071828	H1N1	12/01/2009	Ethiopia	A/Addis Ababa/WR2848T/2009(H1N1)
NA	KC781112	H1N1	12/01/2009	USA	A/New Hampshire/27/2009
NA	KC780187	H1N1	12/01/2009	USA	A/Montana/22/2009
NA	CY055170	H1N1	12/01/2009	Mongolia	A/Dundgobi/9746/2009(H1N1)
NA	CY065842	H1N1	12/01/2009	Netherlan	A/Netherlands/2636/2009
NA	CY056773	H1N1	12/01/2009	USA	A/New York/6669/2009(H1N1)
NA	CY066116	H1N1	12/01/2009	Japan	A/Kyoto/09K174/2009
NA	HQ247731	H1N1	12/01/2009	Finland	A/Finland/705/2009
NA	CY066126	H1N1	12/01/2009	Japan	A/Hyogo/09K305/2009
NA	CY060482	H1N1	12/01/2009	South Kor	A/Daegu/1853/2009(H1N1)
NA	CY060470	H1N1	12/02/2009	South Kor	A/Seoul/2883/2009(H1N1)
NA	HM568122	H1N1	12/02/2009	United Ki	A/England/941/2009
NA	KX651755	H1N1	12/02/2009	USA	A/USA/2050-1T/2009
NA	KX651642	H1N1	12/02/2009	France	A/France/1214-5T/2009
NA	KX651645	H1N1	12/02/2009	France	A/France/730-1T/2009
NA	CY063670	H1N1	12/02/2009	China	A/Shandong-Zoucheng/SWL44/2009(H1N1)
NA	CY056837	H1N1	12/02/2009	USA	A/New York/6864/2009(H1N1)
NA	CY063608	H1N1	12/02/2009	Denmark	A/Aalborg/INS133/2009(H1N1)
NA	CY063774	H1N1	12/02/2009	Singapore	A/Singapore/GP4581/2009(H1N1)
NA	KF687934	H1N1	12/02/2009	Cyprus	A/Cyprus/545/2009
NA	HQ239874	H1N1	12/02/2009	Canada	A/Canada-ON/RV2017-10/2009(H1N1)
NA	CY066042	H1N1	12/02/2009	Japan	A/Fukushima/09FY031/2009
NA	CY053707	H1N1	12/02/2009	Spain	A/Catalonia/S2055/2009(H1N1)
NA	CY053711	H1N1	12/02/2009	Spain	A/Catalonia/S2056/2009(H1N1)
NA	CY071081	H1N1	12/02/2009	Germany	A/Bochum/INS375/2009(H1N1)
NA	CY072272	H1N1	12/02/2009	Poland	A/Warsaw/INS311/2009(H1N1)
NA	CY071089	H1N1	12/02/2009	Germany	A/Bochum/INS376/2009(H1N1)
NA	CY073106	H1N1	12/02/2009	Taiwan	A/Taipei/WRAIR1470T/2009(H1N1)
NA	CY071436	H1N1	12/02/2009	Kyrgyzsta	A/Bishkek/WR0881T/2009(H1N1)
NA	CY072264	H1N1	12/02/2009	Poland	A/Warsaw/INS310/2009(H1N1)
NA	CY071193	H1N1	12/02/2009	Germany	A/Frankfurt/INS399/2009(H1N1)
NA	CY075638	H1N1	12/02/2009	USA	A/Boston/703/2009(H1N1)
NA	CY062765	H1N1	12/02/2009	Spain	A/Madrid/INS134/2009(H1N1)
NA	CY067793	H1N1	12/02/2009	Portugal	A/Lisboa/171/2009(H1N1)
NA	CY056981	H1N1	12/02/2009	Denmark	A/Copenhagen/INS98/2009(H1N1)
NA	KC780684	H1N1	12/02/2009	USA	A/California/42/2009
NA	AB704520	H1N1	12/02/2009	Japan	A/Yamaguchi/247/2009
NA	CY128429	H1N1	12/02/2009	Viet Nam	A/Viet Nam/12032005/2009

NA	CY065898	H1N1	12/02/2009	Netherlar	A/Netherlands/2822/2009
NA	CY057992	H1N1	12/02/2009	USA	A/Wisconsin/629-D00935/2009(H1N1)
NA	CY058000	H1N1	12/02/2009	USA	A/Wisconsin/629-D02299/2009(H1N1)
NA	KC780459	H1N1	12/02/2009	USA	A/North Dakota/16/2009
NA	KP019729	H1N1	12/02/2009	China	A/FuZhou/SWL12139/2009
NA	KX651751	H1N1	12/02/2009	USA	A/USA/1615-5T/2009
NA	CY062829	H1N1	12/03/2009	Poland	A/Warsaw/INS146/2009(H1N1)
NA	KT181558	H1N1	12/03/2009	Singapore	A/Singapore/DMS56/2009
NA	CY053715	H1N1	12/03/2009	Spain	A/Catalonia/S2060/2009(H1N1)
NA	KR870135	H1N1	12/03/2009	India	A/Bangalore/10695-10/2009
NA	KX651630	H1N1	12/03/2009	France	A/France/3489-1T/2009
NA	KR870136	H1N1	12/03/2009	India	A/Bangalore/10698-02/2009
NA	CY072280	H1N1	12/03/2009	Poland	A/Warsaw/INS312/2009(H1N1)
NA	CY072288	H1N1	12/03/2009	Poland	A/Warsaw/INS313/2009(H1N1)
NA	CY083827	H1N1	12/03/2009	Poland	A/Wroclaw/INS435/2009
NA	CY083920	H1N1	12/03/2009	Poland	A/Warsaw/INS147/2009(H1N1)
NA	CY073767	H1N1	12/03/2009	Spain	A/Barcelona/INS378/2009(H1N1)
NA	CY075646	H1N1	12/03/2009	USA	A/Boston/704/2009(H1N1)
NA	KC782230	H1N1	12/03/2009	USA	A/Illinois/33/2009
NA	KC781979	H1N1	12/03/2009	USA	A/Nevada/20/2009
NA	KC780791	H1N1	12/03/2009	USA	A/Utah/55/2009
NA	CY055375	H1N1	12/03/2009	Italy	A/Rome/651/2009(H1N1)
NA	CY066026	H1N1	12/03/2009	Japan	A/Niigata/09F086/2009
NA	CY123881	H1N1	12/03/2009	Singapore	A/Singapore/ON2383/2009
NA	CY053370	H1N1	12/03/2009	Spain	A/Catalonia/NS7745/2009(H1N1)
NA	KX651752	H1N1	12/04/2009	USA	A/USA/1615-7T/2009
NA	JN187193	H1N1	12/04/2009	Taiwan	A/Taiwan/6341/2009
NA	KX651620	H1N1	12/04/2009	France	A/France/3667-1T/2009
NA	CY066313	H1N1	12/04/2009	USA	A/California/VRDL117/2009(H1N1)
NA	MF196266	H1N1	12/04/2009	Iran	A/Tehran/88-9-16/2009
NA	HQ239828	H1N1	12/04/2009	Canada	A/Canada-NB/RV0005-10/2009
NA	CY060997	H1N1	12/04/2009	USA	A/Texas/JMS385/2009(H1N1)
NA	MF535118	H1N1	12/04/2009	India	A/Jodhpur/526/2009
NA	CY087863	H1N1	12/04/2009	Thailand	A/Thailand/CU-H1065/2009
NA	KP317308	H1N1	12/04/2009	India	A/Jammu & Kashmir/013/2009
NA	CY066130	H1N1	12/04/2009	Japan	A/Kyoto/09K328-2/2009
NA	CY083561	H1N1	12/04/2009	Hungary	A/Budapest/WRAIR2411N/2009
NA	CY088603	H1N1	12/04/2009	USA	A/Boston/705/2009
NA	CY066289	H1N1	12/04/2009	USA	A/California/VRDL114/2009(H1N1)
NA	CY065786	H1N1	12/04/2009	Netherlar	A/Netherlands/2629/2009
NA	CY066305	H1N1	12/04/2009	USA	A/California/VRDL116/2009(H1N1)
NA	CY069228	H1N1	12/04/2009	Germany	A/Munich/INS364/2009(H1N1)
NA	CY062773	H1N1	12/04/2009	Spain	A/Madrid/INS135/2009(H1N1)
NA	HM157259	H1N1	12/04/2009	Russia	A/Yaroslavl/IIV-196/2009(H1N1)
NA	CY066321	H1N1	12/04/2009	USA	A/California/VRDL118/2009(H1N1)
NA	CY066297	H1N1	12/04/2009	USA	A/California/VRDL115/2009(H1N1)
NA	KC780613	H1N1	12/04/2009	USA	A/Alabama/08/2009
NA	KC780780	H1N1	12/04/2009	USA	A/Alaska/47/2009
NA	GU727826	H1N1	12/04/2009	Russia	A/IIV-Anadyr/177/2009(H1N1)
NA	CY107535	H1N1	12/04/2009	United Ki	A/Scotland/Edinburgh_23151/2009
NA	CY056797	H1N1	12/04/2009	USA	A/New York/6776/2009(H1N1)
NA	CY057352	H1N1	12/04/2009	Germany	A/Hamburg/INS92/2009(H1N1)
NA	KC780304	H1N1	12/04/2009	USA	A/Pennsylvania/31/2009
NA	KX651646	H1N1	12/04/2009	France	A/France/730-3R/2009

NA	CY060479	H1N1	12/05/2009	South Ko	A/Daejeon/1841/2009(H1N1)
NA	KX651643	H1N1	12/05/2009	France	A/France/1214-P2T/2009
NA	HM104478	H1N1	12/05/2009	Russia	A/Yaroslavl/IIV-198/2009(H1N1)
NA	HQ239829	H1N1	12/05/2009	Canada	A/Canada-MB/RV0062-10/2009
NA	AB704528	H1N1	12/05/2009	Japan	A/Yamaguchi/248/2009
NA	CY189542	H1N1	12/05/2009	Japan	A/Yokohama/UT-K1205T/2009
NA	HM072413	H1N1	12/05/2009	Taiwan	A/Taiwan/2342/2009
NA	JQ714188	H1N1	12/05/2009	China	A/Tianjinninghe/SWL49/2009
NA	CY055379	H1N1	12/05/2009	Italy	A/Rome/652/2009(H1N1)
NA	CY056789	H1N1	12/05/2009	USA	A/New York/6775/2009(H1N1)
NA	CY061005	H1N1	12/06/2009	USA	A/Texas/JMS386/2009(H1N1)
NA	KX651631	H1N1	12/06/2009	France	A/France/3489-3T/2009
NA	CY055381	H1N1	12/06/2009	Italy	A/Rome/654/2009(H1N1)
NA	KP317299	H1N1	12/06/2009	India	A/West Bengal/004/2009
NA	CY084448	H1N1	12/06/2009	USA	A/New York/6902/2009
NA	CY061940	H1N1	12/06/2009	USA	A/New York/6941/2009(H1N1)
NA	HQ247733	H1N1	12/06/2009	Finland	A/Finland/708/2009
NA	KC780598	H1N1	12/06/2009	USA	A/Arizona/20/2009
NA	KC780190	H1N1	12/06/2009	USA	A/Maine/15/2009
NA	KC781016	H1N1	12/06/2009	USA	A/Arizona/20/2009
NA	CY123885	H1N1	12/06/2009	Singapore	A/Singapore/ON2394/2009
NA	CY065077	H1N1	12/06/2009	USA	A/New York/7421/2009(H1N1)
NA	KC780371	H1N1	12/06/2009	USA	A/Florida/30/2009
NA	CY060485	H1N1	12/07/2009	South Ko	A/Daegu/1854/2009(H1N1)
NA	KT181560	H1N1	12/07/2009	Singapore	A/Singapore/DMS57/2009
NA	CY058008	H1N1	12/07/2009	USA	A/Wisconsin/629-D01473/2009(H1N1)
NA	CY061213	H1N1	12/07/2009	USA	A/New York/6771/2009(H1N1)
NA	CY063624	H1N1	12/07/2009	USA	A/New York/INS150/2009(H1N1)
NA	CY062821	H1N1	12/07/2009	Denmark	A/Copenhagen/INS144/2009(H1N1)
NA	KF687935	H1N1	12/07/2009	Cyprus	A/Cyprus/562/2009
NA	CY061596	H1N1	12/07/2009	USA	A/New York/6806/2009(H1N1)
NA	CY055384	H1N1	12/07/2009	Italy	A/Rome/656/2009(H1N1)
NA	CY061221	H1N1	12/07/2009	USA	A/New York/6772/2009(H1N1)
NA	CY053723	H1N1	12/07/2009	Spain	A/Catalonia/S2068/2009(H1N1)
NA	CY053719	H1N1	12/07/2009	Spain	A/Catalonia/S2065/2009(H1N1)
NA	CY072304	H1N1	12/07/2009	Poland	A/Warsaw/INS315/2009(H1N1)
NA	CY072296	H1N1	12/07/2009	Poland	A/Warsaw/INS314/2009(H1N1)
NA	CY073727	H1N1	12/07/2009	Denmark	A/Aalborg/INS283/2009(H1N1)
NA	HQ256663	H1N1	12/07/2009	Myanmar	A/Myanmar/M543/2009(H1N1)
NA	CY061948	H1N1	12/07/2009	USA	A/New York/6943/2009(H1N1)
NA	CY058398	H1N1	12/07/2009	USA	A/Wisconsin/629-D01591/2009(H1N1)
NA	CY056989	H1N1	12/07/2009	Poland	A/Warsaw/INS100/2009(H1N1)
NA	KC781092	H1N1	12/07/2009	USA	A/Iowa/14/2009
NA	KC780337	H1N1	12/07/2009	USA	A/Wisconsin/59/2009
NA	KC782010	H1N1	12/07/2009	USA	A/New Jersey/13/2009
NA	CY065890	H1N1	12/07/2009	Netherlar	A/Netherlands/2815/2009
NA	CY056965	H1N1	12/07/2009	Denmark	A/Copenhagen/INS95/2009(H1N1)
NA	KC780403	H1N1	12/07/2009	USA	A/North Carolina/56/2009
NA	KC780593	H1N1	12/07/2009	USA	A/Texas/75/2009
NA	CY066044	H1N1	12/07/2009	Japan	A/Fukushima/09FY038/2009
NA	CY066122	H1N1	12/07/2009	Japan	A/Kyoto/09K266-2/2009
NA	CY080248	H1N1	12/07/2009	China	A/Nanchang/8014/2009
NA	CY063669	H1N1	12/07/2009	China	A/Shandong-Shizhong/SWL25/2009(H1N1
NA	KX651644	H1N1	12/08/2009	France	A/France/1214-P5T/2009

NA	CY080252	H1N1	12/08/2009	China	A/Nanchang/8021/2009
NA	CY061013	H1N1	12/08/2009	USA	A/Texas/JMS387/2009(H1N1)
NA	KP317309	H1N1	12/08/2009	India	A/Uttrakhand/014/2009
NA	KX651632	H1N1	12/08/2009	France	A/France/3489-5T/2009
NA	CY066921	H1N1	12/08/2009	USA	A/Pensacola/INS234/2009
NA	CY066929	H1N1	12/08/2009	USA	A/Pensacola/INS235/2009
NA	CY072312	H1N1	12/08/2009	Poland	A/Warsaw/INS316/2009(H1N1)
NA	CY065101	H1N1	12/08/2009	USA	A/New York/7426/2009(H1N1)
NA	CY067169	H1N1	12/08/2009	Germany	A/Bonn/INS280/2009(H1N1)
NA	KJ958970	H1N1	12/08/2009	India	A/Mumbai/4467/2009
NA	CY061916	H1N1	12/08/2009	USA	A/New York/6907/2009(H1N1)
NA	CY058016	H1N1	12/08/2009	USA	A/Wisconsin/629-D00672/2009(H1N1)
NA	CY058382	H1N1	12/08/2009	USA	A/Wisconsin/629-D00780/2009(H1N1)
NA	KC780839	H1N1	12/08/2009	USA	A/Oregon/42/2009
NA	KC780211	H1N1	12/08/2009	USA	A/Indiana/20/2009
NA	CY066118	H1N1	12/08/2009	Japan	A/Kyoto/09K206/2009
NA	LN846552	H1N1	12/08/2009	United Ki	A/England/9/2009
NA	LN845784	H1N1	12/08/2009	United Ki	A/England/8/2009
NA	CY058024	H1N1	12/08/2009	USA	A/Wisconsin/629-D00401/2009(H1N1)
NA	CY060488	H1N1	12/09/2009	South Kor	A/Daegu/1855/2009(H1N1)
NA	CY128381	H1N1	12/09/2009	Viet Nam	A/Viet Nam/11032006/2009
NA	KX651756	H1N1	12/09/2009	USA	A/USA/3423-1T/2009
NA	KX651647	H1N1	12/09/2009	France	A/France/1641-3T/2009
NA	HQ286465	H1N1	12/09/2009	Japan	A/Kurume/31/2009
NA	CY069212	H1N1	12/09/2009	Germany	A/Berlin/INS361/2009(H1N1)
NA	HQ286415	H1N1	12/09/2009	Japan	A/Kurume/26/2009
NA	HQ286412	H1N1	12/09/2009	Japan	A/Kurume/23/2009
NA	HQ286463	H1N1	12/09/2009	Japan	A/Kurume/29/2009
NA	HQ286414	H1N1	12/09/2009	Japan	A/Kurume/25/2009
NA	HQ286411	H1N1	12/09/2009	Japan	A/Kurume/22/2009
NA	HQ286413	H1N1	12/09/2009	Japan	A/Kurume/24/2009
NA	CY063592	H1N1	12/09/2009	Greece	A/Athens/INS122/2009(H1N1)
NA	CY055390	H1N1	12/09/2009	Italy	A/Rome/659/2009(H1N1)
NA	CY078389	H1N1	12/09/2009	Romania	A/Botosani/557/2009
NA	LC093006	H1N1	12/09/2009	Saudi Ara	A/Riyadh/4570/2009
NA	HQ286416	H1N1	12/09/2009	Japan	A/Kurume/27/2009
NA	CY053703	H1N1	12/09/2009	Spain	A/Catalonia/NS7922/2009(H1N1)
NA	HQ286462	H1N1	12/09/2009	Japan	A/Kurume/28/2009
NA	HQ286464	H1N1	12/09/2009	Japan	A/Kurume/30/2009
NA	CY072552	H1N1	12/09/2009	Poland	A/Wroclaw/INS437/2009(H1N1)
NA	CY072544	H1N1	12/09/2009	Poland	A/Wroclaw/INS436/2009(H1N1)
NA	CY067177	H1N1	12/09/2009	Germany	A/Bonn/INS284/2009(H1N1)
NA	CY069236	H1N1	12/09/2009	Germany	A/Munich/INS365/2009(H1N1)
NA	KF687936	H1N1	12/09/2009	Cyprus	A/Cyprus/592/2009
NA	CY058390	H1N1	12/09/2009	USA	A/Wisconsin/629-D00147/2009(H1N1)
NA	KC780197	H1N1	12/09/2009	USA	A/Colorado/17/2009
NA	CY058366	H1N1	12/09/2009	USA	A/Wisconsin/629-D00689/2009(H1N1)
NA	CY066066	H1N1	12/09/2009	Japan	A/Gunma/09G025/2009
NA	CY089610	H1N1	12/09/2009	China	A/Nanchang/8002/2009
NA	KX651753	H1N1	12/09/2009	USA	A/USA/1615-P2T/2009
NA	KC780406	H1N1	12/10/2009	USA	A/Indiana/21/2009
NA	CY066673	H1N1	12/10/2009	Belgium	A/Tessenderlo/INS287/2009(H1N1)
NA	CY056973	H1N1	12/10/2009	Denmark	A/Copenhagen/INS96/2009(H1N1)
NA	CY066174	H1N1	12/10/2009	Japan	A/Nagasaki/09N110/2009

NA	CY066028	H1N1	12/10/2009	Japan	A/Niigata/09F098/2009
NA	KP019730	H1N1	12/10/2009	China	A/FuZhou/SWL12273/2009
NA	KM213302	H1N1	12/10/2009	Oman	A/Oman/SQUH-20/2009
NA	KT181561	H1N1	12/10/2009	Singapore	A/Singapore/DMS58/2009
NA	CY073232	H1N1	12/10/2009	Serbia	A/Belgrade/WRAIR2384T/2009(H1N1)
NA	CY073224	H1N1	12/10/2009	Serbia	A/Belgrade/WRAIR2384N/2009(H1N1)
NA	CY083770	H1N1	12/10/2009	Greece	A/Athens/INS256/2009(H1N1)
NA	CY063776	H1N1	12/10/2009	Singapore	A/Singapore/GP4697/2009(H1N1)
NA	CY065997	H1N1	12/10/2009	Mongolia	A/Ulgii/9911/2009(H1N1)
NA	CY062781	H1N1	12/10/2009	Belgium	A/Tessenderlo/INS138/2009(H1N1)
NA	KC780221	H1N1	12/10/2009	USA	A/Florida/31/2009
NA	CY063045	H1N1	12/10/2009	Germany	A/Bochum/INS187/2009(H1N1)
NA	CY061908	H1N1	12/10/2009	USA	A/New York/6903/2009(H1N1)
NA	CY058032	H1N1	12/10/2009	USA	A/Wisconsin/629-D00636/2009(H1N1)
NA	HQ286476	H1N1	12/10/2009	Japan	A/Kurume/42/2009
NA	HQ286475	H1N1	12/10/2009	Japan	A/Kurume/41/2009
NA	HQ286469	H1N1	12/10/2009	Japan	A/Kurume/35/2009
NA	HQ286473	H1N1	12/10/2009	Japan	A/Kurume/39/2009
NA	HQ286471	H1N1	12/10/2009	Japan	A/Kurume/37/2009
NA	HQ286474	H1N1	12/10/2009	Japan	A/Kurume/40/2009
NA	HQ286472	H1N1	12/10/2009	Japan	A/Kurume/38/2009
NA	HQ286467	H1N1	12/10/2009	Japan	A/Kurume/33/2009
NA	HQ286470	H1N1	12/10/2009	Japan	A/Kurume/36/2009
NA	MF196269	H1N1	12/10/2009	Iran	A/Tehran/88-9-52/2009
NA	LC093009	H1N1	12/10/2009	Saudi Ara	A/Riyadh/4573/2009
NA	HQ286466	H1N1	12/10/2009	Japan	A/Kurume/32/2009
NA	CY053700	H1N1	12/10/2009	Spain	A/Catalonia/NS092145714/2009(H1N1)
NA	HQ286468	H1N1	12/10/2009	Japan	A/Kurume/34/2009
NA	CY053696	H1N1	12/10/2009	Spain	A/Catalonia/NS092145684/2009(H1N1)
NA	CY055392	H1N1	12/10/2009	Italy	A/Rome/660/2009(H1N1)
NA	CY063666	H1N1	12/10/2009	China	A/Shandong-Shizhong/SWL213/2009(H1N
NA	CY071097	H1N1	12/11/2009	Spain	A/Barcelona/INS379/2009(H1N1)
NA	CY061924	H1N1	12/11/2009	USA	A/New York/6937/2009(H1N1)
NA	CY099331	H1N1	12/11/2009	USA	A/Kentucky/136/2009
NA	KC780628	H1N1	12/11/2009	USA	A/Vermont/29/2009
NA	CY064774	H1N1	12/11/2009	Germany	A/Berlin/INS170/2009(H1N1)
NA	CY061932	H1N1	12/11/2009	USA	A/New York/6939/2009(H1N1)
NA	CY061956	H1N1	12/11/2009	USA	A/New York/6945/2009(H1N1)
NA	CY063820	H1N1	12/11/2009	Singapore	A/Singapore/ON2400/2009
NA	CY083928	H1N1	12/11/2009	Poland	A/Warsaw/INS148/2009(H1N1)
NA	CY083936	H1N1	12/11/2009	Poland	A/Warsaw/INS149/2009(H1N1)
NA	CY083747	H1N1	12/11/2009	Spain	A/Barcelona/INS189/2009(H1N1)
NA	GU562469	H1N1	12/11/2009	China	A/Guangdong/1202/2009
NA	KX651648	H1N1	12/11/2009	France	A/France/1641-3R/2009
NA	HM998905	H1N1	12/11/2009	Russia	A/Chelyabinsk/08/2009(H1N1)
NA	CY061021	H1N1	12/11/2009	USA	A/Texas/JMS388/2009(H1N1)
NA	CY063632	H1N1	12/11/2009	USA	A/New York/INS151/2009(H1N1)
NA	CY055395	H1N1	12/11/2009	Italy	A/Rome/661/2009(H1N1)
NA	CY055393	H1N1	12/11/2009	Italy	A/Rome/671/2009(H1N1)
NA	CY061980	H1N1	12/11/2009	USA	A/New York/6977/2009(H1N1)
NA	CY058374	H1N1	12/12/2009	USA	A/Wisconsin/629-D01244/2009(H1N1)
NA	KX651757	H1N1	12/12/2009	USA	A/USA/3423-3T/2009
NA	CY080250	H1N1	12/12/2009	China	A/Nanchang/8015/2009
NA	CY123304	H1N1	12/12/2009	Singapore	A/Singapore/GP4728/2009

NA	CY063691	H1N1	12/12/2009	China	A/Shandong-Shizhong/SWL1887/2009(H1N
NA	KM213303	H1N1	12/12/2009	Oman	A/Oman/SQUH-22/2009
NA	CY066128	H1N1	12/12/2009	Japan	A/Hyogo/09K314/2009
NA	CY083799	H1N1	12/12/2009	Germany	A/Berlin/INS362/2009(H1N1)
NA	CY067796	H1N1	12/12/2009	Portugal	A/Lisboa/172/2009(H1N1)
NA	KC780700	H1N1	12/12/2009	USA	A/Vermont/28/2009
NA	KC780392	H1N1	12/13/2009	USA	A/Maryland/36/2009
NA	KP317312	H1N1	12/13/2009	India	A/Delhi/017/2009
NA	CY067113	H1N1	12/13/2009	Greece	A/Athens/INS264/2009(H1N1)
NA	LC093039	H1N1	12/13/2009	Saudi Ara	A/Riyadh/4578/2009
NA	KM213304	H1N1	12/13/2009	Oman	A/Oman/SQUH-31/2009
NA	CY062717	H1N1	12/13/2009	Greece	A/Athens/INS124/2009(H1N1)
NA	CY080254	H1N1	12/13/2009	China	A/Shantou/8027/2009
NA	CY063822	H1N1	12/13/2009	Singapore	A/Singapore/ON2407/2009
NA	CY067121	H1N1	12/13/2009	Greece	A/Athens/INS266/2009(H1N1)
NA	CY067097	H1N1	12/13/2009	Greece	A/Athens/INS262/2009(H1N1)
NA	CY062709	H1N1	12/13/2009	Greece	A/Athens/INS123/2009(H1N1)
NA	CY079539	H1N1	12/13/2009	Switzerla	A/Switzerland/9356/2009
NA	CY067065	H1N1	12/13/2009	Greece	A/Athens/INS257/2009(H1N1)
NA	CY056957	H1N1	12/13/2009	Greece	A/Athens/INS85/2009(H1N1)
NA	CY067089	H1N1	12/13/2009	Greece	A/Athens/INS261/2009(H1N1)
NA	CY067081	H1N1	12/13/2009	Greece	A/Athens/INS260/2009(H1N1)
NA	CY062725	H1N1	12/13/2009	Greece	A/Athens/INS125/2009(H1N1)
NA	CY067073	H1N1	12/13/2009	Greece	A/Athens/INS259/2009(H1N1)
NA	CY083904	H1N1	12/13/2009	Greece	A/Athens/INS86/2009(H1N1)
NA	CY072344	H1N1	12/13/2009	Greece	A/Athens/INS320/2009(H1N1)
NA	CY067105	H1N1	12/13/2009	Greece	A/Athens/INS263/2009(H1N1)
NA	CY072352	H1N1	12/13/2009	Greece	A/Athens/INS322/2009(H1N1)
NA	CY061996	H1N1	12/14/2009	USA	A/New York/7020/2009(H1N1)
NA	CY061964	H1N1	12/14/2009	USA	A/New York/6949/2009(H1N1)
NA	CY054559	H1N1	12/14/2009	Spain	A/Catalonia/S2099/2009(H1N1)
NA	CY054570	H1N1	12/14/2009	Spain	A/Catalonia/S2117/2009(H1N1)
NA	KC683497	H1N1	12/14/2009	China	A/Liaoning/14/2009
NA	KP317311	H1N1	12/14/2009	India	A/Delhi/016/2009
NA	CY054556	H1N1	12/14/2009	Spain	A/Catalonia/S2098/2009(H1N1)
NA	CY054562	H1N1	12/14/2009	Spain	A/Catalonia/S2101/2009(H1N1)
NA	CY063678	H1N1	12/14/2009	China	A/Shandong-Qufu/SWL331/2009(H1N1)
NA	CY063681	H1N1	12/14/2009	China	A/Shandong-Wenshan/SWL47/2009(H1N1)
NA	KX651650	H1N1	12/14/2009	France	A/France/2707-1T/2009
NA	CY063675	H1N1	12/14/2009	China	A/Shandong-Shizhong/SWL1828/2009(H1N
NA	CY063673	H1N1	12/14/2009	China	A/Shandong-Shizhong/SWL1813/2009(H1N
NA	CY054573	H1N1	12/14/2009	Spain	A/Catalonia/S2118/2009(H1N1)
NA	KM985428	H1N1	12/14/2009	India	A/Malappuram/MCVR2376/2009
NA	CY073216	H1N1	12/14/2009	Serbia	A/Belgrade/WRAIR2375T/2009(H1N1)
NA	CY061972	H1N1	12/14/2009	USA	A/New York/6976/2009(H1N1)
NA	CY058422	H1N1	12/14/2009	USA	A/Wisconsin/629-D01904/2009(H1N1)
NA	CY058454	H1N1	12/14/2009	USA	A/Wisconsin/629-D01846/2009(H1N1)
NA	GU592907	H1N1	12/14/2009	Russia	A/Magadan/02/2009(H1N1)
NA	CY107420	H1N1	12/14/2009	United Ki	A/Scotland/Dundee_09V502215/2009
NA	KC780941	H1N1	12/14/2009	USA	A/Washington/73/2009
NA	KT181562	H1N1	12/14/2009	Singapore	A/Singapore/DMS59/2009
NA	CY073208	H1N1	12/14/2009	Serbia	A/Belgrade/WRAIR2375N/2009(H1N1)
NA	CY054567	H1N1	12/15/2009	Spain	A/Catalonia/S2114/2009(H1N1)
NA	CY067153	H1N1	12/15/2009	Greece	A/Athens/INS270/2009(H1N1)

NA	CY090099	H1N1	12/15/2009	USA	A/Boston/706/2009
NA	CY072328	H1N1	12/15/2009	USA	A/Newark/INS318/2009(H1N1)
NA	CY066068	H1N1	12/15/2009	Japan	A/Gunma/09G029/2009
NA	CY066353	H1N1	12/15/2009	USA	A/California/VRDL122/2009(H1N1)
NA	CY066329	H1N1	12/15/2009	USA	A/California/VRDL119/2009(H1N1)
NA	CY066337	H1N1	12/15/2009	USA	A/California/VRDL120/2009(H1N1)
NA	CY066345	H1N1	12/15/2009	USA	A/California/VRDL121/2009(H1N1)
NA	CY073491	H1N1	12/15/2009	Greece	A/Athens/INS271/2009(H1N1)
NA	CY073184	H1N1	12/15/2009	Serbia	A/Belgrade/WRAIR2366N/2009(H1N1)
NA	CY073879	H1N1	12/15/2009	Greece	A/Athens/INS323/2009(H1N1)
NA	CY062733	H1N1	12/15/2009	Greece	A/Athens/INS126/2009(H1N1)
NA	CY062661	H1N1	12/15/2009	Greece	A/Athens/INS87/2009(H1N1)
NA	CY063021	H1N1	12/15/2009	Spain	A/Madrid/INS184/2009(H1N1)
NA	CY058478	H1N1	12/15/2009	USA	A/Wisconsin/629-D02424/2009(H1N1)
NA	CY058470	H1N1	12/15/2009	USA	A/Wisconsin/629-D00337/2009(H1N1)
NA	CY066361	H1N1	12/15/2009	USA	A/California/VRDL123/2009(H1N1)
NA	CY067137	H1N1	12/15/2009	Greece	A/Athens/INS268/2009(H1N1)
NA	CY063053	H1N1	12/15/2009	Belgium	A/Tessenderlo/INS191/2009(H1N1)
NA	CY063824	H1N1	12/15/2009	Singapore	A/Singapore/ON2416/2009
NA	CY067145	H1N1	12/15/2009	Greece	A/Athens/INS269/2009(H1N1)
NA	CY066609	H1N1	12/15/2009	Greece	A/Athens/INS273/2009(H1N1)
NA	CY066601	H1N1	12/15/2009	Greece	A/Athens/INS272/2009(H1N1)
NA	CY066617	H1N1	12/15/2009	Greece	A/Athens/INS274/2009(H1N1)
NA	CY066625	H1N1	12/15/2009	Greece	A/Athens/INS275/2009(H1N1)
NA	CY058430	H1N1	12/15/2009	USA	A/Wisconsin/629-D01752/2009(H1N1)
NA	AB704456	H1N1	12/15/2009	Japan	A/Gunma/293/2009
NA	CY123306	H1N1	12/15/2009	Singapore	A/Singapore/GP4757/2009
NA	CY055403	H1N1	12/15/2009	Italy	A/Rome/665/2009(H1N1)
NA	CY066120	H1N1	12/15/2009	Japan	A/Kyoto/09K254/2009
NA	KM213295	H1N1	12/15/2009	Oman	A/Oman/SQUH-1/2009
NA	KR870137	H1N1	12/15/2009	India	A/Bangalore/11210-01/2009
NA	AB649899	H1N1	12/15/2009	Japan	A/Toyama/437/2009
NA	CY060494	H1N1	12/15/2009	South Ko	A/Daejeon/1871/2009(H1N1)
NA	CY061988	H1N1	12/16/2009	USA	A/New York/7019/2009(H1N1)
NA	KT181563	H1N1	12/16/2009	Singapore	A/Singapore/DMS60/2009
NA	CY054576	H1N1	12/16/2009	Spain	A/Catalonia/S2120/2009(H1N1)
NA	HQ239863	H1N1	12/16/2009	Canada	A/Canada-MB/RV2006-10/2009
NA	HQ239866	H1N1	12/16/2009	Canada	A/Canada-MB/RV2010-10/2009
NA	HQ239860	H1N1	12/16/2009	Canada	A/Canada-BC/RV2004-10/2009
NA	HQ239862	H1N1	12/16/2009	Canada	A/Canada-BC/RV2005-10/2009
NA	HQ239868	H1N1	12/16/2009	Canada	A/Canada-MB/RV2012-10/2009
NA	HQ239867	H1N1	12/16/2009	Canada	A/Canada-MB/RV2011-10/2009
NA	HQ239864	H1N1	12/16/2009	Canada	A/Canada-MB/RV2007-10/2009
NA	KP317310	H1N1	12/16/2009	India	A/Haryana/015/2009
NA	CY060497	H1N1	12/16/2009	South Ko	A/Daegu/1873/2009(H1N1)
NA	KX651621	H1N1	12/16/2009	France	A/France/556-1T/2009
NA	CY063692	H1N1	12/16/2009	China	A/Shandong-Shizhong/SWL1930/2009(H1N
NA	CY080258	H1N1	12/16/2009	China	A/Nanchang/8029/2009
NA	CY071257	H1N1	12/16/2009	Spain	A/Madrid/INS408/2009(H1N1)
NA	CY071105	H1N1	12/16/2009	Belgium	A/Tessenderlo/INS380/2009(H1N1)
NA	CY067129	H1N1	12/16/2009	Greece	A/Athens/INS267/2009(H1N1)
NA	CY062941	H1N1	12/16/2009	Spain	A/Terrassa/INS172/2009(H1N1)
NA	CY058462	H1N1	12/16/2009	USA	A/Wisconsin/629-D00996/2009(H1N1)
NA	CY061029	H1N1	12/16/2009	USA	A/Texas/JMS389/2009(H1N1)

NA	KC780642	H1N1	12/16/2009	USA	A/Connecticut/18/2009
NA	KC780461	H1N1	12/16/2009	USA	A/Mississippi/09/2009
NA	CY058406	H1N1	12/16/2009	USA	A/Wisconsin/629-D01038/2009(H1N1)
NA	CY058446	H1N1	12/16/2009	USA	A/Wisconsin/629-D02073/2009(H1N1)
NA	CY066046	H1N1	12/16/2009	Japan	A/Fukushima/09FY058/2009
NA	CY123308	H1N1	12/16/2009	Singapore	A/Singapore/GP4778/2009
NA	KC782236	H1N1	12/16/2009	USA	A/Michigan/29/2009
NA	KC780485	H1N1	12/16/2009	USA	A/Tennessee/19/2009
NA	HQ247715	H1N1	12/16/2009	Finland	A/Finland/688/2009
NA	CY062004	H1N1	12/16/2009	USA	A/New York/7036/2009(H1N1)
NA	MF196267	H1N1	12/17/2009	Iran	A/Tehran/88-9-221/2009
NA	CY072472	H1N1	12/17/2009	Greece	A/Athens/INS342/2009(H1N1)
NA	CY063671	H1N1	12/17/2009	China	A/Shandong-Wenshan/SWL41/2009(H1N1)
NA	CY063672	H1N1	12/17/2009	China	A/Shandong-Wenshan/SWL42/2009(H1N1)
NA	CY072464	H1N1	12/17/2009	Greece	A/Athens/INS341/2009(H1N1)
NA	CY083807	H1N1	12/17/2009	Spain	A/Madrid/INS377/2009(H1N1)
NA	CY072456	H1N1	12/17/2009	Greece	A/Athens/INS340/2009(H1N1)
NA	CY062949	H1N1	12/17/2009	Spain	A/Terrassa/INS173/2009(H1N1)
NA	CY067778	H1N1	12/17/2009	Portugal	A/Lisboa/153/2009(H1N1)
NA	CY062853	H1N1	12/17/2009	Greece	A/Athens/INS158/2009(H1N1)
NA	KC780428	H1N1	12/17/2009	USA	A/Virginia/27/2009
NA	KC782330	H1N1	12/17/2009	USA	A/Kansas/25/2009
NA	CY058438	H1N1	12/17/2009	USA	A/Wisconsin/629-D01556/2009(H1N1)
NA	CY128389	H1N1	12/17/2009	Viet Nam	A/Viet Nam/11032010/2009
NA	CY128056	H1N1	12/17/2009	Viet Nam	A/Viet Nam/11032011/2009
NA	CY067161	H1N1	12/17/2009	Greece	A/Athens/INS276/2009(H1N1)
NA	KC780445	H1N1	12/17/2009	USA	A/Virginia/27/2009
NA	CY072320	H1N1	12/17/2009	USA	A/New York/INS317/2009(H1N1)
NA	KX651649	H1N1	12/17/2009	France	A/France/1641-P5T/2009
NA	CY062933	H1N1	12/18/2009	Germany	A/Berlin/INS171/2009(H1N1)
NA	CY072488	H1N1	12/18/2009	Greece	A/Athens/INS345/2009(H1N1)
NA	KX651654	H1N1	12/18/2009	France	A/France/365-1T/2009
NA	KX651622	H1N1	12/18/2009	France	A/France/556-P2T/2009
NA	CY078369	H1N1	12/18/2009	Romania	A/Ilfov/138/2009
NA	CY071812	H1N1	12/18/2009	Mali	A/Bamako/WR2361N/2009(H1N1)
NA	KF687937	H1N1	12/18/2009	Cyprus	A/Cyprus/625/2009
NA	CY062869	H1N1	12/18/2009	Greece	A/Athens/INS160/2009(H1N1)
NA	CY062861	H1N1	12/18/2009	Greece	A/Athens/INS159/2009(H1N1)
NA	CY063656	H1N1	12/18/2009	Greece	A/Athens/INS157/2009(H1N1)
NA	CY058414	H1N1	12/18/2009	USA	A/Wisconsin/629-D00459/2009(H1N1)
NA	AB704536	H1N1	12/18/2009	Japan	A/Yamaguchi/273/2009
NA	GU592891	H1N1	12/18/2009	Russia	A/Barnaul/04/2009(H1N1)
NA	KC781233	H1N1	12/18/2009	USA	A/Maine/14/2009
NA	CY080260	H1N1	12/18/2009	China	A/Nanchang/8009/2009
NA	JN187194	H1N1	12/18/2009	Taiwan	A/Taiwan/11706/2009
NA	CY072480	H1N1	12/18/2009	Greece	A/Athens/INS344/2009(H1N1)
NA	CY060491	H1N1	12/18/2009	South Ko	A/Seoul/1870/2009(H1N1)
NA	CY062877	H1N1	12/19/2009	Greece	A/Athens/INS161/2009(H1N1)
NA	CY087869	H1N1	12/19/2009	Thailand	A/Thailand/CU-H1127/2009
NA	KR870138	H1N1	12/19/2009	India	A/Bangalore/11406-11/2009
NA	CY089618	H1N1	12/19/2009	China	A/Nanchang/8008/2009
NA	HM230703	H1N1	12/19/2009	China	A/Zhoushan/52/2009(H1N1)
NA	KC780571	H1N1	12/19/2009	USA	A/Delaware/18/2009
NA	CY066172	H1N1	12/19/2009	Japan	A/Nagasaki/09N105/2009

NA	HM072414	H1N1	12/19/2009	Taiwan	A/Taiwan/2343/2009
NA	CY072496	H1N1	12/20/2009	Greece	A/Athens/INS346/2009(H1N1)
NA	CY062885	H1N1	12/20/2009	Greece	A/Athens/INS162/2009(H1N1)
NA	CY063694	H1N1	12/20/2009	China	A/Shandong-Shizhong/SWL45/2009(H1N1)
NA	CY063693	H1N1	12/20/2009	China	A/Shandong-Shizhong/SWL1944/2009(H1N
NA	CY062893	H1N1	12/20/2009	Greece	A/Athens/INS163/2009(H1N1)
NA	CY069156	H1N1	12/20/2009	Greece	A/Athens/INS352/2009(H1N1)
NA	CY072512	H1N1	12/20/2009	Greece	A/Athens/INS348/2009(H1N1)
NA	CY072368	H1N1	12/20/2009	Greece	A/Athens/INS327/2009(H1N1)
NA	CY072360	H1N1	12/20/2009	Greece	A/Athens/INS325/2009(H1N1)
NA	CY073507	H1N1	12/20/2009	Greece	A/Athens/INS350/2009(H1N1)
NA	CY072504	H1N1	12/20/2009	Greece	A/Athens/INS347/2009(H1N1)
NA	CY062901	H1N1	12/20/2009	Greece	A/Athens/INS164/2009(H1N1)
NA	CY065093	H1N1	12/20/2009	USA	A/New York/7425/2009(H1N1)
NA	CY107427	H1N1	12/20/2009	United Ki	A/Scotland/Dundee_09V502385/2009
NA	CY061037	H1N1	12/20/2009	USA	A/Texas/JMS390/2009(H1N1)
NA	CY072520	H1N1	12/20/2009	Greece	A/Athens/INS349/2009(H1N1)
NA	CY054582	H1N1	12/21/2009	Spain	A/Catalonia/S2144/2009(H1N1)
NA	CY054579	H1N1	12/21/2009	Spain	A/Catalonia/S2143/2009(H1N1)
NA	CY062997	H1N1	12/21/2009	Estonia	A/Tallinn/INS181/2009(H1N1)
NA	CY062909	H1N1	12/21/2009	Greece	A/Athens/INS165/2009(H1N1)
NA	CY062020	H1N1	12/21/2009	USA	A/New York/7216/2009(H1N1)
NA	CY067772	H1N1	12/21/2009	Portugal	A/Lisboa/120/2009(H1N1)
NA	CY069172	H1N1	12/21/2009	Greece	A/Athens/INS354/2009(H1N1)
NA	CY069164	H1N1	12/21/2009	Greece	A/Athens/INS353/2009(H1N1)
NA	CY088623	H1N1	12/21/2009	Denmark	A/Odense/INS176/2009
NA	AB649900	H1N1	12/21/2009	Japan	A/Toyama/444/2009
NA	KT181564	H1N1	12/21/2009	Singapore	A/Singapore/DMS61/2009
NA	CY088633	H1N1	12/21/2009	India	A/Pune/NIV0953725/2009(H1N1)
NA	CY066132	H1N1	12/21/2009	Japan	A/Kyoto/09K347/2009
NA	KC781270	H1N1	12/21/2009	USA	A/New York/109/2009
NA	CY107434	H1N1	12/21/2009	United Ki	A/Scotland/Dundee_09V502390/2009
NA	KX651651	H1N1	12/21/2009	France	A/France/3971-3R/2009
NA	CY066393	H1N1	12/22/2009	USA	A/California/VRDL127/2009(H1N1)
NA	CY054585	H1N1	12/22/2009	Spain	A/Catalonia/S2151/2009(H1N1)
NA	CY069244	H1N1	12/22/2009	Austria	A/Vienna/INS366/2009(H1N1)
NA	KC780294	H1N1	12/22/2009	USA	A/Vermont/30/2009
NA	CY069180	H1N1	12/22/2009	Greece	A/Athens/INS355/2009(H1N1)
NA	CY067760	H1N1	12/22/2009	Portugal	A/Lisboa/105/2009(H1N1)
NA	CY066385	H1N1	12/22/2009	USA	A/California/VRDL126/2009(H1N1)
NA	CY066369	H1N1	12/22/2009	USA	A/California/VRDL124/2009(H1N1)
NA	CY066377	H1N1	12/22/2009	USA	A/California/VRDL125/2009(H1N1)
NA	HQ239870	H1N1	12/22/2009	Canada	A/Canada-MB/RV2014-10/2009
NA	CY089626	H1N1	12/22/2009	China	A/Nanchang/8011/2009
NA	HQ239869	H1N1	12/22/2009	Canada	A/Canada-MB/RV2013-10/2009
NA	CY071113	H1N1	12/22/2009	Germany	A/Wurzburg/INS381/2009(H1N1)
NA	AB649912	H1N1	12/22/2009	Japan	A/Toyama/446/2009
NA	CY063695	H1N1	12/22/2009	China	A/Shandong-Rencheng/SWL412/2009(H1N
NA	CY066401	H1N1	12/22/2009	USA	A/California/VRDL128/2009(H1N1)
NA	CY067757	H1N1	12/22/2009	Portugal	A/Lisboa/104/2009(H1N1)
NA	CY071065	H1N1	12/23/2009	Estonia	A/Tallinn/INS372/2009(H1N1)
NA	CY062012	H1N1	12/23/2009	USA	A/New York/7107/2009(H1N1)
NA	CY065085	H1N1	12/23/2009	USA	A/New York/7424/2009(H1N1)
NA	CY061045	H1N1	12/23/2009	USA	A/Texas/JMS391/2009(H1N1)

NA	LC093024	H1N1	12/23/2009	Saudi Ara	A/Riyadh/4783/2009
NA	LC093034	H1N1	12/23/2009	Saudi Ara	A/Riyadh/5086/2009
NA	CY087875	H1N1	12/23/2009	Thailand	A/Thailand/CU-H1140/2009
NA	CY054588	H1N1	12/23/2009	Spain	A/Catalonia/S2169/2009(H1N1)
NA	CY078388	H1N1	12/23/2009	Romania	A/Botosani/552/2009
NA	HM581924	H1N1	12/23/2009	Iran	A/Iran/1519/2009(H1N1)
NA	CY107441	H1N1	12/23/2009	United Ki	A/Scotland/Perth_09V502475/2009
NA	CY128397	H1N1	12/23/2009	Viet Nam	A/Viet Nam/11032016/2009
NA	CY128405	H1N1	12/23/2009	Viet Nam	A/Viet Nam/11032017/2009
NA	KC780331	H1N1	12/23/2009	USA	A/South Carolina/46/2009
NA	CY066048	H1N1	12/23/2009	Japan	A/Fukushima/09FY071/2009
NA	LN867654	H1N1	12/23/2009	United Ki	A/England/01220639/2009
NA	CY071201	H1N1	12/23/2009	Germany	A/Frankfurt/INS401/2009(H1N1)
NA	CY073256	H1N1	12/23/2009	Greece	A/Athens/WRAIR2962N/2009(H1N1)
NA	CY062965	H1N1	12/23/2009	Spain	A/Terrassa/INS175/2009(H1N1)
NA	HQ247727	H1N1	12/24/2009	Finland	A/Finland/701/2009
NA	CY062957	H1N1	12/24/2009	Spain	A/Terrassa/INS174/2009(H1N1)
NA	KX651666	H1N1	12/24/2009	Russia	A/Russia/519-1T/2009
NA	LC093023	H1N1	12/24/2009	Saudi Ara	A/Riyadh/1639/2010
NA	CY067781	H1N1	12/24/2009	Portugal	A/Lisboa/154/2009(H1N1)
NA	CY066176	H1N1	12/24/2009	Japan	A/Nagasaki/09N114/2009
NA	CY072376	H1N1	12/25/2009	Greece	A/Athens/INS329/2009(H1N1)
NA	KT181567	H1N1	12/25/2009	Singapore	A/Singapore/DMS62/2009
NA	KC780529	H1N1	12/25/2009	USA	A/Maryland/37/2009
NA	CY061053	H1N1	12/25/2009	USA	A/Texas/JMS392/2009(H1N1)
NA	CY071121	H1N1	12/25/2009	Germany	A/Wurzburg/INS382/2009(H1N1)
NA	CY062925	H1N1	12/26/2009	Greece	A/Athens/INS167/2009(H1N1)
NA	CY062917	H1N1	12/26/2009	Greece	A/Athens/INS166/2009(H1N1)
NA	CY069188	H1N1	12/26/2009	Greece	A/Athens/INS357/2009(H1N1)
NA	CY069204	H1N1	12/26/2009	Greece	A/Athens/INS359/2009(H1N1)
NA	CY062036	H1N1	12/26/2009	USA	A/New York/0259/2009(H1N1)
NA	CY072392	H1N1	12/26/2009	Greece	A/Athens/INS332/2009(H1N1)
NA	CY069196	H1N1	12/26/2009	Greece	A/Athens/INS358/2009(H1N1)
NA	CY073008	H1N1	12/26/2009	Greece	A/Athens/INS330/2009(H1N1)
NA	CY072384	H1N1	12/26/2009	Greece	A/Athens/INS331/2009(H1N1)
NA	KR870139	H1N1	12/26/2009	India	A/Bangalore/11658-15/2009
NA	KC780233	H1N1	12/27/2009	USA	A/Montana/25/2009
NA	CY063676	H1N1	12/27/2009	China	A/Shandong-Qufu/SWL326/2009(H1N1)
NA	CY063668	H1N1	12/27/2009	China	A/Shandong-Shizhong/SWL21/2009(H1N1
NA	LC093019	H1N1	12/27/2009	Saudi Ara	A/Riyadh/4972/2009
NA	CY063680	H1N1	12/27/2009	China	A/Shandong-Qufu/SWL339/2009(H1N1)
NA	CY088610	H1N1	12/27/2009	Greece	A/Athens/INS152/2009
NA	CY063679	H1N1	12/27/2009	China	A/Shandong-Qufu/SWL338/2009(H1N1)
NA	HM780490	H1N1	12/27/2009	China	A/Guangdong/0872/2009(H1N1)
NA	CY063682	H1N1	12/27/2009	China	A/Shandong-Rencheng/SWL44/2009(H1N1
NA	LC093016	H1N1	12/27/2009	Saudi Ara	A/Riyadh/4994/2009
NA	CY062044	H1N1	12/27/2009	USA	A/New York/0352/2009(H1N1)
NA	CY063677	H1N1	12/27/2009	China	A/Shandong-Qufu/SWL329/2009(H1N1)
NA	CY061061	H1N1	12/27/2009	USA	A/Texas/JMS393/2009(H1N1)
NA	CY072400	H1N1	12/28/2009	Greece	A/Athens/INS333/2009(H1N1)
NA	CY067763	H1N1	12/28/2009	Portugal	A/Lisboa/106/2009(H1N1)
NA	AB649902	H1N1	12/28/2009	Japan	A/Toyama/451/2009
NA	KT181569	H1N1	12/28/2009	Singapore	A/Singapore/DMS63/2009
NA	CY066178	H1N1	12/28/2009	Japan	A/Nagasaki/09N116/2009

NA	CY062028	H1N1	12/28/2009	USA	A/New York/7236/2009(H1N1)
NA	KC780482	H1N1	12/28/2009	USA	A/Michigan/28/2009
NA	KC780320	H1N1	12/28/2009	USA	A/Georgia/25/2009
NA	AB649903	H1N1	12/28/2009	Japan	A/Toyama/458/2009
NA	LC093030	H1N1	12/28/2009	Saudi Ara	A/Riyadh/5019/2009
NA	CY066050	H1N1	12/28/2009	Japan	A/Fukushima/09FY079/2009
NA	CY062973	H1N1	12/28/2009	Denmark	A/Odense/INS177/2009(H1N1)
NA	CY072336	H1N1	12/28/2009	USA	A/Newark/INS319/2009(H1N1)
NA	CY063640	H1N1	12/29/2009	Greece	A/Athens/INS153/2009(H1N1)
NA	KX651655	H1N1	12/29/2009	France	A/France/2580-1T/2009
NA	CY071241	H1N1	12/29/2009	Poland	A/Warsaw/INS406/2009(H1N1)
NA	JN187195	H1N1	12/29/2009	Taiwan	A/Taiwan/1017/2009
NA	CY064782	H1N1	12/29/2009	Spain	A/Barcelona/INS190/2009(H1N1)
NA	CY072408	H1N1	12/29/2009	Greece	A/Athens/INS334/2009(H1N1)
NA	KX651681	H1N1	12/29/2009	Spain	A/Spain/55-1T/2009
NA	CY071233	H1N1	12/29/2009	Poland	A/Warsaw/INS405/2009(H1N1)
NA	CY063648	H1N1	12/29/2009	Greece	A/Athens/INS154/2009(H1N1)
NA	CY078385	H1N1	12/30/2009	Romania	A/Constanta/363/2009
NA	KC780670	H1N1	12/30/2009	USA	A/Nebraska/23/2009
NA	KC780630	H1N1	12/30/2009	USA	A/Rhode Island/25/2009
NA	CY123314	H1N1	12/30/2009	Singapore	A/Singapore/GP4895/2009
NA	CY128413	H1N1	12/30/2009	Viet Nam	A/Viet Nam/11032021/2009
NA	JN716360	H1N1	12/30/2009	China	A/Suzhou/J1461/2009
NA	HM568130	H1N1	12/30/2009	United Ki	A/England/938/2009
NA	LN846369	H1N1	12/30/2009	United Ki	A/England/WTSI1778/2009
NA	CY072440	H1N1	12/30/2009	Greece	A/Athens/INS338/2009(H1N1)
NA	CY072416	H1N1	12/30/2009	Greece	A/Athens/INS335/2009(H1N1)
NA	CY072424	H1N1	12/30/2009	Greece	A/Athens/INS336/2009(H1N1)
NA	CY072448	H1N1	12/30/2009	Greece	A/Athens/INS339/2009(H1N1)
NA	CY072432	H1N1	12/30/2009	Greece	A/Athens/INS337/2009(H1N1)
NA	CY062837	H1N1	12/30/2009	Greece	A/Athens/INS155/2009(H1N1)
NA	KF411186	H1N1	12/30/2009	China	A/Qingdao/FF86/2009
NA	KF411185	H1N1	12/30/2009	China	A/Qingdao/FF85/2009
NA	KF687938	H1N1	12/30/2009	Cyprus	A/Cyprus/653/2009
NA	JQ714189	H1N1	12/30/2009	China	A/Tianjinjinnan/SWL4141/2009
NA	CY062052	H1N1	12/30/2009	USA	A/New York/0357/2009(H1N1)
NA	CY063029	H1N1	12/30/2009	Spain	A/Madrid/INS185/2009(H1N1)
NA	CY066409	H1N1	12/30/2009	USA	A/California/VRDL129/2009(H1N1)
NA	CY066417	H1N1	12/30/2009	USA	A/California/VRDL130/2009(H1N1)
NA	CY066433	H1N1	12/30/2009	USA	A/California/VRDL132/2009(H1N1)
NA	CY066441	H1N1	12/30/2009	USA	A/California/VRDL133/2009(H1N1)
NA	CY061069	H1N1	12/30/2009	USA	A/Texas/JMS394/2009(H1N1)
NA	CY063037	H1N1	12/30/2009	Spain	A/Madrid/INS186/2009(H1N1)
NA	CY066425	H1N1	12/30/2009	USA	A/California/VRDL131/2009(H1N1)
NA	CY062060	H1N1	12/30/2009	USA	A/New York/0461/2009(H1N1)
NA	CY061077	H1N1	12/30/2009	USA	A/Texas/JMS395/2009(H1N1)
NA	CY061093	H1N1	12/30/2009	USA	A/Texas/JMS398/2009(H1N1)
NA	CY062845	H1N1	12/30/2009	Greece	A/Athens/INS156/2009(H1N1)
NA	CY063061	H1N1	12/30/2009	Belgium	A/Tessenderlo/INS192/2009(H1N1)
NA	CY061085	H1N1	12/30/2009	USA	A/Texas/JMS397/2009(H1N1)
NA	CY078390	H1N1	12/30/2009	Romania	A/Botosani/565/2009
NA	CY087881	H1N1	12/30/2009	Thailand	A/Thailand/CU-H1167/2009
NA	KX651656	H1N1	12/30/2009	France	A/France/3739-1T/2009
NA	JN037763	H1N1	12/30/2009	Tunisia	A/Tunisia/21516/2009

NA	KC780868	H1N1	12/31/2009	USA	A/Idaho/17/2009
NA	KP233640	H1N1	12/31/2009	China	A/JiangxiDonghu/SWL11181/2009
NA	CY123318	H1N1	12/31/2009	Singapore	A/Singapore/GP4907/2009
NA	CY107448	H1N1	12/31/2009	United Ki	A/Scotland/Dundee_09V502666/2009
NA	GU576541	H1N1	12/31/2009	Italy	A/Ancona/508/2009(H1N1)
NA	KP019731	H1N1	12/31/2009	China	A/FuZhou/SWL12496/2009
NA	KT181571	H1N1	12/31/2009	Singapore	A/Singapore/DMS64/2009
NA	CY071129	H1N1	12/31/2009	Greece	A/Athens/INS383/2009(H1N1)
NA	CY067787	H1N1	12/31/2009	Portugal	A/Lisboa/160/2009(H1N1)
NA	CY061101	H1N1	12/31/2009	USA	A/Texas/JMS399/2009(H1N1)
NA	KC780662	H1N1	12/31/2009	USA	A/Minnesota/38/2009
NA	KP233580	H1N1	12/31/2009	China	A/JiangxiDonghu/1123/2009
NA	KP233579	H1N1	12/31/2009	China	A/JiangxiGuixi/174/2009
NA	KP233582	H1N1	12/31/2009	China	A/JiangxiDonghu/1816/2009
NA	CY061572	H1N1	12/31/2009	USA	A/Texas/JMS400/2009(H1N1)
NA	KP233578	H1N1	12/31/2009	China	A/JiangxiYuanzhou/14/2009
NA	HM625671	H1N1	12/31/2009	Italy	A/Rome/PTV25/2009
NA	KP233650	H1N1	12/31/2009	China	A/JiangxiXingzi/SWL57/2009
NA	KP233653	H1N1	12/31/2009	China	A/JiangxiZhushan/SWL1/2009
NA	KP233655	H1N1	12/31/2009	China	A/JiangxiDonghu/SWL536/2009
NA	KP233581	H1N1	12/31/2009	China	A/JiangxiGuixi/5126/2009
NA	KP233651	H1N1	12/31/2009	China	A/JiangxiAnyuan/SWL511/2009
NA	KP233652	H1N1	12/31/2009	China	A/JiangxiDonghu/SWL11116/2009
NA	KR870140	H1N1	12/31/2009	India	A/Bangalore/12220-18/2009
NA	KP233658	H1N1	12/31/2009	China	A/JiangxiDonghu/SWL11171/2009
NA	KP233657	H1N1	12/31/2009	China	A/JiangxiQingshanhu/SWL55/2009
NA	KP233656	H1N1	12/31/2009	China	A/JiangxiAnyuan/SWL541/2009
NA	CY123324	H1N1	12/31/2009	Singapore	A/Singapore/GP4915/2009
NA	HQ190930	H1N1	01/2010	Cuba	A/Habana/7374/2010
NA	CY060453	H1N1	01/2010	South Ko	A/Gwangju/61/2010(H1N1)
NA	CY060455	H1N1	01/2010	South Ko	A/Seoul/7/2010(H1N1)
NA	HM598349	H1N1	01/2010	Thailand	A/Thailand/CU-MV46/2010
NA	CY078372	H1N1	01/2010	Romania	A/Prahova/146/2010
NA	CY078386	H1N1	01/2010	Romania	A/Prahova/404/2010
NA	JN006487	H1N1	01/2010	China	A/Hangzhou/111/2010
NA	JN006486	H1N1	01/2010	China	A/Hangzhou/110/2010
NA	JN006485	H1N1	01/2010	China	A/Hangzhou/15/2010
NA	JN006488	H1N1	01/2010	China	A/Hangzhou/115/2010
NA	CY120026	H1N1	01/2010	Mexico	A/Mexico/InDRECTRLA/2010
NA	JN381401	H1N1	01/2010	Taiwan	A/Taiwan/65052/2010
NA	JN381404	H1N1	01/2010	Taiwan	A/Taiwan/65211/2010
NA	JN381402	H1N1	01/2010	Taiwan	A/Taiwan/65078/2010
NA	JN381399	H1N1	01/2010	Taiwan	A/Taiwan/90001/2010
NA	JN381405	H1N1	01/2010	Taiwan	A/Taiwan/65229/2010
NA	JN381403	H1N1	01/2010	Taiwan	A/Taiwan/90019/2010
NA	JN381400	H1N1	01/2010	Taiwan	A/Taiwan/65048/2010
NA	JN381406	H1N1	01/2010	Taiwan	A/Taiwan/90027/2010
NA	KC780545	H1N1	01/01/2010	USA	A/New Jersey/01/2010
NA	KC780407	H1N1	01/01/2010	USA	A/Utah/01/2010
NA	KT424160	H1N1	01/01/2010	China	A/Shandong-Shizhong/SWL11/2010
NA	CY082964	H1N1	01/01/2010	Thailand	A/Thailand/CU-H1222r/2010
NA	CY087936	H1N1	01/01/2010	Thailand	A/Thailand/CU-H1379/2010
NA	KC780309	H1N1	01/01/2010	USA	A/Minnesota/01/2010
NA	KC780526	H1N1	01/02/2010	USA	A/Kansas/01/2010

NA	CY067784	H1N1	01/02/2010	Portugal	A/Lisboa/159/2009(H1N1)
NA	JQ714190	H1N1	01/02/2010	China	A/Tianjinnankai/SWL49/2010
NA	KX651682	H1N1	01/02/2010	Spain	A/Spain/55-3R/2010
NA	KM213312	H1N1	01/02/2010	Oman	A/Oman/SQUH-40/2010
NA	KC781434	H1N1	01/02/2010	USA	A/Hawaii/02/2010
NA	KM985432	H1N1	01/02/2010	India	A/Trivandrum/MCVR2613/2010
NA	KC781360	H1N1	01/02/2010	USA	A/Hawaii/02/2010
NA	KM213305	H1N1	01/02/2010	Oman	A/Oman/SQUH-32/2010
NA	KM213307	H1N1	01/02/2010	Oman	A/Oman/SQUH-34/2010
NA	CY080266	H1N1	01/02/2010	China	A/Shantou/8064/2010
NA	CY065069	H1N1	01/02/2010	USA	A/New York/7420/2010(H1N1)
NA	CY061580	H1N1	01/03/2010	USA	A/Texas/JMS401/2010(H1N1)
NA	KC781237	H1N1	01/03/2010	USA	A/Hawaii/01/2010
NA	LC093045	H1N1	01/03/2010	Saudi Ara	A/Riyadh/5107/2010
NA	KM213306	H1N1	01/03/2010	Oman	A/Oman/SQUH-33/2010
NA	KC780573	H1N1	01/03/2010	USA	A/New Hampshire/01/2010
NA	KC781178	H1N1	01/03/2010	USA	A/Hawaii/01/2010
NA	CY078384	H1N1	01/04/2010	Romania	A/Covasna/357/2010
NA	HM598322	H1N1	01/04/2010	Thailand	A/Thailand/CU-MV9/2010
NA	CY063005	H1N1	01/04/2010	Estonia	A/Tallinn/INS182/2010(H1N1)
NA	CY061588	H1N1	01/04/2010	USA	A/Texas/JMS403/2010(H1N1)
NA	CY096588	H1N1	01/04/2010	USA	A/District of Columbia/INS525/2010(I
NA	KT181572	H1N1	01/04/2010	Singapore	A/Singapore/DMS65/2010
NA	CY061109	H1N1	01/04/2010	USA	A/Texas/JMS402/2010(H1N1)
NA	CY067799	H1N1	01/04/2010	Portugal	A/Lisboa/2/2010(H1N1)
NA	HM145748	H1N1	01/04/2010	China	A/Haishu/SWL110/2010(H1N1)
NA	GU562460	H1N1	01/04/2010	Russia	A/Karasuk/01/2010(H1N1)
NA	KC780619	H1N1	01/04/2010	USA	A/Alabama/01/2010
NA	KC781901	H1N1	01/04/2010	USA	A/Alabama/01/2010
NA	CY065874	H1N1	01/04/2010	Netherlar	A/Netherlands/2631b/2010
NA	JX308951	H1N1	01/04/2010	Singapore	A/Singapore/GP10/2010
NA	JX309123	H1N1	01/05/2010	Singapore	A/Singapore/KK04/2010
NA	KC782016	H1N1	01/05/2010	USA	A/Iowa/01/2010
NA	KC780225	H1N1	01/05/2010	USA	A/Colorado/02/2010
NA	KC781019	H1N1	01/05/2010	USA	A/New Mexico/01/2010
NA	JX308959	H1N1	01/05/2010	Singapore	A/Singapore/GP33/2010
NA	JX308987	H1N1	01/05/2010	Singapore	A/Singapore/TT01/2010
NA	CY062981	H1N1	01/05/2010	Germany	A/Bonn/INS178/2010(H1N1)
NA	CY071057	H1N1	01/05/2010	Austria	A/Vienna/INS368/2010(H1N1)
NA	CY080262	H1N1	01/05/2010	China	A/Shantou/8049/2010
NA	CY080256	H1N1	01/05/2010	China	A/Shantou/8028/2010
NA	CY062989	H1N1	01/05/2010	Austria	A/Vienna/INS179/2010(H1N1)
NA	CY073775	H1N1	01/05/2010	Austria	A/Vienna/INS369/2010(H1N1)
NA	JQ714191	H1N1	01/05/2010	China	A/Tianjinnankai/SWL413/2010
NA	MF196261	H1N1	01/05/2010	Iran	A/Tehran/88-10-72/2010
NA	AB649901	H1N1	01/05/2010	Japan	A/Toyama/1/2010
NA	CY080264	H1N1	01/06/2010	China	A/Shantou/8012/2010
NA	JX309127	H1N1	01/06/2010	Singapore	A/Singapore/KK12/2010
NA	CY075061	H1N1	01/06/2010	Greece	A/Athens/INS392/2010(H1N1)
NA	CY075069	H1N1	01/06/2010	Greece	A/Athens/INS393/2010(H1N1)
NA	JX309083	H1N1	01/06/2010	Singapore	A/Singapore/TT10/2010
NA	JX309079	H1N1	01/06/2010	Singapore	A/Singapore/EN008/2010
NA	JX308967	H1N1	01/06/2010	Singapore	A/Singapore/GP64/2010
NA	CY067814	H1N1	01/06/2010	Portugal	A/Lisboa/4/2010(H1N1)

NA	KM213308	H1N1	01/06/2010	Oman	A/Oman/SQUH-35/2010
NA	KC780412	H1N1	01/06/2010	USA	A/Florida/01/2010
NA	CY066180	H1N1	01/06/2010	Japan	A/Nagasaki/09N117/2010
NA	CY071073	H1N1	01/07/2010	Estonia	A/Tallinn/INS373/2010(H1N1)
NA	CY100510	H1N1	01/07/2010	Mexico	A/Mexico City/INER17/2010
NA	CY066052	H1N1	01/07/2010	Japan	A/Fukushima/09FY084/2010
NA	CY072528	H1N1	01/07/2010	Estonia	A/Tallinn/INS374/2010(H1N1)
NA	JX309095	H1N1	01/07/2010	Singapore	A/Singapore/GP95/2010
NA	CY063013	H1N1	01/07/2010	Estonia	A/Tallinn/INS183/2010(H1N1)
NA	KX651683	H1N1	01/07/2010	Spain	A/Spain/55-10T/2010
NA	LC093033	H1N1	01/07/2010	Saudi Ara	A/Riyadh/5212/2010
NA	JX309091	H1N1	01/07/2010	Singapore	A/Singapore/GP75/2010
NA	KT181574	H1N1	01/07/2010	Singapore	A/Singapore/DMS66/2010
NA	KX651657	H1N1	01/07/2010	France	A/France/3619-1T/2010
NA	CY061117	H1N1	01/08/2010	USA	A/Texas/JMS404/2010(H1N1)
NA	CY071209	H1N1	01/08/2010	Germany	A/Frankfurt/INS402/2010(H1N1)
NA	KP019732	H1N1	01/08/2010	China	A/FuZhou/SWL172/2010
NA	CY056295	H1N1	01/08/2010	China	A/Henan/1/2010(H1N1)
NA	LC093022	H1N1	01/08/2010	Saudi Ara	A/Riyadh/5230/2009
NA	HM598323	H1N1	01/08/2010	Thailand	A/Thailand/CU-MV10/2010
NA	CY063856	H1N1	01/08/2010	Singapore	A/Singapore/TT017/2010(H1N1)
NA	CY066449	H1N1	01/09/2010	USA	A/California/VRDL1/2010(H1N1)
NA	KC780616	H1N1	01/09/2010	USA	A/North Carolina/02/2010
NA	GU576543	H1N1	01/09/2010	Italy	A/Ancona/01/2010(H1N1)
NA	KM213309	H1N1	01/09/2010	Oman	A/Oman/SQUH-36/2010
NA	CY067817	H1N1	01/09/2010	Portugal	A/Lisboa/5/2010(H1N1)
NA	CY087887	H1N1	01/09/2010	Thailand	A/Thailand/CU-H1210/2010
NA	CY061125	H1N1	01/09/2010	USA	A/Texas/JMS405/2010(H1N1)
NA	KJ958969	H1N1	01/10/2010	India	A/Mumbai/4593/2010
NA	CY061133	H1N1	01/10/2010	USA	A/Texas/JMS406/2010(H1N1)
NA	LC093020	H1N1	01/10/2010	Saudi Ara	A/Riyadh/5281/2010
NA	KP317314	H1N1	01/10/2010	India	A/Rajasthan/019/2010
NA	KX651658	H1N1	01/10/2010	France	A/France/3619-3T/2010
NA	CY066457	H1N1	01/11/2010	USA	A/California/VRDL2/2010(H1N1)
NA	CY071161	H1N1	01/11/2010	Greece	A/Athens/INS394/2010(H1N1)
NA	CY061141	H1N1	01/11/2010	USA	A/Texas/JMS407/2010(H1N1)
NA	CY071217	H1N1	01/11/2010	Germany	A/Frankfurt/INS403/2010(H1N1)
NA	JX308975	H1N1	01/11/2010	Singapore	A/Singapore/GP132/2010
NA	KT181576	H1N1	01/11/2010	Singapore	A/Singapore/DMS67/2010
NA	KM213310	H1N1	01/11/2010	Oman	A/Oman/SQUH-37/2010
NA	CY100516	H1N1	01/11/2010	Mexico	A/Mexico City/INER18/2010
NA	JX308979	H1N1	01/11/2010	Singapore	A/Singapore/GP155/2010
NA	JX309087	H1N1	01/12/2010	Singapore	A/Singapore/TT31/2010
NA	JQ612509	H1N1	01/12/2010	Hungary	A/Hungary/2/2010
NA	KX651659	H1N1	01/12/2010	France	A/France/3619-5T/2010
NA	JX308983	H1N1	01/12/2010	Singapore	A/Singapore/GP174/2010
NA	AB704488	H1N1	01/12/2010	Japan	A/Tochigi/10/2010
NA	KP637672	H1N1	01/12/2010	Thailand	A/Thailand/SP08358/2010
NA	HQ247734	H1N1	01/12/2010	Finland	A/Finland/2/2010
NA	CY061149	H1N1	01/12/2010	USA	A/Texas/JMS408/2010(H1N1)
NA	KC780312	H1N1	01/12/2010	USA	A/New York/02/2010
NA	KC780889	H1N1	01/12/2010	USA	A/Nevada/01/2010
NA	KC781893	H1N1	01/12/2010	USA	A/North Carolina/01/2010
NA	JX309099	H1N1	01/12/2010	Singapore	A/Singapore/GP166/2010

NA	CY062068	H1N1	01/12/2010	USA	A/New York/1680/2010(H1N1)
NA	JX309131	H1N1	01/12/2010	Singapore	A/Singapore/KK25/2010
NA	JX309107	H1N1	01/13/2010	Singapore	A/Singapore/GP205/2010
NA	CY074984	H1N1	01/13/2010	Thailand	A/Thailand/CU-H1222/2010(H1N1)
NA	CY071137	H1N1	01/13/2010	Greece	A/Athens/INS389/2010(H1N1)
NA	KC780645	H1N1	01/13/2010	USA	A/Maryland/03/2010
NA	KC780946	H1N1	01/13/2010	USA	A/Texas/01/2010
NA	JX309103	H1N1	01/13/2010	Singapore	A/Singapore/GP189/2010
NA	CY087893	H1N1	01/13/2010	Thailand	A/Thailand/CU-H1224/2010
NA	CY056386	H1N1	01/13/2010	Greece	A/Thessaloniki/225/2010
NA	HQ247735	H1N1	01/13/2010	Finland	A/Finland/3/2010
NA	AB704480	H1N1	01/13/2010	Japan	A/Tochigi/2/2010
NA	CY088668	H1N1	01/13/2010	India	A/Ngp/NIV22704/2010(H1N1)
NA	LC093044	H1N1	01/13/2010	Saudi Ara	A/Riyadh/5325/2010
NA	KT181578	H1N1	01/14/2010	Singapore	A/Singapore/DMS68/2010
NA	KM213311	H1N1	01/14/2010	Oman	A/Oman/SQUH-38/2010
NA	LN867806	H1N1	01/14/2010	United Ki	A/England/01220740/2010
NA	JX309135	H1N1	01/14/2010	Singapore	A/Singapore/TT37/2010
NA	CY066054	H1N1	01/14/2010	Japan	A/Fukushima/09FY088/2010
NA	KC780420	H1N1	01/14/2010	USA	A/Indiana/01/2010
NA	KC780236	H1N1	01/14/2010	USA	A/Kentucky/01/2010
NA	CY071145	H1N1	01/14/2010	Greece	A/Athens/INS390/2010(H1N1)
NA	CY072536	H1N1	01/14/2010	USA	A/New York/INS432/2010(H1N1)
NA	JX309003	H1N1	01/14/2010	Singapore	A/Singapore/GP220/2010
NA	JX309019	H1N1	01/15/2010	Singapore	A/Singapore/GP256/2010
NA	JN631040	H1N1	01/15/2010	China	A/Shanghai/2167T/2010
NA	CY062076	H1N1	01/15/2010	USA	A/New York/1796/2010(H1N1)
NA	CY073969	H1N1	01/15/2010	Argentina	A/Argentina/HNRG102/2010
NA	JX309011	H1N1	01/15/2010	Singapore	A/Singapore/GP241/2010
NA	KC781919	H1N1	01/15/2010	USA	A/Delaware/01/2010
NA	KP317317	H1N1	01/15/2010	India	A/Jammu & Kashmir/022/2010
NA	HM598319	H1N1	01/16/2010	Thailand	A/Thailand/CU-MV6/2010
NA	KT181579	H1N1	01/16/2010	Singapore	A/Singapore/DMS69/2010
NA	KC781244	H1N1	01/16/2010	USA	A/Arizona/01/2010
NA	KX651660	H1N1	01/16/2010	France	A/France/3619-P5T/2010
NA	KC782006	H1N1	01/16/2010	USA	A/Connecticut/01/2010
NA	KC780364	H1N1	01/17/2010	USA	A/Pennsylvania/01/2010
NA	CY062092	H1N1	01/18/2010	USA	A/New York/1999/2010(H1N1)
NA	HQ286458	H1N1	01/18/2010	Japan	A/Kurume/L12/2010
NA	HQ286445	H1N1	01/18/2010	Japan	A/Kurume/L21/2010
NA	HQ286432	H1N1	01/18/2010	Japan	A/Kurume/N10/2010
NA	HQ286421	H1N1	01/18/2010	Japan	A/Kurume/R5/2010
NA	HQ286454	H1N1	01/18/2010	Japan	A/Kurume/L8/2010
NA	HQ286419	H1N1	01/18/2010	Japan	A/Kurume/R3/2010
NA	HQ286428	H1N1	01/18/2010	Japan	A/Kurume/N6/2010
NA	HQ286430	H1N1	01/18/2010	Japan	A/Kurume/N8/2010
NA	HQ286442	H1N1	01/18/2010	Japan	A/Kurume/L18/2010
NA	HQ286450	H1N1	01/18/2010	Japan	A/Kurume/L4/2010
NA	HQ286424	H1N1	01/18/2010	Japan	A/Kurume/N2/2010
NA	HQ286456	H1N1	01/18/2010	Japan	A/Kurume/L10/2010
NA	HQ286434	H1N1	01/18/2010	Japan	A/Kurume/R7/2010
NA	HQ286436	H1N1	01/18/2010	Japan	A/Kurume/R9/2010
NA	HQ286459	H1N1	01/18/2010	Japan	A/Kurume/L13/2010
NA	HQ286457	H1N1	01/18/2010	Japan	A/Kurume/L11/2010

NA	HQ286443	H1N1	01/18/2010	Japan	A/Kurume/L19/2010
NA	HQ286439	H1N1	01/18/2010	Japan	A/Kurume/R12/2010
NA	HQ286446	H1N1	01/18/2010	Japan	A/Kurume/L22/2010
NA	HQ286449	H1N1	01/18/2010	Japan	A/Kurume/L3/2010
NA	HQ286427	H1N1	01/18/2010	Japan	A/Kurume/N5/2010
NA	HQ286461	H1N1	01/18/2010	Japan	A/Kurume/L15/2010
NA	HQ286460	H1N1	01/18/2010	Japan	A/Kurume/L14/2010
NA	HQ286420	H1N1	01/18/2010	Japan	A/Kurume/R4/2010
NA	HQ286425	H1N1	01/18/2010	Japan	A/Kurume/N3/2010
NA	HQ286433	H1N1	01/18/2010	Japan	A/Kurume/N11/2010
NA	HQ286429	H1N1	01/18/2010	Japan	A/Kurume/N7/2010
NA	HQ286440	H1N1	01/18/2010	Japan	A/Kurume/L16/2010
NA	HQ286453	H1N1	01/18/2010	Japan	A/Kurume/L7/2010
NA	HQ286447	H1N1	01/18/2010	Japan	A/Kurume/L1/2010
NA	HQ286422	H1N1	01/18/2010	Japan	A/Kurume/R6/2010
NA	HQ286451	H1N1	01/18/2010	Japan	A/Kurume/L5/2010
NA	HQ286435	H1N1	01/18/2010	Japan	A/Kurume/R8/2010
NA	HQ286431	H1N1	01/18/2010	Japan	A/Kurume/N9/2010
NA	KT424159	H1N1	01/18/2010	China	A/Shandong-Lixia/SWL123/2010
NA	HQ286438	H1N1	01/18/2010	Japan	A/Kurume/R11/2010
NA	HQ286423	H1N1	01/18/2010	Japan	A/Kurume/N1/2010
NA	JN037764	H1N1	01/18/2010	Tunisia	A/Tunisia/1064/2010
NA	HQ286417	H1N1	01/18/2010	Japan	A/Kurume/R1/2010
NA	HQ286418	H1N1	01/18/2010	Japan	A/Kurume/R2/2010
NA	HQ286426	H1N1	01/18/2010	Japan	A/Kurume/N4/2010
NA	HQ286437	H1N1	01/18/2010	Japan	A/Kurume/R10/2010
NA	HQ286444	H1N1	01/18/2010	Japan	A/Kurume/L20/2010
NA	HQ286452	H1N1	01/18/2010	Japan	A/Kurume/L6/2010
NA	HQ286455	H1N1	01/18/2010	Japan	A/Kurume/L9/2010
NA	CY087899	H1N1	01/18/2010	Thailand	A/Thailand/CU-H1256/2010
NA	CY078363	H1N1	01/18/2010	Romania	A/Prahova/026/2010
NA	CY100524	H1N1	01/18/2010	Mexico	A/Mexico City/INER9/2010
NA	CY129501	H1N1	01/18/2010	Germany	A/Wurzburg/INS578/2010
NA	KC780568	H1N1	01/18/2010	USA	A/Georgia/03/2010
NA	LN846440	H1N1	01/18/2010	United Ki	A/England/32/2010
NA	JX309111	H1N1	01/18/2010	Singapore	A/Singapore/GP266/2010
NA	CY071297	H1N1	01/18/2010	Greece	A/Athens/INS417/2010(H1N1)
NA	CY080317	H1N1	01/18/2010	Thailand	A/Thailand/H1255/2010
NA	CY062084	H1N1	01/18/2010	USA	A/New York/1998/2010(H1N1)
NA	CY066465	H1N1	01/19/2010	USA	A/California/VRDL3/2010(H1N1)
NA	CY167774	H1N1	01/19/2010	USA	A/Tennessee/F1035/2010
NA	JX309119	H1N1	01/19/2010	Singapore	A/Singapore/GP311/2010
NA	CY071153	H1N1	01/19/2010	Greece	A/Athens/INS391/2010(H1N1)
NA	KC782180	H1N1	01/19/2010	USA	A/Mississippi/01/2010
NA	JX309115	H1N1	01/19/2010	Singapore	A/Singapore/GP293/2010
NA	CY062100	H1N1	01/19/2010	USA	A/New York/2175/2010(H1N1)
NA	KC782066	H1N1	01/19/2010	USA	A/California/01/2010
NA	JX308995	H1N1	01/20/2010	Singapore	A/Singapore/GP329/2010
NA	CY062108	H1N1	01/20/2010	USA	A/New York/2372/2010(H1N1)
NA	KC780333	H1N1	01/20/2010	USA	A/North Carolina/03/2010
NA	CY062116	H1N1	01/20/2010	USA	A/New York/2598/2010(H1N1)
NA	KX651704	H1N1	01/20/2010	Thailand	A/Thailand/2355-1T/2010
NA	CY061157	H1N1	01/20/2010	USA	A/Texas/JMS409/2010(H1N1)
NA	CY063674	H1N1	01/20/2010	China	A/Shandong-Shizhong/SWL134/2010(H1N

NA	CY062132	H1N1	01/20/2010	USA	A/New York/2876/2010(H1N1)
NA	KC780581	H1N1	01/21/2010	USA	A/Virginia/01/2010
NA	KT424158	H1N1	01/21/2010	China	A/Shandong-Bincheng/SWL1155/2010
NA	HM598321	H1N1	01/21/2010	Thailand	A/Thailand/CU-MV8/2010
NA	HM598320	H1N1	01/21/2010	Thailand	A/Thailand/CU-MV7/2010
NA	KT424157	H1N1	01/21/2010	China	A/Shandong-Fushan/SWL131/2010
NA	KF849747	H1N1	01/21/2010	Thailand	A/Thailand/SirirajICRC_SEA-001(34)/2
NA	CY071169	H1N1	01/21/2010	Greece	A/Athens/INS396/2010(H1N1)
NA	KF849745	H1N1	01/21/2010	Thailand	A/Thailand/SirirajICRC_SEA-002(33)/2
NA	KP019733	H1N1	01/21/2010	China	A/FuZhou/SWL1223/2010
NA	JX309027	H1N1	01/21/2010	Singapore	A/Singapore/GP368/2010
NA	HM598350	H1N1	01/22/2010	Thailand	A/Thailand/CU-MV47/2010
NA	KX651705	H1N1	01/22/2010	Thailand	A/Thailand/2355-3T/2010
NA	CY061165	H1N1	01/22/2010	USA	A/Texas/JMS410/2010(H1N1)
NA	CY062148	H1N1	01/22/2010	USA	A/New York/2963/2010(H1N1)
NA	KT181581	H1N1	01/22/2010	Singapore	A/Singapore/DMS71/2010
NA	CY062124	H1N1	01/22/2010	USA	A/New York/2648/2010(H1N1)
NA	KC781420	H1N1	01/23/2010	USA	A/Kansas/02/2010
NA	KC780352	H1N1	01/23/2010	USA	A/Alabama/02/2010
NA	CY062156	H1N1	01/23/2010	USA	A/New York/2971/2010(H1N1)
NA	CY087905	H1N1	01/23/2010	Thailand	A/Thailand/CU-H1301/2010
NA	KC780443	H1N1	01/24/2010	USA	A/Georgia/02/2010
NA	CY063698	H1N1	01/24/2010	China	A/Shandong-Shizhong/SWL24/2010(H1N1)
NA	CY063697	H1N1	01/24/2010	China	A/Shandong-Shizhong/SWL14/2010(H1N1)
NA	KC780465	H1N1	01/24/2010	USA	A/Wisconsin/01/2010
NA	CY071177	H1N1	01/24/2010	Greece	A/Athens/INS397/2010(H1N1)
NA	CY063667	H1N1	01/24/2010	China	A/Shandong-Wenshan/SWL42/2010(H1N1)
NA	CY063696	H1N1	01/24/2010	China	A/Shandong-Shizhong/SWL11/2010(H1N1)
NA	KC780496	H1N1	01/24/2010	USA	A/Wisconsin/01/2010
NA	CY071185	H1N1	01/24/2010	Greece	A/Athens/INS398/2010(H1N1)
NA	CY063205	H1N1	01/25/2010	USA	A/California/VRDL13/2010(H1N1)
NA	CY087918	H1N1	01/25/2010	Thailand	A/Thailand/CU-H1313/2010
NA	CY087912	H1N1	01/25/2010	Thailand	A/Thailand/CU-H1310/2010
NA	KX651706	H1N1	01/25/2010	Thailand	A/Thailand/2355-5T/2010
NA	KC780425	H1N1	01/25/2010	USA	A/Kentucky/02/2010
NA	CY083026	H1N1	01/25/2010	Senegal	A/Dakar/WRAIR0018T/2010
NA	CY083018	H1N1	01/25/2010	Senegal	A/Dakar/WRAIR0018N/2010
NA	CY073448	H1N1	01/25/2010	Mongolia	A/Dundgobi/381/2010
NA	KT181582	H1N1	01/25/2010	Singapore	A/Singapore/DMS72/2010
NA	JX309043	H1N1	01/25/2010	Singapore	A/Singapore/GP398/2010
NA	JX309035	H1N1	01/25/2010	Singapore	A/Singapore/GP413/2010
NA	CY063213	H1N1	01/25/2010	USA	A/California/VRDL14/2010(H1N1)
NA	CY062164	H1N1	01/25/2010	USA	A/New York/3230/2010(H1N1)
NA	JF340084	H1N1	01/25/2010	Russia	A/St.Petersburg/14/2010
NA	CY061173	H1N1	01/25/2010	USA	A/Texas/JMS411/2010(H1N1)
NA	CY071305	H1N1	01/25/2010	Greece	A/Athens/INS419/2010(H1N1)
NA	CY073128	H1N1	01/25/2010	Mexico	A/Mexico City/WRAIR1773T/2010(H1N1)
NA	JX309059	H1N1	01/25/2010	Singapore	A/Singapore/GP437/2010(H1N1)
NA	CY083537	H1N1	01/26/2010	Mexico	A/Mexico City/WRAIR1752N/2010(H1N1)
NA	JX309051	H1N1	01/26/2010	Singapore	A/Singapore/GP449/2010(H1N1)
NA	CY083034	H1N1	01/26/2010	Senegal	A/Dakar/WRAIR0019T/2010
NA	CY062172	H1N1	01/26/2010	USA	A/New York/3236/2010(H1N1)
NA	HQ247737	H1N1	01/26/2010	Finland	A/Finland/5/2010
NA	GU931313	H1N1	01/26/2010	Italy	A/Italy/Pisa254/2010(H1N1)

NA	CY062188	H1N1	01/26/2010	USA	A/New York/3251/2010(H1N1)
NA	CY062140	H1N1	01/26/2010	USA	A/New York/2960/2010(H1N1)
NA	KC780872	H1N1	01/26/2010	USA	A/California/03/2010
NA	CY080309	H1N1	01/26/2010	Thailand	A/Thailand/C602/2010(H1N1)
NA	CY063189	H1N1	01/26/2010	USA	A/California/VRDL11/2010(H1N1)
NA	JX308945	H1N1	01/27/2010	Singapore	A/Singapore/GP479/2010
NA	CY063850	H1N1	01/27/2010	Singapore	A/Singapore/KK066/2010(H1N1)
NA	KP019734	H1N1	01/27/2010	China	A/FuZhou/SWL1286/2010
NA	KC780659	H1N1	01/27/2010	USA	A/South Carolina/01/2010
NA	JX309067	H1N1	01/27/2010	Singapore	A/Singapore/GP489/2010(H1N1)
NA	CY065021	H1N1	01/27/2010	USA	A/New York/3834/2010(H1N1)
NA	KC781449	H1N1	01/27/2010	USA	A/Michigan/01/2010
NA	CY087924	H1N1	01/27/2010	Thailand	A/Thailand/CU-H1340/2010
NA	KX651707	H1N1	01/27/2010	Thailand	A/Thailand/3563-1T/2010
NA	CY057074	H1N1	01/27/2010	Mexico	A/Mexico/InDRE797/2010
NA	CY063197	H1N1	01/27/2010	USA	A/California/VRDL12/2010(H1N1)
NA	CY071313	H1N1	01/27/2010	Greece	A/Athens/INS420/2010(H1N1)
NA	CY065029	H1N1	01/27/2010	USA	A/New York/3835/2010(H1N1)
NA	CY092640	H1N1	01/27/2010	Australia	A/Sydney/DD3-37/2010
NA	CY062180	H1N1	01/27/2010	USA	A/New York/3250/2010(H1N1)
NA	KP317315	H1N1	01/28/2010	India	A/Assam/020/2010
NA	KF849748	H1N1	01/28/2010	Thailand	A/Thailand/SirirajICRC_SEA-003(33)/2
NA	KP317319	H1N1	01/28/2010	India	A/Madhya Pradesh/024/2010
NA	CY087930	H1N1	01/28/2010	Thailand	A/Thailand/CU-H1351/2010
NA	KC780448	H1N1	01/28/2010	USA	A/Nebraska/01/2010
NA	JX309075	H1N1	01/28/2010	Singapore	A/Singapore/GP512/2010(H1N1)
NA	CY065037	H1N1	01/29/2010	USA	A/New York/3866/2010(H1N1)
NA	JX309138	H1N1	01/29/2010	Singapore	A/Singapore/GP521/2010
NA	CY066182	H1N1	01/30/2010	Japan	A/Nagasaki/09N121/2010
NA	CY071265	H1N1	01/30/2010	Greece	A/Athens/INS411/2010(H1N1)
NA	CY078362	H1N1	01/30/2010	Romania	A/Neamt/01/2010
NA	CY087942	H1N1	01/30/2010	Thailand	A/Thailand/CU-H1392/2010
NA	KT181585	H1N1	01/31/2010	Singapore	A/Singapore/DMS74/2010
NA	HQ606473	H1N1	02/2010	Iran	A/Shiraz/14/2010
NA	JN006491	H1N1	02/2010	China	A/Hangzhou/125/2010
NA	JN006489	H1N1	02/2010	China	A/Hangzhou/117/2010
NA	JN006492	H1N1	02/2010	China	A/Hangzhou/129/2010
NA	JN006490	H1N1	02/2010	China	A/Hangzhou/124/2010
NA	JN381408	H1N1	02/2010	Taiwan	A/Taiwan/65337/2010
NA	JN381407	H1N1	02/2010	Taiwan	A/Taiwan/65299/2010
NA	KX651708	H1N1	02/01/2010	Thailand	A/Thailand/3563-5T/2010
NA	KC780603	H1N1	02/01/2010	USA	A/California/02/2010
NA	KC780291	H1N1	02/01/2010	USA	A/Colorado/03/2010
NA	KC780314	H1N1	02/01/2010	USA	A/Massachusetts/01/2010
NA	CY065013	H1N1	02/01/2010	USA	A/New York/3683/2010(H1N1)
NA	KC780950	H1N1	02/01/2010	USA	A/New Mexico/02/2010
NA	JX309212	H1N1	02/01/2010	Singapore	A/Singapore/KK70/2010
NA	CY064997	H1N1	02/01/2010	USA	A/New York/3681/2010(H1N1)
NA	CY061181	H1N1	02/01/2010	USA	A/Texas/JMS412/2010(H1N1)
NA	CY066489	H1N1	02/01/2010	USA	A/California/VRDL7/2010(H1N1)
NA	CY065005	H1N1	02/01/2010	USA	A/New York/3682/2010(H1N1)
NA	JX465469	H1N1	02/01/2010	South Ko	A/Gyeonggi/42/2010
NA	HM598333	H1N1	02/01/2010	Thailand	A/Thailand/CU-MV26/2010
NA	HM598334	H1N1	02/01/2010	Thailand	A/Thailand/CU-MV27/2010

NA	CY064989	H1N1	02/02/2010	USA	A/California/VRDL6/2010(H1N1)
NA	KC781410	H1N1	02/02/2010	USA	A/Georgia/09/2010
NA	KC782069	H1N1	02/02/2010	USA	A/Arizona/02/2010
NA	KC780388	H1N1	02/02/2010	USA	A/North Carolina/04/2010
NA	CY063832	H1N1	02/02/2010	Singapore	A/Singapore/GP562/2010(H1N1)
NA	JX309158	H1N1	02/02/2010	Singapore	A/Singapore/GP567/2010
NA	KC780539	H1N1	02/02/2010	USA	A/Florida/02/2010
NA	HQ247736	H1N1	02/02/2010	Finland	A/Finland/4/2010
NA	KP019735	H1N1	02/02/2010	China	A/FuZhou/SWL1338/2010
NA	KT181594	H1N1	02/02/2010	Singapore	A/Singapore/DMS82/2010
NA	CY071273	H1N1	02/02/2010	Greece	A/Athens/INS412/2010(H1N1)
NA	KF849744	H1N1	02/02/2010	Thailand	A/Thailand/SirirajICRC_SEA-002(34)/2
NA	HM598335	H1N1	02/03/2010	Thailand	A/Thailand/CU-MV28/2010
NA	KC781457	H1N1	02/03/2010	USA	A/Louisiana/01/2010
NA	CY061189	H1N1	02/03/2010	USA	A/Texas/JMS413/2010(H1N1)
NA	KC780693	H1N1	02/03/2010	USA	A/Indiana/03/2010
NA	CY071321	H1N1	02/03/2010	Greece	A/Athens/INS421/2010(H1N1)
NA	KT181586	H1N1	02/03/2010	Singapore	A/Singapore/DMS75/2010
NA	KX651709	H1N1	02/03/2010	Thailand	A/Thailand/2396-1T/2010
NA	CY073000	H1N1	02/03/2010	Nicaragua	A/Managua/07193_09/2010(H1N1)
NA	CY065053	H1N1	02/03/2010	USA	A/New York/4662/2010(H1N1)
NA	CY065045	H1N1	02/04/2010	USA	A/New York/4294/2010(H1N1)
NA	CY167326	H1N1	02/04/2010	USA	A/Tennessee/F1042A/2010
NA	CY066056	H1N1	02/04/2010	Japan	A/Fukushima/09FY090/2010
NA	CY066481	H1N1	02/04/2010	USA	A/California/VRDL5/2010(H1N1)
NA	MF196262	H1N1	02/04/2010	Iran	A/Tehran/88-11-70/2010
NA	JX556123	H1N1	02/04/2010	Cuba	A/Habana/8232/2010
NA	LN850032	H1N1	02/04/2010	United Ki	A/England/29/2010
NA	CY107455	H1N1	02/04/2010	United Ki	A/Scotland/Dundee_10V070791/2010
NA	LN846496	H1N1	02/04/2010	United Ki	A/England/01220736/2010
NA	JF929788	H1N1	02/04/2010	China	A/Guangdong/2315/2010
NA	KC782021	H1N1	02/04/2010	USA	A/Vermont/01/2010
NA	CY061197	H1N1	02/04/2010	USA	A/Texas/JMS414/2010(H1N1)
NA	KF849746	H1N1	02/05/2010	Thailand	A/Thailand/SirirajICRC_SEA-003(34)/2
NA	CY073735	H1N1	02/05/2010	Estonia	A/Tallinn/INS431/2010(H1N1)
NA	CY071377	H1N1	02/05/2010	Germany	A/Berlin/INS430/2010(H1N1)
NA	CY071281	H1N1	02/05/2010	Greece	A/Athens/INS413/2010(H1N1)
NA	CY071289	H1N1	02/05/2010	Greece	A/Athens/INS414/2010(H1N1)
NA	KT181588	H1N1	02/05/2010	Singapore	A/Singapore/DMS76/2010
NA	CY083819	H1N1	02/05/2010	USA	A/Los Angeles/INS423/2010(H1N1)
NA	JX309179	H1N1	02/05/2010	Singapore	A/Singapore/GP643/2010
NA	KC781256	H1N1	02/05/2010	USA	A/Georgia/06/2010
NA	KC781298	H1N1	02/05/2010	USA	A/Georgia/05/2010
NA	KC781283	H1N1	02/06/2010	USA	A/Georgia/07/2010
NA	KC781267	H1N1	02/06/2010	USA	A/Georgia/04/2010
NA	CY061205	H1N1	02/06/2010	USA	A/Texas/JMS415/2010(H1N1)
NA	KC780241	H1N1	02/06/2010	USA	A/Georgia/01/2010
NA	KC781387	H1N1	02/07/2010	USA	A/Alaska/01/2010
NA	JX309182	H1N1	02/07/2010	Singapore	A/Singapore/KK77/2010
NA	KP637288	H1N1	02/07/2010	Thailand	A/Thailand/IY07115/2010
NA	KP317320	H1N1	02/07/2010	India	A/Chattisgarh/025/2010
NA	KC780723	H1N1	02/08/2010	USA	A/Hawaii/03/2010
NA	KC780585	H1N1	02/08/2010	USA	A/Maryland/04/2010
NA	HM598336	H1N1	02/08/2010	Thailand	A/Thailand/CU-MV29/2010

NA	JX309169	H1N1	02/08/2010	Singapore	A/Singapore/TT106/2010
NA	JX309162	H1N1	02/08/2010	Singapore	A/Singapore/GP661/2010
NA	KT181589	H1N1	02/08/2010	Singapore	A/Singapore/DMS77/2010
NA	CY066473	H1N1	02/08/2010	USA	A/California/VRDL4/2010(H1N1)
NA	HM598337	H1N1	02/08/2010	Thailand	A/Thailand/CU-MV30/2010
NA	KC781183	H1N1	02/08/2010	USA	A/Wyoming/01/2010
NA	CY167334	H1N1	02/09/2010	USA	A/Tennessee/F1052A/2010
NA	KP637840	H1N1	02/09/2010	Thailand	A/Thailand/IY07144/2010
NA	CY100532	H1N1	02/09/2010	Mexico	A/Mexico City/INER10/2010
NA	JX309203	H1N1	02/09/2010	Singapore	A/Singapore/GP680/2010
NA	CY090863	H1N1	02/09/2010	Netherlar	A/Netherlands/2631c/2010(H1N1)
NA	KC261328	H1N1	02/09/2010	Mexico	A/Mexico City/INERPHAC3/2010(H1N1)
NA	CY066505	H1N1	02/09/2010	USA	A/California/VRDL9/2010(H1N1)
NA	CY100540	H1N1	02/09/2010	Mexico	A/Mexico City/INER11/2010
NA	KC780451	H1N1	02/09/2010	USA	A/New York/03/2010
NA	KT181590	H1N1	02/10/2010	Singapore	A/Singapore/DMS78/2010
NA	JX309206	H1N1	02/10/2010	Singapore	A/Singapore/GP690/2010
NA	KC780503	H1N1	02/10/2010	USA	A/Utah/02/2010
NA	KC781429	H1N1	02/10/2010	USA	A/Maine/01/2010
NA	KP019736	H1N1	02/10/2010	China	A/FuZhou/SWL1395/2010
NA	KC780351	H1N1	02/11/2010	USA	A/Delaware/02/2010
NA	KC780626	H1N1	02/11/2010	USA	A/Indiana/02/2010
NA	KC781035	H1N1	02/11/2010	USA	A/California/04/2010
NA	CY063181	H1N1	02/11/2010	USA	A/California/VRDL10/2010(H1N1)
NA	JX556124	H1N1	02/11/2010	Cuba	A/Habana/8427/2010
NA	JX309188	H1N1	02/11/2010	Singapore	A/Singapore/GP720/2010
NA	JX309173	H1N1	02/12/2010	Singapore	A/Singapore/GP735/2010
NA	JX309209	H1N1	02/12/2010	Singapore	A/Singapore/KK80/2010
NA	CY063836	H1N1	02/12/2010	Singapore	A/Singapore/GP737/2010(H1N1)
NA	CY063854	H1N1	02/12/2010	Singapore	A/Singapore/KK081/2010(H1N1)
NA	JF906185	H1N1	02/12/2010	Netherlar	A/Netherlands/2631_1202/2010
NA	KJ856116	H1N1	02/12/2010	Sri Lanka	A/Sri Lanka/20887/2010
NA	CY066497	H1N1	02/13/2010	USA	A/California/VRDL8/2010(H1N1)
NA	KX651710	H1N1	02/14/2010	Thailand	A/Thailand/2429-1T/2010
NA	JQ247202	H1N1	02/14/2010	Philippir	A/Philippines/TMC10-8/2010
NA	KC780368	H1N1	02/15/2010	USA	A/Iowa/03/2010
NA	CY066058	H1N1	02/15/2010	Japan	A/Fukushima/09FY091/2010
NA	KX651711	H1N1	02/16/2010	Thailand	A/Thailand/2429-3T/2010
NA	CY097791	H1N1	02/16/2010	Senegal	A/Dakar/WRAIR0020T/2010
NA	CY083042	H1N1	02/16/2010	Senegal	A/Dakar/WRAIR0020T/2010
NA	KX651714	H1N1	02/16/2010	Thailand	A/Thailand/550-1T/2010
NA	CY075077	H1N1	02/17/2010	Greece	A/Athens/INS416/2010(H1N1)
NA	JX309185	H1N1	02/17/2010	Singapore	A/Singapore/KK87/2010
NA	KC782002	H1N1	02/17/2010	USA	A/Minnesota/02/2010
NA	KC780523	H1N1	02/17/2010	USA	A/Wisconsin/02/2010
NA	KX651715	H1N1	02/18/2010	Thailand	A/Thailand/550-3T/2010
NA	KP637864	H1N1	02/18/2010	Thailand	A/Thailand/SS08248/2010
NA	CY167350	H1N1	02/18/2010	USA	A/Tennessee/F1060/2010
NA	CY167342	H1N1	02/18/2010	USA	A/Tennessee/F1057c56/2010
NA	KC781198	H1N1	02/19/2010	USA	A/Kentucky/03/2010
NA	KC780070	H1N1	02/19/2010	USA	A/New Hampshire/02/2010
NA	KC781922	H1N1	02/19/2010	USA	A/New Hampshire/02/2010
NA	HM598353	H1N1	02/19/2010	Thailand	A/Thailand/CU-MV50/2010
NA	LN867351	H1N1	02/20/2010	United Ki	A/England/01220741/2010

NA	KT181591	H1N1	02/20/2010	Singapore	A/Singapore/DMS80/2010
NA	KX651716	H1N1	02/20/2010	Thailand	A/Thailand/550-5T/2010
NA	KX651712	H1N1	02/20/2010	Thailand	A/Thailand/2429-7T/2010
NA	CY065061	H1N1	02/21/2010	USA	A/New York/6530/2010(H1N1)
NA	HM598352	H1N1	02/22/2010	Thailand	A/Thailand/CU-MV49/2010
NA	KC781369	H1N1	02/22/2010	USA	A/Wisconsin/04/2010
NA	KC781162	H1N1	02/22/2010	USA	A/Kentucky/04/2010
NA	JX309176	H1N1	02/22/2010	Singapore	A/Singapore/GP758/2010
NA	KX651782	H1N1	02/23/2010	USA	A/USA/3061-1T/2010
NA	AB649913	H1N1	02/23/2010	Japan	A/Toyama/62/2010
NA	CY096596	H1N1	02/23/2010	USA	A/District of Columbia/INS527/2010(H
NA	KC782138	H1N1	02/23/2010	USA	A/Tennessee/01/2010
NA	CY071329	H1N1	02/23/2010	Thailand	A/Bangkok/INS424/2010(H1N1)
NA	HM598351	H1N1	02/23/2010	Thailand	A/Thailand/CU-MV48/2010
NA	KX651713	H1N1	02/23/2010	Thailand	A/Thailand/2429-10T/2010
NA	CY129696	H1N1	02/24/2010	Greece	A/Athens/INS555/2010
NA	KC781257	H1N1	02/24/2010	USA	A/Michigan/02/2010
NA	KC780359	H1N1	02/24/2010	USA	A/North Carolina/05/2010
NA	JX309197	H1N1	02/24/2010	Singapore	A/Singapore/GP784/2010
NA	KT181593	H1N1	02/24/2010	Singapore	A/Singapore/DMS81/2010
NA	CY071345	H1N1	02/25/2010	Thailand	A/Bangkok/INS426/2010(H1N1)
NA	KP019737	H1N1	02/25/2010	China	A/FuZhou/SWL1494/2010
NA	CY071337	H1N1	02/25/2010	Thailand	A/Bangkok/INS425/2010(H1N1)
NA	JX309191	H1N1	02/25/2010	Singapore	A/Singapore/TT114/2010
NA	KC781914	H1N1	02/25/2010	USA	A/New York/04/2010
NA	CY063842	H1N1	02/26/2010	Singapore	A/Singapore/GP999/2010(H1N1)
NA	JX309200	H1N1	02/26/2010	Singapore	A/Singapore/GP811/2010
NA	JX309194	H1N1	02/26/2010	Singapore	A/Singapore/EN35/2010
NA	CY167358	H1N1	02/26/2010	USA	A/Tennessee/F1063A/2010
NA	CY167366	H1N1	02/26/2010	USA	A/Tennessee/F1066c65/2010
NA	CY167374	H1N1	02/26/2010	USA	A/Tennessee/F1067/2010
NA	HM149350	H1N1	02/26/2010	USA	A/Georgia/10/2010(H1N1)
NA	HQ190932	H1N1	03/2010	Cuba	A/Habana/9937/2010
NA	HQ190931	H1N1	03/2010	Cuba	A/Habana/9765/2010
NA	JN006496	H1N1	03/2010	China	A/Hangzhou/178/2010
NA	JN006493	H1N1	03/2010	China	A/Hangzhou/141/2010
NA	JN006495	H1N1	03/2010	China	A/Hangzhou/157/2010
NA	JN006494	H1N1	03/2010	China	A/Hangzhou/156/2010
NA	AB649904	H1N1	03/01/2010	Japan	A/Toyama/68/2010
NA	KC781168	H1N1	03/01/2010	USA	A/Alabama/03/2010
NA	KT181595	H1N1	03/01/2010	Singapore	A/Singapore/DMS83/2010
NA	JX309281	H1N1	03/01/2010	Singapore	A/Singapore/GP820/2010
NA	JX309260	H1N1	03/01/2010	Singapore	A/Singapore/EN39/2010
NA	CY065109	H1N1	03/01/2010	USA	A/New York/7480/2010(H1N1)
NA	KT424161	H1N1	03/01/2010	China	A/Shandong-Yinyuan/SWL52/2010
NA	CY167382	H1N1	03/02/2010	USA	A/Tennessee/F1068/2010
NA	HM569742	H1N1	03/02/2010	Russia	A/Orenburg/IIV-13/2010(H1N1)
NA	HM124385	H1N1	03/02/2010	Russia	A/Orenburg/IIV-13/2010(H1N1)
NA	KC781912	H1N1	03/02/2010	USA	A/Massachusetts/02/2010
NA	JX309272	H1N1	03/02/2010	Singapore	A/Singapore/KK90/2010
NA	JQ247199	H1N1	03/02/2010	Philippir	A/Philippines/TMC10-27/2010
NA	KC781414	H1N1	03/02/2010	USA	A/Pennsylvania/03/2010
NA	CY167406	H1N1	03/02/2010	USA	A/Tennessee/F1072c71/2010
NA	CY167390	H1N1	03/02/2010	USA	A/Tennessee/F1071/2010

NA	HM598357	H1N1	03/03/2010	Thailand	A/Thailand/CU-MV54/2010
NA	KC781350	H1N1	03/03/2010	USA	A/Iowa/04/2010
NA	HM598358	H1N1	03/03/2010	Thailand	A/Thailand/CU-MV55/2010
NA	KX651717	H1N1	03/03/2010	Thailand	A/Thailand/3426-1T/2010
NA	CY071361	H1N1	03/04/2010	Thailand	A/Bangkok/INS428/2010(H1N1)
NA	CY071353	H1N1	03/04/2010	Thailand	A/Bangkok/INS427/2010(H1N1)
NA	KC780737	H1N1	03/04/2010	USA	A/California/05/2010
NA	CY167398	H1N1	03/05/2010	USA	A/Tennessee/F1071B/2010
NA	KX651718	H1N1	03/05/2010	Thailand	A/Thailand/3426-3T/2010
NA	KC781371	H1N1	03/05/2010	USA	A/South Carolina/02/2010
NA	CY071369	H1N1	03/05/2010	USA	A/Newark/INS429/2010(H1N1)
NA	HM598355	H1N1	03/05/2010	Thailand	A/Thailand/CU-MV52/2010
NA	KC780611	H1N1	03/06/2010	USA	A/Arizona/03/2010
NA	KC780728	H1N1	03/07/2010	USA	A/New Mexico/03/2010
NA	KC781342	H1N1	03/07/2010	USA	A/Kansas/03/2010
NA	JF929791	H1N1	03/07/2010	China	A/Guangdong/2989/2010
NA	HM598356	H1N1	03/08/2010	Thailand	A/Thailand/CU-MV53/2010
NA	KX651719	H1N1	03/08/2010	Thailand	A/Thailand/3426-5T/2010
NA	KC781864	H1N1	03/08/2010	USA	A/Mississippi/02/2010
NA	KC781408	H1N1	03/08/2010	USA	A/Utah/03/2010
NA	JX309257	H1N1	03/08/2010	Singapore	A/Singapore/KK98/2010
NA	JX309269	H1N1	03/08/2010	Singapore	A/Singapore/GP866/2010
NA	KC780589	H1N1	03/08/2010	USA	A/Hawaii/04/2010
NA	KP019738	H1N1	03/08/2010	China	A/FuZhou/SWL1549/2010
NA	KP019739	H1N1	03/08/2010	China	A/FuZhou/SWL1565/2010
NA	KC781174	H1N1	03/09/2010	USA	A/North Carolina/06/2010
NA	HM598359	H1N1	03/09/2010	Thailand	A/Thailand/CU-MV56/2010
NA	KP019740	H1N1	03/10/2010	China	A/FuZhou/SWL1563/2010
NA	JX309236	H1N1	03/10/2010	Singapore	A/Singapore/KK100/2010(H1N1)
NA	HM598354	H1N1	03/11/2010	Thailand	A/Thailand/CU-MV51/2010
NA	KC781400	H1N1	03/11/2010	USA	A/Nebraska/02/2010
NA	CY090132	H1N1	03/11/2010	Thailand	A/Thailand/CU-B2296/2010
NA	KC781336	H1N1	03/12/2010	USA	A/Minnesota/03/2010
NA	KC781209	H1N1	03/12/2010	USA	A/Wisconsin/03/2010
NA	KC781160	H1N1	03/12/2010	USA	A/Tennessee/02/2010
NA	KT181598	H1N1	03/12/2010	Singapore	A/Singapore/DMS86/2010
NA	JX309275	H1N1	03/12/2010	Singapore	A/Singapore/GP903/2010
NA	KX651742	H1N1	03/13/2010	USA	A/USA/16-1T/2010
NA	CY081175	H1N1	03/14/2010	Thailand	A/Thailand/CU-H1782/2010
NA	JX309247	H1N1	03/15/2010	Singapore	A/Singapore/TT123/2010
NA	KC781176	H1N1	03/15/2010	USA	A/Alabama/04/2010
NA	CY167414	H1N1	03/15/2010	USA	A/Tennessee/F1074/2010
NA	CY167422	H1N1	03/16/2010	USA	A/Tennessee/F1076/2010
NA	CY167438	H1N1	03/16/2010	USA	A/Tennessee/F1078/2010
NA	KT181601	H1N1	03/16/2010	Singapore	A/Singapore/DMS88/2010
NA	KC781141	H1N1	03/16/2010	USA	A/Florida/05/2010
NA	LN867375	H1N1	03/18/2010	United Ki	A/England/WTSI1774/2010
NA	LN850127	H1N1	03/18/2010	United Ki	A/England/11/2010
NA	LN850347	H1N1	03/18/2010	United Ki	A/England/01180018/2010
NA	LN845776	H1N1	03/18/2010	United Ki	A/England/12/2010
NA	LN867782	H1N1	03/18/2010	United Ki	A/England/01180011/2010
NA	LN867425	H1N1	03/18/2010	United Ki	A/England/01180015/2010
NA	LN846789	H1N1	03/18/2010	United Ki	A/England/10/2010
NA	LN867488	H1N1	03/18/2010	United Ki	A/England/01180017/2010

NA	LN850080	H1N1	03/18/2010	United Ki	A/England/00380710/2010
NA	LN867822	H1N1	03/18/2010	United Ki	A/England/WTSI2076/2010
NA	LN850423	H1N1	03/18/2010	United Ki	A/England/01180007/2010
NA	LN846512	H1N1	03/18/2010	United Ki	A/England/01180016/2010
NA	KC781273	H1N1	03/18/2010	USA	A/Florida/04/2010
NA	KC781431	H1N1	03/18/2010	USA	A/Florida/04/2010
NA	CY081169	H1N1	03/18/2010	Thailand	A/Thailand/CU-H1774/2010
NA	LN846536	H1N1	03/18/2010	United Ki	A/England/00380719/2010
NA	LN846592	H1N1	03/18/2010	United Ki	A/England/00380718/2010
NA	LN867678	H1N1	03/18/2010	United Ki	A/England/WTSI2067/2010
NA	LN850104	H1N1	03/18/2010	United Ki	A/England/WTSI1807/2010
NA	LN850339	H1N1	03/18/2010	United Ki	A/England/00380709/2010
NA	LN867399	H1N1	03/18/2010	United Ki	A/England/WTSI1783/2010
NA	CY167782	H1N1	03/19/2010	USA	A/Tennessee/F1080/2010
NA	JX309251	H1N1	03/19/2010	Singapore	A/Singapore/KK103/2010
NA	CY167446	H1N1	03/19/2010	USA	A/Tennessee/F1083c80/2010
NA	KC781241	H1N1	03/19/2010	USA	A/Georgia/11/2010
NA	JX309254	H1N1	03/19/2010	Singapore	A/Singapore/GP937/2010
NA	CY167430	H1N1	03/19/2010	USA	A/Tennessee/F1076A/2010
NA	KC781139	H1N1	03/19/2010	USA	A/Virginia/02/2010
NA	CY089373	H1N1	03/19/2010	Nicaragua	A/Managua/5708.01/2010(H1N1)
NA	CY080325	H1N1	03/20/2010	Thailand	A/Thailand/CU-H1786/2010(H1N1)
NA	KT181602	H1N1	03/20/2010	Singapore	A/Singapore/DMS89/2010
NA	CY096604	H1N1	03/21/2010	USA	A/Cambridge/INS528/2010(H1N1)
NA	JX556125	H1N1	03/22/2010	Cuba	A/Habana/9696/2010
NA	CY090138	H1N1	03/22/2010	Thailand	A/Thailand/CU-B2323/2010
NA	KC782127	H1N1	03/22/2010	USA	A/Minnesota/04/2010
NA	JF265672	H1N1	03/22/2010	India	A/India/Blore/2010
NA	KT181603	H1N1	03/23/2010	Singapore	A/Singapore/DMS90/2010
NA	CY090839	H1N1	03/23/2010	Nicaragua	A/Managua/0305_10/2010(H1N1)
NA	KM013721	H1N1	03/23/2010	Thailand	A/Bangkok/SIMI508/2010
NA	JX309263	H1N1	03/24/2010	Singapore	A/Singapore/GP965/2010
NA	JX556126	H1N1	03/24/2010	Cuba	A/Habana/9790/2010
NA	CY167454	H1N1	03/24/2010	USA	A/Tennessee/F1089/2010
NA	JF929798	H1N1	03/24/2010	China	A/Guangdong/378/2010
NA	KX651691	H1N1	03/25/2010	Spain	A/Spain/3438-1T/2010
NA	JX309266	H1N1	03/25/2010	Singapore	A/Singapore/GP982/2010
NA	KT181605	H1N1	03/25/2010	Singapore	A/Singapore/DMS91/2010
NA	KC261329	H1N1	03/25/2010	Mexico	A/Mexico City/INERPHAC4/2010(H1N1)
NA	CY089381	H1N1	03/25/2010	Nicaragua	A/Managua/3246.01/2010(H1N1)
NA	KC781423	H1N1	03/26/2010	USA	A/Delaware/03/2010
NA	LN867391	H1N1	03/26/2010	United Ki	A/England/01580005/2010
NA	JX309215	H1N1	03/26/2010	Singapore	A/Singapore/GP1003/2010
NA	KC781135	H1N1	03/27/2010	USA	A/West Virginia/01/2010
NA	KX651692	H1N1	03/27/2010	Spain	A/Spain/3438-3T/2010
NA	AB649905	H1N1	03/29/2010	Japan	A/Toyama/92/2010
NA	HM849000	H1N1	03/29/2010	Thailand	A/Thailand/CU-MV63/2010
NA	KT181607	H1N1	03/29/2010	Singapore	A/Singapore/DMS92/2010
NA	KC780913	H1N1	03/29/2010	USA	A/Oregon/01/2010
NA	CY080333	H1N1	03/29/2010	Thailand	A/Thailand/H1818/2010
NA	JX309218	H1N1	03/29/2010	Singapore	A/Singapore/GP1012/2010
NA	KC780259	H1N1	03/30/2010	USA	A/Hawaii/05/2010
NA	JX309225	H1N1	03/30/2010	Singapore	A/Singapore/GP1035/2010
NA	JX309278	H1N1	03/30/2010	Singapore	A/Singapore/TT128/2010

NA	KX651693	H1N1	03/30/2010	Spain	A/Spain/3438-5T/2010
NA	CY080341	H1N1	03/31/2010	Thailand	A/Thailand/CU-H1821/2010(H1N1)
NA	KC781120	H1N1	03/31/2010	USA	A/Maryland/05/2010
NA	JX309229	H1N1	03/31/2010	Singapore	A/Singapore/GP1040/2010
NA	JX309240	H1N1	03/31/2010	Singapore	A/Singapore/KK111/2010
NA	KC781225	H1N1	03/31/2010	USA	A/Michigan/03/2010

)

)

H1N1)

H1N1)
1N1)
H1N1)

H1N1)
1N1)

1)

1)

1）

1)

1)
1)

1)

1)

1)

1)

1)

1）

-1649/2009

2009

1)
1)
1)

)

N1)

1)

1N1)

1)

1N1)

N1)
N1)

N1)

1N1)

9

1N1)

1N1)

1N1)
1N1)

1N1)

1N1)
1N1)

1N1)

1N1)

1N1)

1N1)

1N1)

1N1)

H1N1)

1N1)

1N1)

1N1)

[illegible]H1N1)

H1N1)

1N1)

)

1)

1)

1）

H1N1)

H1N1)

H1N1)

H1N1)

N1)

N1)

)

)

1)

N1)

N1)
N1)

N1)

)

N1)

1)

)

)

H1N1)

1)

2010

2010

)
)

)

2010

2010

2010

H1N1)

Name	Sequence	Subtype	Collection Date	Country	Strain Name
PB2	GQ200260	H1N1	04/01/2009	USA	A/Nebraska/02/2009(H1N1)
PB2	FJ969516	H1N1	04/01/2009	USA	A/California/04/2009(H1N1)
PB2	FJ966079	H1N1	04/01/2009	USA	A/California/04/2009(H1N1)
PB2	GQ162180	H1N1	04/03/2009	Mexico	A/Mexico/4108/2009(H1N1)
PB2	GQ200202	H1N1	04/03/2009	Mexico	A/Mexico/4108/2009(H1N1)
PB2	GQ379815	H1N1	04/03/2009	Mexico	A/Mexico/4108/2009(H1N1)
PB2	GQ303347	H1N1	04/07/2009	Mexico	A/Mexico/4115/2009(H1N1)
PB2	FJ969530	H1N1	04/09/2009	USA	A/California/07/2009(H1N1)
PB2	CY181400	H1N1	04/09/2009	USA	A/California/07-JR002/2009
PB2	CY181432	H1N1	04/09/2009	USA	A/California/07-JR010/2009
PB2	KU933482	H1N1	04/09/2009	USA	A/California/07/2009
PB2	KC780061	H1N1	04/09/2009	USA	A/California/08/2009
PB2	CY266198	H1N1	04/09/2009	USA	A/California/07/2009
PB2	KC781783	H1N1	04/09/2009	USA	A/California/07/2009
PB2	GQ377051	H1N1	04/09/2009	USA	A/California/08/2009(H1N1)
PB2	CY181424	H1N1	04/09/2009	USA	A/California/07-JR009/2009
PB2	FJ984387	H1N1	04/09/2009	USA	A/California/07/2009(H1N1)
PB2	FJ984365	H1N1	04/09/2009	USA	A/California/08/2009(H1N1)
PB2	FJ966976	H1N1	04/09/2009	USA	A/California/07/2009(H1N1)
PB2	CY181408	H1N1	04/09/2009	USA	A/California/07-JR003/2009
PB2	CY181416	H1N1	04/09/2009	USA	A/California/07-JR008/2009
PB2	FJ969525	H1N1	04/14/2009	USA	A/Texas/04/2009(H1N1)
PB2	CY098505	H1N1	04/14/2009	Mexico	A/Mexico/4482/2009
PB2	GQ168885	H1N1	04/14/2009	USA	A/Texas/04/2009(H1N1)
PB2	CY044192	H1N1	04/14/2009	USA	A/San Antonio/PR921/2009(H1N1)
PB2	CY147778	H1N1	04/14/2009	Mexico	A/Mexico/24033/2009
PB2	FJ998206	H1N1	04/14/2009	Mexico	A/Mexico/InDRE4487/2009(H1N1)
PB2	GQ162177	H1N1	04/14/2009	Mexico	A/Mexico/4486/2009(H1N1)
PB2	GQ397115	H1N1	04/15/2009	Mexico	A/Mexico/4269/2009(H1N1)
PB2	GQ200256	H1N1	04/15/2009	USA	A/Texas/15/2009(H1N1)
PB2	GQ200240	H1N1	04/15/2009	USA	A/Texas/05/2009(H1N1)
PB2	KC781718	H1N1	04/16/2009	USA	A/California/06/2009
PB2	GQ402174	H1N1	04/16/2009	Canada	A/Canada-QC/RV1595/2009(H1N1)
PB2	FJ966963	H1N1	04/16/2009	USA	A/California/06/2009(H1N1)
PB2	CY147770	H1N1	04/17/2009	Mexico	A/Mexico/24032/2009
PB2	CY147826	H1N1	04/17/2009	Mexico	A/Mexico/24043/2009
PB2	GQ149632	H1N1	04/19/2009	Mexico	A/Mexico/4604/2009(H1N1)
PB2	GQ162198	H1N1	04/19/2009	Mexico	A/Mexico/4603/2009(H1N1)
PB2	GQ162168	H1N1	04/19/2009	Mexico	A/Mexico/4603/2009(H1N1)
PB2	GQ200201	H1N1	04/19/2009	Mexico	A/Mexico/4603/2009(H1N1)
PB2	CY147842	H1N1	04/20/2009	Mexico	A/Mexico/24045/2009
PB2	CY147834	H1N1	04/20/2009	Mexico	A/Mexico/24044/2009
PB2	GQ168870	H1N1	04/22/2009	USA	A/Indiana/09/2009(H1N1)
PB2	GQ200241	H1N1	04/22/2009	USA	A/Arizona/01/2009(H1N1)
PB2	CY147706	H1N1	04/23/2009	Mexico	A/Mexico/24021/2009
PB2	CY147618	H1N1	04/23/2009	Mexico	A/Mexico/24007/2009
PB2	GQ160545	H1N1	04/24/2009	USA	A/Ohio/07/2009(H1N1)
PB2	GQ117047	H1N1	04/24/2009	USA	A/Texas/08/2009(H1N1)
PB2	GQ168639	H1N1	04/24/2009	USA	A/Iowa/04/2009(H1N1)
PB2	GQ200254	H1N1	04/24/2009	USA	A/New York/23/2009(H1N1)
PB2	GQ200246	H1N1	04/24/2009	USA	A/New York/21/2009(H1N1)
PB2	GQ200232	H1N1	04/24/2009	USA	A/New York/31/2009(H1N1)
PB2	GQ200223	H1N1	04/24/2009	USA	A/Kansas/02/2009(H1N1)

PB2	FJ998205	H1N1	04/24/2009	Canada	A/Canada-ON/RV1527/2009(H1N1)
PB2	GQ120442	H1N1	04/24/2009	Canada	A/Canada-NS/RV1535/2009(H1N1)
PB2	CY065147	H1N1	04/24/2009	United Ki	A/Scotland/91800799/2009
PB2	GQ373259	H1N1	04/24/2009	Canada	A/Toronto/3141/2009(H1N1)
PB2	CY041556	H1N1	04/24/2009	USA	A/New York/3210/2009(H1N1)
PB2	GQ377099	H1N1	04/24/2009	USA	A/Ohio/07/2009(H1N1)
PB2	GQ377080	H1N1	04/24/2009	USA	A/Ohio/07/2009(H1N1)
PB2	GQ200224	H1N1	04/24/2009	USA	A/Kansas/03/2009(H1N1)
PB2	GQ117021	H1N1	04/24/2009	USA	A/New York/22/2009(H1N1)
PB2	GQ457503	H1N1	04/25/2009	USA	A/New York/19/2009(H1N1)
PB2	CY041137	H1N1	04/25/2009	USA	A/New York/3215/2009(H1N1)
PB2	CY041548	H1N1	04/25/2009	USA	A/New York/3209/2009(H1N1)
PB2	CY086984	H1N1	04/25/2009	USA	A/New York/3217/2009(H1N1)
PB2	CY097873	H1N1	04/25/2009	Mexico	A/Mexico/47N/2009
PB2	CY044160	H1N1	04/25/2009	Mexico	A/Mexico/48N/2009(H1N1)
PB2	CY147682	H1N1	04/25/2009	Mexico	A/Mexico/24015/2009
PB2	GQ168878	H1N1	04/25/2009	USA	A/New York/15/2009(H1N1)
PB2	GQ168876	H1N1	04/25/2009	USA	A/New York/10/2009(H1N1)
PB2	CY041620	H1N1	04/25/2009	USA	A/New York/3002/2009(H1N1)
PB2	CY041129	H1N1	04/25/2009	USA	A/New York/3214/2009(H1N1)
PB2	GQ377076	H1N1	04/25/2009	USA	A/New York/20/2009(H1N1)
PB2	GQ377089	H1N1	04/25/2009	USA	A/New York/13/2009(H1N1)
PB2	GQ200258	H1N1	04/25/2009	USA	A/New York/20/2009(H1N1)
PB2	FJ984351	H1N1	04/25/2009	USA	A/New York/18/2009(H1N1)
PB2	GQ200212	H1N1	04/25/2009	USA	A/New York/11/2009(H1N1)
PB2	GQ200210	H1N1	04/25/2009	USA	A/New York/06/2009(H1N1)
PB2	GQ117027	H1N1	04/25/2009	USA	A/Texas/09/2009(H1N1)
PB2	GQ117035	H1N1	04/25/2009	USA	A/California/14/2009(H1N1)
PB2	CY065139	H1N1	04/25/2009	United Ki	A/Scotland/91800797/2009
PB2	CY040885	H1N1	04/25/2009	Mexico	A/Mexico/47N/2009(H1N1)
PB2	GQ377097	H1N1	04/25/2009	USA	A/New York/09/2009(H1N1)
PB2	GQ396736	H1N1	04/26/2009	USA	A/New Mexico/04/2009(H1N1)
PB2	CY041057	H1N1	04/26/2009	USA	A/New York/3166/2009(H1N1)
PB2	CY041065	H1N1	04/26/2009	USA	A/New York/3168/2009(H1N1)
PB2	GQ377038	H1N1	04/26/2009	USA	A/Massachusetts/06/2009(H1N1)
PB2	CY039900	H1N1	04/26/2009	USA	A/New York/1669/2009(H1N1)
PB2	GQ200262	H1N1	04/26/2009	USA	A/Michigan/02/2009(H1N1)
PB2	GQ338388	H1N1	04/26/2009	USA	A/Texas/22/2009(H1N1)
PB2	CY041741	H1N1	04/26/2009	USA	A/New York/3206/2009(H1N1)
PB2	GQ465665	H1N1	04/26/2009	Canada	A/Canada-PQ/RV1586/2009(H1N1)
PB2	GQ200222	H1N1	04/26/2009	USA	A/South Carolina/09/2009(H1N1)
PB2	GQ117076	H1N1	04/26/2009	USA	A/Arizona/02/2009(H1N1)
PB2	CY041105	H1N1	04/26/2009	USA	A/New York/3176/2009(H1N1)
PB2	CY041177	H1N1	04/26/2009	USA	A/New York/3186/2009(H1N1)
PB2	CY065243	H1N1	04/27/2009	United Ki	A/England/XFL00071/2009
PB2	CY041089	H1N1	04/27/2009	USA	A/New York/3172/2009(H1N1)
PB2	CY040845	H1N1	04/27/2009	USA	A/New York/3262/2009(H1N1)
PB2	CY041521	H1N1	04/27/2009	USA	A/New York/3203/2009(H1N1)
PB2	GQ465666	H1N1	04/27/2009	Canada	A/Canada-ON/RV1589/2009(H1N1)
PB2	CY147858	H1N1	04/27/2009	Mexico	A/Mexico/24048/2009
PB2	CY147866	H1N1	04/27/2009	Mexico	A/Mexico/24049/2009
PB2	CY147890	H1N1	04/27/2009	Mexico	A/Mexico/24052/2009
PB2	CY065219	H1N1	04/27/2009	United Ki	A/England/207/2009
PB2	CY147714	H1N1	04/27/2009	Mexico	A/Mexico/24022/2009

PB2	CY147850	H1N1	04/27/2009	Mexico	A/Mexico/24047/2009
PB2	CY147786	H1N1	04/27/2009	Mexico	A/Mexico/24036/2009
PB2	CY147874	H1N1	04/27/2009	Mexico	A/Mexico/24050/2009
PB2	CY039908	H1N1	04/27/2009	USA	A/New York/1682/2009(H1N1)
PB2	GQ200263	H1N1	04/27/2009	USA	A/Colorado/03/2009(H1N1)
PB2	CY069770	H1N1	04/27/2009	United Ki	A/England/209/2009
PB2	CY040829	H1N1	04/27/2009	USA	A/New York/3260/2009(H1N1)
PB2	GQ402171	H1N1	04/27/2009	Mexico	A/Mexico/InDRE13547/2009(H1N1)
PB2	CY065187	H1N1	04/27/2009	United Ki	A/England/202/2009
PB2	CY065195	H1N1	04/27/2009	United Ki	A/England/197/2009
PB2	CY065235	H1N1	04/27/2009	United Ki	A/England/551/2009
PB2	CY147722	H1N1	04/27/2009	Mexico	A/Mexico/24024/2009
PB2	CY147882	H1N1	04/27/2009	Mexico	A/Mexico/24051/2009
PB2	CY147898	H1N1	04/27/2009	Mexico	A/Mexico/24053/2009
PB2	CY147906	H1N1	04/27/2009	Mexico	A/Mexico/24054/2009
PB2	CY065211	H1N1	04/27/2009	United Ki	A/England/205/2009
PB2	GQ160523	H1N1	04/27/2009	USA	A/Florida/04/2009(H1N1)
PB2	GQ200229	H1N1	04/27/2009	USA	A/Alabama/02/2009(H1N1)
PB2	GQ117070	H1N1	04/27/2009	USA	A/Minnesota/02/2009(H1N1)
PB2	CY041529	H1N1	04/27/2009	USA	A/New York/3204/2009(H1N1)
PB2	CY041837	H1N1	04/28/2009	USA	A/New York/3348/2009(H1N1)
PB2	CY041580	H1N1	04/28/2009	USA	A/New York/3227/2009(H1N1)
PB2	CY041588	H1N1	04/28/2009	USA	A/New York/3230/2009(H1N1)
PB2	GQ402172	H1N1	04/28/2009	Mexico	A/Mexico/InDRE13551/2009(H1N1)
PB2	CY047333	H1N1	04/28/2009	USA	A/New York/3167/2009(H1N1)
PB2	CY047325	H1N1	04/28/2009	USA	A/New York/3124/2009(H1N1)
PB2	CY041497	H1N1	04/28/2009	USA	A/New York/3184/2009(H1N1)
PB2	CY046338	H1N1	04/28/2009	USA	A/Wisconsin/629-D01505/2009(H1N
PB2	CY147914	H1N1	04/28/2009	Mexico	A/Mexico/24055/2009
PB2	CY147746	H1N1	04/28/2009	Mexico	A/Mexico/24027/2009
PB2	CY147794	H1N1	04/28/2009	Mexico	A/Mexico/24038/2009
PB2	CY147946	H1N1	04/28/2009	Mexico	A/Mexico/24060/2009
PB2	CY147738	H1N1	04/28/2009	Mexico	A/Mexico/24026/2009
PB2	CY147954	H1N1	04/28/2009	Mexico	A/Mexico/24061/2009
PB2	CY041596	H1N1	04/28/2009	USA	A/New York/3236/2009(H1N1)
PB2	GQ465661	H1N1	04/28/2009	Canada	A/Canada-AB/RV1531/2009(H1N1)
PB2	GQ396729	H1N1	04/28/2009	USA	A/Tennessee/05/2009(H1N1)
PB2	CY053078	H1N1	04/28/2009	USA	A/Houston/130S/2009(H1N1)
PB2	CY065203	H1N1	04/28/2009	United Ki	A/England/204/2009
PB2	CY065251	H1N1	04/28/2009	United Ki	A/England/211/2009
PB2	GQ166656	H1N1	04/28/2009	United Ki	A/England/195/2009
PB2	CY065227	H1N1	04/28/2009	United Ki	A/England/200/2009
PB2	CY147754	H1N1	04/28/2009	Mexico	A/Mexico/24028/2009
PB2	CY147922	H1N1	04/28/2009	Mexico	A/Mexico/24056/2009
PB2	CY147930	H1N1	04/28/2009	Mexico	A/Mexico/24057/2009
PB2	CY147938	H1N1	04/28/2009	Mexico	A/Mexico/24058/2009
PB2	CY041097	H1N1	04/28/2009	USA	A/New York/3173/2009(H1N1)
PB2	CY041121	H1N1	04/28/2009	USA	A/New York/3193/2009(H1N1)
PB2	CY040708	H1N1	04/28/2009	USA	A/New York/3235/2009(H1N1)
PB2	GQ232045	H1N1	04/28/2009	USA	A/Rhode Island/02/2009(H1N1)
PB2	CY041193	H1N1	04/28/2009	USA	A/New York/3188/2009(H1N1)
PB2	CY041209	H1N1	04/28/2009	USA	A/New York/3199/2009(H1N1)
PB2	CY041513	H1N1	04/28/2009	USA	A/New York/3196/2009(H1N1)
PB2	CY041505	H1N1	04/28/2009	USA	A/New York/3195/2009(H1N1)

PB2	CY041153	H1N1	04/28/2009	USA	A/New York/3181/2009(H1N1)
PB2	CY041564	H1N1	04/28/2009	USA	A/New York/3211/2009(H1N1)
PB2	GQ402170	H1N1	04/29/2009	Mexico	A/Mexico/InDRE13495/2009(H1N1)
PB2	GQ214155	H1N1	04/29/2009	France	A/Paris/2573/2009(H1N1)
PB2	GQ465663	H1N1	04/29/2009	Canada	A/Canada-NS/RV1554/2009(H1N1)
PB2	CY046940	H1N1	04/29/2009	Netherlar	A/Netherlands/602/2009
PB2	CY147810	H1N1	04/29/2009	Mexico	A/Mexico/24040/2009
PB2	CY147802	H1N1	04/29/2009	Mexico	A/Mexico/24039/2009
PB2	GQ160563	H1N1	04/29/2009	USA	A/Maryland/05/2009(H1N1)
PB2	CY041733	H1N1	04/29/2009	USA	A/New York/3247/2009(H1N1)
PB2	GQ465662	H1N1	04/29/2009	Canada	A/Canada-NS/RV1551/2009(H1N1)
PB2	GQ402173	H1N1	04/29/2009	Mexico	A/Mexico/InDRE13555/2009(H1N1)
PB2	CY047317	H1N1	04/29/2009	USA	A/New York/3074/2009(H1N1)
PB2	CY086976	H1N1	04/29/2009	USA	A/New York/3198/2009(H1N1)
PB2	CY065339	H1N1	04/29/2009	United Ki	A/England/223/2009
PB2	CY065259	H1N1	04/29/2009	United Ki	A/England/XFL00137/2009
PB2	CY147762	H1N1	04/29/2009	Mexico	A/Mexico/24030/2009
PB2	CY040869	H1N1	04/29/2009	USA	A/New York/3265/2009(H1N1)
PB2	GQ402169	H1N1	04/29/2009	Mexico	A/Mexico/InDRE13494/2009(H1N1)
PB2	CY041628	H1N1	04/30/2009	USA	A/New York/3100/2009(H1N1)
PB2	CY040700	H1N1	04/30/2009	USA	A/New York/3232/2009(H1N1)
PB2	CY046458	H1N1	04/30/2009	USA	A/Wisconsin/629-D01558/2009(H1N
PB2	CY046850	H1N1	04/30/2009	USA	A/Wisconsin/629-D02361/2009(H1N
PB2	GQ214143	H1N1	04/30/2009	France	A/Paris/2590/2009(H1N1)
PB2	CY065275	H1N1	04/30/2009	United Ki	A/England/215/2009
PB2	CY046266	H1N1	04/30/2009	USA	A/Wisconsin/629-D01773/2009(H1N
PB2	CY046522	H1N1	04/30/2009	USA	A/Wisconsin/629-D02141/2009(H1N
PB2	CY065283	H1N1	04/30/2009	United Ki	A/England/216/2009
PB2	CY065267	H1N1	04/30/2009	United Ki	A/England/214/2009
PB2	GU931811	H1N1	04/30/2009	Hong Kong	A/Hong Kong/415742/2009(H1N1)
PB2	CY064747	H1N1	04/30/2009	Mexico	A/Mexico City/024/2009(H1N1)
PB2	GQ465669	H1N1	04/30/2009	Canada	A/Canada-SK/RV1788/2009(H1N1)
PB2	GQ465664	H1N1	04/30/2009	Canada	A/Canada-NS/RV1565/2009(H1N1)
PB2	CY047357	H1N1	04/30/2009	USA	A/New York/3365/2009(H1N1)
PB2	CY046362	H1N1	04/30/2009	USA	A/Wisconsin/629-D01529/2009(H1N
PB2	CY046394	H1N1	04/30/2009	USA	A/Wisconsin/629-D00750/2009(H1N
PB2	CY046418	H1N1	04/30/2009	USA	A/Wisconsin/629-D00447/2009(H1N
PB2	CY046482	H1N1	04/30/2009	USA	A/Wisconsin/629-D00015/2009(H1N
PB2	CY064707	H1N1	04/30/2009	Mexico	A/Mexico City/019/2009
PB2	CY064715	H1N1	04/30/2009	Mexico	A/Mexico City/020/2009
PB2	GQ214149	H1N1	04/30/2009	France	A/Paris/2580/2009(H1N1)
PB2	CY040652	H1N1	04/30/2009	USA	A/New York/3194/2009(H1N1)
PB2	CY046426	H1N1	04/30/2009	USA	A/Wisconsin/629-D00767/2009(H1N
PB2	CY040039	H1N1	05/2009	Thailand	A/Nonthaburi/102/2009
PB2	GQ254713	H1N1	05/01/2009	France	A/Paris/2591/2009(H1N1)
PB2	CY064755	H1N1	05/01/2009	Mexico	A/Mexico City/025/2009(H1N1)
PB2	CY041644	H1N1	05/01/2009	USA	A/New York/3307/2009(H1N1)
PB2	GQ465667	H1N1	05/01/2009	Canada	A/Canada-AB/RV1644/2009(H1N1)
PB2	CY053110	H1N1	05/01/2009	USA	A/Houston/15H/2009(H1N1)
PB2	CY047349	H1N1	05/01/2009	USA	A/New York/3345/2009(H1N1)
PB2	CY046978	H1N1	05/01/2009	USA	A/New York/3443/2009(H1N1)
PB2	CY046498	H1N1	05/01/2009	USA	A/Wisconsin/629-D02028/2009(H1N
PB2	CY046194	H1N1	05/01/2009	USA	A/Wisconsin/629-D01793/2009(H1N
PB2	CY046434	H1N1	05/01/2009	USA	A/Wisconsin/629-D00677/2009(H1N

PB2	CY046466	H1N1	05/01/2009	USA	A/Wisconsin/629-D01861/2009(H1N
PB2	CY064723	H1N1	05/01/2009	Mexico	A/Mexico City/021/2009
PB2	CY046322	H1N1	05/01/2009	USA	A/Wisconsin/629-D01308/2009(H1N
PB2	CY053094	H1N1	05/01/2009	USA	A/Houston/14H/2009(H1N1)
PB2	GQ214160	H1N1	05/01/2009	France	A/Paris/2592/2009(H1N1)
PB2	CY053102	H1N1	05/01/2009	USA	A/Houston/15OS/2009(H1N1)
PB2	CY053086	H1N1	05/01/2009	USA	A/Houston/14OS/2009H1N1)
PB2	CY064739	H1N1	05/01/2009	Mexico	A/Mexico City/023/2009(H1N1)
PB2	CY069778	H1N1	05/02/2009	United Ki	A/England/221/2009
PB2	CY064731	H1N1	05/02/2009	Mexico	A/Mexico City/022/2009
PB2	CY065331	H1N1	05/02/2009	United Ki	A/England/201/2009
PB2	CY065299	H1N1	05/02/2009	United Ki	A/England/218/2009
PB2	CY049872	H1N1	05/02/2009	USA	A/Cherry Point/WR0102/2009(H1N1)
PB2	GQ160811	H1N1	05/02/2009	South Ko	A/Korea/01/2009
PB2	HM567565	H1N1	05/02/2009	United Ki	A/England/221/2009
PB2	CY065323	H1N1	05/02/2009	United Ki	A/England/220/2009
PB2	CY065315	H1N1	05/02/2009	United Ki	A/England/219/2009
PB2	CY046858	H1N1	05/02/2009	USA	A/Wisconsin/629-D00244/2009(H1N
PB2	CY046442	H1N1	05/02/2009	USA	A/Wisconsin/629-D02262/2009(H1N
PB2	CY049856	H1N1	05/02/2009	USA	A/Cherry Point/WR0100/2009(H1N1)
PB2	CY046242	H1N1	05/02/2009	USA	A/Wisconsin/629-D01735/2009(H1N
PB2	CY046402	H1N1	05/02/2009	USA	A/Wisconsin/629-D01189/2009(H1N
PB2	CY046210	H1N1	05/02/2009	USA	A/Wisconsin/629-D02408/2009(H1N
PB2	CY046386	H1N1	05/02/2009	USA	A/Wisconsin/629-D01980/2009(H1N
PB2	CY046202	H1N1	05/02/2009	USA	A/Wisconsin/629-D02312/2009(H1N
PB2	CY046274	H1N1	05/02/2009	USA	A/Wisconsin/629-D00933/2009(H1N
PB2	CY046346	H1N1	05/02/2009	USA	A/Wisconsin/629-D02455/2009(H1N
PB2	CY065307	H1N1	05/02/2009	United Ki	A/England/246/2009
PB2	CY041081	H1N1	05/02/2009	USA	A/New York/3170/2009(H1N1)
PB2	CY049864	H1N1	05/02/2009	USA	A/Cherry Point/WR0101/2009(H1N1)
PB2	CY064763	H1N1	05/02/2009	Mexico	A/Mexico City/026/2009(H1N1)
PB2	CY041813	H1N1	05/02/2009	USA	A/New York/3408/2009(H1N1)
PB2	CY041604	H1N1	05/02/2009	USA	A/New York/3177/2009(H1N1)
PB2	GQ323502	H1N1	05/02/2009	USA	A/Michigan/06/2009(H1N1)
PB2	GQ251032	H1N1	05/03/2009	Italy	A/Italy/05/2009(H1N1)
PB2	CY065291	H1N1	05/03/2009	United Ki	A/Scotland/4/2009
PB2	CY065347	H1N1	05/03/2009	United Ki	A/England/247/2009
PB2	CY046218	H1N1	05/03/2009	USA	A/Wisconsin/629-D00869/2009(H1N
PB2	CY046234	H1N1	05/03/2009	USA	A/Wisconsin/629-D01296/2009(H1N
PB2	CY069786	H1N1	05/03/2009	United Ki	A/England/222/2009
PB2	CY046282	H1N1	05/03/2009	USA	A/Wisconsin/629-D01092/2009(H1N
PB2	CY046250	H1N1	05/03/2009	USA	A/Wisconsin/629-D01606/2009(H1N
PB2	CY046378	H1N1	05/03/2009	USA	A/Wisconsin/629-D01705/2009(H1N
PB2	CY046290	H1N1	05/03/2009	USA	A/Wisconsin/629-D02473/2009(H1N
PB2	CY046354	H1N1	05/03/2009	USA	A/Wisconsin/629-D01017/2009(H1N
PB2	CY046890	H1N1	05/03/2009	USA	A/Wisconsin/629-D00498/2009(H1N
PB2	CY043210	H1N1	05/04/2009	USA	A/New York/3338/2009(H1N1)
PB2	CY069818	H1N1	05/04/2009	United Ki	A/England/250/2009
PB2	CY041652	H1N1	05/04/2009	USA	A/New York/3313/2009(H1N1)
PB2	CY069802	H1N1	05/04/2009	United Ki	A/England/XFL00230/2009
PB2	CY046474	H1N1	05/04/2009	USA	A/Wisconsin/629-D01962/2009(H1N
PB2	CY065355	H1N1	05/04/2009	United Ki	A/England/XFL00200/2009
PB2	CY046514	H1N1	05/04/2009	USA	A/Wisconsin/629-D02425/2009(H1N
PB2	CY046370	H1N1	05/04/2009	USA	A/Wisconsin/629-D00790/2009(H1N

PB2	CY046410	H1N1	05/04/2009	USA	A/Wisconsin/629-D00807/2009(H1N
PB2	CY046898	H1N1	05/04/2009	USA	A/Wisconsin/629-D01880/2009(H1N
PB2	CY046922	H1N1	05/04/2009	USA	A/Wisconsin/629-D01764/2009(H1N
PB2	CY046330	H1N1	05/04/2009	USA	A/Wisconsin/629-D01810/2009(H1N
PB2	CY046226	H1N1	05/04/2009	USA	A/Wisconsin/629-D01251/2009(H1N
PB2	CY045479	H1N1	05/04/2009	Germany	A/Sachsen-Anhalt/101/2009(H1N1)
PB2	CY046530	H1N1	05/04/2009	USA	A/Wisconsin/629-D00183/2009(H1N
PB2	CY046258	H1N1	05/04/2009	USA	A/Wisconsin/629-D02432/2009(H1N
PB2	CY046490	H1N1	05/04/2009	USA	A/Wisconsin/629-D00349/2009(H1N
PB2	CY046450	H1N1	05/04/2009	USA	A/Wisconsin/629-D01226/2009(H1N
PB2	CY046874	H1N1	05/04/2009	USA	A/Wisconsin/629-D02272/2009(H1N
PB2	CY046866	H1N1	05/04/2009	USA	A/Wisconsin/629-D01415/2009(H1N
PB2	CY046882	H1N1	05/04/2009	USA	A/Wisconsin/629-D00396/2009(H1N
PB2	CY046538	H1N1	05/04/2009	USA	A/Wisconsin/629-D00714/2009(H1N
PB2	CY050149	H1N1	05/04/2009	USA	A/Wisconsin/629-D01058/2009(H1N
PB2	CY053149	H1N1	05/04/2009	USA	A/Houston/220S/2009(H1N1)
PB2	CY053157	H1N1	05/04/2009	USA	A/Houston/22H/2009(H1N1)
PB2	CY041789	H1N1	05/04/2009	USA	A/New York/3337/2009(H1N1)
PB2	GQ402176	H1N1	05/04/2009	Canada	A/Canada-SK/RV1767/2009(H1N1)
PB2	CY046506	H1N1	05/04/2009	USA	A/Wisconsin/629-D00378/2009(H1N
PB2	CY043178	H1N1	05/04/2009	USA	A/New York/3460/2009(H1N1)
PB2	CY069826	H1N1	05/05/2009	United Ki	A/England/XFL00303/2009
PB2	CY065411	H1N1	05/05/2009	United Ki	A/England/254/2009
PB2	CY049817	H1N1	05/05/2009	USA	A/Craven/WR0019/2009(H1N1)
PB2	CY065379	H1N1	05/05/2009	United Ki	A/England/XFL00304/2009
PB2	CY065363	H1N1	05/05/2009	United Ki	A/England/XFL00275/2009
PB2	HM567573	H1N1	05/05/2009	United Ki	A/England/249/2009
PB2	CY069794	H1N1	05/05/2009	United Ki	A/England/XFL00228/2009
PB2	CY069810	H1N1	05/05/2009	United Ki	A/England/249/2009
PB2	CY041829	H1N1	05/06/2009	USA	A/New York/3352/2009(H1N1)
PB2	GQ205443	H1N1	05/06/2009	Thailand	A/Thailand/104/2009
PB2	CY047341	H1N1	05/06/2009	USA	A/New York/3364/2009(H1N1)
PB2	CY046562	H1N1	05/06/2009	USA	A/Wisconsin/629-D00228/2009(H1N
PB2	CY041757	H1N1	05/06/2009	USA	A/New York/3323/2009(H1N1)
PB2	CY041821	H1N1	05/06/2009	USA	A/New York/3351/2009(H1N1)
PB2	CY043202	H1N1	05/06/2009	USA	A/New York/3324/2009(H1N1)
PB2	GQ402178	H1N1	05/06/2009	Canada	A/Canada-SK/RV1798/2009(H1N1)
PB2	GQ465670	H1N1	05/07/2009	Canada	A/Canada-SK/RV1794/2009(H1N1)
PB2	CY100445	H1N1	05/07/2009	Mexico	A/Mexico City/IBT22/2009
PB2	CY065395	H1N1	05/07/2009	United Ki	A/England/XFL00311/2009
PB2	CY046610	H1N1	05/07/2009	USA	A/Wisconsin/629-D00487/2009(H1N
PB2	CY065403	H1N1	05/07/2009	United Ki	A/England/253/2009
PB2	CY050205	H1N1	05/07/2009	Mexico	A/Mexico City/001/2009
PB2	GQ402175	H1N1	05/07/2009	Canada	A/Canada-QC/RV1759/2009(H1N1)
PB2	CY065371	H1N1	05/07/2009	United Ki	A/England/XFL00300/2009
PB2	GQ465671	H1N1	05/07/2009	Canada	A/Canada-SK/RV1797/2009(H1N1)
PB2	GQ402177	H1N1	05/08/2009	Canada	A/Canada-SK/RV1793/2009(H1N1)
PB2	CY065427	H1N1	05/08/2009	United Ki	A/England/256/2009
PB2	CY065387	H1N1	05/08/2009	United Ki	A/England/252/2009
PB2	GQ169307	H1N1	05/08/2009	Japan	A/Narita/1/2009
PB2	CY069834	H1N1	05/08/2009	United Ki	A/England/XFL00364/2009
PB2	GQ465668	H1N1	05/08/2009	Canada	A/Canada-PQ/RV1758/2009(H1N1)
PB2	CY043130	H1N1	05/08/2009	USA	A/New York/3389/2009(H1N1)
PB2	CY050221	H1N1	05/08/2009	Mexico	A/Mexico City/003/2009(H1N1)

PB2	CY041773	H1N1	05/08/2009	USA	A/New York/3354/2009(H1N1)
PB2	CY046594	H1N1	05/08/2009	USA	A/Wisconsin/629-D02452/2009(H1N
PB2	CY046586	H1N1	05/08/2009	USA	A/Wisconsin/629-D01909/2009(H1N
PB2	CY046546	H1N1	05/08/2009	USA	A/Wisconsin/629-D01313/2009(H1N
PB2	CY050229	H1N1	05/08/2009	Mexico	A/Mexico City/004/2009(H1N1)
PB2	CY050213	H1N1	05/08/2009	Mexico	A/Mexico City/002/2009(H1N1)
PB2	CY050886	H1N1	05/08/2009	Mexico	A/Mexico City/005/2009(H1N1)
PB2	CY046578	H1N1	05/08/2009	USA	A/Wisconsin/629-D00905/2009(H1N
PB2	CY050253	H1N1	05/09/2009	Mexico	A/Mexico City/011/2009
PB2	CY050245	H1N1	05/09/2009	Mexico	A/Mexico City/010/2009
PB2	CY046618	H1N1	05/09/2009	USA	A/Wisconsin/629-D01055/2009(H1N
PB2	CY050870	H1N1	05/09/2009	Mexico	A/Mexico City/013/2009
PB2	CY069842	H1N1	05/09/2009	United Ki	A/England/XFL00376/2009
PB2	CY050878	H1N1	05/09/2009	Mexico	A/Mexico City/017/2009
PB2	CY050854	H1N1	05/09/2009	Mexico	A/Mexico City/007/2009(H1N1)
PB2	CY050237	H1N1	05/09/2009	Mexico	A/Mexico City/006/2009(H1N1)
PB2	CY050261	H1N1	05/09/2009	Mexico	A/Mexico City/012/2009
PB2	CY065435	H1N1	05/09/2009	United Ki	A/England/257/2009
PB2	CY062642	H1N1	05/09/2009	Mexico	A/Mexico City/014/2009
PB2	CY045943	H1N1	05/09/2009	Canada	A/Toronto/C2781/2009(H1N1)
PB2	CY046570	H1N1	05/09/2009	USA	A/Wisconsin/629-D01919/2009(H1N
PB2	CY050181	H1N1	05/09/2009	Mexico	A/Mexico City/015/2009
PB2	GQ200292	H1N1	05/10/2009	China	A/Shandong/1/2009(H1N1)
PB2	GQ166228	H1N1	05/10/2009	China	A/Sichuan/1/2009(H1N1)
PB2	JQ173153	H1N1	05/10/2009	Finland	A/Helsinki/P15/2009
PB2	CY065419	H1N1	05/10/2009	United Ki	A/England/XFL00354/2009
PB2	CY046602	H1N1	05/10/2009	USA	A/Wisconsin/629-D01482/2009(H1N
PB2	CY050173	H1N1	05/10/2009	Mexico	A/Mexico City/008/2009(H1N1)
PB2	CY050451	H1N1	05/10/2009	USA	A/Wisconsin/629-D01482/2009(H1N
PB2	CY046554	H1N1	05/10/2009	USA	A/Wisconsin/629-D00179/2009(H1N
PB2	CY050894	H1N1	05/10/2009	Mexico	A/Mexico City/018/2009
PB2	CY065443	H1N1	05/11/2009	United Ki	A/England/259/2009
PB2	CY043170	H1N1	05/11/2009	USA	A/New York/3454/2009(H1N1)
PB2	CY065451	H1N1	05/11/2009	United Ki	A/England/XFL00418/2009
PB2	GQ254716	H1N1	05/11/2009	France	A/Paris/2629/2009(H1N1)
PB2	CY041765	H1N1	05/11/2009	USA	A/New York/3413/2009(H1N1)
PB2	CY069850	H1N1	05/11/2009	United Ki	A/England/260/2009
PB2	CY043226	H1N1	05/11/2009	USA	A/New York/3455/2009(H1N1)
PB2	CY065467	H1N1	05/12/2009	United Ki	A/England/XFL00473/2009
PB2	CY043162	H1N1	05/12/2009	USA	A/New York/3418/2009(H1N1)
PB2	CY065491	H1N1	05/12/2009	United Ki	A/England/265/2009
PB2	CY065483	H1N1	05/12/2009	United Ki	A/England/264/2009
PB2	CY050862	H1N1	05/12/2009	Mexico	A/Mexico City/009/2009(H1N1)
PB2	CY069858	H1N1	05/12/2009	United Ki	A/England/XFL00421/2009
PB2	CY065475	H1N1	05/12/2009	United Ki	A/England/263/2009
PB2	CY050335	H1N1	05/12/2009	USA	A/Wisconsin/629-D01851/2009(H1N
PB2	CY065459	H1N1	05/12/2009	United Ki	A/England/XFL00419/2009
PB2	CY043186	H1N1	05/12/2009	USA	A/New York/3488/2009(H1N1)
PB2	CY043306	H1N1	05/12/2009	USA	A/New York/3498/2009(H1N1)
PB2	KC781316	H1N1	05/13/2009	USA	A/Mississippi/01/2009
PB2	CY050379	H1N1	05/13/2009	USA	A/Wisconsin/629-D01154/2009(H1N
PB2	CY043218	H1N1	05/13/2009	USA	A/New York/3420/2009(H1N1)
PB2	CY046682	H1N1	05/13/2009	USA	A/Wisconsin/629-D01305/2009(H1N
PB2	CY065507	H1N1	05/13/2009	United Ki	A/England/XFL00482/2009

PB2	CY051910	H1N1	05/13/2009	USA	A/Houston/10S/2009(H1N1)
PB2	CY052966	H1N1	05/13/2009	USA	A/Houston/1H/2009(H1N1)
PB2	CY053062	H1N1	05/13/2009	USA	A/Houston/100S/2009(H1N1)
PB2	CY053070	H1N1	05/13/2009	USA	A/Houston/10H/2009(H1N1)
PB2	CY043290	H1N1	05/13/2009	USA	A/New York/3449/2009(H1N1)
PB2	CY065499	H1N1	05/13/2009	United Ki	A/England/XFL00481/2009
PB2	CY046658	H1N1	05/14/2009	USA	A/Wisconsin/629-D01837/2009(H1N
PB2	CY041797	H1N1	05/14/2009	USA	A/New York/3463/2009(H1N1)
PB2	CY046690	H1N1	05/14/2009	USA	A/Wisconsin/629-D00978/2009(H1N
PB2	CY046674	H1N1	05/14/2009	USA	A/Wisconsin/629-D00064/2009(H1N
PB2	CY043138	H1N1	05/14/2009	USA	A/New York/3514/2009(H1N1)
PB2	CY046666	H1N1	05/14/2009	USA	A/Wisconsin/629-D00997/2009(H1N
PB2	CY065523	H1N1	05/15/2009	United Ki	A/England/XFL00532/2009
PB2	GQ225370	H1N1	05/15/2009	China	A/Beijing/01/2009(H1N1)
PB2	CY083293	H1N1	05/15/2009	USA	A/San Diego/WRAIR1647P/2009
PB2	CY044980	H1N1	05/15/2009	USA	A/New York/3744/2009(H1N1)
PB2	CY046634	H1N1	05/15/2009	USA	A/Wisconsin/629-D01839/2009(H1N
PB2	CY050395	H1N1	05/15/2009	USA	A/Wisconsin/629-D01839/2009(H1N
PB2	CY050387	H1N1	05/15/2009	USA	A/Wisconsin/629-D01295/2009(H1N
PB2	CY065515	H1N1	05/15/2009	United Ki	A/England/266/2009
PB2	CY048922	H1N1	05/15/2009	Malaysia	A/Malaysia/820/2009(H1N1)
PB2	CY041781	H1N1	05/16/2009	USA	A/New York/3532/2009(H1N1)
PB2	CY043194	H1N1	05/16/2009	USA	A/New York/3499/2009(H1N1)
PB2	GQ222057	H1N1	05/16/2009	Japan	A/Osaka-C/1/2009(H1N1)
PB2	CY062537	H1N1	05/16/2009	Mexico	A/Mexico city/CIA9/2009(H1N1)
PB2	GQ183632	H1N1	05/16/2009	China	A/Beijing/02/2009(H1N1)
PB2	GQ222056	H1N1	05/16/2009	Japan	A/Osaka/2/2009(H1N1)
PB2	GQ183624	H1N1	05/16/2009	China	A/Beijing/01/2009(H1N1)
PB2	CY046650	H1N1	05/16/2009	USA	A/Wisconsin/629-D02227/2009(H1N
PB2	CY046698	H1N1	05/16/2009	USA	A/Wisconsin/629-D00434/2009(H1N
PB2	CY043242	H1N1	05/16/2009	USA	A/New York/3502/2009(H1N1)
PB2	CY044988	H1N1	05/16/2009	USA	A/New York/3747/2009(H1N1)
PB2	CY048930	H1N1	05/16/2009	Malaysia	A/Malaysia/854/2009(H1N1)
PB2	CY043298	H1N1	05/16/2009	USA	A/New York/3469/2009(H1N1)
PB2	CY046642	H1N1	05/16/2009	USA	A/Wisconsin/629-D00967/2009(H1N
PB2	CY043250	H1N1	05/16/2009	USA	A/New York/3509/2009(H1N1)
PB2	CY043258	H1N1	05/16/2009	USA	A/New York/3545/2009(H1N1)
PB2	GQ222055	H1N1	05/16/2009	Japan	A/Osaka/1/2009(H1N1)
PB2	GQ222052	H1N1	05/17/2009	Japan	A/Hyogo/1/2009(H1N1)
PB2	CY043234	H1N1	05/17/2009	USA	A/New York/3501/2009(H1N1)
PB2	CY083285	H1N1	05/17/2009	USA	A/San Diego/WRAIR1646P/2009
PB2	GQ402179	H1N1	05/17/2009	Canada	A/Canada-QC/RV1954/2009(H1N1)
PB2	GQ221691	H1N1	05/17/2009	China	A/Guangdong/1/2009(H1N1)
PB2	GQ222058	H1N1	05/17/2009	Japan	A/Osaka-C/2/2009(H1N1)
PB2	CY069866	H1N1	05/17/2009	United Ki	A/England/271/2009
PB2	GQ222053	H1N1	05/17/2009	Japan	A/Hyogo/2/2009(H1N1)
PB2	CY050403	H1N1	05/17/2009	USA	A/Wisconsin/629-D00989/2009(H1N
PB2	CY044135	H1N1	05/17/2009	USA	A/New York/3516/2009(H1N1)
PB2	CY043314	H1N1	05/17/2009	USA	A/New York/3534/2009(H1N1)
PB2	CY045092	H1N1	05/17/2009	USA	A/New York/3574/2009(H1N1)
PB2	CY041805	H1N1	05/17/2009	USA	A/New York/3468/2009(H1N1)
PB2	GQ267838	H1N1	05/18/2009	Japan	A/Himeji/1/2009(H1N1)
PB2	GQ223441	H1N1	05/18/2009	China	A/Guangdong/1/2009(H1N1)
PB2	CY051918	H1N1	05/18/2009	USA	A/Houston/2H/2009(H1N1)

PB2	CY052990	H1N1	05/18/2009	USA	A/Houston/30S/2009(H1N1)
PB2	CY044916	H1N1	05/18/2009	USA	A/New York/3612/2009(H1N1)
PB2	CY052998	H1N1	05/18/2009	USA	A/Houston/3H/2009(H1N1)
PB2	CY044095	H1N1	05/18/2009	USA	A/New York/3740/2009(H1N1)
PB2	CY052974	H1N1	05/18/2009	USA	A/Houston/20S/2009(H1N1)
PB2	HQ011411	H1N1	05/18/2009	China	A/Guangdong/01/2009(H1N1)
PB2	CY046746	H1N1	05/19/2009	USA	A/Wisconsin/629-D00084/2009(H1N
PB2	CY044924	H1N1	05/19/2009	USA	A/New York/3613/2009(H1N1)
PB2	CY044143	H1N1	05/19/2009	USA	A/New York/3528/2009(H1N1)
PB2	CY050419	H1N1	05/19/2009	USA	A/Wisconsin/629-D00752/2009(H1N
PB2	CY046778	H1N1	05/19/2009	USA	A/Wisconsin/629-D00722/2009(H1N
PB2	CY043146	H1N1	05/19/2009	USA	A/New York/3529/2009(H1N1)
PB2	CY053014	H1N1	05/19/2009	USA	A/Houston/5H/2009(H1N1)
PB2	CY053006	H1N1	05/19/2009	USA	A/Houston/50S/2009(H1N1)
PB2	GQ293356	H1N1	05/19/2009	Japan	A/Shiga/2/2009(H1N1)
PB2	CY047365	H1N1	05/19/2009	USA	A/New York/3741/2009(H1N1)
PB2	CY044071	H1N1	05/19/2009	USA	A/New York/3568/2009(H1N1)
PB2	CY046762	H1N1	05/19/2009	USA	A/Wisconsin/629-D00160/2009(H1N
PB2	CY046722	H1N1	05/19/2009	USA	A/Wisconsin/629-D00752/2009(H1N
PB2	CY043274	H1N1	05/19/2009	USA	A/New York/3563/2009(H1N1)
PB2	CY044892	H1N1	05/19/2009	USA	A/New York/3580/2009(H1N1)
PB2	CY045150	H1N1	05/19/2009	USA	A/New York/3577/2009(H1N1)
PB2	CY044876	H1N1	05/20/2009	USA	A/New York/3571/2009(H1N1)
PB2	CY046738	H1N1	05/20/2009	USA	A/Wisconsin/629-D01521/2009(H1N
PB2	CY044900	H1N1	05/20/2009	USA	A/New York/3582/2009(H1N1)
PB2	CY044940	H1N1	05/20/2009	USA	A/New York/3623/2009(H1N1)
PB2	CY046730	H1N1	05/20/2009	USA	A/Wisconsin/629-D02427/2009(H1N
PB2	CY044063	H1N1	05/20/2009	USA	A/New York/3567/2009(H1N1)
PB2	GQ225378	H1N1	05/20/2009	China	A/Beijing/3/2009(H1N1)
PB2	CY053181	H1N1	05/20/2009	USA	A/Brownsville/260S/2009(H1N1)
PB2	CY053189	H1N1	05/20/2009	USA	A/Brownsville/270S/2009(H1N1)
PB2	GQ402180	H1N1	05/20/2009	Canada	A/Canada-MB/RV1964/2009(H1N1)
PB2	CY053022	H1N1	05/20/2009	USA	A/Houston/60S/2009(H1N1)
PB2	CY053030	H1N1	05/20/2009	USA	A/Houston/6H/2009(H1N1)
PB2	CY044055	H1N1	05/20/2009	USA	A/New York/3566/2009(H1N1)
PB2	CY043266	H1N1	05/20/2009	USA	A/New York/3552/2009(H1N1)
PB2	CY043154	H1N1	05/20/2009	USA	A/New York/3551/2009(H1N1)
PB2	CY046714	H1N1	05/20/2009	USA	A/Wisconsin/629-D01787/2009(H1N
PB2	CY044884	H1N1	05/21/2009	USA	A/New York/3573/2009(H1N1)
PB2	CY044908	H1N1	05/21/2009	USA	A/New York/3611/2009(H1N1)
PB2	CY044972	H1N1	05/21/2009	USA	A/New York/3653/2009(H1N1)
PB2	CY051926	H1N1	05/21/2009	USA	A/Houston/90S/2009(H1N1)
PB2	CY053197	H1N1	05/21/2009	USA	A/Brownsville/290S/2009(H1N1)
PB2	GQ267845	H1N1	05/21/2009	Japan	A/Sakai/1/2009(H1N1)
PB2	GQ402183	H1N1	05/21/2009	Canada	A/Canada-MB/RV1982/2009(H1N1)
PB2	CY052982	H1N1	05/21/2009	USA	A/Houston/20S/2009(H1N1)
PB2	CY053038	H1N1	05/21/2009	USA	A/Houston/70S/2009(H1N1)
PB2	CY053205	H1N1	05/21/2009	USA	A/Brownsville/300S/2009(H1N1)
PB2	CY053046	H1N1	05/21/2009	USA	A/Houston/7H/2009(H1N1)
PB2	CY046970	H1N1	05/21/2009	USA	A/New York/3576/2009(H1N1)
PB2	GQ219589	H1N1	05/21/2009	Russia	A/Moscow/IIV01/2009(H1N1)
PB2	GQ246613	H1N1	05/21/2009	Russia	A/Moscow/01/2009(H1N1)
PB2	GQ267851	H1N1	05/21/2009	Japan	A/Sakai/2/2009(H1N1)
PB2	GQ402182	H1N1	05/22/2009	Canada	A/Canada-MB/RV1977/2009(H1N1)

PB2	CY053221	H1N1	05/22/2009	USA	A/Brownsville/31H/2009(H1N1)
PB2	CY089186	H1N1	05/22/2009	Chile	A/Chile/19/2009
PB2	GQ267857	H1N1	05/22/2009	Japan	A/Shiga/1/2009(H1N1)
PB2	GQ225362	H1N1	05/22/2009	China	A/Fujian/1/2009(H1N1)
PB2	CY053213	H1N1	05/22/2009	USA	A/Brownsville/31OS/2009(H1N1)
PB2	CY046754	H1N1	05/22/2009	USA	A/Wisconsin/629-D02064/2009(H1N
PB2	CY044956	H1N1	05/22/2009	USA	A/New York/3627/2009(H1N1)
PB2	CY045100	H1N1	05/22/2009	USA	A/New York/3614/2009(H1N1)
PB2	CY046770	H1N1	05/22/2009	USA	A/Wisconsin/629-D02298/2009(H1N
PB2	CY050411	H1N1	05/23/2009	USA	A/Wisconsin/629-D00451/2009(H1N
PB2	CY053366	H1N1	05/23/2009	USA	A/Houston/20H/2009(H1N1)
PB2	CY053229	H1N1	05/23/2009	USA	A/Brownsville/34OS/2009(H1N1)
PB2	CY049912	H1N1	05/23/2009	Dominicar	A/Santo Domingo/0574T/2009(H1N1)
PB2	CY050427	H1N1	05/23/2009	USA	A/Wisconsin/629-D02206/2009(H1N
PB2	GQ225354	H1N1	05/23/2009	China	A/Shanghai/1/2009(H1N1)
PB2	CY075090	H1N1	05/23/2009	Chile	A/Chile/28/2009(H1N1)
PB2	GQ225346	H1N1	05/23/2009	China	A/Zhejiang/1/2009(H1N1)
PB2	CY053237	H1N1	05/23/2009	USA	A/Brownsville/34H/2009(H1N1)
PB2	GQ232090	H1N1	05/23/2009	China	A/Beijing/4/2009(H1N1)
PB2	CY053134	H1N1	05/23/2009	USA	A/Houston/20OS/2009(H1N1)
PB2	CY041980	H1N1	05/24/2009	Dominicar	A/Santo Domingo/0573N/2009(H1N1)
PB2	GQ402167	H1N1	05/24/2009	Canada	A/Canada-MB/RV2023/2009(H1N1)
PB2	CY046706	H1N1	05/24/2009	USA	A/Wisconsin/629-D01445/2009(H1N
PB2	CY044964	H1N1	05/24/2009	USA	A/New York/3629/2009(H1N1)
PB2	CY041957	H1N1	05/24/2009	Dominicar	A/Santo Domingo/572N/2009(H1N1)
PB2	CY045935	H1N1	05/24/2009	Canada	A/Toronto/C0270/2009(H1N1)
PB2	GQ402181	H1N1	05/24/2009	Canada	A/Canada-MB/RV1975/2009(H1N1)
PB2	GQ253489	H1N1	05/24/2009	China	A/Shanghai/37T/2009(H1N1)
PB2	CY075106	H1N1	05/25/2009	Chile	A/Chile/32/2009(H1N1)
PB2	CY075130	H1N1	05/25/2009	Chile	A/Chile/42/2009(H1N1)
PB2	CY050435	H1N1	05/25/2009	USA	A/Wisconsin/629-D02324/2009(H1N
PB2	CY043282	H1N1	05/25/2009	USA	A/New York/3651/2009(H1N1)
PB2	CY044932	H1N1	05/25/2009	USA	A/New York/3617/2009(H1N1)
PB2	CY075114	H1N1	05/25/2009	Chile	A/Chile/36/2009(H1N1)
PB2	JQ409123	H1N1	05/25/2009	Finland	A/Helsinki/Vi1/2009
PB2	CY075098	H1N1	05/25/2009	Chile	A/Chile/31/2009
PB2	CY075122	H1N1	05/25/2009	Chile	A/Chile/40/2009(H1N1)
PB2	GQ375281	H1N1	05/26/2009	Russia	A/Moscow/03/2009(H1N1)
PB2	CY043107	H1N1	05/26/2009	Dominicar	A/Santo Domingo/568T/2009(H1N1)
PB2	GU136085	H1N1	05/26/2009	Japan	A/Shizuoka-C/97/2009(H1N1)
PB2	CY046962	H1N1	05/26/2009	USA	A/New York/3654/2009(H1N1)
PB2	CY045158	H1N1	05/26/2009	USA	A/New York/3655/2009(H1N1)
PB2	CY044079	H1N1	05/26/2009	USA	A/New York/3702/2009(H1N1)
PB2	CY043099	H1N1	05/26/2009	Dominicar	A/Santo Domingo/565T/2009(H1N1)
PB2	CY044948	H1N1	05/26/2009	USA	A/New York/3625/2009(H1N1)
PB2	CY046786	H1N1	05/26/2009	USA	A/Wisconsin/629-D01894/2009(H1N
PB2	GQ247731	H1N1	05/26/2009	Russia	A/Moscow/IIV02/2009(H1N1)
PB2	GQ385297	H1N1	05/26/2009	Canada	A/Toronto/0462/2009(H1N1)
PB2	CY075138	H1N1	05/26/2009	Chile	A/Chile/56/2009(H1N1)
PB2	CY051254	H1N1	05/26/2009	USA	A/Wisconsin/629-S0035/2009(H1N1)
PB2	CY046842	H1N1	05/27/2009	USA	A/Wisconsin/629-D01883/2009(H1N
PB2	HM567685	H1N1	05/27/2009	United Ki	A/England/350/2009
PB2	CY050902	H1N1	05/27/2009	USA	A/Wisconsin/629-D00949/2009(H1N
PB2	GQ283485	H1N1	05/27/2009	Italy	A/Italy/49/2009(H1N1)

PB2	CY046818	H1N1	05/27/2009	USA	A/Wisconsin/629-D00698/2009(H1N
PB2	GQ232082	H1N1	05/27/2009	China	A/Guangdong/02/2009(H1N1)
PB2	CY076752	H1N1	05/27/2009	Canada	A/Ontario/142358/2009
PB2	CY049928	H1N1	05/27/2009	USA	A/Craven/WR1001/2009(H1N1)
PB2	CY075146	H1N1	05/27/2009	Chile	A/Chile/88/2009(H1N1)
PB2	CY075154	H1N1	05/27/2009	Chile	A/Chile/95/2009(H1N1)
PB2	CY053367	H1N1	05/27/2009	USA	A/Brownsville/36H/2009(H1N1)
PB2	CY053245	H1N1	05/27/2009	USA	A/Brownsville/350S/2009(H1N1)
PB2	CY069874	H1N1	05/27/2009	United Ki	A/England/350/2009
PB2	CY050443	H1N1	05/27/2009	USA	A/Wisconsin/629-D01886/2009(H1N
PB2	CY053253	H1N1	05/27/2009	USA	A/Brownsville/35H/2009(H1N1)
PB2	CY046794	H1N1	05/27/2009	USA	A/Wisconsin/629-D02276/2009(H1N
PB2	CY075162	H1N1	05/28/2009	Chile	A/Chile/158/2009(H1N1)
PB2	GQ402184	H1N1	05/28/2009	Canada	A/Canada-MB/RV2013/2009(H1N1)
PB2	CY046906	H1N1	05/28/2009	USA	A/Wisconsin/629-D00829/2009(H1N
PB2	CY046802	H1N1	05/28/2009	USA	A/Wisconsin/629-D01026/2009(H1N
PB2	GQ290434	H1N1	05/28/2009	China	A/Shanghai/60T/2009(H1N1)
PB2	CY045012	H1N1	05/28/2009	USA	A/New York/3709/2009(H1N1)
PB2	CY046834	H1N1	05/28/2009	USA	A/Wisconsin/629-D02144/2009(H1N
PB2	CY046914	H1N1	05/28/2009	USA	A/Wisconsin/629-D01083/2009(H1N
PB2	CY050157	H1N1	05/28/2009	USA	A/Wisconsin/629-D00117/2009(H1N
PB2	CY053316	H1N1	05/28/2009	USA	A/Brownsville/370S/2009(H1N1)
PB2	CY064788	H1N1	05/28/2009	China	A/Guangdong/SB1/2009(H1N1)
PB2	GQ288369	H1N1	05/28/2009	China	A/Fuzhou/01/2009(H1N1)
PB2	CY050918	H1N1	05/28/2009	USA	A/Wisconsin/629-D01262/2009(H1N
PB2	CY050910	H1N1	05/28/2009	USA	A/Wisconsin/629-D01779/2009(H1N
PB2	CY053268	H1N1	05/28/2009	USA	A/Brownsville/37H/2009(H1N1)
PB2	GQ402185	H1N1	05/29/2009	Canada	A/Canada-MB/RV2018/2009(H1N1)
PB2	GQ250158	H1N1	05/29/2009	China	A/Guangdong/03/2009(H1N1)
PB2	CY044087	H1N1	05/29/2009	USA	A/New York/3715/2009(H1N1)
PB2	CY050926	H1N1	05/29/2009	USA	A/Wisconsin/629-D02213/2009(H1N
PB2	CY051262	H1N1	05/29/2009	USA	A/Wisconsin/629-S0086/2009(H1N1)
PB2	CY050942	H1N1	05/29/2009	USA	A/Wisconsin/629-D01847/2009(H1N
PB2	CY075170	H1N1	05/29/2009	Chile	A/Chile/180/2009(H1N1)
PB2	CY071038	H1N1	05/29/2009	USA	A/New York/NHRC0002/2009(H1N1)
PB2	CY050934	H1N1	05/29/2009	USA	A/Wisconsin/629-D00426/2009(H1N
PB2	CY046826	H1N1	05/30/2009	USA	A/Wisconsin/629-D02013/2009(H1N
PB2	GQ402186	H1N1	05/30/2009	Canada	A/Canada-MB/RV2020/2009(H1N1)
PB2	CY055300	H1N1	05/30/2009	Singapore	A/Singapore/GN285/2009(H1N1)
PB2	HM569666	H1N1	05/30/2009	Argentina	A/Argentina/07-09GP/2009(H1N1)
PB2	CY045959	H1N1	05/30/2009	Canada	A/Toronto/T5294/2009(H1N1)
PB2	CY046810	H1N1	05/30/2009	USA	A/Wisconsin/629-D00223/2009(H1N
PB2	CY053276	H1N1	05/30/2009	USA	A/Brownsville/390S/2009(H1N1)
PB2	CY053284	H1N1	05/30/2009	USA	A/Brownsville/39H/2009(H1N1)
PB2	CY065531	H1N1	05/31/2009	United Ki	A/England/359/2009
PB2	GQ253501	H1N1	05/31/2009	China	A/Shanghai/71T/2009(H1N1)
PB2	GQ293075	H1N1	05/31/2009	China	A/Zhejiang/2/2009(H1N1)
PB2	CY044868	H1N1	05/31/2009	USA	A/New York/3726/2009(H1N1)
PB2	CY045190	H1N1	05/31/2009	USA	A/New York/3960/2009(H1N1)
PB2	CY045036	H1N1	05/31/2009	USA	A/New York/3812/2009(H1N1)
PB2	CY071030	H1N1	05/31/2009	USA	A/New York/NHRC0001/2009(H1N1)
PB2	CY089839	H1N1	06/2009	Singapore	A/Singapore/471/2009
PB2	GQ387380	H1N1	06/2009	China	A/Zhejiang/DTID-ZJU01/2009(H1N1)
PB2	CY069631	H1N1	06/2009	Singapore	A/Singapore/478/2009(H1N1)

PB2	CY089840	H1N1	06/2009	Singapore	A/Singapore/527/2009
PB2	CY047784	H1N1	06/2009	Argentina	A/Argentina/7785/2009
PB2	CY045231	H1N1	06/2009	Taiwan	A/Taiwan/126/2009(H1N1)
PB2	CY089838	H1N1	06/2009	Singapore	A/Singapore/276/2009
PB2	HQ664706	H1N1	06/2009	Taiwan	A/Taiwan/14/2009
PB2	CY045239	H1N1	06/2009	Taiwan	A/Taiwan/137/2009(H1N1)
PB2	CY054698	H1N1	06/01/2009	Nicaragua	A/Managua/4467.05/2009(H1N1)
PB2	CY045182	H1N1	06/01/2009	USA	A/New York/3959/2009(H1N1)
PB2	CY047373	H1N1	06/01/2009	USA	A/New York/3795/2009(H1N1)
PB2	CY076760	H1N1	06/01/2009	Canada	A/Ontario/147265/2009
PB2	CY050966	H1N1	06/01/2009	USA	A/Wisconsin/629-D00941/2009(H1N
PB2	CY065579	H1N1	06/01/2009	United Ki	A/England/386/2009
PB2	HM567765	H1N1	06/01/2009	United Ki	A/England/383/2009
PB2	CY050974	H1N1	06/01/2009	USA	A/Wisconsin/629-D01725/2009(H1N
PB2	CY053292	H1N1	06/01/2009	USA	A/Brownsville/400S/2009(H1N1)
PB2	CY050950	H1N1	06/01/2009	USA	A/Wisconsin/629-D00374/2009(H1N
PB2	CY069882	H1N1	06/01/2009	United Ki	A/England/383/2009
PB2	KC800977	H1N1	06/01/2009	France	A/Lyon/969/2009
PB2	CY065555	H1N1	06/01/2009	United Ki	A/England/382/2009
PB2	CY065571	H1N1	06/01/2009	United Ki	A/England/385/2009
PB2	CY065563	H1N1	06/01/2009	United Ki	A/England/384/2009
PB2	CY065547	H1N1	06/01/2009	United Ki	A/England/381/2009
PB2	CY071046	H1N1	06/01/2009	USA	A/New York/NHRC0003/2009(H1N1)
PB2	FN423711	H1N1	06/01/2009	Luxembour	A/Luxembourg/43/2009
PB2	CY065619	H1N1	06/01/2009	United Ki	A/England/395/2009
PB2	CY065539	H1N1	06/01/2009	United Ki	A/England/361/2009
PB2	CY065643	H1N1	06/01/2009	United Ki	A/England/402/2009
PB2	CY050958	H1N1	06/01/2009	USA	A/Wisconsin/629-D00724/2009(H1N
PB2	CY056394	H1N1	06/01/2009	Nicaragua	A/Managua/3119.01/2009(H1N1)
PB2	CY044996	H1N1	06/01/2009	USA	A/New York/3751/2009(H1N1)
PB2	CY045028	H1N1	06/02/2009	USA	A/New York/3803/2009(H1N1)
PB2	CY045004	H1N1	06/02/2009	USA	A/New York/3828/2009(H1N1)
PB2	CY045020	H1N1	06/02/2009	USA	A/New York/3796/2009(H1N1)
PB2	CY051230	H1N1	06/02/2009	USA	A/Wisconsin/629-D00022/2009(H1N
PB2	CY050982	H1N1	06/02/2009	USA	A/Wisconsin/629-D00367/2009(H1N
PB2	CY065611	H1N1	06/02/2009	United Ki	A/England/394/2009
PB2	CY051270	H1N1	06/02/2009	USA	A/Wisconsin/629-S0143/2009(H1N1)
PB2	CY069898	H1N1	06/02/2009	United Ki	A/England/393/2009
PB2	CY053300	H1N1	06/02/2009	USA	A/Brownsville/430S/2009(H1N1)
PB2	CY053308	H1N1	06/02/2009	USA	A/Brownsville/43H/2009(H1N1)
PB2	GQ324577	H1N1	06/02/2009	Japan	A/Tokushima/1/2009(H1N1)
PB2	HM567781	H1N1	06/02/2009	United Ki	A/England/393/2009
PB2	CY065587	H1N1	06/02/2009	United Ki	A/England/388/2009
PB2	HM567773	H1N1	06/02/2009	United Ki	A/England/391/2009
PB2	CY069890	H1N1	06/02/2009	United Ki	A/England/391/2009
PB2	CY076768	H1N1	06/02/2009	Canada	A/Ontario/147723/2009(H1N1)
PB2	CY050990	H1N1	06/02/2009	USA	A/Wisconsin/629-D00592/2009(H1N
PB2	CY051286	H1N1	06/02/2009	USA	A/Wisconsin/629-S0148/2009(H1N1)
PB2	CY051278	H1N1	06/02/2009	USA	A/Wisconsin/629-S0146/2009(H1N1)
PB2	GQ334352	H1N1	06/02/2009	Japan	A/Shizuoka/759/2009(H1N1)
PB2	CY045166	H1N1	06/02/2009	USA	A/New York/3818/2009(H1N1)
PB2	CY045174	H1N1	06/02/2009	USA	A/New York/3819/2009(H1N1)
PB2	CY054690	H1N1	06/03/2009	USA	A/Wisconsin/629-D00589/2009(H1N
PB2	CY051238	H1N1	06/03/2009	USA	A/Wisconsin/629-D01664/2009(H1N

PB2	CY065627	H1N1	06/03/2009	United Ki	A/England/398/2009
PB2	CY107575	H1N1	06/03/2009	United Ki	A/Scotland/Glasgow_412313/2009
PB2	KC781927	H1N1	06/03/2009	USA	A/Virginia/24/2009
PB2	KC781437	H1N1	06/03/2009	USA	A/Utah/27/2009
PB2	CY065603	H1N1	06/03/2009	United Ki	A/England/392/2009
PB2	CY071054	H1N1	06/03/2009	USA	A/New York/NHRC0004/2009(H1N1)
PB2	GQ330652	H1N1	06/03/2009	Russia	A/Moscow/IIV03/2009(H1N1)
PB2	CY044103	H1N1	06/03/2009	USA	A/New York/3895/2009(H1N1)
PB2	GQ411904	H1N1	06/03/2009	Canada	A/Toronto/T5308/2009(H1N1)
PB2	GU136088	H1N1	06/03/2009	Japan	A/Yamaguchi/21/2009(H1N1)
PB2	CY065595	H1N1	06/03/2009	United Ki	A/England/389/2009
PB2	CY076776	H1N1	06/04/2009	Canada	A/Ontario/152439/2009
PB2	CY051054	H1N1	06/04/2009	USA	A/Wisconsin/629-D00008/2009(H1N
PB2	CY051038	H1N1	06/04/2009	USA	A/Wisconsin/629-D00658/2009(H1N
PB2	CY089903	H1N1	06/04/2009	USA	A/Wisconsin/629-D01420/2009(H1N
PB2	CY051294	H1N1	06/04/2009	USA	A/Wisconsin/629-S0173/2009(H1N1)
PB2	CY043339	H1N1	06/04/2009	Denmark	A/Denmark/524/2009(H1N1)
PB2	CY065691	H1N1	06/04/2009	United Ki	A/England/428/2009
PB2	CY065683	H1N1	06/04/2009	United Ki	A/England/423/2009
PB2	CY065659	H1N1	06/04/2009	United Ki	A/England/414/2009
PB2	CY051030	H1N1	06/04/2009	USA	A/Wisconsin/629-D00017/2009(H1N
PB2	CY065651	H1N1	06/04/2009	United Ki	A/England/411/2009
PB2	CY051022	H1N1	06/04/2009	USA	A/Wisconsin/629-D00453/2009(H1N
PB2	CY051046	H1N1	06/04/2009	USA	A/Wisconsin/629-D00725/2009(H1N
PB2	CY076784	H1N1	06/04/2009	Canada	A/Ontario/152846/2009
PB2	CY044111	H1N1	06/05/2009	USA	A/New York/3898/2009(H1N1)
PB2	CY052145	H1N1	06/05/2009	USA	A/New York/3980/2009(H1N1)
PB2	CY065699	H1N1	06/05/2009	United Ki	A/England/430/2009
PB2	CY072917	H1N1	06/05/2009	Nicaragua	A/Managua/2652.01/2009(H1N1)
PB2	CY065667	H1N1	06/05/2009	United Ki	A/England/418/2009
PB2	CY065635	H1N1	06/05/2009	United Ki	A/England/400/2009
PB2	CY065715	H1N1	06/05/2009	United Ki	A/England/433/2009
PB2	CY065707	H1N1	06/05/2009	United Ki	A/England/431/2009
PB2	CY058171	H1N1	06/05/2009	Nicaragua	A/Managua/4016.02/2009(H1N1)
PB2	CY065675	H1N1	06/05/2009	United Ki	A/England/419/2009
PB2	CY058531	H1N1	06/05/2009	Nicaragua	A/Managua/4905.02/2009(H1N1)
PB2	CY072973	H1N1	06/05/2009	Nicaragua	A/Managua/2750.01/2009(H1N1)
PB2	CY050998	H1N1	06/05/2009	USA	A/Wisconsin/629-D01903/2009(H1N
PB2	CY065723	H1N1	06/06/2009	United Ki	A/England/434/2009
PB2	CY083596	H1N1	06/06/2009	Nicaragua	A/Managua/WRAIR8964F/2009(H1N1)
PB2	HM567869	H1N1	06/06/2009	United Ki	A/England/421/2009
PB2	CY058091	H1N1	06/06/2009	Nicaragua	A/Managua/1455.01/2009(H1N1)
PB2	JX625979	H1N1	06/06/2009	United Ki	A/England/421/2009
PB2	GQ324571	H1N1	06/06/2009	Japan	A/Shiga/3/2009(H1N1)
PB2	CY053126	H1N1	06/07/2009	USA	A/Houston/170S/2009(H1N1)
PB2	CY058083	H1N1	06/07/2009	Nicaragua	A/Managua/171.01/2009(H1N1)
PB2	GQ334337	H1N1	06/07/2009	Japan	A/Fukuoka-C/2/2009(H1N1)
PB2	GQ334345	H1N1	06/07/2009	Japan	A/Fukuoka-C/3/2009(H1N1)
PB2	GQ300860	H1N1	06/07/2009	Japan	A/Fukuoka-C/1/2009(H1N1)
PB2	CY051318	H1N1	06/08/2009	USA	A/Wisconsin/629-S0201/2009(H1N1)
PB2	CY045214	H1N1	06/08/2009	USA	A/New York/4057/2009(H1N1)
PB2	CY051510	H1N1	06/08/2009	USA	A/Wisconsin/629-S0206/2009(H1N1)
PB2	CY051502	H1N1	06/08/2009	USA	A/Wisconsin/629-S0189/2009(H1N1)
PB2	CY051070	H1N1	06/08/2009	USA	A/Wisconsin/629-D00101/2009(H1N

PB2	CY051078	H1N1	06/08/2009	USA	A/Wisconsin/629-D00579/2009(H1N
PB2	KC781795	H1N1	06/08/2009	USA	A/Wisconsin/26/2009
PB2	CY051334	H1N1	06/08/2009	USA	A/Wisconsin/629-S0203/2009(H1N1)
PB2	CY051310	H1N1	06/08/2009	USA	A/Wisconsin/629-S0197/2009(H1N1)
PB2	CY051302	H1N1	06/08/2009	USA	A/Wisconsin/629-S0186/2009(H1N1)
PB2	CY051326	H1N1	06/08/2009	USA	A/Wisconsin/629-S0202/2009(H1N1)
PB2	CY076792	H1N1	06/08/2009	Canada	A/Ontario/156785/2009(H1N1)
PB2	CY058139	H1N1	06/08/2009	Nicaragua	A/Managua/516.01/2009(H1N1)
PB2	CY051062	H1N1	06/08/2009	USA	A/Wisconsin/629-D02068/2009(H1N
PB2	CY052137	H1N1	06/08/2009	USA	A/New York/3976/2009(H1N1)
PB2	CY083149	H1N1	06/08/2009	Dominican	A/Santo Domingo/WRAIR1044N/2009
PB2	CY043347	H1N1	06/09/2009	Denmark	A/Denmark/528/2009(H1N1)
PB2	CY045044	H1N1	06/09/2009	USA	A/New York/3967/2009(H1N1)
PB2	CY045060	H1N1	06/09/2009	USA	A/New York/4052/2009(H1N1)
PB2	GQ375888	H1N1	06/09/2009	Japan	A/Iwate/1/2009(H1N1)
PB2	CY051350	H1N1	06/09/2009	USA	A/Wisconsin/629-S0222/2009(H1N1)
PB2	CY051342	H1N1	06/09/2009	USA	A/Wisconsin/629-S0220/2009(H1N1)
PB2	CY051358	H1N1	06/09/2009	USA	A/Wisconsin/629-S0226/2009(H1N1)
PB2	CY051246	H1N1	06/09/2009	USA	A/Wisconsin/629-D01199/2009(H1N
PB2	GQ324583	H1N1	06/09/2009	Japan	A/Yokohama/1/2009(H1N1)
PB2	CY052297	H1N1	06/09/2009	USA	A/Texas/42091791/2009(H1N1)
PB2	CY065731	H1N1	06/09/2009	United Ki	A/England/445/2009
PB2	CY045967	H1N1	06/09/2009	Canada	A/Toronto/T5362/2009(H1N1)
PB2	CY052129	H1N1	06/09/2009	USA	A/New York/3969/2009(H1N1)
PB2	CY058107	H1N1	06/09/2009	Nicaragua	A/Managua/168.02/2009(H1N1)
PB2	CY051518	H1N1	06/09/2009	USA	A/Wisconsin/629-S0208/2009(H1N1)
PB2	CY045222	H1N1	06/10/2009	USA	A/New York/4074/2009(H1N1)
PB2	CY051374	H1N1	06/10/2009	USA	A/Wisconsin/629-S0248/2009(H1N1)
PB2	CY051406	H1N1	06/10/2009	USA	A/Wisconsin/629-S0252/2009(H1N1)
PB2	CY052273	H1N1	06/10/2009	USA	A/Texas/42102708/2009(H1N1)
PB2	CY045052	H1N1	06/10/2009	USA	A/New York/4006/2009(H1N1)
PB2	CY044119	H1N1	06/10/2009	USA	A/New York/3992/2009(H1N1)
PB2	CY052305	H1N1	06/10/2009	USA	A/Texas/42103399/2009(H1N1)
PB2	CY051382	H1N1	06/10/2009	USA	A/Wisconsin/629-S0247/2009(H1N1)
PB2	CY051398	H1N1	06/10/2009	USA	A/Wisconsin/629-S0255/2009(H1N1)
PB2	CY045206	H1N1	06/10/2009	USA	A/New York/4048/2009(H1N1)
PB2	CY051366	H1N1	06/10/2009	USA	A/Wisconsin/629-S0245/2009(H1N1)
PB2	CY051390	H1N1	06/10/2009	USA	A/Wisconsin/629-S0254/2009(H1N1)
PB2	CY045198	H1N1	06/10/2009	USA	A/New York/4014/2009(H1N1)
PB2	CY057213	H1N1	06/11/2009	Nicaragua	A/Managua/167.01/2009(H1N1)
PB2	CY051414	H1N1	06/11/2009	USA	A/Wisconsin/629-S0269/2009(H1N1)
PB2	CY051886	H1N1	06/11/2009	USA	A/Texas/42113095/2009(H1N1)
PB2	CY051894	H1N1	06/11/2009	USA	A/Texas/42114261/2009(H1N1)
PB2	CY053118	H1N1	06/11/2009	USA	A/Houston/160S/2009(H1N1)
PB2	GQ365453	H1N1	06/11/2009	Japan	A/Sapporo/1/2009(H1N1)
PB2	CY065739	H1N1	06/11/2009	United Ki	A/Scotland/10/2009
PB2	CY063493	H1N1	06/11/2009	USA	A/Boston/110/2009(H1N1)
PB2	CY051086	H1N1	06/11/2009	USA	A/Wisconsin/629-D01326/2009(H1N
PB2	GQ365417	H1N1	06/11/2009	Japan	A/Akita/1/2009(H1N1)
PB2	GU136070	H1N1	06/12/2009	Japan	A/Kanagawa/137/2009(H1N1)
PB2	CY058099	H1N1	06/12/2009	Nicaragua	A/Managua/655.02/2009(H1N1)
PB2	CY045084	H1N1	06/12/2009	USA	A/New York/4072/2009(H1N1)
PB2	KT180549	H1N1	06/12/2009	Singapore	A/Singapore/DMS1/2009
PB2	CY058219	H1N1	06/12/2009	Nicaragua	A/Managua/578.01/2009(H1N1)

PB2	CY051862	H1N1	06/12/2009	USA	A/Texas/42122969/2009(H1N1)
PB2	CY050165	H1N1	06/12/2009	USA	A/New York/4095/2009(H1N1)
PB2	CY052201	H1N1	06/12/2009	USA	A/Texas/42121926/2009(H1N1)
PB2	HM569690	H1N1	06/12/2009	Argentina	A/Argentina/19618/2009(H1N1)
PB2	CY051118	H1N1	06/12/2009	USA	A/Wisconsin/629-D02263/2009(H1N
PB2	CY051846	H1N1	06/12/2009	USA	A/Texas/42123701/2009(H1N1)
PB2	GU136063	H1N1	06/13/2009	Japan	A/Hiroshima/200/2009(H1N1)
PB2	CY057221	H1N1	06/13/2009	Nicaragua	A/Managua/109.01/2009(H1N1)
PB2	CY052321	H1N1	06/13/2009	USA	A/Texas/42132413/2009(H1N1)
PB2	CY054754	H1N1	06/13/2009	USA	A/California/VRDL9/2009(H1N1)
PB2	CY058123	H1N1	06/13/2009	Nicaragua	A/Managua/1637.01/2009(H1N1)
PB2	CY064507	H1N1	06/13/2009	USA	A/Boston/115/2009(H1N1)
PB2	GQ365443	H1N1	06/13/2009	Japan	A/Kagoshima/1/2009(H1N1)
PB2	CY058179	H1N1	06/13/2009	Nicaragua	A/Managua/792.01/2009(H1N1)
PB2	CY058115	H1N1	06/13/2009	Nicaragua	A/Managua/2152.01/2009(H1N1)
PB2	CY058235	H1N1	06/13/2009	Nicaragua	A/Managua/5363.02/2009(H1N1)
PB2	CY058227	H1N1	06/13/2009	Nicaragua	A/Managua/5221.03/2009(H1N1)
PB2	KC782099	H1N1	06/13/2009	USA	A/Rhode Island/15/2009
PB2	CY075178	H1N1	06/13/2009	Chile	A/Chile/1586/2009(H1N1)
PB2	CY051102	H1N1	06/13/2009	USA	A/Wisconsin/629-D00196/2009(H1N
PB2	CY051006	H1N1	06/13/2009	USA	A/Wisconsin/629-D00665/2009(H1N
PB2	CY063501	H1N1	06/13/2009	USA	A/Boston/112/2009(H1N1)
PB2	CY058155	H1N1	06/13/2009	Nicaragua	A/Managua/4085.02/2009(H1N1)
PB2	GQ866948	H1N1	06/13/2009	Thailand	A/Thailand/CU-B5/2009(H1N1)
PB2	GQ463197	H1N1	06/13/2009	China	A/Hunan/SWL3/2009(H1N1)
PB2	CY054730	H1N1	06/13/2009	USA	A/California/VRDL6/2009(H1N1)
PB2	CY051110	H1N1	06/13/2009	USA	A/Wisconsin/629-D01618/2009(H1N
PB2	JQ409131	H1N1	06/13/2009	Finland	A/Helsinki/Vi2/2009
PB2	JQ409139	H1N1	06/13/2009	Finland	A/Helsinki/Vi3/2009(H1N1)
PB2	CY064467	H1N1	06/13/2009	USA	A/Boston/96/2009(H1N1)
PB2	CY083500	H1N1	06/13/2009	Nicaragua	A/Managua/WRAIR1693P/2009(H1N1)
PB2	CY051134	H1N1	06/14/2009	USA	A/Wisconsin/629-D00119/2009(H1N
PB2	CY049920	H1N1	06/14/2009	USA	A/New Bern/WR0670/2009(H1N1)
PB2	CY052398	H1N1	06/14/2009	USA	A/Texas/42142537/2009(H1N1)
PB2	CY054746	H1N1	06/14/2009	USA	A/California/VRDL8/2009(H1N1)
PB2	GQ351287	H1N1	06/14/2009	Italy	A/Italy/85/2009(H1N1)
PB2	GQ457462	H1N1	06/14/2009	USA	A/Utah/05/2009(H1N1)
PB2	KJ130177	H1N1	06/14/2009	New Zeala	A/New Zealand/1212d/2009
PB2	KJ130161	H1N1	06/14/2009	New Zeala	A/New Zealand/1212b/2009
PB2	CY054714	H1N1	06/14/2009	USA	A/California/VRDL4/2009(H1N1)
PB2	CY051158	H1N1	06/14/2009	USA	A/Wisconsin/629-D01147/2009(H1N
PB2	KJ130169	H1N1	06/14/2009	New Zeala	A/New Zealand/1212a/2009(H1N1)
PB2	CY051126	H1N1	06/14/2009	USA	A/Wisconsin/629-D00916/2009(H1N
PB2	GU136079	H1N1	06/15/2009	Japan	A/Niigata/690/2009(H1N1)
PB2	CY063509	H1N1	06/15/2009	USA	A/Boston/113/2009(H1N1)
PB2	CY075210	H1N1	06/15/2009	Chile	A/Chile/1603/2009(H1N1)
PB2	CY075218	H1N1	06/15/2009	Chile	A/Chile/1624/2009(H1N1)
PB2	CY051094	H1N1	06/15/2009	USA	A/Wisconsin/629-D01844/2009(H1N
PB2	CY051142	H1N1	06/15/2009	USA	A/Wisconsin/629-D01152/2009(H1N
PB2	CY051430	H1N1	06/15/2009	USA	A/Wisconsin/629-S0279/2009(H1N1)
PB2	CY051438	H1N1	06/15/2009	USA	A/Wisconsin/629-S0293/2009(H1N1)
PB2	CY051822	H1N1	06/15/2009	USA	A/Texas/42152486/2009(H1N1)
PB2	CY052281	H1N1	06/15/2009	USA	A/Texas/42151049/2009(H1N1)
PB2	HM569682	H1N1	06/15/2009	Argentina	A/Argentina/19527/2009(H1N1)

PB2	CY058131	H1N1	06/15/2009	Nicaragua	A/Managua/5453.01/2009(H1N1)
PB2	CY064475	H1N1	06/15/2009	USA	A/Boston/103/2009(H1N1)
PB2	GQ433894	H1N1	06/15/2009	China	A/Jiangsu/1/2009(H1N1)
PB2	CY075194	H1N1	06/15/2009	Chile	A/Chile/1599/2009(H1N1)
PB2	CY060739	H1N1	06/15/2009	Canada	A/Ontario/9698/2009(H1N1)
PB2	CY075202	H1N1	06/15/2009	Chile	A/Chile/1600/2009(H1N1)
PB2	CY054722	H1N1	06/15/2009	USA	A/California/VRDL5/2009(H1N1)
PB2	CY058555	H1N1	06/15/2009	Nicaragua	A/Managua/4935.03/2009(H1N1)
PB2	CY075186	H1N1	06/15/2009	Chile	A/Chile/1598/2009(H1N1)
PB2	KC781546	H1N1	06/15/2009	USA	A/Massachusetts/16/2009
PB2	CY060747	H1N1	06/15/2009	Canada	A/Ontario/9739/2009(H1N1)
PB2	CY051422	H1N1	06/15/2009	USA	A/Wisconsin/629-S0280/2009(H1N1)
PB2	CY051014	H1N1	06/15/2009	USA	A/Wisconsin/629-D01619/2009(H1N
PB2	CY064483	H1N1	06/15/2009	USA	A/Boston/106/2009(H1N1)
PB2	CY055462	H1N1	06/15/2009	USA	A/California/VRDL12/2009(H1N1)
PB2	CY064499	H1N1	06/15/2009	USA	A/Boston/109/2009(H1N1)
PB2	KC781700	H1N1	06/15/2009	USA	A/Oregon/10/2009
PB2	CY044168	H1N1	06/16/2009	USA	A/Bethesda/SP506/2009(H1N1)
PB2	CY051470	H1N1	06/16/2009	USA	A/Wisconsin/629-S0314/2009(H1N1)
PB2	CY064491	H1N1	06/16/2009	USA	A/Boston/108/2009(H1N1)
PB2	GQ365454	H1N1	06/16/2009	Japan	A/Utsunomiya/2/2009(H1N1)
PB2	GU136059	H1N1	06/16/2009	Japan	A/Aichi/202/2009(H1N1)
PB2	GQ334354	H1N1	06/16/2009	Japan	A/Utsunomiya/1/2009(H1N1)
PB2	CY052217	H1N1	06/16/2009	USA	A/Texas/42163291/2009(H1N1)
PB2	CY051150	H1N1	06/16/2009	USA	A/Wisconsin/629-D01734/2009(H1N
PB2	CY051166	H1N1	06/16/2009	USA	A/Wisconsin/629-D00541/2009(H1N
PB2	CY052390	H1N1	06/16/2009	USA	A/Texas/42163295/2009(H1N1)
PB2	CY058163	H1N1	06/16/2009	Nicaragua	A/Managua/2330.02/2009(H1N1)
PB2	CY054850	H1N1	06/17/2009	USA	A/California/VRDL31/2009(H1N1)
PB2	CY064595	H1N1	06/17/2009	USA	A/Boston/127/2009(H1N1)
PB2	CY064579	H1N1	06/17/2009	USA	A/Boston/125/2009(H1N1)
PB2	GQ455029	H1N1	06/17/2009	China	A/Nanjing/2/2009(H1N1)
PB2	CY044127	H1N1	06/17/2009	USA	A/New York/4197/2009(H1N1)
PB2	CY047776	H1N1	06/17/2009	Argentina	A/Argentina/7649/2009
PB2	CY052105	H1N1	06/17/2009	USA	A/New York/4238/2009(H1N1)
PB2	GQ392026	H1N1	06/17/2009	Italy	A/Italy/127/2009(H1N1)
PB2	GQ866956	H1N1	06/17/2009	Thailand	A/Thailand/CU-H9/2009
PB2	GU136064	H1N1	06/17/2009	Japan	A/Hiroshima/201/2009(H1N1)
PB2	CY045975	H1N1	06/17/2009	Canada	A/Toronto/T9842/2009(H1N1)
PB2	CY107190	H1N1	06/17/2009	United Ki	A/Scotland/Paisley_417557/2009
PB2	CY055454	H1N1	06/17/2009	USA	A/California/VRDL11/2009(H1N1)
PB2	CY047808	H1N1	06/17/2009	Argentina	A/Argentina/7967/2009
PB2	CY047816	H1N1	06/17/2009	Argentina	A/Argentina/7980/2009
PB2	CY051878	H1N1	06/17/2009	USA	A/Texas/42173957/2009(H1N1)
PB2	CY054738	H1N1	06/17/2009	USA	A/California/VRDL7/2009(H1N1)
PB2	CY051446	H1N1	06/17/2009	USA	A/Wisconsin/629-S0319/2009(H1N1)
PB2	CY051550	H1N1	06/17/2009	USA	A/New York/4403/2009(H1N1)
PB2	CY064635	H1N1	06/18/2009	USA	A/Boston/133/2009(H1N1)
PB2	GQ504749	H1N1	06/18/2009	China	A/Nanjing/1/2009(H1N1)
PB2	CY043115	H1N1	06/18/2009	USA	A/Bethesda/SP508/2009(H1N1)
PB2	CY073275	H1N1	06/18/2009	USA	A/Silver Spring/WRAIRSP510P18/2(
PB2	CY075226	H1N1	06/18/2009	Chile	A/Chile/2009/2009(H1N1)
PB2	CY064651	H1N1	06/18/2009	USA	A/Boston/135/2009(H1N1)
PB2	CY051462	H1N1	06/18/2009	USA	A/Wisconsin/629-S0339/2009(H1N1)

PB2	CY051454	H1N1	06/18/2009	USA	A/Wisconsin/629-S0345/2009(H1N1)
PB2	CY051174	H1N1	06/18/2009	USA	A/Wisconsin/629-D02063/2009(H1N
PB2	GQ365435	H1N1	06/18/2009	Japan	A/Iwate/2/2009(H1N1)
PB2	CY064667	H1N1	06/18/2009	USA	A/Boston/138/2009(H1N1)
PB2	GU136071	H1N1	06/18/2009	Japan	A/Kanagawa/140/2009(H1N1)
PB2	CY073291	H1N1	06/18/2009	USA	A/Silver Spring/WRAIRSP510P20/2(
PB2	GU136061	H1N1	06/18/2009	Japan	A/Chiba-C/51/2009(H1N1)
PB2	CY064563	H1N1	06/18/2009	USA	A/Boston/123/2009(H1N1)
PB2	KC781805	H1N1	06/18/2009	USA	A/Oregon/11/2009
PB2	HM569698	H1N1	06/18/2009	Argentina	A/Argentina/19656/2009(H1N1)
PB2	CY047824	H1N1	06/18/2009	Argentina	A/Argentina/8019/2009
PB2	KC781844	H1N1	06/18/2009	USA	A/Oregon/13/2009
PB2	CY054810	H1N1	06/18/2009	USA	A/California/VRDL26/2009(H1N1)
PB2	CY123918	H1N1	06/19/2009	Singapore	A/Singapore/ON313/2009
PB2	CY064547	H1N1	06/19/2009	USA	A/Boston/121/2009(H1N1)
PB2	CY083085	H1N1	06/19/2009	Colombia	A/Bogota/WRAIR0442T/2009
PB2	CY045076	H1N1	06/19/2009	USA	A/New York/4290/2009(H1N1)
PB2	CY047800	H1N1	06/19/2009	Argentina	A/Argentina/7953/2009
PB2	CY047848	H1N1	06/19/2009	Argentina	A/Argentina/8673/2009
PB2	CY051566	H1N1	06/19/2009	USA	A/New York/4459/2009(H1N1)
PB2	CY064627	H1N1	06/19/2009	USA	A/Boston/132/2009(H1N1)
PB2	CY064539	H1N1	06/19/2009	USA	A/Boston/120/2009(H1N1)
PB2	CY052422	H1N1	06/19/2009	USA	A/Texas/42192947/2009(H1N1)
PB2	CY052241	H1N1	06/19/2009	USA	A/Texas/42191647/2009(H1N1)
PB2	CY075234	H1N1	06/19/2009	Chile	A/Chile/2239/2009(H1N1)
PB2	CY051854	H1N1	06/19/2009	USA	A/Texas/42191653/2009(H1N1)
PB2	CY052249	H1N1	06/20/2009	USA	A/Texas/42201798/2009(H1N1)
PB2	CY051870	H1N1	06/20/2009	USA	A/Texas/42201824/2009(H1N1)
PB2	CY072989	H1N1	06/20/2009	Nicaragua	A/Managua/5273.02/2009(H1N1)
PB2	CY052329	H1N1	06/20/2009	USA	A/Texas/42202026/2009(H1N1)
PB2	CY064531	H1N1	06/20/2009	USA	A/Boston/118/2009(H1N1)
PB2	CY064659	H1N1	06/20/2009	USA	A/Boston/137/2009(H1N1)
PB2	CY054770	H1N1	06/20/2009	USA	A/California/VRDL20/2009(H1N1)
PB2	CY051198	H1N1	06/20/2009	USA	A/Wisconsin/629-D00496/2009(H1N
PB2	GQ494357	H1N1	06/20/2009	Russia	A/Moscow/IIV05/2009(H1N1)
PB2	CY051182	H1N1	06/20/2009	USA	A/Wisconsin/629-D00428/2009(H1N
PB2	CY075242	H1N1	06/20/2009	Chile	A/Chile/2851/2009(H1N1)
PB2	CY049057	H1N1	06/20/2009	Singapore	A/Singapore/ON305/2009(H1N1)
PB2	CY047872	H1N1	06/20/2009	Argentina	A/Argentina/9004/2009
PB2	KT180684	H1N1	06/20/2009	Singapore	A/Singapore/DMS385/2009
PB2	CY064587	H1N1	06/20/2009	USA	A/Boston/126/2009(H1N1)
PB2	CY047856	H1N1	06/21/2009	Argentina	A/Argentina/8989/2009
PB2	CY064611	H1N1	06/21/2009	USA	A/Boston/130/2009(H1N1)
PB2	CY051190	H1N1	06/21/2009	USA	A/Wisconsin/629-D02220/2009(H1N
PB2	CY058539	H1N1	06/21/2009	Nicaragua	A/Managua/4514.03/2009(H1N1)
PB2	CY047840	H1N1	06/21/2009	Argentina	A/Argentina/8574/2009
PB2	CY052337	H1N1	06/21/2009	USA	A/Texas/42211898/2009(H1N1)
PB2	CY051542	H1N1	06/21/2009	USA	A/New York/4402/2009(H1N1)
PB2	CY052265	H1N1	06/22/2009	USA	A/Texas/42221280/2009(H1N1)
PB2	CY047864	H1N1	06/22/2009	Argentina	A/Argentina/8994/2009
PB2	CY123343	H1N1	06/22/2009	Singapore	A/Singapore/GP687/2009
PB2	CY075250	H1N1	06/22/2009	Chile	A/Chile/2362/2009(H1N1)
PB2	CY123926	H1N1	06/22/2009	Singapore	A/Singapore/ON355/2009(H1N1)
PB2	CY111618	H1N1	06/22/2009	Hong Kong	A/Hong Kong/H090-661-V10/2009

PB2	CY120500	H1N1	06/22/2009	Hong Kong	A/Hong Kong/H090-661-V10/2009
PB2	CY054826	H1N1	06/22/2009	USA	A/California/VRDL28/2009(H1N1)
PB2	CY055478	H1N1	06/22/2009	USA	A/California/VRDL18/2009(H1N1)
PB2	CY058147	H1N1	06/22/2009	Nicaragua	A/Managua/5665.01/2009(H1N1)
PB2	GU136080	H1N1	06/22/2009	Japan	A/Niigata/700/2009(H1N1)
PB2	KC781714	H1N1	06/22/2009	USA	A/New York/98/2009
PB2	CY051206	H1N1	06/22/2009	USA	A/Wisconsin/629-D02024/2009(H1N1
PB2	KT180703	H1N1	06/22/2009	Singapore	A/Singapore/DMS562/2009
PB2	KT180550	H1N1	06/22/2009	Singapore	A/Singapore/DMS4/2009
PB2	KT180697	H1N1	06/22/2009	Singapore	A/Singapore/DMS516/2009
PB2	CY064603	H1N1	06/22/2009	USA	A/Boston/129/2009(H1N1)
PB2	CY064555	H1N1	06/22/2009	USA	A/Boston/122/2009(H1N1)
PB2	CY064675	H1N1	06/22/2009	USA	A/Boston/140/2009(H1N1)
PB2	CY054778	H1N1	06/22/2009	USA	A/California/VRDL21/2009(H1N1)
PB2	CY047968	H1N1	06/23/2009	Argentina	A/Argentina/9721/2009
PB2	CY051558	H1N1	06/23/2009	USA	A/New York/4434/2009(H1N1)
PB2	JX625408	H1N1	06/23/2009	United Ki	A/England/92880002/2009
PB2	CY047896	H1N1	06/23/2009	Argentina	A/Argentina/9384/2009
PB2	KT180700	H1N1	06/23/2009	Singapore	A/Singapore/DMS547/2009
PB2	CY055470	H1N1	06/23/2009	USA	A/California/VRDL14/2009(H1N1)
PB2	GQ365425	H1N1	06/23/2009	Japan	A/Fukushima/1/2009(H1N1)
PB2	CY047880	H1N1	06/23/2009	Argentina	A/Argentina/9180/2009
PB2	CY047888	H1N1	06/23/2009	Argentina	A/Argentina/9333/2009
PB2	CY051486	H1N1	06/23/2009	USA	A/Wisconsin/629-S0410/2009(H1N1)
PB2	GU136081	H1N1	06/23/2009	Japan	A/Niigata/717/2009(H1N1)
PB2	CY065155	H1N1	06/23/2009	United Ki	A/England/541/2009
PB2	CY052153	H1N1	06/24/2009	USA	A/New York/4398/2009(H1N1)
PB2	CY054834	H1N1	06/24/2009	USA	A/California/VRDL29/2009(H1N1)
PB2	CY083620	H1N1	06/24/2009	Peru	A/Trujillo/WRAIR9309F/2009
PB2	CY064619	H1N1	06/24/2009	USA	A/Boston/131/2009(H1N1)
PB2	KF612038	H1N1	06/24/2009	Bolivia	A/Santa Cruz/46631/2009
PB2	KT180671	H1N1	06/24/2009	Singapore	A/Singapore/DMS223/2009
PB2	KT180551	H1N1	06/24/2009	Singapore	A/Singapore/DMS5/2009
PB2	CY047920	H1N1	06/24/2009	Argentina	A/Argentina/9579/2009
PB2	CY087223	H1N1	06/24/2009	USA	A/Houston/210S/2009
PB2	CY107816	H1N1	06/24/2009	United Ki	A/Scotland/StAndrews_17693/2009
PB2	CY051478	H1N1	06/24/2009	USA	A/Wisconsin/629-S0435/2009(H1N1)
PB2	CY054786	H1N1	06/24/2009	USA	A/California/VRDL22/2009(H1N1)
PB2	GU136069	H1N1	06/24/2009	Japan	A/Iwate/3/2009(H1N1)
PB2	CY047952	H1N1	06/24/2009	Argentina	A/Argentina/9705/2009
PB2	CY107323	H1N1	06/24/2009	United Ki	A/Scotland/Edinburgh_17715/2009
PB2	CY083564	H1N1	06/25/2009	Peru	A/Lima/WRAIR8511F/2009
PB2	CY064683	H1N1	06/25/2009	USA	A/Boston/141/2009(H1N1)
PB2	CY075266	H1N1	06/25/2009	Chile	A/Chile/2911/2009(H1N1)
PB2	CY052161	H1N1	06/25/2009	USA	A/New York/4401/2009(H1N1)
PB2	CY050189	H1N1	06/25/2009	USA	A/New York/4438/2009(H1N1)
PB2	CY058211	H1N1	06/25/2009	Nicaragua	A/Managua/791.03/2009(H1N1)
PB2	CY044144	H1N1	06/25/2009	Colombia	A/Bogota/0466N/2009(H1N1)
PB2	CY057229	H1N1	06/25/2009	Nicaragua	A/Managua/611.01/2009(H1N1)
PB2	CY049393	H1N1	06/25/2009	Singapore	A/Singapore/ON506/2009(H1N1)
PB2	KJ130193	H1N1	06/25/2009	New Zeala	A/New Zealand/2047/2009
PB2	GU136068	H1N1	06/25/2009	Japan	A/Hiroshima/230/2009(H1N1)
PB2	CY051534	H1N1	06/25/2009	USA	A/New York/4400/2009(H1N1)
PB2	CY083524	H1N1	06/25/2009	Peru	A/Lima/WRAIR1696P/2009

PB2	CY075258	H1N1	06/25/2009	Chile	A/Chile/2909/2009(H1N1)
PB2	CY052313	H1N1	06/25/2009	USA	A/Texas/42254309/2009(H1N1)
PB2	CY075290	H1N1	06/26/2009	Chile	A/Chile/3056/2009
PB2	CY075274	H1N1	06/26/2009	Chile	A/Chile/2994/2009(H1N1)
PB2	CY053173	H1N1	06/26/2009	USA	A/Houston/240S/2009(H1N1)
PB2	CY124001	H1N1	06/26/2009	Singapore	A/Singapore/ON506/2009
PB2	GU136074	H1N1	06/26/2009	Myanmar	A/Myanmar/60/2009(H1N1)
PB2	CY064523	H1N1	06/26/2009	USA	A/Boston/117/2009(H1N1)
PB2	CY064571	H1N1	06/26/2009	USA	A/Boston/124/2009(H1N1)
PB2	CY064643	H1N1	06/26/2009	USA	A/Boston/134/2009(H1N1)
PB2	CY058547	H1N1	06/26/2009	Nicaragua	A/Managua/462.01/2009(H1N1)
PB2	KC782288	H1N1	06/26/2009	USA	A/Texas/48/2009
PB2	CY052121	H1N1	06/26/2009	USA	A/Wisconsin/629-D01973/2009(H1N
PB2	JX625384	H1N1	06/26/2009	United Ki	A/England/506/2009
PB2	CY075298	H1N1	06/26/2009	Chile	A/Chile/3123/2009(H1N1)
PB2	CY075282	H1N1	06/26/2009	Chile	A/Chile/3019/2009(H1N1)
PB2	CY064902	H1N1	06/26/2009	USA	A/Boston/136/2009(H1N1)
PB2	CY054762	H1N1	06/26/2009	USA	A/California/VRDL19/2009(H1N1)
PB2	CY083452	H1N1	06/27/2009	Peru	A/Lima/WRAIR1687P/2009
PB2	CY051214	H1N1	06/27/2009	USA	A/Wisconsin/629-D01414/2009(H1N
PB2	CY054818	H1N1	06/27/2009	USA	A/California/VRDL27/2009(H1N1)
PB2	CY054842	H1N1	06/28/2009	USA	A/California/VRDL30/2009(H1N1)
PB2	CY052193	H1N1	06/28/2009	USA	A/Texas/42281289/2009(H1N1)
PB2	KT180552	H1N1	06/28/2009	Singapore	A/Singapore/DMS7/2009
PB2	CY064515	H1N1	06/29/2009	USA	A/Boston/116/2009(H1N1)
PB2	CY054794	H1N1	06/29/2009	USA	A/California/VRDL23/2009(H1N1)
PB2	GU136083	H1N1	06/29/2009	Japan	A/Saitama/85/2009(H1N1)
PB2	CY049992	H1N1	06/29/2009	Mexico	A/Mexico City/WR1100N/2009(H1N1)
PB2	CY058187	H1N1	06/29/2009	Nicaragua	A/Managua/3189.01/2009(H1N1)
PB2	CY058203	H1N1	06/29/2009	Nicaragua	A/Managua/1127.02/2009(H1N1)
PB2	CY052406	H1N1	06/29/2009	USA	A/Texas/42291877/2009(H1N1)
PB2	CY063565	H1N1	06/29/2009	USA	A/Boston/152/2009(H1N1)
PB2	CY051654	H1N1	06/30/2009	USA	A/New York/4691/2009(H1N1)
PB2	CY051494	H1N1	06/30/2009	USA	A/Wisconsin/629-S0464/2009(H1N1)
PB2	CY054874	H1N1	06/30/2009	USA	A/California/VRDL34/2009(H1N1)
PB2	CY075322	H1N1	06/30/2009	Chile	A/Chile/3244/2009(H1N1)
PB2	CY075330	H1N1	06/30/2009	Chile	A/Chile/3295/2009(H1N1)
PB2	CY111626	H1N1	06/30/2009	Hong Kong	A/Hong Kong/H090-663-V10/2009
PB2	CY120108	H1N1	06/30/2009	Hong Kong	A/Hong Kong/H090-663-V10/2009
PB2	CY123359	H1N1	06/30/2009	Singapore	A/Singapore/GP875/2009
PB2	CY069754	H1N1	06/30/2009	United Ki	A/England/633/2009
PB2	CY069746	H1N1	06/30/2009	United Ki	A/England/527/2009
PB2	CY064691	H1N1	06/30/2009	USA	A/Boston/142/2009(H1N1)
PB2	GU136066	H1N1	06/30/2009	Japan	A/Hiroshima/216/2009(H1N1)
PB2	CY052209	H1N1	06/30/2009	USA	A/Texas/42303371/2009(H1N1)
PB2	CY058195	H1N1	06/30/2009	Nicaragua	A/Managua/5586.01/2009(H1N1)
PB2	CY075306	H1N1	06/30/2009	Chile	A/Chile/3220/2009(H1N1)
PB2	CY075314	H1N1	06/30/2009	Chile	A/Chile/3242/2009(H1N1)
PB2	JX625392	H1N1	06/30/2009	United Ki	A/England/527/2009
PB2	CY051222	H1N1	06/30/2009	USA	A/Wisconsin/629-D02292/2009(H1N
PB2	CY054802	H1N1	06/30/2009	USA	A/California/VRDL25/2009(H1N1)
PB2	CY047741	H1N1	07/2009	Taiwan	A/Taiwan/526/2009(H1N1)
PB2	CY065171	H1N1	07/01/2009	United Ki	A/England/634/2009
PB2	JX625416	H1N1	07/01/2009	United Ki	A/England/667/2009

PB2	CY051838	H1N1	07/01/2009	USA	A/Texas/43011033/2009(H1N1)
PB2	GQ465660	H1N1	07/01/2009	Canada	A/Canada-SK/RV2486/2009(H1N1)
PB2	CY063525	H1N1	07/01/2009	USA	A/Boston/146/2009(H1N1)
PB2	CY083588	H1N1	07/01/2009	Peru	A/Lima/WRAIR8893F/2009(H1N1)
PB2	KF612037	H1N1	07/01/2009	Bolivia	A/Santa Cruz/12541/2009
PB2	KP637523	H1N1	07/01/2009	Thailand	A/Thailand/SN08270/2009
PB2	CY054914	H1N1	07/01/2009	USA	A/California/VRDL40/2009(H1N1)
PB2	JX625400	H1N1	07/01/2009	United Ki	A/England/685/2009
PB2	GU136065	H1N1	07/01/2009	Japan	A/Hiroshima/207/2009(H1N1)
PB2	CY054938	H1N1	07/01/2009	USA	A/California/VRDL45/2009(H1N1)
PB2	CY054858	H1N1	07/01/2009	USA	A/California/VRDL32/2009(H1N1)
PB2	CY051574	H1N1	07/01/2009	USA	A/New York/4476/2009(H1N1)
PB2	CY063549	H1N1	07/01/2009	USA	A/Boston/149/2009(H1N1)
PB2	CY054954	H1N1	07/01/2009	USA	A/California/VRDL50/2009(H1N1)
PB2	JX625424	H1N1	07/01/2009	United Ki	A/England/92920018/2009
PB2	CY075362	H1N1	07/01/2009	Chile	A/Chile/3375/2009(H1N1)
PB2	CY075346	H1N1	07/01/2009	Chile	A/Chile/3361/2009(H1N1)
PB2	CY051582	H1N1	07/01/2009	USA	A/New York/4500/2009(H1N1)
PB2	CY075354	H1N1	07/01/2009	Chile	A/Chile/3369/2009(H1N1)
PB2	CY075338	H1N1	07/01/2009	Chile	A/Chile/3349/2009(H1N1)
PB2	CY120116	H1N1	07/02/2009	Hong Kong	A/Hong Kong/H090-665-V10/2009
PB2	KF612039	H1N1	07/02/2009	Bolivia	A/Santa Cruz/94841/2009
PB2	CY120124	H1N1	07/02/2009	Hong Kong	A/Hong Kong/H090-667-V10/2009
PB2	CY111634	H1N1	07/02/2009	Hong Kong	A/Hong Kong/H090-665-V10/2009
PB2	CY054866	H1N1	07/02/2009	USA	A/California/VRDL33/2009(H1N1)
PB2	CY120067	H1N1	07/02/2009	Hong Kong	A/Hong Kong/H090-667-V23/2009
PB2	CY072765	H1N1	07/02/2009	Nicaragua	A/Managua/435.01/2009(H1N1)
PB2	CY075378	H1N1	07/02/2009	Chile	A/Chile/3553/2009(H1N1)
PB2	CY072781	H1N1	07/02/2009	Nicaragua	A/Managua/5235.03/2009(H1N1)
PB2	CY124120	H1N1	07/02/2009	Singapore	A/Singapore/ON812/2009
PB2	CY124104	H1N1	07/02/2009	Singapore	A/Singapore/ON809/2009
PB2	CY111642	H1N1	07/02/2009	Hong Kong	A/Hong Kong/H090-667-V10/2009
PB2	CY075370	H1N1	07/02/2009	Chile	A/Chile/3467/2009(H1N1)
PB2	CY054898	H1N1	07/02/2009	USA	A/California/VRDL38/2009(H1N1)
PB2	CY111650	H1N1	07/02/2009	Hong Kong	A/Hong Kong/H090-667-V23/2009
PB2	CY083460	H1N1	07/03/2009	Peru	A/Lima/WRAIR1688P/2009
PB2	CY075386	H1N1	07/03/2009	Chile	A/Chile/3586/2009(H1N1)
PB2	CY083612	H1N1	07/03/2009	Peru	A/Lima/WRAIR9202F/2009(H1N1)
PB2	JX625448	H1N1	07/05/2009	United Ki	A/England/671/2009
PB2	CY054882	H1N1	07/05/2009	USA	A/California/VRDL35/2009(H1N1)
PB2	KT180553	H1N1	07/05/2009	Singapore	A/Singapore/DMS10/2009
PB2	CY122592	H1N1	07/06/2009	Singapore	A/Singapore/GP1094/2009
PB2	CY122600	H1N1	07/06/2009	Singapore	A/Singapore/GP1097/2009
PB2	CY069257	H1N1	07/06/2009	Nicaragua	A/Managua/4943.09/2009(H1N1)
PB2	CY072573	H1N1	07/06/2009	Nicaragua	A/Managua/4197.01/2009(H1N1)
PB2	CY063557	H1N1	07/06/2009	USA	A/Boston/151/2009(H1N1)
PB2	CY075394	H1N1	07/06/2009	Chile	A/Chile/3760/2009(H1N1)
PB2	CY075410	H1N1	07/06/2009	Chile	A/Chile/3819/2009(H1N1)
PB2	CY054930	H1N1	07/06/2009	USA	A/California/VRDL43/2009(H1N1)
PB2	CY122608	H1N1	07/06/2009	Singapore	A/Singapore/GP1099/2009
PB2	CY065163	H1N1	07/06/2009	United Ki	A/England/92860032/2009
PB2	CY075402	H1N1	07/06/2009	Chile	A/Chile/3766/2009(H1N1)
PB2	KP222548	H1N1	07/06/2009	Singapore	A/Singapore/376Y/2009
PB2	KC781694	H1N1	07/06/2009	USA	A/Georgia/08/2009

PB2	GU136082	H1N1	07/07/2009	Japan	A/Niigata/749/2009(H1N1)
PB2	GU136062	H1N1	07/07/2009	Japan	A/Gifu-C/67/2009(H1N1)
PB2	CY088678	H1N1	07/07/2009	India	A/Mum/NIV398/2009(H1N1)
PB2	CY122616	H1N1	07/07/2009	Singapore	A/Singapore/GP1102/2009
PB2	CY075418	H1N1	07/07/2009	Chile	A/Chile/3905/2009(H1N1)
PB2	CY120132	H1N1	07/07/2009	Hong Kong	A/Hong Kong/H090-678-V10/2009
PB2	CY075426	H1N1	07/07/2009	Chile	A/Chile/3935/2009(H1N1)
PB2	CY063541	H1N1	07/07/2009	USA	A/Boston/148/2009(H1N1)
PB2	CY073892	H1N1	07/07/2009	Nicaragua	A/Managua/2729.02/2009(H1N1)
PB2	CY053165	H1N1	07/07/2009	USA	A/Houston/23H/2009(H1N1)
PB2	CY111658	H1N1	07/07/2009	Hong Kong	A/Hong Kong/H090-678-V10/2009
PB2	CY045951	H1N1	07/07/2009	Canada	A/Toronto/T0106/2009(H1N1)
PB2	CY064699	H1N1	07/08/2009	USA	A/Boston/143/2009(H1N1)
PB2	CY051598	H1N1	07/08/2009	USA	A/New York/4565/2009(H1N1)
PB2	CY120140	H1N1	07/08/2009	Hong Kong	A/Hong Kong/H090-681-V10/2009
PB2	CY051614	H1N1	07/08/2009	USA	A/New York/4567/2009(H1N1)
PB2	CY051606	H1N1	07/08/2009	USA	A/New York/4566/2009(H1N1)
PB2	CY122700	H1N1	07/08/2009	Singapore	A/Singapore/GP1146/2009
PB2	CY050197	H1N1	07/08/2009	USA	A/New York/4576/2009(H1N1)
PB2	GU136075	H1N1	07/08/2009	Myanmar	A/Myanmar/JP90/2009(H1N1)
PB2	CY111666	H1N1	07/08/2009	Hong Kong	A/Hong Kong/H090-681-V10/2009
PB2	KT180561	H1N1	07/08/2009	Singapore	A/Singapore/DMS11/2009
PB2	CY095745	H1N1	07/09/2009	China	A/Zhejiang/X2/2009
PB2	GQ392019	H1N1	07/09/2009	Russia	A/Moscow/IIV04/2009(H1N1)
PB2	CY111706	H1N1	07/09/2009	Hong Kong	A/Hong Kong/H090-684-V10/2009
PB2	CY095718	H1N1	07/09/2009	China	A/Zhejiang/X1/2009
PB2	CY095774	H1N1	07/09/2009	China	A/Zhejiang/4/2009
PB2	CY095838	H1N1	07/09/2009	China	A/Zhejiang/7/2009
PB2	CY122776	H1N1	07/09/2009	Singapore	A/Singapore/GP1182/2009
PB2	CY120172	H1N1	07/09/2009	Hong Kong	A/Hong Kong/H090-684-V10/2009
PB2	CY075434	H1N1	07/09/2009	Chile	A/Chile/4064/2009(H1N1)
PB2	CY054906	H1N1	07/09/2009	USA	A/California/VRDL39/2009(H1N1)
PB2	CY095976	H1N1	07/09/2009	China	A/Zhejiang/9/2009
PB2	CY063517	H1N1	07/09/2009	USA	A/Boston/145/2009(H1N1)
PB2	CY066190	H1N1	07/09/2009	USA	A/Boston/144/2009(H1N1)
PB2	CY095814	H1N1	07/09/2009	China	A/Zhejiang/5/2009
PB2	CY095760	H1N1	07/09/2009	China	A/Zhejiang/3/2009
PB2	CY075442	H1N1	07/09/2009	Chile	A/Chile/4181/2009(H1N1)
PB2	KP637795	H1N1	07/09/2009	Thailand	A/Thailand/SS08193/2009
PB2	CY051622	H1N1	07/09/2009	USA	A/New York/4568/2009(H1N1)
PB2	CY051590	H1N1	07/09/2009	USA	A/New York/4556/2009(H1N1)
PB2	KP637715	H1N1	07/09/2009	Thailand	A/Thailand/SR08021/2009
PB2	CY063533	H1N1	07/09/2009	USA	A/Boston/147/2009(H1N1)
PB2	CY120188	H1N1	07/10/2009	Hong Kong	A/Hong Kong/H090-693-V10/2009
PB2	CY075450	H1N1	07/10/2009	Chile	A/Chile/4182/2009(H1N1)
PB2	CY054946	H1N1	07/10/2009	USA	A/California/VRDL48/2009(H1N1)
PB2	CY111674	H1N1	07/10/2009	Hong Kong	A/Hong Kong/H090-681-V22/2009
PB2	CY111722	H1N1	07/10/2009	Hong Kong	A/Hong Kong/H090-693-V10/2009
PB2	CY065179	H1N1	07/10/2009	United Ki	A/England/675/2009
PB2	JX625440	H1N1	07/11/2009	United Ki	A/England/687/2009
PB2	GU136072	H1N1	07/11/2009	Japan	A/Mie/41/2009(H1N1)
PB2	CY054962	H1N1	07/11/2009	USA	A/California/VRDL51/2009(H1N1)
PB2	CY120156	H1N1	07/11/2009	Hong Kong	A/Hong Kong/H090-683-V10/2009
PB2	KT180599	H1N1	07/11/2009	Singapore	A/Singapore/DMS12/2009

PB2	CY106575	H1N1	07/11/2009	Mexico	A/Merida/2189-CIR/2009
PB2	CY111690	H1N1	07/11/2009	Hong Kong	A/Hong Kong/H090-683-V10/2009
PB2	CY054922	H1N1	07/11/2009	USA	A/California/VRDL41/2009(H1N1)
PB2	CY072885	H1N1	07/12/2009	Nicaragua	A/Managua/4606.06/2009(H1N1)
PB2	CY072965	H1N1	07/12/2009	Nicaragua	A/Managua/585.01/2009(H1N1)
PB2	CY052430	H1N1	07/12/2009	USA	A/Texas/43122467/2009(H1N1)
PB2	CY052414	H1N1	07/13/2009	USA	A/Texas/43132503/2009(H1N1)
PB2	GU136087	H1N1	07/13/2009	Japan	A/Wakayama/57/2009(H1N1)
PB2	CY111754	H1N1	07/13/2009	Hong Kong	A/Hong Kong/H090-701-V10/2009
PB2	CY083101	H1N1	07/13/2009	Peru	A/Piura/WRAIR0603F/2009(H1N1)
PB2	JX625456	H1N1	07/13/2009	United Ki	A/England/688/2009
PB2	CY111762	H1N1	07/13/2009	Hong Kong	A/Hong Kong/H090-704-V10/2009
PB2	CY120204	H1N1	07/13/2009	Hong Kong	A/Hong Kong/H090-701-V10/2009
PB2	CY111682	H1N1	07/13/2009	Hong Kong	A/Hong Kong/H090-681-V32/2009
PB2	CY111738	H1N1	07/13/2009	Hong Kong	A/Hong Kong/H090-698-V10/2009
PB2	CY075458	H1N1	07/13/2009	Chile	A/Chile/4257/2009(H1N1)
PB2	KP637651	H1N1	07/13/2009	Thailand	A/Thailand/SP08279/2009
PB2	CY083508	H1N1	07/13/2009	Peru	A/Piura/WRAIR1694P/2009(H1N1)
PB2	CY123371	H1N1	07/13/2009	Singapore	A/Singapore/ON1058/2009
PB2	CY111730	H1N1	07/13/2009	Hong Kong	A/Hong Kong/H090-693-V20/2009
PB2	CY123379	H1N1	07/13/2009	Singapore	A/Singapore/ON1063/2009
PB2	CY120075	H1N1	07/13/2009	Hong Kong	A/Hong Kong/H090-693-V20/2009
PB2	CY120148	H1N1	07/13/2009	Hong Kong	A/Hong Kong/H090-681-V32/2009
PB2	CY120212	H1N1	07/13/2009	Hong Kong	A/Hong Kong/H090-704-V10/2009
PB2	CY073704	H1N1	07/14/2009	Nicaragua	A/Managua/3490.03/2009(H1N1)
PB2	CY051638	H1N1	07/14/2009	USA	A/New York/4620/2009(H1N1)
PB2	CY054986	H1N1	07/14/2009	USA	A/California/VRDL55/2009(H1N1)
PB2	CY111714	H1N1	07/14/2009	Hong Kong	A/Hong Kong/H090-684-V23/2009
PB2	GU136076	H1N1	07/14/2009	Myanmar	A/Myanmar/JP101/2009(H1N1)
PB2	CY120220	H1N1	07/14/2009	Hong Kong	A/Hong Kong/H090-708-V10/2009
PB2	KT180643	H1N1	07/14/2009	Singapore	A/Singapore/DMS13/2009
PB2	CY122784	H1N1	07/14/2009	Singapore	A/Singapore/GP1358/2009
PB2	CY111698	H1N1	07/14/2009	Hong Kong	A/Hong Kong/H090-683-V20/2009
PB2	CY052257	H1N1	07/14/2009	USA	A/Texas/43143450/2009(H1N1)
PB2	CY054970	H1N1	07/14/2009	USA	A/California/VRDL52/2009(H1N1)
PB2	CY051630	H1N1	07/14/2009	USA	A/New York/4607/2009(H1N1)
PB2	CY111770	H1N1	07/14/2009	Hong Kong	A/Hong Kong/H090-708-V10/2009
PB2	CY120180	H1N1	07/14/2009	Hong Kong	A/Hong Kong/H090-684-V23/2009
PB2	CY123411	H1N1	07/14/2009	Singapore	A/Singapore/ON1091/2009
PB2	CY123403	H1N1	07/14/2009	Singapore	A/Singapore/ON1079/2009
PB2	CY120164	H1N1	07/14/2009	Hong Kong	A/Hong Kong/H090-683-V20/2009
PB2	CY123427	H1N1	07/14/2009	Singapore	A/Singapore/ON1102/2009
PB2	CY120228	H1N1	07/15/2009	Hong Kong	A/Hong Kong/H090-712-V10/2009
PB2	CY120236	H1N1	07/15/2009	Hong Kong	A/Hong Kong/H090-712-V14/2009
PB2	CY063573	H1N1	07/15/2009	USA	A/Boston/153/2009(H1N1)
PB2	CY111778	H1N1	07/15/2009	Hong Kong	A/Hong Kong/H090-712-V10/2009
PB2	CY111786	H1N1	07/15/2009	Hong Kong	A/Hong Kong/H090-712-V1_4/2009
PB2	JX625432	H1N1	07/15/2009	United Ki	A/England/92960004/2009
PB2	CY055533	H1N1	07/16/2009	Australia	A/Australia/1/2009(H1N1)
PB2	CY120196	H1N1	07/16/2009	Hong Kong	A/Hong Kong/H090-698-V20/2009
PB2	CY072901	H1N1	07/16/2009	Nicaragua	A/Managua/3214.04/2009(H1N1)
PB2	CY111746	H1N1	07/16/2009	Hong Kong	A/Hong Kong/H090-698-V20/2009
PB2	CY051646	H1N1	07/16/2009	USA	A/New York/4677/2009(H1N1)
PB2	CY083356	H1N1	07/16/2009	USA	A/San Diego/WRAIR1658P/2009

PB2	CY111810	H1N1	07/16/2009	Hong Kong	A/Hong Kong/H090-715-V10/2009
PB2	CY072845	H1N1	07/16/2009	Nicaragua	A/Managua/265.01/2009(H1N1)
PB2	CY075466	H1N1	07/17/2009	Chile	A/Chile/4406/2009(H1N1)
PB2	CY097881	H1N1	07/17/2009	USA	A/South Carolina/WRAIR1645P/2009
PB2	CY055066	H1N1	07/17/2009	Australia	A/Australia/3/2009(H1N1)
PB2	CY055074	H1N1	07/17/2009	Australia	A/Australia/4/2009(H1N1)
PB2	CY111826	H1N1	07/17/2009	Hong Kong	A/Hong Kong/H090-722-V10/2009(H
PB2	CY111818	H1N1	07/17/2009	Hong Kong	A/Hong Kong/H090-721-V10/2009
PB2	CY120260	H1N1	07/17/2009	Hong Kong	A/Hong Kong/H090-721-V10/2009
PB2	JX625464	H1N1	07/17/2009	United Ki	A/England/92980479/2009
PB2	CY120268	H1N1	07/17/2009	Hong Kong	A/Hong Kong/H090-722-V10/2009
PB2	CY083277	H1N1	07/17/2009	USA	A/South Carolina/WRAIR1645P/2009
PB2	KT180652	H1N1	07/17/2009	Singapore	A/Singapore/DMS14/2009
PB2	GU136086	H1N1	07/18/2009	Japan	A/Tokushima/2/2009(H1N1)
PB2	CY120244	H1N1	07/18/2009	Hong Kong	A/Hong Kong/H090-712-V20/2009
PB2	CY120252	H1N1	07/18/2009	Hong Kong	A/Hong Kong/H090-712-V24/2009
PB2	CY111794	H1N1	07/18/2009	Hong Kong	A/Hong Kong/H090-712-V20/2009
PB2	CY083636	H1N1	07/18/2009	USA	A/San Diego/WRAIR1657P/2009
PB2	CY111802	H1N1	07/18/2009	Hong Kong	A/Hong Kong/H090-712-V24/2009
PB2	CY055082	H1N1	07/18/2009	Australia	A/Australia/6/2009(H1N1)
PB2	CY055541	H1N1	07/18/2009	Australia	A/Australia/7/2009(H1N1)
PB2	GU433032	H1N1	07/19/2009	Russia	A/Tomsk/03/2009(H1N1)
PB2	CY083125	H1N1	07/19/2009	Peru	A/Lima/WRAIR0672F/2009
PB2	KP637395	H1N1	07/20/2009	Thailand	A/Thailand/MR08208/2009
PB2	CY052382	H1N1	07/20/2009	USA	A/Texas/43200999/2009(H1N1)
PB2	CY055549	H1N1	07/20/2009	Australia	A/Australia/9/2009(H1N1)
PB2	CY055596	H1N1	07/20/2009	Australia	A/Australia/15/2009(H1N1)
PB2	CY055557	H1N1	07/20/2009	Australia	A/Australia/10/2009(H1N1)
PB2	CY055564	H1N1	07/20/2009	Australia	A/Australia/11/2009(H1N1)
PB2	CY055588	H1N1	07/20/2009	Australia	A/Australia/14/2009(H1N1)
PB2	CY055572	H1N1	07/20/2009	Australia	A/Australia/12/2009(H1N1)
PB2	CY071006	H1N1	07/20/2009	Guam	A/Guam/NHRC0030/2009(H1N1)
PB2	CY055580	H1N1	07/20/2009	Australia	A/Australia/13/2009(H1N1)
PB2	CY075474	H1N1	07/20/2009	Chile	A/Chile/4438/2009(H1N1)
PB2	CY092192	H1N1	07/21/2009	Australia	A/Australia/17/2009
PB2	CY069057	H1N1	07/21/2009	Guam	A/Guam/NHRC0021/2009(H1N1)
PB2	CY083205	H1N1	07/21/2009	USA	A/New Jersey/WRAIR1504P/2009(H1N
PB2	CY055604	H1N1	07/21/2009	Australia	A/Australia/16/2009(H1N1)
PB2	CY054994	H1N1	07/21/2009	USA	A/California/VRDL58/2009(H1N1)
PB2	HM569674	H1N1	07/21/2009	Argentina	A/Argentina/08AR/2009(H1N1)
PB2	CY055284	H1N1	07/21/2009	Malaysia	A/Malaysia/5259/2009(H1N1)
PB2	CY055292	H1N1	07/21/2009	Malaysia	A/Malaysia/5259/2009(H1N1)
PB2	CY055620	H1N1	07/21/2009	Australia	A/Australia/21/2009(H1N1)
PB2	CY072861	H1N1	07/21/2009	Nicaragua	A/Managua/3438.02/2009(H1N1)
PB2	CY055636	H1N1	07/21/2009	Australia	A/Australia/24/2009(H1N1)
PB2	CY054978	H1N1	07/21/2009	USA	A/California/VRDL53/2009(H1N1)
PB2	CY055612	H1N1	07/21/2009	Australia	A/Australia/20/2009(H1N1)
PB2	CY068977	H1N1	07/21/2009	Guam	A/Guam/NHRC0011/2009(H1N1)
PB2	CY069265	H1N1	07/21/2009	Nicaragua	A/Managua/5364.01/2009(H1N1)
PB2	CY069041	H1N1	07/21/2009	Guam	A/Guam/NHRC0019/2009(H1N1)
PB2	CY069097	H1N1	07/21/2009	Guam	A/Guam/NHRC0026/2009(H1N1)
PB2	CY068969	H1N1	07/21/2009	Guam	A/Guam/NHRC0010/2009(H1N1)
PB2	CY111834	H1N1	07/21/2009	Hong Kong	A/Hong Kong/H090-722-V20/2009(H
PB2	KC620383	H1N1	07/21/2009	Germany	A/Giessen/6/2009

PB2	GU433040	H1N1	07/21/2009	Russia	A/Novosibirsk/02/2009(H1N1)
PB2	GU136073	H1N1	07/21/2009	Japan	A/Mie/52/2009(H1N1)
PB2	KC222633	H1N1	07/22/2009	Germany	A/Jena/VI5258/2009(H1N1)
PB2	CY055628	H1N1	07/22/2009	Australia	A/Australia/23/2009(H1N1)
PB2	KJ690421	H1N1	07/22/2009	Uganda	A/Uganda/MUWRP-059/2009
PB2	CY055652	H1N1	07/22/2009	Australia	A/Australia/26/2009(H1N1)
PB2	CY069033	H1N1	07/22/2009	Guam	A/Guam/NHRC0018/2009(H1N1)
PB2	CY073648	H1N1	07/22/2009	Nicaragua	A/Managua/1973.02/2009(H1N1)
PB2	CY073656	H1N1	07/22/2009	Nicaragua	A/Managua/4590.03/2009(H1N1)
PB2	KP222549	H1N1	07/22/2009	Singapore	A/Singapore/384P/2009
PB2	GU136078	H1N1	07/22/2009	Myanmar	A/Myanmar/JP133/2009(H1N1)
PB2	KX013002	H1N1	07/22/2009	Germany	A/Jena/MSV-VI5258/2009
PB2	KJ549775	H1N1	07/22/2009	Germany	A/Jena/5258/2009
PB2	CY055644	H1N1	07/22/2009	Australia	A/Australia/25/2009(H1N1)
PB2	CY068945	H1N1	07/22/2009	Guam	A/Guam/NHRC0008/2009(H1N1)
PB2	CY073624	H1N1	07/22/2009	Nicaragua	A/Managua/4747.01/2009(H1N1)
PB2	CY055660	H1N1	07/22/2009	Australia	A/Australia/27/2009(H1N1)
PB2	GQ496139	H1N1	07/22/2009	Russia	A/Vladivostok/IIV17/2009(H1N1)
PB2	GU136077	H1N1	07/22/2009	Myanmar	A/Myanmar/JP131/2009(H1N1)
PB2	CY056246	H1N1	07/23/2009	Australia	A/Australia/28/2009(H1N1)
PB2	CY068921	H1N1	07/23/2009	Japan	A/Japan/NHRC0003/2009(H1N1)
PB2	CY065959	H1N1	07/23/2009	Japan	A/Japan/NHRC0001/2009(H1N1)
PB2	KT180661	H1N1	07/23/2009	Singapore	A/Singapore/DMS16/2009
PB2	CY073600	H1N1	07/23/2009	Nicaragua	A/Managua/5295.02/2009(H1N1)
PB2	CY119361	H1N1	07/23/2009	Malaysia	A/Malaysia/2050837/2009
PB2	JX625495	H1N1	07/23/2009	United Ki	A/England/93760756/2009
PB2	CY068985	H1N1	07/23/2009	Guam	A/Guam/NHRC0012/2009(H1N1)
PB2	CY055675	H1N1	07/23/2009	Australia	A/Australia/29/2009(H1N1)
PB2	CY055683	H1N1	07/23/2009	Australia	A/Australia/31/2009(H1N1)
PB2	CY065943	H1N1	07/23/2009	Guam	A/Guam/NHRC0002/2009(H1N1)
PB2	CY065967	H1N1	07/23/2009	Japan	A/Japan/NHRC0002/2009(H1N1)
PB2	CY055715	H1N1	07/24/2009	Australia	A/Australia/37/2009(H1N1)
PB2	CY069289	H1N1	07/24/2009	Nicaragua	A/Managua/3467.01/2009(H1N1)
PB2	CY051662	H1N1	07/24/2009	USA	A/New York/4728/2009(H1N1)
PB2	KP637451	H1N1	07/24/2009	Thailand	A/Thailand/PY08016/2009
PB2	KP637659	H1N1	07/24/2009	Thailand	A/Thailand/SP08294/2009
PB2	CY055723	H1N1	07/24/2009	Australia	A/Australia/39/2009(H1N1)
PB2	CY120284	H1N1	07/24/2009	Hong Kong	A/Hong Kong/H090-742-V10/2009
PB2	CY120276	H1N1	07/24/2009	Hong Kong	A/Hong Kong/H090-738-V10/2009
PB2	CY128090	H1N1	07/24/2009	Viet Nam	A/Viet Nam/811/2009
PB2	CY128082	H1N1	07/24/2009	Viet Nam	A/Viet Nam/817/2009
PB2	CY069297	H1N1	07/24/2009	Nicaragua	A/Managua/4951.02/2009(H1N1)
PB2	CY072581	H1N1	07/24/2009	Nicaragua	A/Managua/3231.02/2009(H1N1)
PB2	CY055731	H1N1	07/24/2009	Australia	A/Australia/40/2009(H1N1)
PB2	CY111842	H1N1	07/24/2009	Hong Kong	A/Hong Kong/H090-738-V10/2009
PB2	CY111850	H1N1	07/24/2009	Hong Kong	A/Hong Kong/H090-742-V10/2009(H
PB2	GQ496152	H1N1	07/24/2009	Russia	A/Vladivostok/IIV18/2009(H1N1)
PB2	CY055699	H1N1	07/24/2009	Australia	A/Australia/35/2009(H1N1)
PB2	CY055707	H1N1	07/24/2009	Australia	A/Australia/36/2009(H1N1)
PB2	CY051830	H1N1	07/24/2009	USA	A/Texas/43242018/2009(H1N1)
PB2	CY128098	H1N1	07/25/2009	Viet Nam	A/Viet Nam/818/2009
PB2	KC782197	H1N1	07/25/2009	USA	A/Utah/20/2009
PB2	CY069273	H1N1	07/25/2009	Nicaragua	A/Managua/1264.03/2009(H1N1)
PB2	CY055739	H1N1	07/25/2009	Australia	A/Australia/42/2009(H1N1)

PB2	CY088692	H1N1	07/25/2009	India	A/Pune/NIV759/2009
PB2	CY051670	H1N1	07/25/2009	USA	A/New York/4735/2009(H1N1)
PB2	CY128114	H1N1	07/26/2009	Viet Nam	A/Viet Nam/823/2009
PB2	CY055755	H1N1	07/26/2009	Australia	A/Australia/44/2009(H1N1)
PB2	KT180665	H1N1	07/26/2009	Singapore	A/Singapore/DMS17/2009
PB2	CY055747	H1N1	07/26/2009	Australia	A/Australia/43/2009(H1N1)
PB2	CY128106	H1N1	07/27/2009	Viet Nam	A/Viet Nam/835/2009
PB2	CY111866	H1N1	07/27/2009	Hong Kong	A/Hong Kong/H090-745-V10/2009(H
PB2	CY055779	H1N1	07/27/2009	Australia	A/Australia/48/2009(H1N1)
PB2	CY065935	H1N1	07/27/2009	Guam	A/Guam/NHRC0001/2009(H1N1)
PB2	CY073536	H1N1	07/27/2009	Nicaragua	A/Managua/466.01/2009(H1N1)
PB2	JQ173145	H1N1	07/27/2009	Finland	A/Helsinki/P14/2009
PB2	KP222551	H1N1	07/27/2009	Singapore	A/Singapore/386T/2009
PB2	KP222550	H1N1	07/27/2009	Singapore	A/Singapore/385K/2009
PB2	CY052374	H1N1	07/27/2009	USA	A/Texas/43272683/2009(H1N1)
PB2	CY120091	H1N1	07/27/2009	Hong Kong	A/Hong Kong/H090-745-V10/2009
PB2	CY055002	H1N1	07/27/2009	USA	A/California/VRDL61/2009(H1N1)
PB2	CY055763	H1N1	07/27/2009	Australia	A/Australia/45/2009(H1N1)
PB2	JN187154	H1N1	07/27/2009	Taiwan	A/Taiwan/2826/2009
PB2	CY128130	H1N1	07/27/2009	Viet Nam	A/Viet Nam/841/2009
PB2	CY072733	H1N1	07/27/2009	Nicaragua	A/Managua/4407.02/2009(H1N1)
PB2	CY072629	H1N1	07/27/2009	Nicaragua	A/Managua/5339.04/2009(H1N1)
PB2	CY073664	H1N1	07/27/2009	Nicaragua	A/Managua/3225.03/2009(H1N1)
PB2	CY055771	H1N1	07/27/2009	Australia	A/Australia/47/2009(H1N1)
PB2	CY128122	H1N1	07/28/2009	Viet Nam	A/Viet Nam/850/2009
PB2	KP222553	H1N1	07/28/2009	Singapore	A/Singapore/392N/2009
PB2	CY111882	H1N1	07/28/2009	Hong Kong	A/Hong Kong/H090-746-V10/2009(H
PB2	CY083237	H1N1	07/28/2009	USA	A/Virginia/WRAIR1511P/2009
PB2	KP222552	H1N1	07/28/2009	Singapore	A/Singapore/390T/2009
PB2	CY055787	H1N1	07/28/2009	Australia	A/Australia/51/2009(H1N1)
PB2	CY073640	H1N1	07/28/2009	Nicaragua	A/Managua/4960.01/2009(H1N1)
PB2	CY120300	H1N1	07/28/2009	Hong Kong	A/Hong Kong/H090-746-V10/2009
PB2	CY119537	H1N1	07/28/2009	Malaysia	A/Malaysia/2072040/2009
PB2	CY111858	H1N1	07/28/2009	Hong Kong	A/Hong Kong/H090-742-V33/2009
PB2	CY120292	H1N1	07/28/2009	Hong Kong	A/Hong Kong/H090-742-V33/2009
PB2	CY051678	H1N1	07/28/2009	USA	A/New York/4738/2009(H1N1)
PB2	CY055795	H1N1	07/29/2009	Australia	A/Australia/53/2009(H1N1)
PB2	CY052289	H1N1	07/29/2009	USA	A/Texas/43292238/2009(H1N1)
PB2	CY111898	H1N1	07/29/2009	Hong Kong	A/Hong Kong/H090-751-V10/2009
PB2	CY073560	H1N1	07/29/2009	Nicaragua	A/Managua/2978.04/2009(H1N1)
PB2	KP222555	H1N1	07/29/2009	Singapore	A/Singapore/395T/2009
PB2	KP222554	H1N1	07/29/2009	Singapore	A/Singapore/394T/2009
PB2	KT180666	H1N1	07/29/2009	Singapore	A/Singapore/DMS18/2009
PB2	CY122792	H1N1	07/29/2009	Singapore	A/Singapore/GP2215/2009
PB2	CY120308	H1N1	07/29/2009	Hong Kong	A/Hong Kong/H090-751-V10/2009
PB2	CY128458	H1N1	07/29/2009	Viet Nam	A/Viet Nam/001-1880/2009
PB2	CY065951	H1N1	07/29/2009	Guam	A/Guam/NHRC0003/2009(H1N1)
PB2	CY083221	H1N1	07/29/2009	USA	A/California/WRAIR1507P/2009
PB2	CY072637	H1N1	07/30/2009	Nicaragua	A/Managua/1836.02/2009(H1N1)
PB2	CY111954	H1N1	07/30/2009	Hong Kong	A/Hong Kong/H090-758-V10/2009
PB2	CY111946	H1N1	07/30/2009	Hong Kong	A/Hong Kong/H090-757-V10/2009
PB2	CY111962	H1N1	07/30/2009	Hong Kong	A/Hong Kong/H090-759-V10/2009
PB2	CY120372	H1N1	07/30/2009	Hong Kong	A/Hong Kong/H090-759-V10/2009
PB2	CY122876	H1N1	07/30/2009	Singapore	A/Singapore/GP2315/2009

PB2	CY120364	H1N1	07/30/2009	Hong Kong	A/Hong Kong/H090-758-V10/2009
PB2	CY122884	H1N1	07/30/2009	Singapore	A/Singapore/GP2317/2009
PB2	CY051686	H1N1	07/30/2009	USA	A/New York/4747/2009(H1N1)
PB2	KP637323	H1N1	07/30/2009	Thailand	A/Thailand/KS08277/2009
PB2	KY924983	H1N1	07/30/2009	Brazil	A/Porto Alegre/LACENRS-1771/2009
PB2	KM013753	H1N1	07/30/2009	Thailand	A/Bangkok/SIMI507/2009
PB2	CY055811	H1N1	07/30/2009	Australia	A/Australia/56/2009(H1N1)
PB2	KM013747	H1N1	07/30/2009	Thailand	A/Bangkok/SIMI501/2009
PB2	CY120356	H1N1	07/30/2009	Hong Kong	A/Hong Kong/H090-757-V10/2009
PB2	CY111890	H1N1	07/30/2009	Hong Kong	A/Hong Kong/H090-746-V20/2009
PB2	CY111906	H1N1	07/31/2009	Hong Kong	A/Hong Kong/H090-751-V20/2009(H1
PB2	CY111986	H1N1	07/31/2009	Hong Kong	A/Hong Kong/H090-765-V10/2009
PB2	CY070990	H1N1	07/31/2009	Guam	A/Guam/NHRC0007/2009(H1N1)
PB2	CY068937	H1N1	07/31/2009	Guam	A/Guam/NHRC0005/2009(H1N1)
PB2	CY083213	H1N1	07/31/2009	USA	A/New Jersey/WRAIR1506P/2009(H1N
PB2	CY120380	H1N1	07/31/2009	Hong Kong	A/Hong Kong/H090-761-V10/2009
PB2	CY120396	H1N1	07/31/2009	Hong Kong	A/Hong Kong/H090-765-V10/2009
PB2	CY120316	H1N1	07/31/2009	Hong Kong	A/Hong Kong/H090-751-V20/2009
PB2	CY120332	H1N1	07/31/2009	Hong Kong	A/Hong Kong/H090-751-V24/2009
PB2	CY119369	H1N1	07/31/2009	Malaysia	A/Malaysia/2068631/2009
PB2	CY120388	H1N1	07/31/2009	Hong Kong	A/Hong Kong/H090-762-V10/2009
PB2	KY926114	H1N1	07/31/2009	Brazil	A/Igrejinha/LACENRS-1803/2009
PB2	CY111978	H1N1	07/31/2009	Hong Kong	A/Hong Kong/H090-762-V10/2009
PB2	KP222558	H1N1	07/31/2009	Singapore	A/Singapore/410W/2009
PB2	KP222561	H1N1	07/31/2009	Singapore	A/Singapore/418W/2009
PB2	KP222556	H1N1	07/31/2009	Singapore	A/Singapore/405S/2009
PB2	CY120324	H1N1	07/31/2009	Hong Kong	A/Hong Kong/H090-751-V23/2009
PB2	KP222559	H1N1	07/31/2009	Singapore	A/Singapore/413Y/2009
PB2	KY925578	H1N1	07/31/2009	Brazil	A/Porto Alegre/LACENRS-1786/2009
PB2	CY120746	H1N1	07/31/2009	Brazil	A/Brazil/AVS07/2009
PB2	CY111970	H1N1	07/31/2009	Hong Kong	A/Hong Kong/H090-761-V10/2009
PB2	KY925361	H1N1	07/31/2009	Brazil	A/Estrela/LACENRS-313/2009
PB2	CY111914	H1N1	07/31/2009	Hong Kong	A/Hong Kong/H090-751-V23/2009
PB2	KP222560	H1N1	07/31/2009	Singapore	A/Singapore/416C/2009
PB2	KP222557	H1N1	07/31/2009	Singapore	A/Singapore/406K/2009
PB2	CY111874	H1N1	07/31/2009	Hong Kong	A/Hong Kong/H090-745-V24/2009
PB2	CY111922	H1N1	07/31/2009	Hong Kong	A/Hong Kong/H090-751-V24/2009(H1
PB2	CY055827	H1N1	08/01/2009	Australia	A/Australia/58/2009(H1N1)
PB2	KC780432	H1N1	08/01/2009	USA	A/Texas/71/2009
PB2	CY055819	H1N1	08/01/2009	Australia	A/Australia/57/2009(H1N1)
PB2	KT180667	H1N1	08/01/2009	Singapore	A/Singapore/DMS19/2009
PB2	CY120340	H1N1	08/02/2009	Hong Kong	A/Hong Kong/H090-751-V31/2009
PB2	CY055835	H1N1	08/02/2009	Australia	A/Australia/59/2009(H1N1)
PB2	CY111930	H1N1	08/02/2009	Hong Kong	A/Hong Kong/H090-751-V31/2009(H1
PB2	CY111938	H1N1	08/02/2009	Hong Kong	A/Hong Kong/H090-751-V34/2009
PB2	CY119377	H1N1	08/02/2009	Malaysia	A/Malaysia/2076204/2009
PB2	CY120348	H1N1	08/02/2009	Hong Kong	A/Hong Kong/H090-751-V34/2009
PB2	CY118207	H1N1	08/02/2009	Malaysia	A/Malaysia/2076212/2009
PB2	CY055843	H1N1	08/02/2009	Australia	A/Australia/60/2009(H1N1)
PB2	CY055859	H1N1	08/03/2009	Australia	A/Australia/63/2009(H1N1)
PB2	CY055867	H1N1	08/03/2009	Australia	A/Australia/64/2009(H1N1)
PB2	CY112002	H1N1	08/03/2009	Hong Kong	A/Hong Kong/H090-771-V10/2009
PB2	CY055851	H1N1	08/03/2009	Australia	A/Australia/61/2009(H1N1)
PB2	CY055875	H1N1	08/03/2009	Australia	A/Australia/65/2009(H1N1)

PB2	CY119393	H1N1	08/03/2009	Malaysia	A/Malaysia/2077462/2009
PB2	KP222563	H1N1	08/03/2009	Singapore	A/Singapore/420T/2009
PB2	CY120412	H1N1	08/03/2009	Hong Kong	A/Hong Kong/H090-771-V10/2009
PB2	CY072565	H1N1	08/03/2009	Nicaragua	A/Managua/451.01/2009(H1N1)
PB2	JX625472	H1N1	08/03/2009	United Ki	A/England/646/2009
PB2	KP222564	H1N1	08/03/2009	Singapore	A/Singapore/426N/2009
PB2	CY072685	H1N1	08/03/2009	Nicaragua	A/Managua/209.01/2009(H1N1)
PB2	KP222567	H1N1	08/03/2009	Singapore	A/Singapore/441T/2009
PB2	CY106983	H1N1	08/03/2009	Hong Kong	A/Hong Kong/H090-770-V10/2009
PB2	KP222565	H1N1	08/03/2009	Singapore	A/Singapore/437Y/2009
PB2	CY119385	H1N1	08/03/2009	Malaysia	A/Malaysia/2076039/2009
PB2	KP222566	H1N1	08/03/2009	Singapore	A/Singapore/440F/2009
PB2	KT180668	H1N1	08/04/2009	Singapore	A/Singapore/DMS20/2009
PB2	KP222569	H1N1	08/04/2009	Singapore	A/Singapore/444W/2009
PB2	CY112026	H1N1	08/04/2009	Hong Kong	A/Hong Kong/H090-774-V10/2009
PB2	CY120428	H1N1	08/04/2009	Hong Kong	A/Hong Kong/H090-774-V10/2009
PB2	CY070982	H1N1	08/04/2009	Guam	A/Guam/NHRC0006/2009(H1N1)
PB2	CY055883	H1N1	08/04/2009	Australia	A/Australia/66/2009(H1N1)
PB2	CY068953	H1N1	08/04/2009	Japan	A/Japan/NHRC0004/2009(H1N1)
PB2	CY073608	H1N1	08/04/2009	Nicaragua	A/Managua/4451.02/2009(H1N1)
PB2	CY073616	H1N1	08/04/2009	Nicaragua	A/Managua/1256.04/2009(H1N1)
PB2	CY119529	H1N1	08/04/2009	Malaysia	A/Malaysia/2076756/2009
PB2	CY120444	H1N1	08/05/2009	Hong Kong	A/Hong Kong/H090-779-V12/2009
PB2	CY055891	H1N1	08/05/2009	Australia	A/Australia/69/2009(H1N1)
PB2	CY055042	H1N1	08/05/2009	USA	A/California/VRDL69/2009(H1N1)
PB2	CY112042	H1N1	08/05/2009	Hong Kong	A/Hong Kong/H090-779-V12/2009
PB2	CY120420	H1N1	08/05/2009	Hong Kong	A/Hong Kong/H090-772-V10/2009
PB2	CY073576	H1N1	08/05/2009	Nicaragua	A/Managua/5230.01/2009(H1N1)
PB2	CY071495	H1N1	08/05/2009	USA	A/California/WR1316P/2009(H1N1)
PB2	CY072605	H1N1	08/05/2009	Nicaragua	A/Managua/1077.04/2009(H1N1)
PB2	CY112010	H1N1	08/05/2009	Hong Kong	A/Hong Kong/H090-771-V20/2009
PB2	CY112018	H1N1	08/05/2009	Hong Kong	A/Hong Kong/H090-772-V10/2009
PB2	CY120436	H1N1	08/05/2009	Hong Kong	A/Hong Kong/H090-779-V10/2009
PB2	KJ690500	H1N1	08/05/2009	Uganda	A/Uganda/MUWRP-067/2009
PB2	KY926041	H1N1	08/05/2009	Brazil	A/Teutonia/LACENRS-711/2009
PB2	KY925585	H1N1	08/05/2009	Brazil	A/Nonoai/LACENRS-714/2009
PB2	KJ690405	H1N1	08/05/2009	Uganda	A/Uganda/MUWRP-066/2009
PB2	CY119401	H1N1	08/05/2009	Malaysia	A/Malaysia/2082543/2009
PB2	CY112034	H1N1	08/05/2009	Hong Kong	A/Hong Kong/H090-779-V10/2009
PB2	CY068929	H1N1	08/06/2009	Guam	A/Guam/NHRC0004/2009(H1N1)
PB2	CY111994	H1N1	08/06/2009	Hong Kong	A/Hong Kong/H090-770-V22/2009
PB2	KP222570	H1N1	08/06/2009	Singapore	A/Singapore/448D/2009
PB2	JN596865	H1N1	08/06/2009	Russia	A/Orenburg/01/2009(H1N1)
PB2	CY055899	H1N1	08/06/2009	Australia	A/Australia/70/2009(H1N1)
PB2	JX625480	H1N1	08/06/2009	United Ki	A/England/93320016/2009
PB2	CY051694	H1N1	08/06/2009	USA	A/New York/4761/2009(H1N1)
PB2	CY120404	H1N1	08/06/2009	Hong Kong	A/Hong Kong/H090-770-V22/2009
PB2	CY071527	H1N1	08/07/2009	USA	A/California/WR1320P/2009(H1N1)
PB2	CY120468	H1N1	08/07/2009	Hong Kong	A/Hong Kong/H090-785-V10/2009
PB2	CY119409	H1N1	08/07/2009	Malaysia	A/Malaysia/2086505/2009
PB2	CY072621	H1N1	08/07/2009	Nicaragua	A/Managua/2604.02/2009(H1N1)
PB2	KT180669	H1N1	08/07/2009	Singapore	A/Singapore/DMS21/2009
PB2	CY112074	H1N1	08/07/2009	Hong Kong	A/Hong Kong/H090-785-V10/2009
PB2	CY073528	H1N1	08/07/2009	Nicaragua	A/Managua/5399.04/2009(H1N1)

PB2	CY112066	H1N1	08/08/2009	Hong Kong	A/Hong Kong/H090-779-V22/2009
PB2	CY120460	H1N1	08/08/2009	Hong Kong	A/Hong Kong/H090-779-V22/2009
PB2	CY073632	H1N1	08/08/2009	Nicaragua	A/Managua/5595.03/2009(H1N1)
PB2	CY112050	H1N1	08/08/2009	Hong Kong	A/Hong Kong/H090-779-V20/2009
PB2	CY073592	H1N1	08/08/2009	Nicaragua	A/Managua/4064.01/2009(H1N1)
PB2	CY120452	H1N1	08/08/2009	Hong Kong	A/Hong Kong/H090-779-V21/2009
PB2	CY120476	H1N1	08/08/2009	Hong Kong	A/Hong Kong/H090-787-V10/2009
PB2	CY112082	H1N1	08/08/2009	Hong Kong	A/Hong Kong/H090-787-V10/2009
PB2	CY112058	H1N1	08/08/2009	Hong Kong	A/Hong Kong/H090-779-V21/2009
PB2	KC781729	H1N1	08/09/2009	USA	A/Oregon/30/2009
PB2	CY119417	H1N1	08/09/2009	Malaysia	A/Malaysia/2089841/2009
PB2	KC781744	H1N1	08/09/2009	USA	A/Wyoming/08/2009
PB2	CY060539	H1N1	08/09/2009	Canada	A/Ontario/25389/2009
PB2	CY119425	H1N1	08/09/2009	Malaysia	A/Malaysia/2089888/2009
PB2	CY072693	H1N1	08/10/2009	Nicaragua	A/Managua/554.01/2009(H1N1)
PB2	CY073552	H1N1	08/10/2009	Nicaragua	A/Managua/3083.01/2009(H1N1)
PB2	CY055010	H1N1	08/10/2009	USA	A/California/VRDL64/2009(H1N1)
PB2	CY055018	H1N1	08/10/2009	USA	A/California/VRDL65/2009(H1N1)
PB2	KT180670	H1N1	08/10/2009	Singapore	A/Singapore/DMS22/2009
PB2	KY935465	H1N1	08/10/2009	Brazil	A/Rio Grande/LACENRS-1406/2009
PB2	HM014331	H1N1	08/10/2009	China	A/Guangzhou/GIRD07/2009(H1N1)
PB2	JN596869	H1N1	08/10/2009	Russia	A/Kurgan/01/2009
PB2	CY119441	H1N1	08/10/2009	Malaysia	A/Malaysia/2098044/2009
PB2	CY119449	H1N1	08/10/2009	Malaysia	A/Malaysia/2098053/2009
PB2	CY119433	H1N1	08/10/2009	Malaysia	A/Malaysia/2098001/2009
PB2	CY055907	H1N1	08/10/2009	Australia	A/Australia/72/2009(H1N1)
PB2	CY071503	H1N1	08/10/2009	USA	A/California/WR1317P/2009(H1N1)
PB2	CY055501	H1N1	08/10/2009	USA	A/California/VRDL74/2009(H1N1)
PB2	CY072653	H1N1	08/11/2009	Nicaragua	A/Managua/2275.01/2009(H1N1)
PB2	CY073568	H1N1	08/11/2009	Nicaragua	A/Managua/5119.06/2009(H1N1)
PB2	CY055034	H1N1	08/11/2009	USA	A/California/VRDL68/2009(H1N1)
PB2	CY073544	H1N1	08/11/2009	Nicaragua	A/Managua/4012.01/2009(H1N1)
PB2	CY118227	H1N1	08/11/2009	Malaysia	A/Malaysia/2098038/2009
PB2	CY119457	H1N1	08/11/2009	Malaysia	A/Malaysia/2097717/2009
PB2	CY115869	H1N1	08/11/2009	Netherlan	A/Netherlands/947b/2009
PB2	CY123503	H1N1	08/11/2009	Singapore	A/Singapore/ON1760/2009
PB2	CY069281	H1N1	08/11/2009	Nicaragua	A/Managua/5212.04/2009(H1N1)
PB2	CY072597	H1N1	08/11/2009	Nicaragua	A/Managua/1356.03/2009(H1N1)
PB2	CY072669	H1N1	08/11/2009	Nicaragua	A/Managua/2339.03/2009(H1N1)
PB2	CY123491	H1N1	08/11/2009	Singapore	A/Singapore/ON1747/2009
PB2	CY060515	H1N1	08/11/2009	Canada	A/Ontario/222656/2009
PB2	CY072709	H1N1	08/11/2009	Nicaragua	A/Managua/5036.01/2009(H1N1)
PB2	CY112090	H1N1	08/12/2009	Hong Kong	A/Hong Kong/H090-793-V10/2009
PB2	CY055915	H1N1	08/12/2009	Australia	A/Australia/73/2009(H1N1)
PB2	KY925198	H1N1	08/12/2009	Brazil	A/Alegrete/LACENRS-1453/2009
PB2	CY128138	H1N1	08/12/2009	Viet Nam	A/Viet Nam/13032001/2009
PB2	KY926232	H1N1	08/12/2009	Brazil	A/Porto Alegre/LACENRS-1598/2009
PB2	CY083229	H1N1	08/12/2009	Guam	A/Guam/WRAIR1510P/2009
PB2	JN596866	H1N1	08/12/2009	Russia	A/Omsk/01/2009
PB2	CY120722	H1N1	08/12/2009	Brazil	A/Brazil/AVS03/2009
PB2	CY120484	H1N1	08/12/2009	Hong Kong	A/Hong Kong/H090-793-V10/2009
PB2	CY128146	H1N1	08/12/2009	Viet Nam	A/Viet Nam/13032002/2009
PB2	CY072725	H1N1	08/12/2009	Nicaragua	A/Managua/3359.01/2009(H1N1)
PB2	CY072589	H1N1	08/12/2009	Nicaragua	A/Managua/4186.02/2009(H1N1)

PB2	KY926166	H1N1	08/13/2009	Brazil	A/Santo Angelo/LACENRS-1537/2009
PB2	CY072997	H1N1	08/13/2009	Nicaragua	A/Managua/527.04/2009(H1N1)
PB2	KY925654	H1N1	08/13/2009	Brazil	A/Vacaria/LACENRS-1720/2009
PB2	KY925526	H1N1	08/13/2009	Brazil	A/Venancio Aires/LACENRS-1539/20
PB2	CY073584	H1N1	08/13/2009	Nicaragua	A/Managua/921.02/2009(H1N1)
PB2	CY120730	H1N1	08/13/2009	Brazil	A/Brazil/AVS04/2009
PB2	CY072645	H1N1	08/13/2009	Nicaragua	A/Managua/3642.04/2009(H1N1)
PB2	KT180672	H1N1	08/13/2009	Singapore	A/Singapore/DMS23/2009
PB2	CY051702	H1N1	08/14/2009	USA	A/New York/4777/2009(H1N1)
PB2	KP637827	H1N1	08/14/2009	Thailand	A/Thailand/IY07083/2009
PB2	CY055268	H1N1	08/14/2009	Malaysia	A/Malaysia/10226/2009(H1N1)
PB2	KY926135	H1N1	08/14/2009	Brazil	A/Pelotas/LACENRS-1658/2009
PB2	KY925445	H1N1	08/14/2009	Brazil	A/Triunfo/LACENRS-1611/2009
PB2	KY925059	H1N1	08/14/2009	Brazil	A/Sao Gabriel/LACENRS-1626/2009
PB2	CY112098	H1N1	08/14/2009	Hong Kong	A/Hong Kong/H090-793-V20/2009
PB2	CY120492	H1N1	08/14/2009	Hong Kong	A/Hong Kong/H090-793-V20/2009
PB2	CY122964	H1N1	08/14/2009	Singapore	A/Singapore/GP2982/2009
PB2	CY058251	H1N1	08/15/2009	Nicaragua	A/Managua/3275.01/2009(H1N1)
PB2	CY058611	H1N1	08/15/2009	Nicaragua	A/Managua/5747.02/2009(H1N1)
PB2	KY926015	H1N1	08/15/2009	Brazil	A/Santo Antonio da Patrulha/LACI
PB2	CY052878	H1N1	08/15/2009	USA	A/Texas/44151841/2009(H1N1)
PB2	CY072717	H1N1	08/15/2009	Nicaragua	A/Managua/5307.01/2009(H1N1)
PB2	CY058243	H1N1	08/15/2009	Nicaragua	A/Managua/473.02/2009(H1N1)
PB2	CY052854	H1N1	08/15/2009	USA	A/Texas/44152535/2009(H1N1)
PB2	CY072661	H1N1	08/16/2009	Nicaragua	A/Managua/4570.01/2009(H1N1)
PB2	CY055803	H1N1	08/16/2009	Australia	A/Australia/54/2009(H1N1)
PB2	CY122972	H1N1	08/16/2009	Singapore	A/Singapore/GP2987/2009
PB2	KT180673	H1N1	08/16/2009	Singapore	A/Singapore/DMS24/2009
PB2	CY072677	H1N1	08/16/2009	Nicaragua	A/Managua/1054.02/2009(H1N1)
PB2	KP222572	H1N1	08/17/2009	Singapore	A/Singapore/464L/2009
PB2	KY925985	H1N1	08/17/2009	Brazil	A/Santa Cruz do Sul/LACENRS-201
PB2	CY058571	H1N1	08/17/2009	Nicaragua	A/Managua/4218.01/2009(H1N1)
PB2	CY123000	H1N1	08/17/2009	Singapore	A/Singapore/GP3026/2009
PB2	CY122992	H1N1	08/17/2009	Singapore	A/Singapore/GP3021/2009
PB2	CY072613	H1N1	08/17/2009	Nicaragua	A/Managua/2155.01/2009(H1N1)
PB2	KP222571	H1N1	08/17/2009	Singapore	A/Singapore/463B/2009
PB2	CY060507	H1N1	08/17/2009	Canada	A/Ontario/10296/2009(H1N1)
PB2	CY123024	H1N1	08/18/2009	Singapore	A/Singapore/GP3069/2009
PB2	JX625488	H1N1	08/18/2009	United Ki	A/England/93520038/2009
PB2	CY123032	H1N1	08/18/2009	Singapore	A/Singapore/GP3074/2009
PB2	CY058267	H1N1	08/18/2009	Nicaragua	A/Managua/164.01/2009(H1N1)
PB2	CY058259	H1N1	08/18/2009	Nicaragua	A/Managua/3244.01/2009(H1N1)
PB2	CY051710	H1N1	08/18/2009	USA	A/New York/4780/2009(H1N1)
PB2	CY063581	H1N1	08/18/2009	USA	A/Boston/154/2009(H1N1)
PB2	CY058579	H1N1	08/18/2009	Nicaragua	A/Managua/4757.02/2009(H1N1)
PB2	CY058603	H1N1	08/18/2009	Nicaragua	A/Managua/3435.01/2009(H1N1)
PB2	CY058619	H1N1	08/18/2009	Nicaragua	A/Managua/2323.02/2009(H1N1)
PB2	CY058587	H1N1	08/18/2009	Nicaragua	A/Managua/989.01/2009(H1N1)
PB2	CY058563	H1N1	08/18/2009	Nicaragua	A/Managua/2093.01/2009(H1N1)
PB2	CY063751	H1N1	08/18/2009	Singapore	A/Singapore/GP3084/2009(H1N1)
PB2	CY055026	H1N1	08/18/2009	USA	A/California/VRDL67/2009(H1N1)
PB2	CY123048	H1N1	08/18/2009	Singapore	A/Singapore/GP3093/2009
PB2	KP222573	H1N1	08/18/2009	Singapore	A/Singapore/468H/2009
PB2	JQ431390	H1N1	08/19/2009	France	A/Reunion/0148-6-M1E/2009

PB2	JQ431389	H1N1	08/19/2009	France	A/Reunion/0148-4-M1E/2009
PB2	CY123056	H1N1	08/19/2009	Singapore	A/Singapore/GP3104/2009
PB2	KT180674	H1N1	08/19/2009	Singapore	A/Singapore/DMS25/2009
PB2	CY123521	H1N1	08/19/2009	Singapore	A/Singapore/ON1802/2009
PB2	CY072853	H1N1	08/19/2009	Nicaragua	A/Managua/4702.04/2009(H1N1)
PB2	CY123529	H1N1	08/19/2009	Singapore	A/Singapore/ON1808/2009
PB2	KY925524	H1N1	08/19/2009	Brazil	A/Tenente Portela/LACENRS-2073/2
PB2	CY055276	H1N1	08/20/2009	Malaysia	A/Malaysia/12617/2009(H1N1)
PB2	CY051726	H1N1	08/20/2009	USA	A/New York/4789/2009(H1N1)
PB2	KP222574	H1N1	08/20/2009	Singapore	A/Singapore/480Y/2009
PB2	CY128154	H1N1	08/20/2009	Viet Nam	A/Viet Nam/13032008/2009
PB2	CY128186	H1N1	08/20/2009	Viet Nam	A/Viet Nam/13032016/2009
PB2	CY128170	H1N1	08/20/2009	Viet Nam	A/Viet Nam/13032011/2009
PB2	CY072773	H1N1	08/20/2009	Nicaragua	A/Managua/287.01/2009(H1N1)
PB2	CY128178	H1N1	08/20/2009	Viet Nam	A/Viet Nam/13032015/2009
PB2	KY925001	H1N1	08/20/2009	Brazil	A/Rosario do Sul/LACENRS-2110/20
PB2	CY055923	H1N1	08/20/2009	Australia	A/Australia/79/2009(H1N1)
PB2	CY128162	H1N1	08/20/2009	Viet Nam	A/Viet Nam/13032009/2009
PB2	CY072877	H1N1	08/20/2009	Nicaragua	A/Managua/2999.03/2009(H1N1)
PB2	JQ431382	H1N1	08/20/2009	France	A/Reunion/2923-3-M1E/2009
PB2	KY925791	H1N1	08/20/2009	Brazil	A/Porto Alegre/LACENRS-2121/2009
PB2	KP222575	H1N1	08/21/2009	Singapore	A/Singapore/483A/2009
PB2	CY051750	H1N1	08/21/2009	USA	A/New York/4810/2009(H1N1)
PB2	CY051734	H1N1	08/21/2009	USA	A/New York/4790/2009(H1N1)
PB2	CY060523	H1N1	08/21/2009	Canada	A/Ontario/235657/2009(H1N1)
PB2	CY123064	H1N1	08/21/2009	Singapore	A/Singapore/GP3175/2009
PB2	CY123080	H1N1	08/21/2009	Singapore	A/Singapore/GP3179/2009
PB2	CY051718	H1N1	08/21/2009	USA	A/New York/4787/2009(H1N1)
PB2	CY128194	H1N1	08/21/2009	Viet Nam	A/Viet Nam/13032019/2009
PB2	CY052169	H1N1	08/21/2009	USA	A/New York/4788/2009(H1N1)
PB2	CY072805	H1N1	08/22/2009	Nicaragua	A/Managua/432.01/2009(H1N1)
PB2	KT180675	H1N1	08/22/2009	Singapore	A/Singapore/DMS26/2009
PB2	JQ431384	H1N1	08/22/2009	France	A/Reunion/1709-4-M2E/2009
PB2	CY072757	H1N1	08/22/2009	Nicaragua	A/Managua/5227.04/2009(H1N1)
PB2	CY072869	H1N1	08/22/2009	Nicaragua	A/Managua/4147.01/2009(H1N1)
PB2	KY926395	H1N1	08/23/2009	Brazil	A/Ijui/LACENRS-2326/2009
PB2	CY119465	H1N1	08/24/2009	Malaysia	A/Malaysia/2126061/2009
PB2	CY060499	H1N1	08/24/2009	Canada	A/Ontario/10016/2009
PB2	CY060531	H1N1	08/24/2009	Canada	A/Ontario/237882/2009
PB2	CY072821	H1N1	08/24/2009	Nicaragua	A/Managua/3421.02/2009(H1N1)
PB2	JQ431381	H1N1	08/24/2009	France	A/Reunion/2133-5-M1E/2009
PB2	CY051742	H1N1	08/24/2009	USA	A/New York/4791/2009(H1N1)
PB2	KT180712	H1N1	08/24/2009	Singapore	A/Singapore/DMS623/2009
PB2	JQ431379	H1N1	08/24/2009	France	A/Reunion/2378-1-M1E/2009
PB2	JQ431391	H1N1	08/24/2009	France	A/Reunion/2133-6-M1E/2009
PB2	CY119545	H1N1	08/24/2009	Malaysia	A/Malaysia/2126082/2009
PB2	CY065799	H1N1	08/25/2009	Netherlan	A/Netherlands/1039/2009(H1N1)
PB2	CY058595	H1N1	08/25/2009	Nicaragua	A/Managua/65.02/2009(H1N1)
PB2	KT180714	H1N1	08/25/2009	Singapore	A/Singapore/DMS638/2009
PB2	KT180710	H1N1	08/25/2009	Singapore	A/Singapore/DMS618/2009
PB2	CY056402	H1N1	08/25/2009	USA	A/New York/4925/2009(H1N1)
PB2	KT180709	H1N1	08/25/2009	Singapore	A/Singapore/DMS617/2009
PB2	KP222576	H1N1	08/25/2009	Singapore	A/Singapore/491H/2009
PB2	KC781515	H1N1	08/25/2009	USA	A/Utah/51/2009

PB2	KC780677	H1N1	08/25/2009	USA	A/West Virginia/13/2009
PB2	KP222578	H1N1	08/26/2009	Singapore	A/Singapore/496T/2009
PB2	CY055931	H1N1	08/26/2009	Australia	A/Australia/80/2009(H1N1)
PB2	JQ431393	H1N1	08/26/2009	France	A/Reunion/2224-3-M3E/2009
PB2	JQ431383	H1N1	08/26/2009	France	A/Reunion/0159-3-M1E/2009
PB2	KP222577	H1N1	08/26/2009	Singapore	A/Singapore/495W/2009
PB2	KT180717	H1N1	08/26/2009	Singapore	A/Singapore/DMS653/2009
PB2	CY072909	H1N1	08/27/2009	Nicaragua	A/Managua/3434.02/2009(H1N1)
PB2	CY123581	H1N1	08/27/2009	Singapore	A/Singapore/ON1892/2009
PB2	CY123088	H1N1	08/27/2009	Singapore	A/Singapore/GP3183/2009
PB2	JN187155	H1N1	08/27/2009	Taiwan	A/Taiwan/6078/2009
PB2	KT180721	H1N1	08/27/2009	Singapore	A/Singapore/DMS676/2009
PB2	KT180719	H1N1	08/27/2009	Singapore	A/Singapore/DMS665/2009
PB2	CY055050	H1N1	08/27/2009	USA	A/California/VRDL70/2009(H1N1)
PB2	KC781963	H1N1	08/27/2009	USA	A/Mississippi/05/2009
PB2	KP222580	H1N1	08/27/2009	Singapore	A/Singapore/502J/2009
PB2	KP222579	H1N1	08/27/2009	Singapore	A/Singapore/501L/2009
PB2	CY119473	H1N1	08/27/2009	Malaysia	A/Malaysia/2135565/2009
PB2	AB538386	H1N1	08/27/2009	Japan	A/Nagano/RC1/2009(H1N1)
PB2	CY073672	H1N1	08/28/2009	Nicaragua	A/Managua/4209.04/2009(H1N1)
PB2	KC780563	H1N1	08/28/2009	USA	A/Minnesota/15/2009
PB2	CY123597	H1N1	08/28/2009	Singapore	A/Singapore/ON1900/2009
PB2	KT180676	H1N1	08/28/2009	Singapore	A/Singapore/DMS28/2009
PB2	KT180724	H1N1	08/28/2009	Singapore	A/Singapore/DMS698/2009
PB2	CY052774	H1N1	08/28/2009	USA	A/Texas/44282651/2009(H1N1)
PB2	KP222581	H1N1	08/28/2009	Singapore	A/Singapore/506T/2009
PB2	CY072949	H1N1	08/28/2009	Nicaragua	A/Managua/1244.01/2009(H1N1)
PB2	CY123613	H1N1	08/28/2009	Singapore	A/Singapore/ON1911/2009
PB2	CY123605	H1N1	08/28/2009	Singapore	A/Singapore/ON1908/2009
PB2	CY055058	H1N1	08/28/2009	USA	A/California/VRDL71/2009(H1N1)
PB2	KC782312	H1N1	08/28/2009	USA	A/Minnesota/16/2009
PB2	CY119481	H1N1	08/29/2009	Malaysia	A/Malaysia/2139971/2009
PB2	KP637843	H1N1	08/29/2009	Thailand	A/Thailand/IY07086/2009
PB2	CY128202	H1N1	08/29/2009	Viet Nam	A/Viet Nam/13032030/2009
PB2	CY052630	H1N1	08/30/2009	USA	A/Texas/44302167/2009(H1N1)
PB2	CY072797	H1N1	08/30/2009	Nicaragua	A/Managua/1984.02/2009(H1N1)
PB2	CY052622	H1N1	08/30/2009	USA	A/Texas/44302533/2009(H1N1)
PB2	CY052225	H1N1	08/30/2009	USA	A/Texas/44301765/2009(H1N1)
PB2	CY052233	H1N1	08/30/2009	USA	A/Texas/44302551/2009(H1N1)
PB2	CY072933	H1N1	08/31/2009	Nicaragua	A/Managua/4063.01/2009(H1N1)
PB2	KT180727	H1N1	08/31/2009	Singapore	A/Singapore/DMS722/2009
PB2	KT180677	H1N1	08/31/2009	Singapore	A/Singapore/DMS29/2009
PB2	KY926204	H1N1	08/31/2009	Brazil	A/Palmitinho/LACENRS-2595/2009
PB2	CY073680	H1N1	08/31/2009	Nicaragua	A/Managua/5248.03/2009(H1N1)
PB2	CY052345	H1N1	08/31/2009	USA	A/Texas/44312415/2009(H1N1)
PB2	CY072981	H1N1	08/31/2009	Nicaragua	A/Managua/3507.01/2009(H1N1)
PB2	CY052678	H1N1	08/31/2009	USA	A/Texas/44313703/2009(H1N1)
PB2	HM189562	H1N1	09/2009	South Ko	A/Korea/CJ04/2009(H1N1)
PB2	CY128210	H1N1	09/01/2009	Viet Nam	A/Viet Nam/13032035/2009
PB2	CY123139	H1N1	09/01/2009	Singapore	A/Singapore/GP3491/2009
PB2	CY065130	H1N1	09/01/2009	Netherlar	A/Netherlands/1064b/2009(H1N1)
PB2	CY123654	H1N1	09/01/2009	Singapore	A/Singapore/ON1934/2009
PB2	CY073688	H1N1	09/01/2009	Nicaragua	A/Managua/4736.02/2009(H1N1)
PB2	CY119353	H1N1	09/01/2009	Malaysia	A/Malaysia/2143696/2009(H1N1)

PB2	CY055485	H1N1	09/01/2009	USA	A/California/VRDL72/2009(H1N1)
PB2	CY052686	H1N1	09/01/2009	USA	A/Texas/45010998/2009(H1N1)
PB2	CY128466	H1N1	09/01/2009	Viet Nam	A/Viet Nam/001-1889/2009
PB2	CY055493	H1N1	09/01/2009	USA	A/California/VRDL73/2009(H1N1)
PB2	CY119345	H1N1	09/01/2009	Malaysia	A/Malaysia/2142299/2009(H1N1)
PB2	CY119337	H1N1	09/01/2009	Malaysia	A/Malaysia/2142295/2009(H1N1)
PB2	KC782305	H1N1	09/01/2009	USA	A/Wisconsin/53/2009
PB2	CY052782	H1N1	09/02/2009	USA	A/Texas/45021632/2009(H1N1)
PB2	JQ431388	H1N1	09/02/2009	France	A/Reunion/2692-2-M2E/2009
PB2	CY052886	H1N1	09/02/2009	USA	A/Texas/45024243/2009(H1N1)
PB2	CY055939	H1N1	09/02/2009	Australia	A/Australia/81/2009(H1N1)
PB2	CY055947	H1N1	09/02/2009	Australia	A/Australia/82/2009(H1N1)
PB2	CY051758	H1N1	09/02/2009	USA	A/New York/4820/2009(H1N1)
PB2	JQ431385	H1N1	09/02/2009	France	A/Reunion/2154-4-M4E/2009
PB2	KY925392	H1N1	09/02/2009	Brazil	A/Osorio/LACENRS-2638/2009
PB2	CY123664	H1N1	09/02/2009	Singapore	A/Singapore/ON1943/2009
PB2	KT180728	H1N1	09/02/2009	Singapore	A/Singapore/DMS737/2009
PB2	CY069105	H1N1	09/02/2009	Guam	A/Guam/NHRC0027/2009(H1N1)
PB2	CY072749	H1N1	09/02/2009	Nicaragua	A/Managua/2958.01/2009(H1N1)
PB2	CY052862	H1N1	09/02/2009	USA	A/Texas/45023717/2009(H1N1)
PB2	KT180731	H1N1	09/02/2009	Singapore	A/Singapore/DMS752/2009
PB2	CY123680	H1N1	09/03/2009	Singapore	A/Singapore/ON1964/2009
PB2	CY052910	H1N1	09/03/2009	USA	A/Texas/45032708/2009(H1N1)
PB2	CY123672	H1N1	09/03/2009	Singapore	A/Singapore/ON1955/2009
PB2	CY128218	H1N1	09/03/2009	Viet Nam	A/Viet Nam/13032036/2009
PB2	CY051782	H1N1	09/03/2009	USA	A/New York/4841/2009(H1N1)
PB2	CY052606	H1N1	09/03/2009	USA	A/Texas/45033567/2009(H1N1)
PB2	JQ431386	H1N1	09/03/2009	France	A/Reunion/2969-5-M1E/2009
PB2	CY051766	H1N1	09/03/2009	USA	A/New York/4823/2009(H1N1)
PB2	CY052942	H1N1	09/03/2009	USA	A/Texas/45032753/2009(H1N1)
PB2	CY051774	H1N1	09/03/2009	USA	A/New York/4824/2009(H1N1)
PB2	CY052598	H1N1	09/03/2009	USA	A/Texas/45033774/2009(H1N1)
PB2	CY052614	H1N1	09/03/2009	USA	A/Texas/45034157/2009(H1N1)
PB2	KT180678	H1N1	09/03/2009	Singapore	A/Singapore/DMS30/2009
PB2	KT180736	H1N1	09/04/2009	Singapore	A/Singapore/DMS804/2009
PB2	JQ431380	H1N1	09/04/2009	France	A/Reunion/0215-4-M1E/2009
PB2	CY065887	H1N1	09/04/2009	Netherlar	A/Netherlands/1093/2009(H1N1)
PB2	CY070998	H1N1	09/04/2009	Guam	A/Guam/NHRC0029/2009(H1N1)
PB2	CY052790	H1N1	09/04/2009	USA	A/Texas/45043852/2009(H1N1)
PB2	CY069017	H1N1	09/04/2009	Guam	A/Guam/NHRC0016/2009(H1N1)
PB2	CY068993	H1N1	09/04/2009	Guam	A/Guam/NHRC0013/2009(H1N1)
PB2	CY052870	H1N1	09/04/2009	USA	A/Texas/45042604/2009(H1N1)
PB2	CY128226	H1N1	09/04/2009	Viet Nam	A/Viet Nam/13032037/2009
PB2	CY071831	H1N1	09/05/2009	USA	A/South Carolina/WRSP520/2009(H
PB2	CY052926	H1N1	09/05/2009	USA	A/Texas/45052569/2009(H1N1)
PB2	CY072813	H1N1	09/05/2009	Nicaragua	A/Managua/572.02/2009(H1N1)
PB2	KT180740	H1N1	09/05/2009	Singapore	A/Singapore/DMS835/2009
PB2	JQ431387	H1N1	09/05/2009	France	A/Reunion/0116-3-M1E/2009
PB2	JQ431392	H1N1	09/05/2009	France	A/Reunion/2154-2-M5E/2009
PB2	CY051790	H1N1	09/06/2009	USA	A/New York/4844/2009(H1N1)
PB2	GU108483	H1N1	09/06/2009	China	A/Zhejiang-Yiwu/11/2009(H1N1)
PB2	CY051902	H1N1	09/06/2009	USA	A/Texas/45062633/2009(H1N1)
PB2	CY052894	H1N1	09/06/2009	USA	A/Texas/45062346/2009(H1N1)
PB2	CY052646	H1N1	09/06/2009	USA	A/Texas/45062584/2009(H1N1)

PB2	CY052638	H1N1	09/06/2009	USA	A/Texas/45061670/2009(H1N1)
PB2	CY052958	H1N1	09/06/2009	USA	A/Texas/45061755/2009(H1N1)
PB2	KT180679	H1N1	09/06/2009	Singapore	A/Singapore/DMS31/2009
PB2	CY060547	H1N1	09/07/2009	Canada	A/Ontario/25913/2009
PB2	CY057885	H1N1	09/07/2009	USA	A/Wisconsin/629-D01987/2009(H1N
PB2	CY052798	H1N1	09/07/2009	USA	A/Texas/45072273/2009(H1N1)
PB2	CY052830	H1N1	09/07/2009	USA	A/Texas/45072128/2009(H1N1)
PB2	HM241715	H1N1	09/07/2009	India	A/Nsk/NIV10348/2009(H1N1)
PB2	CY052822	H1N1	09/07/2009	USA	A/Texas/45072656/2009(H1N1)
PB2	CY052814	H1N1	09/07/2009	USA	A/Texas/45071344/2009(H1N1)
PB2	GU112097	H1N1	09/07/2009	China	A/Zhejiang/DTID-ZJU02/2009(H1N1)
PB2	GU189656	H1N1	09/07/2009	China	A/Zhejiang/DTID-ZJU03/2009(H1N1)
PB2	CY052694	H1N1	09/07/2009	USA	A/Texas/45071524/2009(H1N1)
PB2	CY119489	H1N1	09/07/2009	Malaysia	A/Malaysia/2156796/2009
PB2	CY057002	H1N1	09/08/2009	USA	A/Wisconsin/629-D00734/2009(H1N
PB2	CY057010	H1N1	09/08/2009	USA	A/Wisconsin/629-D02367/2009(H1N
PB2	CY123698	H1N1	09/08/2009	Singapore	A/Singapore/ON2009/2009
PB2	CY073696	H1N1	09/08/2009	Nicaragua	A/Managua/5953.01/2009(H1N1)
PB2	KT180741	H1N1	09/08/2009	Singapore	A/Singapore/DMS847/2009
PB2	KT180743	H1N1	09/08/2009	Singapore	A/Singapore/DMS869/2009
PB2	CY128234	H1N1	09/08/2009	Viet Nam	A/Viet Nam/13032040/2009
PB2	CY052702	H1N1	09/08/2009	USA	A/Texas/45083819/2009(H1N1)
PB2	JN187156	H1N1	09/09/2009	Taiwan	A/Taiwan/937/2009
PB2	CY052662	H1N1	09/09/2009	USA	A/Texas/45093846/2009(H1N1)
PB2	HM568133	H1N1	09/09/2009	United Ki	A/England/H093780041/2009
PB2	KT180744	H1N1	09/09/2009	Singapore	A/Singapore/DMS870/2009
PB2	CY057018	H1N1	09/09/2009	USA	A/Wisconsin/629-D02041/2009(H1N
PB2	CY057026	H1N1	09/09/2009	USA	A/Wisconsin/629-D02133/2009(H1N
PB2	CY069089	H1N1	09/09/2009	Guam	A/Guam/NHRC0025/2009(H1N1)
PB2	CY051806	H1N1	09/09/2009	USA	A/New York/4866/2009(H1N1)
PB2	CY052670	H1N1	09/09/2009	USA	A/Texas/45093670/2009(H1N1)
PB2	CY052742	H1N1	09/09/2009	USA	A/Texas/45091417/2009(H1N1)
PB2	CY052734	H1N1	09/09/2009	USA	A/Texas/45091405/2009(H1N1)
PB2	CY051798	H1N1	09/09/2009	USA	A/New York/4856/2009(H1N1)
PB2	CY052710	H1N1	09/09/2009	USA	A/Texas/45093214/2009(H1N1)
PB2	CY052177	H1N1	09/09/2009	USA	A/New York/4855/2009(H1N1)
PB2	CY052726	H1N1	09/09/2009	USA	A/Texas/45091402/2009(H1N1)
PB2	CY069113	H1N1	09/09/2009	Guam	A/Guam/NHRC0028/2009(H1N1)
PB2	CY052518	H1N1	09/10/2009	USA	A/Texas/45103737/2009(H1N1)
PB2	JN187157	H1N1	09/10/2009	Taiwan	A/Taiwan/940/2009
PB2	CY052534	H1N1	09/10/2009	USA	A/Texas/45103998/2009(H1N1)
PB2	CY052750	H1N1	09/10/2009	USA	A/Texas/45101424/2009(H1N1)
PB2	CY051814	H1N1	09/10/2009	USA	A/New York/4870/2009(H1N1)
PB2	CY052758	H1N1	09/10/2009	USA	A/Texas/45103759/2009(H1N1)
PB2	CY052526	H1N1	09/10/2009	USA	A/Texas/45104048/2009(H1N1)
PB2	KT180748	H1N1	09/10/2009	Singapore	A/Singapore/DMS905/2009
PB2	CY119521	H1N1	09/10/2009	Malaysia	A/Malaysia/2097960/2009
PB2	CY118265	H1N1	09/10/2009	Malaysia	A/Malaysia/2089302/2009
PB2	KC781752	H1N1	09/10/2009	USA	A/North Carolina/37/2009
PB2	CY065919	H1N1	09/10/2009	Netherlan	A/Netherlands/1084/2009(H1N1)
PB2	KY925254	H1N1	09/10/2009	Brazil	A/Campina das Missoes/LACENRS-2
PB2	CY052766	H1N1	09/10/2009	USA	A/Texas/45101422/2009(H1N1)
PB2	CY052902	H1N1	09/10/2009	USA	A/Texas/45102952/2009(H1N1)
PB2	CY052510	H1N1	09/10/2009	USA	A/Texas/45104026/2009(H1N1)

PB2	CY072925	H1N1	09/10/2009	Nicaragua	A/Managua/4972.02/2009(H1N1)
PB2	CY057034	H1N1	09/10/2009	USA	A/Wisconsin/629-D00357/2009(H1N
PB2	CY052654	H1N1	09/10/2009	USA	A/Texas/45103259/2009(H1N1)
PB2	HM568141	H1N1	09/10/2009	United Ki	A/England/H093780034/2009
PB2	CY065911	H1N1	09/10/2009	Netherlan	A/Netherlands/1083/2009(H1N1)
PB2	CY128242	H1N1	09/11/2009	Viet Nam	A/Viet Nam/13032044/2009
PB2	KC780109	H1N1	09/11/2009	USA	A/Alaska/38/2009
PB2	CY083069	H1N1	09/11/2009	Colombia	A/Bogota/WRAIR0435T/2009
PB2	CY052542	H1N1	09/11/2009	USA	A/Texas/45113882/2009(H1N1)
PB2	KT180750	H1N1	09/11/2009	Singapore	A/Singapore/DMS913/2009
PB2	CY052502	H1N1	09/11/2009	USA	A/Texas/45113371/2009(H1N1)
PB2	CY052918	H1N1	09/11/2009	USA	A/Texas/45113911/2009(H1N1)
PB2	CY123151	H1N1	09/11/2009	Singapore	A/Singapore/GP3545/2009
PB2	CY083061	H1N1	09/11/2009	Colombia	A/Bogota/WRAIR0435N/2009
PB2	CY073520	H1N1	09/11/2009	Nicaragua	A/Managua/3014.02/2009(H1N1)
PB2	CY057365	H1N1	09/11/2009	USA	A/Wisconsin/629-D02485/2009(H1N
PB2	CY072829	H1N1	09/11/2009	Nicaragua	A/Managua/5115.01/2009(H1N1)
PB2	CY052185	H1N1	09/12/2009	USA	A/New York/4881/2009(H1N1)
PB2	CY052558	H1N1	09/12/2009	USA	A/Texas/45122774/2009(H1N1)
PB2	CY052566	H1N1	09/12/2009	USA	A/Texas/45122722/2009(H1N1)
PB2	CY052934	H1N1	09/12/2009	USA	A/Texas/45121004/2009(H1N1)
PB2	CY057373	H1N1	09/12/2009	USA	A/Wisconsin/629-D00643/2009(H1N
PB2	CY052462	H1N1	09/12/2009	USA	A/Texas/45122369/2009(H1N1)
PB2	CY052478	H1N1	09/12/2009	USA	A/Texas/45122033/2009(H1N1)
PB2	KT180680	H1N1	09/12/2009	Singapore	A/Singapore/DMS33/2009
PB2	CY072741	H1N1	09/12/2009	Nicaragua	A/Managua/1419.02/2009(H1N1)
PB2	CY072837	H1N1	09/12/2009	Nicaragua	A/Managua/1909.02/2009(H1N1)
PB2	CY052446	H1N1	09/12/2009	USA	A/Texas/45122538/2009(H1N1)
PB2	CY052494	H1N1	09/12/2009	USA	A/Texas/45122036/2009(H1N1)
PB2	CY057381	H1N1	09/12/2009	USA	A/Wisconsin/629-D01850/2009(H1N
PB2	CY052950	H1N1	09/12/2009	USA	A/Texas/45122886/2009(H1N1)
PB2	CY052470	H1N1	09/12/2009	USA	A/Texas/45121606/2009(H1N1)
PB2	CY052486	H1N1	09/12/2009	USA	A/Texas/45122282/2009(H1N1)
PB2	CY052438	H1N1	09/12/2009	USA	A/Texas/45120922/2009(H1N1)
PB2	CY052838	H1N1	09/13/2009	USA	A/Texas/45131305/2009(H1N1)
PB2	CY052846	H1N1	09/13/2009	USA	A/Texas/45132202/2009(H1N1)
PB2	CY052550	H1N1	09/13/2009	USA	A/Texas/45132214/2009(H1N1)
PB2	CY052582	H1N1	09/13/2009	USA	A/Texas/45131774/2009(H1N1)
PB2	CY052806	H1N1	09/13/2009	USA	A/Texas/45131576/2009(H1N1)
PB2	CY052454	H1N1	09/13/2009	USA	A/Texas/45130742/2009(H1N1)
PB2	CY052590	H1N1	09/13/2009	USA	A/Texas/45132647/2009(H1N1)
PB2	CY052574	H1N1	09/13/2009	USA	A/Texas/45132788/2009(H1N1)
PB2	CY069025	H1N1	09/13/2009	Guam	A/Guam/NHRC0017/2009(H1N1)
PB2	CY123742	H1N1	09/14/2009	Singapore	A/Singapore/ON2081/2009
PB2	CY071014	H1N1	09/14/2009	Guam	A/Guam/NHRC0031/2009(H1N1)
PB2	CY069049	H1N1	09/14/2009	Guam	A/Guam/NHRC0020/2009(H1N1)
PB2	CY072941	H1N1	09/14/2009	Nicaragua	A/Managua/5590.02/2009(H1N1)
PB2	KF897769	H1N1	09/14/2009	France	A/StEtienne/1691/2009
PB2	KC780923	H1N1	09/14/2009	USA	A/Kansas/19/2009
PB2	JX625557	H1N1	09/14/2009	United Ki	A/England/94560024/2009
PB2	CY071022	H1N1	09/14/2009	Guam	A/Guam/NHRC0032/2009(H1N1)
PB2	CY072893	H1N1	09/14/2009	Nicaragua	A/Managua/12.01/2009(H1N1)
PB2	CY052718	H1N1	09/14/2009	USA	A/Texas/45140902/2009(H1N1)
PB2	KC780828	H1N1	09/14/2009	USA	A/Kansas/20/2009

PB2	CY055430	H1N1	09/14/2009	USA	A/New York/4887/2009(H1N1)
PB2	KC781045	H1N1	09/15/2009	USA	A/Massachusetts/31/2009
PB2	CY090096	H1N1	09/15/2009	Nicaragua	A/Managua/3755.03/2009
PB2	KT180754	H1N1	09/15/2009	Singapore	A/Singapore/DMS952/2009
PB2	KT180753	H1N1	09/15/2009	Singapore	A/Singapore/DMS946/2009
PB2	KT180681	H1N1	09/15/2009	Singapore	A/Singapore/DMS34/2009
PB2	CY123750	H1N1	09/15/2009	Singapore	A/Singapore/ON2095/2009
PB2	CY119497	H1N1	09/15/2009	Malaysia	A/Malaysia/2173411/2009
PB2	CY057397	H1N1	09/15/2009	USA	A/Wisconsin/629-D00036/2009(H1N
PB2	CY053471	H1N1	09/15/2009	Taiwan	A/Taiwan/143/2009(H1N1)
PB2	CY057389	H1N1	09/15/2009	USA	A/Wisconsin/629-D02192/2009(H1N
PB2	CY057405	H1N1	09/15/2009	USA	A/Wisconsin/629-D01469/2009(H1N
PB2	CY069073	H1N1	09/15/2009	Guam	A/Guam/NHRC0023/2009(H1N1)
PB2	CY057413	H1N1	09/15/2009	USA	A/Wisconsin/629-D00832/2009(H1N
PB2	KT180756	H1N1	09/16/2009	Singapore	A/Singapore/DMS962/2009
PB2	CY120738	H1N1	09/16/2009	Brazil	A/Brazil/AVS06/2009
PB2	KC780850	H1N1	09/16/2009	USA	A/Kansas/21/2009
PB2	KC780084	H1N1	09/16/2009	USA	A/South Carolina/18/2009
PB2	KC782205	H1N1	09/16/2009	USA	A/South Carolina/18/2009
PB2	KC782213	H1N1	09/16/2009	USA	A/South Carolina/18/2009
PB2	KC781311	H1N1	09/16/2009	USA	A/Wisconsin/66/2009
PB2	CY088650	H1N1	09/16/2009	India	A/Rtng/NIV11650/2009
PB2	CY072789	H1N1	09/16/2009	Nicaragua	A/Managua/5401.01/2009(H1N1)
PB2	CY057437	H1N1	09/16/2009	USA	A/Wisconsin/629-D01935/2009(H1N
PB2	CY057429	H1N1	09/16/2009	USA	A/Wisconsin/629-D00485/2009(H1N
PB2	CY057421	H1N1	09/16/2009	USA	A/Wisconsin/629-D01802/2009(H1N
PB2	CY128250	H1N1	09/17/2009	Viet Nam	A/Viet Nam/13032048/2009
PB2	KP637539	H1N1	09/18/2009	Thailand	A/Thailand/SN08312/2009
PB2	CY053495	H1N1	09/18/2009	Taiwan	A/Taiwan/177/2009(H1N1)
PB2	KT180758	H1N1	09/18/2009	Singapore	A/Singapore/DMS978/2009
PB2	KT180682	H1N1	09/18/2009	Singapore	A/Singapore/DMS35/2009
PB2	KT180760	H1N1	09/18/2009	Singapore	A/Singapore/DMS980/2009
PB2	CY119505	H1N1	09/18/2009	Malaysia	A/Malaysia/2180812/2009
PB2	CY057445	H1N1	09/18/2009	USA	A/Wisconsin/629-D01522/2009(H1N
PB2	HM568149	H1N1	09/18/2009	United Ki	A/England/H093900113/2009
PB2	KY925018	H1N1	09/18/2009	Brazil	A/Pelotas/LACENRS-3045/2009
PB2	KT180761	H1N1	09/18/2009	Singapore	A/Singapore/DMS982/2009
PB2	CY053487	H1N1	09/18/2009	Taiwan	A/Taiwan/167/2009(H1N1)
PB2	CY055509	H1N1	09/19/2009	USA	A/California/VRDL75/2009(H1N1)
PB2	CY069001	H1N1	09/20/2009	Guam	A/Guam/NHRC0014/2009(H1N1)
PB2	JN187158	H1N1	09/20/2009	Taiwan	A/Taiwan/8575/2009
PB2	KC781059	H1N1	09/20/2009	USA	A/North Carolina/44/2009
PB2	AB539736	H1N1	09/21/2009	China	A/Shanghai/P1/2009(H1N1)
PB2	CY057453	H1N1	09/21/2009	USA	A/Wisconsin/629-D01355/2009(H1N
PB2	KT180555	H1N1	09/21/2009	Singapore	A/Singapore/DMS1006/2009
PB2	CY057461	H1N1	09/21/2009	USA	A/Wisconsin/629-D00748/2009(H1N
PB2	CY055517	H1N1	09/21/2009	USA	A/California/VRDL76/2009(H1N1)
PB2	KC781986	H1N1	09/22/2009	USA	A/Kansas/22/2009
PB2	CY057485	H1N1	09/22/2009	USA	A/Wisconsin/629-D01237/2009(H1N
PB2	GU211234	H1N1	09/22/2009	Russia	A/Russia/01/2009(H1N1)
PB2	CY057477	H1N1	09/22/2009	USA	A/Wisconsin/629-D00402/2009(H1N
PB2	CY128450	H1N1	09/22/2009	Viet Nam	A/Viet Nam/001-1716/2009
PB2	CY057469	H1N1	09/22/2009	USA	A/Wisconsin/629-D00839/2009(H1N
PB2	CY055525	H1N1	09/22/2009	USA	A/California/VRDL78/2009(H1N1)

PB2	CY057501	H1N1	09/23/2009	USA	A/Wisconsin/629-D00287/2009(H1N
PB2	CY057493	H1N1	09/23/2009	USA	A/Wisconsin/629-D00346/2009(H1N
PB2	CY056418	H1N1	09/23/2009	USA	A/New York/4945/2009(H1N1)
PB2	KC780976	H1N1	09/23/2009	USA	A/Kansas/23/2009
PB2	CY123764	H1N1	09/23/2009	Singapore	A/Singapore/ON2135/2009
PB2	CY123772	H1N1	09/23/2009	Singapore	A/Singapore/ON2136/2009
PB2	CY056426	H1N1	09/23/2009	USA	A/New York/4947/2009(H1N1)
PB2	CY056410	H1N1	09/23/2009	USA	A/New York/4944/2009(H1N1)
PB2	CY123185	H1N1	09/23/2009	Singapore	A/Singapore/GP3839/2009
PB2	KJ690397	H1N1	09/23/2009	Uganda	A/Uganda/MUWRP-093/2009
PB2	KT180683	H1N1	09/24/2009	Singapore	A/Singapore/DMS37/2009
PB2	CY119513	H1N1	09/24/2009	Malaysia	A/Malaysia/2190140/2009
PB2	CY071735	H1N1	09/24/2009	Mexico	A/Mexico City/WR1701T/2009(H1N1)
PB2	CY057509	H1N1	09/24/2009	USA	A/Wisconsin/629-D00565/2009(H1N
PB2	CY054890	H1N1	09/24/2009	USA	A/California/VRDL36/2009(H1N1)
PB2	CY057517	H1N1	09/24/2009	USA	A/Wisconsin/629-D00410/2009(H1N
PB2	CY069065	H1N1	09/25/2009	Guam	A/Guam/NHRC0022/2009(H1N1)
PB2	KC780150	H1N1	09/25/2009	USA	A/Mississippi/03/2009
PB2	CY069081	H1N1	09/26/2009	Guam	A/Guam/NHRC0024/2009(H1N1)
PB2	CY057525	H1N1	09/26/2009	USA	A/Wisconsin/629-D00891/2009(H1N
PB2	KC780901	H1N1	09/27/2009	USA	A/Wisconsin/51/2009
PB2	CY123780	H1N1	09/28/2009	Singapore	A/Singapore/ON2145/2009
PB2	KJ690476	H1N1	09/28/2009	Uganda	A/Uganda/MUWRP-098/2009
PB2	JN187159	H1N1	09/28/2009	Taiwan	A/Taiwan/4909/2009
PB2	CY057533	H1N1	09/28/2009	USA	A/Wisconsin/629-D01391/2009(H1N
PB2	JN187160	H1N1	09/28/2009	Taiwan	A/Taiwan/8542/2009
PB2	KC780988	H1N1	09/29/2009	USA	A/Texas/66/2009
PB2	CY058283	H1N1	09/29/2009	USA	A/Wisconsin/629-D01015/2009(H1N
PB2	CY072957	H1N1	09/29/2009	Nicaragua	A/Managua/5258.02/2009(H1N1)
PB2	CY060555	H1N1	09/29/2009	Canada	A/Ontario/26184/2009(H1N1)
PB2	KC780999	H1N1	09/29/2009	USA	A/Texas/66/2009
PB2	CY083436	H1N1	09/30/2009	USA	A/Ft.Benning/WRAIR1669P/2009
PB2	KT180685	H1N1	09/30/2009	Singapore	A/Singapore/DMS39/2009
PB2	CY065927	H1N1	09/30/2009	Netherlar	A/Netherlands/2290/2009
PB2	KC780028	H1N1	09/30/2009	USA	A/Wisconsin/67/2009
PB2	CY123195	H1N1	09/30/2009	Singapore	A/Singapore/GP3912/2009
PB2	HM189569	H1N1	10/2009	South Ko	A/Korea/CJ68/2009(H1N1)
PB2	HM189568	H1N1	10/2009	South Ko	A/Korea/CJ63/2009(H1N1)
PB2	HM189567	H1N1	10/2009	South Ko	A/Korea/CJ40/2009(H1N1)
PB2	CY062209	H1N1	10/01/2009	USA	A/California/VRDL80/2009(H1N1)
PB2	CY068961	H1N1	10/01/2009	Guam	A/Guam/NHRC0009/2009(H1N1)
PB2	CY128258	H1N1	10/01/2009	Viet Nam	A/Viet Nam/13032051/2009
PB2	KC781995	H1N1	10/01/2009	USA	A/North Carolina/45/2009
PB2	CY062201	H1N1	10/01/2009	USA	A/California/VRDL79/2009(H1N1)
PB2	CY062233	H1N1	10/01/2009	USA	A/California/VRDL83/2009(H1N1)
PB2	CY056578	H1N1	10/01/2009	USA	A/New York/5755/2009(H1N1)
PB2	CY069009	H1N1	10/01/2009	Guam	A/Guam/NHRC0015/2009(H1N1)
PB2	CY118274	H1N1	10/01/2009	Malaysia	A/Malaysia/2205822/2009
PB2	CY063226	H1N1	10/01/2009	USA	A/Wisconsin/629-D01351/2009(H1N
PB2	CY062225	H1N1	10/01/2009	USA	A/California/VRDL82/2009(H1N1)
PB2	JX625541	H1N1	10/01/2009	United Ki	A/Northern Ireland/94480417/200
PB2	CY062217	H1N1	10/01/2009	USA	A/California/VRDL81/2009(H1N1)
PB2	KC780141	H1N1	10/02/2009	USA	A/New Mexico/08/2009
PB2	JX625503	H1N1	10/02/2009	United Ki	A/England/94200009/2009

PB2	CY057549	H1N1	10/02/2009	USA	A/Wisconsin/629-D00853/2009(H1N
PB2	CY057541	H1N1	10/02/2009	USA	A/Wisconsin/629-D00250/2009(H1N
PB2	KT180686	H1N1	10/02/2009	Singapore	A/Singapore/DMS40/2009
PB2	CY055438	H1N1	10/03/2009	USA	A/New York/4977/2009(H1N1)
PB2	CY057557	H1N1	10/03/2009	USA	A/Wisconsin/629-D02337/2009(H1N
PB2	CY056490	H1N1	10/04/2009	USA	A/New York/4995/2009(H1N1)
PB2	CY057565	H1N1	10/04/2009	USA	A/Wisconsin/629-D00690/2009(H1N
PB2	CY055446	H1N1	10/05/2009	USA	A/New York/4979/2009(H1N1)
PB2	CY056482	H1N1	10/05/2009	USA	A/New York/4990/2009(H1N1)
PB2	CY056434	H1N1	10/05/2009	USA	A/New York/4981/2009(H1N1)
PB2	KJ690539	H1N1	10/05/2009	Uganda	A/Uganda/MUWRP-102/2009
PB2	KT180687	H1N1	10/05/2009	Singapore	A/Singapore/DMS41/2009
PB2	JX625525	H1N1	10/05/2009	United Ki	A/Northern Ireland/94480397/200
PB2	CY056450	H1N1	10/05/2009	USA	A/New York/4985/2009(H1N1)
PB2	CY057573	H1N1	10/05/2009	USA	A/Wisconsin/629-D00834/2009(H1N
PB2	CY056458	H1N1	10/05/2009	USA	A/New York/4986/2009(H1N1)
PB2	CY056442	H1N1	10/06/2009	USA	A/New York/4984/2009(H1N1)
PB2	CY057581	H1N1	10/06/2009	USA	A/Wisconsin/629-S1252/2009(H1N1)
PB2	KY888158	H1N1	10/06/2009	India	A/Kerala/RGCBA923/2009
PB2	CY056474	H1N1	10/06/2009	USA	A/New York/4988/2009(H1N1)
PB2	CY056466	H1N1	10/06/2009	USA	A/New York/4987/2009(H1N1)
PB2	CY058291	H1N1	10/06/2009	USA	A/Wisconsin/629-D01102/2009(H1N
PB2	KC780927	H1N1	10/06/2009	USA	A/Colorado/13/2009
PB2	CY075499	H1N1	10/07/2009	USA	A/Boston/584/2009(H1N1)
PB2	CY058299	H1N1	10/07/2009	USA	A/Wisconsin/629-D02100/2009(H1N
PB2	CY083109	H1N1	10/07/2009	Ecuador	A/Quito/WRAIR0617N/2009
PB2	JN187161	H1N1	10/08/2009	Taiwan	A/Taiwan/7418/2009
PB2	CY128266	H1N1	10/08/2009	Viet Nam	A/Viet Nam/13032059/2009
PB2	KT180688	H1N1	10/08/2009	Singapore	A/Singapore/DMS42/2009
PB2	CY128274	H1N1	10/08/2009	Viet Nam	A/Viet Nam/13032060/2009
PB2	CY128282	H1N1	10/09/2009	Viet Nam	A/Viet Nam/13032061/2009
PB2	CY062257	H1N1	10/09/2009	USA	A/California/VRDL86/2009(H1N1)
PB2	CY063082	H1N1	10/09/2009	USA	A/California/VRDL88/2009(H1N1)
PB2	JX625603	H1N1	10/09/2009	United Ki	A/Northern Ireland/94940020/200
PB2	KP637459	H1N1	10/09/2009	Thailand	A/Thailand/PY08063/2009
PB2	CY062241	H1N1	10/09/2009	USA	A/California/VRDL84/2009(H1N1)
PB2	CY062249	H1N1	10/09/2009	USA	A/California/VRDL85/2009(H1N1)
PB2	JX625533	H1N1	10/09/2009	United Ki	A/Northern Ireland/94480404/200
PB2	JX625510	H1N1	10/09/2009	United Ki	A/England/1072/2009
PB2	CY057605	H1N1	10/09/2009	USA	A/Wisconsin/629-D01080/2009(H1N
PB2	CY057589	H1N1	10/09/2009	USA	A/Wisconsin/629-D01749/2009(H1N
PB2	CY057597	H1N1	10/09/2009	USA	A/Wisconsin/629-D02090/2009(H1N
PB2	CY063074	H1N1	10/09/2009	USA	A/California/VRDL87/2009(H1N1)
PB2	CY057613	H1N1	10/10/2009	USA	A/Wisconsin/629-D00557/2009(H1N
PB2	KC780398	H1N1	10/10/2009	USA	A/New Mexico/06/2009
PB2	CY089194	H1N1	10/10/2009	USA	A/Boston/583/2009(H1N1)
PB2	CY095960	H1N1	10/11/2009	China	A/Zhejiang/87/2009
PB2	CY096042	H1N1	10/11/2009	China	A/Hubei/99/2009
PB2	CY095932	H1N1	10/11/2009	China	A/Zhejiang/83/2009
PB2	CY072701	H1N1	10/11/2009	Nicaragua	A/Managua/3013.01/2009(H1N1)
PB2	CY095968	H1N1	10/11/2009	China	A/Zhejiang/88/2009
PB2	CY057621	H1N1	10/12/2009	USA	A/Wisconsin/629-S1309/2009(H1N1)
PB2	CY063250	H1N1	10/12/2009	USA	A/Wisconsin/629-D00830/2009(H1N
PB2	CY056890	H1N1	10/12/2009	USA	A/San Diego/INS54/2009(H1N1)

PB2	CY063234	H1N1	10/12/2009	USA	A/Wisconsin/629-D00675/2009(H1N1
PB2	CY057237	H1N1	10/12/2009	USA	A/New York/5045/2009(H1N1)
PB2	HM568069	H1N1	10/12/2009	United Ki	A/England/H094220579/2009
PB2	CY055963	H1N1	10/12/2009	USA	A/San Diego/INS02/2009(H1N1)
PB2	CY055979	H1N1	10/12/2009	USA	A/San Diego/INS04/2009(H1N1)
PB2	CY055971	H1N1	10/12/2009	USA	A/San Diego/INS03/2009(H1N1)
PB2	CY056211	H1N1	10/12/2009	USA	A/San Diego/INS52/2009(H1N1)
PB2	CY055995	H1N1	10/12/2009	USA	A/San Diego/INS06/2009(H1N1)
PB2	KT180689	H1N1	10/12/2009	Singapore	A/Singapore/DMS43/2009
PB2	KP637275	H1N1	10/12/2009	Thailand	A/Thailand/IY07093/2009
PB2	KC261306	H1N1	10/12/2009	Mexico	A/Mexico City/INERPHAC1/2009(H1N
PB2	HM568061	H1N1	10/12/2009	United Ki	A/England/H094240645/2009
PB2	CY083949	H1N1	10/12/2009	USA	A/San Diego/INS194/2009(H1N1)
PB2	CY063242	H1N1	10/12/2009	USA	A/Wisconsin/629-D02442/2009(H1N1
PB2	CY057629	H1N1	10/12/2009	USA	A/Wisconsin/629-S1313/2009(H1N1)
PB2	CY066534	H1N1	10/12/2009	USA	A/San Diego/INS193/2009(H1N1)
PB2	CY055987	H1N1	10/12/2009	USA	A/San Diego/INS05/2009(H1N1)
PB2	CY055955	H1N1	10/12/2009	USA	A/San Diego/INS01/2009(H1N1)
PB2	CY056219	H1N1	10/12/2009	USA	A/San Diego/INS53/2009(H1N1)
PB2	CY056882	H1N1	10/12/2009	USA	A/San Diego/INS51/2009(H1N1)
PB2	CY056003	H1N1	10/13/2009	USA	A/San Diego/INS09/2009(H1N1)
PB2	CY056530	H1N1	10/13/2009	USA	A/New York/5130/2009(H1N1)
PB2	CY057325	H1N1	10/13/2009	USA	A/New York/5667/2009(H1N1)
PB2	CY056506	H1N1	10/13/2009	USA	A/New York/5079/2009(H1N1)
PB2	CY056011	H1N1	10/13/2009	USA	A/San Diego/INS10/2009(H1N1)
PB2	CY057341	H1N1	10/13/2009	USA	A/San Diego/INS08/2009(H1N1)
PB2	CY083837	H1N1	10/13/2009	USA	A/San Diego/INS07/2009(H1N1)
PB2	CY056498	H1N1	10/13/2009	USA	A/New York/5074/2009(H1N1)
PB2	CY071607	H1N1	10/13/2009	USA	A/Ft Carson/WR1448P/2009(H1N1)
PB2	CY056514	H1N1	10/13/2009	USA	A/New York/5083/2009(H1N1)
PB2	CY056522	H1N1	10/13/2009	USA	A/New York/5086/2009(H1N1)
PB2	CY081525	H1N1	10/14/2009	Cambodia	A/Cambodia/NHRCC00005/2009(H1N1)
PB2	CY081493	H1N1	10/14/2009	Cambodia	A/Cambodia/NHRCC00001/2009(H1N1)
PB2	CY083476	H1N1	10/14/2009	USA	A/Chicago/WRAIR1690P/2009
PB2	CY056898	H1N1	10/14/2009	USA	A/San Diego/INS55/2009(H1N1)
PB2	CY081517	H1N1	10/14/2009	Cambodia	A/Cambodia/NHRCC00004/2009(H1N1)
PB2	CY081549	H1N1	10/14/2009	Cambodia	A/Cambodia/NHRCC00008/2009(H1N1)
PB2	KT180690	H1N1	10/14/2009	Singapore	A/Singapore/DMS44/2009
PB2	CY056019	H1N1	10/14/2009	USA	A/San Diego/INS11/2009(H1N1)
PB2	CY057645	H1N1	10/14/2009	USA	A/Wisconsin/629-S1350/2009(H1N1)
PB2	CY061234	H1N1	10/14/2009	USA	A/San Diego/INS101/2009(H1N1)
PB2	CY081533	H1N1	10/14/2009	Cambodia	A/Cambodia/NHRCC00006/2009(H1N1)
PB2	CY057637	H1N1	10/14/2009	USA	A/Wisconsin/629-S1348/2009(H1N1)
PB2	CY081501	H1N1	10/14/2009	Cambodia	A/Cambodia/NHRCC00002/2009(H1N1)
PB2	CY083492	H1N1	10/14/2009	USA	A/Chicago/WRAIR1691P/2009
PB2	CY061242	H1N1	10/14/2009	USA	A/San Diego/INS102/2009(H1N1)
PB2	CY056027	H1N1	10/14/2009	USA	A/San Diego/INS12/2009(H1N1)
PB2	CY081541	H1N1	10/14/2009	Cambodia	A/Cambodia/NHRCC00007/2009(H1N1)
PB2	CY081509	H1N1	10/14/2009	Cambodia	A/Cambodia/NHRCC00003/2009(H1N1)
PB2	CY056083	H1N1	10/15/2009	USA	A/District of Columbia/INS24/200
PB2	CY082991	H1N1	10/15/2009	Cambodia	A/Cambodia/NHRCC00009/2009(H1N1)
PB2	KC780343	H1N1	10/15/2009	USA	A/New York/102/2009
PB2	CY081557	H1N1	10/15/2009	Cambodia	A/Cambodia/NHRCC00010/2009(H1N1)
PB2	CY057245	H1N1	10/15/2009	USA	A/New York/5158/2009(H1N1)

PB2	CY092927	H1N1	10/15/2009	USA	A/Maryland/NHRC0002/2009
PB2	CY066542	H1N1	10/15/2009	USA	A/San Diego/INS195/2009(H1N1)
PB2	CY063258	H1N1	10/15/2009	USA	A/Wisconsin/629-D00692/2009(H1N
PB2	CY056906	H1N1	10/15/2009	USA	A/San Diego/INS57/2009(H1N1)
PB2	JX625565	H1N1	10/15/2009	United Ki	A/Northern Ireland/1/2009
PB2	CY080351	H1N1	10/15/2009	Cambodia	A/Cambodia/NHRCC00011/2009(H1N1)
PB2	KC781024	H1N1	10/16/2009	USA	A/North Carolina/43/2009
PB2	KC780509	H1N1	10/16/2009	USA	A/North Carolina/46/2009
PB2	CY056538	H1N1	10/16/2009	USA	A/New York/5141/2009(H1N1)
PB2	CY063266	H1N1	10/16/2009	USA	A/Wisconsin/629-D00740/2009(H1N
PB2	CY056546	H1N1	10/16/2009	USA	A/New York/5150/2009(H1N1)
PB2	KC782323	H1N1	10/16/2009	USA	A/North Carolina/46/2009
PB2	CY092935	H1N1	10/16/2009	USA	A/Maryland/NHRC0003/2009
PB2	CY056650	H1N1	10/16/2009	USA	A/New York/6079/2009(H1N1)
PB2	CY057253	H1N1	10/16/2009	USA	A/New York/5161/2009(H1N1)
PB2	GU198198	H1N1	10/17/2009	China	A/Nanjing/3/2009(H1N1)
PB2	CY063090	H1N1	10/17/2009	USA	A/California/VRDL89/2009(H1N1)
PB2	CY063114	H1N1	10/17/2009	USA	A/California/VRDL92/2009(H1N1)
PB2	CY058053	H1N1	10/17/2009	USA	A/Texas/46172734/2009(H1N1)
PB2	CY063098	H1N1	10/17/2009	USA	A/California/VRDL90/2009(H1N1)
PB2	CY056554	H1N1	10/17/2009	USA	A/New York/5173/2009(H1N1)
PB2	CY058045	H1N1	10/17/2009	USA	A/Texas/46172731/2009(H1N1)
PB2	CY063122	H1N1	10/17/2009	USA	A/California/VRDL93/2009(H1N1)
PB2	CY063106	H1N1	10/17/2009	USA	A/California/VRDL91/2009(H1N1)
PB2	CY128290	H1N1	10/18/2009	Viet Nam	A/Viet Nam/13032071/2009
PB2	CY060563	H1N1	10/18/2009	Canada	A/Ontario/296008/2009
PB2	KC780116	H1N1	10/18/2009	USA	A/New Mexico/07/2009
PB2	CY058069	H1N1	10/18/2009	USA	A/Texas/46181292/2009(H1N1)
PB2	CY075507	H1N1	10/18/2009	USA	A/Boston/591/2009(H1N1)
PB2	CY058691	H1N1	10/18/2009	USA	A/Texas/46182018/2009(H1N1)
PB2	CY058061	H1N1	10/18/2009	USA	A/Texas/46181235/2009(H1N1)
PB2	CY057669	H1N1	10/18/2009	USA	A/Wisconsin/629-D00099/2009(H1N
PB2	CY057653	H1N1	10/18/2009	USA	A/Wisconsin/629-D02342/2009(H1N
PB2	CY057661	H1N1	10/18/2009	USA	A/Wisconsin/629-D00544/2009(H1N
PB2	CY057685	H1N1	10/19/2009	USA	A/Wisconsin/629-S1384/2009(H1N1)
PB2	JX403975	H1N1	10/19/2009	China	A/Liaoning/1/2009(H1N1)
PB2	CY057677	H1N1	10/19/2009	USA	A/Wisconsin/629-D00132/2009(H1N
PB2	CY083646	H1N1	10/19/2009	USA	A/San Diego/INS13/2009
PB2	CY083845	H1N1	10/19/2009	USA	A/San Diego/INS14/2009(H1N1)
PB2	CY056914	H1N1	10/19/2009	USA	A/San Diego/INS60/2009(H1N1)
PB2	CY060762	H1N1	10/19/2009	USA	A/Texas/46193632/2009(H1N1)
PB2	CY061250	H1N1	10/19/2009	USA	A/San Diego/INS103/2009(H1N1)
PB2	CY058715	H1N1	10/19/2009	USA	A/Texas/46193311/2009(H1N1)
PB2	CY057261	H1N1	10/19/2009	USA	A/New York/5186/2009(H1N1)
PB2	CY063274	H1N1	10/19/2009	USA	A/Wisconsin/629-D02082/2009(H1N
PB2	CY058707	H1N1	10/19/2009	USA	A/Texas/46192760/2009(H1N1)
PB2	CY058699	H1N1	10/19/2009	USA	A/Texas/461917783/2009(H1N1)
PB2	CY060571	H1N1	10/19/2009	Canada	A/Ontario/29801/2009
PB2	CY057693	H1N1	10/19/2009	USA	A/Wisconsin/629-S1388/2009(H1N1)
PB2	KJ690453	H1N1	10/19/2009	Uganda	A/Uganda/MUWRP-111/2009
PB2	CY056067	H1N1	10/19/2009	USA	A/District of Columbia/INS20/20
PB2	CY056570	H1N1	10/20/2009	USA	A/New York/5227/2009(H1N1)
PB2	CY061258	H1N1	10/20/2009	USA	A/San Diego/INS104/2009(H1N1)
PB2	CY057269	H1N1	10/20/2009	USA	A/New York/5196/2009(H1N1)

PB2	CY123806	H1N1	10/20/2009	Singapore	A/Singapore/ON2224/2009
PB2	HM568045	H1N1	10/20/2009	United Ki	A/England/950/2009
PB2	CY056562	H1N1	10/20/2009	USA	A/New York/5199/2009(H1N1)
PB2	CY056866	H1N1	10/20/2009	USA	A/San Diego/INS15/2009(H1N1)
PB2	CY057285	H1N1	10/20/2009	USA	A/New York/5221/2009(H1N1)
PB2	CY057277	H1N1	10/20/2009	USA	A/New York/5217/2009(H1N1)
PB2	CY056035	H1N1	10/20/2009	USA	A/San Diego/INS16/2009(H1N1)
PB2	CY060770	H1N1	10/20/2009	USA	A/Texas/46201823/2009(H1N1)
PB2	JX625619	H1N1	10/20/2009	United Ki	A/Northern Ireland/94940033/2009
PB2	JX625518	H1N1	10/20/2009	United Ki	A/England/94400003/2009
PB2	JX625588	H1N1	10/20/2009	United Ki	A/Northern Ireland/94620068/2009
PB2	JX625611	H1N1	10/21/2009	United Ki	A/Northern Ireland/94940023/2009
PB2	CY083676	H1N1	10/21/2009	USA	A/San Diego/INS62/2009(H1N1)
PB2	CY057701	H1N1	10/21/2009	USA	A/Wisconsin/629-S1398/2009(H1N1)
PB2	HM568093	H1N1	10/21/2009	United Ki	A/England/766/2009
PB2	CY060778	H1N1	10/21/2009	USA	A/Texas/46214103/2009(H1N1)
PB2	CY057301	H1N1	10/21/2009	USA	A/New York/5276/2009(H1N1)
PB2	CY056099	H1N1	10/21/2009	USA	A/District of Columbia/INS27/20
PB2	CY066558	H1N1	10/21/2009	USA	A/San Diego/INS198/2009(H1N1)
PB2	HM567973	H1N1	10/21/2009	United Ki	A/England/1111/2009
PB2	CY056139	H1N1	10/21/2009	USA	A/San Diego/INS34/2009(H1N1)
PB2	CY066550	H1N1	10/21/2009	USA	A/San Diego/INS197/2009(H1N1)
PB2	CY056922	H1N1	10/21/2009	USA	A/San Diego/INS61/2009(H1N1)
PB2	CY057709	H1N1	10/21/2009	USA	A/Wisconsin/629-S1397/2009(H1N1)
PB2	CY071631	H1N1	10/21/2009	Taiwan	A/Taipei/WR1485T/2009(H1N1)
PB2	HM568053	H1N1	10/21/2009	United Ki	A/England/1116/2009
PB2	CY057309	H1N1	10/22/2009	USA	A/New York/5297/2009(H1N1)
PB2	CY060802	H1N1	10/22/2009	USA	A/Texas/46223444/2009(H1N1)
PB2	CY066566	H1N1	10/22/2009	USA	A/San Diego/INS199/2009(H1N1)
PB2	CY060786	H1N1	10/22/2009	USA	A/Texas/46221665/2009(H1N1)
PB2	CY060794	H1N1	10/22/2009	USA	A/Texas/46222134/2009(H1N1)
PB2	CY057717	H1N1	10/22/2009	USA	A/Wisconsin/629-S1407/2009(H1N1)
PB2	CY060810	H1N1	10/22/2009	USA	A/Texas/46223582/2009(H1N1)
PB2	HM568109	H1N1	10/22/2009	United Ki	A/England/798/2009
PB2	CY057293	H1N1	10/22/2009	USA	A/New York/5271/2009(H1N1)
PB2	CY060818	H1N1	10/22/2009	USA	A/Texas/46224042/2009(H1N1)
PB2	CY065984	H1N1	10/22/2009	Mongolia	A/Ulaanbaatar/6525/2009(H1N1)
PB2	CY089210	H1N1	10/22/2009	USA	A/Boston/594/2009(H1N1)
PB2	CY089202	H1N1	10/22/2009	USA	A/Boston/593/2009(H1N1)
PB2	KC780552	H1N1	10/23/2009	USA	A/West Virginia/12/2009
PB2	CY092943	H1N1	10/23/2009	USA	A/Maryland/NHRC0004/2009
PB2	CY128298	H1N1	10/23/2009	Viet Nam	A/Viet Nam/13032074/2009
PB2	CY056107	H1N1	10/23/2009	USA	A/District of Columbia/INS28/20
PB2	CY065871	H1N1	10/23/2009	Netherlar	A/Netherlands/1493b/2009
PB2	CY058307	H1N1	10/23/2009	USA	A/Wisconsin/629-D02018/2009(H1N
PB2	CY060826	H1N1	10/23/2009	USA	A/Texas/46233104/2009(H1N1)
PB2	CY057317	H1N1	10/23/2009	USA	A/New York/5447/2009(H1N1)
PB2	CY075515	H1N1	10/24/2009	USA	A/Boston/595/2009(H1N1)
PB2	CY060842	H1N1	10/24/2009	USA	A/Texas/46241654/2009(H1N1)
PB2	CY060834	H1N1	10/24/2009	USA	A/Texas/46240925/2009(H1N1)
PB2	CY060579	H1N1	10/24/2009	Canada	A/Ontario/304434/2009
PB2	HQ263287	H1N1	10/24/2009	USA	A/Bethesda/NIH107-D0/2009
PB2	CY128306	H1N1	10/24/2009	Viet Nam	A/Viet Nam/13032076/2009
PB2	CY090023	H1N1	10/25/2009	USA	A/Wisconsin/629-D02330/2009(H1N

PB2	CY092919	H1N1	10/25/2009	USA	A/Maryland/NHRC0001/2009
PB2	CY056147	H1N1	10/26/2009	USA	A/San Diego/INS35/2009(H1N1)
PB2	CY056930	H1N1	10/26/2009	USA	A/San Diego/INS63/2009(H1N1)
PB2	CY056043	H1N1	10/26/2009	USA	A/District of Columbia/INS17/20(
PB2	CY056131	H1N1	10/26/2009	USA	A/District of Columbia/INS33/20(
PB2	CY056187	H1N1	10/26/2009	USA	A/District of Columbia/INS43/20(
PB2	CY064771	H1N1	10/26/2009	USA	A/District of Columbia/INS32/20(
PB2	HM568101	H1N1	10/26/2009	United Ki	A/England/797/2009
PB2	JQ612496	H1N1	10/26/2009	Hungary	A/Hungary/40/2009
PB2	CY058315	H1N1	10/26/2009	USA	A/Wisconsin/629-S1417/2009(H1N1)
PB2	CY058331	H1N1	10/26/2009	USA	A/Wisconsin/629-S1416/2009(H1N1)
PB2	CY058323	H1N1	10/26/2009	USA	A/Wisconsin/629-S1414/2009(H1N1)
PB2	CY056115	H1N1	10/26/2009	USA	A/District of Columbia/INS29/20(
PB2	KC780050	H1N1	10/26/2009	USA	A/Washington/75/2009
PB2	CY058627	H1N1	10/27/2009	USA	A/Wisconsin/629-S1433/2009(H1N1)
PB2	JN187162	H1N1	10/27/2009	Taiwan	A/Taiwan/6200/2009
PB2	CY060587	H1N1	10/27/2009	Canada	A/Ontario/305139/2009
PB2	CY058643	H1N1	10/27/2009	USA	A/Wisconsin/629-S1425/2009(H1N1)
PB2	KC780717	H1N1	10/27/2009	USA	A/Rhode Island/18/2009
PB2	CY060595	H1N1	10/27/2009	Canada	A/Ontario/305598/2009(H1N1)
PB2	CY058635	H1N1	10/27/2009	USA	A/Wisconsin/629-S1431/2009(H1N1)
PB2	CY067642	H1N1	10/27/2009	Norway	A/Oslo/INS219/2009(H1N1)
PB2	KT180691	H1N1	10/27/2009	Singapore	A/Singapore/DMS46/2009
PB2	CY056123	H1N1	10/27/2009	USA	A/District of Columbia/INS31/20(
PB2	CY056059	H1N1	10/27/2009	USA	A/District of Columbia/INS19/20(
PB2	CY056938	H1N1	10/28/2009	USA	A/San Diego/INS65/2009(H1N1)
PB2	CY066822	H1N1	10/28/2009	USA	A/San Diego/INS218/2009
PB2	CY066830	H1N1	10/28/2009	Belgium	A/Antwerp/INS221/2009(H1N1)
PB2	JN187163	H1N1	10/28/2009	Taiwan	A/Taiwan/7873/2009
PB2	HM568077	H1N1	10/28/2009	United Ki	A/England/1213/2009
PB2	JX625549	H1N1	10/28/2009	United Ki	A/England/1397/2009
PB2	CY056051	H1N1	10/28/2009	USA	A/District of Columbia/INS18/20(
PB2	KY924964	H1N1	10/28/2009	Brazil	A/Porto Alegre/LACENRS-3434/200
PB2	AB704459	H1N1	10/29/2009	Japan	A/Tochigi/350/2009
PB2	CY063138	H1N1	10/29/2009	USA	A/California/VRDL97/2009(H1N1)
PB2	CY061274	H1N1	10/29/2009	Belgium	A/Brussels/INS106/2009(H1N1)
PB2	CY066198	H1N1	10/29/2009	USA	A/California/VRDL94/2009(H1N1)
PB2	CY075523	H1N1	10/29/2009	USA	A/Boston/602/2009(H1N1)
PB2	CY063130	H1N1	10/29/2009	USA	A/California/VRDL96/2009(H1N1)
PB2	CY128314	H1N1	10/29/2009	Viet Nam	A/Viet Nam/13032079/2009
PB2	CY060611	H1N1	10/29/2009	Canada	A/Ontario/309862/2009
PB2	CY063146	H1N1	10/29/2009	USA	A/California/VRDL98/2009(H1N1)
PB2	CY060619	H1N1	10/30/2009	Canada	A/Ontario/313762/2009
PB2	JQ173100	H1N1	10/30/2009	China	A/Nanjing/NJU-108/2009
PB2	JN207841	H1N1	10/30/2009	China	A/Zhejiang/89w/2009(H1N1)
PB2	CY056946	H1N1	10/30/2009	Belgium	A/Brussels/INS71/2009(H1N1)
PB2	CY066734	H1N1	10/30/2009	Belgium	A/Brussels/INS206/2009(H1N1)
PB2	CY083853	H1N1	10/30/2009	USA	A/District of Columbia/INS23/20(
PB2	CY083760	H1N1	10/30/2009	Belgium	A/Brussels/INS205/2009(H1N1)
PB2	CY128322	H1N1	10/31/2009	Viet Nam	A/Viet Nam/13032088/2009
PB2	CY065815	H1N1	10/31/2009	Netherlar	A/Netherlands/1715b/2009
PB2	CY058651	H1N1	10/31/2009	USA	A/Wisconsin/629-D02329/2009(H1N
PB2	CY058659	H1N1	10/31/2009	USA	A/Wisconsin/629-D02002/2009(H1N
PB2	CY058667	H1N1	10/31/2009	USA	A/Wisconsin/629-D01913/2009(H1N

PB2	CY095875	H1N1	11/2009	China	A/Hubei/76/2009
PB2	CY095917	H1N1	11/2009	China	A/Zhejiang/81/2009
PB2	CY095851	H1N1	11/2009	China	A/Hubei/73/2009
PB2	CY095867	H1N1	11/2009	China	A/Hubei/75/2009
PB2	CY095859	H1N1	11/2009	China	A/Hubei/74/2009
PB2	CY054627	H1N1	11/01/2009	Russia	A/Russia/4/2009(H1N1)
PB2	CY054635	H1N1	11/01/2009	Russia	A/Russia/12/2009(H1N1)
PB2	CY053678	H1N1	11/01/2009	Russia	A/Russia/14/2009(H1N1)
PB2	KT180692	H1N1	11/01/2009	Singapore	A/Singapore/DMS47/2009
PB2	JX625573	H1N1	11/01/2009	United Ki	A/England/94580011/2009
PB2	CY058675	H1N1	11/01/2009	USA	A/Wisconsin/629-D00193/2009(H1N
PB2	CY053749	H1N1	11/01/2009	Russia	A/Russia/200/2009(H1N1)
PB2	CY053733	H1N1	11/01/2009	Russia	A/Russia/178/2009(H1N1)
PB2	CY053741	H1N1	11/01/2009	Russia	A/Russia/190/2009(H1N1)
PB2	CY053725	H1N1	11/01/2009	Russia	A/Russia/74/2009(H1N1)
PB2	CY053686	H1N1	11/01/2009	Russia	A/Russia/191/2009(H1N1)
PB2	CY054659	H1N1	11/01/2009	Russia	A/Russia/100/2009(H1N1)
PB2	CY054651	H1N1	11/01/2009	Russia	A/Russia/61/2009(H1N1)
PB2	CY054643	H1N1	11/01/2009	Russia	A/Russia/19/2009(H1N1)
PB2	CY123235	H1N1	11/02/2009	Singapore	A/Singapore/GP4218/2009
PB2	CY060627	H1N1	11/02/2009	Canada	A/Ontario/314095/2009
PB2	KC780861	H1N1	11/02/2009	USA	A/Wyoming/16/2009
PB2	CY066886	H1N1	11/02/2009	USA	A/District of Columbia/INS229/2(
PB2	CY075531	H1N1	11/02/2009	USA	A/Boston/606/2009(H1N1)
PB2	CY058275	H1N1	11/02/2009	USA	A/New York/5620/2009(H1N1)
PB2	CY060850	H1N1	11/02/2009	USA	A/Texas/JMS356/2009(H1N1)
PB2	CY066574	H1N1	11/02/2009	USA	A/San Diego/INS201/2009(H1N1)
PB2	CY063282	H1N1	11/02/2009	USA	A/Wisconsin/629-D00888/2009(H1N
PB2	CY058683	H1N1	11/02/2009	USA	A/Wisconsin/629-D00938/2009(H1N
PB2	CY060667	H1N1	11/02/2009	Canada	A/Ontario/315047/2009(H1N1)
PB2	CY056626	H1N1	11/02/2009	USA	A/New York/5988/2009(H1N1)
PB2	CY075539	H1N1	11/02/2009	USA	A/Boston/612/2009(H1N1)
PB2	CY060643	H1N1	11/02/2009	Canada	A/Ontario/314603/2009
PB2	JF714139	H1N1	11/02/2009	South Ko	A/Korea/NAP-1/2009(H1N1)
PB2	HM189309	H1N1	11/02/2009	Russia	A/Chita/CRIE-8/2009
PB2	CY083245	H1N1	11/03/2009	Russia	A/Moscow/WRAIR1627T/2009(H1N1)
PB2	GU931812	H1N1	11/03/2009	Hong Kong	A/Hong Kong/415742M/2009(H1N1)
PB2	CY060651	H1N1	11/03/2009	Canada	A/Ontario/315003/2009
PB2	CY060699	H1N1	11/03/2009	Canada	A/Ontario/315613/2009
PB2	CY066742	H1N1	11/03/2009	Belgium	A/Brussels/INS208/2009(H1N1)
PB2	HM100234	H1N1	11/03/2009	Hong Kong	A/Hong Kong/415742Md/2009(H1N1)
PB2	CY056874	H1N1	11/03/2009	USA	A/District of Columbia/INS44/20(
PB2	CY056227	H1N1	11/03/2009	USA	A/San Diego/INS68/2009(H1N1)
PB2	CY060635	H1N1	11/03/2009	Canada	A/Ontario/314137/2009(H1N1)
PB2	CY056195	H1N1	11/03/2009	USA	A/District of Columbia/INS46/20(
PB2	CY066838	H1N1	11/03/2009	Spain	A/Madrid/INS222/2009
PB2	CY062650	H1N1	11/03/2009	USA	A/District of Columbia/INS45/20(
PB2	CY056586	H1N1	11/03/2009	USA	A/New York/5782/2009(H1N1)
PB2	CY060683	H1N1	11/03/2009	Canada	A/Ontario/315181/2009
PB2	CY060691	H1N1	11/03/2009	Canada	A/Ontario/315187/2009
PB2	CY089218	H1N1	11/03/2009	USA	A/Boston/618/2009
PB2	KJ690523	H1N1	11/03/2009	Uganda	A/Uganda/MUWRP-137/2009
PB2	CY056203	H1N1	11/04/2009	USA	A/San Diego/INS48/2009(H1N1)
PB2	CY056179	H1N1	11/04/2009	USA	A/San Diego/INS42/2009(H1N1)

PB2	CY056634	H1N1	11/04/2009	USA	A/New York/6018/2009(H1N1)
PB2	KC780474	H1N1	11/04/2009	USA	A/Florida/25/2009
PB2	CY066750	H1N1	11/04/2009	Belgium	A/Brussels/INS209/2009(H1N1)
PB2	JX625581	H1N1	11/04/2009	United Ki	A/England/94620043/2009
PB2	KT180693	H1N1	11/04/2009	Singapore	A/Singapore/DMS48/2009
PB2	CY066590	H1N1	11/04/2009	USA	A/San Diego/INS203/2009(H1N1)
PB2	CY066582	H1N1	11/04/2009	USA	A/San Diego/INS202/2009(H1N1)
PB2	CY056163	H1N1	11/04/2009	USA	A/Pensacola/INS38/2009(H1N1)
PB2	CY061266	H1N1	11/04/2009	USA	A/San Diego/INS105/2009(H1N1)
PB2	CY060858	H1N1	11/04/2009	USA	A/Texas/JMS358/2009(H1N1)
PB2	CY062690	H1N1	11/04/2009	USA	A/District of Columbia/INS114/2(
PB2	CY060675	H1N1	11/04/2009	Canada	A/Ontario/315107/2009
PB2	CY060707	H1N1	11/04/2009	Canada	A/Ontario/315637/2009
PB2	CY056155	H1N1	11/04/2009	USA	A/Pensacola/INS37/2009(H1N1)
PB2	CY060659	H1N1	11/04/2009	Canada	A/Ontario/315015/2009
PB2	CY063170	H1N1	11/05/2009	USA	A/California/VRDL101/2009(H1N1)
PB2	CY056243	H1N1	11/05/2009	USA	A/San Diego/INS70/2009(H1N1)
PB2	CY057733	H1N1	11/05/2009	USA	A/Wisconsin/629-D01915/2009(H1N
PB2	CY057725	H1N1	11/05/2009	USA	A/Wisconsin/629-D02317/2009(H1N
PB2	CY066598	H1N1	11/05/2009	USA	A/San Diego/INS204/2009(H1N1)
PB2	CY066206	H1N1	11/05/2009	USA	A/California/VRDL103/2009(H1N1)
PB2	CY057773	H1N1	11/05/2009	USA	A/Wisconsin/629-D01346/2009(H1N
PB2	CY066902	H1N1	11/05/2009	USA	A/District of Columbia/INS231/2(
PB2	CY063154	H1N1	11/05/2009	USA	A/California/VRDL99/2009(H1N1)
PB2	CY063178	H1N1	11/05/2009	USA	A/California/VRDL102/2009(H1N1)
PB2	CY063162	H1N1	11/05/2009	USA	A/California/VRDL100/2009(H1N1)
PB2	CY054675	H1N1	11/05/2009	Russia	A/Russia/180/2009(H1N1)
PB2	CY065823	H1N1	11/05/2009	Netherlar	A/Netherlands/2442b/2009
PB2	CY057749	H1N1	11/05/2009	USA	A/Wisconsin/629-D00151/2009(H1N
PB2	CY057765	H1N1	11/05/2009	USA	A/Wisconsin/629-D02370/2009(H1N
PB2	CY057757	H1N1	11/05/2009	USA	A/Wisconsin/629-D01570/2009(H1N
PB2	CY057741	H1N1	11/05/2009	USA	A/Wisconsin/629-D01140/2009(H1N
PB2	HQ263280	H1N1	11/05/2009	USA	A/Bethesda/NIH106-D14/2009
PB2	CY083861	H1N1	11/05/2009	USA	A/Pensacola/INS39/2009(H1N1)
PB2	CY083728	H1N1	11/05/2009	USA	A/District of Columbia/INS115/2(
PB2	CY083877	H1N1	11/05/2009	USA	A/San Diego/INS49/2009(H1N1)
PB2	CY056235	H1N1	11/05/2009	USA	A/San Diego/INS69/2009(H1N1)
PB2	KT180694	H1N1	11/06/2009	Singapore	A/Singapore/DMS49/2009
PB2	CY056602	H1N1	11/06/2009	USA	A/New York/5916/2009(H1N1)
PB2	KC780753	H1N1	11/06/2009	USA	A/Illinois/15/2009
PB2	CY083869	H1N1	11/06/2009	USA	A/District of Columbia/INS47/20(
PB2	KC261307	H1N1	11/06/2009	Mexico	A/Mexico City/INERPHAC2/2009(H1N
PB2	CY065839	H1N1	11/07/2009	Netherlar	A/Netherlands/2457b/2009
PB2	CY057781	H1N1	11/07/2009	USA	A/Wisconsin/629-D00802/2009(H1N
PB2	KC782167	H1N1	11/07/2009	USA	A/New Mexico/16/2009
PB2	CY060866	H1N1	11/07/2009	USA	A/Texas/JMS359/2009(H1N1)
PB2	CY060882	H1N1	11/07/2009	USA	A/Texas/JMS362/2009(H1N1)
PB2	CY087207	H1N1	11/07/2009	USA	A/Texas/JMS360/2009
PB2	CY089226	H1N1	11/07/2009	USA	A/Boston/630/2009
PB2	CY089234	H1N1	11/07/2009	USA	A/Boston/632/2009
PB2	CY060874	H1N1	11/07/2009	USA	A/Texas/JMS361/2009(H1N1)
PB2	CY060898	H1N1	11/08/2009	USA	A/Texas/JMS364/2009(H1N1)
PB2	CY057789	H1N1	11/08/2009	USA	A/Wisconsin/629-D01014/2009(H1N
PB2	CY060890	H1N1	11/08/2009	USA	A/Texas/JMS363/2009(H1N1)

PB2	CY060906	H1N1	11/08/2009	USA	A/Texas/JMS365/2009(H1N1)
PB2	CY060715	H1N1	11/08/2009	Canada	A/Ontario/320266/2009
PB2	KC782065	H1N1	11/08/2009	USA	A/Wisconsin/55/2009
PB2	CY061561	H1N1	11/08/2009	USA	A/Texas/JMS366/2009(H1N1)
PB2	CY057805	H1N1	11/08/2009	USA	A/Wisconsin/629-D01466/2009(H1N
PB2	CY057813	H1N1	11/08/2009	USA	A/Wisconsin/629-D02392/2009(H1N
PB2	CY089258	H1N1	11/08/2009	USA	A/Boston/637/2009
PB2	CY056658	H1N1	11/08/2009	USA	A/New York/6110/2009(H1N1)
PB2	CY057797	H1N1	11/08/2009	USA	A/Wisconsin/629-D01369/2009(H1N
PB2	CY075547	H1N1	11/09/2009	USA	A/Boston/634/2009(H1N1)
PB2	CY056642	H1N1	11/09/2009	USA	A/New York/6019/2009(H1N1)
PB2	CY066790	H1N1	11/09/2009	USA	A/San Diego/INS214/2009
PB2	CY083901	H1N1	11/09/2009	USA	A/San Diego/INS73/2009(H1N1)
PB2	CY057333	H1N1	11/09/2009	USA	A/New York/6072/2009(H1N1)
PB2	CY056610	H1N1	11/09/2009	USA	A/New York/5931/2009(H1N1)
PB2	CY089250	H1N1	11/09/2009	USA	A/Boston/635/2009
PB2	CY066990	H1N1	11/09/2009	Belgium	A/Brussels/INS243/2009(H1N1)
PB2	CY056594	H1N1	11/09/2009	USA	A/New York/5886/2009(H1N1)
PB2	KC780793	H1N1	11/09/2009	USA	A/Maryland/31/2009
PB2	CY083885	H1N1	11/09/2009	USA	A/San Diego/INS50/2009(H1N1)
PB2	KC782246	H1N1	11/09/2009	USA	A/Minnesota/40/2009
PB2	KC780384	H1N1	11/09/2009	USA	A/Washington/62/2009
PB2	CY089242	H1N1	11/09/2009	USA	A/Boston/633/2009
PB2	AB704491	H1N1	11/09/2009	Japan	A/Yamagata/473/2009
PB2	CY083893	H1N1	11/09/2009	USA	A/San Diego/INS72/2009(H1N1)
PB2	CY056171	H1N1	11/09/2009	USA	A/Pensacola/INS40/2009(H1N1)
PB2	CY056618	H1N1	11/09/2009	USA	A/New York/5976/2009(H1N1)
PB2	KC780200	H1N1	11/09/2009	USA	A/Maine/13/2009
PB2	CY066774	H1N1	11/10/2009	USA	A/Pensacola/INS212/2009
PB2	CY062794	H1N1	11/10/2009	Denmark	A/Hvidovre/INS139/2009(H1N1)
PB2	CY066766	H1N1	11/10/2009	USA	A/Pensacola/INS211/2009
PB2	HQ652617	H1N1	11/10/2009	China	A/Jiangsu/S62/2009
PB2	CY053624	H1N1	11/10/2009	Russia	A/Russia/165/2009(H1N1)
PB2	CY128330	H1N1	11/10/2009	Viet Nam	A/Viet Nam/13032093/2009
PB2	CY063589	H1N1	11/10/2009	Norway	A/Oslo/INS110/2009(H1N1)
PB2	HM189349	H1N1	11/10/2009	Russia	A/Lipetsk/CRIE-BVV/2009
PB2	HQ652616	H1N1	11/10/2009	China	A/Jiangsu/S61/2009
PB2	CY065855	H1N1	11/10/2009	Netherlar	A/Netherlands/1324/2009(H1N1)
PB2	CY066758	H1N1	11/10/2009	USA	A/Pensacola/INS210/2009
PB2	CY066798	H1N1	11/10/2009	USA	A/San Diego/INS215/2009
PB2	CY062818	H1N1	11/11/2009	Denmark	A/Odense/INS143/2009(H1N1)
PB2	CY066846	H1N1	11/11/2009	Spain	A/Madrid/INS223/2009
PB2	CY063290	H1N1	11/11/2009	USA	A/Wisconsin/629-D02008/2009(H1N
PB2	JN032403	H1N1	11/11/2009	China	A/Changchun/01/2009
PB2	CY084445	H1N1	11/11/2009	USA	A/New York/6064/2009
PB2	CY066862	H1N1	11/12/2009	USA	A/District of Columbia/INS226/2(
PB2	CY066806	H1N1	11/12/2009	USA	A/San Diego/INS216/2009
PB2	CY067650	H1N1	11/12/2009	Spain	A/Madrid/INS224/2009(H1N1)
PB2	CY066870	H1N1	11/12/2009	USA	A/District of Columbia/INS227/2(
PB2	CY075555	H1N1	11/12/2009	USA	A/Boston/648/2009(H1N1)
PB2	CY057349	H1N1	11/12/2009	USA	A/San Diego/INS75/2009(H1N1)
PB2	CY063298	H1N1	11/12/2009	USA	A/Wisconsin/629-D00292/2009(H1N
PB2	CY061569	H1N1	11/12/2009	USA	A/Texas/JMS367/2009(H1N1)
PB2	CY054667	H1N1	11/12/2009	Russia	A/Russia/171/2009(H1N1)

PB2	HM173606	H1N1	11/12/2009	Russia	A/Blagovechensk/01/2009(H1N1)
PB2	CY066814	H1N1	11/12/2009	USA	A/San Diego/INS217/2009
PB2	CY089266	H1N1	11/12/2009	USA	A/Boston/650/2009
PB2	CY062658	H1N1	11/12/2009	USA	A/San Diego/INS74/2009(H1N1)
PB2	KC781074	H1N1	11/12/2009	USA	A/Oklahoma/05/2009
PB2	KT180695	H1N1	11/12/2009	Singapore	A/Singapore/DMS50/2009
PB2	CY061282	H1N1	11/12/2009	USA	A/Pensacola/INS107/2009(H1N1)
PB2	CY062682	H1N1	11/13/2009	Spain	A/Madrid/INS113/2009(H1N1)
PB2	CY087215	H1N1	11/13/2009	USA	A/Texas/JMS368/2009
PB2	CY128378	H1N1	11/13/2009	Viet Nam	A/Viet Nam/11032001/2009
PB2	JX625596	H1N1	11/13/2009	United Ki	A/England/94720002/2009
PB2	HM773207	H1N1	11/13/2009	China	A/Jiangsu/2/2009(H1N1)
PB2	CY075579	H1N1	11/13/2009	USA	A/Boston/658/2009(H1N1)
PB2	CY075571	H1N1	11/13/2009	USA	A/Boston/655/2009(H1N1)
PB2	CY067030	H1N1	11/13/2009	Germany	A/Bochum/INS250/2009(H1N1)
PB2	CY066878	H1N1	11/13/2009	USA	A/District of Columbia/INS228/2(
PB2	CY060914	H1N1	11/13/2009	USA	A/Texas/JMS369/2009(H1N1)
PB2	CY075563	H1N1	11/13/2009	USA	A/Boston/653/2009(H1N1)
PB2	CY066854	H1N1	11/13/2009	Spain	A/Madrid/INS225/2009
PB2	CY073488	H1N1	11/13/2009	Germany	A/Bochum/INS248/2009(H1N1)
PB2	CY067022	H1N1	11/13/2009	Germany	A/Bochum/INS249/2009(H1N1)
PB2	CY073085	H1N1	11/13/2009	Turkey	A/Ankara/WRAIR1435T/2009
PB2	KC780772	H1N1	11/13/2009	USA	A/Arkansas/09/2009
PB2	CY060922	H1N1	11/14/2009	USA	A/Texas/JMS370/2009(H1N1)
PB2	CY128346	H1N1	11/14/2009	Viet Nam	A/Viet Nam/13032097/2009
PB2	CY089274	H1N1	11/14/2009	USA	A/Boston/657/2009
PB2	GU451255	H1N1	11/14/2009	Russia	A/Tver/IIV2969/2009(H1N1)
PB2	CY128338	H1N1	11/14/2009	Viet Nam	A/Viet Nam/13032096/2009
PB2	CY057821	H1N1	11/14/2009	USA	A/Wisconsin/629-D01826/2009(H1N
PB2	CY128426	H1N1	11/14/2009	Viet Nam	A/Viet Nam/12032001/2009
PB2	CY060930	H1N1	11/14/2009	USA	A/Texas/JMS371/2009(H1N1)
PB2	CY057829	H1N1	11/14/2009	USA	A/Wisconsin/629-D00965/2009(H1N
PB2	CY128354	H1N1	11/15/2009	Viet Nam	A/Viet Nam/13032100/2009
PB2	CY128474	H1N1	11/15/2009	Viet Nam	A/Viet Nam/001-1020/2009
PB2	CY066214	H1N1	11/15/2009	USA	A/California/VRDL104/2009(H1N1)
PB2	KT180696	H1N1	11/15/2009	Singapore	A/Singapore/DMS51/2009
PB2	CY065122	H1N1	11/15/2009	Netherlar	A/Netherlands/2464b/2009
PB2	CY089306	H1N1	11/15/2009	USA	A/Boston/671/2009
PB2	CY066246	H1N1	11/15/2009	USA	A/California/VRDL108/2009(H1N1)
PB2	CY075603	H1N1	11/15/2009	USA	A/Boston/672/2009(H1N1)
PB2	CY057837	H1N1	11/15/2009	USA	A/Wisconsin/629-D00911/2009(H1N
PB2	CY057853	H1N1	11/15/2009	USA	A/Wisconsin/629-D02060/2009(H1N
PB2	CY057861	H1N1	11/15/2009	USA	A/Wisconsin/629-D00295/2009(H1N
PB2	CY057869	H1N1	11/15/2009	USA	A/Wisconsin/629-D00936/2009(H1N
PB2	CY057845	H1N1	11/15/2009	USA	A/Wisconsin/629-D00128/2009(H1N
PB2	CY066238	H1N1	11/15/2009	USA	A/California/VRDL107/2009(H1N1)
PB2	CY066230	H1N1	11/15/2009	USA	A/California/VRDL106/2009(H1N1)
PB2	CY056690	H1N1	11/15/2009	USA	A/New York/6257/2009(H1N1)
PB2	CY066222	H1N1	11/15/2009	USA	A/California/VRDL105/2009(H1N1)
PB2	CY060938	H1N1	11/15/2009	USA	A/Texas/JMS372/2009(H1N1)
PB2	CY089298	H1N1	11/16/2009	USA	A/Boston/670/2009
PB2	CY075595	H1N1	11/16/2009	USA	A/Boston/666/2009(H1N1)
PB2	HQ698624	H1N1	11/16/2009	China	A/Beijing/16/2009
PB2	CY056706	H1N1	11/16/2009	USA	A/New York/6293/2009(H1N1)

PB2	CY067006	H1N1	11/16/2009	Germany	A/Munich/INS246/2009
PB2	KP637403	H1N1	11/16/2009	Thailand	A/Thailand/MR08303/2009
PB2	CY089314	H1N1	11/16/2009	USA	A/Boston/673/2009
PB2	CY065138	H1N1	11/16/2009	Netherlar	A/Netherlands/2143/2009
PB2	CY056674	H1N1	11/16/2009	USA	A/New York/6213/2009(H1N1)
PB2	CY056666	H1N1	11/16/2009	USA	A/New York/6182/2009(H1N1)
PB2	CY075587	H1N1	11/16/2009	USA	A/Boston/663/2009(H1N1)
PB2	CY058339	H1N1	11/16/2009	USA	A/Wisconsin/629-D01347/2009(H1N
PB2	CY089290	H1N1	11/16/2009	USA	A/Boston/667/2009
PB2	CY057877	H1N1	11/16/2009	USA	A/Wisconsin/629-D01661/2009(H1N
PB2	AB704427	H1N1	11/16/2009	Japan	A/Gunma/263/2009
PB2	CY089282	H1N1	11/16/2009	USA	A/Boston/665/2009
PB2	GU371258	H1N1	11/16/2009	Russia	A/Orenburg/IIV2974/2009(H1N1)
PB2	CY056682	H1N1	11/16/2009	USA	A/New York/6214/2009(H1N1)
PB2	KP637803	H1N1	11/17/2009	Thailand	A/Thailand/SS08207/2009
PB2	CY060723	H1N1	11/17/2009	Canada	A/Ontario/328474/2009
PB2	CY075619	H1N1	11/17/2009	USA	A/Boston/682/2009(H1N1)
PB2	CY065831	H1N1	11/17/2009	Netherlar	A/Netherlands/2243/2009
PB2	CY058355	H1N1	11/17/2009	USA	A/Wisconsin/629-D01582/2009(H1N
PB2	CY058347	H1N1	11/17/2009	USA	A/Wisconsin/629-D00503/2009(H1N
PB2	CY069121	H1N1	11/17/2009	Spain	A/Madrid/INS296/2009(H1N1)
PB2	CY089338	H1N1	11/17/2009	USA	A/Boston/683/2009
PB2	CY066782	H1N1	11/17/2009	USA	A/Pensacola/INS213/2009
PB2	CY062674	H1N1	11/17/2009	USA	A/Pensacola/INS109/2009(H1N1)
PB2	CY056714	H1N1	11/17/2009	USA	A/New York/6300/2009(H1N1)
PB2	CY123848	H1N1	11/17/2009	Singapore	A/Singapore/ON2313/2009
PB2	CY089330	H1N1	11/17/2009	USA	A/Boston/680/2009
PB2	CY066726	H1N1	11/17/2009	Spain	A/Madrid/INS295/2009(H1N1)
PB2	CY056698	H1N1	11/17/2009	USA	A/New York/6292/2009(H1N1)
PB2	CY061290	H1N1	11/17/2009	USA	A/Pensacola/INS108/2009(H1N1)
PB2	CY089322	H1N1	11/17/2009	USA	A/Boston/678/2009
PB2	CY075611	H1N1	11/17/2009	USA	A/Boston/681/2009(H1N1)
PB2	CY056722	H1N1	11/18/2009	USA	A/New York/6346/2009(H1N1)
PB2	CY058363	H1N1	11/18/2009	USA	A/Wisconsin/629-D02489/2009(H1N
PB2	CY088736	H1N1	11/18/2009	Netherlar	A/Netherlands/2223b/2009
PB2	CY089346	H1N1	11/18/2009	USA	A/Boston/685/2009
PB2	HM567981	H1N1	11/18/2009	United Ki	A/England/869/2009
PB2	CY067658	H1N1	11/18/2009	Belgium	A/Brussels/INS244/2009(H1N1)
PB2	CY128362	H1N1	11/18/2009	Viet Nam	A/Viet Nam/15032003/2009
PB2	CY089354	H1N1	11/18/2009	USA	A/Boston/686/2009
PB2	CY073748	H1N1	11/18/2009	Denmark	A/Copenhagen/INS433/2009(H1N1)
PB2	CY066998	H1N1	11/18/2009	Belgium	A/Brussels/INS245/2009(H1N1)
PB2	GU560015	H1N1	11/18/2009	Russia	A/Tomsk/05/2009(H1N1)
PB2	CY123257	H1N1	11/18/2009	Singapore	A/Singapore/GP4381/2009
PB2	CY063314	H1N1	11/19/2009	USA	A/Wisconsin/629-D01572/2009(H1N
PB2	CY066942	H1N1	11/19/2009	Denmark	A/Aarhus/INS236/2009(H1N1)
PB2	CY066710	H1N1	11/19/2009	Austria	A/Vienna/INS291/2009(H1N1)
PB2	CY065863	H1N1	11/19/2009	Netherlar	A/Netherlands/2229/2009
PB2	CY063306	H1N1	11/19/2009	USA	A/Wisconsin/629-D00859/2009(H1N
PB2	AB704419	H1N1	11/19/2009	Japan	A/Gunma/262/2009
PB2	CY100485	H1N1	11/19/2009	Mexico	A/Mexico City/INER14/2009
PB2	CY062754	H1N1	11/19/2009	Spain	A/Madrid/INS129/2009(H1N1)
PB2	CY066638	H1N1	11/19/2009	Germany	A/Bonn/INS277/2009(H1N1)
PB2	CY071230	H1N1	11/19/2009	Norway	A/Oslo/INS404/2009(H1N1)

PB2	CY062746	H1N1	11/19/2009	Germany	A/Bonn/INS127/2009(H1N1)
PB2	HM568029	H1N1	11/20/2009	United Ki	A/England/902/2009
PB2	HM230700	H1N1	11/20/2009	China	A/Zhoushan/1/2009(H1N1)
PB2	HM568013	H1N1	11/20/2009	United Ki	A/England/872/2009
PB2	HM754228	H1N1	11/20/2009	China	A/Jiangsu/1/2009(H1N1)
PB2	HM567965	H1N1	11/20/2009	United Ki	A/England/876/2009
PB2	CY066686	H1N1	11/20/2009	Denmark	A/Hvidovre/INS288/2009(H1N1)
PB2	CY066646	H1N1	11/20/2009	Germany	A/Bonn/INS278/2009(H1N1)
PB2	CY062802	H1N1	11/20/2009	Denmark	A/Hvidovre/INS141/2009(H1N1)
PB2	CY083744	H1N1	11/20/2009	Spain	A/Madrid/INS130/2009(H1N1)
PB2	CY089362	H1N1	11/20/2009	USA	A/Boston/690/2009
PB2	CY056954	H1N1	11/20/2009	Denmark	A/Aarhus/INS82/2009(H1N1)
PB2	CY066654	H1N1	11/20/2009	Germany	A/Bonn/INS279/2009(H1N1)
PB2	CY063605	H1N1	11/20/2009	Germany	A/Bonn/INS128/2009(H1N1)
PB2	CY060946	H1N1	11/21/2009	USA	A/Texas/JMS373/2009(H1N1)
PB2	CY067046	H1N1	11/21/2009	Denmark	A/Aarhus/INS252/2009(H1N1)
PB2	CY089370	H1N1	11/21/2009	USA	A/Boston/691/2009
PB2	CY067038	H1N1	11/21/2009	Denmark	A/Aarhus/INS251/2009
PB2	AB704507	H1N1	11/21/2009	Japan	A/Yamaguchi/217/2009
PB2	CY088592	H1N1	11/21/2009	USA	A/Boston/694/2009
PB2	GU560023	H1N1	11/22/2009	Russia	A/Tomsk/06/2009(H1N1)
PB2	CY056730	H1N1	11/22/2009	USA	A/New York/6473/2009(H1N1)
PB2	CY128370	H1N1	11/22/2009	Viet Nam	A/Viet Nam/15032004/2009
PB2	CY057893	H1N1	11/22/2009	USA	A/Wisconsin/629-D00533/2009(H1N
PB2	CY057901	H1N1	11/22/2009	USA	A/Wisconsin/629-D00908/2009(H1N
PB2	CY067054	H1N1	11/22/2009	Denmark	A/Aarhus/INS253/2009(H1N1)
PB2	CY084461	H1N1	11/22/2009	USA	A/New York/6418/2009
PB2	HM567957	H1N1	11/23/2009	United Ki	A/England/865/2009
PB2	CY066950	H1N1	11/23/2009	Denmark	A/Aarhus/INS237/2009(H1N1)
PB2	CY056762	H1N1	11/23/2009	USA	A/New York/6663/2009(H1N1)
PB2	CY075627	H1N1	11/23/2009	USA	A/Boston/698/2009(H1N1)
PB2	CY083917	H1N1	11/23/2009	Denmark	A/Aalborg/INS132/2009(H1N1)
PB2	CY056850	H1N1	11/23/2009	USA	A/New York/6868/2009(H1N1)
PB2	CY056738	H1N1	11/23/2009	USA	A/New York/6546/2009(H1N1)
PB2	CY069153	H1N1	11/23/2009	Germany	A/Frankfurt/INS301/2009(H1N1)
PB2	CY057909	H1N1	11/23/2009	USA	A/Wisconsin/629-D02448/2009(H1N
PB2	CY057925	H1N1	11/23/2009	USA	A/Wisconsin/629-D01330/2009(H1N
PB2	CY057917	H1N1	11/23/2009	USA	A/Wisconsin/629-D00134/2009(H1N
PB2	CY066894	H1N1	11/23/2009	USA	A/District of Columbia/INS230/2(
PB2	CY066910	H1N1	11/23/2009	USA	A/District of Columbia/INS232/2(
PB2	CY065807	H1N1	11/23/2009	Netherlar	A/Netherlands/2445b/2009
PB2	CY072253	H1N1	11/23/2009	Denmark	A/Odense/INS307/2009(H1N1)
PB2	CY058723	H1N1	11/23/2009	USA	A/Texas/JMS375/2009(H1N1)
PB2	HM568021	H1N1	11/23/2009	United Ki	A/England/862/2009
PB2	HM568005	H1N1	11/23/2009	United Ki	A/England/854/2009
PB2	CY066958	H1N1	11/24/2009	Denmark	A/Aarhus/INS238/2009(H1N1)
PB2	CY071254	H1N1	11/24/2009	Spain	A/Madrid/INS407/2009(H1N1)
PB2	CY066262	H1N1	11/24/2009	USA	A/California/VRDL110/2009(H1N1)
PB2	KT180698	H1N1	11/24/2009	Singapore	A/Singapore/DMS53/2009
PB2	KC780702	H1N1	11/24/2009	USA	A/California/41/2009
PB2	CY072261	H1N1	11/24/2009	Denmark	A/Odense/INS308/2009(H1N1)
PB2	CY062698	H1N1	11/24/2009	Denmark	A/Aarhus/INS116/2009(H1N1)
PB2	CY058731	H1N1	11/24/2009	USA	A/Texas/JMS376/2009(H1N1)
PB2	CY069129	H1N1	11/24/2009	Spain	A/Madrid/INS298/2009(H1N1)

PB2	CY066278	H1N1	11/24/2009	USA	A/California/VRDL112/2009(H1N1)
PB2	CY057933	H1N1	11/24/2009	USA	A/Wisconsin/629-D01190/2009(H1N
PB2	AB704467	H1N1	11/24/2009	Japan	A/Tochigi/445/2009
PB2	CY066718	H1N1	11/24/2009	Austria	A/Vienna/INS293/2009(H1N1)
PB2	CY057941	H1N1	11/24/2009	USA	A/Wisconsin/629-D00968/2009(H1N
PB2	CY056754	H1N1	11/24/2009	USA	A/New York/6607/2009(H1N1)
PB2	CY056786	H1N1	11/24/2009	USA	A/New York/6675/2009(H1N1)
PB2	CY066662	H1N1	11/24/2009	Spain	A/Madrid/INS281/2009(H1N1)
PB2	CY066270	H1N1	11/24/2009	USA	A/California/VRDL111/2009(H1N1)
PB2	CY066254	H1N1	11/24/2009	USA	A/California/VRDL109/2009(H1N1)
PB2	CY066286	H1N1	11/24/2009	USA	A/California/VRDL113/2009(H1N1)
PB2	HM567997	H1N1	11/24/2009	United Ki	A/England/900/2009
PB2	CY066918	H1N1	11/24/2009	USA	A/Pensacola/INS233/2009
PB2	CY066982	H1N1	11/24/2009	Denmark	A/Aarhus/INS242/2009(H1N1)
PB2	CY084469	H1N1	11/24/2009	USA	A/New York/6537/2009
PB2	CY092445	H1N1	11/24/2009	Australia	A/Sydney/DD3-02/2009(H1N1)
PB2	HQ263294	H1N1	11/24/2009	USA	A/Bethesda/NIH107-D31/2009
PB2	HM189317	H1N1	11/25/2009	Russia	A/Arkhangelsk/CRIE-GNY/2009
PB2	HM101148	H1N1	11/25/2009	Russia	A/Tver/IIV-183/2009(H1N1)
PB2	CY067190	H1N1	11/25/2009	Austria	A/Vienna/INS294/2009
PB2	CY072229	H1N1	11/25/2009	Germany	A/Frankfurt/INS302/2009(H1N1)
PB2	CY056834	H1N1	11/25/2009	USA	A/New York/6846/2009(H1N1)
PB2	AB704443	H1N1	11/25/2009	Japan	A/Gunma/287/2009
PB2	CY066694	H1N1	11/25/2009	Denmark	A/Hvidovre/INS289/2009(H1N1)
PB2	CY056826	H1N1	11/25/2009	USA	A/New York/6810/2009(H1N1)
PB2	GU562457	H1N1	11/25/2009	Russia	A/Perm/01/2009(H1N1)
PB2	CY067014	H1N1	11/25/2009	Germany	A/Munich/INS247/2009
PB2	AB704435	H1N1	11/25/2009	Japan	A/Gunma/267/2009
PB2	HM567989	H1N1	11/25/2009	United Ki	A/England/859/2009
PB2	JQ173161	H1N1	11/25/2009	Finland	A/Helsinki/P18/2009(H1N1)
PB2	HM568037	H1N1	11/25/2009	United Ki	A/England/898/2009
PB2	HM567949	H1N1	11/25/2009	United Ki	A/England/857/2009
PB2	CY066966	H1N1	11/26/2009	Denmark	A/Aarhus/INS240/2009(H1N1)
PB2	KT180699	H1N1	11/26/2009	Singapore	A/Singapore/DMS54/2009
PB2	CY057949	H1N1	11/26/2009	USA	A/Wisconsin/629-D00629/2009(H1N
PB2	CY062810	H1N1	11/26/2009	Austria	A/Vienna/INS142/2009(H1N1)
PB2	CY069137	H1N1	11/26/2009	Spain	A/Madrid/INS299/2009(H1N1)
PB2	CY062762	H1N1	11/26/2009	Spain	A/Madrid/INS131/2009(H1N1)
PB2	CY060954	H1N1	11/26/2009	USA	A/Texas/JMS379/2009(H1N1)
PB2	CY058747	H1N1	11/26/2009	USA	A/Texas/JMS378/2009(H1N1)
PB2	CY058739	H1N1	11/26/2009	USA	A/Texas/JMS377/2009(H1N1)
PB2	CY066974	H1N1	11/26/2009	Denmark	A/Aarhus/INS241/2009(H1N1)
PB2	CY072237	H1N1	11/26/2009	Germany	A/Frankfurt/INS303/2009(H1N1)
PB2	CY088620	H1N1	11/26/2009	Germany	A/Frankfurt/INS93/2009
PB2	CY056746	H1N1	11/26/2009	USA	A/New York/6571/2009(H1N1)
PB2	CY073756	H1N1	11/26/2009	Denmark	A/Copenhagen/INS434/2009(H1N1)
PB2	KJ856064	H1N1	11/27/2009	Sri Lanka	A/Sri Lanka/2005/2009
PB2	CY060962	H1N1	11/27/2009	USA	A/Texas/JMS380/2009(H1N1)
PB2	CY057957	H1N1	11/27/2009	USA	A/Wisconsin/629-D00050/2009(H1N
PB2	AB704499	H1N1	11/27/2009	Japan	A/Yamagata/674/2009
PB2	CY062706	H1N1	11/27/2009	Denmark	A/Aarhus/INS118/2009(H1N1)
PB2	CY057965	H1N1	11/27/2009	USA	A/Wisconsin/629-D00970/2009(H1N
PB2	CY075635	H1N1	11/27/2009	USA	A/Boston/701/2009(H1N1)
PB2	CY066702	H1N1	11/27/2009	Denmark	A/Hvidovre/INS290/2009(H1N1)

PB2	CY067062	H1N1	11/28/2009	Denmark	A/Aarhus/INS254/2009(H1N1)
PB2	GU592904	H1N1	11/28/2009	Kyrgyzsta	A/Bishkek/03/2009(H1N1)
PB2	CY060970	H1N1	11/28/2009	USA	A/Texas/JMS381/2009(H1N1)
PB2	CY060978	H1N1	11/28/2009	USA	A/Texas/JMS382/2009(H1N1)
PB2	CY057973	H1N1	11/29/2009	USA	A/Wisconsin/629-D01528/2009(H1N
PB2	CY060986	H1N1	11/29/2009	USA	A/Texas/JMS383/2009(H1N1)
PB2	CY056810	H1N1	11/29/2009	USA	A/New York/6808/2009(H1N1)
PB2	CY057981	H1N1	11/29/2009	USA	A/Wisconsin/629-D01434/2009(H1N
PB2	CY088600	H1N1	11/29/2009	USA	A/Boston/702/2009
PB2	CY056858	H1N1	11/29/2009	USA	A/New York/6909/2009(H1N1)
PB2	CY066670	H1N1	11/30/2009	Germany	A/Cologne/INS286/2009(H1N1)
PB2	CY056818	H1N1	11/30/2009	USA	A/New York/6809/2009(H1N1)
PB2	CY069225	H1N1	11/30/2009	Germany	A/Munich/INS363/2009(H1N1)
PB2	CY063621	H1N1	11/30/2009	Germany	A/Cologne/INS136/2009(H1N1)
PB2	KJ856071	H1N1	11/30/2009	Sri Lanka	A/Sri Lanka/2008/2009
PB2	CY056770	H1N1	11/30/2009	USA	A/New York/6668/2009(H1N1)
PB2	CY060994	H1N1	11/30/2009	USA	A/Texas/JMS384/2009(H1N1)
PB2	CY069145	H1N1	11/30/2009	Spain	A/Madrid/INS300/2009(H1N1)
PB2	CY072245	H1N1	11/30/2009	Germany	A/Frankfurt/INS305/2009(H1N1)
PB2	KT180701	H1N1	11/30/2009	Singapore	A/Singapore/DMS55/2009
PB2	KJ856235	H1N1	12/01/2009	Sri Lanka	A/Sri Lanka/24588/2009
PB2	CY057191	H1N1	12/01/2009	Mongolia	A/Dundgobi/9746/2009(H1N1)
PB2	CY057989	H1N1	12/01/2009	USA	A/Wisconsin/629-D01412/2009(H1N
PB2	CY065847	H1N1	12/01/2009	Netherlar	A/Netherlands/2636/2009
PB2	CY123291	H1N1	12/01/2009	Singapore	A/Singapore/GP4556/2009
PB2	CY073504	H1N1	12/01/2009	Germany	A/Frankfurt/INS304/2009(H1N1)
PB2	KC780183	H1N1	12/01/2009	USA	A/Montana/22/2009
PB2	KJ856079	H1N1	12/01/2009	Sri Lanka	A/Sri Lanka/2009/2009
PB2	CY056778	H1N1	12/01/2009	USA	A/New York/6669/2009(H1N1)
PB2	CY072269	H1N1	12/02/2009	Poland	A/Warsaw/INS310/2009(H1N1)
PB2	CY075643	H1N1	12/02/2009	USA	A/Boston/703/2009(H1N1)
PB2	CY128434	H1N1	12/02/2009	Viet Nam	A/Viet Nam/12032005/2009
PB2	CY058005	H1N1	12/02/2009	USA	A/Wisconsin/629-D02299/2009(H1N
PB2	CY056842	H1N1	12/02/2009	USA	A/New York/6864/2009(H1N1)
PB2	CY056986	H1N1	12/02/2009	Denmark	A/Copenhagen/INS98/2009(H1N1)
PB2	CY062770	H1N1	12/02/2009	Spain	A/Madrid/INS134/2009(H1N1)
PB2	CY063613	H1N1	12/02/2009	Denmark	A/Aalborg/INS133/2009(H1N1)
PB2	CY057997	H1N1	12/02/2009	USA	A/Wisconsin/629-D00935/2009(H1N
PB2	CY065903	H1N1	12/02/2009	Netherlar	A/Netherlands/2822/2009
PB2	CY071094	H1N1	12/02/2009	Germany	A/Bochum/INS376/2009(H1N1)
PB2	CY071086	H1N1	12/02/2009	Germany	A/Bochum/INS375/2009(H1N1)
PB2	CY071198	H1N1	12/02/2009	Germany	A/Frankfurt/INS399/2009(H1N1)
PB2	AB704515	H1N1	12/02/2009	Japan	A/Yamaguchi/247/2009
PB2	CY072277	H1N1	12/02/2009	Poland	A/Warsaw/INS311/2009(H1N1)
PB2	HM568117	H1N1	12/02/2009	United Ki	A/England/941/2009
PB2	CY123297	H1N1	12/02/2009	Singapore	A/Singapore/GP4581/2009
PB2	CY083925	H1N1	12/03/2009	Poland	A/Warsaw/INS147/2009(H1N1)
PB2	CY075651	H1N1	12/03/2009	USA	A/Boston/704/2009(H1N1)
PB2	CY062834	H1N1	12/03/2009	Poland	A/Warsaw/INS146/2009(H1N1)
PB2	KT180702	H1N1	12/03/2009	Singapore	A/Singapore/DMS56/2009
PB2	CY072293	H1N1	12/03/2009	Poland	A/Warsaw/INS313/2009(H1N1)
PB2	CY123876	H1N1	12/03/2009	Singapore	A/Singapore/ON2383/2009
PB2	CY072285	H1N1	12/03/2009	Poland	A/Warsaw/INS312/2009(H1N1)
PB2	CY088608	H1N1	12/04/2009	USA	A/Boston/705/2009

PB2	HM157255	H1N1	12/04/2009	Russia	A/Yaroslavl/IIV-196/2009(H1N1)
PB2	KC780778	H1N1	12/04/2009	USA	A/Alaska/47/2009
PB2	CY066310	H1N1	12/04/2009	USA	A/California/VRDL116/2009(H1N1)
PB2	GU727830	H1N1	12/04/2009	Russia	A/IIV-Anadyr/177/2009(H1N1)
PB2	CY066318	H1N1	12/04/2009	USA	A/California/VRDL117/2009(H1N1)
PB2	CY057357	H1N1	12/04/2009	Germany	A/Hamburg/INS92/2009(H1N1)
PB2	CY062778	H1N1	12/04/2009	Spain	A/Madrid/INS135/2009(H1N1)
PB2	CY066326	H1N1	12/04/2009	USA	A/California/VRDL118/2009(H1N1)
PB2	CY069233	H1N1	12/04/2009	Germany	A/Munich/INS364/2009(H1N1)
PB2	CY061002	H1N1	12/04/2009	USA	A/Texas/JMS385/2009(H1N1)
PB2	CY056802	H1N1	12/04/2009	USA	A/New York/6776/2009(H1N1)
PB2	CY065791	H1N1	12/04/2009	Netherlar	A/Netherlands/2629/2009
PB2	CY083556	H1N1	12/04/2009	Hungary	A/Budapest/WRAIR2411N/2009
PB2	CY066294	H1N1	12/04/2009	USA	A/California/VRDL114/2009(H1N1)
PB2	CY066302	H1N1	12/04/2009	USA	A/California/VRDL115/2009(H1N1)
PB2	KC780305	H1N1	12/04/2009	USA	A/Pennsylvania/31/2009
PB2	JN187164	H1N1	12/04/2009	Taiwan	A/Taiwan/6341/2009
PB2	HM104484	H1N1	12/05/2009	Russia	A/Yaroslavl/IIV-198/2009(H1N1)
PB2	AB704523	H1N1	12/05/2009	Japan	A/Yamaguchi/248/2009
PB2	CY056794	H1N1	12/05/2009	USA	A/New York/6775/2009(H1N1)
PB2	KC781012	H1N1	12/06/2009	USA	A/Arizona/20/2009
PB2	CY061010	H1N1	12/06/2009	USA	A/Texas/JMS386/2009(H1N1)
PB2	CY065082	H1N1	12/06/2009	USA	A/New York/7421/2009(H1N1)
PB2	CY084453	H1N1	12/06/2009	USA	A/New York/6902/2009
PB2	CY061945	H1N1	12/06/2009	USA	A/New York/6941/2009(H1N1)
PB2	CY056970	H1N1	12/07/2009	Denmark	A/Copenhagen/INS95/2009(H1N1)
PB2	KC781089	H1N1	12/07/2009	USA	A/Iowa/14/2009
PB2	CY061218	H1N1	12/07/2009	USA	A/New York/6771/2009(H1N1)
PB2	KT180704	H1N1	12/07/2009	Singapore	A/Singapore/DMS57/2009
PB2	CY058013	H1N1	12/07/2009	USA	A/Wisconsin/629-D01473/2009(H1N
PB2	CY062826	H1N1	12/07/2009	Denmark	A/Copenhagen/INS144/2009(H1N1)
PB2	CY061226	H1N1	12/07/2009	USA	A/New York/6772/2009(H1N1)
PB2	CY058403	H1N1	12/07/2009	USA	A/Wisconsin/629-D01591/2009(H1N
PB2	CY073732	H1N1	12/07/2009	Denmark	A/Aalborg/INS283/2009(H1N1)
PB2	CY072309	H1N1	12/07/2009	Poland	A/Warsaw/INS315/2009(H1N1)
PB2	CY065895	H1N1	12/07/2009	Netherlar	A/Netherlands/2815/2009
PB2	CY056994	H1N1	12/07/2009	Poland	A/Warsaw/INS100/2009(H1N1)
PB2	CY063629	H1N1	12/07/2009	USA	A/New York/INS150/2009(H1N1)
PB2	CY061953	H1N1	12/07/2009	USA	A/New York/6943/2009(H1N1)
PB2	CY072301	H1N1	12/07/2009	Poland	A/Warsaw/INS314/2009(H1N1)
PB2	KC780597	H1N1	12/07/2009	USA	A/Texas/75/2009
PB2	CY061601	H1N1	12/07/2009	USA	A/New York/6806/2009(H1N1)
PB2	LN846547	H1N1	12/08/2009	United Ki	A/England/9/2009
PB2	CY058387	H1N1	12/08/2009	USA	A/Wisconsin/629-D00780/2009(H1N
PB2	LN845779	H1N1	12/08/2009	United Ki	A/England/8/2009
PB2	KC780840	H1N1	12/08/2009	USA	A/Oregon/42/2009
PB2	CY058021	H1N1	12/08/2009	USA	A/Wisconsin/629-D00672/2009(H1N
PB2	CY061921	H1N1	12/08/2009	USA	A/New York/6907/2009(H1N1)
PB2	CY065106	H1N1	12/08/2009	USA	A/New York/7426/2009(H1N1)
PB2	CY058029	H1N1	12/08/2009	USA	A/Wisconsin/629-D00401/2009(H1N
PB2	CY066926	H1N1	12/08/2009	USA	A/Pensacola/INS234/2009
PB2	CY067174	H1N1	12/08/2009	Germany	A/Bonn/INS280/2009(H1N1)
PB2	CY066934	H1N1	12/08/2009	USA	A/Pensacola/INS235/2009
PB2	CY072317	H1N1	12/08/2009	Poland	A/Warsaw/INS316/2009(H1N1)

PB2	CY061018	H1N1	12/08/2009	USA	A/Texas/JMS387/2009(H1N1)
PB2	CY058371	H1N1	12/09/2009	USA	A/Wisconsin/629-D00689/2009(H1N
PB2	CY069217	H1N1	12/09/2009	Germany	A/Berlin/INS361/2009(H1N1)
PB2	CY069241	H1N1	12/09/2009	Germany	A/Munich/INS365/2009(H1N1)
PB2	CY058395	H1N1	12/09/2009	USA	A/Wisconsin/629-D00147/2009(H1N
PB2	JF800142	H1N1	12/09/2009	China	A/Nanchang/8002/2009
PB2	CY072557	H1N1	12/09/2009	Poland	A/Wroclaw/INS437/2009(H1N1)
PB2	CY072549	H1N1	12/09/2009	Poland	A/Wroclaw/INS436/2009(H1N1)
PB2	CY063597	H1N1	12/09/2009	Greece	A/Athens/INS122/2009(H1N1)
PB2	CY128386	H1N1	12/09/2009	Viet Nam	A/Viet Nam/11032006/2009
PB2	CY067182	H1N1	12/09/2009	Germany	A/Bonn/INS284/2009(H1N1)
PB2	CY063050	H1N1	12/10/2009	Germany	A/Bochum/INS187/2009(H1N1)
PB2	CY061913	H1N1	12/10/2009	USA	A/New York/6903/2009(H1N1)
PB2	CY065992	H1N1	12/10/2009	Mongolia	A/Ulgii/9911/2009(H1N1)
PB2	CY058037	H1N1	12/10/2009	USA	A/Wisconsin/629-D00636/2009(H1N
PB2	CY066678	H1N1	12/10/2009	Belgium	A/Tessenderlo/INS287/2009(H1N1)
PB2	KT180705	H1N1	12/10/2009	Singapore	A/Singapore/DMS58/2009
PB2	CY062786	H1N1	12/10/2009	Belgium	A/Tessenderlo/INS138/2009(H1N1)
PB2	CY056978	H1N1	12/10/2009	Denmark	A/Copenhagen/INS96/2009(H1N1)
PB2	CY061026	H1N1	12/11/2009	USA	A/Texas/JMS388/2009(H1N1)
PB2	CY061929	H1N1	12/11/2009	USA	A/New York/6937/2009(H1N1)
PB2	CY061961	H1N1	12/11/2009	USA	A/New York/6945/2009(H1N1)
PB2	CY064779	H1N1	12/11/2009	Germany	A/Berlin/INS170/2009(H1N1)
PB2	CY071102	H1N1	12/11/2009	Spain	A/Barcelona/INS379/2009(H1N1)
PB2	CY061937	H1N1	12/11/2009	USA	A/New York/6939/2009(H1N1)
PB2	CY063637	H1N1	12/11/2009	USA	A/New York/INS151/2009(H1N1)
PB2	CY061985	H1N1	12/11/2009	USA	A/New York/6977/2009(H1N1)
PB2	CY123886	H1N1	12/11/2009	Singapore	A/Singapore/ON2400/2009
PB2	CY083933	H1N1	12/11/2009	Poland	A/Warsaw/INS148/2009(H1N1)
PB2	CY083941	H1N1	12/11/2009	Poland	A/Warsaw/INS149/2009(H1N1)
PB2	CY083804	H1N1	12/12/2009	Germany	A/Berlin/INS362/2009(H1N1)
PB2	CY058379	H1N1	12/12/2009	USA	A/Wisconsin/629-D01244/2009(H1N
PB2	CY067094	H1N1	12/13/2009	Greece	A/Athens/INS261/2009(H1N1)
PB2	CY056962	H1N1	12/13/2009	Greece	A/Athens/INS85/2009(H1N1)
PB2	CY072357	H1N1	12/13/2009	Greece	A/Athens/INS322/2009(H1N1)
PB2	CY067126	H1N1	12/13/2009	Greece	A/Athens/INS266/2009(H1N1)
PB2	CY072349	H1N1	12/13/2009	Greece	A/Athens/INS320/2009(H1N1)
PB2	CY067102	H1N1	12/13/2009	Greece	A/Athens/INS262/2009(H1N1)
PB2	CY067118	H1N1	12/13/2009	Greece	A/Athens/INS264/2009(H1N1)
PB2	CY067110	H1N1	12/13/2009	Greece	A/Athens/INS263/2009(H1N1)
PB2	CY062730	H1N1	12/13/2009	Greece	A/Athens/INS125/2009(H1N1)
PB2	CY062722	H1N1	12/13/2009	Greece	A/Athens/INS124/2009(H1N1)
PB2	CY067086	H1N1	12/13/2009	Greece	A/Athens/INS260/2009(H1N1)
PB2	CY067078	H1N1	12/13/2009	Greece	A/Athens/INS259/2009(H1N1)
PB2	CY062714	H1N1	12/13/2009	Greece	A/Athens/INS123/2009(H1N1)
PB2	CY067070	H1N1	12/13/2009	Greece	A/Athens/INS257/2009(H1N1)
PB2	CY083909	H1N1	12/13/2009	Greece	A/Athens/INS86/2009(H1N1)
PB2	CY058459	H1N1	12/14/2009	USA	A/Wisconsin/629-D01846/2009(H1N
PB2	CY061977	H1N1	12/14/2009	USA	A/New York/6976/2009(H1N1)
PB2	GU592912	H1N1	12/14/2009	Russia	A/Magadan/02/2009(H1N1)
PB2	CY058427	H1N1	12/14/2009	USA	A/Wisconsin/629-D01904/2009(H1N
PB2	KC683492	H1N1	12/14/2009	China	A/Liaoning/14/2009
PB2	CY062001	H1N1	12/14/2009	USA	A/New York/7020/2009(H1N1)
PB2	CY061969	H1N1	12/14/2009	USA	A/New York/6949/2009(H1N1)

PB2	KT180706	H1N1	12/14/2009	Singapore	A/Singapore/DMS59/2009
PB2	CY066350	H1N1	12/15/2009	USA	A/California/VRDL121/2009(H1N1)
PB2	CY067158	H1N1	12/15/2009	Greece	A/Athens/INS270/2009(H1N1)
PB2	CY067142	H1N1	12/15/2009	Greece	A/Athens/INS268/2009(H1N1)
PB2	CY123892	H1N1	12/15/2009	Singapore	A/Singapore/ON2416/2009
PB2	AB704451	H1N1	12/15/2009	Japan	A/Gunma/293/2009
PB2	CY066334	H1N1	12/15/2009	USA	A/California/VRDL119/2009(H1N1)
PB2	CY067150	H1N1	12/15/2009	Greece	A/Athens/INS269/2009(H1N1)
PB2	CY066366	H1N1	12/15/2009	USA	A/California/VRDL123/2009(H1N1)
PB2	CY072333	H1N1	12/15/2009	USA	A/Newark/INS318/2009(H1N1)
PB2	CY073884	H1N1	12/15/2009	Greece	A/Athens/INS323/2009(H1N1)
PB2	CY073496	H1N1	12/15/2009	Greece	A/Athens/INS271/2009(H1N1)
PB2	CY066630	H1N1	12/15/2009	Greece	A/Athens/INS275/2009(H1N1)
PB2	CY066622	H1N1	12/15/2009	Greece	A/Athens/INS274/2009(H1N1)
PB2	CY066606	H1N1	12/15/2009	Greece	A/Athens/INS272/2009(H1N1)
PB2	CY058475	H1N1	12/15/2009	USA	A/Wisconsin/629-D00337/2009(H1N
PB2	CY066358	H1N1	12/15/2009	USA	A/California/VRDL122/2009(H1N1)
PB2	CY066342	H1N1	12/15/2009	USA	A/California/VRDL120/2009(H1N1)
PB2	CY062738	H1N1	12/15/2009	Greece	A/Athens/INS126/2009(H1N1)
PB2	CY062666	H1N1	12/15/2009	Greece	A/Athens/INS87/2009(H1N1)
PB2	CY063058	H1N1	12/15/2009	Belgium	A/Tessenderlo/INS191/2009(H1N1)
PB2	CY066614	H1N1	12/15/2009	Greece	A/Athens/INS273/2009(H1N1)
PB2	CY063026	H1N1	12/15/2009	Spain	A/Madrid/INS184/2009(H1N1)
PB2	CY058435	H1N1	12/15/2009	USA	A/Wisconsin/629-D01752/2009(H1N
PB2	CY058483	H1N1	12/15/2009	USA	A/Wisconsin/629-D02424/2009(H1N
PB2	CY058451	H1N1	12/16/2009	USA	A/Wisconsin/629-D02073/2009(H1N
PB2	CY061993	H1N1	12/16/2009	USA	A/New York/7019/2009(H1N1)
PB2	CY062946	H1N1	12/16/2009	Spain	A/Terrassa/INS172/2009(H1N1)
PB2	CY061034	H1N1	12/16/2009	USA	A/Texas/JMS389/2009(H1N1)
PB2	CY071262	H1N1	12/16/2009	Spain	A/Madrid/INS408/2009(H1N1)
PB2	CY071110	H1N1	12/16/2009	Belgium	A/Tessenderlo/INS380/2009(H1N1)
PB2	CY062009	H1N1	12/16/2009	USA	A/New York/7036/2009(H1N1)
PB2	CY067134	H1N1	12/16/2009	Greece	A/Athens/INS267/2009(H1N1)
PB2	KT180707	H1N1	12/16/2009	Singapore	A/Singapore/DMS60/2009
PB2	CY058467	H1N1	12/16/2009	USA	A/Wisconsin/629-D00996/2009(H1N
PB2	CY058411	H1N1	12/16/2009	USA	A/Wisconsin/629-D01038/2009(H1N
PB2	CY058443	H1N1	12/17/2009	USA	A/Wisconsin/629-D01556/2009(H1N
PB2	CY072469	H1N1	12/17/2009	Greece	A/Athens/INS341/2009(H1N1)
PB2	CY072325	H1N1	12/17/2009	USA	A/New York/INS317/2009(H1N1)
PB2	CY128394	H1N1	12/17/2009	Viet Nam	A/Viet Nam/11032010/2009
PB2	CY072477	H1N1	12/17/2009	Greece	A/Athens/INS342/2009(H1N1)
PB2	CY067166	H1N1	12/17/2009	Greece	A/Athens/INS276/2009(H1N1)
PB2	CY062954	H1N1	12/17/2009	Spain	A/Terrassa/INS173/2009(H1N1)
PB2	CY072461	H1N1	12/17/2009	Greece	A/Athens/INS340/2009(H1N1)
PB2	CY062858	H1N1	12/17/2009	Greece	A/Athens/INS158/2009(H1N1)
PB2	CY058419	H1N1	12/18/2009	USA	A/Wisconsin/629-D00459/2009(H1N
PB2	AB704531	H1N1	12/18/2009	Japan	A/Yamaguchi/273/2009
PB2	CY072485	H1N1	12/18/2009	Greece	A/Athens/INS344/2009(H1N1)
PB2	CY062866	H1N1	12/18/2009	Greece	A/Athens/INS159/2009(H1N1)
PB2	JN187165	H1N1	12/18/2009	Taiwan	A/Taiwan/11706/2009
PB2	CY062938	H1N1	12/18/2009	Germany	A/Berlin/INS171/2009(H1N1)
PB2	CY062874	H1N1	12/18/2009	Greece	A/Athens/INS160/2009(H1N1)
PB2	CY072493	H1N1	12/18/2009	Greece	A/Athens/INS345/2009(H1N1)
PB2	CY063661	H1N1	12/18/2009	Greece	A/Athens/INS157/2009(H1N1)

PB2	GU592896	H1N1	12/18/2009	Russia	A/Barnaul/04/2009(H1N1)
PB2	CY062882	H1N1	12/19/2009	Greece	A/Athens/INS161/2009(H1N1)
PB2	HM230708	H1N1	12/19/2009	China	A/Zhoushan/52/2009(H1N1)
PB2	CY089613	H1N1	12/19/2009	China	A/Nanchang/8008/2009
PB2	CY069161	H1N1	12/20/2009	Greece	A/Athens/INS352/2009(H1N1)
PB2	CY072509	H1N1	12/20/2009	Greece	A/Athens/INS347/2009(H1N1)
PB2	CY072365	H1N1	12/20/2009	Greece	A/Athens/INS325/2009(H1N1)
PB2	CY062898	H1N1	12/20/2009	Greece	A/Athens/INS163/2009(H1N1)
PB2	CY062906	H1N1	12/20/2009	Greece	A/Athens/INS164/2009(H1N1)
PB2	CY061042	H1N1	12/20/2009	USA	A/Texas/JMS390/2009(H1N1)
PB2	CY065098	H1N1	12/20/2009	USA	A/New York/7425/2009(H1N1)
PB2	CY072517	H1N1	12/20/2009	Greece	A/Athens/INS348/2009(H1N1)
PB2	CY073512	H1N1	12/20/2009	Greece	A/Athens/INS350/2009(H1N1)
PB2	CY072373	H1N1	12/20/2009	Greece	A/Athens/INS327/2009(H1N1)
PB2	CY062890	H1N1	12/20/2009	Greece	A/Athens/INS162/2009(H1N1)
PB2	CY072501	H1N1	12/20/2009	Greece	A/Athens/INS346/2009(H1N1)
PB2	CY072525	H1N1	12/20/2009	Greece	A/Athens/INS349/2009(H1N1)
PB2	CY062025	H1N1	12/21/2009	USA	A/New York/7216/2009(H1N1)
PB2	CY063002	H1N1	12/21/2009	Estonia	A/Tallinn/INS181/2009(H1N1)
PB2	KT180708	H1N1	12/21/2009	Singapore	A/Singapore/DMS61/2009
PB2	CY069177	H1N1	12/21/2009	Greece	A/Athens/INS354/2009(H1N1)
PB2	CY062914	H1N1	12/21/2009	Greece	A/Athens/INS165/2009(H1N1)
PB2	CY088628	H1N1	12/21/2009	Denmark	A/Odense/INS176/2009
PB2	CY069169	H1N1	12/21/2009	Greece	A/Athens/INS353/2009(H1N1)
PB2	CY066374	H1N1	12/22/2009	USA	A/California/VRDL124/2009(H1N1)
PB2	CY066382	H1N1	12/22/2009	USA	A/California/VRDL125/2009(H1N1)
PB2	CY069249	H1N1	12/22/2009	Austria	A/Vienna/INS366/2009(H1N1)
PB2	CY069185	H1N1	12/22/2009	Greece	A/Athens/INS355/2009(H1N1)
PB2	CY066398	H1N1	12/22/2009	USA	A/California/VRDL127/2009(H1N1)
PB2	CY066390	H1N1	12/22/2009	USA	A/California/VRDL126/2009(H1N1)
PB2	CY089621	H1N1	12/22/2009	China	A/Nanchang/8011/2009
PB2	CY066406	H1N1	12/22/2009	USA	A/California/VRDL128/2009(H1N1)
PB2	CY071118	H1N1	12/22/2009	Germany	A/Wurzburg/INS381/2009(H1N1)
PB2	CY071070	H1N1	12/23/2009	Estonia	A/Tallinn/INS372/2009(H1N1)
PB2	CY061050	H1N1	12/23/2009	USA	A/Texas/JMS391/2009(H1N1)
PB2	CY128402	H1N1	12/23/2009	Viet Nam	A/Viet Nam/11032016/2009
PB2	CY062970	H1N1	12/23/2009	Spain	A/Terrassa/INS175/2009(H1N1)
PB2	CY065090	H1N1	12/23/2009	USA	A/New York/7424/2009(H1N1)
PB2	LN867649	H1N1	12/23/2009	United Ki	A/England/01220639/2009
PB2	CY128410	H1N1	12/23/2009	Viet Nam	A/Viet Nam/11032017/2009
PB2	CY062017	H1N1	12/23/2009	USA	A/New York/7107/2009(H1N1)
PB2	CY071206	H1N1	12/23/2009	Germany	A/Frankfurt/INS401/2009(H1N1)
PB2	CY062962	H1N1	12/24/2009	Spain	A/Terrassa/INS174/2009(H1N1)
PB2	KT180711	H1N1	12/25/2009	Singapore	A/Singapore/DMS62/2009
PB2	CY072381	H1N1	12/25/2009	Greece	A/Athens/INS329/2009(H1N1)
PB2	CY061058	H1N1	12/25/2009	USA	A/Texas/JMS392/2009(H1N1)
PB2	CY071126	H1N1	12/25/2009	Germany	A/Wurzburg/INS382/2009(H1N1)
PB2	CY062930	H1N1	12/26/2009	Greece	A/Athens/INS167/2009(H1N1)
PB2	CY062922	H1N1	12/26/2009	Greece	A/Athens/INS166/2009(H1N1)
PB2	CY062041	H1N1	12/26/2009	USA	A/New York/0259/2009(H1N1)
PB2	CY069193	H1N1	12/26/2009	Greece	A/Athens/INS357/2009(H1N1)
PB2	CY072397	H1N1	12/26/2009	Greece	A/Athens/INS332/2009(H1N1)
PB2	CY072389	H1N1	12/26/2009	Greece	A/Athens/INS331/2009(H1N1)
PB2	CY073013	H1N1	12/26/2009	Greece	A/Athens/INS330/2009(H1N1)

PB2	CY069201	H1N1	12/26/2009	Greece	A/Athens/INS358/2009(H1N1)
PB2	CY069209	H1N1	12/26/2009	Greece	A/Athens/INS359/2009(H1N1)
PB2	CY062049	H1N1	12/27/2009	USA	A/New York/0352/2009(H1N1)
PB2	CY061066	H1N1	12/27/2009	USA	A/Texas/JMS393/2009(H1N1)
PB2	CY062978	H1N1	12/28/2009	Denmark	A/Odense/INS177/2009(H1N1)
PB2	CY072341	H1N1	12/28/2009	USA	A/Newark/INS319/2009(H1N1)
PB2	KT180713	H1N1	12/28/2009	Singapore	A/Singapore/DMS63/2009
PB2	KC780319	H1N1	12/28/2009	USA	A/Georgia/25/2009
PB2	CY062033	H1N1	12/28/2009	USA	A/New York/7236/2009(H1N1)
PB2	CY072405	H1N1	12/28/2009	Greece	A/Athens/INS333/2009(H1N1)
PB2	CY071246	H1N1	12/29/2009	Poland	A/Warsaw/INS406/2009(H1N1)
PB2	CY064787	H1N1	12/29/2009	Spain	A/Barcelona/INS190/2009(H1N1)
PB2	JN187166	H1N1	12/29/2009	Taiwan	A/Taiwan/1017/2009
PB2	CY072413	H1N1	12/29/2009	Greece	A/Athens/INS334/2009(H1N1)
PB2	CY071238	H1N1	12/29/2009	Poland	A/Warsaw/INS405/2009(H1N1)
PB2	CY063645	H1N1	12/29/2009	Greece	A/Athens/INS153/2009(H1N1)
PB2	CY063653	H1N1	12/29/2009	Greece	A/Athens/INS154/2009(H1N1)
PB2	CY062850	H1N1	12/30/2009	Greece	A/Athens/INS156/2009(H1N1)
PB2	CY062057	H1N1	12/30/2009	USA	A/New York/0357/2009(H1N1)
PB2	CY061082	H1N1	12/30/2009	USA	A/Texas/JMS395/2009(H1N1)
PB2	CY066438	H1N1	12/30/2009	USA	A/California/VRDL132/2009(H1N1)
PB2	CY128418	H1N1	12/30/2009	Viet Nam	A/Viet Nam/11032021/2009
PB2	CY063066	H1N1	12/30/2009	Belgium	A/Tessenderlo/INS192/2009(H1N1)
PB2	CY066414	H1N1	12/30/2009	USA	A/California/VRDL129/2009(H1N1)
PB2	CY063034	H1N1	12/30/2009	Spain	A/Madrid/INS185/2009(H1N1)
PB2	CY063042	H1N1	12/30/2009	Spain	A/Madrid/INS186/2009(H1N1)
PB2	CY061074	H1N1	12/30/2009	USA	A/Texas/JMS394/2009(H1N1)
PB2	CY061090	H1N1	12/30/2009	USA	A/Texas/JMS397/2009(H1N1)
PB2	CY072437	H1N1	12/30/2009	Greece	A/Athens/INS337/2009(H1N1)
PB2	CY072445	H1N1	12/30/2009	Greece	A/Athens/INS338/2009(H1N1)
PB2	CY072453	H1N1	12/30/2009	Greece	A/Athens/INS339/2009(H1N1)
PB2	CY072429	H1N1	12/30/2009	Greece	A/Athens/INS336/2009(H1N1)
PB2	CY066446	H1N1	12/30/2009	USA	A/California/VRDL133/2009(H1N1)
PB2	CY066422	H1N1	12/30/2009	USA	A/California/VRDL130/2009(H1N1)
PB2	KC780665	H1N1	12/30/2009	USA	A/Nebraska/23/2009
PB2	CY062065	H1N1	12/30/2009	USA	A/New York/0461/2009(H1N1)
PB2	HM568125	H1N1	12/30/2009	United Ki	A/England/938/2009
PB2	CY062842	H1N1	12/30/2009	Greece	A/Athens/INS155/2009(H1N1)
PB2	LN846364	H1N1	12/30/2009	United Ki	A/England/WTSI1778/2009
PB2	CY066430	H1N1	12/30/2009	USA	A/California/VRDL131/2009(H1N1)
PB2	CY072421	H1N1	12/30/2009	Greece	A/Athens/INS335/2009(H1N1)
PB2	CY061098	H1N1	12/30/2009	USA	A/Texas/JMS398/2009(H1N1)
PB2	CY061106	H1N1	12/31/2009	USA	A/Texas/JMS399/2009(H1N1)
PB2	CY061577	H1N1	12/31/2009	USA	A/Texas/JMS400/2009(H1N1)
PB2	CY123319	H1N1	12/31/2009	Singapore	A/Singapore/GP4915/2009
PB2	CY071134	H1N1	12/31/2009	Greece	A/Athens/INS383/2009(H1N1)
PB2	KT180715	H1N1	12/31/2009	Singapore	A/Singapore/DMS64/2009
PB2	CY120021	H1N1	01/2010	Mexico	A/Mexico/InDRECTRLA/2010
PB2	KC780540	H1N1	01/01/2010	USA	A/New Jersey/01/2010
PB2	CY065074	H1N1	01/02/2010	USA	A/New York/7420/2010(H1N1)
PB2	CY061585	H1N1	01/03/2010	USA	A/Texas/JMS401/2010(H1N1)
PB2	CY061593	H1N1	01/04/2010	USA	A/Texas/JMS403/2010(H1N1)
PB2	CY096593	H1N1	01/04/2010	USA	A/District of Columbia/INS525/2(
PB2	JX308946	H1N1	01/04/2010	Singapore	A/Singapore/GP10/2010

PB2	GU562465	H1N1	01/04/2010	Russia	A/Karasuk/01/2010(H1N1)
PB2	KC781898	H1N1	01/04/2010	USA	A/Alabama/01/2010
PB2	KT180716	H1N1	01/04/2010	Singapore	A/Singapore/DMS65/2010
PB2	CY065879	H1N1	01/04/2010	Netherlar	A/Netherlands/2631b/2010
PB2	CY061114	H1N1	01/04/2010	USA	A/Texas/JMS402/2010(H1N1)
PB2	CY063010	H1N1	01/04/2010	Estonia	A/Tallinn/INS182/2010(H1N1)
PB2	KC782013	H1N1	01/05/2010	USA	A/Iowa/01/2010
PB2	CY073780	H1N1	01/05/2010	Austria	A/Vienna/INS369/2010(H1N1)
PB2	CY062986	H1N1	01/05/2010	Germany	A/Bonn/INS178/2010(H1N1)
PB2	CY071062	H1N1	01/05/2010	Austria	A/Vienna/INS368/2010(H1N1)
PB2	CY062994	H1N1	01/05/2010	Austria	A/Vienna/INS179/2010(H1N1)
PB2	JX308954	H1N1	01/05/2010	Singapore	A/Singapore/GP33/2010
PB2	KC780228	H1N1	01/05/2010	USA	A/Colorado/02/2010
PB2	KC780414	H1N1	01/06/2010	USA	A/Florida/01/2010
PB2	JX308962	H1N1	01/06/2010	Singapore	A/Singapore/GP64/2010
PB2	CY075066	H1N1	01/06/2010	Greece	A/Athens/INS392/2010(H1N1)
PB2	CY075074	H1N1	01/06/2010	Greece	A/Athens/INS393/2010(H1N1)
PB2	CY072533	H1N1	01/07/2010	Estonia	A/Tallinn/INS374/2010(H1N1)
PB2	CY063018	H1N1	01/07/2010	Estonia	A/Tallinn/INS183/2010(H1N1)
PB2	CY071078	H1N1	01/07/2010	Estonia	A/Tallinn/INS373/2010(H1N1)
PB2	KT180718	H1N1	01/07/2010	Singapore	A/Singapore/DMS66/2010
PB2	CY071214	H1N1	01/08/2010	Germany	A/Frankfurt/INS402/2010(H1N1)
PB2	CY061122	H1N1	01/08/2010	USA	A/Texas/JMS404/2010(H1N1)
PB2	CY061130	H1N1	01/09/2010	USA	A/Texas/JMS405/2010(H1N1)
PB2	CY066454	H1N1	01/09/2010	USA	A/California/VRDL1/2010(H1N1)
PB2	CY061138	H1N1	01/10/2010	USA	A/Texas/JMS406/2010(H1N1)
PB2	CY071222	H1N1	01/11/2010	Germany	A/Frankfurt/INS403/2010(H1N1)
PB2	CY061146	H1N1	01/11/2010	USA	A/Texas/JMS407/2010(H1N1)
PB2	CY071166	H1N1	01/11/2010	Greece	A/Athens/INS394/2010(H1N1)
PB2	KT180720	H1N1	01/11/2010	Singapore	A/Singapore/DMS67/2010
PB2	CY066462	H1N1	01/11/2010	USA	A/California/VRDL2/2010(H1N1)
PB2	CY061154	H1N1	01/12/2010	USA	A/Texas/JMS408/2010(H1N1)
PB2	JQ612504	H1N1	01/12/2010	Hungary	A/Hungary/2/2010
PB2	KC780893	H1N1	01/12/2010	USA	A/Nevada/01/2010
PB2	CY062073	H1N1	01/12/2010	USA	A/New York/1680/2010(H1N1)
PB2	AB704483	H1N1	01/12/2010	Japan	A/Tochigi/10/2010
PB2	KP637667	H1N1	01/12/2010	Thailand	A/Thailand/SP08358/2010
PB2	CY071142	H1N1	01/13/2010	Greece	A/Athens/INS389/2010(H1N1)
PB2	AB704475	H1N1	01/13/2010	Japan	A/Tochigi/2/2010
PB2	KT180722	H1N1	01/14/2010	Singapore	A/Singapore/DMS68/2010
PB2	LN867801	H1N1	01/14/2010	United Ki	A/England/01220740/2010
PB2	CY072541	H1N1	01/14/2010	USA	A/New York/INS432/2010(H1N1)
PB2	CY071150	H1N1	01/14/2010	Greece	A/Athens/INS390/2010(H1N1)
PB2	JX309014	H1N1	01/15/2010	Singapore	A/Singapore/GP256/2010
PB2	CY062081	H1N1	01/15/2010	USA	A/New York/1796/2010(H1N1)
PB2	KT180723	H1N1	01/16/2010	Singapore	A/Singapore/DMS69/2010
PB2	CY080312	H1N1	01/18/2010	Thailand	A/Thailand/H1255/2010
PB2	CY071302	H1N1	01/18/2010	Greece	A/Athens/INS417/2010(H1N1)
PB2	LN846435	H1N1	01/18/2010	United Ki	A/England/32/2010
PB2	CY062089	H1N1	01/18/2010	USA	A/New York/1998/2010(H1N1)
PB2	CY062097	H1N1	01/18/2010	USA	A/New York/1999/2010(H1N1)
PB2	CY066470	H1N1	01/19/2010	USA	A/California/VRDL3/2010(H1N1)
PB2	CY062105	H1N1	01/19/2010	USA	A/New York/2175/2010(H1N1)
PB2	CY167779	H1N1	01/19/2010	USA	A/Tennessee/F1035/2010

PB2	CY062113	H1N1	01/20/2010	USA	A/New York/2372/2010(H1N1)
PB2	CY062121	H1N1	01/20/2010	USA	A/New York/2598/2010(H1N1)
PB2	CY061162	H1N1	01/20/2010	USA	A/Texas/JMS409/2010(H1N1)
PB2	JX308990	H1N1	01/20/2010	Singapore	A/Singapore/GP329/2010
PB2	CY062137	H1N1	01/20/2010	USA	A/New York/2876/2010(H1N1)
PB2	KC780579	H1N1	01/21/2010	USA	A/Virginia/01/2010
PB2	JX309022	H1N1	01/21/2010	Singapore	A/Singapore/GP368/2010
PB2	CY071174	H1N1	01/21/2010	Greece	A/Athens/INS396/2010(H1N1)
PB2	KT180725	H1N1	01/22/2010	Singapore	A/Singapore/DMS71/2010
PB2	CY062129	H1N1	01/22/2010	USA	A/New York/2648/2010(H1N1)
PB2	CY062153	H1N1	01/22/2010	USA	A/New York/2963/2010(H1N1)
PB2	CY061170	H1N1	01/22/2010	USA	A/Texas/JMS410/2010(H1N1)
PB2	CY062161	H1N1	01/23/2010	USA	A/New York/2971/2010(H1N1)
PB2	KC780491	H1N1	01/24/2010	USA	A/Wisconsin/01/2010
PB2	CY071190	H1N1	01/24/2010	Greece	A/Athens/INS398/2010(H1N1)
PB2	CY071182	H1N1	01/24/2010	Greece	A/Athens/INS397/2010(H1N1)
PB2	CY063210	H1N1	01/25/2010	USA	A/California/VRDL13/2010(H1N1)
PB2	CY063218	H1N1	01/25/2010	USA	A/California/VRDL14/2010(H1N1)
PB2	CY062169	H1N1	01/25/2010	USA	A/New York/3230/2010(H1N1)
PB2	JX309030	H1N1	01/25/2010	Singapore	A/Singapore/GP413/2010
PB2	CY071310	H1N1	01/25/2010	Greece	A/Athens/INS419/2010(H1N1)
PB2	KT180726	H1N1	01/25/2010	Singapore	A/Singapore/DMS72/2010
PB2	JX309038	H1N1	01/25/2010	Singapore	A/Singapore/GP398/2010
PB2	CY061178	H1N1	01/25/2010	USA	A/Texas/JMS411/2010(H1N1)
PB2	CY062145	H1N1	01/26/2010	USA	A/New York/2960/2010(H1N1)
PB2	CY063194	H1N1	01/26/2010	USA	A/California/VRDL11/2010(H1N1)
PB2	CY083029	H1N1	01/26/2010	Senegal	A/Dakar/WRAIR0019T/2010
PB2	CY083532	H1N1	01/26/2010	Mexico	A/Mexico City/WRAIR1752N/2010(H
PB2	CY062193	H1N1	01/26/2010	USA	A/New York/3251/2010(H1N1)
PB2	JX309046	H1N1	01/26/2010	Singapore	A/Singapore/GP449/2010(H1N1)
PB2	CY062177	H1N1	01/26/2010	USA	A/New York/3236/2010(H1N1)
PB2	CY063202	H1N1	01/27/2010	USA	A/California/VRDL12/2010(H1N1)
PB2	CY065026	H1N1	01/27/2010	USA	A/New York/3834/2010(H1N1)
PB2	CY092645	H1N1	01/27/2010	Australia	A/Sydney/DD3-37/2010
PB2	CY062185	H1N1	01/27/2010	USA	A/New York/3250/2010(H1N1)
PB2	JX309062	H1N1	01/27/2010	Singapore	A/Singapore/GP489/2010(H1N1)
PB2	CY071318	H1N1	01/27/2010	Greece	A/Athens/INS420/2010(H1N1)
PB2	CY065034	H1N1	01/27/2010	USA	A/New York/3835/2010(H1N1)
PB2	KC780655	H1N1	01/27/2010	USA	A/South Carolina/01/2010
PB2	JX309070	H1N1	01/28/2010	Singapore	A/Singapore/GP512/2010(H1N1)
PB2	CY065042	H1N1	01/29/2010	USA	A/New York/3866/2010(H1N1)
PB2	CY071270	H1N1	01/30/2010	Greece	A/Athens/INS411/2010(H1N1)
PB2	KT180729	H1N1	01/31/2010	Singapore	A/Singapore/DMS74/2010
PB2	CY065018	H1N1	02/01/2010	USA	A/New York/3683/2010(H1N1)
PB2	KC780607	H1N1	02/01/2010	USA	A/California/02/2010
PB2	CY065010	H1N1	02/01/2010	USA	A/New York/3682/2010(H1N1)
PB2	CY066494	H1N1	02/01/2010	USA	A/California/VRDL7/2010(H1N1)
PB2	CY061186	H1N1	02/01/2010	USA	A/Texas/JMS412/2010(H1N1)
PB2	CY065002	H1N1	02/01/2010	USA	A/New York/3681/2010(H1N1)
PB2	KT180738	H1N1	02/02/2010	Singapore	A/Singapore/DMS82/2010
PB2	CY064994	H1N1	02/02/2010	USA	A/California/VRDL6/2010(H1N1)
PB2	CY071278	H1N1	02/02/2010	Greece	A/Athens/INS412/2010(H1N1)
PB2	KC780532	H1N1	02/02/2010	USA	A/Florida/02/2010
PB2	JX309153	H1N1	02/02/2010	Singapore	A/Singapore/GP567/2010

PB2	CY065058	H1N1	02/03/2010	USA	A/New York/4662/2010(H1N1)
PB2	KC780696	H1N1	02/03/2010	USA	A/Indiana/03/2010
PB2	CY073005	H1N1	02/03/2010	Nicaragua	A/Managua/07193_09/2010(H1N1)
PB2	CY061194	H1N1	02/03/2010	USA	A/Texas/JMS413/2010(H1N1)
PB2	KT180730	H1N1	02/03/2010	Singapore	A/Singapore/DMS75/2010
PB2	KC781453	H1N1	02/03/2010	USA	A/Louisiana/01/2010
PB2	CY071326	H1N1	02/03/2010	Greece	A/Athens/INS421/2010(H1N1)
PB2	CY167331	H1N1	02/04/2010	USA	A/Tennessee/F1042A/2010
PB2	KC782024	H1N1	02/04/2010	USA	A/Vermont/01/2010
PB2	CY066486	H1N1	02/04/2010	USA	A/California/VRDL5/2010(H1N1)
PB2	CY065050	H1N1	02/04/2010	USA	A/New York/4294/2010(H1N1)
PB2	CY061202	H1N1	02/04/2010	USA	A/Texas/JMS414/2010(H1N1)
PB2	LN846491	H1N1	02/04/2010	United Ki	A/England/01220736/2010
PB2	LN850027	H1N1	02/04/2010	United Ki	A/England/29/2010
PB2	KC781254	H1N1	02/05/2010	USA	A/Georgia/06/2010
PB2	KT180732	H1N1	02/05/2010	Singapore	A/Singapore/DMS76/2010
PB2	CY083824	H1N1	02/05/2010	USA	A/Los Angeles/INS423/2010(H1N1)
PB2	CY071294	H1N1	02/05/2010	Greece	A/Athens/INS414/2010(H1N1)
PB2	CY071382	H1N1	02/05/2010	Germany	A/Berlin/INS430/2010(H1N1)
PB2	CY073740	H1N1	02/05/2010	Estonia	A/Tallinn/INS431/2010(H1N1)
PB2	CY071286	H1N1	02/05/2010	Greece	A/Athens/INS413/2010(H1N1)
PB2	KC781262	H1N1	02/06/2010	USA	A/Georgia/04/2010
PB2	CY061210	H1N1	02/06/2010	USA	A/Texas/JMS415/2010(H1N1)
PB2	KC780242	H1N1	02/06/2010	USA	A/Georgia/01/2010
PB2	KP637283	H1N1	02/07/2010	Thailand	A/Thailand/IY07115/2010
PB2	KC781391	H1N1	02/07/2010	USA	A/Alaska/01/2010
PB2	CY066478	H1N1	02/08/2010	USA	A/California/VRDL4/2010(H1N1)
PB2	KC781188	H1N1	02/08/2010	USA	A/Wyoming/01/2010
PB2	KT180733	H1N1	02/08/2010	Singapore	A/Singapore/DMS77/2010
PB2	CY090868	H1N1	02/09/2010	Netherlar	A/Netherlands/2631c/2010(H1N1)
PB2	KP637835	H1N1	02/09/2010	Thailand	A/Thailand/IY07144/2010
PB2	KC261308	H1N1	02/09/2010	Mexico	A/Mexico City/INERPHAC3/2010(H1N
PB2	CY100535	H1N1	02/09/2010	Mexico	A/Mexico City/INER11/2010
PB2	KC780454	H1N1	02/09/2010	USA	A/New York/03/2010
PB2	CY167339	H1N1	02/09/2010	USA	A/Tennessee/F1052A/2010
PB2	CY066510	H1N1	02/09/2010	USA	A/California/VRDL9/2010(H1N1)
PB2	KC780501	H1N1	02/10/2010	USA	A/Utah/02/2010
PB2	KT180734	H1N1	02/10/2010	Singapore	A/Singapore/DMS78/2010
PB2	CY063186	H1N1	02/11/2010	USA	A/California/VRDL10/2010(H1N1)
PB2	KJ856111	H1N1	02/12/2010	Sri Lanka	A/Sri Lanka/20887/2010
PB2	CY066502	H1N1	02/13/2010	USA	A/California/VRDL8/2010(H1N1)
PB2	CY083037	H1N1	02/16/2010	Senegal	A/Dakar/WRAIR0020T/2010
PB2	CY097786	H1N1	02/16/2010	Senegal	A/Dakar/WRAIR0020T/2010
PB2	KC780521	H1N1	02/17/2010	USA	A/Wisconsin/02/2010
PB2	CY075082	H1N1	02/17/2010	Greece	A/Athens/INS416/2010(H1N1)
PB2	CY167347	H1N1	02/18/2010	USA	A/Tennessee/F1057c56/2010
PB2	CY167355	H1N1	02/18/2010	USA	A/Tennessee/F1060/2010
PB2	KP637859	H1N1	02/18/2010	Thailand	A/Thailand/SS08248/2010
PB2	KT180735	H1N1	02/20/2010	Singapore	A/Singapore/DMS80/2010
PB2	LN867346	H1N1	02/20/2010	United Ki	A/England/01220741/2010
PB2	CY065066	H1N1	02/21/2010	USA	A/New York/6530/2010(H1N1)
PB2	KC782141	H1N1	02/23/2010	USA	A/Tennessee/01/2010
PB2	CY096601	H1N1	02/23/2010	USA	A/District of Columbia/INS527/2(
PB2	CY071334	H1N1	02/23/2010	Thailand	A/Bangkok/INS424/2010(H1N1)

PB2	KT180737	H1N1	02/24/2010	Singapore	A/Singapore/DMS81/2010
PB2	CY129701	H1N1	02/24/2010	Greece	A/Athens/INS555/2010
PB2	KC780362	H1N1	02/24/2010	USA	A/North Carolina/05/2010
PB2	CY071342	H1N1	02/25/2010	Thailand	A/Bangkok/INS425/2010(H1N1)
PB2	CY071350	H1N1	02/25/2010	Thailand	A/Bangkok/INS426/2010(H1N1)
PB2	CY063837	H1N1	02/26/2010	Singapore	A/Singapore/GP999/2010(H1N1)
PB2	CY167371	H1N1	02/26/2010	USA	A/Tennessee/F1066c65/2010
PB2	CY167363	H1N1	02/26/2010	USA	A/Tennessee/F1063A/2010
PB2	CY167379	H1N1	02/26/2010	USA	A/Tennessee/F1067/2010
PB2	KC781165	H1N1	03/01/2010	USA	A/Alabama/03/2010
PB2	KT180739	H1N1	03/01/2010	Singapore	A/Singapore/DMS83/2010
PB2	CY065114	H1N1	03/01/2010	USA	A/New York/7480/2010(H1N1)
PB2	HM124380	H1N1	03/02/2010	Russia	A/Orenburg/IIV-13/2010(H1N1)
PB2	CY167395	H1N1	03/02/2010	USA	A/Tennessee/F1071/2010
PB2	HM569737	H1N1	03/02/2010	Russia	A/Orenburg/IIV-13/2010(H1N1)
PB2	CY167387	H1N1	03/02/2010	USA	A/Tennessee/F1068/2010
PB2	CY167411	H1N1	03/02/2010	USA	A/Tennessee/F1072c71/2010
PB2	KC781354	H1N1	03/03/2010	USA	A/Iowa/04/2010
PB2	CY071358	H1N1	03/04/2010	Thailand	A/Bangkok/INS427/2010(H1N1)
PB2	CY071366	H1N1	03/04/2010	Thailand	A/Bangkok/INS428/2010(H1N1)
PB2	KC780740	H1N1	03/04/2010	USA	A/California/05/2010
PB2	CY167403	H1N1	03/05/2010	USA	A/Tennessee/F1071B/2010
PB2	CY071374	H1N1	03/05/2010	USA	A/Newark/INS429/2010(H1N1)
PB2	KC781405	H1N1	03/08/2010	USA	A/Utah/03/2010
PB2	JX309231	H1N1	03/10/2010	Singapore	A/Singapore/KK100/2010(H1N1)
PB2	KC781395	H1N1	03/11/2010	USA	A/Nebraska/02/2010
PB2	KT180742	H1N1	03/12/2010	Singapore	A/Singapore/DMS86/2010
PB2	CY167419	H1N1	03/15/2010	USA	A/Tennessee/F1074/2010
PB2	KT180745	H1N1	03/16/2010	Singapore	A/Singapore/DMS88/2010
PB2	CY167443	H1N1	03/16/2010	USA	A/Tennessee/F1078/2010
PB2	CY167427	H1N1	03/16/2010	USA	A/Tennessee/F1076/2010
PB2	LN867817	H1N1	03/18/2010	United Ki	A/England/WTSI2076/2010
PB2	LN850342	H1N1	03/18/2010	United Ki	A/England/01180018/2010
PB2	LN850418	H1N1	03/18/2010	United Ki	A/England/01180007/2010
PB2	LN850099	H1N1	03/18/2010	United Ki	A/England/WTSI1807/2010
PB2	LN845771	H1N1	03/18/2010	United Ki	A/England/12/2010
PB2	LN867394	H1N1	03/18/2010	United Ki	A/England/WTSI1783/2010
PB2	LN867420	H1N1	03/18/2010	United Ki	A/England/01180015/2010
PB2	LN867483	H1N1	03/18/2010	United Ki	A/England/01180017/2010
PB2	LN846531	H1N1	03/18/2010	United Ki	A/England/00380719/2010
PB2	LN846587	H1N1	03/18/2010	United Ki	A/England/00380718/2010
PB2	LN850122	H1N1	03/18/2010	United Ki	A/England/11/2010
PB2	LN850075	H1N1	03/18/2010	United Ki	A/England/00380710/2010
PB2	LN850334	H1N1	03/18/2010	United Ki	A/England/00380709/2010
PB2	LN846507	H1N1	03/18/2010	United Ki	A/England/01180016/2010
PB2	LN867673	H1N1	03/18/2010	United Ki	A/England/WTSI2067/2010
PB2	LN867777	H1N1	03/18/2010	United Ki	A/England/01180011/2010
PB2	LN846784	H1N1	03/18/2010	United Ki	A/England/10/2010
PB2	LN867370	H1N1	03/18/2010	United Ki	A/England/WTSI1774/2010
PB2	CY089378	H1N1	03/19/2010	Nicaragua	A/Managua/5708.01/2010(H1N1)
PB2	CY167435	H1N1	03/19/2010	USA	A/Tennessee/F1076A/2010
PB2	CY167451	H1N1	03/19/2010	USA	A/Tennessee/F1083c80/2010
PB2	CY167787	H1N1	03/19/2010	USA	A/Tennessee/F1080/2010
PB2	KT180746	H1N1	03/20/2010	Singapore	A/Singapore/DMS89/2010

PB2	CY080320	H1N1	03/20/2010	Thailand	A/Thailand/CU-H1786/2010(H1N1)
PB2	CY096609	H1N1	03/21/2010	USA	A/Cambridge/INS528/2010(H1N1)
PB2	JF265677	H1N1	03/22/2010	India	A/India/Blore/2010
PB2	KM013754	H1N1	03/23/2010	Thailand	A/Bangkok/SIMI508/2010
PB2	CY090844	H1N1	03/23/2010	Nicaragua	A/Managua/0305_10/2010(H1N1)
PB2	KT180747	H1N1	03/23/2010	Singapore	A/Singapore/DMS90/2010
PB2	CY167459	H1N1	03/24/2010	USA	A/Tennessee/F1089/2010
PB2	KT180749	H1N1	03/25/2010	Singapore	A/Singapore/DMS91/2010
PB2	KC261309	H1N1	03/25/2010	Mexico	A/Mexico City/INERPHAC4/2010(H1N
PB2	CY089386	H1N1	03/25/2010	Nicaragua	A/Managua/3246.01/2010(H1N1)
PB2	LN867386	H1N1	03/26/2010	United Ki	A/England/01580005/2010
PB2	KT180751	H1N1	03/29/2010	Singapore	A/Singapore/DMS92/2010
PB2	CY080328	H1N1	03/29/2010	Thailand	A/Thailand/H1818/2010
PB2	CY080336	H1N1	03/31/2010	Thailand	A/Thailand/CU-H1821/2010(H1N1)

1)

1)
1)

1)
1)

1)
1)
1)
1)

1)

1)
1)
1)

1)

1)

)

1)
1)
)
1)
1)
1)
1)
1)
1)
1)

)

1)
1)

1)
1)
1)
1)
1)
1)

1)

1)
1)

1)
1)
1)
1)
1)

1)
1)
1)
1)
1)
1)
1)
1)
1)

1)

1)

1)

1)
1)
1)

1)

1)

1)

1)

1)
1)

1)

1)

1)

1)

1)
1)

1)

1)
1)
1)

1)
1)

1)

1)

1)

1)
1)

1)
1)

1)

1)

1)

1)

1)
1)

)
1)

)

1)

1)

1)

)
1)

1)

1)

1)

1)

1)
1)

1)
1)
1)

1)
1)

1)
)
1)

1)
1)

1)

)

1)

1)

1)

1)

1)
1)

)

1)
)
)

1)
1)

1)
1)
1)
)

1)

1)
1)

1)

)

)
)
1)

1)

)
)
)
)

1)

)
)
)
1)

)

)
)

)
)

)
)

)

1)

1)

1)
1)

1)

1)

1)

1)

1)
1)
)
)

)
1)

)

1)
1)

)

009(H1N1)

)

)
1)

)09(H1N1)

1)

1)

1)

1)

)

)

1)

1)

)

)

1)

9

1N1)

9

N1)

1N1)

1N1)

1N1)

1N1)

9

1N1)

N1)

9

1N1)

1N1)

9

009

ENRS-1649/2009

3/2009

2009

)09

9

1N1)

1)

)
)

1)
1)

1)
1)

1)

1)

1)

1)

1)

1)
1)

1)

1)
1)
1)

1)

1)

1)

1)

1)

1)

1)
1)

)
1)

1)

1)

1)

1)

1)

9

1)
1)

1)

1)

9

1)

)

1)

1)

9(H1N1)

9

1)
1)
1)

1)

)
1)

1)

N1)

1)
)

)
)

)
)

)

)
)
)

)
)
09 (H1N1)
)

)

1)

)

1)

1)
1)
1)
)

1)

1)

)

09(H1N1)

Ә(H1N1)

Ә
Ә(H1N1)

)

09(H1N1)

)

)

09(H1N1)

1)

1)

)9 (H1N1)
)9 (H1N1)
)9 (H1N1)
)9 (H1N1)

)
)
)
)9 (H1N1)

)

)

)

)9 (H1N1)
)9 (H1N1)

)9 (H1N1)
9

)9 (H1N1)

1)
1)
1)

1)

)09(H1N1)

1)
1)

)9(H1N1)

)9(H1N1)

)9(H1N1)

)09(H1N1)

1)
1)

1)
)09

1)
1)
1)
1)

)09(H1N1)

)9(H1N1)
N1)

1)

1)

1)
1)

1)

1)

009 (H1N1)

009 (H1N1)

1)

)09(H1N1)

1)

1)

1)
1)
1)
1)
1)

1)

1)

1)
1)

1)

1)

1)

1)
1)

1)
1)
1)
)09(H1N1)
)09

1)

1)

1)

1)

1)

1)

1)

1)

1)

1)

1)

1)

1)

1)

1)

1)

1)

1)

1)

1)

1)

1)

1)
1)
1)

1)
1)
1)

1)

)10 (H1N1)

1N1)

N1)

010(H1N1)

N1)

Name	Sequence Acce	Subtype	Collection Date	Country	Strain Name
PB1	FJ966080	H1N1	04/01/2009	USA	A/California/04/2009(H1N1)
PB1	GQ377049	H1N1	04/01/2009	USA	A/California/04/2009(H1N1)
PB1	GQ303345	H1N1	04/03/2009	Mexico	A/Mexico/4108/2009(H1N1)
PB1	GQ339879	H1N1	04/03/2009	Mexico	A/Mexico/4108/2009(H1N1)
PB1	GQ149652	H1N1	04/03/2009	Mexico	A/Mexico/4108/2009(H1N1)
PB1	GQ463207	H1N1	04/03/2009	Mexico	A/Mexico/4108/2009(H1N1)
PB1	GQ162199	H1N1	04/07/2009	Mexico	A/Mexico/4115/2009(H1N1)
PB1	CY266197	H1N1	04/09/2009	USA	A/California/07/2009
PB1	GQ377054	H1N1	04/09/2009	USA	A/California/08/2009(H1N1)
PB1	KC781787	H1N1	04/09/2009	USA	A/California/07/2009
PB1	FJ969531	H1N1	04/09/2009	USA	A/California/07/2009(H1N1)
PB1	GQ323558	H1N1	04/09/2009	USA	A/California/07/2009(H1N1)
PB1	KC780064	H1N1	04/09/2009	USA	A/California/08/2009
PB1	KU933483	H1N1	04/09/2009	USA	A/California/07/2009
PB1	CY181407	H1N1	04/09/2009	USA	A/California/07-JR003/2009
PB1	CY181431	H1N1	04/09/2009	USA	A/California/07-JR010/2009
PB1	CY181423	H1N1	04/09/2009	USA	A/California/07-JR009/2009
PB1	CY181415	H1N1	04/09/2009	USA	A/California/07-JR008/2009
PB1	CY181399	H1N1	04/09/2009	USA	A/California/07-JR002/2009
PB1	FJ984367	H1N1	04/09/2009	USA	A/California/08/2009(H1N1)
PB1	FJ966978	H1N1	04/09/2009	USA	A/California/07/2009(H1N1)
PB1	CY044233	H1N1	04/14/2009	USA	A/San Antonio/PR921/2009(H1N1)
PB1	GQ149675	H1N1	04/14/2009	Mexico	A/Mexico/4482/2009(H1N1)
PB1	GQ162186	H1N1	04/14/2009	Mexico	A/Mexico/4486/2009(H1N1)
PB1	GQ162176	H1N1	04/14/2009	Mexico	A/Mexico/4486/2009(H1N1)
PB1	CY147777	H1N1	04/14/2009	Mexico	A/Mexico/24033/2009
PB1	GQ379819	H1N1	04/14/2009	Mexico	A/Mexico/4482/2009(H1N1)
PB1	FJ998226	H1N1	04/14/2009	Mexico	A/Mexico/InDRE4487/2009(H1N1)
PB1	FJ969526	H1N1	04/14/2009	USA	A/Texas/04/2009(H1N1)
PB1	FJ981616	H1N1	04/14/2009	USA	A/Texas/04/2009(H1N1)
PB1	GQ303337	H1N1	04/15/2009	Mexico	A/Mexico/4269/2009(H1N1)
PB1	FJ969522	H1N1	04/15/2009	USA	A/Texas/05/2009(H1N1)
PB1	GQ122094	H1N1	04/15/2009	USA	A/Texas/15/2009(H1N1)
PB1	GQ168860	H1N1	04/15/2009	USA	A/Texas/05/2009(H1N1)
PB1	GQ402314	H1N1	04/16/2009	Canada	A/Canada-QC/RV1595/2009(H1N1)
PB1	FJ966965	H1N1	04/16/2009	USA	A/California/06/2009(H1N1)
PB1	KC781717	H1N1	04/16/2009	USA	A/California/06/2009
PB1	CY147769	H1N1	04/17/2009	Mexico	A/Mexico/24032/2009
PB1	CY147825	H1N1	04/17/2009	Mexico	A/Mexico/24043/2009
PB1	GQ339878	H1N1	04/19/2009	Mexico	A/Mexico/4603/2009(H1N1)
PB1	GQ162167	H1N1	04/19/2009	Mexico	A/Mexico/4603/2009(H1N1)
PB1	GQ149683	H1N1	04/19/2009	Mexico	A/Mexico/4603/2009(H1N1)
PB1	GQ149633	H1N1	04/19/2009	Mexico	A/Mexico/4604/2009(H1N1)
PB1	CY147833	H1N1	04/20/2009	Mexico	A/Mexico/24044/2009
PB1	CY147841	H1N1	04/20/2009	Mexico	A/Mexico/24045/2009
PB1	GQ377066	H1N1	04/22/2009	USA	A/Arizona/01/2009(H1N1)
PB1	GQ117093	H1N1	04/22/2009	USA	A/Indiana/09/2009(H1N1)
PB1	CY147617	H1N1	04/23/2009	Mexico	A/Mexico/24007/2009
PB1	CY147705	H1N1	04/23/2009	Mexico	A/Mexico/24021/2009
PB1	GQ117020	H1N1	04/24/2009	USA	A/New York/22/2009(H1N1)
PB1	GQ160544	H1N1	04/24/2009	USA	A/Ohio/07/2009(H1N1)
PB1	GQ117046	H1N1	04/24/2009	USA	A/Texas/08/2009(H1N1)
PB1	FJ998227	H1N1	04/24/2009	Canada	A/Canada-ON/RV1527/2009(H1N1)

PB1	GQ168864	H1N1	04/24/2009	USA	A/New York/23/2009(H1N1)
PB1	GQ338347	H1N1	04/24/2009	USA	A/New York/31/2009(H1N1)
PB1	FJ998225	H1N1	04/24/2009	Canada	A/Canada-NS/RV1535/2009(H1N1)
PB1	GQ168855	H1N1	04/24/2009	USA	A/Kansas/02/2009(H1N1)
PB1	CY065148	H1N1	04/24/2009	United Ki	A/Scotland/91800799/2009
PB1	GQ168638	H1N1	04/24/2009	USA	A/Iowa/04/2009(H1N1)
PB1	GQ168857	H1N1	04/24/2009	USA	A/Kansas/03/2009(H1N1)
PB1	CY041555	H1N1	04/24/2009	USA	A/New York/3210/2009(H1N1)
PB1	GQ200247	H1N1	04/24/2009	USA	A/New York/21/2009(H1N1)
PB1	GQ377081	H1N1	04/24/2009	USA	A/Ohio/07/2009(H1N1)
PB1	GQ168887	H1N1	04/24/2009	USA	A/Ohio/07/2009(H1N1)
PB1	GQ373260	H1N1	04/24/2009	Canada	A/Toronto/3141/2009(H1N1)
PB1	CY086983	H1N1	04/25/2009	USA	A/New York/3217/2009(H1N1)
PB1	GQ117120	H1N1	04/25/2009	USA	A/New York/09/2009(H1N1)
PB1	GQ117026	H1N1	04/25/2009	USA	A/Texas/09/2009(H1N1)
PB1	GQ168850	H1N1	04/25/2009	USA	A/New York/06/2009(H1N1)
PB1	GQ168852	H1N1	04/25/2009	USA	A/New York/11/2009(H1N1)
PB1	FJ984373	H1N1	04/25/2009	USA	A/New York/10/2009(H1N1)
PB1	GQ168867	H1N1	04/25/2009	USA	A/New York/20/2009(H1N1)
PB1	CY041619	H1N1	04/25/2009	USA	A/New York/3002/2009(H1N1)
PB1	CY041547	H1N1	04/25/2009	USA	A/New York/3209/2009(H1N1)
PB1	CY040886	H1N1	04/25/2009	Mexico	A/Mexico/47N/2009(H1N1)
PB1	GQ457491	H1N1	04/25/2009	USA	A/New York/20/2009(H1N1)
PB1	CY041128	H1N1	04/25/2009	USA	A/New York/3214/2009(H1N1)
PB1	FJ984353	H1N1	04/25/2009	USA	A/New York/18/2009(H1N1)
PB1	GQ168881	H1N1	04/25/2009	USA	A/New York/13/2009(H1N1)
PB1	FJ984392	H1N1	04/25/2009	USA	A/New York/19/2009(H1N1)
PB1	CY097874	H1N1	04/25/2009	Mexico	A/Mexico/47N/2009
PB1	GQ117034	H1N1	04/25/2009	USA	A/California/14/2009(H1N1)
PB1	GQ168877	H1N1	04/25/2009	USA	A/New York/15/2009(H1N1)
PB1	CY044161	H1N1	04/25/2009	Mexico	A/Mexico/48N/2009(H1N1)
PB1	CY147681	H1N1	04/25/2009	Mexico	A/Mexico/24015/2009
PB1	CY041136	H1N1	04/25/2009	USA	A/New York/3215/2009(H1N1)
PB1	CY065140	H1N1	04/25/2009	United Ki	A/Scotland/91800797/2009
PB1	GQ465749	H1N1	04/26/2009	Canada	A/Canada-PQ/RV1586/2009(H1N1)
PB1	GQ168847	H1N1	04/26/2009	USA	A/Massachusetts/06/2009(H1N1)
PB1	CY041740	H1N1	04/26/2009	USA	A/New York/3206/2009(H1N1)
PB1	CY039899	H1N1	04/26/2009	USA	A/New York/1669/2009(H1N1)
PB1	CY041064	H1N1	04/26/2009	USA	A/New York/3168/2009(H1N1)
PB1	GQ117075	H1N1	04/26/2009	USA	A/Arizona/02/2009(H1N1)
PB1	CY041176	H1N1	04/26/2009	USA	A/New York/3186/2009(H1N1)
PB1	CY041104	H1N1	04/26/2009	USA	A/New York/3176/2009(H1N1)
PB1	CY041056	H1N1	04/26/2009	USA	A/New York/3166/2009(H1N1)
PB1	GQ168880	H1N1	04/26/2009	USA	A/Michigan/02/2009(H1N1)
PB1	GQ160570	H1N1	04/26/2009	USA	A/Texas/22/2009(H1N1)
PB1	GQ168854	H1N1	04/26/2009	USA	A/South Carolina/09/2009(H1N1)
PB1	CY041520	H1N1	04/27/2009	USA	A/New York/3203/2009(H1N1)
PB1	GQ465750	H1N1	04/27/2009	Canada	A/Canada-ON/RV1589/2009(H1N1)
PB1	GQ894930	H1N1	04/27/2009	USA	A/Alabama/02/2009(H1N1)
PB1	CY040844	H1N1	04/27/2009	USA	A/New York/3262/2009(H1N1)
PB1	GQ117069	H1N1	04/27/2009	USA	A/Minnesota/02/2009(H1N1)
PB1	CY041528	H1N1	04/27/2009	USA	A/New York/3204/2009(H1N1)
PB1	CY069771	H1N1	04/27/2009	United Ki	A/England/209/2009
PB1	CY147849	H1N1	04/27/2009	Mexico	A/Mexico/24047/2009

PB1	CY065188	H1N1	04/27/2009	United Ki	A/England/202/2009
PB1	CY065212	H1N1	04/27/2009	United Ki	A/England/205/2009
PB1	CY147889	H1N1	04/27/2009	Mexico	A/Mexico/24052/2009
PB1	CY147865	H1N1	04/27/2009	Mexico	A/Mexico/24049/2009
PB1	CY147905	H1N1	04/27/2009	Mexico	A/Mexico/24054/2009
PB1	CY147897	H1N1	04/27/2009	Mexico	A/Mexico/24053/2009
PB1	CY147857	H1N1	04/27/2009	Mexico	A/Mexico/24048/2009
PB1	CY040828	H1N1	04/27/2009	USA	A/New York/3260/2009(H1N1)
PB1	GQ402311	H1N1	04/27/2009	Mexico	A/Mexico/InDRE13547/2009(H1N1)
PB1	CY041088	H1N1	04/27/2009	USA	A/New York/3172/2009(H1N1)
PB1	GQ168884	H1N1	04/27/2009	USA	A/Colorado/03/2009(H1N1)
PB1	CY039907	H1N1	04/27/2009	USA	A/New York/1682/2009(H1N1)
PB1	GQ160522	H1N1	04/27/2009	USA	A/Florida/04/2009(H1N1)
PB1	CY147873	H1N1	04/27/2009	Mexico	A/Mexico/24050/2009
PB1	CY147881	H1N1	04/27/2009	Mexico	A/Mexico/24051/2009
PB1	CY147785	H1N1	04/27/2009	Mexico	A/Mexico/24036/2009
PB1	CY147721	H1N1	04/27/2009	Mexico	A/Mexico/24024/2009
PB1	CY147713	H1N1	04/27/2009	Mexico	A/Mexico/24022/2009
PB1	CY065244	H1N1	04/27/2009	United Ki	A/England/XFL00071/2009
PB1	CY065220	H1N1	04/27/2009	United Ki	A/England/207/2009
PB1	CY065196	H1N1	04/27/2009	United Ki	A/England/197/2009
PB1	CY065236	H1N1	04/27/2009	United Ki	A/England/551/2009
PB1	CY065204	H1N1	04/28/2009	United Ki	A/England/204/2009
PB1	CY047324	H1N1	04/28/2009	USA	A/New York/3124/2009(H1N1)
PB1	CY053077	H1N1	04/28/2009	USA	A/Houston/130S/2009(H1N1)
PB1	CY147793	H1N1	04/28/2009	Mexico	A/Mexico/24038/2009
PB1	CY147753	H1N1	04/28/2009	Mexico	A/Mexico/24028/2009
PB1	CY147745	H1N1	04/28/2009	Mexico	A/Mexico/24027/2009
PB1	CY040707	H1N1	04/28/2009	USA	A/New York/3235/2009(H1N1)
PB1	CY041120	H1N1	04/28/2009	USA	A/New York/3193/2009(H1N1)
PB1	CY041192	H1N1	04/28/2009	USA	A/New York/3188/2009(H1N1)
PB1	CY041208	H1N1	04/28/2009	USA	A/New York/3199/2009(H1N1)
PB1	CY041512	H1N1	04/28/2009	USA	A/New York/3196/2009(H1N1)
PB1	CY041496	H1N1	04/28/2009	USA	A/New York/3184/2009(H1N1)
PB1	CY041504	H1N1	04/28/2009	USA	A/New York/3195/2009(H1N1)
PB1	GQ166655	H1N1	04/28/2009	United Ki	A/England/195/2009
PB1	CY065252	H1N1	04/28/2009	United Ki	A/England/211/2009
PB1	GQ465745	H1N1	04/28/2009	Canada	A/Canada-AB/RV1531/2009(H1N1)
PB1	CY065228	H1N1	04/28/2009	United Ki	A/England/200/2009
PB1	CY147921	H1N1	04/28/2009	Mexico	A/Mexico/24056/2009
PB1	CY147929	H1N1	04/28/2009	Mexico	A/Mexico/24057/2009
PB1	CY147913	H1N1	04/28/2009	Mexico	A/Mexico/24055/2009
PB1	CY041836	H1N1	04/28/2009	USA	A/New York/3348/2009(H1N1)
PB1	CY147945	H1N1	04/28/2009	Mexico	A/Mexico/24060/2009
PB1	CY041579	H1N1	04/28/2009	USA	A/New York/3227/2009(H1N1)
PB1	CY041587	H1N1	04/28/2009	USA	A/New York/3230/2009(H1N1)
PB1	CY041563	H1N1	04/28/2009	USA	A/New York/3211/2009(H1N1)
PB1	GQ402312	H1N1	04/28/2009	Mexico	A/Mexico/InDRE13551/2009(H1N1)
PB1	CY047332	H1N1	04/28/2009	USA	A/New York/3167/2009(H1N1)
PB1	CY046337	H1N1	04/28/2009	USA	A/Wisconsin/629-D01505/2009(H1N
PB1	CY147737	H1N1	04/28/2009	Mexico	A/Mexico/24026/2009
PB1	CY147953	H1N1	04/28/2009	Mexico	A/Mexico/24061/2009
PB1	CY147937	H1N1	04/28/2009	Mexico	A/Mexico/24058/2009
PB1	CY041096	H1N1	04/28/2009	USA	A/New York/3173/2009(H1N1)

PB1	GQ232042	H1N1	04/28/2009	USA	A/Rhode Island/02/2009(H1N1)
PB1	CY041152	H1N1	04/28/2009	USA	A/New York/3181/2009(H1N1)
PB1	CY041595	H1N1	04/28/2009	USA	A/New York/3236/2009(H1N1)
PB1	GQ396730	H1N1	04/28/2009	USA	A/Tennessee/05/2009(H1N1)
PB1	GQ465746	H1N1	04/29/2009	Canada	A/Canada-NS/RV1551/2009(H1N1)
PB1	CY041732	H1N1	04/29/2009	USA	A/New York/3247/2009(H1N1)
PB1	CY047316	H1N1	04/29/2009	USA	A/New York/3074/2009(H1N1)
PB1	CY040868	H1N1	04/29/2009	USA	A/New York/3265/2009(H1N1)
PB1	GQ222422	H1N1	04/29/2009	France	A/Paris/2573/2009(H1N1)
PB1	GQ160562	H1N1	04/29/2009	USA	A/Maryland/05/2009(H1N1)
PB1	GQ465747	H1N1	04/29/2009	Canada	A/Canada-NS/RV1554/2009(H1N1)
PB1	CY147801	H1N1	04/29/2009	Mexico	A/Mexico/24039/2009
PB1	CY147761	H1N1	04/29/2009	Mexico	A/Mexico/24030/2009
PB1	GQ402313	H1N1	04/29/2009	Mexico	A/Mexico/InDRE13555/2009(H1N1)
PB1	CY065260	H1N1	04/29/2009	United Ki	A/England/XFL00137/2009
PB1	CY046941	H1N1	04/29/2009	Netherlar	A/Netherlands/602/2009
PB1	GQ402310	H1N1	04/29/2009	Mexico	A/Mexico/InDRE13495/2009(H1N1)
PB1	CY065340	H1N1	04/29/2009	United Ki	A/England/223/2009
PB1	GQ402309	H1N1	04/29/2009	Mexico	A/Mexico/InDRE13494/2009(H1N1)
PB1	CY086975	H1N1	04/29/2009	USA	A/New York/3198/2009(H1N1)
PB1	CY147809	H1N1	04/29/2009	Mexico	A/Mexico/24040/2009
PB1	GQ465753	H1N1	04/30/2009	Canada	A/Canada-SK/RV1788/2009(H1N1)
PB1	CY041627	H1N1	04/30/2009	USA	A/New York/3100/2009(H1N1)
PB1	CY040699	H1N1	04/30/2009	USA	A/New York/3232/2009(H1N1)
PB1	CY046393	H1N1	04/30/2009	USA	A/Wisconsin/629-D00750/2009(H1N
PB1	GQ222421	H1N1	04/30/2009	France	A/Paris/2580/2009(H1N1)
PB1	GQ222419	H1N1	04/30/2009	France	A/Paris/2590/2009(H1N1)
PB1	CY064714	H1N1	04/30/2009	Mexico	A/Mexico City/020/2009
PB1	CY065276	H1N1	04/30/2009	United Ki	A/England/215/2009
PB1	CY065284	H1N1	04/30/2009	United Ki	A/England/216/2009
PB1	GU931815	H1N1	04/30/2009	Hong Kong	A/Hong Kong/415742/2009(H1N1)
PB1	CY064746	H1N1	04/30/2009	Mexico	A/Mexico City/024/2009(H1N1)
PB1	CY065268	H1N1	04/30/2009	United Ki	A/England/214/2009
PB1	CY046417	H1N1	04/30/2009	USA	A/Wisconsin/629-D00447/2009(H1N
PB1	CY046265	H1N1	04/30/2009	USA	A/Wisconsin/629-D01773/2009(H1N
PB1	CY046849	H1N1	04/30/2009	USA	A/Wisconsin/629-D02361/2009(H1N
PB1	CY046521	H1N1	04/30/2009	USA	A/Wisconsin/629-D02141/2009(H1N
PB1	CY047356	H1N1	04/30/2009	USA	A/New York/3365/2009(H1N1)
PB1	CY046481	H1N1	04/30/2009	USA	A/Wisconsin/629-D00015/2009(H1N
PB1	CY064706	H1N1	04/30/2009	Mexico	A/Mexico City/019/2009
PB1	CY046457	H1N1	04/30/2009	USA	A/Wisconsin/629-D01558/2009(H1N
PB1	CY040651	H1N1	04/30/2009	USA	A/New York/3194/2009(H1N1)
PB1	CY046361	H1N1	04/30/2009	USA	A/Wisconsin/629-D01529/2009(H1N
PB1	GQ465748	H1N1	04/30/2009	Canada	A/Canada-NS/RV1565/2009(H1N1)
PB1	CY046425	H1N1	04/30/2009	USA	A/Wisconsin/629-D00767/2009(H1N
PB1	CY040040	H1N1	05/2009	Thailand	A/Nonthaburi/102/2009
PB1	CY046321	H1N1	05/01/2009	USA	A/Wisconsin/629-D01308/2009(H1N
PB1	CY064754	H1N1	05/01/2009	Mexico	A/Mexico City/025/2009(H1N1)
PB1	GQ249336	H1N1	05/01/2009	France	A/Paris/2591/2009(H1N1)
PB1	CY064722	H1N1	05/01/2009	Mexico	A/Mexico City/021/2009
PB1	CY046497	H1N1	05/01/2009	USA	A/Wisconsin/629-D02028/2009(H1N
PB1	CY053101	H1N1	05/01/2009	USA	A/Houston/150S/2009(H1N1)
PB1	CY053085	H1N1	05/01/2009	USA	A/Houston/140S/2009H1N1)
PB1	CY046193	H1N1	05/01/2009	USA	A/Wisconsin/629-D01793/2009(H1N

PB1	CY046433	H1N1	05/01/2009	USA	A/Wisconsin/629-D00677/2009(H1N
PB1	GQ465751	H1N1	05/01/2009	Canada	A/Canada-AB/RV1644/2009(H1N1)
PB1	CY046465	H1N1	05/01/2009	USA	A/Wisconsin/629-D01861/2009(H1N
PB1	CY047348	H1N1	05/01/2009	USA	A/New York/3345/2009(H1N1)
PB1	CY046977	H1N1	05/01/2009	USA	A/New York/3443/2009(H1N1)
PB1	CY053109	H1N1	05/01/2009	USA	A/Houston/15H/2009(H1N1)
PB1	GQ222425	H1N1	05/01/2009	France	A/Paris/2592/2009(H1N1)
PB1	CY041643	H1N1	05/01/2009	USA	A/New York/3307/2009(H1N1)
PB1	CY053093	H1N1	05/01/2009	USA	A/Houston/14H/2009(H1N1)
PB1	CY064738	H1N1	05/01/2009	Mexico	A/Mexico City/023/2009(H1N1)
PB1	CY049865	H1N1	05/02/2009	USA	A/Cherry Point/WR0101/2009(H1N1)
PB1	CY041603	H1N1	05/02/2009	USA	A/New York/3177/2009(H1N1)
PB1	CY041812	H1N1	05/02/2009	USA	A/New York/3408/2009(H1N1)
PB1	GQ323500	H1N1	05/02/2009	USA	A/Michigan/06/2009(H1N1)
PB1	CY065324	H1N1	05/02/2009	United Ki	A/England/220/2009
PB1	CY069779	H1N1	05/02/2009	United Ki	A/England/221/2009
PB1	CY064730	H1N1	05/02/2009	Mexico	A/Mexico City/022/2009
PB1	CY046273	H1N1	05/02/2009	USA	A/Wisconsin/629-D00933/2009(H1N
PB1	CY065316	H1N1	05/02/2009	United Ki	A/England/219/2009
PB1	CY065308	H1N1	05/02/2009	United Ki	A/England/246/2009
PB1	GQ160813	H1N1	05/02/2009	South Ko	A/Korea/01/2009
PB1	HM567566	H1N1	05/02/2009	United Ki	A/England/221/2009
PB1	CY065332	H1N1	05/02/2009	United Ki	A/England/201/2009
PB1	CY065300	H1N1	05/02/2009	United Ki	A/England/218/2009
PB1	CY046345	H1N1	05/02/2009	USA	A/Wisconsin/629-D02455/2009(H1N
PB1	CY046441	H1N1	05/02/2009	USA	A/Wisconsin/629-D02262/2009(H1N
PB1	CY046241	H1N1	05/02/2009	USA	A/Wisconsin/629-D01735/2009(H1N
PB1	CY046401	H1N1	05/02/2009	USA	A/Wisconsin/629-D01189/2009(H1N
PB1	CY046201	H1N1	05/02/2009	USA	A/Wisconsin/629-D02312/2009(H1N
PB1	CY046209	H1N1	05/02/2009	USA	A/Wisconsin/629-D02408/2009(H1N
PB1	CY046385	H1N1	05/02/2009	USA	A/Wisconsin/629-D01980/2009(H1N
PB1	CY046857	H1N1	05/02/2009	USA	A/Wisconsin/629-D00244/2009(H1N
PB1	CY041080	H1N1	05/02/2009	USA	A/New York/3170/2009(H1N1)
PB1	CY049857	H1N1	05/02/2009	USA	A/Cherry Point/WR0100/2009(H1N1)
PB1	CY049873	H1N1	05/02/2009	USA	A/Cherry Point/WR0102/2009(H1N1)
PB1	CY064762	H1N1	05/02/2009	Mexico	A/Mexico City/026/2009(H1N1)
PB1	CY069787	H1N1	05/03/2009	United Ki	A/England/222/2009
PB1	CY046281	H1N1	05/03/2009	USA	A/Wisconsin/629-D01092/2009(H1N
PB1	CY065348	H1N1	05/03/2009	United Ki	A/England/247/2009
PB1	CY065292	H1N1	05/03/2009	United Ki	A/Scotland/4/2009
PB1	CY046377	H1N1	05/03/2009	USA	A/Wisconsin/629-D01705/2009(H1N
PB1	CY046353	H1N1	05/03/2009	USA	A/Wisconsin/629-D01017/2009(H1N
PB1	CY046889	H1N1	05/03/2009	USA	A/Wisconsin/629-D00498/2009(H1N
PB1	CY046289	H1N1	05/03/2009	USA	A/Wisconsin/629-D02473/2009(H1N
PB1	GQ251033	H1N1	05/03/2009	Italy	A/Italy/05/2009(H1N1)
PB1	CY046217	H1N1	05/03/2009	USA	A/Wisconsin/629-D00869/2009(H1N
PB1	CY046233	H1N1	05/03/2009	USA	A/Wisconsin/629-D01296/2009(H1N
PB1	CY046249	H1N1	05/03/2009	USA	A/Wisconsin/629-D01606/2009(H1N
PB1	CY041651	H1N1	05/04/2009	USA	A/New York/3313/2009(H1N1)
PB1	CY046881	H1N1	05/04/2009	USA	A/Wisconsin/629-D00396/2009(H1N
PB1	CY046473	H1N1	05/04/2009	USA	A/Wisconsin/629-D01962/2009(H1N
PB1	CY069803	H1N1	05/04/2009	United Ki	A/England/XFL00230/2009
PB1	CY050148	H1N1	05/04/2009	USA	A/Wisconsin/629-D01058/2009(H1N
PB1	CY065356	H1N1	05/04/2009	United Ki	A/England/XFL00200/2009

PB1	CY069819	H1N1	05/04/2009	United Ki	A/England/250/2009
PB1	CY053148	H1N1	05/04/2009	USA	A/Houston/220S/2009(H1N1)
PB1	CY046329	H1N1	05/04/2009	USA	A/Wisconsin/629-D01810/2009(H1N
PB1	CY046489	H1N1	05/04/2009	USA	A/Wisconsin/629-D00349/2009(H1N
PB1	CY046505	H1N1	05/04/2009	USA	A/Wisconsin/629-D00378/2009(H1N
PB1	CY046529	H1N1	05/04/2009	USA	A/Wisconsin/629-D00183/2009(H1N
PB1	CY046537	H1N1	05/04/2009	USA	A/Wisconsin/629-D00714/2009(H1N
PB1	CY046865	H1N1	05/04/2009	USA	A/Wisconsin/629-D01415/2009(H1N
PB1	CY046257	H1N1	05/04/2009	USA	A/Wisconsin/629-D02432/2009(H1N
PB1	CY046449	H1N1	05/04/2009	USA	A/Wisconsin/629-D01226/2009(H1N
PB1	CY046369	H1N1	05/04/2009	USA	A/Wisconsin/629-D00790/2009(H1N
PB1	CY046409	H1N1	05/04/2009	USA	A/Wisconsin/629-D00807/2009(H1N
PB1	CY046225	H1N1	05/04/2009	USA	A/Wisconsin/629-D01251/2009(H1N
PB1	CY045480	H1N1	05/04/2009	Germany	A/Sachsen-Anhalt/101/2009(H1N1)
PB1	CY046897	H1N1	05/04/2009	USA	A/Wisconsin/629-D01880/2009(H1N
PB1	CY043209	H1N1	05/04/2009	USA	A/New York/3338/2009(H1N1)
PB1	CY046921	H1N1	05/04/2009	USA	A/Wisconsin/629-D01764/2009(H1N
PB1	CY043177	H1N1	05/04/2009	USA	A/New York/3460/2009(H1N1)
PB1	CY046873	H1N1	05/04/2009	USA	A/Wisconsin/629-D02272/2009(H1N
PB1	CY041788	H1N1	05/04/2009	USA	A/New York/3337/2009(H1N1)
PB1	CY053156	H1N1	05/04/2009	USA	A/Houston/22H/2009(H1N1)
PB1	CY046513	H1N1	05/04/2009	USA	A/Wisconsin/629-D02425/2009(H1N
PB1	GQ402316	H1N1	05/04/2009	Canada	A/Canada-SK/RV1767/2009(H1N1)
PB1	CY069811	H1N1	05/05/2009	United Ki	A/England/249/2009
PB1	CY065380	H1N1	05/05/2009	United Ki	A/England/XFL00304/2009
PB1	CY065412	H1N1	05/05/2009	United Ki	A/England/254/2009
PB1	CY069827	H1N1	05/05/2009	United Ki	A/England/XFL00303/2009
PB1	HM567574	H1N1	05/05/2009	United Ki	A/England/249/2009
PB1	CY049818	H1N1	05/05/2009	USA	A/Craven/WR0019/2009(H1N1)
PB1	CY069795	H1N1	05/05/2009	United Ki	A/England/XFL00228/2009
PB1	CY065364	H1N1	05/05/2009	United Ki	A/England/XFL00275/2009
PB1	CY041756	H1N1	05/06/2009	USA	A/New York/3323/2009(H1N1)
PB1	GQ259597	H1N1	05/06/2009	Thailand	A/Thailand/104/2009(H1N1)
PB1	GQ402318	H1N1	05/06/2009	Canada	A/Canada-SK/RV1798/2009(H1N1)
PB1	CY046561	H1N1	05/06/2009	USA	A/Wisconsin/629-D00228/2009(H1N
PB1	CY043201	H1N1	05/06/2009	USA	A/New York/3324/2009(H1N1)
PB1	CY047340	H1N1	05/06/2009	USA	A/New York/3364/2009(H1N1)
PB1	CY041828	H1N1	05/06/2009	USA	A/New York/3352/2009(H1N1)
PB1	CY041820	H1N1	05/06/2009	USA	A/New York/3351/2009(H1N1)
PB1	CY065372	H1N1	05/07/2009	United Ki	A/England/XFL00300/2009
PB1	CY065404	H1N1	05/07/2009	United Ki	A/England/253/2009
PB1	CY050204	H1N1	05/07/2009	Mexico	A/Mexico City/001/2009
PB1	CY065396	H1N1	05/07/2009	United Ki	A/England/XFL00311/2009
PB1	GQ465754	H1N1	05/07/2009	Canada	A/Canada-SK/RV1794/2009(H1N1)
PB1	GQ465755	H1N1	05/07/2009	Canada	A/Canada-SK/RV1797/2009(H1N1)
PB1	CY100446	H1N1	05/07/2009	Mexico	A/Mexico City/IBT22/2009
PB1	GQ402315	H1N1	05/07/2009	Canada	A/Canada-QC/RV1759/2009(H1N1)
PB1	CY046609	H1N1	05/07/2009	USA	A/Wisconsin/629-D00487/2009(H1N
PB1	CY069835	H1N1	05/08/2009	United Ki	A/England/XFL00364/2009
PB1	GQ402317	H1N1	05/08/2009	Canada	A/Canada-SK/RV1793/2009(H1N1)
PB1	GQ465752	H1N1	05/08/2009	Canada	A/Canada-PQ/RV1758/2009(H1N1)
PB1	CY041772	H1N1	05/08/2009	USA	A/New York/3354/2009(H1N1)
PB1	CY043129	H1N1	05/08/2009	USA	A/New York/3389/2009(H1N1)
PB1	CY050228	H1N1	05/08/2009	Mexico	A/Mexico City/004/2009(H1N1)

PB1	CY050220	H1N1	05/08/2009	Mexico	A/Mexico City/003/2009(H1N1)
PB1	GQ169306	H1N1	05/08/2009	Japan	A/Narita/1/2009
PB1	CY050212	H1N1	05/08/2009	Mexico	A/Mexico City/002/2009(H1N1)
PB1	CY046577	H1N1	05/08/2009	USA	A/Wisconsin/629-D00905/2009(H1N
PB1	CY046545	H1N1	05/08/2009	USA	A/Wisconsin/629-D01313/2009(H1N
PB1	CY046593	H1N1	05/08/2009	USA	A/Wisconsin/629-D02452/2009(H1N
PB1	CY046585	H1N1	05/08/2009	USA	A/Wisconsin/629-D01909/2009(H1N
PB1	CY065388	H1N1	05/08/2009	United Ki	A/England/252/2009
PB1	CY065428	H1N1	05/08/2009	United Ki	A/England/256/2009
PB1	CY050885	H1N1	05/08/2009	Mexico	A/Mexico City/005/2009(H1N1)
PB1	CY050252	H1N1	05/09/2009	Mexico	A/Mexico City/011/2009
PB1	CY050180	H1N1	05/09/2009	Mexico	A/Mexico City/015/2009
PB1	CY050853	H1N1	05/09/2009	Mexico	A/Mexico City/007/2009(H1N1)
PB1	CY050877	H1N1	05/09/2009	Mexico	A/Mexico City/017/2009
PB1	CY046617	H1N1	05/09/2009	USA	A/Wisconsin/629-D01055/2009(H1N
PB1	CY050260	H1N1	05/09/2009	Mexico	A/Mexico City/012/2009
PB1	CY050244	H1N1	05/09/2009	Mexico	A/Mexico City/010/2009
PB1	CY062641	H1N1	05/09/2009	Mexico	A/Mexico City/014/2009
PB1	CY045944	H1N1	05/09/2009	Canada	A/Toronto/C2781/2009(H1N1)
PB1	CY046569	H1N1	05/09/2009	USA	A/Wisconsin/629-D01919/2009(H1N
PB1	CY069843	H1N1	05/09/2009	United Ki	A/England/XFL00376/2009
PB1	CY065436	H1N1	05/09/2009	United Ki	A/England/257/2009
PB1	CY050869	H1N1	05/09/2009	Mexico	A/Mexico City/013/2009
PB1	CY050236	H1N1	05/09/2009	Mexico	A/Mexico City/006/2009(H1N1)
PB1	JQ173154	H1N1	05/10/2009	Finland	A/Helsinki/P15/2009
PB1	CY046553	H1N1	05/10/2009	USA	A/Wisconsin/629-D00179/2009(H1N
PB1	CY050450	H1N1	05/10/2009	USA	A/Wisconsin/629-D01482/2009(H1N
PB1	CY046601	H1N1	05/10/2009	USA	A/Wisconsin/629-D01482/2009(H1N
PB1	CY050172	H1N1	05/10/2009	Mexico	A/Mexico City/008/2009(H1N1)
PB1	GQ200291	H1N1	05/10/2009	China	A/Shandong/1/2009(H1N1)
PB1	CY050893	H1N1	05/10/2009	Mexico	A/Mexico City/018/2009
PB1	CY065420	H1N1	05/10/2009	United Ki	A/England/XFL00354/2009
PB1	GQ166227	H1N1	05/10/2009	China	A/Sichuan/1/2009(H1N1)
PB1	CY043169	H1N1	05/11/2009	USA	A/New York/3454/2009(H1N1)
PB1	CY043225	H1N1	05/11/2009	USA	A/New York/3455/2009(H1N1)
PB1	CY065444	H1N1	05/11/2009	United Ki	A/England/259/2009
PB1	CY069851	H1N1	05/11/2009	United Ki	A/England/260/2009
PB1	GQ249341	H1N1	05/11/2009	France	A/Paris/2629/2009(H1N1)
PB1	CY065452	H1N1	05/11/2009	United Ki	A/England/XFL00418/2009
PB1	CY041764	H1N1	05/11/2009	USA	A/New York/3413/2009(H1N1)
PB1	CY065468	H1N1	05/12/2009	United Ki	A/England/XFL00473/2009
PB1	CY043161	H1N1	05/12/2009	USA	A/New York/3418/2009(H1N1)
PB1	CY069859	H1N1	05/12/2009	United Ki	A/England/XFL00421/2009
PB1	CY050334	H1N1	05/12/2009	USA	A/Wisconsin/629-D01851/2009(H1N
PB1	CY043305	H1N1	05/12/2009	USA	A/New York/3498/2009(H1N1)
PB1	CY065460	H1N1	05/12/2009	United Ki	A/England/XFL00419/2009
PB1	CY050861	H1N1	05/12/2009	Mexico	A/Mexico City/009/2009(H1N1)
PB1	CY065484	H1N1	05/12/2009	United Ki	A/England/264/2009
PB1	CY043185	H1N1	05/12/2009	USA	A/New York/3488/2009(H1N1)
PB1	CY065492	H1N1	05/12/2009	United Ki	A/England/265/2009
PB1	CY065476	H1N1	05/12/2009	United Ki	A/England/263/2009
PB1	CY053069	H1N1	05/13/2009	USA	A/Houston/10H/2009(H1N1)
PB1	CY065500	H1N1	05/13/2009	United Ki	A/England/XFL00481/2009
PB1	CY053061	H1N1	05/13/2009	USA	A/Houston/10OS/2009(H1N1)

PB1	CY052965	H1N1	05/13/2009	USA	A/Houston/1H/2009(H1N1)
PB1	KC781317	H1N1	05/13/2009	USA	A/Mississippi/01/2009
PB1	CY050378	H1N1	05/13/2009	USA	A/Wisconsin/629-D01154/2009(H1N
PB1	CY043217	H1N1	05/13/2009	USA	A/New York/3420/2009(H1N1)
PB1	CY046681	H1N1	05/13/2009	USA	A/Wisconsin/629-D01305/2009(H1N
PB1	CY065508	H1N1	05/13/2009	United Ki	A/England/XFL00482/2009
PB1	CY043289	H1N1	05/13/2009	USA	A/New York/3449/2009(H1N1)
PB1	CY051909	H1N1	05/13/2009	USA	A/Houston/10S/2009(H1N1)
PB1	CY041796	H1N1	05/14/2009	USA	A/New York/3463/2009(H1N1)
PB1	CY046689	H1N1	05/14/2009	USA	A/Wisconsin/629-D00978/2009(H1N
PB1	CY046657	H1N1	05/14/2009	USA	A/Wisconsin/629-D01837/2009(H1N
PB1	CY043137	H1N1	05/14/2009	USA	A/New York/3514/2009(H1N1)
PB1	CY046673	H1N1	05/14/2009	USA	A/Wisconsin/629-D00064/2009(H1N
PB1	CY046665	H1N1	05/14/2009	USA	A/Wisconsin/629-D00997/2009(H1N
PB1	CY083294	H1N1	05/15/2009	USA	A/San Diego/WRAIR1647P/2009
PB1	CY048923	H1N1	05/15/2009	Malaysia	A/Malaysia/820/2009(H1N1)
PB1	CY065524	H1N1	05/15/2009	United Ki	A/England/XFL00532/2009
PB1	CY044979	H1N1	05/15/2009	USA	A/New York/3744/2009(H1N1)
PB1	CY065516	H1N1	05/15/2009	United Ki	A/England/266/2009
PB1	CY050394	H1N1	05/15/2009	USA	A/Wisconsin/629-D01839/2009(H1N
PB1	CY050386	H1N1	05/15/2009	USA	A/Wisconsin/629-D01295/2009(H1N
PB1	CY046633	H1N1	05/15/2009	USA	A/Wisconsin/629-D01839/2009(H1N
PB1	GQ225371	H1N1	05/15/2009	China	A/Beijing/01/2009(H1N1)
PB1	GQ222046	H1N1	05/16/2009	Japan	A/Osaka/1/2009(H1N1)
PB1	CY046641	H1N1	05/16/2009	USA	A/Wisconsin/629-D00967/2009(H1N
PB1	CY046649	H1N1	05/16/2009	USA	A/Wisconsin/629-D02227/2009(H1N
PB1	CY046697	H1N1	05/16/2009	USA	A/Wisconsin/629-D00434/2009(H1N
PB1	GQ222047	H1N1	05/16/2009	Japan	A/Osaka/2/2009(H1N1)
PB1	CY043249	H1N1	05/16/2009	USA	A/New York/3509/2009(H1N1)
PB1	GQ183631	H1N1	05/16/2009	China	A/Beijing/02/2009(H1N1)
PB1	CY062538	H1N1	05/16/2009	Mexico	A/Mexico city/CIA9/2009(H1N1)
PB1	CY048931	H1N1	05/16/2009	Malaysia	A/Malaysia/854/2009(H1N1)
PB1	GQ183623	H1N1	05/16/2009	China	A/Beijing/01/2009(H1N1)
PB1	CY043257	H1N1	05/16/2009	USA	A/New York/3545/2009(H1N1)
PB1	CY043241	H1N1	05/16/2009	USA	A/New York/3502/2009(H1N1)
PB1	CY043193	H1N1	05/16/2009	USA	A/New York/3499/2009(H1N1)
PB1	GQ222048	H1N1	05/16/2009	Japan	A/Osaka-C/1/2009(H1N1)
PB1	CY043297	H1N1	05/16/2009	USA	A/New York/3469/2009(H1N1)
PB1	CY041780	H1N1	05/16/2009	USA	A/New York/3532/2009(H1N1)
PB1	CY044987	H1N1	05/16/2009	USA	A/New York/3747/2009(H1N1)
PB1	CY069867	H1N1	05/17/2009	United Ki	A/England/271/2009
PB1	CY044134	H1N1	05/17/2009	USA	A/New York/3516/2009(H1N1)
PB1	CY043313	H1N1	05/17/2009	USA	A/New York/3534/2009(H1N1)
PB1	CY045091	H1N1	05/17/2009	USA	A/New York/3574/2009(H1N1)
PB1	GQ402319	H1N1	05/17/2009	Canada	A/Canada-QC/RV1954/2009(H1N1)
PB1	GQ222049	H1N1	05/17/2009	Japan	A/Osaka-C/2/2009(H1N1)
PB1	CY083286	H1N1	05/17/2009	USA	A/San Diego/WRAIR1646P/2009
PB1	CY050402	H1N1	05/17/2009	USA	A/Wisconsin/629-D00989/2009(H1N
PB1	GQ221692	H1N1	05/17/2009	China	A/Guangdong/1/2009(H1N1)
PB1	CY043233	H1N1	05/17/2009	USA	A/New York/3501/2009(H1N1)
PB1	CY041804	H1N1	05/17/2009	USA	A/New York/3468/2009(H1N1)
PB1	GQ222044	H1N1	05/17/2009	Japan	A/Hyogo/2/2009(H1N1)
PB1	GQ222043	H1N1	05/17/2009	Japan	A/Hyogo/1/2009(H1N1)
PB1	CY044915	H1N1	05/18/2009	USA	A/New York/3612/2009(H1N1)

PB1	CY052989	H1N1	05/18/2009	USA	A/Houston/30S/2009(H1N1)
PB1	CY051917	H1N1	05/18/2009	USA	A/Houston/2H/2009(H1N1)
PB1	HQ011409	H1N1	05/18/2009	China	A/Guangdong/01/2009(H1N1)
PB1	CY052997	H1N1	05/18/2009	USA	A/Houston/3H/2009(H1N1)
PB1	GQ223442	H1N1	05/18/2009	China	A/Guangdong/1/2009(H1N1)
PB1	GQ267837	H1N1	05/18/2009	Japan	A/Himeji/1/2009(H1N1)
PB1	CY044094	H1N1	05/18/2009	USA	A/New York/3740/2009(H1N1)
PB1	CY052973	H1N1	05/18/2009	USA	A/Houston/20S/2009(H1N1)
PB1	CY044891	H1N1	05/19/2009	USA	A/New York/3580/2009(H1N1)
PB1	CY043145	H1N1	05/19/2009	USA	A/New York/3529/2009(H1N1)
PB1	CY043273	H1N1	05/19/2009	USA	A/New York/3563/2009(H1N1)
PB1	CY050418	H1N1	05/19/2009	USA	A/Wisconsin/629-D00752/2009(H1N
PB1	CY046761	H1N1	05/19/2009	USA	A/Wisconsin/629-D00160/2009(H1N
PB1	CY046745	H1N1	05/19/2009	USA	A/Wisconsin/629-D00084/2009(H1N
PB1	CY053005	H1N1	05/19/2009	USA	A/Houston/50S/2009(H1N1)
PB1	CY053013	H1N1	05/19/2009	USA	A/Houston/5H/2009(H1N1)
PB1	CY045149	H1N1	05/19/2009	USA	A/New York/3577/2009(H1N1)
PB1	GQ300865	H1N1	05/19/2009	Japan	A/Shiga/2/2009(H1N1)
PB1	CY044070	H1N1	05/19/2009	USA	A/New York/3568/2009(H1N1)
PB1	CY044142	H1N1	05/19/2009	USA	A/New York/3528/2009(H1N1)
PB1	CY047364	H1N1	05/19/2009	USA	A/New York/3741/2009(H1N1)
PB1	CY046777	H1N1	05/19/2009	USA	A/Wisconsin/629-D00722/2009(H1N
PB1	CY044923	H1N1	05/19/2009	USA	A/New York/3613/2009(H1N1)
PB1	CY046721	H1N1	05/19/2009	USA	A/Wisconsin/629-D00752/2009(H1N
PB1	CY043265	H1N1	05/20/2009	USA	A/New York/3552/2009(H1N1)
PB1	GQ225379	H1N1	05/20/2009	China	A/Beijing/3/2009(H1N1)
PB1	CY046737	H1N1	05/20/2009	USA	A/Wisconsin/629-D01521/2009(H1N
PB1	CY044875	H1N1	05/20/2009	USA	A/New York/3571/2009(H1N1)
PB1	GQ402320	H1N1	05/20/2009	Canada	A/Canada-MB/RV1964/2009(H1N1)
PB1	CY044899	H1N1	05/20/2009	USA	A/New York/3582/2009(H1N1)
PB1	CY044054	H1N1	05/20/2009	USA	A/New York/3566/2009(H1N1)
PB1	CY044062	H1N1	05/20/2009	USA	A/New York/3567/2009(H1N1)
PB1	CY046713	H1N1	05/20/2009	USA	A/Wisconsin/629-D01787/2009(H1N
PB1	CY043153	H1N1	05/20/2009	USA	A/New York/3551/2009(H1N1)
PB1	CY046729	H1N1	05/20/2009	USA	A/Wisconsin/629-D02427/2009(H1N
PB1	CY044939	H1N1	05/20/2009	USA	A/New York/3623/2009(H1N1)
PB1	CY053021	H1N1	05/20/2009	USA	A/Houston/60S/2009(H1N1)
PB1	CY053188	H1N1	05/20/2009	USA	A/Brownsville/270S/2009(H1N1)
PB1	CY053029	H1N1	05/20/2009	USA	A/Houston/6H/2009(H1N1)
PB1	CY053180	H1N1	05/20/2009	USA	A/Brownsville/260S/2009(H1N1)
PB1	CY052981	H1N1	05/21/2009	USA	A/Houston/20S/2009(H1N1)
PB1	CY053037	H1N1	05/21/2009	USA	A/Houston/70S/2009(H1N1)
PB1	GQ267850	H1N1	05/21/2009	Japan	A/Sakai/2/2009(H1N1)
PB1	GQ219590	H1N1	05/21/2009	Russia	A/Moscow/IIV01/2009(H1N1)
PB1	CY053204	H1N1	05/21/2009	USA	A/Brownsville/300S/2009(H1N1)
PB1	CY053045	H1N1	05/21/2009	USA	A/Houston/7H/2009(H1N1)
PB1	CY044907	H1N1	05/21/2009	USA	A/New York/3611/2009(H1N1)
PB1	CY044971	H1N1	05/21/2009	USA	A/New York/3653/2009(H1N1)
PB1	GQ246617	H1N1	05/21/2009	Russia	A/Moscow/01/2009(H1N1)
PB1	CY051925	H1N1	05/21/2009	USA	A/Houston/90S/2009(H1N1)
PB1	CY046969	H1N1	05/21/2009	USA	A/New York/3576/2009(H1N1)
PB1	GQ402323	H1N1	05/21/2009	Canada	A/Canada-MB/RV1982/2009(H1N1)
PB1	CY053196	H1N1	05/21/2009	USA	A/Brownsville/290S/2009(H1N1)
PB1	GQ267844	H1N1	05/21/2009	Japan	A/Sakai/1/2009(H1N1)

PB1	CY044883	H1N1	05/21/2009	USA	A/New York/3573/2009(H1N1)
PB1	CY044955	H1N1	05/22/2009	USA	A/New York/3627/2009(H1N1)
PB1	CY089185	H1N1	05/22/2009	Chile	A/Chile/19/2009
PB1	GQ402322	H1N1	05/22/2009	Canada	A/Canada-MB/RV1977/2009(H1N1)
PB1	GQ267856	H1N1	05/22/2009	Japan	A/Shiga/1/2009(H1N1)
PB1	CY046753	H1N1	05/22/2009	USA	A/Wisconsin/629-D02064/2009(H1N
PB1	CY046769	H1N1	05/22/2009	USA	A/Wisconsin/629-D02298/2009(H1N
PB1	CY053220	H1N1	05/22/2009	USA	A/Brownsville/31H/2009(H1N1)
PB1	GQ225363	H1N1	05/22/2009	China	A/Fujian/1/2009(H1N1)
PB1	CY053212	H1N1	05/22/2009	USA	A/Brownsville/31OS/2009(H1N1)
PB1	CY045099	H1N1	05/22/2009	USA	A/New York/3614/2009(H1N1)
PB1	CY053236	H1N1	05/23/2009	USA	A/Brownsville/34H/2009(H1N1)
PB1	CY053141	H1N1	05/23/2009	USA	A/Houston/20H/2009(H1N1)
PB1	GQ225347	H1N1	05/23/2009	China	A/Zhejiang/1/2009(H1N1)
PB1	GQ232091	H1N1	05/23/2009	China	A/Beijing/4/2009(H1N1)
PB1	CY053133	H1N1	05/23/2009	USA	A/Houston/20OS/2009(H1N1)
PB1	CY053228	H1N1	05/23/2009	USA	A/Brownsville/34OS/2009(H1N1)
PB1	CY050426	H1N1	05/23/2009	USA	A/Wisconsin/629-D02206/2009(H1N
PB1	GQ225355	H1N1	05/23/2009	China	A/Shanghai/1/2009(H1N1)
PB1	CY049913	H1N1	05/23/2009	Dominican	A/Santo Domingo/0574T/2009(H1N1)
PB1	CY075089	H1N1	05/23/2009	Chile	A/Chile/28/2009(H1N1)
PB1	CY050410	H1N1	05/23/2009	USA	A/Wisconsin/629-D00451/2009(H1N
PB1	GQ402307	H1N1	05/24/2009	Canada	A/Canada-MB/RV2023/2009(H1N1)
PB1	CY041981	H1N1	05/24/2009	Dominican	A/Santo Domingo/0573N/2009(H1N1)
PB1	CY046705	H1N1	05/24/2009	USA	A/Wisconsin/629-D01445/2009(H1N
PB1	GQ402321	H1N1	05/24/2009	Canada	A/Canada-MB/RV1975/2009(H1N1)
PB1	CY045936	H1N1	05/24/2009	Canada	A/Toronto/C0270/2009(H1N1)
PB1	GQ253490	H1N1	05/24/2009	China	A/Shanghai/37T/2009(H1N1)
PB1	CY041958	H1N1	05/24/2009	Dominican	A/Santo Domingo/572N/2009(H1N1)
PB1	CY044963	H1N1	05/24/2009	USA	A/New York/3629/2009(H1N1)
PB1	JQ409124	H1N1	05/25/2009	Finland	A/Helsinki/Vi1/2009
PB1	CY075105	H1N1	05/25/2009	Chile	A/Chile/32/2009(H1N1)
PB1	CY075113	H1N1	05/25/2009	Chile	A/Chile/36/2009(H1N1)
PB1	CY075097	H1N1	05/25/2009	Chile	A/Chile/31/2009
PB1	CY075129	H1N1	05/25/2009	Chile	A/Chile/42/2009(H1N1)
PB1	CY044931	H1N1	05/25/2009	USA	A/New York/3617/2009(H1N1)
PB1	CY050434	H1N1	05/25/2009	USA	A/Wisconsin/629-D02324/2009(H1N
PB1	CY075121	H1N1	05/25/2009	Chile	A/Chile/40/2009(H1N1)
PB1	CY043281	H1N1	05/25/2009	USA	A/New York/3651/2009(H1N1)
PB1	CY043100	H1N1	05/26/2009	Dominican	A/Santo Domingo/565T/2009(H1N1)
PB1	GQ247730	H1N1	05/26/2009	Russia	A/Moscow/IIV02/2009(H1N1)
PB1	GU136052	H1N1	05/26/2009	Japan	A/Shizuoka-C/97/2009(H1N1)
PB1	CY075137	H1N1	05/26/2009	Chile	A/Chile/56/2009(H1N1)
PB1	CY046785	H1N1	05/26/2009	USA	A/Wisconsin/629-D01894/2009(H1N
PB1	GQ385298	H1N1	05/26/2009	Canada	A/Toronto/0462/2009(H1N1)
PB1	CY044947	H1N1	05/26/2009	USA	A/New York/3625/2009(H1N1)
PB1	CY045157	H1N1	05/26/2009	USA	A/New York/3655/2009(H1N1)
PB1	CY046961	H1N1	05/26/2009	USA	A/New York/3654/2009(H1N1)
PB1	CY044078	H1N1	05/26/2009	USA	A/New York/3702/2009(H1N1)
PB1	CY051253	H1N1	05/26/2009	USA	A/Wisconsin/629-S0035/2009(H1N1)
PB1	GQ375282	H1N1	05/26/2009	Russia	A/Moscow/03/2009(H1N1)
PB1	CY043108	H1N1	05/26/2009	Dominican	A/Santo Domingo/568T/2009(H1N1)
PB1	CY069875	H1N1	05/27/2009	United Ki	A/England/350/2009
PB1	HM567686	H1N1	05/27/2009	United Ki	A/England/350/2009

PB1	CY050901	H1N1	05/27/2009	USA	A/Wisconsin/629-D00949/2009(H1N
PB1	CY075145	H1N1	05/27/2009	Chile	A/Chile/88/2009(H1N1)
PB1	CY075153	H1N1	05/27/2009	Chile	A/Chile/95/2009(H1N1)
PB1	CY046817	H1N1	05/27/2009	USA	A/Wisconsin/629-D00698/2009(H1N
PB1	CY046841	H1N1	05/27/2009	USA	A/Wisconsin/629-D01883/2009(H1N
PB1	CY050442	H1N1	05/27/2009	USA	A/Wisconsin/629-D01886/2009(H1N
PB1	CY049929	H1N1	05/27/2009	USA	A/Craven/WR1001/2009(H1N1)
PB1	GQ232083	H1N1	05/27/2009	China	A/Guangdong/02/2009(H1N1)
PB1	GQ283486	H1N1	05/27/2009	Italy	A/Italy/49/2009(H1N1)
PB1	CY046793	H1N1	05/27/2009	USA	A/Wisconsin/629-D02276/2009(H1N
PB1	CY076753	H1N1	05/27/2009	Canada	A/Ontario/142358/2009(H1N1)
PB1	CY053260	H1N1	05/27/2009	USA	A/Brownsville/36H/2009(H1N1)
PB1	CY053244	H1N1	05/27/2009	USA	A/Brownsville/350S/2009(H1N1)
PB1	CY053252	H1N1	05/27/2009	USA	A/Brownsville/35H/2009(H1N1)
PB1	GQ288370	H1N1	05/28/2009	China	A/Fuzhou/01/2009(H1N1)
PB1	CY050909	H1N1	05/28/2009	USA	A/Wisconsin/629-D01779/2009(H1N
PB1	CY053315	H1N1	05/28/2009	USA	A/Brownsville/370S/2009(H1N1)
PB1	CY064789	H1N1	05/28/2009	China	A/Guangdong/SB1/2009(H1N1)
PB1	CY075161	H1N1	05/28/2009	Chile	A/Chile/158/2009(H1N1)
PB1	GQ402324	H1N1	05/28/2009	Canada	A/Canada-MB/RV2013/2009(H1N1)
PB1	CY046905	H1N1	05/28/2009	USA	A/Wisconsin/629-D00829/2009(H1N
PB1	CY046913	H1N1	05/28/2009	USA	A/Wisconsin/629-D01083/2009(H1N
PB1	CY050917	H1N1	05/28/2009	USA	A/Wisconsin/629-D01262/2009(H1N
PB1	CY050156	H1N1	05/28/2009	USA	A/Wisconsin/629-D00117/2009(H1N
PB1	CY046833	H1N1	05/28/2009	USA	A/Wisconsin/629-D02144/2009(H1N
PB1	CY045011	H1N1	05/28/2009	USA	A/New York/3709/2009(H1N1)
PB1	CY046801	H1N1	05/28/2009	USA	A/Wisconsin/629-D01026/2009(H1N
PB1	GQ290435	H1N1	05/28/2009	China	A/Shanghai/60T/2009(H1N1)
PB1	CY053267	H1N1	05/28/2009	USA	A/Brownsville/37H/2009(H1N1)
PB1	CY044086	H1N1	05/29/2009	USA	A/New York/3715/2009(H1N1)
PB1	CY051261	H1N1	05/29/2009	USA	A/Wisconsin/629-S0086/2009(H1N1)
PB1	CY075169	H1N1	05/29/2009	Chile	A/Chile/180/2009(H1N1)
PB1	CY050925	H1N1	05/29/2009	USA	A/Wisconsin/629-D02213/2009(H1N
PB1	CY050941	H1N1	05/29/2009	USA	A/Wisconsin/629-D01847/2009(H1N
PB1	CY050933	H1N1	05/29/2009	USA	A/Wisconsin/629-D00426/2009(H1N
PB1	GQ250159	H1N1	05/29/2009	China	A/Guangdong/03/2009(H1N1)
PB1	CY071037	H1N1	05/29/2009	USA	A/New York/NHRC0002/2009(H1N1)
PB1	GQ402325	H1N1	05/29/2009	Canada	A/Canada-MB/RV2018/2009(H1N1)
PB1	CY055301	H1N1	05/30/2009	Singapore	A/Singapore/GN285/2009(H1N1)
PB1	CY046825	H1N1	05/30/2009	USA	A/Wisconsin/629-D02013/2009(H1N
PB1	CY046809	H1N1	05/30/2009	USA	A/Wisconsin/629-D00223/2009(H1N
PB1	GQ402326	H1N1	05/30/2009	Canada	A/Canada-MB/RV2020/2009(H1N1)
PB1	CY053283	H1N1	05/30/2009	USA	A/Brownsville/39H/2009(H1N1)
PB1	HM569665	H1N1	05/30/2009	Argentina	A/Argentina/07-09GP/2009(H1N1)
PB1	CY053275	H1N1	05/30/2009	USA	A/Brownsville/390S/2009(H1N1)
PB1	CY045960	H1N1	05/30/2009	Canada	A/Toronto/T5294/2009(H1N1)
PB1	GQ253500	H1N1	05/31/2009	China	A/Shanghai/71T/2009(H1N1)
PB1	CY071029	H1N1	05/31/2009	USA	A/New York/NHRC0001/2009(H1N1)
PB1	CY065532	H1N1	05/31/2009	United Ki	A/England/359/2009
PB1	GQ293076	H1N1	05/31/2009	China	A/Zhejiang/2/2009(H1N1)
PB1	CY044867	H1N1	05/31/2009	USA	A/New York/3726/2009(H1N1)
PB1	CY045035	H1N1	05/31/2009	USA	A/New York/3812/2009(H1N1)
PB1	CY045189	H1N1	05/31/2009	USA	A/New York/3960/2009(H1N1)
PB1	GQ359764	H1N1	06/2009	China	A/Zhejiang/DTID-ZJU01/2009(H1N1)

PB1	CY069632	H1N1	06/2009	Singapore	A/Singapore/478/2009(H1N1)
PB1	HQ695918	H1N1	06/2009	Taiwan	A/Taiwan/14/2009
PB1	CY096725	H1N1	06/2009	Singapore	A/Singapore/276/2009
PB1	CY069624	H1N1	06/2009	Singapore	A/Singapore/471/2009(H1N1)
PB1	CY045240	H1N1	06/2009	Taiwan	A/Taiwan/137/2009(H1N1)
PB1	CY069639	H1N1	06/2009	Singapore	A/Singapore/527/2009(H1N1)
PB1	CY047785	H1N1	06/2009	Argentina	A/Argentina/7785/2009
PB1	CY045232	H1N1	06/2009	Taiwan	A/Taiwan/126/2009(H1N1)
PB1	CY050949	H1N1	06/01/2009	USA	A/Wisconsin/629-D00374/2009(H1N
PB1	CY045181	H1N1	06/01/2009	USA	A/New York/3959/2009(H1N1)
PB1	CY047372	H1N1	06/01/2009	USA	A/New York/3795/2009(H1N1)
PB1	CY050973	H1N1	06/01/2009	USA	A/Wisconsin/629-D01725/2009(H1N
PB1	KC800978	H1N1	06/01/2009	France	A/Lyon/969/2009
PB1	CY065540	H1N1	06/01/2009	United Ki	A/England/361/2009
PB1	CY065572	H1N1	06/01/2009	United Ki	A/England/385/2009
PB1	CY065644	H1N1	06/01/2009	United Ki	A/England/402/2009
PB1	HM567766	H1N1	06/01/2009	United Ki	A/England/383/2009
PB1	CY065556	H1N1	06/01/2009	United Ki	A/England/382/2009
PB1	CY065580	H1N1	06/01/2009	United Ki	A/England/386/2009
PB1	CY065620	H1N1	06/01/2009	United Ki	A/England/395/2009
PB1	CY069883	H1N1	06/01/2009	United Ki	A/England/383/2009
PB1	CY071045	H1N1	06/01/2009	USA	A/New York/NHRC0003/2009(H1N1)
PB1	CY076761	H1N1	06/01/2009	Canada	A/Ontario/147265/2009(H1N1)
PB1	FN423710	H1N1	06/01/2009	Luxembour	A/Luxembourg/43/2009
PB1	CY065548	H1N1	06/01/2009	United Ki	A/England/381/2009
PB1	CY054697	H1N1	06/01/2009	Nicaragua	A/Managua/4467.05/2009(H1N1)
PB1	CY065564	H1N1	06/01/2009	United Ki	A/England/384/2009
PB1	CY050965	H1N1	06/01/2009	USA	A/Wisconsin/629-D00941/2009(H1N
PB1	CY050957	H1N1	06/01/2009	USA	A/Wisconsin/629-D00724/2009(H1N
PB1	CY053291	H1N1	06/01/2009	USA	A/Brownsville/400S/2009(H1N1)
PB1	CY056393	H1N1	06/01/2009	Nicaragua	A/Managua/3119.01/2009(H1N1)
PB1	CY044995	H1N1	06/01/2009	USA	A/New York/3751/2009(H1N1)
PB1	CY050989	H1N1	06/02/2009	USA	A/Wisconsin/629-D00592/2009(H1N
PB1	CY053299	H1N1	06/02/2009	USA	A/Brownsville/430S/2009(H1N1)
PB1	CY053307	H1N1	06/02/2009	USA	A/Brownsville/43H/2009(H1N1)
PB1	CY051269	H1N1	06/02/2009	USA	A/Wisconsin/629-S0143/2009(H1N1)
PB1	CY051277	H1N1	06/02/2009	USA	A/Wisconsin/629-S0146/2009(H1N1)
PB1	CY045003	H1N1	06/02/2009	USA	A/New York/3828/2009(H1N1)
PB1	CY045019	H1N1	06/02/2009	USA	A/New York/3796/2009(H1N1)
PB1	CY045027	H1N1	06/02/2009	USA	A/New York/3803/2009(H1N1)
PB1	GQ324576	H1N1	06/02/2009	Japan	A/Tokushima/1/2009(H1N1)
PB1	CY045165	H1N1	06/02/2009	USA	A/New York/3818/2009(H1N1)
PB1	CY065612	H1N1	06/02/2009	United Ki	A/England/394/2009
PB1	CY050981	H1N1	06/02/2009	USA	A/Wisconsin/629-D00367/2009(H1N
PB1	HM567782	H1N1	06/02/2009	United Ki	A/England/393/2009
PB1	CY069891	H1N1	06/02/2009	United Ki	A/England/391/2009
PB1	HM567774	H1N1	06/02/2009	United Ki	A/England/391/2009
PB1	CY045173	H1N1	06/02/2009	USA	A/New York/3819/2009(H1N1)
PB1	CY069899	H1N1	06/02/2009	United Ki	A/England/393/2009
PB1	GQ334353	H1N1	06/02/2009	Japan	A/Shizuoka/759/2009(H1N1)
PB1	CY076769	H1N1	06/02/2009	Canada	A/Ontario/147723/2009(H1N1)
PB1	CY051285	H1N1	06/02/2009	USA	A/Wisconsin/629-S0148/2009(H1N1)
PB1	CY051229	H1N1	06/02/2009	USA	A/Wisconsin/629-D00022/2009(H1N
PB1	CY065588	H1N1	06/02/2009	United Ki	A/England/388/2009

PB1	CY051237	H1N1	06/03/2009	USA	A/Wisconsin/629-D01664/2009(H1N
PB1	CY065604	H1N1	06/03/2009	United Ki	A/England/392/2009
PB1	CY065596	H1N1	06/03/2009	United Ki	A/England/389/2009
PB1	CY107576	H1N1	06/03/2009	United Ki	A/Scotland/Glasgow_412313/2009
PB1	CY065628	H1N1	06/03/2009	United Ki	A/England/398/2009
PB1	CY054689	H1N1	06/03/2009	USA	A/Wisconsin/629-D00589/2009(H1N
PB1	CY071053	H1N1	06/03/2009	USA	A/New York/NHRC0004/2009(H1N1)
PB1	CY044102	H1N1	06/03/2009	USA	A/New York/3895/2009(H1N1)
PB1	GQ330651	H1N1	06/03/2009	Russia	A/Moscow/IIV03/2009(H1N1)
PB1	GQ411903	H1N1	06/03/2009	Canada	A/Toronto/T5308/2009(H1N1)
PB1	GU136055	H1N1	06/03/2009	Japan	A/Yamaguchi/21/2009(H1N1)
PB1	KC781930	H1N1	06/03/2009	USA	A/Virginia/24/2009
PB1	KC781443	H1N1	06/03/2009	USA	A/Utah/27/2009
PB1	CY051037	H1N1	06/04/2009	USA	A/Wisconsin/629-D00658/2009(H1N
PB1	CY051045	H1N1	06/04/2009	USA	A/Wisconsin/629-D00725/2009(H1N
PB1	CY043340	H1N1	06/04/2009	Denmark	A/Denmark/524/2009(H1N1)
PB1	CY065692	H1N1	06/04/2009	United Ki	A/England/428/2009
PB1	CY065660	H1N1	06/04/2009	United Ki	A/England/414/2009
PB1	CY065684	H1N1	06/04/2009	United Ki	A/England/423/2009
PB1	CY076777	H1N1	06/04/2009	Canada	A/Ontario/152439/2009(H1N1)
PB1	CY089902	H1N1	06/04/2009	USA	A/Wisconsin/629-D01420/2009(H1N
PB1	CY065652	H1N1	06/04/2009	United Ki	A/England/411/2009
PB1	CY051021	H1N1	06/04/2009	USA	A/Wisconsin/629-D00453/2009(H1N
PB1	CY051029	H1N1	06/04/2009	USA	A/Wisconsin/629-D00017/2009(H1N
PB1	CY076785	H1N1	06/04/2009	Canada	A/Ontario/152846/2009(H1N1)
PB1	CY051293	H1N1	06/04/2009	USA	A/Wisconsin/629-S0173/2009(H1N1)
PB1	CY051053	H1N1	06/04/2009	USA	A/Wisconsin/629-D00008/2009(H1N
PB1	CY044110	H1N1	06/05/2009	USA	A/New York/3898/2009(H1N1)
PB1	CY065708	H1N1	06/05/2009	United Ki	A/England/431/2009
PB1	CY065668	H1N1	06/05/2009	United Ki	A/England/418/2009
PB1	CY065716	H1N1	06/05/2009	United Ki	A/England/433/2009
PB1	CY065636	H1N1	06/05/2009	United Ki	A/England/400/2009
PB1	CY072972	H1N1	06/05/2009	Nicaragua	A/Managua/2750.01/2009(H1N1)
PB1	CY072916	H1N1	06/05/2009	Nicaragua	A/Managua/2652.01/2009(H1N1)
PB1	CY065676	H1N1	06/05/2009	United Ki	A/England/419/2009
PB1	CY065700	H1N1	06/05/2009	United Ki	A/England/430/2009
PB1	CY058530	H1N1	06/05/2009	Nicaragua	A/Managua/4905.02/2009(H1N1)
PB1	CY052144	H1N1	06/05/2009	USA	A/New York/3980/2009(H1N1)
PB1	CY058170	H1N1	06/05/2009	Nicaragua	A/Managua/4016.02/2009(H1N1)
PB1	CY050997	H1N1	06/05/2009	USA	A/Wisconsin/629-D01903/2009(H1N
PB1	GQ324570	H1N1	06/06/2009	Japan	A/Shiga/3/2009(H1N1)
PB1	JX625980	H1N1	06/06/2009	United Ki	A/England/421/2009
PB1	CY058090	H1N1	06/06/2009	Nicaragua	A/Managua/1455.01/2009(H1N1)
PB1	CY083597	H1N1	06/06/2009	Nicaragua	A/Managua/WRAIR8964F/2009(H1N1)
PB1	CY065724	H1N1	06/06/2009	United Ki	A/England/434/2009
PB1	HM567870	H1N1	06/06/2009	United Ki	A/England/421/2009
PB1	CY053125	H1N1	06/07/2009	USA	A/Houston/170S/2009(H1N1)
PB1	CY058082	H1N1	06/07/2009	Nicaragua	A/Managua/171.01/2009(H1N1)
PB1	GQ300859	H1N1	06/07/2009	Japan	A/Fukuoka-C/1/2009(H1N1)
PB1	GQ334336	H1N1	06/07/2009	Japan	A/Fukuoka-C/2/2009(H1N1)
PB1	GQ334344	H1N1	06/07/2009	Japan	A/Fukuoka-C/3/2009(H1N1)
PB1	CY051061	H1N1	06/08/2009	USA	A/Wisconsin/629-D02068/2009(H1N
PB1	CY076793	H1N1	06/08/2009	Canada	A/Ontario/156785/2009(H1N1)
PB1	CY051309	H1N1	06/08/2009	USA	A/Wisconsin/629-S0197/2009(H1N1)

PB1	CY052136	H1N1	06/08/2009	USA	A/New York/3976/2009(H1N1)
PB1	CY083150	H1N1	06/08/2009	Dominican	A/Santo Domingo/WRAIR1044N/2009
PB1	CY051325	H1N1	06/08/2009	USA	A/Wisconsin/629-S0202/2009(H1N1)
PB1	CY051301	H1N1	06/08/2009	USA	A/Wisconsin/629-S0186/2009(H1N1)
PB1	CY051317	H1N1	06/08/2009	USA	A/Wisconsin/629-S0201/2009(H1N1)
PB1	CY051077	H1N1	06/08/2009	USA	A/Wisconsin/629-D00579/2009(H1N
PB1	CY045213	H1N1	06/08/2009	USA	A/New York/4057/2009(H1N1)
PB1	CY051501	H1N1	06/08/2009	USA	A/Wisconsin/629-S0189/2009(H1N1)
PB1	CY051509	H1N1	06/08/2009	USA	A/Wisconsin/629-S0206/2009(H1N1)
PB1	CY058138	H1N1	06/08/2009	Nicaragua	A/Managua/516.01/2009(H1N1)
PB1	KC781790	H1N1	06/08/2009	USA	A/Wisconsin/26/2009
PB1	CY051333	H1N1	06/08/2009	USA	A/Wisconsin/629-S0203/2009(H1N1)
PB1	CY051069	H1N1	06/08/2009	USA	A/Wisconsin/629-D00101/2009(H1N
PB1	CY043348	H1N1	06/09/2009	Denmark	A/Denmark/528/2009(H1N1)
PB1	CY051349	H1N1	06/09/2009	USA	A/Wisconsin/629-S0222/2009(H1N1)
PB1	CY052128	H1N1	06/09/2009	USA	A/New York/3969/2009(H1N1)
PB1	CY051341	H1N1	06/09/2009	USA	A/Wisconsin/629-S0220/2009(H1N1)
PB1	CY058106	H1N1	06/09/2009	Nicaragua	A/Managua/168.02/2009(H1N1)
PB1	GQ375887	H1N1	06/09/2009	Japan	A/Iwate/1/2009(H1N1)
PB1	CY045968	H1N1	06/09/2009	Canada	A/Toronto/T5362/2009(H1N1)
PB1	CY065732	H1N1	06/09/2009	United Ki	A/England/445/2009
PB1	CY045043	H1N1	06/09/2009	USA	A/New York/3967/2009(H1N1)
PB1	CY045059	H1N1	06/09/2009	USA	A/New York/4052/2009(H1N1)
PB1	CY051357	H1N1	06/09/2009	USA	A/Wisconsin/629-S0226/2009(H1N1)
PB1	CY052296	H1N1	06/09/2009	USA	A/Texas/42091791/2009(H1N1)
PB1	CY051517	H1N1	06/09/2009	USA	A/Wisconsin/629-S0208/2009(H1N1)
PB1	GQ324582	H1N1	06/09/2009	Japan	A/Yokohama/1/2009(H1N1)
PB1	CY051245	H1N1	06/09/2009	USA	A/Wisconsin/629-D01199/2009(H1N
PB1	CY044118	H1N1	06/10/2009	USA	A/New York/3992/2009(H1N1)
PB1	CY045197	H1N1	06/10/2009	USA	A/New York/4014/2009(H1N1)
PB1	CY045051	H1N1	06/10/2009	USA	A/New York/4006/2009(H1N1)
PB1	CY045221	H1N1	06/10/2009	USA	A/New York/4074/2009(H1N1)
PB1	CY051381	H1N1	06/10/2009	USA	A/Wisconsin/629-S0247/2009(H1N1)
PB1	CY045205	H1N1	06/10/2009	USA	A/New York/4048/2009(H1N1)
PB1	CY051365	H1N1	06/10/2009	USA	A/Wisconsin/629-S0245/2009(H1N1)
PB1	CY051405	H1N1	06/10/2009	USA	A/Wisconsin/629-S0252/2009(H1N1)
PB1	CY051397	H1N1	06/10/2009	USA	A/Wisconsin/629-S0255/2009(H1N1)
PB1	CY051373	H1N1	06/10/2009	USA	A/Wisconsin/629-S0248/2009(H1N1)
PB1	CY051389	H1N1	06/10/2009	USA	A/Wisconsin/629-S0254/2009(H1N1)
PB1	CY052272	H1N1	06/10/2009	USA	A/Texas/42102708/2009(H1N1)
PB1	CY052304	H1N1	06/10/2009	USA	A/Texas/42103399/2009(H1N1)
PB1	CY051893	H1N1	06/11/2009	USA	A/Texas/42114261/2009(H1N1)
PB1	CY063492	H1N1	06/11/2009	USA	A/Boston/110/2009(H1N1)
PB1	GQ365416	H1N1	06/11/2009	Japan	A/Akita/1/2009(H1N1)
PB1	CY065740	H1N1	06/11/2009	United Ki	A/Scotland/10/2009
PB1	CY057212	H1N1	06/11/2009	Nicaragua	A/Managua/167.01/2009(H1N1)
PB1	GQ365452	H1N1	06/11/2009	Japan	A/Sapporo/1/2009(H1N1)
PB1	CY051413	H1N1	06/11/2009	USA	A/Wisconsin/629-S0269/2009(H1N1)
PB1	CY053117	H1N1	06/11/2009	USA	A/Houston/160S/2009(H1N1)
PB1	CY051085	H1N1	06/11/2009	USA	A/Wisconsin/629-D01326/2009(H1N
PB1	CY051885	H1N1	06/11/2009	USA	A/Texas/42113095/2009(H1N1)
PB1	CY052200	H1N1	06/12/2009	USA	A/Texas/42121926/2009(H1N1)
PB1	HM569689	H1N1	06/12/2009	Argentina	A/Argentina/19618/2009(H1N1)
PB1	CY051117	H1N1	06/12/2009	USA	A/Wisconsin/629-D02263/2009(H1N

PB1	CY058098	H1N1	06/12/2009	Nicaragua	A/Managua/655.02/2009(H1N1)
PB1	CY051845	H1N1	06/12/2009	USA	A/Texas/42123701/2009(H1N1)
PB1	CY050164	H1N1	06/12/2009	USA	A/New York/4095/2009(H1N1)
PB1	CY051861	H1N1	06/12/2009	USA	A/Texas/42122969/2009(H1N1)
PB1	KT180335	H1N1	06/12/2009	Singapore	A/Singapore/DMS1/2009
PB1	CY058218	H1N1	06/12/2009	Nicaragua	A/Managua/578.01/2009(H1N1)
PB1	GU136037	H1N1	06/12/2009	Japan	A/Kanagawa/137/2009(H1N1)
PB1	CY045083	H1N1	06/12/2009	USA	A/New York/4072/2009(H1N1)
PB1	GQ866949	H1N1	06/13/2009	Thailand	A/Thailand/CU-B5/2009(H1N1)
PB1	KC782096	H1N1	06/13/2009	USA	A/Rhode Island/15/2009
PB1	GQ365442	H1N1	06/13/2009	Japan	A/Kagoshima/1/2009(H1N1)
PB1	GQ463198	H1N1	06/13/2009	China	A/Hunan/SWL3/2009(H1N1)
PB1	CY058114	H1N1	06/13/2009	Nicaragua	A/Managua/2152.01/2009(H1N1)
PB1	CY058234	H1N1	06/13/2009	Nicaragua	A/Managua/5363.02/2009(H1N1)
PB1	CY058226	H1N1	06/13/2009	Nicaragua	A/Managua/5221.03/2009(H1N1)
PB1	CY075177	H1N1	06/13/2009	Chile	A/Chile/1586/2009(H1N1)
PB1	CY051005	H1N1	06/13/2009	USA	A/Wisconsin/629-D00665/2009(H1N
PB1	CY051109	H1N1	06/13/2009	USA	A/Wisconsin/629-D01618/2009(H1N
PB1	CY051101	H1N1	06/13/2009	USA	A/Wisconsin/629-D00196/2009(H1N
PB1	CY054753	H1N1	06/13/2009	USA	A/California/VRDL9/2009(H1N1)
PB1	CY058122	H1N1	06/13/2009	Nicaragua	A/Managua/1637.01/2009(H1N1)
PB1	CY057220	H1N1	06/13/2009	Nicaragua	A/Managua/109.01/2009(H1N1)
PB1	CY058178	H1N1	06/13/2009	Nicaragua	A/Managua/792.01/2009(H1N1)
PB1	CY064466	H1N1	06/13/2009	USA	A/Boston/96/2009(H1N1)
PB1	CY052320	H1N1	06/13/2009	USA	A/Texas/42132413/2009(H1N1)
PB1	CY054729	H1N1	06/13/2009	USA	A/California/VRDL6/2009(H1N1)
PB1	JQ409140	H1N1	06/13/2009	Finland	A/Helsinki/Vi3/2009(H1N1)
PB1	CY083501	H1N1	06/13/2009	Nicaragua	A/Managua/WRAIR1693P/2009(H1N1)
PB1	GU136030	H1N1	06/13/2009	Japan	A/Hiroshima/200/2009(H1N1)
PB1	JQ409132	H1N1	06/13/2009	Finland	A/Helsinki/Vi2/2009(H1N1)
PB1	CY063500	H1N1	06/13/2009	USA	A/Boston/112/2009(H1N1)
PB1	CY064506	H1N1	06/13/2009	USA	A/Boston/115/2009(H1N1)
PB1	CY058154	H1N1	06/13/2009	Nicaragua	A/Managua/4085.02/2009(H1N1)
PB1	CY052397	H1N1	06/14/2009	USA	A/Texas/42142537/2009(H1N1)
PB1	CY051133	H1N1	06/14/2009	USA	A/Wisconsin/629-D00119/2009(H1N
PB1	GQ457463	H1N1	06/14/2009	USA	A/Utah/05/2009(H1N1)
PB1	CY054713	H1N1	06/14/2009	USA	A/California/VRDL4/2009(H1N1)
PB1	CY051125	H1N1	06/14/2009	USA	A/Wisconsin/629-D00916/2009(H1N
PB1	GQ351288	H1N1	06/14/2009	Italy	A/Italy/85/2009(H1N1)
PB1	CY049921	H1N1	06/14/2009	USA	A/New Bern/WR0670/2009(H1N1)
PB1	KJ130170	H1N1	06/14/2009	New Zeala	A/New Zealand/1212a/2009(H1N1)
PB1	KJ130186	H1N1	06/14/2009	New Zeala	A/New Zealand/1212f/2009
PB1	KJ130146	H1N1	06/14/2009	New Zeala	A/New Zealand/1212c/2009
PB1	CY051157	H1N1	06/14/2009	USA	A/Wisconsin/629-D01147/2009(H1N
PB1	CY054745	H1N1	06/14/2009	USA	A/California/VRDL8/2009(H1N1)
PB1	GQ433895	H1N1	06/15/2009	China	A/Jiangsu/1/2009(H1N1)
PB1	CY064498	H1N1	06/15/2009	USA	A/Boston/109/2009(H1N1)
PB1	CY064474	H1N1	06/15/2009	USA	A/Boston/103/2009(H1N1)
PB1	CY058554	H1N1	06/15/2009	Nicaragua	A/Managua/4935.03/2009(H1N1)
PB1	HM569681	H1N1	06/15/2009	Argentina	A/Argentina/19527/2009(H1N1)
PB1	CY051421	H1N1	06/15/2009	USA	A/Wisconsin/629-S0280/2009(H1N1)
PB1	CY051429	H1N1	06/15/2009	USA	A/Wisconsin/629-S0279/2009(H1N1)
PB1	CY051013	H1N1	06/15/2009	USA	A/Wisconsin/629-D01619/2009(H1N
PB1	CY075185	H1N1	06/15/2009	Chile	A/Chile/1598/2009(H1N1)

PB1	CY075217	H1N1	06/15/2009	Chile	A/Chile/1624/2009(H1N1)
PB1	CY051821	H1N1	06/15/2009	USA	A/Texas/42152486/2009(H1N1)
PB1	GU136046	H1N1	06/15/2009	Japan	A/Niigata/690/2009(H1N1)
PB1	KC781553	H1N1	06/15/2009	USA	A/Massachusetts/16/2009
PB1	CY058130	H1N1	06/15/2009	Nicaragua	A/Managua/5453.01/2009(H1N1)
PB1	CY064482	H1N1	06/15/2009	USA	A/Boston/106/2009(H1N1)
PB1	CY063508	H1N1	06/15/2009	USA	A/Boston/113/2009(H1N1)
PB1	CY060748	H1N1	06/15/2009	Canada	A/Ontario/9739/2009(H1N1)
PB1	KC781702	H1N1	06/15/2009	USA	A/Oregon/10/2009
PB1	CY060740	H1N1	06/15/2009	Canada	A/Ontario/9698/2009(H1N1)
PB1	CY055461	H1N1	06/15/2009	USA	A/California/VRDL12/2009(H1N1)
PB1	CY075201	H1N1	06/15/2009	Chile	A/Chile/1600/2009(H1N1)
PB1	CY075193	H1N1	06/15/2009	Chile	A/Chile/1599/2009(H1N1)
PB1	CY075209	H1N1	06/15/2009	Chile	A/Chile/1603/2009(H1N1)
PB1	CY052280	H1N1	06/15/2009	USA	A/Texas/42151049/2009(H1N1)
PB1	CY054721	H1N1	06/15/2009	USA	A/California/VRDL5/2009(H1N1)
PB1	CY051437	H1N1	06/15/2009	USA	A/Wisconsin/629-S0293/2009(H1N1)
PB1	CY051093	H1N1	06/15/2009	USA	A/Wisconsin/629-D01844/2009(H1N
PB1	CY051141	H1N1	06/15/2009	USA	A/Wisconsin/629-D01152/2009(H1N
PB1	CY058162	H1N1	06/16/2009	Nicaragua	A/Managua/2330.02/2009(H1N1)
PB1	CY051165	H1N1	06/16/2009	USA	A/Wisconsin/629-D00541/2009(H1N
PB1	GQ334361	H1N1	06/16/2009	Japan	A/Utsunomiya/1/2009(H1N1)
PB1	CY051469	H1N1	06/16/2009	USA	A/Wisconsin/629-S0314/2009(H1N1)
PB1	CY052389	H1N1	06/16/2009	USA	A/Texas/42163295/2009(H1N1)
PB1	GQ365461	H1N1	06/16/2009	Japan	A/Utsunomiya/2/2009(H1N1)
PB1	CY052216	H1N1	06/16/2009	USA	A/Texas/42163291/2009(H1N1)
PB1	CY064490	H1N1	06/16/2009	USA	A/Boston/108/2009(H1N1)
PB1	CY044169	H1N1	06/16/2009	USA	A/Bethesda/SP506/2009(H1N1)
PB1	GU136026	H1N1	06/16/2009	Japan	A/Aichi/202/2009(H1N1)
PB1	CY051149	H1N1	06/16/2009	USA	A/Wisconsin/629-D01734/2009(H1N
PB1	CY107191	H1N1	06/17/2009	United Ki	A/Scotland/Paisley_417557/2009
PB1	CY047777	H1N1	06/17/2009	Argentina	A/Argentina/7649/2009
PB1	GQ455030	H1N1	06/17/2009	China	A/Nanjing/2/2009(H1N1)
PB1	CY054737	H1N1	06/17/2009	USA	A/California/VRDL7/2009(H1N1)
PB1	CY054849	H1N1	06/17/2009	USA	A/California/VRDL31/2009(H1N1)
PB1	GQ392027	H1N1	06/17/2009	Italy	A/Italy/127/2009(H1N1)
PB1	CY052104	H1N1	06/17/2009	USA	A/New York/4238/2009(H1N1)
PB1	CY047809	H1N1	06/17/2009	Argentina	A/Argentina/7967/2009
PB1	CY047817	H1N1	06/17/2009	Argentina	A/Argentina/7980/2009
PB1	CY055453	H1N1	06/17/2009	USA	A/California/VRDL11/2009(H1N1)
PB1	CY051549	H1N1	06/17/2009	USA	A/New York/4403/2009(H1N1)
PB1	CY051445	H1N1	06/17/2009	USA	A/Wisconsin/629-S0319/2009(H1N1)
PB1	CY051877	H1N1	06/17/2009	USA	A/Texas/42173957/2009(H1N1)
PB1	CY064594	H1N1	06/17/2009	USA	A/Boston/127/2009(H1N1)
PB1	CY064578	H1N1	06/17/2009	USA	A/Boston/125/2009(H1N1)
PB1	CY044126	H1N1	06/17/2009	USA	A/New York/4197/2009(H1N1)
PB1	GU136031	H1N1	06/17/2009	Japan	A/Hiroshima/201/2009(H1N1)
PB1	CY045976	H1N1	06/17/2009	Canada	A/Toronto/T9842/2009(H1N1)
PB1	GQ866957	H1N1	06/17/2009	Thailand	A/Thailand/CU-H9/2009(H1N1)
PB1	GQ365434	H1N1	06/18/2009	Japan	A/Iwate/2/2009(H1N1)
PB1	CY073276	H1N1	06/18/2009	USA	A/Silver Spring/WRAIRSP510P18/20
PB1	CY064650	H1N1	06/18/2009	USA	A/Boston/135/2009(H1N1)
PB1	CY064634	H1N1	06/18/2009	USA	A/Boston/133/2009(H1N1)
PB1	CY051453	H1N1	06/18/2009	USA	A/Wisconsin/629-S0345/2009(H1N1)

PB1	CY064666	H1N1	06/18/2009	USA	A/Boston/138/2009(H1N1)
PB1	CY054809	H1N1	06/18/2009	USA	A/California/VRDL26/2009(H1N1)
PB1	CY051461	H1N1	06/18/2009	USA	A/Wisconsin/629-S0339/2009(H1N1)
PB1	CY051173	H1N1	06/18/2009	USA	A/Wisconsin/629-D02063/2009(H1N
PB1	HM569697	H1N1	06/18/2009	Argentina	A/Argentina/19656/2009(H1N1)
PB1	CY075225	H1N1	06/18/2009	Chile	A/Chile/2009/2009(H1N1)
PB1	GU136038	H1N1	06/18/2009	Japan	A/Kanagawa/140/2009(H1N1)
PB1	CY047825	H1N1	06/18/2009	Argentina	A/Argentina/8019/2009
PB1	CY064562	H1N1	06/18/2009	USA	A/Boston/123/2009(H1N1)
PB1	CY043116	H1N1	06/18/2009	USA	A/Bethesda/SP508/2009(H1N1)
PB1	GU136028	H1N1	06/18/2009	Japan	A/Chiba-C/51/2009(H1N1)
PB1	KC781850	H1N1	06/18/2009	USA	A/Oregon/13/2009
PB1	KC781809	H1N1	06/18/2009	USA	A/Oregon/11/2009
PB1	CY073292	H1N1	06/18/2009	USA	A/Silver Spring/WRAIRSP510P20/2(
PB1	GQ504750	H1N1	06/18/2009	China	A/Nanjing/1/2009(H1N1)
PB1	CY064538	H1N1	06/19/2009	USA	A/Boston/120/2009(H1N1)
PB1	CY047849	H1N1	06/19/2009	Argentina	A/Argentina/8673/2009
PB1	CY123919	H1N1	06/19/2009	Singapore	A/Singapore/ON313/2009
PB1	CY064626	H1N1	06/19/2009	USA	A/Boston/132/2009(H1N1)
PB1	CY047801	H1N1	06/19/2009	Argentina	A/Argentina/7953/2009
PB1	CY052421	H1N1	06/19/2009	USA	A/Texas/42192947/2009(H1N1)
PB1	CY051565	H1N1	06/19/2009	USA	A/New York/4459/2009(H1N1)
PB1	CY052240	H1N1	06/19/2009	USA	A/Texas/42191647/2009(H1N1)
PB1	CY075233	H1N1	06/19/2009	Chile	A/Chile/2239/2009(H1N1)
PB1	CY064546	H1N1	06/19/2009	USA	A/Boston/121/2009(H1N1)
PB1	CY083086	H1N1	06/19/2009	Colombia	A/Bogota/WRAIR0442T/2009
PB1	CY051853	H1N1	06/19/2009	USA	A/Texas/42191653/2009(H1N1)
PB1	CY045075	H1N1	06/19/2009	USA	A/New York/4290/2009(H1N1)
PB1	CY064530	H1N1	06/20/2009	USA	A/Boston/118/2009(H1N1)
PB1	KT180470	H1N1	06/20/2009	Singapore	A/Singapore/DMS385/2009
PB1	GQ496137	H1N1	06/20/2009	Russia	A/Moscow/IIV05/2009(H1N1)
PB1	CY051181	H1N1	06/20/2009	USA	A/Wisconsin/629-D00428/2009(H1N
PB1	CY052248	H1N1	06/20/2009	USA	A/Texas/42201798/2009(H1N1)
PB1	CY072988	H1N1	06/20/2009	Nicaragua	A/Managua/5273.02/2009(H1N1)
PB1	CY052328	H1N1	06/20/2009	USA	A/Texas/42202026/2009(H1N1)
PB1	CY064658	H1N1	06/20/2009	USA	A/Boston/137/2009(H1N1)
PB1	CY051869	H1N1	06/20/2009	USA	A/Texas/42201824/2009(H1N1)
PB1	CY051197	H1N1	06/20/2009	USA	A/Wisconsin/629-D00496/2009(H1N
PB1	CY054769	H1N1	06/20/2009	USA	A/California/VRDL20/2009(H1N1)
PB1	CY075241	H1N1	06/20/2009	Chile	A/Chile/2851/2009(H1N1)
PB1	CY047873	H1N1	06/20/2009	Argentina	A/Argentina/9004/2009
PB1	CY049058	H1N1	06/20/2009	Singapore	A/Singapore/ON305/2009(H1N1)
PB1	CY064586	H1N1	06/20/2009	USA	A/Boston/126/2009(H1N1)
PB1	CY058538	H1N1	06/21/2009	Nicaragua	A/Managua/4514.03/2009(H1N1)
PB1	CY064610	H1N1	06/21/2009	USA	A/Boston/130/2009(H1N1)
PB1	CY051541	H1N1	06/21/2009	USA	A/New York/4402/2009(H1N1)
PB1	CY047841	H1N1	06/21/2009	Argentina	A/Argentina/8574/2009
PB1	CY051189	H1N1	06/21/2009	USA	A/Wisconsin/629-D02220/2009(H1N
PB1	CY047857	H1N1	06/21/2009	Argentina	A/Argentina/8989/2009
PB1	CY052336	H1N1	06/21/2009	USA	A/Texas/42211898/2009(H1N1)
PB1	CY054777	H1N1	06/22/2009	USA	A/California/VRDL21/2009(H1N1)
PB1	KC781715	H1N1	06/22/2009	USA	A/New York/98/2009
PB1	CY064602	H1N1	06/22/2009	USA	A/Boston/129/2009(H1N1)
PB1	KT180483	H1N1	06/22/2009	Singapore	A/Singapore/DMS516/2009

PB1	CY111617	H1N1	06/22/2009	Hong Kong	A/Hong Kong/H090-661-V10/2009
PB1	CY055477	H1N1	06/22/2009	USA	A/California/VRDL18/2009(H1N1)
PB1	KT180336	H1N1	06/22/2009	Singapore	A/Singapore/DMS4/2009
PB1	CY120499	H1N1	06/22/2009	Hong Kong	A/Hong Kong/H090-661-V10/2009
PB1	CY123344	H1N1	06/22/2009	Singapore	A/Singapore/GP687/2009
PB1	CY123927	H1N1	06/22/2009	Singapore	A/Singapore/ON355/2009(H1N1)
PB1	CY047865	H1N1	06/22/2009	Argentina	A/Argentina/8994/2009
PB1	CY064674	H1N1	06/22/2009	USA	A/Boston/140/2009(H1N1)
PB1	CY051205	H1N1	06/22/2009	USA	A/Wisconsin/629-D02024/2009(H1N
PB1	CY054825	H1N1	06/22/2009	USA	A/California/VRDL28/2009(H1N1)
PB1	KT180489	H1N1	06/22/2009	Singapore	A/Singapore/DMS562/2009
PB1	CY075249	H1N1	06/22/2009	Chile	A/Chile/2362/2009(H1N1)
PB1	CY064554	H1N1	06/22/2009	USA	A/Boston/122/2009(H1N1)
PB1	CY052264	H1N1	06/22/2009	USA	A/Texas/42221280/2009(H1N1)
PB1	GU136047	H1N1	06/22/2009	Japan	A/Niigata/700/2009(H1N1)
PB1	CY058146	H1N1	06/22/2009	Nicaragua	A/Managua/5665.01/2009(H1N1)
PB1	GQ365424	H1N1	06/23/2009	Japan	A/Fukushima/1/2009(H1N1)
PB1	CY051485	H1N1	06/23/2009	USA	A/Wisconsin/629-S0410/2009(H1N1)
PB1	KT180486	H1N1	06/23/2009	Singapore	A/Singapore/DMS547/2009
PB1	CY047969	H1N1	06/23/2009	Argentina	A/Argentina/9721/2009
PB1	CY047897	H1N1	06/23/2009	Argentina	A/Argentina/9384/2009
PB1	JX625409	H1N1	06/23/2009	United Ki	A/England/92880002/2009
PB1	CY065156	H1N1	06/23/2009	United Ki	A/England/541/2009
PB1	CY047889	H1N1	06/23/2009	Argentina	A/Argentina/9333/2009
PB1	GU136048	H1N1	06/23/2009	Japan	A/Niigata/717/2009(H1N1)
PB1	CY047881	H1N1	06/23/2009	Argentina	A/Argentina/9180/2009
PB1	CY055469	H1N1	06/23/2009	USA	A/California/VRDL14/2009(H1N1)
PB1	CY051557	H1N1	06/23/2009	USA	A/New York/4434/2009(H1N1)
PB1	CY064618	H1N1	06/24/2009	USA	A/Boston/131/2009(H1N1)
PB1	CY051477	H1N1	06/24/2009	USA	A/Wisconsin/629-S0435/2009(H1N1)
PB1	CY107324	H1N1	06/24/2009	United Ki	A/Scotland/Edinburgh_17715/2009
PB1	KT180337	H1N1	06/24/2009	Singapore	A/Singapore/DMS5/2009
PB1	CY047953	H1N1	06/24/2009	Argentina	A/Argentina/9705/2009
PB1	CY083621	H1N1	06/24/2009	Peru	A/Trujillo/WRAIR9309F/2009
PB1	GU136036	H1N1	06/24/2009	Japan	A/Iwate/3/2009(H1N1)
PB1	CY054785	H1N1	06/24/2009	USA	A/California/VRDL22/2009(H1N1)
PB1	CY047921	H1N1	06/24/2009	Argentina	A/Argentina/9579/2009
PB1	CY054833	H1N1	06/24/2009	USA	A/California/VRDL29/2009(H1N1)
PB1	CY087222	H1N1	06/24/2009	USA	A/Houston/210S/2009
PB1	KT180457	H1N1	06/24/2009	Singapore	A/Singapore/DMS223/2009
PB1	CY107817	H1N1	06/24/2009	United Ki	A/Scotland/StAndrews_17693/2009
PB1	KF612056	H1N1	06/24/2009	Bolivia	A/Santa Cruz/46631/2009
PB1	CY052152	H1N1	06/24/2009	USA	A/New York/4398/2009(H1N1)
PB1	CY057228	H1N1	06/25/2009	Nicaragua	A/Managua/611.01/2009(H1N1)
PB1	CY064682	H1N1	06/25/2009	USA	A/Boston/141/2009(H1N1)
PB1	CY058210	H1N1	06/25/2009	Nicaragua	A/Managua/791.03/2009(H1N1)
PB1	CY075257	H1N1	06/25/2009	Chile	A/Chile/2909/2009(H1N1)
PB1	CY050188	H1N1	06/25/2009	USA	A/New York/4438/2009(H1N1)
PB1	CY051533	H1N1	06/25/2009	USA	A/New York/4400/2009(H1N1)
PB1	CY083565	H1N1	06/25/2009	Peru	A/Lima/WRAIR8511F/2009
PB1	CY075265	H1N1	06/25/2009	Chile	A/Chile/2911/2009(H1N1)
PB1	CY083525	H1N1	06/25/2009	Peru	A/Lima/WRAIR1696P/2009
PB1	CY052312	H1N1	06/25/2009	USA	A/Texas/42254309/2009(H1N1)
PB1	KJ130194	H1N1	06/25/2009	New Zeala	A/New Zealand/2047/2009

PB1	CY052160	H1N1	06/25/2009	USA	A/New York/4401/2009(H1N1)
PB1	GU136035	H1N1	06/25/2009	Japan	A/Hiroshima/230/2009(H1N1)
PB1	CY044145	H1N1	06/25/2009	Colombia	A/Bogota/0466N/2009(H1N1)
PB1	CY064570	H1N1	06/26/2009	USA	A/Boston/124/2009(H1N1)
PB1	CY075297	H1N1	06/26/2009	Chile	A/Chile/3123/2009(H1N1)
PB1	CY075273	H1N1	06/26/2009	Chile	A/Chile/2994/2009(H1N1)
PB1	CY124002	H1N1	06/26/2009	Singapore	A/Singapore/ON506/2009
PB1	CY064901	H1N1	06/26/2009	USA	A/Boston/136/2009(H1N1)
PB1	CY058546	H1N1	06/26/2009	Nicaragua	A/Managua/462.01/2009(H1N1)
PB1	CY075281	H1N1	06/26/2009	Chile	A/Chile/3019/2009(H1N1)
PB1	CY054761	H1N1	06/26/2009	USA	A/California/VRDL19/2009(H1N1)
PB1	GU136041	H1N1	06/26/2009	Myanmar	A/Myanmar/60/2009(H1N1)
PB1	CY064642	H1N1	06/26/2009	USA	A/Boston/134/2009(H1N1)
PB1	JX625385	H1N1	06/26/2009	United Ki	A/England/506/2009
PB1	KC782291	H1N1	06/26/2009	USA	A/Texas/48/2009
PB1	CY052120	H1N1	06/26/2009	USA	A/Wisconsin/629-D01973/2009(H1N
PB1	CY053172	H1N1	06/26/2009	USA	A/Houston/240S/2009(H1N1)
PB1	CY075289	H1N1	06/26/2009	Chile	A/Chile/3056/2009
PB1	CY064522	H1N1	06/26/2009	USA	A/Boston/117/2009(H1N1)
PB1	CY051213	H1N1	06/27/2009	USA	A/Wisconsin/629-D01414/2009(H1N
PB1	CY054817	H1N1	06/27/2009	USA	A/California/VRDL27/2009(H1N1)
PB1	CY083453	H1N1	06/27/2009	Peru	A/Lima/WRAIR1687P/2009
PB1	CY054841	H1N1	06/28/2009	USA	A/California/VRDL30/2009(H1N1)
PB1	KT180338	H1N1	06/28/2009	Singapore	A/Singapore/DMS7/2009
PB1	CY052192	H1N1	06/28/2009	USA	A/Texas/42281289/2009(H1N1)
PB1	CY054793	H1N1	06/29/2009	USA	A/California/VRDL23/2009(H1N1)
PB1	CY058202	H1N1	06/29/2009	Nicaragua	A/Managua/1127.02/2009(H1N1)
PB1	CY064514	H1N1	06/29/2009	USA	A/Boston/116/2009(H1N1)
PB1	CY049993	H1N1	06/29/2009	Mexico	A/Mexico City/WR1100N/2009(H1N1)
PB1	CY052405	H1N1	06/29/2009	USA	A/Texas/42291877/2009(H1N1)
PB1	CY058186	H1N1	06/29/2009	Nicaragua	A/Managua/3189.01/2009(H1N1)
PB1	GU136050	H1N1	06/29/2009	Japan	A/Saitama/85/2009(H1N1)
PB1	CY063564	H1N1	06/29/2009	USA	A/Boston/152/2009(H1N1)
PB1	CY123360	H1N1	06/30/2009	Singapore	A/Singapore/GP875/2009
PB1	GU136033	H1N1	06/30/2009	Japan	A/Hiroshima/216/2009(H1N1)
PB1	CY064690	H1N1	06/30/2009	USA	A/Boston/142/2009(H1N1)
PB1	CY052208	H1N1	06/30/2009	USA	A/Texas/42303371/2009(H1N1)
PB1	CY075329	H1N1	06/30/2009	Chile	A/Chile/3295/2009(H1N1)
PB1	CY111625	H1N1	06/30/2009	Hong Kong	A/Hong Kong/H090-663-V10/2009
PB1	CY051653	H1N1	06/30/2009	USA	A/New York/4691/2009(H1N1)
PB1	CY051493	H1N1	06/30/2009	USA	A/Wisconsin/629-S0464/2009(H1N1)
PB1	CY069747	H1N1	06/30/2009	United Ki	A/England/527/2009
PB1	CY075321	H1N1	06/30/2009	Chile	A/Chile/3244/2009(H1N1)
PB1	CY120107	H1N1	06/30/2009	Hong Kong	A/Hong Kong/H090-663-V10/2009
PB1	CY069755	H1N1	06/30/2009	United Ki	A/England/633/2009
PB1	JX625393	H1N1	06/30/2009	United Ki	A/England/527/2009
PB1	CY058194	H1N1	06/30/2009	Nicaragua	A/Managua/5586.01/2009(H1N1)
PB1	CY075305	H1N1	06/30/2009	Chile	A/Chile/3220/2009(H1N1)
PB1	CY075313	H1N1	06/30/2009	Chile	A/Chile/3242/2009(H1N1)
PB1	CY051221	H1N1	06/30/2009	USA	A/Wisconsin/629-D02292/2009(H1N
PB1	CY054873	H1N1	06/30/2009	USA	A/California/VRDL34/2009(H1N1)
PB1	CY054801	H1N1	06/30/2009	USA	A/California/VRDL25/2009(H1N1)
PB1	CY047742	H1N1	07/2009	Taiwan	A/Taiwan/526/2009(H1N1)
PB1	CY051581	H1N1	07/01/2009	USA	A/New York/4500/2009(H1N1)

PB1	JX625417	H1N1	07/01/2009	United Ki	A/England/667/2009
PB1	KF612055	H1N1	07/01/2009	Bolivia	A/Santa Cruz/12541/2009
PB1	KP637524	H1N1	07/01/2009	Thailand	A/Thailand/SN08270/2009
PB1	CY051573	H1N1	07/01/2009	USA	A/New York/4476/2009(H1N1)
PB1	CY063548	H1N1	07/01/2009	USA	A/Boston/149/2009(H1N1)
PB1	CY075345	H1N1	07/01/2009	Chile	A/Chile/3361/2009(H1N1)
PB1	CY083589	H1N1	07/01/2009	Peru	A/Lima/WRAIR8893F/2009(H1N1)
PB1	CY065172	H1N1	07/01/2009	United Ki	A/England/634/2009
PB1	GQ465744	H1N1	07/01/2009	Canada	A/Canada-SK/RV2486/2009(H1N1)
PB1	CY054913	H1N1	07/01/2009	USA	A/California/VRDL40/2009(H1N1)
PB1	CY054937	H1N1	07/01/2009	USA	A/California/VRDL45/2009(H1N1)
PB1	CY054857	H1N1	07/01/2009	USA	A/California/VRDL32/2009(H1N1)
PB1	JX625401	H1N1	07/01/2009	United Ki	A/England/685/2009
PB1	JX625425	H1N1	07/01/2009	United Ki	A/England/92920018/2009
PB1	CY051837	H1N1	07/01/2009	USA	A/Texas/43011033/2009(H1N1)
PB1	GU136032	H1N1	07/01/2009	Japan	A/Hiroshima/207/2009(H1N1)
PB1	CY063524	H1N1	07/01/2009	USA	A/Boston/146/2009(H1N1)
PB1	CY075337	H1N1	07/01/2009	Chile	A/Chile/3349/2009(H1N1)
PB1	CY075353	H1N1	07/01/2009	Chile	A/Chile/3369/2009(H1N1)
PB1	CY075361	H1N1	07/01/2009	Chile	A/Chile/3375/2009(H1N1)
PB1	CY054953	H1N1	07/01/2009	USA	A/California/VRDL50/2009(H1N1)
PB1	CY111641	H1N1	07/02/2009	Hong Kong	A/Hong Kong/H090-667-V10/2009
PB1	CY054897	H1N1	07/02/2009	USA	A/California/VRDL38/2009(H1N1)
PB1	CY120115	H1N1	07/02/2009	Hong Kong	A/Hong Kong/H090-665-V10/2009
PB1	CY120123	H1N1	07/02/2009	Hong Kong	A/Hong Kong/H090-667-V10/2009
PB1	CY124105	H1N1	07/02/2009	Singapore	A/Singapore/ON809/2009
PB1	CY072764	H1N1	07/02/2009	Nicaragua	A/Managua/435.01/2009(H1N1)
PB1	CY111649	H1N1	07/02/2009	Hong Kong	A/Hong Kong/H090-667-V23/2009
PB1	CY072780	H1N1	07/02/2009	Nicaragua	A/Managua/5235.03/2009(H1N1)
PB1	CY111633	H1N1	07/02/2009	Hong Kong	A/Hong Kong/H090-665-V10/2009
PB1	CY124121	H1N1	07/02/2009	Singapore	A/Singapore/ON812/2009
PB1	CY075377	H1N1	07/02/2009	Chile	A/Chile/3553/2009(H1N1)
PB1	CY075369	H1N1	07/02/2009	Chile	A/Chile/3467/2009(H1N1)
PB1	CY054865	H1N1	07/02/2009	USA	A/California/VRDL33/2009(H1N1)
PB1	CY075385	H1N1	07/03/2009	Chile	A/Chile/3586/2009(H1N1)
PB1	CY083613	H1N1	07/03/2009	Peru	A/Lima/WRAIR9202F/2009(H1N1)
PB1	CY083461	H1N1	07/03/2009	Peru	A/Lima/WRAIR1688P/2009
PB1	KT180339	H1N1	07/05/2009	Singapore	A/Singapore/DMS10/2009
PB1	JX625449	H1N1	07/05/2009	United Ki	A/England/671/2009
PB1	CY054881	H1N1	07/05/2009	USA	A/California/VRDL35/2009(H1N1)
PB1	CY065164	H1N1	07/06/2009	United Ki	A/England/92860032/2009
PB1	CY054929	H1N1	07/06/2009	USA	A/California/VRDL43/2009(H1N1)
PB1	CY122609	H1N1	07/06/2009	Singapore	A/Singapore/GP1099/2009
PB1	CY063556	H1N1	07/06/2009	USA	A/Boston/151/2009(H1N1)
PB1	CY072572	H1N1	07/06/2009	Nicaragua	A/Managua/4197.01/2009(H1N1)
PB1	CY075401	H1N1	07/06/2009	Chile	A/Chile/3766/2009(H1N1)
PB1	CY075409	H1N1	07/06/2009	Chile	A/Chile/3819/2009(H1N1)
PB1	CY069256	H1N1	07/06/2009	Nicaragua	A/Managua/4943.09/2009(H1N1)
PB1	CY122601	H1N1	07/06/2009	Singapore	A/Singapore/GP1097/2009
PB1	CY122593	H1N1	07/06/2009	Singapore	A/Singapore/GP1094/2009
PB1	KC781699	H1N1	07/06/2009	USA	A/Georgia/08/2009
PB1	CY075393	H1N1	07/06/2009	Chile	A/Chile/3760/2009(H1N1)
PB1	KP222582	H1N1	07/06/2009	Singapore	A/Singapore/376Y/2009
PB1	GU136049	H1N1	07/07/2009	Japan	A/Niigata/749/2009(H1N1)

PB1	CY053164	H1N1	07/07/2009	USA	A/Houston/23H/2009(H1N1)
PB1	CY063540	H1N1	07/07/2009	USA	A/Boston/148/2009(H1N1)
PB1	CY073891	H1N1	07/07/2009	Nicaragua	A/Managua/2729.02/2009(H1N1)
PB1	CY075425	H1N1	07/07/2009	Chile	A/Chile/3935/2009(H1N1)
PB1	CY088679	H1N1	07/07/2009	India	A/Mum/NIV398/2009(H1N1)
PB1	CY111657	H1N1	07/07/2009	Hong Kong	A/Hong Kong/H090-678-V10/2009
PB1	GU136029	H1N1	07/07/2009	Japan	A/Gifu-C/67/2009(H1N1)
PB1	CY045952	H1N1	07/07/2009	Canada	A/Toronto/T0106/2009(H1N1)
PB1	CY122617	H1N1	07/07/2009	Singapore	A/Singapore/GP1102/2009
PB1	CY075417	H1N1	07/07/2009	Chile	A/Chile/3905/2009(H1N1)
PB1	CY120131	H1N1	07/07/2009	Hong Kong	A/Hong Kong/H090-678-V10/2009
PB1	CY111665	H1N1	07/08/2009	Hong Kong	A/Hong Kong/H090-681-V10/2009
PB1	CY064698	H1N1	07/08/2009	USA	A/Boston/143/2009(H1N1)
PB1	GU136042	H1N1	07/08/2009	Myanmar	A/Myanmar/JP90/2009(H1N1)
PB1	CY051613	H1N1	07/08/2009	USA	A/New York/4567/2009(H1N1)
PB1	KT180347	H1N1	07/08/2009	Singapore	A/Singapore/DMS11/2009
PB1	CY051605	H1N1	07/08/2009	USA	A/New York/4566/2009(H1N1)
PB1	CY051597	H1N1	07/08/2009	USA	A/New York/4565/2009(H1N1)
PB1	CY120139	H1N1	07/08/2009	Hong Kong	A/Hong Kong/H090-681-V10/2009
PB1	CY122701	H1N1	07/08/2009	Singapore	A/Singapore/GP1146/2009
PB1	CY050196	H1N1	07/08/2009	USA	A/New York/4576/2009(H1N1)
PB1	KP637796	H1N1	07/09/2009	Thailand	A/Thailand/SS08193/2009
PB1	CY120171	H1N1	07/09/2009	Hong Kong	A/Hong Kong/H090-684-V10/2009
PB1	CY054905	H1N1	07/09/2009	USA	A/California/VRDL39/2009(H1N1)
PB1	CY122777	H1N1	07/09/2009	Singapore	A/Singapore/GP1182/2009
PB1	CY095839	H1N1	07/09/2009	China	A/Zhejiang/7/2009
PB1	CY095719	H1N1	07/09/2009	China	A/Zhejiang/X1/2009
PB1	CY095746	H1N1	07/09/2009	China	A/Zhejiang/X2/2009
PB1	CY095977	H1N1	07/09/2009	China	A/Zhejiang/9/2009
PB1	CY095815	H1N1	07/09/2009	China	A/Zhejiang/5/2009
PB1	CY095761	H1N1	07/09/2009	China	A/Zhejiang/3/2009
PB1	GQ392018	H1N1	07/09/2009	Russia	A/Moscow/IIV04/2009(H1N1)
PB1	CY111705	H1N1	07/09/2009	Hong Kong	A/Hong Kong/H090-684-V10/2009
PB1	CY075441	H1N1	07/09/2009	Chile	A/Chile/4181/2009(H1N1)
PB1	CY075433	H1N1	07/09/2009	Chile	A/Chile/4064/2009(H1N1)
PB1	CY066189	H1N1	07/09/2009	USA	A/Boston/144/2009(H1N1)
PB1	CY063532	H1N1	07/09/2009	USA	A/Boston/147/2009(H1N1)
PB1	CY095775	H1N1	07/09/2009	China	A/Zhejiang/4/2009
PB1	KP637716	H1N1	07/09/2009	Thailand	A/Thailand/SR08021/2009
PB1	CY063516	H1N1	07/09/2009	USA	A/Boston/145/2009(H1N1)
PB1	CY051589	H1N1	07/09/2009	USA	A/New York/4556/2009(H1N1)
PB1	CY051621	H1N1	07/09/2009	USA	A/New York/4568/2009(H1N1)
PB1	CY120187	H1N1	07/10/2009	Hong Kong	A/Hong Kong/H090-693-V10/2009
PB1	CY065180	H1N1	07/10/2009	United Ki	A/England/675/2009
PB1	CY075449	H1N1	07/10/2009	Chile	A/Chile/4182/2009(H1N1)
PB1	CY111673	H1N1	07/10/2009	Hong Kong	A/Hong Kong/H090-681-V22/2009
PB1	CY054945	H1N1	07/10/2009	USA	A/California/VRDL48/2009(H1N1)
PB1	CY111721	H1N1	07/10/2009	Hong Kong	A/Hong Kong/H090-693-V10/2009
PB1	CY106574	H1N1	07/11/2009	Mexico	A/Merida/2189-CIR/2009
PB1	CY054961	H1N1	07/11/2009	USA	A/California/VRDL51/2009(H1N1)
PB1	KT180385	H1N1	07/11/2009	Singapore	A/Singapore/DMS12/2009
PB1	CY054921	H1N1	07/11/2009	USA	A/California/VRDL41/2009(H1N1)
PB1	GU136039	H1N1	07/11/2009	Japan	A/Mie/41/2009(H1N1)
PB1	CY111689	H1N1	07/11/2009	Hong Kong	A/Hong Kong/H090-683-V10/2009

PB1	JX625441	H1N1	07/11/2009	United Ki	A/England/687/2009
PB1	CY120155	H1N1	07/11/2009	Hong Kong	A/Hong Kong/H090-683-V10/2009
PB1	CY072964	H1N1	07/12/2009	Nicaragua	A/Managua/585.01/2009(H1N1)
PB1	CY052429	H1N1	07/12/2009	USA	A/Texas/43122467/2009(H1N1)
PB1	CY072884	H1N1	07/12/2009	Nicaragua	A/Managua/4606.06/2009(H1N1)
PB1	CY120082	H1N1	07/13/2009	Hong Kong	A/Hong Kong/H090-698-V10/2009
PB1	CY120147	H1N1	07/13/2009	Hong Kong	A/Hong Kong/H090-681-V32/2009
PB1	CY120211	H1N1	07/13/2009	Hong Kong	A/Hong Kong/H090-704-V10/2009
PB1	CY120203	H1N1	07/13/2009	Hong Kong	A/Hong Kong/H090-701-V10/2009
PB1	CY111681	H1N1	07/13/2009	Hong Kong	A/Hong Kong/H090-681-V32/2009
PB1	CY111737	H1N1	07/13/2009	Hong Kong	A/Hong Kong/H090-698-V10/2009
PB1	CY111753	H1N1	07/13/2009	Hong Kong	A/Hong Kong/H090-701-V10/2009
PB1	JX625457	H1N1	07/13/2009	United Ki	A/England/688/2009
PB1	KP637652	H1N1	07/13/2009	Thailand	A/Thailand/SP08279/2009
PB1	CY075457	H1N1	07/13/2009	Chile	A/Chile/4257/2009(H1N1)
PB1	CY123380	H1N1	07/13/2009	Singapore	A/Singapore/ON1063/2009
PB1	CY123372	H1N1	07/13/2009	Singapore	A/Singapore/ON1058/2009
PB1	CY083102	H1N1	07/13/2009	Peru	A/Piura/WRAIR0603F/2009(H1N1)
PB1	CY083509	H1N1	07/13/2009	Peru	A/Piura/WRAIR1694P/2009(H1N1)
PB1	CY052413	H1N1	07/13/2009	USA	A/Texas/43132503/2009(H1N1)
PB1	GU136054	H1N1	07/13/2009	Japan	A/Wakayama/57/2009(H1N1)
PB1	CY111729	H1N1	07/13/2009	Hong Kong	A/Hong Kong/H090-693-V20/2009
PB1	CY111761	H1N1	07/13/2009	Hong Kong	A/Hong Kong/H090-704-V10/2009
PB1	CY073703	H1N1	07/14/2009	Nicaragua	A/Managua/3490.03/2009(H1N1)
PB1	CY051629	H1N1	07/14/2009	USA	A/New York/4607/2009(H1N1)
PB1	GU136043	H1N1	07/14/2009	Myanmar	A/Myanmar/JP101/2009(H1N1)
PB1	CY054969	H1N1	07/14/2009	USA	A/California/VRDL52/2009(H1N1)
PB1	CY111713	H1N1	07/14/2009	Hong Kong	A/Hong Kong/H090-684-V23/2009
PB1	CY111769	H1N1	07/14/2009	Hong Kong	A/Hong Kong/H090-708-V10/2009
PB1	CY052256	H1N1	07/14/2009	USA	A/Texas/43143450/2009(H1N1)
PB1	CY123428	H1N1	07/14/2009	Singapore	A/Singapore/ON1102/2009
PB1	CY123412	H1N1	07/14/2009	Singapore	A/Singapore/ON1091/2009
PB1	CY122785	H1N1	07/14/2009	Singapore	A/Singapore/GP1358/2009
PB1	CY111697	H1N1	07/14/2009	Hong Kong	A/Hong Kong/H090-683-V20/2009
PB1	KT180429	H1N1	07/14/2009	Singapore	A/Singapore/DMS13/2009
PB1	CY054985	H1N1	07/14/2009	USA	A/California/VRDL55/2009(H1N1)
PB1	CY051637	H1N1	07/14/2009	USA	A/New York/4620/2009(H1N1)
PB1	CY120179	H1N1	07/14/2009	Hong Kong	A/Hong Kong/H090-684-V23/2009
PB1	CY120219	H1N1	07/14/2009	Hong Kong	A/Hong Kong/H090-708-V10/2009
PB1	CY120163	H1N1	07/14/2009	Hong Kong	A/Hong Kong/H090-683-V20/2009
PB1	CY123404	H1N1	07/14/2009	Singapore	A/Singapore/ON1079/2009
PB1	CY120235	H1N1	07/15/2009	Hong Kong	A/Hong Kong/H090-712-V14/2009
PB1	CY111785	H1N1	07/15/2009	Hong Kong	A/Hong Kong/H090-712-V1_4/2009
PB1	CY063572	H1N1	07/15/2009	USA	A/Boston/153/2009(H1N1)
PB1	JX625433	H1N1	07/15/2009	United Ki	A/England/92960004/2009
PB1	CY120227	H1N1	07/15/2009	Hong Kong	A/Hong Kong/H090-712-V10/2009
PB1	CY111777	H1N1	07/15/2009	Hong Kong	A/Hong Kong/H090-712-V10/2009
PB1	CY055532	H1N1	07/16/2009	Australia	A/Australia/1/2009(H1N1)
PB1	CY111809	H1N1	07/16/2009	Hong Kong	A/Hong Kong/H090-715-V10/2009
PB1	CY072844	H1N1	07/16/2009	Nicaragua	A/Managua/265.01/2009(H1N1)
PB1	CY120195	H1N1	07/16/2009	Hong Kong	A/Hong Kong/H090-698-V20/2009
PB1	CY051645	H1N1	07/16/2009	USA	A/New York/4677/2009(H1N1)
PB1	CY072900	H1N1	07/16/2009	Nicaragua	A/Managua/3214.04/2009(H1N1)
PB1	CY111745	H1N1	07/16/2009	Hong Kong	A/Hong Kong/H090-698-V20/2009

PB1	CY083357	H1N1	07/16/2009	USA	A/San Diego/WRAIR1658P/2009
PB1	CY120267	H1N1	07/17/2009	Hong Kong	A/Hong Kong/H090-722-V10/2009
PB1	CY111817	H1N1	07/17/2009	Hong Kong	A/Hong Kong/H090-721-V10/2009
PB1	KT180438	H1N1	07/17/2009	Singapore	A/Singapore/DMS14/2009
PB1	CY111825	H1N1	07/17/2009	Hong Kong	A/Hong Kong/H090-722-V10/2009(H
PB1	CY055065	H1N1	07/17/2009	Australia	A/Australia/3/2009(H1N1)
PB1	CY055073	H1N1	07/17/2009	Australia	A/Australia/4/2009(H1N1)
PB1	CY120259	H1N1	07/17/2009	Hong Kong	A/Hong Kong/H090-721-V10/2009
PB1	JX625465	H1N1	07/17/2009	United Ki	A/England/92980479/2009
PB1	CY083278	H1N1	07/17/2009	USA	A/South Carolina/WRAIR1645P/2009
PB1	CY097882	H1N1	07/17/2009	USA	A/South Carolina/WRAIR1645P/2009
PB1	CY075465	H1N1	07/17/2009	Chile	A/Chile/4406/2009(H1N1)
PB1	CY083349	H1N1	07/18/2009	USA	A/San Diego/WRAIR1657P/2009
PB1	CY055081	H1N1	07/18/2009	Australia	A/Australia/6/2009(H1N1)
PB1	CY120251	H1N1	07/18/2009	Hong Kong	A/Hong Kong/H090-712-V24/2009
PB1	CY055540	H1N1	07/18/2009	Australia	A/Australia/7/2009(H1N1)
PB1	CY120243	H1N1	07/18/2009	Hong Kong	A/Hong Kong/H090-712-V20/2009
PB1	CY111793	H1N1	07/18/2009	Hong Kong	A/Hong Kong/H090-712-V20/2009
PB1	CY111801	H1N1	07/18/2009	Hong Kong	A/Hong Kong/H090-712-V24/2009
PB1	GU136053	H1N1	07/18/2009	Japan	A/Tokushima/2/2009(H1N1)
PB1	GU433031	H1N1	07/19/2009	Russia	A/Tomsk/03/2009(H1N1)
PB1	CY083126	H1N1	07/19/2009	Peru	A/Lima/WRAIR0672F/2009
PB1	CY055571	H1N1	07/20/2009	Australia	A/Australia/12/2009(H1N1)
PB1	CY055579	H1N1	07/20/2009	Australia	A/Australia/13/2009(H1N1)
PB1	CY055587	H1N1	07/20/2009	Australia	A/Australia/14/2009(H1N1)
PB1	CY055556	H1N1	07/20/2009	Australia	A/Australia/10/2009(H1N1)
PB1	CY052381	H1N1	07/20/2009	USA	A/Texas/43200999/2009(H1N1)
PB1	KP637396	H1N1	07/20/2009	Thailand	A/Thailand/MR08208/2009
PB1	CY075473	H1N1	07/20/2009	Chile	A/Chile/4438/2009(H1N1)
PB1	CY071005	H1N1	07/20/2009	Guam	A/Guam/NHRC0030/2009(H1N1)
PB1	CY055563	H1N1	07/20/2009	Australia	A/Australia/11/2009(H1N1)
PB1	CY055595	H1N1	07/20/2009	Australia	A/Australia/15/2009(H1N1)
PB1	CY055548	H1N1	07/20/2009	Australia	A/Australia/9/2009(H1N1)
PB1	GU433039	H1N1	07/21/2009	Russia	A/Novosibirsk/02/2009(H1N1)
PB1	CY072860	H1N1	07/21/2009	Nicaragua	A/Managua/3438.02/2009(H1N1)
PB1	KC620384	H1N1	07/21/2009	Germany	A/Giessen/6/2009
PB1	CY054977	H1N1	07/21/2009	USA	A/California/VRDL53/2009(H1N1)
PB1	CY069264	H1N1	07/21/2009	Nicaragua	A/Managua/5364.01/2009(H1N1)
PB1	CY068968	H1N1	07/21/2009	Guam	A/Guam/NHRC0010/2009(H1N1)
PB1	CY069040	H1N1	07/21/2009	Guam	A/Guam/NHRC0019/2009(H1N1)
PB1	CY069056	H1N1	07/21/2009	Guam	A/Guam/NHRC0021/2009(H1N1)
PB1	CY083206	H1N1	07/21/2009	USA	A/New Jersey/WRAIR1504P/2009(H1N
PB1	CY092191	H1N1	07/21/2009	Australia	A/Australia/17/2009
PB1	CY111833	H1N1	07/21/2009	Hong Kong	A/Hong Kong/H090-722-V20/2009(H
PB1	CY068976	H1N1	07/21/2009	Guam	A/Guam/NHRC0011/2009(H1N1)
PB1	CY055603	H1N1	07/21/2009	Australia	A/Australia/16/2009(H1N1)
PB1	CY055293	H1N1	07/21/2009	Malaysia	A/Malaysia/5259/2009(H1N1)
PB1	CY055619	H1N1	07/21/2009	Australia	A/Australia/21/2009(H1N1)
PB1	CY055285	H1N1	07/21/2009	Malaysia	A/Malaysia/5259/2009(H1N1)
PB1	CY054993	H1N1	07/21/2009	USA	A/California/VRDL58/2009(H1N1)
PB1	CY055611	H1N1	07/21/2009	Australia	A/Australia/20/2009(H1N1)
PB1	CY055635	H1N1	07/21/2009	Australia	A/Australia/24/2009(H1N1)
PB1	GU136040	H1N1	07/21/2009	Japan	A/Mie/52/2009(H1N1)
PB1	HM569673	H1N1	07/21/2009	Argentina	A/Argentina/08AR/2009(H1N1)

PB1	CY069096	H1N1	07/21/2009	Guam	A/Guam/NHRC0026/2009(H1N1)
PB1	CY055627	H1N1	07/22/2009	Australia	A/Australia/23/2009(H1N1)
PB1	KJ690422	H1N1	07/22/2009	Uganda	A/Uganda/MUWRP-059/2009
PB1	KJ549776	H1N1	07/22/2009	Germany	A/Jena/5258/2009
PB1	KP222583	H1N1	07/22/2009	Singapore	A/Singapore/384P/2009
PB1	KX013003	H1N1	07/22/2009	Germany	A/Jena/MSV-VI5258/2009
PB1	CY069032	H1N1	07/22/2009	Guam	A/Guam/NHRC0018/2009(H1N1)
PB1	CY068944	H1N1	07/22/2009	Guam	A/Guam/NHRC0008/2009(H1N1)
PB1	CY073647	H1N1	07/22/2009	Nicaragua	A/Managua/1973.02/2009(H1N1)
PB1	CY073655	H1N1	07/22/2009	Nicaragua	A/Managua/4590.03/2009(H1N1)
PB1	KC222634	H1N1	07/22/2009	Germany	A/Jena/VI5258/2009(H1N1)
PB1	GU136044	H1N1	07/22/2009	Myanmar	A/Myanmar/JP131/2009(H1N1)
PB1	GQ496138	H1N1	07/22/2009	Russia	A/Vladivostok/IIV17/2009(H1N1)
PB1	CY073623	H1N1	07/22/2009	Nicaragua	A/Managua/4747.01/2009(H1N1)
PB1	CY055659	H1N1	07/22/2009	Australia	A/Australia/27/2009(H1N1)
PB1	CY055651	H1N1	07/22/2009	Australia	A/Australia/26/2009(H1N1)
PB1	GU136045	H1N1	07/22/2009	Myanmar	A/Myanmar/JP133/2009(H1N1)
PB1	CY055643	H1N1	07/22/2009	Australia	A/Australia/25/2009(H1N1)
PB1	CY065966	H1N1	07/23/2009	Japan	A/Japan/NHRC0002/2009(H1N1)
PB1	JX625496	H1N1	07/23/2009	United Ki	A/England/93760756/2009
PB1	KT180447	H1N1	07/23/2009	Singapore	A/Singapore/DMS16/2009
PB1	CY119360	H1N1	07/23/2009	Malaysia	A/Malaysia/2050837/2009
PB1	CY055667	H1N1	07/23/2009	Australia	A/Australia/28/2009(H1N1)
PB1	CY065958	H1N1	07/23/2009	Japan	A/Japan/NHRC0001/2009(H1N1)
PB1	CY073599	H1N1	07/23/2009	Nicaragua	A/Managua/5295.02/2009(H1N1)
PB1	CY065942	H1N1	07/23/2009	Guam	A/Guam/NHRC0002/2009(H1N1)
PB1	CY068984	H1N1	07/23/2009	Guam	A/Guam/NHRC0012/2009(H1N1)
PB1	CY055682	H1N1	07/23/2009	Australia	A/Australia/31/2009(H1N1)
PB1	CY055674	H1N1	07/23/2009	Australia	A/Australia/29/2009(H1N1)
PB1	CY068920	H1N1	07/23/2009	Japan	A/Japan/NHRC0003/2009(H1N1)
PB1	CY055706	H1N1	07/24/2009	Australia	A/Australia/36/2009(H1N1)
PB1	CY055722	H1N1	07/24/2009	Australia	A/Australia/39/2009(H1N1)
PB1	CY051661	H1N1	07/24/2009	USA	A/New York/4728/2009(H1N1)
PB1	CY120275	H1N1	07/24/2009	Hong Kong	A/Hong Kong/H090-738-V10/2009
PB1	CY128089	H1N1	07/24/2009	Viet Nam	A/Viet Nam/811/2009
PB1	CY128081	H1N1	07/24/2009	Viet Nam	A/Viet Nam/817/2009
PB1	CY069288	H1N1	07/24/2009	Nicaragua	A/Managua/3467.01/2009(H1N1)
PB1	CY072580	H1N1	07/24/2009	Nicaragua	A/Managua/3231.02/2009(H1N1)
PB1	CY111841	H1N1	07/24/2009	Hong Kong	A/Hong Kong/H090-738-V10/2009
PB1	GQ496153	H1N1	07/24/2009	Russia	A/Vladivostok/IIV18/2009(H1N1)
PB1	CY120283	H1N1	07/24/2009	Hong Kong	A/Hong Kong/H090-742-V10/2009
PB1	KP637660	H1N1	07/24/2009	Thailand	A/Thailand/SP08294/2009
PB1	CY111849	H1N1	07/24/2009	Hong Kong	A/Hong Kong/H090-742-V10/2009(H
PB1	CY051829	H1N1	07/24/2009	USA	A/Texas/43242018/2009(H1N1)
PB1	CY069296	H1N1	07/24/2009	Nicaragua	A/Managua/4951.02/2009(H1N1)
PB1	CY055698	H1N1	07/24/2009	Australia	A/Australia/35/2009(H1N1)
PB1	KP637452	H1N1	07/24/2009	Thailand	A/Thailand/PY08016/2009
PB1	CY055730	H1N1	07/24/2009	Australia	A/Australia/40/2009(H1N1)
PB1	CY055714	H1N1	07/24/2009	Australia	A/Australia/37/2009(H1N1)
PB1	KC782200	H1N1	07/25/2009	USA	A/Utah/20/2009
PB1	CY069272	H1N1	07/25/2009	Nicaragua	A/Managua/1264.03/2009(H1N1)
PB1	CY051669	H1N1	07/25/2009	USA	A/New York/4735/2009(H1N1)
PB1	CY088693	H1N1	07/25/2009	India	A/Pune/NIV759/2009
PB1	CY055738	H1N1	07/25/2009	Australia	A/Australia/42/2009(H1N1)

PB1	CY128097	H1N1	07/25/2009	Viet Nam	A/Viet Nam/818/2009
PB1	CY055754	H1N1	07/26/2009	Australia	A/Australia/44/2009(H1N1)
PB1	KT180451	H1N1	07/26/2009	Singapore	A/Singapore/DMS17/2009
PB1	CY055746	H1N1	07/26/2009	Australia	A/Australia/43/2009(H1N1)
PB1	CY128113	H1N1	07/26/2009	Viet Nam	A/Viet Nam/823/2009
PB1	KP222585	H1N1	07/27/2009	Singapore	A/Singapore/386T/2009
PB1	CY055762	H1N1	07/27/2009	Australia	A/Australia/45/2009(H1N1)
PB1	CY128129	H1N1	07/27/2009	Viet Nam	A/Viet Nam/841/2009
PB1	CY065934	H1N1	07/27/2009	Guam	A/Guam/NHRC0001/2009(H1N1)
PB1	CY111865	H1N1	07/27/2009	Hong Kong	A/Hong Kong/H090-745-V10/2009(H
PB1	JQ173146	H1N1	07/27/2009	Finland	A/Helsinki/P14/2009
PB1	CY073663	H1N1	07/27/2009	Nicaragua	A/Managua/3225.03/2009(H1N1)
PB1	CY055001	H1N1	07/27/2009	USA	A/California/VRDL61/2009(H1N1)
PB1	CY055770	H1N1	07/27/2009	Australia	A/Australia/47/2009(H1N1)
PB1	CY055778	H1N1	07/27/2009	Australia	A/Australia/48/2009(H1N1)
PB1	CY073535	H1N1	07/27/2009	Nicaragua	A/Managua/466.01/2009(H1N1)
PB1	CY072628	H1N1	07/27/2009	Nicaragua	A/Managua/5339.04/2009(H1N1)
PB1	CY128105	H1N1	07/27/2009	Viet Nam	A/Viet Nam/835/2009
PB1	JN187212	H1N1	07/27/2009	Taiwan	A/Taiwan/2826/2009
PB1	CY120090	H1N1	07/27/2009	Hong Kong	A/Hong Kong/H090-745-V10/2009
PB1	CY052373	H1N1	07/27/2009	USA	A/Texas/43272683/2009(H1N1)
PB1	KP222584	H1N1	07/27/2009	Singapore	A/Singapore/385K/2009
PB1	CY072732	H1N1	07/27/2009	Nicaragua	A/Managua/4407.02/2009(H1N1)
PB1	CY055786	H1N1	07/28/2009	Australia	A/Australia/51/2009(H1N1)
PB1	CY119536	H1N1	07/28/2009	Malaysia	A/Malaysia/2072040/2009
PB1	CY111881	H1N1	07/28/2009	Hong Kong	A/Hong Kong/H090-746-V10/2009(H
PB1	CY111857	H1N1	07/28/2009	Hong Kong	A/Hong Kong/H090-742-V33/2009
PB1	CY073639	H1N1	07/28/2009	Nicaragua	A/Managua/4960.01/2009(H1N1)
PB1	CY083238	H1N1	07/28/2009	USA	A/Virginia/WRAIR1511P/2009
PB1	CY120299	H1N1	07/28/2009	Hong Kong	A/Hong Kong/H090-746-V10/2009
PB1	CY128121	H1N1	07/28/2009	Viet Nam	A/Viet Nam/850/2009
PB1	CY051677	H1N1	07/28/2009	USA	A/New York/4738/2009(H1N1)
PB1	KP222587	H1N1	07/28/2009	Singapore	A/Singapore/392N/2009
PB1	KP222586	H1N1	07/28/2009	Singapore	A/Singapore/390T/2009
PB1	CY120291	H1N1	07/28/2009	Hong Kong	A/Hong Kong/H090-742-V33/2009
PB1	CY120307	H1N1	07/29/2009	Hong Kong	A/Hong Kong/H090-751-V10/2009
PB1	CY122793	H1N1	07/29/2009	Singapore	A/Singapore/GP2215/2009
PB1	KP222588	H1N1	07/29/2009	Singapore	A/Singapore/394T/2009
PB1	CY083222	H1N1	07/29/2009	USA	A/California/WRAIR1507P/2009
PB1	CY111897	H1N1	07/29/2009	Hong Kong	A/Hong Kong/H090-751-V10/2009
PB1	CY073559	H1N1	07/29/2009	Nicaragua	A/Managua/2978.04/2009(H1N1)
PB1	KP222589	H1N1	07/29/2009	Singapore	A/Singapore/395T/2009
PB1	KT180452	H1N1	07/29/2009	Singapore	A/Singapore/DMS18/2009
PB1	CY128457	H1N1	07/29/2009	Viet Nam	A/Viet Nam/001-1880/2009
PB1	CY055794	H1N1	07/29/2009	Australia	A/Australia/53/2009(H1N1)
PB1	CY065950	H1N1	07/29/2009	Guam	A/Guam/NHRC0003/2009(H1N1)
PB1	CY052288	H1N1	07/29/2009	USA	A/Texas/43292238/2009(H1N1)
PB1	CY111889	H1N1	07/30/2009	Hong Kong	A/Hong Kong/H090-746-V20/2009
PB1	KM013742	H1N1	07/30/2009	Thailand	A/Bangkok/SIMI507/2009
PB1	CY120371	H1N1	07/30/2009	Hong Kong	A/Hong Kong/H090-759-V10/2009
PB1	CY055810	H1N1	07/30/2009	Australia	A/Australia/56/2009(H1N1)
PB1	CY122885	H1N1	07/30/2009	Singapore	A/Singapore/GP2317/2009
PB1	CY120355	H1N1	07/30/2009	Hong Kong	A/Hong Kong/H090-757-V10/2009
PB1	KP637324	H1N1	07/30/2009	Thailand	A/Thailand/KS08277/2009

PB1	KY925892	H1N1	07/30/2009	Brazil	A/Porto Alegre/LACENRS-1771/2009
PB1	CY072636	H1N1	07/30/2009	Nicaragua	A/Managua/1836.02/2009(H1N1)
PB1	CY120363	H1N1	07/30/2009	Hong Kong	A/Hong Kong/H090-758-V10/2009
PB1	KM013736	H1N1	07/30/2009	Thailand	A/Bangkok/SIMI501/2009
PB1	CY122877	H1N1	07/30/2009	Singapore	A/Singapore/GP2315/2009
PB1	CY051685	H1N1	07/30/2009	USA	A/New York/4747/2009(H1N1)
PB1	CY111961	H1N1	07/30/2009	Hong Kong	A/Hong Kong/H090-759-V10/2009
PB1	CY111953	H1N1	07/30/2009	Hong Kong	A/Hong Kong/H090-758-V10/2009
PB1	CY111945	H1N1	07/30/2009	Hong Kong	A/Hong Kong/H090-757-V10/2009
PB1	KY926170	H1N1	07/31/2009	Brazil	A/Porto Alegre/LACENRS-1786/2009
PB1	KY925014	H1N1	07/31/2009	Brazil	A/Estrela/LACENRS-313/2009
PB1	KP222594	H1N1	07/31/2009	Singapore	A/Singapore/416C/2009
PB1	CY111969	H1N1	07/31/2009	Hong Kong	A/Hong Kong/H090-761-V10/2009
PB1	CY111913	H1N1	07/31/2009	Hong Kong	A/Hong Kong/H090-751-V23/2009
PB1	CY120331	H1N1	07/31/2009	Hong Kong	A/Hong Kong/H090-751-V24/2009
PB1	CY119368	H1N1	07/31/2009	Malaysia	A/Malaysia/2068631/2009
PB1	CY120745	H1N1	07/31/2009	Brazil	A/Brazil/AVS07/2009
PB1	CY120387	H1N1	07/31/2009	Hong Kong	A/Hong Kong/H090-762-V10/2009
PB1	CY120315	H1N1	07/31/2009	Hong Kong	A/Hong Kong/H090-751-V20/2009
PB1	CY083214	H1N1	07/31/2009	USA	A/New Jersey/WRAIR1506P/2009(H1N
PB1	KP222592	H1N1	07/31/2009	Singapore	A/Singapore/410W/2009
PB1	KP222590	H1N1	07/31/2009	Singapore	A/Singapore/405S/2009
PB1	KP222591	H1N1	07/31/2009	Singapore	A/Singapore/406K/2009
PB1	KP222593	H1N1	07/31/2009	Singapore	A/Singapore/413Y/2009
PB1	CY120323	H1N1	07/31/2009	Hong Kong	A/Hong Kong/H090-751-V23/2009
PB1	CY068936	H1N1	07/31/2009	Guam	A/Guam/NHRC0005/2009(H1N1)
PB1	CY111905	H1N1	07/31/2009	Hong Kong	A/Hong Kong/H090-751-V20/2009(H
PB1	CY070989	H1N1	07/31/2009	Guam	A/Guam/NHRC0007/2009(H1N1)
PB1	CY111921	H1N1	07/31/2009	Hong Kong	A/Hong Kong/H090-751-V24/2009(H
PB1	KP222595	H1N1	07/31/2009	Singapore	A/Singapore/418W/2009
PB1	CY111985	H1N1	07/31/2009	Hong Kong	A/Hong Kong/H090-765-V10/2009
PB1	CY111873	H1N1	07/31/2009	Hong Kong	A/Hong Kong/H090-745-V24/2009
PB1	CY111977	H1N1	07/31/2009	Hong Kong	A/Hong Kong/H090-762-V10/2009
PB1	CY120379	H1N1	07/31/2009	Hong Kong	A/Hong Kong/H090-761-V10/2009
PB1	CY120395	H1N1	07/31/2009	Hong Kong	A/Hong Kong/H090-765-V10/2009
PB1	KY925645	H1N1	07/31/2009	Brazil	A/Igrejinha/LACENRS-1803/2009
PB1	KT180453	H1N1	08/01/2009	Singapore	A/Singapore/DMS19/2009
PB1	KC780430	H1N1	08/01/2009	USA	A/Texas/71/2009
PB1	CY055818	H1N1	08/01/2009	Australia	A/Australia/57/2009(H1N1)
PB1	CY055826	H1N1	08/01/2009	Australia	A/Australia/58/2009(H1N1)
PB1	CY119376	H1N1	08/02/2009	Malaysia	A/Malaysia/2076204/2009
PB1	CY118205	H1N1	08/02/2009	Malaysia	A/Malaysia/2076212/2009
PB1	CY111929	H1N1	08/02/2009	Hong Kong	A/Hong Kong/H090-751-V31/2009(H
PB1	CY055842	H1N1	08/02/2009	Australia	A/Australia/60/2009(H1N1)
PB1	CY120339	H1N1	08/02/2009	Hong Kong	A/Hong Kong/H090-751-V31/2009
PB1	CY111937	H1N1	08/02/2009	Hong Kong	A/Hong Kong/H090-751-V34/2009
PB1	CY120347	H1N1	08/02/2009	Hong Kong	A/Hong Kong/H090-751-V34/2009
PB1	CY055834	H1N1	08/02/2009	Australia	A/Australia/59/2009(H1N1)
PB1	CY055874	H1N1	08/03/2009	Australia	A/Australia/65/2009(H1N1)
PB1	CY072684	H1N1	08/03/2009	Nicaragua	A/Managua/209.01/2009(H1N1)
PB1	CY119384	H1N1	08/03/2009	Malaysia	A/Malaysia/2076039/2009
PB1	CY112001	H1N1	08/03/2009	Hong Kong	A/Hong Kong/H090-771-V10/2009
PB1	CY119392	H1N1	08/03/2009	Malaysia	A/Malaysia/2077462/2009
PB1	CY055858	H1N1	08/03/2009	Australia	A/Australia/63/2009(H1N1)

PB1	CY055850	H1N1	08/03/2009	Australia	A/Australia/61/2009(H1N1)
PB1	CY072564	H1N1	08/03/2009	Nicaragua	A/Managua/451.01/2009(H1N1)
PB1	KP222601	H1N1	08/03/2009	Singapore	A/Singapore/441T/2009
PB1	KP222597	H1N1	08/03/2009	Singapore	A/Singapore/420T/2009
PB1	KP222598	H1N1	08/03/2009	Singapore	A/Singapore/426N/2009
PB1	KP222600	H1N1	08/03/2009	Singapore	A/Singapore/440F/2009
PB1	CY055866	H1N1	08/03/2009	Australia	A/Australia/64/2009(H1N1)
PB1	JX625473	H1N1	08/03/2009	United Ki	A/England/646/2009
PB1	CY120411	H1N1	08/03/2009	Hong Kong	A/Hong Kong/H090-771-V10/2009
PB1	CY106982	H1N1	08/03/2009	Hong Kong	A/Hong Kong/H090-770-V10/2009
PB1	KP222599	H1N1	08/03/2009	Singapore	A/Singapore/437Y/2009
PB1	KP222603	H1N1	08/04/2009	Singapore	A/Singapore/444W/2009
PB1	KT180454	H1N1	08/04/2009	Singapore	A/Singapore/DMS20/2009
PB1	CY119528	H1N1	08/04/2009	Malaysia	A/Malaysia/2076756/2009
PB1	CY055882	H1N1	08/04/2009	Australia	A/Australia/66/2009(H1N1)
PB1	CY120427	H1N1	08/04/2009	Hong Kong	A/Hong Kong/H090-774-V10/2009
PB1	CY068952	H1N1	08/04/2009	Japan	A/Japan/NHRC0004/2009(H1N1)
PB1	CY070981	H1N1	08/04/2009	Guam	A/Guam/NHRC0006/2009(H1N1)
PB1	CY073615	H1N1	08/04/2009	Nicaragua	A/Managua/1256.04/2009(H1N1)
PB1	CY073607	H1N1	08/04/2009	Nicaragua	A/Managua/4451.02/2009(H1N1)
PB1	CY112025	H1N1	08/04/2009	Hong Kong	A/Hong Kong/H090-774-V10/2009
PB1	KY925290	H1N1	08/05/2009	Brazil	A/Teutonia/LACENRS-711/2009
PB1	CY120419	H1N1	08/05/2009	Hong Kong	A/Hong Kong/H090-772-V10/2009
PB1	CY112033	H1N1	08/05/2009	Hong Kong	A/Hong Kong/H090-779-V10/2009
PB1	CY112009	H1N1	08/05/2009	Hong Kong	A/Hong Kong/H090-771-V20/2009
PB1	CY073575	H1N1	08/05/2009	Nicaragua	A/Managua/5230.01/2009(H1N1)
PB1	CY071496	H1N1	08/05/2009	USA	A/California/WR1316P/2009(H1N1)
PB1	CY120435	H1N1	08/05/2009	Hong Kong	A/Hong Kong/H090-779-V10/2009
PB1	CY055890	H1N1	08/05/2009	Australia	A/Australia/69/2009(H1N1)
PB1	CY072604	H1N1	08/05/2009	Nicaragua	A/Managua/1077.04/2009(H1N1)
PB1	KY925788	H1N1	08/05/2009	Brazil	A/Nonoai/LACENRS-714/2009
PB1	KJ690406	H1N1	08/05/2009	Uganda	A/Uganda/MUWRP-066/2009
PB1	CY055041	H1N1	08/05/2009	USA	A/California/VRDL69/2009(H1N1)
PB1	KJ690501	H1N1	08/05/2009	Uganda	A/Uganda/MUWRP-067/2009
PB1	CY112041	H1N1	08/05/2009	Hong Kong	A/Hong Kong/H090-779-V12/2009
PB1	CY112017	H1N1	08/05/2009	Hong Kong	A/Hong Kong/H090-772-V10/2009
PB1	CY119400	H1N1	08/05/2009	Malaysia	A/Malaysia/2082543/2009
PB1	CY120443	H1N1	08/05/2009	Hong Kong	A/Hong Kong/H090-779-V12/2009
PB1	CY051693	H1N1	08/06/2009	USA	A/New York/4761/2009(H1N1)
PB1	CY120403	H1N1	08/06/2009	Hong Kong	A/Hong Kong/H090-770-V22/2009
PB1	JX625481	H1N1	08/06/2009	United Ki	A/England/93320016/2009
PB1	KP222604	H1N1	08/06/2009	Singapore	A/Singapore/448D/2009
PB1	JN596861	H1N1	08/06/2009	Russia	A/Orenburg/01/2009(H1N1)
PB1	CY111993	H1N1	08/06/2009	Hong Kong	A/Hong Kong/H090-770-V22/2009
PB1	CY068928	H1N1	08/06/2009	Guam	A/Guam/NHRC0004/2009(H1N1)
PB1	CY055898	H1N1	08/06/2009	Australia	A/Australia/70/2009(H1N1)
PB1	CY112073	H1N1	08/07/2009	Hong Kong	A/Hong Kong/H090-785-V10/2009
PB1	KT180455	H1N1	08/07/2009	Singapore	A/Singapore/DMS21/2009
PB1	CY119408	H1N1	08/07/2009	Malaysia	A/Malaysia/2086505/2009
PB1	CY071528	H1N1	08/07/2009	USA	A/California/WR1320P/2009(H1N1)
PB1	CY073527	H1N1	08/07/2009	Nicaragua	A/Managua/5399.04/2009(H1N1)
PB1	CY072620	H1N1	08/07/2009	Nicaragua	A/Managua/2604.02/2009(H1N1)
PB1	CY120467	H1N1	08/07/2009	Hong Kong	A/Hong Kong/H090-785-V10/2009
PB1	CY073631	H1N1	08/08/2009	Nicaragua	A/Managua/5595.03/2009(H1N1)

PB1	CY112081	H1N1	08/08/2009	Hong Kong	A/Hong Kong/H090-787-V10/2009
PB1	CY112049	H1N1	08/08/2009	Hong Kong	A/Hong Kong/H090-779-V20/2009
PB1	CY120475	H1N1	08/08/2009	Hong Kong	A/Hong Kong/H090-787-V10/2009
PB1	CY120459	H1N1	08/08/2009	Hong Kong	A/Hong Kong/H090-779-V22/2009
PB1	CY073591	H1N1	08/08/2009	Nicaragua	A/Managua/4064.01/2009(H1N1)
PB1	CY112057	H1N1	08/08/2009	Hong Kong	A/Hong Kong/H090-779-V21/2009
PB1	CY112065	H1N1	08/08/2009	Hong Kong	A/Hong Kong/H090-779-V22/2009
PB1	CY120451	H1N1	08/08/2009	Hong Kong	A/Hong Kong/H090-779-V21/2009
PB1	CY120098	H1N1	08/08/2009	Hong Kong	A/Hong Kong/H090-779-V20/2009
PB1	CY119424	H1N1	08/09/2009	Malaysia	A/Malaysia/2089888/2009
PB1	KC781727	H1N1	08/09/2009	USA	A/Oregon/30/2009
PB1	CY119416	H1N1	08/09/2009	Malaysia	A/Malaysia/2089841/2009
PB1	CY060540	H1N1	08/09/2009	Canada	A/Ontario/25389/2009(H1N1)
PB1	KC781739	H1N1	08/09/2009	USA	A/Wyoming/08/2009
PB1	CY055500	H1N1	08/10/2009	USA	A/California/VRDL74/2009(H1N1)
PB1	CY055009	H1N1	08/10/2009	USA	A/California/VRDL64/2009(H1N1)
PB1	KT180456	H1N1	08/10/2009	Singapore	A/Singapore/DMS22/2009
PB1	KY935466	H1N1	08/10/2009	Brazil	A/Rio Grande/LACENRS-1406/2009
PB1	JN596870	H1N1	08/10/2009	Russia	A/Kurgan/01/2009
PB1	CY073551	H1N1	08/10/2009	Nicaragua	A/Managua/3083.01/2009(H1N1)
PB1	HM014330	H1N1	08/10/2009	China	A/Guangzhou/GIRD07/2009(H1N1)
PB1	CY119440	H1N1	08/10/2009	Malaysia	A/Malaysia/2098044/2009
PB1	CY055017	H1N1	08/10/2009	USA	A/California/VRDL65/2009(H1N1)
PB1	CY072692	H1N1	08/10/2009	Nicaragua	A/Managua/554.01/2009(H1N1)
PB1	CY071504	H1N1	08/10/2009	USA	A/California/WR1317P/2009(H1N1)
PB1	CY055906	H1N1	08/10/2009	Australia	A/Australia/72/2009(H1N1)
PB1	CY119432	H1N1	08/10/2009	Malaysia	A/Malaysia/2098001/2009
PB1	CY119448	H1N1	08/10/2009	Malaysia	A/Malaysia/2098053/2009
PB1	CY123504	H1N1	08/11/2009	Singapore	A/Singapore/ON1760/2009
PB1	CY073543	H1N1	08/11/2009	Nicaragua	A/Managua/4012.01/2009(H1N1)
PB1	CY115868	H1N1	08/11/2009	Netherlands	A/Netherlands/947b/2009
PB1	CY119456	H1N1	08/11/2009	Malaysia	A/Malaysia/2097717/2009
PB1	CY123492	H1N1	08/11/2009	Singapore	A/Singapore/ON1747/2009
PB1	CY118226	H1N1	08/11/2009	Malaysia	A/Malaysia/2098038/2009
PB1	CY055033	H1N1	08/11/2009	USA	A/California/VRDL68/2009(H1N1)
PB1	CY072596	H1N1	08/11/2009	Nicaragua	A/Managua/1356.03/2009(H1N1)
PB1	CY072652	H1N1	08/11/2009	Nicaragua	A/Managua/2275.01/2009(H1N1)
PB1	CY073567	H1N1	08/11/2009	Nicaragua	A/Managua/5119.06/2009(H1N1)
PB1	CY072708	H1N1	08/11/2009	Nicaragua	A/Managua/5036.01/2009(H1N1)
PB1	CY069280	H1N1	08/11/2009	Nicaragua	A/Managua/5212.04/2009(H1N1)
PB1	CY072668	H1N1	08/11/2009	Nicaragua	A/Managua/2339.03/2009(H1N1)
PB1	CY060516	H1N1	08/11/2009	Canada	A/Ontario/222656/2009(H1N1)
PB1	CY112089	H1N1	08/12/2009	Hong Kong	A/Hong Kong/H090-793-V10/2009
PB1	CY055914	H1N1	08/12/2009	Australia	A/Australia/73/2009(H1N1)
PB1	KY925727	H1N1	08/12/2009	Brazil	A/Porto Alegre/LACENRS-1598/2009
PB1	JN596867	H1N1	08/12/2009	Russia	A/Omsk/01/2009
PB1	KY926172	H1N1	08/12/2009	Brazil	A/Alegrete/LACENRS-1453/2009
PB1	CY128137	H1N1	08/12/2009	Viet Nam	A/Viet Nam/13032001/2009
PB1	CY128145	H1N1	08/12/2009	Viet Nam	A/Viet Nam/13032002/2009
PB1	CY120483	H1N1	08/12/2009	Hong Kong	A/Hong Kong/H090-793-V10/2009
PB1	CY083230	H1N1	08/12/2009	Guam	A/Guam/WRAIR1510P/2009
PB1	CY120721	H1N1	08/12/2009	Brazil	A/Brazil/AVS03/2009
PB1	CY072588	H1N1	08/12/2009	Nicaragua	A/Managua/4186.02/2009(H1N1)
PB1	CY072724	H1N1	08/12/2009	Nicaragua	A/Managua/3359.01/2009(H1N1)

PB1	KY925724	H1N1	08/13/2009	Brazil	A/Vacaria/LACENRS-1720/2009
PB1	KY925825	H1N1	08/13/2009	Brazil	A/Venancio Aires/LACENRS-1539/2(
PB1	KT180458	H1N1	08/13/2009	Singapore	A/Singapore/DMS23/2009
PB1	CY073583	H1N1	08/13/2009	Nicaragua	A/Managua/921.02/2009(H1N1)
PB1	CY120729	H1N1	08/13/2009	Brazil	A/Brazil/AVS04/2009
PB1	KY925829	H1N1	08/13/2009	Brazil	A/Santo Angelo/LACENRS-1537/200
PB1	CY072644	H1N1	08/13/2009	Nicaragua	A/Managua/3642.04/2009(H1N1)
PB1	CY072996	H1N1	08/13/2009	Nicaragua	A/Managua/527.04/2009(H1N1)
PB1	CY112097	H1N1	08/14/2009	Hong Kong	A/Hong Kong/H090-793-V20/2009
PB1	CY051701	H1N1	08/14/2009	USA	A/New York/4777/2009(H1N1)
PB1	CY055269	H1N1	08/14/2009	Malaysia	A/Malaysia/10226/2009(H1N1)
PB1	CY120491	H1N1	08/14/2009	Hong Kong	A/Hong Kong/H090-793-V20/2009
PB1	KY925210	H1N1	08/14/2009	Brazil	A/Pelotas/LACENRS-1658/2009
PB1	CY122965	H1N1	08/14/2009	Singapore	A/Singapore/GP2982/2009
PB1	KP637828	H1N1	08/14/2009	Thailand	A/Thailand/IY07083/2009
PB1	KY926409	H1N1	08/14/2009	Brazil	A/Triunfo/LACENRS-1611/2009
PB1	KY926250	H1N1	08/14/2009	Brazil	A/Sao Gabriel/LACENRS-1626/2009
PB1	CY072716	H1N1	08/15/2009	Nicaragua	A/Managua/5307.01/2009(H1N1)
PB1	CY058250	H1N1	08/15/2009	Nicaragua	A/Managua/3275.01/2009(H1N1)
PB1	CY058242	H1N1	08/15/2009	Nicaragua	A/Managua/473.02/2009(H1N1)
PB1	CY052877	H1N1	08/15/2009	USA	A/Texas/44151841/2009(H1N1)
PB1	CY058610	H1N1	08/15/2009	Nicaragua	A/Managua/5747.02/2009(H1N1)
PB1	CY052853	H1N1	08/15/2009	USA	A/Texas/44152535/2009(H1N1)
PB1	KY925476	H1N1	08/15/2009	Brazil	A/Santo Antonio da Patrulha/LACI
PB1	CY055802	H1N1	08/16/2009	Australia	A/Australia/54/2009(H1N1)
PB1	CY072660	H1N1	08/16/2009	Nicaragua	A/Managua/4570.01/2009(H1N1)
PB1	CY072676	H1N1	08/16/2009	Nicaragua	A/Managua/1054.02/2009(H1N1)
PB1	KT180459	H1N1	08/16/2009	Singapore	A/Singapore/DMS24/2009
PB1	CY122973	H1N1	08/16/2009	Singapore	A/Singapore/GP2987/2009
PB1	KP222605	H1N1	08/17/2009	Singapore	A/Singapore/463B/2009
PB1	KY925395	H1N1	08/17/2009	Brazil	A/Santa Cruz do Sul/LACENRS-201
PB1	CY072612	H1N1	08/17/2009	Nicaragua	A/Managua/2155.01/2009(H1N1)
PB1	CY122993	H1N1	08/17/2009	Singapore	A/Singapore/GP3021/2009
PB1	KP222606	H1N1	08/17/2009	Singapore	A/Singapore/464L/2009
PB1	CY123001	H1N1	08/17/2009	Singapore	A/Singapore/GP3026/2009
PB1	CY058570	H1N1	08/17/2009	Nicaragua	A/Managua/4218.01/2009(H1N1)
PB1	CY060508	H1N1	08/17/2009	Canada	A/Ontario/10296/2009(H1N1)
PB1	CY058266	H1N1	08/18/2009	Nicaragua	A/Managua/164.01/2009(H1N1)
PB1	CY055025	H1N1	08/18/2009	USA	A/California/VRDL67/2009(H1N1)
PB1	CY051709	H1N1	08/18/2009	USA	A/New York/4780/2009(H1N1)
PB1	CY063752	H1N1	08/18/2009	Singapore	A/Singapore/GP3084/2009(H1N1)
PB1	CY058258	H1N1	08/18/2009	Nicaragua	A/Managua/3244.01/2009(H1N1)
PB1	CY123049	H1N1	08/18/2009	Singapore	A/Singapore/GP3093/2009
PB1	CY123033	H1N1	08/18/2009	Singapore	A/Singapore/GP3074/2009
PB1	KP222607	H1N1	08/18/2009	Singapore	A/Singapore/468H/2009
PB1	CY058562	H1N1	08/18/2009	Nicaragua	A/Managua/2093.01/2009(H1N1)
PB1	CY058586	H1N1	08/18/2009	Nicaragua	A/Managua/989.01/2009(H1N1)
PB1	CY058602	H1N1	08/18/2009	Nicaragua	A/Managua/3435.01/2009(H1N1)
PB1	JX625489	H1N1	08/18/2009	United Ki	A/England/93520038/2009
PB1	CY123025	H1N1	08/18/2009	Singapore	A/Singapore/GP3069/2009
PB1	CY063580	H1N1	08/18/2009	USA	A/Boston/154/2009(H1N1)
PB1	CY058618	H1N1	08/18/2009	Nicaragua	A/Managua/2323.02/2009(H1N1)
PB1	CY058578	H1N1	08/18/2009	Nicaragua	A/Managua/4757.02/2009(H1N1)
PB1	CY123057	H1N1	08/19/2009	Singapore	A/Singapore/GP3104/2009

PB1	CY123522	H1N1	08/19/2009	Singapore	A/Singapore/ON1802/2009
PB1	CY072852	H1N1	08/19/2009	Nicaragua	A/Managua/4702.04/2009(H1N1)
PB1	KY925932	H1N1	08/19/2009	Brazil	A/Tenente Portela/LACENRS-2073/2
PB1	JQ431376	H1N1	08/19/2009	France	A/Reunion/0148-6-M1E/2009
PB1	JQ431374	H1N1	08/19/2009	France	A/Reunion/0148-4-M1E/2009
PB1	CY123530	H1N1	08/19/2009	Singapore	A/Singapore/ON1808/2009
PB1	KT180460	H1N1	08/19/2009	Singapore	A/Singapore/DMS25/2009
PB1	CY055922	H1N1	08/20/2009	Australia	A/Australia/79/2009(H1N1)
PB1	CY128169	H1N1	08/20/2009	Viet Nam	A/Viet Nam/13032011/2009
PB1	CY072772	H1N1	08/20/2009	Nicaragua	A/Managua/287.01/2009(H1N1)
PB1	CY051725	H1N1	08/20/2009	USA	A/New York/4789/2009(H1N1)
PB1	CY072876	H1N1	08/20/2009	Nicaragua	A/Managua/2999.03/2009(H1N1)
PB1	CY055277	H1N1	08/20/2009	Malaysia	A/Malaysia/12617/2009(H1N1)
PB1	KP222608	H1N1	08/20/2009	Singapore	A/Singapore/480Y/2009
PB1	CY128161	H1N1	08/20/2009	Viet Nam	A/Viet Nam/13032009/2009
PB1	CY128185	H1N1	08/20/2009	Viet Nam	A/Viet Nam/13032016/2009
PB1	CY128177	H1N1	08/20/2009	Viet Nam	A/Viet Nam/13032015/2009
PB1	KY925797	H1N1	08/20/2009	Brazil	A/Rosario do Sul/LACENRS-2110/2(
PB1	KY925412	H1N1	08/20/2009	Brazil	A/Porto Alegre/LACENRS-2121/2009
PB1	CY128153	H1N1	08/20/2009	Viet Nam	A/Viet Nam/13032008/2009
PB1	JQ431373	H1N1	08/20/2009	France	A/Reunion/2923-3-M1E/2009
PB1	CY060524	H1N1	08/21/2009	Canada	A/Ontario/235657/2009(H1N1)
PB1	CY051733	H1N1	08/21/2009	USA	A/New York/4790/2009(H1N1)
PB1	CY051717	H1N1	08/21/2009	USA	A/New York/4787/2009(H1N1)
PB1	CY123065	H1N1	08/21/2009	Singapore	A/Singapore/GP3175/2009
PB1	CY128193	H1N1	08/21/2009	Viet Nam	A/Viet Nam/13032019/2009
PB1	CY123081	H1N1	08/21/2009	Singapore	A/Singapore/GP3179/2009
PB1	KP222609	H1N1	08/21/2009	Singapore	A/Singapore/483A/2009
PB1	CY052168	H1N1	08/21/2009	USA	A/New York/4788/2009(H1N1)
PB1	CY051749	H1N1	08/21/2009	USA	A/New York/4810/2009(H1N1)
PB1	CY072804	H1N1	08/22/2009	Nicaragua	A/Managua/432.01/2009(H1N1)
PB1	CY072756	H1N1	08/22/2009	Nicaragua	A/Managua/5227.04/2009(H1N1)
PB1	JQ431378	H1N1	08/22/2009	France	A/Reunion/1709-4-M2E/2009
PB1	KT180461	H1N1	08/22/2009	Singapore	A/Singapore/DMS26/2009
PB1	CY072868	H1N1	08/22/2009	Nicaragua	A/Managua/4147.01/2009(H1N1)
PB1	KY926078	H1N1	08/23/2009	Brazil	A/Ijui/LACENRS-2326/2009
PB1	JQ431369	H1N1	08/24/2009	France	A/Reunion/2133-5-M1E/2009
PB1	JQ431368	H1N1	08/24/2009	France	A/Reunion/2133-6-M1E/2009
PB1	CY051741	H1N1	08/24/2009	USA	A/New York/4791/2009(H1N1)
PB1	CY119544	H1N1	08/24/2009	Malaysia	A/Malaysia/2126082/2009
PB1	CY060532	H1N1	08/24/2009	Canada	A/Ontario/237882/2009(H1N1)
PB1	KT180498	H1N1	08/24/2009	Singapore	A/Singapore/DMS623/2009
PB1	CY072820	H1N1	08/24/2009	Nicaragua	A/Managua/3421.02/2009(H1N1)
PB1	CY119464	H1N1	08/24/2009	Malaysia	A/Malaysia/2126061/2009
PB1	JQ431366	H1N1	08/24/2009	France	A/Reunion/2378-1-M1E/2009
PB1	CY060500	H1N1	08/24/2009	Canada	A/Ontario/10016/2009(H1N1)
PB1	KT180496	H1N1	08/25/2009	Singapore	A/Singapore/DMS618/2009
PB1	CY056401	H1N1	08/25/2009	USA	A/New York/4925/2009(H1N1)
PB1	CY065798	H1N1	08/25/2009	Netherlan	A/Netherlands/1039/2009(H1N1)
PB1	KC781518	H1N1	08/25/2009	USA	A/Utah/51/2009
PB1	KP222610	H1N1	08/25/2009	Singapore	A/Singapore/491H/2009
PB1	KC780680	H1N1	08/25/2009	USA	A/West Virginia/13/2009
PB1	KT180500	H1N1	08/25/2009	Singapore	A/Singapore/DMS638/2009
PB1	CY058594	H1N1	08/25/2009	Nicaragua	A/Managua/65.02/2009(H1N1)

PB1	KT180495	H1N1	08/25/2009	Singapore	A/Singapore/DMS617/2009
PB1	JQ431371	H1N1	08/26/2009	France	A/Reunion/2224-3-M3E/2009
PB1	JQ431377	H1N1	08/26/2009	France	A/Reunion/0159-3-M1E/2009
PB1	KT180503	H1N1	08/26/2009	Singapore	A/Singapore/DMS653/2009
PB1	KP222612	H1N1	08/26/2009	Singapore	A/Singapore/496T/2009
PB1	CY055930	H1N1	08/26/2009	Australia	A/Australia/80/2009(H1N1)
PB1	KP222611	H1N1	08/26/2009	Singapore	A/Singapore/495W/2009
PB1	KT180507	H1N1	08/27/2009	Singapore	A/Singapore/DMS676/2009
PB1	JN187213	H1N1	08/27/2009	Taiwan	A/Taiwan/6078/2009
PB1	CY055049	H1N1	08/27/2009	USA	A/California/VRDL70/2009(H1N1)
PB1	KP222613	H1N1	08/27/2009	Singapore	A/Singapore/501L/2009
PB1	KP222614	H1N1	08/27/2009	Singapore	A/Singapore/502J/2009
PB1	KT180505	H1N1	08/27/2009	Singapore	A/Singapore/DMS665/2009
PB1	CY072908	H1N1	08/27/2009	Nicaragua	A/Managua/3434.02/2009(H1N1)
PB1	CY123582	H1N1	08/27/2009	Singapore	A/Singapore/ON1892/2009
PB1	CY123089	H1N1	08/27/2009	Singapore	A/Singapore/GP3183/2009
PB1	AB538387	H1N1	08/27/2009	Japan	A/Nagano/RC1/2009(H1N1)
PB1	KC781961	H1N1	08/27/2009	USA	A/Mississippi/05/2009
PB1	CY119472	H1N1	08/27/2009	Malaysia	A/Malaysia/2135565/2009
PB1	CY123614	H1N1	08/28/2009	Singapore	A/Singapore/ON1911/2009
PB1	CY123606	H1N1	08/28/2009	Singapore	A/Singapore/ON1908/2009
PB1	CY072948	H1N1	08/28/2009	Nicaragua	A/Managua/1244.01/2009(H1N1)
PB1	CY055057	H1N1	08/28/2009	USA	A/California/VRDL71/2009(H1N1)
PB1	KC780566	H1N1	08/28/2009	USA	A/Minnesota/15/2009
PB1	KT180462	H1N1	08/28/2009	Singapore	A/Singapore/DMS28/2009
PB1	KC782316	H1N1	08/28/2009	USA	A/Minnesota/16/2009
PB1	CY073671	H1N1	08/28/2009	Nicaragua	A/Managua/4209.04/2009(H1N1)
PB1	KP222615	H1N1	08/28/2009	Singapore	A/Singapore/506T/2009
PB1	CY123598	H1N1	08/28/2009	Singapore	A/Singapore/ON1900/2009
PB1	CY052773	H1N1	08/28/2009	USA	A/Texas/44282651/2009(H1N1)
PB1	KT180510	H1N1	08/28/2009	Singapore	A/Singapore/DMS698/2009
PB1	KP637844	H1N1	08/29/2009	Thailand	A/Thailand/IY07086/2009
PB1	CY119480	H1N1	08/29/2009	Malaysia	A/Malaysia/2139971/2009
PB1	CY128201	H1N1	08/29/2009	Viet Nam	A/Viet Nam/13032030/2009
PB1	CY052224	H1N1	08/30/2009	USA	A/Texas/44301765/2009(H1N1)
PB1	CY052629	H1N1	08/30/2009	USA	A/Texas/44302167/2009(H1N1)
PB1	CY052232	H1N1	08/30/2009	USA	A/Texas/44302551/2009(H1N1)
PB1	CY052621	H1N1	08/30/2009	USA	A/Texas/44302533/2009(H1N1)
PB1	CY072796	H1N1	08/30/2009	Nicaragua	A/Managua/1984.02/2009(H1N1)
PB1	CY052677	H1N1	08/31/2009	USA	A/Texas/44313703/2009(H1N1)
PB1	KT180463	H1N1	08/31/2009	Singapore	A/Singapore/DMS29/2009
PB1	CY052344	H1N1	08/31/2009	USA	A/Texas/44312415/2009(H1N1)
PB1	CY072932	H1N1	08/31/2009	Nicaragua	A/Managua/4063.01/2009(H1N1)
PB1	CY073679	H1N1	08/31/2009	Nicaragua	A/Managua/5248.03/2009(H1N1)
PB1	KT180513	H1N1	08/31/2009	Singapore	A/Singapore/DMS722/2009
PB1	CY072980	H1N1	08/31/2009	Nicaragua	A/Managua/3507.01/2009(H1N1)
PB1	KY926077	H1N1	08/31/2009	Brazil	A/Palmitinho/LACENRS-2595/2009
PB1	HM189649	H1N1	09/2009	South Ko	A/Korea/CJ04/2009(H1N1)
PB1	KC782309	H1N1	09/01/2009	USA	A/Wisconsin/53/2009
PB1	CY073687	H1N1	09/01/2009	Nicaragua	A/Managua/4736.02/2009(H1N1)
PB1	CY065129	H1N1	09/01/2009	Netherlan	A/Netherlands/1064b/2009(H1N1)
PB1	CY119336	H1N1	09/01/2009	Malaysia	A/Malaysia/2142295/2009(H1N1)
PB1	CY119352	H1N1	09/01/2009	Malaysia	A/Malaysia/2143696/2009(H1N1)
PB1	CY119344	H1N1	09/01/2009	Malaysia	A/Malaysia/2142299/2009(H1N1)

PB1	CY123140	H1N1	09/01/2009	Singapore	A/Singapore/GP3491/2009
PB1	CY055484	H1N1	09/01/2009	USA	A/California/VRDL72/2009(H1N1)
PB1	CY128209	H1N1	09/01/2009	Viet Nam	A/Viet Nam/13032035/2009
PB1	CY052685	H1N1	09/01/2009	USA	A/Texas/45010998/2009(H1N1)
PB1	CY055492	H1N1	09/01/2009	USA	A/California/VRDL73/2009(H1N1)
PB1	CY123655	H1N1	09/01/2009	Singapore	A/Singapore/ON1934/2009
PB1	CY128465	H1N1	09/01/2009	Viet Nam	A/Viet Nam/001-1889/2009
PB1	CY055938	H1N1	09/02/2009	Australia	A/Australia/81/2009(H1N1)
PB1	JQ431367	H1N1	09/02/2009	France	A/Reunion/2692-2-M2E/2009
PB1	CY052781	H1N1	09/02/2009	USA	A/Texas/45021632/2009(H1N1)
PB1	CY052861	H1N1	09/02/2009	USA	A/Texas/45023717/2009(H1N1)
PB1	CY069104	H1N1	09/02/2009	Guam	A/Guam/NHRC0027/2009(H1N1)
PB1	CY123665	H1N1	09/02/2009	Singapore	A/Singapore/ON1943/2009
PB1	JQ431365	H1N1	09/02/2009	France	A/Reunion/2154-4-M4E/2009
PB1	KY926421	H1N1	09/02/2009	Brazil	A/Osorio/LACENRS-2638/2009
PB1	KT180517	H1N1	09/02/2009	Singapore	A/Singapore/DMS752/2009
PB1	KT180514	H1N1	09/02/2009	Singapore	A/Singapore/DMS737/2009
PB1	CY072748	H1N1	09/02/2009	Nicaragua	A/Managua/2958.01/2009(H1N1)
PB1	CY051757	H1N1	09/02/2009	USA	A/New York/4820/2009(H1N1)
PB1	CY055946	H1N1	09/02/2009	Australia	A/Australia/82/2009(H1N1)
PB1	CY052885	H1N1	09/02/2009	USA	A/Texas/45024243/2009(H1N1)
PB1	CY051765	H1N1	09/03/2009	USA	A/New York/4823/2009(H1N1)
PB1	CY052597	H1N1	09/03/2009	USA	A/Texas/45033774/2009(H1N1)
PB1	CY052909	H1N1	09/03/2009	USA	A/Texas/45032708/2009(H1N1)
PB1	CY052941	H1N1	09/03/2009	USA	A/Texas/45032753/2009(H1N1)
PB1	CY051781	H1N1	09/03/2009	USA	A/New York/4841/2009(H1N1)
PB1	CY052613	H1N1	09/03/2009	USA	A/Texas/45034157/2009(H1N1)
PB1	JQ431372	H1N1	09/03/2009	France	A/Reunion/2969-5-M1E/2009
PB1	CY128217	H1N1	09/03/2009	Viet Nam	A/Viet Nam/13032036/2009
PB1	CY123681	H1N1	09/03/2009	Singapore	A/Singapore/ON1964/2009
PB1	CY123673	H1N1	09/03/2009	Singapore	A/Singapore/ON1955/2009
PB1	CY052605	H1N1	09/03/2009	USA	A/Texas/45033567/2009(H1N1)
PB1	KT180464	H1N1	09/03/2009	Singapore	A/Singapore/DMS30/2009
PB1	CY051773	H1N1	09/03/2009	USA	A/New York/4824/2009(H1N1)
PB1	CY128225	H1N1	09/04/2009	Viet Nam	A/Viet Nam/13032037/2009
PB1	CY052789	H1N1	09/04/2009	USA	A/Texas/45043852/2009(H1N1)
PB1	JQ431370	H1N1	09/04/2009	France	A/Reunion/0215-4-M1E/2009
PB1	CY065886	H1N1	09/04/2009	Netherlan	A/Netherlands/1093/2009(H1N1)
PB1	CY068992	H1N1	09/04/2009	Guam	A/Guam/NHRC0013/2009(H1N1)
PB1	CY069016	H1N1	09/04/2009	Guam	A/Guam/NHRC0016/2009(H1N1)
PB1	CY052869	H1N1	09/04/2009	USA	A/Texas/45042604/2009(H1N1)
PB1	KT180522	H1N1	09/04/2009	Singapore	A/Singapore/DMS804/2009
PB1	CY070997	H1N1	09/04/2009	Guam	A/Guam/NHRC0029/2009(H1N1)
PB1	KT180526	H1N1	09/05/2009	Singapore	A/Singapore/DMS835/2009
PB1	CY052925	H1N1	09/05/2009	USA	A/Texas/45052569/2009(H1N1)
PB1	CY072812	H1N1	09/05/2009	Nicaragua	A/Managua/572.02/2009(H1N1)
PB1	JQ431364	H1N1	09/05/2009	France	A/Reunion/2154-2-M5E/2009
PB1	JQ431375	H1N1	09/05/2009	France	A/Reunion/0116-3-M1E/2009
PB1	CY071832	H1N1	09/05/2009	USA	A/South Carolina/WRSP520/2009(H
PB1	CY052637	H1N1	09/06/2009	USA	A/Texas/45061670/2009(H1N1)
PB1	CY051789	H1N1	09/06/2009	USA	A/New York/4844/2009(H1N1)
PB1	CY052645	H1N1	09/06/2009	USA	A/Texas/45062584/2009(H1N1)
PB1	KT180465	H1N1	09/06/2009	Singapore	A/Singapore/DMS31/2009
PB1	GU108484	H1N1	09/06/2009	China	A/Zhejiang-Yiwu/11/2009(H1N1)

PB1	CY052893	H1N1	09/06/2009	USA	A/Texas/45062346/2009(H1N1)
PB1	CY052957	H1N1	09/06/2009	USA	A/Texas/45061755/2009(H1N1)
PB1	CY051901	H1N1	09/06/2009	USA	A/Texas/45062633/2009(H1N1)
PB1	CY052693	H1N1	09/07/2009	USA	A/Texas/45071524/2009(H1N1)
PB1	CY052829	H1N1	09/07/2009	USA	A/Texas/45072128/2009(H1N1)
PB1	GU189655	H1N1	09/07/2009	China	A/Zhejiang/DTID-ZJU03/2009(H1N1)
PB1	CY057884	H1N1	09/07/2009	USA	A/Wisconsin/629-D01987/2009(H1N
PB1	GU112096	H1N1	09/07/2009	China	A/Zhejiang/DTID-ZJU02/2009(H1N1)
PB1	CY052821	H1N1	09/07/2009	USA	A/Texas/45072656/2009(H1N1)
PB1	HM241716	H1N1	09/07/2009	India	A/Nsk/NIV10348/2009(H1N1)
PB1	CY119488	H1N1	09/07/2009	Malaysia	A/Malaysia/2156796/2009
PB1	CY052813	H1N1	09/07/2009	USA	A/Texas/45071344/2009(H1N1)
PB1	CY060548	H1N1	09/07/2009	Canada	A/Ontario/25913/2009(H1N1)
PB1	CY052797	H1N1	09/07/2009	USA	A/Texas/45072273/2009(H1N1)
PB1	CY057001	H1N1	09/08/2009	USA	A/Wisconsin/629-D00734/2009(H1N
PB1	CY052701	H1N1	09/08/2009	USA	A/Texas/45083819/2009(H1N1)
PB1	CY128233	H1N1	09/08/2009	Viet Nam	A/Viet Nam/13032040/2009
PB1	KT180527	H1N1	09/08/2009	Singapore	A/Singapore/DMS847/2009
PB1	CY057009	H1N1	09/08/2009	USA	A/Wisconsin/629-D02367/2009(H1N
PB1	CY123699	H1N1	09/08/2009	Singapore	A/Singapore/ON2009/2009
PB1	KT180529	H1N1	09/08/2009	Singapore	A/Singapore/DMS869/2009
PB1	CY073695	H1N1	09/08/2009	Nicaragua	A/Managua/5953.01/2009(H1N1)
PB1	CY057017	H1N1	09/09/2009	USA	A/Wisconsin/629-D02041/2009(H1N
PB1	CY069088	H1N1	09/09/2009	Guam	A/Guam/NHRC0025/2009(H1N1)
PB1	CY069112	H1N1	09/09/2009	Guam	A/Guam/NHRC0028/2009(H1N1)
PB1	CY057025	H1N1	09/09/2009	USA	A/Wisconsin/629-D02133/2009(H1N
PB1	CY052741	H1N1	09/09/2009	USA	A/Texas/45091417/2009(H1N1)
PB1	CY052725	H1N1	09/09/2009	USA	A/Texas/45091402/2009(H1N1)
PB1	CY052733	H1N1	09/09/2009	USA	A/Texas/45091405/2009(H1N1)
PB1	CY052709	H1N1	09/09/2009	USA	A/Texas/45093214/2009(H1N1)
PB1	CY051805	H1N1	09/09/2009	USA	A/New York/4866/2009(H1N1)
PB1	CY051797	H1N1	09/09/2009	USA	A/New York/4856/2009(H1N1)
PB1	CY052669	H1N1	09/09/2009	USA	A/Texas/45093670/2009(H1N1)
PB1	JN187214	H1N1	09/09/2009	Taiwan	A/Taiwan/937/2009
PB1	KT180530	H1N1	09/09/2009	Singapore	A/Singapore/DMS870/2009
PB1	HM568134	H1N1	09/09/2009	United Ki	A/England/H093780041/2009
PB1	CY052661	H1N1	09/09/2009	USA	A/Texas/45093846/2009(H1N1)
PB1	CY052176	H1N1	09/09/2009	USA	A/New York/4855/2009(H1N1)
PB1	KC781748	H1N1	09/10/2009	USA	A/North Carolina/37/2009
PB1	CY119520	H1N1	09/10/2009	Malaysia	A/Malaysia/2097960/2009
PB1	CY057033	H1N1	09/10/2009	USA	A/Wisconsin/629-D00357/2009(H1N
PB1	CY052901	H1N1	09/10/2009	USA	A/Texas/45102952/2009(H1N1)
PB1	CY052757	H1N1	09/10/2009	USA	A/Texas/45103759/2009(H1N1)
PB1	CY052765	H1N1	09/10/2009	USA	A/Texas/45101422/2009(H1N1)
PB1	JN187215	H1N1	09/10/2009	Taiwan	A/Taiwan/940/2009
PB1	CY052517	H1N1	09/10/2009	USA	A/Texas/45103737/2009(H1N1)
PB1	CY051813	H1N1	09/10/2009	USA	A/New York/4870/2009(H1N1)
PB1	CY052653	H1N1	09/10/2009	USA	A/Texas/45103259/2009(H1N1)
PB1	CY052509	H1N1	09/10/2009	USA	A/Texas/45104026/2009(H1N1)
PB1	CY052525	H1N1	09/10/2009	USA	A/Texas/45104048/2009(H1N1)
PB1	CY052533	H1N1	09/10/2009	USA	A/Texas/45103998/2009(H1N1)
PB1	CY072924	H1N1	09/10/2009	Nicaragua	A/Managua/4972.02/2009(H1N1)
PB1	CY065918	H1N1	09/10/2009	Netherlar	A/Netherlands/1084/2009(H1N1)
PB1	CY065910	H1N1	09/10/2009	Netherlar	A/Netherlands/1083/2009(H1N1)

PB1	CY052749	H1N1	09/10/2009	USA	A/Texas/45101424/2009(H1N1)
PB1	KY925997	H1N1	09/10/2009	Brazil	A/Campina das Missoes/LACENRS-28
PB1	HM568142	H1N1	09/10/2009	United Ki	A/England/H093780034/2009
PB1	KT180534	H1N1	09/10/2009	Singapore	A/Singapore/DMS905/2009
PB1	KC780110	H1N1	09/11/2009	USA	A/Alaska/38/2009
PB1	CY052917	H1N1	09/11/2009	USA	A/Texas/45113911/2009(H1N1)
PB1	CY128241	H1N1	09/11/2009	Viet Nam	A/Viet Nam/13032044/2009
PB1	CY052501	H1N1	09/11/2009	USA	A/Texas/45113371/2009(H1N1)
PB1	CY057364	H1N1	09/11/2009	USA	A/Wisconsin/629-D02485/2009(H1N
PB1	KT180536	H1N1	09/11/2009	Singapore	A/Singapore/DMS913/2009
PB1	CY123152	H1N1	09/11/2009	Singapore	A/Singapore/GP3545/2009
PB1	CY083062	H1N1	09/11/2009	Colombia	A/Bogota/WRAIR0435N/2009
PB1	CY083070	H1N1	09/11/2009	Colombia	A/Bogota/WRAIR0435T/2009
PB1	CY073519	H1N1	09/11/2009	Nicaragua	A/Managua/3014.02/2009(H1N1)
PB1	CY072828	H1N1	09/11/2009	Nicaragua	A/Managua/5115.01/2009(H1N1)
PB1	CY052541	H1N1	09/11/2009	USA	A/Texas/45113882/2009(H1N1)
PB1	CY072740	H1N1	09/12/2009	Nicaragua	A/Managua/1419.02/2009(H1N1)
PB1	KT180466	H1N1	09/12/2009	Singapore	A/Singapore/DMS33/2009
PB1	CY052461	H1N1	09/12/2009	USA	A/Texas/45122369/2009(H1N1)
PB1	CY052485	H1N1	09/12/2009	USA	A/Texas/45122282/2009(H1N1)
PB1	CY052477	H1N1	09/12/2009	USA	A/Texas/45122033/2009(H1N1)
PB1	CY052445	H1N1	09/12/2009	USA	A/Texas/45122538/2009(H1N1)
PB1	CY052184	H1N1	09/12/2009	USA	A/New York/4881/2009(H1N1)
PB1	CY052933	H1N1	09/12/2009	USA	A/Texas/45121004/2009(H1N1)
PB1	CY057380	H1N1	09/12/2009	USA	A/Wisconsin/629-D01850/2009(H1N
PB1	CY052565	H1N1	09/12/2009	USA	A/Texas/45122722/2009(H1N1)
PB1	CY052949	H1N1	09/12/2009	USA	A/Texas/45122886/2009(H1N1)
PB1	CY052437	H1N1	09/12/2009	USA	A/Texas/45120922/2009(H1N1)
PB1	CY052469	H1N1	09/12/2009	USA	A/Texas/45121606/2009(H1N1)
PB1	CY052557	H1N1	09/12/2009	USA	A/Texas/45122774/2009(H1N1)
PB1	CY052493	H1N1	09/12/2009	USA	A/Texas/45122036/2009(H1N1)
PB1	CY057372	H1N1	09/12/2009	USA	A/Wisconsin/629-D00643/2009(H1N
PB1	CY072836	H1N1	09/12/2009	Nicaragua	A/Managua/1909.02/2009(H1N1)
PB1	CY069024	H1N1	09/13/2009	Guam	A/Guam/NHRC0017/2009(H1N1)
PB1	CY052845	H1N1	09/13/2009	USA	A/Texas/45132202/2009(H1N1)
PB1	CY052453	H1N1	09/13/2009	USA	A/Texas/45130742/2009(H1N1)
PB1	CY052573	H1N1	09/13/2009	USA	A/Texas/45132788/2009(H1N1)
PB1	CY052805	H1N1	09/13/2009	USA	A/Texas/45131576/2009(H1N1)
PB1	CY052549	H1N1	09/13/2009	USA	A/Texas/45132214/2009(H1N1)
PB1	CY052581	H1N1	09/13/2009	USA	A/Texas/45131774/2009(H1N1)
PB1	CY052589	H1N1	09/13/2009	USA	A/Texas/45132647/2009(H1N1)
PB1	CY052837	H1N1	09/13/2009	USA	A/Texas/45131305/2009(H1N1)
PB1	CY049649	H1N1	09/13/2009	Singapore	A/Singapore/ON2081/2009(H1N1)
PB1	CY072892	H1N1	09/14/2009	Nicaragua	A/Managua/12.01/2009(H1N1)
PB1	CY072940	H1N1	09/14/2009	Nicaragua	A/Managua/5590.02/2009(H1N1)
PB1	CY069048	H1N1	09/14/2009	Guam	A/Guam/NHRC0020/2009(H1N1)
PB1	CY071013	H1N1	09/14/2009	Guam	A/Guam/NHRC0031/2009(H1N1)
PB1	CY052717	H1N1	09/14/2009	USA	A/Texas/45140902/2009(H1N1)
PB1	KF897770	H1N1	09/14/2009	France	A/StEtienne/1691/2009
PB1	CY055429	H1N1	09/14/2009	USA	A/New York/4887/2009(H1N1)
PB1	KC780831	H1N1	09/14/2009	USA	A/Kansas/20/2009
PB1	JX625558	H1N1	09/14/2009	United Ki	A/England/94560024/2009
PB1	KC780921	H1N1	09/14/2009	USA	A/Kansas/19/2009
PB1	CY123743	H1N1	09/14/2009	Singapore	A/Singapore/ON2081/2009

PB1	CY071021	H1N1	09/14/2009	Guam	A/Guam/NHRC0032/2009(H1N1)
PB1	KT180539	H1N1	09/15/2009	Singapore	A/Singapore/DMS946/2009
PB1	CY053472	H1N1	09/15/2009	Taiwan	A/Taiwan/143/2009(H1N1)
PB1	CY123751	H1N1	09/15/2009	Singapore	A/Singapore/ON2095/2009
PB1	CY057412	H1N1	09/15/2009	USA	A/Wisconsin/629-D00832/2009(H1N
PB1	CY057388	H1N1	09/15/2009	USA	A/Wisconsin/629-D02192/2009(H1N
PB1	CY057396	H1N1	09/15/2009	USA	A/Wisconsin/629-D00036/2009(H1N
PB1	KT180540	H1N1	09/15/2009	Singapore	A/Singapore/DMS952/2009
PB1	CY090095	H1N1	09/15/2009	Nicaragua	A/Managua/3755.03/2009
PB1	KC781044	H1N1	09/15/2009	USA	A/Massachusetts/31/2009
PB1	CY069072	H1N1	09/15/2009	Guam	A/Guam/NHRC0023/2009(H1N1)
PB1	CY119496	H1N1	09/15/2009	Malaysia	A/Malaysia/2173411/2009
PB1	KT180467	H1N1	09/15/2009	Singapore	A/Singapore/DMS34/2009
PB1	CY057404	H1N1	09/15/2009	USA	A/Wisconsin/629-D01469/2009(H1N
PB1	KC782216	H1N1	09/16/2009	USA	A/South Carolina/18/2009
PB1	CY088651	H1N1	09/16/2009	India	A/Rtng/NIV11650/2009
PB1	KC782210	H1N1	09/16/2009	USA	A/South Carolina/18/2009
PB1	KT180542	H1N1	09/16/2009	Singapore	A/Singapore/DMS962/2009
PB1	KC780853	H1N1	09/16/2009	USA	A/Kansas/21/2009
PB1	CY057428	H1N1	09/16/2009	USA	A/Wisconsin/629-D00485/2009(H1N
PB1	CY057420	H1N1	09/16/2009	USA	A/Wisconsin/629-D01802/2009(H1N
PB1	CY120737	H1N1	09/16/2009	Brazil	A/Brazil/AVS06/2009
PB1	CY057436	H1N1	09/16/2009	USA	A/Wisconsin/629-D01935/2009(H1N
PB1	KC781312	H1N1	09/16/2009	USA	A/Wisconsin/66/2009
PB1	KC780078	H1N1	09/16/2009	USA	A/South Carolina/18/2009
PB1	CY072788	H1N1	09/16/2009	Nicaragua	A/Managua/5401.01/2009(H1N1)
PB1	CY128249	H1N1	09/17/2009	Viet Nam	A/Viet Nam/13032048/2009
PB1	CY119504	H1N1	09/18/2009	Malaysia	A/Malaysia/2180812/2009
PB1	CY053496	H1N1	09/18/2009	Taiwan	A/Taiwan/177/2009(H1N1)
PB1	CY053488	H1N1	09/18/2009	Taiwan	A/Taiwan/167/2009(H1N1)
PB1	KY925012	H1N1	09/18/2009	Brazil	A/Pelotas/LACENRS-3045/2009
PB1	KT180468	H1N1	09/18/2009	Singapore	A/Singapore/DMS35/2009
PB1	CY057444	H1N1	09/18/2009	USA	A/Wisconsin/629-D01522/2009(H1N
PB1	KP637540	H1N1	09/18/2009	Thailand	A/Thailand/SN08312/2009
PB1	HM568150	H1N1	09/18/2009	United Ki	A/England/H093900113/2009
PB1	KT180547	H1N1	09/18/2009	Singapore	A/Singapore/DMS982/2009
PB1	KT180544	H1N1	09/18/2009	Singapore	A/Singapore/DMS978/2009
PB1	KT180546	H1N1	09/18/2009	Singapore	A/Singapore/DMS980/2009
PB1	CY055508	H1N1	09/19/2009	USA	A/California/VRDL75/2009(H1N1)
PB1	KC781057	H1N1	09/20/2009	USA	A/North Carolina/44/2009
PB1	CY069000	H1N1	09/20/2009	Guam	A/Guam/NHRC0014/2009(H1N1)
PB1	JN187216	H1N1	09/20/2009	Taiwan	A/Taiwan/8575/2009
PB1	KT180341	H1N1	09/21/2009	Singapore	A/Singapore/DMS1006/2009
PB1	CY057452	H1N1	09/21/2009	USA	A/Wisconsin/629-D01355/2009(H1N
PB1	AB539737	H1N1	09/21/2009	China	A/Shanghai/P1/2009(H1N1)
PB1	CY055516	H1N1	09/21/2009	USA	A/California/VRDL76/2009(H1N1)
PB1	CY057460	H1N1	09/21/2009	USA	A/Wisconsin/629-D00748/2009(H1N
PB1	CY057468	H1N1	09/22/2009	USA	A/Wisconsin/629-D00839/2009(H1N
PB1	KC781983	H1N1	09/22/2009	USA	A/Kansas/22/2009
PB1	CY057484	H1N1	09/22/2009	USA	A/Wisconsin/629-D01237/2009(H1N
PB1	CY055524	H1N1	09/22/2009	USA	A/California/VRDL78/2009(H1N1)
PB1	GU211233	H1N1	09/22/2009	Russia	A/Russia/01/2009(H1N1)
PB1	CY128449	H1N1	09/22/2009	Viet Nam	A/Viet Nam/001-1716/2009
PB1	CY057476	H1N1	09/22/2009	USA	A/Wisconsin/629-D00402/2009(H1N

PB1	KJ690398	H1N1	09/23/2009	Uganda	A/Uganda/MUWRP-093/2009
PB1	CY056409	H1N1	09/23/2009	USA	A/New York/4944/2009(H1N1)
PB1	CY123765	H1N1	09/23/2009	Singapore	A/Singapore/ON2135/2009
PB1	KC780972	H1N1	09/23/2009	USA	A/Kansas/23/2009
PB1	CY057492	H1N1	09/23/2009	USA	A/Wisconsin/629-D00346/2009(H1N
PB1	CY123773	H1N1	09/23/2009	Singapore	A/Singapore/ON2136/2009
PB1	CY123186	H1N1	09/23/2009	Singapore	A/Singapore/GP3839/2009
PB1	CY057500	H1N1	09/23/2009	USA	A/Wisconsin/629-D00287/2009(H1N
PB1	CY056425	H1N1	09/23/2009	USA	A/New York/4947/2009(H1N1)
PB1	CY056417	H1N1	09/23/2009	USA	A/New York/4945/2009(H1N1)
PB1	KT180469	H1N1	09/24/2009	Singapore	A/Singapore/DMS37/2009
PB1	CY119512	H1N1	09/24/2009	Malaysia	A/Malaysia/2190140/2009
PB1	CY071736	H1N1	09/24/2009	Mexico	A/Mexico City/WR1701T/2009(H1N1)
PB1	CY054889	H1N1	09/24/2009	USA	A/California/VRDL36/2009(H1N1)
PB1	CY057508	H1N1	09/24/2009	USA	A/Wisconsin/629-D00565/2009(H1N
PB1	CY057516	H1N1	09/24/2009	USA	A/Wisconsin/629-D00410/2009(H1N
PB1	CY069064	H1N1	09/25/2009	Guam	A/Guam/NHRC0022/2009(H1N1)
PB1	KC780155	H1N1	09/25/2009	USA	A/Mississippi/03/2009
PB1	CY057524	H1N1	09/26/2009	USA	A/Wisconsin/629-D00891/2009(H1N
PB1	CY069080	H1N1	09/26/2009	Guam	A/Guam/NHRC0024/2009(H1N1)
PB1	KC780904	H1N1	09/27/2009	USA	A/Wisconsin/51/2009
PB1	KJ690477	H1N1	09/28/2009	Uganda	A/Uganda/MUWRP-098/2009
PB1	CY123781	H1N1	09/28/2009	Singapore	A/Singapore/ON2145/2009
PB1	JN187218	H1N1	09/28/2009	Taiwan	A/Taiwan/8542/2009
PB1	JN187217	H1N1	09/28/2009	Taiwan	A/Taiwan/4909/2009
PB1	CY057532	H1N1	09/28/2009	USA	A/Wisconsin/629-D01391/2009(H1N
PB1	KC781001	H1N1	09/29/2009	USA	A/Texas/66/2009
PB1	CY060556	H1N1	09/29/2009	Canada	A/Ontario/26184/2009(H1N1)
PB1	CY058282	H1N1	09/29/2009	USA	A/Wisconsin/629-D01015/2009(H1N
PB1	CY072956	H1N1	09/29/2009	Nicaragua	A/Managua/5258.02/2009(H1N1)
PB1	KC780989	H1N1	09/29/2009	USA	A/Texas/66/2009
PB1	KC780032	H1N1	09/30/2009	USA	A/Wisconsin/67/2009
PB1	CY123196	H1N1	09/30/2009	Singapore	A/Singapore/GP3912/2009
PB1	CY065926	H1N1	09/30/2009	Netherlar	A/Netherlands/2290/2009
PB1	CY083437	H1N1	09/30/2009	USA	A/Ft.Benning/WRAIR1669P/2009
PB1	KT180471	H1N1	09/30/2009	Singapore	A/Singapore/DMS39/2009
PB1	HM189656	H1N1	10/2009	South Kor	A/Korea/CJ68/2009(H1N1)
PB1	HM189654	H1N1	10/2009	South Kor	A/Korea/CJ40/2009(H1N1)
PB1	HM189655	H1N1	10/2009	South Kor	A/Korea/CJ63/2009(H1N1)
PB1	CY062232	H1N1	10/01/2009	USA	A/California/VRDL83/2009(H1N1)
PB1	CY063225	H1N1	10/01/2009	USA	A/Wisconsin/629-D01351/2009(H1N
PB1	CY062216	H1N1	10/01/2009	USA	A/California/VRDL81/2009(H1N1)
PB1	CY069008	H1N1	10/01/2009	Guam	A/Guam/NHRC0015/2009(H1N1)
PB1	CY062200	H1N1	10/01/2009	USA	A/California/VRDL79/2009(H1N1)
PB1	CY062208	H1N1	10/01/2009	USA	A/California/VRDL80/2009(H1N1)
PB1	CY128257	H1N1	10/01/2009	Viet Nam	A/Viet Nam/13032051/2009
PB1	CY068960	H1N1	10/01/2009	Guam	A/Guam/NHRC0009/2009(H1N1)
PB1	CY118273	H1N1	10/01/2009	Malaysia	A/Malaysia/2205822/2009
PB1	JX625542	H1N1	10/01/2009	United Ki	A/Northern Ireland/94480417/200
PB1	KC781997	H1N1	10/01/2009	USA	A/North Carolina/45/2009
PB1	CY062224	H1N1	10/01/2009	USA	A/California/VRDL82/2009(H1N1)
PB1	CY056577	H1N1	10/01/2009	USA	A/New York/5755/2009(H1N1)
PB1	KC780135	H1N1	10/02/2009	USA	A/New Mexico/08/2009
PB1	KT180472	H1N1	10/02/2009	Singapore	A/Singapore/DMS40/2009

PB1	CY057548	H1N1	10/02/2009	USA	A/Wisconsin/629-D00853/2009(H1N
PB1	JX625504	H1N1	10/02/2009	United Ki	A/England/94200009/2009
PB1	CY057540	H1N1	10/02/2009	USA	A/Wisconsin/629-D00250/2009(H1N
PB1	CY055437	H1N1	10/03/2009	USA	A/New York/4977/2009(H1N1)
PB1	CY057556	H1N1	10/03/2009	USA	A/Wisconsin/629-D02337/2009(H1N
PB1	CY056489	H1N1	10/04/2009	USA	A/New York/4995/2009(H1N1)
PB1	CY057564	H1N1	10/04/2009	USA	A/Wisconsin/629-D00690/2009(H1N
PB1	CY056449	H1N1	10/05/2009	USA	A/New York/4985/2009(H1N1)
PB1	CY056433	H1N1	10/05/2009	USA	A/New York/4981/2009(H1N1)
PB1	CY055445	H1N1	10/05/2009	USA	A/New York/4979/2009(H1N1)
PB1	JX625526	H1N1	10/05/2009	United Ki	A/Northern Ireland/94480397/2009
PB1	CY056457	H1N1	10/05/2009	USA	A/New York/4986/2009(H1N1)
PB1	CY057572	H1N1	10/05/2009	USA	A/Wisconsin/629-D00834/2009(H1N
PB1	KT180473	H1N1	10/05/2009	Singapore	A/Singapore/DMS41/2009
PB1	KJ690540	H1N1	10/05/2009	Uganda	A/Uganda/MUWRP-102/2009
PB1	CY056481	H1N1	10/05/2009	USA	A/New York/4990/2009(H1N1)
PB1	CY058290	H1N1	10/06/2009	USA	A/Wisconsin/629-D01102/2009(H1N
PB1	CY056465	H1N1	10/06/2009	USA	A/New York/4987/2009(H1N1)
PB1	CY057580	H1N1	10/06/2009	USA	A/Wisconsin/629-S1252/2009(H1N1)
PB1	KC780930	H1N1	10/06/2009	USA	A/Colorado/13/2009
PB1	CY056441	H1N1	10/06/2009	USA	A/New York/4984/2009(H1N1)
PB1	CY056473	H1N1	10/06/2009	USA	A/New York/4988/2009(H1N1)
PB1	CY075498	H1N1	10/07/2009	USA	A/Boston/584/2009(H1N1)
PB1	CY058298	H1N1	10/07/2009	USA	A/Wisconsin/629-D02100/2009(H1N
PB1	KU922533	H1N1	10/07/2009	India	A/Kerala/RGCBA923/2009
PB1	CY083110	H1N1	10/07/2009	Ecuador	A/Quito/WRAIR0617N/2009
PB1	CY128265	H1N1	10/08/2009	Viet Nam	A/Viet Nam/13032059/2009
PB1	CY128273	H1N1	10/08/2009	Viet Nam	A/Viet Nam/13032060/2009
PB1	JN187219	H1N1	10/08/2009	Taiwan	A/Taiwan/7418/2009
PB1	KT180474	H1N1	10/08/2009	Singapore	A/Singapore/DMS42/2009
PB1	CY062256	H1N1	10/09/2009	USA	A/California/VRDL86/2009(H1N1)
PB1	CY063081	H1N1	10/09/2009	USA	A/California/VRDL88/2009(H1N1)
PB1	CY062248	H1N1	10/09/2009	USA	A/California/VRDL85/2009(H1N1)
PB1	CY128281	H1N1	10/09/2009	Viet Nam	A/Viet Nam/13032061/2009
PB1	CY057604	H1N1	10/09/2009	USA	A/Wisconsin/629-D01080/2009(H1N
PB1	JX625604	H1N1	10/09/2009	United Ki	A/Northern Ireland/94940020/2009
PB1	JX625511	H1N1	10/09/2009	United Ki	A/England/1072/2009
PB1	JX625534	H1N1	10/09/2009	United Ki	A/Northern Ireland/94480404/2009
PB1	CY063073	H1N1	10/09/2009	USA	A/California/VRDL87/2009(H1N1)
PB1	CY062240	H1N1	10/09/2009	USA	A/California/VRDL84/2009(H1N1)
PB1	KP637460	H1N1	10/09/2009	Thailand	A/Thailand/PY08063/2009
PB1	CY057596	H1N1	10/09/2009	USA	A/Wisconsin/629-D02090/2009(H1N
PB1	CY057588	H1N1	10/09/2009	USA	A/Wisconsin/629-D01749/2009(H1N
PB1	KC780400	H1N1	10/10/2009	USA	A/New Mexico/06/2009
PB1	CY057612	H1N1	10/10/2009	USA	A/Wisconsin/629-D00557/2009(H1N
PB1	CY089193	H1N1	10/10/2009	USA	A/Boston/583/2009(H1N1)
PB1	CY072700	H1N1	10/11/2009	Nicaragua	A/Managua/3013.01/2009(H1N1)
PB1	CY096043	H1N1	10/11/2009	China	A/Hubei/99/2009
PB1	CY095961	H1N1	10/11/2009	China	A/Zhejiang/87/2009
PB1	CY095969	H1N1	10/11/2009	China	A/Zhejiang/88/2009
PB1	CY095933	H1N1	10/11/2009	China	A/Zhejiang/83/2009
PB1	HM568062	H1N1	10/12/2009	United Ki	A/England/H094240645/2009
PB1	CY056881	H1N1	10/12/2009	USA	A/San Diego/INS51/2009(H1N1)
PB1	CY063241	H1N1	10/12/2009	USA	A/Wisconsin/629-D02442/2009(H1N

PB1	CY057236	H1N1	10/12/2009	USA	A/New York/5045/2009(H1N1)
PB1	CY063249	H1N1	10/12/2009	USA	A/Wisconsin/629-D00830/2009(H1N
PB1	CY057628	H1N1	10/12/2009	USA	A/Wisconsin/629-S1313/2009(H1N1)
PB1	CY057620	H1N1	10/12/2009	USA	A/Wisconsin/629-S1309/2009(H1N1)
PB1	KC261310	H1N1	10/12/2009	Mexico	A/Mexico City/INERPHAC1/2009(H1N
PB1	CY055954	H1N1	10/12/2009	USA	A/San Diego/INS01/2009(H1N1)
PB1	CY055978	H1N1	10/12/2009	USA	A/San Diego/INS04/2009(H1N1)
PB1	CY056210	H1N1	10/12/2009	USA	A/San Diego/INS52/2009(H1N1)
PB1	KT180475	H1N1	10/12/2009	Singapore	A/Singapore/DMS43/2009
PB1	HM568070	H1N1	10/12/2009	United Ki	A/England/H094220579/2009
PB1	CY055962	H1N1	10/12/2009	USA	A/San Diego/INS02/2009(H1N1)
PB1	CY056218	H1N1	10/12/2009	USA	A/San Diego/INS53/2009(H1N1)
PB1	KP637276	H1N1	10/12/2009	Thailand	A/Thailand/IY07093/2009
PB1	CY066533	H1N1	10/12/2009	USA	A/San Diego/INS193/2009(H1N1)
PB1	CY055994	H1N1	10/12/2009	USA	A/San Diego/INS06/2009(H1N1)
PB1	CY055970	H1N1	10/12/2009	USA	A/San Diego/INS03/2009(H1N1)
PB1	CY055986	H1N1	10/12/2009	USA	A/San Diego/INS05/2009(H1N1)
PB1	CY056889	H1N1	10/12/2009	USA	A/San Diego/INS54/2009(H1N1)
PB1	CY083948	H1N1	10/12/2009	USA	A/San Diego/INS194/2009(H1N1)
PB1	CY063233	H1N1	10/12/2009	USA	A/Wisconsin/629-D00675/2009(H1N
PB1	CY056529	H1N1	10/13/2009	USA	A/New York/5130/2009(H1N1)
PB1	CY056505	H1N1	10/13/2009	USA	A/New York/5079/2009(H1N1)
PB1	CY071608	H1N1	10/13/2009	USA	A/Ft Carson/WR1448P/2009(H1N1)
PB1	CY056513	H1N1	10/13/2009	USA	A/New York/5083/2009(H1N1)
PB1	CY056521	H1N1	10/13/2009	USA	A/New York/5086/2009(H1N1)
PB1	CY083836	H1N1	10/13/2009	USA	A/San Diego/INS07/2009(H1N1)
PB1	CY056497	H1N1	10/13/2009	USA	A/New York/5074/2009(H1N1)
PB1	CY056010	H1N1	10/13/2009	USA	A/San Diego/INS10/2009(H1N1)
PB1	CY057324	H1N1	10/13/2009	USA	A/New York/5667/2009(H1N1)
PB1	CY056002	H1N1	10/13/2009	USA	A/San Diego/INS09/2009(H1N1)
PB1	CY057340	H1N1	10/13/2009	USA	A/San Diego/INS08/2009(H1N1)
PB1	CY081524	H1N1	10/14/2009	Cambodia	A/Cambodia/NHRCC00005/2009(H1N1)
PB1	CY081516	H1N1	10/14/2009	Cambodia	A/Cambodia/NHRCC00004/2009(H1N1)
PB1	CY081548	H1N1	10/14/2009	Cambodia	A/Cambodia/NHRCC00008/2009(H1N1)
PB1	CY083493	H1N1	10/14/2009	USA	A/Chicago/WRAIR1691P/2009
PB1	CY081492	H1N1	10/14/2009	Cambodia	A/Cambodia/NHRCC00001/2009(H1N1)
PB1	CY057636	H1N1	10/14/2009	USA	A/Wisconsin/629-S1348/2009(H1N1)
PB1	CY057644	H1N1	10/14/2009	USA	A/Wisconsin/629-S1350/2009(H1N1)
PB1	CY083477	H1N1	10/14/2009	USA	A/Chicago/WRAIR1690P/2009
PB1	KT180476	H1N1	10/14/2009	Singapore	A/Singapore/DMS44/2009
PB1	CY081508	H1N1	10/14/2009	Cambodia	A/Cambodia/NHRCC00003/2009(H1N1)
PB1	CY081532	H1N1	10/14/2009	Cambodia	A/Cambodia/NHRCC00006/2009(H1N1)
PB1	CY061241	H1N1	10/14/2009	USA	A/San Diego/INS102/2009(H1N1)
PB1	CY056018	H1N1	10/14/2009	USA	A/San Diego/INS11/2009(H1N1)
PB1	CY056026	H1N1	10/14/2009	USA	A/San Diego/INS12/2009(H1N1)
PB1	CY081500	H1N1	10/14/2009	Cambodia	A/Cambodia/NHRCC00002/2009(H1N1)
PB1	CY081540	H1N1	10/14/2009	Cambodia	A/Cambodia/NHRCC00007/2009(H1N1)
PB1	CY056897	H1N1	10/14/2009	USA	A/San Diego/INS55/2009(H1N1)
PB1	CY061233	H1N1	10/14/2009	USA	A/San Diego/INS101/2009(H1N1)
PB1	CY063257	H1N1	10/15/2009	USA	A/Wisconsin/629-D00692/2009(H1N
PB1	KC780339	H1N1	10/15/2009	USA	A/New York/102/2009
PB1	CY080350	H1N1	10/15/2009	Cambodia	A/Cambodia/NHRCC00011/2009(H1N1)
PB1	CY092926	H1N1	10/15/2009	USA	A/Maryland/NHRC0002/2009
PB1	CY057244	H1N1	10/15/2009	USA	A/New York/5158/2009(H1N1)

PB1	CY082990	H1N1	10/15/2009	Cambodia	A/Cambodia/NHRCC00009/2009(H1N1)
PB1	CY056905	H1N1	10/15/2009	USA	A/San Diego/INS57/2009(H1N1)
PB1	CY066541	H1N1	10/15/2009	USA	A/San Diego/INS195/2009(H1N1)
PB1	CY081556	H1N1	10/15/2009	Cambodia	A/Cambodia/NHRCC00010/2009(H1N1)
PB1	CY056082	H1N1	10/15/2009	USA	A/District of Columbia/INS24/20(
PB1	JX625566	H1N1	10/15/2009	United Ki	A/Northern Ireland/1/2009
PB1	KC781026	H1N1	10/16/2009	USA	A/North Carolina/43/2009
PB1	CY056649	H1N1	10/16/2009	USA	A/New York/6079/2009(H1N1)
PB1	CY057252	H1N1	10/16/2009	USA	A/New York/5161/2009(H1N1)
PB1	KC780511	H1N1	10/16/2009	USA	A/North Carolina/46/2009
PB1	CY056545	H1N1	10/16/2009	USA	A/New York/5150/2009(H1N1)
PB1	CY056537	H1N1	10/16/2009	USA	A/New York/5141/2009(H1N1)
PB1	CY063265	H1N1	10/16/2009	USA	A/Wisconsin/629-D00740/2009(H1N
PB1	CY092934	H1N1	10/16/2009	USA	A/Maryland/NHRC0003/2009
PB1	CY063089	H1N1	10/17/2009	USA	A/California/VRDL89/2009(H1N1)
PB1	CY058044	H1N1	10/17/2009	USA	A/Texas/46172731/2009(H1N1)
PB1	CY063113	H1N1	10/17/2009	USA	A/California/VRDL92/2009(H1N1)
PB1	GU198199	H1N1	10/17/2009	China	A/Nanjing/3/2009(H1N1)
PB1	CY063105	H1N1	10/17/2009	USA	A/California/VRDL91/2009(H1N1)
PB1	CY063097	H1N1	10/17/2009	USA	A/California/VRDL90/2009(H1N1)
PB1	CY063121	H1N1	10/17/2009	USA	A/California/VRDL93/2009(H1N1)
PB1	CY058052	H1N1	10/17/2009	USA	A/Texas/46172734/2009(H1N1)
PB1	CY056553	H1N1	10/17/2009	USA	A/New York/5173/2009(H1N1)
PB1	KC780113	H1N1	10/18/2009	USA	A/New Mexico/07/2009
PB1	CY128289	H1N1	10/18/2009	Viet Nam	A/Viet Nam/13032071/2009
PB1	CY058068	H1N1	10/18/2009	USA	A/Texas/46181292/2009(H1N1)
PB1	CY057668	H1N1	10/18/2009	USA	A/Wisconsin/629-D00099/2009(H1N
PB1	CY057652	H1N1	10/18/2009	USA	A/Wisconsin/629-D02342/2009(H1N
PB1	CY075506	H1N1	10/18/2009	USA	A/Boston/591/2009(H1N1)
PB1	CY058690	H1N1	10/18/2009	USA	A/Texas/46182018/2009(H1N1)
PB1	CY058060	H1N1	10/18/2009	USA	A/Texas/46181235/2009(H1N1)
PB1	CY060564	H1N1	10/18/2009	Canada	A/Ontario/296008/2009(H1N1)
PB1	CY057660	H1N1	10/18/2009	USA	A/Wisconsin/629-D00544/2009(H1N
PB1	CY057676	H1N1	10/19/2009	USA	A/Wisconsin/629-D00132/2009(H1N
PB1	CY060761	H1N1	10/19/2009	USA	A/Texas/46193632/2009(H1N1)
PB1	CY058698	H1N1	10/19/2009	USA	A/Texas/461917783/2009(H1N1)
PB1	CY058714	H1N1	10/19/2009	USA	A/Texas/46193311/2009(H1N1)
PB1	CY061249	H1N1	10/19/2009	USA	A/San Diego/INS103/2009(H1N1)
PB1	CY083844	H1N1	10/19/2009	USA	A/San Diego/INS14/2009(H1N1)
PB1	CY083645	H1N1	10/19/2009	USA	A/San Diego/INS13/2009
PB1	CY056066	H1N1	10/19/2009	USA	A/District of Columbia/INS20/20(
PB1	KJ690454	H1N1	10/19/2009	Uganda	A/Uganda/MUWRP-111/2009
PB1	CY060572	H1N1	10/19/2009	Canada	A/Ontario/29801/2009(H1N1)
PB1	CY057260	H1N1	10/19/2009	USA	A/New York/5186/2009(H1N1)
PB1	CY058706	H1N1	10/19/2009	USA	A/Texas/46192760/2009(H1N1)
PB1	JX403976	H1N1	10/19/2009	China	A/Liaoning/1/2009(H1N1)
PB1	CY063273	H1N1	10/19/2009	USA	A/Wisconsin/629-D02082/2009(H1N
PB1	CY057684	H1N1	10/19/2009	USA	A/Wisconsin/629-S1384/2009(H1N1)
PB1	CY057692	H1N1	10/19/2009	USA	A/Wisconsin/629-S1388/2009(H1N1)
PB1	CY056913	H1N1	10/19/2009	USA	A/San Diego/INS60/2009(H1N1)
PB1	JX625589	H1N1	10/20/2009	United Ki	A/Northern Ireland/94620068/200
PB1	JX625519	H1N1	10/20/2009	United Ki	A/England/94400003/2009
PB1	CY123807	H1N1	10/20/2009	Singapore	A/Singapore/ON2224/2009
PB1	CY056034	H1N1	10/20/2009	USA	A/San Diego/INS16/2009(H1N1)

PB1	CY060769	H1N1	10/20/2009	USA	A/Texas/46201823/2009(H1N1)
PB1	CY061257	H1N1	10/20/2009	USA	A/San Diego/INS104/2009(H1N1)
PB1	CY057276	H1N1	10/20/2009	USA	A/New York/5217/2009(H1N1)
PB1	CY056865	H1N1	10/20/2009	USA	A/San Diego/INS15/2009(H1N1)
PB1	JX625620	H1N1	10/20/2009	United Ki	A/Northern Ireland/94940033/2009
PB1	CY056569	H1N1	10/20/2009	USA	A/New York/5227/2009(H1N1)
PB1	CY056561	H1N1	10/20/2009	USA	A/New York/5199/2009(H1N1)
PB1	CY057268	H1N1	10/20/2009	USA	A/New York/5196/2009(H1N1)
PB1	CY057284	H1N1	10/20/2009	USA	A/New York/5221/2009(H1N1)
PB1	HM568046	H1N1	10/20/2009	United Ki	A/England/950/2009
PB1	JX625612	H1N1	10/21/2009	United Ki	A/Northern Ireland/94940023/2009
PB1	CY066549	H1N1	10/21/2009	USA	A/San Diego/INS197/2009(H1N1)
PB1	CY060777	H1N1	10/21/2009	USA	A/Texas/46214103/2009(H1N1)
PB1	CY057700	H1N1	10/21/2009	USA	A/Wisconsin/629-S1398/2009(H1N1)
PB1	CY057708	H1N1	10/21/2009	USA	A/Wisconsin/629-S1397/2009(H1N1)
PB1	CY056921	H1N1	10/21/2009	USA	A/San Diego/INS61/2009(H1N1)
PB1	CY057300	H1N1	10/21/2009	USA	A/New York/5276/2009(H1N1)
PB1	HM567974	H1N1	10/21/2009	United Ki	A/England/1111/2009
PB1	HM568054	H1N1	10/21/2009	United Ki	A/England/1116/2009
PB1	CY071632	H1N1	10/21/2009	Taiwan	A/Taipei/WR1485T/2009(H1N1)
PB1	CY066557	H1N1	10/21/2009	USA	A/San Diego/INS198/2009(H1N1)
PB1	CY056138	H1N1	10/21/2009	USA	A/San Diego/INS34/2009(H1N1)
PB1	HM568094	H1N1	10/21/2009	United Ki	A/England/766/2009
PB1	CY056098	H1N1	10/21/2009	USA	A/District of Columbia/INS27/20
PB1	CY066565	H1N1	10/22/2009	USA	A/San Diego/INS199/2009(H1N1)
PB1	CY060801	H1N1	10/22/2009	USA	A/Texas/46223444/2009(H1N1)
PB1	CY060809	H1N1	10/22/2009	USA	A/Texas/46223582/2009(H1N1)
PB1	CY060785	H1N1	10/22/2009	USA	A/Texas/46221665/2009(H1N1)
PB1	CY057292	H1N1	10/22/2009	USA	A/New York/5271/2009(H1N1)
PB1	CY089209	H1N1	10/22/2009	USA	A/Boston/594/2009(H1N1)
PB1	CY057716	H1N1	10/22/2009	USA	A/Wisconsin/629-S1407/2009(H1N1)
PB1	CY057308	H1N1	10/22/2009	USA	A/New York/5297/2009(H1N1)
PB1	CY089201	H1N1	10/22/2009	USA	A/Boston/593/2009(H1N1)
PB1	CY065985	H1N1	10/22/2009	Mongolia	A/Ulaanbaatar/6525/2009(H1N1)
PB1	CY060817	H1N1	10/22/2009	USA	A/Texas/46224042/2009(H1N1)
PB1	HM568110	H1N1	10/22/2009	United Ki	A/England/798/2009
PB1	CY060793	H1N1	10/22/2009	USA	A/Texas/46222134/2009(H1N1)
PB1	CY057316	H1N1	10/23/2009	USA	A/New York/5447/2009(H1N1)
PB1	CY056106	H1N1	10/23/2009	USA	A/District of Columbia/INS28/20
PB1	CY060825	H1N1	10/23/2009	USA	A/Texas/46233104/2009(H1N1)
PB1	CY065870	H1N1	10/23/2009	Netherlar	A/Netherlands/1493b/2009
PB1	CY128297	H1N1	10/23/2009	Viet Nam	A/Viet Nam/13032074/2009
PB1	CY058306	H1N1	10/23/2009	USA	A/Wisconsin/629-D02018/2009(H1N
PB1	CY092942	H1N1	10/23/2009	USA	A/Maryland/NHRC0004/2009
PB1	KC780551	H1N1	10/23/2009	USA	A/West Virginia/12/2009
PB1	CY075514	H1N1	10/24/2009	USA	A/Boston/595/2009(H1N1)
PB1	CY060833	H1N1	10/24/2009	USA	A/Texas/46240925/2009(H1N1)
PB1	CY060841	H1N1	10/24/2009	USA	A/Texas/46241654/2009(H1N1)
PB1	CY128305	H1N1	10/24/2009	Viet Nam	A/Viet Nam/13032076/2009
PB1	CY060580	H1N1	10/24/2009	Canada	A/Ontario/304434/2009(H1N1)
PB1	HQ263286	H1N1	10/24/2009	USA	A/Bethesda/NIH107-D0/2009
PB1	CY092918	H1N1	10/25/2009	USA	A/Maryland/NHRC0001/2009
PB1	CY090022	H1N1	10/25/2009	USA	A/Wisconsin/629-D02330/2009(H1N
PB1	HM568102	H1N1	10/26/2009	United Ki	A/England/797/2009

PB1	CY056929	H1N1	10/26/2009	USA	A/San Diego/INS63/2009(H1N1)
PB1	CY058314	H1N1	10/26/2009	USA	A/Wisconsin/629-S1417/2009(H1N1)
PB1	CY056042	H1N1	10/26/2009	USA	A/District of Columbia/INS17/20(
PB1	CY056146	H1N1	10/26/2009	USA	A/San Diego/INS35/2009(H1N1)
PB1	CY056186	H1N1	10/26/2009	USA	A/District of Columbia/INS43/20(
PB1	CY058330	H1N1	10/26/2009	USA	A/Wisconsin/629-S1416/2009(H1N1)
PB1	CY064770	H1N1	10/26/2009	USA	A/District of Columbia/INS32/20(
PB1	JQ612497	H1N1	10/26/2009	Hungary	A/Hungary/40/2009
PB1	CY056130	H1N1	10/26/2009	USA	A/District of Columbia/INS33/20(
PB1	CY058322	H1N1	10/26/2009	USA	A/Wisconsin/629-S1414/2009(H1N1)
PB1	KC780056	H1N1	10/26/2009	USA	A/Washington/75/2009
PB1	CY056114	H1N1	10/26/2009	USA	A/District of Columbia/INS29/20(
PB1	JN187220	H1N1	10/27/2009	Taiwan	A/Taiwan/6200/2009
PB1	CY058642	H1N1	10/27/2009	USA	A/Wisconsin/629-S1425/2009(H1N1)
PB1	CY058634	H1N1	10/27/2009	USA	A/Wisconsin/629-S1431/2009(H1N1)
PB1	CY067641	H1N1	10/27/2009	Norway	A/Oslo/INS219/2009(H1N1)
PB1	CY060596	H1N1	10/27/2009	Canada	A/Ontario/305598/2009(H1N1)
PB1	CY060588	H1N1	10/27/2009	Canada	A/Ontario/305139/2009(H1N1)
PB1	KC780716	H1N1	10/27/2009	USA	A/Rhode Island/18/2009
PB1	CY056122	H1N1	10/27/2009	USA	A/District of Columbia/INS31/20(
PB1	KT180477	H1N1	10/27/2009	Singapore	A/Singapore/DMS46/2009
PB1	CY056058	H1N1	10/27/2009	USA	A/District of Columbia/INS19/20(
PB1	CY058626	H1N1	10/27/2009	USA	A/Wisconsin/629-S1433/2009(H1N1)
PB1	CY056937	H1N1	10/28/2009	USA	A/San Diego/INS65/2009(H1N1)
PB1	CY066821	H1N1	10/28/2009	USA	A/San Diego/INS218/2009
PB1	JN187221	H1N1	10/28/2009	Taiwan	A/Taiwan/7873/2009
PB1	CY056050	H1N1	10/28/2009	USA	A/District of Columbia/INS18/20(
PB1	CY066829	H1N1	10/28/2009	Belgium	A/Antwerp/INS221/2009(H1N1)
PB1	JX625550	H1N1	10/28/2009	United Ki	A/England/1397/2009
PB1	HM568078	H1N1	10/28/2009	United Ki	A/England/1213/2009
PB1	KY926060	H1N1	10/28/2009	Brazil	A/Porto Alegre/LACENRS-3434/200(
PB1	CY061273	H1N1	10/29/2009	Belgium	A/Brussels/INS106/2009(H1N1)
PB1	CY063129	H1N1	10/29/2009	USA	A/California/VRDL96/2009(H1N1)
PB1	CY128313	H1N1	10/29/2009	Viet Nam	A/Viet Nam/13032079/2009
PB1	CY063145	H1N1	10/29/2009	USA	A/California/VRDL98/2009(H1N1)
PB1	CY066197	H1N1	10/29/2009	USA	A/California/VRDL94/2009(H1N1)
PB1	AB704460	H1N1	10/29/2009	Japan	A/Tochigi/350/2009
PB1	CY063137	H1N1	10/29/2009	USA	A/California/VRDL97/2009(H1N1)
PB1	CY060612	H1N1	10/29/2009	Canada	A/Ontario/309862/2009(H1N1)
PB1	CY075522	H1N1	10/29/2009	USA	A/Boston/602/2009(H1N1)
PB1	CY066733	H1N1	10/30/2009	Belgium	A/Brussels/INS206/2009(H1N1)
PB1	CY060620	H1N1	10/30/2009	Canada	A/Ontario/313762/2009(H1N1)
PB1	JQ173101	H1N1	10/30/2009	China	A/Nanjing/NJU-108/2009
PB1	JN207842	H1N1	10/30/2009	China	A/Zhejiang/89w/2009(H1N1)
PB1	CY083852	H1N1	10/30/2009	USA	A/District of Columbia/INS23/20(
PB1	CY056945	H1N1	10/30/2009	Belgium	A/Brussels/INS71/2009(H1N1)
PB1	CY128321	H1N1	10/31/2009	Viet Nam	A/Viet Nam/13032088/2009
PB1	CY058666	H1N1	10/31/2009	USA	A/Wisconsin/629-D01913/2009(H1N
PB1	CY058658	H1N1	10/31/2009	USA	A/Wisconsin/629-D02002/2009(H1N
PB1	CY058650	H1N1	10/31/2009	USA	A/Wisconsin/629-D02329/2009(H1N
PB1	CY065814	H1N1	10/31/2009	Netherlar	A/Netherlands/1715b/2009
PB1	CY095868	H1N1	11/2009	China	A/Hubei/75/2009
PB1	CY095918	H1N1	11/2009	China	A/Zhejiang/81/2009
PB1	CY095852	H1N1	11/2009	China	A/Hubei/73/2009

PB1	CY095860	H1N1	11/2009	China	A/Hubei/74/2009
PB1	CY095876	H1N1	11/2009	China	A/Hubei/76/2009
PB1	CY054660	H1N1	11/01/2009	Russia	A/Russia/100/2009(H1N1)
PB1	CY054652	H1N1	11/01/2009	Russia	A/Russia/61/2009(H1N1)
PB1	JX625574	H1N1	11/01/2009	United Ki	A/England/94580011/2009
PB1	CY053679	H1N1	11/01/2009	Russia	A/Russia/14/2009(H1N1)
PB1	CY054636	H1N1	11/01/2009	Russia	A/Russia/12/2009(H1N1)
PB1	CY053742	H1N1	11/01/2009	Russia	A/Russia/190/2009(H1N1)
PB1	CY053734	H1N1	11/01/2009	Russia	A/Russia/178/2009(H1N1)
PB1	CY053726	H1N1	11/01/2009	Russia	A/Russia/74/2009(H1N1)
PB1	CY054628	H1N1	11/01/2009	Russia	A/Russia/4/2009(H1N1)
PB1	CY058674	H1N1	11/01/2009	USA	A/Wisconsin/629-D00193/2009(H1N
PB1	CY054644	H1N1	11/01/2009	Russia	A/Russia/19/2009(H1N1)
PB1	CY053687	H1N1	11/01/2009	Russia	A/Russia/191/2009(H1N1)
PB1	CY053750	H1N1	11/01/2009	Russia	A/Russia/200/2009(H1N1)
PB1	KT180478	H1N1	11/01/2009	Singapore	A/Singapore/DMS47/2009
PB1	CY060849	H1N1	11/02/2009	USA	A/Texas/JMS356/2009(H1N1)
PB1	CY058274	H1N1	11/02/2009	USA	A/New York/5620/2009(H1N1)
PB1	JF714140	H1N1	11/02/2009	South Ko	A/Korea/NAP-1/2009(H1N1)
PB1	CY075538	H1N1	11/02/2009	USA	A/Boston/612/2009(H1N1)
PB1	CY066885	H1N1	11/02/2009	USA	A/District of Columbia/INS229/2(
PB1	CY066573	H1N1	11/02/2009	USA	A/San Diego/INS201/2009(H1N1)
PB1	HM189310	H1N1	11/02/2009	Russia	A/Chita/CRIE-8/2009
PB1	CY060644	H1N1	11/02/2009	Canada	A/Ontario/314603/2009(H1N1)
PB1	CY060628	H1N1	11/02/2009	Canada	A/Ontario/314095/2009(H1N1)
PB1	CY058682	H1N1	11/02/2009	USA	A/Wisconsin/629-D00938/2009(H1N
PB1	CY123236	H1N1	11/02/2009	Singapore	A/Singapore/GP4218/2009
PB1	CY075530	H1N1	11/02/2009	USA	A/Boston/606/2009(H1N1)
PB1	CY056625	H1N1	11/02/2009	USA	A/New York/5988/2009(H1N1)
PB1	CY063281	H1N1	11/02/2009	USA	A/Wisconsin/629-D00888/2009(H1N
PB1	CY060668	H1N1	11/02/2009	Canada	A/Ontario/315047/2009(H1N1)
PB1	KC780860	H1N1	11/02/2009	USA	A/Wyoming/16/2009
PB1	CY056873	H1N1	11/03/2009	USA	A/District of Columbia/INS44/20(
PB1	HM100236	H1N1	11/03/2009	Hong Kong	A/Hong Kong/415742Md/2009(H1N1)
PB1	GU931816	H1N1	11/03/2009	Hong Kong	A/Hong Kong/415742M/2009(H1N1)
PB1	CY066837	H1N1	11/03/2009	Spain	A/Madrid/INS222/2009
PB1	CY089217	H1N1	11/03/2009	USA	A/Boston/618/2009
PB1	CY060636	H1N1	11/03/2009	Canada	A/Ontario/314137/2009(H1N1)
PB1	CY066741	H1N1	11/03/2009	Belgium	A/Brussels/INS208/2009(H1N1)
PB1	CY056226	H1N1	11/03/2009	USA	A/San Diego/INS68/2009(H1N1)
PB1	CY083246	H1N1	11/03/2009	Russia	A/Moscow/WRAIR1627T/2009(H1N1)
PB1	CY060684	H1N1	11/03/2009	Canada	A/Ontario/315181/2009(H1N1)
PB1	CY060692	H1N1	11/03/2009	Canada	A/Ontario/315187/2009(H1N1)
PB1	CY060652	H1N1	11/03/2009	Canada	A/Ontario/315003/2009(H1N1)
PB1	CY062649	H1N1	11/03/2009	USA	A/District of Columbia/INS45/20(
PB1	KJ690524	H1N1	11/03/2009	Uganda	A/Uganda/MUWRP-137/2009
PB1	CY056585	H1N1	11/03/2009	USA	A/New York/5782/2009(H1N1)
PB1	CY056194	H1N1	11/03/2009	USA	A/District of Columbia/INS46/20(
PB1	CY060700	H1N1	11/03/2009	Canada	A/Ontario/315613/2009(H1N1)
PB1	CY061265	H1N1	11/04/2009	USA	A/San Diego/INS105/2009(H1N1)
PB1	CY060857	H1N1	11/04/2009	USA	A/Texas/JMS358/2009(H1N1)
PB1	CY060676	H1N1	11/04/2009	Canada	A/Ontario/315107/2009(H1N1)
PB1	CY066589	H1N1	11/04/2009	USA	A/San Diego/INS203/2009(H1N1)
PB1	JX625582	H1N1	11/04/2009	United Ki	A/England/94620043/2009

PB1	CY062689	H1N1	11/04/2009	USA	A/District of Columbia/INS114/2(
PB1	KC780471	H1N1	11/04/2009	USA	A/Florida/25/2009
PB1	CY056178	H1N1	11/04/2009	USA	A/San Diego/INS42/2009(H1N1)
PB1	CY056633	H1N1	11/04/2009	USA	A/New York/6018/2009(H1N1)
PB1	CY056202	H1N1	11/04/2009	USA	A/San Diego/INS48/2009(H1N1)
PB1	CY060660	H1N1	11/04/2009	Canada	A/Ontario/315015/2009(H1N1)
PB1	CY056154	H1N1	11/04/2009	USA	A/Pensacola/INS37/2009(H1N1)
PB1	CY056162	H1N1	11/04/2009	USA	A/Pensacola/INS38/2009(H1N1)
PB1	CY060708	H1N1	11/04/2009	Canada	A/Ontario/315637/2009(H1N1)
PB1	CY066749	H1N1	11/04/2009	Belgium	A/Brussels/INS209/2009(H1N1)
PB1	CY066581	H1N1	11/04/2009	USA	A/San Diego/INS202/2009(H1N1)
PB1	KT180479	H1N1	11/04/2009	Singapore	A/Singapore/DMS48/2009
PB1	CY057748	H1N1	11/05/2009	USA	A/Wisconsin/629-D00151/2009(H1N
PB1	CY057756	H1N1	11/05/2009	USA	A/Wisconsin/629-D01570/2009(H1N
PB1	CY054676	H1N1	11/05/2009	Russia	A/Russia/180/2009(H1N1)
PB1	HQ263279	H1N1	11/05/2009	USA	A/Bethesda/NIH106-D14/2009
PB1	CY083860	H1N1	11/05/2009	USA	A/Pensacola/INS39/2009(H1N1)
PB1	CY083876	H1N1	11/05/2009	USA	A/San Diego/INS49/2009(H1N1)
PB1	CY083727	H1N1	11/05/2009	USA	A/District of Columbia/INS115/2(
PB1	CY056234	H1N1	11/05/2009	USA	A/San Diego/INS69/2009(H1N1)
PB1	CY066205	H1N1	11/05/2009	USA	A/California/VRDL103/2009(H1N1)
PB1	CY057740	H1N1	11/05/2009	USA	A/Wisconsin/629-D01140/2009(H1N
PB1	CY066901	H1N1	11/05/2009	USA	A/District of Columbia/INS231/2(
PB1	CY057764	H1N1	11/05/2009	USA	A/Wisconsin/629-D02370/2009(H1N
PB1	CY057724	H1N1	11/05/2009	USA	A/Wisconsin/629-D02317/2009(H1N
PB1	CY065822	H1N1	11/05/2009	Netherlar	A/Netherlands/2442b/2009
PB1	CY063177	H1N1	11/05/2009	USA	A/California/VRDL102/2009(H1N1)
PB1	CY063169	H1N1	11/05/2009	USA	A/California/VRDL101/2009(H1N1)
PB1	CY063161	H1N1	11/05/2009	USA	A/California/VRDL100/2009(H1N1)
PB1	CY066597	H1N1	11/05/2009	USA	A/San Diego/INS204/2009(H1N1)
PB1	CY056242	H1N1	11/05/2009	USA	A/San Diego/INS70/2009(H1N1)
PB1	CY063153	H1N1	11/05/2009	USA	A/California/VRDL99/2009(H1N1)
PB1	CY057732	H1N1	11/05/2009	USA	A/Wisconsin/629-D01915/2009(H1N
PB1	CY057772	H1N1	11/05/2009	USA	A/Wisconsin/629-D01346/2009(H1N
PB1	KC261311	H1N1	11/06/2009	Mexico	A/Mexico City/INERPHAC2/2009(H1N
PB1	KC780747	H1N1	11/06/2009	USA	A/Illinois/15/2009
PB1	CY056601	H1N1	11/06/2009	USA	A/New York/5916/2009(H1N1)
PB1	CY083868	H1N1	11/06/2009	USA	A/District of Columbia/INS47/20(
PB1	KT180480	H1N1	11/06/2009	Singapore	A/Singapore/DMS49/2009
PB1	KC782166	H1N1	11/07/2009	USA	A/New Mexico/16/2009
PB1	CY060873	H1N1	11/07/2009	USA	A/Texas/JMS361/2009(H1N1)
PB1	CY087206	H1N1	11/07/2009	USA	A/Texas/JMS360/2009
PB1	CY089233	H1N1	11/07/2009	USA	A/Boston/632/2009
PB1	CY057780	H1N1	11/07/2009	USA	A/Wisconsin/629-D00802/2009(H1N
PB1	CY060881	H1N1	11/07/2009	USA	A/Texas/JMS362/2009(H1N1)
PB1	CY060865	H1N1	11/07/2009	USA	A/Texas/JMS359/2009(H1N1)
PB1	CY065838	H1N1	11/07/2009	Netherlar	A/Netherlands/2457b/2009
PB1	CY089225	H1N1	11/07/2009	USA	A/Boston/630/2009
PB1	CY060897	H1N1	11/08/2009	USA	A/Texas/JMS364/2009(H1N1)
PB1	KC782062	H1N1	11/08/2009	USA	A/Wisconsin/55/2009
PB1	CY061560	H1N1	11/08/2009	USA	A/Texas/JMS366/2009(H1N1)
PB1	CY060905	H1N1	11/08/2009	USA	A/Texas/JMS365/2009(H1N1)
PB1	CY057812	H1N1	11/08/2009	USA	A/Wisconsin/629-D02392/2009(H1N
PB1	CY056657	H1N1	11/08/2009	USA	A/New York/6110/2009(H1N1)

PB1	CY057796	H1N1	11/08/2009	USA	A/Wisconsin/629-D01369/2009(H1N
PB1	CY060889	H1N1	11/08/2009	USA	A/Texas/JMS363/2009(H1N1)
PB1	CY060716	H1N1	11/08/2009	Canada	A/Ontario/320266/2009(H1N1)
PB1	CY089257	H1N1	11/08/2009	USA	A/Boston/637/2009
PB1	CY057804	H1N1	11/08/2009	USA	A/Wisconsin/629-D01466/2009(H1N
PB1	CY057788	H1N1	11/08/2009	USA	A/Wisconsin/629-D01014/2009(H1N
PB1	CY083900	H1N1	11/09/2009	USA	A/San Diego/INS73/2009(H1N1)
PB1	KC780206	H1N1	11/09/2009	USA	A/Maine/13/2009
PB1	KC782247	H1N1	11/09/2009	USA	A/Minnesota/40/2009
PB1	CY083892	H1N1	11/09/2009	USA	A/San Diego/INS72/2009(H1N1)
PB1	CY083884	H1N1	11/09/2009	USA	A/San Diego/INS50/2009(H1N1)
PB1	CY056609	H1N1	11/09/2009	USA	A/New York/5931/2009(H1N1)
PB1	KC780797	H1N1	11/09/2009	USA	A/Maryland/31/2009
PB1	CY056641	H1N1	11/09/2009	USA	A/New York/6019/2009(H1N1)
PB1	CY056593	H1N1	11/09/2009	USA	A/New York/5886/2009(H1N1)
PB1	CY089249	H1N1	11/09/2009	USA	A/Boston/635/2009
PB1	CY089241	H1N1	11/09/2009	USA	A/Boston/633/2009
PB1	CY056617	H1N1	11/09/2009	USA	A/New York/5976/2009(H1N1)
PB1	CY057332	H1N1	11/09/2009	USA	A/New York/6072/2009(H1N1)
PB1	CY066789	H1N1	11/09/2009	USA	A/San Diego/INS214/2009
PB1	CY056170	H1N1	11/09/2009	USA	A/Pensacola/INS40/2009(H1N1)
PB1	CY075546	H1N1	11/09/2009	USA	A/Boston/634/2009(H1N1)
PB1	CY066989	H1N1	11/09/2009	Belgium	A/Brussels/INS243/2009(H1N1)
PB1	KC780386	H1N1	11/09/2009	USA	A/Washington/62/2009
PB1	AB704492	H1N1	11/09/2009	Japan	A/Yamagata/473/2009
PB1	CY053625	H1N1	11/10/2009	Russia	A/Russia/165/2009(H1N1)
PB1	CY063588	H1N1	11/10/2009	Norway	A/Oslo/INS110/2009(H1N1)
PB1	HQ652620	H1N1	11/10/2009	China	A/Jiangsu/S61/2009
PB1	HM189350	H1N1	11/10/2009	Russia	A/Lipetsk/CRIE-BVV/2009
PB1	CY128329	H1N1	11/10/2009	Viet Nam	A/Viet Nam/13032093/2009
PB1	CY066757	H1N1	11/10/2009	USA	A/Pensacola/INS210/2009
PB1	CY066797	H1N1	11/10/2009	USA	A/San Diego/INS215/2009
PB1	HQ652621	H1N1	11/10/2009	China	A/Jiangsu/S62/2009
PB1	CY066773	H1N1	11/10/2009	USA	A/Pensacola/INS212/2009
PB1	CY065854	H1N1	11/10/2009	Netherlar	A/Netherlands/1324/2009(H1N1)
PB1	CY066765	H1N1	11/10/2009	USA	A/Pensacola/INS211/2009
PB1	CY062793	H1N1	11/10/2009	Denmark	A/Hvidovre/INS139/2009(H1N1)
PB1	CY084444	H1N1	11/11/2009	USA	A/New York/6064/2009
PB1	CY063289	H1N1	11/11/2009	USA	A/Wisconsin/629-D02008/2009(H1N
PB1	CY062817	H1N1	11/11/2009	Denmark	A/Odense/INS143/2009(H1N1)
PB1	CY066845	H1N1	11/11/2009	Spain	A/Madrid/INS223/2009
PB1	JN032404	H1N1	11/11/2009	China	A/Changchun/01/2009
PB1	CY066805	H1N1	11/12/2009	USA	A/San Diego/INS216/2009
PB1	CY061281	H1N1	11/12/2009	USA	A/Pensacola/INS107/2009(H1N1)
PB1	CY075554	H1N1	11/12/2009	USA	A/Boston/648/2009(H1N1)
PB1	CY067649	H1N1	11/12/2009	Spain	A/Madrid/INS224/2009(H1N1)
PB1	KC781071	H1N1	11/12/2009	USA	A/Oklahoma/05/2009
PB1	KT180481	H1N1	11/12/2009	Singapore	A/Singapore/DMS50/2009
PB1	CY063297	H1N1	11/12/2009	USA	A/Wisconsin/629-D00292/2009(H1N
PB1	CY066813	H1N1	11/12/2009	USA	A/San Diego/INS217/2009
PB1	CY054668	H1N1	11/12/2009	Russia	A/Russia/171/2009(H1N1)
PB1	CY066861	H1N1	11/12/2009	USA	A/District of Columbia/INS226/2(
PB1	CY061568	H1N1	11/12/2009	USA	A/Texas/JMS367/2009(H1N1)
PB1	CY062657	H1N1	11/12/2009	USA	A/San Diego/INS74/2009(H1N1)

PB1	HM173605	H1N1	11/12/2009	Russia	A/Blagovechensk/01/2009(H1N1)
PB1	CY057348	H1N1	11/12/2009	USA	A/San Diego/INS75/2009(H1N1)
PB1	CY089265	H1N1	11/12/2009	USA	A/Boston/650/2009
PB1	CY066869	H1N1	11/12/2009	USA	A/District of Columbia/INS227/2(
PB1	CY073086	H1N1	11/13/2009	Turkey	A/Ankara/WRAIR1435T/2009
PB1	KC780773	H1N1	11/13/2009	USA	A/Arkansas/09/2009
PB1	JX625597	H1N1	11/13/2009	United Ki	A/England/94720002/2009
PB1	CY128377	H1N1	11/13/2009	Viet Nam	A/Viet Nam/11032001/2009
PB1	CY075562	H1N1	11/13/2009	USA	A/Boston/653/2009(H1N1)
PB1	CY087214	H1N1	11/13/2009	USA	A/Texas/JMS368/2009
PB1	CY066877	H1N1	11/13/2009	USA	A/District of Columbia/INS228/2(
PB1	CY073487	H1N1	11/13/2009	Germany	A/Bochum/INS248/2009(H1N1)
PB1	CY062681	H1N1	11/13/2009	Spain	A/Madrid/INS113/2009(H1N1)
PB1	CY060913	H1N1	11/13/2009	USA	A/Texas/JMS369/2009(H1N1)
PB1	HM773208	H1N1	11/13/2009	China	A/Jiangsu/2/2009(H1N1)
PB1	CY066853	H1N1	11/13/2009	Spain	A/Madrid/INS225/2009
PB1	CY075578	H1N1	11/13/2009	USA	A/Boston/658/2009(H1N1)
PB1	CY067021	H1N1	11/13/2009	Germany	A/Bochum/INS249/2009(H1N1)
PB1	CY067029	H1N1	11/13/2009	Germany	A/Bochum/INS250/2009(H1N1)
PB1	CY075570	H1N1	11/13/2009	USA	A/Boston/655/2009(H1N1)
PB1	CY128345	H1N1	11/14/2009	Viet Nam	A/Viet Nam/13032097/2009
PB1	CY060921	H1N1	11/14/2009	USA	A/Texas/JMS370/2009(H1N1)
PB1	CY128425	H1N1	11/14/2009	Viet Nam	A/Viet Nam/12032001/2009
PB1	CY128337	H1N1	11/14/2009	Viet Nam	A/Viet Nam/13032096/2009
PB1	CY089273	H1N1	11/14/2009	USA	A/Boston/657/2009
PB1	CY060929	H1N1	11/14/2009	USA	A/Texas/JMS371/2009(H1N1)
PB1	GU451258	H1N1	11/14/2009	Russia	A/Tver/IIV2969/2009(H1N1)
PB1	CY057828	H1N1	11/14/2009	USA	A/Wisconsin/629-D00965/2009(H1N
PB1	CY057820	H1N1	11/14/2009	USA	A/Wisconsin/629-D01826/2009(H1N
PB1	CY057844	H1N1	11/15/2009	USA	A/Wisconsin/629-D00128/2009(H1N
PB1	CY057868	H1N1	11/15/2009	USA	A/Wisconsin/629-D00936/2009(H1N
PB1	CY089305	H1N1	11/15/2009	USA	A/Boston/671/2009
PB1	CY065121	H1N1	11/15/2009	Netherlar	A/Netherlands/2464b/2009
PB1	CY128473	H1N1	11/15/2009	Viet Nam	A/Viet Nam/001-1020/2009
PB1	CY075602	H1N1	11/15/2009	USA	A/Boston/672/2009(H1N1)
PB1	KT180482	H1N1	11/15/2009	Singapore	A/Singapore/DMS51/2009
PB1	CY066245	H1N1	11/15/2009	USA	A/California/VRDL108/2009(H1N1)
PB1	CY066221	H1N1	11/15/2009	USA	A/California/VRDL105/2009(H1N1)
PB1	CY066229	H1N1	11/15/2009	USA	A/California/VRDL106/2009(H1N1)
PB1	CY057860	H1N1	11/15/2009	USA	A/Wisconsin/629-D00295/2009(H1N
PB1	CY057836	H1N1	11/15/2009	USA	A/Wisconsin/629-D00911/2009(H1N
PB1	CY057852	H1N1	11/15/2009	USA	A/Wisconsin/629-D02060/2009(H1N
PB1	CY066237	H1N1	11/15/2009	USA	A/California/VRDL107/2009(H1N1)
PB1	CY128353	H1N1	11/15/2009	Viet Nam	A/Viet Nam/13032100/2009
PB1	CY060937	H1N1	11/15/2009	USA	A/Texas/JMS372/2009(H1N1)
PB1	CY066213	H1N1	11/15/2009	USA	A/California/VRDL104/2009(H1N1)
PB1	CY056689	H1N1	11/15/2009	USA	A/New York/6257/2009(H1N1)
PB1	CY056681	H1N1	11/16/2009	USA	A/New York/6214/2009(H1N1)
PB1	AB704428	H1N1	11/16/2009	Japan	A/Gunma/263/2009
PB1	HQ698625	H1N1	11/16/2009	China	A/Beijing/16/2009
PB1	CY056673	H1N1	11/16/2009	USA	A/New York/6213/2009(H1N1)
PB1	GU570428	H1N1	11/16/2009	Russia	A/Orenburg/IIV2974/2009(H1N1)
PB1	CY056705	H1N1	11/16/2009	USA	A/New York/6293/2009(H1N1)
PB1	CY057876	H1N1	11/16/2009	USA	A/Wisconsin/629-D01661/2009(H1N

PB1	CY056665	H1N1	11/16/2009	USA	A/New York/6182/2009(H1N1)
PB1	CY067005	H1N1	11/16/2009	Germany	A/Munich/INS246/2009
PB1	CY065137	H1N1	11/16/2009	Netherlar	A/Netherlands/2143/2009
PB1	CY075594	H1N1	11/16/2009	USA	A/Boston/666/2009(H1N1)
PB1	CY089297	H1N1	11/16/2009	USA	A/Boston/670/2009
PB1	CY089281	H1N1	11/16/2009	USA	A/Boston/665/2009
PB1	CY089313	H1N1	11/16/2009	USA	A/Boston/673/2009
PB1	CY089289	H1N1	11/16/2009	USA	A/Boston/667/2009
PB1	CY058338	H1N1	11/16/2009	USA	A/Wisconsin/629-D01347/2009(H1N
PB1	CY075586	H1N1	11/16/2009	USA	A/Boston/663/2009(H1N1)
PB1	KP637404	H1N1	11/16/2009	Thailand	A/Thailand/MR08303/2009
PB1	CY075610	H1N1	11/17/2009	USA	A/Boston/681/2009(H1N1)
PB1	CY062673	H1N1	11/17/2009	USA	A/Pensacola/INS109/2009(H1N1)
PB1	CY066781	H1N1	11/17/2009	USA	A/Pensacola/INS213/2009
PB1	CY056697	H1N1	11/17/2009	USA	A/New York/6292/2009(H1N1)
PB1	CY089337	H1N1	11/17/2009	USA	A/Boston/683/2009
PB1	CY123849	H1N1	11/17/2009	Singapore	A/Singapore/ON2313/2009
PB1	CY069120	H1N1	11/17/2009	Spain	A/Madrid/INS296/2009(H1N1)
PB1	CY061289	H1N1	11/17/2009	USA	A/Pensacola/INS108/2009(H1N1)
PB1	CY089321	H1N1	11/17/2009	USA	A/Boston/678/2009
PB1	CY066725	H1N1	11/17/2009	Spain	A/Madrid/INS295/2009(H1N1)
PB1	CY058346	H1N1	11/17/2009	USA	A/Wisconsin/629-D00503/2009(H1N
PB1	CY058354	H1N1	11/17/2009	USA	A/Wisconsin/629-D01582/2009(H1N
PB1	CY065830	H1N1	11/17/2009	Netherlar	A/Netherlands/2243/2009
PB1	CY089329	H1N1	11/17/2009	USA	A/Boston/680/2009
PB1	KP637804	H1N1	11/17/2009	Thailand	A/Thailand/SS08207/2009
PB1	CY056713	H1N1	11/17/2009	USA	A/New York/6300/2009(H1N1)
PB1	CY060724	H1N1	11/17/2009	Canada	A/Ontario/328474/2009(H1N1)
PB1	CY075618	H1N1	11/17/2009	USA	A/Boston/682/2009(H1N1)
PB1	CY088735	H1N1	11/18/2009	Netherlar	A/Netherlands/2223b/2009
PB1	CY123258	H1N1	11/18/2009	Singapore	A/Singapore/GP4381/2009
PB1	HM567982	H1N1	11/18/2009	United Ki	A/England/869/2009
PB1	CY128361	H1N1	11/18/2009	Viet Nam	A/Viet Nam/15032003/2009
PB1	CY058362	H1N1	11/18/2009	USA	A/Wisconsin/629-D02489/2009(H1N
PB1	CY089345	H1N1	11/18/2009	USA	A/Boston/685/2009
PB1	CY066997	H1N1	11/18/2009	Belgium	A/Brussels/INS245/2009(H1N1)
PB1	CY056721	H1N1	11/18/2009	USA	A/New York/6346/2009(H1N1)
PB1	GU560014	H1N1	11/18/2009	Russia	A/Tomsk/05/2009(H1N1)
PB1	CY067657	H1N1	11/18/2009	Belgium	A/Brussels/INS244/2009(H1N1)
PB1	CY073747	H1N1	11/18/2009	Denmark	A/Copenhagen/INS433/2009(H1N1)
PB1	CY089353	H1N1	11/18/2009	USA	A/Boston/686/2009
PB1	AB704420	H1N1	11/19/2009	Japan	A/Gunma/262/2009
PB1	CY066637	H1N1	11/19/2009	Germany	A/Bonn/INS277/2009(H1N1)
PB1	CY066709	H1N1	11/19/2009	Austria	A/Vienna/INS291/2009(H1N1)
PB1	CY066941	H1N1	11/19/2009	Denmark	A/Aarhus/INS236/2009(H1N1)
PB1	CY062745	H1N1	11/19/2009	Germany	A/Bonn/INS127/2009(H1N1)
PB1	CY063305	H1N1	11/19/2009	USA	A/Wisconsin/629-D00859/2009(H1N
PB1	CY065862	H1N1	11/19/2009	Netherlar	A/Netherlands/2229/2009
PB1	CY063313	H1N1	11/19/2009	USA	A/Wisconsin/629-D01572/2009(H1N
PB1	CY100486	H1N1	11/19/2009	Mexico	A/Mexico City/INER14/2009
PB1	CY071229	H1N1	11/19/2009	Norway	A/Oslo/INS404/2009(H1N1)
PB1	CY062753	H1N1	11/19/2009	Spain	A/Madrid/INS129/2009(H1N1)
PB1	HM568014	H1N1	11/20/2009	United Ki	A/England/872/2009
PB1	CY066685	H1N1	11/20/2009	Denmark	A/Hvidovre/INS288/2009(H1N1)

PB1	CY066645	H1N1	11/20/2009	Germany	A/Bonn/INS278/2009(H1N1)
PB1	HM568030	H1N1	11/20/2009	United Ki	A/England/902/2009
PB1	HM754227	H1N1	11/20/2009	China	A/Jiangsu/1/2009(H1N1)
PB1	CY062801	H1N1	11/20/2009	Denmark	A/Hvidovre/INS141/2009(H1N1)
PB1	HM230699	H1N1	11/20/2009	China	A/Zhoushan/1/2009(H1N1)
PB1	CY063604	H1N1	11/20/2009	Germany	A/Bonn/INS128/2009(H1N1)
PB1	CY066653	H1N1	11/20/2009	Germany	A/Bonn/INS279/2009(H1N1)
PB1	CY056953	H1N1	11/20/2009	Denmark	A/Aarhus/INS82/2009(H1N1)
PB1	CY089361	H1N1	11/20/2009	USA	A/Boston/690/2009
PB1	HM567966	H1N1	11/20/2009	United Ki	A/England/876/2009
PB1	CY083743	H1N1	11/20/2009	Spain	A/Madrid/INS130/2009(H1N1)
PB1	CY067037	H1N1	11/21/2009	Denmark	A/Aarhus/INS251/2009
PB1	CY088591	H1N1	11/21/2009	USA	A/Boston/694/2009
PB1	CY067045	H1N1	11/21/2009	Denmark	A/Aarhus/INS252/2009(H1N1)
PB1	CY060945	H1N1	11/21/2009	USA	A/Texas/JMS373/2009(H1N1)
PB1	CY089369	H1N1	11/21/2009	USA	A/Boston/691/2009
PB1	AB704508	H1N1	11/21/2009	Japan	A/Yamaguchi/217/2009
PB1	CY057900	H1N1	11/22/2009	USA	A/Wisconsin/629-D00908/2009(H1N
PB1	CY067053	H1N1	11/22/2009	Denmark	A/Aarhus/INS253/2009(H1N1)
PB1	CY128369	H1N1	11/22/2009	Viet Nam	A/Viet Nam/15032004/2009
PB1	CY056729	H1N1	11/22/2009	USA	A/New York/6473/2009(H1N1)
PB1	GU560022	H1N1	11/22/2009	Russia	A/Tomsk/06/2009(H1N1)
PB1	CY084460	H1N1	11/22/2009	USA	A/New York/6418/2009
PB1	CY057892	H1N1	11/22/2009	USA	A/Wisconsin/629-D00533/2009(H1N
PB1	CY072252	H1N1	11/23/2009	Denmark	A/Odense/INS307/2009(H1N1)
PB1	HM568006	H1N1	11/23/2009	United Ki	A/England/854/2009
PB1	CY057916	H1N1	11/23/2009	USA	A/Wisconsin/629-D00134/2009(H1N
PB1	HM567958	H1N1	11/23/2009	United Ki	A/England/865/2009
PB1	CY058722	H1N1	11/23/2009	USA	A/Texas/JMS375/2009(H1N1)
PB1	CY056849	H1N1	11/23/2009	USA	A/New York/6868/2009(H1N1)
PB1	CY075626	H1N1	11/23/2009	USA	A/Boston/698/2009(H1N1)
PB1	CY066893	H1N1	11/23/2009	USA	A/District of Columbia/INS230/2(
PB1	CY057908	H1N1	11/23/2009	USA	A/Wisconsin/629-D02448/2009(H1N
PB1	CY057924	H1N1	11/23/2009	USA	A/Wisconsin/629-D01330/2009(H1N
PB1	CY066949	H1N1	11/23/2009	Denmark	A/Aarhus/INS237/2009(H1N1)
PB1	CY065806	H1N1	11/23/2009	Netherlar	A/Netherlands/2445b/2009
PB1	CY056761	H1N1	11/23/2009	USA	A/New York/6663/2009(H1N1)
PB1	CY056737	H1N1	11/23/2009	USA	A/New York/6546/2009(H1N1)
PB1	HM568022	H1N1	11/23/2009	United Ki	A/England/862/2009
PB1	CY066909	H1N1	11/23/2009	USA	A/District of Columbia/INS232/2(
PB1	CY069152	H1N1	11/23/2009	Germany	A/Frankfurt/INS301/2009(H1N1)
PB1	CY083916	H1N1	11/23/2009	Denmark	A/Aalborg/INS132/2009(H1N1)
PB1	CY056753	H1N1	11/24/2009	USA	A/New York/6607/2009(H1N1)
PB1	CY084468	H1N1	11/24/2009	USA	A/New York/6537/2009
PB1	KC780705	H1N1	11/24/2009	USA	A/California/41/2009
PB1	CY072260	H1N1	11/24/2009	Denmark	A/Odense/INS308/2009(H1N1)
PB1	CY066957	H1N1	11/24/2009	Denmark	A/Aarhus/INS238/2009(H1N1)
PB1	CY066269	H1N1	11/24/2009	USA	A/California/VRDL111/2009(H1N1)
PB1	HQ263293	H1N1	11/24/2009	USA	A/Bethesda/NIH107-D31/2009
PB1	CY057940	H1N1	11/24/2009	USA	A/Wisconsin/629-D00968/2009(H1N
PB1	CY066661	H1N1	11/24/2009	Spain	A/Madrid/INS281/2009(H1N1)
PB1	CY057932	H1N1	11/24/2009	USA	A/Wisconsin/629-D01190/2009(H1N
PB1	CY066285	H1N1	11/24/2009	USA	A/California/VRDL113/2009(H1N1)
PB1	CY066261	H1N1	11/24/2009	USA	A/California/VRDL110/2009(H1N1)

PB1	CY066277	H1N1	11/24/2009	USA	A/California/VRDL112/2009(H1N1)
PB1	CY066253	H1N1	11/24/2009	USA	A/California/VRDL109/2009(H1N1)
PB1	CY062697	H1N1	11/24/2009	Denmark	A/Aarhus/INS116/2009(H1N1)
PB1	CY058730	H1N1	11/24/2009	USA	A/Texas/JMS376/2009(H1N1)
PB1	CY066717	H1N1	11/24/2009	Austria	A/Vienna/INS293/2009(H1N1)
PB1	CY092444	H1N1	11/24/2009	Australia	A/Sydney/DD3-02/2009(H1N1)
PB1	HM567998	H1N1	11/24/2009	United Ki	A/England/900/2009
PB1	CY071253	H1N1	11/24/2009	Spain	A/Madrid/INS407/2009(H1N1)
PB1	CY066917	H1N1	11/24/2009	USA	A/Pensacola/INS233/2009
PB1	CY066981	H1N1	11/24/2009	Denmark	A/Aarhus/INS242/2009(H1N1)
PB1	CY069128	H1N1	11/24/2009	Spain	A/Madrid/INS298/2009(H1N1)
PB1	KT180484	H1N1	11/24/2009	Singapore	A/Singapore/DMS53/2009
PB1	AB704468	H1N1	11/24/2009	Japan	A/Tochigi/445/2009
PB1	CY056785	H1N1	11/24/2009	USA	A/New York/6675/2009(H1N1)
PB1	JQ173162	H1N1	11/25/2009	Finland	A/Helsinki/P18/2009(H1N1)
PB1	CY056833	H1N1	11/25/2009	USA	A/New York/6846/2009(H1N1)
PB1	CY056825	H1N1	11/25/2009	USA	A/New York/6810/2009(H1N1)
PB1	CY072228	H1N1	11/25/2009	Germany	A/Frankfurt/INS302/2009(H1N1)
PB1	GU562456	H1N1	11/25/2009	Russia	A/Perm/01/2009(H1N1)
PB1	HM189318	H1N1	11/25/2009	Russia	A/Arkhangelsk/CRIE-GNY/2009
PB1	HM567950	H1N1	11/25/2009	United Ki	A/England/857/2009
PB1	AB704444	H1N1	11/25/2009	Japan	A/Gunma/287/2009
PB1	HM567990	H1N1	11/25/2009	United Ki	A/England/859/2009
PB1	CY066693	H1N1	11/25/2009	Denmark	A/Hvidovre/INS289/2009(H1N1)
PB1	CY067189	H1N1	11/25/2009	Austria	A/Vienna/INS294/2009
PB1	CY067013	H1N1	11/25/2009	Germany	A/Munich/INS247/2009
PB1	HM568038	H1N1	11/25/2009	United Ki	A/England/898/2009
PB1	AB704436	H1N1	11/25/2009	Japan	A/Gunma/267/2009
PB1	HM101149	H1N1	11/25/2009	Russia	A/Tver/IIV-183/2009(H1N1)
PB1	CY058746	H1N1	11/26/2009	USA	A/Texas/JMS378/2009(H1N1)
PB1	KT180485	H1N1	11/26/2009	Singapore	A/Singapore/DMS54/2009
PB1	CY088619	H1N1	11/26/2009	Germany	A/Frankfurt/INS93/2009
PB1	CY058738	H1N1	11/26/2009	USA	A/Texas/JMS377/2009(H1N1)
PB1	CY060953	H1N1	11/26/2009	USA	A/Texas/JMS379/2009(H1N1)
PB1	CY066973	H1N1	11/26/2009	Denmark	A/Aarhus/INS241/2009(H1N1)
PB1	CY062809	H1N1	11/26/2009	Austria	A/Vienna/INS142/2009(H1N1)
PB1	CY066965	H1N1	11/26/2009	Denmark	A/Aarhus/INS240/2009(H1N1)
PB1	CY056745	H1N1	11/26/2009	USA	A/New York/6571/2009(H1N1)
PB1	CY069136	H1N1	11/26/2009	Spain	A/Madrid/INS299/2009(H1N1)
PB1	CY073755	H1N1	11/26/2009	Denmark	A/Copenhagen/INS434/2009(H1N1)
PB1	CY072236	H1N1	11/26/2009	Germany	A/Frankfurt/INS303/2009(H1N1)
PB1	CY057948	H1N1	11/26/2009	USA	A/Wisconsin/629-D00629/2009(H1N
PB1	CY062761	H1N1	11/26/2009	Spain	A/Madrid/INS131/2009(H1N1)
PB1	CY075634	H1N1	11/27/2009	USA	A/Boston/701/2009(H1N1)
PB1	CY060961	H1N1	11/27/2009	USA	A/Texas/JMS380/2009(H1N1)
PB1	CY057956	H1N1	11/27/2009	USA	A/Wisconsin/629-D00050/2009(H1N
PB1	CY066701	H1N1	11/27/2009	Denmark	A/Hvidovre/INS290/2009(H1N1)
PB1	AB704500	H1N1	11/27/2009	Japan	A/Yamagata/674/2009
PB1	CY057964	H1N1	11/27/2009	USA	A/Wisconsin/629-D00970/2009(H1N
PB1	CY062705	H1N1	11/27/2009	Denmark	A/Aarhus/INS118/2009(H1N1)
PB1	KJ856065	H1N1	11/27/2009	Sri Lanka	A/Sri Lanka/2005/2009
PB1	GU592903	H1N1	11/28/2009	Kyrgyzsta	A/Bishkek/03/2009(H1N1)
PB1	CY060977	H1N1	11/28/2009	USA	A/Texas/JMS382/2009(H1N1)
PB1	CY060969	H1N1	11/28/2009	USA	A/Texas/JMS381/2009(H1N1)

PB1	CY067061	H1N1	11/28/2009	Denmark	A/Aarhus/INS254/2009(H1N1)
PB1	CY088599	H1N1	11/29/2009	USA	A/Boston/702/2009
PB1	CY056809	H1N1	11/29/2009	USA	A/New York/6808/2009(H1N1)
PB1	CY057972	H1N1	11/29/2009	USA	A/Wisconsin/629-D01528/2009(H1N
PB1	CY057980	H1N1	11/29/2009	USA	A/Wisconsin/629-D01434/2009(H1N
PB1	CY056857	H1N1	11/29/2009	USA	A/New York/6909/2009(H1N1)
PB1	CY060985	H1N1	11/29/2009	USA	A/Texas/JMS383/2009(H1N1)
PB1	CY060993	H1N1	11/30/2009	USA	A/Texas/JMS384/2009(H1N1)
PB1	CY069144	H1N1	11/30/2009	Spain	A/Madrid/INS300/2009(H1N1)
PB1	CY066669	H1N1	11/30/2009	Germany	A/Cologne/INS286/2009(H1N1)
PB1	KT180487	H1N1	11/30/2009	Singapore	A/Singapore/DMS55/2009
PB1	CY069224	H1N1	11/30/2009	Germany	A/Munich/INS363/2009(H1N1)
PB1	CY056769	H1N1	11/30/2009	USA	A/New York/6668/2009(H1N1)
PB1	CY072244	H1N1	11/30/2009	Germany	A/Frankfurt/INS305/2009(H1N1)
PB1	CY056817	H1N1	11/30/2009	USA	A/New York/6809/2009(H1N1)
PB1	CY063620	H1N1	11/30/2009	Germany	A/Cologne/INS136/2009(H1N1)
PB1	KJ856072	H1N1	11/30/2009	Sri Lanka	A/Sri Lanka/2008/2009
PB1	CY057988	H1N1	12/01/2009	USA	A/Wisconsin/629-D01412/2009(H1N
PB1	KJ856080	H1N1	12/01/2009	Sri Lanka	A/Sri Lanka/2009/2009
PB1	KJ856236	H1N1	12/01/2009	Sri Lanka	A/Sri Lanka/24588/2009
PB1	CY073503	H1N1	12/01/2009	Germany	A/Frankfurt/INS304/2009(H1N1)
PB1	CY080270	H1N1	12/01/2009	Mongolia	A/Dundgobi/9746/2009
PB1	KC780184	H1N1	12/01/2009	USA	A/Montana/22/2009
PB1	CY123292	H1N1	12/01/2009	Singapore	A/Singapore/GP4556/2009
PB1	CY056777	H1N1	12/01/2009	USA	A/New York/6669/2009(H1N1)
PB1	CY065846	H1N1	12/01/2009	Netherlar	A/Netherlands/2636/2009
PB1	AB704516	H1N1	12/02/2009	Japan	A/Yamaguchi/247/2009
PB1	CY056841	H1N1	12/02/2009	USA	A/New York/6864/2009(H1N1)
PB1	CY058004	H1N1	12/02/2009	USA	A/Wisconsin/629-D02299/2009(H1N
PB1	CY063612	H1N1	12/02/2009	Denmark	A/Aalborg/INS133/2009(H1N1)
PB1	CY062769	H1N1	12/02/2009	Spain	A/Madrid/INS134/2009(H1N1)
PB1	CY071197	H1N1	12/02/2009	Germany	A/Frankfurt/INS399/2009(H1N1)
PB1	CY072276	H1N1	12/02/2009	Poland	A/Warsaw/INS311/2009(H1N1)
PB1	CY057996	H1N1	12/02/2009	USA	A/Wisconsin/629-D00935/2009(H1N
PB1	CY065902	H1N1	12/02/2009	Netherlar	A/Netherlands/2822/2009
PB1	CY071093	H1N1	12/02/2009	Germany	A/Bochum/INS376/2009(H1N1)
PB1	CY056985	H1N1	12/02/2009	Denmark	A/Copenhagen/INS98/2009(H1N1)
PB1	CY123298	H1N1	12/02/2009	Singapore	A/Singapore/GP4581/2009
PB1	CY072268	H1N1	12/02/2009	Poland	A/Warsaw/INS310/2009(H1N1)
PB1	CY071085	H1N1	12/02/2009	Germany	A/Bochum/INS375/2009(H1N1)
PB1	HM568118	H1N1	12/02/2009	United Ki	A/England/941/2009
PB1	CY075642	H1N1	12/02/2009	USA	A/Boston/703/2009(H1N1)
PB1	CY128433	H1N1	12/02/2009	Viet Nam	A/Viet Nam/12032005/2009
PB1	CY123877	H1N1	12/03/2009	Singapore	A/Singapore/ON2383/2009
PB1	CY083924	H1N1	12/03/2009	Poland	A/Warsaw/INS147/2009(H1N1)
PB1	CY072292	H1N1	12/03/2009	Poland	A/Warsaw/INS313/2009(H1N1)
PB1	CY075650	H1N1	12/03/2009	USA	A/Boston/704/2009(H1N1)
PB1	CY072284	H1N1	12/03/2009	Poland	A/Warsaw/INS312/2009(H1N1)
PB1	CY062833	H1N1	12/03/2009	Poland	A/Warsaw/INS146/2009(H1N1)
PB1	KT180488	H1N1	12/03/2009	Singapore	A/Singapore/DMS56/2009
PB1	CY066309	H1N1	12/04/2009	USA	A/California/VRDL116/2009(H1N1)
PB1	CY062777	H1N1	12/04/2009	Spain	A/Madrid/INS135/2009(H1N1)
PB1	CY083557	H1N1	12/04/2009	Hungary	A/Budapest/WRAIR2411N/2009
PB1	CY065790	H1N1	12/04/2009	Netherlar	A/Netherlands/2629/2009

PB1	CY066293	H1N1	12/04/2009	USA	A/California/VRDL114/2009(H1N1)
PB1	CY066317	H1N1	12/04/2009	USA	A/California/VRDL117/2009(H1N1)
PB1	JN187222	H1N1	12/04/2009	Taiwan	A/Taiwan/6341/2009
PB1	CY057356	H1N1	12/04/2009	Germany	A/Hamburg/INS92/2009(H1N1)
PB1	CY066301	H1N1	12/04/2009	USA	A/California/VRDL115/2009(H1N1)
PB1	HM157256	H1N1	12/04/2009	Russia	A/Yaroslavl/IIV-196/2009(H1N1)
PB1	CY056801	H1N1	12/04/2009	USA	A/New York/6776/2009(H1N1)
PB1	CY066325	H1N1	12/04/2009	USA	A/California/VRDL118/2009(H1N1)
PB1	CY061001	H1N1	12/04/2009	USA	A/Texas/JMS385/2009(H1N1)
PB1	GU727829	H1N1	12/04/2009	Russia	A/IIV-Anadyr/177/2009(H1N1)
PB1	CY069232	H1N1	12/04/2009	Germany	A/Munich/INS364/2009(H1N1)
PB1	KC780775	H1N1	12/04/2009	USA	A/Alaska/47/2009
PB1	CY088607	H1N1	12/04/2009	USA	A/Boston/705/2009
PB1	KC780300	H1N1	12/04/2009	USA	A/Pennsylvania/31/2009
PB1	HM104483	H1N1	12/05/2009	Russia	A/Yaroslavl/IIV-198/2009(H1N1)
PB1	CY056793	H1N1	12/05/2009	USA	A/New York/6775/2009(H1N1)
PB1	AB704524	H1N1	12/05/2009	Japan	A/Yamaguchi/248/2009
PB1	CY065081	H1N1	12/06/2009	USA	A/New York/7421/2009(H1N1)
PB1	CY061009	H1N1	12/06/2009	USA	A/Texas/JMS386/2009(H1N1)
PB1	CY084452	H1N1	12/06/2009	USA	A/New York/6902/2009
PB1	CY061944	H1N1	12/06/2009	USA	A/New York/6941/2009(H1N1)
PB1	KC781015	H1N1	12/06/2009	USA	A/Arizona/20/2009
PB1	CY072300	H1N1	12/07/2009	Poland	A/Warsaw/INS314/2009(H1N1)
PB1	CY056993	H1N1	12/07/2009	Poland	A/Warsaw/INS100/2009(H1N1)
PB1	CY058402	H1N1	12/07/2009	USA	A/Wisconsin/629-D01591/2009(H1N
PB1	CY073731	H1N1	12/07/2009	Denmark	A/Aalborg/INS283/2009(H1N1)
PB1	KC781090	H1N1	12/07/2009	USA	A/Iowa/14/2009
PB1	CY061225	H1N1	12/07/2009	USA	A/New York/6772/2009(H1N1)
PB1	CY065894	H1N1	12/07/2009	Netherlar	A/Netherlands/2815/2009
PB1	CY061952	H1N1	12/07/2009	USA	A/New York/6943/2009(H1N1)
PB1	CY063628	H1N1	12/07/2009	USA	A/New York/INS150/2009(H1N1)
PB1	CY072308	H1N1	12/07/2009	Poland	A/Warsaw/INS315/2009(H1N1)
PB1	CY056969	H1N1	12/07/2009	Denmark	A/Copenhagen/INS95/2009(H1N1)
PB1	CY058012	H1N1	12/07/2009	USA	A/Wisconsin/629-D01473/2009(H1N
PB1	KC780596	H1N1	12/07/2009	USA	A/Texas/75/2009
PB1	KT180490	H1N1	12/07/2009	Singapore	A/Singapore/DMS57/2009
PB1	CY061600	H1N1	12/07/2009	USA	A/New York/6806/2009(H1N1)
PB1	CY062825	H1N1	12/07/2009	Denmark	A/Copenhagen/INS144/2009(H1N1)
PB1	CY061217	H1N1	12/07/2009	USA	A/New York/6771/2009(H1N1)
PB1	LN845780	H1N1	12/08/2009	United Ki	A/England/8/2009
PB1	CY067173	H1N1	12/08/2009	Germany	A/Bonn/INS280/2009(H1N1)
PB1	KC780843	H1N1	12/08/2009	USA	A/Oregon/42/2009
PB1	CY065105	H1N1	12/08/2009	USA	A/New York/7426/2009(H1N1)
PB1	CY058028	H1N1	12/08/2009	USA	A/Wisconsin/629-D00401/2009(H1N
PB1	LN846548	H1N1	12/08/2009	United Ki	A/England/9/2009
PB1	CY066925	H1N1	12/08/2009	USA	A/Pensacola/INS234/2009
PB1	CY061017	H1N1	12/08/2009	USA	A/Texas/JMS387/2009(H1N1)
PB1	CY072316	H1N1	12/08/2009	Poland	A/Warsaw/INS316/2009(H1N1)
PB1	CY058386	H1N1	12/08/2009	USA	A/Wisconsin/629-D00780/2009(H1N
PB1	CY058020	H1N1	12/08/2009	USA	A/Wisconsin/629-D00672/2009(H1N
PB1	CY061920	H1N1	12/08/2009	USA	A/New York/6907/2009(H1N1)
PB1	CY066933	H1N1	12/08/2009	USA	A/Pensacola/INS235/2009
PB1	CY072548	H1N1	12/09/2009	Poland	A/Wroclaw/INS436/2009(H1N1)
PB1	CY069216	H1N1	12/09/2009	Germany	A/Berlin/INS361/2009(H1N1)

PB1	CY069240	H1N1	12/09/2009	Germany	A/Munich/INS365/2009(H1N1)
PB1	JF800143	H1N1	12/09/2009	China	A/Nanchang/8002/2009
PB1	CY128385	H1N1	12/09/2009	Viet Nam	A/Viet Nam/11032006/2009
PB1	CY058394	H1N1	12/09/2009	USA	A/Wisconsin/629-D00147/2009(H1N
PB1	CY067181	H1N1	12/09/2009	Germany	A/Bonn/INS284/2009(H1N1)
PB1	CY063596	H1N1	12/09/2009	Greece	A/Athens/INS122/2009(H1N1)
PB1	CY072556	H1N1	12/09/2009	Poland	A/Wroclaw/INS437/2009(H1N1)
PB1	CY058370	H1N1	12/09/2009	USA	A/Wisconsin/629-D00689/2009(H1N
PB1	CY061912	H1N1	12/10/2009	USA	A/New York/6903/2009(H1N1)
PB1	CY065993	H1N1	12/10/2009	Mongolia	A/Ulgii/9911/2009(H1N1)
PB1	CY056977	H1N1	12/10/2009	Denmark	A/Copenhagen/INS96/2009(H1N1)
PB1	KT180491	H1N1	12/10/2009	Singapore	A/Singapore/DMS58/2009
PB1	CY058036	H1N1	12/10/2009	USA	A/Wisconsin/629-D00636/2009(H1N
PB1	CY062785	H1N1	12/10/2009	Belgium	A/Tessenderlo/INS138/2009(H1N1)
PB1	CY066677	H1N1	12/10/2009	Belgium	A/Tessenderlo/INS287/2009(H1N1)
PB1	CY063049	H1N1	12/10/2009	Germany	A/Bochum/INS187/2009(H1N1)
PB1	CY064778	H1N1	12/11/2009	Germany	A/Berlin/INS170/2009(H1N1)
PB1	CY071101	H1N1	12/11/2009	Spain	A/Barcelona/INS379/2009(H1N1)
PB1	CY123887	H1N1	12/11/2009	Singapore	A/Singapore/ON2400/2009
PB1	CY061984	H1N1	12/11/2009	USA	A/New York/6977/2009(H1N1)
PB1	CY061928	H1N1	12/11/2009	USA	A/New York/6937/2009(H1N1)
PB1	CY061936	H1N1	12/11/2009	USA	A/New York/6939/2009(H1N1)
PB1	CY083932	H1N1	12/11/2009	Poland	A/Warsaw/INS148/2009(H1N1)
PB1	CY083940	H1N1	12/11/2009	Poland	A/Warsaw/INS149/2009(H1N1)
PB1	CY061025	H1N1	12/11/2009	USA	A/Texas/JMS388/2009(H1N1)
PB1	CY061960	H1N1	12/11/2009	USA	A/New York/6945/2009(H1N1)
PB1	CY063636	H1N1	12/11/2009	USA	A/New York/INS151/2009(H1N1)
PB1	CY058378	H1N1	12/12/2009	USA	A/Wisconsin/629-D01244/2009(H1N
PB1	CY083803	H1N1	12/12/2009	Germany	A/Berlin/INS362/2009(H1N1)
PB1	CY067069	H1N1	12/13/2009	Greece	A/Athens/INS257/2009(H1N1)
PB1	CY067093	H1N1	12/13/2009	Greece	A/Athens/INS261/2009(H1N1)
PB1	CY067085	H1N1	12/13/2009	Greece	A/Athens/INS260/2009(H1N1)
PB1	CY083908	H1N1	12/13/2009	Greece	A/Athens/INS86/2009(H1N1)
PB1	CY067077	H1N1	12/13/2009	Greece	A/Athens/INS259/2009(H1N1)
PB1	CY056961	H1N1	12/13/2009	Greece	A/Athens/INS85/2009(H1N1)
PB1	CY062721	H1N1	12/13/2009	Greece	A/Athens/INS124/2009(H1N1)
PB1	CY067125	H1N1	12/13/2009	Greece	A/Athens/INS266/2009(H1N1)
PB1	CY067117	H1N1	12/13/2009	Greece	A/Athens/INS264/2009(H1N1)
PB1	CY072356	H1N1	12/13/2009	Greece	A/Athens/INS322/2009(H1N1)
PB1	CY062713	H1N1	12/13/2009	Greece	A/Athens/INS123/2009(H1N1)
PB1	CY062729	H1N1	12/13/2009	Greece	A/Athens/INS125/2009(H1N1)
PB1	CY067101	H1N1	12/13/2009	Greece	A/Athens/INS262/2009(H1N1)
PB1	CY067109	H1N1	12/13/2009	Greece	A/Athens/INS263/2009(H1N1)
PB1	CY072348	H1N1	12/13/2009	Greece	A/Athens/INS320/2009(H1N1)
PB1	CY058426	H1N1	12/14/2009	USA	A/Wisconsin/629-D01904/2009(H1N
PB1	CY061968	H1N1	12/14/2009	USA	A/New York/6949/2009(H1N1)
PB1	CY061976	H1N1	12/14/2009	USA	A/New York/6976/2009(H1N1)
PB1	CY062000	H1N1	12/14/2009	USA	A/New York/7020/2009(H1N1)
PB1	KT180492	H1N1	12/14/2009	Singapore	A/Singapore/DMS59/2009
PB1	KC683493	H1N1	12/14/2009	China	A/Liaoning/14/2009
PB1	GU592911	H1N1	12/14/2009	Russia	A/Magadan/02/2009(H1N1)
PB1	CY058458	H1N1	12/14/2009	USA	A/Wisconsin/629-D01846/2009(H1N
PB1	CY067157	H1N1	12/15/2009	Greece	A/Athens/INS270/2009(H1N1)
PB1	CY066357	H1N1	12/15/2009	USA	A/California/VRDL122/2009(H1N1)

PB1	CY058474	H1N1	12/15/2009	USA	A/Wisconsin/629-D00337/2009(H1N
PB1	CY058482	H1N1	12/15/2009	USA	A/Wisconsin/629-D02424/2009(H1N
PB1	CY066605	H1N1	12/15/2009	Greece	A/Athens/INS272/2009(H1N1)
PB1	CY066333	H1N1	12/15/2009	USA	A/California/VRDL119/2009(H1N1)
PB1	CY062665	H1N1	12/15/2009	Greece	A/Athens/INS87/2009(H1N1)
PB1	CY066613	H1N1	12/15/2009	Greece	A/Athens/INS273/2009(H1N1)
PB1	CY063057	H1N1	12/15/2009	Belgium	A/Tessenderlo/INS191/2009(H1N1)
PB1	CY066629	H1N1	12/15/2009	Greece	A/Athens/INS275/2009(H1N1)
PB1	CY058434	H1N1	12/15/2009	USA	A/Wisconsin/629-D01752/2009(H1N
PB1	CY066365	H1N1	12/15/2009	USA	A/California/VRDL123/2009(H1N1)
PB1	CY066349	H1N1	12/15/2009	USA	A/California/VRDL121/2009(H1N1)
PB1	CY063025	H1N1	12/15/2009	Spain	A/Madrid/INS184/2009(H1N1)
PB1	CY062737	H1N1	12/15/2009	Greece	A/Athens/INS126/2009(H1N1)
PB1	CY066621	H1N1	12/15/2009	Greece	A/Athens/INS274/2009(H1N1)
PB1	AB704452	H1N1	12/15/2009	Japan	A/Gunma/293/2009
PB1	CY073883	H1N1	12/15/2009	Greece	A/Athens/INS323/2009(H1N1)
PB1	CY067141	H1N1	12/15/2009	Greece	A/Athens/INS268/2009(H1N1)
PB1	CY123893	H1N1	12/15/2009	Singapore	A/Singapore/ON2416/2009
PB1	CY073495	H1N1	12/15/2009	Greece	A/Athens/INS271/2009(H1N1)
PB1	CY066341	H1N1	12/15/2009	USA	A/California/VRDL120/2009(H1N1)
PB1	CY072332	H1N1	12/15/2009	USA	A/Newark/INS318/2009(H1N1)
PB1	CY067149	H1N1	12/15/2009	Greece	A/Athens/INS269/2009(H1N1)
PB1	CY071109	H1N1	12/16/2009	Belgium	A/Tessenderlo/INS380/2009(H1N1)
PB1	CY058466	H1N1	12/16/2009	USA	A/Wisconsin/629-D00996/2009(H1N
PB1	CY061992	H1N1	12/16/2009	USA	A/New York/7019/2009(H1N1)
PB1	CY058410	H1N1	12/16/2009	USA	A/Wisconsin/629-D01038/2009(H1N
PB1	CY058450	H1N1	12/16/2009	USA	A/Wisconsin/629-D02073/2009(H1N
PB1	CY061033	H1N1	12/16/2009	USA	A/Texas/JMS389/2009(H1N1)
PB1	CY062945	H1N1	12/16/2009	Spain	A/Terrassa/INS172/2009(H1N1)
PB1	CY067133	H1N1	12/16/2009	Greece	A/Athens/INS267/2009(H1N1)
PB1	CY062008	H1N1	12/16/2009	USA	A/New York/7036/2009(H1N1)
PB1	KT180493	H1N1	12/16/2009	Singapore	A/Singapore/DMS60/2009
PB1	CY071261	H1N1	12/16/2009	Spain	A/Madrid/INS408/2009(H1N1)
PB1	CY062857	H1N1	12/17/2009	Greece	A/Athens/INS158/2009(H1N1)
PB1	CY128393	H1N1	12/17/2009	Viet Nam	A/Viet Nam/11032010/2009
PB1	CY058442	H1N1	12/17/2009	USA	A/Wisconsin/629-D01556/2009(H1N
PB1	CY072476	H1N1	12/17/2009	Greece	A/Athens/INS342/2009(H1N1)
PB1	CY072460	H1N1	12/17/2009	Greece	A/Athens/INS340/2009(H1N1)
PB1	CY072324	H1N1	12/17/2009	USA	A/New York/INS317/2009(H1N1)
PB1	CY067165	H1N1	12/17/2009	Greece	A/Athens/INS276/2009(H1N1)
PB1	CY072468	H1N1	12/17/2009	Greece	A/Athens/INS341/2009(H1N1)
PB1	CY062953	H1N1	12/17/2009	Spain	A/Terrassa/INS173/2009(H1N1)
PB1	CY058418	H1N1	12/18/2009	USA	A/Wisconsin/629-D00459/2009(H1N
PB1	JN187223	H1N1	12/18/2009	Taiwan	A/Taiwan/11706/2009
PB1	AB704532	H1N1	12/18/2009	Japan	A/Yamaguchi/273/2009
PB1	GU592895	H1N1	12/18/2009	Russia	A/Barnaul/04/2009(H1N1)
PB1	CY062873	H1N1	12/18/2009	Greece	A/Athens/INS160/2009(H1N1)
PB1	CY062937	H1N1	12/18/2009	Germany	A/Berlin/INS171/2009(H1N1)
PB1	CY072484	H1N1	12/18/2009	Greece	A/Athens/INS344/2009(H1N1)
PB1	CY063660	H1N1	12/18/2009	Greece	A/Athens/INS157/2009(H1N1)
PB1	CY072492	H1N1	12/18/2009	Greece	A/Athens/INS345/2009(H1N1)
PB1	CY062865	H1N1	12/18/2009	Greece	A/Athens/INS159/2009(H1N1)
PB1	CY089614	H1N1	12/19/2009	China	A/Nanchang/8008/2009
PB1	HM230707	H1N1	12/19/2009	China	A/Zhoushan/52/2009(H1N1)

PB1	CY062881	H1N1	12/19/2009	Greece	A/Athens/INS161/2009(H1N1)
PB1	CY072508	H1N1	12/20/2009	Greece	A/Athens/INS347/2009(H1N1)
PB1	CY062889	H1N1	12/20/2009	Greece	A/Athens/INS162/2009(H1N1)
PB1	CY062897	H1N1	12/20/2009	Greece	A/Athens/INS163/2009(H1N1)
PB1	CY073511	H1N1	12/20/2009	Greece	A/Athens/INS350/2009(H1N1)
PB1	CY069160	H1N1	12/20/2009	Greece	A/Athens/INS352/2009(H1N1)
PB1	CY065097	H1N1	12/20/2009	USA	A/New York/7425/2009(H1N1)
PB1	CY072364	H1N1	12/20/2009	Greece	A/Athens/INS325/2009(H1N1)
PB1	CY061041	H1N1	12/20/2009	USA	A/Texas/JMS390/2009(H1N1)
PB1	CY062905	H1N1	12/20/2009	Greece	A/Athens/INS164/2009(H1N1)
PB1	CY072524	H1N1	12/20/2009	Greece	A/Athens/INS349/2009(H1N1)
PB1	CY072372	H1N1	12/20/2009	Greece	A/Athens/INS327/2009(H1N1)
PB1	CY072500	H1N1	12/20/2009	Greece	A/Athens/INS346/2009(H1N1)
PB1	CY072516	H1N1	12/20/2009	Greece	A/Athens/INS348/2009(H1N1)
PB1	CY062024	H1N1	12/21/2009	USA	A/New York/7216/2009(H1N1)
PB1	CY069176	H1N1	12/21/2009	Greece	A/Athens/INS354/2009(H1N1)
PB1	KT180494	H1N1	12/21/2009	Singapore	A/Singapore/DMS61/2009
PB1	CY069168	H1N1	12/21/2009	Greece	A/Athens/INS353/2009(H1N1)
PB1	CY063001	H1N1	12/21/2009	Estonia	A/Tallinn/INS181/2009(H1N1)
PB1	CY088627	H1N1	12/21/2009	Denmark	A/Odense/INS176/2009
PB1	CY062913	H1N1	12/21/2009	Greece	A/Athens/INS165/2009(H1N1)
PB1	CY071117	H1N1	12/22/2009	Germany	A/Wurzburg/INS381/2009(H1N1)
PB1	CY066389	H1N1	12/22/2009	USA	A/California/VRDL126/2009(H1N1)
PB1	CY066373	H1N1	12/22/2009	USA	A/California/VRDL124/2009(H1N1)
PB1	CY089622	H1N1	12/22/2009	China	A/Nanchang/8011/2009
PB1	CY069248	H1N1	12/22/2009	Austria	A/Vienna/INS366/2009(H1N1)
PB1	CY069184	H1N1	12/22/2009	Greece	A/Athens/INS355/2009(H1N1)
PB1	CY066381	H1N1	12/22/2009	USA	A/California/VRDL125/2009(H1N1)
PB1	CY066405	H1N1	12/22/2009	USA	A/California/VRDL128/2009(H1N1)
PB1	CY066397	H1N1	12/22/2009	USA	A/California/VRDL127/2009(H1N1)
PB1	CY071205	H1N1	12/23/2009	Germany	A/Frankfurt/INS401/2009(H1N1)
PB1	CY062016	H1N1	12/23/2009	USA	A/New York/7107/2009(H1N1)
PB1	CY071069	H1N1	12/23/2009	Estonia	A/Tallinn/INS372/2009(H1N1)
PB1	CY128401	H1N1	12/23/2009	Viet Nam	A/Viet Nam/11032016/2009
PB1	CY061049	H1N1	12/23/2009	USA	A/Texas/JMS391/2009(H1N1)
PB1	CY062969	H1N1	12/23/2009	Spain	A/Terrassa/INS175/2009(H1N1)
PB1	CY128409	H1N1	12/23/2009	Viet Nam	A/Viet Nam/11032017/2009
PB1	LN867650	H1N1	12/23/2009	United Ki	A/England/01220639/2009
PB1	CY065089	H1N1	12/23/2009	USA	A/New York/7424/2009(H1N1)
PB1	CY062961	H1N1	12/24/2009	Spain	A/Terrassa/INS174/2009(H1N1)
PB1	KT180497	H1N1	12/25/2009	Singapore	A/Singapore/DMS62/2009
PB1	CY072380	H1N1	12/25/2009	Greece	A/Athens/INS329/2009(H1N1)
PB1	CY061057	H1N1	12/25/2009	USA	A/Texas/JMS392/2009(H1N1)
PB1	CY071125	H1N1	12/25/2009	Germany	A/Wurzburg/INS382/2009(H1N1)
PB1	CY062040	H1N1	12/26/2009	USA	A/New York/0259/2009(H1N1)
PB1	CY069208	H1N1	12/26/2009	Greece	A/Athens/INS359/2009(H1N1)
PB1	CY072396	H1N1	12/26/2009	Greece	A/Athens/INS332/2009(H1N1)
PB1	CY072388	H1N1	12/26/2009	Greece	A/Athens/INS331/2009(H1N1)
PB1	CY069200	H1N1	12/26/2009	Greece	A/Athens/INS358/2009(H1N1)
PB1	CY069192	H1N1	12/26/2009	Greece	A/Athens/INS357/2009(H1N1)
PB1	CY062921	H1N1	12/26/2009	Greece	A/Athens/INS166/2009(H1N1)
PB1	CY062929	H1N1	12/26/2009	Greece	A/Athens/INS167/2009(H1N1)
PB1	CY073012	H1N1	12/26/2009	Greece	A/Athens/INS330/2009(H1N1)
PB1	CY061065	H1N1	12/27/2009	USA	A/Texas/JMS393/2009(H1N1)

PB1	CY062048	H1N1	12/27/2009	USA	A/New York/0352/2009(H1N1)
PB1	CY062977	H1N1	12/28/2009	Denmark	A/Odense/INS177/2009(H1N1)
PB1	CY062032	H1N1	12/28/2009	USA	A/New York/7236/2009(H1N1)
PB1	KT180499	H1N1	12/28/2009	Singapore	A/Singapore/DMS63/2009
PB1	KC780318	H1N1	12/28/2009	USA	A/Georgia/25/2009
PB1	CY072404	H1N1	12/28/2009	Greece	A/Athens/INS333/2009(H1N1)
PB1	CY072340	H1N1	12/28/2009	USA	A/Newark/INS319/2009(H1N1)
PB1	CY063652	H1N1	12/29/2009	Greece	A/Athens/INS154/2009(H1N1)
PB1	CY063644	H1N1	12/29/2009	Greece	A/Athens/INS153/2009(H1N1)
PB1	JN187224	H1N1	12/29/2009	Taiwan	A/Taiwan/1017/2009
PB1	CY071245	H1N1	12/29/2009	Poland	A/Warsaw/INS406/2009(H1N1)
PB1	CY064786	H1N1	12/29/2009	Spain	A/Barcelona/INS190/2009(H1N1)
PB1	CY072412	H1N1	12/29/2009	Greece	A/Athens/INS334/2009(H1N1)
PB1	CY071237	H1N1	12/29/2009	Poland	A/Warsaw/INS405/2009(H1N1)
PB1	CY072452	H1N1	12/30/2009	Greece	A/Athens/INS339/2009(H1N1)
PB1	CY063041	H1N1	12/30/2009	Spain	A/Madrid/INS186/2009(H1N1)
PB1	CY062849	H1N1	12/30/2009	Greece	A/Athens/INS156/2009(H1N1)
PB1	CY062056	H1N1	12/30/2009	USA	A/New York/0357/2009(H1N1)
PB1	CY063065	H1N1	12/30/2009	Belgium	A/Tessenderlo/INS192/2009(H1N1)
PB1	CY072444	H1N1	12/30/2009	Greece	A/Athens/INS338/2009(H1N1)
PB1	CY061089	H1N1	12/30/2009	USA	A/Texas/JMS397/2009(H1N1)
PB1	CY061097	H1N1	12/30/2009	USA	A/Texas/JMS398/2009(H1N1)
PB1	CY061073	H1N1	12/30/2009	USA	A/Texas/JMS394/2009(H1N1)
PB1	CY128417	H1N1	12/30/2009	Viet Nam	A/Viet Nam/11032021/2009
PB1	CY066445	H1N1	12/30/2009	USA	A/California/VRDL133/2009(H1N1)
PB1	CY066429	H1N1	12/30/2009	USA	A/California/VRDL131/2009(H1N1)
PB1	CY062064	H1N1	12/30/2009	USA	A/New York/0461/2009(H1N1)
PB1	CY066421	H1N1	12/30/2009	USA	A/California/VRDL130/2009(H1N1)
PB1	CY061081	H1N1	12/30/2009	USA	A/Texas/JMS395/2009(H1N1)
PB1	CY063033	H1N1	12/30/2009	Spain	A/Madrid/INS185/2009(H1N1)
PB1	CY062841	H1N1	12/30/2009	Greece	A/Athens/INS155/2009(H1N1)
PB1	CY072436	H1N1	12/30/2009	Greece	A/Athens/INS337/2009(H1N1)
PB1	HM568126	H1N1	12/30/2009	United Ki	A/England/938/2009
PB1	LN846365	H1N1	12/30/2009	United Ki	A/England/WTSI1778/2009
PB1	KC780669	H1N1	12/30/2009	USA	A/Nebraska/23/2009
PB1	CY072428	H1N1	12/30/2009	Greece	A/Athens/INS336/2009(H1N1)
PB1	CY072420	H1N1	12/30/2009	Greece	A/Athens/INS335/2009(H1N1)
PB1	CY066413	H1N1	12/30/2009	USA	A/California/VRDL129/2009(H1N1)
PB1	CY066437	H1N1	12/30/2009	USA	A/California/VRDL132/2009(H1N1)
PB1	CY123320	H1N1	12/31/2009	Singapore	A/Singapore/GP4915/2009
PB1	CY071133	H1N1	12/31/2009	Greece	A/Athens/INS383/2009(H1N1)
PB1	CY061105	H1N1	12/31/2009	USA	A/Texas/JMS399/2009(H1N1)
PB1	KT180501	H1N1	12/31/2009	Singapore	A/Singapore/DMS64/2009
PB1	CY061576	H1N1	12/31/2009	USA	A/Texas/JMS400/2009(H1N1)
PB1	CY120022	H1N1	01/2010	Mexico	A/Mexico/InDRECTRLA/2010
PB1	KC780542	H1N1	01/01/2010	USA	A/New Jersey/01/2010
PB1	CY065073	H1N1	01/02/2010	USA	A/New York/7420/2010(H1N1)
PB1	CY061584	H1N1	01/03/2010	USA	A/Texas/JMS401/2010(H1N1)
PB1	KC781897	H1N1	01/04/2010	USA	A/Alabama/01/2010
PB1	JX308947	H1N1	01/04/2010	Singapore	A/Singapore/GP10/2010
PB1	CY061113	H1N1	01/04/2010	USA	A/Texas/JMS402/2010(H1N1)
PB1	CY061592	H1N1	01/04/2010	USA	A/Texas/JMS403/2010(H1N1)
PB1	CY065878	H1N1	01/04/2010	Netherlar	A/Netherlands/2631b/2010
PB1	CY063009	H1N1	01/04/2010	Estonia	A/Tallinn/INS182/2010(H1N1)

PB1	GU562464	H1N1	01/04/2010	Russia	A/Karasuk/01/2010(H1N1)
PB1	CY096592	H1N1	01/04/2010	USA	A/District of Columbia/INS525/2(
PB1	KT180502	H1N1	01/04/2010	Singapore	A/Singapore/DMS65/2010
PB1	JX308955	H1N1	01/05/2010	Singapore	A/Singapore/GP33/2010
PB1	CY062985	H1N1	01/05/2010	Germany	A/Bonn/INS178/2010(H1N1)
PB1	CY062993	H1N1	01/05/2010	Austria	A/Vienna/INS179/2010(H1N1)
PB1	KC782012	H1N1	01/05/2010	USA	A/Iowa/01/2010
PB1	KC780227	H1N1	01/05/2010	USA	A/Colorado/02/2010
PB1	CY073779	H1N1	01/05/2010	Austria	A/Vienna/INS369/2010(H1N1)
PB1	CY071061	H1N1	01/05/2010	Austria	A/Vienna/INS368/2010(H1N1)
PB1	CY075065	H1N1	01/06/2010	Greece	A/Athens/INS392/2010(H1N1)
PB1	CY075073	H1N1	01/06/2010	Greece	A/Athens/INS393/2010(H1N1)
PB1	KC780413	H1N1	01/06/2010	USA	A/Florida/01/2010
PB1	JX308963	H1N1	01/06/2010	Singapore	A/Singapore/GP64/2010
PB1	CY063017	H1N1	01/07/2010	Estonia	A/Tallinn/INS183/2010(H1N1)
PB1	KT180504	H1N1	01/07/2010	Singapore	A/Singapore/DMS66/2010
PB1	CY071077	H1N1	01/07/2010	Estonia	A/Tallinn/INS373/2010(H1N1)
PB1	CY072532	H1N1	01/07/2010	Estonia	A/Tallinn/INS374/2010(H1N1)
PB1	CY061121	H1N1	01/08/2010	USA	A/Texas/JMS404/2010(H1N1)
PB1	CY071213	H1N1	01/08/2010	Germany	A/Frankfurt/INS402/2010(H1N1)
PB1	CY066453	H1N1	01/09/2010	USA	A/California/VRDL1/2010(H1N1)
PB1	CY061129	H1N1	01/09/2010	USA	A/Texas/JMS405/2010(H1N1)
PB1	CY061137	H1N1	01/10/2010	USA	A/Texas/JMS406/2010(H1N1)
PB1	CY071221	H1N1	01/11/2010	Germany	A/Frankfurt/INS403/2010(H1N1)
PB1	KT180506	H1N1	01/11/2010	Singapore	A/Singapore/DMS67/2010
PB1	CY061145	H1N1	01/11/2010	USA	A/Texas/JMS407/2010(H1N1)
PB1	CY066461	H1N1	01/11/2010	USA	A/California/VRDL2/2010(H1N1)
PB1	CY071165	H1N1	01/11/2010	Greece	A/Athens/INS394/2010(H1N1)
PB1	KC780892	H1N1	01/12/2010	USA	A/Nevada/01/2010
PB1	AB704484	H1N1	01/12/2010	Japan	A/Tochigi/10/2010
PB1	CY062072	H1N1	01/12/2010	USA	A/New York/1680/2010(H1N1)
PB1	CY061153	H1N1	01/12/2010	USA	A/Texas/JMS408/2010(H1N1)
PB1	KP637668	H1N1	01/12/2010	Thailand	A/Thailand/SP08358/2010
PB1	JQ612505	H1N1	01/12/2010	Hungary	A/Hungary/2/2010
PB1	AB704476	H1N1	01/13/2010	Japan	A/Tochigi/2/2010
PB1	CY071141	H1N1	01/13/2010	Greece	A/Athens/INS389/2010(H1N1)
PB1	CY072540	H1N1	01/14/2010	USA	A/New York/INS432/2010(H1N1)
PB1	CY071149	H1N1	01/14/2010	Greece	A/Athens/INS390/2010(H1N1)
PB1	LN867802	H1N1	01/14/2010	United Ki	A/England/01220740/2010
PB1	KT180508	H1N1	01/14/2010	Singapore	A/Singapore/DMS68/2010
PB1	JX309015	H1N1	01/15/2010	Singapore	A/Singapore/GP256/2010
PB1	CY062080	H1N1	01/15/2010	USA	A/New York/1796/2010(H1N1)
PB1	KT180509	H1N1	01/16/2010	Singapore	A/Singapore/DMS69/2010
PB1	LN846436	H1N1	01/18/2010	United Ki	A/England/32/2010
PB1	CY080313	H1N1	01/18/2010	Thailand	A/Thailand/H1255/2010
PB1	CY062096	H1N1	01/18/2010	USA	A/New York/1999/2010(H1N1)
PB1	CY062088	H1N1	01/18/2010	USA	A/New York/1998/2010(H1N1)
PB1	CY071301	H1N1	01/18/2010	Greece	A/Athens/INS417/2010(H1N1)
PB1	CY167778	H1N1	01/19/2010	USA	A/Tennessee/F1035/2010
PB1	CY062104	H1N1	01/19/2010	USA	A/New York/2175/2010(H1N1)
PB1	CY066469	H1N1	01/19/2010	USA	A/California/VRDL3/2010(H1N1)
PB1	CY062120	H1N1	01/20/2010	USA	A/New York/2598/2010(H1N1)
PB1	JX308991	H1N1	01/20/2010	Singapore	A/Singapore/GP329/2010
PB1	CY062136	H1N1	01/20/2010	USA	A/New York/2876/2010(H1N1)

PB1	CY062112	H1N1	01/20/2010	USA	A/New York/2372/2010(H1N1)
PB1	CY061161	H1N1	01/20/2010	USA	A/Texas/JMS409/2010(H1N1)
PB1	KC780577	H1N1	01/21/2010	USA	A/Virginia/01/2010
PB1	CY071173	H1N1	01/21/2010	Greece	A/Athens/INS396/2010(H1N1)
PB1	JX309023	H1N1	01/21/2010	Singapore	A/Singapore/GP368/2010
PB1	CY062128	H1N1	01/22/2010	USA	A/New York/2648/2010(H1N1)
PB1	KT180511	H1N1	01/22/2010	Singapore	A/Singapore/DMS71/2010
PB1	CY062152	H1N1	01/22/2010	USA	A/New York/2963/2010(H1N1)
PB1	CY061169	H1N1	01/22/2010	USA	A/Texas/JMS410/2010(H1N1)
PB1	CY062160	H1N1	01/23/2010	USA	A/New York/2971/2010(H1N1)
PB1	KC780493	H1N1	01/24/2010	USA	A/Wisconsin/01/2010
PB1	CY071189	H1N1	01/24/2010	Greece	A/Athens/INS398/2010(H1N1)
PB1	CY071181	H1N1	01/24/2010	Greece	A/Athens/INS397/2010(H1N1)
PB1	CY061177	H1N1	01/25/2010	USA	A/Texas/JMS411/2010(H1N1)
PB1	JX309031	H1N1	01/25/2010	Singapore	A/Singapore/GP413/2010
PB1	CY063217	H1N1	01/25/2010	USA	A/California/VRDL14/2010(H1N1)
PB1	JX309039	H1N1	01/25/2010	Singapore	A/Singapore/GP398/2010
PB1	CY062168	H1N1	01/25/2010	USA	A/New York/3230/2010(H1N1)
PB1	CY063209	H1N1	01/25/2010	USA	A/California/VRDL13/2010(H1N1)
PB1	CY071309	H1N1	01/25/2010	Greece	A/Athens/INS419/2010(H1N1)
PB1	KT180512	H1N1	01/25/2010	Singapore	A/Singapore/DMS72/2010
PB1	CY062192	H1N1	01/26/2010	USA	A/New York/3251/2010(H1N1)
PB1	CY083030	H1N1	01/26/2010	Senegal	A/Dakar/WRAIR0019T/2010
PB1	CY083533	H1N1	01/26/2010	Mexico	A/Mexico City/WRAIR1752N/2010(H
PB1	JX309047	H1N1	01/26/2010	Singapore	A/Singapore/GP449/2010(H1N1)
PB1	CY062144	H1N1	01/26/2010	USA	A/New York/2960/2010(H1N1)
PB1	CY063193	H1N1	01/26/2010	USA	A/California/VRDL11/2010(H1N1)
PB1	CY062176	H1N1	01/26/2010	USA	A/New York/3236/2010(H1N1)
PB1	CY065025	H1N1	01/27/2010	USA	A/New York/3834/2010(H1N1)
PB1	CY092644	H1N1	01/27/2010	Australia	A/Sydney/DD3-37/2010
PB1	JX309063	H1N1	01/27/2010	Singapore	A/Singapore/GP489/2010(H1N1)
PB1	CY071317	H1N1	01/27/2010	Greece	A/Athens/INS420/2010(H1N1)
PB1	CY062184	H1N1	01/27/2010	USA	A/New York/3250/2010(H1N1)
PB1	CY063201	H1N1	01/27/2010	USA	A/California/VRDL12/2010(H1N1)
PB1	CY065033	H1N1	01/27/2010	USA	A/New York/3835/2010(H1N1)
PB1	KC780654	H1N1	01/27/2010	USA	A/South Carolina/01/2010
PB1	JX309071	H1N1	01/28/2010	Singapore	A/Singapore/GP512/2010(H1N1)
PB1	CY065041	H1N1	01/29/2010	USA	A/New York/3866/2010(H1N1)
PB1	CY071269	H1N1	01/30/2010	Greece	A/Athens/INS411/2010(H1N1)
PB1	KT180515	H1N1	01/31/2010	Singapore	A/Singapore/DMS74/2010
PB1	CY061185	H1N1	02/01/2010	USA	A/Texas/JMS412/2010(H1N1)
PB1	CY065001	H1N1	02/01/2010	USA	A/New York/3681/2010(H1N1)
PB1	CY065017	H1N1	02/01/2010	USA	A/New York/3683/2010(H1N1)
PB1	KC780605	H1N1	02/01/2010	USA	A/California/02/2010
PB1	CY065009	H1N1	02/01/2010	USA	A/New York/3682/2010(H1N1)
PB1	CY066493	H1N1	02/01/2010	USA	A/California/VRDL7/2010(H1N1)
PB1	KC780535	H1N1	02/02/2010	USA	A/Florida/02/2010
PB1	JX309154	H1N1	02/02/2010	Singapore	A/Singapore/GP567/2010
PB1	CY064993	H1N1	02/02/2010	USA	A/California/VRDL6/2010(H1N1)
PB1	KT180524	H1N1	02/02/2010	Singapore	A/Singapore/DMS82/2010
PB1	CY071277	H1N1	02/02/2010	Greece	A/Athens/INS412/2010(H1N1)
PB1	CY073004	H1N1	02/03/2010	Nicaragua	A/Managua/07193_09/2010(H1N1)
PB1	CY065057	H1N1	02/03/2010	USA	A/New York/4662/2010(H1N1)
PB1	CY071325	H1N1	02/03/2010	Greece	A/Athens/INS421/2010(H1N1)

PB1	KC781455	H1N1	02/03/2010	USA	A/Louisiana/01/2010
PB1	CY061193	H1N1	02/03/2010	USA	A/Texas/JMS413/2010(H1N1)
PB1	KT180516	H1N1	02/03/2010	Singapore	A/Singapore/DMS75/2010
PB1	KC780691	H1N1	02/03/2010	USA	A/Indiana/03/2010
PB1	KC782025	H1N1	02/04/2010	USA	A/Vermont/01/2010
PB1	CY167330	H1N1	02/04/2010	USA	A/Tennessee/F1042A/2010
PB1	LN846492	H1N1	02/04/2010	United Ki	A/England/01220736/2010
PB1	LN850028	H1N1	02/04/2010	United Ki	A/England/29/2010
PB1	CY065049	H1N1	02/04/2010	USA	A/New York/4294/2010(H1N1)
PB1	CY066485	H1N1	02/04/2010	USA	A/California/VRDL5/2010(H1N1)
PB1	CY061201	H1N1	02/04/2010	USA	A/Texas/JMS414/2010(H1N1)
PB1	CY071293	H1N1	02/05/2010	Greece	A/Athens/INS414/2010(H1N1)
PB1	CY071285	H1N1	02/05/2010	Greece	A/Athens/INS413/2010(H1N1)
PB1	CY073739	H1N1	02/05/2010	Estonia	A/Tallinn/INS431/2010(H1N1)
PB1	CY071381	H1N1	02/05/2010	Germany	A/Berlin/INS430/2010(H1N1)
PB1	CY083823	H1N1	02/05/2010	USA	A/Los Angeles/INS423/2010(H1N1)
PB1	KC781249	H1N1	02/05/2010	USA	A/Georgia/06/2010
PB1	KT180518	H1N1	02/05/2010	Singapore	A/Singapore/DMS76/2010
PB1	CY061209	H1N1	02/06/2010	USA	A/Texas/JMS415/2010(H1N1)
PB1	KC781260	H1N1	02/06/2010	USA	A/Georgia/04/2010
PB1	KC780238	H1N1	02/06/2010	USA	A/Georgia/01/2010
PB1	KC781385	H1N1	02/07/2010	USA	A/Alaska/01/2010
PB1	KP637284	H1N1	02/07/2010	Thailand	A/Thailand/IY07115/2010
PB1	KT180519	H1N1	02/08/2010	Singapore	A/Singapore/DMS77/2010
PB1	KC781184	H1N1	02/08/2010	USA	A/Wyoming/01/2010
PB1	CY066477	H1N1	02/08/2010	USA	A/California/VRDL4/2010(H1N1)
PB1	KC261312	H1N1	02/09/2010	Mexico	A/Mexico City/INERPHAC3/2010(H1N
PB1	CY167338	H1N1	02/09/2010	USA	A/Tennessee/F1052A/2010
PB1	KP637836	H1N1	02/09/2010	Thailand	A/Thailand/IY07144/2010
PB1	KC780455	H1N1	02/09/2010	USA	A/New York/03/2010
PB1	CY100536	H1N1	02/09/2010	Mexico	A/Mexico City/INER11/2010
PB1	CY090867	H1N1	02/09/2010	Netherlar	A/Netherlands/2631c/2010(H1N1)
PB1	CY066509	H1N1	02/09/2010	USA	A/California/VRDL9/2010(H1N1)
PB1	KT180520	H1N1	02/10/2010	Singapore	A/Singapore/DMS78/2010
PB1	KC780499	H1N1	02/10/2010	USA	A/Utah/02/2010
PB1	CY063185	H1N1	02/11/2010	USA	A/California/VRDL10/2010(H1N1)
PB1	KJ856112	H1N1	02/12/2010	Sri Lanka	A/Sri Lanka/20887/2010
PB1	CY066501	H1N1	02/13/2010	USA	A/California/VRDL8/2010(H1N1)
PB1	CY083038	H1N1	02/16/2010	Senegal	A/Dakar/WRAIR0020T/2010
PB1	CY097787	H1N1	02/16/2010	Senegal	A/Dakar/WRAIR0020T/2010
PB1	CY075081	H1N1	02/17/2010	Greece	A/Athens/INS416/2010(H1N1)
PB1	KC780519	H1N1	02/17/2010	USA	A/Wisconsin/02/2010
PB1	CY167346	H1N1	02/18/2010	USA	A/Tennessee/F1057c56/2010
PB1	CY167354	H1N1	02/18/2010	USA	A/Tennessee/F1060/2010
PB1	KP637860	H1N1	02/18/2010	Thailand	A/Thailand/SS08248/2010
PB1	LN867347	H1N1	02/20/2010	United Ki	A/England/01220741/2010
PB1	KT180521	H1N1	02/20/2010	Singapore	A/Singapore/DMS80/2010
PB1	CY065065	H1N1	02/21/2010	USA	A/New York/6530/2010(H1N1)
PB1	CY071333	H1N1	02/23/2010	Thailand	A/Bangkok/INS424/2010(H1N1)
PB1	CY096600	H1N1	02/23/2010	USA	A/District of Columbia/INS527/2(
PB1	KC782137	H1N1	02/23/2010	USA	A/Tennessee/01/2010
PB1	KT180523	H1N1	02/24/2010	Singapore	A/Singapore/DMS81/2010
PB1	CY129700	H1N1	02/24/2010	Greece	A/Athens/INS555/2010
PB1	KC780358	H1N1	02/24/2010	USA	A/North Carolina/05/2010

PB1	CY071341	H1N1	02/25/2010	Thailand	A/Bangkok/INS425/2010(H1N1)
PB1	CY071349	H1N1	02/25/2010	Thailand	A/Bangkok/INS426/2010(H1N1)
PB1	CY167370	H1N1	02/26/2010	USA	A/Tennessee/F1066c65/2010
PB1	CY063838	H1N1	02/26/2010	Singapore	A/Singapore/GP999/2010(H1N1)
PB1	CY167362	H1N1	02/26/2010	USA	A/Tennessee/F1063A/2010
PB1	CY167378	H1N1	02/26/2010	USA	A/Tennessee/F1067/2010
PB1	KC781164	H1N1	03/01/2010	USA	A/Alabama/03/2010
PB1	CY065113	H1N1	03/01/2010	USA	A/New York/7480/2010(H1N1)
PB1	KT180525	H1N1	03/01/2010	Singapore	A/Singapore/DMS83/2010
PB1	CY167410	H1N1	03/02/2010	USA	A/Tennessee/F1072c71/2010
PB1	CY167386	H1N1	03/02/2010	USA	A/Tennessee/F1068/2010
PB1	HM124381	H1N1	03/02/2010	Russia	A/Orenburg/IIV-13/2010(H1N1)
PB1	CY167394	H1N1	03/02/2010	USA	A/Tennessee/F1071/2010
PB1	HM569738	H1N1	03/02/2010	Russia	A/Orenburg/IIV-13/2010(H1N1)
PB1	KC781357	H1N1	03/03/2010	USA	A/Iowa/04/2010
PB1	CY071357	H1N1	03/04/2010	Thailand	A/Bangkok/INS427/2010(H1N1)
PB1	CY071365	H1N1	03/04/2010	Thailand	A/Bangkok/INS428/2010(H1N1)
PB1	KC780736	H1N1	03/04/2010	USA	A/California/05/2010
PB1	CY071373	H1N1	03/05/2010	USA	A/Newark/INS429/2010(H1N1)
PB1	CY167402	H1N1	03/05/2010	USA	A/Tennessee/F1071B/2010
PB1	KC781403	H1N1	03/08/2010	USA	A/Utah/03/2010
PB1	JX309232	H1N1	03/10/2010	Singapore	A/Singapore/KK100/2010(H1N1)
PB1	KC781397	H1N1	03/11/2010	USA	A/Nebraska/02/2010
PB1	KT180528	H1N1	03/12/2010	Singapore	A/Singapore/DMS86/2010
PB1	CY167418	H1N1	03/15/2010	USA	A/Tennessee/F1074/2010
PB1	KT180531	H1N1	03/16/2010	Singapore	A/Singapore/DMS88/2010
PB1	CY167426	H1N1	03/16/2010	USA	A/Tennessee/F1076/2010
PB1	CY167442	H1N1	03/16/2010	USA	A/Tennessee/F1078/2010
PB1	LN846588	H1N1	03/18/2010	United Ki	A/England/00380718/2010
PB1	LN867778	H1N1	03/18/2010	United Ki	A/England/01180011/2010
PB1	LN846508	H1N1	03/18/2010	United Ki	A/England/01180016/2010
PB1	LN867395	H1N1	03/18/2010	United Ki	A/England/WTSI1783/2010
PB1	LN846785	H1N1	03/18/2010	United Ki	A/England/10/2010
PB1	LN850123	H1N1	03/18/2010	United Ki	A/England/11/2010
PB1	LN846532	H1N1	03/18/2010	United Ki	A/England/00380719/2010
PB1	LN850335	H1N1	03/18/2010	United Ki	A/England/00380709/2010
PB1	LN867484	H1N1	03/18/2010	United Ki	A/England/01180017/2010
PB1	LN850076	H1N1	03/18/2010	United Ki	A/England/00380710/2010
PB1	LN867818	H1N1	03/18/2010	United Ki	A/England/WTSI2076/2010
PB1	LN867421	H1N1	03/18/2010	United Ki	A/England/01180015/2010
PB1	LN850100	H1N1	03/18/2010	United Ki	A/England/WTSI1807/2010
PB1	LN850343	H1N1	03/18/2010	United Ki	A/England/01180018/2010
PB1	LN867674	H1N1	03/18/2010	United Ki	A/England/WTSI2067/2010
PB1	LN850419	H1N1	03/18/2010	United Ki	A/England/01180007/2010
PB1	LN867371	H1N1	03/18/2010	United Ki	A/England/WTSI1774/2010
PB1	LN845772	H1N1	03/18/2010	United Ki	A/England/12/2010
PB1	CY167434	H1N1	03/19/2010	USA	A/Tennessee/F1076A/2010
PB1	CY167450	H1N1	03/19/2010	USA	A/Tennessee/F1083c80/2010
PB1	CY167786	H1N1	03/19/2010	USA	A/Tennessee/F1080/2010
PB1	CY089377	H1N1	03/19/2010	Nicaragua	A/Managua/5708.01/2010(H1N1)
PB1	KT180532	H1N1	03/20/2010	Singapore	A/Singapore/DMS89/2010
PB1	CY080321	H1N1	03/20/2010	Thailand	A/Thailand/CU-H1786/2010(H1N1)
PB1	CY096608	H1N1	03/21/2010	USA	A/Cambridge/INS528/2010(H1N1)
PB1	JF764086	H1N1	03/22/2010	India	A/India/Blore/2010

PB1	KM013743	H1N1	03/23/2010	Thailand	A/Bangkok/SIMI508/2010
PB1	KT180533	H1N1	03/23/2010	Singapore	A/Singapore/DMS90/2010
PB1	CY090843	H1N1	03/23/2010	Nicaragua	A/Managua/0305_10/2010(H1N1)
PB1	CY167458	H1N1	03/24/2010	USA	A/Tennessee/F1089/2010
PB1	KC261313	H1N1	03/25/2010	Mexico	A/Mexico City/INERPHAC4/2010(H1N
PB1	CY089385	H1N1	03/25/2010	Nicaragua	A/Managua/3246.01/2010(H1N1)
PB1	KT180535	H1N1	03/25/2010	Singapore	A/Singapore/DMS91/2010
PB1	LN867387	H1N1	03/26/2010	United Ki	A/England/01580005/2010
PB1	CY080329	H1N1	03/29/2010	Thailand	A/Thailand/H1818/2010
PB1	KT180537	H1N1	03/29/2010	Singapore	A/Singapore/DMS92/2010
PB1	CY080337	H1N1	03/31/2010	Thailand	A/Thailand/CU-H1821/2010(H1N1)

1)

1)

1)
1)
1)
1)

1)

1)

1)

1)

1)

1)

1)

1)

1)

)

1)

1)
1)
1)
1)
1)
1)
1)
1)

)
)

1)

1)
1)
1)
1)

1)
1)
1)

1)
1)

1)

1)
1)
1)
1)
1)
1)
1)
1)
1)
1)
1)

1)

1)

1)

1)

1)

1)

1)
1)
1)
1)

1)

1)

1)
1)
1)

1)

1)

1)

1)
1)

1)
1)

1)
1)
1)

1)
1)
1)

1)

1)
1)
1)

1)

1)

1)

1)

1)

1)
1)

1)

)

1)

)
1)

1)

1)

)

1)

1)
1)
1)

1)

1)

1)
1)
1)
1)
1)

1)

)

1)
1)
1)

1)
1)

)

1)

1)

1)
1)

1)

)
)

1)

)
1)

1)

1)

1)
1)

1)

1)
1)

)
1)

1)

1)

)

)
)
)
1)

)
)

)
1)

)

)

)

)

1)

)

)
)
)
)
)

)

1)

1)

1)
1)
1)

1)

1)

1)

)
)
1)

)
1)
1)

1)

)

1)

)

)09(H1N1)

)

)
1)

)09(H1N1)

1)

1)

1)

1)

)

)

1)

1)

)

)

1)

1N1)

ə
ə

N1)

1N1)

1N1)

1N1)

1N1)

9

9

N1)

1N1)

1N1)

1N1)

ϑ

)09

9

ENRS-1649/2009

3/2009

2009

009
9

1N1)

1N1)

)
1)
)

1)

1)

1)

1)

1)

1)

1)

1)

1)
1)
1)

1)

1)
1)

1)

1)

1)

1)
1)

1)

1)

1)

1)

)

1)
1)

1)

1)

1)

1)

9

1)

1)

1)

1)

9

1)

1)

)

1)

1)
9 (H1N1)

9

1)
1)

1)

1)

1)
)
)
N1)

1)

)
)
)

)
)
)

)
)

)
)

1)

)

)

)
)9 (H1N1)

1)

1)
1)

1)
1)

)9 (H1N1)

1)
)
)

9 (H1N1)

9(H1N1)

9(H1N1)

)
)

09(H1N1)

)

09(H1N1)

1)

1)

)
09(H1N1)

09(H1N1)
)
09(H1N1)

09(H1N1)
)

09(H1N1)

)
)

09(H1N1)

09(H1N1)
)

09(H1N1)

9

09(H1N1)

1)
1)
1)

1)

009(H1N1)

1)

1)

09(H1N1)

09(H1N1)

09(H1N1)

)09 (H1N1)

1)
1)

)09 (H1N1)

1)
)09
1)
1)

1)
1)
N1)

)9 (H1N1)

1)

1)

1)

1)
1)

1)

1)

009 (H1N1)

)09(H1N1)

)09(H1N1)

1)
1)
1)
1)

1)
1)
1)

1)

1)

1)
1)

1)

1)

1)

1)

1)

1)

)09(H1N1)
1)
1)

)09

1)

1)

1）

1）

1）

1)
1)
1)
1)
1)

1)

1)

1)

1)
1)

1)

1)

1)

1)

1)

1)

1)
1)

1)

1)

1)
1)

1)

1)

)10 (H1N1)

1N1)

N1)

010(H1N1)

N1)

Name	Sequence Ac	Subtype	Collection Date	Country	Strain Name
PA	CY080615	H1N1	04/01/2009	USA	A/Boston/95/2009
PA	CY100843	H1N1	04/01/2009	USA	A/Alabama/WRAIR1235P/2009(H1N1)
PA	FJ969515	H1N1	04/01/2009	USA	A/California/04/2009(H1N1)
PA	FJ966081	H1N1	04/01/2009	USA	A/California/04/2009(H1N1)
PA	CY077594	H1N1	04/03/2009	Mexico	A/Mexico/LaGloria-3/2009(H1N1)
PA	GQ149653	H1N1	04/03/2009	Mexico	A/Mexico/4108/2009(H1N1)
PA	GQ463206	H1N1	04/03/2009	Mexico	A/Mexico/4108/2009(H1N1)
PA	GQ149661	H1N1	04/03/2009	Mexico	A/Mexico/4108/2009(H1N1)
PA	GQ149686	H1N1	04/03/2009	Mexico	A/Mexico/4108/2009(H1N1)
PA	GQ303346	H1N1	04/07/2009	Mexico	A/Mexico/4115/2009(H1N1)
PA	CY181398	H1N1	04/09/2009	USA	A/California/07-JR002/2009
PA	KC780062	H1N1	04/09/2009	USA	A/California/08/2009
PA	FJ984368	H1N1	04/09/2009	USA	A/California/08/2009(H1N1)
PA	CY181406	H1N1	04/09/2009	USA	A/California/07-JR003/2009
PA	FJ969529	H1N1	04/09/2009	USA	A/California/07/2009(H1N1)
PA	FJ969539	H1N1	04/09/2009	USA	A/California/07/2009(H1N1)
PA	FJ966977	H1N1	04/09/2009	USA	A/California/07/2009(H1N1)
PA	KC781786	H1N1	04/09/2009	USA	A/California/07/2009
PA	KU933484	H1N1	04/09/2009	USA	A/California/07/2009
PA	CY266196	H1N1	04/09/2009	USA	A/California/07/2009
PA	CY181422	H1N1	04/09/2009	USA	A/California/07-JR009/2009
PA	CY181430	H1N1	04/09/2009	USA	A/California/07-JR010/2009
PA	CY181414	H1N1	04/09/2009	USA	A/California/07-JR008/2009
PA	GQ377053	H1N1	04/09/2009	USA	A/California/08/2009(H1N1)
PA	GQ397123	H1N1	04/13/2009	Mexico	A/Mexico/4176/2009(H1N1)
PA	FJ998223	H1N1	04/14/2009	Mexico	A/Mexico/InDRE4487/2009(H1N1)
PA	FJ981619	H1N1	04/14/2009	USA	A/Texas/04/2009(H1N1)
PA	FJ969524	H1N1	04/14/2009	USA	A/Texas/04/2009(H1N1)
PA	CY044234	H1N1	04/14/2009	USA	A/San Antonio/PR921/2009(H1N1)
PA	GQ149676	H1N1	04/14/2009	Mexico	A/Mexico/4482/2009(H1N1)
PA	GQ149646	H1N1	04/14/2009	Mexico	A/Mexico/4486/2009(H1N1)
PA	GQ149620	H1N1	04/14/2009	Mexico	A/Mexico/4486/2009(H1N1)
PA	CY147776	H1N1	04/14/2009	Mexico	A/Mexico/24033/2009
PA	CY044242	H1N1	04/15/2009	USA	A/San Antonio/PR922/2009(H1N1)
PA	GQ339882	H1N1	04/15/2009	Mexico	A/Mexico/4283/2009(H1N1)
PA	GQ200257	H1N1	04/15/2009	USA	A/Texas/15/2009(H1N1)
PA	GQ303338	H1N1	04/15/2009	Mexico	A/Mexico/4269/2009(H1N1)
PA	FJ981610	H1N1	04/15/2009	USA	A/Texas/05/2009(H1N1)
PA	FJ966970	H1N1	04/15/2009	USA	A/Texas/05/2009(H1N1)
PA	GQ402294	H1N1	04/16/2009	Canada	A/Canada-QC/RV1595/2009(H1N1)
PA	FJ966964	H1N1	04/16/2009	USA	A/California/06/2009(H1N1)
PA	KC781716	H1N1	04/16/2009	USA	A/California/06/2009
PA	CY147824	H1N1	04/17/2009	Mexico	A/Mexico/24043/2009
PA	CY147768	H1N1	04/17/2009	Mexico	A/Mexico/24032/2009
PA	GQ149627	H1N1	04/19/2009	Mexico	A/Mexico/4603/2009(H1N1)
PA	GQ379813	H1N1	04/19/2009	Mexico	A/Mexico/4603/2009(H1N1)
PA	GQ149636	H1N1	04/19/2009	Mexico	A/Mexico/4604/2009(H1N1)
PA	GQ397118	H1N1	04/20/2009	Mexico	A/Mexico/4635/2009(H1N1)
PA	CY147832	H1N1	04/20/2009	Mexico	A/Mexico/24044/2009
PA	CY147840	H1N1	04/20/2009	Mexico	A/Mexico/24045/2009
PA	CY043093	H1N1	04/21/2009	USA	A/Brawley/40082/2009(H1N1)
PA	GQ117095	H1N1	04/22/2009	USA	A/Indiana/09/2009(H1N1)
PA	GQ377065	H1N1	04/22/2009	USA	A/Arizona/01/2009(H1N1)

PA	CY044250	H1N1	04/23/2009	USA	A/San Antonio/PR923/2009(H1N1)
PA	CY147616	H1N1	04/23/2009	Mexico	A/Mexico/24007/2009
PA	CY147704	H1N1	04/23/2009	Mexico	A/Mexico/24021/2009
PA	GQ117049	H1N1	04/24/2009	USA	A/Texas/08/2009(H1N1)
PA	CY041554	H1N1	04/24/2009	USA	A/New York/3210/2009(H1N1)
PA	FJ998224	H1N1	04/24/2009	Canada	A/Canada-ON/RV1527/2009(H1N1)
PA	GQ200244	H1N1	04/24/2009	USA	A/Iowa/04/2009(H1N1)
PA	GQ168865	H1N1	04/24/2009	USA	A/New York/23/2009(H1N1)
PA	FJ998222	H1N1	04/24/2009	Canada	A/Canada-NS/RV1535/2009(H1N1)
PA	CY065149	H1N1	04/24/2009	United Ki	A/Scotland/91800799/2009
PA	GQ373261	H1N1	04/24/2009	Canada	A/Toronto/3141/2009(H1N1)
PA	FJ984400	H1N1	04/24/2009	USA	A/Ohio/07/2009(H1N1)
PA	GQ338337	H1N1	04/24/2009	USA	A/New York/22/2009(H1N1)
PA	GQ200249	H1N1	04/24/2009	USA	A/New York/21/2009(H1N1)
PA	FJ984359	H1N1	04/24/2009	USA	A/New York/31/2009(H1N1)
PA	GQ200225	H1N1	04/24/2009	USA	A/Kansas/03/2009(H1N1)
PA	GQ377055	H1N1	04/24/2009	USA	A/Kansas/02/2009(H1N1)
PA	GQ117029	H1N1	04/25/2009	USA	A/Texas/09/2009(H1N1)
PA	CY147680	H1N1	04/25/2009	Mexico	A/Mexico/24015/2009
PA	CY040887	H1N1	04/25/2009	Mexico	A/Mexico/47N/2009(H1N1)
PA	GQ200211	H1N1	04/25/2009	USA	A/New York/06/2009(H1N1)
PA	CY044162	H1N1	04/25/2009	Mexico	A/Mexico/48N/2009(H1N1)
PA	GQ117037	H1N1	04/25/2009	USA	A/California/14/2009(H1N1)
PA	CY041618	H1N1	04/25/2009	USA	A/New York/3002/2009(H1N1)
PA	FJ984374	H1N1	04/25/2009	USA	A/New York/10/2009(H1N1)
PA	FJ984380	H1N1	04/25/2009	USA	A/New York/15/2009(H1N1)
PA	FJ984393	H1N1	04/25/2009	USA	A/New York/19/2009(H1N1)
PA	GQ200259	H1N1	04/25/2009	USA	A/New York/20/2009(H1N1)
PA	FJ984346	H1N1	04/25/2009	USA	A/New York/11/2009(H1N1)
PA	CY040642	H1N1	04/25/2009	USA	A/New York/3191/2009(H1N1)
PA	FJ984354	H1N1	04/25/2009	USA	A/New York/18/2009(H1N1)
PA	CY041135	H1N1	04/25/2009	USA	A/New York/3215/2009(H1N1)
PA	GQ457457	H1N1	04/25/2009	USA	A/New York/12/2009(H1N1)
PA	GQ117115	H1N1	04/25/2009	USA	A/New York/13/2009(H1N1)
PA	CY041127	H1N1	04/25/2009	USA	A/New York/3214/2009(H1N1)
PA	CY041546	H1N1	04/25/2009	USA	A/New York/3209/2009(H1N1)
PA	GQ457508	H1N1	04/25/2009	USA	A/New York/09/2009(H1N1)
PA	CY064876	H1N1	04/25/2009	USA	A/California/VRDL146/2009(H1N1)
PA	CY086982	H1N1	04/25/2009	USA	A/New York/3217/2009(H1N1)
PA	CY065141	H1N1	04/25/2009	United Ki	A/Scotland/91800797/2009
PA	CY097875	H1N1	04/25/2009	Mexico	A/Mexico/47N/2009
PA	GQ457494	H1N1	04/26/2009	USA	A/Massachusetts/07/2009(H1N1)
PA	CY074632	H1N1	04/26/2009	USA	A/California/VRDL371/2009(H1N1)
PA	CY041175	H1N1	04/26/2009	USA	A/New York/3186/2009(H1N1)
PA	CY041055	H1N1	04/26/2009	USA	A/New York/3166/2009(H1N1)
PA	CY041103	H1N1	04/26/2009	USA	A/New York/3176/2009(H1N1)
PA	CY041610	H1N1	04/26/2009	USA	A/New York/3207/2009(H1N1)
PA	GQ457472	H1N1	04/26/2009	USA	A/South Carolina/09/2009(H1N1)
PA	GQ117109	H1N1	04/26/2009	USA	A/Michigan/02/2009(H1N1)
PA	GQ457490	H1N1	04/26/2009	USA	A/Arizona/02/2009(H1N1)
PA	CY041535	H1N1	04/26/2009	USA	A/New York/3205/2009(H1N1)
PA	GQ465737	H1N1	04/26/2009	Canada	A/Canada-PQ/RV1586/2009(H1N1)
PA	GQ396737	H1N1	04/26/2009	USA	A/New Mexico/04/2009(H1N1)
PA	CY039898	H1N1	04/26/2009	USA	A/New York/1669/2009(H1N1)

PA	CY041063	H1N1	04/26/2009	USA	A/New York/3168/2009(H1N1)
PA	GQ377039	H1N1	04/26/2009	USA	A/Massachusetts/06/2009(H1N1)
PA	CY041739	H1N1	04/26/2009	USA	A/New York/3206/2009(H1N1)
PA	CY041071	H1N1	04/26/2009	USA	A/New York/3169/2009(H1N1)
PA	GQ457498	H1N1	04/26/2009	USA	A/Texas/22/2009(H1N1)
PA	CY074616	H1N1	04/26/2009	USA	A/California/VRDL366/2009(H1N1)
PA	CY074648	H1N1	04/26/2009	USA	A/California/VRDL378/2009(H1N1)
PA	GQ396741	H1N1	04/26/2009	USA	A/Nevada/03/2009(H1N1)
PA	CY065221	H1N1	04/27/2009	United Ki	A/England/207/2009
PA	CY065245	H1N1	04/27/2009	United Ki	A/England/XFL00071/2009
PA	CY065197	H1N1	04/27/2009	United Ki	A/England/197/2009
PA	CY065237	H1N1	04/27/2009	United Ki	A/England/551/2009
PA	CY065189	H1N1	04/27/2009	United Ki	A/England/202/2009
PA	CY087148	H1N1	04/27/2009	USA	A/California/VRDL137/2009
PA	CY087156	H1N1	04/27/2009	USA	A/California/VRDL148/2009
PA	CY069772	H1N1	04/27/2009	United Ki	A/England/209/2009
PA	CY147720	H1N1	04/27/2009	Mexico	A/Mexico/24024/2009
PA	CY147904	H1N1	04/27/2009	Mexico	A/Mexico/24054/2009
PA	CY147864	H1N1	04/27/2009	Mexico	A/Mexico/24049/2009
PA	CY147848	H1N1	04/27/2009	Mexico	A/Mexico/24047/2009
PA	CY147896	H1N1	04/27/2009	Mexico	A/Mexico/24053/2009
PA	CY147784	H1N1	04/27/2009	Mexico	A/Mexico/24036/2009
PA	CY073834	H1N1	04/27/2009	USA	A/California/VRDL155/2009(H1N1)
PA	CY074640	H1N1	04/27/2009	USA	A/California/VRDL377/2009(H1N1)
PA	CY074400	H1N1	04/27/2009	USA	A/California/VRDL267/2009(H1N1)
PA	CY074672	H1N1	04/27/2009	USA	A/California/VRDL382/2009(H1N1)
PA	CY074128	H1N1	04/27/2009	USA	A/California/VRDL194/2009(H1N1)
PA	CY041519	H1N1	04/27/2009	USA	A/New York/3203/2009(H1N1)
PA	GQ465738	H1N1	04/27/2009	Canada	A/Canada-ON/RV1589/2009(H1N1)
PA	GQ200204	H1N1	04/27/2009	USA	A/Florida/04/2009(H1N1)
PA	CY041087	H1N1	04/27/2009	USA	A/New York/3172/2009(H1N1)
PA	CY041527	H1N1	04/27/2009	USA	A/New York/3204/2009(H1N1)
PA	GQ200264	H1N1	04/27/2009	USA	A/Colorado/03/2009(H1N1)
PA	CY040020	H1N1	04/27/2009	USA	A/New York/3014/2009(H1N1)
PA	CY039906	H1N1	04/27/2009	USA	A/New York/1682/2009(H1N1)
PA	GQ200269	H1N1	04/27/2009	USA	A/Nebraska/03/2009(H1N1)
PA	GQ200230	H1N1	04/27/2009	USA	A/Alabama/02/2009(H1N1)
PA	GQ457488	H1N1	04/27/2009	USA	A/Minnesota/02/2009(H1N1)
PA	CY040047	H1N1	04/27/2009	USA	A/New York/3007/2009(H1N1)
PA	CY040827	H1N1	04/27/2009	USA	A/New York/3260/2009(H1N1)
PA	CY041570	H1N1	04/27/2009	USA	A/New York/3225/2009(H1N1)
PA	CY040028	H1N1	04/27/2009	USA	A/New York/3049/2009(H1N1)
PA	GQ402291	H1N1	04/27/2009	Mexico	A/Mexico/InDRE13547/2009(H1N1)
PA	CY040012	H1N1	04/27/2009	USA	A/New York/3012/2009(H1N1)
PA	CY040004	H1N1	04/27/2009	USA	A/New York/3008/2009(H1N1)
PA	CY040843	H1N1	04/27/2009	USA	A/New York/3262/2009(H1N1)
PA	CY074656	H1N1	04/27/2009	USA	A/California/VRDL379/2009(H1N1)
PA	CY074608	H1N1	04/27/2009	USA	A/California/VRDL365/2009(H1N1)
PA	CY074624	H1N1	04/27/2009	USA	A/California/VRDL369/2009(H1N1)
PA	CY074664	H1N1	04/27/2009	USA	A/California/VRDL380/2009(H1N1)
PA	CY080623	H1N1	04/27/2009	USA	A/Boston/100/2009
PA	CY070900	H1N1	04/27/2009	USA	A/California/VRDL149/2009(H1N1)
PA	CY070908	H1N1	04/27/2009	USA	A/California/VRDL150/2009(H1N1)
PA	CY073818	H1N1	04/27/2009	USA	A/California/VRDL153/2009(H1N1)

PA	CY073810	H1N1	04/27/2009	USA	A/California/VRDL152/2009(H1N1)
PA	CY050481	H1N1	04/27/2009	USA	A/New York/1692/2009(H1N1)
PA	CY050665	H1N1	04/27/2009	USA	A/New York/3150/2009
PA	CY064804	H1N1	04/27/2009	USA	A/California/VRDL134/2009(H1N1)
PA	CY064812	H1N1	04/27/2009	USA	A/California/VRDL135/2009(H1N1)
PA	CY064836	H1N1	04/27/2009	USA	A/California/VRDL140/2009(H1N1)
PA	CY147712	H1N1	04/27/2009	Mexico	A/Mexico/24022/2009
PA	CY147888	H1N1	04/27/2009	Mexico	A/Mexico/24052/2009
PA	CY147872	H1N1	04/27/2009	Mexico	A/Mexico/24050/2009
PA	CY147880	H1N1	04/27/2009	Mexico	A/Mexico/24051/2009
PA	CY147856	H1N1	04/27/2009	Mexico	A/Mexico/24048/2009
PA	CY065213	H1N1	04/27/2009	United Ki	A/England/205/2009
PA	GQ465733	H1N1	04/28/2009	Canada	A/Canada-AB/RV1531/2009(H1N1)
PA	GQ200267	H1N1	04/28/2009	USA	A/North Carolina/05/2009(H1N1)
PA	GQ166654	H1N1	04/28/2009	United Ki	A/England/195/2009
PA	CY041503	H1N1	04/28/2009	USA	A/New York/3195/2009(H1N1)
PA	CY041151	H1N1	04/28/2009	USA	A/New York/3181/2009(H1N1)
PA	CY041594	H1N1	04/28/2009	USA	A/New York/3236/2009(H1N1)
PA	CY040706	H1N1	04/28/2009	USA	A/New York/3235/2009(H1N1)
PA	CY041191	H1N1	04/28/2009	USA	A/New York/3188/2009(H1N1)
PA	CY041835	H1N1	04/28/2009	USA	A/New York/3348/2009(H1N1)
PA	CY040722	H1N1	04/28/2009	USA	A/New York/3238/2009(H1N1)
PA	CY041095	H1N1	04/28/2009	USA	A/New York/3173/2009(H1N1)
PA	CY040634	H1N1	04/28/2009	USA	A/New York/3234/2009(H1N1)
PA	GQ338399	H1N1	04/28/2009	USA	A/Rhode Island/02/2009(H1N1)
PA	GQ200205	H1N1	04/28/2009	USA	A/Tennessee/05/2009(H1N1)
PA	CY040714	H1N1	04/28/2009	USA	A/New York/3237/2009(H1N1)
PA	CY041586	H1N1	04/28/2009	USA	A/New York/3230/2009(H1N1)
PA	CY041562	H1N1	04/28/2009	USA	A/New York/3211/2009(H1N1)
PA	CY064844	H1N1	04/28/2009	USA	A/California/VRDL141/2009(H1N1)
PA	GQ200265	H1N1	04/28/2009	USA	A/New Hampshire/02/2009(H1N1)
PA	GQ200261	H1N1	04/28/2009	USA	A/Washington/09/2009(H1N1)
PA	CY041159	H1N1	04/28/2009	USA	A/New York/3183/2009(H1N1)
PA	CY041495	H1N1	04/28/2009	USA	A/New York/3184/2009(H1N1)
PA	CY041207	H1N1	04/28/2009	USA	A/New York/3199/2009(H1N1)
PA	CY041111	H1N1	04/28/2009	USA	A/New York/3178/2009(H1N1)
PA	CY041578	H1N1	04/28/2009	USA	A/New York/3227/2009(H1N1)
PA	GQ402292	H1N1	04/28/2009	Mexico	A/Mexico/InDRE13551/2009(H1N1)
PA	CY046336	H1N1	04/28/2009	USA	A/Wisconsin/629-D01505/2009(H1N1)
PA	CY047331	H1N1	04/28/2009	USA	A/New York/3167/2009(H1N1)
PA	CY047323	H1N1	04/28/2009	USA	A/New York/3124/2009(H1N1)
PA	CY050066	H1N1	04/28/2009	USA	A/Cherry Point/WR1355/2009(H1N1)
PA	CY041511	H1N1	04/28/2009	USA	A/New York/3196/2009(H1N1)
PA	CY041119	H1N1	04/28/2009	USA	A/New York/3193/2009(H1N1)
PA	GQ464407	H1N1	04/28/2009	Spain	A/Catalonia/63/2009(H1N1)
PA	CY073393	H1N1	04/28/2009	USA	A/Brawley/WR1635P/2009(H1N1)
PA	CY070916	H1N1	04/28/2009	USA	A/California/VRDL151/2009(H1N1)
PA	CY073850	H1N1	04/28/2009	USA	A/California/VRDL167/2009(H1N1)
PA	CY073858	H1N1	04/28/2009	USA	A/California/VRDL175/2009(H1N1)
PA	CY073826	H1N1	04/28/2009	USA	A/California/VRDL154/2009(H1N1)
PA	CY053076	H1N1	04/28/2009	USA	A/Houston/130S/2009(H1N1)
PA	CY147944	H1N1	04/28/2009	Mexico	A/Mexico/24060/2009
PA	CY147920	H1N1	04/28/2009	Mexico	A/Mexico/24056/2009
PA	CY083303	H1N1	04/28/2009	USA	A/San Diego/WRAIR1648P/2009

PA	CY092350	H1N1	04/28/2009	USA	A/California/VRDL191/2009
PA	CY065205	H1N1	04/28/2009	United Ki	A/England/204/2009
PA	CY065229	H1N1	04/28/2009	United Ki	A/England/200/2009
PA	CY065253	H1N1	04/28/2009	United Ki	A/England/211/2009
PA	CY147736	H1N1	04/28/2009	Mexico	A/Mexico/24026/2009
PA	CY147952	H1N1	04/28/2009	Mexico	A/Mexico/24061/2009
PA	CY147912	H1N1	04/28/2009	Mexico	A/Mexico/24055/2009
PA	CY147792	H1N1	04/28/2009	Mexico	A/Mexico/24038/2009
PA	CY147928	H1N1	04/28/2009	Mexico	A/Mexico/24057/2009
PA	CY147744	H1N1	04/28/2009	Mexico	A/Mexico/24027/2009
PA	CY147752	H1N1	04/28/2009	Mexico	A/Mexico/24028/2009
PA	CY147936	H1N1	04/28/2009	Mexico	A/Mexico/24058/2009
PA	CY074112	H1N1	04/28/2009	USA	A/California/VRDL192/2009(H1N1)
PA	CY074088	H1N1	04/28/2009	USA	A/California/VRDL180/2009(H1N1)
PA	CY074152	H1N1	04/28/2009	USA	A/California/VRDL206/2009(H1N1)
PA	CY074176	H1N1	04/28/2009	USA	A/California/VRDL215/2009(H1N1)
PA	CY147808	H1N1	04/29/2009	Mexico	A/Mexico/24040/2009
PA	CY086974	H1N1	04/29/2009	USA	A/New York/3198/2009(H1N1)
PA	HM567551	H1N1	04/29/2009	United Ki	A/England/213/2009
PA	CY092342	H1N1	04/29/2009	USA	A/California/VRDL190/2009
PA	CY065261	H1N1	04/29/2009	United Ki	A/England/XFL00137/2009
PA	CY065341	H1N1	04/29/2009	United Ki	A/England/223/2009
PA	CY147800	H1N1	04/29/2009	Mexico	A/Mexico/24039/2009
PA	CY147760	H1N1	04/29/2009	Mexico	A/Mexico/24030/2009
PA	CY073866	H1N1	04/29/2009	USA	A/California/VRDL229/2009(H1N1)
PA	CY074184	H1N1	04/29/2009	USA	A/California/VRDL217/2009(H1N1)
PA	CY074192	H1N1	04/29/2009	USA	A/California/VRDL218/2009(H1N1)
PA	CY074216	H1N1	04/29/2009	USA	A/California/VRDL221/2009(H1N1)
PA	CY074272	H1N1	04/29/2009	USA	A/California/VRDL228/2009(H1N1)
PA	CY074360	H1N1	04/29/2009	USA	A/California/VRDL252/2009(H1N1)
PA	CY074200	H1N1	04/29/2009	USA	A/California/VRDL219/2009(H1N1)
PA	CY074376	H1N1	04/29/2009	USA	A/California/VRDL256/2009(H1N1)
PA	CY074168	H1N1	04/29/2009	USA	A/California/VRDL213/2009(H1N1)
PA	CY074232	H1N1	04/29/2009	USA	A/California/VRDL223/2009(H1N1)
PA	CY074264	H1N1	04/29/2009	USA	A/California/VRDL227/2009(H1N1)
PA	CY074368	H1N1	04/29/2009	USA	A/California/VRDL254/2009(H1N1)
PA	CY074224	H1N1	04/29/2009	USA	A/California/VRDL222/2009(H1N1)
PA	CY074352	H1N1	04/29/2009	USA	A/California/VRDL251/2009(H1N1)
PA	CY074104	H1N1	04/29/2009	USA	A/California/VRDL189/2009(H1N1)
PA	CY074240	H1N1	04/29/2009	USA	A/California/VRDL224/2009(H1N1)
PA	CY074256	H1N1	04/29/2009	USA	A/California/VRDL226/2009(H1N1)
PA	CY074296	H1N1	04/29/2009	USA	A/California/VRDL236/2009(H1N1)
PA	CY074312	H1N1	04/29/2009	USA	A/California/VRDL238/2009(H1N1)
PA	CY074320	H1N1	04/29/2009	USA	A/California/VRDL239/2009(H1N1)
PA	CY074120	H1N1	04/29/2009	USA	A/California/VRDL193/2009(H1N1)
PA	CY074208	H1N1	04/29/2009	USA	A/California/VRDL220/2009(H1N1)
PA	CY074288	H1N1	04/29/2009	USA	A/California/VRDL235/2009(H1N1)
PA	CY074336	H1N1	04/29/2009	USA	A/California/VRDL245/2009(H1N1)
PA	CY074096	H1N1	04/29/2009	USA	A/California/VRDL186/2009(H1N1)
PA	CY074144	H1N1	04/29/2009	USA	A/California/VRDL202/2009(H1N1)
PA	CY074160	H1N1	04/29/2009	USA	A/California/VRDL209/2009(H1N1)
PA	CY074304	H1N1	04/29/2009	USA	A/California/VRDL237/2009(H1N1)
PA	CY074344	H1N1	04/29/2009	USA	A/California/VRDL249/2009(H1N1)
PA	CY073401	H1N1	04/29/2009	USA	A/Brawley/WR1636P/2009(H1N1)

PA	CY073842	H1N1	04/29/2009	USA	A/California/VRDL163/2009
PA	CY041167	H1N1	04/29/2009	USA	A/New York/3185/2009(H1N1)
PA	CY040867	H1N1	04/29/2009	USA	A/New York/3265/2009(H1N1)
PA	CY041487	H1N1	04/29/2009	USA	A/New York/3189/2009(H1N1)
PA	GQ465735	H1N1	04/29/2009	Canada	A/Canada-NS/RV1554/2009(H1N1)
PA	CY041731	H1N1	04/29/2009	USA	A/New York/3247/2009(H1N1)
PA	CY040036	H1N1	04/29/2009	USA	A/New York/3099/2009(H1N1)
PA	CY046304	H1N1	04/29/2009	USA	A/Wisconsin/629-D01486/2009(H1N1)
PA	CY046312	H1N1	04/29/2009	USA	A/Wisconsin/629-D01642/2009(H1N1)
PA	CY047315	H1N1	04/29/2009	USA	A/New York/3074/2009(H1N1)
PA	CY050553	H1N1	04/29/2009	USA	A/New York/3095/2009(H1N1)
PA	GQ402289	H1N1	04/29/2009	Mexico	A/Mexico/InDRE13494/2009(H1N1)
PA	CY040835	H1N1	04/29/2009	USA	A/New York/3261/2009(H1N1)
PA	GQ329081	H1N1	04/29/2009	France	A/Paris/2573/2009(H1N1)
PA	CY041199	H1N1	04/29/2009	USA	A/New York/3190/2009(H1N1)
PA	GQ402290	H1N1	04/29/2009	Mexico	A/Mexico/InDRE13495/2009(H1N1)
PA	GQ465734	H1N1	04/29/2009	Canada	A/Canada-NS/RV1551/2009(H1N1)
PA	CY040859	H1N1	04/29/2009	USA	A/New York/3264/2009(H1N1)
PA	GQ200253	H1N1	04/29/2009	USA	A/Maryland/05/2009(H1N1)
PA	CY046942	H1N1	04/29/2009	Netherlar	A/Netherlands/602/2009
PA	GQ402293	H1N1	04/29/2009	Mexico	A/Mexico/InDRE13555/2009(H1N1)
PA	CY041626	H1N1	04/30/2009	USA	A/New York/3100/2009(H1N1)
PA	GQ222418	H1N1	04/30/2009	France	A/Paris/2590/2009(H1N1)
PA	GU931809	H1N1	04/30/2009	Hong Kong	A/Hong Kong/415742/2009(H1N1)
PA	HM567559	H1N1	04/30/2009	United Ki	A/England/217/2009
PA	CY065277	H1N1	04/30/2009	United Ki	A/England/215/2009
PA	CY065285	H1N1	04/30/2009	United Ki	A/England/216/2009
PA	CY065269	H1N1	04/30/2009	United Ki	A/England/214/2009
PA	CY147555	H1N1	04/30/2009	Mexico	A/Mexico/24029/2009
PA	CY074456	H1N1	04/30/2009	USA	A/California/VRDL283/2009(H1N1)
PA	CY074496	H1N1	04/30/2009	USA	A/California/VRDL295/2009(H1N1)
PA	CY074424	H1N1	04/30/2009	USA	A/California/VRDL275/2009(H1N1)
PA	CY074432	H1N1	04/30/2009	USA	A/California/VRDL276/2009(H1N1)
PA	CY074440	H1N1	04/30/2009	USA	A/California/VRDL280/2009(H1N1)
PA	CY074480	H1N1	04/30/2009	USA	A/California/VRDL290/2009(H1N1)
PA	CY074488	H1N1	04/30/2009	USA	A/California/VRDL292/2009(H1N1)
PA	CY074504	H1N1	04/30/2009	USA	A/California/VRDL297/2009(H1N1)
PA	CY074528	H1N1	04/30/2009	USA	A/California/VRDL300/2009(H1N1)
PA	CY074536	H1N1	04/30/2009	USA	A/California/VRDL301/2009(H1N1)
PA	CY074328	H1N1	04/30/2009	USA	A/California/VRDL244/2009(H1N1)
PA	CY074136	H1N1	04/30/2009	USA	A/California/VRDL200/2009(H1N1)
PA	CY074408	H1N1	04/30/2009	USA	A/California/VRDL270/2009(H1N1)
PA	CY074416	H1N1	04/30/2009	USA	A/California/VRDL273/2009(H1N1)
PA	CY074512	H1N1	04/30/2009	USA	A/California/VRDL298/2009(H1N1)
PA	CY073349	H1N1	04/30/2009	USA	A/San Diego/WR1629P/2009(H1N1)
PA	CY073365	H1N1	04/30/2009	USA	A/San Diego/WR1631P/2009(H1N1)
PA	CY064713	H1N1	04/30/2009	Mexico	A/Mexico City/020/2009
PA	CY064745	H1N1	04/30/2009	Mexico	A/Mexico City/024/2009(H1N1)
PA	CY064705	H1N1	04/30/2009	Mexico	A/Mexico City/019/2009
PA	GQ465741	H1N1	04/30/2009	Canada	A/Canada-SK/RV1788/2009(H1N1)
PA	CY046392	H1N1	04/30/2009	USA	A/Wisconsin/629-D00750/2009(H1N1)
PA	CY046424	H1N1	04/30/2009	USA	A/Wisconsin/629-D00767/2009(H1N1)
PA	CY046416	H1N1	04/30/2009	USA	A/Wisconsin/629-D00447/2009(H1N1)
PA	CY046520	H1N1	04/30/2009	USA	A/Wisconsin/629-D02141/2009(H1N1)

PA	CY046480	H1N1	04/30/2009	USA	A/Wisconsin/629-D00015/2009(H1N1)
PA	CY046264	H1N1	04/30/2009	USA	A/Wisconsin/629-D01773/2009(H1N1)
PA	CY046360	H1N1	04/30/2009	USA	A/Wisconsin/629-D01529/2009(H1N1)
PA	CY046456	H1N1	04/30/2009	USA	A/Wisconsin/629-D01558/2009(H1N1)
PA	CY046848	H1N1	04/30/2009	USA	A/Wisconsin/629-D02361/2009(H1N1)
PA	CY047355	H1N1	04/30/2009	USA	A/New York/3365/2009(H1N1)
PA	CY049827	H1N1	04/30/2009	USA	A/Cherry Point/WR0080/2009(H1N1)
PA	CY040698	H1N1	04/30/2009	USA	A/New York/3232/2009(H1N1)
PA	GQ465736	H1N1	04/30/2009	Canada	A/Canada-NS/RV1565/2009(H1N1)
PA	GQ222420	H1N1	04/30/2009	France	A/Paris/2580/2009(H1N1)
PA	CY040650	H1N1	04/30/2009	USA	A/New York/3194/2009(H1N1)
PA	CY040041	H1N1	05/2009	Thailand	A/Nonthaburi/102/2009
PA	CY107557	H1N1	05/2009	United Ki	A/Scotland/Glasgow_412118/2009
PA	CY107184	H1N1	05/2009	United Ki	A/Scotland/Paisley_409499/2009
PA	GQ254712	H1N1	05/01/2009	France	A/Paris/2591/2009(H1N1)
PA	CY074384	H1N1	05/01/2009	USA	A/California/VRDL258/2009(H1N1)
PA	CY041540	H1N1	05/01/2009	USA	A/Tennessee/1-560/2009(H1N1)
PA	CY041183	H1N1	05/01/2009	USA	A/New York/3187/2009(H1N1)
PA	CY046320	H1N1	05/01/2009	USA	A/Wisconsin/629-D01308/2009(H1N1)
PA	CY046464	H1N1	05/01/2009	USA	A/Wisconsin/629-D01861/2009(H1N1)
PA	CY046432	H1N1	05/01/2009	USA	A/Wisconsin/629-D00677/2009(H1N1)
PA	CY046976	H1N1	05/01/2009	USA	A/New York/3443/2009(H1N1)
PA	CY047347	H1N1	05/01/2009	USA	A/New York/3345/2009(H1N1)
PA	CY041642	H1N1	05/01/2009	USA	A/New York/3307/2009(H1N1)
PA	CY046296	H1N1	05/01/2009	USA	A/Wisconsin/629-D01817/2009(H1N1)
PA	CY046496	H1N1	05/01/2009	USA	A/Wisconsin/629-D02028/2009(H1N1)
PA	GQ465739	H1N1	05/01/2009	Canada	A/Canada-AB/RV1644/2009(H1N1)
PA	CY074472	H1N1	05/01/2009	USA	A/California/VRDL289/2009(H1N1)
PA	CY074520	H1N1	05/01/2009	USA	A/California/VRDL299/2009(H1N1)
PA	CY053100	H1N1	05/01/2009	USA	A/Houston/150S/2009(H1N1)
PA	CY053092	H1N1	05/01/2009	USA	A/Houston/14H/2009(H1N1)
PA	CY064753	H1N1	05/01/2009	Mexico	A/Mexico City/025/2009(H1N1)
PA	CY064737	H1N1	05/01/2009	Mexico	A/Mexico City/023/2009(H1N1)
PA	CY064721	H1N1	05/01/2009	Mexico	A/Mexico City/021/2009
PA	CY053084	H1N1	05/01/2009	USA	A/Houston/140S/2009H1N1)
PA	CY053108	H1N1	05/01/2009	USA	A/Houston/15H/2009(H1N1)
PA	CY046192	H1N1	05/01/2009	USA	A/Wisconsin/629-D01793/2009(H1N1)
PA	CY073377	H1N1	05/01/2009	USA	A/San Diego/WR1633P/2009(H1N1)
PA	GQ222424	H1N1	05/01/2009	France	A/Paris/2592/2009(H1N1)
PA	GQ365665	H1N1	05/02/2009	Germany	A/Brandenburg/19/2009(H1N1)
PA	CY073385	H1N1	05/02/2009	USA	A/San Diego/WR1634P/2009(H1N1)
PA	GQ323501	H1N1	05/02/2009	USA	A/Michigan/06/2009(H1N1)
PA	GQ160812	H1N1	05/02/2009	South Ko	A/Korea/01/2009
PA	CY046208	H1N1	05/02/2009	USA	A/Wisconsin/629-D02408/2009(H1N1)
PA	CY046856	H1N1	05/02/2009	USA	A/Wisconsin/629-D00244/2009(H1N1)
PA	CY046200	H1N1	05/02/2009	USA	A/Wisconsin/629-D02312/2009(H1N1)
PA	CY046272	H1N1	05/02/2009	USA	A/Wisconsin/629-D00933/2009(H1N1)
PA	CY049874	H1N1	05/02/2009	USA	A/Cherry Point/WR0102/2009(H1N1)
PA	CY046400	H1N1	05/02/2009	USA	A/Wisconsin/629-D01189/2009(H1N1)
PA	CY046344	H1N1	05/02/2009	USA	A/Wisconsin/629-D02455/2009(H1N1)
PA	CY041811	H1N1	05/02/2009	USA	A/New York/3408/2009(H1N1)
PA	CY041602	H1N1	05/02/2009	USA	A/New York/3177/2009(H1N1)
PA	CY074544	H1N1	05/02/2009	USA	A/California/VRDL311/2009(H1N1)
PA	CY074464	H1N1	05/02/2009	USA	A/California/VRDL284/2009(H1N1)

PA	CY064761	H1N1	05/02/2009	Mexico	A/Mexico City/026/2009(H1N1)
PA	CY064417	H1N1	05/02/2009	Mexico	A/Mexico city/CIA11/2009(H1N1)
PA	CY064729	H1N1	05/02/2009	Mexico	A/Mexico City/022/2009
PA	CY049858	H1N1	05/02/2009	USA	A/Cherry Point/WR0100/2009(H1N1)
PA	CY049866	H1N1	05/02/2009	USA	A/Cherry Point/WR0101/2009(H1N1)
PA	CY046240	H1N1	05/02/2009	USA	A/Wisconsin/629-D01735/2009(H1N1)
PA	CY046384	H1N1	05/02/2009	USA	A/Wisconsin/629-D01980/2009(H1N1)
PA	CY046440	H1N1	05/02/2009	USA	A/Wisconsin/629-D02262/2009(H1N1)
PA	HM567567	H1N1	05/02/2009	United Ki	A/England/221/2009
PA	CY069780	H1N1	05/02/2009	United Ki	A/England/221/2009
PA	CY065325	H1N1	05/02/2009	United Ki	A/England/220/2009
PA	CY065309	H1N1	05/02/2009	United Ki	A/England/246/2009
PA	CY065333	H1N1	05/02/2009	United Ki	A/England/201/2009
PA	CY065317	H1N1	05/02/2009	United Ki	A/England/219/2009
PA	CY065301	H1N1	05/02/2009	United Ki	A/England/218/2009
PA	CY073425	H1N1	05/02/2009	USA	A/San Diego/WR1641P/2009(H1N1)
PA	CY041079	H1N1	05/02/2009	USA	A/New York/3170/2009(H1N1)
PA	GQ251034	H1N1	05/03/2009	Italy	A/Italy/05/2009(H1N1)
PA	CY046232	H1N1	05/03/2009	USA	A/Wisconsin/629-D01296/2009(H1N1)
PA	CY046288	H1N1	05/03/2009	USA	A/Wisconsin/629-D02473/2009(H1N1)
PA	CY046888	H1N1	05/03/2009	USA	A/Wisconsin/629-D00498/2009(H1N1)
PA	CY046352	H1N1	05/03/2009	USA	A/Wisconsin/629-D01017/2009(H1N1)
PA	CY046248	H1N1	05/03/2009	USA	A/Wisconsin/629-D01606/2009(H1N1)
PA	CY046376	H1N1	05/03/2009	USA	A/Wisconsin/629-D01705/2009(H1N1)
PA	CY064410	H1N1	05/03/2009	Mexico	A/Mexico city/CIA10/2009(H1N1)
PA	CY046280	H1N1	05/03/2009	USA	A/Wisconsin/629-D01092/2009(H1N1)
PA	CY065293	H1N1	05/03/2009	United Ki	A/Scotland/4/2009
PA	CY069788	H1N1	05/03/2009	United Ki	A/England/222/2009
PA	CY065349	H1N1	05/03/2009	United Ki	A/England/247/2009
PA	CY046216	H1N1	05/03/2009	USA	A/Wisconsin/629-D00869/2009(H1N1)
PA	GQ323467	H1N1	05/04/2009	USA	A/Kentucky/07/2009(H1N1)
PA	GQ402296	H1N1	05/04/2009	Canada	A/Canada-SK/RV1767/2009(H1N1)
PA	CY043208	H1N1	05/04/2009	USA	A/New York/3338/2009(H1N1)
PA	CY041650	H1N1	05/04/2009	USA	A/New York/3313/2009(H1N1)
PA	CY046488	H1N1	05/04/2009	USA	A/Wisconsin/629-D00349/2009(H1N1)
PA	CY046512	H1N1	05/04/2009	USA	A/Wisconsin/629-D02425/2009(H1N1)
PA	CY046504	H1N1	05/04/2009	USA	A/Wisconsin/629-D00378/2009(H1N1)
PA	CY046880	H1N1	05/04/2009	USA	A/Wisconsin/629-D00396/2009(H1N1)
PA	CY046256	H1N1	05/04/2009	USA	A/Wisconsin/629-D02432/2009(H1N1)
PA	CY045481	H1N1	05/04/2009	Germany	A/Sachsen-Anhalt/101/2009(H1N1)
PA	CY046864	H1N1	05/04/2009	USA	A/Wisconsin/629-D01415/2009(H1N1)
PA	CY049842	H1N1	05/04/2009	USA	A/Craven/WR0093/2009(H1N1)
PA	CY046448	H1N1	05/04/2009	USA	A/Wisconsin/629-D01226/2009(H1N1)
PA	CY046528	H1N1	05/04/2009	USA	A/Wisconsin/629-D00183/2009(H1N1)
PA	CY046896	H1N1	05/04/2009	USA	A/Wisconsin/629-D01880/2009(H1N1)
PA	CY046224	H1N1	05/04/2009	USA	A/Wisconsin/629-D01251/2009(H1N1)
PA	CY046368	H1N1	05/04/2009	USA	A/Wisconsin/629-D00790/2009(H1N1)
PA	CY046408	H1N1	05/04/2009	USA	A/Wisconsin/629-D00807/2009(H1N1)
PA	CY046472	H1N1	05/04/2009	USA	A/Wisconsin/629-D01962/2009(H1N1)
PA	CY046872	H1N1	05/04/2009	USA	A/Wisconsin/629-D02272/2009(H1N1)
PA	CY046328	H1N1	05/04/2009	USA	A/Wisconsin/629-D01810/2009(H1N1)
PA	CY046920	H1N1	05/04/2009	USA	A/Wisconsin/629-D01764/2009(H1N1)
PA	CY046536	H1N1	05/04/2009	USA	A/Wisconsin/629-D00714/2009(H1N1)
PA	CY041787	H1N1	05/04/2009	USA	A/New York/3337/2009(H1N1)

PA	CY043176	H1N1	05/04/2009	USA	A/New York/3460/2009(H1N1)
PA	CY074560	H1N1	05/04/2009	USA	A/California/VRDL324/2009(H1N1)
PA	CY053155	H1N1	05/04/2009	USA	A/Houston/22H/2009(H1N1)
PA	CY053147	H1N1	05/04/2009	USA	A/Houston/22OS/2009(H1N1)
PA	CY050147	H1N1	05/04/2009	USA	A/Wisconsin/629-D01058/2009(H1N1)
PA	CY069820	H1N1	05/04/2009	United Ki	A/England/250/2009
PA	CY069804	H1N1	05/04/2009	United Ki	A/England/XFL00230/2009
PA	CY065357	H1N1	05/04/2009	United Ki	A/England/XFL00200/2009
PA	CY049819	H1N1	05/05/2009	USA	A/Craven/WR0019/2009(H1N1)
PA	CY074448	H1N1	05/05/2009	USA	A/California/VRDL281/2009(H1N1)
PA	CY065413	H1N1	05/05/2009	United Ki	A/England/254/2009
PA	CY069796	H1N1	05/05/2009	United Ki	A/England/XFL00228/2009
PA	CY069812	H1N1	05/05/2009	United Ki	A/England/249/2009
PA	HM567575	H1N1	05/05/2009	United Ki	A/England/249/2009
PA	CY065365	H1N1	05/05/2009	United Ki	A/England/XFL00275/2009
PA	CY065381	H1N1	05/05/2009	United Ki	A/England/XFL00304/2009
PA	CY069828	H1N1	05/05/2009	United Ki	A/England/XFL00303/2009
PA	GQ329110	H1N1	05/06/2009	France	A/Strasbourg/2609/2009(H1N1)
PA	GQ169383	H1N1	05/06/2009	Thailand	A/Thailand/104/2009
PA	CY041827	H1N1	05/06/2009	USA	A/New York/3352/2009(H1N1)
PA	CY043200	H1N1	05/06/2009	USA	A/New York/3324/2009(H1N1)
PA	CY045489	H1N1	05/06/2009	Germany	A/Sachsen-Anhalt/97/2009(H1N1)
PA	CY047339	H1N1	05/06/2009	USA	A/New York/3364/2009(H1N1)
PA	CY046560	H1N1	05/06/2009	USA	A/Wisconsin/629-D00228/2009(H1N1)
PA	GQ402298	H1N1	05/06/2009	Canada	A/Canada-SK/RV1798/2009(H1N1)
PA	CY041819	H1N1	05/06/2009	USA	A/New York/3351/2009(H1N1)
PA	CY041755	H1N1	05/06/2009	USA	A/New York/3323/2009(H1N1)
PA	GQ465743	H1N1	05/07/2009	Canada	A/Canada-SK/RV1797/2009(H1N1)
PA	CY065373	H1N1	05/07/2009	United Ki	A/England/XFL00300/2009
PA	GQ402295	H1N1	05/07/2009	Canada	A/Canada-QC/RV1759/2009(H1N1)
PA	GQ323492	H1N1	05/07/2009	USA	A/Arkansas/03/2009(H1N1)
PA	CY046608	H1N1	05/07/2009	USA	A/Wisconsin/629-D00487/2009(H1N1)
PA	GQ465742	H1N1	05/07/2009	Canada	A/Canada-SK/RV1794/2009(H1N1)
PA	CY074552	H1N1	05/07/2009	USA	A/California/VRDL320/2009(H1N1)
PA	CY050203	H1N1	05/07/2009	Mexico	A/Mexico City/001/2009
PA	CY065405	H1N1	05/07/2009	United Ki	A/England/253/2009
PA	CY100447	H1N1	05/07/2009	Mexico	A/Mexico City/IBT22/2009
PA	CY100455	H1N1	05/07/2009	Mexico	A/Mexico City/IBT23/2009
PA	KC782030	H1N1	05/07/2009	USA	A/Washington/51/2009
PA	CY065397	H1N1	05/07/2009	United Ki	A/England/XFL00311/2009
PA	GQ323506	H1N1	05/07/2009	USA	A/Washington/14/2009(H1N1)
PA	GQ465740	H1N1	05/08/2009	Canada	A/Canada-PQ/RV1758/2009(H1N1)
PA	CY073357	H1N1	05/08/2009	USA	A/San Diego/WR1630P/2009(H1N1)
PA	CY046592	H1N1	05/08/2009	USA	A/Wisconsin/629-D02452/2009(H1N1)
PA	CY046584	H1N1	05/08/2009	USA	A/Wisconsin/629-D01909/2009(H1N1)
PA	CY046544	H1N1	05/08/2009	USA	A/Wisconsin/629-D01313/2009(H1N1)
PA	CY046576	H1N1	05/08/2009	USA	A/Wisconsin/629-D00905/2009(H1N1)
PA	GQ402297	H1N1	05/08/2009	Canada	A/Canada-SK/RV1793/2009(H1N1)
PA	CY043128	H1N1	05/08/2009	USA	A/New York/3389/2009(H1N1)
PA	CY041771	H1N1	05/08/2009	USA	A/New York/3354/2009(H1N1)
PA	CY074592	H1N1	05/08/2009	USA	A/California/VRDL348/2009(H1N1)
PA	CY074584	H1N1	05/08/2009	USA	A/California/VRDL346/2009(H1N1)
PA	CY050219	H1N1	05/08/2009	Mexico	A/Mexico City/003/2009(H1N1)
PA	CY050227	H1N1	05/08/2009	Mexico	A/Mexico City/004/2009(H1N1)

PA	CY050211	H1N1	05/08/2009	Mexico	A/Mexico City/002/2009(H1N1)
PA	CY050884	H1N1	05/08/2009	Mexico	A/Mexico City/005/2009(H1N1)
PA	CY065389	H1N1	05/08/2009	United Ki	A/England/252/2009
PA	CY069836	H1N1	05/08/2009	United Ki	A/England/XFL00364/2009
PA	CY065429	H1N1	05/08/2009	United Ki	A/England/256/2009
PA	GQ169305	H1N1	05/08/2009	Japan	A/Narita/1/2009
PA	CY046568	H1N1	05/09/2009	USA	A/Wisconsin/629-D01919/2009(H1N1)
PA	CY046616	H1N1	05/09/2009	USA	A/Wisconsin/629-D01055/2009(H1N1)
PA	CY062640	H1N1	05/09/2009	Mexico	A/Mexico City/014/2009
PA	CY050243	H1N1	05/09/2009	Mexico	A/Mexico City/010/2009
PA	CY050251	H1N1	05/09/2009	Mexico	A/Mexico City/011/2009
PA	CY050235	H1N1	05/09/2009	Mexico	A/Mexico City/006/2009(H1N1)
PA	CY050259	H1N1	05/09/2009	Mexico	A/Mexico City/012/2009
PA	CY050179	H1N1	05/09/2009	Mexico	A/Mexico City/015/2009
PA	CY050777	H1N1	05/09/2009	USA	A/New York/3442/2009(H1N1)
PA	CY050852	H1N1	05/09/2009	Mexico	A/Mexico City/007/2009(H1N1)
PA	CY050868	H1N1	05/09/2009	Mexico	A/Mexico City/013/2009
PA	CY050876	H1N1	05/09/2009	Mexico	A/Mexico City/017/2009
PA	CY069844	H1N1	05/09/2009	United Ki	A/England/XFL00376/2009
PA	CY092106	H1N1	05/09/2009	Mexico	A/Mexico City/016/2009
PA	CY065437	H1N1	05/09/2009	United Ki	A/England/257/2009
PA	CY045945	H1N1	05/09/2009	Canada	A/Toronto/C2781/2009(H1N1)
PA	GQ323536	H1N1	05/10/2009	USA	A/Missouri/04/2009(H1N1)
PA	GQ200290	H1N1	05/10/2009	China	A/Shandong/1/2009(H1N1)
PA	CY065421	H1N1	05/10/2009	United Ki	A/England/XFL00354/2009
PA	JQ173155	H1N1	05/10/2009	Finland	A/Helsinki/P15/2009
PA	CY074568	H1N1	05/10/2009	USA	A/California/VRDL326/2009(H1N1)
PA	GQ166226	H1N1	05/10/2009	China	A/Sichuan/1/2009(H1N1)
PA	CY050892	H1N1	05/10/2009	Mexico	A/Mexico City/018/2009
PA	CY046600	H1N1	05/10/2009	USA	A/Wisconsin/629-D01482/2009(H1N1)
PA	CY050449	H1N1	05/10/2009	USA	A/Wisconsin/629-D01482/2009(H1N1)
PA	CY046552	H1N1	05/10/2009	USA	A/Wisconsin/629-D00179/2009(H1N1)
PA	GQ328864	H1N1	05/10/2009	Finland	A/Finland/553/2009(H1N1)
PA	CY050171	H1N1	05/10/2009	Mexico	A/Mexico City/008/2009(H1N1)
PA	CY041763	H1N1	05/11/2009	USA	A/New York/3413/2009(H1N1)
PA	CY043224	H1N1	05/11/2009	USA	A/New York/3455/2009(H1N1)
PA	CY065445	H1N1	05/11/2009	United Ki	A/England/259/2009
PA	CY069852	H1N1	05/11/2009	United Ki	A/England/260/2009
PA	CY065453	H1N1	05/11/2009	United Ki	A/England/XFL00418/2009
PA	GQ254715	H1N1	05/11/2009	France	A/Paris/2629/2009(H1N1)
PA	CY043168	H1N1	05/11/2009	USA	A/New York/3454/2009(H1N1)
PA	CY065493	H1N1	05/12/2009	United Ki	A/England/265/2009
PA	CY065485	H1N1	05/12/2009	United Ki	A/England/264/2009
PA	CY043160	H1N1	05/12/2009	USA	A/New York/3418/2009(H1N1)
PA	CY050333	H1N1	05/12/2009	USA	A/Wisconsin/629-D01851/2009(H1N1)
PA	CY065469	H1N1	05/12/2009	United Ki	A/England/XFL00473/2009
PA	CY043304	H1N1	05/12/2009	USA	A/New York/3498/2009(H1N1)
PA	CY065477	H1N1	05/12/2009	United Ki	A/England/263/2009
PA	CY069860	H1N1	05/12/2009	United Ki	A/England/XFL00421/2009
PA	CY050860	H1N1	05/12/2009	Mexico	A/Mexico City/009/2009(H1N1)
PA	CY065461	H1N1	05/12/2009	United Ki	A/England/XFL00419/2009
PA	CY043184	H1N1	05/12/2009	USA	A/New York/3488/2009(H1N1)
PA	KC781319	H1N1	05/13/2009	USA	A/Mississippi/01/2009
PA	CY043288	H1N1	05/13/2009	USA	A/New York/3449/2009(H1N1)

PA	CY065509	H1N1	05/13/2009	United Ki	A/England/XFL00482/2009
PA	CY046680	H1N1	05/13/2009	USA	A/Wisconsin/629-D01305/2009(H1N1)
PA	CY053060	H1N1	05/13/2009	USA	A/Houston/100S/2009(H1N1)
PA	CY051908	H1N1	05/13/2009	USA	A/Houston/10S/2009(H1N1)
PA	CY050761	H1N1	05/13/2009	USA	A/New York/3467/2009(H1N1)
PA	CY050377	H1N1	05/13/2009	USA	A/Wisconsin/629-D01154/2009(H1N1)
PA	CY053068	H1N1	05/13/2009	USA	A/Houston/10H/2009(H1N1)
PA	CY052964	H1N1	05/13/2009	USA	A/Houston/1H/2009(H1N1)
PA	CY043216	H1N1	05/13/2009	USA	A/New York/3420/2009(H1N1)
PA	CY065501	H1N1	05/13/2009	United Ki	A/England/XFL00481/2009
PA	CY046664	H1N1	05/14/2009	USA	A/Wisconsin/629-D00997/2009(H1N1)
PA	CY046656	H1N1	05/14/2009	USA	A/Wisconsin/629-D01837/2009(H1N1)
PA	CY043136	H1N1	05/14/2009	USA	A/New York/3514/2009(H1N1)
PA	CY046688	H1N1	05/14/2009	USA	A/Wisconsin/629-D00978/2009(H1N1)
PA	CY041795	H1N1	05/14/2009	USA	A/New York/3463/2009(H1N1)
PA	CY046672	H1N1	05/14/2009	USA	A/Wisconsin/629-D00064/2009(H1N1)
PA	GQ225372	H1N1	05/15/2009	China	A/Beijing/01/2009(H1N1)
PA	CY065517	H1N1	05/15/2009	United Ki	A/England/266/2009
PA	CY065525	H1N1	05/15/2009	United Ki	A/England/XFL00532/2009
PA	CY046632	H1N1	05/15/2009	USA	A/Wisconsin/629-D01839/2009(H1N1)
PA	CY050393	H1N1	05/15/2009	USA	A/Wisconsin/629-D01839/2009(H1N1)
PA	CY050385	H1N1	05/15/2009	USA	A/Wisconsin/629-D01295/2009(H1N1)
PA	CY044978	H1N1	05/15/2009	USA	A/New York/3744/2009(H1N1)
PA	CY083295	H1N1	05/15/2009	USA	A/San Diego/WRAIR1647P/2009
PA	GQ323476	H1N1	05/15/2009	USA	A/Utah/03/2009(H1N1)
PA	CY048924	H1N1	05/15/2009	Malaysia	A/Malaysia/820/2009(H1N1)
PA	CY062539	H1N1	05/16/2009	Mexico	A/Mexico city/CIA9/2009(H1N1)
PA	CY046624	H1N1	05/16/2009	USA	A/Wisconsin/629-D00575/2009(H1N1)
PA	CY046640	H1N1	05/16/2009	USA	A/Wisconsin/629-D00967/2009(H1N1)
PA	CY046648	H1N1	05/16/2009	USA	A/Wisconsin/629-D02227/2009(H1N1)
PA	GQ183630	H1N1	05/16/2009	China	A/Beijing/02/2009(H1N1)
PA	CY048932	H1N1	05/16/2009	Malaysia	A/Malaysia/854/2009(H1N1)
PA	CY044986	H1N1	05/16/2009	USA	A/New York/3747/2009(H1N1)
PA	CY046696	H1N1	05/16/2009	USA	A/Wisconsin/629-D00434/2009(H1N1)
PA	GQ222039	H1N1	05/16/2009	Japan	A/Osaka-C/1/2009(H1N1)
PA	CY043256	H1N1	05/16/2009	USA	A/New York/3545/2009(H1N1)
PA	GQ222038	H1N1	05/16/2009	Japan	A/Osaka/2/2009(H1N1)
PA	CY043192	H1N1	05/16/2009	USA	A/New York/3499/2009(H1N1)
PA	CY041779	H1N1	05/16/2009	USA	A/New York/3532/2009(H1N1)
PA	CY043248	H1N1	05/16/2009	USA	A/New York/3509/2009(H1N1)
PA	GQ222037	H1N1	05/16/2009	Japan	A/Osaka/1/2009(H1N1)
PA	CY043296	H1N1	05/16/2009	USA	A/New York/3469/2009(H1N1)
PA	GQ183622	H1N1	05/16/2009	China	A/Beijing/01/2009(H1N1)
PA	CY043240	H1N1	05/16/2009	USA	A/New York/3502/2009(H1N1)
PA	CY069868	H1N1	05/17/2009	United Ki	A/England/271/2009
PA	CY043312	H1N1	05/17/2009	USA	A/New York/3534/2009(H1N1)
PA	CY074576	H1N1	05/17/2009	USA	A/California/VRDL341/2009(H1N1)
PA	GQ221693	H1N1	05/17/2009	China	A/Guangdong/1/2009(H1N1)
PA	CY045090	H1N1	05/17/2009	USA	A/New York/3574/2009(H1N1)
PA	CY050401	H1N1	05/17/2009	USA	A/Wisconsin/629-D00989/2009(H1N1)
PA	GQ222040	H1N1	05/17/2009	Japan	A/Osaka-C/2/2009(H1N1)
PA	GQ222035	H1N1	05/17/2009	Japan	A/Hyogo/2/2009(H1N1)
PA	GQ402299	H1N1	05/17/2009	Canada	A/Canada-QC/RV1954/2009(H1N1)
PA	CY041803	H1N1	05/17/2009	USA	A/New York/3468/2009(H1N1)

PA	CY083287	H1N1	05/17/2009	USA	A/San Diego/WRAIR1646P/2009
PA	GQ222034	H1N1	05/17/2009	Japan	A/Hyogo/1/2009(H1N1)
PA	CY044133	H1N1	05/17/2009	USA	A/New York/3516/2009(H1N1)
PA	CY043232	H1N1	05/17/2009	USA	A/New York/3501/2009(H1N1)
PA	CY052972	H1N1	05/18/2009	USA	A/Houston/20S/2009(H1N1)
PA	CY052988	H1N1	05/18/2009	USA	A/Houston/30S/2009(H1N1)
PA	CY044914	H1N1	05/18/2009	USA	A/New York/3612/2009(H1N1)
PA	HQ011413	H1N1	05/18/2009	China	A/Guangdong/01/2009(H1N1)
PA	CY052996	H1N1	05/18/2009	USA	A/Houston/3H/2009(H1N1)
PA	CY044093	H1N1	05/18/2009	USA	A/New York/3740/2009(H1N1)
PA	GQ267836	H1N1	05/18/2009	Japan	A/Himeji/1/2009(H1N1)
PA	CY051916	H1N1	05/18/2009	USA	A/Houston/2H/2009(H1N1)
PA	GQ223443	H1N1	05/18/2009	China	A/Guangdong/1/2009(H1N1)
PA	CY043272	H1N1	05/19/2009	USA	A/New York/3563/2009(H1N1)
PA	CY044069	H1N1	05/19/2009	USA	A/New York/3568/2009(H1N1)
PA	CY044141	H1N1	05/19/2009	USA	A/New York/3528/2009(H1N1)
PA	GQ323517	H1N1	05/19/2009	USA	A/Arizona/09/2009(H1N1)
PA	GQ300864	H1N1	05/19/2009	Japan	A/Shiga/2/2009(H1N1)
PA	CY043144	H1N1	05/19/2009	USA	A/New York/3529/2009(H1N1)
PA	CY050417	H1N1	05/19/2009	USA	A/Wisconsin/629-D00752/2009(H1N1)
PA	CY046720	H1N1	05/19/2009	USA	A/Wisconsin/629-D00752/2009(H1N1)
PA	CY046760	H1N1	05/19/2009	USA	A/Wisconsin/629-D00160/2009(H1N1)
PA	CY045148	H1N1	05/19/2009	USA	A/New York/3577/2009(H1N1)
PA	CY046744	H1N1	05/19/2009	USA	A/Wisconsin/629-D00084/2009(H1N1)
PA	CY044195	H1N1	05/19/2009	Taiwan	A/Taiwan/T0724/2009(H1N1)
PA	CY044890	H1N1	05/19/2009	USA	A/New York/3580/2009(H1N1)
PA	CY047363	H1N1	05/19/2009	USA	A/New York/3741/2009(H1N1)
PA	CY046776	H1N1	05/19/2009	USA	A/Wisconsin/629-D00722/2009(H1N1)
PA	CY053012	H1N1	05/19/2009	USA	A/Houston/5H/2009(H1N1)
PA	CY053004	H1N1	05/19/2009	USA	A/Houston/50S/2009(H1N1)
PA	CY044922	H1N1	05/19/2009	USA	A/New York/3613/2009(H1N1)
PA	CY044053	H1N1	05/20/2009	USA	A/New York/3566/2009(H1N1)
PA	CY053020	H1N1	05/20/2009	USA	A/Houston/60S/2009(H1N1)
PA	CY053187	H1N1	05/20/2009	USA	A/Brownsville/270S/2009(H1N1)
PA	CY053179	H1N1	05/20/2009	USA	A/Brownsville/260S/2009(H1N1)
PA	CY053028	H1N1	05/20/2009	USA	A/Houston/6H/2009(H1N1)
PA	GQ225380	H1N1	05/20/2009	China	A/Beijing/3/2009(H1N1)
PA	GQ402300	H1N1	05/20/2009	Canada	A/Canada-MB/RV1964/2009(H1N1)
PA	CY044874	H1N1	05/20/2009	USA	A/New York/3571/2009(H1N1)
PA	CY044898	H1N1	05/20/2009	USA	A/New York/3582/2009(H1N1)
PA	CY044938	H1N1	05/20/2009	USA	A/New York/3623/2009(H1N1)
PA	CY046712	H1N1	05/20/2009	USA	A/Wisconsin/629-D01787/2009(H1N1)
PA	CY046736	H1N1	05/20/2009	USA	A/Wisconsin/629-D01521/2009(H1N1)
PA	CY046728	H1N1	05/20/2009	USA	A/Wisconsin/629-D02427/2009(H1N1)
PA	CY043152	H1N1	05/20/2009	USA	A/New York/3551/2009(H1N1)
PA	GQ290105	H1N1	05/20/2009	China	A/Beijing/502/2009(H1N1)
PA	CY044061	H1N1	05/20/2009	USA	A/New York/3567/2009(H1N1)
PA	CY043264	H1N1	05/20/2009	USA	A/New York/3552/2009(H1N1)
PA	CY053203	H1N1	05/21/2009	USA	A/Brownsville/300S/2009(H1N1)
PA	CY053195	H1N1	05/21/2009	USA	A/Brownsville/290S/2009(H1N1)
PA	GQ219588	H1N1	05/21/2009	Russia	A/Moscow/IIV01/2009(H1N1)
PA	GQ267849	H1N1	05/21/2009	Japan	A/Sakai/2/2009(H1N1)
PA	GQ246616	H1N1	05/21/2009	Russia	A/Moscow/01/2009(H1N1)
PA	GQ267843	H1N1	05/21/2009	Japan	A/Sakai/1/2009(H1N1)

PA	CY051924	H1N1	05/21/2009	USA	A/Houston/90S/2009(H1N1)
PA	CY053036	H1N1	05/21/2009	USA	A/Houston/70S/2009(H1N1)
PA	CY053044	H1N1	05/21/2009	USA	A/Houston/7H/2009(H1N1)
PA	GQ402303	H1N1	05/21/2009	Canada	A/Canada-MB/RV1982/2009(H1N1)
PA	CY053052	H1N1	05/21/2009	USA	A/Houston/9H/2009(H1N1)
PA	CY052980	H1N1	05/21/2009	USA	A/Houston/20S/2009(H1N1)
PA	CY089877	H1N1	05/21/2009	USA	A/Wisconsin/629-D02275/2009(H1N1)
PA	CY044970	H1N1	05/21/2009	USA	A/New York/3653/2009(H1N1)
PA	CY044882	H1N1	05/21/2009	USA	A/New York/3573/2009(H1N1)
PA	CY044906	H1N1	05/21/2009	USA	A/New York/3611/2009(H1N1)
PA	CY046968	H1N1	05/21/2009	USA	A/New York/3576/2009(H1N1)
PA	CY089184	H1N1	05/22/2009	Chile	A/Chile/19/2009
PA	CY046752	H1N1	05/22/2009	USA	A/Wisconsin/629-D02064/2009(H1N1)
PA	CY044211	H1N1	05/22/2009	Taiwan	A/Taiwan/T1339/2009(H1N1)
PA	CY046768	H1N1	05/22/2009	USA	A/Wisconsin/629-D02298/2009(H1N1)
PA	CY053219	H1N1	05/22/2009	USA	A/Brownsville/31H/2009(H1N1)
PA	CY045098	H1N1	05/22/2009	USA	A/New York/3614/2009(H1N1)
PA	GQ267855	H1N1	05/22/2009	Japan	A/Shiga/1/2009(H1N1)
PA	CY044954	H1N1	05/22/2009	USA	A/New York/3627/2009(H1N1)
PA	GQ402302	H1N1	05/22/2009	Canada	A/Canada-MB/RV1977/2009(H1N1)
PA	CY044203	H1N1	05/22/2009	Taiwan	A/Taiwan/T1338/2009(H1N1)
PA	GQ225364	H1N1	05/22/2009	China	A/Fujian/1/2009(H1N1)
PA	CY053211	H1N1	05/22/2009	USA	A/Brownsville/310S/2009(H1N1)
PA	GQ225356	H1N1	05/23/2009	China	A/Shanghai/1/2009(H1N1)
PA	GQ323528	H1N1	05/23/2009	USA	A/Illinois/04/2009(H1N1)
PA	GQ225348	H1N1	05/23/2009	China	A/Zhejiang/1/2009(H1N1)
PA	CY050409	H1N1	05/23/2009	USA	A/Wisconsin/629-D00451/2009(H1N1)
PA	CY050425	H1N1	05/23/2009	USA	A/Wisconsin/629-D02206/2009(H1N1)
PA	CY053132	H1N1	05/23/2009	USA	A/Houston/200S/2009(H1N1)
PA	CY053227	H1N1	05/23/2009	USA	A/Brownsville/340S/2009(H1N1)
PA	CY049914	H1N1	05/23/2009	Dominicar	A/Santo Domingo/0574T/2009(H1N1)
PA	CY049906	H1N1	05/23/2009	Dominicar	A/Santo Domingo/0574/2009(H1N1)
PA	GQ232092	H1N1	05/23/2009	China	A/Beijing/4/2009(H1N1)
PA	CY075088	H1N1	05/23/2009	Chile	A/Chile/28/2009(H1N1)
PA	CY053235	H1N1	05/23/2009	USA	A/Brownsville/34H/2009(H1N1)
PA	CY053140	H1N1	05/23/2009	USA	A/Houston/20H/2009(H1N1)
PA	GQ402287	H1N1	05/24/2009	Canada	A/Canada-MB/RV2023/2009(H1N1)
PA	GQ402301	H1N1	05/24/2009	Canada	A/Canada-MB/RV1975/2009(H1N1)
PA	CY041982	H1N1	05/24/2009	Dominicar	A/Santo Domingo/0573N/2009(H1N1)
PA	CY045937	H1N1	05/24/2009	Canada	A/Toronto/C0270/2009(H1N1)
PA	CY044962	H1N1	05/24/2009	USA	A/New York/3629/2009(H1N1)
PA	CY041959	H1N1	05/24/2009	Dominicar	A/Santo Domingo/572N/2009(H1N1)
PA	GQ253496	H1N1	05/24/2009	China	A/Shanghai/37T/2009(H1N1)
PA	HM567591	H1N1	05/24/2009	Ireland	A/Ireland/1/2009
PA	CY046704	H1N1	05/24/2009	USA	A/Wisconsin/629-D01445/2009(H1N1)
PA	CY050433	H1N1	05/25/2009	USA	A/Wisconsin/629-D02324/2009(H1N1)
PA	CY044930	H1N1	05/25/2009	USA	A/New York/3617/2009(H1N1)
PA	CY043280	H1N1	05/25/2009	USA	A/New York/3651/2009(H1N1)
PA	JQ409125	H1N1	05/25/2009	Finland	A/Helsinki/Vi1/2009(H1N1)
PA	CY075104	H1N1	05/25/2009	Chile	A/Chile/32/2009(H1N1)
PA	CY075128	H1N1	05/25/2009	Chile	A/Chile/42/2009(H1N1)
PA	CY075112	H1N1	05/25/2009	Chile	A/Chile/36/2009(H1N1)
PA	CY075096	H1N1	05/25/2009	Chile	A/Chile/31/2009
PA	CY075120	H1N1	05/25/2009	Chile	A/Chile/40/2009(H1N1)

PA	GQ385299	H1N1	05/26/2009	Canada	A/Toronto/0462/2009(H1N1)
PA	CY044077	H1N1	05/26/2009	USA	A/New York/3702/2009(H1N1)
PA	CY043109	H1N1	05/26/2009	Dominicar	A/Santo Domingo/568T/2009(H1N1)
PA	GQ247729	H1N1	05/26/2009	Russia	A/Moscow/IIV02/2009(H1N1)
PA	CY043101	H1N1	05/26/2009	Dominicar	A/Santo Domingo/565T/2009(H1N1)
PA	GQ375283	H1N1	05/26/2009	Russia	A/Moscow/03/2009(H1N1)
PA	CY050753	H1N1	05/26/2009	USA	A/New York/3768/2009(H1N1)
PA	CY051252	H1N1	05/26/2009	USA	A/Wisconsin/629-S0035/2009(H1N1)
PA	CY046960	H1N1	05/26/2009	USA	A/New York/3654/2009(H1N1)
PA	GU138962	H1N1	05/26/2009	Japan	A/Shizuoka-C/97/2009(H1N1)
PA	CY044946	H1N1	05/26/2009	USA	A/New York/3625/2009(H1N1)
PA	CY045156	H1N1	05/26/2009	USA	A/New York/3655/2009(H1N1)
PA	CY046784	H1N1	05/26/2009	USA	A/Wisconsin/629-D01894/2009(H1N1)
PA	HM567583	H1N1	05/26/2009	United Ki	A/England/303/2009
PA	HM567807	H1N1	05/26/2009	Ireland	A/Ireland/2/2009
PA	HM567831	H1N1	05/26/2009	Ireland	A/Ireland/3/2009
PA	HM567599	H1N1	05/26/2009	United Ki	A/England/313/2009
PA	CY075136	H1N1	05/26/2009	Chile	A/Chile/56/2009(H1N1)
PA	GQ328866	H1N1	05/26/2009	Finland	A/Finland/554/2009(H1N1)
PA	GQ232084	H1N1	05/27/2009	China	A/Guangdong/02/2009(H1N1)
PA	CY049930	H1N1	05/27/2009	USA	A/Craven/WR1001/2009(H1N1)
PA	CY050441	H1N1	05/27/2009	USA	A/Wisconsin/629-D01886/2009(H1N1)
PA	CY050900	H1N1	05/27/2009	USA	A/Wisconsin/629-D00949/2009(H1N1)
PA	CY046816	H1N1	05/27/2009	USA	A/Wisconsin/629-D00698/2009(H1N1)
PA	CY046840	H1N1	05/27/2009	USA	A/Wisconsin/629-D01883/2009(H1N1)
PA	CY046792	H1N1	05/27/2009	USA	A/Wisconsin/629-D02276/2009(H1N1)
PA	CY069876	H1N1	05/27/2009	United Ki	A/England/350/2009
PA	HM567687	H1N1	05/27/2009	United Ki	A/England/350/2009
PA	CY075144	H1N1	05/27/2009	Chile	A/Chile/88/2009(H1N1)
PA	CY075152	H1N1	05/27/2009	Chile	A/Chile/95/2009(H1N1)
PA	CY076754	H1N1	05/27/2009	Canada	A/Ontario/142358/2009(H1N1)
PA	CY053251	H1N1	05/27/2009	USA	A/Brownsville/35H/2009(H1N1)
PA	CY053243	H1N1	05/27/2009	USA	A/Brownsville/350S/2009(H1N1)
PA	CY053259	H1N1	05/27/2009	USA	A/Brownsville/36H/2009(H1N1)
PA	CY074002	H1N1	05/27/2009	Argentina	A/Argentina/HNRG14/2009(H1N1)
PA	GQ283483	H1N1	05/27/2009	Italy	A/Italy/49/2009(H1N1)
PA	GQ288371	H1N1	05/28/2009	China	A/Fuzhou/01/2009(H1N1)
PA	CY050155	H1N1	05/28/2009	USA	A/Wisconsin/629-D00117/2009(H1N1)
PA	GQ402304	H1N1	05/28/2009	Canada	A/Canada-MB/RV2013/2009(H1N1)
PA	CY050908	H1N1	05/28/2009	USA	A/Wisconsin/629-D01779/2009(H1N1)
PA	CY050916	H1N1	05/28/2009	USA	A/Wisconsin/629-D01262/2009(H1N1)
PA	CY046912	H1N1	05/28/2009	USA	A/Wisconsin/629-D01083/2009(H1N1)
PA	CY046904	H1N1	05/28/2009	USA	A/Wisconsin/629-D00829/2009(H1N1)
PA	CY049067	H1N1	05/28/2009	Singapore	A/Singapore/ON129/2009(H1N1)
PA	CY045010	H1N1	05/28/2009	USA	A/New York/3709/2009(H1N1)
PA	CY046832	H1N1	05/28/2009	USA	A/Wisconsin/629-D02144/2009(H1N1)
PA	CY046800	H1N1	05/28/2009	USA	A/Wisconsin/629-D01026/2009(H1N1)
PA	HM567607	H1N1	05/28/2009	United Ki	A/England/328/2009
PA	CY075160	H1N1	05/28/2009	Chile	A/Chile/158/2009(H1N1)
PA	CY053314	H1N1	05/28/2009	USA	A/Brownsville/370S/2009(H1N1)
PA	CY053266	H1N1	05/28/2009	USA	A/Brownsville/37H/2009(H1N1)
PA	CY064790	H1N1	05/28/2009	China	A/Guangdong/SB1/2009(H1N1)
PA	GQ290436	H1N1	05/28/2009	China	A/Shanghai/60T/2009(H1N1)
PA	CY044085	H1N1	05/29/2009	USA	A/New York/3715/2009(H1N1)

PA	GQ244322	H1N1	05/29/2009	China	A/Guangdong/05/2009(H1N1)
PA	GQ250160	H1N1	05/29/2009	China	A/Guangdong/03/2009(H1N1)
PA	CY050924	H1N1	05/29/2009	USA	A/Wisconsin/629-D02213/2009(H1N1)
PA	CY050940	H1N1	05/29/2009	USA	A/Wisconsin/629-D01847/2009(H1N1)
PA	CY075168	H1N1	05/29/2009	Chile	A/Chile/180/2009(H1N1)
PA	CY050932	H1N1	05/29/2009	USA	A/Wisconsin/629-D00426/2009(H1N1)
PA	GQ402305	H1N1	05/29/2009	Canada	A/Canada-MB/RV2018/2009(H1N1)
PA	HM567631	H1N1	05/29/2009	United Ki	A/England/342/2009
PA	HM567839	H1N1	05/29/2009	Ireland	A/Ireland/4/2009
PA	CY071036	H1N1	05/29/2009	USA	A/New York/NHRC0002/2009(H1N1)
PA	CY051260	H1N1	05/29/2009	USA	A/Wisconsin/629-S0086/2009(H1N1)
PA	CY089885	H1N1	05/30/2009	USA	A/Wisconsin/629-D01829/2009(H1N1)
PA	HM567647	H1N1	05/30/2009	United Ki	A/England/345/2009
PA	CY044227	H1N1	05/30/2009	Taiwan	A/Taiwan/T1821/2009(H1N1)
PA	GQ402306	H1N1	05/30/2009	Canada	A/Canada-MB/RV2020/2009(H1N1)
PA	CY046824	H1N1	05/30/2009	USA	A/Wisconsin/629-D02013/2009(H1N1)
PA	CY046808	H1N1	05/30/2009	USA	A/Wisconsin/629-D00223/2009(H1N1)
PA	CY045961	H1N1	05/30/2009	Canada	A/Toronto/T5294/2009(H1N1)
PA	HM569664	H1N1	05/30/2009	Argentina	A/Argentina/07-09GP/2009(H1N1)
PA	CY055302	H1N1	05/30/2009	Singapore	A/Singapore/GN285/2009(H1N1)
PA	CY053282	H1N1	05/30/2009	USA	A/Brownsville/39H/2009(H1N1)
PA	CY053274	H1N1	05/30/2009	USA	A/Brownsville/39OS/2009(H1N1)
PA	HM567639	H1N1	05/30/2009	United Ki	A/England/343/2009
PA	HM567663	H1N1	05/30/2009	United Ki	A/England/347/2009
PA	HM567615	H1N1	05/30/2009	United Ki	A/England/337/2009
PA	HM567655	H1N1	05/30/2009	United Ki	A/England/346/2009
PA	HM567671	H1N1	05/30/2009	United Ki	A/England/348/2009
PA	HM567623	H1N1	05/30/2009	United Ki	A/England/341/2009
PA	HM567679	H1N1	05/30/2009	United Ki	A/England/349/2009
PA	GQ253488	H1N1	05/31/2009	China	A/Zhejiang/2/2009(H1N1)
PA	CY045188	H1N1	05/31/2009	USA	A/New York/3960/2009(H1N1)
PA	HM567711	H1N1	05/31/2009	United Ki	A/Scotland/8/2009
PA	HM567735	H1N1	05/31/2009	United Ki	A/England/366/2009
PA	HM567695	H1N1	05/31/2009	United Ki	A/England/351/2009
PA	CY044866	H1N1	05/31/2009	USA	A/New York/3726/2009(H1N1)
PA	GQ253497	H1N1	05/31/2009	China	A/Shanghai/71T/2009(H1N1)
PA	HM567847	H1N1	05/31/2009	Ireland	A/Ireland/5/2009
PA	CY065533	H1N1	05/31/2009	United Ki	A/England/359/2009
PA	CY045034	H1N1	05/31/2009	USA	A/New York/3812/2009(H1N1)
PA	HM567727	H1N1	05/31/2009	United Ki	A/England/365/2009
PA	HM567719	H1N1	05/31/2009	United Ki	A/England/364/2009
PA	CY071028	H1N1	05/31/2009	USA	A/New York/NHRC0001/2009(H1N1)
PA	GQ359763	H1N1	06/2009	China	A/Zhejiang/DTID-ZJU01/2009(H1N1)
PA	CY045225	H1N1	06/2009	Taiwan	A/Taiwan/115/2009(H1N1)
PA	CY045233	H1N1	06/2009	Taiwan	A/Taiwan/126/2009(H1N1)
PA	CY045241	H1N1	06/2009	Taiwan	A/Taiwan/137/2009(H1N1)
PA	HQ104925	H1N1	06/2009	Germany	A/Hamburg/NY1580/2009
PA	CY069625	H1N1	06/2009	Singapore	A/Singapore/471/2009(H1N1)
PA	CY069618	H1N1	06/2009	Singapore	A/Singapore/276/2009(H1N1)
PA	CY069640	H1N1	06/2009	Singapore	A/Singapore/527/2009(H1N1)
PA	CY069633	H1N1	06/2009	Singapore	A/Singapore/478/2009(H1N1)
PA	HQ695919	H1N1	06/2009	Taiwan	A/Taiwan/14/2009
PA	CY047786	H1N1	06/2009	Argentina	A/Argentina/7785/2009
PA	FN423712	H1N1	06/01/2009	Luxembour	A/Luxembourg/43/2009

PA	HM567815	H1N1	06/01/2009	Ireland	A/Ireland/7/2009
PA	CY044994	H1N1	06/01/2009	USA	A/New York/3751/2009(H1N1)
PA	CY045180	H1N1	06/01/2009	USA	A/New York/3959/2009(H1N1)
PA	CY050956	H1N1	06/01/2009	USA	A/Wisconsin/629-D00724/2009(H1N1)
PA	CY050964	H1N1	06/01/2009	USA	A/Wisconsin/629-D00941/2009(H1N1)
PA	CY050948	H1N1	06/01/2009	USA	A/Wisconsin/629-D00374/2009(H1N1)
PA	CY050972	H1N1	06/01/2009	USA	A/Wisconsin/629-D01725/2009(H1N1)
PA	CY053290	H1N1	06/01/2009	USA	A/Brownsville/400S/2009(H1N1)
PA	CY047379	H1N1	06/01/2009	USA	A/New York/3753/2009(H1N1)
PA	CY047371	H1N1	06/01/2009	USA	A/New York/3795/2009(H1N1)
PA	GQ338383	H1N1	06/01/2009	USA	A/Rhode Island/04/2009(H1N1)
PA	HM567767	H1N1	06/01/2009	United Ki	A/England/383/2009
PA	CY069884	H1N1	06/01/2009	United Ki	A/England/383/2009
PA	CY100811	H1N1	06/01/2009	USA	A/Texas/WRAIR1118P/2009(H1N1)
PA	CY065581	H1N1	06/01/2009	United Ki	A/England/386/2009
PA	CY065573	H1N1	06/01/2009	United Ki	A/England/385/2009
PA	CY065565	H1N1	06/01/2009	United Ki	A/England/384/2009
PA	CY065549	H1N1	06/01/2009	United Ki	A/England/381/2009
PA	CY065541	H1N1	06/01/2009	United Ki	A/England/361/2009
PA	CY065645	H1N1	06/01/2009	United Ki	A/England/402/2009
PA	CY065621	H1N1	06/01/2009	United Ki	A/England/395/2009
PA	CY071044	H1N1	06/01/2009	USA	A/New York/NHRC0003/2009(H1N1)
PA	CY076762	H1N1	06/01/2009	Canada	A/Ontario/147265/2009(H1N1)
PA	KC800979	H1N1	06/01/2009	France	A/Lyon/969/2009
PA	CY054696	H1N1	06/01/2009	Nicaragua	A/Managua/4467.05/2009(H1N1)
PA	CY056392	H1N1	06/01/2009	Nicaragua	A/Managua/3119.01/2009(H1N1)
PA	CY065557	H1N1	06/01/2009	United Ki	A/England/382/2009
PA	CY089893	H1N1	06/01/2009	USA	A/Wisconsin/629-D00881/2009(H1N1)
PA	HM567703	H1N1	06/01/2009	United Ki	A/England/360/2009
PA	HM567743	H1N1	06/01/2009	United Ki	A/England/374/2009
PA	FN423712	H1N1	06/01/2009	Luxembour	A/Luxembourg/43/2009
PA	GQ324575	H1N1	06/02/2009	Japan	A/Tokushima/1/2009(H1N1)
PA	GQ334351	H1N1	06/02/2009	Japan	A/Shizuoka/759/2009(H1N1)
PA	GQ329097	H1N1	06/02/2009	France	A/Paris/2709/2009(H1N1)
PA	CY045026	H1N1	06/02/2009	USA	A/New York/3803/2009(H1N1)
PA	CY045002	H1N1	06/02/2009	USA	A/New York/3828/2009(H1N1)
PA	CY045018	H1N1	06/02/2009	USA	A/New York/3796/2009(H1N1)
PA	CY045172	H1N1	06/02/2009	USA	A/New York/3819/2009(H1N1)
PA	CY045164	H1N1	06/02/2009	USA	A/New York/3818/2009(H1N1)
PA	CY050988	H1N1	06/02/2009	USA	A/Wisconsin/629-D00592/2009(H1N1)
PA	CY051268	H1N1	06/02/2009	USA	A/Wisconsin/629-S0143/2009(H1N1)
PA	CY053306	H1N1	06/02/2009	USA	A/Brownsville/43H/2009(H1N1)
PA	CY053298	H1N1	06/02/2009	USA	A/Brownsville/430S/2009(H1N1)
PA	CY051284	H1N1	06/02/2009	USA	A/Wisconsin/629-S0148/2009(H1N1)
PA	CY051228	H1N1	06/02/2009	USA	A/Wisconsin/629-D00022/2009(H1N1)
PA	CY051276	H1N1	06/02/2009	USA	A/Wisconsin/629-S0146/2009(H1N1)
PA	CY050980	H1N1	06/02/2009	USA	A/Wisconsin/629-D00367/2009(H1N1)
PA	GU138937	H1N1	06/02/2009	Japan	A/Chiba-C/48/2009(H1N1)
PA	CY049075	H1N1	06/02/2009	Singapore	A/Singapore/ON141/2009(H1N1)
PA	GQ329074	H1N1	06/02/2009	France	A/Caen/2716/2009(H1N1)
PA	HM567783	H1N1	06/02/2009	United Ki	A/England/393/2009
PA	CY069900	H1N1	06/02/2009	United Ki	A/England/393/2009
PA	HM567863	H1N1	06/02/2009	United Ki	A/England/416/2009
PA	CY065613	H1N1	06/02/2009	United Ki	A/England/394/2009

PA	CY076770	H1N1	06/02/2009	Canada	A/Ontario/147723/2009(H1N1)
PA	CY065589	H1N1	06/02/2009	United Ki	A/England/388/2009
PA	HM567751	H1N1	06/02/2009	United Ki	A/England/377/2009
PA	HM567759	H1N1	06/02/2009	United Ki	A/England/380/2009
PA	HM567775	H1N1	06/02/2009	United Ki	A/England/391/2009
PA	CY069892	H1N1	06/02/2009	United Ki	A/England/391/2009
PA	GQ411902	H1N1	06/03/2009	Canada	A/Toronto/T5308/2009(H1N1)
PA	HM567799	H1N1	06/03/2009	United Ki	A/England/401/2009
PA	GU138965	H1N1	06/03/2009	Japan	A/Yamaguchi/21/2009(H1N1)
PA	CY044101	H1N1	06/03/2009	USA	A/New York/3895/2009(H1N1)
PA	GQ330650	H1N1	06/03/2009	Russia	A/Moscow/IIV03/2009(H1N1)
PA	CY065597	H1N1	06/03/2009	United Ki	A/England/389/2009
PA	CY065629	H1N1	06/03/2009	United Ki	A/England/398/2009
PA	CY071052	H1N1	06/03/2009	USA	A/New York/NHRC0004/2009(H1N1)
PA	CY054688	H1N1	06/03/2009	USA	A/Wisconsin/629-D00589/2009(H1N1)
PA	CY055420	H1N1	06/03/2009	USA	A/Wisconsin/629-D00809/2009
PA	CY107571	H1N1	06/03/2009	United Ki	A/Scotland/Glasgow_412311/2009
PA	CY107606	H1N1	06/03/2009	United Ki	A/Scotland/Glasgow_413608/2009
PA	CY107750	H1N1	06/03/2009	United Ki	A/Scotland/Paisley_413960/2009
PA	CY107620	H1N1	06/03/2009	United Ki	A/Scotland/Glasgow_413848/2009
PA	CY107584	H1N1	06/03/2009	United Ki	A/Scotland/Paisley_412666/2009
PA	CY107564	H1N1	06/03/2009	United Ki	A/Scotland/Glasgow_412286/2009
PA	CY107577	H1N1	06/03/2009	United Ki	A/Scotland/Glasgow_412313/2009
PA	CY069931	H1N1	06/03/2009	United Ki	A/England/425/2009
PA	KC781440	H1N1	06/03/2009	USA	A/Utah/27/2009
PA	KC781932	H1N1	06/03/2009	USA	A/Virginia/24/2009
PA	CY065605	H1N1	06/03/2009	United Ki	A/England/392/2009
PA	CY107546	H1N1	06/03/2009	United Ki	A/Scotland/Livingston_412757/2009
PA	CY107591	H1N1	06/03/2009	United Ki	A/Scotland/EastKilbride_412770/20
PA	CY107744	H1N1	06/03/2009	United Ki	A/Scotland/Paisley_412182/2009
PA	CY107598	H1N1	06/03/2009	United Ki	A/Scotland/Glasgow_413364/2009
PA	HM567791	H1N1	06/03/2009	United Ki	A/England/399/2009
PA	CY051236	H1N1	06/03/2009	USA	A/Wisconsin/629-D01664/2009(H1N1)
PA	CY051292	H1N1	06/04/2009	USA	A/Wisconsin/629-S0173/2009(H1N1)
PA	HM567879	H1N1	06/04/2009	United Ki	A/England/424/2009
PA	CY051020	H1N1	06/04/2009	USA	A/Wisconsin/629-D00453/2009(H1N1)
PA	CY051044	H1N1	06/04/2009	USA	A/Wisconsin/629-D00725/2009(H1N1)
PA	CY051028	H1N1	06/04/2009	USA	A/Wisconsin/629-D00017/2009(H1N1)
PA	CY051052	H1N1	06/04/2009	USA	A/Wisconsin/629-D00008/2009(H1N1)
PA	CY043341	H1N1	06/04/2009	Denmark	A/Denmark/524/2009(H1N1)
PA	CY043333	H1N1	06/04/2009	Denmark	A/Denmark/523/2009(H1N1)
PA	CY065693	H1N1	06/04/2009	United Ki	A/England/428/2009
PA	CY065685	H1N1	06/04/2009	United Ki	A/England/423/2009
PA	CY065653	H1N1	06/04/2009	United Ki	A/England/411/2009
PA	CY076778	H1N1	06/04/2009	Canada	A/Ontario/152439/2009(H1N1)
PA	CY076786	H1N1	06/04/2009	Canada	A/Ontario/152846/2009(H1N1)
PA	CY083263	H1N1	06/04/2009	USA	A/San Diego/WRAIR1643P/2009(H1N1)
PA	CY069915	H1N1	06/04/2009	United Ki	A/England/413/2009
PA	CY069908	H1N1	06/04/2009	United Ki	A/England/412/2009
PA	CY065661	H1N1	06/04/2009	United Ki	A/England/414/2009
PA	CY089901	H1N1	06/04/2009	USA	A/Wisconsin/629-D01420/2009(H1N1)
PA	HM567855	H1N1	06/04/2009	United Ki	A/England/415/2009
PA	CY051036	H1N1	06/04/2009	USA	A/Wisconsin/629-D00658/2009(H1N1)
PA	CY058169	H1N1	06/05/2009	Nicaragua	A/Managua/4016.02/2009(H1N1)

PA	HM567543	H1N1	06/05/2009	United Ki	A/England/456/2009
PA	CY050996	H1N1	06/05/2009	USA	A/Wisconsin/629-D01903/2009(H1N1)
PA	CY044109	H1N1	06/05/2009	USA	A/New York/3898/2009(H1N1)
PA	HM567895	H1N1	06/05/2009	United Ki	A/England/432/2009
PA	HM567911	H1N1	06/05/2009	United Ki	A/England/436/2009
PA	HM567919	H1N1	06/05/2009	United Ki	A/England/443/2009
PA	CY065669	H1N1	06/05/2009	United Ki	A/England/418/2009
PA	CY065717	H1N1	06/05/2009	United Ki	A/England/433/2009
PA	CY065701	H1N1	06/05/2009	United Ki	A/England/430/2009
PA	CY065677	H1N1	06/05/2009	United Ki	A/England/419/2009
PA	CY065709	H1N1	06/05/2009	United Ki	A/England/431/2009
PA	CY065637	H1N1	06/05/2009	United Ki	A/England/400/2009
PA	CY072971	H1N1	06/05/2009	Nicaragua	A/Managua/2750.01/2009(H1N1)
PA	CY058529	H1N1	06/05/2009	Nicaragua	A/Managua/4905.02/2009(H1N1)
PA	CY072915	H1N1	06/05/2009	Nicaragua	A/Managua/2652.01/2009(H1N1)
PA	CY053895	H1N1	06/05/2009	Argentina	A/Argentina/HNRG13/2009(H1N1)
PA	CY069923	H1N1	06/05/2009	United Ki	A/England/417/2009
PA	HM567823	H1N1	06/05/2009	Ireland	A/Ireland/10/2009
PA	CY052143	H1N1	06/05/2009	USA	A/New York/3980/2009(H1N1)
PA	GQ324569	H1N1	06/06/2009	Japan	A/Shiga/3/2009(H1N1)
PA	GQ411906	H1N1	06/06/2009	China	A/Shanghai/143T/2009(H1N1)
PA	CY065725	H1N1	06/06/2009	United Ki	A/England/434/2009
PA	CY083255	H1N1	06/06/2009	USA	A/San Diego/WRAIR1642P/2009(H1N1)
PA	HM567903	H1N1	06/06/2009	United Ki	A/England/435/2009
PA	CY083598	H1N1	06/06/2009	Nicaragua	A/Managua/WRAIR8964F/2009(H1N1)
PA	CY058089	H1N1	06/06/2009	Nicaragua	A/Managua/1455.01/2009(H1N1)
PA	HM567871	H1N1	06/06/2009	United Ki	A/England/421/2009
PA	JX625981	H1N1	06/06/2009	United Ki	A/England/421/2009
PA	CY083470	H1N1	06/06/2009	Nicaragua	A/Managua/WRAIR1689P/2009(H1N1)
PA	GQ334335	H1N1	06/07/2009	Japan	A/Fukuoka-C/2/2009(H1N1)
PA	GQ334343	H1N1	06/07/2009	Japan	A/Fukuoka-C/3/2009(H1N1)
PA	CY058081	H1N1	06/07/2009	Nicaragua	A/Managua/171.01/2009(H1N1)
PA	CY053124	H1N1	06/07/2009	USA	A/Houston/170S/2009(H1N1)
PA	HM567943	H1N1	06/07/2009	United Ki	A/England/451/2009
PA	CY074600	H1N1	06/07/2009	USA	A/California/VRDL358/2009(H1N1)
PA	CY069939	H1N1	06/07/2009	United Ki	A/England/438/2009
PA	CY089917	H1N1	06/07/2009	USA	A/Wisconsin/629-D00112/2009(H1N1)
PA	HM567935	H1N1	06/07/2009	United Ki	A/England/450/2009
PA	GQ300858	H1N1	06/07/2009	Japan	A/Fukuoka-C/1/2009(H1N1)
PA	CY045212	H1N1	06/08/2009	USA	A/New York/4057/2009(H1N1)
PA	HM567887	H1N1	06/08/2009	United Ki	A/England/429/2009
PA	CY051300	H1N1	06/08/2009	USA	A/Wisconsin/629-S0186/2009(H1N1)
PA	CY051076	H1N1	06/08/2009	USA	A/Wisconsin/629-D00579/2009(H1N1)
PA	CY051324	H1N1	06/08/2009	USA	A/Wisconsin/629-S0202/2009(H1N1)
PA	CY051508	H1N1	06/08/2009	USA	A/Wisconsin/629-S0206/2009(H1N1)
PA	CY052135	H1N1	06/08/2009	USA	A/New York/3976/2009(H1N1)
PA	CY051316	H1N1	06/08/2009	USA	A/Wisconsin/629-S0201/2009(H1N1)
PA	CY051500	H1N1	06/08/2009	USA	A/Wisconsin/629-S0189/2009(H1N1)
PA	CY051332	H1N1	06/08/2009	USA	A/Wisconsin/629-S0203/2009(H1N1)
PA	CY051068	H1N1	06/08/2009	USA	A/Wisconsin/629-D00101/2009(H1N1)
PA	CY051308	H1N1	06/08/2009	USA	A/Wisconsin/629-S0197/2009(H1N1)
PA	CY051060	H1N1	06/08/2009	USA	A/Wisconsin/629-D02068/2009(H1N1)
PA	CY076794	H1N1	06/08/2009	Canada	A/Ontario/156785/2009(H1N1)
PA	CY083151	H1N1	06/08/2009	Dominican	A/Santo Domingo/WRAIR1044N/2009

PA	CY071545	H1N1	06/08/2009	USA	A/California/WR1323P/2009(H1N1)
PA	KC781794	H1N1	06/08/2009	USA	A/Wisconsin/26/2009
PA	CY089989	H1N1	06/08/2009	USA	A/Wisconsin/629-D01822/2009(H1N1)
PA	CY058137	H1N1	06/08/2009	Nicaragua	A/Managua/516.01/2009(H1N1)
PA	CY045058	H1N1	06/09/2009	USA	A/New York/4052/2009(H1N1)
PA	HM567927	H1N1	06/09/2009	United Ki	A/England/446/2009
PA	CY045969	H1N1	06/09/2009	Canada	A/Toronto/T5362/2009(H1N1)
PA	CY051516	H1N1	06/09/2009	USA	A/Wisconsin/629-S0208/2009(H1N1)
PA	CY051244	H1N1	06/09/2009	USA	A/Wisconsin/629-D01199/2009(H1N1)
PA	CY052295	H1N1	06/09/2009	USA	A/Texas/42091791/2009(H1N1)
PA	CY052127	H1N1	06/09/2009	USA	A/New York/3969/2009(H1N1)
PA	CY051356	H1N1	06/09/2009	USA	A/Wisconsin/629-S0226/2009(H1N1)
PA	CY051340	H1N1	06/09/2009	USA	A/Wisconsin/629-S0220/2009(H1N1)
PA	CY051348	H1N1	06/09/2009	USA	A/Wisconsin/629-S0222/2009(H1N1)
PA	CY049882	H1N1	06/09/2009	El Salvac	A/San Salvador/WR0167N/2009(H1N1)
PA	GQ324581	H1N1	06/09/2009	Japan	A/Yokohama/1/2009(H1N1)
PA	GQ375886	H1N1	06/09/2009	Japan	A/Iwate/1/2009(H1N1)
PA	CY043349	H1N1	06/09/2009	Denmark	A/Denmark/528/2009(H1N1)
PA	CY065733	H1N1	06/09/2009	United Ki	A/England/445/2009
PA	CY058105	H1N1	06/09/2009	Nicaragua	A/Managua/168.02/2009(H1N1)
PA	CY069947	H1N1	06/09/2009	United Ki	A/England/444/2009
PA	CY095800	H1N1	06/09/2009	China	A/Shenzhen/45/2009
PA	CY095808	H1N1	06/09/2009	China	A/Shenzhen/48/2009
PA	CY095804	H1N1	06/09/2009	China	A/Shenzhen/47/2009
PA	CY095756	H1N1	06/09/2009	China	A/Shenzhen/25/2009
PA	CY095835	H1N1	06/09/2009	China	A/Shenzhen/67/2009
PA	CY095846	H1N1	06/09/2009	China	A/Shenzhen/71/2009
PA	CY095812	H1N1	06/09/2009	China	A/Shenzhen/49/2009
PA	CY095796	H1N1	06/09/2009	China	A/Shenzhen/44/2009
PA	CY095791	H1N1	06/09/2009	China	A/Shenzhen/42/2009
PA	CY095823	H1N1	06/09/2009	China	A/Shenzhen/54/2009
PA	CY095827	H1N1	06/09/2009	China	A/Shenzhen/61/2009
PA	CY095828	H1N1	06/09/2009	China	A/Shenzhen/62/2009
PA	CY095832	H1N1	06/09/2009	China	A/Shenzhen/63/2009
PA	CY095784	H1N1	06/09/2009	China	A/Shenzhen/40/2009
PA	CY095770	H1N1	06/09/2009	China	A/Shenzhen/37/2009
PA	CY045042	H1N1	06/09/2009	USA	A/New York/3967/2009(H1N1)
PA	CY051372	H1N1	06/10/2009	USA	A/Wisconsin/629-S0248/2009(H1N1)
PA	CY090005	H1N1	06/10/2009	USA	A/Wisconsin/629-S0235/2009(H1N1)
PA	CY051388	H1N1	06/10/2009	USA	A/Wisconsin/629-S0254/2009(H1N1)
PA	CY051404	H1N1	06/10/2009	USA	A/Wisconsin/629-S0252/2009(H1N1)
PA	CY044117	H1N1	06/10/2009	USA	A/New York/3992/2009(H1N1)
PA	CY045050	H1N1	06/10/2009	USA	A/New York/4006/2009(H1N1)
PA	CY045220	H1N1	06/10/2009	USA	A/New York/4074/2009(H1N1)
PA	CY045196	H1N1	06/10/2009	USA	A/New York/4014/2009(H1N1)
PA	CY045204	H1N1	06/10/2009	USA	A/New York/4048/2009(H1N1)
PA	CY069308	H1N1	06/10/2009	Colombia	A/Bogota/WRAIR0088N/2009(H1N1)
PA	CY073341	H1N1	06/10/2009	USA	A/San Diego/WR1628P/2009(H1N1)
PA	CY052271	H1N1	06/10/2009	USA	A/Texas/42102708/2009(H1N1)
PA	CY052303	H1N1	06/10/2009	USA	A/Texas/42103399/2009(H1N1)
PA	CY051364	H1N1	06/10/2009	USA	A/Wisconsin/629-S0245/2009(H1N1)
PA	CY051396	H1N1	06/10/2009	USA	A/Wisconsin/629-S0255/2009(H1N1)
PA	CY107471	H1N1	06/10/2009	United Ki	A/Scotland/Edinburgh_17343/2009
PA	CY107641	H1N1	06/10/2009	United Ki	A/Scotland/Glasgow_413941/2009

PA	CY107756	H1N1	06/10/2009	United Ki	A/Scotland/Paisley_415110/2009
PA	CY107552	H1N1	06/10/2009	United Ki	A/Scotland/Livingston_416138/2009
PA	CY107648	H1N1	06/10/2009	United Ki	A/Scotland/Glasgow_414159/2009
PA	CY051380	H1N1	06/10/2009	USA	A/Wisconsin/629-S0247/2009(H1N1)
PA	CY051884	H1N1	06/11/2009	USA	A/Texas/42113095/2009(H1N1)
PA	GQ365451	H1N1	06/11/2009	Japan	A/Sapporo/1/2009(H1N1)
PA	GQ365415	H1N1	06/11/2009	Japan	A/Akita/1/2009(H1N1)
PA	CY074060	H1N1	06/11/2009	Argentina	A/Argentina/HNRG8/2009
PA	CY057211	H1N1	06/11/2009	Nicaragua	A/Managua/167.01/2009(H1N1)
PA	CY051412	H1N1	06/11/2009	USA	A/Wisconsin/629-S0269/2009(H1N1)
PA	CY063491	H1N1	06/11/2009	USA	A/Boston/110/2009(H1N1)
PA	CY051892	H1N1	06/11/2009	USA	A/Texas/42114261/2009(H1N1)
PA	CY051084	H1N1	06/11/2009	USA	A/Wisconsin/629-D01326/2009(H1N1)
PA	CY089997	H1N1	06/11/2009	USA	A/Wisconsin/629-D01183/2009(H1N1)
PA	CY065741	H1N1	06/11/2009	United Ki	A/Scotland/10/2009
PA	CY053116	H1N1	06/11/2009	USA	A/Houston/160S/2009(H1N1)
PA	CY051860	H1N1	06/12/2009	USA	A/Texas/42122969/2009(H1N1)
PA	CY058097	H1N1	06/12/2009	Nicaragua	A/Managua/655.02/2009(H1N1)
PA	CY049890	H1N1	06/12/2009	El Salvad	A/San Salvador/0169T/2009(H1N1)
PA	CY045082	H1N1	06/12/2009	USA	A/New York/4072/2009(H1N1)
PA	HM569688	H1N1	06/12/2009	Argentina	A/Argentina/19618/2009(H1N1)
PA	CY051116	H1N1	06/12/2009	USA	A/Wisconsin/629-D02263/2009(H1N1)
PA	CY052199	H1N1	06/12/2009	USA	A/Texas/42121926/2009(H1N1)
PA	CY050163	H1N1	06/12/2009	USA	A/New York/4095/2009(H1N1)
PA	CY051844	H1N1	06/12/2009	USA	A/Texas/42123701/2009(H1N1)
PA	KT180763	H1N1	06/12/2009	Singapore	A/Singapore/DMS1/2009
PA	CY073325	H1N1	06/12/2009	USA	A/San Diego/WR1626P/2009(H1N1)
PA	CY073317	H1N1	06/12/2009	USA	A/San Diego/WR1625P/2009(H1N1)
PA	CY058217	H1N1	06/12/2009	Nicaragua	A/Managua/578.01/2009(H1N1)
PA	GU138947	H1N1	06/12/2009	Japan	A/Kanagawa/137/2009(H1N1)
PA	CY051108	H1N1	06/13/2009	USA	A/Wisconsin/629-D01618/2009(H1N1)
PA	CY064505	H1N1	06/13/2009	USA	A/Boston/115/2009(H1N1)
PA	CY047387	H1N1	06/13/2009	USA	A/New York/4100/2009(H1N1)
PA	GQ365441	H1N1	06/13/2009	Japan	A/Kagoshima/1/2009(H1N1)
PA	GQ463199	H1N1	06/13/2009	China	A/Hunan/SWL3/2009(H1N1)
PA	CY045066	H1N1	06/13/2009	USA	A/New York/4099/2009(H1N1)
PA	GQ866950	H1N1	06/13/2009	Thailand	A/Thailand/CU-B5/2009(H1N1)
PA	CY064465	H1N1	06/13/2009	USA	A/Boston/96/2009(H1N1)
PA	CY073309	H1N1	06/13/2009	USA	A/San Diego/WR1624P/2009(H1N1)
PA	CY073333	H1N1	06/13/2009	USA	A/San Diego/WR1627P/2009(H1N1)
PA	CY054752	H1N1	06/13/2009	USA	A/California/VRDL9/2009(H1N1)
PA	CY054728	H1N1	06/13/2009	USA	A/California/VRDL6/2009(H1N1)
PA	CY057219	H1N1	06/13/2009	Nicaragua	A/Managua/109.01/2009(H1N1)
PA	CY058225	H1N1	06/13/2009	Nicaragua	A/Managua/5221.03/2009(H1N1)
PA	CY058153	H1N1	06/13/2009	Nicaragua	A/Managua/4085.02/2009(H1N1)
PA	CY058177	H1N1	06/13/2009	Nicaragua	A/Managua/792.01/2009(H1N1)
PA	CY058233	H1N1	06/13/2009	Nicaragua	A/Managua/5363.02/2009(H1N1)
PA	CY058121	H1N1	06/13/2009	Nicaragua	A/Managua/1637.01/2009(H1N1)
PA	CY051004	H1N1	06/13/2009	USA	A/Wisconsin/629-D00665/2009(H1N1)
PA	CY052319	H1N1	06/13/2009	USA	A/Texas/42132413/2009(H1N1)
PA	CY051100	H1N1	06/13/2009	USA	A/Wisconsin/629-D00196/2009(H1N1)
PA	KC782098	H1N1	06/13/2009	USA	A/Rhode Island/15/2009
PA	JQ409141	H1N1	06/13/2009	Finland	A/Helsinki/Vi3/2009(H1N1)
PA	JQ409133	H1N1	06/13/2009	Finland	A/Helsinki/Vi2/2009

PA	CY075176	H1N1	06/13/2009	Chile	A/Chile/1586/2009(H1N1)
PA	CY083502	H1N1	06/13/2009	Nicaragua	A/Managua/WRAIR1693P/2009(H1N1)
PA	CY083606	H1N1	06/13/2009	Nicaragua	A/Managua/WRAIR8997F/2009(H1N1)
PA	CY058113	H1N1	06/13/2009	Nicaragua	A/Managua/2152.01/2009(H1N1)
PA	CY063499	H1N1	06/13/2009	USA	A/Boston/112/2009(H1N1)
PA	GU138940	H1N1	06/13/2009	Japan	A/Hiroshima/200/2009(H1N1)
PA	CY123648	H1N1	06/14/2009	Singapore	A/Singapore/ON193/2009
PA	KJ130163	H1N1	06/14/2009	New Zeala	A/New Zealand/1212b/2009
PA	GQ457466	H1N1	06/14/2009	USA	A/Utah/11/2009(H1N1)
PA	GQ457461	H1N1	06/14/2009	USA	A/Utah/05/2009(H1N1)
PA	GQ351289	H1N1	06/14/2009	Italy	A/Italy/85/2009(H1N1)
PA	CY054712	H1N1	06/14/2009	USA	A/California/VRDL4/2009(H1N1)
PA	CY054744	H1N1	06/14/2009	USA	A/California/VRDL8/2009(H1N1)
PA	CY051132	H1N1	06/14/2009	USA	A/Wisconsin/629-D00119/2009(H1N1)
PA	CY052396	H1N1	06/14/2009	USA	A/Texas/42142537/2009(H1N1)
PA	CY051156	H1N1	06/14/2009	USA	A/Wisconsin/629-D01147/2009(H1N1)
PA	CY051124	H1N1	06/14/2009	USA	A/Wisconsin/629-D00916/2009(H1N1)
PA	KJ130203	H1N1	06/14/2009	New Zeala	A/New Zealand/1212e/2009
PA	KJ130171	H1N1	06/14/2009	New Zeala	A/New Zealand/1212a/2009(H1N1)
PA	KJ130179	H1N1	06/14/2009	New Zeala	A/New Zealand/1212d/2009
PA	KJ130187	H1N1	06/14/2009	New Zeala	A/New Zealand/1212f/2009
PA	KJ130147	H1N1	06/14/2009	New Zeala	A/New Zealand/1212c/2009
PA	CY049922	H1N1	06/14/2009	USA	A/New Bern/WR0670/2009(H1N1)
PA	CY051428	H1N1	06/15/2009	USA	A/Wisconsin/629-S0279/2009(H1N1)
PA	CY051140	H1N1	06/15/2009	USA	A/Wisconsin/629-D01152/2009(H1N1)
PA	CY051012	H1N1	06/15/2009	USA	A/Wisconsin/629-D01619/2009(H1N1)
PA	CY051436	H1N1	06/15/2009	USA	A/Wisconsin/629-S0293/2009(H1N1)
PA	GU138935	H1N1	06/15/2009	Japan	A/Aichi/198/2009(H1N1)
PA	GU138961	H1N1	06/15/2009	Japan	A/Shizuoka/793/2009(H1N1)
PA	GU138956	H1N1	06/15/2009	Japan	A/Niigata/690/2009(H1N1)
PA	CY049131	H1N1	06/15/2009	Singapore	A/Singapore/ON226/2009(H1N1)
PA	GQ433896	H1N1	06/15/2009	China	A/Jiangsu/1/2009(H1N1)
PA	HM569680	H1N1	06/15/2009	Argentina	A/Argentina/19527/2009(H1N1)
PA	CY054720	H1N1	06/15/2009	USA	A/California/VRDL5/2009(H1N1)
PA	CY055460	H1N1	06/15/2009	USA	A/California/VRDL12/2009(H1N1)
PA	CY058129	H1N1	06/15/2009	Nicaragua	A/Managua/5453.01/2009(H1N1)
PA	CY051420	H1N1	06/15/2009	USA	A/Wisconsin/629-S0280/2009(H1N1)
PA	CY052279	H1N1	06/15/2009	USA	A/Texas/42151049/2009(H1N1)
PA	CY052111	H1N1	06/15/2009	USA	A/Wisconsin/629-D00739/2009(H1N1)
PA	CY051092	H1N1	06/15/2009	USA	A/Wisconsin/629-D01844/2009(H1N1)
PA	CY051820	H1N1	06/15/2009	USA	A/Texas/42152486/2009(H1N1)
PA	KC781548	H1N1	06/15/2009	USA	A/Massachusetts/16/2009
PA	KC781703	H1N1	06/15/2009	USA	A/Oregon/10/2009
PA	CY075216	H1N1	06/15/2009	Chile	A/Chile/1624/2009(H1N1)
PA	CY075200	H1N1	06/15/2009	Chile	A/Chile/1600/2009(H1N1)
PA	CY075192	H1N1	06/15/2009	Chile	A/Chile/1599/2009(H1N1)
PA	CY075184	H1N1	06/15/2009	Chile	A/Chile/1598/2009(H1N1)
PA	CY075208	H1N1	06/15/2009	Chile	A/Chile/1603/2009(H1N1)
PA	CY083335	H1N1	06/15/2009	USA	A/San Diego/WRAIR1654P/2009
PA	CY089722	H1N1	06/15/2009	USA	A/Boston/107/2009
PA	CY089933	H1N1	06/15/2009	USA	A/Wisconsin/629-D00889/2009(H1N1)
PA	CY089925	H1N1	06/15/2009	USA	A/Wisconsin/629-D01717/2009(H1N1)
PA	CY058553	H1N1	06/15/2009	Nicaragua	A/Managua/4935.03/2009(H1N1)
PA	CY060749	H1N1	06/15/2009	Canada	A/Ontario/9739/2009(H1N1)

PA	CY060741	H1N1	06/15/2009	Canada	A/Ontario/9698/2009(H1N1)
PA	CY063507	H1N1	06/15/2009	USA	A/Boston/113/2009(H1N1)
PA	CY064497	H1N1	06/15/2009	USA	A/Boston/109/2009(H1N1)
PA	CY064473	H1N1	06/15/2009	USA	A/Boston/103/2009(H1N1)
PA	CY064481	H1N1	06/15/2009	USA	A/Boston/106/2009(H1N1)
PA	GQ365460	H1N1	06/16/2009	Japan	A/Utsunomiya/2/2009(H1N1)
PA	CY049835	H1N1	06/16/2009	Colombia	A/Bogota/WR0090N/2009(H1N1)
PA	CY052215	H1N1	06/16/2009	USA	A/Texas/42163291/2009(H1N1)
PA	GQ902800	H1N1	06/16/2009	Thailand	A/Thailand/CU-B42/2009(H1N1)
PA	CY089941	H1N1	06/16/2009	USA	A/Wisconsin/629-D00744/2009(H1N1)
PA	CY052388	H1N1	06/16/2009	USA	A/Texas/42163295/2009(H1N1)
PA	CY074042	H1N1	06/16/2009	Argentina	A/Argentina/HNRG45/2009(H1N1)
PA	GU138936	H1N1	06/16/2009	Japan	A/Aichi/202/2009(H1N1)
PA	CY051164	H1N1	06/16/2009	USA	A/Wisconsin/629-D00541/2009(H1N1)
PA	CY058161	H1N1	06/16/2009	Nicaragua	A/Managua/2330.02/2009(H1N1)
PA	GQ334360	H1N1	06/16/2009	Japan	A/Utsunomiya/1/2009(H1N1)
PA	CY044170	H1N1	06/16/2009	USA	A/Bethesda/SP506/2009(H1N1)
PA	CY051148	H1N1	06/16/2009	USA	A/Wisconsin/629-D01734/2009(H1N1)
PA	CY051468	H1N1	06/16/2009	USA	A/Wisconsin/629-S0314/2009(H1N1)
PA	CY064489	H1N1	06/16/2009	USA	A/Boston/108/2009(H1N1)
PA	CY107674	H1N1	06/17/2009	United Ki	A/Scotland/Glasgow_419977/2009
PA	CY047778	H1N1	06/17/2009	Argentina	A/Argentina/7649/2009
PA	CY047810	H1N1	06/17/2009	Argentina	A/Argentina/7967/2009
PA	CY047818	H1N1	06/17/2009	Argentina	A/Argentina/7980/2009
PA	CY107771	H1N1	06/17/2009	United Ki	A/Scotland/EastKilbride_419809/20
PA	CY107192	H1N1	06/17/2009	United Ki	A/Scotland/Paisley_417557/2009
PA	CY074071	H1N1	06/17/2009	Argentina	A/Argentina/HNRG83/2009
PA	CY089730	H1N1	06/17/2009	USA	A/Boston/139/2009
PA	CY064577	H1N1	06/17/2009	USA	A/Boston/125/2009(H1N1)
PA	CY107667	H1N1	06/17/2009	United Ki	A/Scotland/Glasgow_419865/2009
PA	CY107660	H1N1	06/17/2009	United Ki	A/Scotland/Glasgow_419707/2009
PA	CY049970	H1N1	06/17/2009	Dominican	A/Santo Domingo/1066N/2009(H1N1)
PA	CY052103	H1N1	06/17/2009	USA	A/New York/4238/2009(H1N1)
PA	CY064593	H1N1	06/17/2009	USA	A/Boston/127/2009(H1N1)
PA	CY051548	H1N1	06/17/2009	USA	A/New York/4403/2009(H1N1)
PA	CY051876	H1N1	06/17/2009	USA	A/Texas/42173957/2009(H1N1)
PA	CY055452	H1N1	06/17/2009	USA	A/California/VRDL11/2009(H1N1)
PA	CY054736	H1N1	06/17/2009	USA	A/California/VRDL7/2009(H1N1)
PA	CY054848	H1N1	06/17/2009	USA	A/California/VRDL31/2009(H1N1)
PA	GQ392028	H1N1	06/17/2009	Italy	A/Italy/127/2009(H1N1)
PA	GQ455031	H1N1	06/17/2009	China	A/Nanjing/2/2009(H1N1)
PA	CY044125	H1N1	06/17/2009	USA	A/New York/4197/2009(H1N1)
PA	GU138941	H1N1	06/17/2009	Japan	A/Hiroshima/201/2009(H1N1)
PA	CY045977	H1N1	06/17/2009	Canada	A/Toronto/T9842/2009(H1N1)
PA	GQ866958	H1N1	06/17/2009	Thailand	A/Thailand/CU-H9/2009(H1N1)
PA	CY051444	H1N1	06/17/2009	USA	A/Wisconsin/629-S0319/2009(H1N1)
PA	KC781848	H1N1	06/18/2009	USA	A/Oregon/13/2009
PA	KC781807	H1N1	06/18/2009	USA	A/Oregon/11/2009
PA	CY047826	H1N1	06/18/2009	Argentina	A/Argentina/8019/2009
PA	CY047794	H1N1	06/18/2009	Argentina	A/Argentina/7937/2009
PA	CY089957	H1N1	06/18/2009	USA	A/Wisconsin/629-D01039/2009(H1N1)
PA	CY089949	H1N1	06/18/2009	USA	A/Wisconsin/629-D01875/2009(H1N1)
PA	CY089909	H1N1	06/18/2009	USA	A/Wisconsin/629-D01115/2009(H1N1)
PA	CY073293	H1N1	06/18/2009	USA	A/Silver Spring/WRAIRSP510P20/200

PA	CY073277	H1N1	06/18/2009	USA	A/Silver Spring/WRAIRSP510P18/200
PA	CY073269	H1N1	06/18/2009	USA	A/Silver Spring/WRAIRSP510P12/200
PA	CY075224	H1N1	06/18/2009	Chile	A/Chile/2009/2009(H1N1)
PA	CY064649	H1N1	06/18/2009	USA	A/Boston/135/2009(H1N1)
PA	CY064665	H1N1	06/18/2009	USA	A/Boston/138/2009(H1N1)
PA	CY064561	H1N1	06/18/2009	USA	A/Boston/123/2009(H1N1)
PA	CY064633	H1N1	06/18/2009	USA	A/Boston/133/2009(H1N1)
PA	HM569696	H1N1	06/18/2009	Argentina	A/Argentina/19656/2009(H1N1)
PA	CY073301	H1N1	06/18/2009	USA	A/Silver Spring/WRAIRSP510P3/2009
PA	CY073285	H1N1	06/18/2009	USA	A/Silver Spring/WRAIRSP510P2/2009
PA	CY073261	H1N1	06/18/2009	USA	A/Silver Spring/WRAIRSP510P10/200
PA	CY053927	H1N1	06/18/2009	Argentina	A/Argentina/HNRG3/2009(H1N1)
PA	GQ902832	H1N1	06/18/2009	Thailand	A/Thailand/CU-H17/2009(H1N1)
PA	CY054808	H1N1	06/18/2009	USA	A/California/VRDL26/2009(H1N1)
PA	GQ457563	H1N1	06/18/2009	Canada	A/Saskatchewan/5350/2009
PA	GQ457564	H1N1	06/18/2009	Canada	A/Saskatchewan/5351/2009
PA	CY051452	H1N1	06/18/2009	USA	A/Wisconsin/629-S0345/2009(H1N1)
PA	CY051172	H1N1	06/18/2009	USA	A/Wisconsin/629-D02063/2009(H1N1)
PA	CY051460	H1N1	06/18/2009	USA	A/Wisconsin/629-S0339/2009(H1N1)
PA	GU138938	H1N1	06/18/2009	Japan	A/Chiba-C/51/2009(H1N1)
PA	GU138948	H1N1	06/18/2009	Japan	A/Kanagawa/140/2009(H1N1)
PA	CY043117	H1N1	06/18/2009	USA	A/Bethesda/SP508/2009(H1N1)
PA	CY044178	H1N1	06/18/2009	USA	A/Silver Spring/SP509/2009(H1N1)
PA	GQ504751	H1N1	06/18/2009	China	A/Nanjing/1/2009(H1N1)
PA	GQ365433	H1N1	06/18/2009	Japan	A/Iwate/2/2009(H1N1)
PA	CY044186	H1N1	06/18/2009	USA	A/Silver Spring/SP510/2009(H1N1)
PA	CY047850	H1N1	06/19/2009	Argentina	A/Argentina/8673/2009
PA	CY045074	H1N1	06/19/2009	USA	A/New York/4290/2009(H1N1)
PA	CY089965	H1N1	06/19/2009	USA	A/Wisconsin/629-D02404/2009(H1N1)
PA	CY075232	H1N1	06/19/2009	Chile	A/Chile/2239/2009(H1N1)
PA	CY073974	H1N1	06/19/2009	Argentina	A/Argentina/HNRG104/2009
PA	CY083087	H1N1	06/19/2009	Colombia	A/Bogota/WRAIR0442T/2009
PA	CY064625	H1N1	06/19/2009	USA	A/Boston/132/2009(H1N1)
PA	CY064537	H1N1	06/19/2009	USA	A/Boston/120/2009(H1N1)
PA	CY064545	H1N1	06/19/2009	USA	A/Boston/121/2009(H1N1)
PA	GQ457562	H1N1	06/19/2009	Canada	A/Saskatchewan/5131/2009
PA	CY051564	H1N1	06/19/2009	USA	A/New York/4459/2009(H1N1)
PA	CY051852	H1N1	06/19/2009	USA	A/Texas/42191653/2009(H1N1)
PA	CY052420	H1N1	06/19/2009	USA	A/Texas/42192947/2009(H1N1)
PA	CY052239	H1N1	06/19/2009	USA	A/Texas/42191647/2009(H1N1)
PA	CY049986	H1N1	06/19/2009	Mexico	A/Mexico City/WR1087T/2009(H1N1)
PA	CY049083	H1N1	06/19/2009	Singapore	A/Singapore/ON279/2009(H1N1)
PA	CY049363	H1N1	06/19/2009	Singapore	A/Singapore/ON313/2009(H1N1)
PA	CY047802	H1N1	06/19/2009	Argentina	A/Argentina/7953/2009
PA	GQ494356	H1N1	06/20/2009	Russia	A/Moscow/IIV05/2009(H1N1)
PA	CY047874	H1N1	06/20/2009	Argentina	A/Argentina/9004/2009
PA	CY052327	H1N1	06/20/2009	USA	A/Texas/42202026/2009(H1N1)
PA	CY052247	H1N1	06/20/2009	USA	A/Texas/42201798/2009(H1N1)
PA	CY051868	H1N1	06/20/2009	USA	A/Texas/42201824/2009(H1N1)
PA	CY049059	H1N1	06/20/2009	Singapore	A/Singapore/ON305/2009(H1N1)
PA	CY072987	H1N1	06/20/2009	Nicaragua	A/Managua/5273.02/2009(H1N1)
PA	CY075240	H1N1	06/20/2009	Chile	A/Chile/2851/2009(H1N1)
PA	CY064529	H1N1	06/20/2009	USA	A/Boston/118/2009(H1N1)
PA	CY064657	H1N1	06/20/2009	USA	A/Boston/137/2009(H1N1)

PA	CY064585	H1N1	06/20/2009	USA	A/Boston/126/2009(H1N1)
PA	CY054768	H1N1	06/20/2009	USA	A/California/VRDL20/2009(H1N1)
PA	CY051196	H1N1	06/20/2009	USA	A/Wisconsin/629-D00496/2009(H1N1)
PA	KT180898	H1N1	06/20/2009	Singapore	A/Singapore/DMS385/2009
PA	CY051180	H1N1	06/20/2009	USA	A/Wisconsin/629-D00428/2009(H1N1)
PA	CY052335	H1N1	06/21/2009	USA	A/Texas/42211898/2009(H1N1)
PA	CY051188	H1N1	06/21/2009	USA	A/Wisconsin/629-D02220/2009(H1N1)
PA	CY089973	H1N1	06/21/2009	USA	A/Wisconsin/629-D02294/2009(H1N1)
PA	CY058537	H1N1	06/21/2009	Nicaragua	A/Managua/4514.03/2009(H1N1)
PA	CY064609	H1N1	06/21/2009	USA	A/Boston/130/2009(H1N1)
PA	CY047834	H1N1	06/21/2009	Argentina	A/Argentina/8551/2009
PA	CY051540	H1N1	06/21/2009	USA	A/New York/4402/2009(H1N1)
PA	CY047858	H1N1	06/21/2009	Argentina	A/Argentina/8989/2009
PA	CY047842	H1N1	06/21/2009	Argentina	A/Argentina/8574/2009
PA	CY074032	H1N1	06/21/2009	Argentina	A/Argentina/HNRG36/2009(H1N1)
PA	CY052263	H1N1	06/22/2009	USA	A/Texas/42221280/2009(H1N1)
PA	CY120498	H1N1	06/22/2009	Hong Kong	A/Hong Kong/H090-661-V10/2009
PA	CY051204	H1N1	06/22/2009	USA	A/Wisconsin/629-D02024/2009(H1N1)
PA	GU138967	H1N1	06/22/2009	Japan	A/Yamaguchi/23/2009(H1N1)
PA	GU138957	H1N1	06/22/2009	Japan	A/Niigata/700/2009(H1N1)
PA	CY123928	H1N1	06/22/2009	Singapore	A/Singapore/ON355/2009(H1N1)
PA	CY049371	H1N1	06/22/2009	Singapore	A/Singapore/ON355/2009(H1N1)
PA	CY049091	H1N1	06/22/2009	Singapore	A/Singapore/ON354/2009(H1N1)
PA	CY049355	H1N1	06/22/2009	Singapore	A/Singapore/ON368/2009(H1N1)
PA	CY075248	H1N1	06/22/2009	Chile	A/Chile/2362/2009(H1N1)
PA	CY064553	H1N1	06/22/2009	USA	A/Boston/122/2009(H1N1)
PA	CY064673	H1N1	06/22/2009	USA	A/Boston/140/2009(H1N1)
PA	CY064601	H1N1	06/22/2009	USA	A/Boston/129/2009(H1N1)
PA	CY074022	H1N1	06/22/2009	Argentina	A/Argentina/HNRG32/2009(H1N1)
PA	CY074050	H1N1	06/22/2009	Argentina	A/Argentina/HNRG5/2009(H1N1)
PA	CY054776	H1N1	06/22/2009	USA	A/California/VRDL21/2009(H1N1)
PA	CY054824	H1N1	06/22/2009	USA	A/California/VRDL28/2009(H1N1)
PA	GU271949	H1N1	06/22/2009	Thailand	A/Thailand/CU-B97/2009(H1N1)
PA	CY055476	H1N1	06/22/2009	USA	A/California/VRDL18/2009(H1N1)
PA	CY058145	H1N1	06/22/2009	Nicaragua	A/Managua/5665.01/2009(H1N1)
PA	KT180911	H1N1	06/22/2009	Singapore	A/Singapore/DMS516/2009
PA	KT180764	H1N1	06/22/2009	Singapore	A/Singapore/DMS4/2009
PA	KT180917	H1N1	06/22/2009	Singapore	A/Singapore/DMS562/2009
PA	CY123345	H1N1	06/22/2009	Singapore	A/Singapore/GP687/2009
PA	KC781887	H1N1	06/22/2009	USA	A/Utah/21/2009
PA	KC781709	H1N1	06/22/2009	USA	A/New York/98/2009
PA	CY047906	H1N1	06/22/2009	Argentina	A/Argentina/9452/2009
PA	CY047866	H1N1	06/22/2009	Argentina	A/Argentina/8994/2009
PA	CY111616	H1N1	06/22/2009	Hong Kong	A/Hong Kong/H090-661-V10/2009
PA	CY049850	H1N1	06/22/2009	Bolivia	A/La Paz/WR0096T/2009(H1N1)
PA	GQ365423	H1N1	06/23/2009	Japan	A/Fukushima/1/2009(H1N1)
PA	CY047890	H1N1	06/23/2009	Argentina	A/Argentina/9333/2009
PA	CY051556	H1N1	06/23/2009	USA	A/New York/4434/2009(H1N1)
PA	GU138958	H1N1	06/23/2009	Japan	A/Niigata/717/2009(H1N1)
PA	CY049291	H1N1	06/23/2009	Singapore	A/Singapore/ON380/2009(H1N1)
PA	CY049299	H1N1	06/23/2009	Singapore	A/Singapore/ON384/2009(H1N1)
PA	CY065157	H1N1	06/23/2009	United Ki	A/England/541/2009
PA	CY055468	H1N1	06/23/2009	USA	A/California/VRDL14/2009(H1N1)
PA	KT180914	H1N1	06/23/2009	Singapore	A/Singapore/DMS547/2009

PA	CY123329	H1N1	06/23/2009	Singapore	A/Singapore/GP669/2009
PA	CY123337	H1N1	06/23/2009	Singapore	A/Singapore/GP674/2009
PA	JX625410	H1N1	06/23/2009	United Ki	A/England/92880002/2009
PA	CY047882	H1N1	06/23/2009	Argentina	A/Argentina/9180/2009
PA	CY047970	H1N1	06/23/2009	Argentina	A/Argentina/9721/2009
PA	CY047898	H1N1	06/23/2009	Argentina	A/Argentina/9384/2009
PA	CY051484	H1N1	06/23/2009	USA	A/Wisconsin/629-S0410/2009(H1N1)
PA	CY052151	H1N1	06/24/2009	USA	A/New York/4398/2009(H1N1)
PA	CY051476	H1N1	06/24/2009	USA	A/Wisconsin/629-S0435/2009(H1N1)
PA	GU138946	H1N1	06/24/2009	Japan	A/Iwate/3/2009(H1N1)
PA	CY049307	H1N1	06/24/2009	Singapore	A/Singapore/ON402/2009
PA	CY123964	H1N1	06/24/2009	Singapore	A/Singapore/ON402/2009(H1N1)
PA	CY107692	H1N1	06/24/2009	United Ki	A/Scotland/Glasgow_422823/2009
PA	CY107796	H1N1	06/24/2009	United Ki	A/Scotland/EastKilbride_420880/20
PA	CY073981	H1N1	06/24/2009	Argentina	A/Argentina/HNRG105/2009
PA	CY087221	H1N1	06/24/2009	USA	A/Houston/210S/2009
PA	CY064617	H1N1	06/24/2009	USA	A/Boston/131/2009(H1N1)
PA	CY074027	H1N1	06/24/2009	Argentina	A/Argentina/HNRG33/2009(H1N1)
PA	CY074037	H1N1	06/24/2009	Argentina	A/Argentina/HNRG40/2009(H1N1)
PA	CY054784	H1N1	06/24/2009	USA	A/California/VRDL22/2009(H1N1)
PA	CY054832	H1N1	06/24/2009	USA	A/California/VRDL29/2009(H1N1)
PA	KF612073	H1N1	06/24/2009	Bolivia	A/Santa Cruz/46631/2009
PA	KT180765	H1N1	06/24/2009	Singapore	A/Singapore/DMS5/2009
PA	KT180885	H1N1	06/24/2009	Singapore	A/Singapore/DMS223/2009
PA	CY107803	H1N1	06/24/2009	United Ki	A/Scotland/EastKilbride_420881/20
PA	CY107486	H1N1	06/24/2009	United Ki	A/Scotland/Edinburgh_17714/2009
PA	CY107539	H1N1	06/24/2009	United Ki	A/Scotland/Fife_17690/2009
PA	CY107789	H1N1	06/24/2009	United Ki	A/Scotland/EastKilbride_420875/20
PA	CY107325	H1N1	06/24/2009	United Ki	A/Scotland/Edinburgh_17715/2009
PA	CY107464	H1N1	06/24/2009	United Ki	A/Scotland/Fife_17712/2009
PA	CY107681	H1N1	06/24/2009	United Ki	A/Scotland/Glasgow_420755/2009
PA	CY107818	H1N1	06/24/2009	United Ki	A/Scotland/StAndrews_17693/2009
PA	CY047922	H1N1	06/24/2009	Argentina	A/Argentina/9579/2009
PA	CY047954	H1N1	06/24/2009	Argentina	A/Argentina/9705/2009
PA	CY047914	H1N1	06/24/2009	Argentina	A/Argentina/9576/2009
PA	CY047946	H1N1	06/24/2009	Argentina	A/Argentina/9597/2009
PA	CY047938	H1N1	06/24/2009	Argentina	A/Argentina/9588/2009
PA	CY047930	H1N1	06/24/2009	Argentina	A/Argentina/9583/2009
PA	CY083622	H1N1	06/24/2009	Peru	A/Trujillo/WRAIR9309F/2009
PA	CY107478	H1N1	06/24/2009	United Ki	A/Scotland/Edinburgh_17645/2009
PA	CY107778	H1N1	06/24/2009	United Ki	A/Scotland/EastKilbride_420703/20
PA	CY044146	H1N1	06/25/2009	Colombia	A/Bogota/0466N/2009(H1N1)
PA	CY083526	H1N1	06/25/2009	Peru	A/Lima/WRAIR1696P/2009
PA	CY052159	H1N1	06/25/2009	USA	A/New York/4401/2009(H1N1)
PA	CY049978	H1N1	06/25/2009	Dominican	A/Santo Domingo/WR1068N/2009(H1N1
PA	CY049938	H1N1	06/25/2009	Dominican	A/Santo Domingo/WR1056T/2009(H1N1
PA	CY050187	H1N1	06/25/2009	USA	A/New York/4438/2009(H1N1)
PA	CY051532	H1N1	06/25/2009	USA	A/New York/4400/2009(H1N1)
PA	GU138945	H1N1	06/25/2009	Japan	A/Hiroshima/230/2009(H1N1)
PA	CY049315	H1N1	06/25/2009	Singapore	A/Singapore/ON439/2009(H1N1)
PA	CY049379	H1N1	06/25/2009	Singapore	A/Singapore/ON442/2009(H1N1)
PA	CY049395	H1N1	06/25/2009	Singapore	A/Singapore/ON506/2009(H1N1)
PA	CY049099	H1N1	06/25/2009	Singapore	A/Singapore/ON511/2009(H1N1)
PA	CY049387	H1N1	06/25/2009	Singapore	A/Singapore/ON496/2009(H1N1)

PA	CY049155	H1N1	06/25/2009	Singapore	A/Singapore/ON507/2009(H1N1)
PA	CY049323	H1N1	06/25/2009	Singapore	A/Singapore/ON478/2009(H1N1)
PA	CY075256	H1N1	06/25/2009	Chile	A/Chile/2909/2009(H1N1)
PA	CY075264	H1N1	06/25/2009	Chile	A/Chile/2911/2009(H1N1)
PA	CY083566	H1N1	06/25/2009	Peru	A/Lima/WRAIR8511F/2009
PA	CY064681	H1N1	06/25/2009	USA	A/Boston/141/2009(H1N1)
PA	CY057227	H1N1	06/25/2009	Nicaragua	A/Managua/611.01/2009(H1N1)
PA	CY058209	H1N1	06/25/2009	Nicaragua	A/Managua/791.03/2009(H1N1)
PA	KJ130195	H1N1	06/25/2009	New Zeala	A/New Zealand/2047/2009
PA	KC781505	H1N1	06/25/2009	USA	A/Utah/22/2009
PA	CY052311	H1N1	06/25/2009	USA	A/Texas/42254309/2009(H1N1)
PA	CY052119	H1N1	06/26/2009	USA	A/Wisconsin/629-D01973/2009(H1N1)
PA	GU138951	H1N1	06/26/2009	Myanmar	A/Myanmar/60/2009(H1N1)
PA	CY049187	H1N1	06/26/2009	Singapore	A/Singapore/ON594/2009(H1N1)
PA	CY049347	H1N1	06/26/2009	Singapore	A/Singapore/ON593/2009(H1N1)
PA	CY049403	H1N1	06/26/2009	Singapore	A/Singapore/ON575/2009(H1N1)
PA	CY049163	H1N1	06/26/2009	Singapore	A/Singapore/ON584/2009(H1N1)
PA	CY049171	H1N1	06/26/2009	Singapore	A/Singapore/ON588/2009(H1N1)
PA	CY049107	H1N1	06/26/2009	Singapore	A/Singapore/ON581/2009(H1N1)
PA	CY049339	H1N1	06/26/2009	Singapore	A/Singapore/ON582/2009(H1N1)
PA	CY123995	H1N1	06/26/2009	Singapore	A/Singapore/ON496/2009
PA	CY049179	H1N1	06/26/2009	Singapore	A/Singapore/ON590/2009(H1N1)
PA	CY044154	H1N1	06/26/2009	Nicaragua	A/Managua/0536N/2009(H1N1)
PA	CY075272	H1N1	06/26/2009	Chile	A/Chile/2994/2009(H1N1)
PA	CY075288	H1N1	06/26/2009	Chile	A/Chile/3056/2009
PA	CY075296	H1N1	06/26/2009	Chile	A/Chile/3123/2009(H1N1)
PA	CY075280	H1N1	06/26/2009	Chile	A/Chile/3019/2009(H1N1)
PA	CY058545	H1N1	06/26/2009	Nicaragua	A/Managua/462.01/2009(H1N1)
PA	CY064569	H1N1	06/26/2009	USA	A/Boston/124/2009(H1N1)
PA	CY064641	H1N1	06/26/2009	USA	A/Boston/134/2009(H1N1)
PA	CY064521	H1N1	06/26/2009	USA	A/Boston/117/2009(H1N1)
PA	CY064900	H1N1	06/26/2009	USA	A/Boston/136/2009(H1N1)
PA	CY054760	H1N1	06/26/2009	USA	A/California/VRDL19/2009(H1N1)
PA	KF612080	H1N1	06/26/2009	Bolivia	A/Santa Cruz/97831/2009
PA	CY124019	H1N1	06/26/2009	Singapore	A/Singapore/ON547/2009
PA	CY123979	H1N1	06/26/2009	Singapore	A/Singapore/ON477/2009
PA	JX625386	H1N1	06/26/2009	United Ki	A/England/506/2009
PA	KC782294	H1N1	06/26/2009	USA	A/Texas/48/2009
PA	CY053171	H1N1	06/26/2009	USA	A/Houston/240S/2009(H1N1)
PA	GU290046	H1N1	06/27/2009	Czech Rep	A/Prague/197-81395/2009(H1N1)
PA	CY051212	H1N1	06/27/2009	USA	A/Wisconsin/629-D01414/2009(H1N1)
PA	CY124027	H1N1	06/27/2009	Singapore	A/Singapore/ON575/2009
PA	CY049211	H1N1	06/27/2009	Singapore	A/Singapore/ON635/2009(H1N1)
PA	CY049203	H1N1	06/27/2009	Singapore	A/Singapore/ON628/2009(H1N1)
PA	CY083454	H1N1	06/27/2009	Peru	A/Lima/WRAIR1687P/2009
PA	CY049195	H1N1	06/27/2009	Singapore	A/Singapore/ON621/2009(H1N1)
PA	CY073988	H1N1	06/27/2009	Argentina	A/Argentina/HNRG106/2009
PA	CY083582	H1N1	06/27/2009	Peru	A/Lima/WRAIR8689F/2009(H1N1)
PA	CY054816	H1N1	06/27/2009	USA	A/California/VRDL27/2009(H1N1)
PA	CY124034	H1N1	06/27/2009	Singapore	A/Singapore/ON580/2009
PA	CY049219	H1N1	06/27/2009	Singapore	A/Singapore/ON639/2009(H1N1)
PA	CY052191	H1N1	06/28/2009	USA	A/Texas/42281289/2009(H1N1)
PA	KT180766	H1N1	06/28/2009	Singapore	A/Singapore/DMS7/2009
PA	CY054840	H1N1	06/28/2009	USA	A/California/VRDL30/2009(H1N1)

PA	CY052404	H1N1	06/29/2009	USA	A/Texas/42291877/2009(H1N1)
PA	GU138960	H1N1	06/29/2009	Japan	A/Saitama/85/2009(H1N1)
PA	CY049994	H1N1	06/29/2009	Mexico	A/Mexico City/WR1100N/2009(H1N1)
PA	CY058201	H1N1	06/29/2009	Nicaragua	A/Managua/1127.02/2009(H1N1)
PA	CY123353	H1N1	06/29/2009	Singapore	A/Singapore/GP835/2009
PA	CY064513	H1N1	06/29/2009	USA	A/Boston/116/2009(H1N1)
PA	CY054792	H1N1	06/29/2009	USA	A/California/VRDL23/2009(H1N1)
PA	CY058185	H1N1	06/29/2009	Nicaragua	A/Managua/3189.01/2009(H1N1)
PA	CY063563	H1N1	06/29/2009	USA	A/Boston/152/2009(H1N1)
PA	JX625394	H1N1	06/30/2009	United Ki	A/England/527/2009
PA	CY123361	H1N1	06/30/2009	Singapore	A/Singapore/GP875/2009
PA	CY069756	H1N1	06/30/2009	United Ki	A/England/633/2009
PA	CY120106	H1N1	06/30/2009	Hong Kong	A/Hong Kong/H090-663-V10/2009
PA	CY111624	H1N1	06/30/2009	Hong Kong	A/Hong Kong/H090-663-V10/2009
PA	CY073417	H1N1	06/30/2009	USA	A/San Diego/WR1640P/2009(H1N1)
PA	CY075328	H1N1	06/30/2009	Chile	A/Chile/3295/2009(H1N1)
PA	CY075304	H1N1	06/30/2009	Chile	A/Chile/3220/2009(H1N1)
PA	CY075312	H1N1	06/30/2009	Chile	A/Chile/3242/2009(H1N1)
PA	CY075320	H1N1	06/30/2009	Chile	A/Chile/3244/2009(H1N1)
PA	CY064689	H1N1	06/30/2009	USA	A/Boston/142/2009(H1N1)
PA	CY054800	H1N1	06/30/2009	USA	A/California/VRDL25/2009(H1N1)
PA	CY054872	H1N1	06/30/2009	USA	A/California/VRDL34/2009(H1N1)
PA	CY058193	H1N1	06/30/2009	Nicaragua	A/Managua/5586.01/2009(H1N1)
PA	CY051220	H1N1	06/30/2009	USA	A/Wisconsin/629-D02292/2009(H1N1)
PA	CY052207	H1N1	06/30/2009	USA	A/Texas/42303371/2009(H1N1)
PA	CY049962	H1N1	06/30/2009	Dominican	A/Santo Domingo/WR1059N/2009(H1N1
PA	CY051652	H1N1	06/30/2009	USA	A/New York/4691/2009(H1N1)
PA	CY051492	H1N1	06/30/2009	USA	A/Wisconsin/629-S0464/2009(H1N1)
PA	GU138943	H1N1	06/30/2009	Japan	A/Hiroshima/216/2009(H1N1)
PA	CY069748	H1N1	06/30/2009	United Ki	A/England/527/2009
PA	GQ866925	H1N1	07/2009	Thailand	A/Thailand/CU-H276/2009(H1N1)
PA	GQ866924	H1N1	07/2009	Thailand	A/Thailand/CU-H106/2009(H1N1)
PA	CY047743	H1N1	07/2009	Taiwan	A/Taiwan/526/2009(H1N1)
PA	CY063547	H1N1	07/01/2009	USA	A/Boston/149/2009(H1N1)
PA	CY107810	H1N1	07/01/2009	United Ki	A/Scotland/EastKilbride_425383/20
PA	CY073409	H1N1	07/01/2009	USA	A/San Diego/WR1639P/2009(H1N1)
PA	CY051580	H1N1	07/01/2009	USA	A/New York/4500/2009(H1N1)
PA	CY051572	H1N1	07/01/2009	USA	A/New York/4476/2009(H1N1)
PA	CY074012	H1N1	07/01/2009	Argentina	A/Argentina/HNRG21/2009(H1N1)
PA	CY054936	H1N1	07/01/2009	USA	A/California/VRDL45/2009(H1N1)
PA	CY054952	H1N1	07/01/2009	USA	A/California/VRDL50/2009(H1N1)
PA	CY054856	H1N1	07/01/2009	USA	A/California/VRDL32/2009(H1N1)
PA	CY054912	H1N1	07/01/2009	USA	A/California/VRDL40/2009(H1N1)
PA	GQ465732	H1N1	07/01/2009	Canada	A/Canada-SK/RV2486/2009(H1N1)
PA	KC781605	H1N1	07/01/2009	USA	A/Rhode Island/07/2009
PA	GU138942	H1N1	07/01/2009	Japan	A/Hiroshima/207/2009(H1N1)
PA	CY051836	H1N1	07/01/2009	USA	A/Texas/43011033/2009(H1N1)
PA	CY083590	H1N1	07/01/2009	Peru	A/Lima/WRAIR8893F/2009(H1N1)
PA	JX625426	H1N1	07/01/2009	United Ki	A/England/92920018/2009
PA	JX625418	H1N1	07/01/2009	United Ki	A/England/667/2009
PA	CY065173	H1N1	07/01/2009	United Ki	A/England/634/2009
PA	CY075336	H1N1	07/01/2009	Chile	A/Chile/3349/2009(H1N1)
PA	CY075344	H1N1	07/01/2009	Chile	A/Chile/3361/2009(H1N1)
PA	CY075352	H1N1	07/01/2009	Chile	A/Chile/3369/2009(H1N1)

PA	CY075360	H1N1	07/01/2009	Chile	A/Chile/3375/2009(H1N1)
PA	CY063523	H1N1	07/01/2009	USA	A/Boston/146/2009(H1N1)
PA	KP637525	H1N1	07/01/2009	Thailand	A/Thailand/SN08270/2009
PA	JX625402	H1N1	07/01/2009	United Ki	A/England/685/2009
PA	KF612072	H1N1	07/01/2009	Bolivia	A/Santa Cruz/12541/2009
PA	KF612076	H1N1	07/01/2009	Bolivia	A/Santa Cruz/21641/2009
PA	CY063779	H1N1	07/01/2009	Singapore	A/Singapore/ON0801/2009(H1N1)
PA	CY054864	H1N1	07/02/2009	USA	A/California/VRDL33/2009(H1N1)
PA	CY124106	H1N1	07/02/2009	Singapore	A/Singapore/ON809/2009
PA	GU271957	H1N1	07/02/2009	Thailand	A/Thailand/CU-B267/2009(H1N1)
PA	CY049954	H1N1	07/02/2009	Dominican	A/Santo Domingo/WR1058N/2009(H1N1
PA	CY049946	H1N1	07/02/2009	Dominican	A/Santo Domingo/WR1057N/2009(H1N1
PA	CY111632	H1N1	07/02/2009	Hong Kong	A/Hong Kong/H090-665-V10/2009
PA	CY120114	H1N1	07/02/2009	Hong Kong	A/Hong Kong/H090-665-V10/2009
PA	CY111648	H1N1	07/02/2009	Hong Kong	A/Hong Kong/H090-667-V23/2009
PA	CY120066	H1N1	07/02/2009	Hong Kong	A/Hong Kong/H090-667-V23/2009
PA	CY111640	H1N1	07/02/2009	Hong Kong	A/Hong Kong/H090-667-V10/2009
PA	CY120122	H1N1	07/02/2009	Hong Kong	A/Hong Kong/H090-667-V10/2009
PA	CY072763	H1N1	07/02/2009	Nicaragua	A/Managua/435.01/2009(H1N1)
PA	CY072779	H1N1	07/02/2009	Nicaragua	A/Managua/5235.03/2009(H1N1)
PA	CY075368	H1N1	07/02/2009	Chile	A/Chile/3467/2009(H1N1)
PA	CY075376	H1N1	07/02/2009	Chile	A/Chile/3553/2009(H1N1)
PA	CY073995	H1N1	07/02/2009	Argentina	A/Argentina/HNRG107/2009
PA	KP637789	H1N1	07/02/2009	Thailand	A/Thailand/SS08191/2009
PA	CY124122	H1N1	07/02/2009	Singapore	A/Singapore/ON812/2009
PA	KF612074	H1N1	07/02/2009	Bolivia	A/Santa Cruz/94841/2009
PA	CY124114	H1N1	07/02/2009	Singapore	A/Singapore/ON811/2009
PA	CY054896	H1N1	07/02/2009	USA	A/California/VRDL38/2009(H1N1)
PA	GU138944	H1N1	07/03/2009	Japan	A/Hiroshima/220/2009(H1N1)
PA	CY083614	H1N1	07/03/2009	Peru	A/Lima/WRAIR9202F/2009(H1N1)
PA	CY075384	H1N1	07/03/2009	Chile	A/Chile/3586/2009(H1N1)
PA	CY122554	H1N1	07/03/2009	Singapore	A/Singapore/GP1020/2009
PA	CY083462	H1N1	07/03/2009	Peru	A/Lima/WRAIR1688P/2009
PA	CY124138	H1N1	07/03/2009	Singapore	A/Singapore/ON819/2009
PA	CY124145	H1N1	07/03/2009	Singapore	A/Singapore/ON822/2009
PA	CY124130	H1N1	07/03/2009	Singapore	A/Singapore/ON816/2009
PA	CY087029	H1N1	07/03/2009	USA	A/New York/4557/2009(H1N1)
PA	CY049235	H1N1	07/04/2009	Singapore	A/Singapore/ON896/2009(H1N1)
PA	CY049251	H1N1	07/04/2009	Singapore	A/Singapore/ON901/2009(H1N1)
PA	CY049442	H1N1	07/04/2009	Singapore	A/Singapore/ON912/2009(H1N1)
PA	CY049418	H1N1	07/04/2009	Singapore	A/Singapore/ON903/2009(H1N1)
PA	CY049227	H1N1	07/04/2009	Singapore	A/Singapore/ON894/2009(H1N1)
PA	CY062516	H1N1	07/04/2009	Mexico	A/Mexico city/CIA5/2009(H1N1)
PA	CY049434	H1N1	07/04/2009	Singapore	A/Singapore/ON907/2009(H1N1)
PA	CY049426	H1N1	07/04/2009	Singapore	A/Singapore/ON905/2009(H1N1)
PA	CY062490	H1N1	07/04/2009	Mexico	A/Mexico city/CIA1/2009(H1N1)
PA	CY049243	H1N1	07/04/2009	Singapore	A/Singapore/ON899/2009(H1N1)
PA	CY054880	H1N1	07/05/2009	USA	A/California/VRDL35/2009(H1N1)
PA	CY049115	H1N1	07/05/2009	Singapore	A/Singapore/ON923/2009(H1N1)
PA	CY049458	H1N1	07/05/2009	Singapore	A/Singapore/ON925/2009(H1N1)
PA	CY049450	H1N1	07/05/2009	Singapore	A/Singapore/ON921/2009(H1N1)
PA	CY074078	H1N1	07/05/2009	Argentina	A/Argentina/HNRG84/2009
PA	KT180767	H1N1	07/05/2009	Singapore	A/Singapore/DMS10/2009
PA	JX625450	H1N1	07/05/2009	United Ki	A/England/671/2009

PA	CY063555	H1N1	07/06/2009	USA	A/Boston/151/2009(H1N1)
PA	CY069255	H1N1	07/06/2009	Nicaragua	A/Managua/4943.09/2009(H1N1)
PA	CY054928	H1N1	07/06/2009	USA	A/California/VRDL43/2009(H1N1)
PA	CY045510	H1N1	07/06/2009	Canada	A/Toronto/R8564/2009(H1N1)
PA	KC781695	H1N1	07/06/2009	USA	A/Georgia/08/2009
PA	CY049267	H1N1	07/06/2009	Singapore	A/Singapore/ON978/2009(H1N1)
PA	CY049275	H1N1	07/06/2009	Singapore	A/Singapore/ON980/2009(H1N1)
PA	CY049259	H1N1	07/06/2009	Singapore	A/Singapore/ON975/2009(H1N1)
PA	CY049283	H1N1	07/06/2009	Singapore	A/Singapore/ON981/2009(H1N1)
PA	CY083343	H1N1	07/06/2009	USA	A/San Diego/WRAIR1656P/2009
PA	CY065165	H1N1	07/06/2009	United Ki	A/England/92860032/2009
PA	CY072571	H1N1	07/06/2009	Nicaragua	A/Managua/4197.01/2009(H1N1)
PA	CY075408	H1N1	07/06/2009	Chile	A/Chile/3819/2009(H1N1)
PA	CY075400	H1N1	07/06/2009	Chile	A/Chile/3766/2009(H1N1)
PA	CY075392	H1N1	07/06/2009	Chile	A/Chile/3760/2009(H1N1)
PA	CY062497	H1N1	07/06/2009	Mexico	A/Mexico city/CIA2/2009(H1N1)
PA	CY062504	H1N1	07/06/2009	Mexico	A/Mexico city/CIA3/2009(H1N1)
PA	KP222616	H1N1	07/06/2009	Singapore	A/Singapore/376Y/2009
PA	CY122586	H1N1	07/06/2009	Singapore	A/Singapore/GP1092/2009
PA	CY122610	H1N1	07/06/2009	Singapore	A/Singapore/GP1099/2009
PA	CY122686	H1N1	07/06/2009	Singapore	A/Singapore/GP1137/2009
PA	CY122594	H1N1	07/06/2009	Singapore	A/Singapore/GP1094/2009
PA	CY122602	H1N1	07/06/2009	Singapore	A/Singapore/GP1097/2009
PA	CY083630	H1N1	07/06/2009	Peru	A/Arequipa/WRAIR9939F/2009
PA	CY063539	H1N1	07/07/2009	USA	A/Boston/148/2009(H1N1)
PA	CY045953	H1N1	07/07/2009	Canada	A/Toronto/T0106/2009(H1N1)
PA	GU138959	H1N1	07/07/2009	Japan	A/Niigata/749/2009(H1N1)
PA	GU138939	H1N1	07/07/2009	Japan	A/Gifu-C/67/2009(H1N1)
PA	CY120130	H1N1	07/07/2009	Hong Kong	A/Hong Kong/H090-678-V10/2009
PA	CY111656	H1N1	07/07/2009	Hong Kong	A/Hong Kong/H090-678-V10/2009
PA	CY088680	H1N1	07/07/2009	India	A/Mum/NIV398/2009(H1N1)
PA	CY073890	H1N1	07/07/2009	Nicaragua	A/Managua/2729.02/2009(H1N1)
PA	CY075424	H1N1	07/07/2009	Chile	A/Chile/3935/2009(H1N1)
PA	CY075416	H1N1	07/07/2009	Chile	A/Chile/3905/2009(H1N1)
PA	CY122654	H1N1	07/07/2009	Singapore	A/Singapore/GP1124/2009
PA	CY122618	H1N1	07/07/2009	Singapore	A/Singapore/GP1102/2009
PA	CY122678	H1N1	07/07/2009	Singapore	A/Singapore/GP1136/2009
PA	CY122570	H1N1	07/07/2009	Singapore	A/Singapore/GP1087/2009
PA	CY122578	H1N1	07/07/2009	Singapore	A/Singapore/GP1090/2009
PA	CY122646	H1N1	07/07/2009	Singapore	A/Singapore/GP1122/2009
PA	CY053163	H1N1	07/07/2009	USA	A/Houston/23H/2009(H1N1)
PA	CY064697	H1N1	07/08/2009	USA	A/Boston/143/2009(H1N1)
PA	CY122770	H1N1	07/08/2009	Singapore	A/Singapore/GP1178/2009
PA	CY051612	H1N1	07/08/2009	USA	A/New York/4567/2009(H1N1)
PA	GU138952	H1N1	07/08/2009	Myanmar	A/Myanmar/JP90/2009(H1N1)
PA	CY050195	H1N1	07/08/2009	USA	A/New York/4576/2009(H1N1)
PA	CY051604	H1N1	07/08/2009	USA	A/New York/4566/2009(H1N1)
PA	CY111664	H1N1	07/08/2009	Hong Kong	A/Hong Kong/H090-681-V10/2009
PA	CY120138	H1N1	07/08/2009	Hong Kong	A/Hong Kong/H090-681-V10/2009
PA	CY071473	H1N1	07/08/2009	USA	A/California/WR1313P/2009(H1N1)
PA	CY071521	H1N1	07/08/2009	USA	A/California/WR1319P/2009(H1N1)
PA	CY083095	H1N1	07/08/2009	Peru	A/Lima/WRAIR0519F/2009
PA	CY122762	H1N1	07/08/2009	Singapore	A/Singapore/GP1174/2009
PA	CY122662	H1N1	07/08/2009	Singapore	A/Singapore/GP1127/2009

PA	CY122702	H1N1	07/08/2009	Singapore	A/Singapore/GP1146/2009
PA	CY122710	H1N1	07/08/2009	Singapore	A/Singapore/GP1147/2009
PA	KT180775	H1N1	07/08/2009	Singapore	A/Singapore/DMS11/2009
PA	CY122738	H1N1	07/08/2009	Singapore	A/Singapore/GP1164/2009
PA	CY122746	H1N1	07/08/2009	Singapore	A/Singapore/GP1167/2009
PA	CY122670	H1N1	07/08/2009	Singapore	A/Singapore/GP1132/2009
PA	CY122694	H1N1	07/08/2009	Singapore	A/Singapore/GP1142/2009
PA	CY122730	H1N1	07/08/2009	Singapore	A/Singapore/GP1163/2009
PA	CY122638	H1N1	07/08/2009	Singapore	A/Singapore/GP1120/2009
PA	CY122754	H1N1	07/08/2009	Singapore	A/Singapore/GP1169/2009
PA	CY051596	H1N1	07/08/2009	USA	A/New York/4565/2009(H1N1)
PA	CY063515	H1N1	07/09/2009	USA	A/Boston/145/2009(H1N1)
PA	CY095978	H1N1	07/09/2009	China	A/Zhejiang/9/2009
PA	CY066188	H1N1	07/09/2009	USA	A/Boston/144/2009(H1N1)
PA	CY054904	H1N1	07/09/2009	USA	A/California/VRDL39/2009(H1N1)
PA	GQ392020	H1N1	07/09/2009	Russia	A/Moscow/IIV04/2009(H1N1)
PA	CY049482	H1N1	07/09/2009	Singapore	A/Singapore/ON1067/2009(H1N1)
PA	CY049538	H1N1	07/09/2009	Singapore	A/Singapore/ON1118/2009(H1N1)
PA	CY049490	H1N1	07/09/2009	Singapore	A/Singapore/ON1072/2009(H1N1)
PA	CY051588	H1N1	07/09/2009	USA	A/New York/4556/2009(H1N1)
PA	CY051620	H1N1	07/09/2009	USA	A/New York/4568/2009(H1N1)
PA	CY111704	H1N1	07/09/2009	Hong Kong	A/Hong Kong/H090-684-V10/2009
PA	CY120170	H1N1	07/09/2009	Hong Kong	A/Hong Kong/H090-684-V10/2009
PA	CY075440	H1N1	07/09/2009	Chile	A/Chile/4181/2009(H1N1)
PA	CY075432	H1N1	07/09/2009	Chile	A/Chile/4064/2009(H1N1)
PA	KP637717	H1N1	07/09/2009	Thailand	A/Thailand/SR08021/2009
PA	KP637797	H1N1	07/09/2009	Thailand	A/Thailand/SS08193/2009
PA	CY095905	H1N1	07/09/2009	China	A/Zhejiang/8/2009
PA	CY123397	H1N1	07/09/2009	Singapore	A/Singapore/ON1074/2009
PA	CY122778	H1N1	07/09/2009	Singapore	A/Singapore/GP1182/2009
PA	CY095720	H1N1	07/09/2009	China	A/Zhejiang/X1/2009
PA	CY095740	H1N1	07/09/2009	China	A/Zhejiang/11/2009
PA	CY095727	H1N1	07/09/2009	China	A/Zhejiang/10/2009
PA	CY095840	H1N1	07/09/2009	China	A/Zhejiang/7/2009
PA	CY095762	H1N1	07/09/2009	China	A/Zhejiang/3/2009
PA	CY095816	H1N1	07/09/2009	China	A/Zhejiang/5/2009
PA	CY095747	H1N1	07/09/2009	China	A/Zhejiang/X2/2009
PA	CY095776	H1N1	07/09/2009	China	A/Zhejiang/4/2009
PA	CY063531	H1N1	07/09/2009	USA	A/Boston/147/2009(H1N1)
PA	CY065181	H1N1	07/10/2009	United Ki	A/England/675/2009
PA	CY075448	H1N1	07/10/2009	Chile	A/Chile/4182/2009(H1N1)
PA	CY120186	H1N1	07/10/2009	Hong Kong	A/Hong Kong/H090-693-V10/2009
PA	CY111720	H1N1	07/10/2009	Hong Kong	A/Hong Kong/H090-693-V10/2009
PA	CY111672	H1N1	07/10/2009	Hong Kong	A/Hong Kong/H090-681-V22/2009
PA	CY054944	H1N1	07/10/2009	USA	A/California/VRDL48/2009(H1N1)
PA	GU183798	H1N1	07/10/2009	Thailand	A/Thailand/CU-B589/2009(H1N1)
PA	KT180813	H1N1	07/11/2009	Singapore	A/Singapore/DMS12/2009
PA	CY111688	H1N1	07/11/2009	Hong Kong	A/Hong Kong/H090-683-V10/2009
PA	JX625442	H1N1	07/11/2009	United Ki	A/England/687/2009
PA	CY054920	H1N1	07/11/2009	USA	A/California/VRDL41/2009(H1N1)
PA	CY054960	H1N1	07/11/2009	USA	A/California/VRDL51/2009(H1N1)
PA	CY106573	H1N1	07/11/2009	Mexico	A/Merida/2189-CIR/2009
PA	GU138949	H1N1	07/11/2009	Japan	A/Mie/41/2009(H1N1)
PA	CY120154	H1N1	07/11/2009	Hong Kong	A/Hong Kong/H090-683-V10/2009

PA	CY049466	H1N1	07/12/2009	Singapore	A/Singapore/ON1050/2009(H1N1)
PA	CY049474	H1N1	07/12/2009	Singapore	A/Singapore/ON1060/2009(H1N1)
PA	GU183806	H1N1	07/12/2009	Thailand	A/Thailand/CU-B685/2009(H1N1)
PA	CY052428	H1N1	07/12/2009	USA	A/Texas/43122467/2009(H1N1)
PA	CY072963	H1N1	07/12/2009	Nicaragua	A/Managua/585.01/2009(H1N1)
PA	CY072883	H1N1	07/12/2009	Nicaragua	A/Managua/4606.06/2009(H1N1)
PA	CY049546	H1N1	07/12/2009	Singapore	A/Singapore/ON1126/2009(H1N1)
PA	KP637653	H1N1	07/13/2009	Thailand	A/Thailand/SP08279/2009
PA	CY123373	H1N1	07/13/2009	Singapore	A/Singapore/ON1058/2009
PA	CY123381	H1N1	07/13/2009	Singapore	A/Singapore/ON1063/2009
PA	CY123389	H1N1	07/13/2009	Singapore	A/Singapore/ON1069/2009
PA	CY111752	H1N1	07/13/2009	Hong Kong	A/Hong Kong/H090-701-V10/2009
PA	CY120202	H1N1	07/13/2009	Hong Kong	A/Hong Kong/H090-701-V10/2009
PA	CY111760	H1N1	07/13/2009	Hong Kong	A/Hong Kong/H090-704-V10/2009
PA	CY120210	H1N1	07/13/2009	Hong Kong	A/Hong Kong/H090-704-V10/2009
PA	CY111728	H1N1	07/13/2009	Hong Kong	A/Hong Kong/H090-693-V20/2009
PA	CY120073	H1N1	07/13/2009	Hong Kong	A/Hong Kong/H090-693-V20/2009
PA	CY120081	H1N1	07/13/2009	Hong Kong	A/Hong Kong/H090-698-V10/2009
PA	CY111736	H1N1	07/13/2009	Hong Kong	A/Hong Kong/H090-698-V10/2009
PA	CY120146	H1N1	07/13/2009	Hong Kong	A/Hong Kong/H090-681-V32/2009
PA	CY111680	H1N1	07/13/2009	Hong Kong	A/Hong Kong/H090-681-V32/2009
PA	CY083510	H1N1	07/13/2009	Peru	A/Piura/WRAIR1694P/2009(H1N1)
PA	JX625458	H1N1	07/13/2009	United Ki	A/England/688/2009
PA	CY075456	H1N1	07/13/2009	Chile	A/Chile/4257/2009(H1N1)
PA	CY083103	H1N1	07/13/2009	Peru	A/Piura/WRAIR0603F/2009(H1N1)
PA	CY083319	H1N1	07/13/2009	USA	A/Great Lakes/WRAIR1652P/2009
PA	CY052412	H1N1	07/13/2009	USA	A/Texas/43132503/2009(H1N1)
PA	KC782033	H1N1	07/13/2009	USA	A/Rhode Island/08/2009
PA	GU138964	H1N1	07/13/2009	Japan	A/Wakayama/57/2009(H1N1)
PA	CY049498	H1N1	07/13/2009	Singapore	A/Singapore/ON1087/2009(H1N1)
PA	CY049514	H1N1	07/13/2009	Singapore	A/Singapore/ON1099/2009(H1N1)
PA	CY049554	H1N1	07/13/2009	Singapore	A/Singapore/ON1138/2009(H1N1)
PA	CY049506	H1N1	07/13/2009	Singapore	A/Singapore/ON1093/2009(H1N1)
PA	CY049522	H1N1	07/13/2009	Singapore	A/Singapore/ON1105/2009(H1N1)
PA	CY049562	H1N1	07/13/2009	Singapore	A/Singapore/ON1144/2009(H1N1)
PA	CY054968	H1N1	07/14/2009	USA	A/California/VRDL52/2009(H1N1)
PA	CY052255	H1N1	07/14/2009	USA	A/Texas/43143450/2009(H1N1)
PA	GU138953	H1N1	07/14/2009	Myanmar	A/Myanmar/JP101/2009(H1N1)
PA	CY049570	H1N1	07/14/2009	Singapore	A/Singapore/ON1156/2009(H1N1)
PA	CY049586	H1N1	07/14/2009	Singapore	A/Singapore/ON1168/2009(H1N1)
PA	CY054984	H1N1	07/14/2009	USA	A/California/VRDL55/2009(H1N1)
PA	CY051636	H1N1	07/14/2009	USA	A/New York/4620/2009(H1N1)
PA	CY073702	H1N1	07/14/2009	Nicaragua	A/Managua/3490.03/2009(H1N1)
PA	CY120162	H1N1	07/14/2009	Hong Kong	A/Hong Kong/H090-683-V20/2009
PA	CY111696	H1N1	07/14/2009	Hong Kong	A/Hong Kong/H090-683-V20/2009
PA	CY120218	H1N1	07/14/2009	Hong Kong	A/Hong Kong/H090-708-V10/2009
PA	CY111768	H1N1	07/14/2009	Hong Kong	A/Hong Kong/H090-708-V10/2009
PA	CY111712	H1N1	07/14/2009	Hong Kong	A/Hong Kong/H090-684-V23/2009
PA	CY120178	H1N1	07/14/2009	Hong Kong	A/Hong Kong/H090-684-V23/2009
PA	CY049594	H1N1	07/14/2009	Singapore	A/Singapore/ON1177/2009(H1N1)
PA	CY049578	H1N1	07/14/2009	Singapore	A/Singapore/ON1163/2009(H1N1)
PA	CY051628	H1N1	07/14/2009	USA	A/New York/4607/2009(H1N1)
PA	CY123445	H1N1	07/14/2009	Singapore	A/Singapore/ON1114/2009
PA	CY122786	H1N1	07/14/2009	Singapore	A/Singapore/GP1358/2009

PA	CY123421	H1N1	07/14/2009	Singapore	A/Singapore/ON1095/2009
PA	CY123453	H1N1	07/14/2009	Singapore	A/Singapore/ON1121/2009
PA	CY123413	H1N1	07/14/2009	Singapore	A/Singapore/ON1091/2009
PA	KT180857	H1N1	07/14/2009	Singapore	A/Singapore/DMS13/2009
PA	CY123429	H1N1	07/14/2009	Singapore	A/Singapore/ON1102/2009
PA	CY123405	H1N1	07/14/2009	Singapore	A/Singapore/ON1079/2009
PA	CY123437	H1N1	07/14/2009	Singapore	A/Singapore/ON1107/2009
PA	CY111784	H1N1	07/15/2009	Hong Kong	A/Hong Kong/H090-712-V1_4/2009
PA	JX625434	H1N1	07/15/2009	United Ki	A/England/92960004/2009
PA	CY063571	H1N1	07/15/2009	USA	A/Boston/153/2009(H1N1)
PA	CY087038	H1N1	07/15/2009	USA	A/New York/4631/2009(H1N1)
PA	CY111776	H1N1	07/15/2009	Hong Kong	A/Hong Kong/H090-712-V10/2009
PA	CY049602	H1N1	07/15/2009	Singapore	A/Singapore/ON1187/2009(H1N1)
PA	GU290054	H1N1	07/15/2009	Czech Rep	A/Prague/196-81511/2009(H1N1)
PA	CY083047	H1N1	07/15/2009	Peru	A/Lima/WRAIR0285F/2009
PA	CY049610	H1N1	07/15/2009	Singapore	A/Singapore/ON1194/2009(H1N1)
PA	CY120234	H1N1	07/15/2009	Hong Kong	A/Hong Kong/H090-712-V14/2009
PA	CY120226	H1N1	07/15/2009	Hong Kong	A/Hong Kong/H090-712-V10/2009
PA	CY055531	H1N1	07/16/2009	Australia	A/Australia/1/2009(H1N1)
PA	CY111744	H1N1	07/16/2009	Hong Kong	A/Hong Kong/H090-698-V20/2009
PA	CY120194	H1N1	07/16/2009	Hong Kong	A/Hong Kong/H090-698-V20/2009
PA	CY111808	H1N1	07/16/2009	Hong Kong	A/Hong Kong/H090-715-V10/2009
PA	CY072899	H1N1	07/16/2009	Nicaragua	A/Managua/3214.04/2009(H1N1)
PA	CY072843	H1N1	07/16/2009	Nicaragua	A/Managua/265.01/2009(H1N1)
PA	CY083358	H1N1	07/16/2009	USA	A/San Diego/WRAIR1658P/2009
PA	CY051644	H1N1	07/16/2009	USA	A/New York/4677/2009(H1N1)
PA	GU183814	H1N1	07/16/2009	Thailand	A/Thailand/CU-H223/2009(H1N1)
PA	CY055064	H1N1	07/17/2009	Australia	A/Australia/3/2009(H1N1)
PA	CY083135	H1N1	07/17/2009	Peru	A/Lima/WRAIR0681F/2009
PA	CY075464	H1N1	07/17/2009	Chile	A/Chile/4406/2009(H1N1)
PA	JX625466	H1N1	07/17/2009	United Ki	A/England/92980479/2009
PA	CY111816	H1N1	07/17/2009	Hong Kong	A/Hong Kong/H090-721-V10/2009
PA	CY120258	H1N1	07/17/2009	Hong Kong	A/Hong Kong/H090-721-V10/2009
PA	CY120266	H1N1	07/17/2009	Hong Kong	A/Hong Kong/H090-722-V10/2009
PA	CY111824	H1N1	07/17/2009	Hong Kong	A/Hong Kong/H090-722-V10/2009(H1N
PA	CY097883	H1N1	07/17/2009	USA	A/South Carolina/WRAIR1645P/2009
PA	CY083279	H1N1	07/17/2009	USA	A/South Carolina/WRAIR1645P/2009
PA	CY055072	H1N1	07/17/2009	Australia	A/Australia/4/2009(H1N1)
PA	KT180866	H1N1	07/17/2009	Singapore	A/Singapore/DMS14/2009
PA	CY111792	H1N1	07/18/2009	Hong Kong	A/Hong Kong/H090-712-V20/2009
PA	CY120242	H1N1	07/18/2009	Hong Kong	A/Hong Kong/H090-712-V20/2009
PA	CY111800	H1N1	07/18/2009	Hong Kong	A/Hong Kong/H090-712-V24/2009
PA	CY120250	H1N1	07/18/2009	Hong Kong	A/Hong Kong/H090-712-V24/2009
PA	CY055539	H1N1	07/18/2009	Australia	A/Australia/7/2009(H1N1)
PA	CY083350	H1N1	07/18/2009	USA	A/San Diego/WRAIR1657P/2009
PA	GU138963	H1N1	07/18/2009	Japan	A/Tokushima/2/2009(H1N1)
PA	CY055080	H1N1	07/18/2009	Australia	A/Australia/6/2009(H1N1)
PA	CY083127	H1N1	07/19/2009	Peru	A/Lima/WRAIR0672F/2009
PA	GU433030	H1N1	07/19/2009	Russia	A/Tomsk/03/2009(H1N1)
PA	CY055547	H1N1	07/20/2009	Australia	A/Australia/9/2009(H1N1)
PA	CY055578	H1N1	07/20/2009	Australia	A/Australia/13/2009(H1N1)
PA	CY055594	H1N1	07/20/2009	Australia	A/Australia/15/2009(H1N1)
PA	CY055570	H1N1	07/20/2009	Australia	A/Australia/12/2009(H1N1)
PA	CY055555	H1N1	07/20/2009	Australia	A/Australia/10/2009(H1N1)

PA	CY055562	H1N1	07/20/2009	Australia	A/Australia/11/2009(H1N1)
PA	HM241696	H1N1	07/20/2009	India	A/Che/NIV658/2009
PA	CY052380	H1N1	07/20/2009	USA	A/Texas/43200999/2009(H1N1)
PA	CY074007	H1N1	07/20/2009	Argentina	A/Argentina/HNRG15/2009(H1N1)
PA	CY071004	H1N1	07/20/2009	Guam	A/Guam/NHRC0030/2009(H1N1)
PA	CY075472	H1N1	07/20/2009	Chile	A/Chile/4438/2009(H1N1)
PA	KP637397	H1N1	07/20/2009	Thailand	A/Thailand/MR08208/2009
PA	KJ130155	H1N1	07/20/2009	New Zeala	A/New Zealand/3958/2009
PA	CY055586	H1N1	07/20/2009	Australia	A/Australia/14/2009(H1N1)
PA	GU138950	H1N1	07/21/2009	Japan	A/Mie/52/2009(H1N1)
PA	CY055602	H1N1	07/21/2009	Australia	A/Australia/16/2009(H1N1)
PA	HM569672	H1N1	07/21/2009	Argentina	A/Argentina/08AR/2009(H1N1)
PA	CY069263	H1N1	07/21/2009	Nicaragua	A/Managua/5364.01/2009(H1N1)
PA	CY069095	H1N1	07/21/2009	Guam	A/Guam/NHRC0026/2009(H1N1)
PA	CY068975	H1N1	07/21/2009	Guam	A/Guam/NHRC0011/2009(H1N1)
PA	CY068967	H1N1	07/21/2009	Guam	A/Guam/NHRC0010/2009(H1N1)
PA	CY069039	H1N1	07/21/2009	Guam	A/Guam/NHRC0019/2009(H1N1)
PA	CY069055	H1N1	07/21/2009	Guam	A/Guam/NHRC0021/2009(H1N1)
PA	GU433038	H1N1	07/21/2009	Russia	A/Novosibirsk/02/2009(H1N1)
PA	CY054976	H1N1	07/21/2009	USA	A/California/VRDL53/2009(H1N1)
PA	CY054992	H1N1	07/21/2009	USA	A/California/VRDL58/2009(H1N1)
PA	CY055286	H1N1	07/21/2009	Malaysia	A/Malaysia/5259/2009(H1N1)
PA	CY055294	H1N1	07/21/2009	Malaysia	A/Malaysia/5259/2009(H1N1)
PA	CY055618	H1N1	07/21/2009	Australia	A/Australia/21/2009(H1N1)
PA	CY055610	H1N1	07/21/2009	Australia	A/Australia/20/2009(H1N1)
PA	CY111832	H1N1	07/21/2009	Hong Kong	A/Hong Kong/H090-722-V20/2009(H1N
PA	CY083207	H1N1	07/21/2009	USA	A/New Jersey/WRAIR1504P/2009(H1N1
PA	CY092190	H1N1	07/21/2009	Australia	A/Australia/17/2009
PA	CY073433	H1N1	07/21/2009	USA	A/San Diego/WR1650P/2009(H1N1)
PA	CY072859	H1N1	07/21/2009	Nicaragua	A/Managua/3438.02/2009(H1N1)
PA	CY073441	H1N1	07/21/2009	USA	A/San Diego/WR1651P/2009(H1N1)
PA	KC620385	H1N1	07/21/2009	Germany	A/Giessen/6/2009
PA	CY055634	H1N1	07/21/2009	Australia	A/Australia/24/2009(H1N1)
PA	CY055626	H1N1	07/22/2009	Australia	A/Australia/23/2009(H1N1)
PA	KJ549777	H1N1	07/22/2009	Germany	A/Jena/5258/2009
PA	GU138955	H1N1	07/22/2009	Myanmar	A/Myanmar/JP133/2009(H1N1)
PA	GU138954	H1N1	07/22/2009	Myanmar	A/Myanmar/JP131/2009(H1N1)
PA	CY055642	H1N1	07/22/2009	Australia	A/Australia/25/2009(H1N1)
PA	CY055650	H1N1	07/22/2009	Australia	A/Australia/26/2009(H1N1)
PA	CY055658	H1N1	07/22/2009	Australia	A/Australia/27/2009(H1N1)
PA	CY068943	H1N1	07/22/2009	Guam	A/Guam/NHRC0008/2009(H1N1)
PA	CY069031	H1N1	07/22/2009	Guam	A/Guam/NHRC0018/2009(H1N1)
PA	CY083366	H1N1	07/22/2009	USA	A/South Carolina/WRAIR1660P/2009
PA	CY073646	H1N1	07/22/2009	Nicaragua	A/Managua/1973.02/2009(H1N1)
PA	CY073622	H1N1	07/22/2009	Nicaragua	A/Managua/4747.01/2009(H1N1)
PA	CY073654	H1N1	07/22/2009	Nicaragua	A/Managua/4590.03/2009(H1N1)
PA	CY074065	H1N1	07/22/2009	Argentina	A/Argentina/HNRG82/2009
PA	KX013004	H1N1	07/22/2009	Germany	A/Jena/MSV-VI5258/2009
PA	KP222617	H1N1	07/22/2009	Singapore	A/Singapore/384P/2009
PA	KC222635	H1N1	07/22/2009	Germany	A/Jena/VI5258/2009(H1N1)
PA	KJ690423	H1N1	07/22/2009	Uganda	A/Uganda/MUWRP-059/2009
PA	GQ496140	H1N1	07/22/2009	Russia	A/Vladivostok/IIV17/2009(H1N1)
PA	CY055681	H1N1	07/23/2009	Australia	A/Australia/31/2009(H1N1)
PA	CY055666	H1N1	07/23/2009	Australia	A/Australia/28/2009(H1N1)

PA	CY055673	H1N1	07/23/2009	Australia	A/Australia/29/2009(H1N1)
PA	CY065957	H1N1	07/23/2009	Japan	A/Japan/NHRC0001/2009(H1N1)
PA	CY065941	H1N1	07/23/2009	Guam	A/Guam/NHRC0002/2009(H1N1)
PA	CY065965	H1N1	07/23/2009	Japan	A/Japan/NHRC0002/2009(H1N1)
PA	CY068983	H1N1	07/23/2009	Guam	A/Guam/NHRC0012/2009(H1N1)
PA	CY068919	H1N1	07/23/2009	Japan	A/Japan/NHRC0003/2009(H1N1)
PA	CY073598	H1N1	07/23/2009	Nicaragua	A/Managua/5295.02/2009(H1N1)
PA	JX625497	H1N1	07/23/2009	United Ki	A/England/93760756/2009
PA	KT180875	H1N1	07/23/2009	Singapore	A/Singapore/DMS16/2009
PA	CY119359	H1N1	07/23/2009	Malaysia	A/Malaysia/2050837/2009
PA	CY055713	H1N1	07/24/2009	Australia	A/Australia/37/2009(H1N1)
PA	CY051660	H1N1	07/24/2009	USA	A/New York/4728/2009(H1N1)
PA	CY055729	H1N1	07/24/2009	Australia	A/Australia/40/2009(H1N1)
PA	CY055705	H1N1	07/24/2009	Australia	A/Australia/36/2009(H1N1)
PA	CY055721	H1N1	07/24/2009	Australia	A/Australia/39/2009(H1N1)
PA	CY055697	H1N1	07/24/2009	Australia	A/Australia/35/2009(H1N1)
PA	CY062528	H1N1	07/24/2009	Mexico	A/Mexico city/CIA7/2009(H1N1)
PA	CY069287	H1N1	07/24/2009	Nicaragua	A/Managua/3467.01/2009(H1N1)
PA	CY069295	H1N1	07/24/2009	Nicaragua	A/Managua/4951.02/2009(H1N1)
PA	CY072579	H1N1	07/24/2009	Nicaragua	A/Managua/3231.02/2009(H1N1)
PA	CY051828	H1N1	07/24/2009	USA	A/Texas/43242018/2009(H1N1)
PA	CY111848	H1N1	07/24/2009	Hong Kong	A/Hong Kong/H090-742-V10/2009(H1N
PA	CY120282	H1N1	07/24/2009	Hong Kong	A/Hong Kong/H090-742-V10/2009
PA	CY111840	H1N1	07/24/2009	Hong Kong	A/Hong Kong/H090-738-V10/2009
PA	CY120274	H1N1	07/24/2009	Hong Kong	A/Hong Kong/H090-738-V10/2009
PA	CY092198	H1N1	07/24/2009	Australia	A/Australia/38/2009
PA	KP637661	H1N1	07/24/2009	Thailand	A/Thailand/SP08294/2009
PA	KP637453	H1N1	07/24/2009	Thailand	A/Thailand/PY08016/2009
PA	CY128088	H1N1	07/24/2009	Viet Nam	A/Viet Nam/811/2009
PA	CY128080	H1N1	07/24/2009	Viet Nam	A/Viet Nam/817/2009
PA	GQ496151	H1N1	07/24/2009	Russia	A/Vladivostok/IIV18/2009(H1N1)
PA	CY055737	H1N1	07/25/2009	Australia	A/Australia/42/2009(H1N1)
PA	CY069271	H1N1	07/25/2009	Nicaragua	A/Managua/1264.03/2009(H1N1)
PA	KC782199	H1N1	07/25/2009	USA	A/Utah/20/2009
PA	CY088694	H1N1	07/25/2009	India	A/Pune/NIV759/2009
PA	CY128096	H1N1	07/25/2009	Viet Nam	A/Viet Nam/818/2009
PA	CY051668	H1N1	07/25/2009	USA	A/New York/4735/2009(H1N1)
PA	CY055745	H1N1	07/26/2009	Australia	A/Australia/43/2009(H1N1)
PA	KT180879	H1N1	07/26/2009	Singapore	A/Singapore/DMS17/2009
PA	CY128112	H1N1	07/26/2009	Viet Nam	A/Viet Nam/823/2009
PA	CY055753	H1N1	07/26/2009	Australia	A/Australia/44/2009(H1N1)
PA	CY055761	H1N1	07/27/2009	Australia	A/Australia/45/2009(H1N1)
PA	CY055777	H1N1	07/27/2009	Australia	A/Australia/48/2009(H1N1)
PA	CY065933	H1N1	07/27/2009	Guam	A/Guam/NHRC0001/2009(H1N1)
PA	CY072627	H1N1	07/27/2009	Nicaragua	A/Managua/5339.04/2009(H1N1)
PA	CY052372	H1N1	07/27/2009	USA	A/Texas/43272683/2009(H1N1)
PA	CY074017	H1N1	07/27/2009	Argentina	A/Argentina/HNRG23/2009(H1N1)
PA	CY055000	H1N1	07/27/2009	USA	A/California/VRDL61/2009(H1N1)
PA	CY120089	H1N1	07/27/2009	Hong Kong	A/Hong Kong/H090-745-V10/2009
PA	CY111864	H1N1	07/27/2009	Hong Kong	A/Hong Kong/H090-745-V10/2009(H1N
PA	CY083327	H1N1	07/27/2009	USA	A/Cape May/WRAIR1653P/2009
PA	JN187241	H1N1	07/27/2009	Taiwan	A/Taiwan/2826/2009
PA	CY072731	H1N1	07/27/2009	Nicaragua	A/Managua/4407.02/2009(H1N1)
PA	CY073534	H1N1	07/27/2009	Nicaragua	A/Managua/466.01/2009(H1N1)

PA	CY073662	H1N1	07/27/2009	Nicaragua	A/Managua/3225.03/2009(H1N1)
PA	CY128104	H1N1	07/27/2009	Viet Nam	A/Viet Nam/835/2009
PA	CY128128	H1N1	07/27/2009	Viet Nam	A/Viet Nam/841/2009
PA	KP222618	H1N1	07/27/2009	Singapore	A/Singapore/385K/2009
PA	KP222619	H1N1	07/27/2009	Singapore	A/Singapore/386T/2009
PA	JQ173147	H1N1	07/27/2009	Finland	A/Helsinki/P14/2009
PA	CY122818	H1N1	07/27/2009	Singapore	A/Singapore/GP2224/2009
PA	CY122810	H1N1	07/27/2009	Singapore	A/Singapore/GP2223/2009
PA	CY055769	H1N1	07/27/2009	Australia	A/Australia/47/2009(H1N1)
PA	CY055785	H1N1	07/28/2009	Australia	A/Australia/51/2009(H1N1)
PA	CY051676	H1N1	07/28/2009	USA	A/New York/4738/2009(H1N1)
PA	CY120298	H1N1	07/28/2009	Hong Kong	A/Hong Kong/H090-746-V10/2009
PA	CY111880	H1N1	07/28/2009	Hong Kong	A/Hong Kong/H090-746-V10/2009(H1N
PA	CY111856	H1N1	07/28/2009	Hong Kong	A/Hong Kong/H090-742-V33/2009
PA	CY120290	H1N1	07/28/2009	Hong Kong	A/Hong Kong/H090-742-V33/2009
PA	CY073638	H1N1	07/28/2009	Nicaragua	A/Managua/4960.01/2009(H1N1)
PA	CY128120	H1N1	07/28/2009	Viet Nam	A/Viet Nam/850/2009
PA	KP222621	H1N1	07/28/2009	Singapore	A/Singapore/392N/2009
PA	KP222620	H1N1	07/28/2009	Singapore	A/Singapore/390T/2009
PA	CY119535	H1N1	07/28/2009	Malaysia	A/Malaysia/2072040/2009
PA	CY122842	H1N1	07/28/2009	Singapore	A/Singapore/GP2252/2009
PA	CY122826	H1N1	07/28/2009	Singapore	A/Singapore/GP2238/2009
PA	CY083239	H1N1	07/28/2009	USA	A/Virginia/WRAIR1511P/2009
PA	CY083223	H1N1	07/29/2009	USA	A/California/WRAIR1507P/2009
PA	CY052287	H1N1	07/29/2009	USA	A/Texas/43292238/2009(H1N1)
PA	CY055793	H1N1	07/29/2009	Australia	A/Australia/53/2009(H1N1)
PA	CY111896	H1N1	07/29/2009	Hong Kong	A/Hong Kong/H090-751-V10/2009
PA	CY120306	H1N1	07/29/2009	Hong Kong	A/Hong Kong/H090-751-V10/2009
PA	CY073558	H1N1	07/29/2009	Nicaragua	A/Managua/2978.04/2009(H1N1)
PA	KP637533	H1N1	07/29/2009	Thailand	A/Thailand/SN08294/2009
PA	CY122794	H1N1	07/29/2009	Singapore	A/Singapore/GP2215/2009
PA	CY128456	H1N1	07/29/2009	Viet Nam	A/Viet Nam/001-1880/2009
PA	KP222623	H1N1	07/29/2009	Singapore	A/Singapore/395T/2009
PA	KP222622	H1N1	07/29/2009	Singapore	A/Singapore/394T/2009
PA	KT180880	H1N1	07/29/2009	Singapore	A/Singapore/DMS18/2009
PA	CY065949	H1N1	07/29/2009	Guam	A/Guam/NHRC0003/2009(H1N1)
PA	KP637325	H1N1	07/30/2009	Thailand	A/Thailand/KS08277/2009
PA	KY925887	H1N1	07/30/2009	Brazil	A/Tramandai/LACENRS-324/2009
PA	CY055809	H1N1	07/30/2009	Australia	A/Australia/56/2009(H1N1)
PA	CY051684	H1N1	07/30/2009	USA	A/New York/4747/2009(H1N1)
PA	CY072635	H1N1	07/30/2009	Nicaragua	A/Managua/1836.02/2009(H1N1)
PA	CY111944	H1N1	07/30/2009	Hong Kong	A/Hong Kong/H090-757-V10/2009
PA	CY120354	H1N1	07/30/2009	Hong Kong	A/Hong Kong/H090-757-V10/2009
PA	CY111888	H1N1	07/30/2009	Hong Kong	A/Hong Kong/H090-746-V20/2009
PA	CY120362	H1N1	07/30/2009	Hong Kong	A/Hong Kong/H090-758-V10/2009
PA	CY111952	H1N1	07/30/2009	Hong Kong	A/Hong Kong/H090-758-V10/2009
PA	CY120370	H1N1	07/30/2009	Hong Kong	A/Hong Kong/H090-759-V10/2009
PA	CY111960	H1N1	07/30/2009	Hong Kong	A/Hong Kong/H090-759-V10/2009
PA	CY122886	H1N1	07/30/2009	Singapore	A/Singapore/GP2317/2009
PA	KM013764	H1N1	07/30/2009	Thailand	A/Bangkok/SIMI507/2009
PA	KM013758	H1N1	07/30/2009	Thailand	A/Bangkok/SIMI501/2009
PA	CY122878	H1N1	07/30/2009	Singapore	A/Singapore/GP2315/2009
PA	KY925842	H1N1	07/30/2009	Brazil	A/Porto Alegre/LACENRS-1771/2009
PA	CY120394	H1N1	07/31/2009	Hong Kong	A/Hong Kong/H090-765-V10/2009

PA	CY049626	H1N1	07/31/2009	Singapore	A/Singapore/GP2316/2009(H1N1)
PA	CY120314	H1N1	07/31/2009	Hong Kong	A/Hong Kong/H090-751-V20/2009
PA	CY111872	H1N1	07/31/2009	Hong Kong	A/Hong Kong/H090-745-V24/2009
PA	CY068935	H1N1	07/31/2009	Guam	A/Guam/NHRC0005/2009(H1N1)
PA	CY111984	H1N1	07/31/2009	Hong Kong	A/Hong Kong/H090-765-V10/2009
PA	CY083215	H1N1	07/31/2009	USA	A/New Jersey/WRAIR1506P/2009(H1N1
PA	CY111912	H1N1	07/31/2009	Hong Kong	A/Hong Kong/H090-751-V23/2009
PA	CY071569	H1N1	07/31/2009	USA	A/South Carolina/WR1325P/2009(H1N
PA	CY071577	H1N1	07/31/2009	USA	A/South Carolina/WR1326P/2009(H1N
PA	CY111968	H1N1	07/31/2009	Hong Kong	A/Hong Kong/H090-761-V10/2009
PA	CY070988	H1N1	07/31/2009	Guam	A/Guam/NHRC0007/2009(H1N1)
PA	CY120744	H1N1	07/31/2009	Brazil	A/Brazil/AVS07/2009
PA	CY119367	H1N1	07/31/2009	Malaysia	A/Malaysia/2068631/2009
PA	CY120322	H1N1	07/31/2009	Hong Kong	A/Hong Kong/H090-751-V23/2009
PA	CY120330	H1N1	07/31/2009	Hong Kong	A/Hong Kong/H090-751-V24/2009
PA	CY111920	H1N1	07/31/2009	Hong Kong	A/Hong Kong/H090-751-V24/2009(H1N
PA	CY120386	H1N1	07/31/2009	Hong Kong	A/Hong Kong/H090-762-V10/2009
PA	KP222626	H1N1	07/31/2009	Singapore	A/Singapore/410W/2009
PA	KP222628	H1N1	07/31/2009	Singapore	A/Singapore/416C/2009
PA	KP222627	H1N1	07/31/2009	Singapore	A/Singapore/413Y/2009
PA	KP222630	H1N1	07/31/2009	Singapore	A/Singapore/419B/2009
PA	KP222629	H1N1	07/31/2009	Singapore	A/Singapore/418W/2009
PA	KP222624	H1N1	07/31/2009	Singapore	A/Singapore/405S/2009
PA	KP222625	H1N1	07/31/2009	Singapore	A/Singapore/406K/2009
PA	CY111976	H1N1	07/31/2009	Hong Kong	A/Hong Kong/H090-762-V10/2009
PA	KY926364	H1N1	07/31/2009	Brazil	A/Estrela/LACENRS-313/2009
PA	KY925894	H1N1	07/31/2009	Brazil	A/Porto Alegre/LACENRS-1786/2009
PA	CY120378	H1N1	07/31/2009	Hong Kong	A/Hong Kong/H090-761-V10/2009
PA	KY926042	H1N1	07/31/2009	Brazil	A/Igrejinha/LACENRS-1803/2009
PA	CY111904	H1N1	07/31/2009	Hong Kong	A/Hong Kong/H090-751-V20/2009(H1N
PA	GQ866927	H1N1	08/2009	Thailand	A/Thailand/CU-B938/2009(H1N1)
PA	GQ866926	H1N1	08/2009	Thailand	A/Thailand/CU-H340/2009(H1N1)
PA	CY050338	H1N1	08/2009	Singapore	A/Singapore/9061364/2009(H1N1)
PA	CY050346	H1N1	08/2009	Singapore	A/Singapore/9061365/2009(H1N1)
PA	CY050354	H1N1	08/2009	Singapore	A/Singapore/9061366/2009(H1N1)
PA	CY055825	H1N1	08/01/2009	Australia	A/Australia/58/2009(H1N1)
PA	CY071561	H1N1	08/01/2009	USA	A/South Carolina/WR1324P/2009(H1N
PA	CY055817	H1N1	08/01/2009	Australia	A/Australia/57/2009(H1N1)
PA	KC780434	H1N1	08/01/2009	USA	A/Texas/71/2009
PA	KT180881	H1N1	08/01/2009	Singapore	A/Singapore/DMS19/2009
PA	CY118204	H1N1	08/02/2009	Malaysia	A/Malaysia/2076212/2009
PA	CY119375	H1N1	08/02/2009	Malaysia	A/Malaysia/2076204/2009
PA	CY083167	H1N1	08/02/2009	USA	A/New Jersey/WRAIR1499P/2009(H1N1
PA	CY055841	H1N1	08/02/2009	Australia	A/Australia/60/2009(H1N1)
PA	CY055833	H1N1	08/02/2009	Australia	A/Australia/59/2009(H1N1)
PA	CY120338	H1N1	08/02/2009	Hong Kong	A/Hong Kong/H090-751-V31/2009
PA	CY111928	H1N1	08/02/2009	Hong Kong	A/Hong Kong/H090-751-V31/2009(H1N
PA	CY120346	H1N1	08/02/2009	Hong Kong	A/Hong Kong/H090-751-V34/2009
PA	CY111936	H1N1	08/02/2009	Hong Kong	A/Hong Kong/H090-751-V34/2009
PA	CY112000	H1N1	08/03/2009	Hong Kong	A/Hong Kong/H090-771-V10/2009
PA	CY120410	H1N1	08/03/2009	Hong Kong	A/Hong Kong/H090-771-V10/2009
PA	CY072683	H1N1	08/03/2009	Nicaragua	A/Managua/209.01/2009(H1N1)
PA	CY072563	H1N1	08/03/2009	Nicaragua	A/Managua/451.01/2009(H1N1)
PA	CY055857	H1N1	08/03/2009	Australia	A/Australia/63/2009(H1N1)

PA	CY055873	H1N1	08/03/2009	Australia	A/Australia/65/2009(H1N1)
PA	CY055865	H1N1	08/03/2009	Australia	A/Australia/64/2009(H1N1)
PA	CY055849	H1N1	08/03/2009	Australia	A/Australia/61/2009(H1N1)
PA	KP222634	H1N1	08/03/2009	Singapore	A/Singapore/440F/2009
PA	KP222632	H1N1	08/03/2009	Singapore	A/Singapore/426N/2009
PA	KP222631	H1N1	08/03/2009	Singapore	A/Singapore/420T/2009
PA	KP222633	H1N1	08/03/2009	Singapore	A/Singapore/437Y/2009
PA	KP222635	H1N1	08/03/2009	Singapore	A/Singapore/441T/2009
PA	CY119391	H1N1	08/03/2009	Malaysia	A/Malaysia/2077462/2009
PA	CY119383	H1N1	08/03/2009	Malaysia	A/Malaysia/2076039/2009
PA	CY122958	H1N1	08/03/2009	Singapore	A/Singapore/GP2714/2009
PA	JX625474	H1N1	08/03/2009	United Ki	A/England/646/2009
PA	CY106981	H1N1	08/03/2009	Hong Kong	A/Hong Kong/H090-770-V10/2009
PA	CY120426	H1N1	08/04/2009	Hong Kong	A/Hong Kong/H090-774-V10/2009
PA	CY112024	H1N1	08/04/2009	Hong Kong	A/Hong Kong/H090-774-V10/2009
PA	CY068951	H1N1	08/04/2009	Japan	A/Japan/NHRC0004/2009(H1N1)
PA	CY070980	H1N1	08/04/2009	Guam	A/Guam/NHRC0006/2009(H1N1)
PA	CY055881	H1N1	08/04/2009	Australia	A/Australia/66/2009(H1N1)
PA	KT180882	H1N1	08/04/2009	Singapore	A/Singapore/DMS20/2009
PA	CY073614	H1N1	08/04/2009	Nicaragua	A/Managua/1256.04/2009(H1N1)
PA	KP222636	H1N1	08/04/2009	Singapore	A/Singapore/443M/2009
PA	KP222637	H1N1	08/04/2009	Singapore	A/Singapore/444W/2009
PA	CY119527	H1N1	08/04/2009	Malaysia	A/Malaysia/2076756/2009
PA	CY122902	H1N1	08/04/2009	Singapore	A/Singapore/GP2649/2009
PA	CY123485	H1N1	08/04/2009	Singapore	A/Singapore/ON1666/2009
PA	CY073606	H1N1	08/04/2009	Nicaragua	A/Managua/4451.02/2009(H1N1)
PA	CY122894	H1N1	08/04/2009	Singapore	A/Singapore/GP2640/2009
PA	CY120434	H1N1	08/05/2009	Hong Kong	A/Hong Kong/H090-779-V10/2009
PA	CY112032	H1N1	08/05/2009	Hong Kong	A/Hong Kong/H090-779-V10/2009
PA	CY120442	H1N1	08/05/2009	Hong Kong	A/Hong Kong/H090-779-V12/2009
PA	CY112040	H1N1	08/05/2009	Hong Kong	A/Hong Kong/H090-779-V12/2009
PA	CY112008	H1N1	08/05/2009	Hong Kong	A/Hong Kong/H090-771-V20/2009
PA	CY071497	H1N1	08/05/2009	USA	A/California/WR1316P/2009(H1N1)
PA	CY071481	H1N1	08/05/2009	USA	A/California/WR1314P/2009(H1N1)
PA	CY071489	H1N1	08/05/2009	USA	A/California/WR1315P/2009(H1N1)
PA	CY072603	H1N1	08/05/2009	Nicaragua	A/Managua/1077.04/2009(H1N1)
PA	CY049634	H1N1	08/05/2009	Singapore	A/Singapore/GP2641/2009(H1N1)
PA	CY055040	H1N1	08/05/2009	USA	A/California/VRDL69/2009(H1N1)
PA	CY055889	H1N1	08/05/2009	Australia	A/Australia/69/2009(H1N1)
PA	KJ690502	H1N1	08/05/2009	Uganda	A/Uganda/MUWRP-067/2009
PA	KJ690407	H1N1	08/05/2009	Uganda	A/Uganda/MUWRP-066/2009
PA	KY926136	H1N1	08/05/2009	Brazil	A/Teutonia/LACENRS-711/2009
PA	KY926197	H1N1	08/05/2009	Brazil	A/Nonoai/LACENRS-714/2009
PA	CY122934	H1N1	08/05/2009	Singapore	A/Singapore/GP2693/2009
PA	CY120418	H1N1	08/05/2009	Hong Kong	A/Hong Kong/H090-772-V10/2009
PA	CY112016	H1N1	08/05/2009	Hong Kong	A/Hong Kong/H090-772-V10/2009
PA	CY119399	H1N1	08/05/2009	Malaysia	A/Malaysia/2082543/2009
PA	CY122926	H1N1	08/05/2009	Singapore	A/Singapore/GP2680/2009
PA	CY122918	H1N1	08/05/2009	Singapore	A/Singapore/GP2672/2009
PA	CY122942	H1N1	08/05/2009	Singapore	A/Singapore/GP2697/2009
PA	CY073574	H1N1	08/05/2009	Nicaragua	A/Managua/5230.01/2009(H1N1)
PA	JN596864	H1N1	08/06/2009	Russia	A/Orenburg/01/2009(H1N1)
PA	CY111992	H1N1	08/06/2009	Hong Kong	A/Hong Kong/H090-770-V22/2009
PA	CY068927	H1N1	08/06/2009	Guam	A/Guam/NHRC0004/2009(H1N1)

PA	CY049674	H1N1	08/06/2009	Singapore	A/Singapore/GP2695/2009(H1N1)
PA	CY049698	H1N1	08/06/2009	Singapore	A/Singapore/GP2715/2009(H1N1)
PA	CY049690	H1N1	08/06/2009	Singapore	A/Singapore/GP2711/2009(H1N1)
PA	CY049658	H1N1	08/06/2009	Singapore	A/Singapore/GP2686/2009(H1N1)
PA	CY049682	H1N1	08/06/2009	Singapore	A/Singapore/GP2703/2009(H1N1)
PA	CY049666	H1N1	08/06/2009	Singapore	A/Singapore/GP2687/2009(H1N1)
PA	CY051692	H1N1	08/06/2009	USA	A/New York/4761/2009(H1N1)
PA	CY055897	H1N1	08/06/2009	Australia	A/Australia/70/2009(H1N1)
PA	JX625482	H1N1	08/06/2009	United Ki	A/England/93320016/2009
PA	KP222638	H1N1	08/06/2009	Singapore	A/Singapore/448D/2009
PA	CY122950	H1N1	08/06/2009	Singapore	A/Singapore/GP2709/2009
PA	CY122910	H1N1	08/06/2009	Singapore	A/Singapore/GP2658/2009
PA	CY120402	H1N1	08/06/2009	Hong Kong	A/Hong Kong/H090-770-V22/2009
PA	CY071537	H1N1	08/07/2009	USA	A/California/WR1321P/2009(H1N1)
PA	CY071529	H1N1	08/07/2009	USA	A/California/WR1320P/2009(H1N1)
PA	CY072619	H1N1	08/07/2009	Nicaragua	A/Managua/2604.02/2009(H1N1)
PA	CY073526	H1N1	08/07/2009	Nicaragua	A/Managua/5399.04/2009(H1N1)
PA	KT180883	H1N1	08/07/2009	Singapore	A/Singapore/DMS21/2009
PA	CY120466	H1N1	08/07/2009	Hong Kong	A/Hong Kong/H090-785-V10/2009
PA	CY112072	H1N1	08/07/2009	Hong Kong	A/Hong Kong/H090-785-V10/2009
PA	CY119407	H1N1	08/07/2009	Malaysia	A/Malaysia/2086505/2009
PA	KJ690462	H1N1	08/07/2009	Uganda	A/Uganda/MUWRP-069/2009
PA	CY112080	H1N1	08/08/2009	Hong Kong	A/Hong Kong/H090-787-V10/2009
PA	CY120474	H1N1	08/08/2009	Hong Kong	A/Hong Kong/H090-787-V10/2009
PA	CY112056	H1N1	08/08/2009	Hong Kong	A/Hong Kong/H090-779-V21/2009
PA	CY120450	H1N1	08/08/2009	Hong Kong	A/Hong Kong/H090-779-V21/2009
PA	CY120097	H1N1	08/08/2009	Hong Kong	A/Hong Kong/H090-779-V20/2009
PA	CY087021	H1N1	08/08/2009	USA	A/New York/4764/2009(H1N1)
PA	CY112064	H1N1	08/08/2009	Hong Kong	A/Hong Kong/H090-779-V22/2009
PA	CY120458	H1N1	08/08/2009	Hong Kong	A/Hong Kong/H090-779-V22/2009
PA	CY071465	H1N1	08/08/2009	USA	A/California/WR1312P/2009(H1N1)
PA	CY073590	H1N1	08/08/2009	Nicaragua	A/Managua/4064.01/2009(H1N1)
PA	CY073630	H1N1	08/08/2009	Nicaragua	A/Managua/5595.03/2009(H1N1)
PA	CY112048	H1N1	08/08/2009	Hong Kong	A/Hong Kong/H090-779-V20/2009
PA	CY060541	H1N1	08/09/2009	Canada	A/Ontario/25389/2009(H1N1)
PA	KC781741	H1N1	08/09/2009	USA	A/Wyoming/08/2009
PA	CY119423	H1N1	08/09/2009	Malaysia	A/Malaysia/2089888/2009
PA	CY119415	H1N1	08/09/2009	Malaysia	A/Malaysia/2089841/2009
PA	KC781731	H1N1	08/09/2009	USA	A/Oregon/30/2009
PA	CY055905	H1N1	08/10/2009	Australia	A/Australia/72/2009(H1N1)
PA	CY055008	H1N1	08/10/2009	USA	A/California/VRDL64/2009(H1N1)
PA	HM014329	H1N1	08/10/2009	China	A/Guangzhou/GIRD07/2009
PA	KT180884	H1N1	08/10/2009	Singapore	A/Singapore/DMS22/2009
PA	KY935481	H1N1	08/10/2009	Brazil	A/Rio Grande/LACENRS-1406/2009
PA	CY119447	H1N1	08/10/2009	Malaysia	A/Malaysia/2098053/2009
PA	CY119431	H1N1	08/10/2009	Malaysia	A/Malaysia/2098001/2009
PA	CY119439	H1N1	08/10/2009	Malaysia	A/Malaysia/2098044/2009
PA	CY073550	H1N1	08/10/2009	Nicaragua	A/Managua/3083.01/2009(H1N1)
PA	CY072691	H1N1	08/10/2009	Nicaragua	A/Managua/554.01/2009(H1N1)
PA	CY071505	H1N1	08/10/2009	USA	A/California/WR1317P/2009(H1N1)
PA	CY053784	H1N1	08/10/2009	Norway	A/Norway/2924/2009(H1N1)
PA	CY055016	H1N1	08/10/2009	USA	A/California/VRDL65/2009(H1N1)
PA	JN596871	H1N1	08/10/2009	Russia	A/Kurgan/01/2009
PA	CY055499	H1N1	08/10/2009	USA	A/California/VRDL74/2009(H1N1)

PA	CY123505	H1N1	08/11/2009	Singapore	A/Singapore/ON1760/2009
PA	JQ431340	H1N1	08/11/2009	France	A/Reunion/2336-4-M1E/2009
PA	CY115867	H1N1	08/11/2009	Netherlar	A/Netherlands/947b/2009
PA	CY119455	H1N1	08/11/2009	Malaysia	A/Malaysia/2097717/2009
PA	CY118225	H1N1	08/11/2009	Malaysia	A/Malaysia/2098038/2009
PA	CY123493	H1N1	08/11/2009	Singapore	A/Singapore/ON1747/2009
PA	CY073566	H1N1	08/11/2009	Nicaragua	A/Managua/5119.06/2009(H1N1)
PA	CY073542	H1N1	08/11/2009	Nicaragua	A/Managua/4012.01/2009(H1N1)
PA	CY060517	H1N1	08/11/2009	Canada	A/Ontario/222656/2009(H1N1)
PA	CY069279	H1N1	08/11/2009	Nicaragua	A/Managua/5212.04/2009(H1N1)
PA	CY072707	H1N1	08/11/2009	Nicaragua	A/Managua/5036.01/2009(H1N1)
PA	CY072651	H1N1	08/11/2009	Nicaragua	A/Managua/2275.01/2009(H1N1)
PA	CY072667	H1N1	08/11/2009	Nicaragua	A/Managua/2339.03/2009(H1N1)
PA	CY072595	H1N1	08/11/2009	Nicaragua	A/Managua/1356.03/2009(H1N1)
PA	CY055032	H1N1	08/11/2009	USA	A/California/VRDL68/2009(H1N1)
PA	CY055913	H1N1	08/12/2009	Australia	A/Australia/73/2009(H1N1)
PA	JN596868	H1N1	08/12/2009	Russia	A/Omsk/01/2009
PA	CY072587	H1N1	08/12/2009	Nicaragua	A/Managua/4186.02/2009(H1N1)
PA	CY072723	H1N1	08/12/2009	Nicaragua	A/Managua/3359.01/2009(H1N1)
PA	CY083390	H1N1	08/12/2009	USA	A/San Diego/WRAIR1663P/2009
PA	CY083231	H1N1	08/12/2009	Guam	A/Guam/WRAIR1510P/2009
PA	CY120720	H1N1	08/12/2009	Brazil	A/Brazil/AVS03/2009
PA	CY112088	H1N1	08/12/2009	Hong Kong	A/Hong Kong/H090-793-V10/2009
PA	CY120482	H1N1	08/12/2009	Hong Kong	A/Hong Kong/H090-793-V10/2009
PA	CY128136	H1N1	08/12/2009	Viet Nam	A/Viet Nam/13032001/2009
PA	CY128144	H1N1	08/12/2009	Viet Nam	A/Viet Nam/13032002/2009
PA	KY925745	H1N1	08/12/2009	Brazil	A/Porto Alegre/LACENRS-1598/2009
PA	KY925537	H1N1	08/12/2009	Brazil	A/Alegrete/LACENRS-1453/2009
PA	JQ431348	H1N1	08/13/2009	France	A/Reunion/1722-7-M1E/2009
PA	CY088673	H1N1	08/13/2009	India	A/Delhi/NIV3610/2009
PA	KF612078	H1N1	08/13/2009	Bolivia	A/Santa Cruz/87212/2009
PA	KT180886	H1N1	08/13/2009	Singapore	A/Singapore/DMS23/2009
PA	CY073582	H1N1	08/13/2009	Nicaragua	A/Managua/921.02/2009(H1N1)
PA	CY060733	H1N1	08/13/2009	Canada	A/Ontario/35273/2009(H1N1)
PA	KY925240	H1N1	08/13/2009	Brazil	A/Santo Angelo/LACENRS-1537/2009
PA	KY925458	H1N1	08/13/2009	Brazil	A/Vacaria/LACENRS-1720/2009
PA	KY926073	H1N1	08/13/2009	Brazil	A/Venancio Aires/LACENRS-1539/200
PA	CY120728	H1N1	08/13/2009	Brazil	A/Brazil/AVS04/2009
PA	CY072995	H1N1	08/13/2009	Nicaragua	A/Managua/527.04/2009(H1N1)
PA	JQ431349	H1N1	08/13/2009	France	A/Reunion/1722-8-M1E/2009
PA	CY072643	H1N1	08/13/2009	Nicaragua	A/Managua/3642.04/2009(H1N1)
PA	KY925540	H1N1	08/14/2009	Brazil	A/Pelotas/LACENRS-1658/2009
PA	CY112096	H1N1	08/14/2009	Hong Kong	A/Hong Kong/H090-793-V20/2009
PA	KY925205	H1N1	08/14/2009	Brazil	A/Sao Gabriel/LACENRS-1626/2009
PA	CY120490	H1N1	08/14/2009	Hong Kong	A/Hong Kong/H090-793-V20/2009
PA	KY925294	H1N1	08/14/2009	Brazil	A/Triunfo/LACENRS-1611/2009
PA	CY122966	H1N1	08/14/2009	Singapore	A/Singapore/GP2982/2009
PA	KP637829	H1N1	08/14/2009	Thailand	A/Thailand/IY07083/2009
PA	CY051700	H1N1	08/14/2009	USA	A/New York/4777/2009(H1N1)
PA	JQ431361	H1N1	08/14/2009	France	A/Reunion/1658-4-M1E/2009
PA	CY055270	H1N1	08/14/2009	Malaysia	A/Malaysia/10226/2009(H1N1)
PA	KY925446	H1N1	08/15/2009	Brazil	A/Santo Antonio da Patrulha/LACEN
PA	CY058609	H1N1	08/15/2009	Nicaragua	A/Managua/5747.02/2009(H1N1)
PA	HM849037	H1N1	08/15/2009	Japan	A/Kurume/K0910/2009(H1N1)

PA	CY072715	H1N1	08/15/2009	Nicaragua	A/Managua/5307.01/2009(H1N1)
PA	CY052852	H1N1	08/15/2009	USA	A/Texas/44152535/2009(H1N1)
PA	CY052876	H1N1	08/15/2009	USA	A/Texas/44151841/2009(H1N1)
PA	CY058241	H1N1	08/15/2009	Nicaragua	A/Managua/473.02/2009(H1N1)
PA	CY058249	H1N1	08/15/2009	Nicaragua	A/Managua/3275.01/2009(H1N1)
PA	CY072675	H1N1	08/16/2009	Nicaragua	A/Managua/1054.02/2009(H1N1)
PA	CY122974	H1N1	08/16/2009	Singapore	A/Singapore/GP2987/2009
PA	CY072659	H1N1	08/16/2009	Nicaragua	A/Managua/4570.01/2009(H1N1)
PA	KT180887	H1N1	08/16/2009	Singapore	A/Singapore/DMS24/2009
PA	CY055801	H1N1	08/16/2009	Australia	A/Australia/54/2009(H1N1)
PA	CY088687	H1N1	08/16/2009	India	A/Mum/NIV5442/2009
PA	CY122986	H1N1	08/16/2009	Singapore	A/Singapore/GP3012/2009
PA	KP222640	H1N1	08/17/2009	Singapore	A/Singapore/464L/2009
PA	KY925260	H1N1	08/17/2009	Brazil	A/Passo Fundo/LACENRS-1972/2009
PA	KY925354	H1N1	08/17/2009	Brazil	A/Santa Cruz do Sul/LACENRS-2013/
PA	CY123002	H1N1	08/17/2009	Singapore	A/Singapore/GP3026/2009
PA	KP222639	H1N1	08/17/2009	Singapore	A/Singapore/463B/2009
PA	JQ431343	H1N1	08/17/2009	France	A/Reunion/2245-3-M1E/2009
PA	CY122994	H1N1	08/17/2009	Singapore	A/Singapore/GP3021/2009
PA	CY072611	H1N1	08/17/2009	Nicaragua	A/Managua/2155.01/2009(H1N1)
PA	CY060509	H1N1	08/17/2009	Canada	A/Ontario/10296/2009(H1N1)
PA	CY058569	H1N1	08/17/2009	Nicaragua	A/Managua/4218.01/2009(H1N1)
PA	CY123018	H1N1	08/17/2009	Singapore	A/Singapore/GP3044/2009
PA	CY123129	H1N1	08/17/2009	Singapore	A/Singapore/GP3278/2009
PA	CY123026	H1N1	08/18/2009	Singapore	A/Singapore/GP3069/2009
PA	CY123034	H1N1	08/18/2009	Singapore	A/Singapore/GP3074/2009
PA	CY123010	H1N1	08/18/2009	Singapore	A/Singapore/GP3040/2009
PA	KP222641	H1N1	08/18/2009	Singapore	A/Singapore/468H/2009
PA	JQ431337	H1N1	08/18/2009	France	A/Reunion/1632-5-M1E/2009
PA	CY123050	H1N1	08/18/2009	Singapore	A/Singapore/GP3093/2009
PA	CY058265	H1N1	08/18/2009	Nicaragua	A/Managua/164.01/2009(H1N1)
PA	CY058617	H1N1	08/18/2009	Nicaragua	A/Managua/2323.02/2009(H1N1)
PA	CY058257	H1N1	08/18/2009	Nicaragua	A/Managua/3244.01/2009(H1N1)
PA	CY055024	H1N1	08/18/2009	USA	A/California/VRDL67/2009(H1N1)
PA	CY051708	H1N1	08/18/2009	USA	A/New York/4780/2009(H1N1)
PA	CY063753	H1N1	08/18/2009	Singapore	A/Singapore/GP3084/2009(H1N1)
PA	CY063579	H1N1	08/18/2009	USA	A/Boston/154/2009(H1N1)
PA	CY058601	H1N1	08/18/2009	Nicaragua	A/Managua/3435.01/2009(H1N1)
PA	CY058577	H1N1	08/18/2009	Nicaragua	A/Managua/4757.02/2009(H1N1)
PA	CY058561	H1N1	08/18/2009	Nicaragua	A/Managua/2093.01/2009(H1N1)
PA	CY058585	H1N1	08/18/2009	Nicaragua	A/Managua/989.01/2009(H1N1)
PA	JX625490	H1N1	08/18/2009	United Ki	A/England/93520038/2009
PA	KY926245	H1N1	08/19/2009	Brazil	A/Tenente Portela/LACENRS-2073/20
PA	CY123531	H1N1	08/19/2009	Singapore	A/Singapore/ON1808/2009
PA	JQ431353	H1N1	08/19/2009	France	A/Reunion/0148-4-M1E/2009
PA	CY072851	H1N1	08/19/2009	Nicaragua	A/Managua/4702.04/2009(H1N1)
PA	JQ431355	H1N1	08/19/2009	France	A/Reunion/0148-6-M1E/2009
PA	CY123523	H1N1	08/19/2009	Singapore	A/Singapore/ON1802/2009
PA	CY123058	H1N1	08/19/2009	Singapore	A/Singapore/GP3104/2009
PA	KT180888	H1N1	08/19/2009	Singapore	A/Singapore/DMS25/2009
PA	CY123042	H1N1	08/19/2009	Singapore	A/Singapore/GP3081/2009
PA	CY123547	H1N1	08/19/2009	Singapore	A/Singapore/ON1813/2009
PA	CY123539	H1N1	08/19/2009	Singapore	A/Singapore/ON1811/2009
PA	KP222642	H1N1	08/20/2009	Singapore	A/Singapore/480Y/2009

PA	CY128152	H1N1	08/20/2009	Viet Nam	A/Viet Nam/13032008/2009
PA	CY128168	H1N1	08/20/2009	Viet Nam	A/Viet Nam/13032011/2009
PA	JQ431336	H1N1	08/20/2009	France	A/Reunion/2923-3-M1E/2009
PA	CY072771	H1N1	08/20/2009	Nicaragua	A/Managua/287.01/2009(H1N1)
PA	CY123074	H1N1	08/20/2009	Singapore	A/Singapore/GP3178/2009
PA	KY925280	H1N1	08/20/2009	Brazil	A/Porto Alegre/LACENRS-2121/2009
PA	KY925503	H1N1	08/20/2009	Brazil	A/Rosario do Sul/LACENRS-2110/200
PA	CY072875	H1N1	08/20/2009	Nicaragua	A/Managua/2999.03/2009(H1N1)
PA	CY055921	H1N1	08/20/2009	Australia	A/Australia/79/2009(H1N1)
PA	CY055278	H1N1	08/20/2009	Malaysia	A/Malaysia/12617/2009(H1N1)
PA	CY128184	H1N1	08/20/2009	Viet Nam	A/Viet Nam/13032016/2009
PA	CY128176	H1N1	08/20/2009	Viet Nam	A/Viet Nam/13032015/2009
PA	CY128160	H1N1	08/20/2009	Viet Nam	A/Viet Nam/13032009/2009
PA	CY051724	H1N1	08/20/2009	USA	A/New York/4789/2009(H1N1)
PA	CY052167	H1N1	08/21/2009	USA	A/New York/4788/2009(H1N1)
PA	CY051732	H1N1	08/21/2009	USA	A/New York/4790/2009(H1N1)
PA	JQ431341	H1N1	08/21/2009	France	A/Reunion/2433-3-M1E/2009
PA	CY051748	H1N1	08/21/2009	USA	A/New York/4810/2009(H1N1)
PA	CY051716	H1N1	08/21/2009	USA	A/New York/4787/2009(H1N1)
PA	KP222643	H1N1	08/21/2009	Singapore	A/Singapore/483A/2009
PA	CY060525	H1N1	08/21/2009	Canada	A/Ontario/235657/2009(H1N1)
PA	CY128192	H1N1	08/21/2009	Viet Nam	A/Viet Nam/13032019/2009
PA	JQ431356	H1N1	08/21/2009	France	A/Reunion/1572-3-M1E/2009
PA	CY123082	H1N1	08/21/2009	Singapore	A/Singapore/GP3179/2009
PA	CY123066	H1N1	08/21/2009	Singapore	A/Singapore/GP3175/2009
PA	JQ431360	H1N1	08/22/2009	France	A/Reunion/1709-4-M2E/2009
PA	KT180889	H1N1	08/22/2009	Singapore	A/Singapore/DMS26/2009
PA	CY072755	H1N1	08/22/2009	Nicaragua	A/Managua/5227.04/2009(H1N1)
PA	CY072803	H1N1	08/22/2009	Nicaragua	A/Managua/432.01/2009(H1N1)
PA	CY072867	H1N1	08/22/2009	Nicaragua	A/Managua/4147.01/2009(H1N1)
PA	KY925224	H1N1	08/23/2009	Brazil	A/Ijui/LACENRS-2326/2009
PA	CY123113	H1N1	08/23/2009	Singapore	A/Singapore/GP3205/2009
PA	CY060533	H1N1	08/24/2009	Canada	A/Ontario/237882/2009(H1N1)
PA	CY060501	H1N1	08/24/2009	Canada	A/Ontario/10016/2009(H1N1)
PA	CY072819	H1N1	08/24/2009	Nicaragua	A/Managua/3421.02/2009(H1N1)
PA	CY051740	H1N1	08/24/2009	USA	A/New York/4791/2009(H1N1)
PA	KT180926	H1N1	08/24/2009	Singapore	A/Singapore/DMS623/2009
PA	JQ431338	H1N1	08/24/2009	France	A/Reunion/2378-1-M1E/2009
PA	JQ431345	H1N1	08/24/2009	France	A/Reunion/2133-5-M1E/2009
PA	JQ431350	H1N1	08/24/2009	France	A/Reunion/2133-6-M1E/2009
PA	CY119543	H1N1	08/24/2009	Malaysia	A/Malaysia/2126082/2009
PA	CY119463	H1N1	08/24/2009	Malaysia	A/Malaysia/2126061/2009
PA	CY123555	H1N1	08/24/2009	Singapore	A/Singapore/ON1852/2009
PA	CY123565	H1N1	08/24/2009	Singapore	A/Singapore/ON1858/2009
PA	CY123098	H1N1	08/24/2009	Singapore	A/Singapore/GP3187/2009
PA	CY056400	H1N1	08/25/2009	USA	A/New York/4925/2009(H1N1)
PA	KC781520	H1N1	08/25/2009	USA	A/Utah/51/2009
PA	KC780674	H1N1	08/25/2009	USA	A/West Virginia/13/2009
PA	CY058593	H1N1	08/25/2009	Nicaragua	A/Managua/65.02/2009(H1N1)
PA	KP222644	H1N1	08/25/2009	Singapore	A/Singapore/491H/2009
PA	KT180923	H1N1	08/25/2009	Singapore	A/Singapore/DMS617/2009
PA	CY065797	H1N1	08/25/2009	Netherlan	A/Netherlands/1039/2009(H1N1)
PA	KT180924	H1N1	08/25/2009	Singapore	A/Singapore/DMS618/2009
PA	KT180928	H1N1	08/25/2009	Singapore	A/Singapore/DMS638/2009

PA	CY073016	H1N1	08/26/2009	Hong Kong	A/Hong Kong/WRAIR0113N/2009(H1N1)
PA	CY074973	H1N1	08/26/2009	Thailand	A/Thailand/CU-C161/2009(H1N1)
PA	KP222646	H1N1	08/26/2009	Singapore	A/Singapore/496T/2009
PA	KP222645	H1N1	08/26/2009	Singapore	A/Singapore/495W/2009
PA	JQ431344	H1N1	08/26/2009	France	A/Reunion/0159-3-M1E/2009
PA	JQ431362	H1N1	08/26/2009	France	A/Reunion/2224-3-M3E/2009
PA	KT180931	H1N1	08/26/2009	Singapore	A/Singapore/DMS653/2009
PA	CY055929	H1N1	08/26/2009	Australia	A/Australia/80/2009(H1N1)
PA	CY123583	H1N1	08/27/2009	Singapore	A/Singapore/ON1892/2009
PA	CY123090	H1N1	08/27/2009	Singapore	A/Singapore/GP3183/2009
PA	JN187242	H1N1	08/27/2009	Taiwan	A/Taiwan/6078/2009
PA	KT180935	H1N1	08/27/2009	Singapore	A/Singapore/DMS676/2009
PA	CY072907	H1N1	08/27/2009	Nicaragua	A/Managua/3434.02/2009(H1N1)
PA	KP222647	H1N1	08/27/2009	Singapore	A/Singapore/501L/2009
PA	CY119471	H1N1	08/27/2009	Malaysia	A/Malaysia/2135565/2009
PA	CY055048	H1N1	08/27/2009	USA	A/California/VRDL70/2009(H1N1)
PA	AB538388	H1N1	08/27/2009	Japan	A/Nagano/RC1/2009(H1N1)
PA	CY073024	H1N1	08/27/2009	Hong Kong	A/Hong Kong/WRAIR0114T/2009(H1N1)
PA	CY123591	H1N1	08/27/2009	Singapore	A/Singapore/ON1896/2009
PA	KT180933	H1N1	08/27/2009	Singapore	A/Singapore/DMS665/2009
PA	KP222648	H1N1	08/27/2009	Singapore	A/Singapore/502J/2009
PA	CY123575	H1N1	08/27/2009	Singapore	A/Singapore/ON1888/2009
PA	KC781962	H1N1	08/27/2009	USA	A/Mississippi/05/2009
PA	KC780560	H1N1	08/28/2009	USA	A/Minnesota/15/2009
PA	KC782319	H1N1	08/28/2009	USA	A/Minnesota/16/2009
PA	CY072947	H1N1	08/28/2009	Nicaragua	A/Managua/1244.01/2009(H1N1)
PA	CY123599	H1N1	08/28/2009	Singapore	A/Singapore/ON1900/2009
PA	CY123615	H1N1	08/28/2009	Singapore	A/Singapore/ON1911/2009
PA	HM241710	H1N1	08/28/2009	India	A/Ytml/NIV9438/2009
PA	KT180938	H1N1	08/28/2009	Singapore	A/Singapore/DMS698/2009
PA	KT180890	H1N1	08/28/2009	Singapore	A/Singapore/DMS28/2009
PA	CY123607	H1N1	08/28/2009	Singapore	A/Singapore/ON1908/2009
PA	CY052772	H1N1	08/28/2009	USA	A/Texas/44282651/2009(H1N1)
PA	CY055056	H1N1	08/28/2009	USA	A/California/VRDL71/2009(H1N1)
PA	CY073670	H1N1	08/28/2009	Nicaragua	A/Managua/4209.04/2009(H1N1)
PA	KP222649	H1N1	08/28/2009	Singapore	A/Singapore/506T/2009
PA	CY088701	H1N1	08/29/2009	India	A/Pune/NIV9355/2009
PA	KP637845	H1N1	08/29/2009	Thailand	A/Thailand/IY07086/2009
PA	CY119479	H1N1	08/29/2009	Malaysia	A/Malaysia/2139971/2009
PA	CY128200	H1N1	08/29/2009	Viet Nam	A/Viet Nam/13032030/2009
PA	HM241703	H1N1	08/30/2009	India	A/Jalna/NIV9436/2009
PA	CY052231	H1N1	08/30/2009	USA	A/Texas/44302551/2009(H1N1)
PA	CY052628	H1N1	08/30/2009	USA	A/Texas/44302167/2009(H1N1)
PA	CY052223	H1N1	08/30/2009	USA	A/Texas/44301765/2009(H1N1)
PA	CY052620	H1N1	08/30/2009	USA	A/Texas/44302533/2009(H1N1)
PA	CY072795	H1N1	08/30/2009	Nicaragua	A/Managua/1984.02/2009(H1N1)
PA	CY123639	H1N1	08/31/2009	Singapore	A/Singapore/ON1924/2009
PA	CY052676	H1N1	08/31/2009	USA	A/Texas/44313703/2009(H1N1)
PA	CY073678	H1N1	08/31/2009	Nicaragua	A/Managua/5248.03/2009(H1N1)
PA	CY052343	H1N1	08/31/2009	USA	A/Texas/44312415/2009(H1N1)
PA	CY071401	H1N1	08/31/2009	Taiwan	A/Taipei/WR0193T/2009(H1N1)
PA	CY072931	H1N1	08/31/2009	Nicaragua	A/Managua/4063.01/2009(H1N1)
PA	CY123623	H1N1	08/31/2009	Singapore	A/Singapore/ON1914/2009
PA	CY123631	H1N1	08/31/2009	Singapore	A/Singapore/ON1921/2009

PA	KY926214	H1N1	08/31/2009	Brazil	A/Palmitinho/LACENRS-2595/2009
PA	KT180941	H1N1	08/31/2009	Singapore	A/Singapore/DMS722/2009
PA	KT180891	H1N1	08/31/2009	Singapore	A/Singapore/DMS29/2009
PA	CY072979	H1N1	08/31/2009	Nicaragua	A/Managua/3507.01/2009(H1N1)
PA	HM189659	H1N1	09/2009	South Ko	A/Korea/CJ01/2009(H1N1)
PA	HM189662	H1N1	09/2009	South Ko	A/Korea/CJ15/2009(H1N1)
PA	HM189660	H1N1	09/2009	South Ko	A/Korea/CJ04/2009(H1N1)
PA	HM189661	H1N1	09/2009	South Ko	A/Korea/CJ09/2009(H1N1)
PA	HM189663	H1N1	09/2009	South Ko	A/Korea/CJ19/2009(H1N1)
PA	HM189664	H1N1	09/2009	South Ko	A/Korea/CJ24/2009(H1N1)
PA	CY052018	H1N1	09/01/2009	Norway	A/Norway/3206-3/2009(H1N1)
PA	CY052684	H1N1	09/01/2009	USA	A/Texas/45010998/2009(H1N1)
PA	CY055491	H1N1	09/01/2009	USA	A/California/VRDL73/2009(H1N1)
PA	CY055483	H1N1	09/01/2009	USA	A/California/VRDL72/2009(H1N1)
PA	CY123141	H1N1	09/01/2009	Singapore	A/Singapore/GP3491/2009
PA	CY065128	H1N1	09/01/2009	Netherlan	A/Netherlands/1064b/2009(H1N1)
PA	KC782307	H1N1	09/01/2009	USA	A/Wisconsin/53/2009
PA	CY119335	H1N1	09/01/2009	Malaysia	A/Malaysia/2142295/2009(H1N1)
PA	CY119343	H1N1	09/01/2009	Malaysia	A/Malaysia/2142299/2009(H1N1)
PA	CY119351	H1N1	09/01/2009	Malaysia	A/Malaysia/2143696/2009(H1N1)
PA	CY123656	H1N1	09/01/2009	Singapore	A/Singapore/ON1934/2009
PA	CY128208	H1N1	09/01/2009	Viet Nam	A/Viet Nam/13032035/2009
PA	CY128464	H1N1	09/01/2009	Viet Nam	A/Viet Nam/001-1889/2009
PA	CY073686	H1N1	09/01/2009	Nicaragua	A/Managua/4736.02/2009(H1N1)
PA	CY050018	H1N1	09/02/2009	Mexico	A/Mexico City/WR1306N/2009(H1N1)
PA	CY051756	H1N1	09/02/2009	USA	A/New York/4820/2009(H1N1)
PA	CY052860	H1N1	09/02/2009	USA	A/Texas/45023717/2009(H1N1)
PA	CY052780	H1N1	09/02/2009	USA	A/Texas/45021632/2009(H1N1)
PA	CY052884	H1N1	09/02/2009	USA	A/Texas/45024243/2009(H1N1)
PA	GU271965	H1N1	09/02/2009	Thailand	A/Thailand/CU-H565/2009(H1N1)
PA	CY055945	H1N1	09/02/2009	Australia	A/Australia/82/2009(H1N1)
PA	CY055937	H1N1	09/02/2009	Australia	A/Australia/81/2009(H1N1)
PA	CY069103	H1N1	09/02/2009	Guam	A/Guam/NHRC0027/2009(H1N1)
PA	CY072747	H1N1	09/02/2009	Nicaragua	A/Managua/2958.01/2009(H1N1)
PA	CY074989	H1N1	09/02/2009	Thailand	A/Thailand/CU-H567/2009
PA	CY123666	H1N1	09/02/2009	Singapore	A/Singapore/ON1943/2009
PA	JQ431347	H1N1	09/02/2009	France	A/Reunion/2956-2-M1E/2009
PA	JQ431352	H1N1	09/02/2009	France	A/Reunion/2154-4-M4E/2009
PA	JQ431363	H1N1	09/02/2009	France	A/Reunion/2692-2-M2E/2009
PA	KT180945	H1N1	09/02/2009	Singapore	A/Singapore/DMS752/2009
PA	KT180942	H1N1	09/02/2009	Singapore	A/Singapore/DMS737/2009
PA	KY926290	H1N1	09/02/2009	Brazil	A/Osorio/LACENRS-2638/2009
PA	CY052604	H1N1	09/03/2009	USA	A/Texas/45033567/2009(H1N1)
PA	CY123674	H1N1	09/03/2009	Singapore	A/Singapore/ON1955/2009
PA	CY123682	H1N1	09/03/2009	Singapore	A/Singapore/ON1964/2009
PA	KT180892	H1N1	09/03/2009	Singapore	A/Singapore/DMS30/2009
PA	CY074997	H1N1	09/03/2009	Thailand	A/Thailand/CU-H572/2009
PA	CY090086	H1N1	09/03/2009	Nicaragua	A/Managua/4032.03/2009
PA	CY128216	H1N1	09/03/2009	Viet Nam	A/Viet Nam/13032036/2009
PA	CY088708	H1N1	09/03/2009	India	A/Mum/NIV9945/2009
PA	CY052940	H1N1	09/03/2009	USA	A/Texas/45032753/2009(H1N1)
PA	CY052908	H1N1	09/03/2009	USA	A/Texas/45032708/2009(H1N1)
PA	CY051772	H1N1	09/03/2009	USA	A/New York/4824/2009(H1N1)
PA	CY051780	H1N1	09/03/2009	USA	A/New York/4841/2009(H1N1)

PA	CY051764	H1N1	09/03/2009	USA	A/New York/4823/2009(H1N1)
PA	CY052596	H1N1	09/03/2009	USA	A/Texas/45033774/2009(H1N1)
PA	CY052612	H1N1	09/03/2009	USA	A/Texas/45034157/2009(H1N1)
PA	JQ431357	H1N1	09/03/2009	France	A/Reunion/2969-5-M1E/2009
PA	CY069015	H1N1	09/04/2009	Guam	A/Guam/NHRC0016/2009(H1N1)
PA	KT180950	H1N1	09/04/2009	Singapore	A/Singapore/DMS804/2009
PA	CY052788	H1N1	09/04/2009	USA	A/Texas/45043852/2009(H1N1)
PA	CY068991	H1N1	09/04/2009	Guam	A/Guam/NHRC0013/2009(H1N1)
PA	JQ431346	H1N1	09/04/2009	France	A/Reunion/2956-1-M2E/2009
PA	CY128224	H1N1	09/04/2009	Viet Nam	A/Viet Nam/13032037/2009
PA	JQ431342	H1N1	09/04/2009	France	A/Reunion/0215-4-M1E/2009
PA	CY052868	H1N1	09/04/2009	USA	A/Texas/45042604/2009(H1N1)
PA	CY065885	H1N1	09/04/2009	Netherlar	A/Netherlands/1093/2009(H1N1)
PA	CY070996	H1N1	09/04/2009	Guam	A/Guam/NHRC0029/2009(H1N1)
PA	CY071833	H1N1	09/05/2009	USA	A/South Carolina/WRSP520/2009(H1N
PA	CY072811	H1N1	09/05/2009	Nicaragua	A/Managua/572.02/2009(H1N1)
PA	KT180954	H1N1	09/05/2009	Singapore	A/Singapore/DMS835/2009
PA	CY052924	H1N1	09/05/2009	USA	A/Texas/45052569/2009(H1N1)
PA	JQ431351	H1N1	09/05/2009	France	A/Reunion/2154-2-M5E/2009
PA	JQ431358	H1N1	09/05/2009	France	A/Reunion/0116-3-M1E/2009
PA	CY052644	H1N1	09/06/2009	USA	A/Texas/45062584/2009(H1N1)
PA	GU108485	H1N1	09/06/2009	China	A/Zhejiang-Yiwu/11/2009(H1N1)
PA	CY052892	H1N1	09/06/2009	USA	A/Texas/45062346/2009(H1N1)
PA	CY051788	H1N1	09/06/2009	USA	A/New York/4844/2009(H1N1)
PA	KT180893	H1N1	09/06/2009	Singapore	A/Singapore/DMS31/2009
PA	CY051900	H1N1	09/06/2009	USA	A/Texas/45062633/2009(H1N1)
PA	CY052956	H1N1	09/06/2009	USA	A/Texas/45061755/2009(H1N1)
PA	CY052636	H1N1	09/06/2009	USA	A/Texas/45061670/2009(H1N1)
PA	CY052812	H1N1	09/07/2009	USA	A/Texas/45071344/2009(H1N1)
PA	CY052796	H1N1	09/07/2009	USA	A/Texas/45072273/2009(H1N1)
PA	GU112095	H1N1	09/07/2009	China	A/Zhejiang/DTID-ZJU02/2009(H1N1)
PA	GU189654	H1N1	09/07/2009	China	A/Zhejiang/DTID-ZJU03/2009(H1N1)
PA	CY052820	H1N1	09/07/2009	USA	A/Texas/45072656/2009(H1N1)
PA	CY052828	H1N1	09/07/2009	USA	A/Texas/45072128/2009(H1N1)
PA	CY052692	H1N1	09/07/2009	USA	A/Texas/45071524/2009(H1N1)
PA	HM241717	H1N1	09/07/2009	India	A/Nsk/NIV10348/2009(H1N1)
PA	CY060549	H1N1	09/07/2009	Canada	A/Ontario/25913/2009(H1N1)
PA	CY057883	H1N1	09/07/2009	USA	A/Wisconsin/629-D01987/2009(H1N1)
PA	CY088638	H1N1	09/07/2009	India	A/Pune/NIV10278/2009
PA	JQ431359	H1N1	09/07/2009	France	A/Reunion/0116-3-M2E/2009
PA	JQ431339	H1N1	09/07/2009	France	A/Reunion/0215-4-M2E/2009
PA	CY119487	H1N1	09/07/2009	Malaysia	A/Malaysia/2156796/2009
PA	KT180957	H1N1	09/08/2009	Singapore	A/Singapore/DMS869/2009
PA	KT180955	H1N1	09/08/2009	Singapore	A/Singapore/DMS847/2009
PA	CY057008	H1N1	09/08/2009	USA	A/Wisconsin/629-D02367/2009(H1N1)
PA	CY128232	H1N1	09/08/2009	Viet Nam	A/Viet Nam/13032040/2009
PA	CY123700	H1N1	09/08/2009	Singapore	A/Singapore/ON2009/2009
PA	CY057000	H1N1	09/08/2009	USA	A/Wisconsin/629-D00734/2009(H1N1)
PA	CY052700	H1N1	09/08/2009	USA	A/Texas/45083819/2009(H1N1)
PA	CY051997	H1N1	09/08/2009	Norway	A/Norway/3364-2/2009(H1N1)
PA	CY073694	H1N1	09/08/2009	Nicaragua	A/Managua/5953.01/2009(H1N1)
PA	CY057016	H1N1	09/09/2009	USA	A/Wisconsin/629-D02041/2009(H1N1)
PA	CY052660	H1N1	09/09/2009	USA	A/Texas/45093846/2009(H1N1)
PA	CY052724	H1N1	09/09/2009	USA	A/Texas/45091402/2009(H1N1)

PA	HM568135	H1N1	09/09/2009	United Ki	A/England/H093780041/2009
PA	JN187243	H1N1	09/09/2009	Taiwan	A/Taiwan/937/2009
PA	CY083374	H1N1	09/09/2009	USA	A/Pendleton/WRAIR1661P/2009(H1N1)
PA	CY083398	H1N1	09/09/2009	USA	A/Great Lakes/WRAIR1664P/2009
PA	CY069087	H1N1	09/09/2009	Guam	A/Guam/NHRC0025/2009(H1N1)
PA	KT180958	H1N1	09/09/2009	Singapore	A/Singapore/DMS870/2009
PA	CY057024	H1N1	09/09/2009	USA	A/Wisconsin/629-D02133/2009(H1N1)
PA	CY069111	H1N1	09/09/2009	Guam	A/Guam/NHRC0028/2009(H1N1)
PA	CY052740	H1N1	09/09/2009	USA	A/Texas/45091417/2009(H1N1)
PA	CY052668	H1N1	09/09/2009	USA	A/Texas/45093670/2009(H1N1)
PA	CY052175	H1N1	09/09/2009	USA	A/New York/4855/2009(H1N1)
PA	CY051796	H1N1	09/09/2009	USA	A/New York/4856/2009(H1N1)
PA	CY051804	H1N1	09/09/2009	USA	A/New York/4866/2009(H1N1)
PA	CY050042	H1N1	09/09/2009	Mexico	A/Mexico City/WR1310N/2009(H1N1)
PA	CY052732	H1N1	09/09/2009	USA	A/Texas/45091405/2009(H1N1)
PA	CY052708	H1N1	09/09/2009	USA	A/Texas/45093214/2009(H1N1)
PA	CY119519	H1N1	09/10/2009	Malaysia	A/Malaysia/2097960/2009
PA	HM241724	H1N1	09/10/2009	India	A/Pune/NIV10604/2009
PA	JN187244	H1N1	09/10/2009	Taiwan	A/Taiwan/940/2009
PA	HM568143	H1N1	09/10/2009	United Ki	A/England/H093780034/2009
PA	KT180962	H1N1	09/10/2009	Singapore	A/Singapore/DMS905/2009
PA	KY925373	H1N1	09/10/2009	Brazil	A/Campina das Missoes/LACENRS-288
PA	KC781747	H1N1	09/10/2009	USA	A/North Carolina/37/2009
PA	CY052516	H1N1	09/10/2009	USA	A/Texas/45103737/2009(H1N1)
PA	CY052652	H1N1	09/10/2009	USA	A/Texas/45103259/2009(H1N1)
PA	CY050058	H1N1	09/10/2009	Mexico	A/Mexico City/WR1312N/2009(H1N1)
PA	CY050050	H1N1	09/10/2009	Mexico	A/Mexico City/WR1311T/2009(H1N1)
PA	CY051812	H1N1	09/10/2009	USA	A/New York/4870/2009(H1N1)
PA	CY052524	H1N1	09/10/2009	USA	A/Texas/45104048/2009(H1N1)
PA	CY052748	H1N1	09/10/2009	USA	A/Texas/45101424/2009(H1N1)
PA	CY052532	H1N1	09/10/2009	USA	A/Texas/45103998/2009(H1N1)
PA	CY052756	H1N1	09/10/2009	USA	A/Texas/45103759/2009(H1N1)
PA	CY052508	H1N1	09/10/2009	USA	A/Texas/45104026/2009(H1N1)
PA	CY052900	H1N1	09/10/2009	USA	A/Texas/45102952/2009(H1N1)
PA	CY052764	H1N1	09/10/2009	USA	A/Texas/45101422/2009(H1N1)
PA	CY072923	H1N1	09/10/2009	Nicaragua	A/Managua/4972.02/2009(H1N1)
PA	CY065917	H1N1	09/10/2009	Netherlan	A/Netherlands/1084/2009(H1N1)
PA	CY065909	H1N1	09/10/2009	Netherlan	A/Netherlands/1083/2009(H1N1)
PA	CY057032	H1N1	09/10/2009	USA	A/Wisconsin/629-D00357/2009(H1N1)
PA	CY118263	H1N1	09/10/2009	Malaysia	A/Malaysia/2089302/2009
PA	CY128240	H1N1	09/11/2009	Viet Nam	A/Viet Nam/13032044/2009
PA	CY123153	H1N1	09/11/2009	Singapore	A/Singapore/GP3545/2009
PA	CY083063	H1N1	09/11/2009	Colombia	A/Bogota/WRAIR0435N/2009
PA	CY083071	H1N1	09/11/2009	Colombia	A/Bogota/WRAIR0435T/2009
PA	CY072827	H1N1	09/11/2009	Nicaragua	A/Managua/5115.01/2009(H1N1)
PA	CY057363	H1N1	09/11/2009	USA	A/Wisconsin/629-D02485/2009(H1N1)
PA	KT180964	H1N1	09/11/2009	Singapore	A/Singapore/DMS913/2009
PA	KC780105	H1N1	09/11/2009	USA	A/Alaska/38/2009
PA	CY052500	H1N1	09/11/2009	USA	A/Texas/45113371/2009(H1N1)
PA	CY052540	H1N1	09/11/2009	USA	A/Texas/45113882/2009(H1N1)
PA	CY052916	H1N1	09/11/2009	USA	A/Texas/45113911/2009(H1N1)
PA	CY073518	H1N1	09/11/2009	Nicaragua	A/Managua/3014.02/2009(H1N1)
PA	CY057371	H1N1	09/12/2009	USA	A/Wisconsin/629-D00643/2009(H1N1)
PA	CY057379	H1N1	09/12/2009	USA	A/Wisconsin/629-D01850/2009(H1N1)

PA	KT180894	H1N1	09/12/2009	Singapore	A/Singapore/DMS33/2009
PA	CY052556	H1N1	09/12/2009	USA	A/Texas/45122774/2009(H1N1)
PA	CY052932	H1N1	09/12/2009	USA	A/Texas/45121004/2009(H1N1)
PA	CY052484	H1N1	09/12/2009	USA	A/Texas/45122282/2009(H1N1)
PA	CY052492	H1N1	09/12/2009	USA	A/Texas/45122036/2009(H1N1)
PA	CY052948	H1N1	09/12/2009	USA	A/Texas/45122886/2009(H1N1)
PA	CY050010	H1N1	09/12/2009	Mexico	A/Mexico City/WR1301N/2009(H1N1)
PA	CY052183	H1N1	09/12/2009	USA	A/New York/4881/2009(H1N1)
PA	CY052436	H1N1	09/12/2009	USA	A/Texas/45120922/2009(H1N1)
PA	CY052444	H1N1	09/12/2009	USA	A/Texas/45122538/2009(H1N1)
PA	CY052460	H1N1	09/12/2009	USA	A/Texas/45122369/2009(H1N1)
PA	CY052564	H1N1	09/12/2009	USA	A/Texas/45122722/2009(H1N1)
PA	CY052476	H1N1	09/12/2009	USA	A/Texas/45122033/2009(H1N1)
PA	CY052468	H1N1	09/12/2009	USA	A/Texas/45121606/2009(H1N1)
PA	CY072835	H1N1	09/12/2009	Nicaragua	A/Managua/1909.02/2009(H1N1)
PA	CY072739	H1N1	09/12/2009	Nicaragua	A/Managua/1419.02/2009(H1N1)
PA	CY052580	H1N1	09/13/2009	USA	A/Texas/45131774/2009(H1N1)
PA	CY069023	H1N1	09/13/2009	Guam	A/Guam/NHRC0017/2009(H1N1)
PA	KC780878	H1N1	09/13/2009	USA	A/Texas/67/2009
PA	CY052588	H1N1	09/13/2009	USA	A/Texas/45132647/2009(H1N1)
PA	CY052452	H1N1	09/13/2009	USA	A/Texas/45130742/2009(H1N1)
PA	CY052572	H1N1	09/13/2009	USA	A/Texas/45132788/2009(H1N1)
PA	CY052804	H1N1	09/13/2009	USA	A/Texas/45131576/2009(H1N1)
PA	CY052836	H1N1	09/13/2009	USA	A/Texas/45131305/2009(H1N1)
PA	CY049650	H1N1	09/13/2009	Singapore	A/Singapore/ON2081/2009(H1N1)
PA	CY052548	H1N1	09/13/2009	USA	A/Texas/45132214/2009(H1N1)
PA	CY052844	H1N1	09/13/2009	USA	A/Texas/45132202/2009(H1N1)
PA	KF897771	H1N1	09/14/2009	France	A/StEtienne/1691/2009
PA	CY071020	H1N1	09/14/2009	Guam	A/Guam/NHRC0032/2009(H1N1)
PA	JX625559	H1N1	09/14/2009	United Ki	A/England/94560024/2009
PA	KC780918	H1N1	09/14/2009	USA	A/Kansas/19/2009
PA	KC780833	H1N1	09/14/2009	USA	A/Kansas/20/2009
PA	CY123173	H1N1	09/14/2009	Singapore	A/Singapore/GP3710/2009
PA	CY052716	H1N1	09/14/2009	USA	A/Texas/45140902/2009(H1N1)
PA	CY123744	H1N1	09/14/2009	Singapore	A/Singapore/ON2081/2009
PA	CY072939	H1N1	09/14/2009	Nicaragua	A/Managua/5590.02/2009(H1N1)
PA	CY071012	H1N1	09/14/2009	Guam	A/Guam/NHRC0031/2009(H1N1)
PA	CY072891	H1N1	09/14/2009	Nicaragua	A/Managua/12.01/2009(H1N1)
PA	CY069047	H1N1	09/14/2009	Guam	A/Guam/NHRC0020/2009(H1N1)
PA	CY055428	H1N1	09/14/2009	USA	A/New York/4887/2009(H1N1)
PA	CY057403	H1N1	09/15/2009	USA	A/Wisconsin/629-D01469/2009(H1N1)
PA	CY057387	H1N1	09/15/2009	USA	A/Wisconsin/629-D02192/2009(H1N1)
PA	CY057395	H1N1	09/15/2009	USA	A/Wisconsin/629-D00036/2009(H1N1)
PA	CY057411	H1N1	09/15/2009	USA	A/Wisconsin/629-D00832/2009(H1N1)
PA	CY069071	H1N1	09/15/2009	Guam	A/Guam/NHRC0023/2009(H1N1)
PA	CY053473	H1N1	09/15/2009	Taiwan	A/Taiwan/143/2009(H1N1)
PA	CY123752	H1N1	09/15/2009	Singapore	A/Singapore/ON2095/2009
PA	KC781043	H1N1	09/15/2009	USA	A/Massachusetts/31/2009
PA	KT180895	H1N1	09/15/2009	Singapore	A/Singapore/DMS34/2009
PA	KT180967	H1N1	09/15/2009	Singapore	A/Singapore/DMS946/2009
PA	KT180968	H1N1	09/15/2009	Singapore	A/Singapore/DMS952/2009
PA	CY090094	H1N1	09/15/2009	Nicaragua	A/Managua/3755.03/2009
PA	CY119495	H1N1	09/15/2009	Malaysia	A/Malaysia/2173411/2009
PA	KT180970	H1N1	09/16/2009	Singapore	A/Singapore/DMS962/2009

PA	CY057435	H1N1	09/16/2009	USA	A/Wisconsin/629-D01935/2009(H1N1)
PA	KC782211	H1N1	09/16/2009	USA	A/South Carolina/18/2009
PA	KC780080	H1N1	09/16/2009	USA	A/South Carolina/18/2009
PA	CY120736	H1N1	09/16/2009	Brazil	A/Brazil/AVS06/2009
PA	CY057419	H1N1	09/16/2009	USA	A/Wisconsin/629-D01802/2009(H1N1)
PA	CY088652	H1N1	09/16/2009	India	A/Rtng/NIV11650/2009
PA	KJ690470	H1N1	09/16/2009	Uganda	A/Uganda/MUWRP-094/2009
PA	KC782209	H1N1	09/16/2009	USA	A/South Carolina/18/2009
PA	CY072787	H1N1	09/16/2009	Nicaragua	A/Managua/5401.01/2009(H1N1)
PA	KC780852	H1N1	09/16/2009	USA	A/Kansas/21/2009
PA	CY057427	H1N1	09/16/2009	USA	A/Wisconsin/629-D00485/2009(H1N1)
PA	KC781307	H1N1	09/16/2009	USA	A/Wisconsin/66/2009
PA	CY090070	H1N1	09/17/2009	Nicaragua	A/Managua/4877.02/2009
PA	CY071721	H1N1	09/17/2009	Mexico	A/Mexico City/WR1696T/2009(H1N1)
PA	CY128248	H1N1	09/17/2009	Viet Nam	A/Viet Nam/13032048/2009
PA	CY119503	H1N1	09/18/2009	Malaysia	A/Malaysia/2180812/2009
PA	HM568151	H1N1	09/18/2009	United Ki	A/England/H093900113/2009
PA	CY053505	H1N1	09/18/2009	Taiwan	A/Taiwan/206/2009(H1N1)
PA	CY071753	H1N1	09/18/2009	Mexico	A/Mexico City/WR1706T/2009(H1N1)
PA	CY053497	H1N1	09/18/2009	Taiwan	A/Taiwan/177/2009(H1N1)
PA	KT180896	H1N1	09/18/2009	Singapore	A/Singapore/DMS35/2009
PA	KT180975	H1N1	09/18/2009	Singapore	A/Singapore/DMS982/2009
PA	KY926426	H1N1	09/18/2009	Brazil	A/Pelotas/LACENRS-3045/2009
PA	KP637541	H1N1	09/18/2009	Thailand	A/Thailand/SN08312/2009
PA	KT180974	H1N1	09/18/2009	Singapore	A/Singapore/DMS980/2009
PA	CY053481	H1N1	09/18/2009	Taiwan	A/Taiwan/156/2009(H1N1)
PA	KT180972	H1N1	09/18/2009	Singapore	A/Singapore/DMS978/2009
PA	CY053489	H1N1	09/18/2009	Taiwan	A/Taiwan/167/2009(H1N1)
PA	CY057443	H1N1	09/18/2009	USA	A/Wisconsin/629-D01522/2009(H1N1)
PA	CY055507	H1N1	09/19/2009	USA	A/California/VRDL75/2009(H1N1)
PA	KC781055	H1N1	09/20/2009	USA	A/North Carolina/44/2009
PA	CY068999	H1N1	09/20/2009	Guam	A/Guam/NHRC0014/2009(H1N1)
PA	JN187245	H1N1	09/20/2009	Taiwan	A/Taiwan/8575/2009
PA	KT180769	H1N1	09/21/2009	Singapore	A/Singapore/DMS1006/2009
PA	CY057451	H1N1	09/21/2009	USA	A/Wisconsin/629-D01355/2009(H1N1)
PA	CY083430	H1N1	09/21/2009	USA	A/San Diego/WRAIR1668P/2009
PA	AB539738	H1N1	09/21/2009	China	A/Shanghai/P1/2009(H1N1)
PA	CY057459	H1N1	09/21/2009	USA	A/Wisconsin/629-D00748/2009(H1N1)
PA	KJ690517	H1N1	09/21/2009	Uganda	A/Uganda/MUWRP-092/2009
PA	CY055515	H1N1	09/21/2009	USA	A/California/VRDL76/2009(H1N1)
PA	CY057467	H1N1	09/22/2009	USA	A/Wisconsin/629-D00839/2009(H1N1)
PA	GU211232	H1N1	09/22/2009	Russia	A/Russia/01/2009(H1N1)
PA	CY055523	H1N1	09/22/2009	USA	A/California/VRDL78/2009(H1N1)
PA	KC781982	H1N1	09/22/2009	USA	A/Kansas/22/2009
PA	CY071705	H1N1	09/22/2009	Mexico	A/Mexico City/WR1692T/2009(H1N1)
PA	CY128448	H1N1	09/22/2009	Viet Nam	A/Viet Nam/001-1716/2009
PA	CY057483	H1N1	09/22/2009	USA	A/Wisconsin/629-D01237/2009(H1N1)
PA	CY057475	H1N1	09/22/2009	USA	A/Wisconsin/629-D00402/2009(H1N1)
PA	CY083406	H1N1	09/23/2009	USA	A/San Diego/WRAIR1665P/2009
PA	CY083486	H1N1	09/23/2009	Mexico	A/Mexico City/WRAIR1691N/2009(H1N
PA	CY056408	H1N1	09/23/2009	USA	A/New York/4944/2009(H1N1)
PA	CY056416	H1N1	09/23/2009	USA	A/New York/4945/2009(H1N1)
PA	CY056424	H1N1	09/23/2009	USA	A/New York/4947/2009(H1N1)
PA	CY057491	H1N1	09/23/2009	USA	A/Wisconsin/629-D00346/2009(H1N1)

PA	CY071697	H1N1	09/23/2009	Mexico	A/Mexico City/WR1687T/2009(H1N1)
PA	CY057499	H1N1	09/23/2009	USA	A/Wisconsin/629-D00287/2009(H1N1)
PA	KJ690399	H1N1	09/23/2009	Uganda	A/Uganda/MUWRP-093/2009
PA	CY123774	H1N1	09/23/2009	Singapore	A/Singapore/ON2136/2009
PA	CY123187	H1N1	09/23/2009	Singapore	A/Singapore/GP3839/2009
PA	CY123766	H1N1	09/23/2009	Singapore	A/Singapore/ON2135/2009
PA	KC780969	H1N1	09/23/2009	USA	A/Kansas/23/2009
PA	CY119511	H1N1	09/24/2009	Malaysia	A/Malaysia/2190140/2009
PA	CY054888	H1N1	09/24/2009	USA	A/California/VRDL36/2009(H1N1)
PA	KT180897	H1N1	09/24/2009	Singapore	A/Singapore/DMS37/2009
PA	CY057515	H1N1	09/24/2009	USA	A/Wisconsin/629-D00410/2009(H1N1)
PA	CY071737	H1N1	09/24/2009	Mexico	A/Mexico City/WR1701T/2009(H1N1)
PA	CY057507	H1N1	09/24/2009	USA	A/Wisconsin/629-D00565/2009(H1N1)
PA	KC780157	H1N1	09/25/2009	USA	A/Mississippi/03/2009
PA	CY071745	H1N1	09/25/2009	Mexico	A/Mexico City/WR1704T/2009(H1N1)
PA	CY069063	H1N1	09/25/2009	Guam	A/Guam/NHRC0022/2009(H1N1)
PA	CY071689	H1N1	09/25/2009	Mexico	A/Mexico City/WR1683T/2009(H1N1)
PA	CY069079	H1N1	09/26/2009	Guam	A/Guam/NHRC0024/2009(H1N1)
PA	CY057523	H1N1	09/26/2009	USA	A/Wisconsin/629-D00891/2009(H1N1)
PA	KC780902	H1N1	09/27/2009	USA	A/Wisconsin/51/2009
PA	JQ431354	H1N1	09/28/2009	France	A/Reunion/1698-3-M1E/2009
PA	KJ690478	H1N1	09/28/2009	Uganda	A/Uganda/MUWRP-098/2009
PA	JN187246	H1N1	09/28/2009	Taiwan	A/Taiwan/4909/2009
PA	CY057531	H1N1	09/28/2009	USA	A/Wisconsin/629-D01391/2009(H1N1)
PA	JN187247	H1N1	09/28/2009	Taiwan	A/Taiwan/8542/2009
PA	CY123782	H1N1	09/28/2009	Singapore	A/Singapore/ON2145/2009
PA	CY060557	H1N1	09/29/2009	Canada	A/Ontario/26184/2009(H1N1)
PA	KC780986	H1N1	09/29/2009	USA	A/Texas/66/2009
PA	KC780997	H1N1	09/29/2009	USA	A/Texas/66/2009
PA	CY072955	H1N1	09/29/2009	Nicaragua	A/Managua/5258.02/2009(H1N1)
PA	CY058281	H1N1	09/29/2009	USA	A/Wisconsin/629-D01015/2009(H1N1)
PA	CY065925	H1N1	09/30/2009	Netherlar	A/Netherlands/2290/2009
PA	CY083438	H1N1	09/30/2009	USA	A/Ft.Benning/WRAIR1669P/2009
PA	CY123197	H1N1	09/30/2009	Singapore	A/Singapore/GP3912/2009
PA	KC780033	H1N1	09/30/2009	USA	A/Wisconsin/67/2009
PA	KT180899	H1N1	09/30/2009	Singapore	A/Singapore/DMS39/2009
PA	HM189668	H1N1	10/2009	South Kor	A/Korea/CJ109/2009(H1N1)
PA	HM189666	H1N1	10/2009	South Kor	A/Korea/CJ63/2009(H1N1)
PA	HM189665	H1N1	10/2009	South Kor	A/Korea/CJ40/2009(H1N1)
PA	HM189667	H1N1	10/2009	South Kor	A/Korea/CJ68/2009(H1N1)
PA	HM189669	H1N1	10/2009	South Kor	A/Korea/CJ112/2009(H1N1)
PA	CY069007	H1N1	10/01/2009	Guam	A/Guam/NHRC0015/2009(H1N1)
PA	CY062207	H1N1	10/01/2009	USA	A/California/VRDL80/2009(H1N1)
PA	CY056576	H1N1	10/01/2009	USA	A/New York/5755/2009(H1N1)
PA	CY062199	H1N1	10/01/2009	USA	A/California/VRDL79/2009(H1N1)
PA	KC781994	H1N1	10/01/2009	USA	A/North Carolina/45/2009
PA	CY083414	H1N1	10/01/2009	USA	A/San Diego/WRAIR1666P/2009
PA	CY063224	H1N1	10/01/2009	USA	A/Wisconsin/629-D01351/2009(H1N1)
PA	CY128256	H1N1	10/01/2009	Viet Nam	A/Viet Nam/13032051/2009
PA	CY118272	H1N1	10/01/2009	Malaysia	A/Malaysia/2205822/2009
PA	JX625543	H1N1	10/01/2009	United Ki	A/Northern Ireland/94480417/2009
PA	CY068959	H1N1	10/01/2009	Guam	A/Guam/NHRC0009/2009(H1N1)
PA	CY062223	H1N1	10/01/2009	USA	A/California/VRDL82/2009(H1N1)
PA	CY062231	H1N1	10/01/2009	USA	A/California/VRDL83/2009(H1N1)

PA	CY062215	H1N1	10/01/2009	USA	A/California/VRDL81/2009(H1N1)
PA	CY071713	H1N1	10/01/2009	Mexico	A/Mexico City/WR1695N/2009(H1N1)
PA	CY057539	H1N1	10/02/2009	USA	A/Wisconsin/629-D00250/2009(H1N1)
PA	JX625505	H1N1	10/02/2009	United Ki	A/England/94200009/2009
PA	CY083422	H1N1	10/02/2009	USA	A/San Diego/WRAIR1667P/2009
PA	KC780136	H1N1	10/02/2009	USA	A/New Mexico/08/2009
PA	KT180900	H1N1	10/02/2009	Singapore	A/Singapore/DMS40/2009
PA	CY057547	H1N1	10/02/2009	USA	A/Wisconsin/629-D00853/2009(H1N1)
PA	CY055436	H1N1	10/03/2009	USA	A/New York/4977/2009(H1N1)
PA	CY057555	H1N1	10/03/2009	USA	A/Wisconsin/629-D02337/2009(H1N1)
PA	CY057563	H1N1	10/04/2009	USA	A/Wisconsin/629-D00690/2009(H1N1)
PA	CY056488	H1N1	10/04/2009	USA	A/New York/4995/2009(H1N1)
PA	KC780376	H1N1	10/04/2009	USA	A/New York/101/2009
PA	CY056432	H1N1	10/05/2009	USA	A/New York/4981/2009(H1N1)
PA	CY056448	H1N1	10/05/2009	USA	A/New York/4985/2009(H1N1)
PA	KJ690541	H1N1	10/05/2009	Uganda	A/Uganda/MUWRP-102/2009
PA	KT180901	H1N1	10/05/2009	Singapore	A/Singapore/DMS41/2009
PA	JX625527	H1N1	10/05/2009	United Ki	A/Northern Ireland/94480397/2009
PA	KC780174	H1N1	10/05/2009	USA	A/Minnesota/24/2009
PA	CY123796	H1N1	10/05/2009	Singapore	A/Singapore/ON2180/2009
PA	CY056456	H1N1	10/05/2009	USA	A/New York/4986/2009(H1N1)
PA	CY055444	H1N1	10/05/2009	USA	A/New York/4979/2009(H1N1)
PA	CY056480	H1N1	10/05/2009	USA	A/New York/4990/2009(H1N1)
PA	CY057571	H1N1	10/05/2009	USA	A/Wisconsin/629-D00834/2009(H1N1)
PA	KC780929	H1N1	10/06/2009	USA	A/Colorado/13/2009
PA	CY056472	H1N1	10/06/2009	USA	A/New York/4988/2009(H1N1)
PA	CY057579	H1N1	10/06/2009	USA	A/Wisconsin/629-S1252/2009(H1N1)
PA	CY056440	H1N1	10/06/2009	USA	A/New York/4984/2009(H1N1)
PA	CY058289	H1N1	10/06/2009	USA	A/Wisconsin/629-D01102/2009(H1N1)
PA	CY083079	H1N1	10/06/2009	Colombia	A/Bogota/WRAIR0440T/2009
PA	CY088659	H1N1	10/06/2009	India	A/Ngp/NIV14725/2009(H1N1)
PA	CY056464	H1N1	10/06/2009	USA	A/New York/4987/2009(H1N1)
PA	CY083111	H1N1	10/07/2009	Ecuador	A/Quito/WRAIR0617N/2009
PA	CY058297	H1N1	10/07/2009	USA	A/Wisconsin/629-D02100/2009(H1N1)
PA	CY075497	H1N1	10/07/2009	USA	A/Boston/584/2009(H1N1)
PA	KU922539	H1N1	10/07/2009	India	A/Kerala/RGCBA923/2009
PA	CY128272	H1N1	10/08/2009	Viet Nam	A/Viet Nam/13032060/2009
PA	JN187248	H1N1	10/08/2009	Taiwan	A/Taiwan/7418/2009
PA	CY128264	H1N1	10/08/2009	Viet Nam	A/Viet Nam/13032059/2009
PA	KT180902	H1N1	10/08/2009	Singapore	A/Singapore/DMS42/2009
PA	CY057595	H1N1	10/09/2009	USA	A/Wisconsin/629-D02090/2009(H1N1)
PA	CY057603	H1N1	10/09/2009	USA	A/Wisconsin/629-D01080/2009(H1N1)
PA	CY057587	H1N1	10/09/2009	USA	A/Wisconsin/629-D01749/2009(H1N1)
PA	CY063080	H1N1	10/09/2009	USA	A/California/VRDL88/2009(H1N1)
PA	CY063072	H1N1	10/09/2009	USA	A/California/VRDL87/2009(H1N1)
PA	CY071761	H1N1	10/09/2009	Mexico	A/Mexico City/WR1708T/2009(H1N1)
PA	CY062255	H1N1	10/09/2009	USA	A/California/VRDL86/2009(H1N1)
PA	CY062247	H1N1	10/09/2009	USA	A/California/VRDL85/2009(H1N1)
PA	CY062239	H1N1	10/09/2009	USA	A/California/VRDL84/2009(H1N1)
PA	KP637461	H1N1	10/09/2009	Thailand	A/Thailand/PY08063/2009
PA	JX625605	H1N1	10/09/2009	United Ki	A/Northern Ireland/94940020/2009(
PA	JX625535	H1N1	10/09/2009	United Ki	A/Northern Ireland/94480404/2009
PA	JX625512	H1N1	10/09/2009	United Ki	A/England/1072/2009
PA	CY128280	H1N1	10/09/2009	Viet Nam	A/Viet Nam/13032061/2009

PA	CY089192	H1N1	10/10/2009	USA	A/Boston/583/2009(H1N1)
PA	KC782177	H1N1	10/10/2009	USA	A/Tennessee/18/2009
PA	KC780394	H1N1	10/10/2009	USA	A/New Mexico/06/2009
PA	CY057611	H1N1	10/10/2009	USA	A/Wisconsin/629-D00557/2009(H1N1)
PA	CY096044	H1N1	10/11/2009	China	A/Hubei/99/2009
PA	CY090062	H1N1	10/11/2009	Nicaragua	A/Managua/4606.07/2009
PA	CY095734	H1N1	10/11/2009	China	A/Hubei/100/2009
PA	CY072699	H1N1	10/11/2009	Nicaragua	A/Managua/3013.01/2009(H1N1)
PA	CY083055	H1N1	10/11/2009	USA	A/Colorado/WRAIR0353S/2009
PA	CY095934	H1N1	10/11/2009	China	A/Zhejiang/83/2009
PA	CY095962	H1N1	10/11/2009	China	A/Zhejiang/87/2009
PA	CY095941	H1N1	10/11/2009	China	A/Zhejiang/84/2009
PA	CY123207	H1N1	10/11/2009	Singapore	A/Singapore/GP4000/2009
PA	CY095970	H1N1	10/11/2009	China	A/Zhejiang/88/2009
PA	CY055969	H1N1	10/12/2009	USA	A/San Diego/INS03/2009(H1N1)
PA	CY055953	H1N1	10/12/2009	USA	A/San Diego/INS01/2009(H1N1)
PA	CY055993	H1N1	10/12/2009	USA	A/San Diego/INS06/2009(H1N1)
PA	CY055961	H1N1	10/12/2009	USA	A/San Diego/INS02/2009(H1N1)
PA	CY056217	H1N1	10/12/2009	USA	A/San Diego/INS53/2009(H1N1)
PA	CY055985	H1N1	10/12/2009	USA	A/San Diego/INS05/2009(H1N1)
PA	CY057619	H1N1	10/12/2009	USA	A/Wisconsin/629-S1309/2009(H1N1)
PA	CY056888	H1N1	10/12/2009	USA	A/San Diego/INS54/2009(H1N1)
PA	CY056880	H1N1	10/12/2009	USA	A/San Diego/INS51/2009(H1N1)
PA	CY057627	H1N1	10/12/2009	USA	A/Wisconsin/629-S1313/2009(H1N1)
PA	CY057235	H1N1	10/12/2009	USA	A/New York/5045/2009(H1N1)
PA	CY055977	H1N1	10/12/2009	USA	A/San Diego/INS04/2009(H1N1)
PA	KC261314	H1N1	10/12/2009	Mexico	A/Mexico City/INERPHAC1/2009(H1N1
PA	CY083947	H1N1	10/12/2009	USA	A/San Diego/INS194/2009(H1N1)
PA	HM568063	H1N1	10/12/2009	United Ki	A/England/H094240645/2009
PA	CY100463	H1N1	10/12/2009	Mexico	A/Mexico City/INER2/2009
PA	KT180903	H1N1	10/12/2009	Singapore	A/Singapore/DMS43/2009
PA	KP637277	H1N1	10/12/2009	Thailand	A/Thailand/IY07093/2009
PA	CY066532	H1N1	10/12/2009	USA	A/San Diego/INS193/2009(H1N1)
PA	CY063240	H1N1	10/12/2009	USA	A/Wisconsin/629-D02442/2009(H1N1)
PA	CY063232	H1N1	10/12/2009	USA	A/Wisconsin/629-D00675/2009(H1N1)
PA	CY063248	H1N1	10/12/2009	USA	A/Wisconsin/629-D00830/2009(H1N1)
PA	HM568071	H1N1	10/12/2009	United Ki	A/England/H094220579/2009
PA	CY056209	H1N1	10/12/2009	USA	A/San Diego/INS52/2009(H1N1)
PA	CY056528	H1N1	10/13/2009	USA	A/New York/5130/2009(H1N1)
PA	CY056001	H1N1	10/13/2009	USA	A/San Diego/INS09/2009(H1N1)
PA	CY071609	H1N1	10/13/2009	USA	A/Ft Carson/WR1448P/2009(H1N1)
PA	CY057323	H1N1	10/13/2009	USA	A/New York/5667/2009(H1N1)
PA	CY056520	H1N1	10/13/2009	USA	A/New York/5086/2009(H1N1)
PA	CY056504	H1N1	10/13/2009	USA	A/New York/5079/2009(H1N1)
PA	CY056512	H1N1	10/13/2009	USA	A/New York/5083/2009(H1N1)
PA	CY057339	H1N1	10/13/2009	USA	A/San Diego/INS08/2009(H1N1)
PA	CY056009	H1N1	10/13/2009	USA	A/San Diego/INS10/2009(H1N1)
PA	CY056496	H1N1	10/13/2009	USA	A/New York/5074/2009(H1N1)
PA	CY083835	H1N1	10/13/2009	USA	A/San Diego/INS07/2009(H1N1)
PA	CY090078	H1N1	10/13/2009	Nicaragua	A/Managua/4076.03/2009
PA	CY081531	H1N1	10/14/2009	Cambodia	A/Cambodia/NHRCC00006/2009(H1N1)
PA	CY081547	H1N1	10/14/2009	Cambodia	A/Cambodia/NHRCC00008/2009(H1N1)
PA	CY081499	H1N1	10/14/2009	Cambodia	A/Cambodia/NHRCC00002/2009(H1N1)
PA	CY081523	H1N1	10/14/2009	Cambodia	A/Cambodia/NHRCC00005/2009(H1N1)

PA	CY081507	H1N1	10/14/2009	Cambodia	A/Cambodia/NHRCC00003/2009(H1N1)
PA	CY081539	H1N1	10/14/2009	Cambodia	A/Cambodia/NHRCC00007/2009(H1N1)
PA	CY081515	H1N1	10/14/2009	Cambodia	A/Cambodia/NHRCC00004/2009(H1N1)
PA	CY081491	H1N1	10/14/2009	Cambodia	A/Cambodia/NHRCC00001/2009(H1N1)
PA	CY057643	H1N1	10/14/2009	USA	A/Wisconsin/629-S1350/2009(H1N1)
PA	CY057635	H1N1	10/14/2009	USA	A/Wisconsin/629-S1348/2009(H1N1)
PA	CY056896	H1N1	10/14/2009	USA	A/San Diego/INS55/2009(H1N1)
PA	CY056025	H1N1	10/14/2009	USA	A/San Diego/INS12/2009(H1N1)
PA	CY056017	H1N1	10/14/2009	USA	A/San Diego/INS11/2009(H1N1)
PA	CY090013	H1N1	10/14/2009	USA	A/Wisconsin/629-S1368/2009(H1N1)
PA	CY083494	H1N1	10/14/2009	USA	A/Chicago/WRAIR1691P/2009
PA	CY083478	H1N1	10/14/2009	USA	A/Chicago/WRAIR1690P/2009
PA	CY061232	H1N1	10/14/2009	USA	A/San Diego/INS101/2009(H1N1)
PA	CY061240	H1N1	10/14/2009	USA	A/San Diego/INS102/2009(H1N1)
PA	KT180904	H1N1	10/14/2009	Singapore	A/Singapore/DMS44/2009
PA	CY066540	H1N1	10/15/2009	USA	A/San Diego/INS195/2009(H1N1)
PA	CY081555	H1N1	10/15/2009	Cambodia	A/Cambodia/NHRCC00010/2009(H1N1)
PA	CY056081	H1N1	10/15/2009	USA	A/District of Columbia/INS24/2009
PA	CY063256	H1N1	10/15/2009	USA	A/Wisconsin/629-D00692/2009(H1N1)
PA	CY057243	H1N1	10/15/2009	USA	A/New York/5158/2009(H1N1)
PA	JX625567	H1N1	10/15/2009	United Ki	A/Northern Ireland/1/2009
PA	CY071385	H1N1	10/15/2009	Jordan	A/Amman/WR0060N/2009(H1N1)
PA	CY056904	H1N1	10/15/2009	USA	A/San Diego/INS57/2009(H1N1)
PA	CY071601	H1N1	10/15/2009	USA	A/Ft Carson/WR1446P/2009(H1N1)
PA	CY092925	H1N1	10/15/2009	USA	A/Maryland/NHRC0002/2009
PA	CY107339	H1N1	10/15/2009	United Ki	A/Scotland/Dundee_09V500203/2009
PA	CY107320	H1N1	10/15/2009	United Ki	A/Scotland/Fife_20234/2009
PA	CY107332	H1N1	10/15/2009	United Ki	A/Scotland/Dundee_09V500199/2009
PA	CY080349	H1N1	10/15/2009	Cambodia	A/Cambodia/NHRCC00011/2009(H1N1)
PA	CY082989	H1N1	10/15/2009	Cambodia	A/Cambodia/NHRCC00009/2009(H1N1)
PA	KC780342	H1N1	10/15/2009	USA	A/New York/102/2009
PA	KC781029	H1N1	10/16/2009	USA	A/North Carolina/43/2009
PA	KC780507	H1N1	10/16/2009	USA	A/North Carolina/46/2009
PA	KC782326	H1N1	10/16/2009	USA	A/North Carolina/46/2009
PA	CY073048	H1N1	10/16/2009	Dominican	A/Santo Domingo/WRAIR1414N/2009(H
PA	CY092933	H1N1	10/16/2009	USA	A/Maryland/NHRC0003/2009
PA	CY056536	H1N1	10/16/2009	USA	A/New York/5141/2009(H1N1)
PA	CY063264	H1N1	10/16/2009	USA	A/Wisconsin/629-D00740/2009(H1N1)
PA	CY056544	H1N1	10/16/2009	USA	A/New York/5150/2009(H1N1)
PA	CY057251	H1N1	10/16/2009	USA	A/New York/5161/2009(H1N1)
PA	CY056648	H1N1	10/16/2009	USA	A/New York/6079/2009(H1N1)
PA	CY063104	H1N1	10/17/2009	USA	A/California/VRDL91/2009(H1N1)
PA	GU198200	H1N1	10/17/2009	China	A/Nanjing/3/2009(H1N1)
PA	CY063120	H1N1	10/17/2009	USA	A/California/VRDL93/2009(H1N1)
PA	CY056552	H1N1	10/17/2009	USA	A/New York/5173/2009(H1N1)
PA	CY058043	H1N1	10/17/2009	USA	A/Texas/46172731/2009(H1N1)
PA	CY107360	H1N1	10/17/2009	United Ki	A/Scotland/Perth_09V500259/2009
PA	CY063096	H1N1	10/17/2009	USA	A/California/VRDL90/2009(H1N1)
PA	CY107353	H1N1	10/17/2009	United Ki	A/Scotland/Perth_09V500257/2009
PA	CY058051	H1N1	10/17/2009	USA	A/Texas/46172734/2009(H1N1)
PA	CY063088	H1N1	10/17/2009	USA	A/California/VRDL89/2009(H1N1)
PA	CY063112	H1N1	10/17/2009	USA	A/California/VRDL92/2009(H1N1)
PA	CY107346	H1N1	10/17/2009	United Ki	A/Scotland/StAndrews_09V500252/20
PA	KC780111	H1N1	10/18/2009	USA	A/New Mexico/07/2009

PA	CY107500	H1N1	10/18/2009	United Ki	A/Scotland/Edinburgh_20204/2009
PA	CY128288	H1N1	10/18/2009	Viet Nam	A/Viet Nam/13032071/2009
PA	CY073040	H1N1	10/18/2009	Jordan	A/Amman/WRAIR1339N/2009(H1N1)
PA	CY058067	H1N1	10/18/2009	USA	A/Texas/46181292/2009(H1N1)
PA	CY057659	H1N1	10/18/2009	USA	A/Wisconsin/629-D00544/2009(H1N1)
PA	CY057651	H1N1	10/18/2009	USA	A/Wisconsin/629-D02342/2009(H1N1)
PA	CY058059	H1N1	10/18/2009	USA	A/Texas/46181235/2009(H1N1)
PA	CY057667	H1N1	10/18/2009	USA	A/Wisconsin/629-D00099/2009(H1N1)
PA	CY058689	H1N1	10/18/2009	USA	A/Texas/46182018/2009(H1N1)
PA	CY083159	H1N1	10/18/2009	Dominican	A/Santo Domingo/WRAIR1404T/2009
PA	CY071553	H1N1	10/18/2009	Jordan	A/Amman/WR1324N/2009(H1N1)
PA	CY075505	H1N1	10/18/2009	USA	A/Boston/591/2009(H1N1)
PA	CY060565	H1N1	10/18/2009	Canada	A/Ontario/296008/2009(H1N1)
PA	CY058697	H1N1	10/19/2009	USA	A/Texas/461917783/2009(H1N1)
PA	CY060573	H1N1	10/19/2009	Canada	A/Ontario/29801/2009(H1N1)
PA	CY063272	H1N1	10/19/2009	USA	A/Wisconsin/629-D02082/2009(H1N1)
PA	CY107502	H1N1	10/19/2009	United Ki	A/Scotland/Livingston_20263/2009
PA	CY107367	H1N1	10/19/2009	United Ki	A/Scotland/Dundee_09V500266/2009
PA	CY107494	H1N1	10/19/2009	United Ki	A/Scotland/Edinburgh_20203/2009
PA	KJ690455	H1N1	10/19/2009	Uganda	A/Uganda/MUWRP-111/2009
PA	CY056065	H1N1	10/19/2009	USA	A/District of Columbia/INS20/2009
PA	CY083644	H1N1	10/19/2009	USA	A/San Diego/INS13/2009
PA	CY083843	H1N1	10/19/2009	USA	A/San Diego/INS14/2009(H1N1)
PA	CY058713	H1N1	10/19/2009	USA	A/Texas/46193311/2009(H1N1)
PA	CY056912	H1N1	10/19/2009	USA	A/San Diego/INS60/2009(H1N1)
PA	CY071593	H1N1	10/19/2009	Turkey	A/Ankara/WR1429T/2009(H1N1)
PA	CY057259	H1N1	10/19/2009	USA	A/New York/5186/2009(H1N1)
PA	CY071641	H1N1	10/19/2009	Taiwan	A/Taipei/WR1489N/2009(H1N1)
PA	JX403977	H1N1	10/19/2009	China	A/Liaoning/1/2009(H1N1)
PA	CY057691	H1N1	10/19/2009	USA	A/Wisconsin/629-S1388/2009(H1N1)
PA	CY057683	H1N1	10/19/2009	USA	A/Wisconsin/629-S1384/2009(H1N1)
PA	CY057675	H1N1	10/19/2009	USA	A/Wisconsin/629-D00132/2009(H1N1)
PA	CY060760	H1N1	10/19/2009	USA	A/Texas/46193632/2009(H1N1)
PA	CY061248	H1N1	10/19/2009	USA	A/San Diego/INS103/2009(H1N1)
PA	CY058705	H1N1	10/19/2009	USA	A/Texas/46192760/2009(H1N1)
PA	CY107508	H1N1	10/19/2009	United Ki	A/Scotland/Edinburgh_20308/2009
PA	CY056864	H1N1	10/20/2009	USA	A/San Diego/INS15/2009(H1N1)
PA	CY057267	H1N1	10/20/2009	USA	A/New York/5196/2009(H1N1)
PA	CY057283	H1N1	10/20/2009	USA	A/New York/5221/2009(H1N1)
PA	CY056033	H1N1	10/20/2009	USA	A/San Diego/INS16/2009(H1N1)
PA	CY056560	H1N1	10/20/2009	USA	A/New York/5199/2009(H1N1)
PA	CY056568	H1N1	10/20/2009	USA	A/New York/5227/2009(H1N1)
PA	CY061256	H1N1	10/20/2009	USA	A/San Diego/INS104/2009(H1N1)
PA	CY057275	H1N1	10/20/2009	USA	A/New York/5217/2009(H1N1)
PA	CY073071	H1N1	10/20/2009	Turkey	A/Ankara/WRAIR1426N/2009(H1N1)
PA	CY060768	H1N1	10/20/2009	USA	A/Texas/46201823/2009(H1N1)
PA	CY107514	H1N1	10/20/2009	United Ki	A/Scotland/Edinburgh_20342/2009
PA	CY107519	H1N1	10/20/2009	United Ki	A/Scotland/Edinburgh_20665/2009
PA	CY107374	H1N1	10/20/2009	United Ki	A/Scotland/Dundee_09V500321/2009
PA	HM568047	H1N1	10/20/2009	United Ki	A/England/950/2009
PA	JX625520	H1N1	10/20/2009	United Ki	A/England/94400003/2009
PA	JX625590	H1N1	10/20/2009	United Ki	A/Northern Ireland/94620068/2009(
PA	JX625621	H1N1	10/20/2009	United Ki	A/Northern Ireland/94940033/2009(
PA	CY123808	H1N1	10/20/2009	Singapore	A/Singapore/ON2224/2009

PA	CY066556	H1N1	10/21/2009	USA	A/San Diego/INS198/2009(H1N1)
PA	CY066548	H1N1	10/21/2009	USA	A/San Diego/INS197/2009(H1N1)
PA	CY073079	H1N1	10/21/2009	Turkey	A/Ankara/WRAIR1428T/2009(H1N1)
PA	CY071633	H1N1	10/21/2009	Taiwan	A/Taipei/WR1485T/2009(H1N1)
PA	CY071625	H1N1	10/21/2009	Taiwan	A/Taipei/WR1472T/2009(H1N1)
PA	CY071649	H1N1	10/21/2009	Ecuador	A/Quito/WR1589N/2009(H1N1)
PA	CY057299	H1N1	10/21/2009	USA	A/New York/5276/2009(H1N1)
PA	CY057707	H1N1	10/21/2009	USA	A/Wisconsin/629-S1397/2009(H1N1)
PA	CY056920	H1N1	10/21/2009	USA	A/San Diego/INS61/2009(H1N1)
PA	CY056097	H1N1	10/21/2009	USA	A/District of Columbia/INS27/2009
PA	CY056137	H1N1	10/21/2009	USA	A/San Diego/INS34/2009(H1N1)
PA	CY057699	H1N1	10/21/2009	USA	A/Wisconsin/629-S1398/2009(H1N1)
PA	JX625613	H1N1	10/21/2009	United Ki	A/Northern Ireland/94940023/2009(
PA	HM568095	H1N1	10/21/2009	United Ki	A/England/766/2009
PA	HM568087	H1N1	10/21/2009	United Ki	A/England/H094380096/2009
PA	HM567975	H1N1	10/21/2009	United Ki	A/England/1111/2009
PA	CY083674	H1N1	10/21/2009	USA	A/San Diego/INS62/2009(H1N1)
PA	HM568055	H1N1	10/21/2009	United Ki	A/England/1116/2009
PA	CY060776	H1N1	10/21/2009	USA	A/Texas/46214103/2009(H1N1)
PA	CY060792	H1N1	10/22/2009	USA	A/Texas/46222134/2009(H1N1)
PA	CY057291	H1N1	10/22/2009	USA	A/New York/5271/2009(H1N1)
PA	CY065986	H1N1	10/22/2009	Mongolia	A/Ulaanbaatar/6525/2009(H1N1)
PA	CY057715	H1N1	10/22/2009	USA	A/Wisconsin/629-S1407/2009(H1N1)
PA	CY060784	H1N1	10/22/2009	USA	A/Texas/46221665/2009(H1N1)
PA	CY060800	H1N1	10/22/2009	USA	A/Texas/46223444/2009(H1N1)
PA	CY089208	H1N1	10/22/2009	USA	A/Boston/594/2009(H1N1)
PA	CY089200	H1N1	10/22/2009	USA	A/Boston/593/2009(H1N1)
PA	HM568111	H1N1	10/22/2009	United Ki	A/England/798/2009
PA	CY071457	H1N1	10/22/2009	Mexico	A/Mexico City/WR1090N/2009(H1N1)
PA	CY066564	H1N1	10/22/2009	USA	A/San Diego/INS199/2009(H1N1)
PA	HQ263271	H1N1	10/22/2009	USA	A/Bethesda/NIH106-D0/2009
PA	CY057307	H1N1	10/22/2009	USA	A/New York/5297/2009(H1N1)
PA	CY060808	H1N1	10/22/2009	USA	A/Texas/46223582/2009(H1N1)
PA	CY060816	H1N1	10/22/2009	USA	A/Texas/46224042/2009(H1N1)
PA	CY128296	H1N1	10/23/2009	Viet Nam	A/Viet Nam/13032074/2009
PA	CY071617	H1N1	10/23/2009	USA	A/Ft Carson/WR1449P/2009(H1N1)
PA	CY058305	H1N1	10/23/2009	USA	A/Wisconsin/629-D02018/2009(H1N1)
PA	CY060824	H1N1	10/23/2009	USA	A/Texas/46233104/2009(H1N1)
PA	KC780555	H1N1	10/23/2009	USA	A/West Virginia/12/2009
PA	CY065869	H1N1	10/23/2009	Netherlar	A/Netherlands/1493b/2009
PA	CY057315	H1N1	10/23/2009	USA	A/New York/5447/2009(H1N1)
PA	CY056105	H1N1	10/23/2009	USA	A/District of Columbia/INS28/2009
PA	CY092941	H1N1	10/23/2009	USA	A/Maryland/NHRC0004/2009
PA	CY060581	H1N1	10/24/2009	Canada	A/Ontario/304434/2009(H1N1)
PA	CY060840	H1N1	10/24/2009	USA	A/Texas/46241654/2009(H1N1)
PA	CY060832	H1N1	10/24/2009	USA	A/Texas/46240925/2009(H1N1)
PA	HQ263285	H1N1	10/24/2009	USA	A/Bethesda/NIH107-D0/2009
PA	CY128304	H1N1	10/24/2009	Viet Nam	A/Viet Nam/13032076/2009
PA	CY075513	H1N1	10/24/2009	USA	A/Boston/595/2009(H1N1)
PA	CY092917	H1N1	10/25/2009	USA	A/Maryland/NHRC0001/2009
PA	CY090021	H1N1	10/25/2009	USA	A/Wisconsin/629-D02330/2009(H1N1)
PA	CY075005	H1N1	10/26/2009	Thailand	A/Thailand/CU-H847/2009
PA	HM568103	H1N1	10/26/2009	United Ki	A/England/797/2009
PA	JQ612498	H1N1	10/26/2009	Hungary	A/Hungary/40/2009

PA	KC780051	H1N1	10/26/2009	USA	A/Washington/75/2009
PA	CY123816	H1N1	10/26/2009	Singapore	A/Singapore/ON2250/2009
PA	CY056145	H1N1	10/26/2009	USA	A/San Diego/INS35/2009(H1N1)
PA	CY056113	H1N1	10/26/2009	USA	A/District of Columbia/INS29/2009
PA	CY056089	H1N1	10/26/2009	USA	A/District of Columbia/INS25/2009
PA	CY056185	H1N1	10/26/2009	USA	A/District of Columbia/INS43/2009
PA	CY056129	H1N1	10/26/2009	USA	A/District of Columbia/INS33/2009
PA	CY056041	H1N1	10/26/2009	USA	A/District of Columbia/INS17/2009
PA	CY056928	H1N1	10/26/2009	USA	A/San Diego/INS63/2009(H1N1)
PA	CY058321	H1N1	10/26/2009	USA	A/Wisconsin/629-S1414/2009(H1N1)
PA	CY058329	H1N1	10/26/2009	USA	A/Wisconsin/629-S1416/2009(H1N1)
PA	CY058313	H1N1	10/26/2009	USA	A/Wisconsin/629-S1417/2009(H1N1)
PA	CY064769	H1N1	10/26/2009	USA	A/District of Columbia/INS32/2009
PA	CY067640	H1N1	10/27/2009	Norway	A/Oslo/INS219/2009(H1N1)
PA	CY058641	H1N1	10/27/2009	USA	A/Wisconsin/629-S1425/2009(H1N1)
PA	CY058633	H1N1	10/27/2009	USA	A/Wisconsin/629-S1431/2009(H1N1)
PA	KC780721	H1N1	10/27/2009	USA	A/Rhode Island/18/2009
PA	CY056121	H1N1	10/27/2009	USA	A/District of Columbia/INS31/2009
PA	JN187249	H1N1	10/27/2009	Taiwan	A/Taiwan/6200/2009
PA	KT180905	H1N1	10/27/2009	Singapore	A/Singapore/DMS46/2009
PA	CY056057	H1N1	10/27/2009	USA	A/District of Columbia/INS19/2009
PA	CY060589	H1N1	10/27/2009	Canada	A/Ontario/305139/2009(H1N1)
PA	CY058625	H1N1	10/27/2009	USA	A/Wisconsin/629-S1433/2009(H1N1)
PA	CY123229	H1N1	10/27/2009	Singapore	A/Singapore/GP4148/2009
PA	CY060597	H1N1	10/27/2009	Canada	A/Ontario/305598/2009(H1N1)
PA	CY071425	H1N1	10/28/2009	Turkmenis	A/Ashgabat/WR0865N/2009(H1N1)
PA	KY925771	H1N1	10/28/2009	Brazil	A/Porto Alegre/LACENRS-3434/2009
PA	CY056936	H1N1	10/28/2009	USA	A/San Diego/INS65/2009(H1N1)
PA	JN187250	H1N1	10/28/2009	Taiwan	A/Taiwan/7873/2009
PA	JX625551	H1N1	10/28/2009	United Ki	A/England/1397/2009
PA	CY066820	H1N1	10/28/2009	USA	A/San Diego/INS218/2009
PA	CY066828	H1N1	10/28/2009	Belgium	A/Antwerp/INS221/2009(H1N1)
PA	CY060605	H1N1	10/28/2009	Canada	A/Ontario/308054/2009(H1N1)
PA	CY056049	H1N1	10/28/2009	USA	A/District of Columbia/INS18/2009
PA	HM568079	H1N1	10/28/2009	United Ki	A/England/1213/2009
PA	AB704461	H1N1	10/29/2009	Japan	A/Tochigi/350/2009
PA	CY128312	H1N1	10/29/2009	Viet Nam	A/Viet Nam/13032079/2009
PA	CY075521	H1N1	10/29/2009	USA	A/Boston/602/2009(H1N1)
PA	CY083652	H1N1	10/29/2009	USA	A/District of Columbia/INS22/2009
PA	CY063128	H1N1	10/29/2009	USA	A/California/VRDL96/2009(H1N1)
PA	CY066196	H1N1	10/29/2009	USA	A/California/VRDL94/2009(H1N1)
PA	CY061272	H1N1	10/29/2009	Belgium	A/Brussels/INS106/2009(H1N1)
PA	CY060613	H1N1	10/29/2009	Canada	A/Ontario/309862/2009(H1N1)
PA	CY063144	H1N1	10/29/2009	USA	A/California/VRDL98/2009(H1N1)
PA	CY063136	H1N1	10/29/2009	USA	A/California/VRDL97/2009(H1N1)
PA	CY056944	H1N1	10/30/2009	Belgium	A/Brussels/INS71/2009(H1N1)
PA	CY083851	H1N1	10/30/2009	USA	A/District of Columbia/INS23/2009
PA	JN207840	H1N1	10/30/2009	China	A/Zhejiang/89w/2009(H1N1)
PA	CY060621	H1N1	10/30/2009	Canada	A/Ontario/313762/2009(H1N1)
PA	CY107526	H1N1	10/30/2009	United Ki	A/Scotland/Edinburgh_21027/2009
PA	CY066732	H1N1	10/30/2009	Belgium	A/Brussels/INS206/2009(H1N1)
PA	CY056073	H1N1	10/30/2009	USA	A/District of Columbia/INS21/2009
PA	CY083758	H1N1	10/30/2009	Belgium	A/Brussels/INS205/2009(H1N1)
PA	JQ173102	H1N1	10/30/2009	China	A/Nanjing/NJU-108/2009

PA	CY128320	H1N1	10/31/2009	Viet Nam	A/Viet Nam/13032088/2009
PA	CY073117	H1N1	10/31/2009	Russia	A/Moscow/WRAIR1632N/2009(H1N1)
PA	CY058657	H1N1	10/31/2009	USA	A/Wisconsin/629-D02002/2009(H1N1)
PA	CY058665	H1N1	10/31/2009	USA	A/Wisconsin/629-D01913/2009(H1N1)
PA	CY058649	H1N1	10/31/2009	USA	A/Wisconsin/629-D02329/2009(H1N1)
PA	CY065813	H1N1	10/31/2009	Netherlar	A/Netherlands/1715b/2009
PA	CY095919	H1N1	11/2009	China	A/Zhejiang/81/2009
PA	CY095869	H1N1	11/2009	China	A/Hubei/75/2009
PA	CY053337	H1N1	11/2009	China	A/Beijing/718/2009(H1N1)
PA	CY053347	H1N1	11/2009	China	A/Beijing/720/2009(H1N1)
PA	CY095927	H1N1	11/2009	China	A/Zhejiang/82/2009
PA	CY095911	H1N1	11/2009	China	A/Zhejiang/80/2009
PA	CY095861	H1N1	11/2009	China	A/Hubei/74/2009
PA	CY095877	H1N1	11/2009	China	A/Hubei/76/2009
PA	CY095853	H1N1	11/2009	China	A/Hubei/73/2009
PA	CY095898	H1N1	11/2009	China	A/Zhejiang/79/2009
PA	CY053727	H1N1	11/01/2009	Russia	A/Russia/74/2009(H1N1)
PA	CY053751	H1N1	11/01/2009	Russia	A/Russia/200/2009(H1N1)
PA	CY053735	H1N1	11/01/2009	Russia	A/Russia/178/2009(H1N1)
PA	CY053743	H1N1	11/01/2009	Russia	A/Russia/190/2009(H1N1)
PA	CY054637	H1N1	11/01/2009	Russia	A/Russia/12/2009(H1N1)
PA	CY054653	H1N1	11/01/2009	Russia	A/Russia/61/2009(H1N1)
PA	CY053415	H1N1	11/01/2009	Russia	A/Russia/149/2009(H1N1)
PA	CY054661	H1N1	11/01/2009	Russia	A/Russia/100/2009(H1N1)
PA	CY054629	H1N1	11/01/2009	Russia	A/Russia/4/2009(H1N1)
PA	CY053680	H1N1	11/01/2009	Russia	A/Russia/14/2009(H1N1)
PA	CY053688	H1N1	11/01/2009	Russia	A/Russia/191/2009(H1N1)
PA	CY054645	H1N1	11/01/2009	Russia	A/Russia/19/2009(H1N1)
PA	JX625575	H1N1	11/01/2009	United Ki	A/England/94580011/2009
PA	KT180906	H1N1	11/01/2009	Singapore	A/Singapore/DMS47/2009
PA	CY107719	H1N1	11/01/2009	United Ki	A/Scotland/Glasgow_444590/2009
PA	CY107704	H1N1	11/01/2009	United Ki	A/Scotland/Glasgow_443734/2009
PA	CY107726	H1N1	11/01/2009	United Ki	A/Scotland/Glasgow_444809/2009
PA	CY058673	H1N1	11/01/2009	USA	A/Wisconsin/629-D00193/2009(H1N1)
PA	CY107711	H1N1	11/01/2009	United Ki	A/Scotland/Glasgow_444125/2009
PA	CY107699	H1N1	11/01/2009	United Ki	A/Scotland/Glasgow_443726/2009
PA	CY107388	H1N1	11/02/2009	United Ki	A/Scotland/StAndrews_09V500630/20
PA	CY107381	H1N1	11/02/2009	United Ki	A/Scotland/Dundee_09V500624/2009
PA	KC780863	H1N1	11/02/2009	USA	A/Wyoming/16/2009
PA	CY123237	H1N1	11/02/2009	Singapore	A/Singapore/GP4218/2009
PA	CY058681	H1N1	11/02/2009	USA	A/Wisconsin/629-D00938/2009(H1N1)
PA	CY056624	H1N1	11/02/2009	USA	A/New York/5988/2009(H1N1)
PA	CY060645	H1N1	11/02/2009	Canada	A/Ontario/314603/2009(H1N1)
PA	CY060669	H1N1	11/02/2009	Canada	A/Ontario/315047/2009(H1N1)
PA	CY060629	H1N1	11/02/2009	Canada	A/Ontario/314095/2009(H1N1)
PA	CY060848	H1N1	11/02/2009	USA	A/Texas/JMS356/2009(H1N1)
PA	CY058273	H1N1	11/02/2009	USA	A/New York/5620/2009(H1N1)
PA	CY075537	H1N1	11/02/2009	USA	A/Boston/612/2009(H1N1)
PA	CY075529	H1N1	11/02/2009	USA	A/Boston/606/2009(H1N1)
PA	HM189311	H1N1	11/02/2009	Russia	A/Chita/CRIE-8/2009
PA	CY066572	H1N1	11/02/2009	USA	A/San Diego/INS201/2009(H1N1)
PA	CY063280	H1N1	11/02/2009	USA	A/Wisconsin/629-D00888/2009(H1N1)
PA	JF714141	H1N1	11/02/2009	South Kor	A/Korea/NAP-1/2009(H1N1)
PA	CY083666	H1N1	11/02/2009	USA	A/San Diego/INS41/2009(H1N1)

PA	CY066884	H1N1	11/02/2009	USA	A/District of Columbia/INS229/200
PA	CY060701	H1N1	11/03/2009	Canada	A/Ontario/315613/2009(H1N1)
PA	CY060685	H1N1	11/03/2009	Canada	A/Ontario/315181/2009(H1N1)
PA	CY066836	H1N1	11/03/2009	Spain	A/Madrid/INS222/2009
PA	CY075657	H1N1	11/03/2009	USA	A/Boston/617/2009(H1N1)
PA	GU931810	H1N1	11/03/2009	Hong Kong	A/Hong Kong/415742M/2009(H1N1)
PA	HM100233	H1N1	11/03/2009	Hong Kong	A/Hong Kong/415742Md/2009(H1N1)
PA	CY089216	H1N1	11/03/2009	USA	A/Boston/618/2009
PA	CY083247	H1N1	11/03/2009	Russia	A/Moscow/WRAIR1627T/2009(H1N1)
PA	CY060637	H1N1	11/03/2009	Canada	A/Ontario/314137/2009(H1N1)
PA	CY062648	H1N1	11/03/2009	USA	A/District of Columbia/INS45/2009
PA	CY060693	H1N1	11/03/2009	Canada	A/Ontario/315187/2009(H1N1)
PA	CY056584	H1N1	11/03/2009	USA	A/New York/5782/2009(H1N1)
PA	CY056872	H1N1	11/03/2009	USA	A/District of Columbia/INS44/2009
PA	CY071777	H1N1	11/03/2009	Mexico	A/Mexico City/WR1762T/2009(H1N1)
PA	CY117626	H1N1	11/03/2009	Mexico	A/Mexico/UASLP-021/2009
PA	CY056225	H1N1	11/03/2009	USA	A/San Diego/INS68/2009(H1N1)
PA	KJ690525	H1N1	11/03/2009	Uganda	A/Uganda/MUWRP-137/2009
PA	CY100471	H1N1	11/03/2009	Mexico	A/Mexico City/INER12/2009
PA	CY060653	H1N1	11/03/2009	Canada	A/Ontario/315003/2009(H1N1)
PA	CY056193	H1N1	11/03/2009	USA	A/District of Columbia/INS46/2009
PA	CY066740	H1N1	11/03/2009	Belgium	A/Brussels/INS208/2009(H1N1)
PA	CY066748	H1N1	11/04/2009	Belgium	A/Brussels/INS209/2009(H1N1)
PA	CY060856	H1N1	11/04/2009	USA	A/Texas/JMS358/2009(H1N1)
PA	CY062688	H1N1	11/04/2009	USA	A/District of Columbia/INS114/200
PA	CY066580	H1N1	11/04/2009	USA	A/San Diego/INS202/2009(H1N1)
PA	CY066588	H1N1	11/04/2009	USA	A/San Diego/INS203/2009(H1N1)
PA	CY073095	H1N1	11/04/2009	Turkey	A/Ankara/WRAIR1440T/2009(H1N1)
PA	CY061264	H1N1	11/04/2009	USA	A/San Diego/INS105/2009(H1N1)
PA	CY060709	H1N1	11/04/2009	Canada	A/Ontario/315637/2009(H1N1)
PA	CY060677	H1N1	11/04/2009	Canada	A/Ontario/315107/2009(H1N1)
PA	CY060661	H1N1	11/04/2009	Canada	A/Ontario/315015/2009(H1N1)
PA	CY056632	H1N1	11/04/2009	USA	A/New York/6018/2009(H1N1)
PA	CY056161	H1N1	11/04/2009	USA	A/Pensacola/INS38/2009(H1N1)
PA	CY056153	H1N1	11/04/2009	USA	A/Pensacola/INS37/2009(H1N1)
PA	CY056177	H1N1	11/04/2009	USA	A/San Diego/INS42/2009(H1N1)
PA	CY056201	H1N1	11/04/2009	USA	A/San Diego/INS48/2009(H1N1)
PA	KT180907	H1N1	11/04/2009	Singapore	A/Singapore/DMS48/2009
PA	JX625583	H1N1	11/04/2009	United Ki	A/England/94620043/2009
PA	KC780467	H1N1	11/04/2009	USA	A/Florida/25/2009
PA	CY128440	H1N1	11/05/2009	Viet Nam	A/Viet Nam/001-1424/2009
PA	CY117640	H1N1	11/05/2009	Mexico	A/Mexico/UASLP-023/2009
PA	CY107402	H1N1	11/05/2009	United Ki	A/Scotland/Dundee_09V500800/2009
PA	CY107395	H1N1	11/05/2009	United Ki	A/Scotland/Dundee_09V500795/2009
PA	CY083859	H1N1	11/05/2009	USA	A/Pensacola/INS39/2009(H1N1)
PA	CY083726	H1N1	11/05/2009	USA	A/District of Columbia/INS115/200
PA	CY083875	H1N1	11/05/2009	USA	A/San Diego/INS49/2009(H1N1)
PA	CY066900	H1N1	11/05/2009	USA	A/District of Columbia/INS231/200
PA	CY075665	H1N1	11/05/2009	USA	A/Boston/628/2009(H1N1)
PA	CY066204	H1N1	11/05/2009	USA	A/California/VRDL103/2009(H1N1)
PA	CY065821	H1N1	11/05/2009	Netherlar	A/Netherlands/2442b/2009
PA	CY066596	H1N1	11/05/2009	USA	A/San Diego/INS204/2009(H1N1)
PA	CY063168	H1N1	11/05/2009	USA	A/California/VRDL101/2009(H1N1)
PA	CY063160	H1N1	11/05/2009	USA	A/California/VRDL100/2009(H1N1)

PA	CY063176	H1N1	11/05/2009	USA	A/California/VRDL102/2009(H1N1)
PA	CY063152	H1N1	11/05/2009	USA	A/California/VRDL99/2009(H1N1)
PA	HQ263278	H1N1	11/05/2009	USA	A/Bethesda/NIH106-D14/2009
PA	CY057739	H1N1	11/05/2009	USA	A/Wisconsin/629-D01140/2009(H1N1)
PA	CY057755	H1N1	11/05/2009	USA	A/Wisconsin/629-D01570/2009(H1N1)
PA	CY057763	H1N1	11/05/2009	USA	A/Wisconsin/629-D02370/2009(H1N1)
PA	CY057731	H1N1	11/05/2009	USA	A/Wisconsin/629-D01915/2009(H1N1)
PA	CY057723	H1N1	11/05/2009	USA	A/Wisconsin/629-D02317/2009(H1N1)
PA	CY057747	H1N1	11/05/2009	USA	A/Wisconsin/629-D00151/2009(H1N1)
PA	CY057771	H1N1	11/05/2009	USA	A/Wisconsin/629-D01346/2009(H1N1)
PA	CY056233	H1N1	11/05/2009	USA	A/San Diego/INS69/2009(H1N1)
PA	CY054677	H1N1	11/05/2009	Russia	A/Russia/180/2009(H1N1)
PA	CY056241	H1N1	11/05/2009	USA	A/San Diego/INS70/2009(H1N1)
PA	CY123832	H1N1	11/06/2009	Singapore	A/Singapore/ON2269/2009
PA	CY073032	H1N1	11/06/2009	El Salvac	A/San Salvador/WRAIR1109N/2009(H1
PA	CY083867	H1N1	11/06/2009	USA	A/District of Columbia/INS47/2009
PA	KT180908	H1N1	11/06/2009	Singapore	A/Singapore/DMS49/2009
PA	CY056600	H1N1	11/06/2009	USA	A/New York/5916/2009(H1N1)
PA	KC261315	H1N1	11/06/2009	Mexico	A/Mexico City/INERPHAC2/2009(H1N1
PA	KC780752	H1N1	11/06/2009	USA	A/Illinois/15/2009
PA	CY060880	H1N1	11/07/2009	USA	A/Texas/JMS362/2009(H1N1)
PA	KC782169	H1N1	11/07/2009	USA	A/New Mexico/16/2009
PA	CY089232	H1N1	11/07/2009	USA	A/Boston/632/2009
PA	CY089224	H1N1	11/07/2009	USA	A/Boston/630/2009
PA	CY060864	H1N1	11/07/2009	USA	A/Texas/JMS359/2009(H1N1)
PA	CY060872	H1N1	11/07/2009	USA	A/Texas/JMS361/2009(H1N1)
PA	CY065837	H1N1	11/07/2009	Netherlar	A/Netherlands/2457b/2009
PA	CY087205	H1N1	11/07/2009	USA	A/Texas/JMS360/2009
PA	CY057779	H1N1	11/07/2009	USA	A/Wisconsin/629-D00802/2009(H1N1)
PA	CY057803	H1N1	11/08/2009	USA	A/Wisconsin/629-D01466/2009(H1N1)
PA	CY057811	H1N1	11/08/2009	USA	A/Wisconsin/629-D02392/2009(H1N1)
PA	CY061559	H1N1	11/08/2009	USA	A/Texas/JMS366/2009(H1N1)
PA	CY060717	H1N1	11/08/2009	Canada	A/Ontario/320266/2009(H1N1)
PA	CY089256	H1N1	11/08/2009	USA	A/Boston/637/2009
PA	CY057795	H1N1	11/08/2009	USA	A/Wisconsin/629-D01369/2009(H1N1)
PA	CY060904	H1N1	11/08/2009	USA	A/Texas/JMS365/2009(H1N1)
PA	CY060896	H1N1	11/08/2009	USA	A/Texas/JMS364/2009(H1N1)
PA	CY056656	H1N1	11/08/2009	USA	A/New York/6110/2009(H1N1)
PA	CY057787	H1N1	11/08/2009	USA	A/Wisconsin/629-D01014/2009(H1N1)
PA	KC782064	H1N1	11/08/2009	USA	A/Wisconsin/55/2009
PA	CY060888	H1N1	11/08/2009	USA	A/Texas/JMS363/2009(H1N1)
PA	KC780798	H1N1	11/09/2009	USA	A/Maryland/31/2009
PA	KC782243	H1N1	11/09/2009	USA	A/Minnesota/40/2009
PA	KC780385	H1N1	11/09/2009	USA	A/Washington/62/2009
PA	KC780205	H1N1	11/09/2009	USA	A/Maine/13/2009
PA	KC781618	H1N1	11/09/2009	USA	A/Idaho/14/2009
PA	CY083891	H1N1	11/09/2009	USA	A/San Diego/INS72/2009(H1N1)
PA	CY083883	H1N1	11/09/2009	USA	A/San Diego/INS50/2009(H1N1)
PA	CY083899	H1N1	11/09/2009	USA	A/San Diego/INS73/2009(H1N1)
PA	CY089248	H1N1	11/09/2009	USA	A/Boston/635/2009
PA	CY089240	H1N1	11/09/2009	USA	A/Boston/633/2009
PA	CY071769	H1N1	11/09/2009	Mexico	A/Mexico City/WR1747N/2009(H1N1)
PA	CY075545	H1N1	11/09/2009	USA	A/Boston/634/2009(H1N1)
PA	CY075013	H1N1	11/09/2009	Thailand	A/Thailand/CU-H910/2009

PA	CY066788	H1N1	11/09/2009	USA	A/San Diego/INS214/2009
PA	CY066988	H1N1	11/09/2009	Belgium	A/Brussels/INS243/2009(H1N1)
PA	CY056608	H1N1	11/09/2009	USA	A/New York/5931/2009(H1N1)
PA	CY056592	H1N1	11/09/2009	USA	A/New York/5886/2009(H1N1)
PA	CY056169	H1N1	11/09/2009	USA	A/Pensacola/INS40/2009(H1N1)
PA	CY056640	H1N1	11/09/2009	USA	A/New York/6019/2009(H1N1)
PA	AB704493	H1N1	11/09/2009	Japan	A/Yamagata/473/2009
PA	CY056616	H1N1	11/09/2009	USA	A/New York/5976/2009(H1N1)
PA	CY057331	H1N1	11/09/2009	USA	A/New York/6072/2009(H1N1)
PA	CY066756	H1N1	11/10/2009	USA	A/Pensacola/INS210/2009
PA	CY083550	H1N1	11/10/2009	Hungary	A/Budapest/WRAIR2393T/2009
PA	CY066796	H1N1	11/10/2009	USA	A/San Diego/INS215/2009
PA	CY107410	H1N1	11/10/2009	United Ki	A/Scotland/Perth_09V501016/2009
PA	CY062792	H1N1	11/10/2009	Denmark	A/Hvidovre/INS139/2009(H1N1)
PA	CY100479	H1N1	11/10/2009	Mexico	A/Mexico City/INER13/2009
PA	CY128328	H1N1	11/10/2009	Viet Nam	A/Viet Nam/13032093/2009
PA	CY053626	H1N1	11/10/2009	Russia	A/Russia/165/2009(H1N1)
PA	CY073141	H1N1	11/10/2009	Mexico	A/Mexico City/WRAIR1781T/2009(H1N
PA	HQ652619	H1N1	11/10/2009	China	A/Jiangsu/S62/2009
PA	HQ652618	H1N1	11/10/2009	China	A/Jiangsu/S61/2009
PA	CY083542	H1N1	11/10/2009	Hungary	A/Budapest/WRAIR2393N/2009
PA	CY063587	H1N1	11/10/2009	Norway	A/Oslo/INS110/2009(H1N1)
PA	CY107210	H1N1	11/10/2009	United Ki	A/Scotland/Aberdeen_1/2009
PA	HM189351	H1N1	11/10/2009	Russia	A/Lipetsk/CRIE-BVV/2009
PA	CY065853	H1N1	11/10/2009	Netherlar	A/Netherlands/1324/2009(H1N1)
PA	CY107217	H1N1	11/10/2009	United Ki	A/Scotland/Inverness_10/2009
PA	CY066764	H1N1	11/10/2009	USA	A/Pensacola/INS211/2009
PA	CY066772	H1N1	11/10/2009	USA	A/Pensacola/INS212/2009
PA	CY063288	H1N1	11/11/2009	USA	A/Wisconsin/629-D02008/2009(H1N1)
PA	CY062816	H1N1	11/11/2009	Denmark	A/Odense/INS143/2009(H1N1)
PA	GU371265	H1N1	11/11/2009	Russia	A/Bryansk/IIV2971/2009(H1N1)
PA	CY107224	H1N1	11/11/2009	United Ki	A/Scotland/Aberdeen_11/2009
PA	CY107280	H1N1	11/11/2009	United Ki	A/Scotland/Aberdeen_3/2009
PA	CY107287	H1N1	11/11/2009	United Ki	A/Scotland/Aberdeen_4/2009
PA	CY107273	H1N1	11/11/2009	United Ki	A/Scotland/Aberdeen_2/2009
PA	CY107301	H1N1	11/11/2009	United Ki	A/Scotland/Aberdeen_6/2009
PA	CY084443	H1N1	11/11/2009	USA	A/New York/6064/2009
PA	JN032405	H1N1	11/11/2009	China	A/Changchun/01/2009
PA	CY107307	H1N1	11/11/2009	United Ki	A/Scotland/Aberdeen_7/2009
PA	CY066844	H1N1	11/11/2009	Spain	A/Madrid/INS223/2009
PA	CY089264	H1N1	11/12/2009	USA	A/Boston/650/2009
PA	CY066860	H1N1	11/12/2009	USA	A/District of Columbia/INS226/200
PA	HM173604	H1N1	11/12/2009	Russia	A/Blagovechensk/01/2009(H1N1)
PA	CY067648	H1N1	11/12/2009	Spain	A/Madrid/INS224/2009(H1N1)
PA	CY107266	H1N1	11/12/2009	United Ki	A/Scotland/Inverness_18/2009
PA	KT180909	H1N1	11/12/2009	Singapore	A/Singapore/DMS50/2009
PA	KC781072	H1N1	11/12/2009	USA	A/Oklahoma/05/2009
PA	CY054669	H1N1	11/12/2009	Russia	A/Russia/171/2009(H1N1)
PA	CY107259	H1N1	11/12/2009	United Ki	A/Scotland/Aberdeen_16/2009
PA	CY061567	H1N1	11/12/2009	USA	A/Texas/JMS367/2009(H1N1)
PA	CY061280	H1N1	11/12/2009	USA	A/Pensacola/INS107/2009(H1N1)
PA	CY123840	H1N1	11/12/2009	Singapore	A/Singapore/ON2291/2009
PA	CY107231	H1N1	11/12/2009	United Ki	A/Scotland/Aberdeen_12/2009
PA	CY063296	H1N1	11/12/2009	USA	A/Wisconsin/629-D00292/2009(H1N1)

PA	JQ714155	H1N1	11/12/2009	China	A/Tianjinheping/SWL313/2009
PA	CY057347	H1N1	11/12/2009	USA	A/San Diego/INS75/2009(H1N1)
PA	CY123249	H1N1	11/12/2009	Singapore	A/Singapore/GP4309/2009
PA	CY075553	H1N1	11/12/2009	USA	A/Boston/648/2009(H1N1)
PA	CY066804	H1N1	11/12/2009	USA	A/San Diego/INS216/2009
PA	CY066812	H1N1	11/12/2009	USA	A/San Diego/INS217/2009
PA	CY062656	H1N1	11/12/2009	USA	A/San Diego/INS74/2009(H1N1)
PA	CY066868	H1N1	11/12/2009	USA	A/District of Columbia/INS227/200
PA	CY107252	H1N1	11/12/2009	United Ki	A/Scotland/Inverness_15/2009
PA	CY107238	H1N1	11/12/2009	United Ki	A/Scotland/Aberdeen_13/2009
PA	CY060912	H1N1	11/13/2009	USA	A/Texas/JMS369/2009(H1N1)
PA	CY062680	H1N1	11/13/2009	Spain	A/Madrid/INS113/2009(H1N1)
PA	CY067020	H1N1	11/13/2009	Germany	A/Bochum/INS249/2009(H1N1)
PA	CY066876	H1N1	11/13/2009	USA	A/District of Columbia/INS228/200
PA	CY066852	H1N1	11/13/2009	Spain	A/Madrid/INS225/2009
PA	CY067028	H1N1	11/13/2009	Germany	A/Bochum/INS250/2009(H1N1)
PA	CY071801	H1N1	11/13/2009	Haiti	A/Port Au Prince/WR2294T/2009(H1N
PA	CY073486	H1N1	11/13/2009	Germany	A/Bochum/INS248/2009(H1N1)
PA	HM773209	H1N1	11/13/2009	China	A/Jiangsu/2/2009(H1N1)
PA	CY075569	H1N1	11/13/2009	USA	A/Boston/655/2009(H1N1)
PA	CY075561	H1N1	11/13/2009	USA	A/Boston/653/2009(H1N1)
PA	CY075577	H1N1	11/13/2009	USA	A/Boston/658/2009(H1N1)
PA	CY073087	H1N1	11/13/2009	Turkey	A/Ankara/WRAIR1435T/2009
PA	JX625598	H1N1	11/13/2009	United Ki	A/England/94720002/2009
PA	CY087213	H1N1	11/13/2009	USA	A/Texas/JMS368/2009
PA	CY083718	H1N1	11/13/2009	Spain	A/Madrid/INS112/2009(H1N1)
PA	KC780774	H1N1	11/13/2009	USA	A/Arkansas/09/2009
PA	CY128376	H1N1	11/13/2009	Viet Nam	A/Viet Nam/11032001/2009
PA	CY107294	H1N1	11/13/2009	United Ki	A/Scotland/Aberdeen_5/2009
PA	CY107245	H1N1	11/13/2009	United Ki	A/Scotland/Aberdeen_14/2009
PA	CY089272	H1N1	11/14/2009	USA	A/Boston/657/2009
PA	CY128424	H1N1	11/14/2009	Viet Nam	A/Viet Nam/12032001/2009
PA	CY128344	H1N1	11/14/2009	Viet Nam	A/Viet Nam/13032097/2009
PA	CY128336	H1N1	11/14/2009	Viet Nam	A/Viet Nam/13032096/2009
PA	CY057819	H1N1	11/14/2009	USA	A/Wisconsin/629-D01826/2009(H1N1)
PA	GU451253	H1N1	11/14/2009	Russia	A/Tver/IIV2969/2009(H1N1)
PA	CY060920	H1N1	11/14/2009	USA	A/Texas/JMS370/2009(H1N1)
PA	CY060928	H1N1	11/14/2009	USA	A/Texas/JMS371/2009(H1N1)
PA	CY057827	H1N1	11/14/2009	USA	A/Wisconsin/629-D00965/2009(H1N1)
PA	CY075601	H1N1	11/15/2009	USA	A/Boston/672/2009(H1N1)
PA	CY089304	H1N1	11/15/2009	USA	A/Boston/671/2009
PA	KT180910	H1N1	11/15/2009	Singapore	A/Singapore/DMS51/2009
PA	CY128472	H1N1	11/15/2009	Viet Nam	A/Viet Nam/001-1020/2009
PA	CY128352	H1N1	11/15/2009	Viet Nam	A/Viet Nam/13032100/2009
PA	CY057851	H1N1	11/15/2009	USA	A/Wisconsin/629-D02060/2009(H1N1)
PA	CY057867	H1N1	11/15/2009	USA	A/Wisconsin/629-D00936/2009(H1N1)
PA	CY060936	H1N1	11/15/2009	USA	A/Texas/JMS372/2009(H1N1)
PA	CY056688	H1N1	11/15/2009	USA	A/New York/6257/2009(H1N1)
PA	CY057835	H1N1	11/15/2009	USA	A/Wisconsin/629-D00911/2009(H1N1)
PA	CY057859	H1N1	11/15/2009	USA	A/Wisconsin/629-D00295/2009(H1N1)
PA	CY057843	H1N1	11/15/2009	USA	A/Wisconsin/629-D00128/2009(H1N1)
PA	CY066244	H1N1	11/15/2009	USA	A/California/VRDL108/2009(H1N1)
PA	CY066228	H1N1	11/15/2009	USA	A/California/VRDL106/2009(H1N1)
PA	CY066212	H1N1	11/15/2009	USA	A/California/VRDL104/2009(H1N1)

PA	CY066236	H1N1	11/15/2009	USA	A/California/VRDL107/2009(H1N1)
PA	CY066220	H1N1	11/15/2009	USA	A/California/VRDL105/2009(H1N1)
PA	CY065120	H1N1	11/15/2009	Netherlar	A/Netherlands/2464b/2009
PA	CY073063	H1N1	11/15/2009	Turkey	A/Ankara/WRAIR1425T/2009(H1N1)
PA	CY075585	H1N1	11/16/2009	USA	A/Boston/663/2009(H1N1)
PA	CY075593	H1N1	11/16/2009	USA	A/Boston/666/2009(H1N1)
PA	CY089296	H1N1	11/16/2009	USA	A/Boston/670/2009
PA	CY089280	H1N1	11/16/2009	USA	A/Boston/665/2009
PA	CY089288	H1N1	11/16/2009	USA	A/Boston/667/2009
PA	CY089312	H1N1	11/16/2009	USA	A/Boston/673/2009
PA	KP637405	H1N1	11/16/2009	Thailand	A/Thailand/MR08303/2009
PA	AB704429	H1N1	11/16/2009	Japan	A/Gunma/263/2009
PA	CY056672	H1N1	11/16/2009	USA	A/New York/6213/2009(H1N1)
PA	CY057875	H1N1	11/16/2009	USA	A/Wisconsin/629-D01661/2009(H1N1)
PA	CY056680	H1N1	11/16/2009	USA	A/New York/6214/2009(H1N1)
PA	CY056704	H1N1	11/16/2009	USA	A/New York/6293/2009(H1N1)
PA	GU371259	H1N1	11/16/2009	Russia	A/Orenburg/IIV2974/2009(H1N1)
PA	CY058337	H1N1	11/16/2009	USA	A/Wisconsin/629-D01347/2009(H1N1)
PA	CY056664	H1N1	11/16/2009	USA	A/New York/6182/2009(H1N1)
PA	CY065136	H1N1	11/16/2009	Netherlar	A/Netherlands/2143/2009
PA	CY067004	H1N1	11/16/2009	Germany	A/Munich/INS246/2009
PA	HQ698626	H1N1	11/16/2009	China	A/Beijing/16/2009
PA	CY089328	H1N1	11/17/2009	USA	A/Boston/680/2009
PA	CY075609	H1N1	11/17/2009	USA	A/Boston/681/2009(H1N1)
PA	CY089320	H1N1	11/17/2009	USA	A/Boston/678/2009
PA	KP637805	H1N1	11/17/2009	Thailand	A/Thailand/SS08207/2009
PA	CY128052	H1N1	11/17/2009	Viet Nam	A/Viet Nam/11032002/2009
PA	CY056696	H1N1	11/17/2009	USA	A/New York/6292/2009(H1N1)
PA	CY062672	H1N1	11/17/2009	USA	A/Pensacola/INS109/2009(H1N1)
PA	CY060725	H1N1	11/17/2009	Canada	A/Ontario/328474/2009(H1N1)
PA	CY061288	H1N1	11/17/2009	USA	A/Pensacola/INS108/2009(H1N1)
PA	CY058345	H1N1	11/17/2009	USA	A/Wisconsin/629-D00503/2009(H1N1)
PA	CY058353	H1N1	11/17/2009	USA	A/Wisconsin/629-D01582/2009(H1N1)
PA	CY056712	H1N1	11/17/2009	USA	A/New York/6300/2009(H1N1)
PA	CY066724	H1N1	11/17/2009	Spain	A/Madrid/INS295/2009(H1N1)
PA	CY069119	H1N1	11/17/2009	Spain	A/Madrid/INS296/2009(H1N1)
PA	CY123850	H1N1	11/17/2009	Singapore	A/Singapore/ON2313/2009
PA	CY065829	H1N1	11/17/2009	Netherlar	A/Netherlands/2243/2009
PA	CY066780	H1N1	11/17/2009	USA	A/Pensacola/INS213/2009
PA	CY075617	H1N1	11/17/2009	USA	A/Boston/682/2009(H1N1)
PA	CY089336	H1N1	11/17/2009	USA	A/Boston/683/2009
PA	CY073165	H1N1	11/18/2009	Nigeria	A/Lagos/WRAIR1984N/2009(H1N1)
PA	CY073746	H1N1	11/18/2009	Denmark	A/Copenhagen/INS433/2009(H1N1)
PA	CY088734	H1N1	11/18/2009	Netherlar	A/Netherlands/2223b/2009
PA	CY089352	H1N1	11/18/2009	USA	A/Boston/686/2009
PA	CY089344	H1N1	11/18/2009	USA	A/Boston/685/2009
PA	CY128360	H1N1	11/18/2009	Viet Nam	A/Viet Nam/15032003/2009
PA	CY056720	H1N1	11/18/2009	USA	A/New York/6346/2009(H1N1)
PA	GU560013	H1N1	11/18/2009	Russia	A/Tomsk/05/2009(H1N1)
PA	CY058361	H1N1	11/18/2009	USA	A/Wisconsin/629-D02489/2009(H1N1)
PA	CY073173	H1N1	11/18/2009	Nigeria	A/Lagos/WRAIR1984T/2009(H1N1)
PA	CY067656	H1N1	11/18/2009	Belgium	A/Brussels/INS244/2009(H1N1)
PA	CY123259	H1N1	11/18/2009	Singapore	A/Singapore/GP4381/2009
PA	CY066996	H1N1	11/18/2009	Belgium	A/Brussels/INS245/2009(H1N1)

PA	CY073189	H1N1	11/18/2009	Serbia	A/Belgrade/WRAIR2368N/2009(H1N1)
PA	CY073197	H1N1	11/18/2009	Serbia	A/Belgrade/WRAIR2368T/2009(H1N1)
PA	HM567983	H1N1	11/18/2009	United Ki	A/England/869/2009
PA	CY071585	H1N1	11/19/2009	Jordan	A/Amman/WR1335T/2009(H1N1)
PA	CY066940	H1N1	11/19/2009	Denmark	A/Aarhus/INS236/2009(H1N1)
PA	CY100487	H1N1	11/19/2009	Mexico	A/Mexico City/INER14/2009
PA	AB704421	H1N1	11/19/2009	Japan	A/Gunma/262/2009
PA	CY063304	H1N1	11/19/2009	USA	A/Wisconsin/629-D00859/2009(H1N1)
PA	CY063312	H1N1	11/19/2009	USA	A/Wisconsin/629-D01572/2009(H1N1)
PA	CY062744	H1N1	11/19/2009	Germany	A/Bonn/INS127/2009(H1N1)
PA	CY071785	H1N1	11/19/2009	Mexico	A/Mexico City/WR1765N/2009(H1N1)
PA	CY071228	H1N1	11/19/2009	Norway	A/Oslo/INS404/2009(H1N1)
PA	CY066708	H1N1	11/19/2009	Austria	A/Vienna/INS291/2009(H1N1)
PA	CY066636	H1N1	11/19/2009	Germany	A/Bonn/INS277/2009(H1N1)
PA	CY065861	H1N1	11/19/2009	Netherlar	A/Netherlands/2229/2009
PA	CY062752	H1N1	11/19/2009	Spain	A/Madrid/INS129/2009(H1N1)
PA	CY083143	H1N1	11/19/2009	Kyrgyzsta	A/Bishkek/WRAIR0883N/2009
PA	JQ714156	H1N1	11/19/2009	China	A/Tianjintanggu/SWL432/2009
PA	HM754226	H1N1	11/20/2009	China	A/Jiangsu/1/2009(H1N1)
PA	CY062800	H1N1	11/20/2009	Denmark	A/Hvidovre/INS141/2009(H1N1)
PA	CY056952	H1N1	11/20/2009	Denmark	A/Aarhus/INS82/2009(H1N1)
PA	HM230698	H1N1	11/20/2009	China	A/Zhoushan/1/2009(H1N1)
PA	CY071441	H1N1	11/20/2009	Kyrgyzsta	A/Bishkek/WR0882N/2009(H1N1)
PA	HM568031	H1N1	11/20/2009	United Ki	A/England/902/2009
PA	CY066652	H1N1	11/20/2009	Germany	A/Bonn/INS279/2009(H1N1)
PA	CY089360	H1N1	11/20/2009	USA	A/Boston/690/2009
PA	CY066684	H1N1	11/20/2009	Denmark	A/Hvidovre/INS288/2009(H1N1)
PA	HM568015	H1N1	11/20/2009	United Ki	A/England/872/2009
PA	HM567967	H1N1	11/20/2009	United Ki	A/England/876/2009
PA	CY083742	H1N1	11/20/2009	Spain	A/Madrid/INS130/2009(H1N1)
PA	CY066644	H1N1	11/20/2009	Germany	A/Bonn/INS278/2009(H1N1)
PA	CY063603	H1N1	11/20/2009	Germany	A/Bonn/INS128/2009(H1N1)
PA	AB704509	H1N1	11/21/2009	Japan	A/Yamaguchi/217/2009
PA	CY088590	H1N1	11/21/2009	USA	A/Boston/694/2009
PA	CY089368	H1N1	11/21/2009	USA	A/Boston/691/2009
PA	CY067044	H1N1	11/21/2009	Denmark	A/Aarhus/INS252/2009(H1N1)
PA	CY067036	H1N1	11/21/2009	Denmark	A/Aarhus/INS251/2009
PA	CY060944	H1N1	11/21/2009	USA	A/Texas/JMS373/2009(H1N1)
PA	CY067052	H1N1	11/22/2009	Denmark	A/Aarhus/INS253/2009(H1N1)
PA	CY057891	H1N1	11/22/2009	USA	A/Wisconsin/629-D00533/2009(H1N1)
PA	CY057899	H1N1	11/22/2009	USA	A/Wisconsin/629-D00908/2009(H1N1)
PA	CY128368	H1N1	11/22/2009	Viet Nam	A/Viet Nam/15032004/2009
PA	CY084459	H1N1	11/22/2009	USA	A/New York/6418/2009
PA	CY056728	H1N1	11/22/2009	USA	A/New York/6473/2009(H1N1)
PA	GU560021	H1N1	11/22/2009	Russia	A/Tomsk/06/2009(H1N1)
PA	CY065805	H1N1	11/23/2009	Netherlar	A/Netherlands/2445b/2009
PA	CY066908	H1N1	11/23/2009	USA	A/District of Columbia/INS232/200
PA	CY066892	H1N1	11/23/2009	USA	A/District of Columbia/INS230/200
PA	CY066948	H1N1	11/23/2009	Denmark	A/Aarhus/INS237/2009(H1N1)
PA	CY075625	H1N1	11/23/2009	USA	A/Boston/698/2009(H1N1)
PA	CY123267	H1N1	11/23/2009	Singapore	A/Singapore/GP4419/2009
PA	HM568007	H1N1	11/23/2009	United Ki	A/England/854/2009
PA	HM567959	H1N1	11/23/2009	United Ki	A/England/865/2009
PA	HM568023	H1N1	11/23/2009	United Ki	A/England/862/2009

PA	CY083915	H1N1	11/23/2009	Denmark	A/Aalborg/INS132/2009(H1N1)
PA	CY123858	H1N1	11/23/2009	Singapore	A/Singapore/ON2326/2009
PA	CY056848	H1N1	11/23/2009	USA	A/New York/6868/2009(H1N1)
PA	CY056760	H1N1	11/23/2009	USA	A/New York/6663/2009(H1N1)
PA	CY056736	H1N1	11/23/2009	USA	A/New York/6546/2009(H1N1)
PA	CY057907	H1N1	11/23/2009	USA	A/Wisconsin/629-D02448/2009(H1N1)
PA	CY057923	H1N1	11/23/2009	USA	A/Wisconsin/629-D01330/2009(H1N1)
PA	CY058721	H1N1	11/23/2009	USA	A/Texas/JMS375/2009(H1N1)
PA	CY057915	H1N1	11/23/2009	USA	A/Wisconsin/629-D00134/2009(H1N1)
PA	CY072251	H1N1	11/23/2009	Denmark	A/Odense/INS307/2009(H1N1)
PA	CY073157	H1N1	11/23/2009	Nigeria	A/Lagos/WRAIR1982N/2009(H1N1)
PA	CY069151	H1N1	11/23/2009	Germany	A/Frankfurt/INS301/2009(H1N1)
PA	CY066660	H1N1	11/24/2009	Spain	A/Madrid/INS281/2009(H1N1)
PA	CY069127	H1N1	11/24/2009	Spain	A/Madrid/INS298/2009(H1N1)
PA	CY066956	H1N1	11/24/2009	Denmark	A/Aarhus/INS238/2009(H1N1)
PA	CY066916	H1N1	11/24/2009	USA	A/Pensacola/INS233/2009
PA	HM567999	H1N1	11/24/2009	United Ki	A/England/900/2009
PA	CY092443	H1N1	11/24/2009	Australia	A/Sydney/DD3-02/2009(H1N1)
PA	CY084467	H1N1	11/24/2009	USA	A/New York/6537/2009
PA	KT180912	H1N1	11/24/2009	Singapore	A/Singapore/DMS53/2009
PA	KC780704	H1N1	11/24/2009	USA	A/California/41/2009
PA	AB704469	H1N1	11/24/2009	Japan	A/Tochigi/445/2009
PA	CY057931	H1N1	11/24/2009	USA	A/Wisconsin/629-D01190/2009(H1N1)
PA	CY056752	H1N1	11/24/2009	USA	A/New York/6607/2009(H1N1)
PA	CY057939	H1N1	11/24/2009	USA	A/Wisconsin/629-D00968/2009(H1N1)
PA	CY062696	H1N1	11/24/2009	Denmark	A/Aarhus/INS116/2009(H1N1)
PA	CY056784	H1N1	11/24/2009	USA	A/New York/6675/2009(H1N1)
PA	CY058729	H1N1	11/24/2009	USA	A/Texas/JMS376/2009(H1N1)
PA	HQ263292	H1N1	11/24/2009	USA	A/Bethesda/NIH107-D31/2009
PA	CY071252	H1N1	11/24/2009	Spain	A/Madrid/INS407/2009(H1N1)
PA	CY072259	H1N1	11/24/2009	Denmark	A/Odense/INS308/2009(H1N1)
PA	CY066252	H1N1	11/24/2009	USA	A/California/VRDL109/2009(H1N1)
PA	CY066284	H1N1	11/24/2009	USA	A/California/VRDL113/2009(H1N1)
PA	CY066268	H1N1	11/24/2009	USA	A/California/VRDL111/2009(H1N1)
PA	CY066276	H1N1	11/24/2009	USA	A/California/VRDL112/2009(H1N1)
PA	CY066260	H1N1	11/24/2009	USA	A/California/VRDL110/2009(H1N1)
PA	CY066716	H1N1	11/24/2009	Austria	A/Vienna/INS293/2009(H1N1)
PA	CY066980	H1N1	11/24/2009	Denmark	A/Aarhus/INS242/2009(H1N1)
PA	CY066692	H1N1	11/25/2009	Denmark	A/Hvidovre/INS289/2009(H1N1)
PA	HM189319	H1N1	11/25/2009	Russia	A/Arkhangelsk/CRIE-GNY/2009
PA	CY067012	H1N1	11/25/2009	Germany	A/Munich/INS247/2009
PA	CY072227	H1N1	11/25/2009	Germany	A/Frankfurt/INS302/2009(H1N1)
PA	CY123285	H1N1	11/25/2009	Singapore	A/Singapore/GP4539/2009
PA	HM567951	H1N1	11/25/2009	United Ki	A/England/857/2009
PA	HM568039	H1N1	11/25/2009	United Ki	A/England/898/2009
PA	HM567991	H1N1	11/25/2009	United Ki	A/England/859/2009
PA	AB704437	H1N1	11/25/2009	Japan	A/Gunma/267/2009
PA	AB704445	H1N1	11/25/2009	Japan	A/Gunma/287/2009
PA	JQ173163	H1N1	11/25/2009	Finland	A/Helsinki/P18/2009(H1N1)
PA	CY056824	H1N1	11/25/2009	USA	A/New York/6810/2009(H1N1)
PA	GU562455	H1N1	11/25/2009	Russia	A/Perm/01/2009(H1N1)
PA	HM101147	H1N1	11/25/2009	Russia	A/Tver/IIV-183/2009(H1N1)
PA	CY056832	H1N1	11/25/2009	USA	A/New York/6846/2009(H1N1)
PA	CY067188	H1N1	11/25/2009	Austria	A/Vienna/INS294/2009

PA	CY062808	H1N1	11/26/2009	Austria	A/Vienna/INS142/2009(H1N1)
PA	CY069135	H1N1	11/26/2009	Spain	A/Madrid/INS299/2009(H1N1)
PA	CY066972	H1N1	11/26/2009	Denmark	A/Aarhus/INS241/2009(H1N1)
PA	CY073754	H1N1	11/26/2009	Denmark	A/Copenhagen/INS434/2009(H1N1)
PA	CY072235	H1N1	11/26/2009	Germany	A/Frankfurt/INS303/2009(H1N1)
PA	CY088618	H1N1	11/26/2009	Germany	A/Frankfurt/INS93/2009
PA	CY083734	H1N1	11/26/2009	Germany	A/Bochum/INS120/2009(H1N1)
PA	KT180913	H1N1	11/26/2009	Singapore	A/Singapore/DMS54/2009
PA	CY056744	H1N1	11/26/2009	USA	A/New York/6571/2009(H1N1)
PA	CY062760	H1N1	11/26/2009	Spain	A/Madrid/INS131/2009(H1N1)
PA	CY060952	H1N1	11/26/2009	USA	A/Texas/JMS379/2009(H1N1)
PA	CY057947	H1N1	11/26/2009	USA	A/Wisconsin/629-D00629/2009(H1N1)
PA	CY058737	H1N1	11/26/2009	USA	A/Texas/JMS377/2009(H1N1)
PA	CY058745	H1N1	11/26/2009	USA	A/Texas/JMS378/2009(H1N1)
PA	CY066964	H1N1	11/26/2009	Denmark	A/Aarhus/INS240/2009(H1N1)
PA	CY066700	H1N1	11/27/2009	Denmark	A/Hvidovre/INS290/2009(H1N1)
PA	CY075633	H1N1	11/27/2009	USA	A/Boston/701/2009(H1N1)
PA	KJ856066	H1N1	11/27/2009	Sri Lanka	A/Sri Lanka/2005/2009
PA	CY057955	H1N1	11/27/2009	USA	A/Wisconsin/629-D00050/2009(H1N1)
PA	CY062704	H1N1	11/27/2009	Denmark	A/Aarhus/INS118/2009(H1N1)
PA	CY060960	H1N1	11/27/2009	USA	A/Texas/JMS380/2009(H1N1)
PA	CY057963	H1N1	11/27/2009	USA	A/Wisconsin/629-D00970/2009(H1N1)
PA	AB704501	H1N1	11/27/2009	Japan	A/Yamagata/674/2009
PA	CY067060	H1N1	11/28/2009	Denmark	A/Aarhus/INS254/2009(H1N1)
PA	CY060976	H1N1	11/28/2009	USA	A/Texas/JMS382/2009(H1N1)
PA	CY060968	H1N1	11/28/2009	USA	A/Texas/JMS381/2009(H1N1)
PA	GU592902	H1N1	11/28/2009	Kyrgyzsta	A/Bishkek/03/2009(H1N1)
PA	CY088598	H1N1	11/29/2009	USA	A/Boston/702/2009
PA	CY090045	H1N1	11/29/2009	USA	A/Wisconsin/629-D01667/2009
PA	CY056808	H1N1	11/29/2009	USA	A/New York/6808/2009(H1N1)
PA	CY057971	H1N1	11/29/2009	USA	A/Wisconsin/629-D01528/2009(H1N1)
PA	CY073245	H1N1	11/29/2009	Serbia	A/Belgrade/WRAIR2385T/2009(H1N1)
PA	CY060984	H1N1	11/29/2009	USA	A/Texas/JMS383/2009(H1N1)
PA	CY056856	H1N1	11/29/2009	USA	A/New York/6909/2009(H1N1)
PA	CY073237	H1N1	11/29/2009	Serbia	A/Belgrade/WRAIR2385N/2009(H1N1)
PA	CY057979	H1N1	11/29/2009	USA	A/Wisconsin/629-D01434/2009(H1N1)
PA	CY056768	H1N1	11/30/2009	USA	A/New York/6668/2009(H1N1)
PA	CY060992	H1N1	11/30/2009	USA	A/Texas/JMS384/2009(H1N1)
PA	CY056816	H1N1	11/30/2009	USA	A/New York/6809/2009(H1N1)
PA	CY069223	H1N1	11/30/2009	Germany	A/Munich/INS363/2009(H1N1)
PA	CY069143	H1N1	11/30/2009	Spain	A/Madrid/INS300/2009(H1N1)
PA	CY066668	H1N1	11/30/2009	Germany	A/Cologne/INS286/2009(H1N1)
PA	CY123872	H1N1	11/30/2009	Singapore	A/Singapore/ON2378/2009
PA	CY072243	H1N1	11/30/2009	Germany	A/Frankfurt/INS305/2009(H1N1)
PA	CY083695	H1N1	11/30/2009	Spain	A/Terrassa/INS94/2009(H1N1)
PA	CY083792	H1N1	11/30/2009	Denmark	A/Odense/INS309/2009(H1N1)
PA	KJ856073	H1N1	11/30/2009	Sri Lanka	A/Sri Lanka/2008/2009
PA	KT180915	H1N1	11/30/2009	Singapore	A/Singapore/DMS55/2009
PA	CY100500	H1N1	11/30/2009	Mexico	A/Mexico City/INER16/2009
PA	CY063619	H1N1	11/30/2009	Germany	A/Cologne/INS136/2009(H1N1)
PA	CY056776	H1N1	12/01/2009	USA	A/New York/6669/2009(H1N1)
PA	CY057083	H1N1	12/01/2009	Mongolia	A/Dundgobi/9746/2009(H1N1)
PA	CY057987	H1N1	12/01/2009	USA	A/Wisconsin/629-D01412/2009(H1N1)
PA	CY123293	H1N1	12/01/2009	Singapore	A/Singapore/GP4556/2009

PA	CY065845	H1N1	12/01/2009	Netherlar	A/Netherlands/2636/2009
PA	CY071817	H1N1	12/01/2009	Ethiopia	A/Addis Ababa/WR2848N/2009(H1N1)
PA	CY073149	H1N1	12/01/2009	Mexico	A/Mexico City/WRAIR1786T/2009(H1N
PA	CY071825	H1N1	12/01/2009	Ethiopia	A/Addis Ababa/WR2848T/2009(H1N1)
PA	CY073502	H1N1	12/01/2009	Germany	A/Frankfurt/INS304/2009(H1N1)
PA	CY073133	H1N1	12/01/2009	Mexico	A/Mexico City/WRAIR1779T/2009(H1N
PA	KJ856081	H1N1	12/01/2009	Sri Lanka	A/Sri Lanka/2009/2009
PA	KJ856237	H1N1	12/01/2009	Sri Lanka	A/Sri Lanka/24588/2009
PA	KC780185	H1N1	12/01/2009	USA	A/Montana/22/2009
PA	CY057995	H1N1	12/02/2009	USA	A/Wisconsin/629-D00935/2009(H1N1)
PA	CY056984	H1N1	12/02/2009	Denmark	A/Copenhagen/INS98/2009(H1N1)
PA	CY058003	H1N1	12/02/2009	USA	A/Wisconsin/629-D02299/2009(H1N1)
PA	CY056840	H1N1	12/02/2009	USA	A/New York/6864/2009(H1N1)
PA	CY062768	H1N1	12/02/2009	Spain	A/Madrid/INS134/2009(H1N1)
PA	CY063611	H1N1	12/02/2009	Denmark	A/Aalborg/INS133/2009(H1N1)
PA	CY123299	H1N1	12/02/2009	Singapore	A/Singapore/GP4581/2009
PA	CY065901	H1N1	12/02/2009	Netherlar	A/Netherlands/2822/2009
PA	CY071092	H1N1	12/02/2009	Germany	A/Bochum/INS376/2009(H1N1)
PA	CY071433	H1N1	12/02/2009	Kyrgyzsta	A/Bishkek/WR0881T/2009(H1N1)
PA	CY072267	H1N1	12/02/2009	Poland	A/Warsaw/INS310/2009(H1N1)
PA	CY071196	H1N1	12/02/2009	Germany	A/Frankfurt/INS399/2009(H1N1)
PA	CY072275	H1N1	12/02/2009	Poland	A/Warsaw/INS311/2009(H1N1)
PA	CY075641	H1N1	12/02/2009	USA	A/Boston/703/2009(H1N1)
PA	CY071084	H1N1	12/02/2009	Germany	A/Bochum/INS375/2009(H1N1)
PA	CY073103	H1N1	12/02/2009	Taiwan	A/Taipei/WRAIR1470T/2009(H1N1)
PA	AB704517	H1N1	12/02/2009	Japan	A/Yamaguchi/247/2009
PA	CY128432	H1N1	12/02/2009	Viet Nam	A/Viet Nam/12032005/2009
PA	HM568119	H1N1	12/02/2009	United Ki	A/England/941/2009
PA	CY062832	H1N1	12/03/2009	Poland	A/Warsaw/INS146/2009(H1N1)
PA	CY072283	H1N1	12/03/2009	Poland	A/Warsaw/INS312/2009(H1N1)
PA	CY083923	H1N1	12/03/2009	Poland	A/Warsaw/INS147/2009(H1N1)
PA	CY072291	H1N1	12/03/2009	Poland	A/Warsaw/INS313/2009(H1N1)
PA	CY073770	H1N1	12/03/2009	Spain	A/Barcelona/INS378/2009(H1N1)
PA	CY075649	H1N1	12/03/2009	USA	A/Boston/704/2009(H1N1)
PA	KT180916	H1N1	12/03/2009	Singapore	A/Singapore/DMS56/2009
PA	CY123878	H1N1	12/03/2009	Singapore	A/Singapore/ON2383/2009
PA	CY057355	H1N1	12/04/2009	Germany	A/Hamburg/INS92/2009(H1N1)
PA	CY056800	H1N1	12/04/2009	USA	A/New York/6776/2009(H1N1)
PA	GU727827	H1N1	12/04/2009	Russia	A/IIV-Anadyr/177/2009(H1N1)
PA	CY062776	H1N1	12/04/2009	Spain	A/Madrid/INS135/2009(H1N1)
PA	HM157257	H1N1	12/04/2009	Russia	A/Yaroslavl/IIV-196/2009(H1N1)
PA	CY061000	H1N1	12/04/2009	USA	A/Texas/JMS385/2009(H1N1)
PA	CY066308	H1N1	12/04/2009	USA	A/California/VRDL116/2009(H1N1)
PA	CY065789	H1N1	12/04/2009	Netherlar	A/Netherlands/2629/2009
PA	CY069231	H1N1	12/04/2009	Germany	A/Munich/INS364/2009(H1N1)
PA	CY066292	H1N1	12/04/2009	USA	A/California/VRDL114/2009(H1N1)
PA	CY066324	H1N1	12/04/2009	USA	A/California/VRDL118/2009(H1N1)
PA	CY066300	H1N1	12/04/2009	USA	A/California/VRDL115/2009(H1N1)
PA	CY066316	H1N1	12/04/2009	USA	A/California/VRDL117/2009(H1N1)
PA	KC780301	H1N1	12/04/2009	USA	A/Pennsylvania/31/2009
PA	KC780779	H1N1	12/04/2009	USA	A/Alaska/47/2009
PA	CY107533	H1N1	12/04/2009	United Ki	A/Scotland/Edinburgh_23151/2009
PA	JN187251	H1N1	12/04/2009	Taiwan	A/Taiwan/6341/2009
PA	CY083558	H1N1	12/04/2009	Hungary	A/Budapest/WRAIR2411N/2009

PA	CY088606	H1N1	12/04/2009	USA	A/Boston/705/2009
PA	CY056792	H1N1	12/05/2009	USA	A/New York/6775/2009(H1N1)
PA	HM104482	H1N1	12/05/2009	Russia	A/Yaroslavl/IIV-198/2009(H1N1)
PA	AB704525	H1N1	12/05/2009	Japan	A/Yamaguchi/248/2009
PA	JQ714157	H1N1	12/05/2009	China	A/Tianjinninghe/SWL49/2009
PA	CY061008	H1N1	12/06/2009	USA	A/Texas/JMS386/2009(H1N1)
PA	CY061943	H1N1	12/06/2009	USA	A/New York/6941/2009(H1N1)
PA	CY065080	H1N1	12/06/2009	USA	A/New York/7421/2009(H1N1)
PA	KC781014	H1N1	12/06/2009	USA	A/Arizona/20/2009
PA	CY056992	H1N1	12/07/2009	Poland	A/Warsaw/INS100/2009(H1N1)
PA	CY058011	H1N1	12/07/2009	USA	A/Wisconsin/629-D01473/2009(H1N1)
PA	CY056968	H1N1	12/07/2009	Denmark	A/Copenhagen/INS95/2009(H1N1)
PA	CY061224	H1N1	12/07/2009	USA	A/New York/6772/2009(H1N1)
PA	CY061599	H1N1	12/07/2009	USA	A/New York/6806/2009(H1N1)
PA	CY061951	H1N1	12/07/2009	USA	A/New York/6943/2009(H1N1)
PA	CY062824	H1N1	12/07/2009	Denmark	A/Copenhagen/INS144/2009(H1N1)
PA	CY061216	H1N1	12/07/2009	USA	A/New York/6771/2009(H1N1)
PA	CY058401	H1N1	12/07/2009	USA	A/Wisconsin/629-D01591/2009(H1N1)
PA	CY063627	H1N1	12/07/2009	USA	A/New York/INS150/2009(H1N1)
PA	CY065893	H1N1	12/07/2009	Netherlar	A/Netherlands/2815/2009
PA	CY072307	H1N1	12/07/2009	Poland	A/Warsaw/INS315/2009(H1N1)
PA	CY072299	H1N1	12/07/2009	Poland	A/Warsaw/INS314/2009(H1N1)
PA	CY073730	H1N1	12/07/2009	Denmark	A/Aalborg/INS283/2009(H1N1)
PA	KT180918	H1N1	12/07/2009	Singapore	A/Singapore/DMS57/2009
PA	KC780595	H1N1	12/07/2009	USA	A/Texas/75/2009
PA	KC781088	H1N1	12/07/2009	USA	A/Iowa/14/2009
PA	CY058019	H1N1	12/08/2009	USA	A/Wisconsin/629-D00672/2009(H1N1)
PA	CY058027	H1N1	12/08/2009	USA	A/Wisconsin/629-D00401/2009(H1N1)
PA	CY061016	H1N1	12/08/2009	USA	A/Texas/JMS387/2009(H1N1)
PA	CY061919	H1N1	12/08/2009	USA	A/New York/6907/2009(H1N1)
PA	CY058385	H1N1	12/08/2009	USA	A/Wisconsin/629-D00780/2009(H1N1)
PA	CY065104	H1N1	12/08/2009	USA	A/New York/7426/2009(H1N1)
PA	CY066932	H1N1	12/08/2009	USA	A/Pensacola/INS235/2009
PA	CY067172	H1N1	12/08/2009	Germany	A/Bonn/INS280/2009(H1N1)
PA	CY066924	H1N1	12/08/2009	USA	A/Pensacola/INS234/2009
PA	CY072315	H1N1	12/08/2009	Poland	A/Warsaw/INS316/2009(H1N1)
PA	KC780841	H1N1	12/08/2009	USA	A/Oregon/42/2009
PA	LN846549	H1N1	12/08/2009	United Ki	A/England/9/2009
PA	LN845781	H1N1	12/08/2009	United Ki	A/England/8/2009
PA	CY058393	H1N1	12/09/2009	USA	A/Wisconsin/629-D00147/2009(H1N1)
PA	CY058369	H1N1	12/09/2009	USA	A/Wisconsin/629-D00689/2009(H1N1)
PA	CY063595	H1N1	12/09/2009	Greece	A/Athens/INS122/2009(H1N1)
PA	CY072547	H1N1	12/09/2009	Poland	A/Wroclaw/INS436/2009(H1N1)
PA	CY072555	H1N1	12/09/2009	Poland	A/Wroclaw/INS437/2009(H1N1)
PA	CY069215	H1N1	12/09/2009	Germany	A/Berlin/INS361/2009(H1N1)
PA	CY069239	H1N1	12/09/2009	Germany	A/Munich/INS365/2009(H1N1)
PA	CY067180	H1N1	12/09/2009	Germany	A/Bonn/INS284/2009(H1N1)
PA	CY128384	H1N1	12/09/2009	Viet Nam	A/Viet Nam/11032006/2009
PA	CY089607	H1N1	12/09/2009	China	A/Nanchang/8002/2009
PA	CY073229	H1N1	12/10/2009	Serbia	A/Belgrade/WRAIR2384T/2009(H1N1)
PA	CY065994	H1N1	12/10/2009	Mongolia	A/Ulgii/9911/2009(H1N1)
PA	CY073221	H1N1	12/10/2009	Serbia	A/Belgrade/WRAIR2384N/2009(H1N1)
PA	KT180919	H1N1	12/10/2009	Singapore	A/Singapore/DMS58/2009
PA	CY062784	H1N1	12/10/2009	Belgium	A/Tessenderlo/INS138/2009(H1N1)

PA	CY058035	H1N1	12/10/2009	USA	A/Wisconsin/629-D00636/2009(H1N1)
PA	CY061911	H1N1	12/10/2009	USA	A/New York/6903/2009(H1N1)
PA	CY063048	H1N1	12/10/2009	Germany	A/Bochum/INS187/2009(H1N1)
PA	CY066676	H1N1	12/10/2009	Belgium	A/Tessenderlo/INS287/2009(H1N1)
PA	CY056976	H1N1	12/10/2009	Denmark	A/Copenhagen/INS96/2009(H1N1)
PA	CY071100	H1N1	12/11/2009	Spain	A/Barcelona/INS379/2009(H1N1)
PA	CY083939	H1N1	12/11/2009	Poland	A/Warsaw/INS149/2009(H1N1)
PA	CY083750	H1N1	12/11/2009	Spain	A/Barcelona/INS189/2009(H1N1)
PA	CY083931	H1N1	12/11/2009	Poland	A/Warsaw/INS148/2009(H1N1)
PA	CY061927	H1N1	12/11/2009	USA	A/New York/6937/2009(H1N1)
PA	CY064777	H1N1	12/11/2009	Germany	A/Berlin/INS170/2009(H1N1)
PA	CY061935	H1N1	12/11/2009	USA	A/New York/6939/2009(H1N1)
PA	CY061959	H1N1	12/11/2009	USA	A/New York/6945/2009(H1N1)
PA	CY061024	H1N1	12/11/2009	USA	A/Texas/JMS388/2009(H1N1)
PA	CY063635	H1N1	12/11/2009	USA	A/New York/INS151/2009(H1N1)
PA	CY123888	H1N1	12/11/2009	Singapore	A/Singapore/ON2400/2009
PA	CY061983	H1N1	12/11/2009	USA	A/New York/6977/2009(H1N1)
PA	CY058377	H1N1	12/12/2009	USA	A/Wisconsin/629-D01244/2009(H1N1)
PA	CY083802	H1N1	12/12/2009	Germany	A/Berlin/INS362/2009(H1N1)
PA	CY079536	H1N1	12/13/2009	Switzerla	A/Switzerland/9356/2009
PA	CY072347	H1N1	12/13/2009	Greece	A/Athens/INS320/2009(H1N1)
PA	CY067100	H1N1	12/13/2009	Greece	A/Athens/INS262/2009(H1N1)
PA	CY067076	H1N1	12/13/2009	Greece	A/Athens/INS259/2009(H1N1)
PA	CY067068	H1N1	12/13/2009	Greece	A/Athens/INS257/2009(H1N1)
PA	CY067116	H1N1	12/13/2009	Greece	A/Athens/INS264/2009(H1N1)
PA	CY067108	H1N1	12/13/2009	Greece	A/Athens/INS263/2009(H1N1)
PA	CY067092	H1N1	12/13/2009	Greece	A/Athens/INS261/2009(H1N1)
PA	CY067124	H1N1	12/13/2009	Greece	A/Athens/INS266/2009(H1N1)
PA	CY067084	H1N1	12/13/2009	Greece	A/Athens/INS260/2009(H1N1)
PA	CY056960	H1N1	12/13/2009	Greece	A/Athens/INS85/2009(H1N1)
PA	CY062728	H1N1	12/13/2009	Greece	A/Athens/INS125/2009(H1N1)
PA	CY062720	H1N1	12/13/2009	Greece	A/Athens/INS124/2009(H1N1)
PA	CY062712	H1N1	12/13/2009	Greece	A/Athens/INS123/2009(H1N1)
PA	CY072355	H1N1	12/13/2009	Greece	A/Athens/INS322/2009(H1N1)
PA	CY083907	H1N1	12/13/2009	Greece	A/Athens/INS86/2009(H1N1)
PA	CY073205	H1N1	12/14/2009	Serbia	A/Belgrade/WRAIR2375N/2009(H1N1)
PA	KT180920	H1N1	12/14/2009	Singapore	A/Singapore/DMS59/2009
PA	KC683494	H1N1	12/14/2009	China	A/Liaoning/14/2009
PA	CY107417	H1N1	12/14/2009	United Ki	A/Scotland/Dundee_09V502215/2009
PA	GU592910	H1N1	12/14/2009	Russia	A/Magadan/02/2009(H1N1)
PA	CY073213	H1N1	12/14/2009	Serbia	A/Belgrade/WRAIR2375T/2009(H1N1)
PA	CY061999	H1N1	12/14/2009	USA	A/New York/7020/2009(H1N1)
PA	CY061967	H1N1	12/14/2009	USA	A/New York/6949/2009(H1N1)
PA	CY058457	H1N1	12/14/2009	USA	A/Wisconsin/629-D01846/2009(H1N1)
PA	CY058425	H1N1	12/14/2009	USA	A/Wisconsin/629-D01904/2009(H1N1)
PA	CY061975	H1N1	12/14/2009	USA	A/New York/6976/2009(H1N1)
PA	CY066332	H1N1	12/15/2009	USA	A/California/VRDL119/2009(H1N1)
PA	CY072331	H1N1	12/15/2009	USA	A/Newark/INS318/2009(H1N1)
PA	CY066340	H1N1	12/15/2009	USA	A/California/VRDL120/2009(H1N1)
PA	CY066348	H1N1	12/15/2009	USA	A/California/VRDL121/2009(H1N1)
PA	CY067140	H1N1	12/15/2009	Greece	A/Athens/INS268/2009(H1N1)
PA	CY067156	H1N1	12/15/2009	Greece	A/Athens/INS270/2009(H1N1)
PA	CY067148	H1N1	12/15/2009	Greece	A/Athens/INS269/2009(H1N1)
PA	CY073494	H1N1	12/15/2009	Greece	A/Athens/INS271/2009(H1N1)

PA	CY073882	H1N1	12/15/2009	Greece	A/Athens/INS323/2009(H1N1)
PA	CY073181	H1N1	12/15/2009	Serbia	A/Belgrade/WRAIR2366N/2009(H1N1)
PA	AB704453	H1N1	12/15/2009	Japan	A/Gunma/293/2009
PA	CY090102	H1N1	12/15/2009	USA	A/Boston/706/2009
PA	CY058433	H1N1	12/15/2009	USA	A/Wisconsin/629-D01752/2009(H1N1)
PA	CY058473	H1N1	12/15/2009	USA	A/Wisconsin/629-D00337/2009(H1N1)
PA	CY058481	H1N1	12/15/2009	USA	A/Wisconsin/629-D02424/2009(H1N1)
PA	CY066364	H1N1	12/15/2009	USA	A/California/VRDL123/2009(H1N1)
PA	CY066612	H1N1	12/15/2009	Greece	A/Athens/INS273/2009(H1N1)
PA	CY066604	H1N1	12/15/2009	Greece	A/Athens/INS272/2009(H1N1)
PA	CY062736	H1N1	12/15/2009	Greece	A/Athens/INS126/2009(H1N1)
PA	CY123894	H1N1	12/15/2009	Singapore	A/Singapore/ON2416/2009
PA	CY066628	H1N1	12/15/2009	Greece	A/Athens/INS275/2009(H1N1)
PA	CY066620	H1N1	12/15/2009	Greece	A/Athens/INS274/2009(H1N1)
PA	CY062664	H1N1	12/15/2009	Greece	A/Athens/INS87/2009(H1N1)
PA	CY063024	H1N1	12/15/2009	Spain	A/Madrid/INS184/2009(H1N1)
PA	CY063056	H1N1	12/15/2009	Belgium	A/Tessenderlo/INS191/2009(H1N1)
PA	CY066356	H1N1	12/15/2009	USA	A/California/VRDL122/2009(H1N1)
PA	CY071108	H1N1	12/16/2009	Belgium	A/Tessenderlo/INS380/2009(H1N1)
PA	CY067132	H1N1	12/16/2009	Greece	A/Athens/INS267/2009(H1N1)
PA	KT180921	H1N1	12/16/2009	Singapore	A/Singapore/DMS60/2009
PA	CY062944	H1N1	12/16/2009	Spain	A/Terrassa/INS172/2009(H1N1)
PA	CY061032	H1N1	12/16/2009	USA	A/Texas/JMS389/2009(H1N1)
PA	CY071260	H1N1	12/16/2009	Spain	A/Madrid/INS408/2009(H1N1)
PA	CY062007	H1N1	12/16/2009	USA	A/New York/7036/2009(H1N1)
PA	CY058409	H1N1	12/16/2009	USA	A/Wisconsin/629-D01038/2009(H1N1)
PA	CY058465	H1N1	12/16/2009	USA	A/Wisconsin/629-D00996/2009(H1N1)
PA	CY058449	H1N1	12/16/2009	USA	A/Wisconsin/629-D02073/2009(H1N1)
PA	CY061991	H1N1	12/16/2009	USA	A/New York/7019/2009(H1N1)
PA	CY072475	H1N1	12/17/2009	Greece	A/Athens/INS342/2009(H1N1)
PA	CY083810	H1N1	12/17/2009	Spain	A/Madrid/INS377/2009(H1N1)
PA	CY067164	H1N1	12/17/2009	Greece	A/Athens/INS276/2009(H1N1)
PA	CY072467	H1N1	12/17/2009	Greece	A/Athens/INS341/2009(H1N1)
PA	CY128058	H1N1	12/17/2009	Viet Nam	A/Viet Nam/11032011/2009
PA	CY128392	H1N1	12/17/2009	Viet Nam	A/Viet Nam/11032010/2009
PA	CY058441	H1N1	12/17/2009	USA	A/Wisconsin/629-D01556/2009(H1N1)
PA	CY062952	H1N1	12/17/2009	Spain	A/Terrassa/INS173/2009(H1N1)
PA	CY062856	H1N1	12/17/2009	Greece	A/Athens/INS158/2009(H1N1)
PA	CY072459	H1N1	12/17/2009	Greece	A/Athens/INS340/2009(H1N1)
PA	CY072323	H1N1	12/17/2009	USA	A/New York/INS317/2009(H1N1)
PA	CY062872	H1N1	12/18/2009	Greece	A/Athens/INS160/2009(H1N1)
PA	AB704533	H1N1	12/18/2009	Japan	A/Yamaguchi/273/2009
PA	JN187252	H1N1	12/18/2009	Taiwan	A/Taiwan/11706/2009
PA	CY072483	H1N1	12/18/2009	Greece	A/Athens/INS344/2009(H1N1)
PA	CY062864	H1N1	12/18/2009	Greece	A/Athens/INS159/2009(H1N1)
PA	CY058417	H1N1	12/18/2009	USA	A/Wisconsin/629-D00459/2009(H1N1)
PA	CY072491	H1N1	12/18/2009	Greece	A/Athens/INS345/2009(H1N1)
PA	CY063659	H1N1	12/18/2009	Greece	A/Athens/INS157/2009(H1N1)
PA	CY062936	H1N1	12/18/2009	Germany	A/Berlin/INS171/2009(H1N1)
PA	GU592894	H1N1	12/18/2009	Russia	A/Barnaul/04/2009(H1N1)
PA	CY062880	H1N1	12/19/2009	Greece	A/Athens/INS161/2009(H1N1)
PA	CY089615	H1N1	12/19/2009	China	A/Nanchang/8008/2009
PA	HM230706	H1N1	12/19/2009	China	A/Zhoushan/52/2009(H1N1)
PA	CY062904	H1N1	12/20/2009	Greece	A/Athens/INS164/2009(H1N1)

PA	CY065096	H1N1	12/20/2009	USA	A/New York/7425/2009(H1N1)
PA	CY072507	H1N1	12/20/2009	Greece	A/Athens/INS347/2009(H1N1)
PA	CY069159	H1N1	12/20/2009	Greece	A/Athens/INS352/2009(H1N1)
PA	CY073510	H1N1	12/20/2009	Greece	A/Athens/INS350/2009(H1N1)
PA	CY072363	H1N1	12/20/2009	Greece	A/Athens/INS325/2009(H1N1)
PA	CY062888	H1N1	12/20/2009	Greece	A/Athens/INS162/2009(H1N1)
PA	CY072371	H1N1	12/20/2009	Greece	A/Athens/INS327/2009(H1N1)
PA	CY062896	H1N1	12/20/2009	Greece	A/Athens/INS163/2009(H1N1)
PA	CY107424	H1N1	12/20/2009	United Ki	A/Scotland/Dundee_09V502385/2009
PA	CY061040	H1N1	12/20/2009	USA	A/Texas/JMS390/2009(H1N1)
PA	CY072515	H1N1	12/20/2009	Greece	A/Athens/INS348/2009(H1N1)
PA	CY072499	H1N1	12/20/2009	Greece	A/Athens/INS346/2009(H1N1)
PA	CY072523	H1N1	12/20/2009	Greece	A/Athens/INS349/2009(H1N1)
PA	CY062912	H1N1	12/21/2009	Greece	A/Athens/INS165/2009(H1N1)
PA	CY088626	H1N1	12/21/2009	Denmark	A/Odense/INS176/2009
PA	CY088631	H1N1	12/21/2009	India	A/Pune/NIV0953725/2009(H1N1)
PA	CY107431	H1N1	12/21/2009	United Ki	A/Scotland/Dundee_09V502390/2009
PA	CY063000	H1N1	12/21/2009	Estonia	A/Tallinn/INS181/2009(H1N1)
PA	KT180922	H1N1	12/21/2009	Singapore	A/Singapore/DMS61/2009
PA	CY069167	H1N1	12/21/2009	Greece	A/Athens/INS353/2009(H1N1)
PA	CY069175	H1N1	12/21/2009	Greece	A/Athens/INS354/2009(H1N1)
PA	CY062023	H1N1	12/21/2009	USA	A/New York/7216/2009(H1N1)
PA	CY069247	H1N1	12/22/2009	Austria	A/Vienna/INS366/2009(H1N1)
PA	CY069183	H1N1	12/22/2009	Greece	A/Athens/INS355/2009(H1N1)
PA	CY071116	H1N1	12/22/2009	Germany	A/Wurzburg/INS381/2009(H1N1)
PA	CY066380	H1N1	12/22/2009	USA	A/California/VRDL125/2009(H1N1)
PA	CY066404	H1N1	12/22/2009	USA	A/California/VRDL128/2009(H1N1)
PA	CY066396	H1N1	12/22/2009	USA	A/California/VRDL127/2009(H1N1)
PA	CY066372	H1N1	12/22/2009	USA	A/California/VRDL124/2009(H1N1)
PA	CY089623	H1N1	12/22/2009	China	A/Nanchang/8011/2009
PA	CY066388	H1N1	12/22/2009	USA	A/California/VRDL126/2009(H1N1)
PA	CY073253	H1N1	12/23/2009	Greece	A/Athens/WRAIR2962N/2009(H1N1)
PA	CY065088	H1N1	12/23/2009	USA	A/New York/7424/2009(H1N1)
PA	CY061048	H1N1	12/23/2009	USA	A/Texas/JMS391/2009(H1N1)
PA	CY062015	H1N1	12/23/2009	USA	A/New York/7107/2009(H1N1)
PA	CY062968	H1N1	12/23/2009	Spain	A/Terrassa/INS175/2009(H1N1)
PA	CY128400	H1N1	12/23/2009	Viet Nam	A/Viet Nam/11032016/2009
PA	CY128408	H1N1	12/23/2009	Viet Nam	A/Viet Nam/11032017/2009
PA	CY107438	H1N1	12/23/2009	United Ki	A/Scotland/Perth_09V502475/2009
PA	LN867651	H1N1	12/23/2009	United Ki	A/England/01220639/2009
PA	CY071068	H1N1	12/23/2009	Estonia	A/Tallinn/INS372/2009(H1N1)
PA	CY071204	H1N1	12/23/2009	Germany	A/Frankfurt/INS401/2009(H1N1)
PA	CY062960	H1N1	12/24/2009	Spain	A/Terrassa/INS174/2009(H1N1)
PA	KT180925	H1N1	12/25/2009	Singapore	A/Singapore/DMS62/2009
PA	CY061056	H1N1	12/25/2009	USA	A/Texas/JMS392/2009(H1N1)
PA	CY071124	H1N1	12/25/2009	Germany	A/Wurzburg/INS382/2009(H1N1)
PA	CY072379	H1N1	12/25/2009	Greece	A/Athens/INS329/2009(H1N1)
PA	CY062039	H1N1	12/26/2009	USA	A/New York/0259/2009(H1N1)
PA	CY069191	H1N1	12/26/2009	Greece	A/Athens/INS357/2009(H1N1)
PA	CY069207	H1N1	12/26/2009	Greece	A/Athens/INS359/2009(H1N1)
PA	CY062928	H1N1	12/26/2009	Greece	A/Athens/INS167/2009(H1N1)
PA	CY062920	H1N1	12/26/2009	Greece	A/Athens/INS166/2009(H1N1)
PA	CY069199	H1N1	12/26/2009	Greece	A/Athens/INS358/2009(H1N1)
PA	CY072395	H1N1	12/26/2009	Greece	A/Athens/INS332/2009(H1N1)

PA	CY072387	H1N1	12/26/2009	Greece	A/Athens/INS331/2009(H1N1)
PA	CY073011	H1N1	12/26/2009	Greece	A/Athens/INS330/2009(H1N1)
PA	CY062047	H1N1	12/27/2009	USA	A/New York/0352/2009(H1N1)
PA	CY061064	H1N1	12/27/2009	USA	A/Texas/JMS393/2009(H1N1)
PA	KC780317	H1N1	12/28/2009	USA	A/Georgia/25/2009
PA	CY062031	H1N1	12/28/2009	USA	A/New York/7236/2009(H1N1)
PA	KT180927	H1N1	12/28/2009	Singapore	A/Singapore/DMS63/2009
PA	CY072403	H1N1	12/28/2009	Greece	A/Athens/INS333/2009(H1N1)
PA	CY072339	H1N1	12/28/2009	USA	A/Newark/INS319/2009(H1N1)
PA	CY062976	H1N1	12/28/2009	Denmark	A/Odense/INS177/2009(H1N1)
PA	CY071236	H1N1	12/29/2009	Poland	A/Warsaw/INS405/2009(H1N1)
PA	CY071244	H1N1	12/29/2009	Poland	A/Warsaw/INS406/2009(H1N1)
PA	CY072411	H1N1	12/29/2009	Greece	A/Athens/INS334/2009(H1N1)
PA	CY064785	H1N1	12/29/2009	Spain	A/Barcelona/INS190/2009(H1N1)
PA	CY063643	H1N1	12/29/2009	Greece	A/Athens/INS153/2009(H1N1)
PA	CY063651	H1N1	12/29/2009	Greece	A/Athens/INS154/2009(H1N1)
PA	JN187253	H1N1	12/29/2009	Taiwan	A/Taiwan/1017/2009
PA	CY063064	H1N1	12/30/2009	Belgium	A/Tessenderlo/INS192/2009(H1N1)
PA	JQ714158	H1N1	12/30/2009	China	A/Tianjinjinnan/SWL4141/2009
PA	CY061080	H1N1	12/30/2009	USA	A/Texas/JMS395/2009(H1N1)
PA	HM568127	H1N1	12/30/2009	United Ki	A/England/938/2009
PA	CY128416	H1N1	12/30/2009	Viet Nam	A/Viet Nam/11032021/2009
PA	CY123311	H1N1	12/30/2009	Singapore	A/Singapore/GP4895/2009
PA	LN846366	H1N1	12/30/2009	United Ki	A/England/WTSI1778/2009
PA	KF411292	H1N1	12/30/2009	China	A/Qingdao/FF85/2009
PA	CY062055	H1N1	12/30/2009	USA	A/New York/0357/2009(H1N1)
PA	CY062063	H1N1	12/30/2009	USA	A/New York/0461/2009(H1N1)
PA	CY061096	H1N1	12/30/2009	USA	A/Texas/JMS398/2009(H1N1)
PA	CY061072	H1N1	12/30/2009	USA	A/Texas/JMS394/2009(H1N1)
PA	CY061088	H1N1	12/30/2009	USA	A/Texas/JMS397/2009(H1N1)
PA	KF411294	H1N1	12/30/2009	China	A/Qingdao/1364/2009
PA	KF411291	H1N1	12/30/2009	China	A/Qingdao/1170/2009
PA	KF411290	H1N1	12/30/2009	China	A/Qingdao/1008/2009
PA	KF411288	H1N1	12/30/2009	China	A/Qingdao/1006/2009
PA	KF411289	H1N1	12/30/2009	China	A/Qingdao/1020/2009
PA	CY063040	H1N1	12/30/2009	Spain	A/Madrid/INS186/2009(H1N1)
PA	CY062848	H1N1	12/30/2009	Greece	A/Athens/INS156/2009(H1N1)
PA	CY062840	H1N1	12/30/2009	Greece	A/Athens/INS155/2009(H1N1)
PA	CY063032	H1N1	12/30/2009	Spain	A/Madrid/INS185/2009(H1N1)
PA	CY072419	H1N1	12/30/2009	Greece	A/Athens/INS335/2009(H1N1)
PA	CY072427	H1N1	12/30/2009	Greece	A/Athens/INS336/2009(H1N1)
PA	CY072435	H1N1	12/30/2009	Greece	A/Athens/INS337/2009(H1N1)
PA	CY072443	H1N1	12/30/2009	Greece	A/Athens/INS338/2009(H1N1)
PA	CY066412	H1N1	12/30/2009	USA	A/California/VRDL129/2009(H1N1)
PA	CY066436	H1N1	12/30/2009	USA	A/California/VRDL132/2009(H1N1)
PA	CY066444	H1N1	12/30/2009	USA	A/California/VRDL133/2009(H1N1)
PA	CY066428	H1N1	12/30/2009	USA	A/California/VRDL131/2009(H1N1)
PA	CY066420	H1N1	12/30/2009	USA	A/California/VRDL130/2009(H1N1)
PA	KF411299	H1N1	12/30/2009	China	A/Qingdao/1609/2009
PA	KF411297	H1N1	12/30/2009	China	A/Qingdao/1568/2009
PA	KF411300	H1N1	12/30/2009	China	A/Qingdao/1610/2009
PA	KF411296	H1N1	12/30/2009	China	A/Qingdao/1530/2009
PA	KF411302	H1N1	12/30/2009	China	A/Qingdao/1631/2009
PA	KF411298	H1N1	12/30/2009	China	A/Qingdao/1577/2009

PA	CY072451	H1N1	12/30/2009	Greece	A/Athens/INS339/2009(H1N1)
PA	KF411301	H1N1	12/30/2009	China	A/Qingdao/1626/2009
PA	KF411295	H1N1	12/30/2009	China	A/Qingdao/1508/2009
PA	KC780666	H1N1	12/30/2009	USA	A/Nebraska/23/2009
PA	KF411293	H1N1	12/30/2009	China	A/Qingdao/FF86/2009
PA	KT180929	H1N1	12/31/2009	Singapore	A/Singapore/DMS64/2009
PA	CY071132	H1N1	12/31/2009	Greece	A/Athens/INS383/2009(H1N1)
PA	CY107445	H1N1	12/31/2009	United Ki	A/Scotland/Dundee_09V502666/2009
PA	CY123321	H1N1	12/31/2009	Singapore	A/Singapore/GP4915/2009
PA	CY061575	H1N1	12/31/2009	USA	A/Texas/JMS400/2009(H1N1)
PA	CY061104	H1N1	12/31/2009	USA	A/Texas/JMS399/2009(H1N1)
PA	CY120023	H1N1	01/2010	Mexico	A/Mexico/InDRECTRLA/2010
PA	KC780541	H1N1	01/01/2010	USA	A/New Jersey/01/2010
PA	JQ714159	H1N1	01/02/2010	China	A/Tianjinnankai/SWL49/2010
PA	CY065072	H1N1	01/02/2010	USA	A/New York/7420/2010(H1N1)
PA	CY061583	H1N1	01/03/2010	USA	A/Texas/JMS401/2010(H1N1)
PA	CY061112	H1N1	01/04/2010	USA	A/Texas/JMS402/2010(H1N1)
PA	CY061591	H1N1	01/04/2010	USA	A/Texas/JMS403/2010(H1N1)
PA	KC781900	H1N1	01/04/2010	USA	A/Alabama/01/2010
PA	JX308948	H1N1	01/04/2010	Singapore	A/Singapore/GP10/2010
PA	KT180930	H1N1	01/04/2010	Singapore	A/Singapore/DMS65/2010
PA	CY063008	H1N1	01/04/2010	Estonia	A/Tallinn/INS182/2010(H1N1)
PA	GU562463	H1N1	01/04/2010	Russia	A/Karasuk/01/2010(H1N1)
PA	CY096591	H1N1	01/04/2010	USA	A/District of Columbia/INS525/201
PA	CY065877	H1N1	01/04/2010	Netherlar	A/Netherlands/2631b/2010
PA	CY071060	H1N1	01/05/2010	Austria	A/Vienna/INS368/2010(H1N1)
PA	CY062992	H1N1	01/05/2010	Austria	A/Vienna/INS179/2010(H1N1)
PA	JQ714160	H1N1	01/05/2010	China	A/Tianjinnankai/SWL413/2010
PA	CY073778	H1N1	01/05/2010	Austria	A/Vienna/INS369/2010(H1N1)
PA	CY062984	H1N1	01/05/2010	Germany	A/Bonn/INS178/2010(H1N1)
PA	JX308956	H1N1	01/05/2010	Singapore	A/Singapore/GP33/2010
PA	KC782017	H1N1	01/05/2010	USA	A/Iowa/01/2010
PA	KC780229	H1N1	01/05/2010	USA	A/Colorado/02/2010
PA	CY075072	H1N1	01/06/2010	Greece	A/Athens/INS393/2010(H1N1)
PA	JX308964	H1N1	01/06/2010	Singapore	A/Singapore/GP64/2010
PA	CY075064	H1N1	01/06/2010	Greece	A/Athens/INS392/2010(H1N1)
PA	KC780415	H1N1	01/06/2010	USA	A/Florida/01/2010
PA	KT180932	H1N1	01/07/2010	Singapore	A/Singapore/DMS66/2010
PA	CY072531	H1N1	01/07/2010	Estonia	A/Tallinn/INS374/2010(H1N1)
PA	CY100507	H1N1	01/07/2010	Mexico	A/Mexico City/INER17/2010
PA	CY071076	H1N1	01/07/2010	Estonia	A/Tallinn/INS373/2010(H1N1)
PA	CY063016	H1N1	01/07/2010	Estonia	A/Tallinn/INS183/2010(H1N1)
PA	CY071212	H1N1	01/08/2010	Germany	A/Frankfurt/INS402/2010(H1N1)
PA	CY061120	H1N1	01/08/2010	USA	A/Texas/JMS404/2010(H1N1)
PA	CY061128	H1N1	01/09/2010	USA	A/Texas/JMS405/2010(H1N1)
PA	CY066452	H1N1	01/09/2010	USA	A/California/VRDL1/2010(H1N1)
PA	CY061136	H1N1	01/10/2010	USA	A/Texas/JMS406/2010(H1N1)
PA	JX308972	H1N1	01/11/2010	Singapore	A/Singapore/GP132/2010
PA	CY066460	H1N1	01/11/2010	USA	A/California/VRDL2/2010(H1N1)
PA	CY071164	H1N1	01/11/2010	Greece	A/Athens/INS394/2010(H1N1)
PA	CY061144	H1N1	01/11/2010	USA	A/Texas/JMS407/2010(H1N1)
PA	KT180934	H1N1	01/11/2010	Singapore	A/Singapore/DMS67/2010
PA	CY071220	H1N1	01/11/2010	Germany	A/Frankfurt/INS403/2010(H1N1)
PA	JQ612506	H1N1	01/12/2010	Hungary	A/Hungary/2/2010

PA	AB704485	H1N1	01/12/2010	Japan	A/Tochigi/10/2010
PA	CY061152	H1N1	01/12/2010	USA	A/Texas/JMS408/2010(H1N1)
PA	CY062071	H1N1	01/12/2010	USA	A/New York/1680/2010(H1N1)
PA	KC780890	H1N1	01/12/2010	USA	A/Nevada/01/2010
PA	KP637669	H1N1	01/12/2010	Thailand	A/Thailand/SP08358/2010
PA	AB704477	H1N1	01/13/2010	Japan	A/Tochigi/2/2010
PA	CY071140	H1N1	01/13/2010	Greece	A/Athens/INS389/2010(H1N1)
PA	CY074981	H1N1	01/13/2010	Thailand	A/Thailand/CU-H1222/2010(H1N1)
PA	CY088666	H1N1	01/13/2010	India	A/Ngp/NIV22704/2010(H1N1)
PA	JX309000	H1N1	01/14/2010	Singapore	A/Singapore/GP220/2010
PA	CY072539	H1N1	01/14/2010	USA	A/New York/INS432/2010(H1N1)
PA	CY071148	H1N1	01/14/2010	Greece	A/Athens/INS390/2010(H1N1)
PA	KT180936	H1N1	01/14/2010	Singapore	A/Singapore/DMS68/2010
PA	LN867803	H1N1	01/14/2010	United Ki	A/England/01220740/2010
PA	CY062079	H1N1	01/15/2010	USA	A/New York/1796/2010(H1N1)
PA	JX309008	H1N1	01/15/2010	Singapore	A/Singapore/GP241/2010
PA	CY073967	H1N1	01/15/2010	Argentina	A/Argentina/HNRG102/2010
PA	JX309016	H1N1	01/15/2010	Singapore	A/Singapore/GP256/2010
PA	KT180937	H1N1	01/16/2010	Singapore	A/Singapore/DMS69/2010
PA	CY062095	H1N1	01/18/2010	USA	A/New York/1999/2010(H1N1)
PA	CY062087	H1N1	01/18/2010	USA	A/New York/1998/2010(H1N1)
PA	LN846437	H1N1	01/18/2010	United Ki	A/England/32/2010
PA	CY100521	H1N1	01/18/2010	Mexico	A/Mexico City/INER9/2010
PA	CY071300	H1N1	01/18/2010	Greece	A/Athens/INS417/2010(H1N1)
PA	CY080314	H1N1	01/18/2010	Thailand	A/Thailand/H1255/2010
PA	CY066468	H1N1	01/19/2010	USA	A/California/VRDL3/2010(H1N1)
PA	CY062103	H1N1	01/19/2010	USA	A/New York/2175/2010(H1N1)
PA	CY167777	H1N1	01/19/2010	USA	A/Tennessee/F1035/2010
PA	CY071156	H1N1	01/19/2010	Greece	A/Athens/INS391/2010(H1N1)
PA	JX308992	H1N1	01/20/2010	Singapore	A/Singapore/GP329/2010
PA	CY061160	H1N1	01/20/2010	USA	A/Texas/JMS409/2010(H1N1)
PA	CY062111	H1N1	01/20/2010	USA	A/New York/2372/2010(H1N1)
PA	CY062135	H1N1	01/20/2010	USA	A/New York/2876/2010(H1N1)
PA	CY062119	H1N1	01/20/2010	USA	A/New York/2598/2010(H1N1)
PA	JX309024	H1N1	01/21/2010	Singapore	A/Singapore/GP368/2010
PA	CY071172	H1N1	01/21/2010	Greece	A/Athens/INS396/2010(H1N1)
PA	KC780578	H1N1	01/21/2010	USA	A/Virginia/01/2010
PA	KT180939	H1N1	01/22/2010	Singapore	A/Singapore/DMS71/2010
PA	CY062151	H1N1	01/22/2010	USA	A/New York/2963/2010(H1N1)
PA	CY062127	H1N1	01/22/2010	USA	A/New York/2648/2010(H1N1)
PA	CY061168	H1N1	01/22/2010	USA	A/Texas/JMS410/2010(H1N1)
PA	CY062159	H1N1	01/23/2010	USA	A/New York/2971/2010(H1N1)
PA	CY071188	H1N1	01/24/2010	Greece	A/Athens/INS398/2010(H1N1)
PA	KC780495	H1N1	01/24/2010	USA	A/Wisconsin/01/2010
PA	CY071180	H1N1	01/24/2010	Greece	A/Athens/INS397/2010(H1N1)
PA	KT180940	H1N1	01/25/2010	Singapore	A/Singapore/DMS72/2010
PA	CY063208	H1N1	01/25/2010	USA	A/California/VRDL13/2010(H1N1)
PA	CY071308	H1N1	01/25/2010	Greece	A/Athens/INS419/2010(H1N1)
PA	JX309032	H1N1	01/25/2010	Singapore	A/Singapore/GP413/2010
PA	CY063216	H1N1	01/25/2010	USA	A/California/VRDL14/2010(H1N1)
PA	CY073125	H1N1	01/25/2010	Mexico	A/Mexico City/WRAIR1773T/2010(H1N
PA	CY062167	H1N1	01/25/2010	USA	A/New York/3230/2010(H1N1)
PA	JX309056	H1N1	01/25/2010	Singapore	A/Singapore/GP437/2010(H1N1)
PA	CY061176	H1N1	01/25/2010	USA	A/Texas/JMS411/2010(H1N1)

PA	JX309040	H1N1	01/25/2010	Singapore	A/Singapore/GP398/2010
PA	JX309048	H1N1	01/26/2010	Singapore	A/Singapore/GP449/2010(H1N1)
PA	CY063192	H1N1	01/26/2010	USA	A/California/VRDL11/2010(H1N1)
PA	CY062143	H1N1	01/26/2010	USA	A/New York/2960/2010(H1N1)
PA	CY062175	H1N1	01/26/2010	USA	A/New York/3236/2010(H1N1)
PA	CY062191	H1N1	01/26/2010	USA	A/New York/3251/2010(H1N1)
PA	CY083031	H1N1	01/26/2010	Senegal	A/Dakar/WRAIR0019T/2010
PA	CY080306	H1N1	01/26/2010	Thailand	A/Thailand/C602/2010(H1N1)
PA	CY083534	H1N1	01/26/2010	Mexico	A/Mexico City/WRAIR1752N/2010(H1N
PA	KC780657	H1N1	01/27/2010	USA	A/South Carolina/01/2010
PA	CY092643	H1N1	01/27/2010	Australia	A/Sydney/DD3-37/2010
PA	CY062183	H1N1	01/27/2010	USA	A/New York/3250/2010(H1N1)
PA	CY071316	H1N1	01/27/2010	Greece	A/Athens/INS420/2010(H1N1)
PA	CY065024	H1N1	01/27/2010	USA	A/New York/3834/2010(H1N1)
PA	CY065032	H1N1	01/27/2010	USA	A/New York/3835/2010(H1N1)
PA	JX309064	H1N1	01/27/2010	Singapore	A/Singapore/GP489/2010(H1N1)
PA	CY063200	H1N1	01/27/2010	USA	A/California/VRDL12/2010(H1N1)
PA	JX309072	H1N1	01/28/2010	Singapore	A/Singapore/GP512/2010(H1N1)
PA	CY065040	H1N1	01/29/2010	USA	A/New York/3866/2010(H1N1)
PA	CY071268	H1N1	01/30/2010	Greece	A/Athens/INS411/2010(H1N1)
PA	KT180943	H1N1	01/31/2010	Singapore	A/Singapore/DMS74/2010
PA	CY061184	H1N1	02/01/2010	USA	A/Texas/JMS412/2010(H1N1)
PA	CY065016	H1N1	02/01/2010	USA	A/New York/3683/2010(H1N1)
PA	CY065000	H1N1	02/01/2010	USA	A/New York/3681/2010(H1N1)
PA	KC780604	H1N1	02/01/2010	USA	A/California/02/2010
PA	CY065008	H1N1	02/01/2010	USA	A/New York/3682/2010(H1N1)
PA	CY066492	H1N1	02/01/2010	USA	A/California/VRDL7/2010(H1N1)
PA	JX309155	H1N1	02/02/2010	Singapore	A/Singapore/GP567/2010
PA	CY064992	H1N1	02/02/2010	USA	A/California/VRDL6/2010(H1N1)
PA	CY071276	H1N1	02/02/2010	Greece	A/Athens/INS412/2010(H1N1)
PA	CY063829	H1N1	02/02/2010	Singapore	A/Singapore/GP562/2010(H1N1)
PA	KT180952	H1N1	02/02/2010	Singapore	A/Singapore/DMS82/2010
PA	KC780536	H1N1	02/02/2010	USA	A/Florida/02/2010
PA	KT180944	H1N1	02/03/2010	Singapore	A/Singapore/DMS75/2010
PA	CY065056	H1N1	02/03/2010	USA	A/New York/4662/2010(H1N1)
PA	CY061192	H1N1	02/03/2010	USA	A/Texas/JMS413/2010(H1N1)
PA	CY073003	H1N1	02/03/2010	Nicaragua	A/Managua/07193_09/2010(H1N1)
PA	CY071324	H1N1	02/03/2010	Greece	A/Athens/INS421/2010(H1N1)
PA	KC780694	H1N1	02/03/2010	USA	A/Indiana/03/2010
PA	KC781454	H1N1	02/03/2010	USA	A/Louisiana/01/2010
PA	CY107452	H1N1	02/04/2010	United Ki	A/Scotland/Dundee_10V070791/2010
PA	CY061200	H1N1	02/04/2010	USA	A/Texas/JMS414/2010(H1N1)
PA	CY167329	H1N1	02/04/2010	USA	A/Tennessee/F1042A/2010
PA	KC782020	H1N1	02/04/2010	USA	A/Vermont/01/2010
PA	LN850029	H1N1	02/04/2010	United Ki	A/England/29/2010
PA	LN846493	H1N1	02/04/2010	United Ki	A/England/01220736/2010
PA	CY066484	H1N1	02/04/2010	USA	A/California/VRDL5/2010(H1N1)
PA	CY065048	H1N1	02/04/2010	USA	A/New York/4294/2010(H1N1)
PA	CY073738	H1N1	02/05/2010	Estonia	A/Tallinn/INS431/2010(H1N1)
PA	CY071380	H1N1	02/05/2010	Germany	A/Berlin/INS430/2010(H1N1)
PA	CY083822	H1N1	02/05/2010	USA	A/Los Angeles/INS423/2010(H1N1)
PA	KC781253	H1N1	02/05/2010	USA	A/Georgia/06/2010
PA	CY071284	H1N1	02/05/2010	Greece	A/Athens/INS413/2010(H1N1)
PA	CY071292	H1N1	02/05/2010	Greece	A/Athens/INS414/2010(H1N1)

PA	KT180946	H1N1	02/05/2010	Singapore	A/Singapore/DMS76/2010
PA	CY061208	H1N1	02/06/2010	USA	A/Texas/JMS415/2010(H1N1)
PA	KC780243	H1N1	02/06/2010	USA	A/Georgia/01/2010
PA	KC781261	H1N1	02/06/2010	USA	A/Georgia/04/2010
PA	KC781392	H1N1	02/07/2010	USA	A/Alaska/01/2010
PA	KP637285	H1N1	02/07/2010	Thailand	A/Thailand/IY07115/2010
PA	KC781185	H1N1	02/08/2010	USA	A/Wyoming/01/2010
PA	JX309166	H1N1	02/08/2010	Singapore	A/Singapore/TT106/2010
PA	KT180947	H1N1	02/08/2010	Singapore	A/Singapore/DMS77/2010
PA	CY066476	H1N1	02/08/2010	USA	A/California/VRDL4/2010(H1N1)
PA	CY090866	H1N1	02/09/2010	Netherlar	A/Netherlands/2631c/2010(H1N1)
PA	CY167337	H1N1	02/09/2010	USA	A/Tennessee/F1052A/2010
PA	CY100529	H1N1	02/09/2010	Mexico	A/Mexico City/INER10/2010
PA	KP637837	H1N1	02/09/2010	Thailand	A/Thailand/IY07144/2010
PA	CY066508	H1N1	02/09/2010	USA	A/California/VRDL9/2010(H1N1)
PA	CY100537	H1N1	02/09/2010	Mexico	A/Mexico City/INER11/2010
PA	KC261316	H1N1	02/09/2010	Mexico	A/Mexico City/INERPHAC3/2010(H1N1
PA	KC780456	H1N1	02/09/2010	USA	A/New York/03/2010
PA	KT180948	H1N1	02/10/2010	Singapore	A/Singapore/DMS78/2010
PA	KC780504	H1N1	02/10/2010	USA	A/Utah/02/2010
PA	CY063184	H1N1	02/11/2010	USA	A/California/VRDL10/2010(H1N1)
PA	JX309143	H1N1	02/12/2010	Singapore	A/Singapore/KK81/2010
PA	JX309149	H1N1	02/12/2010	Singapore	A/Singapore/GP737/2010
PA	KJ856113	H1N1	02/12/2010	Sri Lanka	A/Sri Lanka/20887/2010
PA	JF906182	H1N1	02/12/2010	Netherlar	A/Netherlands/2631_1202/2010
PA	CY066500	H1N1	02/13/2010	USA	A/California/VRDL8/2010(H1N1)
PA	CY083039	H1N1	02/16/2010	Senegal	A/Dakar/WRAIR0020T/2010
PA	CY097788	H1N1	02/16/2010	Senegal	A/Dakar/WRAIR0020T/2010
PA	KC780522	H1N1	02/17/2010	USA	A/Wisconsin/02/2010
PA	CY075080	H1N1	02/17/2010	Greece	A/Athens/INS416/2010(H1N1)
PA	KP637861	H1N1	02/18/2010	Thailand	A/Thailand/SS08248/2010
PA	CY167345	H1N1	02/18/2010	USA	A/Tennessee/F1057c56/2010
PA	CY167353	H1N1	02/18/2010	USA	A/Tennessee/F1060/2010
PA	LN867348	H1N1	02/20/2010	United Ki	A/England/01220741/2010
PA	KT180949	H1N1	02/20/2010	Singapore	A/Singapore/DMS80/2010
PA	CY065064	H1N1	02/21/2010	USA	A/New York/6530/2010(H1N1)
PA	KC782142	H1N1	02/23/2010	USA	A/Tennessee/01/2010
PA	CY071332	H1N1	02/23/2010	Thailand	A/Bangkok/INS424/2010(H1N1)
PA	CY096599	H1N1	02/23/2010	USA	A/District of Columbia/INS527/201
PA	KC780357	H1N1	02/24/2010	USA	A/North Carolina/05/2010
PA	CY129699	H1N1	02/24/2010	Greece	A/Athens/INS555/2010
PA	KT180951	H1N1	02/24/2010	Singapore	A/Singapore/DMS81/2010
PA	CY071340	H1N1	02/25/2010	Thailand	A/Bangkok/INS425/2010(H1N1)
PA	CY071348	H1N1	02/25/2010	Thailand	A/Bangkok/INS426/2010(H1N1)
PA	CY167369	H1N1	02/26/2010	USA	A/Tennessee/F1066c65/2010
PA	CY167377	H1N1	02/26/2010	USA	A/Tennessee/F1067/2010
PA	CY167361	H1N1	02/26/2010	USA	A/Tennessee/F1063A/2010
PA	CY063839	H1N1	02/26/2010	Singapore	A/Singapore/GP999/2010(H1N1)
PA	CY065112	H1N1	03/01/2010	USA	A/New York/7480/2010(H1N1)
PA	KC781166	H1N1	03/01/2010	USA	A/Alabama/03/2010
PA	KT180953	H1N1	03/01/2010	Singapore	A/Singapore/DMS83/2010
PA	CY167409	H1N1	03/02/2010	USA	A/Tennessee/F1072c71/2010
PA	CY167385	H1N1	03/02/2010	USA	A/Tennessee/F1068/2010
PA	HM124382	H1N1	03/02/2010	Russia	A/Orenburg/IIV-13/2010(H1N1)

PA	HM569739	H1N1	03/02/2010	Russia	A/Orenburg/IIV-13/2010(H1N1)
PA	CY167393	H1N1	03/02/2010	USA	A/Tennessee/F1071/2010
PA	KC781352	H1N1	03/03/2010	USA	A/Iowa/04/2010
PA	CY071356	H1N1	03/04/2010	Thailand	A/Bangkok/INS427/2010(H1N1)
PA	KC780742	H1N1	03/04/2010	USA	A/California/05/2010
PA	CY071364	H1N1	03/04/2010	Thailand	A/Bangkok/INS428/2010(H1N1)
PA	CY167401	H1N1	03/05/2010	USA	A/Tennessee/F1071B/2010
PA	CY071372	H1N1	03/05/2010	USA	A/Newark/INS429/2010(H1N1)
PA	KC781402	H1N1	03/08/2010	USA	A/Utah/03/2010
PA	KC781861	H1N1	03/08/2010	USA	A/Mississippi/02/2010
PA	JX309233	H1N1	03/10/2010	Singapor	A/Singapore/KK100/2010(H1N1)
PA	KC781396	H1N1	03/11/2010	USA	A/Nebraska/02/2010
PA	KT180956	H1N1	03/12/2010	Singapor	A/Singapore/DMS86/2010
PA	CY167417	H1N1	03/15/2010	USA	A/Tennessee/F1074/2010
PA	CY167441	H1N1	03/16/2010	USA	A/Tennessee/F1078/2010
PA	CY167425	H1N1	03/16/2010	USA	A/Tennessee/F1076/2010
PA	KT180959	H1N1	03/16/2010	Singapor	A/Singapore/DMS88/2010
PA	LN846786	H1N1	03/18/2010	United Ki	A/England/10/2010
PA	LN846533	H1N1	03/18/2010	United Ki	A/England/00380719/2010
PA	LN846589	H1N1	03/18/2010	United Ki	A/England/00380718/2010
PA	LN846509	H1N1	03/18/2010	United Ki	A/England/01180016/2010
PA	LN845773	H1N1	03/18/2010	United Ki	A/England/12/2010
PA	LN867396	H1N1	03/18/2010	United Ki	A/England/WTSI1783/2010
PA	LN867675	H1N1	03/18/2010	United Ki	A/England/WTSI2067/2010
PA	LN867422	H1N1	03/18/2010	United Ki	A/England/01180015/2010
PA	LN867372	H1N1	03/18/2010	United Ki	A/England/WTSI1774/2010
PA	LN867485	H1N1	03/18/2010	United Ki	A/England/01180017/2010
PA	LN867779	H1N1	03/18/2010	United Ki	A/England/01180011/2010
PA	LN867819	H1N1	03/18/2010	United Ki	A/England/WTSI2076/2010
PA	LN850124	H1N1	03/18/2010	United Ki	A/England/11/2010
PA	LN850344	H1N1	03/18/2010	United Ki	A/England/01180018/2010
PA	LN850336	H1N1	03/18/2010	United Ki	A/England/00380709/2010
PA	LN850077	H1N1	03/18/2010	United Ki	A/England/00380710/2010
PA	LN850101	H1N1	03/18/2010	United Ki	A/England/WTSI1807/2010
PA	LN850420	H1N1	03/18/2010	United Ki	A/England/01180007/2010
PA	CY089376	H1N1	03/19/2010	Nicaragua	A/Managua/5708.01/2010(H1N1)
PA	CY167449	H1N1	03/19/2010	USA	A/Tennessee/F1083c80/2010
PA	CY167433	H1N1	03/19/2010	USA	A/Tennessee/F1076A/2010
PA	CY167785	H1N1	03/19/2010	USA	A/Tennessee/F1080/2010
PA	CY080322	H1N1	03/20/2010	Thailand	A/Thailand/CU-H1786/2010(H1N1)
PA	KT180960	H1N1	03/20/2010	Singapor	A/Singapore/DMS89/2010
PA	CY096607	H1N1	03/21/2010	USA	A/Cambridge/INS528/2010(H1N1)
PA	JF265676	H1N1	03/22/2010	India	A/India/Blore/2010
PA	KT180961	H1N1	03/23/2010	Singapor	A/Singapore/DMS90/2010
PA	CY090842	H1N1	03/23/2010	Nicaragua	A/Managua/0305_10/2010(H1N1)
PA	KM013765	H1N1	03/23/2010	Thailand	A/Bangkok/SIMI508/2010
PA	CY167457	H1N1	03/24/2010	USA	A/Tennessee/F1089/2010
PA	KT180963	H1N1	03/25/2010	Singapor	A/Singapore/DMS91/2010
PA	CY089384	H1N1	03/25/2010	Nicaragua	A/Managua/3246.01/2010(H1N1)
PA	KC261317	H1N1	03/25/2010	Mexico	A/Mexico City/INERPHAC4/2010(H1N1
PA	LN867388	H1N1	03/26/2010	United Ki	A/England/01580005/2010
PA	CY080330	H1N1	03/29/2010	Thailand	A/Thailand/H1818/2010
PA	KT180965	H1N1	03/29/2010	Singapor	A/Singapore/DMS92/2010
PA	JX309222	H1N1	03/30/2010	Singapor	A/Singapore/GP1035/2010

PA	CY080338	H1N1	03/31/2010	Thailand	A/Thailand/CU-H1821/2010(H1N1)
PA	JQ340006	H1N2	08/24/2009	India	A/Eastern India/N-1289/2009

60

09

9(H1N1)

9(H1N1)
9(H1N1)

(H1N1)
(H1N1)
9(H1N1)

09

09

09

09

)
)

)

09

)
)

1)

1)
)

1)

1)

1)

)

1)
1)

1)

1)

1)

)

1)

RS-1649/2009

9

2009

09

1)

2/2009

1)

1)

H1N1)

(H1N1)

1N1)

09

(H1N1)

H1N1)
H1N1)

(H1N1)

H1N1)

(H1N1)

(H1N1)
(H1N1)
(H1N1)
(H1N1)
(H1N1)

(H1N1)

(H1N1)

(H1N1)

(H1N1)

(H1N1)

(H1N1)

(H1N1)

09

9(H1N1)

(H1N1)

(H1N1)

(H1N1)

9(H1N1)

9(H1N1)

9

N1)
(H1N1)

)

1)

9(H1N1)

9(H1N1)

9(H1N1)

1)

9
9(H1N1)

1)

1)

0(H1N1)

1)

1)

)

0(H1N1)

)

Name	Sequence Acc	Subtype	Collection Da	Country	Strain Name
NP	CY080613	H1N1	04/01/2009	USA	A/Boston/95/2009
NP	FJ966083	H1N1	04/01/2009	USA	A/California/04/2009(H1N1)
NP	FJ969512	H1N1	04/01/2009	USA	A/California/04/2009(H1N1)
NP	GQ149660	H1N1	04/03/2009	Mexico	A/Mexico/4108/2009(H1N1)
NP	GQ149667	H1N1	04/03/2009	Mexico	A/Mexico/4108/2009(H1N1)
NP	GQ149655	H1N1	04/03/2009	Mexico	A/Mexico/4108/2009(H1N1)
NP	CY077596	H1N1	04/03/2009	Mexico	A/Mexico/LaGloria-3/2009(H1N1)
NP	GQ149685	H1N1	04/03/2009	Mexico	A/Mexico/4108/2009(H1N1)
NP	GQ149693	H1N1	04/07/2009	Mexico	A/Mexico/4115/2009(H1N1)
NP	FJ969536	H1N1	04/09/2009	USA	A/California/07/2009(H1N1)
NP	FJ984366	H1N1	04/09/2009	USA	A/California/08/2009(H1N1)
NP	KC780063	H1N1	04/09/2009	USA	A/California/08/2009
NP	CY266194	H1N1	04/09/2009	USA	A/California/07/2009
NP	KU933486	H1N1	04/09/2009	USA	A/California/07/2009
NP	CY181404	H1N1	04/09/2009	USA	A/California/07-JR003/2009
NP	CY181412	H1N1	04/09/2009	USA	A/California/07-JR008/2009
NP	GQ338390	H1N1	04/09/2009	USA	A/California/07/2009(H1N1)
NP	CY181428	H1N1	04/09/2009	USA	A/California/07-JR010/2009
NP	CY181396	H1N1	04/09/2009	USA	A/California/07-JR002/2009
NP	CY181420	H1N1	04/09/2009	USA	A/California/07-JR009/2009
NP	GQ338345	H1N1	04/09/2009	USA	A/California/08/2009(H1N1)
NP	GQ303352	H1N1	04/13/2009	Mexico	A/Mexico/4176/2009(H1N1)
NP	CY147774	H1N1	04/14/2009	Mexico	A/Mexico/24033/2009
NP	CY044236	H1N1	04/14/2009	USA	A/San Antonio/PR921/2009(H1N1)
NP	GQ149645	H1N1	04/14/2009	Mexico	A/Mexico/4486/2009(H1N1)
NP	GQ149619	H1N1	04/14/2009	Mexico	A/Mexico/4486/2009(H1N1)
NP	GQ149678	H1N1	04/14/2009	Mexico	A/Mexico/4482/2009(H1N1)
NP	FJ966979	H1N1	04/14/2009	USA	A/Texas/04/2009(H1N1)
NP	FJ981618	H1N1	04/14/2009	USA	A/Texas/04/2009(H1N1)
NP	GQ303342	H1N1	04/14/2009	Mexico	A/Mexico/InDRE4487/2009(H1N1)
NP	GQ149673	H1N1	04/14/2009	Mexico	A/Mexico/4482/2009(H1N1)
NP	FJ998217	H1N1	04/14/2009	Mexico	A/Mexico/InDRE4487/2009
NP	GQ162184	H1N1	04/14/2009	Mexico	A/Mexico/4486/2009(H1N1)
NP	GQ303339	H1N1	04/15/2009	Mexico	A/Mexico/4269/2009(H1N1)
NP	GQ339880	H1N1	04/15/2009	Mexico	A/Mexico/4283/2009(H1N1)
NP	GQ122092	H1N1	04/15/2009	USA	A/Texas/15/2009(H1N1)
NP	GQ457484	H1N1	04/15/2009	USA	A/Texas/05/2009(H1N1)
NP	FJ966967	H1N1	04/15/2009	USA	A/Texas/05/2009(H1N1)
NP	FJ981609	H1N1	04/15/2009	USA	A/Texas/05/2009(H1N1)
NP	CY044244	H1N1	04/15/2009	USA	A/San Antonio/PR922/2009(H1N1)
NP	KC781722	H1N1	04/16/2009	USA	A/California/06/2009
NP	GQ402254	H1N1	04/16/2009	Canada	A/Canada-QC/RV1595/2009
NP	FJ966961	H1N1	04/16/2009	USA	A/California/06/2009(H1N1)
NP	CY147766	H1N1	04/17/2009	Mexico	A/Mexico/24032/2009
NP	CY147822	H1N1	04/17/2009	Mexico	A/Mexico/24043/2009
NP	GQ149637	H1N1	04/19/2009	Mexico	A/Mexico/4604/2009(H1N1)
NP	GQ149682	H1N1	04/19/2009	Mexico	A/Mexico/4603/2009(H1N1)
NP	GQ149639	H1N1	04/19/2009	Mexico	A/Mexico/4603/2009(H1N1)
NP	GQ149628	H1N1	04/19/2009	Mexico	A/Mexico/4603/2009(H1N1)
NP	GQ303353	H1N1	04/20/2009	Mexico	A/Mexico/4627/2009(H1N1)
NP	GQ463209	H1N1	04/20/2009	Mexico	A/Mexico/4595/2009(H1N1)
NP	GQ397122	H1N1	04/20/2009	Mexico	A/Mexico/4646/2009(H1N1)
NP	CY147830	H1N1	04/20/2009	Mexico	A/Mexico/24044/2009

NP	CY147838	H1N1	04/20/2009	Mexico	A/Mexico/24045/2009
NP	GQ339884	H1N1	04/20/2009	Mexico	A/Mexico/4635/2009(H1N1)
NP	GQ232011	H1N1	04/21/2009	USA	A/California/13/2009(H1N1)
NP	CY043095	H1N1	04/21/2009	USA	A/Brawley/40082/2009(H1N1)
NP	CY043087	H1N1	04/22/2009	USA	A/Brawley/40081/2009(H1N1)
NP	GQ117063	H1N1	04/22/2009	USA	A/Arizona/01/2009(H1N1)
NP	GQ117092	H1N1	04/22/2009	USA	A/Indiana/09/2009(H1N1)
NP	CY044252	H1N1	04/23/2009	USA	A/San Antonio/PR923/2009(H1N1)
NP	GQ232001	H1N1	04/23/2009	USA	A/Texas/10/2009(H1N1)
NP	FJ984384	H1N1	04/23/2009	USA	A/Texas/06/2009(H1N1)
NP	GQ232075	H1N1	04/23/2009	USA	A/Texas/11/2009(H1N1)
NP	CY147614	H1N1	04/23/2009	Mexico	A/Mexico/24007/2009
NP	CY147702	H1N1	04/23/2009	Mexico	A/Mexico/24021/2009
NP	FJ998216	H1N1	04/24/2009	Canada	A/Canada-NS/RV1535/2009
NP	CY041552	H1N1	04/24/2009	USA	A/New York/3210/2009(H1N1)
NP	CY065151	H1N1	04/24/2009	United Ki	A/Scotland/91800799/2009
NP	GQ168637	H1N1	04/24/2009	USA	A/Iowa/04/2009(H1N1)
NP	GQ117060	H1N1	04/24/2009	USA	A/Kansas/03/2009(H1N1)
NP	GQ338338	H1N1	04/24/2009	USA	A/Texas/12/2009(H1N1)
NP	GQ377091	H1N1	04/24/2009	USA	A/New York/25/2009(H1N1)
NP	FJ984363	H1N1	04/24/2009	USA	A/New York/23/2009(H1N1)
NP	GQ232018	H1N1	04/24/2009	USA	A/Texas/17/2009(H1N1)
NP	GQ117098	H1N1	04/24/2009	USA	A/Ohio/07/2009(H1N1)
NP	FJ984396	H1N1	04/24/2009	USA	A/Ohio/07/2009(H1N1)
NP	FJ998218	H1N1	04/24/2009	Canada	A/Canada-ON/RV1527/2009
NP	GQ232024	H1N1	04/24/2009	USA	A/Illinois/01/2009(H1N1)
NP	GQ117045	H1N1	04/24/2009	USA	A/Texas/08/2009(H1N1)
NP	GQ117019	H1N1	04/24/2009	USA	A/New York/22/2009(H1N1)
NP	GQ200248	H1N1	04/24/2009	USA	A/New York/21/2009(H1N1)
NP	GQ117057	H1N1	04/24/2009	USA	A/Kansas/02/2009(H1N1)
NP	GQ373262	H1N1	04/24/2009	Canada	A/Toronto/3141/2009(H1N1)
NP	GQ377056	H1N1	04/24/2009	USA	A/Ohio/07/2009(H1N1)
NP	FJ984358	H1N1	04/24/2009	USA	A/New York/31/2009(H1N1)
NP	CY040889	H1N1	04/25/2009	Mexico	A/Mexico/47N/2009(H1N1)
NP	CY064874	H1N1	04/25/2009	USA	A/California/VRDL146/2009(H1N1)
NP	CY074390	H1N1	04/25/2009	USA	A/California/VRDL264/2009(H1N1)
NP	GQ149648	H1N1	04/25/2009	Mexico	A/Mexico/4516/2009(H1N1)
NP	GQ117083	H1N1	04/25/2009	USA	A/New York/20/2009(H1N1)
NP	FJ984379	H1N1	04/25/2009	USA	A/New York/15/2009(H1N1)
NP	GQ117101	H1N1	04/25/2009	USA	A/Massachusetts/07/2009(H1N1)
NP	FJ984391	H1N1	04/25/2009	USA	A/New York/19/2009(H1N1)
NP	FJ984352	H1N1	04/25/2009	USA	A/New York/18/2009(H1N1)
NP	CY041125	H1N1	04/25/2009	USA	A/New York/3214/2009(H1N1)
NP	GQ232000	H1N1	04/25/2009	USA	A/New York/37/2009(H1N1)
NP	CY040640	H1N1	04/25/2009	USA	A/New York/3191/2009(H1N1)
NP	FJ984341	H1N1	04/25/2009	USA	A/New York/06/2009(H1N1)
NP	FJ984372	H1N1	04/25/2009	USA	A/New York/10/2009(H1N1)
NP	FJ984345	H1N1	04/25/2009	USA	A/New York/11/2009(H1N1)
NP	GQ117025	H1N1	04/25/2009	USA	A/Texas/09/2009(H1N1)
NP	GQ338407	H1N1	04/25/2009	USA	A/New York/09/2009(H1N1)
NP	GQ117087	H1N1	04/25/2009	USA	A/Texas/07/2009(H1N1)
NP	GQ117033	H1N1	04/25/2009	USA	A/California/14/2009(H1N1)
NP	GQ232062	H1N1	04/25/2009	USA	A/New York/18/2009(H1N1)
NP	GQ231994	H1N1	04/25/2009	USA	A/New York/35/2009(H1N1)

NP	GQ232016	H1N1	04/25/2009	USA	A/New York/39/2009(H1N1)
NP	GQ117113	H1N1	04/25/2009	USA	A/New York/13/2009(H1N1)
NP	GQ258466	H1N1	04/25/2009	New Zeala	A/Auckland/1/2009(H1N1)
NP	CY041133	H1N1	04/25/2009	USA	A/New York/3215/2009(H1N1)
NP	CY044164	H1N1	04/25/2009	Mexico	A/Mexico/48N/2009(H1N1)
NP	CY147678	H1N1	04/25/2009	Mexico	A/Mexico/24015/2009
NP	GQ117080	H1N1	04/25/2009	USA	A/New York/20/2009(H1N1)
NP	CY065143	H1N1	04/25/2009	United Ki	A/Scotland/91800797/2009
NP	CY086980	H1N1	04/25/2009	USA	A/New York/3217/2009(H1N1)
NP	CY097877	H1N1	04/25/2009	Mexico	A/Mexico/47N/2009
NP	GQ231998	H1N1	04/25/2009	USA	A/New York/33/2009(H1N1)
NP	CY041616	H1N1	04/25/2009	USA	A/New York/3002/2009(H1N1)
NP	CY041544	H1N1	04/25/2009	USA	A/New York/3209/2009(H1N1)
NP	GQ457480	H1N1	04/26/2009	USA	A/Nevada/03/2009(H1N1)
NP	GQ303356	H1N1	04/26/2009	Mexico	A/Mexico/6882/2009(H1N1)
NP	FJ985766	H1N1	04/26/2009	Spain	A/Castilla-La Mancha/GP9/2009(H1
NP	FJ985756	H1N1	04/26/2009	Spain	A/Valencia/GP4/2009(H1N1)
NP	GQ168618	H1N1	04/26/2009	USA	A/Texas/19/2009(H1N1)
NP	GQ117052	H1N1	04/26/2009	USA	A/South Carolina/09/2009(H1N1)
NP	CY041053	H1N1	04/26/2009	USA	A/New York/3166/2009(H1N1)
NP	CY041061	H1N1	04/26/2009	USA	A/New York/3168/2009(H1N1)
NP	CY041069	H1N1	04/26/2009	USA	A/New York/3169/2009(H1N1)
NP	CY041533	H1N1	04/26/2009	USA	A/New York/3205/2009(H1N1)
NP	CY041173	H1N1	04/26/2009	USA	A/New York/3186/2009(H1N1)
NP	GQ117041	H1N1	04/26/2009	USA	A/Massachusetts/06/2009(H1N1)
NP	GQ160569	H1N1	04/26/2009	USA	A/Texas/22/2009(H1N1)
NP	CY041101	H1N1	04/26/2009	USA	A/New York/3176/2009(H1N1)
NP	GQ117074	H1N1	04/26/2009	USA	A/Arizona/02/2009(H1N1)
NP	CY041737	H1N1	04/26/2009	USA	A/New York/3206/2009(H1N1)
NP	GQ396589	H1N1	04/26/2009	Spain	A/Madrid/GP62/2009(H1N1)
NP	CY074630	H1N1	04/26/2009	USA	A/California/VRDL371/2009(H1N1)
NP	CY074646	H1N1	04/26/2009	USA	A/California/VRDL378/2009(H1N1)
NP	FJ985761	H1N1	04/26/2009	Spain	A/Pais Vasco/GP20/2009(H1N1)
NP	GQ338408	H1N1	04/26/2009	USA	A/Washington/11/2009(H1N1)
NP	FJ985751	H1N1	04/26/2009	Spain	A/Castilla-La Mancha/GP13/2009(H
NP	CY039896	H1N1	04/26/2009	USA	A/New York/1669/2009(H1N1)
NP	CY074614	H1N1	04/26/2009	USA	A/California/VRDL366/2009(H1N1)
NP	CY092096	H1N1	04/26/2009	USA	A/California/VRDL372/2009
NP	GQ117107	H1N1	04/26/2009	USA	A/Michigan/02/2009(H1N1)
NP	GQ465713	H1N1	04/26/2009	Canada	A/Canada-PQ/RV1586/2009(H1N1)
NP	CY041608	H1N1	04/26/2009	USA	A/New York/3207/2009(H1N1)
NP	GQ160535	H1N1	04/26/2009	USA	A/New Mexico/04/2009(H1N1)
NP	CY069774	H1N1	04/27/2009	United Ki	A/England/209/2009
NP	CY087146	H1N1	04/27/2009	USA	A/California/VRDL137/2009
NP	CY074638	H1N1	04/27/2009	USA	A/California/VRDL377/2009(H1N1)
NP	CY040002	H1N1	04/27/2009	USA	A/New York/3008/2009(H1N1)
NP	CY064802	H1N1	04/27/2009	USA	A/California/VRDL134/2009(H1N1)
NP	CY074606	H1N1	04/27/2009	USA	A/California/VRDL365/2009(H1N1)
NP	CY073832	H1N1	04/27/2009	USA	A/California/VRDL155/2009(H1N1)
NP	GQ200271	H1N1	04/27/2009	USA	A/Wisconsin/09/2009(H1N1)
NP	CY040841	H1N1	04/27/2009	USA	A/New York/3262/2009(H1N1)
NP	GQ231983	H1N1	04/27/2009	USA	A/Missouri/01/2009(H1N1)
NP	CY041517	H1N1	04/27/2009	USA	A/New York/3203/2009(H1N1)
NP	CY073816	H1N1	04/27/2009	USA	A/California/VRDL153/2009(H1N1)

NP	CY040825	H1N1	04/27/2009	USA	A/New York/3260/2009(H1N1)
NP	GQ338398	H1N1	04/27/2009	USA	A/District of Columbia/03/2009(H
NP	CY074398	H1N1	04/27/2009	USA	A/California/VRDL267/2009(H1N1)
NP	GQ396517	H1N1	04/27/2009	Spain	A/Murcia/GP78/2009(H1N1)
NP	CY074126	H1N1	04/27/2009	USA	A/California/VRDL194/2009(H1N1)
NP	CY073808	H1N1	04/27/2009	USA	A/California/VRDL152/2009(H1N1)
NP	CY147846	H1N1	04/27/2009	Mexico	A/Mexico/24047/2009
NP	CY065239	H1N1	04/27/2009	United Ki	A/England/551/2009
NP	CY065199	H1N1	04/27/2009	United Ki	A/England/197/2009
NP	CY147710	H1N1	04/27/2009	Mexico	A/Mexico/24022/2009
NP	GQ160587	H1N1	04/27/2009	USA	A/Virginia/04/2009(H1N1)
NP	GQ160521	H1N1	04/27/2009	USA	A/Florida/04/2009(H1N1)
NP	GQ160603	H1N1	04/27/2009	USA	A/Wisconsin/08/2009(H1N1)
NP	CY040010	H1N1	04/27/2009	USA	A/New York/3012/2009(H1N1)
NP	CY147854	H1N1	04/27/2009	Mexico	A/Mexico/24048/2009
NP	GQ168662	H1N1	04/27/2009	USA	A/Washington/12/2009(H1N1)
NP	CY147870	H1N1	04/27/2009	Mexico	A/Mexico/24050/2009
NP	CY074662	H1N1	04/27/2009	USA	A/California/VRDL380/2009(H1N1)
NP	CY147886	H1N1	04/27/2009	Mexico	A/Mexico/24052/2009
NP	CY074670	H1N1	04/27/2009	USA	A/California/VRDL382/2009(H1N1)
NP	CY074622	H1N1	04/27/2009	USA	A/California/VRDL369/2009(H1N1)
NP	CY050479	H1N1	04/27/2009	USA	A/New York/1692/2009(H1N1)
NP	CY074654	H1N1	04/27/2009	USA	A/California/VRDL379/2009(H1N1)
NP	CY065215	H1N1	04/27/2009	United Ki	A/England/205/2009
NP	CY065247	H1N1	04/27/2009	United Ki	A/England/XFL00071/2009
NP	CY065223	H1N1	04/27/2009	United Ki	A/England/207/2009
NP	CY065191	H1N1	04/27/2009	United Ki	A/England/202/2009
NP	CY080621	H1N1	04/27/2009	USA	A/Boston/100/2009
NP	CY087154	H1N1	04/27/2009	USA	A/California/VRDL148/2009
NP	CY069766	H1N1	04/27/2009	United Ki	A/England/196/2009
NP	CY050663	H1N1	04/27/2009	USA	A/New York/3150/2009
NP	GQ402251	H1N1	04/27/2009	Mexico	A/Mexico/InDRE13547/2009
NP	CY147718	H1N1	04/27/2009	Mexico	A/Mexico/24024/2009
NP	CY147902	H1N1	04/27/2009	Mexico	A/Mexico/24054/2009
NP	CY147782	H1N1	04/27/2009	Mexico	A/Mexico/24036/2009
NP	CY147878	H1N1	04/27/2009	Mexico	A/Mexico/24051/2009
NP	CY147894	H1N1	04/27/2009	Mexico	A/Mexico/24053/2009
NP	CY147862	H1N1	04/27/2009	Mexico	A/Mexico/24049/2009
NP	GQ396739	H1N1	04/27/2009	USA	A/Texas/23/2009(H1N1)
NP	GQ232021	H1N1	04/27/2009	USA	A/District of Columbia/01/2009(H
NP	CY041568	H1N1	04/27/2009	USA	A/New York/3225/2009(H1N1)
NP	GQ338352	H1N1	04/27/2009	USA	A/Oregon/04/2009(H1N1)
NP	GQ338331	H1N1	04/27/2009	USA	A/Connecticut/01/2009(H1N1)
NP	GQ465714	H1N1	04/27/2009	Canada	A/Canada-ON/RV1589/2009(H1N1)
NP	CY064834	H1N1	04/27/2009	USA	A/California/VRDL140/2009(H1N1)
NP	GQ894824	H1N1	04/27/2009	USA	A/Maine/01/2009(H1N1)
NP	GQ894931	H1N1	04/27/2009	USA	A/Alabama/02/2009(H1N1)
NP	CY064810	H1N1	04/27/2009	USA	A/California/VRDL135/2009(H1N1)
NP	CY070906	H1N1	04/27/2009	USA	A/California/VRDL150/2009(H1N1)
NP	CY070898	H1N1	04/27/2009	USA	A/California/VRDL149/2009(H1N1)
NP	CY040018	H1N1	04/27/2009	USA	A/New York/3014/2009(H1N1)
NP	CY040026	H1N1	04/27/2009	USA	A/New York/3049/2009(H1N1)
NP	CY040045	H1N1	04/27/2009	USA	A/New York/3007/2009(H1N1)
NP	CY039904	H1N1	04/27/2009	USA	A/New York/1682/2009(H1N1)

NP	GQ200235	H1N1	04/27/2009	USA	A/Georgia/01/2009(H1N1)
NP	GQ117068	H1N1	04/27/2009	USA	A/Minnesota/02/2009(H1N1)
NP	GQ160595	H1N1	04/27/2009	USA	A/Nebraska/03/2009(H1N1)
NP	GQ117117	H1N1	04/27/2009	USA	A/Colorado/03/2009(H1N1)
NP	CY041085	H1N1	04/27/2009	USA	A/New York/3172/2009(H1N1)
NP	CY041525	H1N1	04/27/2009	USA	A/New York/3204/2009(H1N1)
NP	GQ160575	H1N1	04/28/2009	USA	A/Washington/09/2009(H1N1)
NP	GQ160580	H1N1	04/28/2009	USA	A/New Hampshire/02/2009(H1N1)
NP	GQ160583	H1N1	04/28/2009	USA	A/North Carolina/05/2009(H1N1)
NP	GQ160592	H1N1	04/28/2009	USA	A/Delaware/02/2009(H1N1)
NP	GQ168621	H1N1	04/28/2009	USA	A/Washington/07/2009(H1N1)
NP	GQ168629	H1N1	04/28/2009	USA	A/North Carolina/06/2009(H1N1)
NP	GQ168634	H1N1	04/28/2009	USA	A/Delaware/04/2009(H1N1)
NP	GQ232036	H1N1	04/28/2009	USA	A/Arizona/06/2009(H1N1)
NP	CY041205	H1N1	04/28/2009	USA	A/New York/3199/2009(H1N1)
NP	CY147790	H1N1	04/28/2009	Mexico	A/Mexico/24038/2009
NP	CY065255	H1N1	04/28/2009	United Ki	A/England/211/2009
NP	GQ160539	H1N1	04/28/2009	USA	A/Delaware/03/2009(H1N1)
NP	GQ200206	H1N1	04/28/2009	USA	A/Oregon/06/2009(H1N1)
NP	CY040720	H1N1	04/28/2009	USA	A/New York/3238/2009(H1N1)
NP	CY083305	H1N1	04/28/2009	USA	A/San Diego/WRAIR1648P/2009
NP	CY147742	H1N1	04/28/2009	Mexico	A/Mexico/24027/2009
NP	CY073856	H1N1	04/28/2009	USA	A/California/VRDL175/2009(H1N1)
NP	CY073824	H1N1	04/28/2009	USA	A/California/VRDL154/2009(H1N1)
NP	CY147734	H1N1	04/28/2009	Mexico	A/Mexico/24026/2009
NP	CY092348	H1N1	04/28/2009	USA	A/California/VRDL191/2009
NP	CY065207	H1N1	04/28/2009	United Ki	A/England/204/2009
NP	GQ166658	H1N1	04/28/2009	United Ki	A/England/195/2009
NP	CY147750	H1N1	04/28/2009	Mexico	A/Mexico/24028/2009
NP	CY147926	H1N1	04/28/2009	Mexico	A/Mexico/24057/2009
NP	CY147934	H1N1	04/28/2009	Mexico	A/Mexico/24058/2009
NP	GQ160528	H1N1	04/28/2009	USA	A/Tennessee/05/2009(H1N1)
NP	CY041584	H1N1	04/28/2009	USA	A/New York/3230/2009(H1N1)
NP	CY041833	H1N1	04/28/2009	USA	A/New York/3348/2009(H1N1)
NP	GQ338397	H1N1	04/28/2009	USA	A/Hawaii/08/2009(H1N1)
NP	GQ465709	H1N1	04/28/2009	Canada	A/Canada-AB/RV1531/2009(H1N1)
NP	GQ396541	H1N1	04/28/2009	Spain	A/Madrid/GP193/2009(H1N1)
NP	CY047329	H1N1	04/28/2009	USA	A/New York/3167/2009(H1N1)
NP	CY074150	H1N1	04/28/2009	USA	A/California/VRDL206/2009(H1N1)
NP	CY050068	H1N1	04/28/2009	USA	A/Cherry Point/WR1355/2009(H1N1)
NP	GQ464409	H1N1	04/28/2009	Spain	A/Catalonia/63/2009(H1N1)
NP	CY041493	H1N1	04/28/2009	USA	A/New York/3184/2009(H1N1)
NP	CY147918	H1N1	04/28/2009	Mexico	A/Mexico/24056/2009
NP	GQ232040	H1N1	04/28/2009	USA	A/Rhode Island/02/2009(H1N1)
NP	GQ232056	H1N1	04/28/2009	USA	A/Delaware/08/2009(H1N1)
NP	CY041501	H1N1	04/28/2009	USA	A/New York/3195/2009(H1N1)
NP	CY070914	H1N1	04/28/2009	USA	A/California/VRDL151/2009(H1N1)
NP	CY147942	H1N1	04/28/2009	Mexico	A/Mexico/24060/2009
NP	CY064842	H1N1	04/28/2009	USA	A/California/VRDL141/2009(H1N1)
NP	CY074110	H1N1	04/28/2009	USA	A/California/VRDL192/2009(H1N1)
NP	CY040704	H1N1	04/28/2009	USA	A/New York/3235/2009(H1N1)
NP	CY040632	H1N1	04/28/2009	USA	A/New York/3234/2009(H1N1)
NP	CY040712	H1N1	04/28/2009	USA	A/New York/3237/2009(H1N1)
NP	CY041117	H1N1	04/28/2009	USA	A/New York/3193/2009(H1N1)

NP	GQ221828	H1N1	04/28/2009	USA	A/Idaho/02/2009(H1N1)
NP	GQ221792	H1N1	04/28/2009	USA	A/Oklahoma/01/2009(H1N1)
NP	CY041093	H1N1	04/28/2009	USA	A/New York/3173/2009(H1N1)
NP	CY041109	H1N1	04/28/2009	USA	A/New York/3178/2009(H1N1)
NP	CY041157	H1N1	04/28/2009	USA	A/New York/3183/2009(H1N1)
NP	CY041189	H1N1	04/28/2009	USA	A/New York/3188/2009(H1N1)
NP	CY041149	H1N1	04/28/2009	USA	A/New York/3181/2009(H1N1)
NP	CY073395	H1N1	04/28/2009	USA	A/Brawley/WR1635P/2009(H1N1)
NP	CY073848	H1N1	04/28/2009	USA	A/California/VRDL167/2009(H1N1)
NP	GQ402252	H1N1	04/28/2009	Mexico	A/Mexico/InDRE13551/2009
NP	CY041509	H1N1	04/28/2009	USA	A/New York/3196/2009(H1N1)
NP	CY041576	H1N1	04/28/2009	USA	A/New York/3227/2009(H1N1)
NP	CY041592	H1N1	04/28/2009	USA	A/New York/3236/2009(H1N1)
NP	CY041560	H1N1	04/28/2009	USA	A/New York/3211/2009(H1N1)
NP	CY074174	H1N1	04/28/2009	USA	A/California/VRDL215/2009(H1N1)
NP	CY074086	H1N1	04/28/2009	USA	A/California/VRDL180/2009(H1N1)
NP	GQ396578	H1N1	04/28/2009	Spain	A/Valencia/GP154/2009(H1N1)
NP	CY147910	H1N1	04/28/2009	Mexico	A/Mexico/24055/2009
NP	CY046334	H1N1	04/28/2009	USA	A/Wisconsin/629-D01505/2009(H1N1
NP	CY047321	H1N1	04/28/2009	USA	A/New York/3124/2009(H1N1)
NP	CY053074	H1N1	04/28/2009	USA	A/Houston/130S/2009(H1N1)
NP	CY147950	H1N1	04/28/2009	Mexico	A/Mexico/24061/2009
NP	CY065231	H1N1	04/28/2009	United Ki	A/England/200/2009
NP	CY074302	H1N1	04/29/2009	USA	A/California/VRDL237/2009(H1N1)
NP	CY047313	H1N1	04/29/2009	USA	A/New York/3074/2009(H1N1)
NP	CY050767	H1N1	04/29/2009	USA	A/New York/3315/2009(H1N1)
NP	CY074318	H1N1	04/29/2009	USA	A/California/VRDL239/2009(H1N1)
NP	CY074214	H1N1	04/29/2009	USA	A/California/VRDL221/2009(H1N1)
NP	GQ402249	H1N1	04/29/2009	Mexico	A/Mexico/InDRE13494/2009
NP	CY041485	H1N1	04/29/2009	USA	A/New York/3189/2009(H1N1)
NP	CY074142	H1N1	04/29/2009	USA	A/California/VRDL202/2009(H1N1)
NP	CY040034	H1N1	04/29/2009	USA	A/New York/3099/2009(H1N1)
NP	GQ160561	H1N1	04/29/2009	USA	A/Maryland/05/2009(H1N1)
NP	GQ214153	H1N1	04/29/2009	France	A/Paris/2573/2009(H1N1)
NP	GQ232013	H1N1	04/29/2009	USA	A/Texas/28/2009(H1N1)
NP	HM567553	H1N1	04/29/2009	United Ki	A/England/213/2009
NP	GQ168653	H1N1	04/29/2009	USA	A/Oklahoma/02/2009(H1N1)
NP	GQ168646	H1N1	04/29/2009	USA	A/Nevada/05/2009(H1N1)
NP	GQ168625	H1N1	04/29/2009	USA	A/District of Columbia/02/2009(H
NP	CY074366	H1N1	04/29/2009	USA	A/California/VRDL254/2009(H1N1)
NP	CY074262	H1N1	04/29/2009	USA	A/California/VRDL227/2009(H1N1)
NP	CY074198	H1N1	04/29/2009	USA	A/California/VRDL219/2009(H1N1)
NP	CY073864	H1N1	04/29/2009	USA	A/California/VRDL229/2009(H1N1)
NP	CY074254	H1N1	04/29/2009	USA	A/California/VRDL226/2009(H1N1)
NP	CY074374	H1N1	04/29/2009	USA	A/California/VRDL256/2009(H1N1)
NP	CY074158	H1N1	04/29/2009	USA	A/California/VRDL209/2009(H1N1)
NP	CY074094	H1N1	04/29/2009	USA	A/California/VRDL186/2009(H1N1)
NP	CY074238	H1N1	04/29/2009	USA	A/California/VRDL224/2009(H1N1)
NP	CY074166	H1N1	04/29/2009	USA	A/California/VRDL213/2009(H1N1)
NP	CY074182	H1N1	04/29/2009	USA	A/California/VRDL217/2009(H1N1)
NP	CY074350	H1N1	04/29/2009	USA	A/California/VRDL251/2009(H1N1)
NP	CY074358	H1N1	04/29/2009	USA	A/California/VRDL252/2009(H1N1)
NP	CY074222	H1N1	04/29/2009	USA	A/California/VRDL222/2009(H1N1)
NP	CY086972	H1N1	04/29/2009	USA	A/New York/3198/2009(H1N1)

NP	CY046943	H1N1	04/29/2009	Netherlar	A/Netherlands/602/2009
NP	CY065263	H1N1	04/29/2009	United Ki	A/England/XFL00137/2009
NP	GQ402250	H1N1	04/29/2009	Mexico	A/Mexico/InDRE13495/2009
NP	CY147798	H1N1	04/29/2009	Mexico	A/Mexico/24039/2009
NP	CY147806	H1N1	04/29/2009	Mexico	A/Mexico/24040/2009
NP	GQ396564	H1N1	04/29/2009	Spain	A/Valencia/GP272/2009(H1N1)
NP	CY065343	H1N1	04/29/2009	United Ki	A/England/223/2009
NP	CY041165	H1N1	04/29/2009	USA	A/New York/3185/2009(H1N1)
NP	GQ338389	H1N1	04/29/2009	USA	A/Pennsylvania/06/2009(H1N1)
NP	GQ457492	H1N1	04/29/2009	USA	A/Nevada/04/2009(H1N1)
NP	GQ465710	H1N1	04/29/2009	Canada	A/Canada-NS/RV1551/2009(H1N1)
NP	GQ465711	H1N1	04/29/2009	Canada	A/Canada-NS/RV1554/2009(H1N1)
NP	GQ396569	H1N1	04/29/2009	Spain	A/Valencia/GP278/2009(H1N1)
NP	GQ396594	H1N1	04/29/2009	Spain	A/Andalucia/GP251/2009(H1N1)
NP	CY046302	H1N1	04/29/2009	USA	A/Wisconsin/629-D01486/2009(H1N1
NP	CY046310	H1N1	04/29/2009	USA	A/Wisconsin/629-D01642/2009(H1N1
NP	CY050551	H1N1	04/29/2009	USA	A/New York/3095/2009(H1N1)
NP	CY073403	H1N1	04/29/2009	USA	A/Brawley/WR1636P/2009(H1N1)
NP	CY074334	H1N1	04/29/2009	USA	A/California/VRDL245/2009(H1N1)
NP	CY041197	H1N1	04/29/2009	USA	A/New York/3190/2009(H1N1)
NP	CY147758	H1N1	04/29/2009	Mexico	A/Mexico/24030/2009
NP	CY074118	H1N1	04/29/2009	USA	A/California/VRDL193/2009(H1N1)
NP	CY074190	H1N1	04/29/2009	USA	A/California/VRDL218/2009(H1N1)
NP	CY073840	H1N1	04/29/2009	USA	A/California/VRDL163/2009
NP	CY074230	H1N1	04/29/2009	USA	A/California/VRDL223/2009(H1N1)
NP	CY074206	H1N1	04/29/2009	USA	A/California/VRDL220/2009(H1N1)
NP	CY074342	H1N1	04/29/2009	USA	A/California/VRDL249/2009(H1N1)
NP	CY040865	H1N1	04/29/2009	USA	A/New York/3265/2009(H1N1)
NP	CY040857	H1N1	04/29/2009	USA	A/New York/3264/2009(H1N1)
NP	CY040833	H1N1	04/29/2009	USA	A/New York/3261/2009(H1N1)
NP	CY041729	H1N1	04/29/2009	USA	A/New York/3247/2009(H1N1)
NP	CY074102	H1N1	04/29/2009	USA	A/California/VRDL189/2009(H1N1)
NP	CY074294	H1N1	04/29/2009	USA	A/California/VRDL236/2009(H1N1)
NP	CY074270	H1N1	04/29/2009	USA	A/California/VRDL228/2009(H1N1)
NP	GQ396523	H1N1	04/29/2009	Spain	A/Galicia/GP224/2009(H1N1)
NP	CY092340	H1N1	04/29/2009	USA	A/California/VRDL190/2009
NP	GQ402253	H1N1	04/29/2009	Mexico	A/Mexico/InDRE13555/2009
NP	CY074286	H1N1	04/29/2009	USA	A/California/VRDL235/2009(H1N1)
NP	CY074310	H1N1	04/29/2009	USA	A/California/VRDL238/2009(H1N1)
NP	GQ396546	H1N1	04/29/2009	Spain	A/CastillaLaMancha/GP248/2009(H1
NP	GQ338354	H1N1	04/30/2009	USA	A/Kentucky/05/2009(H1N1)
NP	GQ232053	H1N1	04/30/2009	USA	A/South Dakota/05/2009(H1N1)
NP	HM567561	H1N1	04/30/2009	United Ki	A/England/217/2009
NP	CY046846	H1N1	04/30/2009	USA	A/Wisconsin/629-D02361/2009(H1N1
NP	CY074454	H1N1	04/30/2009	USA	A/California/VRDL283/2009(H1N1)
NP	CY074526	H1N1	04/30/2009	USA	A/California/VRDL300/2009(H1N1)
NP	CY074494	H1N1	04/30/2009	USA	A/California/VRDL295/2009(H1N1)
NP	CY040648	H1N1	04/30/2009	USA	A/New York/3194/2009(H1N1)
NP	GQ214147	H1N1	04/30/2009	France	A/Paris/2580/2009(H1N1)
NP	GQ232032	H1N1	04/30/2009	USA	A/Louisiana/04/2009(H1N1)
NP	CY074534	H1N1	04/30/2009	USA	A/California/VRDL301/2009(H1N1)
NP	CY073367	H1N1	04/30/2009	USA	A/San Diego/WR1631P/2009(H1N1)
NP	CY074406	H1N1	04/30/2009	USA	A/California/VRDL270/2009(H1N1)
NP	CY041624	H1N1	04/30/2009	USA	A/New York/3100/2009(H1N1)

NP	CY074414	H1N1	04/30/2009	USA	A/California/VRDL273/2009(H1N1)
NP	CY074486	H1N1	04/30/2009	USA	A/California/VRDL292/2009(H1N1)
NP	CY046422	H1N1	04/30/2009	USA	A/Wisconsin/629-D00767/2009(H1N1
NP	CY046414	H1N1	04/30/2009	USA	A/Wisconsin/629-D00447/2009(H1N1
NP	CY046390	H1N1	04/30/2009	USA	A/Wisconsin/629-D00750/2009(H1N1
NP	CY046478	H1N1	04/30/2009	USA	A/Wisconsin/629-D00015/2009(H1N1
NP	CY065287	H1N1	04/30/2009	United Ki	A/England/216/2009
NP	CY073351	H1N1	04/30/2009	USA	A/San Diego/WR1629P/2009(H1N1)
NP	CY065279	H1N1	04/30/2009	United Ki	A/England/215/2009
NP	CY074510	H1N1	04/30/2009	USA	A/California/VRDL298/2009(H1N1)
NP	CY074422	H1N1	04/30/2009	USA	A/California/VRDL275/2009(H1N1)
NP	CY074478	H1N1	04/30/2009	USA	A/California/VRDL290/2009(H1N1)
NP	CY074326	H1N1	04/30/2009	USA	A/California/VRDL244/2009(H1N1)
NP	GU931813	H1N1	04/30/2009	Hong Kong	A/Hong Kong/415742/2009(H1N1)
NP	CY065271	H1N1	04/30/2009	United Ki	A/England/214/2009
NP	CY064711	H1N1	04/30/2009	Mexico	A/Mexico City/020/2009
NP	CY064703	H1N1	04/30/2009	Mexico	A/Mexico City/019/2009
NP	CY064743	H1N1	04/30/2009	Mexico	A/Mexico City/024/2009(H1N1)
NP	GQ465712	H1N1	04/30/2009	Canada	A/Canada-NS/RV1565/2009(H1N1)
NP	GQ465717	H1N1	04/30/2009	Canada	A/Canada-SK/RV1788/2009(H1N1)
NP	CY047353	H1N1	04/30/2009	USA	A/New York/3365/2009(H1N1)
NP	CY046358	H1N1	04/30/2009	USA	A/Wisconsin/629-D01529/2009(H1N1
NP	CY046518	H1N1	04/30/2009	USA	A/Wisconsin/629-D02141/2009(H1N1
NP	CY046262	H1N1	04/30/2009	USA	A/Wisconsin/629-D01773/2009(H1N1
NP	CY046454	H1N1	04/30/2009	USA	A/Wisconsin/629-D01558/2009(H1N1
NP	CY049829	H1N1	04/30/2009	USA	A/Cherry Point/WR0080/2009(H1N1)
NP	CY074430	H1N1	04/30/2009	USA	A/California/VRDL276/2009(H1N1)
NP	CY074438	H1N1	04/30/2009	USA	A/California/VRDL280/2009(H1N1)
NP	CY074502	H1N1	04/30/2009	USA	A/California/VRDL297/2009(H1N1)
NP	CY074134	H1N1	04/30/2009	USA	A/California/VRDL200/2009(H1N1)
NP	GQ214141	H1N1	04/30/2009	France	A/Paris/2590/2009(H1N1)
NP	CY040696	H1N1	04/30/2009	USA	A/New York/3232/2009(H1N1)
NP	GQ232051	H1N1	04/30/2009	USA	A/Ohio/10/2009(H1N1)
NP	GQ377034	H1N1	05/2009	USA	A/Alaska/04/2009(H1N1)
NP	CY107186	H1N1	05/2009	United Ki	A/Scotland/Paisley_409499/2009
NP	CY107559	H1N1	05/2009	United Ki	A/Scotland/Glasgow_412118/2009
NP	CY039987	H1N1	05/2009	Thailand	A/Nonthaburi/102/2009
NP	GQ465715	H1N1	05/01/2009	Canada	A/Canada-AB/RV1644/2009(H1N1)
NP	CY074382	H1N1	05/01/2009	USA	A/California/VRDL258/2009(H1N1)
NP	CY046494	H1N1	05/01/2009	USA	A/Wisconsin/629-D02028/2009(H1N1
NP	CY046294	H1N1	05/01/2009	USA	A/Wisconsin/629-D01817/2009(H1N1
NP	CY046462	H1N1	05/01/2009	USA	A/Wisconsin/629-D01861/2009(H1N1
NP	CY053090	H1N1	05/01/2009	USA	A/Houston/14H/2009(H1N1)
NP	CY064751	H1N1	05/01/2009	Mexico	A/Mexico City/025/2009(H1N1)
NP	CY064735	H1N1	05/01/2009	Mexico	A/Mexico City/023/2009(H1N1)
NP	GQ214158	H1N1	05/01/2009	France	A/Paris/2592/2009(H1N1)
NP	GQ231986	H1N1	05/01/2009	USA	A/Missouri/02/2009(H1N1)
NP	GQ232066	H1N1	05/01/2009	USA	A/Louisiana/03/2009(H1N1)
NP	CY041640	H1N1	05/01/2009	USA	A/New York/3307/2009(H1N1)
NP	CY073379	H1N1	05/01/2009	USA	A/San Diego/WR1633P/2009(H1N1)
NP	GQ254711	H1N1	05/01/2009	France	A/Paris/2591/2009(H1N1)
NP	CY046430	H1N1	05/01/2009	USA	A/Wisconsin/629-D00677/2009(H1N1
NP	CY064719	H1N1	05/01/2009	Mexico	A/Mexico City/021/2009
NP	CY074470	H1N1	05/01/2009	USA	A/California/VRDL289/2009(H1N1)

NP	CY074518	H1N1	05/01/2009	USA	A/California/VRDL299/2009(H1N1)
NP	CY046190	H1N1	05/01/2009	USA	A/Wisconsin/629-D01793/2009(H1N1
NP	CY046318	H1N1	05/01/2009	USA	A/Wisconsin/629-D01308/2009(H1N1
NP	CY041181	H1N1	05/01/2009	USA	A/New York/3187/2009(H1N1)
NP	CY053082	H1N1	05/01/2009	USA	A/Houston/140S/2009H1N1)
NP	CY053106	H1N1	05/01/2009	USA	A/Houston/15H/2009(H1N1)
NP	CY053098	H1N1	05/01/2009	USA	A/Houston/150S/2009(H1N1)
NP	CY046974	H1N1	05/01/2009	USA	A/New York/3443/2009(H1N1)
NP	CY047345	H1N1	05/01/2009	USA	A/New York/3345/2009(H1N1)
NP	GQ396534	H1N1	05/01/2009	Spain	A/CastillaLaMancha/GP369/2009(H1
NP	GQ365667	H1N1	05/02/2009	Germany	A/Brandenburg/19/2009(H1N1)
NP	CY041809	H1N1	05/02/2009	USA	A/New York/3408/2009(H1N1)
NP	CY046198	H1N1	05/02/2009	USA	A/Wisconsin/629-D02312/2009(H1N1
NP	CY046854	H1N1	05/02/2009	USA	A/Wisconsin/629-D00244/2009(H1N1
NP	CY046382	H1N1	05/02/2009	USA	A/Wisconsin/629-D01980/2009(H1N1
NP	CY046438	H1N1	05/02/2009	USA	A/Wisconsin/629-D02262/2009(H1N1
NP	CY065335	H1N1	05/02/2009	United Ki	A/England/201/2009
NP	CY041077	H1N1	05/02/2009	USA	A/New York/3170/2009(H1N1)
NP	CY041600	H1N1	05/02/2009	USA	A/New York/3177/2009(H1N1)
NP	GQ323503	H1N1	05/02/2009	USA	A/Michigan/06/2009(H1N1)
NP	CY074542	H1N1	05/02/2009	USA	A/California/VRDL311/2009(H1N1)
NP	CY073427	H1N1	05/02/2009	USA	A/San Diego/WR1641P/2009(H1N1)
NP	CY064727	H1N1	05/02/2009	Mexico	A/Mexico City/022/2009
NP	GQ131024	H1N1	05/02/2009	South Ko	A/Korea/01/2009
NP	CY065303	H1N1	05/02/2009	United Ki	A/England/218/2009
NP	CY065319	H1N1	05/02/2009	United Ki	A/England/219/2009
NP	CY073387	H1N1	05/02/2009	USA	A/San Diego/WR1634P/2009(H1N1)
NP	CY065311	H1N1	05/02/2009	United Ki	A/England/246/2009
NP	CY065327	H1N1	05/02/2009	United Ki	A/England/220/2009
NP	CY069782	H1N1	05/02/2009	United Ki	A/England/221/2009
NP	CY046238	H1N1	05/02/2009	USA	A/Wisconsin/629-D01735/2009(H1N1
NP	CY046206	H1N1	05/02/2009	USA	A/Wisconsin/629-D02408/2009(H1N1
NP	CY046398	H1N1	05/02/2009	USA	A/Wisconsin/629-D01189/2009(H1N1
NP	HM567569	H1N1	05/02/2009	United Ki	A/England/221/2009
NP	CY049860	H1N1	05/02/2009	USA	A/Cherry Point/WR0100/2009(H1N1)
NP	CY049868	H1N1	05/02/2009	USA	A/Cherry Point/WR0101/2009(H1N1)
NP	CY074462	H1N1	05/02/2009	USA	A/California/VRDL284/2009(H1N1)
NP	CY046270	H1N1	05/02/2009	USA	A/Wisconsin/629-D00933/2009(H1N1
NP	CY049876	H1N1	05/02/2009	USA	A/Cherry Point/WR0102/2009(H1N1)
NP	CY064759	H1N1	05/02/2009	Mexico	A/Mexico City/026/2009(H1N1)
NP	CY046342	H1N1	05/02/2009	USA	A/Wisconsin/629-D02455/2009(H1N1
NP	CY046350	H1N1	05/03/2009	USA	A/Wisconsin/629-D01017/2009(H1N1
NP	CY046246	H1N1	05/03/2009	USA	A/Wisconsin/629-D01606/2009(H1N1
NP	CY046886	H1N1	05/03/2009	USA	A/Wisconsin/629-D00498/2009(H1N1
NP	CY046278	H1N1	05/03/2009	USA	A/Wisconsin/629-D01092/2009(H1N1
NP	CY046286	H1N1	05/03/2009	USA	A/Wisconsin/629-D02473/2009(H1N1
NP	CY046230	H1N1	05/03/2009	USA	A/Wisconsin/629-D01296/2009(H1N1
NP	CY065351	H1N1	05/03/2009	United Ki	A/England/247/2009
NP	CY065295	H1N1	05/03/2009	United Ki	A/Scotland/4/2009
NP	CY069790	H1N1	05/03/2009	United Ki	A/England/222/2009
NP	CY046214	H1N1	05/03/2009	USA	A/Wisconsin/629-D00869/2009(H1N1
NP	CY046374	H1N1	05/03/2009	USA	A/Wisconsin/629-D01705/2009(H1N1
NP	GQ251036	H1N1	05/03/2009	Italy	A/Italy/05/2009
NP	CY046326	H1N1	05/04/2009	USA	A/Wisconsin/629-D01810/2009(H1N1

NP	CY041785	H1N1	05/04/2009	USA	A/New York/3337/2009(H1N1)
NP	CY046510	H1N1	05/04/2009	USA	A/Wisconsin/629-D02425/2009(H1N1
NP	CY046366	H1N1	05/04/2009	USA	A/Wisconsin/629-D00790/2009(H1N1
NP	CY046526	H1N1	05/04/2009	USA	A/Wisconsin/629-D00183/2009(H1N1
NP	CY046406	H1N1	05/04/2009	USA	A/Wisconsin/629-D00807/2009(H1N1
NP	CY049844	H1N1	05/04/2009	USA	A/Craven/WR0093/2009(H1N1)
NP	CY050145	H1N1	05/04/2009	USA	A/Wisconsin/629-D01058/2009(H1N1
NP	GQ232069	H1N1	05/04/2009	USA	A/Missouri/03/2009(H1N1)
NP	CY043206	H1N1	05/04/2009	USA	A/New York/3338/2009(H1N1)
NP	GQ232004	H1N1	05/04/2009	USA	A/Vermont/03/2009(H1N1)
NP	GQ232072	H1N1	05/04/2009	USA	A/Wisconsin/10/2009(H1N1)
NP	CY041648	H1N1	05/04/2009	USA	A/New York/3313/2009(H1N1)
NP	GQ323471	H1N1	05/04/2009	USA	A/Kentucky/07/2009(H1N1)
NP	GQ377041	H1N1	05/04/2009	USA	A/Montana/07/2009(H1N1)
NP	CY069822	H1N1	05/04/2009	United Ki	A/England/250/2009
NP	CY069806	H1N1	05/04/2009	United Ki	A/England/XFL00230/2009
NP	CY046446	H1N1	05/04/2009	USA	A/Wisconsin/629-D01226/2009(H1N1
NP	GQ402256	H1N1	05/04/2009	Canada	A/Canada-SK/RV1767/2009
NP	CY053153	H1N1	05/04/2009	USA	A/Houston/22H/2009(H1N1)
NP	CY074558	H1N1	05/04/2009	USA	A/California/VRDL324/2009(H1N1)
NP	CY046222	H1N1	05/04/2009	USA	A/Wisconsin/629-D01251/2009(H1N1
NP	CY046534	H1N1	05/04/2009	USA	A/Wisconsin/629-D00714/2009(H1N1
NP	CY046254	H1N1	05/04/2009	USA	A/Wisconsin/629-D02432/2009(H1N1
NP	GQ232034	H1N1	05/04/2009	USA	A/Wisconsin/11/2009(H1N1)
NP	CY065359	H1N1	05/04/2009	United Ki	A/England/XFL00200/2009
NP	CY053145	H1N1	05/04/2009	USA	A/Houston/220S/2009(H1N1)
NP	CY046918	H1N1	05/04/2009	USA	A/Wisconsin/629-D01764/2009(H1N1
NP	CY046870	H1N1	05/04/2009	USA	A/Wisconsin/629-D02272/2009(H1N1
NP	CY046502	H1N1	05/04/2009	USA	A/Wisconsin/629-D00378/2009(H1N1
NP	CY046486	H1N1	05/04/2009	USA	A/Wisconsin/629-D00349/2009(H1N1
NP	CY046878	H1N1	05/04/2009	USA	A/Wisconsin/629-D00396/2009(H1N1
NP	CY046862	H1N1	05/04/2009	USA	A/Wisconsin/629-D01415/2009(H1N1
NP	CY046470	H1N1	05/04/2009	USA	A/Wisconsin/629-D01962/2009(H1N1
NP	CY046894	H1N1	05/04/2009	USA	A/Wisconsin/629-D01880/2009(H1N1
NP	CY043174	H1N1	05/04/2009	USA	A/New York/3460/2009(H1N1)
NP	CY045483	H1N1	05/04/2009	Germany	A/Sachsen-Anhalt/101/2009(H1N1)
NP	GQ231992	H1N1	05/05/2009	USA	A/Ohio/09/2009(H1N1)
NP	GQ231989	H1N1	05/05/2009	USA	A/South Dakota/04/2009(H1N1)
NP	CY049821	H1N1	05/05/2009	USA	A/Craven/WR0019/2009(H1N1)
NP	CY069830	H1N1	05/05/2009	United Ki	A/England/XFL00303/2009
NP	CY065383	H1N1	05/05/2009	United Ki	A/England/XFL00304/2009
NP	GQ457500	H1N1	05/05/2009	USA	A/Texas/30/2009(H1N1)
NP	CY069814	H1N1	05/05/2009	United Ki	A/England/249/2009
NP	CY074446	H1N1	05/05/2009	USA	A/California/VRDL281/2009(H1N1)
NP	CY065367	H1N1	05/05/2009	United Ki	A/England/XFL00275/2009
NP	CY065415	H1N1	05/05/2009	United Ki	A/England/254/2009
NP	CY069798	H1N1	05/05/2009	United Ki	A/England/XFL00228/2009
NP	HM567577	H1N1	05/05/2009	United Ki	A/England/249/2009
NP	CY041825	H1N1	05/06/2009	USA	A/New York/3352/2009(H1N1)
NP	GQ402258	H1N1	05/06/2009	Canada	A/Canada-SK/RV1798/2009
NP	GQ169385	H1N1	05/06/2009	Thailand	A/Thailand/104/2009
NP	CY041753	H1N1	05/06/2009	USA	A/New York/3323/2009(H1N1)
NP	CY043198	H1N1	05/06/2009	USA	A/New York/3324/2009(H1N1)
NP	CY047337	H1N1	05/06/2009	USA	A/New York/3364/2009(H1N1)

NP	CY041817	H1N1	05/06/2009	USA	A/New York/3351/2009(H1N1)
NP	CY046558	H1N1	05/06/2009	USA	A/Wisconsin/629-D00228/2009(H1N1
NP	GQ465718	H1N1	05/07/2009	Canada	A/Canada-SK/RV1794/2009(H1N1)
NP	GQ323496	H1N1	05/07/2009	USA	A/Arkansas/03/2009(H1N1)
NP	GQ323511	H1N1	05/07/2009	USA	A/Washington/14/2009(H1N1)
NP	CY074550	H1N1	05/07/2009	USA	A/California/VRDL320/2009(H1N1)
NP	CY050201	H1N1	05/07/2009	Mexico	A/Mexico City/001/2009
NP	CY100449	H1N1	05/07/2009	Mexico	A/Mexico City/IBT22/2009
NP	GQ402255	H1N1	05/07/2009	Canada	A/Canada-QC/RV1759/2009
NP	KC782029	H1N1	05/07/2009	USA	A/Washington/51/2009
NP	CY100457	H1N1	05/07/2009	Mexico	A/Mexico City/IBT23/2009
NP	CY065407	H1N1	05/07/2009	United Ki	A/England/253/2009
NP	CY065399	H1N1	05/07/2009	United Ki	A/England/XFL00311/2009
NP	CY040873	H1N1	05/07/2009	Norway	A/Norway/1168/2009(H1N1)
NP	GQ232029	H1N1	05/07/2009	USA	A/Arizona/07/2009(H1N1)
NP	GQ465719	H1N1	05/07/2009	Canada	A/Canada-SK/RV1797/2009(H1N1)
NP	CY046606	H1N1	05/07/2009	USA	A/Wisconsin/629-D00487/2009(H1N1
NP	CY065375	H1N1	05/07/2009	United Ki	A/England/XFL00300/2009
NP	CY050217	H1N1	05/08/2009	Mexico	A/Mexico City/003/2009(H1N1)
NP	GQ169303	H1N1	05/08/2009	Japan	A/Narita/1/2009
NP	CY043126	H1N1	05/08/2009	USA	A/New York/3389/2009(H1N1)
NP	GQ323541	H1N1	05/08/2009	USA	A/Texas/35/2009(H1N1)
NP	CY046574	H1N1	05/08/2009	USA	A/Wisconsin/629-D00905/2009(H1N1
NP	CY046542	H1N1	05/08/2009	USA	A/Wisconsin/629-D01313/2009(H1N1
NP	GQ465716	H1N1	05/08/2009	Canada	A/Canada-PQ/RV1758/2009(H1N1)
NP	CY040881	H1N1	05/08/2009	Norway	A/Norway/1177/2009(H1N1)
NP	CY050882	H1N1	05/08/2009	Mexico	A/Mexico City/005/2009(H1N1)
NP	CY074590	H1N1	05/08/2009	USA	A/California/VRDL348/2009(H1N1)
NP	CY050209	H1N1	05/08/2009	Mexico	A/Mexico City/002/2009(H1N1)
NP	CY046590	H1N1	05/08/2009	USA	A/Wisconsin/629-D02452/2009(H1N1
NP	CY065391	H1N1	05/08/2009	United Ki	A/England/252/2009
NP	GQ402257	H1N1	05/08/2009	Canada	A/Canada-SK/RV1793/2009
NP	CY065431	H1N1	05/08/2009	United Ki	A/England/256/2009
NP	CY050225	H1N1	05/08/2009	Mexico	A/Mexico City/004/2009(H1N1)
NP	CY069838	H1N1	05/08/2009	United Ki	A/England/XFL00364/2009
NP	CY046582	H1N1	05/08/2009	USA	A/Wisconsin/629-D01909/2009(H1N1
NP	CY041769	H1N1	05/08/2009	USA	A/New York/3354/2009(H1N1)
NP	CY073359	H1N1	05/08/2009	USA	A/San Diego/WR1630P/2009(H1N1)
NP	CY074582	H1N1	05/08/2009	USA	A/California/VRDL346/2009(H1N1)
NP	CY050257	H1N1	05/09/2009	Mexico	A/Mexico City/012/2009
NP	CY050775	H1N1	05/09/2009	USA	A/New York/3442/2009(H1N1)
NP	CY046566	H1N1	05/09/2009	USA	A/Wisconsin/629-D01919/2009(H1N1
NP	CY045947	H1N1	05/09/2009	Canada	A/Toronto/C2781/2009(H1N1)
NP	CY092104	H1N1	05/09/2009	Mexico	A/Mexico City/016/2009
NP	CY050241	H1N1	05/09/2009	Mexico	A/Mexico City/010/2009
NP	CY050874	H1N1	05/09/2009	Mexico	A/Mexico City/017/2009
NP	CY050249	H1N1	05/09/2009	Mexico	A/Mexico City/011/2009
NP	CY050177	H1N1	05/09/2009	Mexico	A/Mexico City/015/2009
NP	CY069846	H1N1	05/09/2009	United Ki	A/England/XFL00376/2009
NP	CY062638	H1N1	05/09/2009	Mexico	A/Mexico City/014/2009
NP	CY050866	H1N1	05/09/2009	Mexico	A/Mexico City/013/2009
NP	CY065439	H1N1	05/09/2009	United Ki	A/England/257/2009
NP	CY050850	H1N1	05/09/2009	Mexico	A/Mexico City/007/2009(H1N1)
NP	CY050233	H1N1	05/09/2009	Mexico	A/Mexico City/006/2009(H1N1)

NP	CY046614	H1N1	05/09/2009	USA	A/Wisconsin/629-D01055/2009(H1N1
NP	GQ166225	H1N1	05/10/2009	China	A/Sichuan/1/2009(H1N1)
NP	CY050169	H1N1	05/10/2009	Mexico	A/Mexico City/008/2009(H1N1)
NP	GQ323538	H1N1	05/10/2009	USA	A/Missouri/04/2009(H1N1)
NP	JQ173157	H1N1	05/10/2009	Finland	A/Helsinki/P15/2009(H1N1)
NP	CY050890	H1N1	05/10/2009	Mexico	A/Mexico City/018/2009
NP	CY065423	H1N1	05/10/2009	United Ki	A/England/XFL00354/2009
NP	CY046550	H1N1	05/10/2009	USA	A/Wisconsin/629-D00179/2009(H1N1
NP	CY074566	H1N1	05/10/2009	USA	A/California/VRDL326/2009(H1N1)
NP	CY050447	H1N1	05/10/2009	USA	A/Wisconsin/629-D01482/2009(H1N1
NP	GQ200289	H1N1	05/10/2009	China	A/Shandong/1/2009(H1N1)
NP	CY046598	H1N1	05/10/2009	USA	A/Wisconsin/629-D01482/2009(H1N1
NP	CY041761	H1N1	05/11/2009	USA	A/New York/3413/2009(H1N1)
NP	CY043222	H1N1	05/11/2009	USA	A/New York/3455/2009(H1N1)
NP	GQ232058	H1N1	05/11/2009	USA	A/North Dakota/04/2009(H1N1)
NP	GQ249339	H1N1	05/11/2009	France	A/Paris/2629/2009(H1N1)
NP	CY043166	H1N1	05/11/2009	USA	A/New York/3454/2009(H1N1)
NP	CY065447	H1N1	05/11/2009	United Ki	A/England/259/2009
NP	CY065455	H1N1	05/11/2009	United Ki	A/England/XFL00418/2009
NP	CY069854	H1N1	05/11/2009	United Ki	A/England/260/2009
NP	CY065479	H1N1	05/12/2009	United Ki	A/England/263/2009
NP	CY065463	H1N1	05/12/2009	United Ki	A/England/XFL00419/2009
NP	CY069862	H1N1	05/12/2009	United Ki	A/England/XFL00421/2009
NP	CY050858	H1N1	05/12/2009	Mexico	A/Mexico City/009/2009(H1N1)
NP	CY043302	H1N1	05/12/2009	USA	A/New York/3498/2009(H1N1)
NP	CY043158	H1N1	05/12/2009	USA	A/New York/3418/2009(H1N1)
NP	CY043182	H1N1	05/12/2009	USA	A/New York/3488/2009(H1N1)
NP	CY050331	H1N1	05/12/2009	USA	A/Wisconsin/629-D01851/2009(H1N1
NP	CY065495	H1N1	05/12/2009	United Ki	A/England/265/2009
NP	CY065471	H1N1	05/12/2009	United Ki	A/England/XFL00473/2009
NP	CY065487	H1N1	05/12/2009	United Ki	A/England/264/2009
NP	CY065503	H1N1	05/13/2009	United Ki	A/England/XFL00481/2009
NP	GQ396528	H1N1	05/13/2009	Spain	A/Madrid/GP523/2009(H1N1)
NP	CY050759	H1N1	05/13/2009	USA	A/New York/3467/2009(H1N1)
NP	CY053058	H1N1	05/13/2009	USA	A/Houston/100S/2009(H1N1)
NP	CY053066	H1N1	05/13/2009	USA	A/Houston/10H/2009(H1N1)
NP	CY043286	H1N1	05/13/2009	USA	A/New York/3449/2009(H1N1)
NP	CY052962	H1N1	05/13/2009	USA	A/Houston/1H/2009(H1N1)
NP	CY051906	H1N1	05/13/2009	USA	A/Houston/10S/2009(H1N1)
NP	CY046678	H1N1	05/13/2009	USA	A/Wisconsin/629-D01305/2009(H1N1
NP	CY043214	H1N1	05/13/2009	USA	A/New York/3420/2009(H1N1)
NP	KC781318	H1N1	05/13/2009	USA	A/Mississippi/01/2009
NP	CY065511	H1N1	05/13/2009	United Ki	A/England/XFL00482/2009
NP	CY050375	H1N1	05/13/2009	USA	A/Wisconsin/629-D01154/2009(H1N1
NP	CY046686	H1N1	05/14/2009	USA	A/Wisconsin/629-D00978/2009(H1N1
NP	CY043134	H1N1	05/14/2009	USA	A/New York/3514/2009(H1N1)
NP	CY041793	H1N1	05/14/2009	USA	A/New York/3463/2009(H1N1)
NP	CY046662	H1N1	05/14/2009	USA	A/Wisconsin/629-D00997/2009(H1N1
NP	CY046670	H1N1	05/14/2009	USA	A/Wisconsin/629-D00064/2009(H1N1
NP	CY046654	H1N1	05/14/2009	USA	A/Wisconsin/629-D01837/2009(H1N1
NP	CY065527	H1N1	05/15/2009	United Ki	A/England/XFL00532/2009
NP	CY065519	H1N1	05/15/2009	United Ki	A/England/266/2009
NP	GQ225374	H1N1	05/15/2009	China	A/Beijing/01/2009(H1N1)
NP	CY044976	H1N1	05/15/2009	USA	A/New York/3744/2009(H1N1)

NP	CY048926	H1N1	05/15/2009	Malaysia	A/Malaysia/820/2009(H1N1)
NP	CY050383	H1N1	05/15/2009	USA	A/Wisconsin/629-D01295/2009(H1N1
NP	CY050391	H1N1	05/15/2009	USA	A/Wisconsin/629-D01839/2009(H1N1
NP	CY083297	H1N1	05/15/2009	USA	A/San Diego/WRAIR1647P/2009
NP	CY046630	H1N1	05/15/2009	USA	A/Wisconsin/629-D01839/2009(H1N1
NP	CY048934	H1N1	05/16/2009	Malaysia	A/Malaysia/854/2009(H1N1)
NP	GQ183620	H1N1	05/16/2009	China	A/Beijing/01/2009(H1N1)
NP	CY043238	H1N1	05/16/2009	USA	A/New York/3502/2009(H1N1)
NP	CY043246	H1N1	05/16/2009	USA	A/New York/3509/2009(H1N1)
NP	CY043254	H1N1	05/16/2009	USA	A/New York/3545/2009(H1N1)
NP	CY043294	H1N1	05/16/2009	USA	A/New York/3469/2009(H1N1)
NP	GQ223422	H1N1	05/16/2009	Japan	A/Osaka/2/2009(H1N1)
NP	GQ223423	H1N1	05/16/2009	Japan	A/Osaka-C/1/2009(H1N1)
NP	CY044984	H1N1	05/16/2009	USA	A/New York/3747/2009(H1N1)
NP	CY046646	H1N1	05/16/2009	USA	A/Wisconsin/629-D02227/2009(H1N1
NP	GQ223421	H1N1	05/16/2009	Japan	A/Osaka/1/2009(H1N1)
NP	CY062541	H1N1	05/16/2009	Mexico	A/Mexico city/CIA9/2009(H1N1)
NP	CY046622	H1N1	05/16/2009	USA	A/Wisconsin/629-D00575/2009(H1N1
NP	CY043190	H1N1	05/16/2009	USA	A/New York/3499/2009(H1N1)
NP	CY041777	H1N1	05/16/2009	USA	A/New York/3532/2009(H1N1)
NP	CY046638	H1N1	05/16/2009	USA	A/Wisconsin/629-D00967/2009(H1N1
NP	CY046694	H1N1	05/16/2009	USA	A/Wisconsin/629-D00434/2009(H1N1
NP	GQ183628	H1N1	05/16/2009	China	A/Beijing/02/2009(H1N1)
NP	GQ223419	H1N1	05/17/2009	Japan	A/Hyogo/2/2009(H1N1)
NP	CY045088	H1N1	05/17/2009	USA	A/New York/3574/2009(H1N1)
NP	GQ223424	H1N1	05/17/2009	Japan	A/Osaka-C/2/2009(H1N1)
NP	GQ223418	H1N1	05/17/2009	Japan	A/Hyogo/1/2009(H1N1)
NP	CY043230	H1N1	05/17/2009	USA	A/New York/3501/2009(H1N1)
NP	CY043310	H1N1	05/17/2009	USA	A/New York/3534/2009(H1N1)
NP	CY083289	H1N1	05/17/2009	USA	A/San Diego/WRAIR1646P/2009
NP	CY050399	H1N1	05/17/2009	USA	A/Wisconsin/629-D00989/2009(H1N1
NP	CY041801	H1N1	05/17/2009	USA	A/New York/3468/2009(H1N1)
NP	GQ221695	H1N1	05/17/2009	China	A/Guangdong/1/2009(H1N1)
NP	GQ402259	H1N1	05/17/2009	Canada	A/Canada-QC/RV1954/2009
NP	CY069870	H1N1	05/17/2009	United Ki	A/England/271/2009
NP	CY074574	H1N1	05/17/2009	USA	A/California/VRDL341/2009(H1N1)
NP	CY044131	H1N1	05/17/2009	USA	A/New York/3516/2009(H1N1)
NP	GQ223444	H1N1	05/18/2009	China	A/Guangdong/1/2009(H1N1)
NP	CY044912	H1N1	05/18/2009	USA	A/New York/3612/2009(H1N1)
NP	CY044091	H1N1	05/18/2009	USA	A/New York/3740/2009(H1N1)
NP	CY047423	H1N1	05/18/2009	Australia	A/Victoria/2005/2009(H1N1)
NP	CY052986	H1N1	05/18/2009	USA	A/Houston/30S/2009(H1N1)
NP	CY052994	H1N1	05/18/2009	USA	A/Houston/3H/2009(H1N1)
NP	CY052970	H1N1	05/18/2009	USA	A/Houston/20S/2009(H1N1)
NP	GQ267834	H1N1	05/18/2009	Japan	A/Himeji/1/2009(H1N1)
NP	HQ011414	H1N1	05/18/2009	China	A/Guangdong/01/2009(H1N1)
NP	CY051914	H1N1	05/18/2009	USA	A/Houston/2H/2009(H1N1)
NP	CY044920	H1N1	05/19/2009	USA	A/New York/3613/2009(H1N1)
NP	CY044888	H1N1	05/19/2009	USA	A/New York/3580/2009(H1N1)
NP	CY046742	H1N1	05/19/2009	USA	A/Wisconsin/629-D00084/2009(H1N1
NP	CY046718	H1N1	05/19/2009	USA	A/Wisconsin/629-D00752/2009(H1N1
NP	CY050415	H1N1	05/19/2009	USA	A/Wisconsin/629-D00752/2009(H1N1
NP	CY044197	H1N1	05/19/2009	Taiwan	A/Taiwan/T0724/2009(H1N1)
NP	CY045146	H1N1	05/19/2009	USA	A/New York/3577/2009(H1N1)

NP	CY046774	H1N1	05/19/2009	USA	A/Wisconsin/629-D00722/2009(H1N1
NP	CY043142	H1N1	05/19/2009	USA	A/New York/3529/2009(H1N1)
NP	GQ300862	H1N1	05/19/2009	Japan	A/Shiga/2/2009(H1N1)
NP	CY047361	H1N1	05/19/2009	USA	A/New York/3741/2009(H1N1)
NP	CY053002	H1N1	05/19/2009	USA	A/Houston/50S/2009(H1N1)
NP	CY053010	H1N1	05/19/2009	USA	A/Houston/5H/2009(H1N1)
NP	CY083313	H1N1	05/19/2009	USA	A/San Diego/WRAIR1649P/2009
NP	CY044067	H1N1	05/19/2009	USA	A/New York/3568/2009(H1N1)
NP	CY046758	H1N1	05/19/2009	USA	A/Wisconsin/629-D00160/2009(H1N1
NP	CY043270	H1N1	05/19/2009	USA	A/New York/3563/2009(H1N1)
NP	GQ323490	H1N1	05/19/2009	USA	A/Arizona/09/2009(H1N1)
NP	CY044139	H1N1	05/19/2009	USA	A/New York/3528/2009(H1N1)
NP	GQ402260	H1N1	05/20/2009	Canada	A/Canada-MB/RV1964/2009
NP	CY044896	H1N1	05/20/2009	USA	A/New York/3582/2009(H1N1)
NP	GQ225382	H1N1	05/20/2009	China	A/Beijing/3/2009(H1N1)
NP	CY044872	H1N1	05/20/2009	USA	A/New York/3571/2009(H1N1)
NP	CY053018	H1N1	05/20/2009	USA	A/Houston/60S/2009(H1N1)
NP	CY053185	H1N1	05/20/2009	USA	A/Brownsville/270S/2009(H1N1)
NP	CY053026	H1N1	05/20/2009	USA	A/Houston/6H/2009(H1N1)
NP	CY053177	H1N1	05/20/2009	USA	A/Brownsville/260S/2009(H1N1)
NP	CY046734	H1N1	05/20/2009	USA	A/Wisconsin/629-D01521/2009(H1N1
NP	CY044059	H1N1	05/20/2009	USA	A/New York/3567/2009(H1N1)
NP	GQ290109	H1N1	05/20/2009	China	A/Beijing/502/2009(H1N1)
NP	CY044051	H1N1	05/20/2009	USA	A/New York/3566/2009(H1N1)
NP	CY046726	H1N1	05/20/2009	USA	A/Wisconsin/629-D02427/2009(H1N1
NP	CY046710	H1N1	05/20/2009	USA	A/Wisconsin/629-D01787/2009(H1N1
NP	CY043262	H1N1	05/20/2009	USA	A/New York/3552/2009(H1N1)
NP	CY044936	H1N1	05/20/2009	USA	A/New York/3623/2009(H1N1)
NP	CY043150	H1N1	05/20/2009	USA	A/New York/3551/2009(H1N1)
NP	CY053042	H1N1	05/21/2009	USA	A/Houston/7H/2009(H1N1)
NP	GQ246614	H1N1	05/21/2009	Russia	A/Moscow/01/2009(H1N1)
NP	CY053050	H1N1	05/21/2009	USA	A/Houston/9H/2009(H1N1)
NP	CY046966	H1N1	05/21/2009	USA	A/New York/3576/2009(H1N1)
NP	CY052978	H1N1	05/21/2009	USA	A/Houston/20S/2009(H1N1)
NP	CY087011	H1N1	05/21/2009	USA	A/New York/3565/2009(H1N1)
NP	CY044904	H1N1	05/21/2009	USA	A/New York/3611/2009(H1N1)
NP	GQ402263	H1N1	05/21/2009	Canada	A/Canada-MB/RV1982/2009
NP	GQ219587	H1N1	05/21/2009	Russia	A/Moscow/IIV01/2009(H1N1)
NP	GQ267841	H1N1	05/21/2009	Japan	A/Sakai/1/2009(H1N1)
NP	CY044968	H1N1	05/21/2009	USA	A/New York/3653/2009(H1N1)
NP	GQ267847	H1N1	05/21/2009	Japan	A/Sakai/2/2009(H1N1)
NP	CY044880	H1N1	05/21/2009	USA	A/New York/3573/2009(H1N1)
NP	CY053034	H1N1	05/21/2009	USA	A/Houston/70S/2009(H1N1)
NP	CY051922	H1N1	05/21/2009	USA	A/Houston/90S/2009(H1N1)
NP	CY089875	H1N1	05/21/2009	USA	A/Wisconsin/629-D02275/2009(H1N1
NP	CY053193	H1N1	05/21/2009	USA	A/Brownsville/290S/2009(H1N1)
NP	CY053201	H1N1	05/21/2009	USA	A/Brownsville/300S/2009(H1N1)
NP	GQ225366	H1N1	05/22/2009	China	A/Fujian/1/2009(H1N1)
NP	CY089182	H1N1	05/22/2009	Chile	A/Chile/19/2009
NP	CY053217	H1N1	05/22/2009	USA	A/Brownsville/31H/2009(H1N1)
NP	CY046766	H1N1	05/22/2009	USA	A/Wisconsin/629-D02298/2009(H1N1
NP	CY053209	H1N1	05/22/2009	USA	A/Brownsville/310S/2009(H1N1)
NP	GQ402262	H1N1	05/22/2009	Canada	A/Canada-MB/RV1977/2009
NP	CY044952	H1N1	05/22/2009	USA	A/New York/3627/2009(H1N1)

NP	CY044213	H1N1	05/22/2009	Taiwan	A/Taiwan/T1339/2009(H1N1)
NP	CY045096	H1N1	05/22/2009	USA	A/New York/3614/2009(H1N1)
NP	GQ267853	H1N1	05/22/2009	Japan	A/Shiga/1/2009(H1N1)
NP	CY046750	H1N1	05/22/2009	USA	A/Wisconsin/629-D02064/2009(H1N1
NP	CY044205	H1N1	05/22/2009	Taiwan	A/Taiwan/T1338/2009(H1N1)
NP	CY050423	H1N1	05/23/2009	USA	A/Wisconsin/629-D02206/2009(H1N1
NP	CY053130	H1N1	05/23/2009	USA	A/Houston/200S/2009(H1N1)
NP	CY053233	H1N1	05/23/2009	USA	A/Brownsville/34H/2009(H1N1)
NP	GQ225350	H1N1	05/23/2009	China	A/Zhejiang/1/2009(H1N1)
NP	CY053225	H1N1	05/23/2009	USA	A/Brownsville/340S/2009(H1N1)
NP	CY050407	H1N1	05/23/2009	USA	A/Wisconsin/629-D00451/2009(H1N1
NP	GQ232094	H1N1	05/23/2009	China	A/Beijing/4/2009(H1N1)
NP	CY053138	H1N1	05/23/2009	USA	A/Houston/20H/2009(H1N1)
NP	CY049908	H1N1	05/23/2009	Dominicar	A/Santo Domingo/0574/2009(H1N1)
NP	GQ323531	H1N1	05/23/2009	USA	A/Illinois/04/2009(H1N1)
NP	CY075086	H1N1	05/23/2009	Chile	A/Chile/28/2009(H1N1)
NP	GQ225358	H1N1	05/23/2009	China	A/Shanghai/1/2009(H1N1)
NP	GQ338334	H1N1	05/23/2009	USA	A/New York/58/2009(H1N1)
NP	CY049916	H1N1	05/23/2009	Dominicar	A/Santo Domingo/0574T/2009(H1N1)
NP	GQ323580	H1N1	05/24/2009	USA	A/West Virginia/01/2009(H1N1)
NP	CY041984	H1N1	05/24/2009	Dominicar	A/Santo Domingo/0573N/2009(H1N1)
NP	GQ402247	H1N1	05/24/2009	Canada	A/Canada-MB/RV2023/2009
NP	CY045939	H1N1	05/24/2009	Canada	A/Toronto/C0270/2009(H1N1)
NP	CY044960	H1N1	05/24/2009	USA	A/New York/3629/2009(H1N1)
NP	CY046702	H1N1	05/24/2009	USA	A/Wisconsin/629-D01445/2009(H1N1
NP	HM567593	H1N1	05/24/2009	Ireland	A/Ireland/1/2009
NP	GQ253494	H1N1	05/24/2009	China	A/Shanghai/37T/2009(H1N1)
NP	CY041961	H1N1	05/24/2009	Dominicar	A/Santo Domingo/572N/2009(H1N1)
NP	GQ402261	H1N1	05/24/2009	Canada	A/Canada-MB/RV1975/2009
NP	CY043278	H1N1	05/25/2009	USA	A/New York/3651/2009(H1N1)
NP	CY044928	H1N1	05/25/2009	USA	A/New York/3617/2009(H1N1)
NP	JQ409127	H1N1	05/25/2009	Finland	A/Helsinki/Vi1/2009(H1N1)
NP	CY075126	H1N1	05/25/2009	Chile	A/Chile/42/2009(H1N1)
NP	CY075094	H1N1	05/25/2009	Chile	A/Chile/31/2009
NP	CY050431	H1N1	05/25/2009	USA	A/Wisconsin/629-D02324/2009(H1N1
NP	CY075118	H1N1	05/25/2009	Chile	A/Chile/40/2009(H1N1)
NP	CY075102	H1N1	05/25/2009	Chile	A/Chile/32/2009(H1N1)
NP	CY075110	H1N1	05/25/2009	Chile	A/Chile/36/2009(H1N1)
NP	GQ385301	H1N1	05/26/2009	Canada	A/Toronto/0462/2009(H1N1)
NP	GQ375285	H1N1	05/26/2009	Russia	A/Moscow/03/2009(H1N1)
NP	CY043111	H1N1	05/26/2009	Dominicar	A/Santo Domingo/568T/2009(H1N1)
NP	GU138928	H1N1	05/26/2009	Japan	A/Shizuoka-C/97/2009(H1N1)
NP	HM567601	H1N1	05/26/2009	United Ki	A/England/313/2009
NP	CY051250	H1N1	05/26/2009	USA	A/Wisconsin/629-S0035/2009(H1N1)
NP	CY075134	H1N1	05/26/2009	Chile	A/Chile/56/2009(H1N1)
NP	HM567833	H1N1	05/26/2009	Ireland	A/Ireland/3/2009
NP	HM567809	H1N1	05/26/2009	Ireland	A/Ireland/2/2009
NP	HM567585	H1N1	05/26/2009	United Ki	A/England/303/2009
NP	CY043103	H1N1	05/26/2009	Dominicar	A/Santo Domingo/565T/2009(H1N1)
NP	GQ247732	H1N1	05/26/2009	Russia	A/Moscow/IIV02/2009(H1N1)
NP	CY050751	H1N1	05/26/2009	USA	A/New York/3768/2009(H1N1)
NP	CY044944	H1N1	05/26/2009	USA	A/New York/3625/2009(H1N1)
NP	CY045154	H1N1	05/26/2009	USA	A/New York/3655/2009(H1N1)
NP	CY046782	H1N1	05/26/2009	USA	A/Wisconsin/629-D01894/2009(H1N1

NP	CY046958	H1N1	05/26/2009	USA	A/New York/3654/2009(H1N1)
NP	CY044075	H1N1	05/26/2009	USA	A/New York/3702/2009(H1N1)
NP	GQ283482	H1N1	05/27/2009	Italy	A/Italy/49/2009(H1N1)
NP	CY075142	H1N1	05/27/2009	Chile	A/Chile/88/2009(H1N1)
NP	CY053257	H1N1	05/27/2009	USA	A/Brownsville/36H/2009(H1N1)
NP	CY075150	H1N1	05/27/2009	Chile	A/Chile/95/2009(H1N1)
NP	CY076756	H1N1	05/27/2009	Canada	A/Ontario/142358/2009(H1N1)
NP	CY074003	H1N1	05/27/2009	Argentina	A/Argentina/HNRG14/2009(H1N1)
NP	CY069878	H1N1	05/27/2009	United Ki	A/England/350/2009
NP	CY053241	H1N1	05/27/2009	USA	A/Brownsville/350S/2009(H1N1)
NP	CY046838	H1N1	05/27/2009	USA	A/Wisconsin/629-D01883/2009(H1N1
NP	CY046814	H1N1	05/27/2009	USA	A/Wisconsin/629-D00698/2009(H1N1
NP	CY046790	H1N1	05/27/2009	USA	A/Wisconsin/629-D02276/2009(H1N1
NP	CY050439	H1N1	05/27/2009	USA	A/Wisconsin/629-D01886/2009(H1N1
NP	CY050898	H1N1	05/27/2009	USA	A/Wisconsin/629-D00949/2009(H1N1
NP	GQ232086	H1N1	05/27/2009	China	A/Guangdong/02/2009(H1N1)
NP	CY049932	H1N1	05/27/2009	USA	A/Craven/WR1001/2009(H1N1)
NP	CY053249	H1N1	05/27/2009	USA	A/Brownsville/35H/2009(H1N1)
NP	HM567689	H1N1	05/27/2009	United Ki	A/England/350/2009
NP	GQ290438	H1N1	05/28/2009	China	A/Shanghai/60T/2009(H1N1)
NP	CY075158	H1N1	05/28/2009	Chile	A/Chile/158/2009(H1N1)
NP	CY046902	H1N1	05/28/2009	USA	A/Wisconsin/629-D00829/2009(H1N1
NP	CY046830	H1N1	05/28/2009	USA	A/Wisconsin/629-D02144/2009(H1N1
NP	GQ402264	H1N1	05/28/2009	Canada	A/Canada-MB/RV2013/2009
NP	CY053312	H1N1	05/28/2009	USA	A/Brownsville/370S/2009(H1N1)
NP	CY053264	H1N1	05/28/2009	USA	A/Brownsville/37H/2009(H1N1)
NP	GQ328869	H1N1	05/28/2009	Finland	A/Finland/555/2009(H1N1)
NP	CY064792	H1N1	05/28/2009	China	A/Guangdong/SB1/2009(H1N1)
NP	HM567609	H1N1	05/28/2009	United Ki	A/England/328/2009
NP	CY046798	H1N1	05/28/2009	USA	A/Wisconsin/629-D01026/2009(H1N1
NP	CY050914	H1N1	05/28/2009	USA	A/Wisconsin/629-D01262/2009(H1N1
NP	CY050153	H1N1	05/28/2009	USA	A/Wisconsin/629-D00117/2009(H1N1
NP	CY050906	H1N1	05/28/2009	USA	A/Wisconsin/629-D01779/2009(H1N1
NP	GQ288373	H1N1	05/28/2009	China	A/Fuzhou/01/2009(H1N1)
NP	CY046910	H1N1	05/28/2009	USA	A/Wisconsin/629-D01083/2009(H1N1
NP	HQ011419	H1N1	05/28/2009	China	A/Guangdong/45/2009(H1N1)
NP	CY049069	H1N1	05/28/2009	Singapore	A/Singapore/ON129/2009(H1N1)
NP	CY044221	H1N1	05/28/2009	Taiwan	A/Taiwan/T1773/2009(H1N1)
NP	CY045008	H1N1	05/28/2009	USA	A/New York/3709/2009(H1N1)
NP	GQ227548	H1N1	05/29/2009	China	A/Guangdong/03/2009(H1N1)
NP	GQ244323	H1N1	05/29/2009	China	A/Guangdong/05/2009(H1N1)
NP	CY044083	H1N1	05/29/2009	USA	A/New York/3715/2009(H1N1)
NP	HM780471	H1N1	05/29/2009	China	A/Guangdong/06/2009(H1N1)
NP	GQ402265	H1N1	05/29/2009	Canada	A/Canada-MB/RV2018/2009
NP	CY051258	H1N1	05/29/2009	USA	A/Wisconsin/629-S0086/2009(H1N1)
NP	CY075166	H1N1	05/29/2009	Chile	A/Chile/180/2009(H1N1)
NP	HM567633	H1N1	05/29/2009	United Ki	A/England/342/2009
NP	CY050938	H1N1	05/29/2009	USA	A/Wisconsin/629-D01847/2009(H1N1
NP	CY050930	H1N1	05/29/2009	USA	A/Wisconsin/629-D00426/2009(H1N1
NP	HM567841	H1N1	05/29/2009	Ireland	A/Ireland/4/2009
NP	CY050922	H1N1	05/29/2009	USA	A/Wisconsin/629-D02213/2009(H1N1
NP	CY071034	H1N1	05/29/2009	USA	A/New York/NHRC0002/2009(H1N1)
NP	CY053280	H1N1	05/30/2009	USA	A/Brownsville/39H/2009(H1N1)
NP	CY044229	H1N1	05/30/2009	Taiwan	A/Taiwan/T1821/2009(H1N1)

NP	CY046806	H1N1	05/30/2009	USA	A/Wisconsin/629-D00223/2009(H1N1
NP	HM567681	H1N1	05/30/2009	United Ki	A/England/349/2009
NP	HM567673	H1N1	05/30/2009	United Ki	A/England/348/2009
NP	HM567665	H1N1	05/30/2009	United Ki	A/England/347/2009
NP	HM567649	H1N1	05/30/2009	United Ki	A/England/345/2009
NP	HM567625	H1N1	05/30/2009	United Ki	A/England/341/2009
NP	CY089883	H1N1	05/30/2009	USA	A/Wisconsin/629-D01829/2009(H1N1
NP	CY055304	H1N1	05/30/2009	Singapore	A/Singapore/GN285/2009(H1N1)
NP	CY053272	H1N1	05/30/2009	USA	A/Brownsville/390S/2009(H1N1)
NP	HM567617	H1N1	05/30/2009	United Ki	A/England/337/2009
NP	CY046822	H1N1	05/30/2009	USA	A/Wisconsin/629-D02013/2009(H1N1
NP	GQ402266	H1N1	05/30/2009	Canada	A/Canada-MB/RV2020/2009
NP	CY045963	H1N1	05/30/2009	Canada	A/Toronto/T5294/2009(H1N1)
NP	HM567641	H1N1	05/30/2009	United Ki	A/England/343/2009
NP	HM567657	H1N1	05/30/2009	United Ki	A/England/346/2009
NP	HM569662	H1N1	05/30/2009	Argentina	A/Argentina/07-09GP/2009(H1N1)
NP	HM567713	H1N1	05/31/2009	United Ki	A/Scotland/8/2009
NP	HM567849	H1N1	05/31/2009	Ireland	A/Ireland/5/2009
NP	HM567729	H1N1	05/31/2009	United Ki	A/England/365/2009
NP	GQ377106	H1N1	05/31/2009	USA	A/Utah/04/2009(H1N1)
NP	GQ253487	H1N1	05/31/2009	China	A/Zhejiang/2/2009(H1N1)
NP	CY071026	H1N1	05/31/2009	USA	A/New York/NHRC0001/2009(H1N1)
NP	CY044864	H1N1	05/31/2009	USA	A/New York/3726/2009(H1N1)
NP	CY065535	H1N1	05/31/2009	United Ki	A/England/359/2009
NP	HM567721	H1N1	05/31/2009	United Ki	A/England/364/2009
NP	GQ253499	H1N1	05/31/2009	China	A/Shanghai/71T/2009(H1N1)
NP	HM567737	H1N1	05/31/2009	United Ki	A/England/366/2009
NP	CY045032	H1N1	05/31/2009	USA	A/New York/3812/2009(H1N1)
NP	CY045186	H1N1	05/31/2009	USA	A/New York/3960/2009(H1N1)
NP	HM567697	H1N1	05/31/2009	United Ki	A/England/351/2009
NP	GQ359761	H1N1	06/2009	China	A/Zhejiang/DTID-ZJU01/2009(H1N1)
NP	CY069642	H1N1	06/2009	Singapore	A/Singapore/527/2009(H1N1)
NP	CY047788	H1N1	06/2009	Argentina	A/Argentina/7785/2009
NP	CY045235	H1N1	06/2009	Taiwan	A/Taiwan/126/2009(H1N1)
NP	HM598305	H1N1	06/2009	Germany	A/Hamburg/NY1580/2009
NP	CY069627	H1N1	06/2009	Singapore	A/Singapore/471/2009(H1N1)
NP	HQ695921	H1N1	06/2009	Taiwan	A/Taiwan/14/2009
NP	CY069620	H1N1	06/2009	Singapore	A/Singapore/276/2009(H1N1)
NP	CY069635	H1N1	06/2009	Singapore	A/Singapore/478/2009(H1N1)
NP	CY045243	H1N1	06/2009	Taiwan	A/Taiwan/137/2009(H1N1)
NP	CY065551	H1N1	06/01/2009	United Ki	A/England/381/2009
NP	CY076764	H1N1	06/01/2009	Canada	A/Ontario/147265/2009(H1N1)
NP	CY065543	H1N1	06/01/2009	United Ki	A/England/361/2009
NP	FN423708	H1N1	06/01/2009	Luxembour	A/Luxembourg/43/2009
NP	CY044992	H1N1	06/01/2009	USA	A/New York/3751/2009(H1N1)
NP	CY047377	H1N1	06/01/2009	USA	A/New York/3753/2009(H1N1)
NP	CY047369	H1N1	06/01/2009	USA	A/New York/3795/2009(H1N1)
NP	CY045178	H1N1	06/01/2009	USA	A/New York/3959/2009(H1N1)
NP	CY050954	H1N1	06/01/2009	USA	A/Wisconsin/629-D00724/2009(H1N1
NP	HM567769	H1N1	06/01/2009	United Ki	A/England/383/2009
NP	CY065647	H1N1	06/01/2009	United Ki	A/England/402/2009
NP	CY089891	H1N1	06/01/2009	USA	A/Wisconsin/629-D00881/2009(H1N1
NP	CY069886	H1N1	06/01/2009	United Ki	A/England/383/2009
NP	CY065583	H1N1	06/01/2009	United Ki	A/England/386/2009

NP	CY100813	H1N1	06/01/2009	USA	A/Texas/WRAIR1118P/2009(H1N1)
NP	HM567745	H1N1	06/01/2009	United Ki	A/England/374/2009
NP	CY065575	H1N1	06/01/2009	United Ki	A/England/385/2009
NP	HM567705	H1N1	06/01/2009	United Ki	A/England/360/2009
NP	KC800980	H1N1	06/01/2009	France	A/Lyon/969/2009
NP	CY053288	H1N1	06/01/2009	USA	A/Brownsville/400S/2009(H1N1)
NP	CY065559	H1N1	06/01/2009	United Ki	A/England/382/2009
NP	CY071042	H1N1	06/01/2009	USA	A/New York/NHRC0003/2009(H1N1)
NP	CY050946	H1N1	06/01/2009	USA	A/Wisconsin/629-D00374/2009(H1N1
NP	CY050962	H1N1	06/01/2009	USA	A/Wisconsin/629-D00941/2009(H1N1
NP	CY050970	H1N1	06/01/2009	USA	A/Wisconsin/629-D01725/2009(H1N1
NP	HM567817	H1N1	06/01/2009	Ireland	A/Ireland/7/2009
NP	CY065567	H1N1	06/01/2009	United Ki	A/England/384/2009
NP	CY054694	H1N1	06/01/2009	Nicaragua	A/Managua/4467.05/2009(H1N1)
NP	CY056390	H1N1	06/01/2009	Nicaragua	A/Managua/3119.01/2009(H1N1)
NP	CY065623	H1N1	06/01/2009	United Ki	A/England/395/2009
NP	CY050986	H1N1	06/02/2009	USA	A/Wisconsin/629-D00592/2009(H1N1
NP	GQ324573	H1N1	06/02/2009	Japan	A/Tokushima/1/2009(H1N1)
NP	CY076772	H1N1	06/02/2009	Canada	A/Ontario/147723/2009(H1N1)
NP	CY051282	H1N1	06/02/2009	USA	A/Wisconsin/629-S0148/2009(H1N1)
NP	CY053296	H1N1	06/02/2009	USA	A/Brownsville/430S/2009(H1N1)
NP	CY051274	H1N1	06/02/2009	USA	A/Wisconsin/629-S0146/2009(H1N1)
NP	GU138904	H1N1	06/02/2009	Japan	A/Chiba-C/48/2009(H1N1)
NP	GQ329095	H1N1	06/02/2009	France	A/Paris/2709/2009(H1N1)
[truncated: 240,440 more chars]
